# Supplementary material for: Quality of care index and gender disparity ratio for stroke and its subtypes from the Global Burden of Disease Study 2021
Source: PLoS One. 2026 Apr 29;21(4):e0345938. doi: 10.1371/journal.pone.0345938 (PMC13127941; doi:10.1371/journal.pone.0345938)
Supplement: S1 File — (PDF) [file pone.0345938.s001.pdf]

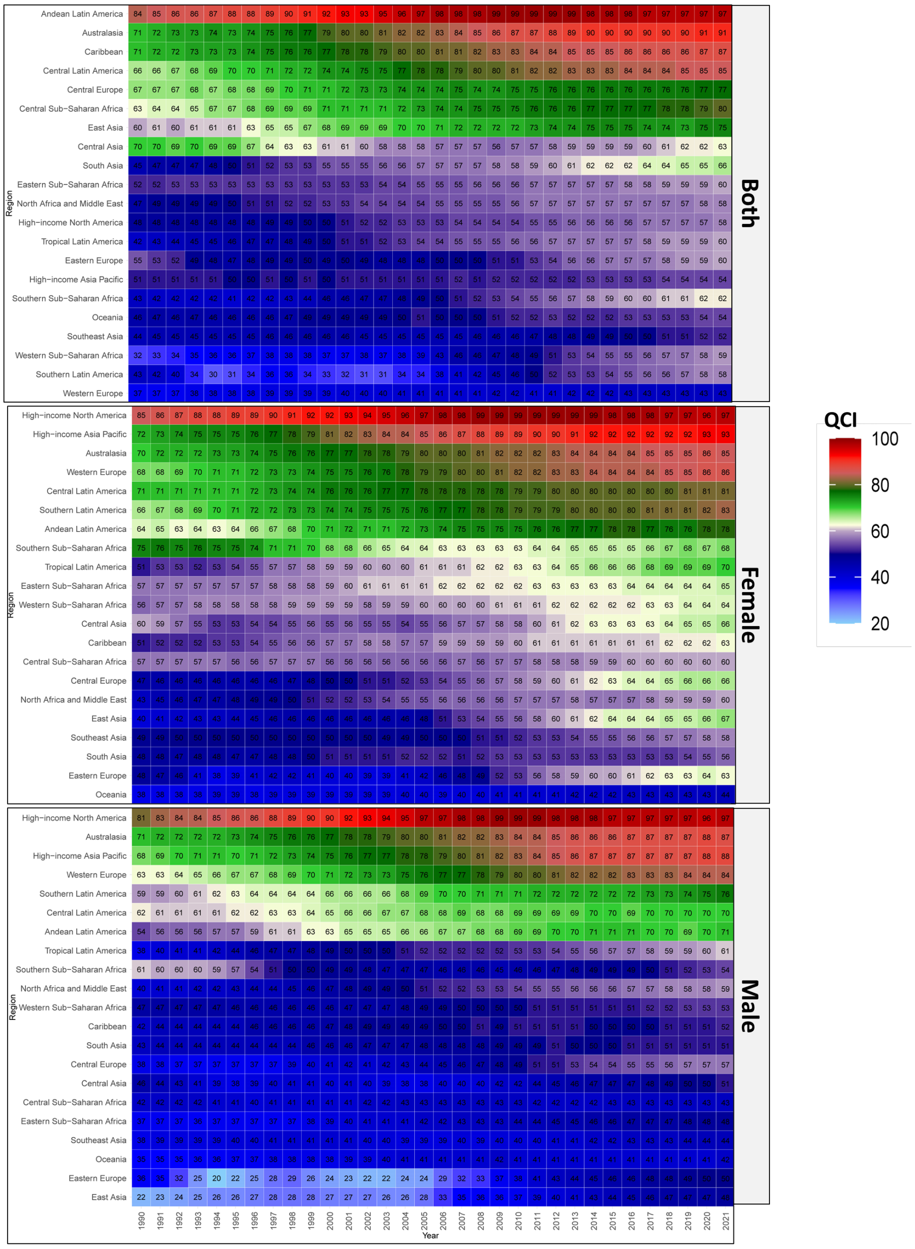

S1 Fig. Ranking of the 21 GBD regions based on age-standardized QCI for stroke in both sexes, female, and male from 1990 to 2021. QCI, quality of care index GBD, Global Burden of Diseases

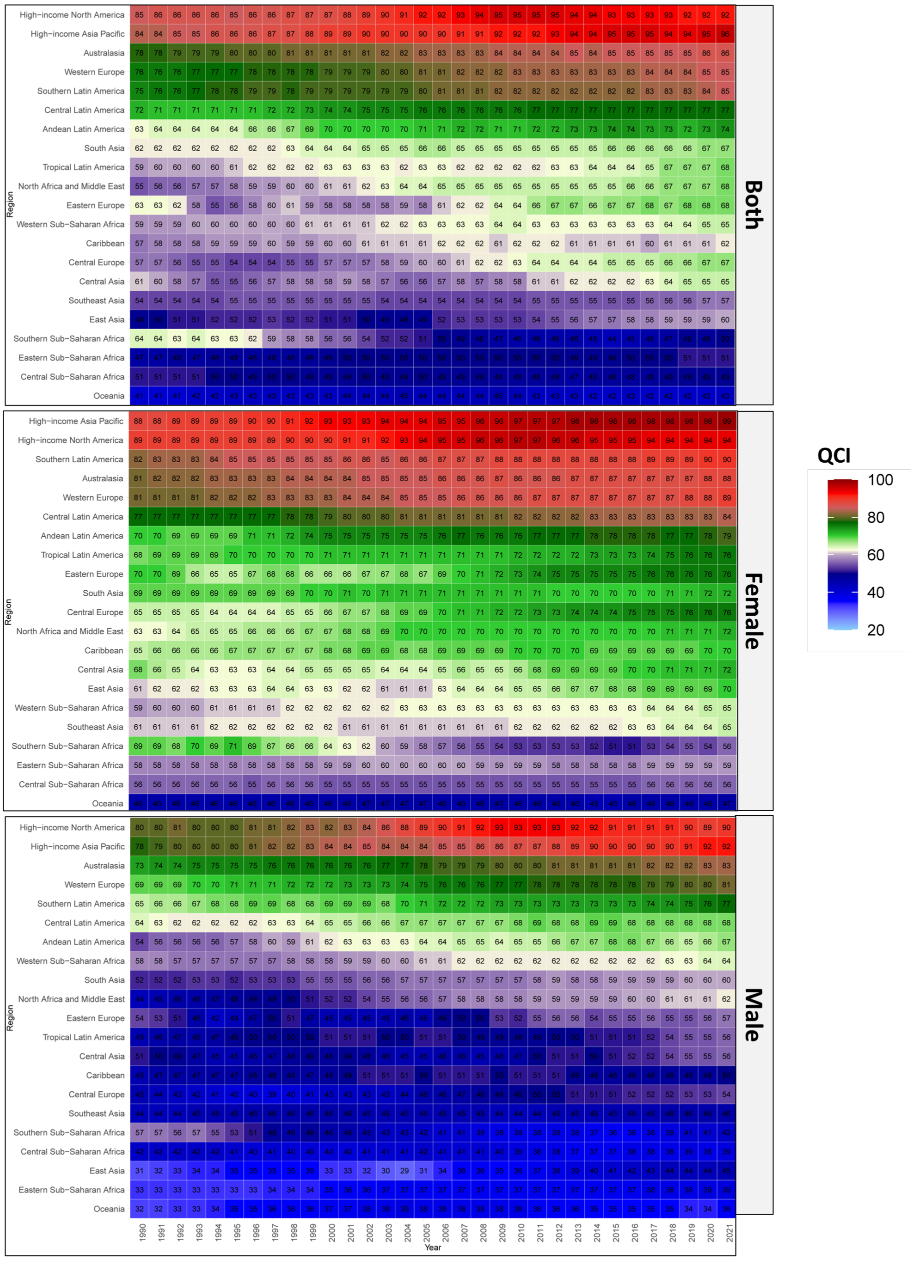

S2 Fig. Ranking of the 21 GBD regions based on age-standardized QCI for ICH in both sexes, female, and male from 1990 to 2021. QCI, quality of care index GBD, Global Burden of Diseases, ICH, intracerebral hemorrhage

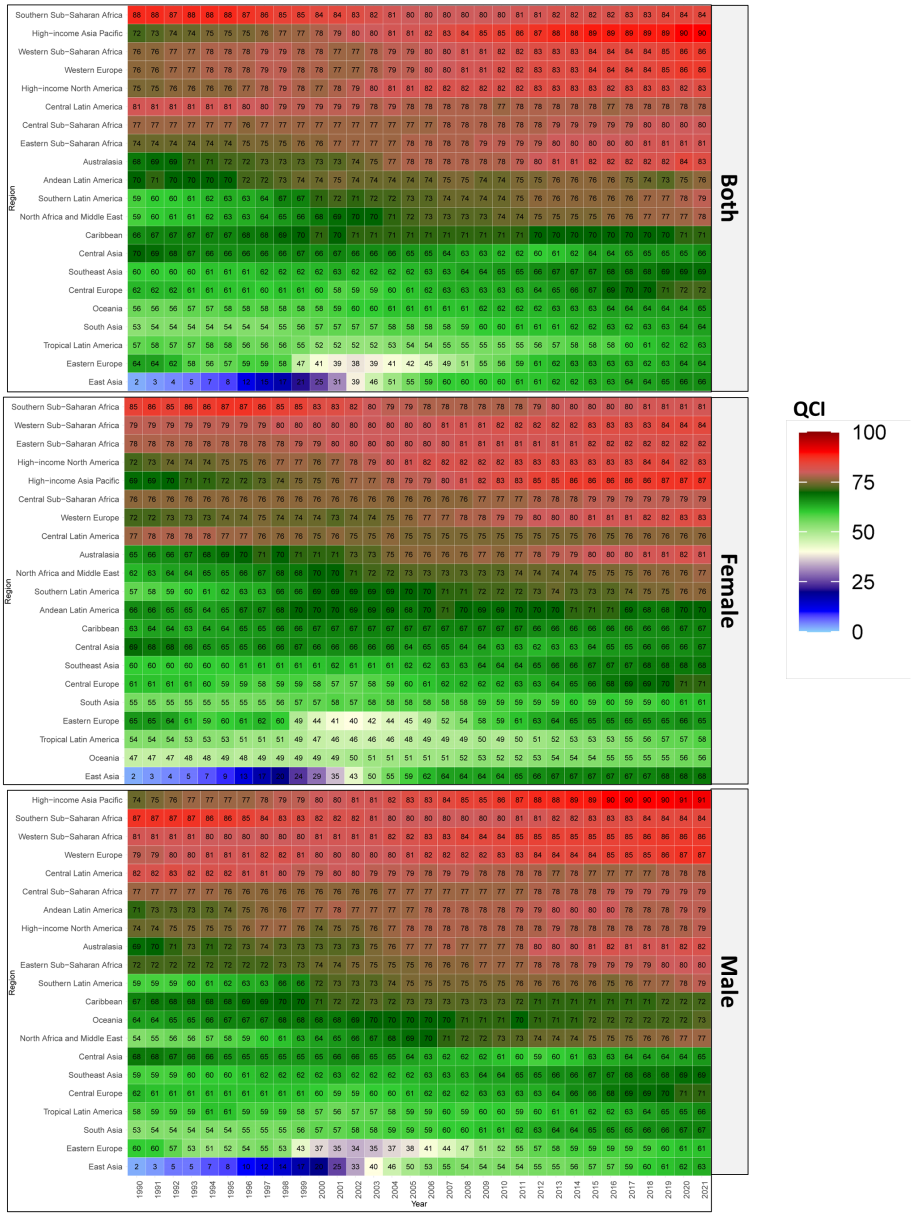

S3 Fig. Ranking of the 21 GBD regions based on age-standardized QCI for SAH in both sexes, female, and male from 1990 to 2021. QCI, quality of care index GBD, Global Burden of Diseases, SAH, subarachnoid hemorrhage

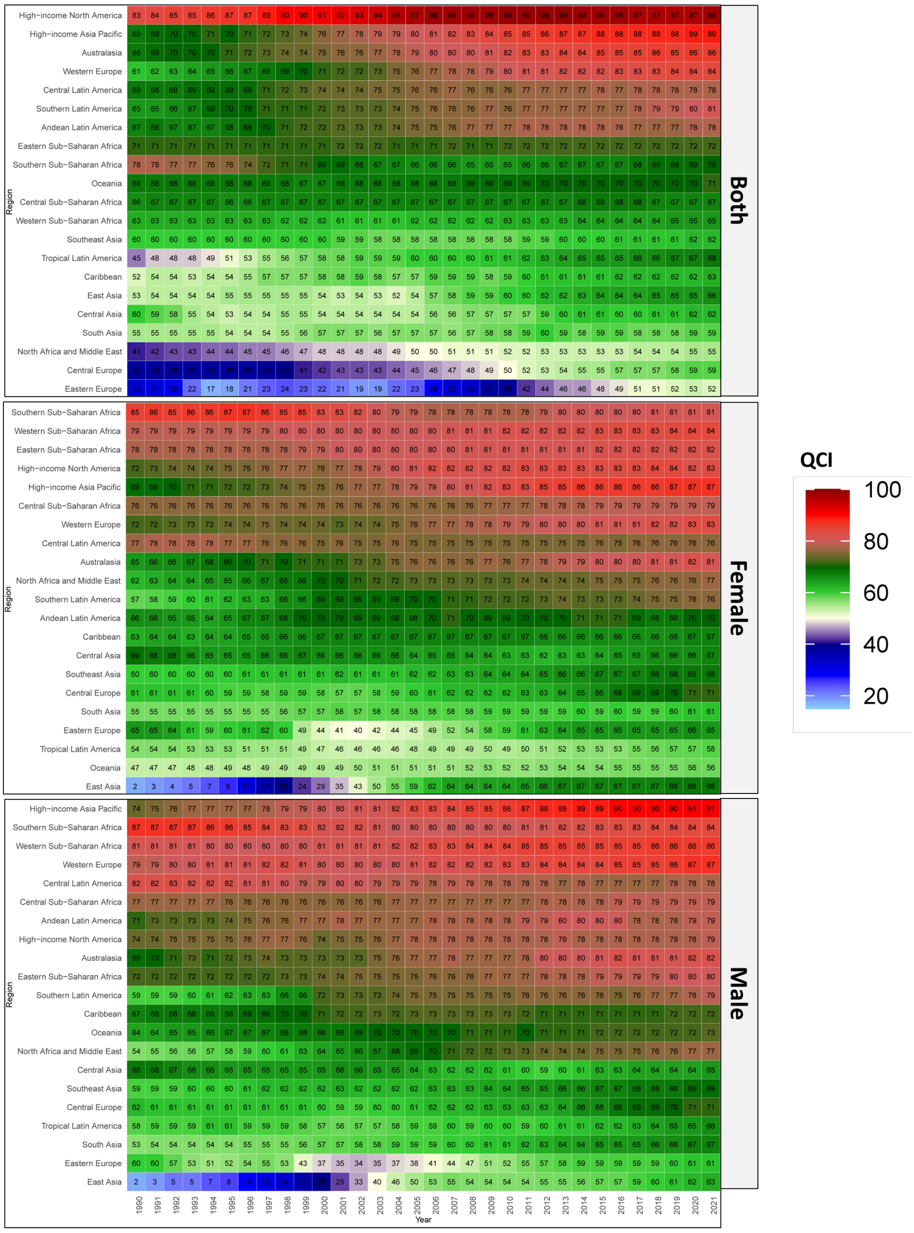

S4 Fig. Ranking of the 21 GBD regions based on age-standardized QCI for IS in both sexes, female, and male from 1990 to 2021. QCI, quality of care index GBD, Global Burden of Diseases, IS, ischemic stroke

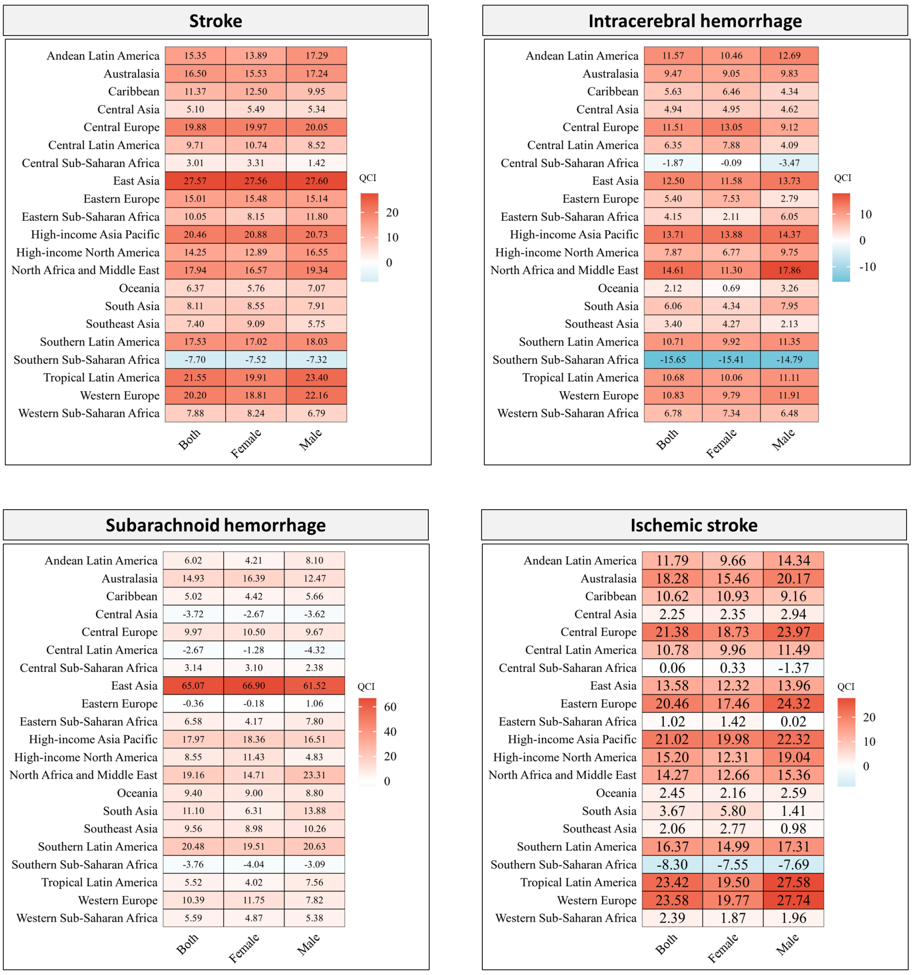

S5 Fig. Age-standardized quality of care index changes for stroke, ICH, SAH, and IS from 1990 to 2021 in 21 regions in both sexes, female, and male. ICH, intracerebral hemorrhage; SAH, subarachnoid hemorrhage; IS, ischemic stroke; QCI, quality of care index. Change represents the difference between the QCI in 2021 and the QCI in 1990

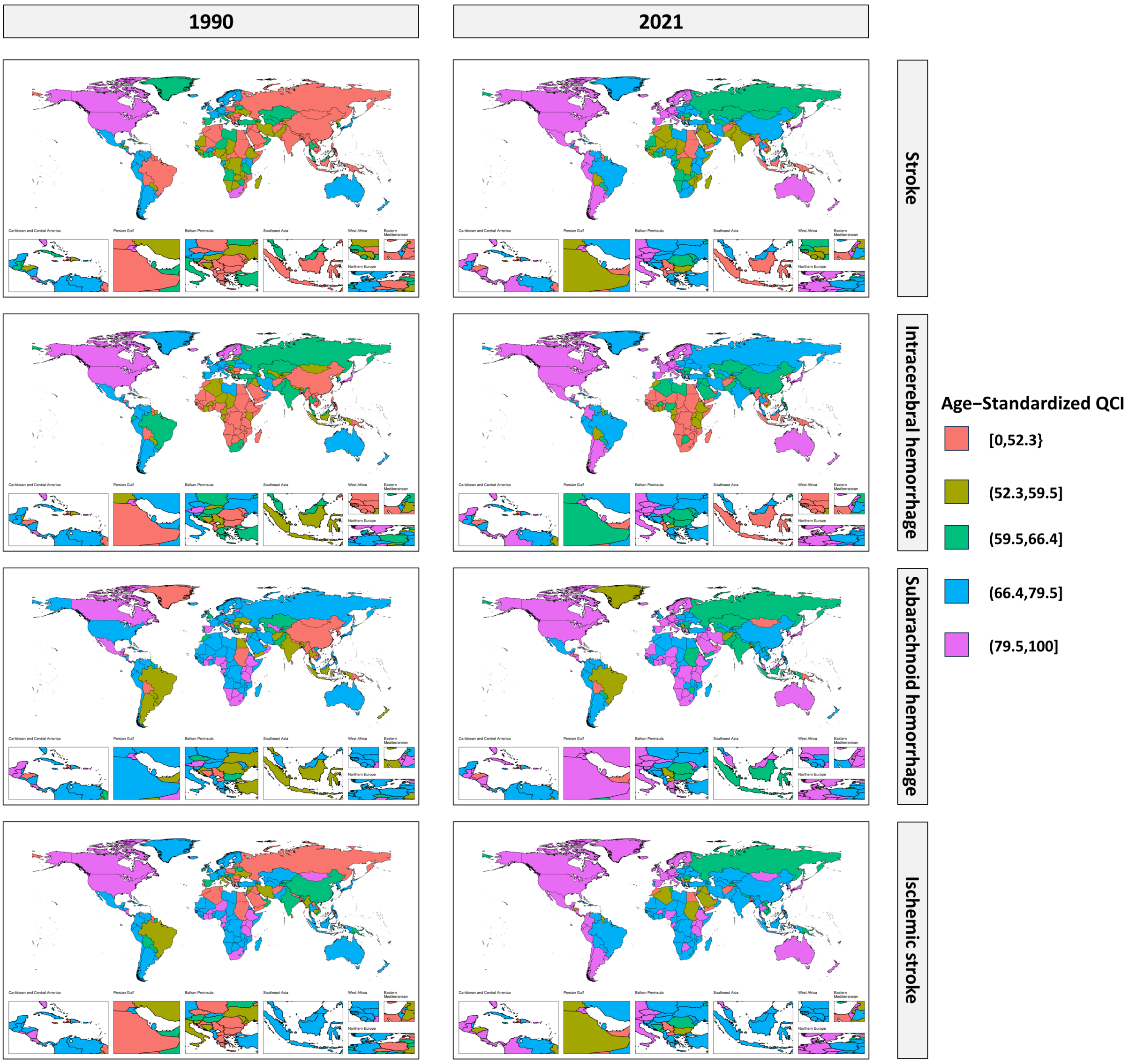

S6 Fig. Geographical distribution of age-standardized quality of care index for stroke, ICH, SAH, and IS in 1990 and 2021 for females. ICH, intracerebral hemorrhage; SAH, subarachnoid hemorrhage; IS, ischemic stroke; QCI, quality of care index

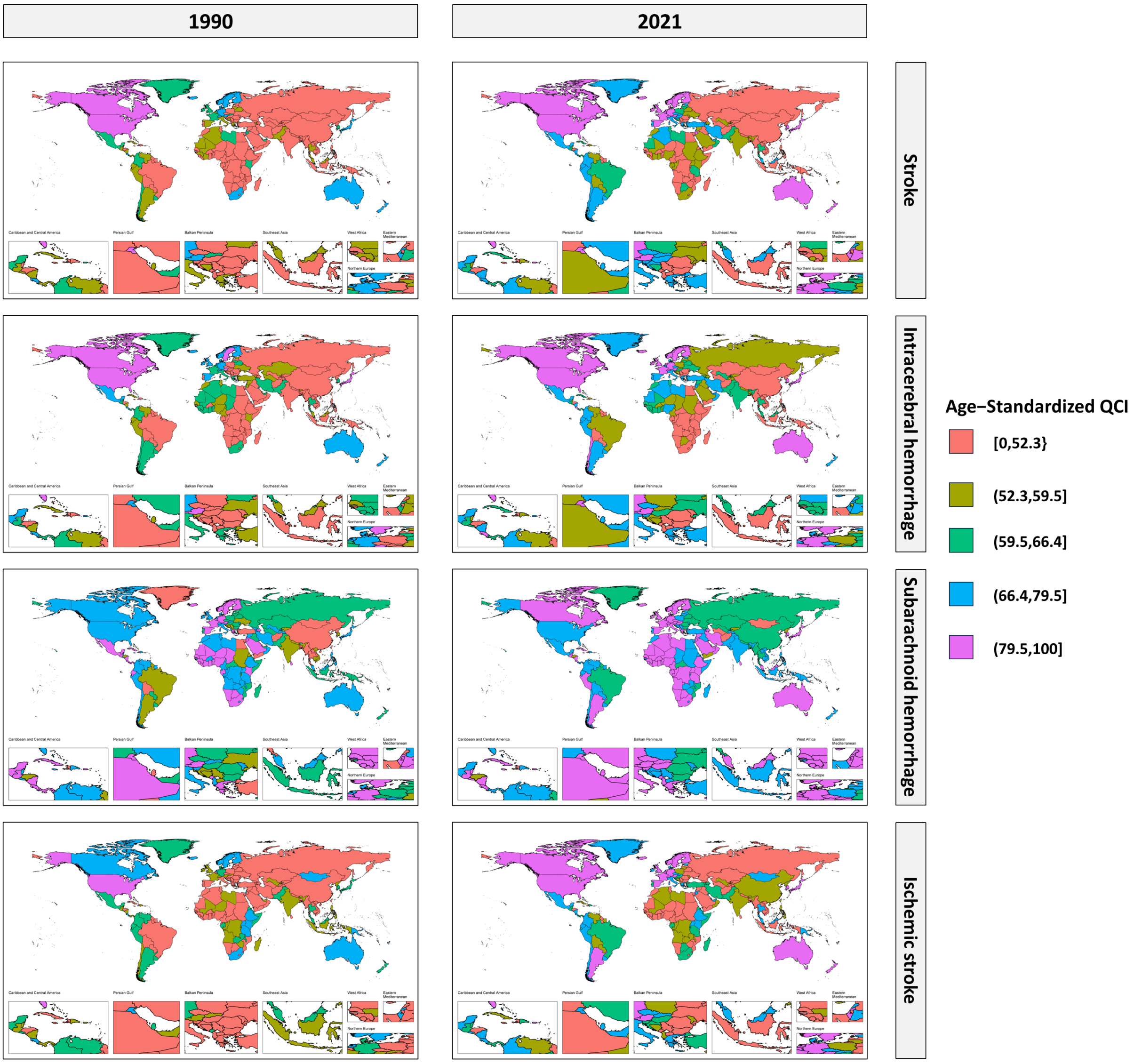

S7 Fig. Geographical distribution of age-standardized quality of care index for stroke, ICH, SAH, and IS in 1990 and 2021 for males. ICH, intracerebral hemorrhage; SAH, subarachnoid hemorrhage; IS, ischemic stroke; QCI, quality of care index

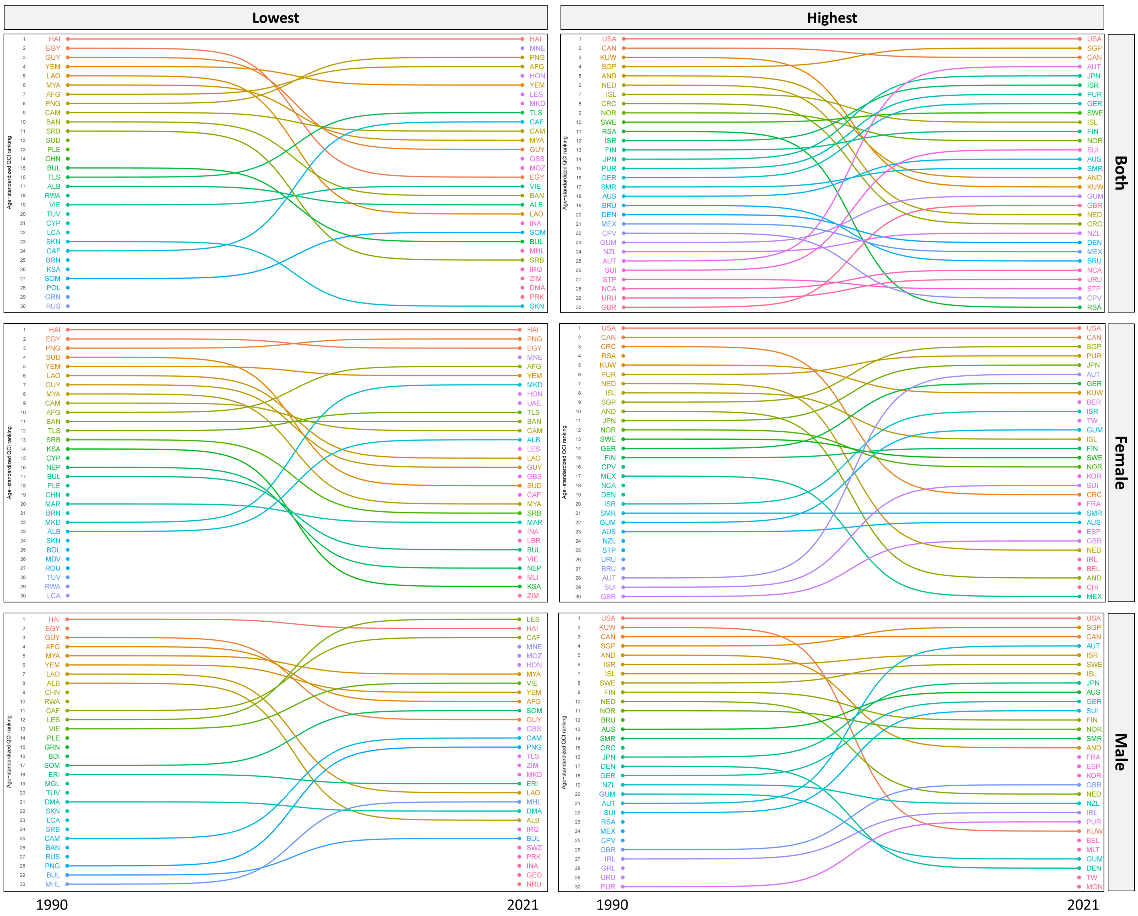

S8 Fig. The top 30 countries with the lowest and highest age-standardized quality of care index for stroke in both sexes, females, and males in 1990 and 2021. HAI, Haiti; EGY, Egypt; GUY, Guyana; YEM, Yemen; LAO, Lao People's Democratic Republic; MYA, Myanmar; AFG, Afghanistan; PNG, Papua New Guinea; CAM, Cambodia; BAN, Bangladesh; SRB, Serbia; SUD, Sudan; PLE, Palestine; CHN, China; BUL, Bulgaria; TLS, Timor-Leste; ALB, Albania; RWA, Rwanda; VIE, Viet Nam; TUV, Tuvalu; CYP, Cyprus; LCA, Saint Lucia; SKN, Saint Kitts and Nevis; CAF, Central African Republic; BRN, Bahrain; KSA, Saudi Arabia; SOM, Somalia; POL, Poland; GRN, Grenada; RUS, Russian Federation; NEP, Nepal; MAR, Morocco; MKD, North Macedonia; BOL, Bolivia (Plurinational State of); MDV, Maldives; ROU, Romania; LES, Lesotho; BDI, Burundi; ERI, Eritrea; MGL, Mongolia; DMA, Dominica; MHL, Marshall Islands; MNE, Montenegro; HDN, Honduras; GBS, Guinea-Bissau; MOZ, Mozambique; INA, Indonesia; IRQ, Iraq; ZIM, Zimbabwe; PRK, Democratic People's Republic of Korea; UAE, United Arab Emirates; LBR, Liberia; MLI, Mali; SWZ, Eswatini; GEO, Georgia; NRU, Nauru; USA, United States of America; CAN, Canada; KUW, Kuwait; SGP, Singapore; AND, Andorra; NED, Netherlands; ISL, Iceland; CRC, Costa Rica; NOR, Norway; SWE, Sweden; RSA, South Africa; ISR, Israel; FIN, Finland; JPN, Japan; PUR, Puerto Rico; GER, Germany; SMR, San Marino; AUS, Australia; BRU, Brunei Darussalam; DEN, Denmark; MEX, Mexico; CPV, Cabo Verde; GUM, Guam; NZL, New Zealand; AUT, Austria; SUI, Switzerland; STP, Sao Tome and Principe; NCA, Nicaragua; URU, Uruguay; GBR, United

Kingdom; IRL, Ireland; GRL, Greenland; BER, Bermuda; TW, Taiwan (Province of China); KOR, Republic of Korea; FRA, France; ESP, Spain; BEL, Belgium; CHI, Chile; MLT, Malta; MON, Monaco; QCI, quality of care index

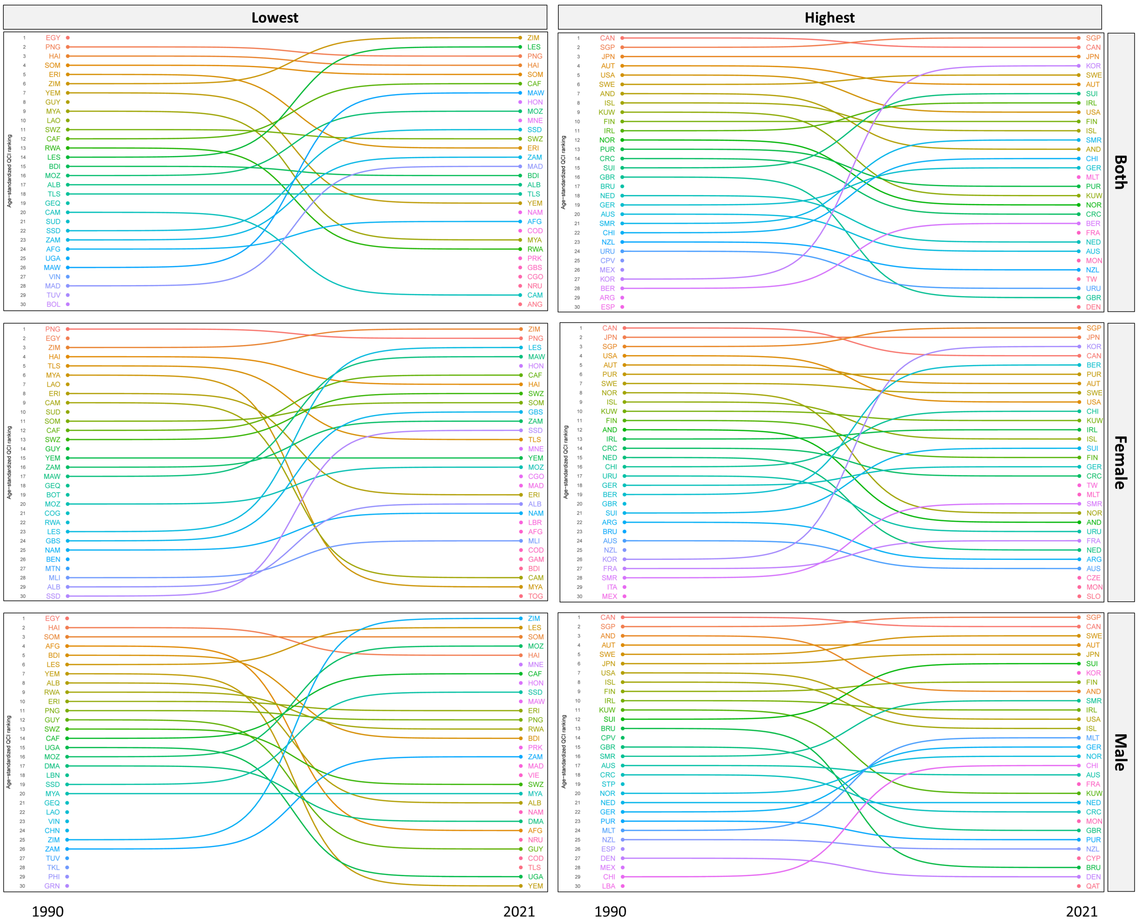

S9 Fig. The top 30 countries with the lowest and highest age-standardized quality of care index for ICH in both sexes, females, and males in 1990 and 2021. EGY, Egypt; PNG, Papua New Guinea; HAI, Haiti; SOM, Somalia; ERI, Eritrea; ZIM, Zimbabwe; YEM, Yemen; GUY, Guyana; MYA, Myanmar; LAO, Lao People's Democratic Republic; SWZ, Eswatini; CAF, Central African Republic; RWA, Rwanda; LES, Lesotho; BDI, Burundi; MOZ, Mozambique; ALB, Albania; TLS, Timor-Leste; GEQ, Equatorial Guinea; CAM, Cambodia; SUD, Sudan; SSD, South Sudan; ZAM, Zambia; AFG, Afghanistan; UGA, Uganda; MAW, Malawi; VIN, Saint Vincent and the Grenadines; MAD, Madagascar; TUV, Tuvalu; BOL, Bolivia (Plurinational State of); BOT, Botswana; CGO, Congo; GBS, Guinea-Bissau; NAM, Namibia; BEN, Benin; MTN, Mauritania; MLI, Mali; DMA, Dominica; LBN, Lebanon; CHN, China; TKL, Tokelau; PHI, Philippines; GRN, Grenada; HON, Honduras; MNE, Montenegro; COD, Democratic Republic of the Congo; PRK, Democratic People's Republic of Korea; NRU, Nauru; ANG, Angola; LBR, Liberia; GAM, Gambia; TOG, Togo; VIE, Viet Nam; CAN, Canada; SGP, Singapore; JPN, Japan; AUT, Austria; USA, United States of America; SWE, Sweden; AND, Andorra; ISL, Iceland; KUW, Kuwait; FIN, Finland; IRL,Ireland; NOR, Norway; PUR, Puerto Rico; CRC, Costa Rica; SUI,

Switzerland; GBR, United Kingdom; BRU, Brunei Darussalam; NED, Netherlands; GER, Germany; AUS, Australia; SMR, San Marino; CHI, Chile; NZL, New Zealand; URU, Uruguay; CPV, Cabo Verde; MEX, Mexico; KOR, Republic of Korea; BER, Bermuda; ARG, Argentina; ESP, Spain; FRA, France; ITA, Italy; STP, Sao Tome and Principe; MLT, Malta; DEN, Denmark; LBA, Libya; MON, Monaco; TW, Taiwan (Province of China); ICH, intracerebral hemorrhage; QCI, quality of care index

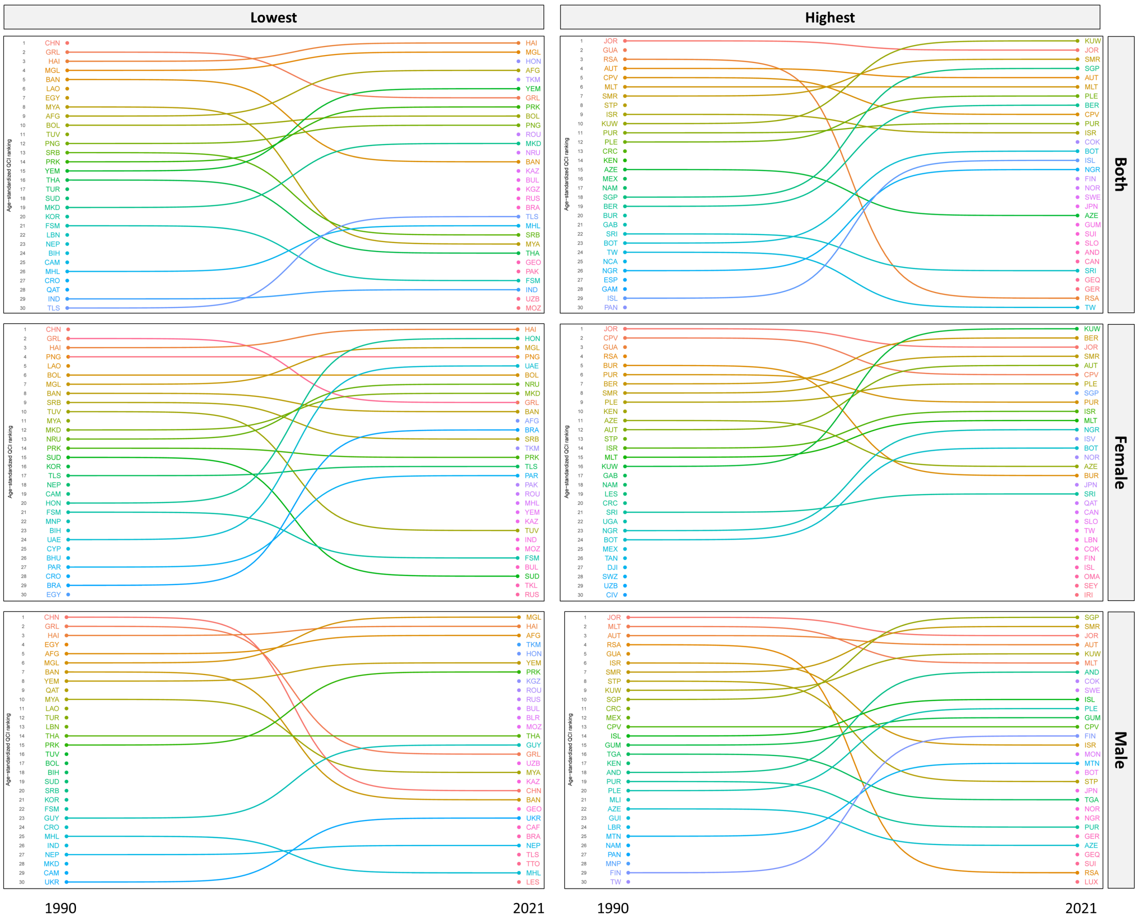

S10 Fig. The top 30 countries with the lowest and highest age-standardized quality of care index for SAH in both sexes, females, and males in 1990 and 2021. CHN, China; GRL, Greenland; HAI, Haiti; MGL, Mongolia; BAN, Bangladesh; LAO, Lao People's Democratic Republic; EGY, Egypt; MYA, Myanmar; AFG, Afghanistan; BOL, Bolivia (Plurinational State of); TUV, Tuvalu; PNG, Papua New Guinea; SRB, Serbia; PRK, Democratic People's Republic of Korea; YEM, Yemen; THA, Thailand; TUR, Turkey; SUD, Sudan; MKD, North Macedonia; KOR, Republic of Korea; FSM, Micronesia (Federated States of); LBN, Lebanon; NEP, Nepal; BIH, Bosnia and Herzegovina; CAM, Cambodia; MHL, Marshall Islands; CRO, Croatia; QAT, Qatar; IND, India; TLS, Timor-Leste; NRU, Nauru; HON, Honduras; MNP, Northern Mariana Islands; UAE, United Arab Emirates; CYP, Cyprus; BHU, Bhutan; PAR, Paraguay; BRA, Brazil; GUY, Guyana; UKR, Ukraine; TKM, Turkmenistan; ROU, Romania; KAZ, Kazakhstan; BUL, Bulgaria; KGZ, Kyrgyzstan; RUS, Russian Federation; GEO, Georgia; PAK, Pakistan; UZB, Uzbekistan; INA, Indonesia; TKL,

Tokelau; BLR, Belarus; MOZ, Mozambique; CAF, Central African Republic; TTO, Trinidad and Tobago; LES, Lesotho; JOR, Jordan; GUA, Guatemala; RSA, South Africa; AUT, Austria; CPV, Cabo Verde; MLT, Malta; SMR, San Marino; STP, Sao Tome and Principe; ISR, Israel; KUW, Kuwait; PUR, Puerto Rico; PLE, Palestine; CRC, Costa Rica; KEN, Kenya; AZE, Azerbaijan; MEX, Mexico; NAM, Namibia; SGP, Singapore; BER, Bermuda; BUR, Burkina Faso; GAB, Gabon; SRI, Sri Lanka; BOT, Botswana; TW, Taiwan (Province of China); NCA, Nicaragua; NGR, Nigeria; ESP, Spain; GAM, Gambia; ISL, Iceland; PAN, Panama; ; LES, Lesotho; UGA, Uganda; TAN, United Republic of Tanzania; DJI, Djibouti; SWZ, Eswatini; UZB, Uzbekistan; CIV, Coted'Ivoire; GUM, Guam; TGA, Tonga; AND, Andorra; MLI, Mali; GEQ, Guinea; LBR, Liberia; MTN, Mauritania; MNP, Northern Mariana Islands; FIN, Finland; COK, Cook Islands; NOR, Norway; SWE, Sweden; JPN, Japan; Switzerland; SLO, Slovenia; CAN, Canada; GEQ, Equatorial Guinea; GER, Germany; ISV, United States Virgin Islands; QAT, Qatar; LB, Lebanon; OMA, Oman; SEY, Seychelles; IRI , Iran (Islamic Republic of); MON, Monaco; LUX, Luxembourg; SAH, subarachnoid hemorrhage; QCI, quality of care index

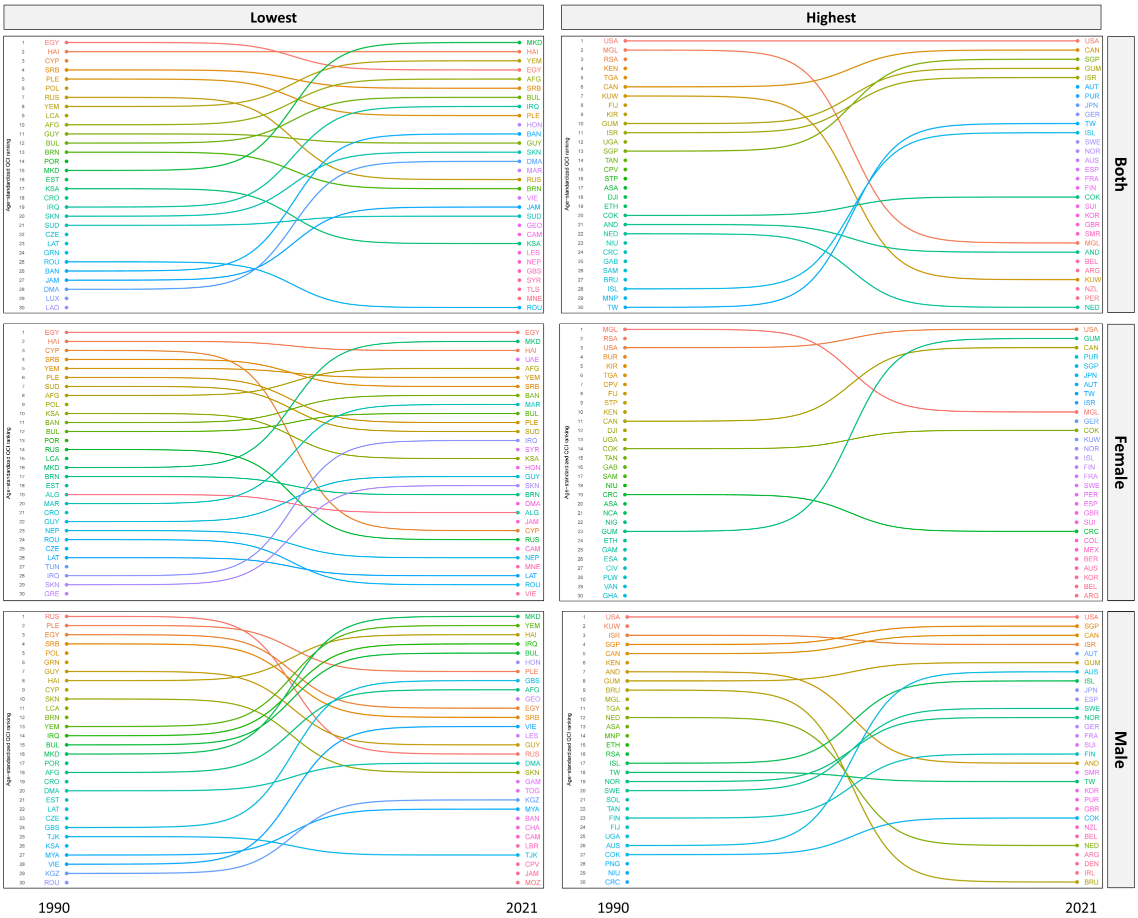

S11 Fig. The top 30 countries with the lowest and highest age-standardized quality of care index for IS in both sexes, females, and males in 1990 and 2021. EGY, Egypt; HAI, Haiti; CYP, Cyprus; SRB, Serbia; PLE, Palestine; POL, Poland; RUS, Russian Federation; YEM, Yemen; LCA, Saint Lucia; AFG, Afghanistan; GUY, Guyana; BUL,

Bulgaria; BRN, Bahrain; POR, Portugal; MKD, North Macedonia; EST, Estonia; KSA, Saudi Arabia; CRO, Croatia; IRQ, Iraq; SKN, Saint Kitts and Nevis; SUD, Sudan; CZE, Czechia; LAT, Latvia; GRN, Grenada; ROU, Romania; BAN, Bangladesh; JAM, Jamaica; DMA, Dominica; LUX, Luxembourg; LAO, Lao People's Democratic Republic; ALG, Algeria; MAR, Morocco; NEP, Nepal; TUN, Tunisia; GRE, Greece; GBS, Guinea-Bissau; TJK, Tajikistan; MYA, Myanmar; VIE, Viet Nam; KGZ, Kyrgyzstan; HON, Honduras; GEO, Georgia; CAM, Cambodia; LES, Lesotho; SYR, Syrian Arab Republic; TLS, Timor-Leste; MNE, Montenegro; UAE, United Arab Emirates; GAM, Gambia; TOG, Togo; CHA, Chad; LBR, Liberia; CVP, Cabo Verde; MOZ, Mozambique; USA, United States of America; MGL, Mongolia; RSV, South Africa; KEN, Kenya; TGA, Tonga; CAN, Canada; KUW, Kuwait; FIJ, Fiji; KIR, Kiribati; GUM, Guam; ISR, Israel; UGA, Uganda; SGP, Singapore; TAN, United Republic of Tanzania; CPV, Cabo Verde; STP, Sao Tome and Principe; ASA, American Samoa; DJI, Djibouti; ETH, Ethiopia; COK, Cook Islands; AND, Andorra; NED, Netherlands; NIU, Niue; CRC, Costa Rica; GAB, Gabon; SAM, Samoa; BRU, Brunei Darussalam; ISL, Iceland; MNP, Northern Mariana Islands; TW, Taiwan (Province of China); ; BUR, Burkina Faso; NCA, Nicaragua; NIG, Niger; GAM, Gambia; ESA, El Salvador; CIV, Coted'Ivoire; PLW, Palau;VAN, Vanuatu; GHA, Ghana; NOR, Norway; SWE, Sweden; SOL, Solomon Islands; FIN, Finland; AUS, Australia; PNG, Papua New Guinea; AUT, Austria; PUR, Puerto Rico; JPN, Japan; GER, Germany; ESP, Spain; FRA, France; SUI, Switzerland; KOR, Republic of Korea; GBR, United Kingdom; SMR, San Marino; BEL, Belgium; ARG, Argentina; NZL, New Zealand; PER, Peru; COL, Colombia; MEX, Mexico; BER, Bermuda; DEN, Denmark; IRL, Ireland; IS, ischemic stroke; QCI, quality of care index

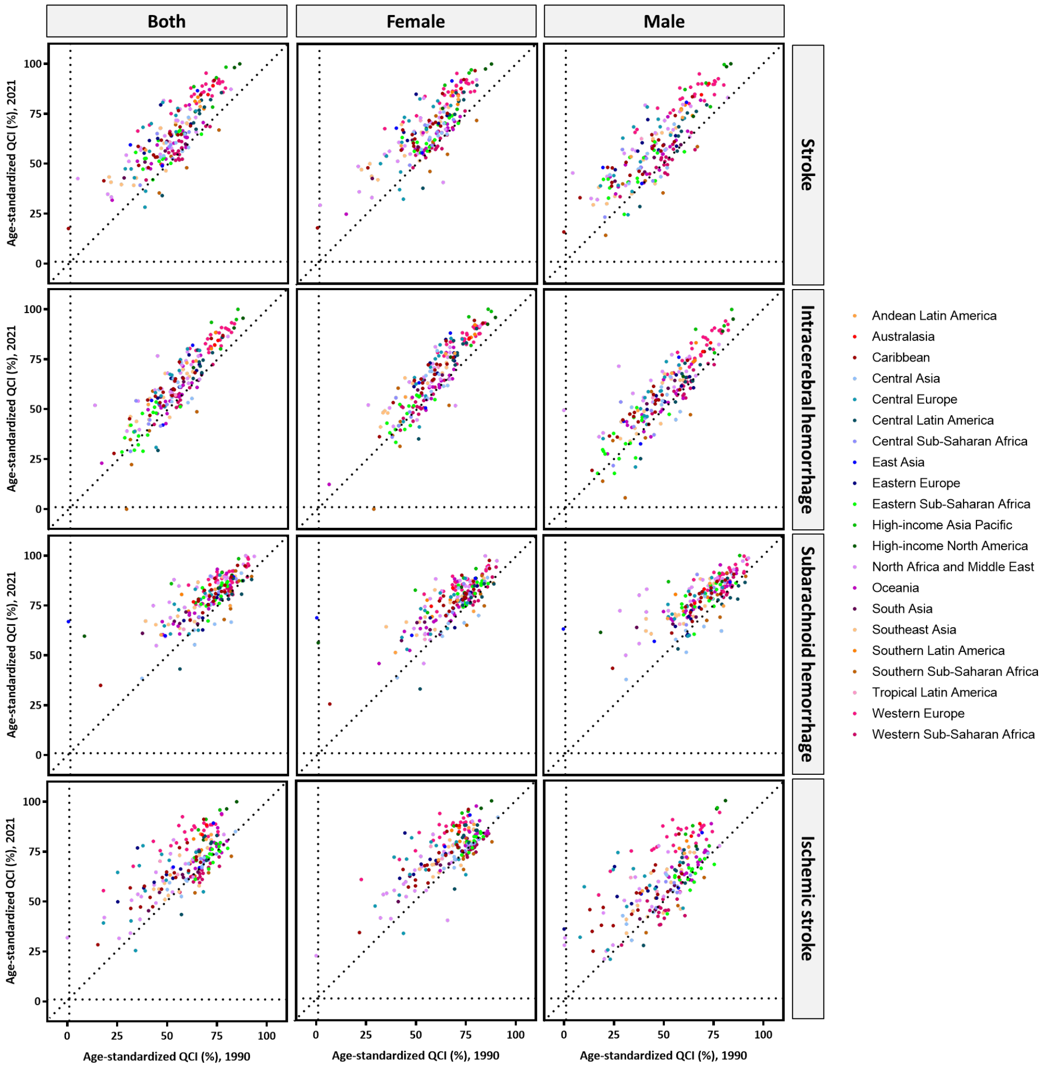

S12 Fig. Age-standardized QCI in 1990 and 2021 in different countries according to the 21 GBD regions values in males, females, and both genders for stroke, ICH, SAH, and IS (each point represents a country color-coded based on the 21 GBD regions). ICH, intracerebral hemorrhage; SAH, subarachnoid hemorrhage; IS, ischemic stroke; SDI, socio-demographic index; QCI, quality of care index

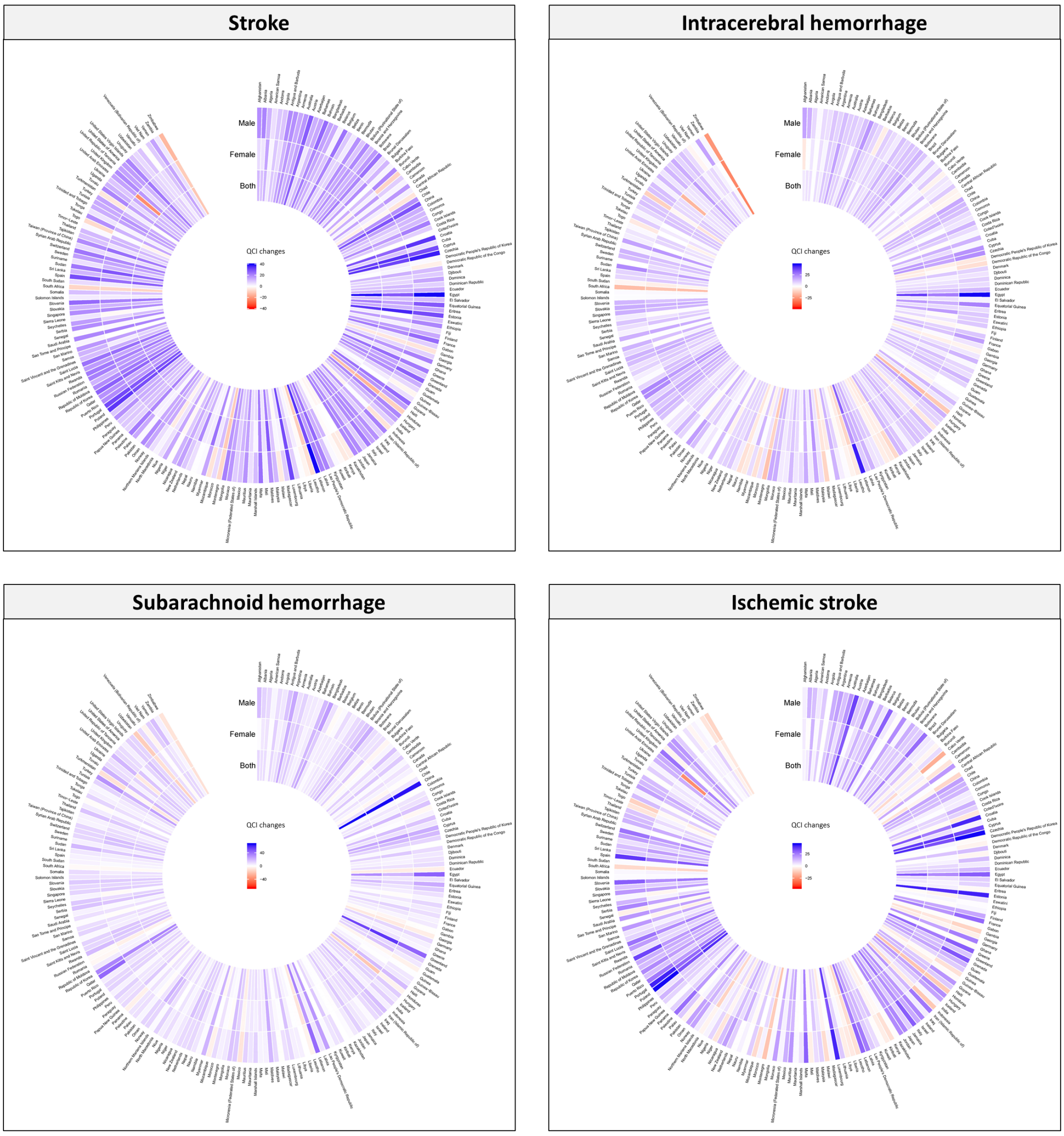

S13 Fig. Age-standardized quality of care index changes for stroke, ICH, SAH, and IS between 1990 and 2021 for both sexes, females, and males. ICH, intracerebral hemorrhage; SAH, subarachnoid hemorrhage; IS, ischemic stroke; QCI, quality of care index. Change represents the difference between the QCI in 2021 and the QCI in 1990

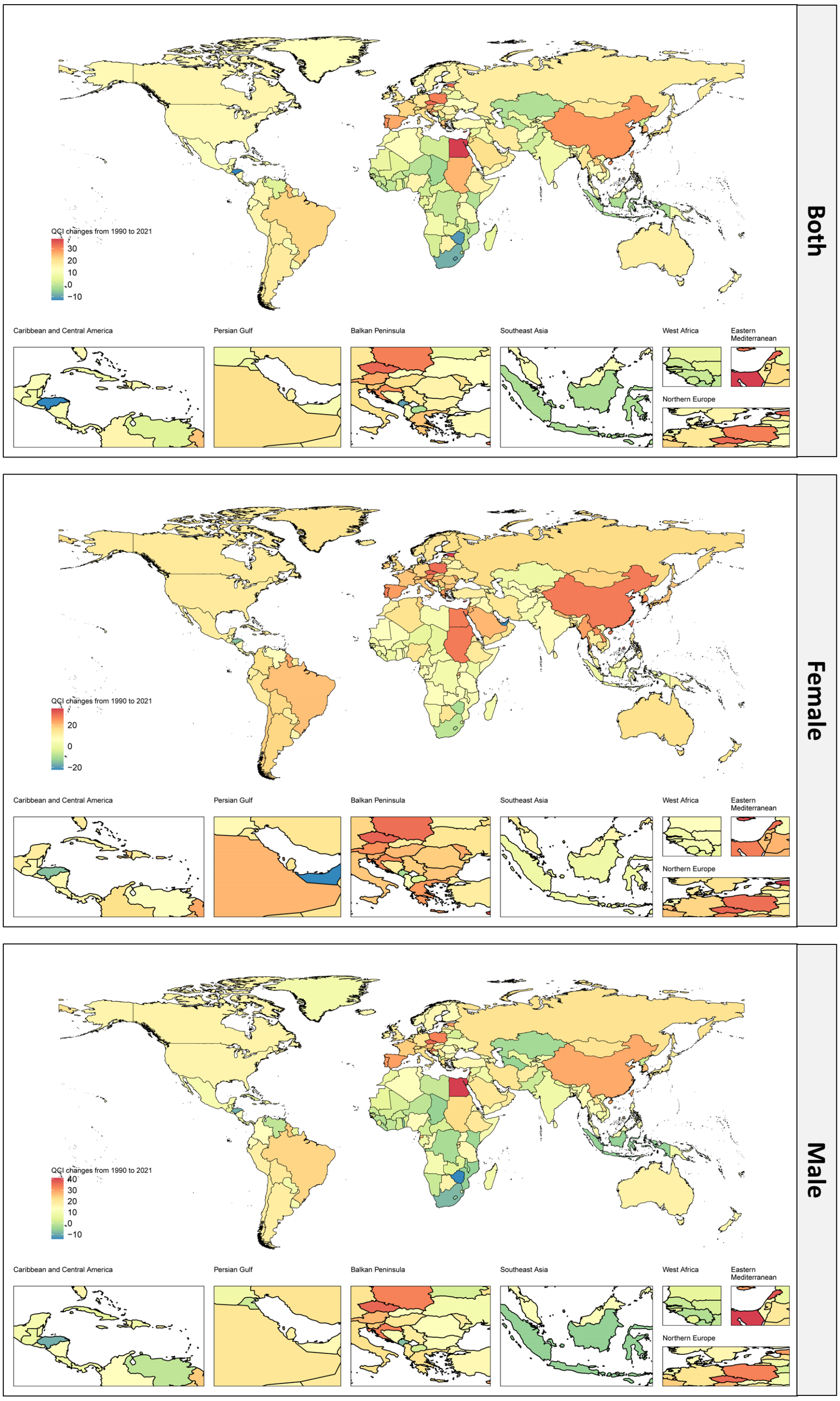

S14 Fig. Geographical distribution of age-standardized quality of care index changes for stroke between 1990 and 2021 for both sexes, females, and males. QCI, quality of care index. Change represents the difference between the QCI in 2021 and the QCI in 1990

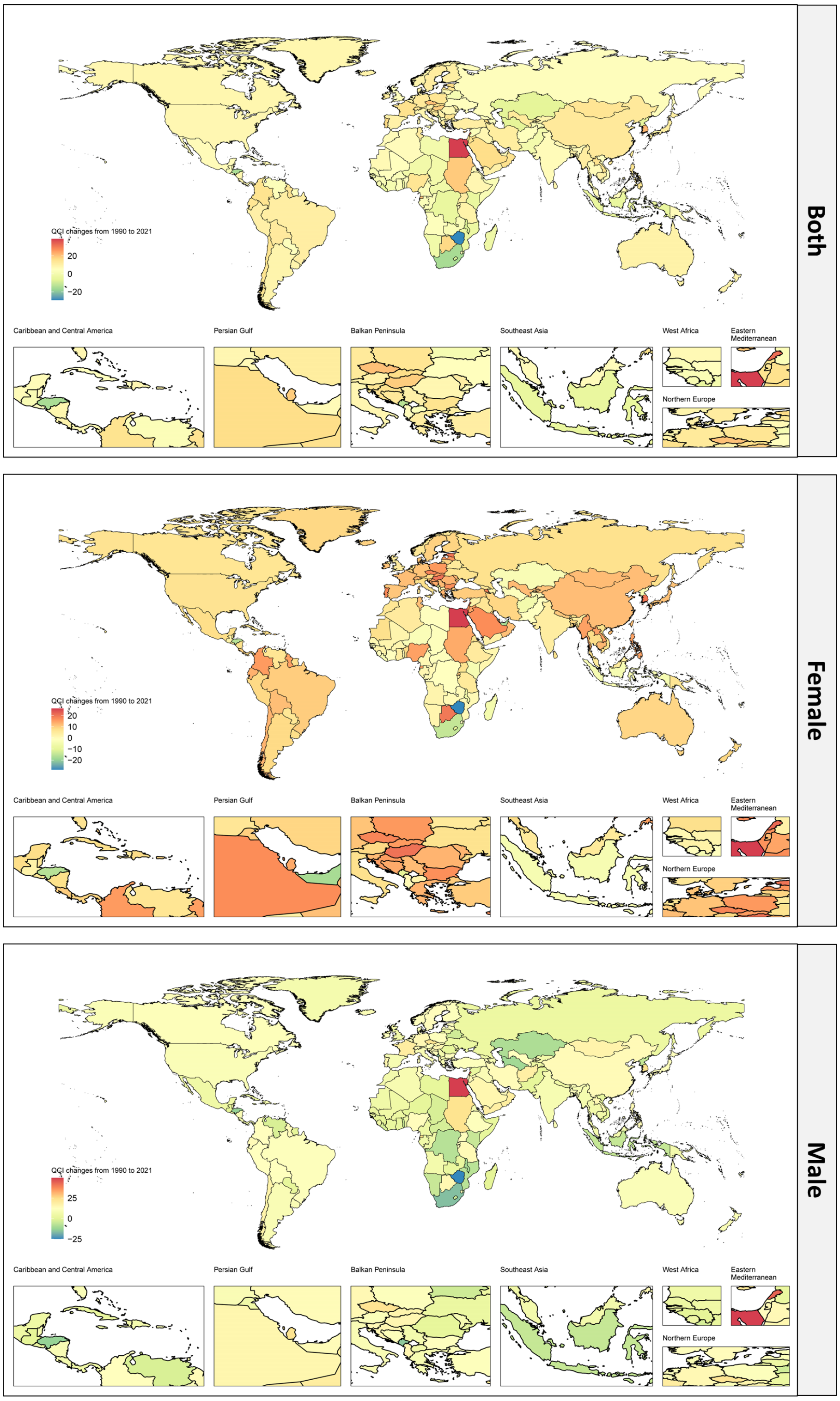

S15 Fig. Geographical distribution of age-standardized quality of care index changes for ICH between 1990 and 2021 for both sexes, females, and males. ICH, intracerebral hemorrhage; QCI, quality of care index. Change represents the difference between the QCI in 2021 and the QCI in 1990

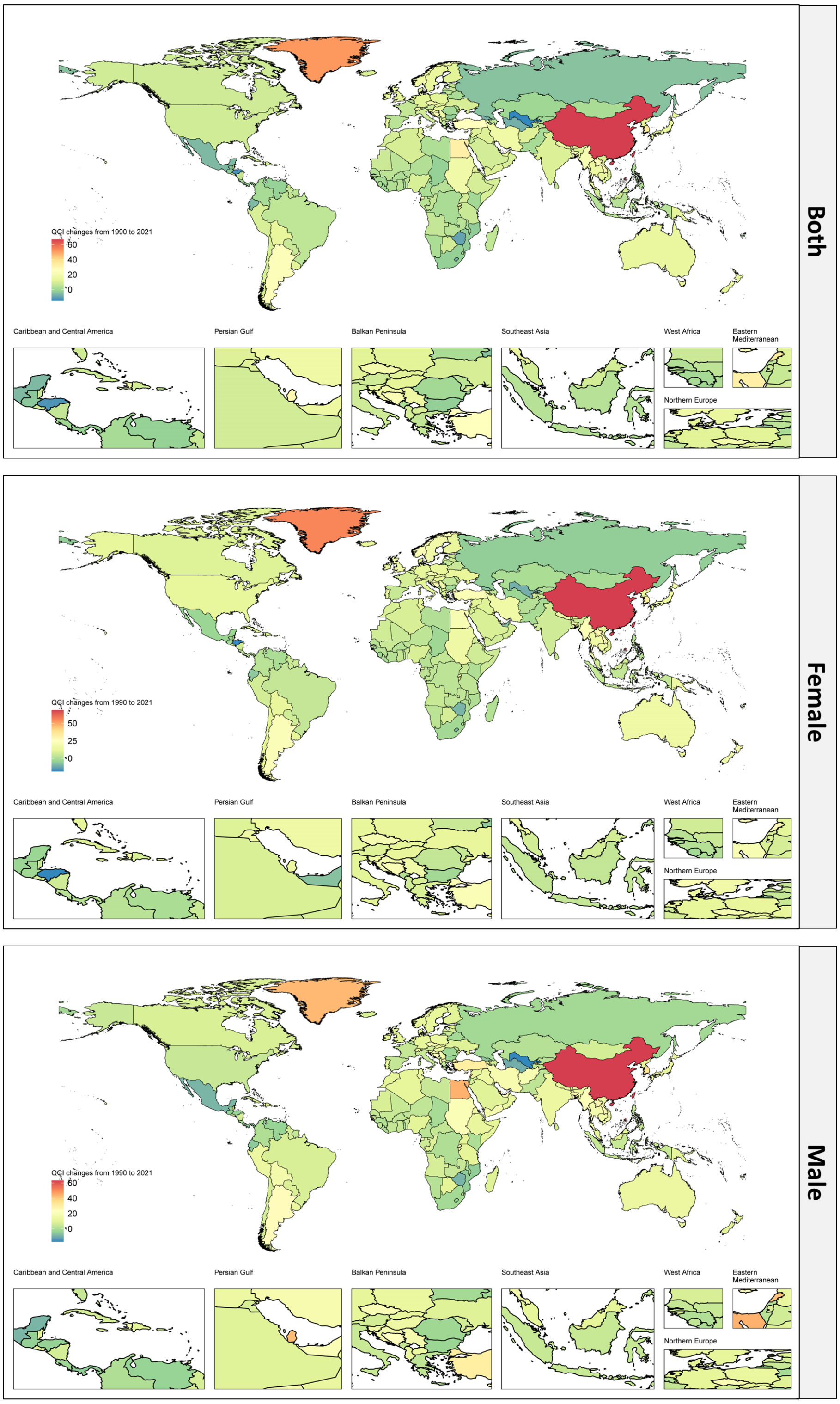

S16 Fig. Geographical distribution of age-standardized quality of care index changes for SAH between 1990 and 2021 for both sexes, females, and males. SAH, subarachnoid hemorrhage; QCI, quality of care index. Change represents the difference between the QCI in 2021 and the QCI in 1990

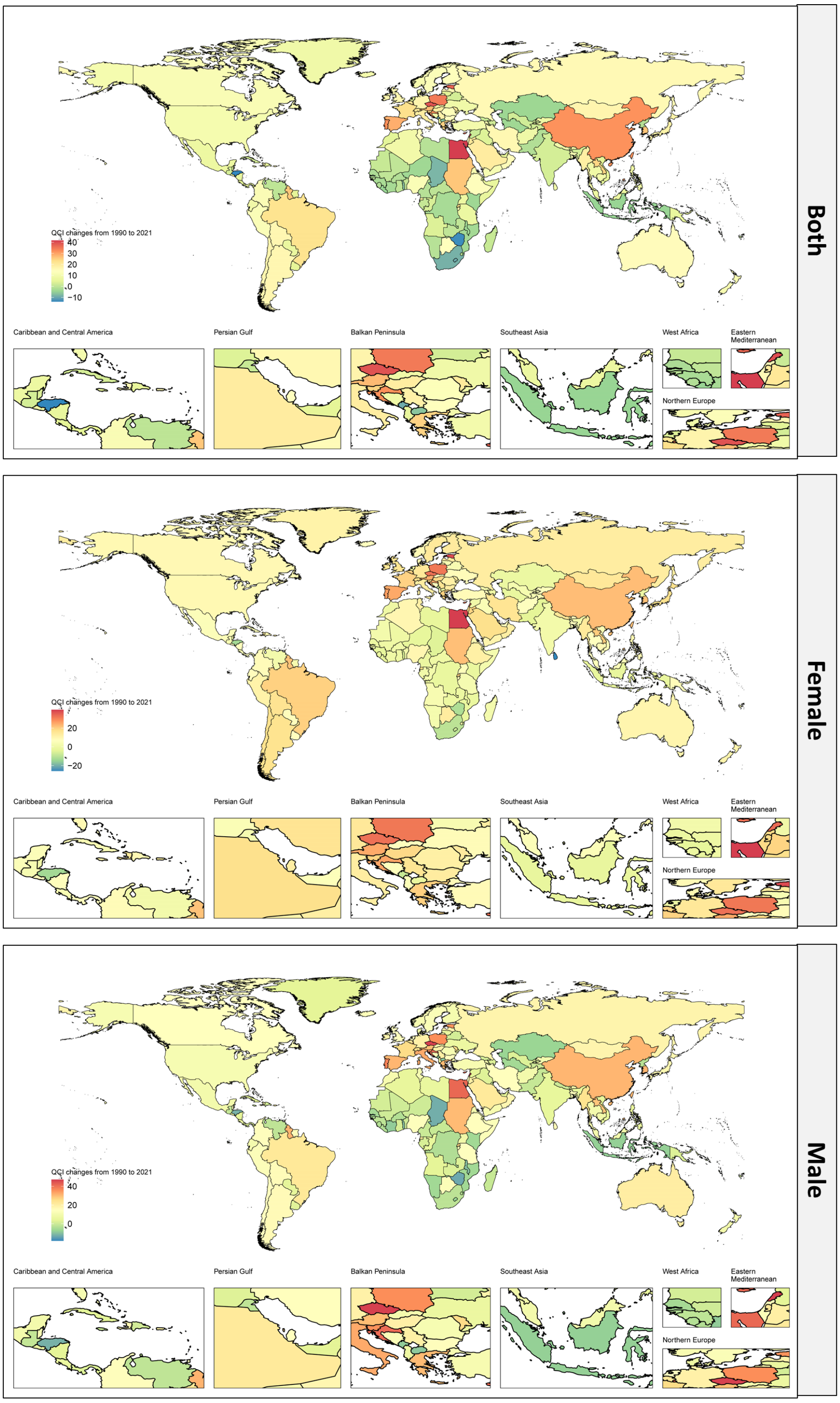

S17 Fig. Geographical distribution of age-standardized quality of care index changes for IS between 1990 and 2021 for both sexes, females, and males. IS, ischemic stroke; QCI, quality of care index. Change represents the difference between the QCI in 2021 and the QCI in 1990

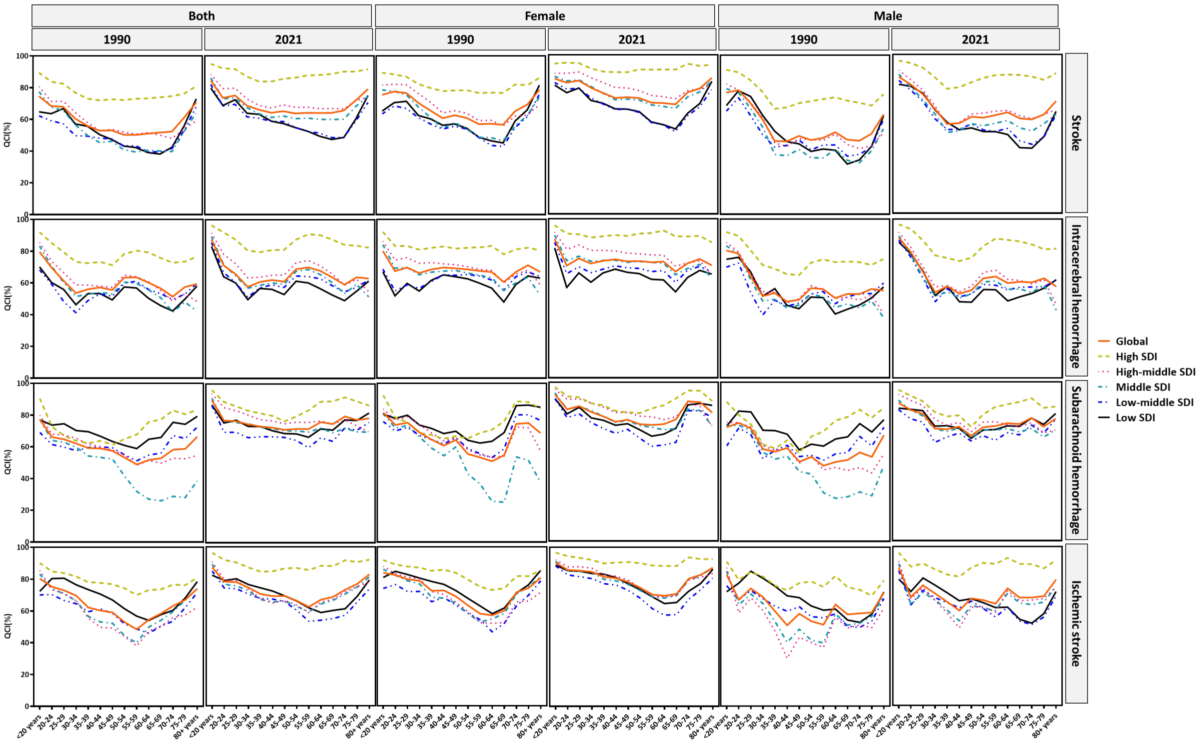

S18 Fig. Age trend of quality of care index in the global and 5 sociodemographic index in both sexes, females, and males for stroke, ICH, SAH, and IS in 1990 and 2021. ICH, intracerebral hemorrhage; SAH, subarachnoid hemorrhage; IS, ischemic stroke; SDI, socio-demographic index; QCI, quality of care index

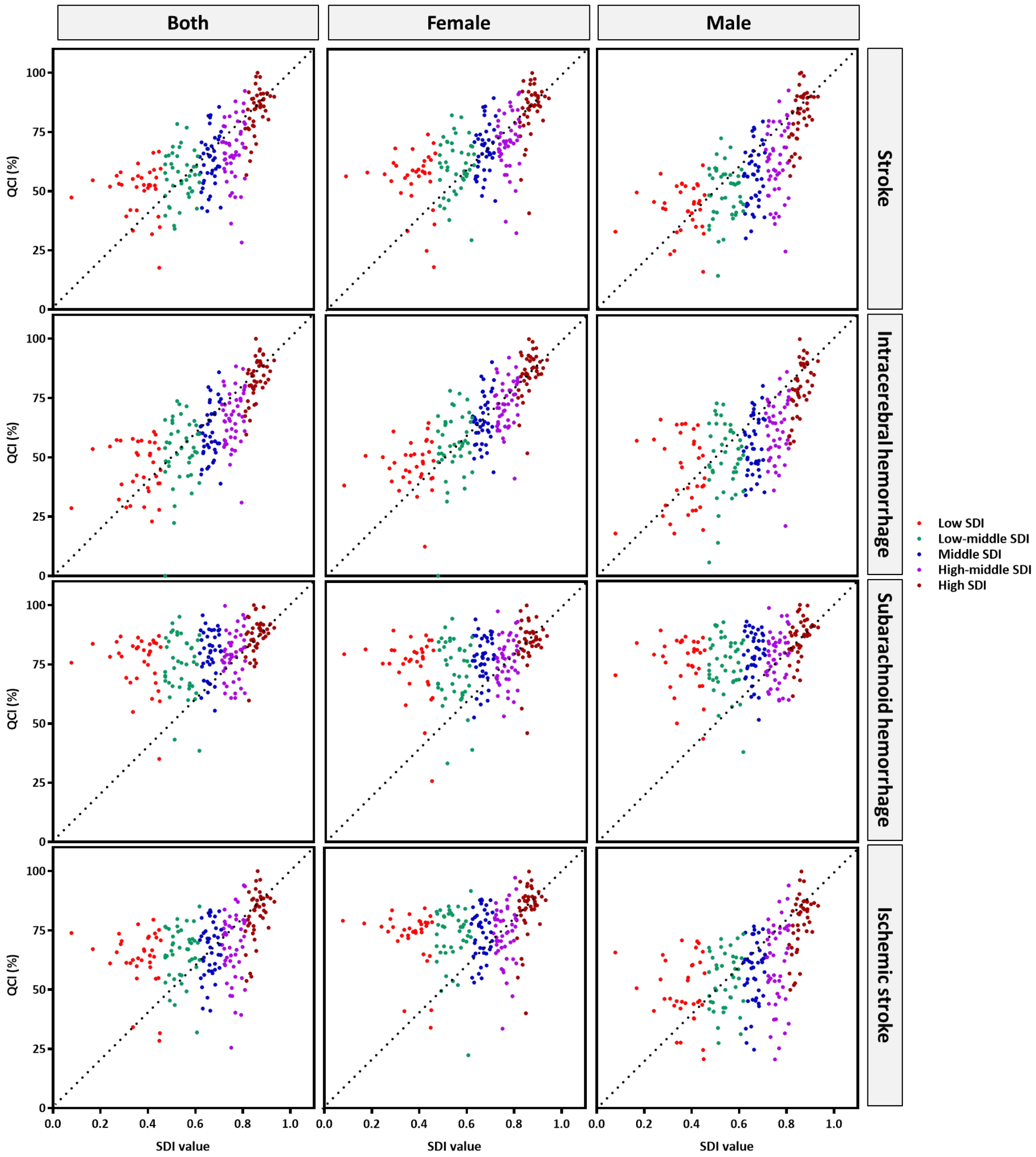

S19 Fig. Age-standardized QCI of different countries according to SDI classification for stroke, ICH, SAH, and IS in 2021 (each point represents a country color-coded based on the 5 SDI classification). ICH, intracerebral hemorrhage; SAH, subarachnoid hemorrhage; IS, ischemic stroke; SDI, QCI, quality of care index

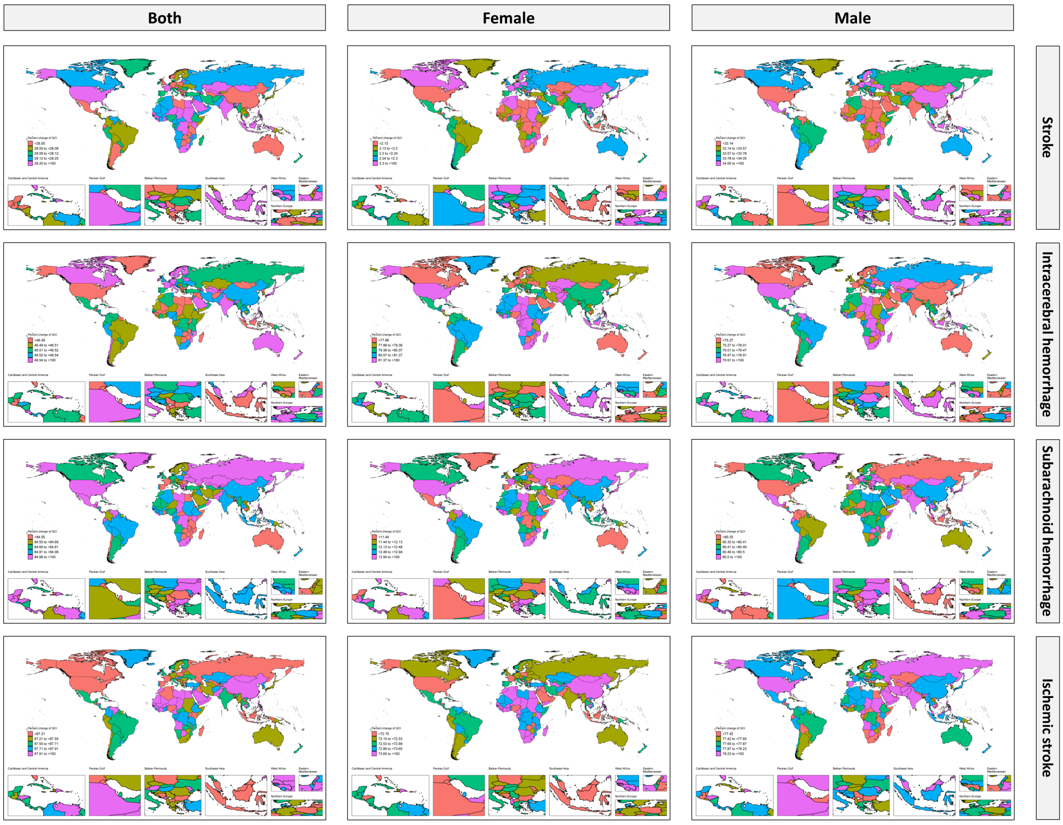

S20 Fig. Percent changes of the QCIs of the countries between 1990 and 2021 for stroke, ICH, SAH, and IS in both sexes, females, and males. ICH, intracerebral hemorrhage; SAH, subarachnoid hemorrhage; IS, ischemic stroke; QCI, quality of care index

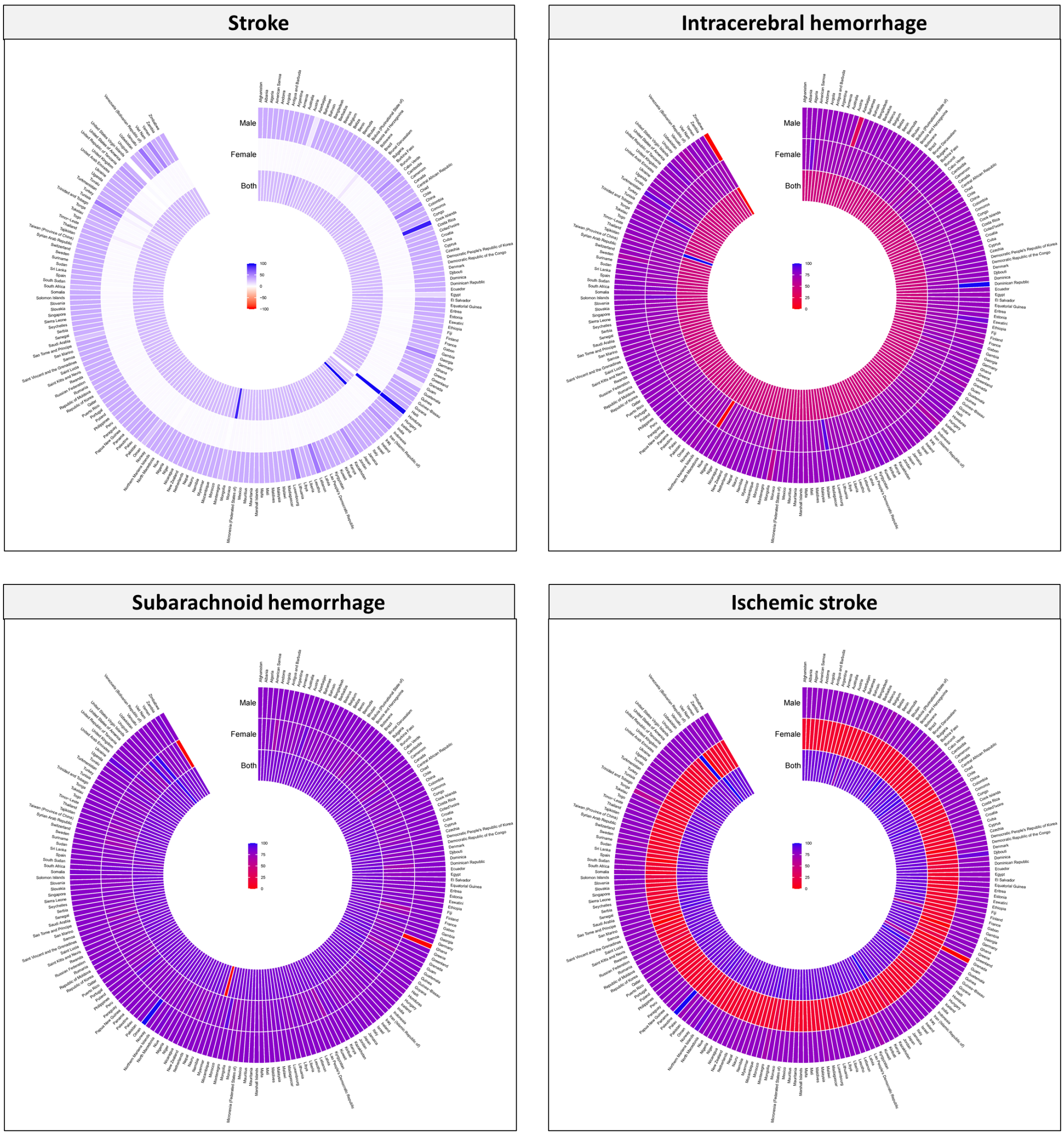

S21 Fig. Percent changes of the QCIs of the countries for stroke, ICH, SAH, and IS from 1990 to 2021 for both sexes, females, and males. ICH, intracerebral hemorrhage; SAH, subarachnoid hemorrhage; IS, ischemic stroke; QCI, quality of care index. Change represents the difference between the QCI in 2021 and the QCI in 1990

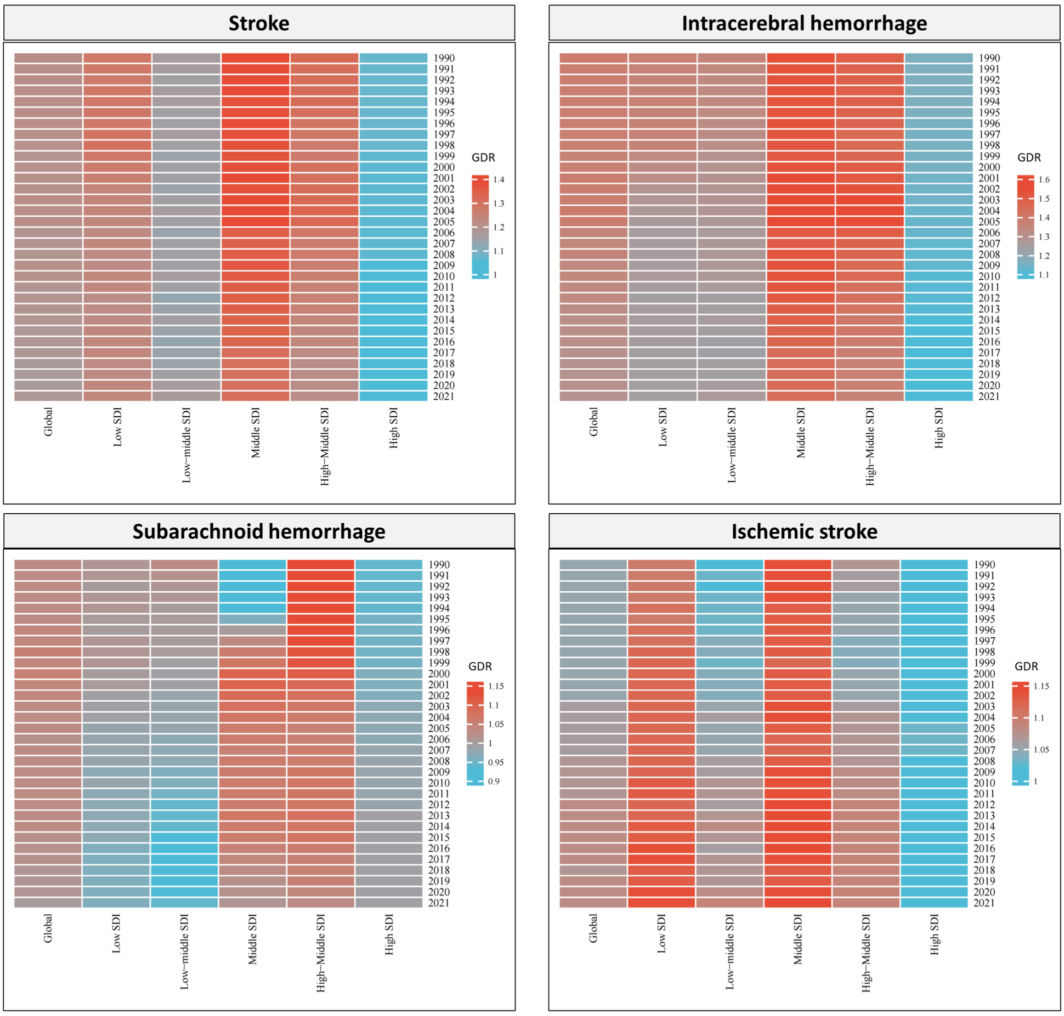

S22 Fig. SDI Regional distribution of gender disparity ratio of stroke, ICH, SAH, and IS from 1990 to 2021. ICH, intracerebral hemorrhage; SAH, subarachnoid hemorrhage; IS, ischemic stroke; SDI, socio-demographic index; GDR, gender disparity ratio

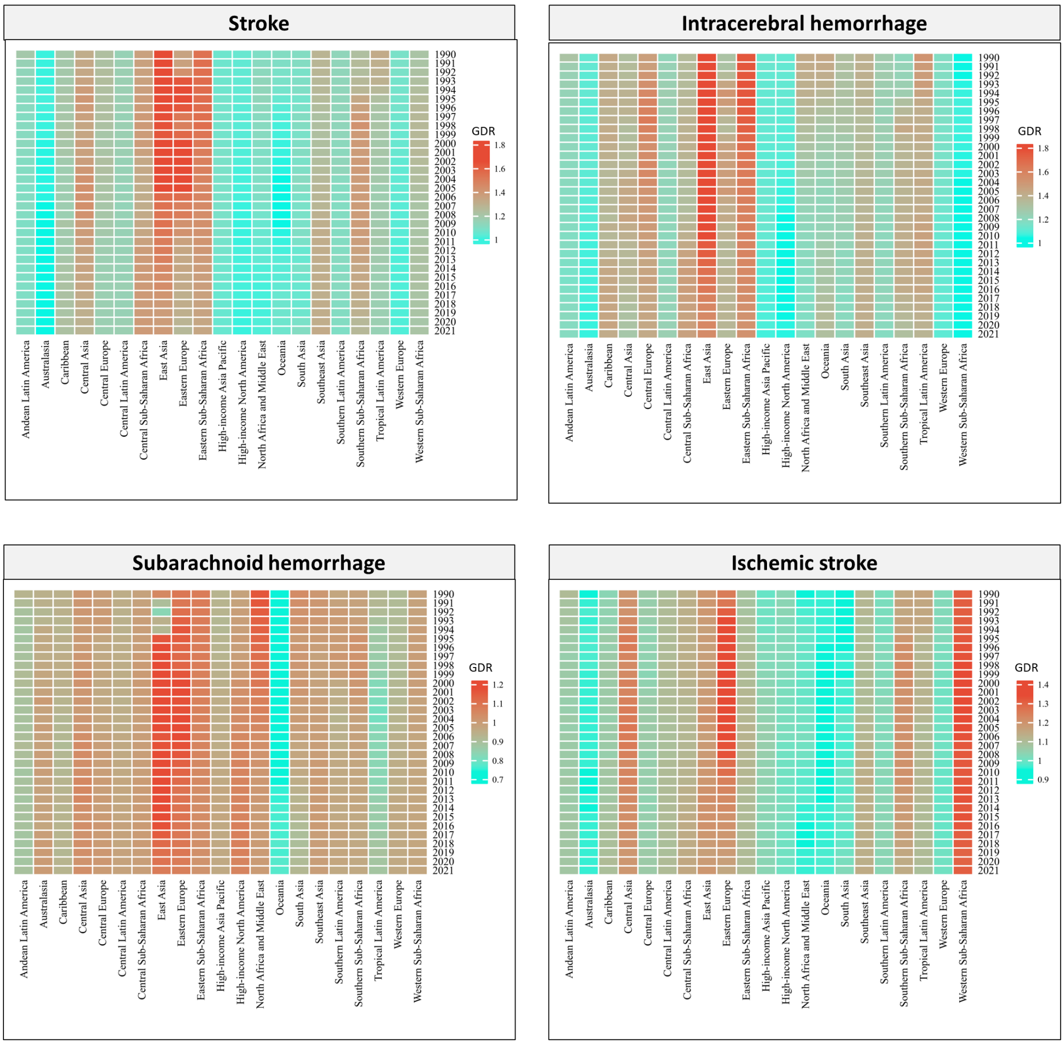

S23 Fig. Regional distribution of gender disparity ratio of stroke, ICH, SAH, and IS from 1990 to 2021. ICH, intracerebral hemorrhage; SAH, subarachnoid hemorrhage; IS, ischemic stroke; GDR, gender disparity ratio

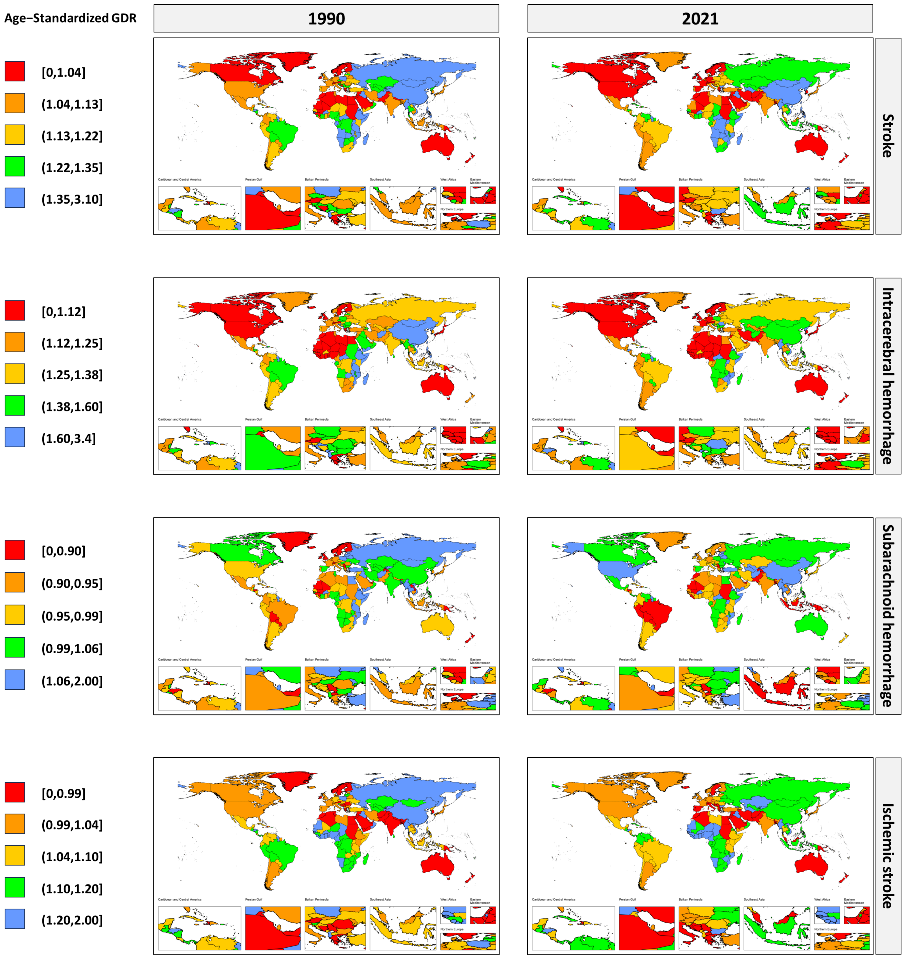

S24 Fig. Global distribution of gender disparity ratio of stroke, ICH, SAH, and IS in 1990 and 2021. ICH, intracerebral hemorrhage; SAH, subarachnoid hemorrhage; IS, ischemic stroke; GDR, gender disparity ratio

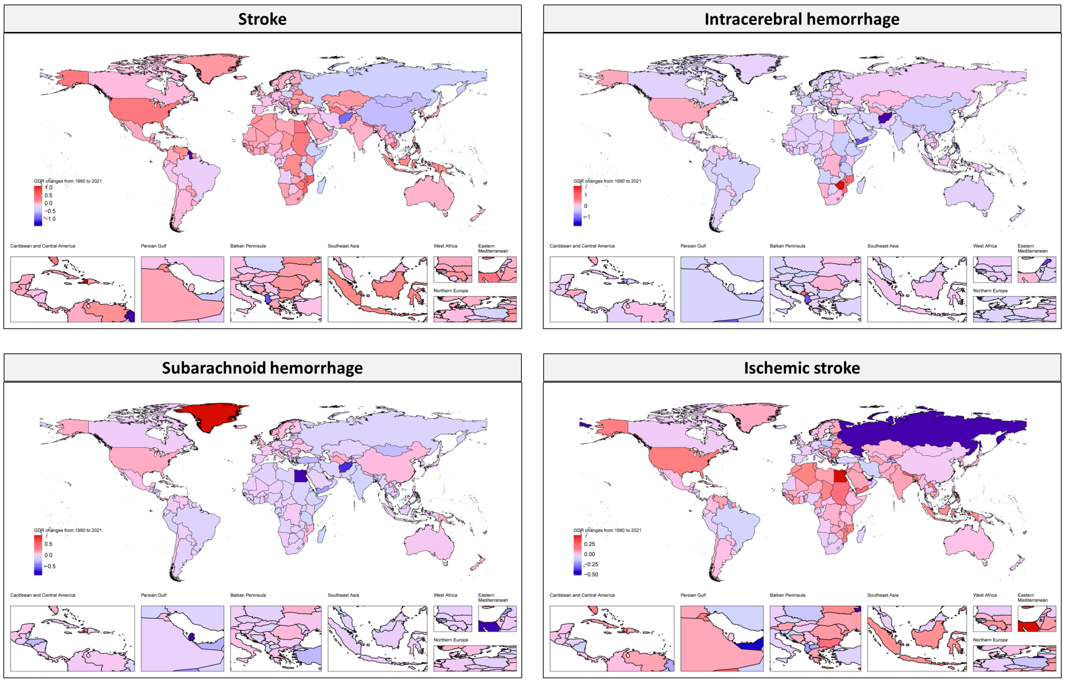

S25 Fig. Geographical distribution of age-standardized GDR changes for stroke, ICH, SAH, and IS from 1990 to 2021. ICH, intracerebral hemorrhage; SAH, subarachnoid hemorrhage; IS, ischemic stroke; GDR, gender disparity ratio. Changes represents the difference between the GDR in 2021 and the QCI in 1990

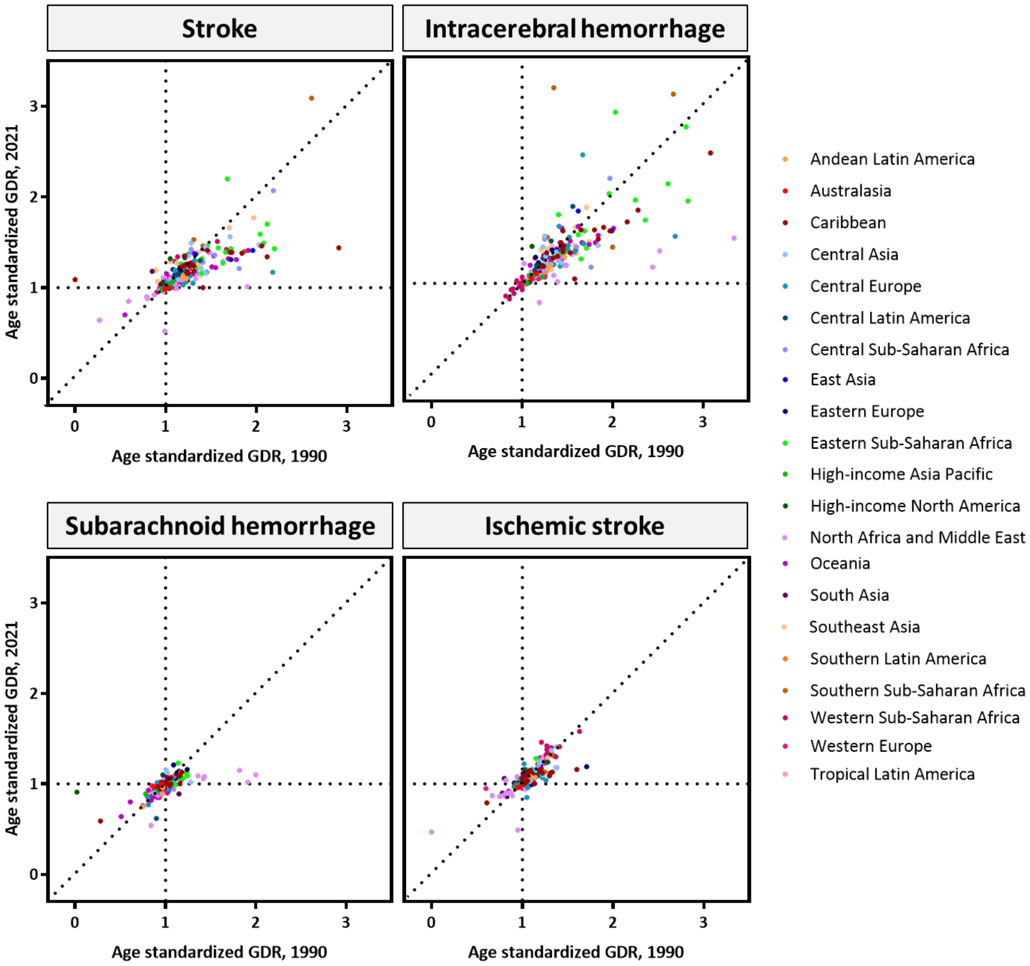

S26 Fig. Age-standardized gender disparity ratio for stroke, ICH, SHA, and IS in 1990 and 2021 in different countries according to the 21 GBD regions (each point represents a country color-coded based on the 21 GBD regions). ICH, intracerebral hemorrhage; SAH, subarachnoid hemorrhage; IS, ischemic stroke; GDR, gender disparity ratio

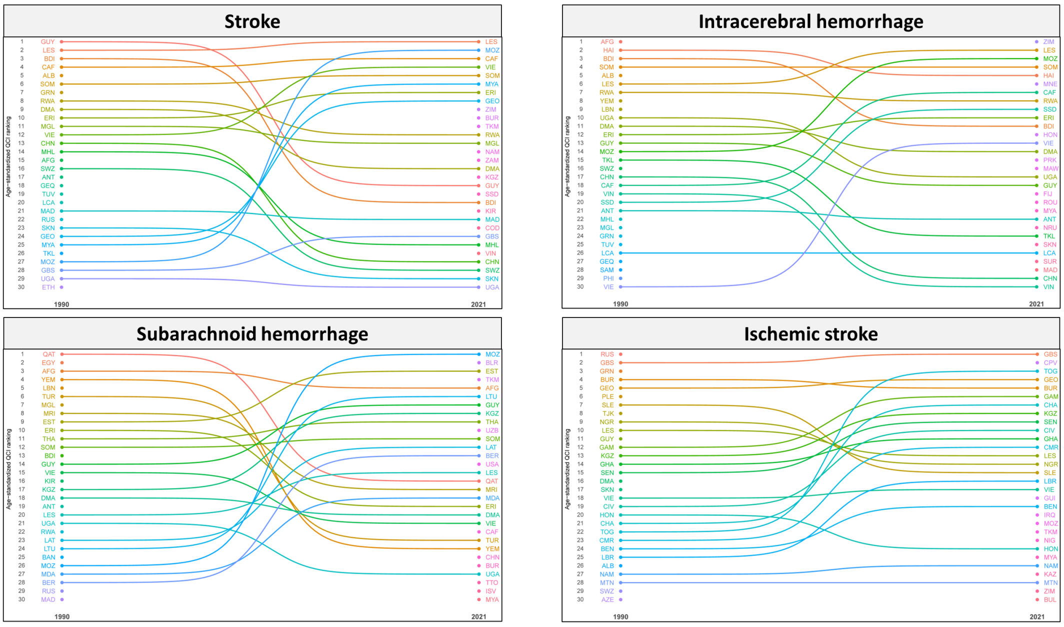

S27 Fig. The top 30 countries with the lowest and highest age-standardized GDR for stroke, ICH, SAH, and IS in 1990 and 2021. GUY, Guyana; LES, Lesotho; BDI, Burundi; CAF, Central African Republic; ALB, Albania; SOM, Somalia; GRN, Grenada; RWA, Rwanda; DMA, Dominica; ERI, Eritrea; MGL, Mongolia; VIE, Viet Nam; CHN, China; MHL, Marshall Islands; AFG, Afghanistan; SWZ, Eswatini; ANT, Antigua and Barbuda; GEQ, Equatorial Guinea; TUV, Tuvalu; LCA, Saint Lucia; MAD, Madagascar; RUS, Russian Federation; SKN, Saint Kitts and Nevis; GEO, Georgia; MYA, Myanmar; TKL, Tokelau; MOZ, Mozambique; GBS, Guinea-Bissau; UGA, Uganda; ETH, Ethiopia; ZIM, Zimbabwe; BUR, Burkina Faso; TKM, Turkmenistan; NAM, Namibia; ZAM, Zambia; KGZ, Kyrgyzstan; SSD, South Sudan; KIR, Kiribati; COD, Democratic Republic of the Congo; VIN, Saint Vincent and the Grenadines; HAI, Haiti; YEM, Yemen; LBN, Lebanon; SAM, Samoa; PHI, Philippines; MNE, Montenegro; HON, Honduras; PRK, Democratic People's Republic of Korea; MAW, Malawi; FIJ, Fiji; ROU, Romania; NAU, Nauru; SUR, Suriname; QAT, Qatar; EGY, Egypt; TUR, Turkey; MRI, Mauritius; EST, Estonia; THA, Thailand; LAT, Latvia; LTU, Lithuania; BAN, Bangladesh; MDA, Republic of Moldova; BER, Bermuda; BLR, Belarus; UZB, Uzbekistan; USA, United States of America; TTO, Trinidad and Tobago; ISV, United States Virgin Islands; PLE, Palestine; SLE, Sierra Leone; TJK, Tajikistan; NGR, Nigeria; GAM, Gambia; GHA, Ghana; SEN, Senegal; CIV, Coted'Ivoire; CHA, Chad; TOG, Togo; CMR, Cameroon; BEN, Benin; LBR, Liberia; MTN, Mauritania;AZE, Azerbaijan; CPV, Cabo Verde; GUI, Guinea; IRQ, Iraq; NIG, Niger; KAZ, Kazakhstan; BUL, Bulgaria. ICH, intracerebral hemorrhage; SAH, subarachnoid hemorrhage; IS, ischemic stroke; GDR, gender disparity ratio

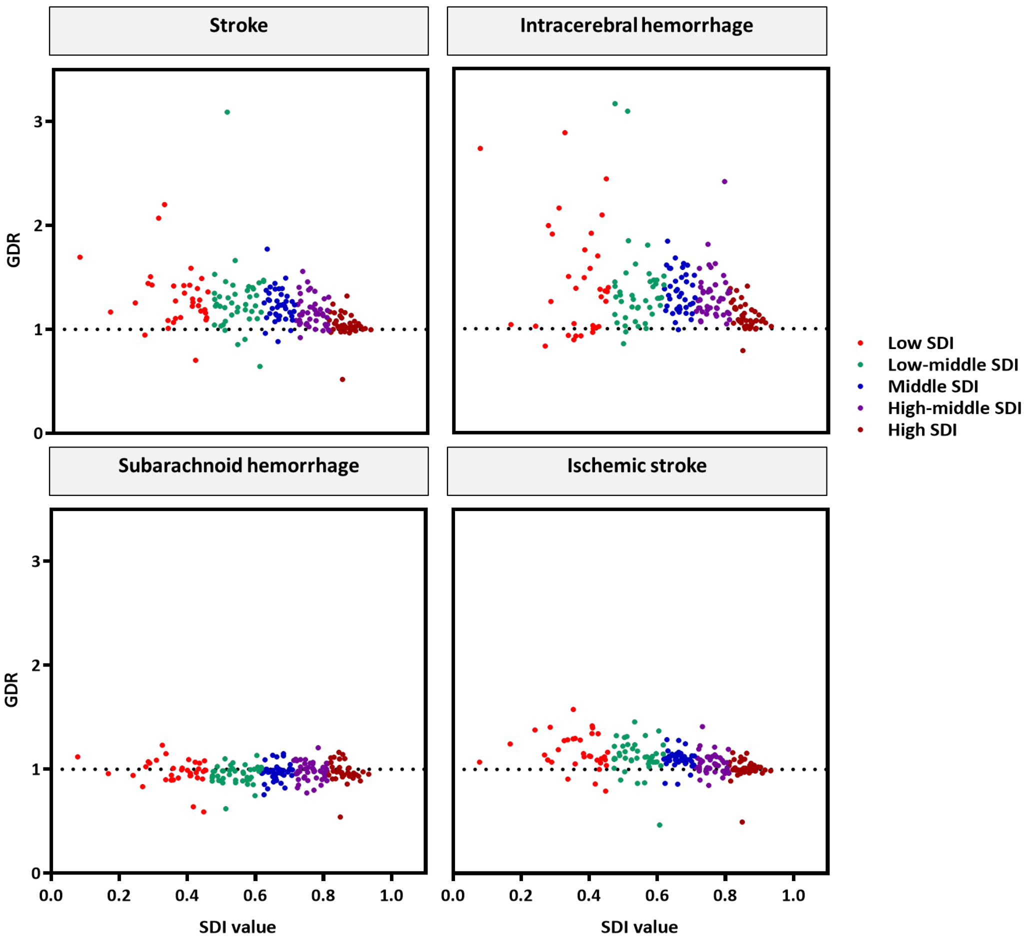

S28 Fig. Age-standardized GDR of different countries according to SDI classification for stroke, ICH, SAH, and IS in 2021 (each point represents a country color-coded based on the 5 SDI classification). ICH, intracerebral hemorrhage; SAH, subarachnoid hemorrhage; IS, ischemic stroke; GDR, gender disparity ratio; ; SDI, socio-demographic index

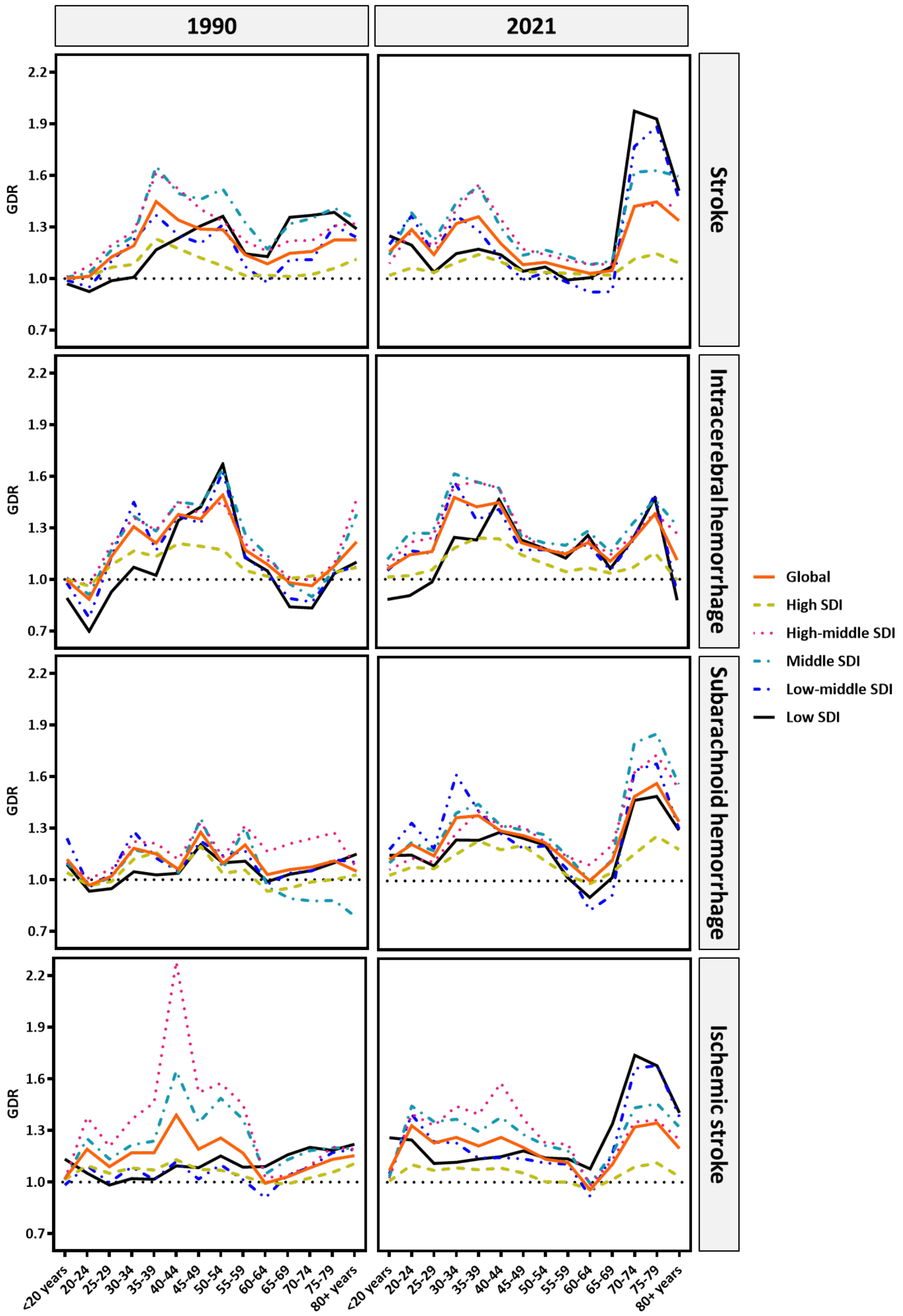

S29 Fig. Age trend of GDR in the global and 5 sociodemographic index regions for stroke, ICH, SAH, and IS in 1990 and 2021. ICH, intracerebral hemorrhage; SAH, subarachnoid hemorrhage; IS, ischemic stroke; GDR, gender disparity ratio; SDI, socio-demographic index
