# Supplementary material for: Quality of care index and gender disparity ratio for stroke and its subtypes from the Global Burden of Disease Study 2021
Source: PLoS One. 2026 Apr 29;21(4):e0345938. doi: 10.1371/journal.pone.0345938 (PMC13127941; doi:10.1371/journal.pone.0345938)
Supplement: S2 File — (PDF) [file pone.0345938.s002.pdf]

SUPPLEMENTAL MATERIAL

Supplementary Methods
Overview Description of GBD 2021
Data sources of GBD 2021
Case Definition
Term description
The models of this study
Estimated annual percentage change (EAPC) analysis
S1 Table Age-standardized prevalence, incidence, and YLDs of stroke, ICH, SAH, and IS from 1990 to 2021 at the global and SDI levels
S2 Table Age-standardized prevalence, incidence, and YLDs of stroke, ICH, SAH, and IS from 1990 to 2021 at regional levels
S3 Table Age-standardized prevalence, incidence, and YLDs of stroke, ICH, SAH, and IS from 1990 to 2021 at national levels
S4 Table Age-Standardized QCI in global and 5 sociodemographic index regions
S5 Table A world map depicting the QCI and its changes in each country for stroke, ICH, SAH, and IS in both sexes, females, and males in 1990 and 2021
S6 Table The number of QCI growing countries stroke, ICH, SAH, and IS in women and men from 1990 to 2021
S7 Table QCI values by sex, age group and SDI regions for stroke, ICH, SAH, and IS in 1990 and 2021
S8 Table QCI values by sex, age group and 21 GBD regions for stroke, ICH, SAH, and IS in 1990 and 2021
S9 Table QCI values by sex, age group and countires for stroke, ICH, SAH, and IS in 1990 and 2021
S10 Table Percent changes of the QCIs for stroke, ICH, SAH, and IS in SDI regions between 1990 and 2021 for both sexes, females, and males
S11 Table Percent changes of the QCI for stroke, ICH, SAH, and IS in 21 GBD regions between 1990 and 2021 for both sexes, females, and males
S12 Table Percent changes of the QCIs for stroke, ICH, SAH, and IS in countries between 1990 and 2021 for both sexes, females, and males
S13 Table Values of age-standardized gender disparity ratio in burden of stroke, ICH, SAH, and IS at global and SDI levels
S14 Table Values of age-standardized gender disparity ratio in burden of stroke, ICH, SAH, and IS at regional levels
S15 Table Values of age-standardized gender disparity ratio in burden of stroke, ICH, SAH, and IS at national levels
S16 Table The number of countries for GDR values near one for stroke, ICH, SAH, and IS in 1990 and 2021
S17 Table GDR values by age group and SDI regions for stroke, ICH, SAH, and IS in 1990 and 2021
S18 Table GDR values by age group and 21 GBD regions for stroke, ICH, SAH, and IS in 1990 and 2021
S19 Table The number of regions for GDR values near one for stroke, ICH, SAH, and IS in different age groups in 1990 and 2021
S20 Table GDR values by age group and countries for stroke, ICH, SAH, and IS in 1990 and 2021
S21 Table The number of countries for GDR values near one for stroke, ICH, SAH, and IS in different age groups in 1990 and 2021
S22 Table The ASPR, ASIR, ASMR, and DALYs rate of stroke and its subtypes in China in 2021
S23 Table The EAPC results in China from 1990 to 2021

Supplementary Methods

Overview Description of GBD 2021

GBD provides estimated metrics and measures of 0-95 years and older and 23 age groups for males, females, and both sexes across 204 countries, 21 regions, and seven super-regions. The metrics includes number, rate, percent, years, probability of death. Measures mainly includes deaths, years of life lost (YLLs), years lived with disability (YLDs), disability-adjusted life years (DALYs), prevalence, incidence. Compared with GBD 2019, GBD 2021 updates the demographic changes across countries and regions for 371 diseases from 1990 to 2021. In addition, it adds the 5,296 additional data sources, and the number of non-fatal disease predictions increase from 250 to 359 [2]. 25 risk outcome pairs are also removed in GBD 2021. Six age groups under 5 years, 0-6 days, 7-27 days, 1-5 months, 6-11 months, 12-23 months, and 2-4 years are added. For the first time, twelve additional causes of death are reported, including COVID-19-related mortality, other pandemic-related mortalities, pulmonary arterial hypertension, and nine types of cancer (hepatoblastoma, Burkitt lymphoma, other non-Hodgkin lymphomas, eye cancer, retinoblastoma, other ocular malignancies, soft tissue and extraosseous sarcomas, malignant neoplasms of bone and articular cartilage, and neuroblastoma along with other peripheral nerve cell tumors).

Data sources of GBD 2021

The data of non-fatal diseases are derived from 75,459 sources, including scientific literature, household survey data, epidemiological surveillance data, disease registry data, clinical informatics data, and other sources. Of them, 36,916 sources and 22,236 sources were used on incidence and prevalence, respectively [2]. Cause-specific literature reviews included searches of online research databases, government and international organisation websites, published reports, primary data sources, and contributions of datasets by GBD Collaborators. A variety of methods, including vital registration, verbal autopsy, registry, survey, police, or surveillance data across all countries and territories, were used to confirm data sources. Each data source is cataloged with a unique identifier and documented in the Global Health Data Exchange (GHDx). The GHDx platform provides access to metadata for each source, subject to the data provider's permissions. All metadata is accessible online via the GHDx platform at ghdx.healthdata.org/gbd-results-tool.

Case Definition

According to WHO criteria, stroke was rapidly developed clinical signs of focal disturbance of cerebral function lasting >24 hours or leading to death. Based on whether they are caused by a blockage or rupture of the blood vessels in the brain, strokes are divided as ischaemic stroke (IS), intracerebral hemorrhage (ICH), and subarachnoid hemorrhage (SAH). ICH is defined by the rupture of a blood vessel, leading to bleeding within the brain's tissue, and occurs spontaneously rather than due to trauma. SAH was is characterized by the rupture of a blood vessel, causing bleeding into the subarachnoid space surrounding the brain. IS results from the obstruction of blood flow to a portion of the brain caused by a thrombus or embolism, leading to neurological impairment.

Term description

In this analysis, stroke prevalence is depicted as the proportion of the population experiencing the condition over a given period. Incidence refers to the number of new stroke cases emerging within a specified population during a defined timeframe. Years Lived with Disability (YLD) are calculated by multiplying the prevalence of different severity levels by their respective disability weights. Years of Life Lost (YLL) are determined by multiplying the number of deaths in each age group by the remaining life expectancy for that age group. Disability-Adjusted Life Years (DALYs) combine YLLs and YLDs to offer a comprehensive metric of the total years of healthy life lost due to stroke, from its onset to death.

The models of this study

The cause of death ensemble model (CODEm), spatiotemporal Gaussian process regression (ST GPR), and DisMod MR was used in our analysis. Briefly, CODEm is designed to assess stroke-related mortality by employing a diverse array of modeling techniques and covariates, refined through out-of-sample predictive validity tests [1]. The Gaussian Process Regression (GPR) model is crafted to bridge the gap between stroke burden as influenced by socioeconomic development and the present scenario, leveraging correlations across different locations and time periods for specific metrics of interest [2]. The DisMod-MR analysis, a Bayesian disease modeling tool, synthesizes data from various sources and accounts for known variability factors to assess stroke incidence, prevalence, and mortality rates, thereby producing internally consistent estimates [8].

Estimated annual percentage change (EAPC) analysis

Assuming a linear relationship between the natural logarithm of age-standardized rates and time, a regression model was expressed as $y = \alpha + \beta x + \epsilon$. Here, y denotes the natural logarithm of rates (such as prevalence or incidence), α represents the intercept, x is the calendar year, ϵ is the error term, and β is the regression coefficient. The Estimated Annual Percentage Change (EAPC) is calculated as $EAPC = 100 \times[\exp(\beta)-1]$, with its 95% confidence interval (CI) derived from the linear regression model. A statistically significant increase or decrease in the age-standardized rate is indicated when both the EAPC estimate and its 95% CI are consistently above or below zero. Conversely, if the EAPC estimate and its 95% CI include zero, the trend is deemed stable, suggesting no significant temporal change.

Table S1 Age-standardized prevalence, incidence, and YLDs of stroke, ICH, SAH, and IS from 1990 to 2021 at the global and SDI levels

		Prevalence (95% uncertainty interval)					incidence (95% uncertainty interval)					YLDs (95% uncertainty interval)				
location	cause	NO. in 1990 (000s)	ASR per 100,000 (1990)	NO. in 2021 (000s)	ASR per 100,000 (2021)	EAPC(1990-2021)	NO. in 1990 (000s)	ASR per 100,000 (1990)	NO. in 2021 (000s)	ASR per 100,000 (2021)	EAPC(1990-2021)	NO. in 1990 (000s)	ASR per 100,000 (1990)	NO.in 2021 (000s)	ASR per 100,000 (2021)	EAPC(1990-2021)
Global	Stroke	50416(47793,53227)	1201.1(1138.0,1271.3)	93816(89030,99335)	1099.3(1044.2,1162.1)	-0.37(-0.4 to -0.35)	7019(6346,7794)	181.0(164.0,200.6)	11946(10772,13220)	141.6(128.0,155.8)	-0.99(-1.08 to -0.9)	8010(5778,10181)	192.9(139.4,245.3)	15210(10986,19425)	178.7(128.8,227.7)	-0.33(-0.35 to -0.3)
Low SDI	Stroke	3306(3149,3481)	1223.7(1158.4,1291.7)	6588(6323,6864)	1056.7(1006.4,1107.2)	-0.57(-0.63 to -0.51)	445(406,489)	188.6(171.9,207.8)	799(737,866)	147.4(135.3,160.2)	-1.75(-1.86 to -1.63)	441(322,553)	168.7(124.0,212.5)	888(652,1122)	147.5(108.8,185.5)	-0.57(-0.63 to -0.51)
Low-middle SDI	Stroke	7456(7038,7936)	1040.8(978.1,1111.3)	15293(14510,16186)	972.9(920.1,1030.8)	-0.38(-0.41 to -0.34)	1054(960,1162)	170.9(155.4,188.5)	2029(1855,2209)	142.3(130.6,154.4)	-1.2(-1.31 to -1.08)	1068(776,1354)	151.9(110.2,192.1)	2193(1597,2774)	141.7(103.3,179.7)	-0.29(-0.32 to -0.26)
Middle SDI	Stroke	13625(12827,14466)	1149.8(1077.8,1233.4)	30207(28379,32296)	1126.9(1060.2,1202.1)	-0.12(-0.14 to -0.1)	1921(1743,2140)	192.2(174.1,211.9)	4215(3795,4707)	165.0(148.5,182.8)	-0.69(-0.77 to -0.62)	2239(1613,2848)	192.0(137.8,243.1)	5079(3651,6520)	190.9(136.7,244.2)	-0.06(-0.08 to -0.03)
High-middle SDI	Stroke	12713(11971,13458)	1242.7(1171.0,1318.0)	21406(20065,22783)	1138.3(1070.8,1208.8)	-0.27(-0.29 to -0.25)	2036(1823,2290)	217.7(196.5,241.9)	3094(2748,3480)	162.0(145.0,180.7)	-0.74(-0.8 to -0.68)	2232(1611,2852)	219.3(158.3,279.5)	3875(2807,4958)	205.6(148.4,262.7)	-0.27(-0.28 to -0.25)
High SDI	Stroke	13262(12588,13972)	1237.9(1175.6,1301.2)	20249(19375,21279)	1072.4(1025.1,1124.2)	-0.56(-0.59 to -0.53)	1555(1390,1742)	143.8(129.2,159.9)	1800(1632,1981)	90.2(82.1,99.2)	-0.95(-1.02 to -0.88)	2022(1440,2577)	188.1(134.3,239.2)	3165(2288,4028)	163.3(117.5,207.4)	-0.5(-0.53 to -0.47)
Global	ICH	11174(10197,12318)	250.2(228.0,275.1)	16604(15159,18183)	194.5(178.0,212.5)	-0.97(-1.04 to -0.9)	2358(2052,2634)	59.5(51.4,66.6)	3444(3053,3812)	40.8(36.2,45.2)	-1.52(-1.67 to -1.38)	1791(1262,2324)	40.4(28.4,52.4)	2687(1909,3470)	31.5(22.5,40.7)	-0.96(-1.03 to -0.89)
Low SDI	ICH	909(838,990)	297.3(272.4,327.8)	1572(1457,1697)	222.0(204.9,241.9)	-0.85(-0.9 to -0.79)	201(177,225)	85.2(75.3,94.6)	312(281,344)	57.8(52.0,63.3)	-2.05(-2.19 to -1.91)	121(86,156)	40.7(29.0,52.2)	212(152,272)	31.1(22.2,40.0)	-0.87(-0.93 to -0.82)
Low-middle SDI	ICH	2062(1880,2281)	265.6(240.5,294.3)	3639(3332,3994)	219.9(200.1,241.2)	-1.37(-1.48 to -1.25)	451(394,508)	72.5(63.1,81.6)	762(674,856)	52.7(46.7,58.5)	-2.16(-2.41 to -1.9)	298(211,388)	39.0(27.8,50.3)	526(375,682)	32.2(23.0,41.6)	-1.27(-1.39 to -1.15)
Middle SDI	ICH	3607(3246,4018)	274.0(246.1,305.8)	5670(5113,6288)	204.7(185.5,226.0)	-1.1(-1.17 to -1.02)	792(681,894)	79.8(68.6,90.4)	1327(1162,1483)	52.3(45.9,58.5)	-1.64(-1.8 to -1.48)	605(420,791)	46.7(32.3,60.8)	968(681,1253)	35.2(24.8,45.6)	-1.05(-1.13 to -0.98)
High-middle SDI	ICH	2584(2352,2833)	246.5(224.2,269.5)	3102(2798,3414)	175.5(159.9,193.0)	-0.72(-0.77 to -0.67)	613(528,691)	64.7(55.9,72.6)	723(634,810)	38.5(33.9,43.0)	-1.25(-1.35 to -1.15)	456(318,591)	43.7(30.6,56.5)	568(400,738)	32.0(22.6,41.7)	-0.71(-0.76 to -0.67)
High SDI	ICH	2002(1851,2178)	195.3(180.2,211.8)	2610(2394,2826)	153.5(141.9,165.2)	-1.11(-1.16 to -1.06)	298(262,333)	28.1(24.8,31.2)	318(280,352)	16.3(14.5,18.0)	-1.49(-1.59 to -1.39)	310(222,395)	30.2(21.6,38.4)	412(297,526)	23.6(17.0,30.3)	-1.01(-1.06 to -0.97)
Global	SAH	4902(4421,5395)	109.9(99.1,121.6)	7853(7165,8579)	92.2(84.1,100.6)	-0.64(-0.67 to -0.61)	509(442,588)	11.7(10.2,13.5)	697(614,796)	8.3(7.3,9.5)	-1.52(-1.66 to -1.37)	765(538,989)	17.2(12.2,22.2)	1205(862,1538)	14.2(10.1,18.1)	-0.7(-0.73 to -0.68)
Low SDI	SAH	292(264,322)	97.3(88.4,107.1)	600(550,653)	85.4(78.6,92.7)	-0.2(-0.25 to -0.15)	28(24,32)	9.0(7.8,10.6)	54(47,62)	7.4(6.5,8.6)	-0.99(-1.06 to -0.92)	39(28,49)	13.1(9.6,16.5)	80(57,102)	11.6(8.3,14.7)	-0.17(-0.22 to -0.11)
Low-middle SDI	SAH	806(724,891)	101.4(91.2,112.6)	1502(1368,1646)	89.4(81.4,98.2)	-0.95(-1 to -0.91)	86(74,98)	11.0(9.6,12.8)	146(127,167)	8.9(7.8,10.2)	-1.96(-2.14 to -1.78)	112(81,145)	14.2(10.2,18.2)	208(149,265)	12.4(8.9,15.9)	-0.95(-1 to -0.9)
Middle SDI	SAH	1542(1375,1703)	117.8(105.3,130.6)	2493(2256,2752)	90.8(82.2,99.9)	-0.94(-1 to -0.88)	176(151,204)	14.9(12.9,17.4)	240(210,276)	9.2(8.1,10.4)	-2.22(-2.47 to -1.97)	248(174,322)	19.1(13.5,24.9)	386(276,496)	14.1(10.1,18.1)	-1.09(-1.16 to -1.02)
High-middle SDI	SAH	1029(924,1139)	97.5(87.6,107.9)	1314(1194,1448)	75.6(68.7,82.7)	-0.44(-0.46 to -0.42)	116(101,134)	11.6(10.2,13.3)	127(113,146)	7.4(6.6,8.4)	-0.92(-1 to -0.84)	179(125,232)	17.0(11.9,22.0)	228(163,295)	13.2(9.4,17.0)	-0.45(-0.48 to -0.43)
High SDI	SAH	1228(1117,1344)	118.6(107.9,129.7)	1937(1781,2088)	112.4(103.7,121.3)	-0.47(-0.49 to -0.45)	103(90,120)	10.1(8.8,11.8)	130(116,148)	7.9(6.9,9.0)	-0.86(-0.93 to -0.79)	186(133,240)	17.9(12.7,23.1)	301(217,388)	17.1(12.2,22.0)	-0.43(-0.45 to -0.41)
Global	IS	34668(32154,37172)	849.5(785.9,913.2)	69945(64789,75010)	819.5(760.3,878.7)	-0.18(-0.21 to -0.16)	4152(3537,4868)	109.8(93.6,127.6)	7804(6720,8944)	92.4(79.8,105.8)	-0.67(-0.76 to -0.58)	5454(3922,6953)	135.3(97.8,172.8)	11318(8184,14472)	133.0(96.0,169.9)	-0.11(-0.14 to -0.08)
Low SDI	IS	2125(1977,2271)	838.1(776.3,901.2)	4448(4174,4700)	755.7(707.7,800.1)	-0.57(-0.64 to -0.5)	216(184,252)	94.4(80.4,110.8)	433(375,494)	82.2(71.0,93.1)	-1.75(-1.86 to -1.64)	282(207,354)	115.0(85.5,144.6)	596(437,745)	104.9(78.1,131.0)	-0.56(-0.63 to -0.49)
Low-middle SDI	IS	4631(4229,5024)	680.8(620.6,741.7)	10234(9419,11004)	669.3(616.3,720.6)	-0.09(-0.14 to -0.04)	517(440,604)	87.3(74.1,102.4)	1120(972,1274)	80.7(70.0,91.7)	-0.74(-0.85 to -0.63)	658(478,836)	98.7(72.0,125.1)	1460(1064,1849)	97.0(71.0,122.8)	0.01(-0.05 to 0.06)
Middle SDI	IS	8556(7763,9310)	765.9(693.0,837.5)	22231(20240,24120)	838.4(764.8,911.0)	0.27(0.24 to 0.3)	954(801,1135)	97.5(81.4,115.6)	2649(2241,3083)	103.5(88.0,120.0)	0.12(0.07 to 0.16)	1386(995,1770)	126.1(91.1,160.8)	3725(2671,4796)	141.6(101.1,181.8)	0.38(0.34 to 0.42)
High-middle SDI	IS	9174(8430,9883)	906.2(834.7,975.0)	17111(15739,18479)	893.3(824.0,960.0)	-0.09(-0.11 to -0.06)	1306(1096,1548)	141.4(119.9,164.1)	2243(1900,2614)	116.0(99.2,133.9)	-0.34(-0.38 to -0.3)	1597(1149,2047)	158.6(114.0,203.3)	3078(2217,3952)	160.5(115.7,206.5)	-0.08(-0.1 to -0.06)
High SDI	IS	10142(9488,10833)	933.8(874.5,998.0)	15865(14926,16873)	813.9(768.6,864.1)	-0.39(-0.43 to -0.36)	1153(993,1333)	105.5(91.2,121.2)	1352(1180,1536)	66.1(58.2,74.7)	-0.51(-0.56 to -0.46)	1526(1084,1953)	140.0(99.7,179.5)	2451(1782,3115)	122.5(88.2,156.1)	-0.34(-0.37 to -0.3)

ASR, Agestandardised rates; EAPC, estimated annual percentage change; SDI, sociodemographic index; ICH, intracerebral hemorrhage; SAH, subarachnoid hemorrhage; IS, ischemic stroke; YLDs， years lived with disability

Table S2 Age-standardized prevalence, incidence, and YLDs of stroke, ICH, SAH, and IS from 1990 to 2021 at regional levels

		Prevalence (95% uncertainty interval)					incidence (95% uncertainty interval)					YLDs (95% uncertainty interval)				
location	cause	NO. in 1990	ASR per 100,000 (1990)	NO. in 2021	ASR per 100,000 (2021)	EAPC(1990-2021)	NO. in 1990	ASR per 100,000 (1990)	NO. in 2021	ASR per 100,000 (2021)	EAPC(1990-2021)	NO. in 1990	ASR per 100,000 (1990)	NO. in 2021	ASR per 100,000 (2021)	EAPC(1990-2021)
Andean Latin America	ICH	53925.8(50499.4 to 57318.9)	195.5(183.5 to 208)	77723.9(72457.7 to 83004.4)	121(112.8 to 129.4)	-1.8(-1.88 to -1.72)	8395.9(7653.2 to 9185.6)	35.5(32.2 to 38.8)	10817.9(9857.6 to 11860.8)	18(16.3 to 19.6)	-2.44(-2.59 to -2.29)	4765.4(3450.2 to 6182.2)	17.8(13 to 22.9)	7178.3(5228.2 to 9386.9)	11.3(8.2 to 14.8)	-1.69(-1.76 to -1.61)

Australasia	ICH	25720.1(23812.6 to 27796.9)	113.6(105.2 to 122.7)	37992.9(34897.8 to 41104.4)	85.2(77.7 to 92.7)	-1.04(-1.11 to -0.97)	4256.3(3909.2 to 4575.9)	18.6(17.1 to 20.1)	5697.9(5118.3 to 6243.4)	10.8(9.6 to 11.9)	-1.8(-1.93 to -1.67)	3749.6(2679.9 to 4826.8)	16.5(11.8 to 21.2)	5890.2(4184 to 7598.2)	12.8(9.1 to 16.8)	-0.93(-0.98 to -0.87)
Caribbean	ICH	64381.9(60454.6 to 68790.3)	220.8(207.6 to 235.6)	87174.7(82051.4 to 92793.9)	167.7(158 to 178.6)	-1.05(-1.1 to -1.01)	12488.8(11535.9 to 13403.2)	46.2(42.7 to 49.5)	17584.1(16192.2 to 18934.7)	33.3(30.7 to 35.9)	-1.25(-1.36 to -1.14)	5661.6(4133.4 to 7246.8)	19.6(14.3 to 25.1)	7879.9(5757 to 10098.9)	15.1(11 to 19.4)	-1.01(-1.06 to -0.97)
Central Asia	ICH	142478.4(133370.9 to 151423.3)	263.8(246.8 to 279.9)	180253.3(168874.8 to 191994.2)	191.3(179.5 to 204.1)	-1.26(-1.45 to -1.08)	39620.6(36705.9 to 42560.6)	86.3(80.1 to 92.5)	55547.7(51306.4 to 59649.6)	74.7(69.3 to 79.9)	-0.6(-0.86 to -0.34)	25576.4(18285 to 32660.7)	47.7(33.9 to 60.9)	32197.6(23093.6 to 41482.9)	34.7(24.8 to 44.6)	-1.24(-1.42 to -1.06)
Central Europe	ICH	246756.2(228537.3 to 265220.5)	175.1(162.5 to 188.4)	151846(140163.2 to 164066.2)	96.5(89.3 to 104.1)	-2.27(-2.38 to -2.17)	70008.6(64093 to 75290.8)	50.3(46.3 to 53.9)	50101(45976.4 to 53822.9)	23.7(22 to 25.4)	-2.86(-3.03 to -2.69)	44277.9(31257.9 to 56821.6)	31.5(22.3 to 40.3)	28356.3(20133.6 to 36613.7)	17.7(12.6 to 23)	-2.19(-2.29 to -2.09)
Central Latin America	ICH	227539.1(207050.2 to 248322)	195.8(179.5 to 212)	339240.4(312470 to 366494.4)	130.9(120.9 to 141.5)	-1.54(-1.61 to -1.47)	31601(28194.1 to 35406.7)	33.8(30.1 to 37)	48325.4(43161.3 to 53249.4)	19.6(17.6 to 21.6)	-2.05(-2.18 to -1.92)	19916.1(14615.7 to 26007.4)	17.7(13.1 to 22.8)	30895.3(22490.3 to 40114.1)	12(8.8 to 15.6)	-1.48(-1.55 to -1.41)
Central Sub-Saharan Africa	ICH	94869.4(88596.8 to 101656.3)	292.8(272.5 to 316)	174316.3(163264.2 to 186749.4)	208.2(193.4 to 225)	-1.27(-1.33 to -1.22)	21106.2(18698.9 to 23579.4)	90(80.5 to 99.1)	37322.8(33808.7 to 41188.4)	63.6(58.1 to 69.4)	-1.32(-1.41 to -1.24)	12496.1(8971.6 to 16161)	40.1(28.5 to 51.6)	23337(16712 to 30063.9)	29.8(21.3 to 39)	-1.09(-1.13 to -1.04)
East Asia	ICH	3252210.8(2895893.6 to 3662720)	310.4(276.9 to 349.4)	4554930.2(4050940.1 to 5081761.1)	223.1(201.5 to 246.9)	-1.27(-1.38 to -1.16)	804381.5(673102.7 to 927435.4)	108.6(91.6 to 124.1)	1218006.8(1046030.9 to 1377277)	61.1(53.1 to 68.8)	-2.23(-2.49 to -1.98)	623294.7(428052.3 to 818659.4)	60.4(41.7 to 79)	894367.3(620364.1 to 1158374.5)	43.9(30.5 to 57.1)	-1.24(-1.35 to -1.12)
Eastern Europe	ICH	408944.7(362616 to 455758.7)	152.5(135.7 to 169.4)	353983.5(313521.9 to 398007.1)	122(109.3 to 136.5)	-0.94(-1.16 to -0.72)	114220.8(95944.6 to 130920.8)	43.3(36.4 to 49.4)	92425.2(80056.6 to 103800.1)	28.7(25.2 to 32.4)	-1.77(-2.08 to -1.45)	73300.4(52277.7 to 95092.1)	27.4(19.5 to 35.4)	63488.1(44564.3 to 82716.6)	21.8(15.3 to 28.5)	-0.94(-1.14 to -0.74)
Eastern Sub-Saharan Africa	ICH	346993.9(320228.3 to 378196.9)	326.4(298.9 to 358.2)	535714.3(497160.6 to 575131.4)	209.8(193.4 to 227.8)	-1.68(-1.75 to -1.61)	81517.2(72264.1 to 90929.9)	100.7(89.6 to 111.6)	112869.8(102527.3 to 124052.7)	62.2(56.5 to 67.9)	-1.84(-1.95 to -1.72)	45420.2(32530.1 to 58725.7)	44.4(31.7 to 57.5)	71439.7(51654 to 91530.5)	29.6(21 to 38.3)	-1.53(-1.59 to -1.46)
High-income Asia Pacific	ICH	732339.4(671235 to 799464.8)	367.1(337.9 to 399.5)	813438.1(735552.3 to 887603.2)	232.2(213.7 to 251.9)	-1.77(-1.93 to -1.61)	88022.2(75966.4 to 99116.8)	45.2(39.2 to 50.7)	77253(67778 to 85468.3)	20(17.7 to 22.5)	-3.15(-3.44 to -2.86)	118220.4(84485.4 to 150121.1)	59.7(42.7 to 75.9)	137022.3(97195.8 to 174515.8)	37.8(27.3 to 48.3)	-1.77(-1.94 to -1.6)
High-income North America	ICH	472495.6(424070 to 525596.2)	146.8(131.6 to 162.7)	843200.1(761614.5 to 932204.9)	154.1(139.9 to 168.2)	0.5(0.21 to 0.78)	60962.1(50848 to 70524.9)	17.8(14.9 to 20.6)	79595.2(68334.4 to 90253)	13.1(11.3 to 14.7)	-0.87(-0.98 to -0.77)	67446.5(47260.3 to 87659.1)	20.8(14.6 to 26.8)	120130.7(87423.8 to 153451.4)	21.7(15.6 to 27.9)	0.46(0.18 to 0.74)
North Africa and Middle East	ICH	515146.1(482050 to 550215.8)	212.3(198.5 to 226.7)	803050.9(752918.3 to 856260.8)	139.1(130.2 to 148.3)	-1.65(-1.74 to -1.57)	85082.1(76375.2 to 93986)	45.7(41.1 to 49.9)	121271.9(109767.2 to 132332.3)	25.9(23.6 to 28.1)	-2.16(-2.29 to -2.04)	71830.7(52239.6 to 91916)	29.9(21.8 to 38.3)	111572.3(80582.1 to 142926.1)	19.7(14.3 to 25.2)	-1.63(-1.72 to -1.55)
Oceania	ICH	12456.8(11664.4 to 13335.4)	308.1(286.6 to 331.7)	24844.8(23415.4 to 26480)	246.8(230.6 to 264)	-0.84(-0.92 to -0.76)	2657.1(2378.6 to 2943.8)	94.9(85.7 to 103.6)	5442.7(4926.4 to 5964)	74.4(68 to 80.5)	-0.93(-0.99 to -0.87)	2274.4(1609.7 to 2918.4)	57.4(40.5 to 73.7)	4550(3214.9 to 5870.4)	46.2(32.5 to 59.1)	-0.82(-0.89 to -0.74)
South Asia	ICH	1751450.4(1559231.5 to 1973549.2)	243(215.5 to 274.1)	3473328.2(3115617.5 to 3881650.7)	210.6(189 to 234.4)	-0.5(-0.54 to -0.47)	387268(328887.1 to 446387.6)	67.2(56.5 to 77.2)	703200.3(607007.6 to 804764.3)	47.6(41 to 54.1)	-1.37(-1.48 to -1.25)	243510.8(172545.7 to 317250.6)	34.5(24.4 to 45)	488135(347482.8 to 632469.2)	30(21.3 to 38.7)	-0.48(-0.51 to -0.45)
Southeast Asia	ICH	1163109.5(1064447.5 to 1271539)	347.6(317.3 to 381.7)	2017274.1(1834696.4 to 2208645.1)	271.6(247.7 to 297.2)	-0.91(-0.99 to -0.83)	244778.5(215410.2 to 272158.2)	93.7(82.9 to 103.7)	450578.3(400978.2 to 497072.1)	69.8(62.4 to 76.2)	-1.09(-1.21 to -0.98)	216870.7(150965.1 to 280981.3)	65.5(45.9 to 84.8)	377043.2(267297.4 to 488702.9)	51.4(36.3 to 66.6)	-0.9(-0.97 to -0.82)
Southern Latin America	ICH	158440.7(149414.4 to 169127.9)	335.8(316.8 to 358.3)	159305.5(150466.3 to 168994.6)	199.3(188.7 to 210.9)	-2(-2.12 to -1.89)	24515.1(22113.2 to 26843.3)	53.3(48.1 to 58.2)	21617.9(19466.1 to 23529.7)	25.8(23.4 to 28.3)	-2.73(-2.9 to -2.55)	22103.8(16047.8 to 28073.4)	47.1(34.2 to 59.7)	22776(16561 to 28830.3)	28.3(20.5 to 35.9)	-1.95(-2.06 to -1.83)
Southern Sub-Saharan Africa	ICH	81028.3(72296 to 90293.6)	237.1(211.1 to 265)	103534.2(93715 to 114464.5)	152.2(137.8 to 168.9)	-1.86(-2.09 to -1.63)	15160.6(13117 to 17200.7)	51.5(44.7 to 58)	20704.6(18235.3 to 23291.5)	36.6(32.1 to 40.9)	-1.45(-1.76 to -1.15)	11039.1(7798.8 to 14059)	33.2(23.3 to 42.4)	14343.2(10215.1 to 18573.3)	21.9(15.5 to 28.5)	-1.73(-1.93 to -1.52)
Tropical Latin America	ICH	274101.2(244197.9 to 307627.8)	239.7(212.9 to 268.6)	291967.4(261326.3 to 323598.2)	113.5(101.9 to 125.7)	-2.95(-3.11 to -2.78)	52463(44397.4 to 60679.3)	51.9(43.4 to 59.8)	52687.6(45342.8 to 60231.9)	20.8(17.9 to 23.7)	-3.43(-3.63 to -3.22)	22984.4(16918.6 to 30142.9)	20.5(15.2 to 26.7)	26250.7(19173.7 to 34482.4)	10.3(7.5 to 13.5)	-2.73(-2.88 to -2.58)
Western Europe	ICH	725773.6(674679.1 to 780202.9)	144.9(134.2 to 155.8)	751957.5(699012.6 to 806480.2)	103.1(95.6 to 111.1)	-1.31(-1.37 to -1.25)	127712.7(113717 to 139441.4)	23.1(20.7 to 25.2)	124278.8(111584 to 135124.2)	13.2(11.9 to 14.5)	-2.07(-2.18 to -1.96)	101007(72543.3 to 128222.4)	19.8(14.2 to 25.2)	112044(80003.3 to 141916.3)	14.6(10.4 to 18.8)	-1.18(-1.23 to -1.12)
Western Sub-Saharan Africa	ICH	424153.5(389984.1 to 461525.5)	349.9(320.8 to 382.7)	828759.9(775625.9 to 889949.6)	274.7(254.3 to 297.7)	-0.94(-1.02 to -0.85)	82130.9(72597 to 91399.8)	89.3(78.5 to 99.8)	139009.1(125318.5 to 152585.7)	65.3(58.5 to 71.5)	-1.17(-1.27 to -1.07)	55067.5(39259.8 to 70761.9)	47(33.7 to 60.3)	108479.3(78560.3 to 138888.9)	37.6(27 to 48.1)	-0.86(-0.94 to -0.79)
Andean Latin America	IS	138242.8(131026.6 to 145937.1)	604.9(573.4 to 638.1)	312484.9(297506.2 to 327199.8)	515.1(490.6 to 539)	-0.6(-0.64 to -0.57)	13562.9(11785.2 to 15387)	63.5(55.4 to 71.9)	27415.5(24041.9 to 31116.2)	46.3(40.6 to 52.4)	-1.13(-1.24 to -1.02)	12311.5(9175.7 to 15573.4)	55.5(41.4 to 70.1)	28367.9(20986.1 to 35909.6)	47.3(35 to 59.9)	-0.59(-0.63 to -0.56)
Australasia	IS	176175.4(169750.4 to 183601.5)	751.3(724.3 to 783.4)	296950.7(286363 to 308252.3)	581.9(560.2 to 603.2)	-0.93(-0.98 to -0.89)	20989.3(19362.1 to 22541)	91.1(84.1 to 97.8)	28159.6(25187.7 to 31109)	52.8(47.3 to 58.5)	-2.05(-2.2 to -1.91)	25417.6(18474 to 32317.7)	108.7(79 to 138.3)	44249.3(32625.4 to 55541.8)	84.7(62.2 to 106.6)	-0.94(-0.99 to -0.89)
Caribbean	IS	178810(169859.7 to 188128.7)	649.1(616.1 to 684.1)	327164.5(312540.5 to 341547.6)	616.9(589.7 to 643.6)	-0.18(-0.2 to -0.16)	19577.4(17215.7 to 21952.6)	76.2(67.5 to 85.7)	36042.3(32142.5 to 39960.6)	67.3(60 to 74.7)	-0.42(-0.46 to -0.37)	15832.3(11821.3 to 20009.6)	58.2(43.5 to 73.7)	29243.6(21879.3 to 36747.2)	55(41.2 to 69.1)	-0.2(-0.21 to -0.18)
Central Asia	IS	550460.5(523473.4 to 577216.8)	1107.9(1052.3 to 1164.9)	866712.4(829519.2 to 902763)	1014.9(969.9 to 1063.6)	-0.31(-0.33 to -0.29)	65958.6(58343.7 to 73949.5)	141.9(124.6 to 159.1)	102572.6(91018.4 to 114462.7)	132.9(118.2 to 148.1)	-0.2(-0.29 to -0.11)	100285.6(72795.4 to 126675.7)	203.4(148.2 to 256.7)	157141.2(113197 to 199677.6)	186.3(134.4 to 236.5)	-0.3(-0.32 to -0.28)
Central Europe	IS	1418231.7(1324818.4 to 1513619.6)	959.6(897.8 to 1022.4)	1631106.1(1537835 to 1733826.7)	776.1(733.5 to 820.8)	-0.77(-0.82 to -0.72)	222510.3(193304.8 to 251309.2)	158(138.4 to 177.7)	238905.2(209922.3 to 266603.5)	107(95.3 to 118.4)	-1.35(-1.4 to -1.3)	253322.1(183981.4 to 324206.5)	172(125.1 to 219.9)	297818.5(214626.1 to 377584.1)	140.7(101.9 to 178.2)	-0.73(-0.78 to -0.68)
Central Latin America	IS	678691.9(630061.6 to 725121)	725.2(673.9 to 777.8)	1387551.7(1296756.3 to 1484316)	552.6(516.9 to 591.3)	-1.03(-1.09 to -0.96)	68642.9(58878.8 to 78850.5)	80.5(69 to 93.2)	127074.9(110155 to 144196.4)	52(45.1 to 58.8)	-1.67(-1.82 to -1.51)	59492.4(44200.8 to 74951.3)	65.7(48.9 to 82.9)	125259.5(93636.1 to 159103.5)	50.5(37.6 to 64.1)	-1(-1.06 to -0.94)
Central Sub-Saharan Africa	IS	266923.7(253028.2 to 281576.5)	1113.6(1052.2 to 1178.4)	596223.3(568897.8 to 624228.7)	995.7(947.7 to 1046.3)	-0.41(-0.43 to -0.38)	27144.3(23222 to 32004.7)	122.7(104.3 to 142.6)	59172.9(51496.2 to 68041.3)	109(95 to 124.8)	-0.41(-0.45 to -0.38)	34711.4(25816.2 to 43387.9)	151.5(112.3 to 189.9)	78364.6(58380.1 to 98780.4)	137.6(103.6 to 172.6)	-0.34(-0.36 to -0.32)
East Asia	IS	6921707.7(6208387 to 7617523.8)	774.2(691.5 to 863.7)	21503822.8(19292836.5 to 23714701.3)	1018(920 to 1120.1)	0.95(0.89 to 1)	800330.3(656281.1 to 982072.7)	101.3(83 to 121.9)	2850089.7(2363157.7 to 3405882.5)	134.8(112.6 to 158.4)	0.86(0.8 to 0.92)	1328866.2(944764.8 to 1705467.9)	150.8(107.5 to 192.9)	4111019.6(2915550.7 to 5333556.2)	195.7(139.1 to 252.5)	0.92(0.86 to 0.98)
Eastern Europe	IS	2869099.4(2565924.6 to 3174157)	1028(921.9 to 1132.8)	3049360.5(2763322.4 to 3340261.4)	920.4(835.8 to 1005.3)	-0.38(-0.41 to -0.35)	515846.2(425258.3 to 623600.8)	197.9(164.9 to 232.4)	490197.1(415356.1 to 571450.5)	142.6(122.1 to 164.7)	-1.13(-1.24 to -1.02)	514403.6(375284.2 to 663562.3)	184.7(134.2 to 237.1)	550043.8(401702.5 to 706747.6)	165.4(120.4 to 212.5)	-0.36(-0.4 to -0.32)
Eastern Sub-Saharan Africa	IS	867800.8(811693.6 to 925063.4)	1066.6(996.2 to 1139.8)	1869299.3(1761941.3 to 1973402.3)	992.1(934.2 to 1049.9)	-0.27(-0.29 to -0.25)	85161.6(72719 to 99207.2)	110.9(94.2 to 130.1)	179317.5(154965.6 to 204334.2)	103.6(89.2 to 118.4)	-0.21(-0.26 to -0.17)	113981.5(83920.3 to 142702)	145.8(109.1 to 181.7)	247641.9(181931.2 to 307559.4)	137.4(102.4 to 170.3)	-0.21(-0.23 to -0.19)
High-income Asia Pacific	IS	2058456.1(1893145.5 to 2220222.2)	1030.5(947.1 to 1109.2)	3295961.7(3048492.7 to 3555085.8)	775.6(723 to 832.2)	-1.04(-1.09 to -0.98)	234946.3(193848 to 280991)	121(100.7 to 144)	284444.7(249282.5 to 322677.8)	64.6(56.4 to 73.7)	-2.53(-2.73 to -2.33)	334299.9(238320.6 to 428823.1)	169.1(120.2 to 216.4)	563941.3(405776.7 to 723399.5)	126.9(90.9 to 163.7)	-1.06(-1.12 to -1)
High-income North America	IS	3403854.6(3107281.7 to 3724886.2)	991.5(904.5 to 1081.9)	5699097.6(5260863.2 to 6152298.3)	950(880.6 to 1024.5)	-0.39(-0.57 to -0.21)	315394.2(259208 to 383545.1)	90(74.4 to 108.3)	352963.3(299100 to 413294.4)	56.9(48.6 to 66.1)	-1.58(-1.73 to -1.43)	481432.5(342541.8 to 620018.5)	139.1(99.4 to 178.2)	792506.2(575212.5 to 1008039.4)	130.4(94.5 to 165.4)	-0.47(-0.65 to -0.3)
North Africa and Middle East	IS	1847829.4(1741882.4 to 1963338.4)	909.3(852.4 to 967.6)	4366507.3(4157817.8 to 4571157.7)	866.3(823.7 to 909.4)	-0.18(-0.2 to -0.17)	199912.2(175527.7 to 229003.4)	117.5(102.6 to 133.5)	461145.6(409815.1 to 516836.6)	103.6(91.6 to 116.1)	-0.43(-0.47 to -0.39)	251672.2(184852.7 to 321035.8)	125.4(92.4 to 159.3)	590461.6(431731.9 to 753591.1)	118.9(87.3 to 151.4)	-0.2(-0.22 to -0.18)
Oceania	IS	26485.1(25138.3 to 27927.3)	809.7(767.7 to 855)	59506.3(57089.1 to 62156.1)	741.4(711.4 to 772)	-0.31(-0.31 to -0.3)	2342.9(2016.9 to 2728.2)	81.5(70 to 94.3)	5311.5(4624.9 to 6003.2)	73.2(63.7 to 82.9)	-0.43(-0.47 to -0.39)	4885.6(3476.4 to 6248.2)	152.4(110.4 to 193.1)	10933.1(7950.6 to 13866)	139(102.1 to 176.3)	-0.31(-0.32 to -0.31)
South Asia	IS	3441863(3041705 to 3832962.8)	526(462.2 to 586.9)	7828397.3(6997064.5 to 8621267.5)	497.7(445.7 to 549)	-0.21(-0.25 to -0.18)	398076.4(331671.9 to 474038.3)	72.6(60.6 to 86.5)	853370.3(727638.3 to 990208.9)	61.2(52.3 to 70.7)	-0.74(-0.82 to -0.66)	475844.7(347227.4 to 607006.1)	74.6(54.8 to 95.2)	1099881.7(811156.8 to 1404190.6)	71.2(52.3 to 90.5)	-0.17(-0.2 to -0.13)
Southeast Asia	IS	2557635.5(2350464.3 to 2762167)	921.2(846.8 to 999.6)	6104966(5651318.6 to 6562857)	919(851.9 to 986.3)	-0.03(-0.04 to -0.02)	265778.9(228020.3 to 310151.1)	107.3(91.5 to 125.6)	667263(582280.6 to 763034.9)	107.3(93.7 to 122.7)	-0.04(-0.07 to 0)	479867.6(344386.4 to 615545.8)	174.7(126.1 to 224.4)	1147516(830589.4 to 1476620.9)	174.7(126.5 to 223.9)	-0.02(-0.02 to -0.01)
Southern Latin America	IS	399118(380518.4 to 419432.1)	859.4(819.6 to 903.1)	546568.7(524188.8 to 570536.2)	640.6(614.8 to 668.1)	-1.05(-1.1 to -1)	44172.3(38720 to 49910.2)	97.8(86.3 to 109.9)	52069.9(46168.9 to 58185)	60.3(53.5 to 67.6)	-1.77(-1.93 to -1.62)	57206.7(41582.9 to 72340.6)	124.3(90.6 to 157.1)	79670.4(57795.5 to 100433.5)	92.6(67 to 116.8)	-1.05(-1.1 to -1)
Southern Sub-Saharan Africa	IS	364210.1(330935.5 to 399540.5)	1274.6(1149.8 to 1404.1)	640169.7(583548.5 to 694855.4)	1122(1019.9 to 1224.9)	-0.49(-0.54 to -0.44)	33295(28141.1 to 39224.7)	122.3(101.9 to 146.7)	64729.4(54456.2 to 74990.5)	121.9(102.5 to 142.2)	-0.11(-0.36 to 0.15)	49904.9(36087 to 63332.6)	179.9(129.3 to 226.5)	87036.1(63129.1 to 109277.9)	157.8(113.2 to 198.6)	-0.5(-0.54 to -0.45)
Tropical Latin America	IS	827663.7(739294.2 to 917728.8)	850(757.8 to 943.6)	1543559.5(1392394.6 to 1692607.6)	605.7(546.6 to 663.9)	-1.24(-1.3 to -1.18)	106000(88155.9 to 125886.1)	119.2(98.3 to 142.3)	166748.8(140997.7 to 194698.4)	66.4(56 to 77.5)	-2.04(-2.16 to -1.92)	69187.4(51836.8 to 88852.1)	73.2(54.6 to 93.1)	134588.6(99756.9 to 171466.5)	53.4(39.5 to 68)	-1.16(-1.21 to -1.1)
Western Europe	IS	4563751.4(4343952.8 to 4819225.2)	806.9(768.4 to 850.6)	5540066.3(5333481.7 to 5752361.9)	625.7(600.1 to 649.9)	-0.86(-0.89 to -0.83)	613766.1(538423.5 to 692797.7)	106(94 to 118)	549190.6(496801.9 to 601605.6)	58.1(52.9 to 63.7)	-2.12(-2.21 to -2.03)	643941.2(465042.1 to 820150.6)	112.2(81.1 to 142.9)	811574.9(595788.3 to 1025596.2)	87.7(63.8 to 111.2)	-0.84(-0.87 to -0.81)
Western Sub-Saharan Africa	IS	1111030.5(1034246.3 to 1188453)	1109.4(1026.9 to 1194.5)	2479408.3(2331911 to 2621732.8)	1046(977.7 to 1112.6)	-0.23(-0.24 to -0.21)	98569.9(83977.2 to 114606.6)	104.1(88.2 to 122.1)	208264.8(181390.6 to 236453.6)	94.7(82.1 to 106.9)	-0.29(-0.33 to -0.25)	147544.7(107322 to 184689)	152.9(112.7 to 191.8)	331107(242067.4 to 414241.4)	146.2(108.3 to 182.4)	-0.17(-0.18 to -0.15)

Andean Latin America	Stroke	244181(236154 to 252779.3)	1002.6(967.9 to 1040.3)	496226.7(481108.9 to 512716.5)	804(779.9 to 831.4)	-0.82(-0.87 to -0.78)	26504.1(24535.4 to 28595.9)	115.3(106.1 to 124.5)	46075.6(42239.1 to 50127)	76.5(70.2 to 83.3)	-1.46(-1.58 to -1.35)	21697.1(16218.7 to 27441.9)	91.6(68.7 to 115.8)	45146.7(33218.4 to 57232.3)	74(54.5 to 93.5)	-0.8(-0.84 to -0.76)
Australasia	Stroke	220497.4(213506.1 to 227974.3)	947.8(917.7 to 980.3)	368278.2(357039.2 to 379889.9)	743.7(720.6 to 769.1)	-0.88(-0.92 to -0.84)	26936.5(25243.4 to 28704.6)	117.4(110.3 to 124.5)	36487.9(33477.1 to 39688.4)	69.6(64 to 75.5)	-1.91(-2.04 to -1.79)	32020.8(23307.9 to 40599.4)	137.8(100.2 to 175)	55337.9(40607.7 to 69972.2)	109.1(79.3 to 138)	-0.87(-0.92 to -0.83)
Caribbean	Stroke	285810.8(276172.7 to 296856)	1015.2(979.7 to 1054.9)	482954.2(467480.1 to 499999.1)	916.6(887.4 to 948.5)	-0.38(-0.41 to -0.36)	35497.6(32779.2 to 38146.4)	133.9(124 to 144.1)	58953.8(54786.7 to 63492.9)	111(103.3 to 119.5)	-0.69(-0.76 to -0.62)	25381.1(18954.6 to 32135.9)	91.3(68.4 to 115.4)	43444(32425.2 to 54609)	82.3(61.4 to 103.5)	-0.39(-0.41 to -0.37)
Central Asia	Stroke	736945.1(708214.8 to 766165)	1451.7(1394.8 to 1511.8)	1119259.1(1081926.5 to 1160256.9)	1281.9(1235.1 to 1330.7)	-0.47(-0.5 to -0.43)	110529(102186.6 to 119687.5)	237.4(219.6 to 256.1)	165934(153819.7 to 178419.8)	216.6(200.7 to 232.7)	-0.34(-0.49 to -0.19)	134587.1(96980.7 to 170278.3)	267.1(193.4 to 338.4)	203293.1(146329.8 to 259234.3)	235.8(170 to 299.9)	-0.46(-0.49 to -0.42)
Central Europe	Stroke	1782941.6(1687986.5 to 1880118.9)	1218.3(1154.7 to 1284)	1890793.4(1797462.6 to 1995682.7)	941.1(897.3 to 986.4)	-0.95(-1 to -0.9)	305510.1(276390.8 to 334458)	217.8(198.2 to 237.2)	301718.3(273240.8 to 328174.9)	138(126 to 149.2)	-1.65(-1.72 to -1.58)	320180.8(231373.7 to 407575.1)	219.5(158.9 to 279.2)	346799.4(250004.9 to 440637.5)	171.3(123.5 to 217.1)	-0.91(-0.97 to -0.86)
Central Latin America	Stroke	1073460.1(1017917.2 to 1126399)	1076.5(1024.7 to 1129.8)	2099762.2(2005678.5 to 2207618)	827.5(789.8 to 869)	-0.99(-1.05 to -0.94)	113930.8(103754.6 to 125004.9)	126.1(114.3 to 138.6)	203866.6(186309.9 to 223014.3)	82.7(75.6 to 90.3)	-1.61(-1.74 to -1.47)	94465.2(69494 to 118795.3)	97.8(72.3 to 122.7)	190349.5(139499 to 242182.6)	75.9(55.6 to 96.8)	-0.97(-1.02 to -0.91)
Central Sub-Saharan Africa	Stroke	387661.2(372090.7 to 404026.3)	1491.6(1426.2 to 1563)	837111(808195.4 to 868179.5)	1289.2(1239.3 to 1343.8)	-0.54(-0.56 to -0.51)	50733.9(46127.8 to 55872.1)	220.5(200 to 244.3)	102567(94093 to 112548.2)	179.9(164.1 to 196.9)	-0.76(-0.8 to -0.72)	50861.1(37622.5 to 64299.8)	204.3(149.4 to 257.1)	110897.4(82462.1 to 139402.5)	179.8(134.4 to 226.2)	-0.46(-0.48 to -0.44)
East Asia	Stroke	11252263.5(10514274.4 to 12081623.1)	1184.4(1101.5 to 1277.7)	27268340.1(25076687.3 to 29587374.1)	1302.7(1204.6 to 1404.1)	0.29(0.26 to 0.32)	1759569.2(1576973.5 to 1975766.8)	227.7(204.2 to 253.1)	4219912.6(3716696 to 4837656)	203.8(180.5 to 230.3)	-0.63(-0.74 to -0.51)	2174089(1546629.6 to 2781275.9)	232.4(164.4 to 297)	5274970.7(3756289.4 to 6833231.9)	253.1(179.8 to 326.3)	0.27(0.24 to 0.3)
Eastern Europe	Stroke	3493914.3(3185792.1 to 3786348.6)	1263.2(1156.1 to 1366)	3632918.4(3341515.9 to 3927824.5)	1123.4(1039.4 to 1208.7)	-0.42(-0.44 to -0.4)	655178.4(567277.2 to 761798)	251.1(219.8 to 287.6)	610359.4(536457.8 to 693426.4)	180.9(160.2 to 204.4)	-1.2(-1.34 to -1.07)	629269.1(455513.2 to 808906)	228(164.3 to 292.6)	657350.8(477853.2 to 842293.7)	202.4(146.4 to 260.2)	-0.41(-0.43 to -0.39)
Eastern Sub-Saharan Africa	Stroke	1314229.7(1253488.4 to 1381295.3)	1491.1(1416.5 to 1571)	2612262.9(2509416.6 to 2719632)	1287.1(1225.6 to 1349.2)	-0.56(-0.58 to -0.53)	176348.9(161275.5 to 192990.5)	220.6(201.5 to 243.7)	310771.2(285228.3 to 338184.4)	173(157.5 to 189.2)	-0.91(-0.98 to -0.84)	173327.2(127112.1 to 217024.6)	204.7(151.9 to 256.5)	347740.5(254655.4 to 434626.2)	179.5(132.9 to 224.4)	-0.48(-0.51 to -0.46)
High-income Asia Pacific	Stroke	3129954.3(2944797 to 3305685.7)	1565.4(1474.3 to 1652.1)	4792697(4538760.8 to 5070582.3)	1188.6(1131.1 to 1250.1)	-1.02(-1.08 to -0.96)	357808.8(315611.7 to 408749.5)	183.5(162.6 to 207.7)	408315.5(371713.9 to 445803.4)	98.7(89.7 to 108.1)	-2.46(-2.66 to -2.26)	515659.5(369021 to 651591.1)	260.4(185.8 to 328.4)	827559.4(593115.9 to 1057719.7)	196.4(140.9 to 251.2)	-1.05(-1.12 to -0.98)
High-income North America	Stroke	4156907.8(3860040.6 to 4491134.3)	1226.6(1141.9 to 1317.1)	7004984.3(6589033.9 to 7466789.1)	1194.8(1125.6 to 1273.3)	-0.25(-0.42 to -0.08)	399086.7(340282.8 to 466911.7)	114.9(99.1 to 133.5)	466207.6(410439.9 to 527804.7)	76.3(67.7 to 85.8)	-1.39(-1.53 to -1.26)	592626.3(421817.6 to 759030.6)	173.4(123.6 to 222)	985263.6(719066.4 to 1253999.3)	165.9(120.6 to 211.3)	-0.32(-0.49 to -0.15)
North Africa and Middle East	Stroke	2578384.8(2460378 to 2696137.6)	1210(1151 to 1270.3)	5573052.9(5372243.1 to 5794177.7)	1074.9(1032.4 to 1119)	-0.45(-0.48 to -0.43)	305469.5(279115.2 to 337160)	172(157.2 to 189.4)	614803.8(560242.9 to 672498.2)	135.5(123.6 to 148.3)	-0.88(-0.93 to -0.83)	355381(258490.4 to 451497.8)	168.7(123.4 to 214.7)	761637.9(557073.9 to 971004.5)	149(109.1 to 190)	-0.47(-0.49 to -0.44)
Oceania	Stroke	45716.6(44164.2 to 47443.1)	1269.3(1222.6 to 1320.7)	99250.6(96398.1 to 102465.6)	1127(1092.1 to 1165.1)	-0.43(-0.45 to -0.41)	5608.5(5173.7 to 6038.8)	191.3(176.5 to 206.2)	12023.5(11188.9 to 12814.4)	160.1(148.6 to 172)	-0.69(-0.74 to -0.64)	8461.5(6046.7 to 10768.9)	239.8(173.4 to 302.5)	18327.9(13258.6 to 23310.1)	212.4(154.6 to 270)	-0.44(-0.46 to -0.42)
South Asia	Stroke	5854732.5(5434644 to 6296783.8)	853.6(790.3 to 920.8)	12592735.4(11788892.4 to 13536968.7)	783.2(732 to 843.7)	-0.31(-0.33 to -0.29)	865083.8(778337.9 to 963087.8)	150.7(136 to 167.5)	1697104.4(1540300.1 to 1860033.8)	117.2(106.3 to 128.2)	-1.04(-1.13 to -0.94)	814979.6(592320.7 to 1038851.5)	121.5(88.3 to 154.8)	1776801.9(1294907.1 to 2253360)	112.3(81.8 to 142.2)	-0.28(-0.3 to -0.25)
Southeast Asia	Stroke	4114338.8(3896233.5 to 4352287.6)	1385.5(1308 to 1471.6)	8864563(8401985.1 to 9372299.8)	1292.1(1223.2 to 1365.5)	-0.27(-0.29 to -0.25)	553393.8(506224.6 to 605962)	214.7(196 to 235.6)	1192653.7(1090117.6 to 1305247.4)	188(173.1 to 204.5)	-0.52(-0.59 to -0.45)	776538.6(555779.6 to 990511.2)	264.5(190.2 to 336.8)	1675756.1(1204360.5 to 2139738.1)	247(178.2 to 315)	-0.26(-0.28 to -0.24)
Southern Latin America	Stroke	625463.2(605302.6 to 648106.3)	1338.5(1295.8 to 1386)	798754.4(773081.5 to 825045.3)	956.2(925.1 to 987)	-1.23(-1.29 to -1.17)	76259(69975.4 to 83096.5)	167.3(154 to 181.8)	82105.3(75749 to 89047.3)	96.8(89.5 to 105.3)	-2.03(-2.18 to -1.87)	89598.8(65481.7 to 113238.8)	193.2(141 to 244.4)	116275.8(84876.5 to 147012.7)	138.1(100.6 to 174.6)	-1.22(-1.28 to -1.16)
Southern Sub-Saharan Africa	Stroke	469700.8(437120.3 to 507506.6)	1581.9(1461.4 to 1712.8)	788654.5(731611.7 to 844689.9)	1339.6(1237 to 1442.7)	-0.66(-0.73 to -0.59)	50552(45239.9 to 56876.2)	179.6(159 to 202.7)	89024.2(78449.1 to 100459.5)	163.7(144 to 184.8)	-0.46(-0.72 to -0.21)	64607.6(46311.4 to 80814.6)	224.3(161.9 to 282.2)	107901.7(78053.1 to 135136.9)	189.7(136.6 to 237.4)	-0.65(-0.72 to -0.59)
Tropical Latin America	Stroke	1258626.7(1168717.2 to 1352459.9)	1226.8(1128.5 to 1326.9)	2104995.6(1949748.7 to 2262246.5)	824.1(762.7 to 885.7)	-1.5(-1.58 to -1.42)	175182.3(154972.9 to 197616.6)	184.9(163 to 210.5)	245437.9(217778.6 to 275023.8)	97.4(86.4 to 109.2)	-2.32(-2.46 to -2.18)	106221.6(79090.8 to 135933.5)	106.4(79.1 to 136.2)	185348.3(138511.3 to 236802.6)	73.2(54.5 to 93.6)	-1.41(-1.48 to -1.33)
Western Europe	Stroke	5753541.5(5527031.9 to 6011759.4)	1045.3(1004.3 to 1090.3)	6857699.7(6651036.6 to 7083693.4)	811(783.7 to 837.5)	-0.88(-0.91 to -0.85)	780854.8(704804 to 861242.7)	137.2(124.9 to 149.8)	717414.7(664324.1 to 771407.7)	77.7(72.1 to 83.6)	-2.03(-2.12 to -1.94)	813114.4(588781.9 to 1035959.2)	145.5(105.8 to 184.5)	1008754.3(737440.4 to 1282738.9)	113.9(82.5 to 144.9)	-0.84(-0.87 to -0.81)
Western Sub-Saharan Africa	Stroke	1636330.4(1555600.7 to 1721770.9)	1540.4(1455.5 to 1631.6)	3531120.4(3383228.6 to 3684837.7)	1392.6(1323.6 to 1460.3)	-0.39(-0.41 to -0.37)	189078.7(173532.9 to 207729.3)	199.4(181.4 to 220.1)	364536.9(335166.5 to 396618.4)	165(151.7 to 179.1)	-0.68(-0.75 to -0.62)	217246.1(158422.8 to 270946.4)	212.3(155.6 to 265.5)	471467.6(345133 to 589816.7)	194.9(143.2 to 243.4)	-0.33(-0.35 to -0.31)
Andean Latin America	SAH	53338.9(50555.1 to 56206.7)	208.6(198.1 to 219.9)	108449.3(103325 to 113846.2)	172(164 to 180.4)	-0.71(-0.75 to -0.68)	4545.3(4013.7 to 5073.1)	16.3(14.4 to 18.4)	7842.2(7001.2 to 8775.5)	12.3(11 to 13.7)	-0.99(-1.05 to -0.93)	4620.2(3438.4 to 6016.4)	18.4(13.7 to 23.9)	9600.6(7118.8 to 12349.9)	15.4(11.3 to 19.8)	-0.67(-0.7 to -0.63)
Australasia	SAH	19830.5(18428.7 to 21454)	88(81.7 to 95.3)	35127(32840.4 to 37589.9)	79.8(74.3 to 85.8)	-0.3(-0.33 to -0.28)	1690.9(1514.2 to 1900.7)	7.6(6.8 to 8.6)	2630.5(2331.8 to 2987.1)	6.1(5.3 to 6.9)	-0.8(-0.85 to -0.76)	2853.6(1997.5 to 3683.8)	12.6(8.8 to 16.3)	5198.3(3747.7 to 6732.1)	11.6(8.4 to 15.1)	-0.25(-0.28 to -0.23)
Caribbean	SAH	44260(41671.3 to 46948.7)	151.7(143 to 160.5)	71255.8(67508.6 to 74882.8)	136.9(129.7 to 143.8)	-0.39(-0.41 to -0.36)	3431.4(3012.4 to 3842.2)	11.5(10.1 to 12.9)	5327.4(4776.9 to 6036.1)	10.4(9.3 to 11.7)	-0.43(-0.5 to -0.37)	3887.2(2833.7 to 5019.7)	13.4(9.8 to 17.4)	6320.6(4668.7 to 8200.2)	12.1(9 to 15.7)	-0.39(-0.42 to -0.37)
Central Asia	SAH	48426.8(44898.3 to 52110.7)	89(82.9 to 95.6)	78142.3(73006.8 to 83491.5)	82.5(77.1 to 88.1)	-0.28(-0.29 to -0.27)	4949.8(4311.6 to 5538.7)	9.3(8.2 to 10.5)	7813.7(6959.8 to 8734.3)	9(8.1 to 10)	-0.05(-0.09 to -0.02)	8725.1(6173.1 to 11204.3)	16(11.3 to 20.7)	13954.2(9977 to 18055)	14.8(10.6 to 19.1)	-0.27(-0.28 to -0.26)
Central Europe	SAH	126143.2(115978.4 to 137367.9)	89(81.7 to 97.1)	113644.9(104927.6 to 123406.1)	71.2(65.3 to 77.4)	-0.83(-0.88 to -0.79)	12991.2(11527.1 to 14599.9)	9.5(8.5 to 10.6)	12712(11517.7 to 13976.9)	7.3(6.5 to 8)	-1.14(-1.23 to -1.04)	22580.7(15854.4 to 29172.2)	16(11.2 to 20.6)	20624.6(14556.3 to 26389.1)	12.9(9.1 to 16.6)	-0.8(-0.84 to -0.75)
Central Latin America	SAH	173017.8(159454.6 to 187101.1)	162.8(151.2 to 175.8)	384033.6(357568.7 to 411853.7)	148.5(138.6 to 159.2)	-0.33(-0.36 to -0.31)	13687(12039.8 to 15342.7)	11.9(10.5 to 13.4)	28466.3(25351 to 32267.7)	11.1(9.9 to 12.5)	-0.28(-0.3 to -0.25)	15056.7(10928.7 to 19409.1)	14.4(10.5 to 18.5)	34194.7(25046.1 to 43639.6)	13.3(9.8 to 17)	-0.29(-0.3 to -0.27)
Central Sub-Saharan Africa	SAH	28247.4(25786.9 to 30696.4)	96.5(88.9 to 104.3)	70727.4(65523.1 to 76565.4)	93.4(86.8 to 100.1)	-0.12(-0.13 to -0.11)	2483.4(2115.2 to 2915.4)	7.8(6.6 to 9.3)	6071.3(5215.3 to 7066.6)	7.3(6.3 to 8.5)	-0.25(-0.29 to -0.22)	3653.5(2669.5 to 4665.6)	12.8(9.4 to 16.2)	9195.8(6556.7 to 11763.7)	12.5(9.1 to 16)	-0.08(-0.09 to -0.07)
East Asia	SAH	1147885.2(1002332.6 to 1288506.2)	108.1(95 to 121.6)	1392548(1241282.8 to 1558662.3)	70.1(62.8 to 78)	-1.59(-1.7 to -1.48)	154857.5(132434.2 to 181540.9)	17.7(15.2 to 20.8)	151816(131564.6 to 176502.4)	7.9(6.9 to 9)	-3.6(-3.97 to -3.22)	221928.1(154645.4 to 289439.9)	21.1(14.7 to 27.7)	269583.8(190560.9 to 352796.5)	13.6(9.6 to 17.7)	-1.62(-1.73 to -1.51)
Eastern Europe	SAH	232592(205338.2 to 264099.8)	88.5(78.2 to 100.1)	244739.3(216483.4 to 276836.1)	85.4(75.9 to 95.6)	-0.12(-0.16 to -0.08)	25111.3(21514.8 to 29212.9)	9.8(8.5 to 11.4)	27737.1(24527.8 to 31834.9)	9.6(8.4 to 10.9)	-0.27(-0.52 to -0.02)	41565.1(29159.6 to 53729.8)	15.8(11.1 to 20.4)	43818.9(30968.8 to 57163.1)	15.2(10.7 to 19.9)	-0.11(-0.15 to -0.07)
Eastern Sub-Saharan Africa	SAH	107959.4(98238.1 to 118535.5)	110.1(100.1 to 120.4)	220523.9(202100.8 to 239702.5)	93.5(86.3 to 101)	-0.58(-0.61 to -0.56)	9670.1(8318.5 to 11211.5)	9(7.7 to 10.7)	18583.9(15962 to 21445.3)	7.3(6.3 to 8.6)	-0.9(-0.97 to -0.83)	13925.5(10184.4 to 17781.9)	14.5(10.7 to 18.3)	28658.9(20461.1 to 36600)	12.4(9 to 15.8)	-0.54(-0.56 to -0.51)
High-income Asia Pacific	SAH	386168.4(343656.5 to 431630.7)	191.5(171.1 to 213.7)	747778.5(674045.6 to 832435.3)	193.6(175.4 to 212.1)	0.05(0.03 to 0.07)	34840.3(30002.7 to 41206.3)	17.3(15 to 20.3)	46617.8(41128 to 53645.4)	14.1(12.3 to 16.4)	-0.73(-0.8 to -0.66)	63139.2(45186.7 to 81405.3)	31.6(22.6 to 40.7)	126595.8(91890.4 to 162503.4)	31.7(22.9 to 40.8)	0.03(0.01 to 0.05)
High-income North America	SAH	305044(273290.3 to 342708.6)	95.1(85.2 to 106.6)	512781(466659.5 to 564387.5)	98.2(89.6 to 107.9)	0.07(-0.03 to 0.17)	22730.4(19663.8 to 26711.3)	7.2(6.2 to 8.4)	33649.1(29722.2 to 38721.7)	6.4(5.6 to 7.3)	-0.55(-0.72 to -0.37)	43747.4(31063.2 to 56654.8)	13.5(9.5 to 17.6)	72626.7(51707.2 to 93625.4)	13.8(9.8 to 17.7)	0.03(-0.07 to 0.12)
North Africa and Middle East	SAH	226025.8(208423 to 245127.6)	94.2(87.3 to 101.6)	422055.6(389157 to 456658.6)	73.4(67.8 to 79.2)	-0.91(-0.95 to -0.87)	20475.2(17698.3 to 23128.1)	8.7(7.7 to 9.9)	32386.3(28114.1 to 36307.4)	6(5.3 to 6.7)	-1.54(-1.63 to -1.44)	31878.1(22266.2 to 41154.8)	13.3(9.4 to 17.2)	59604(42296.6 to 76707.6)	10.4(7.4 to 13.5)	-0.89(-0.93 to -0.85)
Oceania	SAH	7073.1(6576.3 to 7540.6)	162.2(152 to 173)	15490.2(14650.9 to 16406.4)	147(138.6 to 155.5)	-0.37(-0.38 to -0.35)	608.5(531.7 to 686.1)	14.9(13.2 to 16.9)	1269.3(1123.1 to 1415.8)	12.5(11.3 to 14)	-0.7(-0.74 to -0.65)	1301.5(915 to 1676)	30(21.1 to 38.5)	2844.8(2007.7 to 3681.7)	27.1(19.3 to 35.1)	-0.37(-0.39 to -0.36)
South Asia	SAH	691952.2(607644.1 to 780839.7)	89.8(79.2 to 101.1)	1355698(1203895.1 to 1512996.1)	79.2(70.6 to 88.8)	-0.41(-0.44 to -0.39)	79739.4(68836.9 to 92811.4)	10.9(9.4 to 12.9)	140533.7(120483.4 to 162708)	8.4(7.3 to 9.7)	-1.12(-1.23 to -1.02)	95624(68980.8 to 123762.4)	12.5(9.1 to 16.2)	188785.2(133812.3 to 243008.3)	11.1(7.9 to 14.2)	-0.38(-0.4 to -0.35)
Southeast Asia	SAH	423364.9(384441.3 to 464831.2)	128(116.6 to 140.5)	804234.8(736667.9 to 876796.4)	110.6(101.7 to 120.4)	-0.52(-0.55 to -0.5)	42836.3(37087.5 to 48904.6)	13.6(12 to 15.7)	74812.4(65465.6 to 85579.6)	10.9(9.7 to 12.4)	-0.89(-0.96 to -0.82)	79800.3(55899.9 to 103445)	24.2(17 to 31.5)	151196.9(107020.8 to 194807.7)	21(14.9 to 26.9)	-0.51(-0.53 to -0.49)
Southern Latin America	SAH	73435.9(68870.2 to 78718.3)	155.3(145.7 to 166.3)	98117.3(90839.9 to 107907)	122.3(113.6 to 133.7)	-0.83(-0.85 to -0.81)	7571.6(6754.7 to 8608)	16.1(14.4 to 18.4)	8417.5(7546.5 to 9475.4)	10.7(9.5 to 11.9)	-1.52(-1.64 to -1.4)	10288.3(7563.6 to 13194.6)	21.8(16 to 27.9)	13829.4(9999.4 to 17552)	17.2(12.4 to 21.8)	-0.8(-0.81 to -0.78)
Southern Sub-Saharan Africa	SAH	27258.6(24371.5 to 30080.6)	81.4(72.9 to 90.4)	48804.4(44313 to 53405.9)	73.1(66.7 to 80)	-0.41(-0.44 to -0.39)	2096.4(1791.1 to 2422.6)	5.8(5 to 6.8)	3590.2(3112.6 to 4139.8)	5.3(4.6 to 6.1)	-0.41(-0.52 to -0.3)	3663.6(2606 to 4693.2)	11.2(8 to 14.2)	6522.4(4671.9 to 8315.9)	10(7.2 to 12.6)	-0.43(-0.45 to -0.4)
Tropical Latin America	SAH	164919.9(145257.6 to 185464.8)	145.9(128.4 to 165.3)	278850.8(248463.7 to 313442)	108.5(96.8 to 121.8)	-1.16(-1.25 to -1.07)	16719.3(14083.9 to 19461.9)	13.9(11.8 to 16.4)	26001.4(22460.4 to 30289.8)	10.2(8.9 to 11.8)	-1.36(-1.51 to -1.22)	14049.8(10021.5 to 18297.7)	12.6(9.1 to 16.4)	24509(17729.1 to 31740)	9.6(6.9 to 12.4)	-1.08(-1.16 to -0.99)
Western Europe	SAH	503131.5(466769.3 to 541147.9)	100(92.5 to 107.5)	608489.6(569044.5 to 647049)	86.4(80.6 to 92.2)	-0.51(-0.53 to -0.49)	39376(34657.7 to 45305.8)	8.1(7.1 to 9.2)	43945.2(39398 to 49547.9)	6.3(5.6 to 7.1)	-0.9(-0.95 to -0.85)	68166.2(48626.3 to 87227.3)	13.4(9.5 to 17.2)	85135.4(60988.5 to 108836.4)	11.7(8.4 to 14.9)	-0.46(-0.48 to -0.44)
Western Sub-Saharan Africa	SAH	111635.8(101453.3 to 122867.1	93.4(85.4 to 102)	241300.6(219739.5 to 264664.8)	81.6(75.4 to 88.1)	-0.49(-0.52 to -0.47)	8377.8(7164.6 to 9575.6)	6(5.2 to 7.1)	17263(14764.8 to 19802.5)	5(4.3 to 5.8)	-0.81(-0.89 to -0.73)	14633.9(10625.7 to 18541)	12.5(9.1 to 15.8)	31881.3(22729.3 to 40630.1)	11.1(8 to 13.9)	-0.44(-0.46 to -0.42)

		)														

ASR, Agestandardised rates; EAPC, estimated annual percentage change; ICH, intracerebral hemorrhage; SAH, subarachnoid hemorrhage; IS, ischemic stroke; YLDs, years lived with disability

Table S3 Age-standardized prevalence, incidence, and YLDs of stroke, ICH, SAH, and IS from 1990 to 2021 at national levels

		Prevalence (95% uncertainty interval)					incidence (95% uncertainty interval)					YLDs (95% uncertainty interval)				
location_name	cause	nunmber in 1990 (000s)	Agestandardised rates per 100,000 (1990)	nunmber in 2021 (000s)	Agestandardised rates per 100,000 (2021)	EAPC(1990-2021)	nunmber in 1990 (000s)	Agestandardised rates per 100,000 (1990)	nunmber in 2021 (000s)	Agestandardised rates per 100,000 (2021)	EAPC(1990-2021)	nunmber in 1990 (000s)	Agestandardised rates per 100,000 (1990)	nunmber in 2021 (000s)	Agestandardised rates per 100,000 (2021)	EAPC(1990-2021)
Afghanistan	Stroke	97126.6(92333.8 to 102038.4)	1288(1219.6 to 1355.7)	170833.2(164259.4 to 177644)	1098.9(1050 to 1149.1)	-0.59(-0.62 to -0.56)	15115.5(13845.9 to 16681.5)	229.3(211.5 to 250.2)	21823.8(20091.2 to 23672.7)	179.8(165.6 to 195.7)	-0.99(-1.1 to -0.89)	13160.7(9641.9 to 16701.5)	176.2(128.9 to 222.7)	22829.3(16851 to 28893.6)	147.6(108.9 to 186.3)	-0.64(-0.67 to -0.61)
Albania	Stroke	22804.3(21987.5 to 23677.6)	950.2(913.1 to 987.4)	31368.3(29992.2 to 32676.2)	804.3(769.3 to 834.8)	-0.6(-0.66 to -0.55)	3526.6(3277.5 to 3790.1)	180.6(168.2 to 194)	6251.7(5760.3 to 6777.5)	155.3(144 to 166.8)	-0.56(-0.59 to -0.52)	4141.1(2960.5 to 5217.8)	174.7(125.4 to 220)	5806.4(4188 to 7404.3)	148.2(106.4 to 188.3)	-0.59(-0.64 to -0.53)
Algeria	Stroke	203833.7(195235.1 to 212467)	1352.6(1289.6 to 1417.9)	458318.7(442888.8 to 476199.4)	1192.3(1149.2 to 1240.8)	-0.46(-0.47 to -0.44)	24140.4(21948.2 to 26706.1)	195.2(177.8 to 216.5)	51525.5(45937.7 to 57238.7)	147.8(131.1 to 164.1)	-1.18(-1.31 to -1.04)	28111.1(20485.6 to 35525.5)	188.9(138.6 to 240.1)	63585.9(46452.6 to 79988.2)	167.6(122.7 to 211.2)	-0.43(-0.45 to -0.42)
American Samoa	Stroke	419.8(405.5 to 435.2)	1527.9(1469.1 to 1585.8)	621.5(602.6 to 640)	1283(1242.7 to 1323.1)	-0.64(-0.66 to -0.61)	47.1(43.2 to 50.9)	199.8(183.2 to 216.8)	69.8(64.3 to 76.2)	157(145 to 169.9)	-0.94(-0.99 to -0.88)	78.2(55.8 to 100)	289.7(210.8 to 368.7)	114.6(83.3 to 145)	239.5(174.7 to 302.1)	-0.68(-0.7 to -0.65)
Andorra	Stroke	468.8(448.2 to 492.1)	810(774.5 to 849.5)	930.3(894.6 to 968.6)	654(629.7 to 681)	-0.82(-0.86 to -0.77)	48(41.7 to 55.1)	86.2(75.8 to 98.1)	93.4(82.7 to 107)	61.9(55.1 to 70.6)	-1.27(-1.4 to -1.15)	63.8(46.7 to 81.4)	112.2(82.4 to 142.7)	130.7(94.1 to 165.1)	90.5(65.4 to 115.1)	-0.82(-0.86 to -0.77)
Angola	Stroke	75951.1(73024 to 78932.3)	1656.8(1589.1 to 1732.7)	201591(194855.4 to 208986.8)	1452.3(1396.7 to 1514.5)	-0.48(-0.51 to -0.46)	9697.1(8833 to 10618.5)	238.4(215.9 to 263)	22650.1(20712.7 to 24842.1)	182.2(165.7 to 201.3)	-0.99(-1.04 to -0.93)	9979.6(7359.1 to 12496.7)	228.3(168.8 to 287.5)	26734.3(19682.9 to 33596.8)	203.2(151 to 253.7)	-0.42(-0.43 to -0.4)
Antigua and Barbuda	Stroke	565.4(542.8 to 591.8)	1046.4(1005.6 to 1094.7)	917.6(885.6 to 951.4)	869.9(839.1 to 900.9)	-0.7(-0.74 to -0.67)	81.5(75.2 to 88.7)	147.1(135.9 to 159)	106.5(97.3 to 115.2)	107.9(99.1 to 116.1)	-1.16(-1.27 to -1.05)	52.6(39.6 to 66.2)	95.6(71.9 to 120.5)	81.4(60.5 to 102.7)	78.6(58.7 to 99.3)	-0.74(-0.77 to -0.7)
Argentina	Stroke	438250.2(423145.2 to 455057.8)	1357.5(1310.9 to 1408.9)	518441.4(500421.3 to 536513.7)	958.5(925.5 to 991.2)	-1.27(-1.33 to -1.21)	53782.3(49429.1 to 58623.4)	170.3(156.7 to 185.1)	53566.2(49366 to 58290.9)	98(90.7 to 106.8)	-2.1(-2.27 to -1.94)	62794.4(45599 to 79547.1)	195.9(142.1 to 248.4)	75506.6(54757.3 to 95418.8)	138.5(100.3 to 175.1)	-1.25(-1.31 to -1.19)
Armenia	Stroke	34436.1(32950.7 to 35895.1)	1187.1(1134.5 to 1241)	40584.3(39055.6 to 42173.3)	990.2(953.9 to 1029.4)	-0.63(-0.68 to -0.59)	4908.8(4447.3 to 5437.6)	187.9(170.6 to 206.4)	4614.2(4223.2 to 5045.7)	111.4(102.1 to 120.8)	-1.96(-2.1 to -1.82)	6260.3(4436 to 7841.7)	217.7(154.4 to 273)	7486.5(5381.8 to 9427)	182.1(130.5 to 229.4)	-0.62(-0.66 to -0.57)
Australia	Stroke	185525(180269 to 191622.6)	956.2(929.9 to 987.8)	313299.7(305057.9 to 322121.7)	750.5(730.6 to 773.1)	-0.87(-0.91 to -0.84)	22495.1(21234.1 to 23860.3)	117.8(111.3 to 124.6)	30923.7(28452.9 to 33682.1)	70(64.6 to 75.7)	-1.93(-2.06 to -1.8)	26950.4(19720.9 to 34188.9)	139.1(101.8 to 175.8)	47170.5(34743.3 to 59500.6)	110.2(80.5 to 139.1)	-0.87(-0.91 to -0.83)
Austria	Stroke	130905.2(127147 to 134888)	1152.4(1117.6 to 1187.6)	195927.4(190523.5 to 202188.1)	1140(1106.6 to 1177.7)	0.05(-0.04 to 0.14)	17217.1(16007.1 to 18456.2)	145.3(136 to 154.7)	16912.5(15142.2 to 19181.4)	95.7(86.7 to 108)	-1.16(-1.37 to -0.95)	18760.3(13982.7 to 23740.4)	161.8(120.5 to 203.9)	29279.2(21437.2 to 36788.6)	163.2(118.9 to 205.6)	0.13(0.03 to 0.23)
Azerbaijan	Stroke	60606.1(57920.2 to 63568.8)	1099.6(1045.6 to 1159.4)	110330.4(106083.1 to 114681)	1030.7(987.3 to 1076.2)	-0.22(-0.24 to -0.19)	9196.4(8467.4 to 9960.4)	185.7(170.7 to 200.6)	17260(15879.8 to 18708.7)	197.1(182.4 to 213.8)	0.49(0.39 to 0.58)	11106.5(7909.1 to 14123.7)	203.6(145.3 to 258.9)	20081(14397.7 to 25513.6)	190.7(138 to 242.7)	-0.21(-0.23 to -0.18)
Bahamas	Stroke	1856.6(1785.5 to 1930.7)	1027.3(989.3 to 1066.9)	3744.9(3612.9 to 3873.3)	899.9(868.5 to 931.5)	-0.48(-0.5 to -0.46)	213.9(197.4 to 233.4)	130.7(119.7 to 142.9)	401.6(369.9 to 434.3)	103(95 to 110.8)	-0.85(-0.91 to -0.78)	164(121.6 to 207.8)	93.3(69 to 117.9)	327.3(243 to 413.3)	80.7(60.1 to 101.6)	-0.52(-0.55 to -0.5)
Bahrain	Stroke	3088.3(2951.4 to 3245.1)	936.1(896.6 to 980.4)	10012.3(9589.4 to 10445.6)	736.5(707.6 to 769.8)	-0.91(-0.96 to -0.85)	265.5(241.9 to 294)	122.6(113.4 to 133.7)	726(650.4 to 809.8)	79(72.4 to 86.5)	-1.56(-1.64 to -1.48)	416.5(296.5 to 534.3)	128.4(93 to 162.3)	1325(985.6 to 1663.5)	100.3(74.6 to 126.5)	-0.92(-0.98 to -0.87)
Bangladesh	Stroke	619920.2(592037.7 to 649855.6)	1101.6(1051.8 to 1157.1)	1448638.2(1395226.2 to 1510663.3)	986(947.2 to 1028.7)	-0.37(-0.4 to -0.33)	96587.5(89511.6 to 103629.6)	197.8(182 to 213.9)	221157.1(203556.5 to 240970.4)	167.3(154.3 to 181.9)	-0.59(-0.72 to -0.46)	87257.5(63623.2 to 110308.8)	157.9(115.7 to 199.8)	208407(152038.4 to 263604.2)	143.9(104.5 to 182.5)	-0.31(-0.35 to -0.27)
Barbados	Stroke	2891.7(2756.2 to 3041.5)	1045.4(1000 to 1095.9)	4182.1(4026.3 to 4354)	912.1(879.1 to 947.2)	-0.49(-0.54 to -0.45)	406.4(370.1 to 444.4)	140.6(129.7 to 152.3)	502.2(455.8 to 548.7)	105.2(96.5 to 114.1)	-0.97(-1.11 to -0.84)	273.5(204.1 to 349.3)	96(71.4 to 122)	386.7(289 to 490.5)	83.2(62 to 105.2)	-0.52(-0.56 to -0.47)
Belarus	Stroke	168020.5(154348.9 to 180331.2)	1316.9(1211.9 to 1412.8)	176671.5(168219.3 to 186485.3)	1185.3(1132.5 to 1246)	-0.46(-0.59 to -0.33)	28988.8(25860.5 to 32406.5)	230.5(206.3 to 255.4)	27034.1(23943.2 to 30070.7)	174.8(155.4 to 192.8)	-0.95(-1.12 to -0.77)	30549.4(22221.8 to 39624.5)	239.6(174.3 to 309.8)	32197.6(23156.3 to 40885.1)	215.3(155.2 to 272.6)	-0.45(-0.58 to -0.32)
Belgium	Stroke	126244.1(119231.8 to 133205.6)	874.4(828.3 to 922.5)	151254.5(146960.9 to 155425.3)	715.9(695.2 to 737.1)	-0.7(-0.79 to -0.6)	19498.7(17361.1 to 21740)	129.8(116.4 to 143.8)	17143.2(15733.7 to 18562.7)	72.6(66.6 to 78.4)	-1.77(-2.04 to -1.5)	17535.2(12892.9 to 22462.8)	119.8(87.9 to 153.3)	21949.2(16228.7 to 27523.9)	99.7(73.5 to 125)	-0.62(-0.73 to -0.52)
Belize	Stroke	914.3(879.2 to 953.4)	820.9(788.7 to 855)	2559.9(2465.7 to 2652.5)	750.2(723.1 to 778.2)	-0.37(-0.4 to -0.33)	110.7(102.6 to 119.9)	105.2(97.1 to 114.3)	276.7(254.9 to 299.7)	89.2(82 to 96.7)	-0.89(-1.02 to -0.76)	82(60.8 to 103.1)	74.6(55.1 to 93.5)	223(166.1 to 281.7)	67.3(50.3 to 85.3)	-0.41(-0.45 to -0.38)
Benin	Stroke	39426.7(37973.7 to 41029.6)	1620.9(1556.1 to 1690.6)	93476.6(90102.7 to 96777.6)	1392.4(1342.1 to 1443.6)	-0.59(-0.62 to -0.56)	4790.9(4404.6 to 5200.3)	214.5(195.8 to 234.9)	10059.4(9253.8 to 10932.7)	171.8(157.7 to 187.1)	-0.85(-0.91 to -0.79)	5283.4(3925.7 to 6555.2)	224.1(166.6 to 278.4)	12531.3(9170.1 to 15873.4)	195.6(144.5 to 247.1)	-0.54(-0.57 to -0.51)
Bermuda	Stroke	655.4(632.9 to 682.8)	1038(1002.1 to 1081.4)	930.5(896.7 to 964.2)	817.3(789.4 to 845.3)	-0.88(-0.92 to -0.83)	72.8(66.4 to 79.7)	121.3(111.4 to 132.6)	92.4(83 to 101.5)	74.5(67.1 to 81.5)	-1.9(-2.02 to -1.77)	59.4(44.4 to 75.8)	95.4(71.5 to 121.5)	88.8(66 to 112.5)	75(55.2 to 95.2)	-0.88(-0.92 to -0.84)
Bhutan	Stroke	2713.9(2592.7 to 2833.8)	878.2(835 to 924.6)	5089.1(4887.4 to 5302.7)	784(750.6 to 819.4)	-0.45(-0.47 to -0.42)	359.3(324.9 to 394.6)	141(127.8 to 154.8)	666.1(607.5 to 726.5)	110(100.6 to 120)	-1(-1.08 to -0.91)	382.6(274.5 to 485.7)	127.9(91.6 to 162.8)	742.4(535.9 to 945.4)	115.8(83.8 to 147.9)	-0.39(-0.42 to -0.37)
Bolivia (Plurinational State of)	Stroke	39356.4(37876.8 to 40911.9)	1011(969.6 to 1056)	75162.7(72334.2 to 77961.8)	757.2(728 to 786.3)	-1.09(-1.15 to -1.04)	4674.6(4310.2 to 5097.1)	133.1(122.1 to 145.1)	8086.3(7352.9 to 8868.5)	90.7(82.5 to 99.3)	-1.49(-1.6 to -1.37)	3443.3(2525.4 to 4350.8)	91.5(67.3 to 115.4)	6666.4(4893 to 8403.4)	69.2(50.9 to 87.2)	-1.05(-1.1 to -1)
Bosnia and Herzegovina	Stroke	58189.6(55338.8 to 60912.6)	1338.8(1272.1 to 1404.7)	70511(67521.7 to 73899.4)	1269.4(1218.2 to 1322.4)	-0.19(-0.21 to -0.16)	7967.9(7116.6 to 8986.7)	206.6(185.1 to 231.2)	10116.8(8941.1 to 11281.6)	171.3(152.6 to 190)	-0.53(-0.59 to -0.47)	10410.9(7485.7 to 13272.6)	241.8(174.6 to 310.9)	12647.8(9218.9 to 16120.5)	226.7(165.6 to 288.3)	-0.22(-0.24 to -0.2)
Botswana	Stroke	10102.8(9644.5 to 10564.8)	1677.5(1594.8 to 1754.1)	24576.6(23727.3 to 25573.3)	1615(1548.4 to 1690)	-0.19(-0.23 to -0.14)	1304.9(1183 to 1457.9)	241(215.8 to 268.6)	3124.5(2788 to 3454.3)	228.5(204.6 to 254.2)	-0.38(-0.53 to -0.24)	1377.6(1011.1 to 1749.4)	240.1(177.6 to 300.6)	3336.9(2446.5 to 4150.8)	230.3(169.2 to 285.1)	-0.17(-0.21 to -0.13)
Brazil	Stroke	1232538(1143459.9 to 1325429.6)	1230.8(1130.6 to 1332.3)	2052713.6(1898196.3 to 2207058.5)	823.3(760.8 to 885.4)	-1.52(-1.6 to -1.43)	171711.7(151718.9 to 193831.6)	185.9(163.6 to 211.7)	239213(211891 to 268367.2)	97.2(86.1 to 109.1)	-2.34(-2.48 to -2.2)	103925.2(77291.2 to 133046.5)	106.7(79.3 to 136.5)	180753.8(135123.8 to 231165.2)	73.2(54.5 to 93.5)	-1.42(-1.5 to -1.35)
Brunei Darussalam	Stroke	2696.2(2598.5 to 2795.8)	2292.8(2209.3 to 2391.9)	5141.6(4991 to 5320.2)	1384.8(1340.6 to 1433.9)	-1.83(-1.92 to -1.74)	310.4(281.2 to 342.5)	264.9(237.2 to 294.9)	509.1(457.4 to 562.1)	144.7(131.3 to 158)	-2.33(-2.52 to -2.14)	432.4(310.4 to 550.8)	376.1(270.4 to 478.9)	810(589.8 to 1021.7)	225.2(165.5 to 284.7)	-1.85(-1.93 to -1.77)
Bulgaria	Stroke	168701.4(156828.2 to 181579)	1416.1(1320.7 to 1517.5)	159097.7(146482.2 to 172646)	1256.2(1170.8 to 1343)	-0.44(-0.46 to -0.42)	31281.7(28255.8 to 34455.5)	285.5(263 to 310.1)	31161.7(27887.6 to 34241.2)	225.9(205 to 246.4)	-0.86(-0.92 to -0.8)	30169.6(22066.3 to 38759.7)	254.5(186 to 326.2)	28705(21002.4 to 36586.8)	225(164.6 to 286.8)	-0.44(-0.46 to -0.42)
Burkina Faso	Stroke	63139(60701.8 to 65985)	1214.1(1164.6 to 1270.5)	131233.6(126749.8 to 135812.8)	1099.6(1060.8 to 1137.3)	-0.38(-0.41 to -0.36)	6960.6(6384.5 to 7636.7)	148.3(136.5 to 161.8)	14338.3(13228 to 15494.4)	136.5(126.3 to 146.8)	-0.27(-0.31 to -0.22)	8411(6146 to 10538.3)	167.8(123 to 210.2)	17631.3(12907.1 to 22038.5)	154.2(113.9 to 191.3)	-0.32(-0.34 to -0.3)
Burundi	Stroke	48047.9(46078.5 to 50098.7)	1791.4(1702.3 to 1876.4)	76440.6(73656.5 to 79329)	1264.1(1216.4 to 1315.9)	-1.28(-1.33 to -1.22)	6999.2(6407.6 to 7625.5)	279.7(254.7 to 306.8)	9374.7(8612.7 to 10255.5)	176.5(160.8 to 193.2)	-1.83(-1.96 to -1.7)	6452.2(4717 to 8094)	248.1(181.6 to 311.8)	10149.5(7485 to 12721.9)	176.6(129.8 to 220.6)	-1.23(-1.28 to -1.18)

Cabo Verde	Stroke	3649.4(3517.7 to 3788.8)	1494.5(1440.7 to 1553.4)	6765.2(6549.5 to 6996)	1392.8(1349.3 to 1442.4)	-0.28(-0.31 to -0.25)	371.5(342 to 403.8)	149.9(137.5 to 162.8)	660.4(605.7 to 724.4)	144.4(131.9 to 158.7)	-0.2(-0.26 to -0.14)	516.2(379.6 to 644.6)	211.4(155.3 to 265.8)	936.1(692.1 to 1166.9)	196.9(145.5 to 245)	-0.27(-0.3 to -0.24)
Cambodia	Stroke	61632.8(59133.9 to 64450.1)	1137.2(1086 to 1188.7)	144798(140057.7 to 150568.8)	1063(1023.1 to 1108.7)	-0.26(-0.27 to -0.25)	10105.4(9320.9 to 10899.2)	229.6(211.9 to 248.9)	23555(21697.6 to 25455.6)	204.2(189.3 to 221.7)	-0.48(-0.52 to -0.43)	11577(8292.3 to 14909.1)	216(156 to 278.6)	27331.6(19443 to 35262.1)	203(145.8 to 260.7)	-0.22(-0.23 to -0.22)
Cameroon	Stroke	80347.1(77408.2 to 83339.3)	1473.2(1415.4 to 1535.1)	233357.6(225763.2 to 241476.1)	1411.2(1362.2 to 1466.6)	-0.18(-0.25 to -0.11)	9070.2(8339.9 to 9847.4)	190.6(174.4 to 208.3)	25159.5(23272.1 to 27203.9)	178.1(163.2 to 192)	-0.29(-0.44 to -0.14)	10683.6(7838.3 to 13298.3)	204.2(150 to 254.4)	30991.7(22939.4 to 38952.4)	197.2(146.4 to 247.3)	-0.15(-0.22 to -0.09)
Canada	Stroke	388326(375204.8 to 400587.2)	1221.8(1181.2 to 1259.8)	704693.3(690601.5 to 719837.4)	1090.9(1066 to 1116.2)	-0.3(-0.32 to -0.27)	41115(36632.9 to 45889)	129.4(115.6 to 144.2)	54091.6(50512.1 to 57866.6)	81.8(76.4 to 87.1)	-1.24(-1.36 to -1.13)	55632.1(41031.9 to 70813.6)	175(129.1 to 222.5)	103131.3(75444.8 to 129878.3)	156.5(113.9 to 197.2)	-0.27(-0.3 to -0.24)
Central African Republic	Stroke	19363.5(18567.6 to 20193.5)	1457.1(1389.2 to 1528.3)	34859.2(33601.3 to 36168.2)	1292.2(1235.3 to 1352)	-0.44(-0.47 to -0.42)	2764.5(2511.8 to 3047.7)	236.2(214.1 to 261.2)	4687.7(4314 to 5118.4)	203.1(185 to 221.9)	-0.59(-0.63 to -0.55)	2512.9(1857.4 to 3169.9)	198.2(146.7 to 249.6)	4524.9(3364.3 to 5726.2)	177.3(131.6 to 223.5)	-0.4(-0.42 to -0.38)
Chad	Stroke	51237.1(49340.2 to 53441.3)	1539.1(1481.1 to 1608.4)	115323.8(111416.8 to 118989.1)	1449.3(1399.6 to 1502)	-0.22(-0.25 to -0.19)	6093.9(5629.6 to 6627)	197(180.9 to 215.1)	12973.9(11990.7 to 14018.2)	186.6(172 to 200)	-0.19(-0.28 to -0.1)	6887.5(5035.3 to 8591.3)	212.8(157.2 to 264.9)	15184.2(11303 to 19099.6)	199.8(148.6 to 250.2)	-0.22(-0.25 to -0.2)
Chile	Stroke	127286(122623.8 to 132184.5)	1191.1(1146.1 to 1237.9)	229533.6(221613.3 to 237756.7)	940.4(909 to 973.5)	-0.88(-0.94 to -0.82)	15495(14064.3 to 16920.2)	151.6(137.6 to 165.7)	22836.9(20675.8 to 25052.6)	92.4(83.8 to 101)	-1.65(-1.8 to -1.49)	18081.4(13402.5 to 22804.4)	171.3(126.4 to 214.8)	33239.6(24402.3 to 41992.7)	135.5(99.6 to 171.3)	-0.87(-0.92 to -0.81)
China	Stroke	10731080.1(10003053.9 to 11542610.1)	1167.4(1082 to 1262.6)	26335402.6(24154963 to 28625610.9)	1301.4(1200.6 to 1405.7)	0.34(0.31 to 0.38)	1685761.8(1506913.7 to 1897237.4)	226.9(202.9 to 252.8)	4090480.1(3593818.7 to 4699827.9)	204.8(181 to 231.5)	-0.6(-0.72 to -0.48)	2072204.4(1474566.1 to 2652676.5)	228.8(161.8 to 292.8)	5089856.8(3624862.6 to 6591427.3)	252.6(179.4 to 325.9)	0.32(0.29 to 0.36)
Colombia	Stroke	233236.8(225265.9 to 241751.1)	1142.7(1104.5 to 1185.6)	428801.1(415725.4 to 443503.9)	781.6(757.4 to 808.5)	-1.42(-1.49 to -1.35)	25743.6(23808.9 to 27985.7)	136.8(125.6 to 148.3)	42000.7(38338.7 to 45662.4)	75.9(69.3 to 82.4)	-2.22(-2.37 to -2.07)	20552.6(15306.4 to 25807.3)	103.9(78 to 129.6)	40021(29717.9 to 50486.2)	72.6(53.8 to 91.6)	-1.34(-1.41 to -1.27)
Comoros	Stroke	3914.1(3761.5 to 4082.1)	1755.5(1682.6 to 1835.3)	7351.6(7079.1 to 7619.8)	1405.4(1351.7 to 1462.5)	-0.82(-0.86 to -0.78)	482.9(439.2 to 528.5)	233.4(211.8 to 255.9)	832.5(751 to 912.7)	170.6(154.2 to 186.5)	-1.24(-1.34 to -1.13)	522.5(387.7 to 662.4)	243.5(180.8 to 304.1)	1002.1(740.1 to 1245)	197.6(146.7 to 246.4)	-0.76(-0.79 to -0.73)
Congo	Stroke	21961.3(21094.1 to 22910.4)	1850.5(1766.9 to 1933.2)	46074.3(44388.7 to 47736.5)	1508.4(1448.8 to 1573.6)	-0.76(-0.8 to -0.73)	2870.6(2619 to 3181.5)	263.9(239.1 to 292)	5351.5(4838.3 to 5879.6)	196.3(176.9 to 215.1)	-1.16(-1.23 to -1.08)	2915.1(2133.3 to 3663.4)	255.8(188.3 to 320.3)	6108.7(4487.8 to 7598.8)	209.9(155.4 to 261.3)	-0.73(-0.76 to -0.7)
Cook Islands	Stroke	192.8(186.1 to 199.4)	1395(1345.8 to 1448.4)	315.5(306 to 325.7)	1326.9(1285.1 to 1367.8)	-0.2(-0.23 to -0.17)	21.7(20 to 23.6)	172.1(158 to 186.4)	32.3(29.4 to 35.5)	134.4(123.3 to 146.5)	-0.98(-1.09 to -0.87)	36(26.1 to 46.1)	263.5(191.6 to 336.9)	59.2(42.6 to 74.5)	249(179.7 to 313.8)	-0.21(-0.24 to -0.18)
Costa Rica	Stroke	20083.9(19349.1 to 20851.1)	989.3(954 to 1026.1)	44483.7(43022.3 to 46107.6)	822.5(795.6 to 851.9)	-0.7(-0.74 to -0.66)	2061.7(1891.8 to 2264.1)	108.2(98.8 to 119.8)	4119.7(3720 to 4569.7)	75.9(68.5 to 83.8)	-1.38(-1.52 to -1.24)	1808.3(1333.5 to 2308.7)	90.9(67 to 115.6)	4106.7(3007.2 to 5153.5)	75.7(55.4 to 95.2)	-0.69(-0.73 to -0.65)
Croatia	Stroke	100552.5(96502.9 to 104646.1)	1784.1(1711.4 to 1864.8)	223680(216805.9 to 231823)	1495(1440.8 to 1549)	-0.68(-0.72 to -0.64)	10379.5(9557.7 to 11317.7)	214.1(195.3 to 233.5)	22552.5(20857.8 to 24350)	175.8(160.8 to 191.4)	-0.8(-0.89 to -0.71)	12802.8(9475.9 to 16050.2)	239.5(177 to 295.6)	29343.9(21483.1 to 36850.4)	207(152.7 to 257.9)	-0.59(-0.62 to -0.55)
Cuba	Stroke	66155.6(62212.2 to 70417)	1113.6(1044.3 to 1183.4)	72488.3(70340.8 to 74542.3)	884.4(860 to 908.3)	-0.79(-0.84 to -0.75)	12222(11450.7 to 13000.8)	214.9(201.1 to 228.8)	11027.1(10421.6 to 11635.9)	122.3(115.8 to 128.9)	-1.9(-1.98 to -1.83)	11859(8491 to 15252.4)	200.6(143.3 to 259.2)	13290(9692 to 16782.5)	160.7(117.2 to 203.5)	-0.77(-0.82 to -0.72)
Cyprus	Stroke	102274.6(98346.7 to 106260.9)	973(935.2 to 1011.2)	145114.2(139791.6 to 150639.7)	833.9(805.6 to 863.9)	-0.56(-0.6 to -0.53)	12044.6(10985.5 to 13096.6)	118.6(108.4 to 128.8)	17006.3(15537.7 to 18570.1)	91.8(84.4 to 99.7)	-0.9(-0.95 to -0.85)	9206.3(6863.9 to 11677.9)	87.8(65.4 to 111.4)	13357.5(9948.7 to 16818)	75.4(56.1 to 95.1)	-0.55(-0.58 to -0.52)
Czechia	Stroke	7075.1(6762.7 to 7451.4)	857.6(818.7 to 903.3)	9959.2(9465.7 to 10598.9)	521.5(495.7 to 553.5)	-1.93(-2.08 to -1.79)	1036.1(907.7 to 1173.9)	130.6(116.5 to 145.9)	1260.9(1160.8 to 1370.7)	67.5(62.6 to 73.1)	-2.82(-3.16 to -2.47)	962.5(690.7 to 1231.4)	118.4(86.1 to 152.1)	1368.5(983.8 to 1748.5)	71.4(50.9 to 91.1)	-2(-2.16 to -1.84)
C 么 te d'Ivoire	Stroke	168993.2(158408.1 to 180368.1)	1268.3(1193.7 to 1349.7)	184479.2(178036.1 to 191802.7)	937.6(905.8 to 971.8)	-0.95(-0.99 to -0.9)	30586.2(26884.7 to 34298.5)	228.7(203.3 to 254.2)	23181(20453 to 26073.8)	110.1(99.1 to 122.1)	-2.53(-2.62 to -2.44)	30446.1(21966.7 to 38823)	228.3(165.1 to 291)	33980.3(24741 to 42901)	171.2(123.3 to 217.6)	-0.9(-0.94 to -0.85)
Democratic People's Republic of Korea	Stroke	264843(254482.5 to 275383.2)	1560.8(1489.5 to 1634.5)	472425.4(454616.3 to 491781.7)	1458.1(1401.9 to 1523.2)	-0.21(-0.22 to -0.19)	40302.1(37049.6 to 44220.7)	270.7(248.5 to 296.4)	78520.7(72000.1 to 85485)	248.6(228.2 to 269.6)	-0.39(-0.5 to -0.28)	51572.3(36481.4 to 66026.8)	309.4(220.2 to 396.2)	92674.5(65591.3 to 118425.3)	287.9(204.1 to 368.5)	-0.21(-0.23 to -0.2)
Democratic Republic of the Congo	Stroke	256495.5(245567.5 to 267984)	1410.4(1343.4 to 1484.4)	529764(509879.5 to 550475.9)	1215(1164.4 to 1268.6)	-0.55(-0.58 to -0.52)	33628.7(30483.6 to 37321.1)	211.1(191.1 to 235)	67180.2(61636.7 to 73701)	176.9(160.9 to 194.4)	-0.66(-0.7 to -0.63)	33585.1(24860.1 to 42531.3)	192.4(140.7 to 242.3)	70232.4(52153.2 to 88545.2)	169.6(126.6 to 214.9)	-0.45(-0.47 to -0.43)
Denmark	Stroke	88968.9(85606.9 to 91886.5)	1144(1104.8 to 1182.3)	79024.6(76348.2 to 82014.5)	744.7(718.4 to 773.1)	-1.47(-1.54 to -1.4)	11352.9(10380.3 to 12413)	142.3(131.2 to 154.8)	8211.2(7405.3 to 9067)	72.4(65.5 to 80.1)	-2.49(-2.58 to -2.4)	12662.8(9220.9 to 15964.5)	159.3(116.6 to 200.6)	11584.6(8441.3 to 14741.9)	105.3(76.6 to 133.8)	-1.4(-1.47 to -1.33)
Djibouti	Stroke	2873.7(2755.9 to 2998.5)	1681.3(1605.2 to 1753.5)	11148.5(10752.9 to 11542.7)	1529.1(1472.3 to 1588.3)	-0.36(-0.38 to -0.34)	332(302.5 to 366.9)	218.4(197 to 242.3)	1216(1103.9 to 1342.4)	186.6(169.2 to 206.1)	-0.61(-0.66 to -0.56)	376.7(276.2 to 474.2)	234.1(172.6 to 293.2)	1467.5(1073.9 to 1824.7)	213(157.3 to 263.3)	-0.35(-0.37 to -0.33)
Dominica	Stroke	551.2(529.1 to 576.8)	907.9(871.3 to 950.4)	638.5(616 to 663.2)	789.4(761.1 to 820.6)	-0.53(-0.56 to -0.51)	69.3(63.1 to 76)	116.7(106.6 to 127.4)	77.3(69.7 to 84.3)	98.7(89.8 to 107.3)	-0.58(-0.64 to -0.52)	50.3(37.4 to 64)	82.7(61.4 to 105.2)	56.7(42.9 to 71.3)	70.9(53.7 to 88.9)	-0.58(-0.6 to -0.56)
Dominican Republic	Stroke	44701.3(42928.7 to 46508.3)	999.4(962 to 1041.2)	111376.5(107632.2 to 115445.8)	1076.5(1039.6 to 1116.4)	0.27(0.25 to 0.3)	4798.4(4439.9 to 5190.3)	116.8(107.6 to 126.2)	13550.1(12470.7 to 14743.3)	133.5(122.6 to 145.3)	0.53(0.48 to 0.58)	3930.7(2914.9 to 4992.8)	90.4(67.1 to 114.9)	9878(7261.4 to 12502.6)	96.3(70.8 to 121.9)	0.24(0.22 to 0.26)
Ecuador	Stroke	68923.1(66426.3 to 71349.5)	1104.9(1062.8 to 1143.2)	152555(147610.2 to 157633.1)	901.5(872.3 to 932.2)	-0.74(-0.78 to -0.71)	7086.9(6562.8 to 7641)	118.1(108.6 to 127.2)	14355.6(13153.4 to 15615.2)	87.6(80.2 to 95)	-1.01(-1.06 to -0.96)	6106.3(4584.4 to 7659.3)	100.4(75.3 to 125.5)	13738.1(10175.3 to 17412.8)	82(60.7 to 103.7)	-0.75(-0.78 to -0.71)
Egypt	Stroke	370355.1(350962.1 to 391061.7)	1035(973 to 1098.3)	897295.4(858768.2 to 935773)	1154.3(1099.4 to 1209.5)	0.35(0.34 to 0.36)	43995.4(40122.9 to 48696.6)	158(142.3 to 175.3)	104553.8(94514.5 to 117362.5)	163.4(147.9 to 182.2)	0.12(0.06 to 0.18)	51499.6(36889.6 to 65869.3)	145.1(104.2 to 186.9)	121551.1(88284.4 to 155296.8)	158.3(115.4 to 201.5)	0.28(0.27 to 0.29)
El Salvador	Stroke	32942.3(31801.8 to 34185.2)	952.2(918.1 to 985.4)	44986.5(43369.9 to 46788.1)	712.3(687 to 740.7)	-1.1(-1.17 to -1.03)	3795(3505 to 4111.5)	114(105.1 to 124.1)	4867.3(4480.5 to 5324.5)	74.4(68.2 to 81.5)	-1.64(-1.82 to -1.47)	2970.5(2221.9 to 3721.1)	87.5(65.5 to 109.2)	4229.6(3133.2 to 5354.9)	66(49 to 84.1)	-1.05(-1.11 to -0.99)
Equatorial Guinea	Stroke	3423.2(3288.5 to 3570.9)	1595.5(1525.4 to 1667.5)	8314.3(8004.5 to 8673.6)	1308.4(1257.2 to 1360.9)	-0.75(-0.79 to -0.7)	480.6(439 to 530.9)	245.3(222.9 to 270.7)	856(772.8 to 950.1)	157.8(141.4 to 175.3)	-1.6(-1.72 to -1.49)	451.1(334.5 to 568.7)	218(161.8 to 274.7)	1096.6(805.3 to 1375.1)	182.6(135.8 to 229)	-0.67(-0.73 to -0.62)
Eritrea	Stroke	22756(21816.7 to 23712.4)	1617.2(1544.6 to 1694.1)	46997(45283.5 to 48631.1)	1411(1354.8 to 1465.6)	-0.53(-0.55 to -0.5)	3139.6(2880.6 to 3448.6)	264.6(241.4 to 290.1)	5631.4(5195.5 to 6139.7)	189.1(172.9 to 208.1)	-1.31(-1.4 to -1.21)	2970.6(2176.6 to 3751.2)	224.8(167.1 to 281.3)	6220.1(4514.8 to 7808.9)	197.9(144.7 to 248)	-0.48(-0.51 to -0.46)
Estonia	Stroke	22082.9(20527.3 to 23680.9)	1112.3(1036.9 to 1188.7)	17503.9(16558.4 to 18459.7)	772.7(736.2 to 809.1)	-1.35(-1.46 to -1.24)	4248.6(3918.6 to 4637.3)	215.3(199.2 to 234.2)	2285(2030.7 to 2562.7)	89(79.2 to 99.2)	-3.29(-3.51 to -3.07)	3967.9(2857.1 to 5036.2)	199.7(144 to 252.9)	3238.4(2360.3 to 4121.1)	140.9(102.6 to 179.7)	-1.29(-1.41 to -1.18)
Eswatini	Stroke	4385.1(4187.3 to 4592.2)	1335.7(1271.5 to 1407.9)	8062.3(7751.6 to 8387.9)	1327.5(1269.7 to 1389.6)	-0.06(-0.11 to -0.01)	579.4(525.1 to 641.2)	202.4(182.7 to 227)	1057.3(955 to 1160.7)	209.1(188.1 to 230.6)	0.12(-0.05 to 0.3)	600.7(439.8 to 750.9)	192.3(139.4 to 240.8)	1077.9(787.3 to 1338.2)	187.8(138.8 to 233.5)	-0.12(-0.16 to -0.07)
Ethiopia	Stroke	321011.8(296177.1 to 347441)	1345.2(1236.9 to 1460.9)	499603.3(467310.3 to 539141.5)	942.4(872.3 to 1030.5)	-1.36(-1.45 to -1.28)	47257.5(42117.6 to 53250.9)	224.5(200.8 to 250.2)	59344.8(53592.4 to 65293.4)	129.8(116.7 to 144)	-2.13(-2.3 to -1.96)	41786.8(30571 to 53270.7)	182.3(134.6 to 230.5)	67019.1(48183.8 to 84608.6)	131.6(95.5 to 166.3)	-1.24(-1.32 to -1.16)
Fiji	Stroke	7596.8(7314.4 to 7882.7)	1694.4(1632.5 to 1762.5)	11607.1(11237.5 to 11967.1)	1448.4(1401.2 to 1495.1)	-0.59(-0.62 to -0.57)	832(768.2 to 897.8)	218.8(200.3 to 236.4)	1211(1108.4 to 1315.6)	168.5(154.8 to 184)	-1.09(-1.18 to -1.01)	1420.1(1016.2 to 1796.8)	322.4(232.6 to 407.6)	2156.5(1554.5 to 2749.7)	273.3(198.1 to 347.1)	-0.61(-0.64 to -0.59)
Finland	Stroke	98757.8(96124.1 to 101537.9)	1422(1385.4 to 1463.9)	126865.4(123373.2 to 130454)	1110.4(1078.9 to 1141.1)	-0.73(-0.87 to -0.6)	11746.5(10758.2 to 12755)	167.3(154.2 to 181.1)	12177(11051.1 to 13394.2)	99.9(91.2 to 109)	-1.73(-1.96 to -1.5)	13961.9(10226.3 to 17644.4)	199.2(145.7 to 251.1)	18770.2(13809.7 to 23626.5)	156.4(114.8 to 196.9)	-0.7(-0.84 to -0.56)
France	Stroke	599559.6(574307.6 to 624780.4)	760.2(727.8 to 791.1)	928694.4(906899 to 954122.5)	737.2(720.7 to 757.4)	-0.1(-0.12 to -0.07)	73347.1(69199.3 to 78112.6)	88.7(83.9 to 94.2)	94240.5(88308.3 to 100727.3)	68.3(64 to 72.9)	-0.92(-0.96 to -0.87)	86618.9(62721.2 to 109263.9)	107.3(78.1 to 135.2)	139843.4(102095.7 to 174248.4)	105.5(76.5 to 132.1)	-0.05(-0.08 to -0.02)
Gabon	Stroke	10466.5(10040 to 10888.3)	1760.1(1688.1 to 1839)	16508.2(15942.2 to 17098.2)	1481.9(1429.3 to 1541.5)	-0.65(-0.68 to -0.62)	1292.5(1164.2 to 1429.4)	226.2(203 to 249.2)	1841.5(1657.9 to 2015.4)	179.1(161 to 197.5)	-0.93(-1.01 to -0.86)	1417.3(1047.9 to 1769.3)	244.8(181.3 to 305.7)	2200.5(1625.9 to 2790.4)	205.9(151.7 to 257.4)	-0.64(-0.67 to -0.62)
Gambia	Stroke	7888.4(7580.5 to 8216.8)	1687.2(1624.5 to 1759.6)	18893.7(18235.7 to 19540)	1513.6(1464.3 to 1568.6)	-0.41(-0.44 to -0.38)	813.2(745.2 to 887.6)	197.7(180.5 to 216.2)	2003.6(1843.1 to 2174.8)	182(167.3 to 196.7)	-0.28(-0.32 to -0.24)	1039.3(763.3 to 1298.6)	233.2(170.5 to 290.1)	2519.1(1855.6 to 3144.6)	210.5(157.1 to 262.9)	-0.38(-0.41 to -0.35)
Georgia	Stroke	83653.2(80136.6 to 87499)	1365.3(1308.4 to 1427.2)	68177.1(65078.9 to 71355.4)	1286.9(1236 to 1342.6)	-0.24(-0.26 to -0.22)	14898.6(13832.4 to 16202.2)	254.9(237.1 to 275.3)	12565.9(11536.1 to 13591)	212.6(196.7 to 228.7)	-0.5(-0.57 to -0.43)	15396.5(11135.3 to 19528.5)	251.8(181.4 to 319.3)	12545.7(9123.4 to 15892.2)	234.6(170.3 to 297.1)	-0.27(-0.29 to -0.25)
Germany	Stroke	1635384.4(1585038.6 to 1690182.8)	1346.3(1306.1 to 1390)	1961477.2(1908356.6 to 2013776.8)	1093.2(1061.8 to 1123.2)	-0.69(-0.7 to -0.67)	199972.1(182787.1 to 217345.3)	161.1(148.3 to 173.7)	185041.5(167804 to 201859.2)	99(90 to 108)	-1.7(-1.77 to -1.62)	231432.1(169893 to 292041.8)	187.3(137.1 to 236.5)	288214.5(209347.9 to 360563.6)	153.1(111.2 to 192.9)	-0.66(-0.68 to -0.65)
Ghana	Stroke	165822(159620.8 to 171880.8)	2094.5(2007.3 to 2177.3)	422090.6(408863.7 to 435617.4)	2045.8(1977.2 to 2120.1)	-0.12(-0.15 to -0.09)	17190(15837.4 to 18711.7)	242.2(222.1 to 263.9)	42079.6(38672.7 to 45364.8)	226.9(207.2 to 245.5)	-0.14(-0.22 to -0.05)	21996.2(16075.6 to 27582.2)	290.1(213.8 to 361.9)	56280.7(41488.3 to 70849.2)	285.3(211.8 to 356.9)	-0.09(-0.11 to -0.07)
Greece	Stroke	164071.7(156169.2 to 172715.2)	1130.6(1078.1 to 1187.5)	184789.5(177807.4 to 192878)	868.5(837.8 to 900.3)	-0.99(-1.05 to -0.92)	26298.3(23858.9 to 29315.5)	180.9(165.3 to 198.9)	26950(24532.4 to 29496.2)	103.2(93.6 to 112.7)	-2.16(-2.31 to -2)	22923.4(16525.8 to 29089.6)	157.1(113.4 to 199.3)	27248.2(20169.9 to 34426)	120.9(89 to 154)	-0.99(-1.05 to -0.93)
Greenland	Stroke	761.5(728.6 to 801.3)	2075(1974.7 to 2200.4)	841.9(811 to 873.8)	1263.4(1219.3 to 1309.5)	-1.84(-1.97 to -1.71)	81.7(73.3 to 90.6)	266.5(240.9 to 295.7)	83.6(75.8 to 93.4)	138.1(127.1 to 150.3)	-2.59(-2.78 to -2.39)	102.7(74.6 to 129.9)	290.4(212.3 to 367.5)	113(83.4 to 143.6)	174.8(130.1 to 221.8)	-1.88(-2.02 to -1.74)
Grenada	Stroke	846.8(795.8 to 904)	1202.1(1135.9 to 1282.7)	1113.3(1050.3 to 1184.6)	970.2(915.8 to 1030.8)	-0.84(-0.89 to -0.79)	133.4(122.6 to 144.1)	178.6(164.4 to 193)	135.3(124.8 to 146.2)	132.8(123.7 to 142.2)	-1.13(-1.28 to -0.98)	78.4(57.6 to 100.3)	108.4(80.2 to 138)	96.5(71.8 to 123)	86.4(64.3 to 110.8)	-0.89(-0.93 to -0.84)
Guam	Stroke	1220.9(1178.3 to 1264.4)	1378.6(1327.8 to 1430.3)	2692(2600.8 to 2779.8)	1354.9(1311.7 to 1398.2)	-0.03(-0.05 to -0.01)	121.7(110.8 to 132.4)	160.1(146.3 to 174)	257.7(236.1 to 281.5)	126.5(116.6 to 137.6)	-0.96(-1.08 to -0.84)	229.9(163.7 to 293.7)	265.5(191.2 to 339.3)	516.7(373.4 to 652.2)	258.9(187 to 327.7)	-0.06(-0.07 to -0.04)

Guatemala	Stroke	39758(38390.7 to 41327.9)	901.1(869.5 to 935.2)	88742.4(85779.5 to 91970.8)	722.1(698.5 to 748.6)	-0.88(-0.93 to -0.83)	4297.3(3985.6 to 4632.7)	108.9(100.6 to 117.7)	9540.3(8850.2 to 10306.8)	84.6(78.3 to 91.2)	-1.01(-1.11 to -0.91)	3424.7(2547.9 to 4348.1)	81(60.8 to 103.1)	7911.2(5903.5 to 9955.3)	65.7(49 to 82.5)	-0.85(-0.9 to -0.79)
Guinea	Stroke	58562.1(56421.9 to 60860.5)	1571.1(1508.8 to 1637.1)	108202.9(104412.5 to 111902.3)	1524.3(1470.2 to 1579.1)	-0.13(-0.14 to -0.11)	7085.8(6501.8 to 7726.6)	204.1(187.1 to 222.1)	12389.7(11511.4 to 13280.8)	195.7(180.8 to 210.9)	-0.11(-0.15 to -0.07)	7941.8(5822.9 to 10005.7)	218.5(160.2 to 272.6)	14504(10661.4 to 18104.5)	212.1(157.1 to 263.7)	-0.12(-0.13 to -0.11)
Guinea-Bissau	Stroke	9432.3(9076.5 to 9797.6)	1857.2(1782 to 1934)	16184.4(15635.8 to 16722.1)	1617.1(1560.5 to 1675.4)	-0.53(-0.57 to -0.49)	1110.9(1027.3 to 1204.5)	246.6(226.8 to 266.6)	1787.4(1657.3 to 1942.4)	209.1(193.2 to 227.7)	-0.6(-0.65 to -0.54)	1238(903.7 to 1552)	254.3(186.6 to 316.8)	2127.4(1575.7 to 2675.7)	224.9(167.6 to 279.7)	-0.48(-0.52 to -0.44)
Guyana	Stroke	6996.8(6716.9 to 7327.7)	1501.8(1431.7 to 1585.1)	7589.5(7336.5 to 7893)	1097.6(1058.1 to 1145.9)	-1.2(-1.26 to -1.15)	1103.1(1023.2 to 1178.6)	271.6(251.1 to 291.2)	1058.7(980.4 to 1145.1)	169.7(157.5 to 181.8)	-1.66(-1.76 to -1.55)	592.4(444.5 to 748.5)	130.2(98.3 to 164.3)	640.5(480.4 to 813.4)	95.4(71.7 to 121)	-1.19(-1.25 to -1.14)
Haiti	Stroke	46290.3(44522.9 to 48261.2)	1179(1122.6 to 1235.4)	89376.7(86277.3 to 92592.3)	1000.7(961.4 to 1041.9)	-0.6(-0.63 to -0.57)	7144.9(6608.2 to 7700.8)	215.5(198.3 to 232.7)	12549.4(11626.1 to 13515.8)	169.7(157.4 to 183.4)	-0.87(-0.93 to -0.81)	3936.7(2899.3 to 5031.1)	104.1(76.5 to 133)	7600.5(5586.3 to 9664.3)	88.6(65.5 to 112.9)	-0.59(-0.62 to -0.56)
Honduras	Stroke	23070.6(22135.1 to 24051.2)	888.4(849.9 to 925.7)	56034.7(53958.2 to 58319.5)	776.6(746.8 to 809.1)	-0.53(-0.56 to -0.5)	2412.9(2205.6 to 2640.7)	105.6(96.5 to 116.2)	6416.7(5823.3 to 7047.6)	106.1(96.9 to 116.2)	-0.06(-0.12 to -0.01)	2023.7(1514.1 to 2557.7)	80.8(60.5 to 101.7)	4957.4(3662.4 to 6244)	71.2(52.8 to 89.3)	-0.5(-0.53 to -0.47)
Hungary	Stroke	217813.5(208015 to 229612)	1543.9(1477.3 to 1622.1)	162162.3(155130.8 to 169438.1)	936.7(899.4 to 975.1)	-1.83(-1.93 to -1.73)	33681.4(30392.6 to 36879.4)	243(221.4 to 265.8)	22139.8(19917 to 24704)	117.2(104.8 to 128.9)	-2.67(-2.8 to -2.53)	38873(28027.8 to 49634.8)	275.6(198.3 to 351.7)	29509.6(21349.9 to 37615.3)	169.2(121.8 to 215.2)	-1.78(-1.88 to -1.68)
Iceland	Stroke	3243.2(3105.6 to 3378.2)	1145.6(1097.3 to 1192.6)	4295.1(4152.9 to 4441)	799.1(772.9 to 826.9)	-1.24(-1.29 to -1.2)	404.4(360.2 to 456.8)	140.1(124.9 to 157.8)	404(362.8 to 454.8)	71.2(63.6 to 80.2)	-2.5(-2.63 to -2.36)	460.4(333.8 to 583.9)	160.6(116.5 to 203.2)	619.2(454 to 781.5)	112.1(81.9 to 142.2)	-1.23(-1.28 to -1.18)
India	Stroke	4394347.7(4057177.2 to 4750446.9)	790.3(725.8 to 857.8)	9337657.6(8686856.6 to 10109670.3)	727.1(674.6 to 788.5)	-0.31(-0.33 to -0.28)	649982.3(579662.4 to 729602.3)	141.2(126.7 to 157.6)	1250948.8(1127370.8 to 1378326.9)	107.4(96.7 to 118.2)	-1.14(-1.24 to -1.03)	609384.2(443275 to 780875.8)	112.1(81.7 to 142.5)	1315776.8(958132.7 to 1675608.9)	104.1(75.7 to 132.4)	-0.26(-0.29 to -0.24)
Indonesia	Stroke	1918555.3(1778585.6 to 2069379.9)	1636.4(1511.3 to 1788.8)	3942162.9(3639487.6 to 4286186.5)	1526.6(1405.3 to 1662.7)	-0.27(-0.29 to -0.25)	246844.1(222330.1 to 275050)	243.5(218.2 to 272.1)	543349.1(486499.9 to 611194.5)	243.3(218.6 to 270.9)	-0.01(-0.06 to 0.04)	361538.2(257849.1 to 463656.6)	312(222.5 to 400.3)	740782.3(532919.3 to 954773.9)	291.2(208.5 to 375.4)	-0.26(-0.29 to -0.24)
Iran (Islamic Republic of)	Stroke	383202.6(349215.9 to 417620.3)	1097.1(996.5 to 1203.1)	787178.5(726667.8 to 855320.2)	934.3(860.3 to 1015.7)	-0.59(-0.62 to -0.55)	41071.2(36126.6 to 47100.6)	147.7(128.6 to 169.7)	76271.6(67431.4 to 86132.3)	100.8(88.4 to 114.2)	-1.16(-1.25 to -1.06)	52312.6(38100 to 66870.9)	151.7(111.4 to 195)	108041.8(78674.6 to 137730.3)	129.6(94.5 to 164.5)	-0.57(-0.6 to -0.53)
Iraq	Stroke	142908.4(136517.8 to 148867.2)	1383.3(1316.8 to 1448.9)	379406.9(365620.2 to 392808)	1284.1(1231.3 to 1336.9)	-0.26(-0.27 to -0.25)	18758.7(17374.8 to 20333.8)	204(186.1 to 223.6)	45127(40965.9 to 49752.7)	187.5(170.1 to 207.1)	-0.28(-0.31 to -0.25)	19534.7(14321 to 24610.6)	189.8(137.6 to 240.2)	51034.5(37257 to 64854.3)	175.1(128.9 to 222.1)	-0.27(-0.28 to -0.26)
Ireland	Stroke	41594.9(39690.9 to 43612.7)	1033.4(986.3 to 1083.6)	42862.7(41229.3 to 44764.5)	596.3(573 to 621.6)	-2.1(-2.22 to -1.98)	5694.6(5058.8 to 6411)	142(127.1 to 158.5)	4462.8(4029.3 to 4969.6)	58.8(53.1 to 65.5)	-3.57(-3.91 to -3.24)	5784.5(4174.3 to 7349.8)	143.2(103.5 to 181.7)	6017.4(4440.4 to 7703.4)	82.3(60.5 to 105)	-2.12(-2.23 to -2)
Israel	Stroke	53679.2(51865.3 to 55405.2)	1101.4(1065 to 1137)	91775.4(88873.2 to 95269.4)	775.2(749.6 to 803.5)	-1.15(-1.21 to -1.08)	6322.6(5607.6 to 7133)	132.4(118.1 to 148.3)	7943.1(7104.2 to 8895.7)	65.1(58.1 to 73.3)	-2.26(-2.35 to -2.17)	7570.2(5524.7 to 9617.1)	155.7(113.4 to 197)	13215.4(9612.6 to 16732.6)	109.4(79.8 to 138.2)	-1.15(-1.21 to -1.08)
Italy	Stroke	669936.9(617848.9 to 728872.5)	816(757.8 to 879.5)	726983.3(670999.4 to 790216.5)	582.8(543 to 626.3)	-1.22(-1.29 to -1.15)	111624.1(93136.2 to 133772.4)	129.6(110.1 to 152.5)	92278.3(83534.2 to 101776.8)	61.6(56.3 to 67.7)	-2.49(-2.66 to -2.31)	91851.2(65349.1 to 118761.1)	110.9(78.8 to 142.6)	106699.2(76632.7 to 137636.5)	81(58.2 to 104)	-1.13(-1.18 to -1.07)
Jamaica	Stroke	18313.1(17623.7 to 19064.5)	975.6(939.7 to 1015.8)	26164.9(25242.5 to 27195)	853.9(824.3 to 887.9)	-0.5(-0.53 to -0.46)	2643(2431.6 to 2873.3)	141.9(130.6 to 154.1)	3700.2(3393.3 to 3970.7)	117(107.2 to 126)	-0.73(-0.89 to -0.58)	1685.5(1247.6 to 2137.2)	89.2(66 to 113.5)	2388.5(1778.5 to 3012.8)	77.4(57.7 to 97.7)	-0.52(-0.55 to -0.49)
Japan	Stroke	2268614.3(2102192.4 to 2424845.2)	1369.2(1271.4 to 1460.2)	3606917.3(3366842 to 3851853.1)	1176(1104.8 to 1247.5)	-0.57(-0.6 to -0.53)	253732.2(217911.8 to 295738.5)	155.8(134.6 to 179.6)	305158.7(275558.9 to 336315.4)	97(88.1 to 106.6)	-1.91(-2.09 to -1.73)	377114.1(272189.6 to 481324.4)	229(164.6 to 291.7)	632314.2(454263.4 to 809099.7)	194.3(139.3 to 249.8)	-0.62(-0.66 to -0.58)
Jordan	Stroke	26542.4(25285 to 27734.8)	1434(1356.8 to 1508.9)	117502.3(112900.1 to 121888.8)	1367.6(1308.4 to 1427.4)	-0.15(-0.23 to -0.08)	3070.4(2775.5 to 3399.3)	208.8(188.6 to 231.1)	11792.6(10576 to 13006.6)	158.5(140.2 to 175.3)	-1.01(-1.14 to -0.88)	3638.1(2650.1 to 4634.7)	198.4(145.8 to 250.5)	16064.5(11815.6 to 20349.9)	191.3(139.9 to 241.6)	-0.1(-0.18 to -0.02)
Kazakhstan	Stroke	250994.1(239927.3 to 261678.7)	1855.9(1770.8 to 1947.9)	275450.9(265495.8 to 286924.2)	1482.6(1423.3 to 1546.1)	-0.84(-0.94 to -0.75)	34337.2(31341.4 to 37686.1)	278.5(254.4 to 305.6)	37266.6(33934.1 to 40644.4)	227.3(208.1 to 246.7)	-0.78(-0.95 to -0.61)	45583.4(33236.7 to 57763.6)	340.1(247.1 to 432.2)	50102.9(36023.6 to 63470.1)	272.8(196.2 to 346.2)	-0.82(-0.91 to -0.74)
Kenya	Stroke	138507.1(128041.2 to 150518.4)	1371.7(1258.2 to 1504.6)	332118.2(308396 to 358450.6)	1251.2(1153.7 to 1364.1)	-0.37(-0.4 to -0.34)	16253.8(14611 to 18064)	178.6(159.2 to 201.8)	37353.1(33661.6 to 41562.2)	162(145 to 180.4)	-0.36(-0.44 to -0.29)	18373.2(13379.3 to 23227.2)	188.8(138 to 238.9)	44101.3(31906.4 to 55646.2)	174.5(128 to 220.3)	-0.31(-0.34 to -0.29)
Kiribati	Stroke	1039(1007.5 to 1070.6)	2269.5(2193 to 2348.2)	1729.5(1686.1 to 1785.6)	1967.7(1913.2 to 2035.3)	-0.51(-0.55 to -0.48)	141.6(132.2 to 152)	337.1(313.9 to 358.8)	221.6(207.3 to 235.5)	281.8(263 to 300.6)	-0.72(-0.77 to -0.66)	190.1(135.4 to 241.4)	421.2(300.6 to 535.1)	317.3(228.8 to 405.1)	366.9(264.3 to 465.5)	-0.5(-0.53 to -0.46)
Kuwait	Stroke	11569(11046.4 to 12158.5)	1170.7(1122.8 to 1219.3)	42593.4(40894.8 to 44166.7)	1077.2(1038 to 1116)	-0.31(-0.42 to -0.2)	896.2(809.6 to 1001.3)	111.3(100.2 to 122.2)	3331.5(3029.6 to 3664.3)	101(91.4 to 110.9)	-0.28(-0.53 to -0.03)	1575(1148 to 2001.9)	162.6(119.7 to 207.2)	5798.8(4221.5 to 7411.8)	149.7(109.9 to 190)	-0.32(-0.43 to -0.21)
Kyrgyzstan	Stroke	48229.5(46142.2 to 50156)	1510.5(1441.4 to 1574.5)	55873.1(53596 to 58113.5)	1029.5(985.3 to 1072.8)	-1.42(-1.49 to -1.35)	7710.9(7093.6 to 8385.4)	258.4(236.4 to 283.3)	7805.6(7129.2 to 8557.9)	159.7(145.1 to 175.8)	-1.86(-2.05 to -1.67)	8825.1(6217.9 to 11124.5)	277.8(196.1 to 351.8)	10146.5(7258.7 to 12935.5)	189.7(135.2 to 241.1)	-1.41(-1.47 to -1.34)
Lao People's Democratic Republic	Stroke	33341.9(32056.8 to 34795.8)	1382.4(1323.5 to 1449.4)	65532.9(63356 to 67857.2)	1232.5(1184.7 to 1283.3)	-0.44(-0.46 to -0.41)	5272.5(4879.1 to 5706.1)	255.1(235.9 to 276.2)	9431.6(8678.2 to 10198.8)	204.9(187.4 to 222.4)	-0.88(-0.96 to -0.79)	6226.4(4443 to 7936.1)	260.9(188.1 to 337.3)	12286.9(8828.3 to 15743.8)	234.1(168.1 to 299.8)	-0.41(-0.44 to -0.38)
Latvia	Stroke	42496.7(39047.5 to 45701.5)	1227.1(1131 to 1315.8)	38722.6(36528.7 to 40718.9)	1085(1034.6 to 1132.7)	-0.33(-0.45 to -0.21)	9274.7(8363.8 to 10401.8)	266(240.4 to 297.4)	6603(5974.7 to 7281.8)	164.1(148.7 to 179.8)	-1.61(-1.63 to -1.59)	7700.5(5599.8 to 9792.3)	222.2(161 to 281.3)	7186(5211.4 to 9147.4)	198.4(144.2 to 252.4)	-0.27(-0.4 to -0.15)
Lebanon	Stroke	26578.2(25471.7 to 27720.8)	1102.7(1052.7 to 1154)	64665.3(61668.7 to 67708.1)	1086.7(1037.8 to 1135.9)	-0.01(-0.04 to 0.02)	3254.1(2946.8 to 3577.6)	154.1(138.7 to 169.2)	7119.8(6409.1 to 7850.5)	116.9(105.3 to 129.1)	-0.93(-0.97 to -0.88)	3665(2666.4 to 4633.3)	153.9(112.3 to 195.1)	8972.7(6610.7 to 11330.3)	149.8(110.4 to 188.9)	-0.05(-0.07 to -0.03)
Lesotho	Stroke	9551.8(9057.3 to 10016.6)	1066.7(1007.1 to 1126.6)	14325.2(13759.9 to 14948.2)	1274.3(1214.3 to 1340.6)	0.64(0.58 to 0.7)	1411.3(1271.9 to 1562.8)	171.3(154.5 to 190)	2274.2(2056.7 to 2504.8)	234.1(212.2 to 256.7)	1.28(1.1 to 1.47)	1357.9(989.9 to 1714.8)	156.4(114.8 to 196.8)	1949(1433.9 to 2445.9)	181.4(134.4 to 227)	0.53(0.48 to 0.59)
Liberia	Stroke	22065.3(21237.3 to 22923.8)	1588.9(1523 to 1657.4)	38105.6(36706.3 to 39399.4)	1301.3(1256 to 1348.1)	-0.7(-0.73 to -0.67)	2537(2342.5 to 2769.2)	200.2(184 to 219.5)	3886.2(3565 to 4228.3)	154.6(141.9 to 166.6)	-0.87(-0.91 to -0.83)	2886.6(2121.2 to 3611.2)	214.6(157.7 to 266.9)	4943.6(3677.4 to 6160.9)	177.9(131.9 to 220.7)	-0.66(-0.68 to -0.63)
Libya	Stroke	27091.5(26010.4 to 28233.9)	1021.7(977.4 to 1069.9)	66040.1(63730.7 to 68302.9)	1030(990.4 to 1070.1)	0.06(0.03 to 0.1)	2560(2355.1 to 2806.6)	110(100.5 to 120.8)	6148.8(5586.9 to 6751)	111.9(101.6 to 123.8)	0.01(-0.02 to 0.04)	3734(2742.8 to 4769.7)	141.9(105.1 to 180.4)	8986.6(6613 to 11337.4)	141.5(104.5 to 178.6)	0.03(-0.01 to 0.07)
Lithuania	Stroke	47836.3(43642.8 to 52170.6)	1091.7(994.1 to 1190.5)	49790.1(43963.2 to 55916.9)	1018.7(917.5 to 1121.7)	1.33(0.76 to 1.89)	10929.3(9743.1 to 12033.4)	245.2(218.6 to 270.5)	10056.4(8819 to 11377.7)	176.1(156.7 to 196)	-0.95(-1.21 to -0.69)	8629.2(6268 to 11087)	196.8(142.9 to 252.7)	9146.7(6427.9 to 11909.5)	184.7(131.2 to 240.5)	1.44(0.84 to 2.05)
Luxembourg	Stroke	4865.4(4447 to 5301.2)	934.7(858 to 1015.9)	5450.1(5265.1 to 5625.5)	561.4(541.4 to 580.9)	-2.05(-2.2 to -1.91)	707.3(637.2 to 780)	134.8(122.6 to 147.1)	609.2(563.8 to 654.1)	57.7(53.5 to 62.1)	-3.07(-3.41 to -2.73)	676.6(486.1 to 871.5)	129.1(93.2 to 165.6)	782.7(571.4 to 995.1)	78.8(57.6 to 100)	-2.02(-2.16 to -1.87)
Madagascar	Stroke	118576.9(113978.8 to 122754)	2013(1931.2 to 2096.2)	251363.8(243123 to 259274.1)	1813.4(1745.7 to 1883.9)	-0.38(-0.4 to -0.37)	15193.9(14071.4 to 16406.4)	274.9(253.2 to 299.1)	29782.5(27520.5 to 32397.5)	238.2(218.1 to 261.4)	-0.59(-0.66 to -0.53)	15723.2(11539.1 to 19658.9)	276.2(203.3 to 344.7)	33197(24220.7 to 41802.9)	252.6(187.1 to 317.2)	-0.31(-0.32 to -0.29)
Malawi	Stroke	66092.8(63369 to 68979.1)	1459.9(1393.8 to 1527.4)	117460.8(113106.3 to 121580.2)	1302.9(1249.8 to 1354.7)	-0.44(-0.47 to -0.4)	8267.2(7559.5 to 9075.5)	204.8(186.3 to 225.8)	13623.2(12477.1 to 14917.8)	173(157.6 to 189.3)	-0.68(-0.76 to -0.6)	8751(6474.9 to 10898.6)	201.7(150.1 to 250.9)	15653.6(11522.5 to 19749.3)	182.6(136 to 230.4)	-0.36(-0.39 to -0.34)
Malaysia	Stroke	162532.8(156827.8 to 168795.6)	1492.6(1432.8 to 1559)	400837.3(388095.3 to 415065.4)	1356.7(1312.2 to 1405.2)	-0.33(-0.35 to -0.31)	22493.3(20693 to 24312.7)	231.5(211.3 to 252)	44739.3(40576.5 to 49456.2)	163.2(148.4 to 179.7)	-1.17(-1.27 to -1.08)	30467(21876.7 to 38508.1)	282.2(203 to 356.8)	75409.3(54619.3 to 95363.3)	257.7(186.4 to 325.6)	-0.3(-0.32 to -0.29)
Maldives	Stroke	1881.3(1817.8 to 1950.9)	1695.2(1631 to 1769.7)	4251.5(4111.1 to 4408.8)	1013.5(978.5 to 1052.2)	-1.9(-1.99 to -1.81)	236.1(216.5 to 258.6)	249.7(228.5 to 271.7)	464.8(427.5 to 506.5)	131.1(120.4 to 143.1)	-2.49(-2.68 to -2.29)	349.3(254.7 to 450)	320.2(233.8 to 412.1)	786.9(566.9 to 1001.2)	192.2(140.1 to 243.8)	-1.87(-1.95 to -1.78)
Mali	Stroke	65328.5(62905.7 to 68039)	1357.1(1303.5 to 1417.6)	137554(132658 to 142571.2)	1158.1(1118.7 to 1199.8)	-0.61(-0.64 to -0.58)	7696.2(7056.1 to 8457.1)	186.7(171.4 to 204)	14494(13322.1 to 15808)	150.5(139.1 to 162.6)	-0.78(-0.83 to -0.72)	8653.6(6368.4 to 10879.6)	187.1(137.9 to 232.7)	18194(13262.8 to 22720.7)	160.4(118 to 199.8)	-0.58(-0.61 to -0.55)
Malta	Stroke	3844.3(3651.5 to 4069.3)	912.7(867.7 to 965.9)	4905.7(4720.9 to 5112.9)	596.5(573.8 to 619.2)	-1.64(-1.76 to -1.52)	553.4(491.5 to 620.3)	133.7(119.6 to 148.8)	559.8(506.1 to 620.6)	60.2(54.8 to 66.5)	-2.83(-3.13 to -2.54)	527.2(378.5 to 681.6)	125.8(90.2 to 162.4)	697.2(502.8 to 887.5)	81.9(58.6 to 104.1)	-1.66(-1.77 to -1.54)
Marshall Islands	Stroke	358.6(345.9 to 372.3)	1692.1(1621.7 to 1763.5)	668.5(648.9 to 689.8)	1597.9(1544 to 1654)	-0.21(-0.26 to -0.16)	48.6(45.2 to 52.2)	272.8(253.2 to 293.8)	85.3(79.5 to 91.5)	246(229.2 to 264.4)	-0.44(-0.54 to -0.33)	66.9(47.6 to 85.3)	320.1(229.6 to 408.6)	122.2(87.6 to 155)	297.9(216.7 to 377.7)	-0.25(-0.3 to -0.21)
Mauritania	Stroke	21851.7(21077.4 to 22660.6)	1881.6(1808.4 to 1959.6)	36287.9(35099.3 to 37589.2)	1419.6(1372.8 to 1476.5)	-1.05(-1.1 to -0.99)	2430.9(2218 to 2666)	224.3(203.5 to 245.3)	3609(3294.7 to 3930.6)	155.2(140.8 to 169.3)	-1.31(-1.41 to -1.2)	2957.5(2169.7 to 3706.9)	263(193.1 to 329.9)	4947.6(3643.9 to 6224.4)	200.6(147.8 to 249.4)	-1(-1.04 to -0.95)
Mauritius	Stroke	12957.8(12450.6 to 13447.4)	1609.8(1541.4 to 1680.5)	18130.4(17505.8 to 18771.8)	1043.8(1008.5 to 1081)	-1.61(-1.69 to -1.52)	1742.7(1582.6 to 1927.7)	241.5(218.9 to 266.6)	2130.8(1902.4 to 2342.5)	125(112.9 to 136.7)	-2.79(-3.04 to -2.54)	2421.2(1734 to 3084)	303.6(218.3 to 388.2)	3404.9(2485.4 to 4287.5)	196.9(144.3 to 248.3)	-1.6(-1.69 to -1.51)
Mexico	Stroke	564966.6(524714.5 to 602836.9)	1091.1(1014.8 to 1167.3)	1099733.9(1028846.5 to 1179296.3)	854.6(800.5 to 917.6)	-0.93(-1 to -0.87)	57400(50958.7 to 64114.5)	123.8(109.5 to 139.3)	99806.2(88981.3 to 111150.2)	81(72.5 to 89.7)	-1.64(-1.8 to -1.48)	49590.2(36392.7 to 63102.5)	98.9(72.9 to 125)	98673.1(72189.2 to 126557.2)	78(56.9 to 100.2)	-0.93(-0.99 to -0.87)
Micronesia (Federated States of)	Stroke	1041.3(1003.5 to 1081.3)	1815.3(1743.2 to 1893.8)	1342.9(1304.4 to 1386.4)	1628.9(1578.1 to 1684.7)	-0.39(-0.41 to -0.36)	146.3(136.1 to 157.8)	287.8(266.3 to 308.1)	176.1(163.3 to 189.7)	248.1(230.6 to 266.6)	-0.56(-0.6 to -0.53)	194.7(139.6 to 247.4)	343.2(246.3 to 436.3)	248.8(178.2 to 316.1)	307.2(221.8 to 390.2)	-0.4(-0.43 to -0.38)
Monaco	Stroke	733.1(699 to 771)	1130.1(1081.4 to 1182.2)	702.3(671.9 to 735.1)	826.1(795.8 to 861.1)	-1.17(-1.23 to -1.11)	101(88.7 to 114.4)	142.7(126.8 to 160.5)	77.5(68.6 to 87.5)	80.9(71.8 to 90.1)	-2.14(-2.33 to -1.96)	109.6(80 to 140)	161.6(117.4 to 206.5)	103.7(76.1 to 131.5)	116(84.9 to 147.5)	-1.22(-1.29 to -1.16)
Mongolia	Stroke	17286.8(16637.6 to 17953)	1319.3(1262.8 to 1374.9)	36020.2(34837.1 to 37288.8)	1309(1261.9 to 1355.9)	-0.06(-0.18 to 0.06)	2646.5(2468.3 to 2820.2)	238.7(222.9 to 253.9)	5513.6(5165.8 to 5881)	240(225.1 to 255)	-0.07(-0.36 to 0.21)	3122.6(2230 to 4000.1)	240(171 to 305.5)	6450.8(4608.5 to 8200.4)	240(171.9 to 305.3)	-0.02(-0.14 to 0.1)
Montenegro	Stroke	6162.5(5908.9 to 6422.4)	978(938.2 to 1020.4)	7802.6(7491.1 to 8151.5)	873.5(839.2 to 911.8)	-0.39(-0.44 to -0.35)	1303.7(1215.1 to 1398.8)	221.9(207.2 to 237.6)	1813.8(1686.3 to 1959)	207.2(193.9 to 221.8)	-0.26(-0.39 to -0.12)	1126.5(810.8 to 1443.1)	179.8(129.3 to 229.7)	1427.7(1039.4 to 1807.6)	160.1(116.2 to 202)	-0.39(-0.43 to -0.34)

Morocco	Stroke	201871.7(192815.7 to 210935.8)	1175.7(1123.2 to 1234)	412728.4(394698.2 to 431284.8)	1134.3(1081.9 to 1187.4)	-0.16(-0.18 to -0.15)	25382.5(23285.8 to 27918.7)	169.5(155 to 186.7)	52561.1(47526.6 to 58097.7)	159.6(144.7 to 176.1)	-0.25(-0.29 to -0.22)	27644.6(20181.5 to 35178.8)	161.6(118.2 to 204.5)	55763.7(40903.5 to 70494.3)	154.5(113.1 to 194.7)	-0.19(-0.2 to -0.17)
Mozambique	Stroke	102244.7(97573.7 to 106833.5)	1490.3(1415.4 to 1570.8)	220751.9(213390.1 to 229068.5)	1620.7(1553.2 to 1695.2)	0.31(0.29 to 0.34)	14284.6(12999.4 to 15743.6)	230.6(208.7 to 253.3)	29336.1(26999.9 to 31736.6)	238.4(217.2 to 259.1)	0.23(0.17 to 0.3)	13608.9(10039.2 to 16977.2)	206.3(153.2 to 256.6)	28989.8(21385.9 to 36394.4)	223.6(165.4 to 278.9)	0.31(0.28 to 0.34)
Myanmar	Stroke	364314.4(348548.3 to 379496.4)	1370.8(1305.7 to 1433.4)	597161.8(577052.6 to 619884.9)	1159.7(1119.6 to 1203.4)	-0.61(-0.67 to -0.55)	57466.9(52910.6 to 62481.7)	251.4(231.8 to 271.5)	88388.8(81815.3 to 95547.2)	187.8(174.3 to 202.4)	-1.16(-1.32 to -1)	68400.2(49254.4 to 87701.4)	260.1(188.3 to 334.5)	112669.5(80375.7 to 143528.4)	221.1(158.8 to 281.1)	-0.58(-0.64 to -0.52)
Namibia	Stroke	10988.6(10511.6 to 11492.3)	1602.3(1529.2 to 1684)	19358.1(18578.9 to 20125.1)	1345.8(1282.9 to 1410.4)	-0.65(-0.69 to -0.61)	1494.5(1343.4 to 1666.5)	241.5(215.9 to 267.4)	2574.3(2323.5 to 2831.5)	201.2(180.4 to 222)	-0.82(-0.92 to -0.72)	1510.2(1113.6 to 1898.4)	230.6(168.9 to 287.2)	2676.9(1981.9 to 3337.7)	195(143.7 to 243.8)	-0.61(-0.65 to -0.58)
Nauru	Stroke	130.4(125.8 to 134.9)	2244(2156 to 2332.7)	146.5(142.4 to 151)	2027.8(1967 to 2095.1)	-0.38(-0.41 to -0.36)	14.4(13.3 to 15.5)	281.8(258.5 to 306.4)	14.9(13.9 to 16)	208.5(192.7 to 223.7)	-1.21(-1.33 to -1.1)	24.2(17.2 to 30.8)	423.7(300.8 to 535.9)	27.2(19.6 to 34.7)	381.7(277.6 to 482.9)	-0.38(-0.4 to -0.36)
Nepal	Stroke	93579.3(89260.2 to 98152.7)	832.2(789.7 to 877.7)	180894(173409.2 to 189168.3)	714.8(683.4 to 749.8)	-0.56(-0.58 to -0.53)	13644.4(12409.8 to 14959.2)	143(130.2 to 156.4)	26152.4(23760.1 to 28481.4)	117.3(107.5 to 127.3)	-0.73(-0.82 to -0.63)	13088.8(9528.2 to 16788)	119.3(87.2 to 153.3)	25849.3(18917.8 to 32726.3)	103.9(75.9 to 131.1)	-0.5(-0.52 to -0.48)
Netherlands	Stroke	245089.2(236127.5 to 254621.2)	1260.3(1212.4 to 1307)	251372.7(241824.2 to 261370.8)	803.8(773.4 to 834)	-1.62(-1.7 to -1.54)	28187.7(25560.1 to 30923.6)	142.9(129.7 to 156.7)	25539.1(22707.3 to 28778.3)	76.4(68.5 to 85.3)	-2.55(-2.77 to -2.33)	35063.4(25305.5 to 44543.3)	178.3(128.6 to 226.4)	36001.1(26371.6 to 45780.4)	111.9(82.8 to 142.1)	-1.67(-1.76 to -1.58)
New Zealand	Stroke	34972.5(31821.6 to 38057.6)	906(828.1 to 981.3)	54978.4(50289.3 to 60353.7)	707.4(651.2 to 766.6)	-0.92(-1.01 to -0.84)	4441.4(3803.6 to 5148.8)	115.4(100.1 to 132.6)	5564.2(4964.1 to 6221.9)	67.8(60.7 to 75.1)	-1.82(-1.95 to -1.69)	5070.4(3627.2 to 6628.3)	131.3(93.8 to 171.9)	8167.4(5836.7 to 10481.3)	103.3(73.4 to 132.7)	-0.9(-0.98 to -0.82)
Nicaragua	Stroke	19621.4(18883.2 to 20409.5)	1034.8(995 to 1078.1)	43051.3(41521 to 44865.2)	812.6(783.5 to 847.1)	-0.9(-0.94 to -0.85)	2324(2124.2 to 2541)	137.8(125.3 to 150.9)	4511.8(4107.8 to 4932.8)	91.3(83.6 to 99.8)	-1.56(-1.67 to -1.45)	1750.3(1301 to 2219)	95.7(71 to 121.2)	3876.1(2887.6 to 4911.8)	75(56 to 94.3)	-0.9(-0.94 to -0.85)
Niger	Stroke	57657.2(55691.4 to 59991)	1540.6(1479.4 to 1608.9)	142595.9(137646.4 to 147432.7)	1275.5(1230.2 to 1326.6)	-0.72(-0.75 to -0.68)	6727.6(6190.1 to 7370.9)	207.1(189.8 to 226.1)	15993.8(14759.3 to 17354.6)	167.9(154.6 to 181.1)	-0.79(-0.85 to -0.73)	7604.3(5570.2 to 9604.3)	213.6(156.7 to 268.6)	19068.8(14105.9 to 24145.7)	179.5(133.9 to 225.5)	-0.65(-0.68 to -0.62)
Nigeria	Stroke	747432.9(691458 to 807852)	1440.6(1324.4 to 1570.5)	1517505.2(1414787.5 to 1632609.8)	1291.6(1195.4 to 1402.4)	-0.41(-0.43 to -0.39)	90649.2(81687.4 to 100955)	196(175.5 to 220.1)	153216.5(138398.7 to 170293.8)	151.2(136 to 168.1)	-0.96(-1.04 to -0.89)	99469.3(72483 to 125471.4)	198.7(145.4 to 250.9)	203472.7(147859.9 to 256200.7)	181.6(132.8 to 228.9)	-0.33(-0.35 to -0.31)
Niue	Stroke	39.3(37.8 to 40.7)	1791(1722.9 to 1857)	31.8(30.8 to 32.9)	1558.4(1509.7 to 1611.6)	-0.51(-0.54 to -0.48)	5.2(4.8 to 5.7)	230.1(211.8 to 249.4)	3.6(3.3 to 4)	178.3(163.4 to 193.4)	-1.01(-1.09 to -0.93)	7.6(5.5 to 9.6)	343.5(249 to 434.7)	6(4.4 to 7.5)	295.3(213.5 to 371.6)	-0.54(-0.57 to -0.52)
North Macedonia	Stroke	29478.7(27497 to 31599.9)	1544.3(1433.4 to 1664)	39574.5(36419.3 to 43178.5)	1254.3(1165.8 to 1360.8)	-0.8(-0.84 to -0.75)	5292.9(4805.2 to 5858.6)	307.8(281 to 340.1)	7486.8(6722.6 to 8279.2)	281.4(256.8 to 308.2)	-0.34(-0.39 to -0.29)	5315.8(3812.2 to 6822.4)	280.2(199.7 to 358.6)	7098.4(5057.6 to 9034.1)	226.8(162.5 to 288.9)	-0.79(-0.84 to -0.75)
Northern Mariana Islands	Stroke	372.3(358.4 to 386.7)	1462.1(1409 to 1519.7)	645.8(625.2 to 667)	1257.9(1218 to 1300)	-0.57(-0.6 to -0.54)	37.3(34.3 to 40.6)	180.6(164.9 to 196.2)	70.1(63.8 to 76.8)	149.6(137.2 to 162.3)	-0.72(-0.76 to -0.67)	68.3(48.4 to 86.5)	277(199.7 to 349.6)	119.6(86.1 to 152.5)	238.2(171.3 to 303.1)	-0.57(-0.6 to -0.54)
Norway	Stroke	87068.9(78839 to 95682.9)	1341.1(1225.3 to 1459.3)	91232.7(83862.8 to 99175.2)	956.5(883.6 to 1029.8)	-1.24(-1.32 to -1.15)	11091.3(9152.9 to 13425.6)	163.9(137.3 to 196)	9367.9(8131.2 to 10745.8)	93(81.9 to 106)	-2.22(-2.44 to -2)	12301.9(8667.1 to 16017.1)	184.8(130.8 to 238.3)	13272(9503.9 to 17007.9)	134.8(96.3 to 173)	-1.17(-1.26 to -1.08)
Oman	Stroke	13357.9(12770 to 13974.4)	1228.1(1169.5 to 1285.8)	39518.8(38059.6 to 40934.1)	1169.9(1128.2 to 1214.3)	-0.17(-0.22 to -0.13)	1425(1298.7 to 1554.8)	172.1(155.4 to 189.1)	3282(3011.8 to 3580.6)	149.6(135.4 to 163.5)	-0.45(-0.51 to -0.39)	1795.2(1333.4 to 2281.6)	166.4(124.3 to 212.4)	5273.7(3811 to 6755.2)	159.1(115.8 to 202.4)	-0.15(-0.2 to -0.1)
Pakistan	Stroke	744171.4(686544.3 to 806235.2)	1153.2(1058.8 to 1258.9)	1620456.5(1499846.6 to 1746020.3)	1088.1(1000.7 to 1188.9)	-0.22(-0.25 to -0.19)	104510.4(93762.9 to 116776.8)	180(160.6 to 201.3)	198179.9(179699.3 to 218781)	152.8(138.2 to 169.4)	-0.72(-0.84 to -0.59)	104866.4(75744.5 to 134024.8)	165(119.6 to 210)	226026.4(163661.9 to 288259.9)	155.4(112.6 to 196.3)	-0.21(-0.24 to -0.19)
Palau	Stroke	201.2(194.8 to 208.9)	1826.4(1764.8 to 1898.8)	395.5(383 to 407.9)	1754.7(1701.1 to 1811.3)	-0.15(-0.17 to -0.12)	24.6(22.7 to 26.7)	246.9(228.5 to 268.2)	43.1(39.5 to 46.7)	210.6(194.6 to 228.1)	-0.63(-0.68 to -0.58)	37.9(26.8 to 48.6)	348.1(246 to 444.6)	72.6(52.4 to 91)	328(236.2 to 411.2)	-0.21(-0.23 to -0.18)
Palestine	Stroke	11174.9(10705.2 to 11757.1)	985.9(939.7 to 1036.9)	29094.4(27885.4 to 30310.3)	863.4(826.6 to 900.8)	-0.52(-0.55 to -0.49)	1626.5(1487.8 to 1791.3)	182.5(166.4 to 201.1)	3661.9(3362 to 4013.4)	158(144.9 to 171.4)	-0.5(-0.56 to -0.45)	1553.8(1145.4 to 1967.3)	137.9(101.6 to 175.3)	3990.9(2903.1 to 5057)	119.9(87.8 to 151.9)	-0.54(-0.56 to -0.51)
Panama	Stroke	17217.4(16575.7 to 17875)	1023.7(985.8 to 1066.7)	37391.5(36196.5 to 38686.3)	844.5(817.7 to 873.8)	-0.73(-0.78 to -0.69)	2020.5(1848.7 to 2206.6)	130.8(119.2 to 143.1)	4059.3(3679.2 to 4433.6)	90.6(82.2 to 99)	-1.37(-1.46 to -1.29)	1553.2(1161.8 to 1954.9)	94.5(71.1 to 119.4)	3486.8(2616.7 to 4394.5)	78.4(58.8 to 99)	-0.72(-0.76 to -0.68)
Papua New Guinea	Stroke	22835.1(21917.3 to 23858.4)	1028.3(985 to 1074.6)	59058.2(57105.5 to 61243.6)	951(917 to 987.1)	-0.31(-0.32 to -0.29)	2821.7(2564.4 to 3066.6)	163.2(149.1 to 178)	7162.1(6629.5 to 7707.3)	141.9(131.4 to 153.4)	-0.55(-0.61 to -0.49)	4187.6(3006.1 to 5324.7)	192.6(138.9 to 242.6)	10829.1(7815.8 to 13797)	178.1(130.2 to 226.8)	-0.3(-0.32 to -0.28)
Paraguay	Stroke	26088.7(25010.9 to 27151.8)	1056.1(1012 to 1103.6)	52282(50219.8 to 54368.3)	855.9(822.7 to 891.1)	-0.81(-0.84 to -0.77)	3470.6(3199.1 to 3778.6)	148.4(135.3 to 162.5)	6224.9(5654.9 to 6749.4)	106(96.5 to 115.2)	-1.25(-1.32 to -1.17)	2296.4(1718.9 to 2908.6)	94.8(71 to 119.9)	4594.4(3393.2 to 5818.6)	76.5(56.9 to 96.5)	-0.81(-0.85 to -0.78)
Peru	Stroke	135901.6(131231.7 to 141060.1)	953.4(920.4 to 991)	268509.1(259891.3 to 277430.9)	767.8(743.1 to 793.5)	-0.81(-0.86 to -0.76)	14742.7(13590.1 to 15977.3)	109.4(100.2 to 119.2)	23633.8(21605.3 to 25877.4)	68.1(62.4 to 74.6)	-1.69(-1.85 to -1.53)	12147.6(9080.8 to 15494.4)	87.6(65.5 to 111.1)	24742.2(18245.6 to 31352.2)	71.2(52.4 to 90.1)	-0.78(-0.82 to -0.73)
Philippines	Stroke	345944.7(321245.2 to 373863.2)	968.5(899.8 to 1055.4)	1051962.8(980977.1 to 1134429.3)	1158.5(1074.2 to 1257.4)	0.7(0.62 to 0.77)	37901.3(33912.1 to 42208.1)	124.4(110.9 to 139.4)	132877.1(120545.6 to 147795.1)	159.1(143.8 to 176)	0.95(0.69 to 1.21)	64523.3(45858.2 to 83177.5)	182.3(130.2 to 235.1)	196904.4(141312 to 251839.1)	219.4(156.8 to 280)	0.72(0.65 to 0.8)
Poland	Stroke	385295.6(355356.7 to 417203.4)	900.5(833.4 to 973.5)	484802.2(440952 to 535182.8)	749.2(688.2 to 811.2)	-0.78(-0.88 to -0.68)	65633.4(57010 to 75681)	158.6(138.3 to 180.7)	72341.4(62221.6 to 82838.2)	102.9(89.6 to 117.2)	-1.61(-1.7 to -1.51)	69169(49283.7 to 88864.5)	161.9(115.3 to 208.4)	89657.1(63101.3 to 115299.1)	137.4(96.9 to 175.6)	-0.72(-0.81 to -0.62)
Portugal	Stroke	175961.9(162608.2 to 190068.2)	1296(1203.8 to 1392.5)	121105(115950.7 to 126771)	579.9(557.4 to 604.3)	-2.91(-3.05 to -2.76)	29140.8(26401.7 to 32284.1)	221.7(202.7 to 242.4)	17984.4(16566.4 to 19519.9)	69.4(63.7 to 75)	-4.53(-4.88 to -4.18)	24289.6(17828.4 to 31054.5)	179.3(131.9 to 229.5)	17498.1(12782 to 22220)	80(58.3 to 101.2)	-2.92(-3.07 to -2.76)
Puerto Rico	Stroke	30611.5(29461.4 to 31881.8)	845.5(814 to 880.8)	43918.5(42434.6 to 45481.8)	745.2(718.3 to 772.4)	-0.41(-0.5 to -0.33)	3144.9(2857.5 to 3461.3)	89.9(82.2 to 99)	4063.6(3676.3 to 4470.1)	60.6(54.9 to 67.4)	-1.55(-1.65 to -1.44)	2776(2057.6 to 3554.7)	77(57.1 to 98.6)	4283.5(3166.3 to 5384.5)	68.1(50 to 86.1)	-0.42(-0.51 to -0.34)
Qatar	Stroke	3782.8(3639.6 to 3949.2)	1610.2(1546.8 to 1670.1)	21884.5(20946.6 to 22905)	1012.2(972.7 to 1054.2)	-1.62(-1.79 to -1.45)	316.1(288.8 to 347.6)	174.7(160 to 191.8)	1456.8(1278.9 to 1666.5)	110.6(101 to 122.6)	-1.65(-1.9 to -1.39)	500.9(366.4 to 640.1)	220.5(160.7 to 281.1)	2871.5(2126.6 to 3637.6)	137.2(101.8 to 172.9)	-1.65(-1.82 to -1.48)
Republic of Korea	Stroke	813629.1(787453.4 to 843561.2)	2552.2(2468.3 to 2653.2)	1097372.3(1062648.1 to 1135171.7)	1240.9(1203.5 to 1281.2)	-2.62(-2.79 to -2.46)	98887.9(89815 to 109104.9)	333.3(302.6 to 366.4)	96104(85891.5 to 106089.8)	109.7(99 to 120.8)	-4.23(-4.49 to -3.97)	130737.6(94531 to 165340.3)	421(305.1 to 534.4)	180566.2(130583.9 to 230567.2)	204.2(147 to 260.5)	-2.63(-2.8 to -2.47)
Republic of Moldova	Stroke	47500.5(45060.2 to 49797.1)	1040.7(987 to 1093.3)	52569.5(50081.5 to 55078)	946.5(906 to 988.8)	-0.32(-0.35 to -0.29)	8967.3(8198.1 to 9717.6)	218.5(200.7 to 236.2)	8583.5(7692.6 to 9501)	148.8(134.3 to 163.8)	-1.27(-1.35 to -1.19)	8571.6(6216.7 to 10916.5)	188.6(137.1 to 240.3)	9562.4(6943.3 to 12170.1)	171.8(124.1 to 218.7)	-0.31(-0.34 to -0.28)
Romania	Stroke	362084.1(338401.9 to 382203.6)	1316.5(1234.9 to 1391)	368874.8(351165.5 to 387194.1)	1082.8(1038.8 to 1126.9)	-0.73(-0.77 to -0.69)	65589.2(58674.7 to 73060.9)	255.8(232.2 to 281.4)	63903.5(57646.6 to 69845.2)	170.5(155.6 to 185.7)	-1.52(-1.63 to -1.42)	64879.6(47533.2 to 83157.2)	237.1(173.4 to 303.6)	67991(49469.5 to 86170.4)	197.8(144.4 to 251)	-0.67(-0.71 to -0.63)
Russian Federation	Stroke	2197815.8(1993185.3 to 2409390.6)	1217.7(1110 to 1329.3)	2453768.6(2247378.6 to 2670137)	1102.1(1014.4 to 1196)	-0.44(-0.48 to -0.4)	417088.1(359292.5 to 489676.7)	250.6(218.7 to 287.6)	421513.9(368384.8 to 480718.4)	183(161.1 to 207.4)	-1.13(-1.29 to -0.97)	394669.5(284328.8 to 509408.9)	219.3(158.3 to 282.3)	443432.3(321470.6 to 567251.6)	198.5(143.1 to 254.8)	-0.43(-0.47 to -0.38)
Rwanda	Stroke	61529.2(59053.5 to 64202.5)	1854.7(1767 to 1953.8)	87783.7(84672.2 to 91015)	1216.5(1163.7 to 1268.4)	-1.59(-1.67 to -1.5)	9238.9(8447.4 to 10084.3)	307.6(279.7 to 336.9)	10842.1(9831 to 11885.8)	173.6(157.5 to 190.2)	-2.34(-2.56 to -2.12)	8209.7(6038.1 to 10362.7)	257.6(188.7 to 326.7)	11871.5(8685 to 14849)	173.1(127.2 to 216.9)	-1.47(-1.54 to -1.39)
Saint Kitts and Nevis	Stroke	590.4(553 to 632.1)	1623.3(1530 to 1727.4)	734.2(706.7 to 767.4)	1041.7(1002.3 to 1090.7)	-1.67(-1.76 to -1.58)	91.8(84.7 to 100.8)	255.4(237.5 to 276.1)	93.8(85.8 to 103)	150.3(138.5 to 163.3)	-2.03(-2.2 to -1.85)	54(40.4 to 68.6)	146.6(109.9 to 186.2)	63.1(47.6 to 80.2)	93.4(70.4 to 118)	-1.69(-1.78 to -1.61)
Saint Lucia	Stroke	1189.9(1132.4 to 1256.3)	1245(1185 to 1315.6)	2150.2(2078.6 to 2234.9)	932.5(900.6 to 967.5)	-1.04(-1.1 to -0.99)	164.9(151.1 to 179.2)	191.8(176.3 to 207.8)	261.8(241.1 to 283.6)	114.2(105.6 to 122.9)	-1.97(-2.13 to -1.8)	106.3(79.2 to 134.2)	112.8(83.9 to 142.8)	191.7(142.1 to 241.1)	83.5(61.9 to 105)	-1.08(-1.14 to -1.03)
Saint Vincent and the Grenadines	Stroke	870.8(830.4 to 917.1)	1116.4(1062.7 to 1178.6)	1223(1179.5 to 1270.5)	882.8(851 to 916.8)	-0.89(-0.94 to -0.84)	110.5(101.2 to 120.7)	151.7(139.3 to 165.6)	155.7(142.4 to 168.8)	115.6(106.8 to 124.6)	-0.99(-1.09 to -0.9)	78.1(58.1 to 99.3)	100.8(74.3 to 128.7)	107.2(80.1 to 134.9)	78.1(58.5 to 97.9)	-0.95(-1 to -0.91)
Samoa	Stroke	1626.5(1568.6 to 1688.2)	1653.7(1588.9 to 1716.3)	2479.2(2412.4 to 2555)	1576.9(1530.4 to 1629.1)	-0.16(-0.19 to -0.14)	201.7(185.9 to 219.3)	230.4(212 to 251.2)	284.4(264.1 to 305.3)	191.8(176.8 to 206.8)	-0.67(-0.71 to -0.64)	306(218.8 to 391)	315(226.6 to 402.4)	465.3(337.8 to 589.3)	299(217.7 to 376.7)	-0.18(-0.2 to -0.15)
San Marino	Stroke	332(317.2 to 348.6)	978.8(937.7 to 1025.3)	496.8(475 to 519.4)	748.9(719.7 to 779)	-0.99(-1.04 to -0.94)	40.2(34.9 to 45.9)	115.1(101 to 130.7)	53.7(47.6 to 60.7)	71.6(63.4 to 80.9)	-1.81(-1.92 to -1.69)	47.8(35 to 60.9)	138.6(101.5 to 177)	73.9(53.2 to 93)	105.4(76.2 to 133.2)	-1.01(-1.06 to -0.95)
Sao Tome and Principe	Stroke	1406.8(1357.3 to 1463.7)	1943.8(1872.5 to 2028.8)	2548.1(2465 to 2632.1)	1867.4(1806 to 1934.6)	-0.16(-0.2 to -0.13)	145.5(133.2 to 158.5)	212.9(194.4 to 232.4)	249.3(229.7 to 270.3)	202.7(186.2 to 220.1)	-0.24(-0.32 to -0.16)	192.5(142.9 to 241.3)	272.1(202.2 to 339.7)	341.3(251 to 426.7)	260(192 to 324.6)	-0.17(-0.21 to -0.14)
Saudi Arabia	Stroke	84102.2(80365.8 to 88198.1)	982.4(933 to 1031.9)	278384.7(267541 to 288945.4)	965.2(924.9 to 1006.4)	-0.13(-0.15 to -0.1)	10607(9705.7 to 11650.2)	166.3(152 to 182.3)	28005.8(25498.7 to 30595.8)	130.7(119.4 to 141.7)	-0.87(-1 to -0.75)	11368.8(8325.5 to 14474.6)	133.5(99.3 to 170)	36818.5(26803.7 to 46999.8)	129.9(95.3 to 165.3)	-0.16(-0.19 to -0.13)
Senegal	Stroke	69320.5(66615.8 to 72022)	1741.9(1673.3 to 1813.5)	139328.7(134657.1 to 144098.7)	1481.8(1432.6 to 1534.8)	-0.59(-0.62 to -0.57)	7280(6635.5 to 8016.9)	198.1(179.8 to 217.5)	13787.1(12691.1 to 14962.6)	163.1(149.3 to 177.5)	-0.63(-0.66 to -0.6)	9238.6(6839.6 to 11549.2)	240.8(178.5 to 301.1)	18762.7(13820.5 to 23590)	207.3(154.8 to 259.1)	-0.55(-0.57 to -0.53)
Serbia	Stroke	151235.7(140172.7 to 163015.1)	1358.4(1259.9 to 1468.6)	151578.4(140681 to 164520.2)	1006.3(941.8 to 1079.9)	-1.13(-1.19 to -1.08)	26465.6(23634.2 to 29432.5)	265.5(241.2 to 291.4)	31800(28992.4 to 34874)	194.2(178.3 to 211.2)	-1.26(-1.37 to -1.16)	27155.5(19876.6 to 35559.7)	245.7(179.2 to 320.8)	27473.2(19678.5 to 34617.7)	181.6(130.9 to 228.2)	-1.15(-1.2 to -1.09)
Seychelles	Stroke	793.4(760.7 to 827.3)	1354.4(1297.8 to 1413.4)	1338.9(1291.5 to 1388.5)	1136.3(1095.7 to 1179.6)	-0.64(-0.67 to -0.61)	102.9(94.4 to 112.5)	179.1(164.2 to 195.6)	148.2(134.4 to 163.5)	133(121 to 146.4)	-1.08(-1.17 to -0.99)	150.3(107.6 to 191)	256.3(183.4 to 324.8)	247(178.3 to 313.3)	211.6(153.4 to 267.3)	-0.68(-0.71 to -0.66)
Sierra Leone	Stroke	42422.5(40942.1 to 44103.3)	1727.7(1665.9 to 1802.6)	76603(73988.9 to 79114.9)	1549.5(1496.1 to 1605.4)	-0.4(-0.42 to -0.39)	4641(4252 to 5089.8)	203(185.6 to 221.7)	7793.3(7186.3 to 8423.8)	177.9(163.5 to 192.5)	-0.42(-0.46 to -0.37)	5645.5(4189.9 to 7032.2)	236.5(174.7 to 293.7)	10183.3(7532.4 to 12725.1)	214.8(159.2 to 267.6)	-0.35(-0.37 to -0.34)
Singapore	Stroke	45014.6(43411.9 to 46761.4)	1945.7(1874.8 to 2026.6)	83265.7(80409.5 to 86242)	1000.4(966.6 to 1035.1)	-2.38(-2.5 to -2.26)	4878.3(4374.9 to 5393.8)	213.5(191.9 to 235.5)	6543.8(5807.3 to 7439.2)	78.8(70.4 to 88.8)	-3.83(-4.09 to -3.58)	7375.3(5294.8 to 9392.3)	327(235.4 to 417.9)	13868.9(10067.7 to 17638.6)	167.8(121.2 to 213.2)	-2.4(-2.52 to -2.28)

Slovakia	Stroke	96017.4(92762.9 to 99758.5)	1624.5(1568.7 to 1687.2)	106246.4(101627.1 to 110737.3)	1185.6(1134.7 to 1232.2)	-1.03(-1.14 to -0.92)	12922.7(11345.4 to 14425.4)	219.6(193.8 to 244.4)	12675.6(11164.7 to 14309.5)	137.8(122.1 to 154)	-1.57(-1.77 to -1.37)	17660.3(12643.4 to 22205.5)	299.2(214.6 to 375.4)	19682(14330.4 to 24834.3)	219(159.1 to 275.8)	-1.01(-1.12 to -0.9)
Slovenia	Stroke	21497.4(19987.8 to 23126.9)	902.5(840.4 to 968.3)	24286.5(23420.2 to 25199.4)	624.3(602.2 to 646.3)	-1.27(-1.37 to -1.16)	4151(3712.4 to 4611.2)	174.2(156.4 to 192.7)	3427.4(3119.5 to 3746.3)	76.5(70.1 to 83.1)	-2.77(-3.05 to -2.49)	3854.1(2782.8 to 4918.1)	162(117.1 to 206.7)	4483.1(3252.1 to 5689.5)	113.9(82 to 144.7)	-1.21(-1.33 to -1.1)
Solomon Islands	Stroke	3371.3(3252.6 to 3499.2)	1988.1(1901.4 to 2081)	8117(7875.1 to 8366.9)	1920.6(1851.1 to 1985.5)	-0.13(-0.15 to -0.11)	511.2(472.9 to 549.7)	369(341.4 to 395.6)	1272.5(1194.4 to 1351.5)	355(332.7 to 378.1)	-0.2(-0.24 to -0.16)	632.6(454.8 to 808.3)	380.6(272.9 to 484)	1523.7(1084 to 1937.3)	367.6(263.7 to 469.5)	-0.13(-0.15 to -0.11)
Somalia	Stroke	48025.8(46083.1 to 50111.9)	1462.8(1386.7 to 1538.9)	107198.5(103389.5 to 111428.2)	1252.8(1199.5 to 1309.8)	-0.56(-0.58 to -0.53)	6617.1(6061.8 to 7244.5)	232.1(211.3 to 256.4)	13455.9(12368 to 14600.4)	184.4(170.2 to 200.3)	-0.86(-0.92 to -0.81)	6220.9(4617.3 to 7889.2)	199.8(148.2 to 253.9)	13964.1(10259.4 to 17499.3)	173.1(128.1 to 216.5)	-0.51(-0.53 to -0.48)
South Africa	Stroke	380009.6(350189.1 to 414046.7)	1682.6(1535 to 1843.2)	620739.2(568784.2 to 673535.5)	1333.4(1215.9 to 1448.3)	-0.91(-0.98 to -0.83)	39770.3(35296.8 to 45193.4)	183.6(160.4 to 208.9)	68729.1(59851.8 to 78606.4)	158.3(137.8 to 180.7)	-0.71(-1 to -0.42)	52274.3(37525.5 to 65598.1)	237.7(171.3 to 299.6)	85104(61371.1 to 106546.5)	188.3(134.9 to 235.8)	-0.9(-0.97 to -0.83)
South Sudan	Stroke	41118.2(39468.6 to 42908.7)	1427.9(1371.9 to 1493)	55195.8(53129.4 to 57408.4)	1201(1151.9 to 1250.5)	-0.59(-0.61 to -0.57)	5318.9(4843.5 to 5871.8)	199.4(181.5 to 220.5)	6405.8(5834.5 to 7014.8)	159.8(145.5 to 175.2)	-0.73(-0.79 to -0.67)	5345.5(3958.4 to 6658.5)	191.3(142.2 to 238.3)	7159.5(5293 to 8902.2)	162.3(120.7 to 200.5)	-0.55(-0.57 to -0.53)
Spain	Stroke	505949.8(470785.7 to 541405.3)	970.1(908.5 to 1033.8)	694496.2(678678.9 to 710508.7)	776.9(757.4 to 796.5)	-0.69(-0.73 to -0.65)	78035.4(68925.5 to 87464.2)	147.2(131.9 to 163.8)	70979.9(66973.2 to 74932.5)	73.7(69.6 to 78.1)	-2.48(-2.67 to -2.28)	71008.2(51910.1 to 91707.8)	135.2(98.6 to 173.6)	102451(75826.3 to 128391.7)	109.6(80.9 to 138)	-0.66(-0.7 to -0.62)
Sri Lanka	Stroke	147355.4(141596.3 to 153192.5)	1210.9(1163.6 to 1263.8)	270518.1(260307.3 to 280772.5)	1027.1(989.2 to 1066.7)	-0.64(-0.68 to -0.6)	17102.1(15480.3 to 18672.8)	166.9(151 to 182.2)	31012.5(27768.2 to 34183.6)	125.4(113.8 to 137.3)	-1.08(-1.2 to -0.96)	27649.4(19980.7 to 35637.9)	229.7(165.6 to 294.9)	51287.2(37174.1 to 64978.9)	195.8(141.9 to 247.7)	-0.61(-0.65 to -0.58)
Sudan	Stroke	139697.1(133271.5 to 146443.4)	1162.4(1100.7 to 1221.9)	292991.3(281403.1 to 304550.2)	1110(1062.4 to 1162.4)	-0.19(-0.21 to -0.16)	18854.7(17337.1 to 20628.6)	187.7(170.3 to 205.4)	33671.2(30717.6 to 36791.3)	155.8(142 to 171)	-0.73(-0.79 to -0.67)	19057.5(14092.1 to 24126)	159.7(118.5 to 202.5)	39843.8(29217.3 to 50720.6)	151.9(111.6 to 194.3)	-0.2(-0.21 to -0.18)
Suriname	Stroke	3254.3(3136.8 to 3372.3)	1133.4(1090.6 to 1177.9)	6255.3(6048.1 to 6472.9)	988.1(953.4 to 1023.1)	-0.51(-0.54 to -0.48)	413.9(384.3 to 445.3)	156.5(144.4 to 168.5)	835.6(769.1 to 901.2)	136.1(125.4 to 146.4)	-0.66(-0.78 to -0.54)	284.1(213.3 to 356.7)	101.3(76 to 127.4)	550.5(408.6 to 688.3)	88.2(65.6 to 110.5)	-0.51(-0.54 to -0.47)
Sweden	Stroke	160222.4(145719.6 to 177192.2)	1119(1027.1 to 1217.6)	179366(164538.7 to 194052.5)	929.5(860.9 to 995.5)	-0.7(-0.75 to -0.66)	19801(16839.2 to 23120.1)	129.7(111.6 to 149.7)	18135.8(15582.5 to 20930.6)	83.2(72.9 to 94.9)	-1.78(-1.94 to -1.63)	23464.6(16833.5 to 30044.1)	158.8(114.6 to 204.3)	26679.7(19167.5 to 34222.7)	131(93 to 169.1)	-0.74(-0.78 to -0.7)
Switzerland	Stroke	77064.5(73830.2 to 80973.7)	773.9(741 to 811.7)	102748.5(99105.9 to 106414.1)	623.4(601 to 646)	-0.74(-0.75 to -0.72)	10439.3(9409.8 to 11544.5)	99.3(90 to 109.1)	10387.9(9338 to 11547.5)	58(52 to 64.8)	-1.79(-1.9 to -1.67)	10739(7801.7 to 13600.2)	105.7(76.9 to 133.8)	14925.4(10773.4 to 18943.4)	87(62.8 to 110)	-0.66(-0.68 to -0.64)
Syrian Arab Republic	Stroke	104629.8(100404.2 to 108695.8)	1380.3(1321.2 to 1438.5)	148316.7(142675.2 to 153864.2)	1044.3(1002.1 to 1086.5)	-1.02(-1.07 to -0.98)	11737.1(10876.2 to 12635)	181.9(167.7 to 196.7)	16059.5(14652.9 to 17592.1)	135.2(124.7 to 146.2)	-1.21(-1.34 to -1.09)	14251.3(10208.8 to 17950)	189(136.6 to 237.6)	20079.1(14750 to 25594.6)	143.4(105.2 to 181.7)	-1.01(-1.06 to -0.97)
Taiwan (Province of China)	Stroke	256340.4(247744.7 to 265518.9)	1609.4(1556.4 to 1669.7)	460512.1(445078.4 to 476771)	1190.1(1153.8 to 1229.1)	-1.14(-1.21 to -1.08)	33505.3(30645.9 to 36410.9)	228.8(209.4 to 248.9)	50911.8(45892 to 56306.4)	126.7(114.2 to 138.9)	-2.39(-2.56 to -2.22)	50312.3(36005.3 to 63927)	322.4(231.4 to 407)	92439.5(66640.1 to 117085.4)	236.9(169.7 to 299)	-1.17(-1.24 to -1.1)
Tajikistan	Stroke	35371.6(33833.8 to 36945.8)	1141.5(1089.1 to 1197.4)	70832.4(67986.9 to 73691.6)	1046.4(996.3 to 1096.1)	-0.36(-0.41 to -0.31)	5658.1(5201.5 to 6176.9)	200(183.2 to 217.8)	11636.5(10687 to 12667.5)	223.7(205.3 to 244.1)	0.52(0.47 to 0.57)	6541.2(4619.7 to 8333.6)	213(150.9 to 269.8)	12955.7(9270.5 to 16590.3)	195.7(140.6 to 250.6)	-0.33(-0.38 to -0.28)
Thailand	Stroke	517268.8(498528.6 to 536912.9)	1283.2(1236.9 to 1336.3)	1070131.8(1037336.7 to 1109028.5)	1050.2(1018.7 to 1087)	-0.74(-0.78 to -0.69)	63422.4(58393.4 to 68983.6)	177.1(163 to 192.7)	117556.9(107504.4 to 127943.8)	114.9(105.7 to 124.4)	-1.74(-1.87 to -1.6)	98514.8(70022.1 to 124528.2)	248.6(177.7 to 314.7)	208559(150880.2 to 264323.6)	204.3(148.1 to 259.9)	-0.72(-0.77 to -0.67)
Timor-Leste	Stroke	3924.3(3769.4 to 4102)	1044.6(998.4 to 1094.1)	10027.6(9653.9 to 10410.2)	1058.5(1016 to 1100.4)	0.04(0.03 to 0.06)	523.2(478.5 to 571.2)	176.5(161.3 to 193.4)	1530(1394.7 to 1679.8)	181.6(166.1 to 199.3)	0.06(0.02 to 0.11)	722.4(518 to 921.3)	194.6(141.1 to 249.8)	1858.1(1346.3 to 2386.7)	197(143.5 to 251.7)	0.06(0.04 to 0.08)
Togo	Stroke	28734.4(27688 to 29848.7)	1705.5(1635.9 to 1780)	71342.3(68987.9 to 73678.8)	1471.9(1421.8 to 1523.6)	-0.56(-0.6 to -0.53)	3098.5(2851.6 to 3388.8)	209.1(191.2 to 227.7)	7499.2(6926.2 to 8128)	178.9(165.1 to 194.1)	-0.56(-0.62 to -0.5)	3791.2(2805.3 to 4720.2)	236(172.6 to 292.8)	9498.4(6978.7 to 11992.8)	206.6(151.3 to 260.2)	-0.51(-0.54 to -0.48)
Tokelau	Stroke	19.4(18.7 to 20.3)	1456.8(1402.3 to 1519.6)	19.1(18.4 to 19.7)	1311.7(1267.4 to 1352.7)	-0.4(-0.42 to -0.37)	2.7(2.4 to 2.9)	210.1(192.9 to 228.1)	2.2(2 to 2.3)	148.6(136.5 to 159.7)	-1.38(-1.48 to -1.28)	3.7(2.7 to 4.8)	281.4(200.5 to 358.3)	3.6(2.6 to 4.6)	250.2(180.4 to 316.2)	-0.43(-0.46 to -0.4)
Tonga	Stroke	754.4(726.4 to 785.7)	1213.4(1165.2 to 1260.9)	953.5(922.2 to 984.6)	1125.8(1088.3 to 1163.4)	-0.29(-0.31 to -0.28)	82.7(75.4 to 90)	147.2(134.2 to 160.1)	106.3(97.3 to 114.7)	130(118.7 to 140.6)	-0.52(-0.57 to -0.47)	143.5(102.5 to 183)	234(169.1 to 298.3)	181.8(131.1 to 228.9)	215.8(155.3 to 271.9)	-0.31(-0.33 to -0.29)
Trinidad and Tobago	Stroke	11962.8(11489.3 to 12452.7)	1288(1236.7 to 1340.8)	17392.8(16761.2 to 18077.6)	957.2(923.4 to 993.3)	-1.12(-1.18 to -1.05)	1461.2(1335.8 to 1593.4)	172.3(157.4 to 187.8)	1938.6(1746.8 to 2130)	107(97.6 to 116.8)	-1.81(-1.91 to -1.7)	1041.5(769.3 to 1323.6)	113.9(84.6 to 144.8)	1540(1148.6 to 1957.8)	85.2(63.6 to 108.2)	-1.09(-1.15 to -1.03)
Tunisia	Stroke	54502.7(52185.9 to 56956.2)	919(878 to 960.6)	123062.8(118417 to 128231.5)	923.1(887.3 to 963.3)	0(-0.04 to 0.04)	6602(5953.2 to 7291.3)	134.4(121 to 147.8)	14944.4(13311.9 to 16757.7)	119.1(107.2 to 133.2)	-0.5(-0.58 to -0.41)	7556.7(5510.4 to 9588)	128.9(94.7 to 163.7)	16908.8(12373 to 21502.2)	127.9(93.5 to 163.3)	-0.02(-0.06 to 0.02)
Turkmenistan	Stroke	583399.7(559163.4 to 606003.4)	1430.3(1366.8 to 1491.7)	926990(894758.1 to 963704.9)	999.1(963.3 to 1039.7)	-1.35(-1.42 to -1.28)	64020.2(58412.2 to 70600.4)	177.8(161.3 to 196)	97866.2(87326.1 to 108254.7)	110.4(99.8 to 121.8)	-1.8(-1.9 to -1.7)	81887.2(59648.1 to 104593.4)	203.7(147.7 to 261.1)	131055.9(95497 to 167134)	142.6(104 to 181.6)	-1.34(-1.41 to -1.27)
Tuvalu	Stroke	29390.1(28075.4 to 30770)	1325.6(1261.6 to 1392.7)	70244.1(67647 to 73213.4)	1571.1(1502.1 to 1638.5)	0.66(0.62 to 0.71)	4190.4(3840.6 to 4530.3)	212.8(194.7 to 232.1)	9318.2(8535.7 to 10158.6)	225.3(206.4 to 245.1)	0.41(0.32 to 0.5)	5340.5(3796.8 to 6809)	243.3(172.9 to 311.6)	12703.4(9119.1 to 16275.7)	288.2(207.5 to 368.3)	0.68(0.63 to 0.73)
T 肩 rkiye	Stroke	115.5(111.2 to 119.6)	1605.8(1544.1 to 1670.1)	158.9(154.1 to 164.1)	1469.1(1422 to 1520.6)	-0.35(-0.38 to -0.32)	16.2(14.9 to 17.6)	253.7(234.8 to 272.9)	20.5(19 to 22.2)	203.6(189.7 to 219.7)	-0.89(-0.96 to -0.83)	21.9(15.8 to 28)	309.4(224.8 to 393)	29.9(21.7 to 38)	279.3(203.4 to 352.9)	-0.37(-0.39 to -0.35)
Uganda	Stroke	115990.2(110851 to 121611.8)	1553(1480.4 to 1630.8)	241988.9(233294.5 to 251171.1)	1364.2(1314 to 1421.6)	-0.51(-0.57 to -0.45)	14279.1(12877.3 to 15859.4)	208.4(187.3 to 231.9)	27992.1(25376.4 to 30716)	176.2(157.6 to 193.4)	-0.77(-0.9 to -0.63)	15381.5(11233.6 to 19327.5)	214.7(158.8 to 267.7)	32443.7(24043.3 to 40865.1)	193(143.3 to 241.2)	-0.41(-0.47 to -0.36)
Ukraine	Stroke	968161.7(867954.1 to 1061315.2)	1396.8(1262.1 to 1527.4)	843892.3(763449 to 924951.3)	1207.9(1108.4 to 1314.5)	-0.44(-0.49 to -0.4)	175681.6(149790.3 to 207698.9)	261.3(225.6 to 301.4)	134283.5(116189.3 to 153495.3)	183.7(161.3 to 207.8)	-1.42(-1.54 to -1.3)	175181.1(127066.2 to 224059)	252.8(183.1 to 322.3)	152587.3(110791.6 to 198677.1)	217.1(156.9 to 280.9)	-0.44(-0.48 to -0.39)
United Arab Emirates	Stroke	15731.3(15036.3 to 16453.1)	1697(1617.5 to 1787)	113378.9(108776.8 to 117974.6)	1429.4(1361.6 to 1493)	-0.64(-0.7 to -0.59)	1495.6(1350.7 to 1653.8)	241.1(218.5 to 267.2)	10210.1(8910.5 to 11682.8)	173.5(157.5 to 191.8)	-1.29(-1.44 to -1.13)	2085(1531.7 to 2641.4)	229.4(168.1 to 291.7)	14591.9(10628.5 to 18568.6)	190.2(139.8 to 240.6)	-0.69(-0.74 to -0.64)
United Kingdom	Stroke	867791.7(810174.2 to 928594.5)	1027.2(963.7 to 1091.7)	894946.8(842584.1 to 952592.7)	759.8(718.5 to 804.2)	-1.1(-1.15 to -1.06)	117553.3(101194.4 to 135248.4)	132.4(115.2 to 151.1)	95969.7(86613.8 to 106312.8)	75.1(67.9 to 82.9)	-2.14(-2.25 to -2.03)	123631(88365.5 to 158863.5)	143.4(102.8 to 183.8)	130441.6(93929.1 to 166056.8)	106.7(76.9 to 136)	-1.09(-1.14 to -1.04)
United Republic of Tanzania	Stroke	171504.2(164841.3 to 178621.4)	1359.2(1300.9 to 1424.3)	432497.9(416756.2 to 449021.2)	1447.5(1391.3 to 1506)	0.2(0.16 to 0.23)	21816.8(19974.6 to 23849.3)	189.4(172.3 to 208)	49446.8(45131.4 to 54011.6)	182.3(164.7 to 200.3)	0(-0.09 to 0.08)	22684.1(16737 to 28356.9)	186.3(137.5 to 232.1)	57940.8(42614.7 to 71999.1)	201.8(149.6 to 250.3)	0.27(0.24 to 0.31)
United States of America	Stroke	3767725(3478373.6 to 4094434.4)	1227.3(1135.6 to 1325.2)	6299339.3(5888661.2 to 6760944.6)	1207(1130.8 to 1293.3)	-0.24(-0.43 to -0.06)	357880.8(303318.8 to 420067.6)	113.4(97.2 to 132.5)	412025.1(358281.3 to 469611.7)	75.6(66.3 to 85.8)	-1.42(-1.58 to -1.27)	536878(381323.2 to 687481.7)	173.3(123.2 to 222.4)	882003.9(641272.8 to 1124842.1)	166.9(121 to 211.9)	-0.32(-0.51 to -0.13)
United States Virgin Islands	Stroke	795.9(762.3 to 827)	855.7(818.9 to 890.6)	1227.7(1178.9 to 1285.7)	795.9(766.6 to 827.6)	-0.25(-0.31 to -0.18)	86(78.3 to 94.2)	102.8(93.9 to 112.2)	152.8(138.7 to 168.6)	93.2(85.4 to 101.8)	-0.32(-0.38 to -0.27)	69.8(52 to 88.1)	77.7(57.8 to 98.1)	112.4(83.9 to 142.9)	71.3(53.6 to 89.8)	-0.29(-0.36 to -0.22)
Uruguay	Stroke	59897.3(57474.3 to 62420.4)	1599.3(1537.2 to 1662.8)	50735.3(48963.8 to 52692.2)	1012.6(980.7 to 1047.3)	-1.7(-1.79 to -1.61)	6978(6323.2 to 7657.2)	188.3(171.8 to 205.3)	5697.7(5198.1 to 6223.4)	105.4(96.3 to 115)	-2.18(-2.31 to -2.04)	8718.7(6327.6 to 10923.9)	231.6(168.2 to 290.4)	7523.3(5539.1 to 9603.1)	146.2(107.6 to 186.4)	-1.7(-1.78 to -1.61)
Uzbekistan	Stroke	176977.4(168447.4 to 185407.3)	1394.5(1321 to 1467.9)	391746.5(376751.6 to 407241.5)	1349.9(1294.6 to 1409.4)	-0.15(-0.2 to -0.1)	26982(25000.8 to 29300.7)	226.5(209.4 to 246)	59953.5(55241.7 to 65435.2)	249(229.6 to 270.3)	0.23(-0.07 to 0.52)	32411(23410.1 to 41411.9)	257(185.5 to 329.1)	70820.5(51008.9 to 90104.7)	248.1(179.5 to 314.8)	-0.14(-0.19 to -0.1)
Vanuatu	Stroke	1465.4(1412.7 to 1523.2)	1850.7(1776.4 to 1928.6)	3812(3703.6 to 3921.1)	1832.8(1774.2 to 1893)	-0.03(-0.06 to -0.01)	174.1(161.2 to 189.2)	258.1(237.6 to 282.9)	450.2(418.7 to 486.4)	244.5(227.8 to 264.1)	-0.23(-0.27 to -0.2)	272.5(193.5 to 346.4)	350.4(249.4 to 445.6)	710.7(508.9 to 903.2)	347.4(250.8 to 441.9)	-0.03(-0.05 to 0)
Venezuela (Bolivarian Republic of)	Stroke	122563.2(118079.6 to 127293.6)	1068.1(1030.1 to 1107.8)	256537.1(247744.3 to 265584.7)	870.5(841.4 to 902.7)	-0.73(-0.76 to -0.7)	13876(12741.5 to 15100.7)	132.6(121.3 to 145.2)	28544.5(26017.2 to 31062.6)	99.1(90.6 to 107.6)	-1.02(-1.11 to -0.93)	10791.6(7976.9 to 13649.7)	96.9(72.2 to 121.9)	23087.6(17108.2 to 29078.7)	79.4(59.1 to 99.7)	-0.7(-0.73 to -0.67)
Viet Nam	Stroke	537884.3(515484.7 to 561768.9)	1213.1(1160.9 to 1268.8)	1275344.9(1237550.8 to 1317875)	1233.3(1193.6 to 1277.2)	0.04(0.02 to 0.05)	89380.5(82606.2 to 96496.5)	224.1(206.6 to 242)	195806.1(183222.4 to 209488.6)	203.4(190.5 to 217.8)	-0.42(-0.46 to -0.39)	102875.9(74288.3 to 132077.5)	233.3(169.1 to 300)	241891.8(174668.4 to 309600.4)	236.3(170.9 to 302.2)	0.03(0.01 to 0.04)
Yemen	Stroke	72428.5(69421.2 to 75684.1)	1055.1(1003.4 to 1105.9)	187658.2(179957.9 to 195656.9)	963.6(916.7 to 1009.1)	-0.3(-0.33 to -0.27)	10108.2(9251.8 to 11020.7)	187(171.9 to 203.6)	24090.8(22029.7 to 26109.5)	157.2(143.8 to 171.5)	-0.63(-0.67 to -0.6)	9838.2(7211 to 12500.6)	144.8(107 to 185)	25539.5(18820.3 to 32168.1)	132.4(98 to 168.9)	-0.29(-0.32 to -0.26)
Zambia	Stroke	51097.8(49158.8 to 53377)	1511.9(1447 to 1584.7)	122088.3(117793.4 to 126344.6)	1475.4(1420.3 to 1539)	-0.09(-0.1 to -0.08)	6741.3(6195.4 to 7367.7)	223.2(204.2 to 246.8)	15863.5(14477 to 17260.3)	220.6(199.2 to 242.1)	-0.07(-0.11 to -0.04)	6796.5(4994.5 to 8482.5)	209.6(152.8 to 259.6)	16258(11874 to 20322)	207.4(154 to 259)	-0.02(-0.02 to -0.01)
Zimbabwe	Stroke	54662.8(52316.6 to 57197.8)	1167.4(1112 to 1223.7)	101593.1(97498.3 to 105549.2)	1287.6(1234.8 to 1341.8)	0.33(0.29 to 0.36)	5991.7(5409.6 to 6642.3)	142.8(128.6 to 157.7)	11264.9(10125.3 to 12487.8)	163.5(147.6 to 181.9)	0.61(0.48 to 0.75)	7486.8(5434.7 to 9322.5)	167.2(121.6 to 208.9)	13757.1(10066 to 17451.7)	183.1(134.8 to 231.8)	0.32(0.29 to 0.34)
Afghanistan	ICH	26168.1(24420.9 to 27986.3)	339.3(317.4 to 362.2)	38568.5(36434.7 to 40948.1)	198.3(185.2 to 212.8)	-2.07(-2.23 to -1.91)	6090.9(5468.6 to 6711.5)	91(81.7 to 99.3)	6824.2(6176.8 to 7493.6)	51.8(47.3 to 56.2)	-2.3(-2.51 to -2.1)	3587.1(2567.6 to 4588)	47(33.5 to 60)	5197.9(3684.2 to 6737.9)	27.1(19.1 to 34.7)	-2.11(-2.26 to -1.95)
Albania	ICH	6235.4(5825.9 to 6669)	234.1(218.3 to 250.8)	5626.1(5248.6 to 6058.1)	163.3(153.6 to 174.2)	-1.38(-1.55 to -1.21)	1786.5(1659.5 to 1904)	98.2(90.9 to 104.2)	3294.1(3034.2 to 3554.1)	81.8(75.7 to 87.8)	-0.79(-0.87 to -0.7)	1142.8(821.4 to 1476.1)	43.9(31.4 to 56.8)	1104.8(776.6 to 1421.1)	31.4(22.2 to 40.3)	-1.29(-1.45 to -1.12)
Algeria	ICH	39381.9(37048.1 to 42074.4)	231.8(216.7 to 248)	57577.9(53885.9 to 61638.5)	136.5(127.4 to 146.3)	-2.07(-2.18 to -1.96)	6494.7(5795.9 to 7198.8)	50.1(44.8 to 54.9)	8459.4(7545.2 to 9361.5)	23.9(21.3 to 26.2)	-2.88(-3.06 to -2.7)	5443.6(3850.7 to 7049.1)	32.4(22.9 to 41.8)	8025.4(5857 to 10435.9)	19.3(14.1 to 24.9)	-2.04(-2.15 to -1.93)
American Samoa	ICH	119.5(112.2 to 127.2)	380.4(356.4 to 407.4)	150.1(141 to 159.2)	287.4(270.2 to 304.7)	-1.02(-1.06 to -0.99)	21.1(19.1 to 23.2)	90.7(81.7 to 98.9)	29(26.2 to 31.8)	65.4(59.6 to 71.6)	-1.2(-1.27 to -1.14)	21.8(15.7 to 27.8)	70.3(50.7 to 90.1)	27.3(19.5 to 35)	52.9(38 to 67.8)	-1.01(-1.05 to -0.98)

Andorra	ICH	63.2(57.8 to 68.8)	107.7(98.8 to 116.9)	123.4(114.5 to 132.8)	93.2(85.6 to 100.8)	-0.64(-0.69 to -0.6)	7.8(6.6 to 8.9)	14.1(12.1 to 16.1)	16.3(14.1 to 18.3)	11(9.5 to 12.4)	-0.94(-1.03 to -0.85)	8.6(6.1 to 11.1)	14.8(10.5 to 19)	17.5(12.6 to 22.7)	13(9.3 to 16.9)	-0.59(-0.63 to -0.55)
Angola	ICH	19030.9(17784.2 to 20360.1)	320.6(297.7 to 348.2)	39372.7(36688.6 to 42098.9)	212.2(195.5 to 228.8)	-1.53(-1.61 to -1.45)	4032.9(3586.5 to 4496.3)	95.9(85.6 to 105.5)	7306.8(6566.7 to 8140.1)	56.3(50.8 to 62.3)	-2.03(-2.16 to -1.9)	2508.4(1815.3 to 3228.4)	44.1(31.9 to 56.6)	5259.7(3738.9 to 6738)	30.2(21.2 to 38.9)	-1.39(-1.46 to -1.32)
Antigua and Barbuda	ICH	115.3(106.7 to 126)	219.8(203.9 to 239.8)	161.1(150.7 to 172.5)	152.4(142.8 to 163.3)	-1.45(-1.52 to -1.38)	28.4(26.1 to 30.7)	52(47.8 to 56.4)	31.3(28.5 to 34)	31.3(28.5 to 33.8)	-1.89(-2.05 to -1.73)	10.7(7.8 to 14)	20(14.6 to 26.1)	14.5(10.5 to 18.6)	13.9(10.1 to 17.9)	-1.45(-1.52 to -1.38)
Argentina	ICH	115912.3(109044 to 123927.4)	358.8(337.5 to 383.1)	101762.7(95927.3 to 108048.6)	196(184.7 to 207.7)	-2.31(-2.44 to -2.18)	18262(16411.8 to 20056.5)	57.6(51.9 to 63.1)	14710.2(13241.4 to 16125.3)	27.2(24.5 to 29.8)	-2.88(-3.08 to -2.68)	16104.2(11570.2 to 20537.5)	50(36 to 63.7)	14462.3(10466.2 to 18505.5)	27.7(20 to 35.3)	-2.26(-2.38 to -2.13)
Armenia	ICH	4433.8(4085.1 to 4787.9)	142.3(131.3 to 153.5)	3593.4(3303.4 to 3888.4)	95.1(87.9 to 102.9)	-1.53(-1.62 to -1.43)	1136.5(1023.6 to 1254.7)	44.6(40.3 to 49.2)	782.2(701.7 to 863.9)	19.1(17.2 to 21.1)	-3.23(-3.49 to -2.96)	807.7(563.4 to 1038.6)	26.2(18.3 to 33.8)	660(449.7 to 863.2)	17.4(11.8 to 22.7)	-1.54(-1.64 to -1.43)
Australia	ICH	21720.7(20170.1 to 23420.4)	115(106.9 to 124.1)	32459.7(29831.2 to 35028.8)	86.3(78.9 to 93.5)	-1.04(-1.09 to -0.98)	3562.2(3287.5 to 3820.8)	18.7(17.3 to 20.1)	4867.3(4374.7 to 5355.4)	10.9(9.7 to 12.1)	-1.76(-1.89 to -1.63)	3170.3(2270.3 to 4111.5)	16.8(12 to 21.7)	5053.4(3580.9 to 6538.3)	13(9.2 to 17)	-0.92(-0.96 to -0.87)
Austria	ICH	27736.6(25609 to 29424.8)	249.8(233.1 to 264.2)	21429.8(20167.8 to 22644.4)	139.2(130.8 to 147.8)	-2.17(-2.26 to -2.08)	4362.8(3820.9 to 4837.8)	37.3(32.8 to 41)	2497.5(2200 to 2803.6)	14.3(12.6 to 16.1)	-3.51(-3.7 to -3.32)	3940(2914.5 to 5008.3)	34.9(25.7 to 44.2)	3144.8(2330.6 to 3965.9)	19.6(14.4 to 24.9)	-2.15(-2.24 to -2.06)
Azerbaijan	ICH	14564.2(13448.4 to 15548)	245.5(226.1 to 263.1)	21124.2(19575.4 to 22739.4)	181.9(168.3 to 196)	-1.09(-1.21 to -0.96)	4159.9(3830.6 to 4521.3)	87.3(80.1 to 94.7)	7622.9(6947.6 to 8238.9)	92.6(84.9 to 100.1)	0.4(0.27 to 0.53)	2627.5(1877.2 to 3377)	44.7(31.9 to 57.2)	3811.7(2656.5 to 4908.3)	33.6(23.7 to 43)	-1.02(-1.15 to -0.9)
Bahamas	ICH	435.1(407.6 to 467.3)	220.2(206.4 to 236.6)	727.8(683.9 to 779.3)	167.6(157.5 to 179.5)	-1.02(-1.06 to -0.98)	75.2(69.3 to 81.3)	44.6(41 to 48.3)	123.8(113.2 to 134.9)	30.9(28.4 to 33.6)	-1.33(-1.44 to -1.23)	37.5(27 to 48.4)	19.4(14 to 25)	62.5(45.8 to 79.8)	14.7(10.7 to 18.8)	-1.05(-1.1 to -1.01)
Bahrain	ICH	832.1(771.6 to 898.9)	207.7(194 to 225)	2197.1(2048.2 to 2354.4)	138.1(129 to 148.2)	-1.6(-1.69 to -1.5)	86.1(76.3 to 96.4)	36.7(32.9 to 40.3)	171.9(151.2 to 195.7)	18(16.2 to 20)	-2.64(-2.79 to -2.49)	112.8(78.7 to 146.3)	28.8(20.3 to 37)	295.4(212.6 to 379.3)	19.2(13.6 to 24.4)	-1.59(-1.69 to -1.49)
Bangladesh	ICH	208497.4(194067.3 to 224592.2)	355(326.9 to 387)	403370.9(375283 to 433403.3)	269(249.7 to 289.1)	-1.01(-1.05 to -0.97)	48511.3(43816.9 to 53207.6)	99.3(88.9 to 109.5)	94995.5(85170.5 to 103928.9)	71(63.9 to 77.6)	-1.15(-1.32 to -0.98)	29067.3(21248.7 to 37452.3)	50.3(36.5 to 64.8)	57892.8(41433.9 to 74067.6)	39.2(28 to 50.1)	-0.92(-0.97 to -0.88)
Barbados	ICH	480.3(445.8 to 518.6)	188.1(175 to 202)	597.2(555.2 to 637.7)	144.9(135.3 to 154.8)	-1.02(-1.07 to -0.96)	106.4(97.2 to 115.7)	37.6(34.3 to 40.7)	116.2(105.1 to 128.9)	24.6(22.4 to 27.2)	-1.5(-1.66 to -1.34)	45.4(32.9 to 58.6)	17.2(12.4 to 22.2)	57.1(41.7 to 73.2)	13.5(9.9 to 17.2)	-0.96(-1.02 to -0.9)
Belarus	ICH	19809.1(18388.8 to 21404.7)	161.1(149.9 to 173.7)	16921.7(15539.8 to 18370.4)	127.7(117.6 to 137.7)	-0.78(-0.93 to -0.64)	4733(4269.2 to 5218.6)	38.7(35 to 42.6)	3899.2(3525 to 4278.1)	26.8(24.3 to 29.3)	-1.27(-1.51 to -1.03)	3521.8(2513.4 to 4565.3)	28.6(20.4 to 37)	3036.5(2138.2 to 3928.5)	22.9(16.1 to 29.6)	-0.74(-0.87 to -0.6)
Belgium	ICH	18491.8(17096.6 to 19896.7)	139.3(129.2 to 149.7)	16494.1(15314.4 to 17806.7)	92.2(85 to 99.9)	-1.61(-1.72 to -1.51)	3559.1(3164.6 to 3911.7)	24.3(21.7 to 26.5)	3134.6(2829.3 to 3427.6)	12.9(11.8 to 14.2)	-2.14(-2.29 to -1.99)	2549(1796.1 to 3316.8)	18.9(13.4 to 24.6)	2409.5(1719.8 to 3152.6)	12.9(9.2 to 16.9)	-1.46(-1.56 to -1.36)
Belize	ICH	210.9(195.9 to 226.9)	175.7(164.3 to 188.3)	518.9(484 to 557.6)	138.6(129.4 to 148.8)	-1(-1.1 to -0.89)	39.3(35.9 to 42.9)	37.6(34.4 to 40.8)	88(80.4 to 96.8)	28.1(25.6 to 30.6)	-1.42(-1.59 to -1.24)	19(13.6 to 24.7)	16.1(11.5 to 20.9)	45.8(33.2 to 59.1)	12.6(9.2 to 16.3)	-1.04(-1.15 to -0.93)
Benin	ICH	10410.9(9798 to 11097.6)	377(352.6 to 405.2)	23763.8(22524.7 to 25213.1)	299.6(281.3 to 319.9)	-0.91(-0.99 to -0.83)	2105.3(1900.1 to 2312.8)	96.3(85.7 to 105.9)	4110.4(3749.8 to 4529.3)	72.6(65.3 to 79.8)	-1.09(-1.18 to -1)	1359.6(990.1 to 1731.6)	50.8(36.7 to 64.6)	3111.8(2216.8 to 3987.4)	41(29.3 to 52.8)	-0.85(-0.93 to -0.78)
Bermuda	ICH	112.3(104.6 to 120.9)	176.2(164.5 to 189.8)	122.3(114.3 to 130.7)	126.4(118.5 to 135.3)	-1.27(-1.34 to -1.21)	17.1(15.2 to 18.9)	28.2(25.2 to 31.2)	17(15.1 to 18.8)	14.3(12.7 to 16.1)	-2.58(-2.75 to -2.42)	10(7.1 to 12.9)	15.9(11.3 to 20.5)	11.9(8.7 to 15.6)	11.6(8.6 to 15.2)	-1.21(-1.27 to -1.15)
Bhutan	ICH	772.7(710.7 to 837.2)	233.9(213.3 to 255.6)	1138.9(1046.8 to 1226.3)	171.3(157.3 to 184.9)	-1.24(-1.32 to -1.17)	150.4(131.8 to 172)	58.8(51.8 to 66.3)	223.5(196.9 to 249.8)	36.9(32.4 to 41.3)	-1.79(-1.91 to -1.68)	108.6(76.2 to 141.5)	33.8(23.5 to 44)	165.7(118.3 to 212.7)	25.2(18 to 32.4)	-1.18(-1.25 to -1.11)
Bolivia (Plurinational State of)	ICH	9053.6(8414.6 to 9665.6)	206.5(191.8 to 221.4)	12495.7(11476.5 to 13468.7)	117.4(108.1 to 126.9)	-2.2(-2.3 to -2.09)	1662(1482.1 to 1846.1)	47(42.1 to 51.7)	2233.5(2000.9 to 2464.8)	25.3(22.6 to 27.7)	-2.37(-2.51 to -2.23)	792.3(577.9 to 1022.7)	18.7(13.7 to 24.1)	1143.8(817.6 to 1493.5)	11.1(7.9 to 14.5)	-2.01(-2.11 to -1.92)
Bosnia and Herzegovina	ICH	5217.4(4787.9 to 5644)	111.1(102.5 to 120.1)	3770.1(3467.2 to 4084.2)	85.5(78.9 to 92.6)	-1.06(-1.13 to -0.99)	1019(906.6 to 1113)	27.1(24.3 to 29.9)	1055.3(950.1 to 1163.3)	18.3(16.5 to 20.2)	-1.33(-1.44 to -1.22)	939.2(648.4 to 1224.7)	20.3(14 to 26.5)	700.3(485.2 to 928)	15.7(10.9 to 20.9)	-1.03(-1.09 to -0.96)
Botswana	ICH	2020.7(1872.2 to 2172.4)	279.7(258.5 to 302.9)	3990.8(3711.1 to 4262.7)	208.7(194.6 to 224.7)	-1.25(-1.44 to -1.06)	454.2(402.1 to 504.7)	79.8(71.1 to 87.9)	880.1(786.5 to 968.9)	59.6(53.4 to 65)	-1.33(-1.56 to -1.1)	273.5(193.6 to 352.7)	39.5(28.1 to 51.6)	535.1(386.9 to 683.7)	29.5(21.1 to 37.7)	-1.2(-1.36 to -1.04)
Brazil	ICH	268261.3(238551.2 to 301531.5)	240.3(213.1 to 269.8)	283407.3(253058.9 to 314723.8)	113.1(101.4 to 125.3)	-2.97(-3.14 to -2.81)	51322.4(43297.6 to 59533.8)	52(43.3 to 60)	51212.2(43933.6 to 58713.5)	20.7(17.8 to 23.7)	-3.45(-3.66 to -3.25)	22474.1(16525.1 to 29507.4)	20.6(15.2 to 26.7)	25488.3(18628.9 to 33495.5)	10.2(7.5 to 13.5)	-2.76(-2.91 to -2.6)
Brunei Darussalam	ICH	703.3(661 to 744.7)	504.5(474.2 to 539.9)	1122.8(1064.5 to 1190.5)	274.5(257.7 to 291.1)	-2.28(-2.4 to -2.15)	90(79.6 to 101.7)	71.3(63 to 79.9)	124.2(109.7 to 139)	34.5(30.7 to 38.2)	-2.74(-2.96 to -2.52)	109.8(79.6 to 139.5)	79.8(57.4 to 101.7)	174.7(123.4 to 222.3)	43.7(31 to 55)	-2.25(-2.37 to -2.13)
Bulgaria	ICH	31524.5(29150.8 to 33947.2)	288.6(269.3 to 307.9)	15142(13944.8 to 16555.2)	154(143.6 to 167.6)	-2.39(-2.52 to -2.26)	11098.1(10306.7 to 11979.3)	103.1(96.4 to 109.6)	6520.4(6089.7 to 6940.4)	48.7(45.6 to 51.6)	-2.99(-3.24 to -2.74)	5608(3932.8 to 7178.1)	51.4(36.4 to 65.7)	2718.1(1843 to 3512.8)	27.4(18.7 to 35.6)	-2.4(-2.53 to -2.27)
Burkina Faso	ICH	17025.3(15994.4 to 18317.5)	288.4(269.8 to 311)	34467.1(32333.9 to 36737.2)	249.3(233.5 to 266.2)	-0.57(-0.64 to -0.51)	3126.2(2788.7 to 3429.8)	69.8(62.3 to 76.2)	6357.2(5774.4 to 6968.2)	63.4(57 to 68.4)	-0.33(-0.38 to -0.27)	2240.9(1597.3 to 2848.8)	39.3(27.9 to 49.9)	4555.3(3264.2 to 5825.4)	34.4(24.8 to 43.5)	-0.51(-0.57 to -0.45)
Burundi	ICH	12607.9(11818.5 to 13462.2)	403.9(376.1 to 433.8)	16505.5(15390 to 17613)	213.4(198.3 to 231.2)	-2.41(-2.52 to -2.3)	3281.6(2972.4 to 3596.8)	128.3(116.1 to 140.4)	3609.6(3277.6 to 3946.7)	66.6(60.3 to 73)	-2.57(-2.74 to -2.39)	1665.6(1208.6 to 2147.2)	54.9(40 to 70.9)	2203.2(1599.5 to 2846.5)	30.5(21.8 to 40.5)	-2.21(-2.31 to -2.11)
Cabo Verde	ICH	968.8(911.8 to 1033.4)	388.6(365.5 to 413.9)	1563.7(1478.3 to 1656.9)	300.3(282.5 to 319.1)	-1(-1.09 to -0.9)	162.8(144.3 to 180.6)	65.7(58.3 to 72.8)	247.4(220.3 to 267.9)	54.5(48.1 to 59.8)	-0.78(-0.86 to -0.7)	132.1(95.6 to 168.9)	53.3(38.4 to 67.2)	209.8(151.2 to 269)	41.2(29.6 to 52.7)	-0.98(-1.06 to -0.89)
Cambodia	ICH	19111.4(17769.2 to 20613.2)	314.2(290.4 to 340.7)	38226.9(35640.8 to 40762.6)	253.9(237.1 to 272)	-0.8(-0.88 to -0.73)	5209.3(4730.6 to 5654.5)	117.6(107.1 to 127.3)	10875(10011.1 to 11788.9)	92.4(85.6 to 99.7)	-0.93(-0.99 to -0.87)	3567.2(2555.4 to 4651.6)	59.4(42.5 to 76.8)	7158.8(5001.9 to 9399.5)	48.3(33.6 to 62.7)	-0.77(-0.85 to -0.7)
Cameroon	ICH	23282.2(21860.4 to 24865.7)	369.7(344.5 to 397.8)	63589.2(60032.2 to 67194.5)	321.6(301.1 to 344.6)	-0.58(-0.74 to -0.41)	4403.3(3970.4 to 4828.5)	95.6(85.9 to 104.8)	11009.7(10105.4 to 12071.6)	80(73.1 to 87.2)	-0.71(-0.87 to -0.54)	3024.5(2190.1 to 3880)	49.9(36.1 to 64)	8292.5(6051.4 to 10576.3)	44(32 to 55.8)	-0.54(-0.69 to -0.39)
Canada	ICH	49798.4(46442.2 to 53324.3)	160(149.2 to 171.6)	89586.4(84486.9 to 94780.3)	147.7(138.8 to 156.9)	0.09(-0.06 to 0.23)	5727.9(5043.5 to 6392.9)	18.2(16.1 to 20.2)	8893.7(7653.7 to 9868)	13.3(11.4 to 15)	-0.62(-0.78 to -0.46)	7155.5(5082.9 to 9220.2)	23(16.3 to 29.6)	13317.7(9556 to 17018.4)	21.5(15.4 to 27.7)	0.14(-0.01 to 0.29)
Central African Republic	ICH	5327.2(4953.3 to 5722.2)	321.5(297 to 350)	8648.6(8121.9 to 9262.8)	243.5(225.2 to 264.2)	-1.02(-1.11 to -0.92)	1326.5(1179.2 to 1479.9)	109.9(98.2 to 120.2)	2057.2(1853.4 to 2260.6)	84.7(77.3 to 92)	-1.03(-1.11 to -0.95)	689.2(502.8 to 866.5)	43.4(31.7 to 54.9)	1128.7(814.8 to 1495.4)	34(24.1 to 45.9)	-0.89(-0.97 to -0.82)
Chad	ICH	13405.9(12574.5 to 14402.7)	362.1(338.2 to 392)	31853.6(30225.4 to 33694.6)	331.3(310.7 to 354.5)	-0.33(-0.43 to -0.22)	2764(2508.1 to 3037.9)	91.8(82.9 to 101)	5783.9(5295.3 to 6293.1)	86.2(78.5 to 93.9)	-0.27(-0.41 to -0.14)	1760.8(1278.8 to 2241.6)	48.9(35.3 to 62.7)	4118(3031.5 to 5314.2)	44.7(32.6 to 57.5)	-0.32(-0.42 to -0.23)
Chile	ICH	32712.8(30781.9 to 34856.5)	291.1(273.8 to 310.5)	49554.9(46531.4 to 52498.8)	211.6(199 to 224.1)	-1.22(-1.31 to -1.14)	4806.5(4272.9 to 5302.4)	46.1(40.9 to 50.7)	5736.7(5110.9 to 6328.1)	23.6(21.1 to 26.1)	-2.29(-2.46 to -2.13)	4594.7(3317.5 to 5874.6)	41.3(29.8 to 52.6)	7135.2(5250.3 to 9091.9)	30.3(22.3 to 38.8)	-1.17(-1.26 to -1.09)
China	ICH	3115040(2764294.2 to 3518251.9)	308.4(274.5 to 348.3)	4385239.7(3892100.8 to 4906564.9)	222.1(200.1 to 246.5)	-1.26(-1.37 to -1.15)	774011.8(644709.2 to 896196.9)	108.9(91.7 to 124.9)	1173288.3(1003993 to 1330455.4)	61.2(53 to 69.1)	-2.24(-2.5 to -1.98)	597049.1(409771.6 to 784636.7)	60.1(41.4 to 78.5)	861429.3(597331.6 to 1116860.1)	43.7(30.3 to 57)	-1.23(-1.34 to -1.11)
Colombia	ICH	47155.3(44125.1 to 50433.4)	198.6(185.7 to 212.9)	60284.4(56115.1 to 64451.6)	111.1(103.3 to 119)	-2.21(-2.32 to -2.1)	7078.9(6458.6 to 7828.1)	36.8(33.4 to 40.4)	8568.2(7662.1 to 9538.3)	15.6(13.9 to 17.4)	-3.21(-3.39 to -3.02)	4112.2(2997.7 to 5287.9)	17.8(13.1 to 22.9)	5680.2(4090.1 to 7330.5)	10.4(7.5 to 13.4)	-2.06(-2.17 to -1.95)
Comoros	ICH	902.1(840.1 to 969)	331.7(305.4 to 358.9)	1246.7(1161.9 to 1341.5)	203.4(188.7 to 219.2)	-1.92(-2.02 to -1.81)	190.7(169.8 to 213)	90.1(81 to 99.9)	257.3(232.4 to 284.6)	52.1(47.3 to 57.1)	-2.14(-2.28 to -1.99)	119.1(84.1 to 152.6)	45.4(32.4 to 58.4)	170.4(122 to 220.5)	28.9(20.6 to 37.6)	-1.76(-1.85 to -1.67)
Congo	ICH	5025.6(4707.2 to 5388)	348.2(324.4 to 375.5)	8343.9(7809.9 to 8941)	210.2(195.3 to 226.9)	-1.93(-2.03 to -1.83)	1165.8(1040.4 to 1302.5)	104.1(93.4 to 114.6)	1710.3(1533.6 to 1895.2)	60.3(54.3 to 66.1)	-2.13(-2.27 to -1.99)	661.5(471.4 to 842)	47.3(33.7 to 60.7)	1108.6(784.8 to 1427)	29.6(20.9 to 38.2)	-1.78(-1.88 to -1.69)
Cook Islands	ICH	49.4(46.2 to 53)	329.7(305.7 to 354.9)	61.1(57.4 to 65.2)	267.9(253.2 to 285.2)	-0.77(-0.84 to -0.71)	9.2(8.2 to 10.2)	73.5(65.7 to 82)	10.9(9.7 to 12.2)	45.9(40.9 to 50.9)	-1.76(-1.9 to -1.62)	9.1(6.4 to 11.8)	60.9(43.5 to 78.7)	11.3(8 to 14.5)	49.3(35.3 to 63.4)	-0.77(-0.84 to -0.71)
Costa Rica	ICH	4063.5(3765.4 to 4395.2)	177.8(166.5 to 191.1)	7084.5(6657.6 to 7585.8)	133.4(125.5 to 142.9)	-1.1(-1.16 to -1.04)	525.8(473.2 to 595.6)	26.8(24.1 to 29.7)	855.2(748.7 to 960.4)	16(14.1 to 18.1)	-1.94(-2.09 to -1.78)	359.6(257.8 to 468)	16.1(11.6 to 20.7)	651.6(469.4 to 838.5)	12.2(8.8 to 15.8)	-1.06(-1.12 to -0.99)
Croatia	ICH	28460.4(26852.8 to 30406.9)	405.4(377.5 to 436.2)	57876.3(54848.5 to 61024.4)	315.3(294.8 to 335.4)	-1.04(-1.13 to -0.94)	4427.3(3991.8 to 4901.9)	92.8(83.2 to 102)	9037.5(8220.3 to 9803.8)	71.7(65.1 to 78.3)	-1.02(-1.13 to -0.91)	3551.4(2629.1 to 4538.6)	52.9(39 to 67.5)	7432.2(5349.3 to 9425.2)	42.5(30.6 to 54)	-0.93(-1.02 to -0.84)
Cuba	ICH	9605(8893 to 10364.4)	163.2(151.1 to 176)	4536.3(4168.9 to 4920.3)	79.6(72.8 to 87)	-2.71(-2.84 to -2.59)	2504.5(2324.5 to 2689)	43.4(40.4 to 46.5)	1390.8(1281.2 to 1506.8)	16.4(15.1 to 17.7)	-3.56(-3.76 to -3.36)	1715.9(1218.9 to 2233.4)	29.3(20.7 to 38.2)	852.3(611.8 to 1102.8)	14.7(10.3 to 19.3)	-2.64(-2.77 to -2.52)
Cyprus	ICH	20452.7(19129.6 to 21985.8)	193.8(181.2 to 208.4)	20360(19023.2 to 21743.5)	129.1(121.1 to 137.9)	-1.57(-1.65 to -1.49)	3654.4(3337.9 to 3978.5)	35.7(32.7 to 38.8)	3752.7(3396.6 to 4151.5)	20.7(18.8 to 22.9)	-2.09(-2.22 to -1.96)	1828.5(1317.7 to 2358.4)	17.4(12.5 to 22.4)	1918.6(1399.4 to 2482.1)	11.8(8.5 to 15.2)	-1.51(-1.59 to -1.43)
Czechia	ICH	1531.4(1424.4 to 1635.2)	187.2(174 to 199.7)	2276.9(2128.1 to 2441.9)	123.5(115.4 to 131.6)	-1.58(-1.65 to -1.51)	265.1(231.2 to 300.5)	33.3(29.3 to 37.3)	356.6(314.8 to 393.3)	18.7(16.5 to 20.5)	-2.3(-2.44 to -2.16)	204.7(148.5 to 263.5)	25.3(18.2 to 32.5)	314.6(223 to 408.1)	17(12.1 to 21.9)	-1.52(-1.6 to -1.45)
C 么 te d'Ivoire	ICH	16715.6(15484.2 to 18073.1)	140.2(129.8 to 151.5)	12859.9(11773.3 to 13944.6)	88.3(81.7 to 95.9)	-1.57(-1.71 to -1.43)	4061.6(3642.6 to 4456.9)	31.3(28.4 to 34.3)	2831.8(2494.3 to 3195.9)	14.4(12.8 to 16.2)	-2.71(-2.91 to -2.5)	3026.8(2133.8 to 3910.4)	25.3(17.8 to 32.7)	2391.8(1671.3 to 3167.4)	16.2(11.4 to 21.4)	-1.49(-1.61 to -1.36)
Democratic People's Republic of Korea	ICH	72700.3(68118.6 to 77799.3)	371.9(347.1 to 399.2)	96479.4(90954.1 to 102494.1)	288.9(272.5 to 306.9)	-0.92(-1.03 to -0.81)	18045.7(16340.2 to 19578.6)	123.1(111.6 to 133.9)	30962.4(28575 to 33440.3)	98.8(91.5 to 106.4)	-0.86(-1.03 to -0.69)	13875.7(9800.5 to 17800.7)	72.2(50.8 to 93.2)	18661.5(12946.6 to 24093.7)	56.3(39.1 to 72.7)	-0.91(-1.02 to -0.8)
Democratic Republic of the Congo	ICH	62530.9(58294.9 to 67357.4)	278.6(257.9 to 303.4)	113491.4(105398.2 to 122149.5)	205(189.2 to 222.4)	-1.14(-1.19 to -1.1)	13891.8(12233.3 to 15569.9)	86.3(76.7 to 95.4)	25441.4(23000 to 28131.1)	65.5(59.5 to 71.5)	-1.05(-1.12 to -0.98)	8244.1(5919.7 to 10599)	38.1(26.9 to 49.2)	15252.2(10766.2 to 19796)	29.5(20.9 to 39.1)	-0.94(-0.97 to -0.91)
Denmark	ICH	10013.3(9290.1 to 10787.4)	142.9(132.7 to 153.1)	9332.6(8690.4 to 10067.9)	101.5(94.3 to 109.3)	-1.22(-1.34 to -1.11)	1938.9(1722.9 to 2121.5)	24.6(22.1 to 26.9)	1500.4(1312.7 to 1678.9)	13.1(11.6 to 14.6)	-2.17(-2.29 to -2.05)	1396.6(986.1 to 1804.4)	19.5(13.7 to 25.3)	1381.1(962.3 to 1768)	14.4(10.1 to 18.6)	-1.06(-1.18 to -0.94)

Djibouti	ICH	714.1(666.8 to 763.9)	304.1(280.1 to 330)	2058.5(1920.5 to 2199.8)	212.7(197.2 to 228.8)	-1.38(-1.45 to -1.31)	130.7(115.2 to 146)	82(73.8 to 90.9)	366.6(323.3 to 407.8)	53.8(48.1 to 59.5)	-1.64(-1.74 to -1.53)	92.2(66.8 to 119.3)	41.8(30.2 to 53.9)	268.6(194.8 to 345.3)	29.5(21.2 to 38.5)	-1.33(-1.39 to -1.27)
Dominica	ICH	104.5(97.4 to 113.3)	174.1(162.4 to 187.9)	103.4(96.1 to 111.3)	130.6(121.3 to 140.3)	-1.12(-1.18 to -1.07)	21.9(19.7 to 23.9)	36.8(33.1 to 39.9)	20.7(18.7 to 22.9)	26.1(23.6 to 28.7)	-1.21(-1.32 to -1.1)	9.5(6.9 to 12.3)	15.8(11.6 to 20.4)	9.3(6.9 to 12.1)	11.9(8.7 to 15.4)	-1.12(-1.17 to -1.06)
Dominican Republic	ICH	10353.8(9721.6 to 11036.7)	210.5(197.2 to 224.3)	21391.2(19978.1 to 22890.3)	200.7(186.8 to 215.4)	-0.21(-0.25 to -0.18)	1742.1(1590.5 to 1902.8)	41.9(38.4 to 45.8)	4416.4(4033.4 to 4793)	43.2(39.5 to 46.9)	0.22(0.14 to 0.3)	910.1(663.4 to 1178)	19.1(13.8 to 24.7)	1897.9(1375.5 to 2479.1)	18(13 to 23.5)	-0.25(-0.3 to -0.21)
Ecuador	ICH	13047.2(12226.8 to 13924.5)	185.4(174.1 to 197.8)	21585.9(20034.7 to 23291.4)	124(115.2 to 133.6)	-1.51(-1.57 to -1.45)	1990.3(1808.3 to 2194.8)	32.2(29.2 to 35.3)	3087.2(2791.5 to 3384.5)	18.8(16.9 to 20.6)	-1.87(-1.95 to -1.78)	1146.9(837.9 to 1487)	16.7(12.1 to 21.6)	1961.9(1438.4 to 2529)	11.4(8.4 to 14.7)	-1.44(-1.5 to -1.38)
Egypt	ICH	65214.5(60620.1 to 70254.2)	156.6(145.4 to 169.5)	104296.5(96402.1 to 113113.9)	117.2(107.9 to 127.3)	-1.17(-1.23 to -1.11)	10958.8(9595.2 to 12317.7)	36.3(31.3 to 40.7)	16886.2(15018.5 to 18785.3)	25.8(23.1 to 28.2)	-1.37(-1.45 to -1.28)	9566.5(6815.4 to 12295.3)	23.2(16.7 to 30.2)	14781.8(10437.8 to 19036.6)	17(12 to 21.7)	-1.22(-1.28 to -1.17)
El Salvador	ICH	8562.1(8076.6 to 9142.7)	221.2(209.8 to 235.5)	7392(6865.8 to 7958.8)	116.3(108.1 to 125.4)	-2.5(-2.66 to -2.34)	1352.1(1236.1 to 1483.8)	39.5(35.9 to 42.9)	1245.8(1117.4 to 1386.5)	19(17.1 to 21)	-2.75(-3 to -2.51)	759.9(556 to 986)	20(14.7 to 25.8)	703.1(513.5 to 918.9)	10.9(8 to 14.3)	-2.32(-2.47 to -2.18)
Equatorial Guinea	ICH	827.7(767.1 to 891.5)	316.4(291.7 to 343.1)	1582.8(1473.1 to 1698.8)	182.1(168.8 to 196.6)	-2.06(-2.15 to -1.97)	203.4(182.5 to 227.5)	101.4(91.3 to 112)	257.7(227.6 to 291.5)	47.1(41.5 to 52.4)	-2.88(-3.1 to -2.66)	109.7(80.1 to 141.3)	43.4(31.1 to 56.3)	208.8(153.8 to 267.6)	25.6(18.7 to 33)	-1.98(-2.06 to -1.89)
Eritrea	ICH	6822.2(6370.3 to 7287.4)	359.4(331.6 to 387.3)	10516.1(9862.3 to 11189.5)	238.4(221.3 to 258.4)	-1.58(-1.67 to -1.49)	1550.3(1373.6 to 1719.5)	125.4(112.3 to 137.3)	2198.9(1978.7 to 2428.6)	70.3(64.5 to 76.6)	-2.22(-2.35 to -2.08)	877.4(646.2 to 1137.3)	49.3(35.6 to 64.6)	1381.3(970.9 to 1779.6)	33.7(24 to 44.4)	-1.45(-1.53 to -1.38)
Estonia	ICH	2517.5(2331.2 to 2710.3)	133.1(122.9 to 143.3)	1224.8(1122.4 to 1335.8)	67.1(61.3 to 73)	-2.63(-2.78 to -2.49)	692.1(630.3 to 750.4)	35.7(32.5 to 38.5)	275.8(241.8 to 308.4)	11.2(9.8 to 12.5)	-4.42(-4.72 to -4.12)	450.9(317.4 to 579)	23.8(16.8 to 30.6)	228.7(159.5 to 300.3)	12.3(8.6 to 16.1)	-2.53(-2.67 to -2.39)
Eswatini	ICH	876.9(809.7 to 949.7)	217.6(199.9 to 238.2)	1511.3(1402.8 to 1637.8)	199(184.4 to 215.1)	-0.37(-0.59 to -0.15)	212.9(190.1 to 233.6)	72.5(65.3 to 79.3)	353.2(319.1 to 383.1)	65.6(59.8 to 70.7)	-0.37(-0.62 to -0.13)	120.6(85.1 to 153.4)	31.6(22.1 to 40.5)	200.9(144.6 to 261.5)	28(20.2 to 35.9)	-0.46(-0.65 to -0.28)
Ethiopia	ICH	105888.5(92098.9 to 121088)	375.6(326.8 to 430.9)	111674.5(99979.3 to 125164.2)	175.6(156.5 to 198.8)	-2.94(-3.09 to -2.79)	25571.7(21612.9 to 29572.9)	121.5(102.6 to 140.6)	23136.4(20178.9 to 26013.5)	51.9(45.1 to 58.7)	-3.28(-3.49 to -3.07)	13626.4(9499.8 to 17819.5)	49.9(35.1 to 65.3)	15115.8(10677 to 19691.6)	25.1(17.4 to 32.8)	-2.65(-2.79 to -2.52)
Fiji	ICH	1853.4(1735.5 to 1974.3)	350(326.1 to 374.2)	2203.4(2072.8 to 2339.4)	246.5(231.4 to 262.2)	-1.36(-1.42 to -1.29)	322.7(290.6 to 354.1)	85.4(76.9 to 93.1)	383(348.3 to 423.7)	54(49.3 to 59)	-1.79(-1.9 to -1.67)	341.6(240.6 to 438.5)	65.5(46.1 to 83.7)	406.4(287.8 to 515.3)	46.2(32.5 to 58.2)	-1.34(-1.41 to -1.28)
Finland	ICH	11258.6(10561.2 to 12026)	171.6(160.7 to 183.2)	15838.5(14952.5 to 16771.6)	154.3(145.5 to 163.7)	-0.21(-0.53 to 0.11)	1603.6(1466.6 to 1746.4)	23.3(21.4 to 25.3)	2051.5(1812 to 2305.2)	17(15.2 to 18.9)	-1.09(-1.31 to -0.87)	1544.3(1126.1 to 1976.7)	23.3(17.1 to 30)	2315.6(1660.7 to 2972.7)	21.4(15.3 to 27.5)	-0.13(-0.45 to 0.19)
France	ICH	79671.6(73594.9 to 86411.9)	111.6(102.6 to 121.3)	102960(95646.2 to 110311.1)	94.5(87.2 to 101.9)	-0.65(-0.72 to -0.57)	14776.5(13486.1 to 15955.9)	18.3(16.7 to 19.7)	17253(15443.9 to 18888.4)	11.9(10.6 to 13.1)	-1.55(-1.67 to -1.43)	11803.9(8564 to 15208)	16(11.6 to 20.9)	16571.1(11695.9 to 21524.9)	14.1(10.1 to 18.4)	-0.49(-0.55 to -0.44)
Gabon	ICH	2127.1(1974.1 to 2310.1)	316.7(292.4 to 344.4)	2876.8(2684.6 to 3090.9)	208.2(193.5 to 225)	-1.62(-1.72 to -1.53)	485.9(431.9 to 539.7)	83.9(74.8 to 92.8)	549.5(487.5 to 617.1)	51.3(45.6 to 56.9)	-1.91(-2.03 to -1.78)	283.1(203.2 to 360.8)	43(31.1 to 54.8)	378.9(271.7 to 488.5)	28.5(20.3 to 37.1)	-1.57(-1.65 to -1.48)
Gambia	ICH	2011.4(1885.5 to 2151)	358.5(333.5 to 387.7)	4254.6(4011.7 to 4526.2)	290.4(271.4 to 311.7)	-0.82(-0.92 to -0.73)	323.8(289.3 to 360.9)	81.5(72.9 to 89.9)	761.5(688.1 to 837.4)	71.7(64.6 to 78.7)	-0.47(-0.53 to -0.42)	258.7(188.4 to 325)	48.1(35.2 to 60.6)	555.7(408.6 to 705.2)	39.5(29 to 49.9)	-0.76(-0.85 to -0.67)
Georgia	ICH	24004.3(22489.9 to 25581.9)	390.1(366.1 to 414.7)	11230.3(10440.8 to 12085.9)	229.5(215.1 to 244.6)	-1.96(-2.21 to -1.71)	7160.6(6624 to 7734.1)	122.1(113.3 to 131.5)	4232.1(3857.3 to 4600)	71.2(65.3 to 77)	-1.81(-2.09 to -1.52)	4301.2(2983.7 to 5520.4)	70(48.9 to 90)	2022.2(1420.1 to 2615.9)	40.8(28.6 to 53)	-1.99(-2.24 to -1.73)
Germany	ICH	158698.5(147344.9 to 170481.8)	145.2(134.7 to 156.1)	179961.3(168862.3 to 191748.5)	116.6(109 to 124.6)	-0.81(-0.85 to -0.76)	25898.5(23132.3 to 28625.5)	21.5(19.3 to 23.7)	24874.4(22250.4 to 27564.8)	13.3(11.9 to 14.9)	-1.74(-1.82 to -1.65)	21796(15520.2 to 27862.7)	19.6(13.9 to 25.2)	26183.3(18598.7 to 33720.1)	16.2(11.6 to 21)	-0.7(-0.75 to -0.65)
Ghana	ICH	41438(39132.4 to 44184.2)	436.4(407.2 to 467.6)	87941.6(83251.6 to 92915.2)	357.9(337.8 to 381.4)	-0.8(-0.92 to -0.68)	6742.9(6128.2 to 7415.9)	95.8(86.6 to 104.8)	14326.1(13067.2 to 15796.9)	77.8(70.6 to 85.2)	-0.69(-0.83 to -0.54)	5319.5(3810.3 to 6774.3)	58(41.6 to 74.1)	11325.4(8219.9 to 14283.7)	47.8(34.8 to 60.1)	-0.77(-0.88 to -0.66)
Greece	ICH	31480.3(29180.9 to 34213.7)	231(214.9 to 249.5)	26945.6(25254.1 to 28703.8)	151(142 to 160.2)	-1.65(-1.81 to -1.49)	6769.7(6143.4 to 7346.2)	47.4(43.2 to 51)	6576.6(5911.2 to 7195.3)	25.7(23.1 to 28)	-2.53(-2.71 to -2.34)	4325(3094 to 5611.6)	31.5(22.6 to 40.9)	3870.8(2785.1 to 4930.4)	20.5(14.8 to 26.2)	-1.67(-1.83 to -1.51)
Greenland	ICH	129.1(121.2 to 137.7)	296.3(277.4 to 317.2)	123.9(115.7 to 132.4)	183.6(171.1 to 196.7)	-1.95(-2.13 to -1.77)	18.4(16.6 to 20.2)	55(49.4 to 60)	19.8(17.8 to 22.1)	32.7(29.7 to 35.7)	-2.06(-2.21 to -1.9)	17.4(12.4 to 22.4)	41.1(29.4 to 52.5)	16.8(11.9 to 21.7)	25.6(18.4 to 32.9)	-1.92(-2.1 to -1.73)
Grenada	ICH	154.4(143.4 to 167.3)	228.6(213.7 to 246.4)	179.6(168.1 to 194)	154.5(144.6 to 166.4)	-1.53(-1.59 to -1.46)	41.4(38.4 to 44.7)	57.1(52.9 to 61.7)	36.4(33.3 to 39.3)	34.1(31.4 to 36.7)	-1.94(-2.1 to -1.78)	14.2(10.4 to 18.6)	20.4(14.8 to 26.6)	15.6(11.2 to 20.5)	13.8(10 to 17.9)	-1.53(-1.6 to -1.46)
Guam	ICH	273.8(255.4 to 292.3)	260.5(241.7 to 279.9)	471.4(440.9 to 504.1)	243.6(228.2 to 259.9)	-0.09(-0.17 to 0)	41.4(37 to 46)	54.6(49.1 to 60.7)	82.3(73.7 to 90.3)	40.1(36.1 to 44)	-1.17(-1.3 to -1.04)	50.6(36.4 to 65.6)	48.9(34.9 to 62.8)	88.8(62.5 to 114.1)	45.7(32.2 to 58.6)	-0.1(-0.18 to -0.01)
Guatemala	ICH	12880(12193.2 to 13629.9)	249.4(236.7 to 264.3)	20698.2(19490.7 to 22014.8)	156.5(147.5 to 166.6)	-1.89(-2.02 to -1.75)	1768.4(1634.5 to 1928.3)	41.1(38.2 to 44.2)	3272.4(3020.1 to 3568)	28.8(26.7 to 31.4)	-1.4(-1.51 to -1.29)	1091.4(787.2 to 1404.1)	21.8(15.8 to 27.7)	1867.3(1358.7 to 2427.8)	14.5(10.6 to 18.6)	-1.69(-1.82 to -1.56)
Guinea	ICH	15790.3(14831.9 to 16911.5)	385.6(361.8 to 415.3)	28477.6(26956.4 to 30191.3)	344.7(322.3 to 368.3)	-0.43(-0.48 to -0.37)	3282(2943 to 3607.9)	95.9(85.6 to 105.2)	5424.5(4991.4 to 5903.3)	87.6(80.2 to 95)	-0.29(-0.36 to -0.22)	2089.9(1507.8 to 2698.4)	52.2(37.3 to 67.3)	3725.5(2746.4 to 4680.2)	46.7(33.9 to 59.1)	-0.41(-0.47 to -0.36)
Guinea-Bissau	ICH	2807.3(2632.8 to 2985.5)	472.9(440.1 to 506.3)	4219.3(4011 to 4482.5)	341.7(320.8 to 364.7)	-1.23(-1.34 to -1.12)	541(487 to 599.5)	122.4(110.9 to 133.4)	775.9(710.2 to 845.6)	92.8(85.1 to 100.7)	-1.01(-1.08 to -0.94)	359(258.1 to 457.8)	62.6(44.9 to 79.8)	545.7(400.8 to 696.5)	46.4(33.6 to 58.7)	-1.14(-1.24 to -1.03)
Guyana	ICH	2043.1(1897.6 to 2199.2)	409.6(379 to 444.2)	1628.4(1534.8 to 1731.9)	223(209.9 to 237.6)	-2.22(-2.3 to -2.13)	505(467.7 to 538.4)	121.2(112 to 129.9)	390.1(358.8 to 420.4)	60(55.4 to 64.2)	-2.43(-2.58 to -2.27)	171.3(124 to 220.2)	35.1(25.5 to 45)	137.1(99.4 to 176.9)	19.3(14 to 24.9)	-2.16(-2.24 to -2.08)
Haiti	ICH	14202(13325.9 to 15260.5)	334.6(311.6 to 362)	22257.5(20891.8 to 23755.1)	223.6(208.4 to 239.8)	-1.49(-1.54 to -1.44)	3440.7(3106.8 to 3729.8)	99.8(90.7 to 108.2)	5047.4(4599.6 to 5478.2)	65.4(59.8 to 71)	-1.56(-1.65 to -1.46)	1204.9(864.9 to 1565.7)	29.4(21.2 to 37.9)	1913.4(1391.5 to 2477.3)	20.1(14.6 to 25.8)	-1.4(-1.45 to -1.35)
Honduras	ICH	5255.9(4924.3 to 5640.2)	172.6(161.7 to 184.1)	9992.4(9264 to 10830.4)	125.1(116.2 to 135.6)	-1.32(-1.42 to -1.23)	776.1(693.5 to 856.7)	33.5(30 to 36.9)	1807.4(1606 to 1997.4)	30.5(27 to 33.6)	-0.46(-0.54 to -0.37)	463.2(330.6 to 602.8)	15.9(11.4 to 20.4)	908.1(651.4 to 1190.8)	11.9(8.6 to 15.5)	-1.17(-1.26 to -1.08)
Hungary	ICH	24766.5(23092.6 to 26496.3)	196.2(183.9 to 209.3)	11912.4(10988.4 to 12944.2)	93(86.2 to 100.3)	-2.86(-3.01 to -2.72)	6564.9(6084.9 to 7097.5)	48.9(45.4 to 52.7)	3050(2730.8 to 3369.4)	17.1(15.3 to 19)	-3.92(-4.17 to -3.67)	4390.9(3139.7 to 5658.6)	34.8(25 to 44.8)	2159.5(1513 to 2819.3)	16.7(11.7 to 21.8)	-2.8(-2.94 to -2.65)
Iceland	ICH	442.7(414.7 to 473.5)	161(150.9 to 172.2)	523.5(491.5 to 558.8)	105.9(98.7 to 113.3)	-1.7(-1.83 to -1.56)	68.3(59.3 to 76.5)	23.7(20.7 to 26.7)	64.5(56.6 to 71.9)	11.4(10 to 12.9)	-2.88(-3.11 to -2.65)	61.3(42.9 to 78.8)	22(15.5 to 28.2)	74.9(54 to 96.2)	14.7(10.5 to 19)	-1.63(-1.77 to -1.5)
India	ICH	1306586.4(1152070 to 1486147.8)	223.9(196.6 to 254.9)	2609906.5(2326121.3 to 2944798.7)	198.8(177.2 to 223.7)	-0.4(-0.44 to -0.37)	286933.8(238808.9 to 334798.6)	62(51.4 to 72.3)	523412.7(446706.1 to 604614.7)	43.9(37.4 to 50.5)	-1.39(-1.52 to -1.26)	181441.6(127650.6 to 237422.7)	31.8(22.4 to 41.8)	366509.7(258840.9 to 475937.3)	28.3(20 to 36.5)	-0.38(-0.41 to -0.35)
Indonesia	ICH	578105.9(514582.2 to 656354.3)	432(382.4 to 490.4)	937897.7(828663.1 to 1062484.7)	321.1(284.1 to 361.5)	-1.1(-1.19 to -1.02)	112617.1(95480.6 to 129820.5)	108.4(90.2 to 124.1)	207346.2(176874.1 to 238265.9)	89.8(77.7 to 101.1)	-0.71(-0.81 to -0.61)	107742.9(75103.2 to 140873.8)	81.4(56.9 to 106.4)	174345.9(121793.7 to 226893.4)	60.7(42.5 to 78.6)	-1.09(-1.18 to -1)
Iran (Islamic Republic of)	ICH	52299.4(45211.4 to 59330.2)	127.8(112.5 to 144.1)	81265.7(72921.4 to 90120.1)	90.8(81.6 to 100.6)	-1.28(-1.33 to -1.22)	6842.8(5785.5 to 8081.6)	22.5(19.2 to 25.8)	10018.6(8702.4 to 11432.4)	13.3(11.5 to 15)	-1.93(-2.02 to -1.84)	7296.2(4975 to 9803.9)	18(12.6 to 23.9)	11514.2(8081.7 to 15129.6)	13(9.2 to 17)	-1.21(-1.27 to -1.16)
Iraq	ICH	33977(31905.7 to 36222.7)	284.3(267.3 to 303.4)	73418.7(69393.8 to 77414.8)	210.9(199.4 to 223.9)	-1.15(-1.24 to -1.06)	5875.3(5340 to 6377.3)	59.4(53.8 to 64.8)	11445.1(10430.8 to 12444.2)	43.4(39.8 to 46.9)	-1.2(-1.28 to -1.12)	4628.5(3333.8 to 5914.6)	38.9(28.4 to 49.3)	9852.8(7130.1 to 12666)	28.7(20.8 to 37)	-1.15(-1.24 to -1.07)
Ireland	ICH	6096.3(5668.9 to 6581)	158.1(147.4 to 169.9)	7062.1(6600.4 to 7577.1)	104.9(97.8 to 112.8)	-1.71(-1.86 to -1.56)	934.3(815.7 to 1048.9)	23.7(20.9 to 26.2)	877(770.3 to 984.6)	11.6(10.3 to 13.1)	-2.77(-2.94 to -2.59)	830.4(604.8 to 1076.4)	21.5(15.5 to 27.8)	998.7(729.1 to 1297.7)	14.5(10.6 to 19)	-1.62(-1.76 to -1.48)
Israel	ICH	6186.4(5706.4 to 6721.9)	130(120.4 to 141.1)	9435.4(8633.2 to 10197.1)	85.5(77.8 to 92.6)	-1.76(-1.91 to -1.61)	1274.1(1114.8 to 1423.8)	26.7(23.6 to 29.6)	1564.8(1381.4 to 1733.4)	12.5(11.1 to 14)	-2.96(-3.22 to -2.69)	861.7(609.9 to 1131.3)	18.1(12.7 to 23.6)	1384.6(964.1 to 1814.1)	12.3(8.6 to 16)	-1.64(-1.78 to -1.49)
Italy	ICH	99116.5(88726.1 to 110657.5)	134.2(119.7 to 149.4)	96753.3(85926.9 to 107521.9)	92.3(83.1 to 102.1)	-1.46(-1.56 to -1.37)	18650.3(15356.2 to 21977.5)	22.6(18.9 to 26.6)	19386.3(17001.1 to 21928.5)	13.1(11.6 to 14.6)	-1.84(-1.96 to -1.73)	13532.1(9459.1 to 17506)	18.1(12.6 to 23.7)	14663(10339.1 to 18945.3)	13.1(9.2 to 17.1)	-1.24(-1.31 to -1.18)
Jamaica	ICH	3950.7(3692.3 to 4263.9)	211.2(197.5 to 227.5)	5252.4(4898.5 to 5607.5)	170.8(159.4 to 182.5)	-0.82(-0.89 to -0.76)	851.7(775.2 to 924.5)	46.8(42.5 to 51)	1119.6(1022.1 to 1220.7)	36.2(33.1 to 39.5)	-0.97(-1.19 to -0.74)	359.3(263.3 to 472.8)	19.1(14 to 25.2)	485.3(346 to 637)	15.7(11.2 to 20.6)	-0.76(-0.82 to -0.7)
Japan	ICH	468341.2(415572.9 to 523645.2)	287.8(257.3 to 320.1)	578707.4(508783.3 to 643655.7)	222.9(200.4 to 246.8)	-0.98(-1.07 to -0.89)	54311.4(44019.7 to 64050.7)	33.8(27.7 to 39.6)	55281(48068.4 to 62042.6)	19.1(16.7 to 21.6)	-2.2(-2.45 to -1.95)	76507.1(54992.2 to 98082.3)	47.3(34 to 60.4)	98572.9(70094.9 to 126998.8)	36.2(25.8 to 46.6)	-1.02(-1.12 to -0.91)
Jordan	ICH	4195.2(3883.1 to 4532.5)	176.8(164.3 to 190.5)	12378.6(11512.2 to 13312.6)	112.9(105.2 to 120.9)	-1.75(-1.83 to -1.66)	621.3(551.8 to 698)	39.2(35 to 43.4)	1510.5(1354.3 to 1705.4)	19.7(17.8 to 21.6)	-2.75(-2.94 to -2.55)	595.7(430.4 to 786.1)	25.2(18.4 to 32.9)	1740.3(1237.9 to 2264.4)	16.2(11.6 to 21)	-1.72(-1.8 to -1.63)
Kazakhstan	ICH	37989.4(35393.7 to 40367.1)	260.9(243.3 to 278.7)	36471.6(34045.3 to 39032.5)	186.6(174.3 to 199.6)	-1.39(-1.68 to -1.09)	9267.6(8480 to 10032.4)	75.9(69.6 to 81.9)	9921.2(9131.8 to 10790.3)	62.4(57.2 to 67.8)	-0.85(-1.06 to -0.63)	6756.2(4761.3 to 8680)	46.8(33 to 59.8)	6532.6(4628.4 to 8504)	33.9(24.1 to 44)	-1.33(-1.59 to -1.06)
Kenya	ICH	30273.9(26491.4 to 34300.3)	245.9(216.4 to 279.1)	64229.7(57605.3 to 72139.8)	194.2(172.3 to 219.2)	-0.94(-1.06 to -0.82)	6300.4(5377.4 to 7243.4)	70.2(58.7 to 80.9)	12821.7(11113.4 to 14655.1)	55(47.6 to 62.3)	-0.9(-1.02 to -0.78)	3995.8(2786.1 to 5226.1)	33.8(23.9 to 44.2)	8493.5(5981.3 to 11089.9)	27.1(18.9 to 35.3)	-0.91(-1.02 to -0.8)
Kiribati	ICH	400.7(379.2 to 423.2)	804.6(759.4 to 856.3)	587.6(559.4 to 619.5)	592.5(560.7 to 625.1)	-1.08(-1.18 to -0.98)	81.5(74.3 to 88.2)	189.5(173.2 to 203.9)	114.9(106.3 to 123.4)	140.6(130.3 to 150)	-1.15(-1.23 to -1.08)	70.8(50.9 to 90.7)	143.1(103.2 to 182.5)	104.9(73.6 to 133.7)	106.9(75.6 to 136.7)	-1.04(-1.13 to -0.94)
Kuwait	ICH	1965.9(1816.5 to 2127.4)	149.6(139.2 to 160.9)	6477.8(6056.1 to 6900.1)	135.9(127.3 to 144.8)	-0.42(-0.59 to -0.26)	168.1(146.1 to 194.2)	18(16.2 to 20)	587.1(524.9 to 654.5)	16.9(15.1 to 18.8)	-0.17(-0.36 to 0.01)	268.5(191 to 350.6)	20.7(14.8 to 26.8)	889(638.7 to 1159.4)	19(13.9 to 24.7)	-0.39(-0.55 to -0.23)
Kyrgyzstan	ICH	9797.1(9180.2 to 10438.1)	293(273.8 to 312.7)	8826.4(8201 to 9456.8)	144.9(134.6 to 155.4)	-2.73(-2.97 to -2.5)	2610.5(2405.6 to 2838.8)	86.6(80.1 to 94)	2156.3(1978.4 to 2334.3)	42.7(39.3 to 46.4)	-2.75(-3.08 to -2.42)	1749.7(1229.5 to 2251.1)	52.5(36.9 to 67.5)	1576.9(1094.3 to 2043.9)	26.2(18.3 to 33.8)	-2.7(-2.93 to -2.47)

Lao People's Democratic Republic	ICH	10332.6(9606.3 to 11054)	386(355.4 to 414.6)	17739.5(16757.2 to 18849.6)	289.2(271.5 to 309)	-1.09(-1.21 to -0.98)	2633(2398 to 2867.9)	124.7(113.8 to 135.2)	4141.5(3786.2 to 4470.2)	87.1(80.3 to 93.9)	-1.41(-1.55 to -1.28)	1901.8(1337.7 to 2458.1)	71.6(50.3 to 92.6)	3288.2(2337.2 to 4260.4)	54.3(38.4 to 70.3)	-1.05(-1.16 to -0.93)
Latvia	ICH	4829.5(4475.9 to 5222.4)	145.3(134.5 to 157.3)	2323.4(2108.5 to 2536.6)	82.7(75.8 to 90.3)	-2.19(-2.33 to -2.06)	1722.1(1588.9 to 1865.5)	49.9(46.1 to 53.9)	725.5(661.3 to 791.4)	19.4(17.7 to 21.2)	-3.6(-3.84 to -3.37)	882.6(629 to 1120.6)	26.5(19 to 33.8)	434.9(305.6 to 573.7)	15.2(10.7 to 20)	-2.16(-2.3 to -2.02)
Lebanon	ICH	4914.3(4571.1 to 5268.6)	190.1(176.7 to 204.4)	7765.1(7288.6 to 8319)	131.3(123.3 to 140.6)	-1.4(-1.49 to -1.32)	794.6(708.3 to 886.2)	36.7(32.5 to 40.6)	1116.8(990.8 to 1244.6)	18.4(16.3 to 20.6)	-2.59(-2.74 to -2.45)	695.3(505.2 to 893.9)	27.4(19.7 to 35.1)	1120.6(814.2 to 1443.5)	18.8(13.7 to 24.3)	-1.41(-1.49 to -1.33)
Lesotho	ICH	1728.5(1579.8 to 1885.5)	176.6(160.6 to 195.2)	2840.2(2631.2 to 3065.1)	216.2(200.5 to 234.4)	0.82(0.6 to 1.04)	536.5(483 to 590.8)	65.3(58.6 to 71.1)	880.4(806 to 950.4)	86.6(79.2 to 93.3)	1.18(0.93 to 1.42)	258.4(179.1 to 336.3)	27.6(19 to 36.7)	386.4(277.8 to 492.7)	30.9(22.2 to 40)	0.5(0.32 to 0.67)
Liberia	ICH	6104.4(5739.3 to 6521.4)	388.3(361.9 to 417.4)	9601(9042.2 to 10170.5)	269.6(252.3 to 289.2)	-1.29(-1.33 to -1.25)	1102.1(1001.6 to 1212.2)	88.1(79.2 to 96.4)	1547.9(1397.6 to 1701.3)	63.5(57.4 to 69.4)	-1.19(-1.25 to -1.13)	780(560.4 to 999.7)	51(37.1 to 64.8)	1232(905.4 to 1568.8)	36.4(26.5 to 46.4)	-1.2(-1.24 to -1.17)
Libya	ICH	5678.1(5325.4 to 6071.2)	182.2(170.8 to 194.6)	9191.2(8656.8 to 9797.1)	126.9(119.1 to 135.4)	-1.35(-1.42 to -1.29)	665.9(592 to 746)	26.1(23.4 to 28.8)	1031.2(924.8 to 1142.7)	18.2(16.2 to 20.1)	-1.47(-1.61 to -1.32)	785.5(562.1 to 1024)	25.5(18.2 to 33.3)	1268.9(925.3 to 1640)	17.8(13 to 22.8)	-1.35(-1.41 to -1.28)
Lithuania	ICH	4578.4(4224.3 to 4956)	107.5(99.1 to 116.3)	3631.6(3331.2 to 3938.7)	86.7(80 to 94.3)	-0.64(-0.69 to -0.6)	1115.3(1017.8 to 1208.2)	25.7(23.5 to 27.9)	919.1(823.9 to 1002.7)	17.6(15.8 to 19.2)	-1.13(-1.2 to -1.06)	824.2(578.4 to 1083.2)	19.4(13.5 to 25.4)	670.8(467.2 to 871.2)	15.8(11.1 to 20.5)	-0.59(-0.63 to -0.55)
Luxembourg	ICH	747.9(687.2 to 817.5)	154(142.4 to 167.6)	775.1(715.1 to 836.2)	86.6(79.3 to 93.9)	-2.44(-2.65 to -2.22)	145.2(133.3 to 157.5)	28.1(25.7 to 30.3)	121.3(109.7 to 133)	11.4(10.2 to 12.5)	-3.38(-3.62 to -3.15)	102.2(72.2 to 133.5)	20.9(14.7 to 27.4)	113.9(80.2 to 146.5)	12.4(8.7 to 15.9)	-2.26(-2.49 to -2.02)
Madagascar	ICH	33924(32090.4 to 35964.1)	485(455.9 to 517.6)	60235.5(56609.7 to 63779.9)	333(310 to 358.2)	-1.38(-1.44 to -1.32)	7186.1(6599.4 to 7759.1)	125.8(115.5 to 136.3)	11931.9(10868.7 to 13043.2)	91(83.3 to 99.4)	-1.28(-1.39 to -1.18)	4393.7(3172.1 to 5617.5)	64.8(46.6 to 82.2)	7920.3(5668.3 to 10269.8)	46.6(32.9 to 60.1)	-1.21(-1.27 to -1.16)
Malawi	ICH	14364.5(13459 to 15393.8)	256.5(238 to 277.2)	21182.5(19771.6 to 22630.9)	182.3(169.9 to 197.5)	-1.34(-1.47 to -1.22)	3271(2941.9 to 3617.4)	79.7(71.6 to 88.2)	4424.5(4032.8 to 4808.7)	54.8(50 to 59.7)	-1.48(-1.61 to -1.35)	1902.9(1384.9 to 2432)	35.6(25.8 to 46.2)	2857.3(2066.3 to 3731.9)	26.3(18.7 to 34.6)	-1.19(-1.29 to -1.09)
Malaysia	ICH	47439(44752.5 to 50564.4)	372.3(348 to 400.6)	87576.6(82471.8 to 92850.8)	274.9(259.3 to 292.2)	-1.04(-1.1 to -0.99)	9462.4(8565.9 to 10339.1)	96.3(86.6 to 105.4)	15319.3(13746.6 to 16799.3)	56.5(50.6 to 61.5)	-1.87(-1.98 to -1.76)	8749.2(6258.6 to 11333.8)	69.3(49.4 to 89)	16291.2(11434.4 to 20889.9)	51.6(36.3 to 66.3)	-1.01(-1.07 to -0.96)
Maldives	ICH	627.7(593 to 666.5)	463(432.5 to 494.6)	1113.8(1054.3 to 1178.2)	218.7(206.4 to 233.1)	-2.85(-3.02 to -2.69)	103.2(93 to 112.6)	106.5(96.3 to 115.3)	161.8(146.2 to 178.8)	44.9(40.3 to 49.2)	-3.25(-3.51 to -2.98)	114.4(81.6 to 148.3)	85.5(60.9 to 111.4)	201.6(139.5 to 258.9)	40.8(28.9 to 52)	-2.8(-2.95 to -2.64)
Mali	ICH	18393.8(17227.7 to 19796.2)	332.7(310.5 to 359.8)	36216.1(34236.9 to 38447.8)	259.8(244.5 to 278.1)	-0.97(-1.03 to -0.92)	3502.3(3154.5 to 3835.4)	88.9(79.5 to 97.2)	6101.3(5541 to 6716.7)	68.5(61.7 to 74.4)	-0.96(-1.03 to -0.89)	2399.5(1722.1 to 3096.4)	45.1(32.2 to 57.7)	4739.7(3394.4 to 6106.7)	35.7(26 to 45.4)	-0.91(-0.96 to -0.86)
Malta	ICH	713.5(667.2 to 763.6)	172.5(161.5 to 184.5)	955(891.8 to 1012.5)	125.6(117.3 to 133.5)	-1.19(-1.31 to -1.07)	121.4(106.8 to 136.4)	29.5(26.1 to 33)	142.1(123.4 to 163)	15.2(13.4 to 17.4)	-2.27(-2.42 to -2.12)	95.5(68.8 to 122.2)	23.2(16.8 to 29.7)	136.9(96.4 to 176.6)	17.3(12.2 to 22.2)	-1.07(-1.17 to -0.96)
Marshall Islands	ICH	109.7(103.4 to 116.2)	486.5(454.8 to 521.6)	207.2(195.3 to 219.6)	426.6(400.3 to 454.1)	-0.45(-0.61 to -0.3)	25(22.7 to 27.2)	144.5(131.1 to 156.3)	42.7(39 to 46.6)	121.5(112.4 to 130.9)	-0.67(-0.8 to -0.55)	20(14 to 25.8)	89.3(62.5 to 115.2)	37.1(26.5 to 46.9)	77.6(55.6 to 98.2)	-0.48(-0.63 to -0.33)
Mauritania	ICH	5277.4(4928.1 to 5649.8)	405(376.8 to 437.6)	7803.6(7323.6 to 8317)	269.8(251.7 to 289.8)	-1.54(-1.61 to -1.47)	943.3(840.1 to 1049.2)	88.7(78.8 to 97.9)	1267.9(1133.6 to 1399.4)	56.4(50.2 to 61.9)	-1.63(-1.72 to -1.53)	691.6(503.2 to 888.5)	54.6(39.3 to 69.9)	1042.8(750.5 to 1330.7)	37.4(26.7 to 47.3)	-1.43(-1.49 to -1.37)
Mauritius	ICH	2825.8(2636 to 3017.9)	312(291.3 to 334.7)	3203.1(3000.4 to 3419.9)	189.8(178.6 to 201.9)	-1.92(-2.04 to -1.8)	560.4(512 to 612.5)	73.3(67.2 to 79.5)	575.2(514.9 to 643.5)	34(30.6 to 37.7)	-3.14(-3.4 to -2.89)	520.2(373.4 to 664.8)	57.8(41.3 to 73.6)	589.2(428.5 to 755.9)	35(25.4 to 45)	-1.92(-2.04 to -1.8)
Mexico	ICH	111630.5(97375.3 to 126039.6)	182.5(162.6 to 202.7)	170533.6(152715 to 189257.9)	128.9(116.2 to 142.6)	-1.35(-1.41 to -1.28)	13976.5(11884.1 to 16180.7)	29.1(24.9 to 33.4)	21742.8(18800.2 to 24734.5)	17.7(15.4 to 20.1)	-1.9(-2.02 to -1.77)	9819.6(7046.1 to 13024.2)	16.6(12.1 to 21.6)	15387.8(11072.1 to 20206.2)	11.8(8.5 to 15.5)	-1.35(-1.42 to -1.28)
Micronesia (Federated States of)	ICH	326.3(306.6 to 348.2)	545.7(509.6 to 583.8)	407.9(383.4 to 433.1)	436.2(407.8 to 465.1)	-0.77(-0.85 to -0.7)	76.3(69.1 to 83)	152.7(138 to 165.7)	87.5(79.4 to 94.6)	121.2(111.2 to 131.1)	-0.84(-0.89 to -0.8)	59.6(42.5 to 76.3)	100.1(70.6 to 128.2)	73.9(51.5 to 95.3)	80.2(55.8 to 103.5)	-0.78(-0.85 to -0.7)
Monaco	ICH	76.5(70.4 to 83.2)	138.8(128.5 to 149.6)	79.3(73.8 to 84.8)	109.9(102.6 to 117.1)	-0.95(-1.02 to -0.89)	14.4(12.4 to 16.4)	21.4(18.6 to 23.9)	11.7(10.1 to 13.3)	12.7(11.1 to 14.2)	-1.94(-2.09 to -1.79)	11.3(8 to 14.5)	19.5(13.8 to 25.2)	11.8(8.4 to 15)	15.4(11 to 19.7)	-0.96(-1.02 to -0.9)
Mongolia	ICH	6240.7(5873 to 6614.4)	467.1(437.3 to 497.7)	11621.7(10920.9 to 12330.4)	382.2(357.3 to 408)	-0.78(-1.05 to -0.51)	1714.1(1569.8 to 1846.8)	162.6(149.3 to 174.7)	3383.5(3155.9 to 3621)	153.3(142.6 to 164.8)	-0.35(-0.69 to 0)	1116.3(785.8 to 1429.6)	84(58.8 to 107.7)	2041.7(1456.1 to 2611.2)	68.9(48.9 to 87.8)	-0.75(-1.02 to -0.49)
Montenegro	ICH	1952.4(1818.3 to 2091.5)	302.2(280.7 to 324.2)	1732.7(1577.1 to 1928)	211.6(193.6 to 233)	-1.28(-1.44 to -1.11)	801.7(743 to 860)	138(128.1 to 148.1)	1057.7(974.2 to 1138.3)	122.1(113.4 to 130.6)	-0.6(-0.8 to -0.41)	360.3(256.9 to 468.9)	56.2(40.1 to 73.3)	330.9(236.1 to 427.8)	40.1(28.8 to 52.2)	-1.2(-1.35 to -1.04)
Morocco	ICH	38362.3(35806.9 to 41070.7)	202.6(188.9 to 217.5)	51538.9(47861 to 55329.9)	136.6(126.6 to 146.6)	-1.58(-1.7 to -1.47)	6940.2(6240.4 to 7659.5)	44.7(40.1 to 48.9)	9577.9(8580.6 to 10592.7)	29(26.1 to 32)	-1.67(-1.78 to -1.57)	5294(3861.5 to 6963)	28.1(20.5 to 36.7)	7015.9(4942 to 8973.9)	18.8(13.2 to 23.9)	-1.58(-1.69 to -1.47)
Mozambique	ICH	23187.1(21535.4 to 24908.4)	287.3(263.9 to 313.9)	47771.5(44976.2 to 50931)	281.2(261.5 to 305.5)	0(-0.07 to 0.08)	6095(5445.1 to 6681.3)	97.6(87.3 to 107.3)	11381.2(10389.2 to 12382.6)	89.2(82 to 96.5)	-0.22(-0.27 to -0.17)	3197.3(2294.7 to 4107.1)	42.1(29.9 to 55.2)	6432.1(4597.1 to 8293.3)	40.8(28.7 to 53.8)	-0.03(-0.09 to 0.02)
Myanmar	ICH	112267.6(105007.1 to 120400.9)	385.1(359.8 to 415.1)	149593(140405.2 to 158786.1)	267.6(250.9 to 284.7)	-1.37(-1.57 to -1.18)	28922.8(26115.4 to 31675.4)	124.6(112.9 to 136.1)	38111.6(35041.3 to 41460.5)	79.1(72.8 to 85.3)	-1.8(-2.03 to -1.56)	20682.9(14454.7 to 26604)	71.5(50 to 92.4)	27821.5(19965.2 to 35997.4)	50.3(36.2 to 65.2)	-1.33(-1.52 to -1.14)
Namibia	ICH	2094.1(1933 to 2262.9)	260(240.4 to 282.2)	3133(2922 to 3372.4)	180.1(167.5 to 195.5)	-1.45(-1.6 to -1.3)	517.8(461.2 to 569.9)	81.5(73.1 to 89.2)	746.8(681.7 to 819.6)	55.9(51.1 to 61.2)	-1.6(-1.78 to -1.41)	290.1(211.4 to 372.7)	37.5(27.1 to 48.7)	440(310 to 567.4)	26.8(18.7 to 35.2)	-1.31(-1.44 to -1.18)
Nauru	ICH	41.5(39.1 to 44)	627.4(584.8 to 669.7)	39.5(37.6 to 41.7)	473.6(448 to 502.7)	-1.06(-1.2 to -0.92)	6.9(6.2 to 7.7)	131.9(118.5 to 144.5)	6.5(5.9 to 7.1)	88(80.1 to 95.1)	-1.54(-1.73 to -1.35)	7.5(5.4 to 9.5)	114.7(81.5 to 146)	7.2(5.1 to 9.3)	86.6(60.8 to 110.6)	-1.05(-1.18 to -0.91)
Nepal	ICH	28424.4(26176.1 to 31008.9)	239.2(218.6 to 261.5)	46846(42948.5 to 50672.7)	180.8(165.2 to 195.6)	-1.05(-1.09 to -1.01)	6276.1(5452.6 to 7077.3)	65.4(57.4 to 73)	10731(9411.2 to 12148.1)	47.6(42.2 to 53.7)	-1.14(-1.24 to -1.03)	3970.4(2830.9 to 5149.3)	34.1(24.3 to 43.7)	6693.7(4798.5 to 8608.8)	26.2(18.8 to 33.6)	-0.99(-1.03 to -0.95)
Netherlands	ICH	24951.2(23302.3 to 26705.9)	136.3(126.9 to 145.6)	28411.8(26438.3 to 30410.2)	103.3(95.9 to 111)	-1.03(-1.16 to -0.9)	4200.7(3805.6 to 4518.8)	21.5(19.6 to 23.2)	4448.7(3919.2 to 4965.8)	13.1(11.6 to 14.6)	-1.78(-1.93 to -1.63)	3504(2498.3 to 4508.1)	18.9(13.5 to 24.4)	4161.7(2991 to 5328.6)	14.6(10.5 to 18.6)	-0.95(-1.08 to -0.81)
New Zealand	ICH	3999.5(3512.3 to 4497.2)	106.6(93.9 to 119.2)	5533.3(4898.9 to 6168)	79.5(71.1 to 88.1)	-1.11(-1.23 to -0.98)	694.1(573.9 to 813.5)	18.1(15 to 21)	830.6(719.9 to 950.1)	10.1(8.8 to 11.5)	-2.02(-2.18 to -1.85)	579.3(419.4 to 765.7)	15.4(11.2 to 20.2)	836.8(588.4 to 1083.3)	11.8(8.3 to 15.4)	-1.02(-1.14 to -0.91)
Nicaragua	ICH	4313.8(4013.4 to 4678.6)	192.4(178.8 to 207.5)	7511.4(7023.4 to 8069.6)	130.1(121.5 to 140.1)	-1.52(-1.59 to -1.45)	727.8(653.2 to 810)	42.7(38.2 to 47.1)	1164.4(1041.9 to 1294.1)	23.7(21.2 to 26.4)	-2.21(-2.35 to -2.07)	385.1(277.1 to 500.5)	17.9(13 to 22.9)	683.3(492.3 to 882.3)	12.2(8.8 to 15.6)	-1.5(-1.57 to -1.43)
Niger	ICH	17380.6(16315.3 to 18459)	383.1(354.4 to 411.2)	38254.2(36244 to 40356.6)	286.2(267.6 to 304.4)	-1.14(-1.19 to -1.08)	3098.7(2800.3 to 3424.8)	97.7(87.7 to 107.5)	6894.7(6319.4 to 7564.8)	76.4(69.1 to 82.7)	-0.93(-0.98 to -0.88)	2250.5(1625.8 to 2885.8)	51.9(37.1 to 65.8)	5086(3699.6 to 6524.3)	40.1(29.1 to 51.6)	-1.01(-1.06 to -0.96)
Nigeria	ICH	186028.2(164098.2 to 212031.7)	321.3(283.3 to 367.1)	330730.7(295042.2 to 371440.8)	238.4(213 to 268.6)	-1.13(-1.21 to -1.05)	39616.7(33629.9 to 45908.3)	88.7(74.2 to 102)	54200.5(46890.6 to 61164.5)	56.4(48.5 to 63.8)	-1.73(-1.86 to -1.6)	24257.6(16929.7 to 31746.4)	43.2(30.6 to 56.2)	43579.5(30971.2 to 56005.7)	32.9(23.1 to 42.2)	-1.04(-1.11 to -0.97)
Niue	ICH	9(8.4 to 9.7)	432.6(401.9 to 464.6)	6.7(6.3 to 7.1)	329.3(310.6 to 348.9)	-1.05(-1.13 to -0.97)	2.3(2 to 2.5)	100(89.4 to 109.5)	1.4(1.3 to 1.5)	68.8(62.5 to 75)	-1.44(-1.53 to -1.34)	1.7(1.2 to 2.2)	80.6(57.4 to 102.8)	1.2(0.9 to 1.6)	60.8(42.9 to 77.8)	-1.07(-1.14 to -0.99)
North Macedonia	ICH	4627(4346.4 to 4936.3)	225.9(212.2 to 240.7)	4139.7(3848.9 to 4441.3)	144.4(134.9 to 154.5)	-1.75(-1.87 to -1.64)	1279.7(1174.1 to 1369.2)	72.2(66.8 to 77.5)	1476(1350.6 to 1592.2)	54.1(49.8 to 57.7)	-1.2(-1.32 to -1.08)	834.1(589 to 1074.1)	41(28.9 to 52.8)	752.1(525.2 to 973.3)	26.4(18.6 to 34.3)	-1.72(-1.83 to -1.61)
Northern Mariana Islands	ICH	116.3(109.4 to 124.3)	369.1(343.2 to 397.1)	158.6(148.3 to 169.5)	271.2(254 to 290.1)	-1.18(-1.25 to -1.11)	17.3(15.1 to 19.6)	82.6(73.5 to 91.1)	28.5(25.4 to 31.9)	60.6(54.5 to 66.5)	-1.09(-1.14 to -1.04)	20.9(14.7 to 26.9)	68.3(47.8 to 86.8)	28.8(20.7 to 37.1)	50.4(36 to 64.8)	-1.17(-1.24 to -1.1)
Norway	ICH	7468.2(6614.4 to 8368)	126.5(112.7 to 141.9)	8632.8(7585.6 to 9605.9)	100.5(90.1 to 111.9)	-0.93(-1.1 to -0.75)	1302.4(1055.8 to 1564.3)	19.4(15.9 to 22.9)	1223.4(1041.7 to 1412.9)	12.3(10.5 to 14.1)	-1.92(-2.15 to -1.69)	1051.6(745.8 to 1360.1)	17.3(12.5 to 22.5)	1258.1(884.4 to 1626)	14.2(10.1 to 18.3)	-0.81(-0.97 to -0.64)
Oman	ICH	2897.9(2696.3 to 3112.5)	215.8(200.9 to 230.5)	6146.4(5749.5 to 6578)	146.7(138 to 156.6)	-1.45(-1.53 to -1.36)	417.1(373.6 to 462.2)	47.7(42.7 to 52.6)	622.3(556.9 to 688)	28.1(25.4 to 31.1)	-1.95(-2.05 to -1.85)	393.7(287.5 to 518.3)	29.7(21.9 to 39)	829.2(597 to 1092.9)	20.3(14.9 to 26.2)	-1.41(-1.5 to -1.33)
Pakistan	ICH	207169.4(181824.7 to 237171.7)	309.5(269.5 to 354.2)	412066(367895.7 to 465591.1)	256(225.9 to 288.3)	-0.77(-0.88 to -0.66)	45396.5(37747.9 to 52577.2)	78.2(64.5 to 90.3)	73837.6(62786.7 to 84518.1)	55.9(47.7 to 63.9)	-1.38(-1.57 to -1.19)	28922.9(19887.6 to 37663)	43.7(30.2 to 56.2)	56873.1(40332.8 to 73503.6)	36(25.6 to 46.5)	-0.78(-0.88 to -0.67)
Palau	ICH	55.2(51.7 to 59.1)	461.7(430.9 to 496)	98.4(92.6 to 105.3)	392.3(369.1 to 419)	-0.59(-0.67 to -0.5)	11.4(10.3 to 12.6)	114.4(102.6 to 125)	17.7(15.9 to 19.6)	85.4(78.2 to 93.7)	-1.09(-1.15 to -1.03)	10.1(7.1 to 13.2)	85.3(59.8 to 111)	17.6(12.4 to 22.3)	71.1(50.5 to 90.1)	-0.64(-0.72 to -0.56)
Palestine	ICH	2076.8(1914.9 to 2253.6)	160.5(148.8 to 174.1)	4227.8(3903.4 to 4559.4)	106.6(99.2 to 114.5)	-1.45(-1.68 to -1.22)	446.4(403.2 to 491.6)	50.2(45.3 to 54.8)	682.9(620.5 to 748.9)	29.6(27.1 to 31.9)	-2.05(-2.49 to -1.61)	297.7(212.7 to 387.8)	23.2(16.8 to 30)	605.3(427.7 to 783.9)	15.6(11 to 20)	-1.41(-1.63 to -1.19)
Panama	ICH	3821.7(3572.9 to 4090)	207.8(194.4 to 222.1)	6592.5(6178.2 to 7027.2)	148.9(139.5 to 158.8)	-1.29(-1.36 to -1.22)	613(553.4 to 673.2)	38.8(34.7 to 42.6)	1002.6(897.9 to 1103.4)	22.5(20.2 to 24.8)	-1.99(-2.1 to -1.89)	343(249 to 445.1)	19.1(13.9 to 24.8)	616.7(450.8 to 799.2)	13.9(10.1 to 18)	-1.25(-1.32 to -1.18)
Papua New Guinea	ICH	6013(5563.4 to 6523.1)	246(225.2 to 267.8)	14669.8(13659.1 to 15820)	209.4(192.9 to 226)	-0.64(-0.72 to -0.56)	1355(1194.9 to 1525.5)	85.5(76.1 to 94.6)	3309.6(2954.4 to 3647.2)	69.7(63 to 76.3)	-0.78(-0.85 to -0.71)	1094.3(762.5 to 1419.2)	46(32 to 59.1)	2685.8(1879.7 to 3508.9)	39.5(27.6 to 50.9)	-0.61(-0.69 to -0.53)
Paraguay	ICH	5839.9(5443.7 to 6248.7)	211.1(196.9 to 225.6)	8560.1(7951 to 9232.1)	129.5(120.5 to 139.7)	-1.89(-1.99 to -1.8)	1140.6(1051.5 to 1236.3)	47.2(43.6 to 51)	1475.4(1341.9 to 1615.9)	24.6(22.3 to 26.8)	-2.41(-2.55 to -2.28)	510.3(373.2 to 658.2)	18.9(13.7 to 24.1)	762.4(538.7 to 990.2)	11.7(8.3 to 15.2)	-1.83(-1.92 to -1.74)
Peru	ICH	31824.9(29799.3 to 33936.1)	197.2(184.5 to 211.1)	43642.2(40725.4 to 46444.5)	120.6(112.4 to 128.4)	-1.82(-1.92 to -1.72)	4743.6(4284.5 to 5234.7)	34(30.5 to 37.4)	5497.2(4955.8 to 6081.1)	15.8(14.2 to 17.5)	-2.73(-2.92 to -2.53)	2826.2(2023.3 to 3699.3)	18(13 to 23.4)	4072.6(2971 to 5293.2)	11.4(8.3 to 14.8)	-1.71(-1.8 to -1.62)
Philippines	ICH	80398.8(71216 to 90627)	194.9(172.9 to 220.1)	260764.2(233404.2 to 292700.8)	258.2(229.5 to 291.4)	1.13(0.9 to 1.37)	14628.2(12301.7 to 16907.9)	49.3(41 to 57)	54562.9(47209.6 to 62615.9)	63.9(55.5 to 72.2)	1.03(0.67 to 1.39)	14993.2(10194.6 to 19804.8)	36.7(25.3 to 48.4)	48066.1(33938.6 to 63207.7)	48.1(34.1 to 63)	1.09(0.86 to 1.32)
Poland	ICH	59442.9(52643.3 to 66630.9)	144(127.8 to 161.4)	40244.9(36015.4 to 45299.9)	77.6(69.7 to 87)	-2.45(-2.58 to -2.32)	14587.1(12195.9 to 16772.8)	35.7(30.1 to 41)	10412.9(8928.1 to 11847.7)	15.6(13.5 to 17.6)	-3.17(-3.36 to -2.97)	10793(7495.3 to 14047.3)	26.2(18.1 to 34.1)	7749.3(5420.4 to 10164.1)	14.6(10.1 to 19.3)	-2.31(-2.44 to -2.18)

Portugal	ICH	28715.2(26519.7 to 30921.3)	226.2(210.5 to 242.3)	17968.8(16440.3 to 19466.8)	101(92.3 to 109.8)	-3.2(-3.49 to -2.9)	6692.4(6042.8 to 7311.9)	51.5(46.8 to 55.7)	3922.3(3569.5 to 4263.8)	15.7(14.2 to 17.1)	-4.61(-4.93 to -4.28)	3941.5(2810.5 to 5059.6)	30.9(22 to 39.7)	2686.6(1914.5 to 3501.6)	14.2(10.2 to 18.7)	-3.07(-3.35 to -2.78)
Puerto Rico	ICH	6013.6(5613.9 to 6473.6)	167.6(156.7 to 180.2)	6306.3(5880.1 to 6742)	125.1(116.9 to 133.9)	-0.9(-1.12 to -0.69)	837.5(747.6 to 926.6)	23.7(21.2 to 26.2)	894.4(791.9 to 990.1)	13.6(12.1 to 15.2)	-2.1(-2.24 to -1.96)	536.5(388.1 to 699.2)	15(10.8 to 19.5)	628.1(449.4 to 797.2)	11.5(8.2 to 14.9)	-0.83(-1.05 to -0.62)
Qatar	ICH	996(929.3 to 1062.4)	285.9(268 to 306.2)	4773.8(4469.3 to 5086.6)	168(157.7 to 179.1)	-1.94(-2.09 to -1.79)	97.3(86.7 to 108.3)	46.9(42.1 to 51.5)	340.3(291.5 to 391.2)	25.7(23.2 to 28.1)	-2.02(-2.1 to -1.94)	131.6(94 to 170)	38.8(27.8 to 49.7)	630.1(447.9 to 833)	23.1(16.7 to 29.6)	-1.91(-2.06 to -1.77)
Republic of Korea	ICH	252574.3(239505.9 to 266773.3)	722.8(683 to 765.8)	216556.9(204152.2 to 228038.2)	260.7(246.3 to 274.2)	-3.89(-4.23 to -3.56)	32436.7(28863.5 to 36141.6)	106(95.1 to 117)	20451.7(17865.9 to 22876.1)	24.2(21.3 to 27.1)	-5.6(-6 to -5.2)	39891.2(29039.6 to 51074.5)	116.3(84.5 to 148.6)	35470.6(25495.6 to 45210)	42.7(30.9 to 54.5)	-3.83(-4.15 to -3.5)
Republic of Moldova	ICH	10581.8(9744.8 to 11462.2)	229.4(211.4 to 247.7)	6267.6(5798.5 to 6765.4)	121.2(112.3 to 130.4)	-2.44(-2.62 to -2.26)	3499.5(3239.4 to 3794.8)	82.5(76.9 to 89)	1905.9(1742.9 to 2086)	33.5(30.7 to 36.6)	-3.39(-3.68 to -3.1)	1902.1(1356.7 to 2427.4)	41.5(29.6 to 53)	1142.6(824 to 1471.6)	22(15.8 to 28.3)	-2.43(-2.61 to -2.25)
Romania	ICH	50557.8(46880.2 to 54430.4)	188.2(175.2 to 202)	29520.8(27280.5 to 32074.7)	112.4(104.5 to 121)	-1.97(-2.15 to -1.79)	16218.1(14949.6 to 17495.8)	62.1(57.7 to 66.8)	11302.3(10511.2 to 12134.5)	31.5(29.3 to 33.7)	-2.61(-2.8 to -2.42)	8986.7(6414.9 to 11548.7)	33.6(24 to 43)	5446.2(3848.9 to 7142.6)	20.5(14.2 to 27)	-1.89(-2.07 to -1.71)
Russian Federation	ICH	268297.1(235228.8 to 301839.4)	152.1(134.1 to 170)	239832.1(210560.6 to 272090.9)	118.4(105.3 to 133.6)	-1.1(-1.39 to -0.8)	74551.1(61573.8 to 86195.9)	44(36.5 to 50.5)	63551.7(54799.3 to 71646.2)	28.7(25 to 32.4)	-1.88(-2.26 to -1.5)	48083.5(33879.7 to 62075.4)	27.4(19.4 to 35.4)	43135.1(30099.5 to 56040.5)	21.2(15 to 27.8)	-1.09(-1.36 to -0.82)
Rwanda	ICH	18493.3(17321.6 to 19690.8)	469(435.9 to 503.7)	17903.7(16730.6 to 19312.4)	201.6(186.6 to 218.6)	-3.29(-3.47 to -3.12)	4713.2(4237.6 to 5161.7)	153(137.7 to 166.4)	4097.3(3676 to 4546.5)	65.6(59.1 to 72)	-3.38(-3.65 to -3.11)	2429.4(1741.3 to 3089.8)	63.8(45.9 to 81.7)	2447(1751.9 to 3196.2)	29.5(21 to 38.9)	-2.98(-3.13 to -2.83)
Saint Kitts and Nevis	ICH	119.6(110.3 to 130.8)	363.2(336.6 to 392.6)	135(123.9 to 147.1)	184.7(169.5 to 201.3)	-2.73(-2.87 to -2.58)	33.7(31.2 to 36.6)	93.7(86.6 to 100.6)	28.7(26.1 to 31.6)	43.8(40.5 to 47.6)	-2.89(-3.11 to -2.66)	10.8(7.7 to 14.1)	32(23.1 to 41.1)	11.6(8.6 to 15.2)	16.5(12.2 to 21.5)	-2.67(-2.81 to -2.53)
Saint Lucia	ICH	247.5(231.3 to 265.7)	251.5(234.9 to 268.1)	366(343 to 392.9)	163(153 to 174.9)	-1.63(-1.7 to -1.57)	53.9(49.5 to 58.7)	60.9(56 to 65.8)	73(66.7 to 79.5)	31.7(29.1 to 34.4)	-2.47(-2.66 to -2.28)	21.9(15.9 to 28.3)	22.4(16.4 to 28.8)	32.8(24.2 to 42.2)	14.7(10.8 to 18.9)	-1.62(-1.69 to -1.55)
Saint Vincent and the Grenadines	ICH	166.6(155.9 to 177.9)	208.3(195.8 to 221.9)	221.6(208.4 to 237.2)	163.8(154.1 to 175.1)	-1(-1.1 to -0.9)	30.5(27.7 to 33)	41.1(37.6 to 44.6)	48.5(44.7 to 52.8)	35.7(32.8 to 38.7)	-0.73(-0.84 to -0.61)	14.9(10.6 to 19.4)	18.7(13.5 to 24.1)	19.5(14.3 to 25.5)	14.5(10.6 to 18.9)	-1.06(-1.15 to -0.96)
Samoa	ICH	449.7(420.6 to 480.8)	425.8(395.9 to 456.2)	627.4(591.2 to 666.7)	368.3(345.9 to 391.7)	-0.51(-0.59 to -0.43)	93.9(84.9 to 103.3)	110(99.3 to 120.3)	121(110.1 to 132)	82.1(74.7 to 89.6)	-1.07(-1.12 to -1.02)	83(58.4 to 107.9)	79.3(56 to 102.5)	115.5(83 to 147.5)	68.4(49.1 to 87.2)	-0.52(-0.59 to -0.44)
San Marino	ICH	40.8(37.8 to 43.9)	128.2(118.5 to 138.1)	61.7(57.1 to 66.2)	104.8(97.1 to 112.8)	-0.83(-0.88 to -0.78)	6.4(5.6 to 7.3)	18.6(16.2 to 21)	9.4(8.2 to 10.5)	12.3(10.7 to 13.7)	-1.59(-1.69 to -1.49)	5.9(4.2 to 7.7)	18.2(13.2 to 23.7)	9.3(6.5 to 11.9)	14.9(10.5 to 19.2)	-0.82(-0.87 to -0.76)
Sao Tome and Principe	ICH	341(320.3 to 365.7)	426.8(399 to 459.4)	574.7(544.4 to 607.8)	360.2(339.4 to 384.1)	-0.69(-0.81 to -0.58)	58(52.3 to 63.9)	86(76.7 to 94.4)	87.4(79.1 to 96)	72.1(65.1 to 78.7)	-0.74(-0.85 to -0.63)	44.8(32.4 to 57.9)	57.2(41.7 to 73.1)	74.8(54.6 to 95.1)	48.4(35.1 to 61.2)	-0.68(-0.79 to -0.57)
Saudi Arabia	ICH	18183.1(16889.1 to 19644.5)	179.4(166.8 to 193.7)	54407.8(50851 to 58235.7)	146.1(137 to 157.1)	-0.82(-0.92 to -0.73)	3308.3(2975.1 to 3641.3)	49.8(44.5 to 54.3)	7380.7(6725.1 to 8116.5)	30.1(27.6 to 32.5)	-1.8(-1.96 to -1.64)	2512.3(1795.4 to 3240.8)	25.2(18 to 32.5)	7324.2(5198.3 to 9613.2)	20.2(14.4 to 26.2)	-0.87(-0.96 to -0.78)
Senegal	ICH	16824.7(15809.2 to 17999.8)	365.3(342.2 to 394.6)	31481.4(29687.2 to 33368.9)	291(272.1 to 310.5)	-0.87(-0.92 to -0.83)	2778(2492.2 to 3076.9)	77.6(69.1 to 85.8)	5076.8(4610.1 to 5592.9)	61.9(55.7 to 68.2)	-0.75(-0.78 to -0.73)	2189(1584 to 2799.9)	49.1(35.5 to 63.1)	4147.2(3019.3 to 5335.9)	39.8(29 to 51.3)	-0.81(-0.85 to -0.77)
Serbia	ICH	21405.1(19982.1 to 22944.5)	189.9(177.8 to 203.1)	11698.6(10745.6 to 12686.2)	99.1(91.2 to 106.8)	-2.51(-2.69 to -2.33)	6136.2(5582.2 to 6656.9)	61.2(56.4 to 66)	4916.9(4522 to 5296.2)	31(28.6 to 33.3)	-2.77(-2.97 to -2.56)	3826.3(2659.9 to 4970.9)	34.2(23.8 to 44.8)	2166.2(1527.2 to 2825.6)	18.1(12.8 to 23.7)	-2.46(-2.64 to -2.29)
Seychelles	ICH	174.9(162.9 to 187.5)	295.9(275.5 to 317.1)	267.2(249.7 to 284)	214.6(200.9 to 228.4)	-1.2(-1.25 to -1.15)	36.1(32.5 to 39.4)	62.9(56.3 to 68.5)	45.6(41 to 49.9)	40.3(36.2 to 43.9)	-1.56(-1.67 to -1.46)	32.2(22.7 to 41.4)	54.3(37.9 to 69.8)	48.4(34.4 to 62.8)	39.2(27.8 to 51)	-1.19(-1.24 to -1.14)
Sierra Leone	ICH	10628.7(10043.4 to 11375.6)	381.4(357.6 to 410.7)	19082.4(18067.8 to 20208.9)	322.2(302.2 to 344.5)	-0.62(-0.69 to -0.56)	1878.4(1696.6 to 2062.7)	82.7(74.2 to 91.3)	3049.4(2762 to 3326.5)	70.7(64.1 to 76.9)	-0.51(-0.6 to -0.42)	1380.6(1014 to 1758.6)	50.8(37 to 64.5)	2482.5(1770.1 to 3169.5)	43.7(31.4 to 55.6)	-0.56(-0.62 to -0.49)
Singapore	ICH	10720.6(10118.4 to 11326.3)	428.5(403.2 to 454.5)	17050.9(16166.4 to 17986.5)	210.3(199.6 to 221.7)	-2.76(-2.93 to -2.59)	1184.1(1058.3 to 1324.1)	49(43.9 to 54.7)	1396.1(1216.2 to 1571.6)	17.2(15.1 to 19.2)	-3.84(-4.08 to -3.6)	1712.3(1229.1 to 2209.8)	69.7(49.8 to 89.5)	2804.1(2047.6 to 3594.2)	34.8(25.5 to 44.6)	-2.71(-2.88 to -2.54)
Slovakia	ICH	7855.2(7270.1 to 8441.4)	139.3(129.4 to 149.2)	6394.5(5883.2 to 6911)	88.6(81.7 to 95.7)	-1.65(-1.71 to -1.58)	2141.6(1938.9 to 2327.8)	37.1(33.7 to 40.2)	1535.8(1393.6 to 1684.2)	17.6(15.9 to 19.2)	-2.5(-2.63 to -2.37)	1430.4(1021.8 to 1850.3)	25.3(18 to 32.8)	1183.6(840.8 to 1545.1)	16.3(11.5 to 21.5)	-1.57(-1.64 to -1.51)
Slovenia	ICH	2905.2(2692.7 to 3143)	127.7(118.3 to 138)	2057.6(1891.4 to 2250.3)	69.8(63.5 to 76.7)	-2.28(-2.4 to -2.17)	690(615.4 to 757.8)	29.5(26.4 to 32.5)	527.8(475.4 to 582.1)	12.2(11.1 to 13.4)	-3.22(-3.44 to -3.01)	515.4(363.1 to 656.3)	22.7(16 to 28.8)	388.5(271.3 to 510.4)	12.9(9 to 16.8)	-2.13(-2.25 to -2.02)
Solomon Islands	ICH	1212.1(1132.7 to 1295.6)	657.2(608.2 to 707.4)	2764.1(2624.9 to 2931.8)	581.6(548.4 to 620.7)	-0.41(-0.5 to -0.32)	299.9(269.1 to 328)	219.4(199 to 238.1)	714(664 to 762.6)	198.1(184.5 to 211.6)	-0.39(-0.44 to -0.34)	221.9(158.5 to 284.7)	121.8(86.3 to 156.5)	505.9(351.8 to 644.2)	108(75.2 to 137.3)	-0.4(-0.48 to -0.31)
Somalia	ICH	14000.6(13063.1 to 14914.8)	327.8(300.6 to 355.6)	24780.8(23195.2 to 26475.2)	220.6(204 to 239.3)	-1.46(-1.53 to -1.39)	3247.5(2885.7 to 3610.5)	112.1(100.4 to 123.1)	5562(4998.5 to 6079.6)	76(68.7 to 82.2)	-1.49(-1.58 to -1.4)	1799.3(1313.7 to 2340.6)	44.7(31.8 to 58.5)	3251.1(2298.1 to 4233)	31.3(22 to 40.7)	-1.31(-1.37 to -1.25)
South Africa	ICH	67173.7(59464.6 to 75798.5)	261.4(231.2 to 296.2)	79659(71380.2 to 89613.5)	151.5(135.5 to 170.3)	-2.27(-2.52 to -2.03)	12027.4(10193.9 to 13799.4)	52.8(45 to 60.3)	15401(13262.4 to 17627.1)	34.3(29.7 to 38.8)	-1.84(-2.19 to -1.5)	9081.1(6411.5 to 11662.9)	36.2(25.4 to 46.4)	11038.5(7884.7 to 14411.5)	21.7(15.5 to 28.3)	-2.12(-2.34 to -1.9)
South Sudan	ICH	9571.9(8929.4 to 10289.9)	285.5(264.2 to 308.5)	11057.7(10357.4 to 11883)	186.4(172.8 to 201.2)	-1.53(-1.58 to -1.48)	2275.1(2034 to 2521.9)	84.8(75.8 to 93.1)	2283.7(2046.5 to 2547.5)	57.2(51.6 to 62.9)	-1.44(-1.53 to -1.35)	1255(897.6 to 1599.1)	38.5(27.6 to 49.6)	1459.5(1047.5 to 1901.6)	26.2(18.5 to 34.2)	-1.39(-1.43 to -1.35)
Spain	ICH	85020.5(79729.6 to 90669.8)	178.4(167.7 to 190.1)	70053.5(65282.6 to 75231.1)	92.5(85 to 100.1)	-2.59(-2.75 to -2.44)	14457.5(12828.9 to 16016.3)	28.5(25.4 to 31.2)	13023.5(11596.9 to 14240.6)	12.6(11.3 to 14)	-3.13(-3.34 to -2.92)	11642.2(8240.4 to 14808.8)	24.1(17 to 30.8)	10512.2(7539.7 to 13336.5)	13.1(9.3 to 16.9)	-2.41(-2.55 to -2.26)
Sri Lanka	ICH	30044.6(28080.9 to 32022.9)	214.8(200 to 230)	38548.7(35754.2 to 41501.3)	149.7(139.4 to 160.6)	-1.37(-1.46 to -1.27)	4841.9(4300.4 to 5423.5)	45.1(39.8 to 50.3)	6494.6(5719.8 to 7210.7)	26.5(23.5 to 29.1)	-1.93(-2.07 to -1.78)	5598.5(3956 to 7250.4)	40.5(28.5 to 52.5)	7377.6(5231.4 to 9475.9)	28.7(20.4 to 36.9)	-1.29(-1.38 to -1.2)
Sudan	ICH	32577.3(30605.5 to 34753.1)	246.7(231.2 to 265)	47126.2(44116.3 to 50529.3)	146.7(137.2 to 158)	-2.02(-2.14 to -1.89)	6489(5851.3 to 7155.5)	62(55.7 to 68)	7238.9(6482.3 to 7972.8)	31.5(28.4 to 34.5)	-2.65(-2.82 to -2.47)	4509.3(3207 to 5773.1)	34.5(24.5 to 43.9)	6515.9(4698.9 to 8546.9)	20.5(14.9 to 26.6)	-2.01(-2.13 to -1.89)
Suriname	ICH	839.7(793.5 to 891)	271.2(255.3 to 288)	1304.1(1230.1 to 1392.2)	202.4(190.6 to 216.2)	-1.12(-1.21 to -1.03)	163.1(149.1 to 174.7)	60.5(55.4 to 65)	301.9(276.8 to 327.1)	48.4(44.4 to 52.4)	-1(-1.16 to -0.84)	72.7(52.4 to 93.4)	24(17.4 to 30.9)	116(84.7 to 147.8)	18.3(13.3 to 23.3)	-1.06(-1.15 to -0.97)
Sweden	ICH	20509.4(17955.7 to 23440.7)	157.5(140 to 176.2)	25397.9(22242.1 to 28496.5)	146.6(131.3 to 163.1)	-0.22(-0.27 to -0.16)	2944(2429.7 to 3493)	19.9(16.6 to 23.3)	2873.3(2391.6 to 3351.4)	13.8(11.7 to 15.9)	-1.42(-1.51 to -1.33)	2973.5(2122.1 to 3844.5)	22.1(15.6 to 28.6)	3740.9(2727.6 to 4831)	20.5(14.6 to 26.6)	-0.25(-0.3 to -0.19)
Switzerland	ICH	11169.5(10364.6 to 11950.3)	120.5(111.5 to 129.3)	13430.8(12576.8 to 14374)	89.3(82.3 to 96.4)	-0.96(-1.16 to -0.77)	1877.1(1689.8 to 2040.6)	18.2(16.4 to 19.9)	1787.7(1563.5 to 2010)	9.9(8.6 to 11.2)	-2.09(-2.22 to -1.96)	1540.7(1115.5 to 1968.7)	16.3(11.7 to 21)	1969.3(1396.3 to 2561.5)	12.5(8.8 to 16.4)	-0.82(-1.01 to -0.63)
Syrian Arab Republic	ICH	28598.9(26881 to 30307.1)	341.1(320.8 to 363.8)	28511(26892.1 to 30199.6)	194.8(184.1 to 207.1)	-2.11(-2.21 to -2.02)	4327.5(3960.8 to 4698.8)	65.2(59.6 to 70)	4653.4(4240.4 to 5064.2)	39.8(36.4 to 42.8)	-1.96(-2.13 to -1.8)	3908.5(2817 to 5003.4)	46.8(33.5 to 59.6)	3898.9(2774.3 to 5044.7)	27.1(19.2 to 35.4)	-2.07(-2.17 to -1.98)
Taiwan (Province of China)	ICH	64470.5(60072.9 to 69287.7)	346.5(322.5 to 374)	73211.1(67738.4 to 78830.7)	216.1(201.8 to 231)	-1.86(-1.96 to -1.75)	12324(11152.4 to 13387.5)	84.6(75.8 to 92)	13756.1(12119.3 to 15396.7)	35.1(31.1 to 39.5)	-3.25(-3.43 to -3.06)	12369.9(8800.1 to 16015.1)	67.2(47.7 to 86.8)	14276.4(10028 to 18556.9)	41.8(29.2 to 54.3)	-1.88(-1.99 to -1.78)
Tajikistan	ICH	7726.8(7166.6 to 8300.8)	232.8(216 to 250.7)	12341.4(11406.9 to 13257)	160.6(148.4 to 173.5)	-1.48(-1.56 to -1.41)	2217.1(2014.3 to 2398.8)	79.8(72.3 to 86.5)	4012.1(3648.4 to 4382.7)	79.9(73.2 to 87.2)	-0.02(-0.12 to 0.07)	1404.1(992 to 1796.4)	42.5(29.7 to 54.7)	2240.7(1554.3 to 2946.9)	29.8(20.8 to 38.8)	-1.39(-1.46 to -1.33)
Thailand	ICH	135140.7(126576.4 to 143817.2)	289.8(270.9 to 311.1)	197961.6(183713 to 210724.2)	205.8(192.3 to 218.4)	-1.29(-1.38 to -1.2)	24474.2(21889.8 to 26962.1)	68.4(61.2 to 75)	36765.4(32600 to 40278.3)	36.8(32.7 to 40.2)	-2.39(-2.54 to -2.23)	25206.5(17661.1 to 31890.9)	55.1(38.3 to 70.5)	38118.9(26759.1 to 49197.4)	39.5(27.7 to 50.8)	-1.25(-1.34 to -1.16)
Timor-Leste	ICH	1191.7(1108.1 to 1273.9)	255.4(236.6 to 275.6)	2354(2196.8 to 2520.3)	232.3(216 to 250)	-0.36(-0.38 to -0.35)	239.2(210.5 to 267.1)	79.6(70.9 to 87.2)	626(570.5 to 687.9)	74.1(67.5 to 80.7)	-0.33(-0.37 to -0.29)	218.5(152.3 to 282.6)	47.6(33.3 to 61.6)	437.8(314.4 to 559.5)	43.4(31.1 to 55.8)	-0.36(-0.37 to -0.34)
Togo	ICH	7560.6(7141.1 to 8080.1)	367.4(340.7 to 395.8)	16999.5(16055.1 to 18109.1)	290.4(271.6 to 311.7)	-0.92(-1 to -0.84)	1272(1152.1 to 1405.3)	87.8(78.9 to 96)	2947.3(2663.3 to 3247)	71.9(64.9 to 78.9)	-0.72(-0.81 to -0.64)	975.9(700.9 to 1248.1)	49.5(35.4 to 63)	2221.7(1610.4 to 2864.7)	39.9(28.9 to 51.4)	-0.84(-0.92 to -0.77)
Tokelau	ICH	4.9(4.5 to 5.3)	374.9(347.2 to 403)	3.9(3.7 to 4.2)	275.6(259 to 293.7)	-1.2(-1.28 to -1.12)	1.3(1.1 to 1.4)	101.5(90.2 to 111)	0.9(0.8 to 0.9)	58.8(52.6 to 64.5)	-2.07(-2.18 to -1.95)	0.9(0.7 to 1.2)	71.3(50.3 to 91.3)	0.7(0.5 to 0.9)	52(36.9 to 66.2)	-1.23(-1.31 to -1.14)
Tonga	ICH	172.6(160.9 to 185.6)	258.2(239.7 to 277.7)	190.9(178.7 to 203.3)	217.3(202.9 to 231.6)	-0.68(-0.73 to -0.62)	32.3(28.8 to 36)	60.1(52.9 to 66.4)	37.9(34.1 to 42)	47(42 to 52.1)	-0.98(-1.05 to -0.92)	32.7(23.1 to 42.5)	49.5(34.9 to 64.1)	36.1(25.8 to 46.2)	41.4(29.5 to 52.9)	-0.69(-0.74 to -0.64)
Trinidad and Tobago	ICH	2041.2(1904.8 to 2200.5)	208.9(195.5 to 224.8)	2424.4(2264.8 to 2606.3)	141.2(132.1 to 151)	-1.52(-1.61 to -1.44)	397.4(364.3 to 434.8)	46.3(42.6 to 50.4)	443.1(398.7 to 490.4)	24.5(22.1 to 27)	-2.41(-2.55 to -2.26)	178.7(131 to 231.6)	18.6(13.6 to 24)	220.7(162.3 to 287.3)	12.8(9.5 to 16.6)	-1.44(-1.52 to -1.36)
Tunisia	ICH	10133.3(9427.1 to 10914.5)	156.8(145.7 to 168.9)	15938.6(14831.4 to 17091.7)	120.7(112.5 to 129.6)	-1.12(-1.21 to -1.03)	1748.2(1554.6 to 1955.7)	35.1(31.2 to 38.7)	2696.4(2377.4 to 3014.2)	21.7(19.2 to 24.1)	-1.91(-2.06 to -1.76)	1429.4(1036.3 to 1844.3)	22.4(16.2 to 28.8)	2229.5(1598.9 to 2890.1)	17(12.2 to 22.1)	-1.13(-1.22 to -1.05)
Turkmenistan	ICH	125764(117970.7 to 133512)	273.6(255.5 to 290.8)	146139.3(136157.6 to 155837.9)	156.8(146.3 to 167.5)	-2.18(-2.3 to -2.06)	18698.6(16823.7 to 20630.1)	50.2(45.1 to 55.2)	22173.2(19642 to 24612.1)	25.1(22.2 to 27.8)	-2.58(-2.74 to -2.42)	17503.8(12642.8 to 22473.3)	38.6(27.8 to 49.6)	20888(15351.6 to 26939.5)	22.6(16.6 to 29.1)	-2.1(-2.21 to -1.98)
Tuvalu	ICH	5576(5199.9 to 5935.2)	229.8(213.8 to 244.3)	11575(10878.6 to 12334.1)	233.5(218.8 to 249.6)	-0.03(-0.17 to 0.1)	1439.9(1318 to 1565.2)	73.2(67.2 to 79.4)	2774.6(2551.5 to 2994.9)	66.1(60.7 to 71.6)	-0.25(-0.36 to -0.14)	993.3(695.8 to 1272.9)	41.1(29 to 52.9)	2039(1433.9 to 2672.3)	41.6(29.2 to 54.4)	-0.03(-0.15 to 0.1)
T 眉 rkiye	ICH	35.8(33.3 to 38.5)	461.6(429.8 to 496.1)	41(38.6 to 43.7)	358.9(337.7 to 383.1)	-0.95(-1.03 to -0.86)	8.6(7.7 to 9.4)	134.7(122.1 to 145.7)	9.2(8.4 to 9.9)	91.2(84 to 98.8)	-1.48(-1.56 to -1.4)	6.7(4.6 to 8.6)	86.8(60.4 to 111.2)	7.6(5.3 to 9.7)	66.7(47 to 85.2)	-0.97(-1.05 to -0.89)
Uganda	ICH	23605.6(21958 to 25403.8)	257.1(238.1 to 278.7)	45269.8(42181.7 to 48592.3)	190.1(176.5 to 204)	-1.24(-1.39 to -1.09)	5568.8(5024.2 to 6184.1)	81.5(73.5 to 89.7)	9466.4(8559.9 to 10392.6)	58.4(52.8 to 63.4)	-1.43(-1.59 to -1.26)	3122.2(2257.7 to 4013.5)	35.4(25.2 to 46.3)	5985(4411 to 7799.1)	26.7(19.5 to 35.3)	-1.16(-1.29 to -1.02)
Ukraine	ICH	98331.5(86081.2 to 110586.4)	151.9(134.3 to 169.8)	83782.2(73586.8 to 94693.3)	138.5(123.5 to 156.2)	-0.34(-0.37 to -0.3)	27907.7(22975.7 to 32441.8)	41.7(34.9 to 48.2)	21148(17998.1 to 24397)	31.1(26.7 to 35.8)	-1.3(-1.46 to -1.14)	17635.2(12616.8 to 22713.6)	27.3(19.5 to 35.1)	14839.5(10162.5 to 19889.6)	24.5(16.8 to 32.2)	-0.37(-0.4 to -0.33)

United Arab Emirates	ICH	3244.9(3005.2 to 3490.1)	241.7(225.4 to 259.3)	17495.8(16419.7 to 18676.4)	162.9(152 to 174.4)	-1.52(-1.59 to -1.44)	359.7(315.3 to 408.8)	50.6(45.3 to 55.5)	1826.4(1558.9 to 2116.3)	29(25.9 to 32.1)	-2.18(-2.37 to -2)	431.8(310.3 to 575.3)	32.9(23.7 to 43.1)	2262.2(1611.3 to 2993.1)	22.2(15.9 to 28.5)	-1.51(-1.59 to -1.44)
United Kingdom	ICH	94977.5(85628.4 to 105404.2)	125.3(113.8 to 138.2)	96392.5(87317.2 to 106281.7)	93.5(85.4 to 102.3)	-1.14(-1.21 to -1.06)	15737.1(13293.2 to 18119.9)	18.4(15.7 to 21)	16452.6(14114.2 to 18774.1)	12.7(11.1 to 14.3)	-1.47(-1.56 to -1.38)	13202(9434.8 to 17143.5)	17.1(12.1 to 21.9)	14015.2(10091.9 to 17940.3)	13.1(9.4 to 16.8)	-1.05(-1.12 to -0.98)
United Republic of Tanzania	ICH	39473.4(36767.9 to 42506.9)	263.7(244.4 to 286)	74982.5(70638.6 to 80009.2)	200.6(187.5 to 215.8)	-1.04(-1.08 to -1)	9064.1(8236.1 to 9956.2)	78.7(71.1 to 86.9)	15215.2(13875.4 to 16727.6)	55.6(50.4 to 60.7)	-1.23(-1.32 to -1.14)	5219.5(3811.1 to 6657.9)	36.2(26.3 to 46.4)	10005(7167.9 to 12814.9)	28.1(19.9 to 35.8)	-0.93(-0.95 to -0.9)
United States of America	ICH	422557.3(376060 to 472546.5)	145.5(129.6 to 162.3)	753476.6(675190.6 to 838583.4)	154.7(140.1 to 169.9)	0.54(0.24 to 0.83)	55214.4(45721.5 to 64379.8)	17.8(14.7 to 20.7)	70680.4(60481.5 to 80920.6)	13(11.2 to 14.8)	-0.92(-1.05 to -0.78)	60272(42336.2 to 78615.8)	20.5(14.4 to 26.6)	106794.3(77800.8 to 136938.6)	21.7(15.7 to 28.1)	0.49(0.19 to 0.79)
United States Virgin Islands	ICH	158.4(146.9 to 169.9)	158.3(147.3 to 170.4)	167.3(152.8 to 182.2)	130.6(122.2 to 141.2)	-0.71(-0.89 to -0.54)	26.3(23.3 to 29.2)	30.4(26.9 to 33.5)	39.8(35.6 to 44.1)	24.4(22 to 26.8)	-0.75(-0.82 to -0.67)	13.8(9.8 to 17.9)	14.3(10.2 to 18.5)	15.7(11.3 to 20.6)	11.8(8.5 to 15.5)	-0.7(-0.87 to -0.54)
Uruguay	ICH	9808(9167.1 to 10502.5)	276.9(259.5 to 295.2)	7979.1(7412.5 to 8531.8)	174.1(163.1 to 185.4)	-1.86(-1.98 to -1.74)	1445.4(1292.1 to 1597.7)	39.5(35.6 to 43.7)	1169.8(1028.4 to 1291.8)	22.2(19.7 to 24.5)	-2.28(-2.51 to -2.05)	1403.8(987.9 to 1782.6)	39.4(27.8 to 50.1)	1177.2(835.7 to 1494.7)	25(17.9 to 31.8)	-1.81(-1.93 to -1.7)
Uzbekistan	ICH	32146(29788 to 34467.7)	236.7(219.3 to 253.5)	63469.3(59236.2 to 67920.4)	199.3(185.1 to 213.8)	-0.7(-0.9 to -0.5)	9914.3(9131.2 to 10703.3)	85.2(78.6 to 92.2)	20662.8(18954.1 to 22275.3)	91.4(84 to 99)	0.11(-0.33 to 0.54)	5820.3(4172.8 to 7439.1)	43(30.6 to 54.9)	11272.8(7960 to 14623.4)	36.1(25.4 to 46.7)	-0.7(-0.91 to -0.5)
Vanuatu	ICH	419.3(390.4 to 447.1)	472.8(437 to 508.8)	1040.4(987.5 to 1100.3)	440.8(415.4 to 469.2)	-0.22(-0.33 to -0.12)	81.6(72.4 to 90.6)	123.8(111.2 to 135.2)	201.4(184.7 to 218.2)	109.2(100.4 to 118.1)	-0.45(-0.49 to -0.41)	76.2(54.5 to 97.5)	86.9(61.5 to 111.2)	189.6(134.7 to 241.3)	81.3(57.9 to 104.4)	-0.22(-0.32 to -0.12)
Venezuela (Bolivarian Republic of)	ICH	29856.2(27811.7 to 32066.8)	229.6(214.7 to 246.1)	49151.5(46161.7 to 52697.9)	165.3(155.4 to 177.3)	-1.18(-1.23 to -1.14)	4782.5(4340.3 to 5278.8)	44.5(40.1 to 48.7)	8666.7(7808.3 to 9553.6)	29.8(26.8 to 32.7)	-1.47(-1.57 to -1.36)	2582(1853.5 to 3358.8)	20.4(14.6 to 26.4)	4397.2(3176 to 5679.5)	14.9(10.8 to 19.3)	-1.12(-1.15 to -1.08)
Viet Nam	ICH	143766.4(135330.4 to 153891.4)	308.4(289.7 to 331)	279214.2(262128.4 to 296736.7)	252.6(236.9 to 269.5)	-0.71(-0.76 to -0.66)	40696.6(36756.5 to 44343.3)	102.3(92.7 to 111.3)	74924.8(68600 to 80667.2)	76.8(70.7 to 82.3)	-1.02(-1.1 to -0.94)	27229.4(19503.2 to 34896.4)	58.7(41.6 to 74.9)	52772.3(37774.3 to 68073.8)	48.4(34.7 to 62.5)	-0.7(-0.75 to -0.64)
Yemen	ICH	17403.3(16243.2 to 18657.7)	225.4(210.3 to 242.2)	32859.2(30597.9 to 35250.9)	140.3(130.5 to 150.9)	-1.76(-1.85 to -1.67)	3604.7(3224.8 to 3990.6)	64.3(57.4 to 70.4)	5915.4(5356.6 to 6478)	36.4(33.2 to 39.5)	-2.24(-2.39 to -2.09)	2399.7(1711.6 to 3167.6)	31.6(22.9 to 41.1)	4582.8(3229.9 to 5983.4)	19.9(14 to 25.8)	-1.72(-1.8 to -1.64)
Zambia	ICH	12916.9(12071.2 to 13832.3)	308.6(286.3 to 332.7)	25832.8(24129.6 to 27523.3)	235.6(217.7 to 254.3)	-1.04(-1.12 to -0.95)	3012.6(2711.2 to 3278.9)	98.9(89.2 to 107.5)	6018.9(5534.2 to 6545.1)	81.3(74.6 to 88.2)	-0.83(-0.91 to -0.74)	1692(1223.9 to 2141.1)	42.2(30.2 to 54.2)	3387.4(2446.1 to 4408.3)	32.8(23.7 to 42.6)	-0.96(-1.03 to -0.88)
Zimbabwe	ICH	7134.3(6479.1 to 7810.6)	123.7(112.9 to 135.3)	12399.9(11493.2 to 13525.8)	123.9(113.9 to 135.6)	0.04(-0.12 to 0.2)	1411.9(1262 to 1580)	32.3(28.9 to 35.3)	2443.1(2197.6 to 2702.4)	33.1(30 to 36.1)	0.27(0.07 to 0.46)	1015.4(709.6 to 1328.2)	18.9(12.9 to 24.6)	1742.3(1205 to 2325.7)	18.9(13 to 25.4)	0.04(-0.09 to 0.17)
Afghanistan	SAH	7822.5(7159.7 to 8493.2)	100.8(92.1 to 109.5)	15607.8(14270.3 to 17128.5)	83.2(76.4 to 89.9)	-0.72(-0.76 to -0.68)	775.3(648.1 to 916.1)	10.4(8.5 to 12.5)	1534.7(1346.3 to 1740.1)	8.9(7.9 to 10.1)	-0.96(-1.14 to -0.79)	1080.7(765.3 to 1402.3)	14.1(10.1 to 18.2)	2152.3(1505.7 to 2773.9)	11.5(8.2 to 15)	-0.73(-0.77 to -0.7)
Albania	SAH	2000.4(1831.6 to 2182.2)	74.6(68.6 to 80.8)	2369(2190.1 to 2571.3)	67.4(62.3 to 73.3)	-0.38(-0.39 to -0.36)	179.9(153.6 to 205)	7.2(6.3 to 8.2)	242(214 to 270.7)	6.7(5.9 to 7.6)	-0.24(-0.31 to -0.17)	360.9(255.8 to 479.6)	13.5(9.6 to 17.9)	429.1(300.9 to 556.3)	12.2(8.5 to 15.9)	-0.34(-0.36 to -0.32)
Algeria	SAH	17138.2(15830.2 to 18573.7)	100.5(92.9 to 108)	32670.1(30268.7 to 35363.6)	77.5(71.8 to 83.7)	-0.96(-1 to -0.91)	1550.7(1340.7 to 1748.9)	9.4(8.2 to 10.7)	2541.1(2187.2 to 2873.7)	6.3(5.5 to 7.1)	-1.65(-1.76 to -1.54)	2411.7(1694 to 3143.4)	14.2(9.9 to 18.6)	4613.3(3250.8 to 6030.1)	11(7.8 to 14.4)	-0.92(-0.96 to -0.88)
American Samoa	SAH	53.2(49.9 to 56.7)	159.8(149.6 to 169.4)	77.5(73.5 to 81.6)	151.6(143.5 to 159.8)	-0.2(-0.22 to -0.19)	4.3(3.8 to 4.8)	12.8(11.4 to 14.3)	5.5(4.9 to 6.1)	11.6(10.4 to 12.8)	-0.38(-0.41 to -0.35)	9.8(6.8 to 12.7)	29.6(20.7 to 38.1)	14(10 to 18.2)	27.7(19.8 to 35.8)	-0.23(-0.24 to -0.22)
Andorra	SAH	56(52.2 to 60.5)	93(86.7 to 100.3)	114.4(106.9 to 122.1)	84.6(78.8 to 90.3)	-0.34(-0.37 to -0.32)	4.2(3.5 to 4.9)	7(6 to 8.1)	8(6.9 to 9.3)	6.1(5.3 to 7)	-0.52(-0.58 to -0.46)	7.4(5.4 to 9.6)	12.4(9 to 16)	15.4(10.8 to 20)	11.4(8 to 14.7)	-0.32(-0.36 to -0.29)
Angola	SAH	5057.1(4599.2 to 5528.9)	95.7(88.1 to 103.6)	14324.6(13146.1 to 15604)	85.4(79 to 91.4)	-0.4(-0.42 to -0.38)	457.3(387 to 537.5)	7.7(6.6 to 9.2)	1209(1026.8 to 1391.5)	6.3(5.5 to 7.4)	-0.84(-0.91 to -0.77)	654.3(460.5 to 844.2)	12.7(9.1 to 16.4)	1878.3(1359.3 to 2414.3)	11.5(8.5 to 14.6)	-0.35(-0.38 to -0.33)
Antigua and Barbuda	SAH	86.2(80.8 to 91.4)	159.8(149.8 to 169.4)	147.8(138.6 to 156)	139.6(131 to 147.4)	-0.5(-0.53 to -0.48)	6.8(6 to 7.6)	12.5(11.1 to 14.2)	10.6(9.3 to 11.8)	10.3(9.2 to 11.5)	-0.74(-0.81 to -0.67)	7.8(5.8 to 10.1)	14.2(10.7 to 18.4)	12.9(9.5 to 16.6)	12.3(9.1 to 15.8)	-0.52(-0.54 to -0.5)
Argentina	SAH	53925.2(50370.1 to 57982.9)	166.4(155.6 to 178.6)	65231.5(60047.7 to 72359.2)	125.3(115.7 to 137.9)	-1.03(-1.08 to -0.99)	5635.3(4996 to 6461.6)	17.6(15.6 to 20.2)	5583.4(4971.8 to 6303.7)	10.9(9.7 to 12.2)	-1.85(-2 to -1.71)	7539.8(5516.2 to 9845.6)	23.3(17 to 30.4)	9170(6664.2 to 11680.1)	17.5(12.7 to 22.4)	-1.01(-1.05 to -0.97)
Armenia	SAH	2647.2(2455.3 to 2845.3)	84.9(78.7 to 91.1)	2678(2491.4 to 2865.1)	71.1(66 to 76.2)	-0.65(-0.68 to -0.62)	254.4(218.3 to 286.2)	8.6(7.5 to 9.6)	225.9(200.6 to 253)	6.2(5.5 to 6.9)	-1.3(-1.44 to -1.17)	476.5(329.3 to 631.3)	15.3(10.6 to 20.2)	484.5(336.1 to 630.3)	12.8(9 to 16.8)	-0.64(-0.67 to -0.61)
Australia	SAH	16335.2(15201.4 to 17575.4)	86.8(80.8 to 93.6)	30089.4(28167.7 to 32117)	81.2(75.8 to 87)	-0.17(-0.21 to -0.14)	1350.6(1213.6 to 1511.3)	7.3(6.6 to 8.2)	2226.4(1969.2 to 2538.9)	6.1(5.4 to 6.9)	-0.63(-0.71 to -0.55)	2352.6(1642.5 to 3054.6)	12.5(8.7 to 16.2)	4460.7(3229.9 to 5773.5)	11.8(8.6 to 15.5)	-0.13(-0.17 to -0.08)
Austria	SAH	18415.3(16874.5 to 19552.6)	172(158.3 to 182.5)	23045.1(21681.7 to 24171.2)	150.2(141.1 to 158.4)	-0.42(-0.55 to -0.3)	1246(1106.7 to 1392.3)	12.2(10.9 to 13.7)	1239.6(1072.2 to 1429)	8.9(7.6 to 10.2)	-0.76(-0.98 to -0.54)	2509.9(1812.3 to 3240.5)	23.1(16.7 to 29.9)	3237.6(2333.2 to 4063.5)	20.5(14.8 to 25.9)	-0.37(-0.5 to -0.25)

Azerbaijan	SAH	4518.6(4106.8 to 4947)	75.4(68.9 to 82.6)	7885.3(7284.5 to 8524.6)	67.1(62 to 72.6)	-0.4(-0.42 to -0.38)	430.4(349.3 to 510.1)	7.3(5.9 to 8.8)	621.8(525 to 725.8)	5.8(5 to 6.7)	-0.65(-0.77 to -0.53)	816.4(565.3 to 1074.8)	13.6(9.6 to 18)	1410.9(963.8 to 1846)	12.1(8.3 to 15.8)	-0.39(-0.42 to -0.37)
Bahamas	SAH	258.2(240.5 to 275.1)	130.1(121.5 to 138.4)	521.7(491.8 to 552)	122.4(115.4 to 129.7)	-0.23(-0.24 to -0.21)	20.2(17.7 to 22.7)	9.9(8.8 to 11.2)	37.4(33 to 42)	9(8 to 10)	-0.37(-0.42 to -0.31)	22.6(16.6 to 29.5)	11.6(8.6 to 15.1)	45.4(33.7 to 59.2)	10.8(8 to 14.1)	-0.26(-0.28 to -0.24)
Bahrain	SAH	299.6(272.7 to 330.2)	74.9(68.5 to 81.5)	955.4(869.4 to 1049.2)	60(54.8 to 65.3)	-0.83(-0.87 to -0.78)	23.4(19.6 to 27.2)	6.4(5.5 to 7.3)	61.6(50.7 to 72.6)	4.5(3.9 to 5)	-1.41(-1.51 to -1.3)	42.2(29.2 to 55)	10.6(7.5 to 13.6)	132.8(92.1 to 174.2)	8.5(6 to 11)	-0.82(-0.86 to -0.78)
Bangladesh	SAH	85093.8(78077.4 to 92587.6)	135.3(124.7 to 146.4)	189240.8(176671.4 to 203827.2)	122.5(114.2 to 132.3)	-0.25(-0.29 to -0.2)	9036.5(7705.2 to 10427.9)	14.3(12.2 to 16.7)	19095.7(16867.1 to 21923.9)	12.7(11.3 to 14.6)	-0.49(-0.57 to -0.42)	11691.5(8300.2 to 15062.1)	18.7(13.1 to 24)	26195.8(18776.2 to 33988.1)	17.1(12.2 to 22.2)	-0.21(-0.25 to -0.17)
Barbados	SAH	404.5(379.3 to 430.2)	152.6(143.1 to 162.4)	604.3(570.7 to 640.3)	141(133 to 149)	-0.29(-0.32 to -0.26)	31.8(28.3 to 35.6)	12.3(10.9 to 13.8)	41.4(37.2 to 47.2)	10.2(9.2 to 11.5)	-0.55(-0.66 to -0.44)	37(27.1 to 48.4)	13.6(10 to 18)	54.3(39.9 to 70.2)	12.5(9.1 to 16.2)	-0.31(-0.34 to -0.28)
Belarus	SAH	9114.8(8406.1 to 9990.4)	75.7(69.8 to 82.8)	9367.1(8724 to 10141.4)	71.7(66.8 to 77.6)	-0.17(-0.21 to -0.13)	990.2(866 to 1116.9)	8.4(7.3 to 9.4)	948.2(848.9 to 1062.9)	7.4(6.6 to 8.2)	-0.46(-0.52 to -0.39)	1655.4(1193.1 to 2145.2)	13.7(9.8 to 17.8)	1709.9(1206 to 2236.3)	13.1(9.2 to 17.1)	-0.15(-0.19 to -0.12)
Belgium	SAH	10296.1(9512.3 to 11149.1)	78.5(72 to 85.2)	12417.3(11560.3 to 13365.9)	71.2(65.7 to 77.2)	-0.43(-0.51 to -0.35)	824.2(718.7 to 940.7)	6.5(5.6 to 7.4)	1027(912 to 1175.8)	5.7(5 to 6.5)	-0.67(-0.91 to -0.43)	1392.9(990.3 to 1811.8)	10.5(7.5 to 13.7)	1719.3(1202.5 to 2205.1)	9.6(6.7 to 12.4)	-0.39(-0.46 to -0.31)
Belize	SAH	151.1(140.4 to 161.9)	126.6(118.5 to 134.8)	419.7(393 to 447.3)	115(108 to 122)	-0.37(-0.39 to -0.35)	12.4(10.9 to 13.8)	9.6(8.6 to 10.9)	31.2(27.7 to 35)	8.4(7.5 to 9.4)	-0.6(-0.66 to -0.53)	13.4(9.7 to 17.3)	11.3(8.2 to 14.7)	36.4(27.1 to 46.7)	10.2(7.7 to 13.1)	-0.39(-0.42 to -0.37)
Benin	SAH	2551.7(2347.1 to 2773.2)	93.4(86.8 to 100.5)	6584.1(6064.1 to 7217.2)	84.7(79.1 to 91)	-0.4(-0.43 to -0.38)	203.3(173.3 to 231.6)	6.3(5.3 to 7.3)	490.3(418.4 to 564.6)	5.4(4.7 to 6.4)	-0.62(-0.68 to -0.57)	336.9(238 to 430.3)	12.5(8.9 to 16.1)	869.4(609.2 to 1112.3)	11.5(8.1 to 14.7)	-0.38(-0.4 to -0.35)
Bermuda	SAH	105.4(99.1 to 111.2)	164.3(154.7 to 173.1)	141.7(134.2 to 149.4)	141.4(134.5 to 149.1)	-0.55(-0.58 to -0.52)	7.1(6.2 to 8)	11.2(9.9 to 12.7)	8(7 to 9.1)	8.6(7.5 to 9.7)	-1.07(-1.16 to -0.99)	9.3(6.9 to 12.2)	14.7(10.8 to 19)	13(9.6 to 16.9)	12.6(9.2 to 16.4)	-0.55(-0.58 to -0.52)
Bhutan	SAH	343.3(311.9 to 375.3)	95.3(87.2 to 104)	549.9(505.9 to 595.2)	78.4(72.2 to 84.8)	-0.66(-0.68 to -0.64)	37(31.6 to 42.3)	10.6(9.2 to 12.3)	52.7(45.3 to 60)	7.6(6.7 to 8.7)	-1.32(-1.41 to -1.22)	47.9(34.8 to 61.5)	13.5(9.9 to 17.4)	78.2(55.4 to 101.9)	11.2(7.9 to 14.7)	-0.61(-0.63 to -0.59)
Bolivia (Plurinational State of)	SAH	9211.1(8666.4 to 9794.2)	225(210.8 to 239.2)	17761.1(16758.6 to 18776.5)	171.8(161.8 to 181.4)	-1(-1.04 to -0.95)	869.7(766.1 to 985.4)	19.9(17.6 to 22.7)	1485.1(1316.3 to 1663.8)	14.4(12.9 to 16.2)	-1.27(-1.36 to -1.19)	789.5(578.6 to 1032.8)	19.7(14.6 to 25.3)	1542.1(1121.7 to 1970.4)	15.2(11.1 to 19.4)	-0.95(-1 to -0.91)
Bosnia and Herzegovina	SAH	4328.6(4001.1 to 4695.5)	92(84.8 to 99.6)	3537(3279.4 to 3802.4)	76.6(70.7 to 82.4)	-0.69(-0.73 to -0.65)	443.3(383.9 to 502.2)	10.4(9.1 to 11.8)	387.1(350.5 to 428.1)	7.7(6.9 to 8.6)	-1.09(-1.15 to -1.02)	780.5(544.8 to 1009.2)	16.7(11.8 to 21.5)	634.7(450 to 834.9)	13.7(9.6 to 18)	-0.71(-0.75 to -0.67)
Botswana	SAH	629.3(570.9 to 691.8)	87(80.1 to 93.9)	1450.4(1326.6 to 1574.5)	79.1(73.1 to 85.2)	-0.37(-0.39 to -0.35)	51(43.7 to 58.3)	6.7(5.7 to 7.8)	109.5(93.3 to 127.8)	5.9(5.1 to 6.7)	-0.6(-0.68 to -0.52)	83.7(58.2 to 106)	11.9(8.5 to 15.1)	193.4(136.5 to 252.4)	10.8(7.7 to 14.1)	-0.36(-0.38 to -0.35)
Brazil	SAH	161141.9(141723 to 181480.7)	146(128.4 to 165.7)	270875.2(240858.1 to 305149)	108.1(96.3 to 121.7)	-1.18(-1.27 to -1.08)	16353.4(13744.8 to 19081.8)	14(11.8 to 16.5)	25289.7(21783.8 to 29544)	10.2(8.8 to 11.8)	-1.38(-1.53 to -1.24)	13721.1(9780.5 to 17873.4)	12.6(9.1 to 16.4)	23816(17194.5 to 30828.1)	9.6(6.9 to 12.4)	-1.09(-1.18 to -1.01)
Brunei Darussalam	SAH	290.3(269.5 to 310.3)	220.7(203.7 to 236.2)	697.5(660.2 to 738.6)	172.3(162.8 to 182.9)	-0.91(-0.96 to -0.85)	34.1(28 to 39.8)	23(18.8 to 27.4)	67.3(58.6 to 78.6)	15.6(13.8 to 17.9)	-1.63(-1.79 to -1.46)	46.1(32.8 to 58.7)	35.4(25.7 to 45.1)	109.5(77.6 to 139.8)	27.6(19.7 to 35.2)	-0.91(-0.96 to -0.86)

Bulgaria	SAH	8269(7635.8 to 8954.6)	76.5(70.5 to 82.9)	7404.5(6836.2 to 7975.1)	75.9(70.1 to 81.7)	-0.05(-0.07 to -0.03)	827.7(736.6 to 916.4)	8.1(7.2 to 8.9)	900.4(823 to 985.3)	8.3(7.5 to 9.1)	0.12(0.08 to 0.15)	1475.6(1044 to 1934)	13.7(9.5 to 18)	1323(913.1 to 1726.6)	13.5(9.3 to 17.6)	-0.04(-0.06 to -0.02)
Burkina Faso	SAH	4967.1(4542.8 to 5446.2)	86.8(79.9 to 94.2)	10882(9977.7 to 11903.5)	80.5(74.7 to 86.9)	-0.29(-0.3 to -0.27)	376(318.5 to 432.9)	5.6(4.7 to 6.6)	797.2(686.9 to 911.7)	5(4.3 to 5.8)	-0.44(-0.48 to -0.39)	656.9(463.5 to 845.2)	11.7(8.3 to 15.1)	1448.5(1051.4 to 1873.1)	11(8.1 to 14.1)	-0.25(-0.26 to -0.23)
Burundi	SAH	3395.6(3117.4 to 3684)	116.2(107.1 to 125.4)	7028.4(6462.2 to 7597.5)	100.9(93.7 to 108)	-0.51(-0.54 to -0.49)	305.9(263.1 to 353.8)	9.6(8.2 to 11.4)	605.4(519.1 to 699.6)	8(6.9 to 9.5)	-0.81(-0.89 to -0.73)	446.2(319.5 to 566.2)	15.5(11.3 to 19.6)	912.3(645.5 to 1176)	13.5(9.8 to 17.1)	-0.51(-0.53 to -0.49)
Cabo Verde	SAH	243.5(224.1 to 262)	98.5(90.9 to 105.9)	467.6(434.9 to 502.6)	91.2(84.7 to 97.6)	-0.27(-0.3 to -0.25)	16(13.7 to 18.3)	6(5 to 7)	27.2(22.7 to 32)	5.2(4.4 to 6.1)	-0.57(-0.64 to -0.51)	33.5(24.2 to 43)	13.6(9.9 to 17.5)	63.1(45 to 80.3)	12.5(9 to 15.8)	-0.29(-0.31 to -0.27)
Cambodia	SAH	7062.7(6502.8 to 7693.4)	114.8(106.1 to 124.4)	15314.4(14188.1 to 16432.5)	102.6(95.1 to 109.8)	-0.41(-0.43 to -0.39)	753.1(660.6 to 845.2)	13.1(11.6 to 14.8)	1495.2(1316.1 to 1692.3)	10.8(9.5 to 12.2)	-0.79(-0.84 to -0.73)	1327.8(941.9 to 1734.5)	21.6(15.5 to 28.1)	2882.1(2076.1 to 3753.6)	19.4(14 to 25.2)	-0.39(-0.41 to -0.37)
Cameroon	SAH	5544.4(5087.5 to 6075.2)	89.7(82.8 to 97.6)	16176.3(14687.7 to 17658.7)	84.4(78 to 90.6)	-0.23(-0.24 to -0.22)	410.3(353.1 to 469.5)	5.7(4.9 to 6.7)	1151.8(983.6 to 1335.4)	5.3(4.6 to 6.1)	-0.34(-0.41 to -0.27)	731(524.8 to 952.9)	12.1(8.7 to 15.6)	2131.8(1523.6 to 2722.1)	11.4(8.2 to 14.3)	-0.23(-0.24 to -0.22)
Canada	SAH	38642.2(36179.5 to 41176.2)	124.1(116.2 to 132.4)	58392.1(54702.1 to 62100.4)	103.2(96.6 to 110)	-0.68(-0.71 to -0.64)	2849.7(2489.7 to 3262.6)	9.3(8.2 to 10.6)	3883.3(3430 to 4441.6)	7(6.1 to 8)	-1.02(-1.08 to -0.96)	5538.7(3919.2 to 7141.8)	17.8(12.5 to 22.9)	8586(6196.3 to 11112.7)	15(10.7 to 19.2)	-0.63(-0.66 to -0.59)
Central African Republic	SAH	1359.4(1232.7 to 1494.2)	91(83.3 to 99.1)	2766.5(2519 to 3015.6)	89.3(81.9 to 96.5)	-0.07(-0.09 to -0.06)	125.1(107.3 to 146.1)	7.8(6.6 to 9.2)	252.2(214.9 to 296.9)	7.5(6.5 to 8.8)	-0.15(-0.16 to -0.13)	175.1(123.2 to 225.1)	12(8.5 to 15.2)	356.4(255.4 to 456.4)	11.8(8.6 to 15)	-0.06(-0.08 to -0.04)
Chad	SAH	3411(3139.5 to 3723)	94.4(87.2 to 102.2)	8199.2(7476.7 to 8932.6)	89.4(83.1 to 95.4)	-0.21(-0.22 to -0.19)	257.6(219.4 to 292.9)	6.1(5.1 to 7.1)	642.3(548.2 to 735.1)	5.7(4.9 to 6.6)	-0.32(-0.38 to -0.25)	452.4(325.7 to 578.1)	12.7(9.2 to 16.4)	1073.1(766 to 1366.1)	12(8.7 to 15.2)	-0.22(-0.24 to -0.21)
Chile	SAH	13160.1(12205.5 to 14167)	115.5(107 to 124.2)	26323.4(24366.2 to 28746)	111.4(103.6 to 121)	-0.02(-0.09 to 0.05)	1324.6(1172.1 to 1514.2)	11.5(10.2 to 13.2)	2225.1(1960.8 to 2514.8)	9.6(8.5 to 10.8)	-0.37(-0.49 to -0.26)	1851.2(1320.7 to 2397.1)	16.3(11.6 to 21)	3722.2(2681 to 4739.9)	15.7(11.4 to 20.1)	0.02(-0.06 to 0.1)
China	SAH	1104538(961687.3 to 1242644.7)	107.9(94.6 to 121.8)	1323286.9(1176081.1 to 1484052.1)	68.9(61.5 to 76.9)	-1.65(-1.76 to -1.54)	150558.7(128668.9 to 176863)	18(15.4 to 21.1)	145138.5(125425.4 to 169016.4)	7.8(6.9 to 8.9)	-3.7(-4.08 to -3.31)	213547.8(148686.2 to 278716.5)	21.1(14.7 to 27.6)	256151.3(180970.7 to 336575.9)	13.4(9.4 to 17.4)	-1.68(-1.8 to -1.56)
Colombia	SAH	44055.1(41196 to 46623.5)	203(190.8 to 214.5)	97518.3(92132.3 to 102356.3)	178.3(168.4 to 187.1)	-0.5(-0.53 to -0.47)	3582(3162.5 to 3985.9)	15.3(13.6 to 17.3)	7570.4(6798 to 8534.6)	14(12.6 to 15.7)	-0.42(-0.5 to -0.34)	3825.1(2834.3 to 4975.4)	18(13.3 to 23.4)	8828.8(6494.6 to 11310.5)	16.1(11.9 to 20.7)	-0.4(-0.43 to -0.37)
Comoros	SAH	290(266.2 to 313)	116(107 to 124.7)	585.7(546.7 to 629.4)	101.1(94.1 to 108.4)	-0.5(-0.52 to -0.47)	24.5(21 to 28.4)	8.9(7.6 to 10.6)	44.5(38.3 to 52.4)	7.4(6.4 to 8.6)	-0.81(-0.88 to -0.74)	37.7(26.4 to 48.7)	15.4(10.9 to 19.8)	76.8(55.6 to 96.6)	13.5(9.8 to 16.9)	-0.47(-0.49 to -0.44)
Congo	SAH	1259(1142.2 to 1371.3)	93.8(86 to 101.7)	3003.3(2760.1 to 3253)	82.6(76.7 to 88.6)	-0.46(-0.48 to -0.43)	110.9(94.5 to 128.4)	7.6(6.5 to 9)	244(206.7 to 283)	6.2(5.3 to 7.1)	-0.9(-0.98 to -0.82)	164.2(120.5 to 209)	12.5(9.3 to 15.9)	391(277 to 500)	11(7.9 to 14.1)	-0.45(-0.47 to -0.42)
Cook Islands	SAH	27.9(26.4 to 29.6)	182.1(172 to 192.8)	33.7(32 to 35.5)	153(145.5 to 160.7)	-0.62(-0.65 to -0.59)	2.1(1.9 to 2.4)	14.1(12.5 to 15.9)	2(1.8 to 2.3)	9.9(8.8 to 11)	-1.37(-1.5 to -1.23)	5.2(3.6 to 6.7)	33.8(23.6 to 43.7)	6.2(4.5 to 8)	28.2(20.3 to 36.7)	-0.64(-0.67 to -0.6)
Costa Rica	SAH	3679.8(3467.4 to 3906.3)	173.3(163.5 to 183.2)	8384(7927.5 to 8836.9)	155.4(146.8 to 163.7)	-0.41(-0.43 to -0.38)	277.8(241.4 to 313)	12.3(10.8 to 14)	541(470.5 to 616)	10.3(8.9 to 11.7)	-0.68(-0.75 to -0.61)	324.5(236.4 to 419)	15.5(11.4 to 20.1)	751.6(551.9 to 978.6)	13.9(10.2 to 18.1)	-0.38(-0.4 to -0.36)

Croatia	SAH	5969.9(5397.6 to 6587.2)	89.8(82.7 to 97.3)	14078.4(12886.9 to 15366.4)	81.2(75.2 to 87.3)	-0.37(-0.38 to -0.35)	465.6(391.7 to 533.6)	5.8(4.9 to 6.8)	1007.7(863.3 to 1152.4)	5.1(4.4 to 5.8)	-0.57(-0.63 to -0.52)	770.7(559.2 to 987)	11.9(8.7 to 15.1)	1844.4(1329.3 to 2383.4)	10.9(7.9 to 13.9)	-0.31(-0.33 to -0.3)
Cuba	SAH	4757.3(4397.6 to 5157.1)	80.6(74.6 to 87.4)	3767.3(3461.9 to 4074.8)	65(59.5 to 70.5)	-0.81(-0.86 to -0.77)	492.6(440.7 to 556.1)	8.7(7.8 to 9.7)	391.6(355.5 to 430.6)	5.9(5.3 to 6.6)	-1.51(-1.63 to -1.39)	856.5(609 to 1114.2)	14.6(10.4 to 18.9)	690.4(478.4 to 890)	11.8(8.1 to 15.2)	-0.78(-0.83 to -0.74)
Cyprus	SAH	16393.9(15369.3 to 17449.7)	154.7(145.1 to 164.5)	21078.9(20088.3 to 22129.1)	130.9(124.3 to 137.6)	-0.61(-0.65 to -0.58)	1143.6(1002.5 to 1287.1)	10.9(9.6 to 12.3)	1277.6(1126.3 to 1483.8)	8.4(7.4 to 9.5)	-1.03(-1.11 to -0.95)	1448.2(1061.8 to 1880.3)	13.7(10.1 to 17.8)	1903.6(1376.5 to 2504)	11.6(8.4 to 15.3)	-0.6(-0.63 to -0.57)
Czechia	SAH	956.3(881.5 to 1015.8)	115.5(106.8 to 122.8)	1634.4(1522.3 to 1735.9)	88(81.9 to 93.7)	-1(-1.06 to -0.93)	84.1(73.5 to 96.7)	10.5(9.3 to 12)	123.4(107.9 to 141.8)	7(6.1 to 8)	-1.87(-2.06 to -1.68)	125.7(91.2 to 162.6)	15.2(11 to 19.7)	218.7(154.6 to 285.2)	11.7(8.3 to 15.3)	-0.97(-1.04 to -0.9)
C 么 te d'Ivoire	SAH	11074.3(10247.2 to 11917.6)	91.1(84.2 to 97.8)	10905.9(10106.9 to 11698)	72.9(67 to 78.3)	-0.79(-0.82 to -0.75)	1076.3(957.5 to 1198.6)	9(7.9 to 9.9)	956.9(857 to 1066)	6.1(5.4 to 6.8)	-1.52(-1.65 to -1.39)	1999.6(1393.5 to 2613.1)	16.4(11.4 to 21.6)	1985.8(1396.7 to 2602.6)	13.2(9.3 to 17.3)	-0.77(-0.81 to -0.73)
Democratic People's Republic of Korea	SAH	21751.7(20105.3 to 23416.2)	113.9(104.9 to 123.2)	32424.8(30231.4 to 34868.7)	99.3(92.3 to 107)	-0.5(-0.52 to -0.48)	2484.7(2171 to 2834)	14.7(13 to 16.7)	3649.4(3191.9 to 4137.1)	11.6(10.3 to 13)	-0.99(-1.07 to -0.91)	4202.4(2960.1 to 5471.2)	22.3(15.8 to 29)	6268.7(4376.8 to 8221.8)	19.3(13.4 to 25.2)	-0.52(-0.54 to -0.5)
Democratic Republic of the Congo	SAH	19752.7(18025.4 to 21513.9)	97.4(89.8 to 105.8)	48958.7(45235.8 to 52954.2)	97.7(90.8 to 104.8)	0(0 to 0)	1719.7(1456 to 2019.2)	7.8(6.6 to 9.3)	4234.4(3629.8 to 4964.3)	7.7(6.7 to 9.1)	-0.02(-0.05 to 0.01)	2552.1(1873.7 to 3277.2)	12.9(9.5 to 16.5)	6352(4554.9 to 8194.9)	13(9.4 to 16.8)	0.04(0.03 to 0.05)
Denmark	SAH	8625.6(8052.6 to 9203.6)	125(116.9 to 133)	9040.9(8486.8 to 9585)	98.9(92.6 to 105.5)	-0.74(-0.77 to -0.71)	708.6(628.9 to 798.1)	10.7(9.5 to 12.1)	654.7(576.9 to 748.2)	7.4(6.5 to 8.5)	-1.32(-1.39 to -1.25)	1169(840.8 to 1507.1)	16.7(12.1 to 21.6)	1257.6(909 to 1616.6)	13.4(9.7 to 17.3)	-0.65(-0.69 to -0.61)
Djibouti	SAH	229.7(209.5 to 250.5)	110(101.6 to 118.4)	897(836 to 967)	101.2(94.5 to 108.4)	-0.3(-0.32 to -0.27)	20(16.9 to 23.4)	8.5(7.3 to 10)	71.7(60.6 to 84.6)	7.5(6.4 to 8.7)	-0.51(-0.57 to -0.46)	29.5(21 to 37.8)	14.6(10.6 to 18.7)	115.5(81.5 to 149.4)	13.4(9.4 to 17.2)	-0.3(-0.32 to -0.28)
Dominica	SAH	86.9(80.7 to 92.6)	143.1(133.3 to 152.5)	98.9(93.1 to 105.1)	124.6(117.1 to 132.6)	-0.52(-0.55 to -0.49)	6.6(5.8 to 7.4)	10.7(9.5 to 12.1)	6.9(6 to 7.8)	9(7.9 to 10.1)	-0.67(-0.74 to -0.6)	7.7(5.7 to 10)	12.8(9.3 to 16.5)	8.6(6.2 to 11.1)	10.9(7.9 to 14.2)	-0.57(-0.6 to -0.54)
Dominican Republic	SAH	6647.8(6173.8 to 7142.7)	137(128.2 to 146.4)	15003.7(14095.7 to 15895.4)	141.8(133.2 to 150.1)	0.13(0.12 to 0.14)	525.1(454.9 to 587.9)	10(8.8 to 11.3)	1175.5(1037.4 to 1336.8)	11(9.7 to 12.5)	0.42(0.36 to 0.47)	584.2(418.1 to 766)	12.3(8.8 to 16.1)	1325.4(971.4 to 1712.5)	12.6(9.2 to 16.2)	0.09(0.08 to 0.1)
Ecuador	SAH	14695.9(13844.4 to 15517)	223.4(210.7 to 235.1)	34297.3(32563.7 to 35907.5)	199.8(189.6 to 209.3)	-0.4(-0.43 to -0.38)	1220.8(1087.9 to 1361)	16.8(15 to 18.9)	2585(2336.5 to 2906.2)	15(13.6 to 16.9)	-0.32(-0.35 to -0.28)	1272.6(936.6 to 1640.2)	19.7(14.6 to 25.2)	3024.9(2212.5 to 3876.1)	17.7(12.9 to 22.7)	-0.38(-0.4 to -0.35)
Egypt	SAH	33063.3(30206.6 to 36373.5)	80.3(73.9 to 88.2)	58523.5(53457.4 to 64093.6)	65.5(60.1 to 71.5)	-0.76(-0.8 to -0.72)	2959.9(2536.6 to 3372.7)	7.5(6.6 to 8.6)	4577.5(3929.5 to 5241.4)	5.5(4.8 to 6.2)	-1.2(-1.27 to -1.13)	4695(3221.7 to 6119.5)	11.5(7.9 to 14.9)	8299(5784.4 to 10771.8)	9.4(6.5 to 12.1)	-0.75(-0.79 to -0.71)
El Salvador	SAH	6260.1(5893.4 to 6654.6)	175.7(165.1 to 186)	8409.9(7931.5 to 8928.8)	134.7(127.1 to 142.9)	-1(-1.08 to -0.93)	545(480.4 to 610.7)	14.1(12.4 to 15.9)	616(548 to 687)	9.7(8.7 to 10.9)	-1.34(-1.55 to -1.13)	553.6(406.9 to 711.3)	15.7(11.5 to 20.3)	768.2(544.9 to 985.6)	12.2(8.6 to 15.7)	-0.94(-1.01 to -0.88)
Equatorial Guinea	SAH	220.8(201.6 to 241)	92.9(85 to 100.5)	597.3(540.6 to 656.8)	74.1(68.5 to 79.9)	-0.82(-0.86 to -0.78)	20.8(17.8 to 24.4)	8(6.8 to 9.5)	48.7(40.6 to 56.4)	5.3(4.5 to 6.2)	-1.65(-1.81 to -1.49)	28.6(20.8 to 36.3)	12.3(9 to 15.7)	77.7(53.9 to 100.3)	9.9(7.1 to 12.7)	-0.78(-0.82 to -0.74)
Eritrea	SAH	1908.3(1745.7 to 2067.8)	115(106.2 to 124.3)	4228.3(3918.7 to 4550.1)	107.7(100.6 to 115)	-0.24(-0.25 to -0.22)	175.3(148.6 to 206.3)	9.4(8 to 11.3)	358.2(304.4 to 419.4)	8.2(7.1 to 9.7)	-0.54(-0.61 to -0.48)	247(175.2 to 315.3)	15.4(11.1 to 19.7)	547.6(390.3 to 700.2)	14.4(10.4 to 18.3)	-0.24(-0.25 to -0.22)

Estonia	SAH	1747.2(1610.3 to 1893.9)	93.7(86.6 to 101.5)	1215.8(1119.6 to 1321.6)	66.8(62.1 to 71.9)	-1.2(-1.27 to -1.13)	173(153 to 196.4)	9.6(8.4 to 10.8)	120.7(107.5 to 135.6)	6.4(5.6 to 7.1)	-1.73(-1.88 to -1.58)	312.9(215.2 to 412.5)	16.8(11.6 to 22)	223.6(158.3 to 292.8)	12.1(8.7 to 15.9)	-1.14(-1.21 to -1.06)
Eswatini	SAH	311.4(277.5 to 348.4)	76.7(69.9 to 83.7)	540.5(493.6 to 594.2)	72.4(67.2 to 78.4)	-0.23(-0.24 to -0.21)	25.5(21.9 to 29.5)	5.9(5 to 6.8)	41.8(35.9 to 48)	5.6(4.9 to 6.4)	-0.16(-0.27 to -0.05)	41.6(29 to 53.2)	10.5(7.5 to 13.5)	72(51.7 to 92.5)	9.9(7.1 to 12.7)	-0.26(-0.29 to -0.24)
Ethiopia	SAH	31941.9(28048.5 to 36052.2)	123.6(106.9 to 141.3)	51392.4(45752.5 to 57503.4)	84.8(75.3 to 95.1)	-1.35(-1.4 to -1.29)	2983.2(2554 to 3493)	10.5(8.9 to 12.6)	4282.1(3638 to 4945.2)	6.5(5.6 to 7.7)	-1.95(-2.11 to -1.79)	4065.4(2913.1 to 5257.1)	16.1(11.7 to 20.6)	6680.7(4703.8 to 8578.6)	11.2(8 to 14.4)	-1.27(-1.32 to -1.22)
Fiji	SAH	1149.2(1080.3 to 1215.1)	213.8(200.3 to 227.5)	1622.7(1533.5 to 1709.5)	183.1(172.5 to 193.1)	-0.59(-0.62 to -0.56)	101.8(89.6 to 114.3)	20.2(18 to 22.5)	132.1(119 to 146.4)	16.5(15 to 18.2)	-0.91(-1 to -0.82)	214.6(150.1 to 277.2)	40.2(28.1 to 52)	300.8(214.8 to 388.8)	34.2(24.4 to 44.1)	-0.6(-0.63 to -0.58)
Finland	SAH	12780.7(12139.1 to 13423.8)	195.2(185 to 204.8)	15604.9(14829.4 to 16368.7)	160.7(152.8 to 168.9)	-0.56(-0.72 to -0.41)	850(772.1 to 932.3)	13.5(12.3 to 14.9)	874.8(771.6 to 993.1)	10.3(9 to 11.7)	-1.14(-1.41 to -0.87)	1699.5(1233.3 to 2209.3)	25.8(18.7 to 33.5)	2173.3(1585.3 to 2735.5)	21.5(15.6 to 27.3)	-0.5(-0.65 to -0.35)
France	SAH	61900.5(57887.4 to 66014.8)	86.3(80.6 to 92.5)	90775.2(85341.5 to 96584)	87.1(81.5 to 93.2)	0.03(0.02 to 0.04)	4571.7(4063.8 to 5128.7)	6.5(5.7 to 7.3)	6760.8(6084.5 to 7534.6)	6.5(5.8 to 7.3)	0.09(0.04 to 0.13)	8489.8(6153 to 10837.2)	11.7(8.5 to 15)	13044.8(9490.7 to 16776)	12(8.6 to 15.5)	0.13(0.11 to 0.15)
Gabon	SAH	598.5(548.9 to 655.4)	92.7(85.1 to 100.7)	1076.9(989.5 to 1161.4)	83.1(76.9 to 89.2)	-0.38(-0.41 to -0.35)	49.6(42.7 to 57.3)	7.2(6.2 to 8.5)	83(70.4 to 96.4)	6(5.2 to 7)	-0.81(-0.88 to -0.73)	79.2(57.5 to 101.8)	12.4(9.1 to 16)	140.3(99.6 to 180.4)	11.1(7.9 to 14.1)	-0.38(-0.41 to -0.36)
Gambia	SAH	501.8(460.2 to 549.2)	91.9(85 to 99)	1229.2(1131.7 to 1348.4)	85.3(79.5 to 91.7)	-0.28(-0.29 to -0.26)	37.9(32.1 to 43.9)	5.8(4.9 to 6.8)	85.9(73.5 to 99.4)	5.3(4.6 to 6.2)	-0.36(-0.4 to -0.31)	65.9(47.2 to 84.1)	12.3(8.9 to 15.8)	161.8(115.6 to 208.3)	11.5(8.2 to 14.7)	-0.26(-0.27 to -0.24)
Georgia	SAH	7735.3(7229.5 to 8250.3)	127(118.6 to 135.4)	6621.4(6177.2 to 7050.5)	136.1(127.1 to 144.8)	0.24(0.22 to 0.26)	803.1(716.1 to 897.3)	13.7(12.2 to 15.2)	831.3(760.8 to 912.4)	16.1(14.7 to 17.8)	0.83(0.73 to 0.94)	1390.8(994.8 to 1778.3)	22.8(16.1 to 29.5)	1185.8(837.4 to 1525)	24.2(17.1 to 31.1)	0.21(0.19 to 0.24)
Germany	SAH	123141.8(114436.4 to 131215)	111.1(103.2 to 118.2)	134982.9(126165 to 142395.8)	93.3(86.8 to 99.3)	-0.58(-0.62 to -0.54)	9095.5(8025.4 to 10367.8)	8.6(7.5 to 9.8)	8639(7600.2 to 10001.4)	6.3(5.4 to 7.3)	-1.05(-1.1 to -1)	16665.6(11922 to 21318.9)	14.9(10.6 to 19.1)	18668.2(13332.6 to 23733.6)	12.5(8.9 to 15.9)	-0.56(-0.61 to -0.52)
Ghana	SAH	9718.2(8935.9 to 10548)	108.9(100.9 to 117.4)	23452.6(21811 to 25292)	101.4(95 to 108.1)	-0.26(-0.28 to -0.25)	686.3(585.6 to 787.3)	6.6(5.6 to 7.7)	1569.9(1334.5 to 1818.7)	6.1(5.2 to 7.1)	-0.29(-0.36 to -0.22)	1273.8(924.4 to 1634.3)	14.6(10.6 to 18.6)	3085.7(2214.5 to 3916.2)	13.7(9.8 to 17.4)	-0.24(-0.25 to -0.22)
Greece	SAH	13430(12527.1 to 14396.9)	99(92 to 106.3)	18452.5(17284.6 to 19551)	105.5(98.7 to 111.7)	0.26(0.23 to 0.29)	958.8(829.8 to 1114.1)	7.4(6.4 to 8.5)	1234.2(1093.1 to 1413.5)	7.2(6.3 to 8.2)	0.2(0.1 to 0.31)	1800.8(1286.4 to 2333.6)	13.2(9.4 to 17.2)	2556.9(1843.5 to 3309.9)	14.1(10.1 to 18.2)	0.25(0.23 to 0.28)
Greenland	SAH	61.2(53 to 82.6)	132.4(115.3 to 175.7)	78.6(73.1 to 83.9)	111.9(104 to 120.2)	-0.67(-0.74 to -0.61)	5(3.9 to 8.5)	11.3(8.9 to 19.4)	7.5(6.7 to 8.4)	11.6(10.6 to 12.9)	0.14(0.02 to 0.26)	8.5(5.8 to 12.2)	18.7(12.9 to 26.1)	10.8(7.6 to 13.9)	15.7(11 to 20.2)	-0.66(-0.71 to -0.6)
Grenada	SAH	109.1(102.1 to 116.7)	155.4(146.2 to 165.9)	155(147 to 163.2)	135(127.8 to 142.4)	-0.51(-0.53 to -0.5)	9.2(8.3 to 10.3)	12.9(11.5 to 14.5)	11.4(10.2 to 13)	10.3(9.2 to 11.5)	-0.86(-0.93 to -0.79)	9.9(7.2 to 12.7)	13.8(10 to 17.6)	13.4(10 to 17.3)	11.9(8.8 to 15.3)	-0.53(-0.55 to -0.51)
Guam	SAH	171.7(162.4 to 182.2)	159.2(150.2 to 168.8)	290.8(275.8 to 305.8)	154.1(146.5 to 161.8)	-0.09(-0.12 to -0.07)	12.9(11.3 to 14.4)	12.3(11 to 13.8)	18.5(16.5 to 20.7)	10.1(9 to 11.2)	-0.79(-0.85 to -0.72)	32(21.8 to 42.4)	29.9(20.6 to 39.1)	54.3(39 to 70.7)	28.7(20.5 to 37.3)	-0.11(-0.14 to -0.08)
Guatemala	SAH	6200.3(5771.3 to 6657.9)	130.2(122.3 to 138.3)	16258.1(15267.9 to 17232)	127.4(120 to 134.5)	-0.09(-0.1 to -0.09)	498(432.4 to 557.1)	9(7.9 to 10.2)	1198.2(1065.6 to 1345)	8.9(8 to 10.1)	-0.04(-0.07 to -0.01)	531.1(386.3 to 691.7)	11.5(8.5 to 14.8)	1418.7(1027.2 to 1847.9)	11.3(8.1 to 14.7)	-0.09(-0.1 to -0.08)

Guinea	SAH	3940.4(3637.7 to 4234.4)	98.6(91.1 to 106)	7527.8(6959.1 to 8150.8)	94.7(88.4 to 101.6)	-0.15(-0.15 to -0.14)	290.4(251 to 332.8)	6.4(5.5 to 7.5)	555(479.7 to 633.1)	6.1(5.2 to 7.1)	-0.22(-0.25 to -0.19)	524.8(372.7 to 669.4)	13.3(9.5 to 17)	994.9(714.4 to 1276.4)	12.8(9.3 to 16.3)	-0.15(-0.16 to -0.14)
Guinea-Bissau	SAH	560.6(512.9 to 611.8)	98.2(90.5 to 106.2)	1024.7(938.3 to 1123.7)	88(81.4 to 95.1)	-0.41(-0.43 to -0.39)	43.6(37.2 to 49.8)	6.5(5.6 to 7.6)	76.5(64.5 to 88.3)	5.7(4.9 to 6.6)	-0.56(-0.62 to -0.5)	73.1(52.8 to 94.2)	13.1(9.5 to 16.7)	134.8(92.9 to 171.9)	11.9(8.3 to 15.1)	-0.38(-0.41 to -0.36)
Guyana	SAH	777.6(723.8 to 834.7)	148.6(138.1 to 159.3)	932.7(874.7 to 993.8)	129.8(121.8 to 138.5)	-0.52(-0.56 to -0.49)	76.1(68.4 to 84.8)	14.2(12.7 to 16.1)	80.8(72.8 to 90.4)	11.3(10.2 to 12.6)	-1.03(-1.14 to -0.92)	65.9(47.7 to 86.1)	12.8(9.4 to 16.8)	79(59.2 to 102.7)	11.2(8.4 to 14.6)	-0.5(-0.54 to -0.47)
Haiti	SAH	6743.8(6279.2 to 7210.4)	158(147 to 169.6)	14614.5(13671.6 to 15557.2)	150(139.9 to 160.2)	-0.2(-0.24 to -0.15)	681.6(563.9 to 796.4)	15.4(12.8 to 18.1)	1464.4(1290.9 to 1660)	14.8(13.1 to 16.9)	-0.28(-0.36 to -0.2)	572.2(420.7 to 741.2)	13.7(10.2 to 17.7)	1238.9(908.3 to 1619.1)	13.1(9.6 to 16.9)	-0.19(-0.24 to -0.15)
Honduras	SAH	4432(4144.2 to 4734.2)	161.1(151.5 to 171.7)	10805.1(10100.3 to 11516.9)	141.5(132.7 to 150.3)	-0.51(-0.54 to -0.48)	367.5(321.8 to 413.5)	12.1(10.7 to 13.8)	920.1(819.2 to 1035.6)	12.4(11.1 to 13.9)	0.02(-0.01 to 0.06)	383.8(280.7 to 496.1)	14.2(10.4 to 18.3)	944.3(697 to 1215.1)	12.6(9.4 to 16.3)	-0.48(-0.51 to -0.45)
Hungary	SAH	10437.4(9621.5 to 11260.2)	82.3(75.7 to 88.8)	9024.5(8324.3 to 9729.8)	67.1(61.8 to 72.2)	-0.74(-0.78 to -0.7)	953.4(857.7 to 1052.9)	7.8(7 to 8.6)	848.2(766.5 to 942.1)	6(5.3 to 6.6)	-1.06(-1.13 to -0.99)	1881.9(1278.6 to 2438.3)	14.8(10 to 19.4)	1646.9(1177 to 2134.3)	12.2(8.7 to 15.9)	-0.71(-0.75 to -0.67)
Iceland	SAH	341.4(318.8 to 363.4)	126.9(118.3 to 135.1)	506.8(474.6 to 536.4)	104.3(97.7 to 110.6)	-0.68(-0.72 to -0.65)	26.2(22.8 to 30.3)	9.8(8.5 to 11.3)	34.4(30.4 to 39.6)	7.2(6.3 to 8.3)	-1.19(-1.26 to -1.12)	46.1(32.5 to 58.9)	17(12 to 21.8)	69.2(49.5 to 89)	14(10 to 18.1)	-0.66(-0.69 to -0.62)
India	SAH	518466.8(449986.3 to 589482.6)	82.8(72.1 to 94.2)	975215(855887.8 to 1105598.1)	71.8(63.1 to 81.2)	-0.48(-0.51 to -0.46)	60704.5(51739 to 71049.9)	10.4(8.9 to 12.3)	101503.4(86895.3 to 118265.6)	7.7(6.6 to 8.9)	-1.3(-1.43 to -1.17)	71646.2(51359.8 to 93380.3)	11.5(8.4 to 15)	136114(96605.9 to 175704.1)	10.1(7.2 to 13)	-0.44(-0.47 to -0.41)
Indonesia	SAH	176217.3(154977.1 to 199409.9)	133.5(117 to 151.6)	313471.2(277711.6 to 352552.1)	109.1(96.7 to 122.7)	-0.74(-0.78 to -0.7)	17481.1(14897.6 to 20258.4)	13.5(11.7 to 15.9)	29375(25055.8 to 34149.6)	10.9(9.5 to 12.6)	-0.83(-0.88 to -0.77)	33045.4(23468 to 43068.5)	25.1(17.8 to 32.9)	58404.2(40792 to 76188.6)	20.5(14.3 to 26.7)	-0.74(-0.78 to -0.7)
Iran (Islamic Republic of)	SAH	28566.1(25386.5 to 32052.7)	73.6(65.5 to 82.8)	54855.1(49044.2 to 61208.9)	61.1(54.8 to 67.9)	-0.68(-0.72 to -0.64)	2591.9(2203.6 to 2978.5)	6.9(6 to 8)	3971.5(3395.1 to 4544.8)	4.8(4.2 to 5.4)	-1.43(-1.54 to -1.32)	4062.2(2786.8 to 5252.2)	10.5(7.4 to 13.6)	7871.9(5388.7 to 10327.2)	8.8(6.1 to 11.5)	-0.65(-0.68 to -0.61)
Iraq	SAH	10619.9(9698.6 to 11569.2)	90.7(83.8 to 98.1)	22567.7(20666.7 to 24840.9)	66(60.7 to 71.9)	-1.14(-1.2 to -1.09)	1020.8(900.3 to 1138.1)	8.7(7.7 to 9.7)	1676(1428.1 to 1905.9)	5.1(4.5 to 5.8)	-2.17(-2.38 to -1.96)	1496.6(1074.9 to 1941.6)	12.8(9.2 to 16.5)	3188.4(2242.7 to 4180.8)	9.4(6.7 to 12.2)	-1.1(-1.16 to -1.05)
Ireland	SAH	5561.2(5166.3 to 5930.1)	145.9(135.5 to 155.6)	6888.7(6439.6 to 7303.9)	101.1(94.5 to 107.3)	-1.34(-1.42 to -1.27)	456.7(394.7 to 523.2)	12.4(10.6 to 14.1)	499.9(442.9 to 575.5)	7.5(6.6 to 8.5)	-1.99(-2.13 to -1.85)	744.7(533.9 to 950.7)	19.4(14 to 24.8)	932.5(684 to 1198.1)	13.5(9.9 to 17.5)	-1.31(-1.38 to -1.24)
Israel	SAH	4342.8(4010.6 to 4674.3)	92(85 to 98.9)	8757.7(8166.7 to 9345.6)	80.7(75 to 86.5)	-0.47(-0.5 to -0.44)	318.5(270.5 to 365.7)	6.8(5.9 to 7.9)	605.8(522.5 to 697.9)	5.7(4.8 to 6.5)	-0.76(-0.82 to -0.7)	587.5(415.4 to 757.6)	12.4(8.7 to 16)	1202.5(862.9 to 1571.2)	10.9(7.8 to 14.3)	-0.44(-0.47 to -0.41)
Italy	SAH	57949.8(50895.1 to 65556.7)	76(67 to 85.3)	64232.3(56972.7 to 71456.6)	62.8(56.3 to 69.7)	-0.65(-0.69 to -0.62)	4946.2(4170.4 to 5911.4)	6.7(5.7 to 7.9)	5151.4(4585.8 to 5770.5)	4.8(4.3 to 5.4)	-1.16(-1.25 to -1.07)	7788(5537.2 to 10015.7)	10.2(7.1 to 13.2)	9168.2(6482 to 11856.5)	8.6(6.1 to 11.2)	-0.54(-0.56 to -0.52)
Jamaica	SAH	3336.8(3134.4 to 3534.3)	175(164.4 to 186)	4838.9(4573.3 to 5110)	158(149.4 to 166.7)	-0.37(-0.39 to -0.35)	253(221.6 to 286.3)	13.2(11.4 to 15)	351(314.6 to 394.3)	11.7(10.6 to 13.1)	-0.48(-0.6 to -0.36)	297.5(217.6 to 391.7)	15.5(11.4 to 20.5)	428.6(314.3 to 554.9)	13.9(10.2 to 18)	-0.37(-0.39 to -0.36)
Japan	SAH	324377.5(285813.3 to 368284.3)	195.9(173.2 to 220.7)	624486.7(552969.1 to 709169.4)	219.6(196.3 to 244.7)	0.41(0.37 to 0.44)	27463(23241.9 to 32955.7)	16.8(14.4 to 19.9)	37011.3(32479.5 to 42503.9)	15.8(13.7 to 18.4)	-0.21(-0.31 to -0.11)	53184.5(38157.7 to 68743.3)	32.3(23.1 to 41.5)	106357.2(76836.1 to 137757.5)	35.8(25.9 to 46.4)	0.36(0.33 to 0.39)

Jordan	SAH	1914(1754.6 to 2108)	81.3(74.9 to 88)	7516.9(6915.9 to 8195.3)	68.5(63.5 to 74.4)	-0.59(-0.63 to -0.55)	157.1(132.2 to 182.7)	6.4(5.6 to 7.3)	490.9(407.6 to 574.8)	4.7(4 to 5.4)	-1.31(-1.43 to -1.18)	268.4(184.9 to 350.5)	11.4(8 to 14.8)	1055.9(740.5 to 1388.1)	9.7(6.8 to 12.7)	-0.55(-0.59 to -0.51)
Kazakhstan	SAH	13107.9(12108.8 to 14153.2)	90.4(83.7 to 97.6)	17356.9(16125.5 to 18619.5)	87.9(81.4 to 94.5)	-0.13(-0.13 to -0.12)	1368.2(1201 to 1545.7)	9.9(8.7 to 11.1)	1852.8(1646 to 2077.1)	10.1(9.1 to 11.3)	0.08(0.04 to 0.12)	2347.7(1654.7 to 3059.2)	16.3(11.4 to 21.4)	3090.8(2227.4 to 3981.1)	15.8(11.4 to 20.3)	-0.13(-0.14 to -0.11)
Kenya	SAH	10772.7(9545.2 to 12080.8)	93.2(82.2 to 104.6)	27041.5(24018.3 to 30047.2)	86(76.7 to 96.1)	-0.29(-0.32 to -0.26)	941(799.9 to 1086)	7.4(6.3 to 8.7)	2161.5(1843.5 to 2498.5)	6.5(5.6 to 7.6)	-0.46(-0.55 to -0.37)	1399.1(995.3 to 1800.9)	12.3(9 to 15.7)	3515.5(2492.8 to 4520.5)	11.5(8.2 to 14.7)	-0.25(-0.28 to -0.23)
Kiribati	SAH	115.2(107.8 to 123.1)	221.5(205.9 to 238.1)	235.5(223 to 247.9)	241.2(227 to 255.3)	0.35(0.31 to 0.39)	10.4(8.6 to 13.5)	20(16.9 to 24.9)	21.3(18.7 to 24.1)	22(19.6 to 24.6)	0.27(0.19 to 0.35)	21.1(14.7 to 27.5)	40.8(29 to 53.2)	42.8(29.7 to 55.1)	44.1(30.9 to 56.2)	0.31(0.28 to 0.35)
Kuwait	SAH	1150(1058.5 to 1254.5)	88.3(81.3 to 95.2)	3886.3(3607.7 to 4216.8)	79.7(73.8 to 85.6)	-0.38(-0.41 to -0.34)	85.5(70.4 to 100.7)	6.4(5.5 to 7.3)	250.1(200.1 to 302.1)	5.5(4.8 to 6.3)	-0.62(-0.69 to -0.55)	161.3(112 to 208.3)	12.4(8.8 to 15.9)	544.4(377.9 to 720)	11.3(8 to 14.8)	-0.34(-0.36 to -0.31)
Kyrgyzstan	SAH	3590.1(3314.4 to 3890.9)	106.4(98.2 to 115.2)	5536.4(5113.2 to 5916.8)	91.7(84.6 to 97.8)	-0.55(-0.58 to -0.53)	379(333.5 to 425.7)	11.2(9.9 to 12.7)	564.3(502.6 to 629.4)	9.6(8.5 to 10.7)	-0.66(-0.77 to -0.56)	644.5(446.8 to 835.5)	19.1(13.3 to 24.8)	987.1(686.2 to 1302.2)	16.5(11.5 to 21.6)	-0.54(-0.56 to -0.52)
Lao People's Democratic Republic	SAH	3128.9(2886.1 to 3391.6)	116.9(107.8 to 127)	5993.9(5590.2 to 6442.5)	99.1(92 to 106.3)	-0.59(-0.62 to -0.57)	338.2(294 to 384.6)	13.3(11.7 to 15.3)	571.3(498.3 to 650.2)	9.9(8.7 to 11.1)	-1.19(-1.3 to -1.09)	585.3(408 to 757.7)	21.9(15.2 to 28.3)	1120.8(795.2 to 1450.6)	18.6(13.3 to 24.3)	-0.57(-0.6 to -0.55)
Latvia	SAH	2866.2(2671.9 to 3112.1)	89.3(83.5 to 96.9)	1915(1767.5 to 2085.5)	70.9(65.8 to 76.3)	-0.67(-0.71 to -0.64)	329.8(295.4 to 371.6)	10.4(9.3 to 11.7)	198.1(176.4 to 222.4)	7.2(6.4 to 8.1)	-1.57(-1.69 to -1.44)	513.1(363.3 to 661.9)	16(11.3 to 20.5)	349.7(244.1 to 460.7)	12.8(8.9 to 16.9)	-0.62(-0.66 to -0.59)
Lebanon	SAH	2522(2343.1 to 2715.6)	98.6(91.6 to 105.7)	4613(4264.7 to 4968.9)	78.3(72.5 to 84.3)	-0.83(-0.87 to -0.79)	211(183.2 to 240)	8.5(7.4 to 9.6)	326.8(279.9 to 369)	5.6(4.8 to 6.3)	-1.71(-1.83 to -1.59)	355.6(250.2 to 463.3)	14(9.9 to 18.1)	664.9(477.3 to 855.2)	11.2(8.1 to 14.5)	-0.79(-0.83 to -0.76)
Lesotho	SAH	738.3(664 to 817.8)	74.4(67.6 to 81.7)	1005.7(915.7 to 1101.8)	77.1(71 to 83.6)	0.1(0.07 to 0.12)	58.8(50.8 to 68.6)	5.7(4.9 to 6.8)	82.3(71.4 to 93.6)	6.5(5.6 to 7.4)	0.54(0.44 to 0.64)	100.9(71.4 to 131.1)	10.4(7.4 to 13.6)	134.2(96.4 to 172.5)	10.6(7.7 to 13.6)	0.03(0.01 to 0.05)
Liberia	SAH	1467.8(1347.7 to 1594.5)	97.1(89.7 to 104.8)	2893.3(2639 to 3171.5)	86.7(80.7 to 93.7)	-0.43(-0.45 to -0.4)	111.7(94.9 to 127.4)	6.3(5.4 to 7.4)	207.2(174.9 to 243.4)	5.5(4.7 to 6.5)	-0.6(-0.65 to -0.55)	191.3(136.9 to 244.4)	12.8(9.3 to 16.3)	374.8(270.2 to 481.6)	11.5(8.4 to 14.6)	-0.4(-0.42 to -0.38)
Libya	SAH	2401.1(2208.6 to 2629.8)	81.4(75.1 to 88.3)	4797.1(4407.3 to 5188.6)	68.7(63.1 to 74.1)	-0.62(-0.66 to -0.59)	203.8(174 to 232.5)	6.7(5.8 to 7.8)	332.9(281.2 to 386.4)	5.1(4.5 to 5.8)	-1.17(-1.28 to -1.05)	341.8(243.1 to 446.3)	11.6(8.2 to 15)	675.8(484.5 to 877.8)	9.7(6.9 to 12.6)	-0.63(-0.66 to -0.59)
Lithuania	SAH	3033.1(2797.7 to 3280.4)	72.5(66.9 to 78.5)	2749.5(2518.8 to 3003.1)	68.7(64 to 74.1)	-0.08(-0.12 to -0.05)	353.7(315.5 to 396.6)	8.5(7.6 to 9.5)	268(238.7 to 303.1)	6.7(6 to 7.6)	-0.72(-0.81 to -0.63)	548(382.6 to 721.1)	13.1(9.1 to 17.2)	504.6(352.4 to 659.5)	12.5(8.7 to 16.3)	-0.03(-0.08 to 0.01)
Luxembourg	SAH	399.8(370.7 to 429.6)	81.2(75.2 to 87.6)	614.9(569.1 to 661.4)	67.9(62.7 to 73.6)	-0.71(-0.8 to -0.62)	33.5(29.3 to 38.5)	7(6.1 to 8)	47.1(41 to 54.6)	5.2(4.5 to 6)	-1.41(-1.67 to -1.15)	54(38.3 to 70.5)	10.9(7.7 to 14.2)	84.6(60.4 to 110.4)	9.2(6.6 to 12.1)	-0.66(-0.73 to -0.58)
Madagascar	SAH	8699.7(8023.2 to 9374.3)	135.4(125.5 to 145.7)	20985.6(19571.3 to 22483.6)	130.4(122 to 139.5)	-0.13(-0.14 to -0.12)	735.7(628.1 to 848.1)	10.4(8.9 to 12.3)	1770.3(1503.5 to 2078.1)	9.9(8.4 to 11.5)	-0.25(-0.29 to -0.21)	1122.3(791.3 to 1452.7)	17.8(12.7 to 22.9)	2705.6(1941.9 to 3545.4)	17.3(12.5 to 22.4)	-0.09(-0.1 to -0.08)
Malawi	SAH	4792.4(4361.6 to 5258.1)	94.1(86.3 to 102.5)	9192.7(8419.7 to 10041.1)	87.8(81.5 to 94.4)	-0.26(-0.28 to -0.25)	422.5(356.6 to 491.1)	7.7(6.5 to 9.2)	783.7(667.2 to 912)	7(5.9 to 8.2)	-0.44(-0.48 to -0.39)	623.1(452.5 to 797.9)	12.5(9.2 to 15.9)	1200.2(855.3 to 1517.2)	11.8(8.4 to 14.9)	-0.22(-0.23 to -0.21)

Malaysia	SAH	16758.3(15605.7 to 17883.3)	134.3(124.7 to 143.5)	35378.7(33143.7 to 37644.1)	111.3(104 to 119)	-0.63(-0.66 to -0.6)	1635(1425.7 to 1832.4)	13.5(12 to 15.3)	2751.4(2408.2 to 3100.6)	9.1(8.1 to 10.1)	-1.48(-1.58 to -1.39)	3142.8(2165.9 to 4053.5)	25.2(17.7 to 32.8)	6617.3(4671.5 to 8537)	20.9(14.7 to 26.8)	-0.61(-0.64 to -0.58)
Maldives	SAH	170.8(159.4 to 184.1)	134.2(125.3 to 143.5)	492.7(456.7 to 529.2)	99.4(92.5 to 106.5)	-1.07(-1.13 to -1.02)	17.4(15.1 to 19.8)	14.6(12.7 to 16.6)	39.6(33.3 to 46.3)	8.5(7.5 to 9.7)	-2.1(-2.25 to -1.94)	31.9(22 to 41.4)	25.1(17.2 to 32.6)	90.9(62.6 to 118.9)	18.6(13 to 23.8)	-1.06(-1.11 to -1)
Mali	SAH	4904.5(4508.5 to 5344.1)	92.3(85.6 to 99.9)	11098.3(10189.1 to 12093.4)	82.8(76.9 to 89.1)	-0.4(-0.42 to -0.38)	377.2(321.5 to 434.9)	6.1(5.2 to 7.1)	826.5(707.6 to 943.2)	5.2(4.5 to 6)	-0.64(-0.69 to -0.6)	639.2(451.8 to 813.7)	12.3(8.7 to 15.6)	1447.8(1000.7 to 1861.5)	11(7.7 to 14.2)	-0.38(-0.4 to -0.36)
Malta	SAH	440.1(407.7 to 470.8)	104.9(97.2 to 112.5)	654.9(613 to 694.6)	89.9(83.5 to 95.6)	-0.56(-0.6 to -0.52)	32(27.2 to 37.3)	7.8(6.7 to 9)	40.4(34.9 to 46.5)	6.1(5.3 to 7.1)	-1.15(-1.28 to -1.01)	58.5(42.4 to 75.1)	14(10.1 to 18)	89.7(64.8 to 115.5)	12(8.7 to 15.5)	-0.55(-0.59 to -0.52)
Marshall Islands	SAH	53.1(49.8 to 56.7)	209.6(195.1 to 223.5)	100.7(94.9 to 106.2)	211.1(199 to 223.3)	0.03(0.01 to 0.04)	5(4.3 to 5.7)	20.3(17.4 to 23.6)	9(7.9 to 10.2)	20.2(18 to 22.5)	-0.16(-0.24 to -0.08)	9.8(6.8 to 12.7)	38.8(27.7 to 50.2)	18.2(12.8 to 23.7)	38.5(27.3 to 49.8)	-0.03(-0.04 to -0.01)
Mauritania	SAH	1279.2(1177.8 to 1383.3)	100(92.4 to 107.6)	2469.1(2271 to 2688.6)	86.5(80.2 to 93.1)	-0.54(-0.56 to -0.51)	93.5(80.1 to 107.7)	6.4(5.5 to 7.5)	164.9(140.1 to 192.7)	5.2(4.4 to 6.1)	-0.87(-0.93 to -0.81)	169.1(123.2 to 220.4)	13.4(9.8 to 17.4)	328.2(229.1 to 424.8)	11.7(8.2 to 15.1)	-0.5(-0.52 to -0.47)
Mauritius	SAH	1274.7(1180.9 to 1367.6)	142.3(131.7 to 152.6)	2241.3(2091.2 to 2389.8)	131.3(122.5 to 140.1)	-0.2(-0.22 to -0.17)	114.4(100.6 to 129)	12.7(11.3 to 14.4)	171.6(151.7 to 195.4)	10.8(9.6 to 12.1)	-0.77(-0.85 to -0.69)	238.2(168.4 to 311.3)	26.6(18.9 to 34.6)	417.7(300.7 to 535.4)	24.5(17.6 to 31.7)	-0.18(-0.21 to -0.15)
Mexico	SAH	79373(70301.3 to 88488.1)	142.2(126.6 to 159.4)	176786.3(158617.1 to 196213)	133.6(120.3 to 148.4)	-0.21(-0.24 to -0.18)	6016.9(5123 to 6924.2)	9.9(8.5 to 11.6)	12882.7(11193.7 to 14846.7)	9.8(8.6 to 11.2)	-0.03(-0.07 to 0.02)	6919.1(4939.1 to 8998.4)	12.6(9 to 16.3)	15674.8(11331.3 to 20230.9)	12(8.7 to 15.4)	-0.17(-0.2 to -0.15)
Micronesia (Federated States of)	SAH	139.7(131.5 to 148)	213.9(200.7 to 227.7)	188.3(177.4 to 198.7)	206.5(194.1 to 218.2)	-0.13(-0.15 to -0.11)	13.1(11.3 to 14.9)	20.7(18 to 23.8)	16.6(14.6 to 18.7)	19.8(17.7 to 22.1)	-0.28(-0.34 to -0.22)	25.8(18.4 to 33.2)	39.6(28 to 50.6)	34.4(24.5 to 44.6)	38(27.3 to 49.2)	-0.16(-0.18 to -0.14)
Monaco	SAH	49.1(45.5 to 52.9)	94.4(87.3 to 101.5)	54.2(50.6 to 57.7)	79.5(73.9 to 85.2)	-0.61(-0.63 to -0.58)	4(3.5 to 4.6)	7.9(6.8 to 9.1)	3.6(3.1 to 4.2)	5.6(4.8 to 6.4)	-1.29(-1.4 to -1.18)	6.9(5 to 9)	12.9(9.2 to 16.8)	7.6(5.5 to 9.9)	10.8(7.9 to 13.9)	-0.62(-0.65 to -0.59)
Mongolia	SAH	1335.9(1218.9 to 1459.8)	97.4(89.3 to 106.1)	2831(2625.4 to 3059.5)	94(87.2 to 101.5)	-0.15(-0.17 to -0.14)	148.9(128.1 to 169.2)	11.6(10.1 to 13.4)	329.7(289.5 to 374)	13(11.6 to 14.6)	0.34(0.19 to 0.49)	239.5(167.9 to 313.3)	17.5(12.4 to 22.8)	503.5(350.5 to 666.7)	17(12 to 22.1)	-0.1(-0.11 to -0.09)
Montenegro	SAH	415.3(379.7 to 453.4)	64(58.4 to 69.8)	468.1(430.3 to 507)	59.1(54.1 to 64)	-0.3(-0.32 to -0.29)	36.1(30.9 to 41.4)	5.8(5 to 6.6)	39.8(34.9 to 45.1)	5.2(4.6 to 5.9)	-0.33(-0.42 to -0.25)	75.2(52 to 97.8)	11.6(8.1 to 15.1)	84.6(57.9 to 111.7)	10.7(7.4 to 14.1)	-0.29(-0.31 to -0.27)
Morocco	SAH	17939.5(16516.7 to 19504.6)	95.4(88.1 to 103.1)	29061.1(26716.7 to 31461.2)	77(70.7 to 83.3)	-0.8(-0.84 to -0.76)	1657.2(1435.3 to 1880.9)	9(7.9 to 10.4)	2445.2(2129.4 to 2778.3)	6.9(6.1 to 7.8)	-1.09(-1.17 to -1.02)	2525.4(1776.6 to 3206.6)	13.4(9.5 to 17)	4061.6(2832.3 to 5327.7)	10.8(7.6 to 14.1)	-0.78(-0.82 to -0.74)
Mozambique	SAH	7636.4(6968.4 to 8334.7)	100.8(92.4 to 109.3)	15885.8(14602 to 17285.2)	102.1(94.4 to 109.9)	0.05(0.03 to 0.06)	681.9(574 to 803.5)	8.5(7.2 to 10.2)	1445.7(1242.6 to 1674.3)	8.5(7.3 to 10.1)	0.03(0.01 to 0.06)	989.7(704.1 to 1273.5)	13.3(9.6 to 17.1)	2052.9(1468.6 to 2612)	13.5(9.8 to 17.2)	0.05(0.03 to 0.07)
Myanmar	SAH	35435.8(32517.7 to 38359)	121.6(111.8 to 131.9)	53564.1(49834 to 57329.7)	97.6(90.8 to 104.4)	-0.79(-0.82 to -0.75)	3750.2(3268.2 to 4283.6)	13.7(12.1 to 15.8)	4975.6(4419.1 to 5618)	9.5(8.5 to 10.7)	-1.52(-1.68 to -1.35)	6655.5(4565 to 8673.1)	22.9(15.9 to 30)	10031.5(6965.5 to 12968.4)	18.4(12.9 to 23.7)	-0.77(-0.81 to -0.73)
Namibia	SAH	720.9(657.3 to 786.7)	89.1(82 to 95.8)	1342.2(1233.5 to 1460.1)	78.1(72.2 to 84.1)	-0.49(-0.52 to -0.47)	58.2(50 to 66.9)	6.8(5.9 to 7.9)	101.4(87.2 to 117.4)	5.8(5 to 6.7)	-0.77(-0.87 to -0.68)	96(69.2 to 124.1)	12.2(8.8 to 15.7)	179.7(127.7 to 232.4)	10.7(7.8 to 13.7)	-0.47(-0.5 to -0.45)

Nauru	SAH	14.1(13.3 to 15.1)	202.5(189.7 to 217.1)	17(16.1 to 17.8)	204.7(192.6 to 216.2)	0.04(0.01 to 0.06)	1.2(1 to 1.4)	17.2(14.9 to 19.8)	1.5(1.3 to 1.6)	16.1(14.4 to 18)	-0.36(-0.48 to -0.24)	2.6(1.9 to 3.4)	37.5(27 to 48.5)	3.1(2.2 to 4)	37.8(27.1 to 48.5)	0.02(-0.01 to 0.05)
Nepal	SAH	12000.8(10863.5 to 13115.6)	95.2(86.3 to 104)	22404.2(20693.7 to 24275.5)	83.3(76.7 to 90.3)	-0.49(-0.52 to -0.47)	1323.2(1134.3 to 1542.2)	10.9(9.4 to 12.8)	2283.8(1968.8 to 2635.5)	8.9(7.7 to 10.3)	-0.79(-0.87 to -0.72)	1671.1(1179.7 to 2167.9)	13.4(9.5 to 17.4)	3151.7(2215 to 4067.2)	11.8(8.3 to 15.2)	-0.45(-0.47 to -0.42)
Netherlands	SAH	20640(19269.1 to 21951.2)	113.1(105.5 to 120.4)	24714.4(23147.7 to 26276.8)	90.4(84.1 to 96.3)	-0.8(-0.84 to -0.76)	1657.9(1458.9 to 1908.1)	9.3(8.1 to 10.7)	1694(1495.6 to 1948.7)	6.5(5.7 to 7.4)	-1.21(-1.28 to -1.14)	2797.1(2020.4 to 3594.8)	15.2(10.9 to 19.5)	3416(2484.8 to 4331.3)	12.2(8.9 to 15.6)	-0.77(-0.81 to -0.73)
New Zealand	SAH	3495.3(3079.6 to 3982.5)	93.8(82.6 to 106.3)	5037.6(4454.6 to 5618.9)	72(64.1 to 80.3)	-1(-1.1 to -0.91)	340.3(287 to 406.9)	9.3(7.9 to 11.1)	404.1(355.8 to 460.2)	5.8(5.1 to 6.6)	-1.65(-1.82 to -1.49)	501(347.7 to 654.6)	13.4(9.3 to 17.5)	737.7(509.3 to 967.2)	10.4(7.1 to 13.6)	-0.95(-1.03 to -0.87)
Nicaragua	SAH	3289(3058.3 to 3520.8)	160.1(149.8 to 170.3)	7785.8(7278.4 to 8249.2)	139.4(130.4 to 146.9)	-0.51(-0.54 to -0.48)	280.7(244.1 to 315.8)	12.5(11 to 14.3)	573(498 to 651.3)	10(8.8 to 11.5)	-0.81(-0.88 to -0.75)	288.6(209 to 373.8)	14.4(10.4 to 18.7)	686.6(503.9 to 883.4)	12.5(9.2 to 16)	-0.51(-0.53 to -0.48)
Niger	SAH	4289.8(3953.1 to 4671.1)	99.9(93 to 107.3)	11721.5(10778.2 to 12773.5)	91(84.4 to 97.8)	-0.34(-0.36 to -0.33)	341(287.8 to 392.4)	6.4(5.5 to 7.6)	916.6(786.2 to 1048.6)	5.8(5.1 to 6.9)	-0.44(-0.49 to -0.39)	562.4(397.1 to 727)	13.5(9.5 to 17.3)	1539.9(1085.4 to 1993.2)	12.3(8.7 to 15.8)	-0.31(-0.32 to -0.29)
Nigeria	SAH	53671.5(47759.8 to 60177.5)	91.5(81.1 to 102.3)	104735.8(93890.3 to 116830.1)	74.3(66.3 to 82.2)	-0.75(-0.79 to -0.71)	4021.9(3434.7 to 4621.9)	6(5.2 to 7.1)	7451.6(6273.5 to 8603)	4.5(3.8 to 5.2)	-1.3(-1.43 to -1.17)	7019.1(5041.4 to 8979.8)	12.2(8.8 to 15.5)	13904.3(9793.9 to 17858.8)	10.1(7.2 to 12.9)	-0.67(-0.7 to -0.63)
Niue	SAH	4.3(4.1 to 4.6)	203.7(191.7 to 216.5)	3.5(3.3 to 3.6)	173.1(163.6 to 182.3)	-0.58(-0.6 to -0.55)	0.4(0.3 to 0.4)	17.7(15.7 to 20)	0.2(0.2 to 0.3)	13.1(11.6 to 14.7)	-1.2(-1.28 to -1.11)	0.8(0.6 to 1.1)	38.2(26.9 to 49.7)	0.6(0.5 to 0.8)	32.2(23.1 to 41.1)	-0.59(-0.61 to -0.57)
North Macedonia	SAH	2340.3(2167.3 to 2504.3)	113.9(105.3 to 122.1)	3038.6(2827.6 to 3261)	102.4(95.6 to 109.5)	-0.43(-0.46 to -0.4)	230.1(199.3 to 259.8)	12(10.5 to 13.5)	344.8(312.6 to 379.4)	12.9(11.8 to 14.1)	0.14(0.02 to 0.25)	418.5(288.8 to 546.8)	20.4(14.1 to 26.7)	541.4(381.8 to 692)	18.3(12.9 to 23.5)	-0.43(-0.46 to -0.39)
Northern Mariana Islands	SAH	62.7(58.8 to 66.6)	187(176.2 to 198.3)	94.9(89.8 to 99.9)	168.9(160.1 to 178.1)	-0.36(-0.38 to -0.34)	5(4.3 to 5.8)	15.1(13.5 to 17.2)	6.6(5.8 to 7.5)	13(11.7 to 14.3)	-0.57(-0.61 to -0.53)	11.5(7.9 to 15)	34.5(24.3 to 44.2)	17.3(12.1 to 22.4)	31.2(21.6 to 40.4)	-0.36(-0.38 to -0.34)
Norway	SAH	7134.3(6236 to 8005.4)	126.7(111.3 to 143.5)	8416.2(7501.7 to 9332.8)	102.8(92 to 113.3)	-0.75(-0.79 to -0.71)	541.4(462.6 to 637.7)	9.9(8.5 to 11.8)	547.7(477.2 to 629.4)	6.9(6 to 8)	-1.22(-1.36 to -1.07)	967.1(696.5 to 1255.4)	16.8(12 to 21.9)	1151.7(816.4 to 1490.1)	13.8(9.8 to 17.8)	-0.72(-0.76 to -0.67)
Oman	SAH	1523.4(1399.5 to 1663.2)	116.4(107.2 to 125.7)	4145.7(3848.7 to 4458.8)	103.7(97 to 110.2)	-0.43(-0.45 to -0.41)	132(107.9 to 184.3)	10.8(8.9 to 16.2)	322.1(277.5 to 363.8)	11.4(10.2 to 12.7)	0.12(0.06 to 0.19)	209.6(146 to 274.3)	16.1(11.4 to 21)	569(402.4 to 744.4)	14.4(10.2 to 18.6)	-0.41(-0.42 to -0.39)
Pakistan	SAH	76047.5(66581.9 to 86152.4)	108.5(94.5 to 123.9)	168288.1(148165.8 to 189881)	99(87 to 112.6)	-0.3(-0.33 to -0.27)	8638.3(7436.8 to 10088)	12.5(10.7 to 14.8)	17598.2(15071.3 to 20382.7)	10.4(9 to 12)	-0.82(-0.95 to -0.69)	10567.4(7509.8 to 13856.8)	15.2(10.8 to 19.9)	23245.5(15930.8 to 30449.2)	13.8(9.6 to 18.2)	-0.3(-0.33 to -0.27)
Palau	SAH	24.9(23.3 to 26.4)	201.7(189 to 214.1)	43.7(41.4 to 46.2)	184.3(174.3 to 194)	-0.33(-0.34 to -0.31)	2.2(1.9 to 2.4)	18.2(16.2 to 20.6)	3.1(2.7 to 3.6)	14.5(13 to 16.2)	-0.87(-0.93 to -0.81)	4.6(3.2 to 5.9)	37.6(26.2 to 48.7)	7.9(5.6 to 10.1)	33.7(23.8 to 42.9)	-0.39(-0.4 to -0.37)
Palestine	SAH	1044.9(946 to 1144.4)	82.1(75.3 to 89.5)	2914.7(2668.9 to 3169.8)	74.1(68.3 to 80.2)	-0.36(-0.38 to -0.34)	89.7(76.5 to 102.8)	6.6(5.7 to 7.5)	207.7(174.5 to 238.7)	5.3(4.6 to 6)	-0.78(-0.85 to -0.71)	148.3(105.2 to 191.5)	11.6(8.4 to 15)	410.4(289.6 to 532.4)	10.5(7.4 to 13.4)	-0.36(-0.39 to -0.34)
Panama	SAH	3061.7(2878.8 to 3249.9)	174.6(164.8 to 184.3)	7068(6693.8 to 7440)	160.1(151.6 to 168.5)	-0.33(-0.35 to -0.31)	241(211.8 to 271.1)	13.4(11.8 to 15.3)	521.9(462 to 588.1)	11.8(10.5 to 13.3)	-0.46(-0.51 to -0.4)	269.9(197.2 to 347.5)	15.6(11.5 to 20)	637.8(478.7 to 822.1)	14.4(10.8 to 18.6)	-0.31(-0.33 to -0.29)

Papua New Guinea	SAH	3858.2(3565.6 to 4156.3)	142.3(131.5 to 153)	10051.6(9440.1 to 10735.3)	131.2(123 to 139.4)	-0.31(-0.33 to -0.29)	328.2(286.4 to 370.5)	13(11.4 to 15)	805.6(705.5 to 903.2)	11(9.8 to 12.3)	-0.65(-0.7 to -0.6)	704.5(492.1 to 914.6)	26.1(18.5 to 33.8)	1841.6(1301.2 to 2396.9)	24.1(17 to 31.2)	-0.31(-0.33 to -0.29)
Paraguay	SAH	3778.1(3532.4 to 4031.8)	140.7(131.5 to 150.4)	7975.6(7462.5 to 8487.7)	123.6(115.7 to 131.3)	-0.5(-0.52 to -0.47)	365.9(323.7 to 408.5)	12.8(11.3 to 14.4)	711.7(632.9 to 793.8)	10.9(9.7 to 12.1)	-0.46(-0.49 to -0.43)	328.7(244.4 to 428.4)	12.4(9.2 to 16.1)	692.9(501.7 to 881)	10.9(7.9 to 13.8)	-0.49(-0.52 to -0.47)
Peru	SAH	29432(27753.6 to 31080.3)	197.4(186.3 to 208.5)	56390.9(53471 to 59396.4)	158.5(150.2 to 167.1)	-0.82(-0.86 to -0.78)	2454.9(2116.4 to 2762.9)	15.1(13.2 to 17.2)	3772.1(3341.2 to 4235.9)	10.5(9.3 to 11.7)	-1.29(-1.39 to -1.19)	2558.1(1899.5 to 3357.6)	17.5(12.9 to 22.8)	5033.6(3720.5 to 6457)	14.2(10.5 to 18.3)	-0.76(-0.8 to -0.72)
Philippines	SAH	36763.3(32534.1 to 41369.9)	86.5(76.3 to 96.8)	105545(94047.3 to 118045.1)	106.6(94.7 to 119.7)	0.82(0.75 to 0.88)	3260.6(2740.4 to 3773.8)	7.7(6.6 to 8.9)	9471.2(8154.8 to 10901.3)	9.6(8.4 to 11)	1.17(0.96 to 1.37)	6900.4(4757.5 to 8992)	16.2(11.3 to 21)	19695(13764 to 25445.6)	20(14 to 25.8)	0.83(0.77 to 0.9)
Poland	SAH	41318.9(36327.2 to 46931.1)	98.5(86.6 to 111.5)	36765.8(32367.5 to 41420)	70(61.8 to 78.8)	-1.27(-1.35 to -1.19)	4090.9(3490.2 to 4765.9)	9.9(8.5 to 11.6)	3318.5(2910.5 to 3855.5)	6.2(5.4 to 7.1)	-1.97(-2.14 to -1.8)	7348.8(5172.7 to 9561.2)	17.5(12.3 to 22.8)	6686.1(4669.6 to 8675.2)	12.7(8.8 to 16.6)	-1.2(-1.28 to -1.12)
Portugal	SAH	11824.9(10998.3 to 12763.7)	93.5(86.7 to 100.9)	12043.1(11150.3 to 12964)	69.3(63.6 to 75.2)	-1.15(-1.21 to -1.09)	1004.5(887.2 to 1152.1)	8.3(7.3 to 9.5)	1092.2(944.4 to 1235.1)	5.6(4.8 to 6.4)	-1.51(-1.62 to -1.4)	1575.4(1119 to 2038.3)	12.4(8.8 to 16.2)	1701.4(1215.8 to 2237.4)	9.5(6.6 to 12.4)	-1.05(-1.11 to -1)
Puerto Rico	SAH	5280.8(4974.1 to 5586.7)	146.1(137.6 to 154.6)	6510.1(6154.8 to 6859.3)	126.9(120 to 133.7)	-0.53(-0.56 to -0.49)	348.1(303.1 to 392.4)	9.8(8.5 to 11)	368.8(321.4 to 421.3)	7.8(6.8 to 8.9)	-0.89(-0.99 to -0.8)	472.3(340.3 to 616.1)	13.1(9.4 to 17)	615.6(453.7 to 783.8)	11.4(8.3 to 14.5)	-0.53(-0.57 to -0.49)
Qatar	SAH	378.8(351.8 to 410)	113.1(105.5 to 121)	2751.1(2565.2 to 2944)	100.8(94.4 to 107.2)	-0.46(-0.49 to -0.42)	30.1(24.9 to 35.2)	8.8(7.8 to 10)	187.7(151.7 to 227.4)	7.8(6.9 to 8.7)	-0.37(-0.41 to -0.33)	51.8(36.1 to 67.6)	15.7(11.2 to 20.2)	373.3(263.6 to 486.5)	13.9(10.1 to 17.7)	-0.48(-0.51 to -0.44)
Republic of Korea	SAH	57294(52961.4 to 61427.6)	169.6(156.2 to 182.6)	111725.3(105687.9 to 117850.1)	127.8(120.9 to 135.4)	-0.89(-1.08 to -0.7)	6988.7(6035.9 to 8206.6)	19.8(17.2 to 23.3)	8825.6(7695.5 to 10359.9)	10.6(9.3 to 12.3)	-2.12(-2.5 to -1.74)	9225.7(6630.9 to 11801.7)	27.7(20.1 to 35.6)	18346.8(13066.8 to 23757.3)	21(15 to 27.2)	-0.85(-1.03 to -0.67)
Republic of Moldova	SAH	2939.6(2688.7 to 3223.5)	64(58.8 to 70.1)	2831.6(2604.7 to 3073.8)	57.2(52.7 to 62)	-0.38(-0.41 to -0.35)	312.8(271 to 354.1)	7.1(6.2 to 8)	272.7(237.2 to 308.7)	5.7(4.9 to 6.4)	-0.88(-0.97 to -0.79)	530.6(368.2 to 691.5)	11.6(8.1 to 15)	514.6(356 to 674.5)	10.4(7.2 to 13.6)	-0.36(-0.4 to -0.33)
Romania	SAH	20859.1(19057 to 22796.7)	79(72.2 to 86.6)	18663.4(17066.8 to 20133.8)	71(64.5 to 77.3)	-0.44(-0.49 to -0.39)	2529(2272.7 to 2804.3)	10.1(9.1 to 11.2)	3152.6(2901.6 to 3424.4)	9.7(8.8 to 10.4)	-0.3(-0.36 to -0.24)	3736.8(2598.1 to 4892.8)	14.2(9.8 to 18.5)	3396.6(2366.9 to 4382)	12.9(9 to 16.8)	-0.41(-0.45 to -0.36)
Russian Federation	SAH	161406.5(141440.4 to 184304)	93.2(82 to 106)	175732.3(154517.1 to 199748.6)	88.5(78.1 to 99.7)	-0.2(-0.25 to -0.14)	16601(14181.6 to 19472.9)	9.9(8.5 to 11.5)	20253.2(17894.6 to 23245.5)	10.1(8.9 to 11.5)	-0.19(-0.55 to 0.18)	28673.5(20216.1 to 37473.2)	16.6(11.7 to 21.6)	31446.3(22235.5 to 41151.4)	15.8(11.1 to 20.7)	-0.17(-0.23 to -0.12)
Rwanda	SAH	4640.1(4270.7 to 5036.7)	127.3(117.2 to 137.3)	7894.9(7320.9 to 8566.5)	94.6(88.2 to 101.5)	-1.07(-1.12 to -1.03)	423.1(357.8 to 487.4)	10.6(9 to 12.6)	639.9(543.5 to 738)	7.3(6.3 to 8.6)	-1.65(-1.8 to -1.5)	603.8(435.6 to 769.8)	17(12.4 to 21.5)	1035.3(742.9 to 1345.7)	12.7(9.2 to 16.4)	-1.01(-1.06 to -0.97)
Saint Kitts and Nevis	SAH	58.9(54.9 to 62.9)	165.8(155.4 to 176.9)	92.2(86.8 to 97.8)	129(121.5 to 137.1)	-0.93(-0.98 to -0.89)	5(4.5 to 5.6)	14.2(12.7 to 16)	6.9(6 to 7.8)	10(8.9 to 11.2)	-1.37(-1.48 to -1.25)	5.3(3.8 to 6.7)	14.6(10.7 to 18.9)	7.9(5.9 to 10.2)	11.4(8.4 to 14.6)	-0.94(-0.98 to -0.9)
Saint Lucia	SAH	154.2(144.3 to 163.9)	154.6(144.8 to 163.9)	300(283.3 to 316.6)	132.7(125.1 to 139.9)	-0.55(-0.58 to -0.52)	12.7(11.3 to 14.3)	12.6(11.1 to 14.4)	21(18.6 to 23.9)	9.6(8.6 to 10.8)	-1.04(-1.13 to -0.95)	13.6(10 to 17.3)	13.7(10.1 to 17.6)	26.4(19.2 to 33.9)	11.7(8.5 to 15.1)	-0.56(-0.59 to -0.54)
Saint Vincent and the Grenadines	SAH	110.6(102.6 to 118.6)	136.4(126.8 to 145.4)	155.4(146.3 to 165.1)	115.4(108.3 to 122.5)	-0.62(-0.65 to -0.58)	8.9(7.8 to 9.9)	10.7(9.5 to 12)	10.8(9.6 to 12.2)	8.3(7.4 to 9.2)	-0.9(-1.02 to -0.78)	9.8(7.2 to 12.6)	12.2(9 to 15.6)	13.5(9.9 to 17.8)	10.1(7.4 to 13.3)	-0.67(-0.7 to -0.64)

Samoa	SAH	214.6(201.8 to 228.1)	194(182.2 to 206.3)	321.4(304.6 to 338.2)	189.6(179.1 to 200.1)	-0.08(-0.08 to -0.07)	18.7(16.5 to 21)	17.3(15.3 to 19.7)	26.2(23.5 to 29.6)	15.4(13.8 to 17.4)	-0.46(-0.5 to -0.42)	39.9(28.9 to 50.8)	36.1(26.2 to 46.2)	59.4(42.1 to 76.7)	35.2(25.2 to 45.3)	-0.09(-0.1 to -0.09)
San Marino	SAH	28(25.9 to 30.2)	90.9(84.1 to 98.1)	45.6(42.3 to 48.9)	82.5(76.6 to 88.9)	-0.34(-0.37 to -0.31)	2(1.7 to 2.3)	6.9(5.8 to 8)	2.9(2.5 to 3.4)	5.6(4.8 to 6.6)	-0.79(-0.86 to -0.71)	3.8(2.7 to 4.9)	12.2(8.8 to 15.8)	6.3(4.5 to 8.2)	11.1(7.9 to 14.3)	-0.34(-0.38 to -0.31)
Sao Tome and Principe	SAH	82.9(76.3 to 89.2)	107.1(99.3 to 114.8)	154.1(143.1 to 166.1)	100.8(93.7 to 107.6)	-0.22(-0.24 to -0.2)	5.6(4.8 to 6.5)	6.4(5.4 to 7.5)	10.1(8.6 to 11.8)	6(5.1 to 7)	-0.37(-0.42 to -0.31)	11.1(7.9 to 14.1)	14.5(10.4 to 18.6)	20.2(14.3 to 26.2)	13.5(9.7 to 17.5)	-0.24(-0.26 to -0.22)
Saudi Arabia	SAH	7226.6(6554.2 to 7962.9)	66.1(60 to 72.4)	22026(20098.6 to 24046.9)	58.7(53.6 to 63.7)	-0.4(-0.45 to -0.36)	639.2(539.8 to 729.6)	5.9(5.1 to 6.6)	1604.3(1316.6 to 1891.5)	4.6(4 to 5.2)	-0.92(-0.99 to -0.85)	1020.7(709.7 to 1334.8)	9.4(6.6 to 12.2)	3094.2(2189.2 to 4075)	8.3(5.9 to 10.9)	-0.38(-0.42 to -0.34)
Senegal	SAH	4142.1(3793.3 to 4522.8)	93(86.1 to 100.8)	8843.6(8136.7 to 9572)	84.9(79.1 to 91.2)	-0.33(-0.35 to -0.31)	311.1(264.3 to 354.8)	5.9(5 to 6.8)	595.5(506.4 to 691.3)	5.1(4.4 to 6)	-0.5(-0.55 to -0.46)	545.6(389.9 to 703.7)	12.5(8.9 to 15.9)	1170.4(846.4 to 1515.3)	11.5(8.4 to 14.8)	-0.3(-0.32 to -0.28)
Serbia	SAH	11484.2(10594.8 to 12446.4)	101.4(93.4 to 109.4)	9111.5(8404.2 to 9834.2)	75.7(69.9 to 81.4)	-1.11(-1.18 to -1.05)	1259.4(1116.2 to 1397.6)	12.1(10.9 to 13.3)	1373.2(1258.1 to 1495.4)	9.9(9 to 10.7)	-1.04(-1.18 to -0.91)	2056.5(1402.3 to 2681.7)	18.2(12.4 to 23.6)	1642.2(1168.7 to 2118.4)	13.6(9.6 to 17.6)	-1.11(-1.17 to -1.04)
Seychelles	SAH	66.9(61.7 to 72.4)	110.5(102 to 119.3)	113.5(105.4 to 121.8)	93.1(86.2 to 100.2)	-0.56(-0.6 to -0.52)	6.1(5.2 to 6.9)	10(8.7 to 11.4)	8.4(7.2 to 9.6)	7.2(6.3 to 8.1)	-1.2(-1.29 to -1.1)	12.7(8.8 to 16.5)	20.9(14.5 to 27.1)	21(14.9 to 27.6)	17.3(12.2 to 22.5)	-0.59(-0.64 to -0.55)
Sierra Leone	SAH	2534.1(2320.7 to 2739.8)	95(87.6 to 102.4)	4965.8(4578.2 to 5400.5)	90.1(83.9 to 96.5)	-0.19(-0.2 to -0.17)	185.4(157.4 to 213.6)	6(5.1 to 7)	351.6(299.3 to 399.7)	5.6(4.8 to 6.6)	-0.26(-0.31 to -0.21)	333.2(235 to 433.2)	12.7(9 to 16.4)	656.4(470.3 to 841.6)	12.2(8.7 to 15.5)	-0.16(-0.18 to -0.14)
Singapore	SAH	4206.6(3934.3 to 4477.9)	169.3(158.3 to 179.6)	10869(10231.9 to 11512.9)	130.5(122.7 to 138.2)	-0.86(-0.91 to -0.81)	354.5(295 to 412.7)	13.1(11.2 to 15.4)	713.6(597.7 to 859.5)	8.7(7.4 to 10.3)	-1.63(-1.75 to -1.52)	682.8(482.2 to 894.7)	28(19.7 to 36.4)	1782.3(1252.5 to 2293.9)	21.5(15.1 to 27.6)	-0.86(-0.9 to -0.81)
Slovakia	SAH	4982.8(4628.1 to 5331.6)	87.1(81 to 93.3)	4992.2(4624.4 to 5400.2)	67.2(62 to 72.7)	-0.89(-0.92 to -0.85)	498.6(442.9 to 553.7)	8.9(7.9 to 9.8)	409.3(361 to 459.3)	5.6(4.9 to 6.3)	-1.96(-2.1 to -1.81)	895.5(620.9 to 1155.9)	15.6(10.9 to 20.1)	909.2(632.6 to 1188.7)	12.2(8.5 to 16)	-0.84(-0.88 to -0.8)
Slovenia	SAH	1858.2(1714 to 2004.6)	80.8(74.4 to 87.3)	1943(1802.9 to 2097.1)	65.6(60.8 to 70.8)	-0.7(-0.72 to -0.67)	166.1(146 to 187.5)	7.4(6.5 to 8.3)	162.5(143 to 186.1)	5.3(4.5 to 6)	-1.5(-1.63 to -1.37)	333.2(236.4 to 433.8)	14.5(10.3 to 18.9)	354.3(245.1 to 465.4)	11.9(8.2 to 15.5)	-0.67(-0.7 to -0.64)
Solomon Islands	SAH	434.5(404.3 to 462.2)	213.6(197.9 to 229.3)	1109.6(1049.2 to 1165.3)	227(213.7 to 240.2)	0.21(0.17 to 0.24)	38.7(31.6 to 48.6)	20(16.4 to 26)	112.8(99.9 to 127.7)	24.2(21.6 to 27.1)	0.38(0.26 to 0.5)	80.8(57.7 to 105.3)	40(28.3 to 51.8)	205.1(145.5 to 265.2)	42.4(30.4 to 54.5)	0.21(0.17 to 0.24)
Somalia	SAH	4310.8(3946.7 to 4712.5)	115.7(106.1 to 126.6)	10958.5(10076.8 to 11851.1)	112.5(103.9 to 120.7)	-0.1(-0.11 to -0.08)	392.7(327 to 465.4)	9.3(7.8 to 11.1)	1001.6(849.8 to 1170.7)	9(7.7 to 10.8)	-0.14(-0.17 to -0.1)	550.7(396.3 to 705.3)	15.2(10.9 to 19.3)	1402.4(1015.7 to 1781.7)	14.9(10.8 to 18.7)	-0.08(-0.09 to -0.07)
South Africa	SAH	19569(17270 to 22001.4)	78.3(68.9 to 88.6)	33948.6(30322.9 to 37814.8)	66.1(59.1 to 73.6)	-0.63(-0.67 to -0.6)	1495(1274.5 to 1729.6)	5.6(4.8 to 6.5)	2390.1(2039.2 to 2797.7)	4.6(4 to 5.4)	-0.78(-0.92 to -0.64)	2642.8(1876.8 to 3377.4)	10.8(7.7 to 13.8)	4572.9(3258.5 to 5874.3)	9(6.5 to 11.6)	-0.64(-0.68 to -0.61)
South Sudan	SAH	3132.2(2848.8 to 3412.5)	97.9(89.7 to 105.7)	4807.3(4429 to 5218.7)	89.4(83.2 to 96.1)	-0.33(-0.35 to -0.31)	267.6(229.7 to 307.3)	7.7(6.5 to 9.1)	399.9(339.7 to 461.1)	6.8(5.9 to 8)	-0.46(-0.54 to -0.38)	399.9(294 to 511.3)	12.7(9.4 to 16.2)	615.1(440.9 to 787.3)	11.6(8.5 to 14.6)	-0.29(-0.3 to -0.27)
Spain	SAH	40034(37139.7 to 43063.8)	82.6(76.5 to 89.4)	61131.6(57345.4 to 65113.4)	81.1(75.7 to 87)	-0.08(-0.11 to -0.05)	3049.7(2638.5 to 3520.7)	6.6(5.6 to 7.6)	4623.6(4083.8 to 5223.3)	6.1(5.3 to 6.9)	-0.29(-0.4 to -0.18)	5409.2(3858.8 to 7087.1)	11.1(7.9 to 14.5)	8585.8(6053.2 to 11148.8)	11(7.8 to 14.3)	-0.04(-0.07 to -0.01)

Sri Lanka	SAH	18132.2(16811.2 to 19463.4)	134.6(124.9 to 144.1)	29339.7(27502.2 to 31106.8)	112.3(105.2 to 119.1)	-0.67(-0.71 to -0.63)	1779.2(1550.4 to 2026)	14.9(13.2 to 16.9)	2615.5(2304.2 to 2954.9)	10.7(9.4 to 12)	-1.28(-1.4 to -1.17)	3392.6(2356.7 to 4476.8)	25.3(17.6 to 33.4)	5527.6(3944.8 to 7200.3)	21.2(15.1 to 27.6)	-0.65(-0.68 to -0.61)
Sudan	SAH	12029.6(11011.2 to 13186.3)	89.5(82.2 to 97.4)	21634.6(19687.7 to 23837.4)	68.7(63.2 to 74.6)	-0.99(-1.04 to -0.94)	1227.4(1070.7 to 1401.2)	9.3(8.1 to 10.9)	1851.3(1607.5 to 2098.6)	6.4(5.6 to 7.2)	-1.57(-1.69 to -1.45)	1683.4(1182.9 to 2185.7)	12.5(8.9 to 16.2)	3068(2168.3 to 3983.6)	9.7(6.9 to 12.7)	-0.94(-0.99 to -0.89)
Suriname	SAH	447.2(416.1 to 477.2)	146.1(136.1 to 155.1)	829.6(780.7 to 877.2)	130.8(122.9 to 138.6)	-0.41(-0.43 to -0.39)	36.2(32 to 40.5)	11.5(10.3 to 13)	64.2(57 to 72.8)	10.3(9.2 to 11.5)	-0.52(-0.57 to -0.47)	38.7(28.7 to 50.3)	12.8(9.6 to 16.7)	72.1(53.1 to 92.3)	11.5(8.4 to 14.7)	-0.4(-0.43 to -0.38)
Sweden	SAH	13709.7(12008.9 to 15619.7)	111.8(98.7 to 126)	14664.4(13015.3 to 16327.4)	89.5(80 to 99.1)	-0.81(-0.85 to -0.76)	1083(928.4 to 1294.1)	9.2(7.9 to 10.9)	1038.1(894.9 to 1202.4)	6.5(5.6 to 7.4)	-1.34(-1.44 to -1.24)	1905.8(1360 to 2458.4)	15.2(10.8 to 19.5)	2080.5(1491.7 to 2689.9)	12.2(8.8 to 15.8)	-0.8(-0.85 to -0.75)
Switzerland	SAH	7370.1(6799.3 to 7919.7)	82.2(75.5 to 88.6)	10472.9(9749.8 to 11168.4)	74.7(69.3 to 80.4)	-0.37(-0.44 to -0.3)	666.3(586.3 to 759.4)	7.5(6.5 to 8.5)	765.5(666.8 to 891.1)	5.5(4.7 to 6.4)	-0.93(-1.2 to -0.66)	995.7(707.5 to 1288.6)	11(7.7 to 14.2)	1447.7(1040.1 to 1869.1)	10.1(7.2 to 13)	-0.31(-0.38 to -0.24)
Syrian Arab Republic	SAH	10563.9(9824.6 to 11360.4)	118.1(109.7 to 126.4)	14352.4(13469.1 to 15255.9)	98.7(92.8 to 105)	-0.63(-0.68 to -0.58)	858.1(697 to 1265.3)	8.7(7.4 to 10.8)	879.7(767.1 to 986.2)	6.7(6 to 7.5)	-1.25(-1.37 to -1.13)	1471.2(1046.4 to 1900.9)	16.4(11.7 to 21.1)	1981.2(1421.2 to 2568.7)	13.8(9.9 to 17.8)	-0.6(-0.64 to -0.56)
Taiwan (Province of China)	SAH	21595.5(20065 to 23314.8)	112.9(104.6 to 121.9)	36836.3(34163.6 to 39654.4)	108.9(101.5 to 116.2)	-0.14(-0.15 to -0.13)	1814.1(1525.7 to 2111.1)	10.4(8.8 to 12.1)	3028.1(2678.9 to 3453.4)	8.9(7.9 to 10)	-0.4(-0.47 to -0.34)	4177.8(2897.6 to 5397.1)	21.9(15.3 to 28.4)	7163.9(5142.4 to 9121.3)	21.1(14.9 to 26.9)	-0.14(-0.16 to -0.13)
Tajikistan	SAH	3171.9(2899.4 to 3438.9)	93.1(85.7 to 101.1)	7154.5(6618.8 to 7685.6)	89.7(82.7 to 96.5)	-0.18(-0.19 to -0.16)	330.8(286.4 to 369.6)	9.8(8.6 to 11.1)	761(667.8 to 854.2)	11.2(10 to 12.4)	0.54(0.44 to 0.63)	575.5(403.8 to 731.9)	16.9(11.8 to 21.5)	1282.6(895.7 to 1713.2)	16.2(11.2 to 21.6)	-0.17(-0.19 to -0.16)
Thailand	SAH	68372.7(63781.7 to 73205.9)	154.6(143.7 to 165.6)	124120.5(116043.5 to 131461.6)	126.1(117.8 to 133.8)	-0.73(-0.77 to -0.7)	7587.6(6639.5 to 8605.9)	19.1(16.9 to 21.5)	12269.7(10909 to 14011.3)	12.6(11.3 to 14.3)	-1.69(-1.83 to -1.54)	13041.9(9145.2 to 16909.8)	29.9(21.1 to 38.7)	24027.2(16863.8 to 31303.1)	24.4(17.2 to 31.7)	-0.72(-0.75 to -0.69)
Timor-Leste	SAH	468.7(430.4 to 514.8)	101.5(94 to 109.8)	983.5(903.1 to 1061.5)	96.7(89.6 to 103.8)	-0.17(-0.19 to -0.15)	47(40.3 to 53.8)	10.6(9.2 to 12.1)	92.6(80.6 to 104.8)	9.4(8.3 to 10.7)	-0.51(-0.58 to -0.43)	87(61.2 to 112.7)	18.8(13.4 to 24.4)	182.9(129.2 to 237.5)	18(12.7 to 23.2)	-0.16(-0.18 to -0.14)
Togo	SAH	1851.4(1695.5 to 2021.6)	94.2(87.5 to 101.7)	4794.6(4417.4 to 5207.2)	86.9(80.8 to 93.3)	-0.3(-0.31 to -0.28)	143(121.1 to 165.5)	6(5.1 to 7)	335(288.5 to 387)	5.5(4.7 to 6.4)	-0.4(-0.44 to -0.37)	243.4(173.2 to 316.9)	12.7(9.2 to 16.4)	631.3(437.7 to 812.9)	11.8(8.3 to 14.9)	-0.27(-0.29 to -0.25)
Tokelau	SAH	2.8(2.6 to 2.9)	204.8(192.4 to 216.1)	2.5(2.4 to 2.7)	175.5(165.5 to 185.7)	-0.53(-0.56 to -0.51)	0.2(0.2 to 0.3)	18.2(15.9 to 20.9)	0.2(0.2 to 0.2)	13.5(12 to 15.2)	-1.2(-1.29 to -1.12)	0.5(0.4 to 0.7)	38.6(26.9 to 49.7)	0.5(0.3 to 0.6)	32.6(23.1 to 42)	-0.57(-0.59 to -0.54)
Tonga	SAH	103.8(97.1 to 110.6)	149.1(139.6 to 158.9)	119.8(113.3 to 126.6)	134.7(127.4 to 142.2)	-0.37(-0.38 to -0.35)	8.4(7.3 to 9.5)	12.2(10.8 to 13.8)	9.1(8.1 to 10.1)	10(8.9 to 11.3)	-0.76(-0.82 to -0.7)	19.5(14 to 25.1)	28.2(20.3 to 36.2)	22.3(15.6 to 28.8)	25.1(17.6 to 32.4)	-0.39(-0.41 to -0.37)
Trinidad and Tobago	SAH	1453.4(1358.9 to 1545.2)	148.6(139.1 to 158.2)	2214.3(2079.5 to 2336.2)	125.5(117.6 to 132.7)	-0.63(-0.66 to -0.6)	118.6(105.3 to 132.9)	12.1(10.8 to 13.6)	166.6(148.9 to 189.4)	9.8(8.8 to 11)	-0.8(-0.85 to -0.76)	126.8(92.3 to 163.9)	13.1(9.5 to 17)	194.8(142.7 to 252.1)	11.1(8.1 to 14.3)	-0.63(-0.66 to -0.6)
Tunisia	SAH	5872.7(5388.9 to 6388.6)	91.3(84.3 to 99)	10295.9(9529.2 to 11100.4)	77.3(71.3 to 83.5)	-0.61(-0.65 to -0.57)	510.2(441.1 to 579.7)	8.3(7.2 to 9.5)	767.9(664.3 to 875.1)	6(5.3 to 6.8)	-1.26(-1.34 to -1.17)	830.6(596.9 to 1092.2)	13(9.4 to 17)	1457.2(1031.8 to 1890.9)	11(7.8 to 14.2)	-0.59(-0.64 to -0.55)
Turkmenistan	SAH	55547.7(51527.1 to 59521.9)	123.9(115.2 to 132.4)	83114(77362.4 to 89138.4)	88.2(82.1 to 94.8)	-1.27(-1.34 to -1.2)	4917.5(4225.8 to 5595.6)	11.3(9.7 to 13)	6169.6(5446.6 to 6954.5)	6.8(6.1 to 7.6)	-1.97(-2.11 to -1.83)	7844.7(5465.4 to 10250.3)	17.6(12.2 to 23)	11794.9(8347 to 15022.8)	12.6(8.9 to 16)	-1.24(-1.31 to -1.17)

Tuvalu	SAH	2219.6(2041.9 to 2410.4)	89.7(83.1 to 97.1)	5225.1(4886.5 to 5587.8)	106.4(99.2 to 113.8)	0.62(0.6 to 0.65)	234(204.3 to 262.7)	9.7(8.6 to 10.9)	563.9(503.3 to 631.1)	12.1(10.9 to 13.5)	1.04(0.93 to 1.15)	400.5(278.3 to 515.9)	16.2(11.4 to 20.9)	919.9(651 to 1195.8)	18.9(13.4 to 24.5)	0.6(0.57 to 0.63)
T 眉 rkiye	SAH	16.2(15.1 to 17.3)	207.2(192.9 to 222.1)	21.3(20.1 to 22.6)	187.4(177 to 199)	-0.36(-0.38 to -0.34)	1.5(1.3 to 1.7)	20.2(17.7 to 23.3)	1.8(1.6 to 2.1)	16.9(15.1 to 19)	-0.78(-0.84 to -0.71)	3(2.1 to 3.9)	39(27.7 to 50.4)	3.9(2.8 to 5.1)	34.8(24.9 to 45)	-0.39(-0.41 to -0.37)
Uganda	SAH	8212.8(7445.7 to 9030.3)	96.7(88.8 to 105.1)	18736.4(17236.2 to 20550.7)	87.5(81.2 to 94.1)	-0.36(-0.38 to -0.35)	729.2(625.4 to 844.8)	7.9(6.7 to 9.4)	1607.5(1384.2 to 1857.6)	6.8(5.8 to 8.1)	-0.63(-0.7 to -0.56)	1068.4(762.6 to 1374.8)	12.8(9.3 to 16.3)	2459.2(1742.7 to 3157.8)	11.8(8.4 to 15.2)	-0.31(-0.32 to -0.3)
Ukraine	SAH	51484.7(45289.2 to 58804.5)	80.4(70.9 to 91.4)	50928.1(44857.5 to 57600.5)	83(73.3 to 93.4)	0.15(0.13 to 0.17)	6350.9(5413.4 to 7472.9)	10.1(8.7 to 11.7)	5676.2(5004.1 to 6503.6)	9.2(8.1 to 10.5)	-0.33(-0.4 to -0.26)	9331.5(6369.2 to 12201.6)	14.6(9.9 to 19.1)	9070.3(6362 to 12077.6)	14.7(10.4 to 19.4)	0.11(0.09 to 0.13)
United Arab Emirates	SAH	1172.6(1065.6 to 1296.1)	86.1(79.1 to 93.9)	7891.8(7244.4 to 8569.2)	70.9(65.2 to 76.6)	-0.68(-0.7 to -0.65)	94.1(77.3 to 110.4)	7.7(6.7 to 8.7)	542.2(427.1 to 668.3)	5.7(5 to 6.4)	-1.2(-1.31 to -1.09)	162.3(110.9 to 211.4)	12(8.5 to 15.3)	1072.5(749.6 to 1403.2)	9.9(7 to 12.8)	-0.69(-0.71 to -0.66)
United Kingdom	SAH	83290.3(74056.5 to 93013.8)	110.5(98.7 to 123.6)	88688.3(79594.2 to 97587.7)	88.4(79.3 to 97.8)	-0.83(-0.87 to -0.78)	7178.7(6229.8 to 8437.7)	9.8(8.5 to 11.5)	7198.5(6369.1 to 8166.7)	7.1(6.2 to 8.1)	-1.35(-1.47 to -1.22)	11309.7(8096.6 to 14527.5)	14.8(10.5 to 19)	12225(8767.8 to 15711.6)	11.9(8.5 to 15.3)	-0.8(-0.84 to -0.76)
United Republic of Tanzania	SAH	13997(12826.6 to 15258.7)	101.8(93.7 to 109.9)	31041.7(28725.7 to 33529)	91.3(85.1 to 97.8)	-0.39(-0.41 to -0.37)	1213.8(1033 to 1416.3)	8.1(6.9 to 9.7)	2552.7(2192.5 to 2944.6)	6.9(5.9 to 8.1)	-0.68(-0.73 to -0.62)	1823(1300.4 to 2352.8)	13.5(9.7 to 17.4)	4066(2874.3 to 5175.9)	12.2(8.7 to 15.5)	-0.34(-0.36 to -0.32)
United States of America	SAH	266333.7(236589.4 to 301773.1)	92(81.6 to 104.2)	454302.2(409929.6 to 503031.5)	97.6(88.4 to 108.1)	0.16(0.05 to 0.27)	19875.1(17051.7 to 23439.6)	6.9(5.9 to 8.1)	29757.8(26203.9 to 34291)	6.3(5.5 to 7.2)	-0.48(-0.67 to -0.29)	38199.2(27207 to 49585.4)	13.1(9.2 to 17.1)	64028.7(45348.6 to 81978.1)	13.7(9.6 to 17.5)	0.1(-0.01 to 0.22)
United States Virgin Islands	SAH	155(145 to 164)	159.1(149 to 168.2)	185(173.3 to 195.3)	135.2(127.1 to 142.7)	-0.6(-0.62 to -0.57)	12.1(10.6 to 13.8)	12.2(10.7 to 13.8)	12.8(11.3 to 14.5)	9.8(8.8 to 11)	-0.82(-0.88 to -0.76)	13.5(9.9 to 17.5)	14.2(10.4 to 18.4)	16.8(12.3 to 21.8)	12(8.8 to 15.6)	-0.59(-0.62 to -0.57)
Uruguay	SAH	6347.1(5894.6 to 6819.3)	180(167.7 to 192.8)	6556.9(6029.1 to 7130.5)	145(135.3 to 155.8)	-0.64(-0.71 to -0.58)	611.3(548.4 to 696.8)	17.9(16.1 to 20.4)	608.6(548.1 to 679.3)	13.4(12 to 15)	-1.06(-1.13 to -0.98)	896.8(647.9 to 1157.8)	25.3(18.2 to 32.6)	936.4(657.1 to 1195.8)	20.4(14.4 to 26.1)	-0.6(-0.67 to -0.52)
Uzbekistan	SAH	10100.2(9254.1 to 11042.8)	70.5(64.8 to 77)	22853.7(21188.9 to 24489.5)	70.1(65 to 75.1)	-0.05(-0.07 to -0.03)	1001.1(838.7 to 1148.5)	6.7(5.7 to 7.7)	2063(1793.1 to 2334.9)	6.9(6.1 to 7.7)	0.18(0.11 to 0.26)	1833.7(1289.6 to 2403.8)	12.8(9 to 16.9)	4089.1(2938.7 to 5383.2)	12.6(9 to 16.7)	-0.04(-0.06 to -0.01)
Vanuatu	SAH	175.8(164.5 to 187.1)	187.8(174.8 to 200.5)	460.2(435.6 to 484.8)	196(185.4 to 207.7)	0.16(0.14 to 0.17)	15.7(13.6 to 17.8)	16.8(14.7 to 19.1)	40.1(35.5 to 45)	17.1(15.3 to 19.2)	0.01(-0.02 to 0.05)	32.4(22.7 to 42)	34.8(24.3 to 44.9)	84.6(58.7 to 110.3)	36.3(25.5 to 47.1)	0.15(0.14 to 0.17)
Venezuela (Bolivarian Republic of)	SAH	22666.8(21196.7 to 24144.8)	184.6(173.4 to 195.7)	51018.2(48400.7 to 53608.2)	170(161.2 to 178.8)	-0.32(-0.35 to -0.29)	1878.1(1658.2 to 2106.5)	13.9(12.3 to 15.7)	3643(3244.5 to 4223.4)	12.3(11 to 14.1)	-0.47(-0.56 to -0.38)	1961.1(1425.7 to 2479.7)	16.2(11.8 to 20.5)	4484(3205.3 to 5790.7)	15.1(10.8 to 19.4)	-0.28(-0.3 to -0.25)
Viet Nam	SAH	58900.1(54589 to 63565.7)	123.7(114.7 to 133)	116554.5(109076.1 to 124410.6)	107.4(100.4 to 114.7)	-0.53(-0.57 to -0.48)	6004.5(5164.3 to 6832.1)	13(11.3 to 14.9)	10871(9514.8 to 12535.7)	10.5(9.3 to 11.9)	-0.91(-0.99 to -0.82)	11223.4(7638.2 to 14639.3)	23.6(16 to 30.5)	21967.7(15401 to 28802.4)	20.4(14.3 to 26.5)	-0.52(-0.56 to -0.48)
Yemen	SAH	7105.6(6462.3 to 7774.5)	89(81.7 to 96.8)	17481.8(15899.4 to 19256.8)	75.8(69.6 to 82.5)	-0.61(-0.66 to -0.57)	729(635.2 to 832.6)	9.3(8.1 to 10.8)	1615.2(1419 to 1807.1)	7.7(6.8 to 8.7)	-0.86(-0.95 to -0.77)	997(700.7 to 1302.2)	12.5(9 to 16.4)	2467.1(1726.6 to 3213.7)	10.7(7.5 to 14.1)	-0.59(-0.63 to -0.54)
Zambia	SAH	3922.5(3583.2 to 4278.9)	102.2(94.3 to 110.9)	9655.7(8844.8 to 10453)	95.3(88.7 to 102.1)	-0.26(-0.27 to -0.25)	346.8(297.8 to 399)	8.3(7.1 to 9.7)	842.9(718.6 to 972)	7.7(6.6 to 9)	-0.35(-0.41 to -0.3)	509.8(363.9 to 651.4)	13.6(9.8 to 17.2)	1248.7(876.8 to 1582.9)	12.7(9.1 to 16)	-0.23(-0.24 to -0.22)

Zimbabwe	SAH	5289.7(4800.3 to 5777.2)	95.4(87.6 to 103)	10516.8(9792.8 to 11248.7)	111.6(104.7 to 118.4)	0.57(0.53 to 0.6)	407.8(348.8 to 468.9)	6.8(5.9 to 8)	865.1(754.1 to 985.6)	8.5(7.4 to 9.7)	0.98(0.84 to 1.11)	698.6(501.1 to 882.5)	12.9(9.3 to 16.4)	1370.3(998.1 to 1755.7)	15(11.1 to 19.1)	0.56(0.53 to 0.6)
Afghanistan	IS	63701.4(59332.2 to 68245.5)	855.3(791.8 to 918.4)	117238.9(111184.4 to 123129.2)	822(773.8 to 869.8)	-0.11(-0.12 to -0.09)	8249.3(7193.8 to 9492.6)	127.9(112.5 to 146.5)	13464.9(11936.5 to 15296.4)	119.1(107.1 to 135.4)	-0.23(-0.29 to -0.18)	8492.8(6270.7 to 10803)	115.1(84.8 to 145.8)	15479.1(11457 to 19736.8)	109(81.1 to 138.1)	-0.15(-0.16 to -0.13)
Albania	IS	14662.9(13862.9 to 15438.5)	646.1(607.8 to 680.3)	23506(22181.7 to 24759.2)	576.6(543.9 to 606.3)	-0.38(-0.42 to -0.35)	1560.2(1348.6 to 1789.9)	75.2(65.2 to 86.4)	2715.7(2339.1 to 3155.4)	66.8(58.6 to 76.7)	-0.28(-0.4 to -0.16)	2637.4(1881.8 to 3333.9)	117.3(84.4 to 147.8)	4272.4(3091.6 to 5492.4)	104.6(75.6 to 133.9)	-0.38(-0.42 to -0.34)
Algeria	IS	148258.8(140039.6 to 157093.2)	1027.6(968.9 to 1090.8)	369745.7(354425 to 385886.6)	982.9(939.4 to 1028.1)	-0.13(-0.15 to -0.12)	16095(13919.7 to 18485.1)	135.7(119.3 to 155.9)	40525.1(34841 to 46588.5)	117.7(100.3 to 135)	-0.68(-0.82 to -0.55)	20255.8(14858.4 to 25627.7)	142.4(105.7 to 181.3)	50947.2(37308.3 to 64144.1)	137.3(100.9 to 173)	-0.1(-0.12 to -0.09)
American Samoa	IS	250.2(238 to 263.7)	1001.4(947.2 to 1054.8)	398.2(382.1 to 415.9)	853.1(818.1 to 891.4)	-0.58(-0.61 to -0.55)	21.7(18.8 to 25.2)	96.4(82.2 to 111.4)	35.3(30.8 to 41)	80.1(69.9 to 92.2)	-0.78(-0.84 to -0.73)	46.6(32.9 to 59.6)	189.8(136.8 to 241.3)	73.3(52.8 to 92.5)	158.9(114.9 to 200.8)	-0.63(-0.65 to -0.61)
Andorra	IS	351.8(330.9 to 373.9)	613.4(578.3 to 651.7)	697(660.8 to 733.5)	478.9(453.5 to 504.4)	-0.93(-0.98 to -0.88)	36(30.2 to 43)	65.1(55 to 77.2)	69.1(58.6 to 82.9)	44.8(38.3 to 53.7)	-1.44(-1.58 to -1.31)	47.8(34.5 to 61.2)	84.9(61.7 to 108.8)	97.8(71.3 to 122.8)	66.2(47.9 to 83.8)	-0.94(-0.98 to -0.89)
Angola	IS	52352.1(49719.8 to 55124.7)	1254(1189.3 to 1322.5)	148898.6(142446 to 156124)	1164.2(1111.7 to 1224.7)	-0.27(-0.28 to -0.26)	5206.9(4457.3 to 6034.5)	134.7(115.4 to 155.8)	14134.3(12326.2 to 16327.5)	119.6(103.2 to 137.6)	-0.38(-0.4 to -0.36)	6817(5047.7 to 8574.5)	171.5(127 to 215.2)	19596.3(14436.1 to 24386.2)	161.5(120.4 to 201)	-0.21(-0.22 to -0.2)
Antigua and Barbuda	IS	367.6(347.5 to 390.9)	673.8(637.6 to 714.8)	613.5(583.3 to 642.8)	582.4(552.5 to 610.2)	-0.54(-0.57 to -0.51)	46.3(40.4 to 52.5)	82.5(71.8 to 93.8)	64.6(56.5 to 72.7)	66.3(58.7 to 74.1)	-0.83(-0.92 to -0.73)	34.1(25.5 to 43.1)	61.3(46 to 77.3)	54(40.3 to 68.9)	52.4(39.2 to 66.7)	-0.58(-0.61 to -0.55)
Argentina	IS	272419.5(258693 to 286702.9)	844.7(802.4 to 889.5)	354764(339411.5 to 370886.9)	643.1(615.6 to 671.1)	-0.95(-1 to -0.91)	29885(25982.4 to 34220.2)	95.1(82.9 to 108.2)	33272.6(29391.1 to 37720.1)	60(53.2 to 68)	-1.74(-1.89 to -1.59)	39150.4(28384.9 to 49533.8)	122.6(88.9 to 155.1)	51874.2(37608.9 to 65118.6)	93.3(67.6 to 117.1)	-0.94(-0.98 to -0.9)
Armenia	IS	27504.6(26040.9 to 28906.1)	965.1(912.9 to 1015.8)	34442.8(32917.6 to 35969.7)	827(790.4 to 863.1)	-0.52(-0.57 to -0.47)	3517.9(3077.3 to 4026.3)	134.8(117.6 to 152.8)	3606.1(3213 to 4011.3)	86.1(76.9 to 95)	-1.64(-1.77 to -1.52)	4976(3551.5 to 6272.9)	176.2(125.9 to 222)	6341.9(4532.4 to 8010.2)	151.8(108.6 to 192.1)	-0.49(-0.54 to -0.43)
Australia	IS	148512.1(143711.8 to 153960.5)	759.6(735.3 to 787.6)	252325.7(244095.4 to 260214.5)	586.3(567.2 to 604.8)	-0.94(-0.99 to -0.9)	17582.4(16333.8 to 18840.3)	91.7(85.6 to 98)	23830(21335.5 to 26509.6)	53(47.7 to 58.8)	-2.1(-2.25 to -1.94)	21427.5(15697.1 to 27172.2)	109.9(80.6 to 139)	37656.4(27829 to 47171.9)	85.3(63.1 to 106.9)	-0.96(-1.01 to -0.91)
Austria	IS	86637.6(83705.1 to 89754.1)	745.2(717.5 to 772.7)	153609.4(148085.5 to 159504.6)	861.2(828.5 to 893.6)	0.64(0.5 to 0.77)	11608.4(10592.6 to 12695)	95.8(87.9 to 104.1)	13175.3(11499.4 to 15430.1)	72.5(64 to 84.2)	-0.6(-0.91 to -0.29)	12310.4(9113.5 to 15583.8)	103.9(76.8 to 131.1)	22896.8(16689.5 to 28748.4)	123.1(89.1 to 155.3)	0.74(0.6 to 0.88)
Azerbaijan	IS	41839.2(39278.5 to 44505.5)	784.6(733.3 to 837.4)	81841(77847.3 to 85845.3)	786.8(747.6 to 830.2)	0.03(-0.02 to 0.09)	4606(3982 to 5302.9)	91.1(79.3 to 104.8)	9015.3(7861 to 10287.8)	98.7(86 to 112.6)	0.65(0.52 to 0.78)	7662.6(5483.6 to 9816.9)	145.2(104.4 to 186.3)	14858.5(10645 to 19018.8)	145(104.3 to 184.4)	0.03(-0.03 to 0.09)
Bahamas	IS	1172.8(1114.4 to 1233.1)	683.3(649.9 to 719.2)	2513.4(2398.2 to 2624.8)	614.4(586.1 to 641.5)	-0.38(-0.4 to -0.36)	118.6(103.7 to 135.8)	76.2(66.5 to 87.5)	240.5(210.5 to 269.4)	63.2(55.5 to 70)	-0.65(-0.7 to -0.6)	103.9(76.3 to 132.1)	62.2(45.8 to 78.8)	219.5(163.9 to 277.2)	55.2(41.2 to 69.8)	-0.42(-0.44 to -0.4)
Bahrain	IS	1965.2(1837.1 to 2099.1)	656.9(619.8 to 697.7)	6883.5(6514.9 to 7326.1)	540.4(511.2 to 573.5)	-0.72(-0.77 to -0.68)	156(132.8 to 182)	79.5(70.5 to 89.2)	492.5(422.4 to 574)	56.5(49.7 to 63.5)	-1.15(-1.21 to -1.1)	261.6(188.5 to 335.6)	89(65 to 112.6)	896.8(666.5 to 1136.3)	72.7(54.4 to 91.4)	-0.74(-0.78 to -0.69)
Bangladesh	IS	330600.6(310024.5 to 351869.3)	620.4(580 to 665.8)	865770.5(820023.9 to 914545.1)	601.5(568 to 638.2)	-0.07(-0.13 to -0.01)	39039.7(33424.1 to 44847.2)	84.2(72.5 to 96.5)	107065.9(92201.3 to 123094.7)	83.6(72.3 to 95.6)	-0.03(-0.13 to 0.08)	46498.7(33961.7 to 59089)	89(65.2 to 113.4)	124318.4(91160.6 to 158475.7)	87.7(64.6 to 111.6)	-0.02(-0.08 to 0.05)
Barbados	IS	2025.3(1894.1 to 2160.6)	711.1(665.9 to 757.1)	3004.3(2848 to 3158.1)	630.8(599.8 to 661.8)	-0.42(-0.46 to -0.37)	268.2(235.5 to 305.7)	90.8(80.1 to 102.5)	344.6(299.9 to 389)	70.3(61.6 to 79)	-0.83(-0.97 to -0.7)	191.1(142.3 to 244)	65.2(48.6 to 83)	275.3(204.1 to 350.7)	57.2(42.4 to 73)	-0.45(-0.5 to -0.41)
Belarus	IS	139881.6(125696.7 to 152451.9)	1086.1(977.1 to 1182.9)	151094.5(142723.6 to 160614.5)	990.3(937.1 to 1047.3)	-0.44(-0.58 to -0.3)	23265.5(20087 to 26739.5)	183.5(159.2 to 208.6)	22186.7(19277.2 to 25304.7)	140.6(122.4 to 159.4)	-0.9(-1.08 to -0.73)	25372.2(18395.9 to 33167.9)	197.2(142.5 to 257.1)	27451.3(19848.5 to 34728.2)	179.4(130.2 to 227.1)	-0.43(-0.57 to -0.3)
Belgium	IS	98149.1(91379.4 to 105337.5)	661(616.2 to 706.5)	123049.9(119043.6 to 126841.2)	555.4(535.8 to 573)	-0.56(-0.66 to -0.47)	15115.5(12938.3 to 17384.4)	99(85.7 to 112.6)	12981.5(11652.2 to 14236)	54(48.3 to 58.8)	-1.76(-2.07 to -1.45)	13593.3(9921.8 to 17316.2)	90.4(66.2 to 115.1)	17820.4(13232.6 to 22235.3)	77.2(57.1 to 96.4)	-0.49(-0.6 to -0.38)
Belize	IS	556.3(521.4 to 591.9)	522.8(493.4 to 555.9)	1631(1549.3 to 1715.6)	499.8(473.7 to 526.2)	-0.18(-0.21 to -0.15)	59(50.8 to 67.2)	58(50.1 to 65.8)	157.4(138.1 to 178)	52.7(46.3 to 59)	-0.63(-0.74 to -0.51)	49.6(36.4 to 62.8)	47.1(34.6 to 59.5)	140.9(104.9 to 179.8)	44.4(33.1 to 56.4)	-0.23(-0.26 to -0.2)
Benin	IS	26736.2(25376.9 to 28204.7)	1164.7(1101.1 to 1228.8)	63632.9(60510.9 to 66667.8)	1018.4(972.3 to 1068.2)	-0.52(-0.55 to -0.48)	2482.3(2130.1 to 2842.4)	111.9(97 to 129.6)	5458.7(4771 to 6227.2)	93.8(82 to 108.2)	-0.67(-0.72 to -0.61)	3587(2639.1 to 4483.3)	160.7(118.6 to 200.5)	8550.1(6253.9 to 10766.3)	143.1(106.2 to 180.9)	-0.46(-0.5 to -0.43)
Bermuda	IS	441.6(417.7 to 468.6)	703.9(665.5 to 747.8)	671.7(639.1 to 704.6)	553.3(525.5 to 580.7)	-0.87(-0.92 to -0.82)	48.7(42.7 to 55.1)	81.9(72 to 92.6)	67.4(58 to 76.7)	51.5(44.6 to 57.9)	-1.81(-1.92 to -1.69)	40.1(29.7 to 50.9)	64.8(48.1 to 82.6)	63.9(47.7 to 80.6)	50.8(37.7 to 64.3)	-0.88(-0.93 to -0.84)
Bhutan	IS	1611.1(1497.1 to 1716.9)	554.3(514.1 to 593.4)	3424.8(3231.5 to 3616.6)	538.3(506.3 to 569.7)	-0.12(-0.14 to -0.11)	172(145.3 to 200.9)	71.6(60.9 to 84.1)	389.9(337.9 to 445.6)	65.5(57 to 74.4)	-0.41(-0.47 to -0.35)	226.1(163.7 to 287.4)	80.6(58.8 to 103.2)	498.4(356 to 635.7)	79.4(57 to 101.4)	-0.07(-0.09 to -0.06)
Bolivia (Plurinational State of)	IS	21307.5(20082.4 to 22651.1)	586.1(550.7 to 623.6)	45241.3(42796.1 to 47846.2)	471.7(445.4 to 499)	-0.81(-0.86 to -0.76)	2142.9(1844.6 to 2479.1)	66.2(56.9 to 76.8)	4367.6(3717.4 to 5066.6)	51(43.5 to 58.8)	-1.01(-1.12 to -0.9)	1861.5(1361.9 to 2360.1)	53.1(38.7 to 67.2)	3980.5(2952.6 to 5025.1)	42.9(31.9 to 54.1)	-0.79(-0.83 to -0.75)
Bosnia and Herzegovina	IS	48842.7(46108.4 to 51528.9)	1140.3(1073.8 to 1205.1)	63409.2(60465.2 to 66821.3)	1110.7(1062.2 to 1164.7)	-0.08(-0.1 to -0.05)	6505.5(5695.3 to 7536.3)	169.1(149 to 193.2)	8674.3(7494.8 to 9801.5)	145.2(126.8 to 164)	-0.38(-0.45 to -0.31)	8691.1(6281.3 to 11040.2)	204.8(147.9 to 261.5)	11312.9(8240.1 to 14364.8)	197.3(144.2 to 250.9)	-0.11(-0.14 to -0.09)
Botswana	IS	7521.5(7078.1 to 7926.2)	1325.1(1246.9 to 1408.4)	19280.3(18418.9 to 20182.9)	1339.1(1275.7 to 1408.7)	0.02(-0.01 to 0.05)	799.7(680.5 to 937.6)	154.5(131.2 to 181.7)	2134.9(1833.3 to 2451.9)	163.1(140.4 to 187.1)	0.05(-0.07 to 0.16)	1020.4(753.9 to 1286.3)	188.6(139.7 to 235.6)	2608.5(1912.3 to 3250.1)	190(139.8 to 235.6)	0.03(0 to 0.06)
Brazil	IS	811042.4(723673 to 900218.2)	853.3(759.5 to 948.5)	1507566.8(1357108.4 to 1655969.3)	605.7(545.7 to 664.7)	-1.25(-1.32 to -1.19)	104035.9(86380.6 to 123697.8)	120(98.8 to 143.4)	162711(137407.1 to 190295.3)	66.3(55.8 to 77.6)	-2.07(-2.19 to -1.95)	67730(50772 to 87126.8)	73.5(54.7 to 93.4)	131449.5(97471.5 to 167377.5)	53.3(39.5 to 68)	-1.17(-1.23 to -1.11)
Brunei Darussalam	IS	1743(1653 to 1828.8)	1612.3(1530.1 to 1698.9)	3372.4(3222 to 3525.6)	954.5(914.2 to 999.3)	-1.88(-1.97 to -1.8)	186.3(161 to 215.6)	170.6(147.1 to 198.2)	317.6(271 to 367.7)	94.6(82.4 to 108.6)	-2.28(-2.46 to -2.1)	276.5(198.8 to 353.2)	261(188.7 to 333.1)	525.9(382.9 to 666.7)	153.9(113.6 to 194)	-1.89(-1.97 to -1.8)
Bulgaria	IS	129984.5(118741.9 to 143136)	1059.6(970.6 to 1161.1)	137218.8(124624.7 to 150670)	1031.3(945 to 1115.8)	-0.08(-0.1 to -0.05)	19355.8(16585.4 to 22494.5)	174.3(153.5 to 197.7)	23740.9(20664 to 26747.5)	168.9(149.2 to 188.3)	0.02(-0.04 to 0.08)	23086(17017.6 to 29904.2)	189.3(139.7 to 245.1)	24663.9(18081.8 to 31460.5)	184(135.5 to 234.6)	-0.07(-0.09 to -0.04)
Burkina Faso	IS	41466(39064.8 to 43773.2)	846.7(801.1 to 895.7)	86464.8(82444 to 90431.9)	776.3(741.6 to 811.4)	-0.33(-0.35 to -0.31)	3458.4(2937.6 to 4065.5)	73(63.3 to 84.1)	7183.9(6205.9 to 8257.8)	68.1(59.6 to 77.3)	-0.19(-0.24 to -0.15)	5513.2(4065.1 to 6907.5)	116.7(86.7 to 146)	11627.5(8605.8 to 14508.5)	108.8(81.2 to 135.5)	-0.27(-0.28 to -0.25)
Burundi	IS	32413.1(30583.6 to 34304.3)	1287.4(1206.8 to 1365.2)	53274.3(50745.5 to 55845.8)	957.7(910.9 to 1008.2)	-1.06(-1.11 to -1.02)	3411.6(2958.6 to 3955.7)	141.8(121.9 to 164.7)	5159.7(4468.3 to 5997.5)	101.9(87.6 to 117.2)	-1.32(-1.42 to -1.23)	4340.5(3214.8 to 5431.8)	177.7(131.3 to 222.4)	7034(5167.5 to 8831.6)	132.7(98.4 to 165.3)	-1.03(-1.08 to -0.99)
Cabo Verde	IS	2464.2(2335.5 to 2587.9)	1019.3(963.6 to 1068.5)	4779.9(4589.2 to 4996.1)	1011.8(970 to 1055.6)	-0.04(-0.05 to -0.03)	192.7(165.9 to 220.3)	78.2(67.3 to 90)	385.8(337.3 to 445.3)	84.7(73.9 to 97.7)	0.26(0.2 to 0.31)	350.5(258.8 to 439.1)	144.6(106.5 to 181)	663.2(492.1 to 825.2)	143.3(106.5 to 178.3)	-0.04(-0.05 to -0.02)
Cambodia	IS	35827.5(33749.6 to 37983.7)	716.1(670.8 to 763.3)	92092.3(87597.4 to 96723.4)	713.1(673 to 753.9)	-0.02(-0.05 to 0.01)	4143(3547.1 to 4807.6)	99(84.6 to 116.2)	11184.7(9612.2 to 12803.7)	101(86.8 to 115.5)	0.05(-0.02 to 0.12)	6682(4750.3 to 8585.5)	134.9(97 to 173.7)	17290.8(12381.3 to 22099.3)	135.4(97.8 to 172.6)	0.02(-0.01 to 0.05)
Cameroon	IS	52024.2(49264.2 to 54813.7)	1025.7(968 to 1082.6)	154862.1(148046.6 to 161887)	1015.7(967.8 to 1064.8)	-0.05(-0.09 to 0)	4256.6(3657.7 to 4950.1)	89.3(76.1 to 104.5)	12997.9(11469.9 to 14822.7)	92.8(81.3 to 105.6)	0.12(-0.03 to 0.26)	6928.1(5129.6 to 8594.5)	142.1(106.2 to 176.7)	20567.3(15308.8 to 25808.5)	141.8(106.1 to 177.5)	-0.02(-0.06 to 0.02)
Canada	IS	302810.3(290985.3 to 313751)	946.6(909.9 to 981)	562109.5(548506.2 to 574789.5)	847.2(825.7 to 869.4)	-0.32(-0.37 to -0.27)	32537.4(28186.3 to 37205.3)	101.9(88.4 to 116.4)	41314.6(37976.5 to 44826.4)	61.5(56.6 to 66.4)	-1.4(-1.54 to -1.25)	42937.9(31691.8 to 54435.4)	134.3(99.2 to 170)	81227.5(59411.3 to 102221.2)	120(87.3 to 151.6)	-0.3(-0.35 to -0.25)
Central African Republic	IS	12795.2(12087 to 13509.6)	1055.5(993 to 1120.8)	23617(22430.2 to 24842)	967.6(912 to 1026.1)	-0.32(-0.34 to -0.3)	1312.9(1114.1 to 1565.8)	118.6(100.3 to 140.9)	2378.4(2033 to 2760.5)	110.9(94.6 to 127.6)	-0.24(-0.26 to -0.22)	1648.5(1204.6 to 2086.8)	142.8(105.8 to 179.2)	3039.7(2248.8 to 3837.6)	131.4(98.4 to 165.9)	-0.29(-0.31 to -0.28)
Chad	IS	34750.9(32898.5 to 36650.6)	1094.5(1036.3 to 1153.9)	75881.4(72357.1 to 78949.6)	1039.2(992.1 to 1084.3)	-0.19(-0.2 to -0.18)	3072.3(2655.5 to 3554.8)	99.2(85.6 to 114.4)	6547.6(5692.5 to 7465)	94.8(82.4 to 106.8)	-0.11(-0.16 to -0.06)	4674.3(3412.6 to 5799.2)	151.2(111.8 to 187.4)	9993.1(7398.8 to 12655.3)	143.1(106.6 to 180.8)	-0.19(-0.2 to -0.18)
Chile	IS	82346.8(78408.2 to 86449.3)	793.9(754.1 to 834.6)	155201.5(148079.5 to 162378.5)	623.5(594.8 to 652.3)	-0.9(-0.97 to -0.84)	9364(8192.3 to 10710.1)	93.9(82.3 to 107.1)	14875.1(12895.3 to 17007.3)	59.1(51.5 to 67.4)	-1.54(-1.69 to -1.38)	11635.6(8544.3 to 14748.7)	113.7(83.1 to 144)	22382.2(16332.7 to 28410)	89.5(65.2 to 113.7)	-0.9(-0.96 to -0.84)
China	IS	6577204.6(5875417.3 to 7262376.3)	759.2(675.2 to 850.3)	20803931.7(18615866.6 to 22995491.3)	1018.8(918.5 to 1123.4)	1.02(0.96 to 1.08)	761191.4(621353.8 to 937712.2)	100(81.5 to 120.9)	2772053.3(2295713.4 to 3319150.1)	135.8(113.3 to 159.8)	0.94(0.88 to 1)	1261607.4(896627.2 to 1622747.7)	147.6(104.9 to 189.2)	3972276.2(2812535 to 5155558.2)	195.6(138.8 to 252.5)	1(0.93 to 1.06)
Colombia	IS	143540.1(136675.6 to 150776.2)	750.2(715.9 to 785.7)	273597.3(261970.3 to 286798.5)	496.9(475.5 to 520.2)	-1.53(-1.61 to -1.45)	15082.6(13148.7 to 17087.4)	84.8(73.8 to 95.3)	25862.1(22398.6 to 29347.8)	46.3(40.2 to 52.5)	-2.28(-2.45 to -2.1)	12615.3(9467.3 to 15887.5)	68.1(51.3 to 85.4)	25512.1(19224.3 to 32302.6)	46.1(34.7 to 58.4)	-1.45(-1.53 to -1.38)
Comoros	IS	2750(2607.7 to 2905)	1322.7(1252 to 1398.8)	5561(5306.8 to 5826.5)	1110(1058.5 to 1162.2)	-0.63(-0.66 to -0.6)	267.6(229.9 to 309.7)	134.4(115 to 156.8)	530.7(459.8 to 605.2)	111.2(96.2 to 127)	-0.75(-0.84 to -0.67)	365.8(275.8 to 454.7)	182.7(137.1 to 227.1)	754.9(558.1 to 945.7)	155.2(115.5 to 194.1)	-0.57(-0.59 to -0.55)
Congo	IS	15835.3(15015.5 to 16635.9)	1424.1(1345.6 to 1499)	34965.1(33299.8 to 36550.3)	1225.3(1168.7 to 1285.4)	-0.55(-0.57 to -0.53)	1593.9(1370.7 to 1868.3)	152.2(130.4 to 177.4)	3397.2(2933.5 to 3894.2)	129.9(112.9 to 148.7)	-0.61(-0.66 to -0.56)	2089.4(1528.9 to 2637.2)	196.1(145.2 to 246.2)	4609.2(3385.1 to 5763.8)	169.3(125.6 to 211.7)	-0.53(-0.55 to -0.51)
Cook Islands	IS	116.9(111.3 to 122.9)	894.5(850.7 to 942.1)	223.1(213.9 to 231.9)	915.7(879.1 to 950.6)	0.06(0.04 to 0.08)	10.4(8.9 to 11.9)	84.5(72.4 to 96.3)	19.3(16.8 to 22.1)	78.6(69.1 to 89.7)	-0.38(-0.45 to -0.3)	21.8(15.8 to 28)	168.8(122.4 to 215.6)	41.7(30.2 to 52.7)	171.5(123.8 to 217.5)	0.05(0.02 to 0.07)
Costa Rica	IS	12454.2(11757.8 to 13149.2)	644.9(611.9 to 678.6)	29272.8(27851.6 to 30793)	538.4(513.2 to 566)	-0.68(-0.72 to -0.64)	1258.1(1087.5 to 1445.9)	69.2(60 to 79.9)	2723.5(2342.6 to 3137.9)	49.6(42.5 to 57.1)	-1.31(-1.46 to -1.16)	1124.2(827.3 to 1432.2)	59.3(43.9 to 75.4)	2703.4(2020.7 to 3408.2)	49.6(37 to 62.4)	-0.68(-0.72 to -0.64)

Croatia	IS	66739.2(63322.3 to 70126.7)	1304.6(1229.9 to 1378.5)	152892.5(146002.2 to 159948.1)	1109.4(1060.8 to 1157.8)	-0.61(-0.63 to -0.58)	5486.6(4761.7 to 6299.3)	115.4(99.9 to 132.8)	12507.4(10916.8 to 14192)	99(85.6 to 112.6)	-0.65(-0.73 to -0.56)	8480.7(6278.4 to 10645.4)	174.7(129.5 to 215.4)	20067.3(14701 to 25244)	153.6(113.4 to 192.3)	-0.51(-0.54 to -0.48)
Cuba	IS	52071.2(48224.6 to 56110.7)	874.1(804.7 to 943.3)	64356.7(62235.6 to 66261)	741.9(718.4 to 763.6)	-0.52(-0.57 to -0.47)	9225(8491.7 to 9966.9)	162.7(149.3 to 175.7)	9244.7(8638.5 to 9821.2)	99.9(93.8 to 105.9)	-1.56(-1.63 to -1.49)	9286.6(6735.8 to 12042.3)	156.7(113.1 to 203.6)	11747.2(8639.8 to 14766.1)	134.2(98.5 to 169.5)	-0.49(-0.55 to -0.43)
Cyprus	IS	66010.4(62529.2 to 69644.5)	630.1(597.6 to 665.2)	104405.8(99313.9 to 109629)	577.6(549.3 to 606.1)	-0.29(-0.32 to -0.27)	7246.6(6352.1 to 8188.4)	72(63.1 to 81.4)	11976(10625.5 to 13407.9)	62.7(55.8 to 69.8)	-0.4(-0.43 to -0.37)	5929.5(4417.4 to 7530.3)	56.7(42.4 to 72)	9535.2(7128.2 to 11986.6)	51.9(39 to 65.4)	-0.29(-0.31 to -0.27)
Czechia	IS	4639(4345 to 4970.4)	561.1(524.8 to 602.6)	6093.8(5641.3 to 6733.5)	312.3(288.4 to 346.1)	-2.3(-2.5 to -2.1)	686.9(569.3 to 808.7)	86.8(73.6 to 100.3)	780.9(688.7 to 878.6)	41.8(37.1 to 46.8)	-3.17(-3.65 to -2.7)	632.1(455.9 to 814.9)	77.9(56.7 to 99.9)	835.1(601.8 to 1058.6)	42.7(30.5 to 54.1)	-2.4(-2.62 to -2.17)
C 么 te d'Ivoire	IS	141880.9(131207.5 to 153274.9)	1042(968.2 to 1125.2)	161271(154894.9 to 168608.5)	779.1(749 to 811)	-0.89(-0.93 to -0.85)	25448.2(21824.1 to 29039.8)	188.4(163.2 to 213.5)	19392.4(16683.6 to 22281)	89.6(78.8 to 101.8)	-2.56(-2.64 to -2.48)	25419.7(18343.9 to 32462.9)	186.6(134.8 to 238.6)	29602.7(21553.4 to 37266.2)	141.8(102.9 to 179.6)	-0.84(-0.88 to -0.8)
Democratic People's Republic of Korea	IS	172358.9(162988.6 to 182711.8)	1087.4(1023.8 to 1153.1)	346744.2(330062.1 to 365846.9)	1079.6(1026.2 to 1142)	0.04(0.01 to 0.06)	19771.7(16992.5 to 23121.4)	132.9(114.6 to 154.5)	43908.8(38064.6 to 50459.2)	138.1(119.8 to 157.8)	0.06(-0.01 to 0.12)	33494.2(23955.2 to 42969.9)	215(154.8 to 275.2)	67744.3(48173.7 to 87235.7)	212.3(151.9 to 273.3)	0.03(0.01 to 0.05)
Democratic Republic of the Congo	IS	175723.1(166073.5 to 185967)	1044.7(980.6 to 1112.4)	369928.7(351658.8 to 388420.6)	920(869.3 to 972.5)	-0.46(-0.49 to -0.43)	18017.2(15203 to 21285.9)	117(98.9 to 136.8)	37504.4(32504 to 43158.8)	103.6(89.5 to 121.1)	-0.44(-0.47 to -0.4)	22788.8(16904.9 to 28779.2)	141.4(104.5 to 177.9)	48628.2(36188 to 61280.1)	127.1(95.2 to 161.9)	-0.38(-0.41 to -0.36)
Denmark	IS	71021.4(67852.5 to 73867.4)	884.2(846 to 918)	61099.4(58487.6 to 63609.1)	547.8(524.6 to 570.4)	-1.64(-1.72 to -1.56)	8705.4(7749.6 to 9747.5)	106.9(96.2 to 118)	6056.2(5211.6 to 6933.6)	51.9(44.9 to 59.2)	-2.71(-2.81 to -2.6)	10097.2(7387.6 to 12726.1)	123.1(89.7 to 154.9)	8945.9(6563.8 to 11331.7)	77.5(56.9 to 98.1)	-1.58(-1.66 to -1.5)
Djibouti	IS	1947.2(1835 to 2064.3)	1281(1210.9 to 1351)	8253.4(7880.5 to 8619.8)	1225.6(1168.8 to 1287.1)	-0.16(-0.18 to -0.13)	181.3(153.5 to 212.3)	127.8(108.7 to 148)	777.7(675.1 to 897.8)	125.3(107.1 to 144.8)	-0.07(-0.11 to -0.03)	254.9(187.1 to 319.3)	177.7(132.4 to 221.6)	1083.4(793.8 to 1347.3)	170.1(126.3 to 209.5)	-0.15(-0.18 to -0.13)
Dominica	IS	362.7(341.3 to 385.6)	595.6(561.9 to 632.7)	439.1(417.6 to 461.6)	537.8(511.6 to 565.5)	-0.38(-0.4 to -0.36)	40.9(35.2 to 47.3)	69.2(59.9 to 79.5)	49.7(42.5 to 57)	63.6(54.8 to 71.9)	-0.27(-0.3 to -0.23)	33.1(24.3 to 42.4)	54.2(39.9 to 69.2)	38.8(29.2 to 49)	48.1(36.2 to 60.8)	-0.44(-0.46 to -0.42)
Dominican Republic	IS	27919.1(26416.3 to 29591.1)	657.8(622.6 to 697.9)	75673.7(72191.2 to 79237.1)	740.9(706.5 to 777.9)	0.45(0.42 to 0.47)	2531.2(2209.2 to 2896.8)	64.9(56.7 to 73.5)	7958.2(6951.4 to 9041.9)	79.3(68.9 to 90.1)	0.73(0.7 to 0.76)	2436.4(1815.8 to 3105.3)	59(43.9 to 75.4)	6654.6(4930.7 to 8437.5)	65.7(48.6 to 83.7)	0.41(0.39 to 0.44)
Ecuador	IS	41592.8(39349.4 to 43718.5)	703.9(665.4 to 738.8)	97527.8(92741.7 to 101861.5)	583(554.5 to 609.3)	-0.68(-0.71 to -0.65)	3875.8(3379 to 4374.9)	69.1(60.2 to 77.7)	8683.4(7647.6 to 9803.8)	53.8(47.7 to 60.5)	-0.84(-0.89 to -0.8)	3686.7(2738.8 to 4645.7)	64.1(47.8 to 81)	8751.3(6509.4 to 11083.6)	52.9(39.3 to 67)	-0.7(-0.73 to -0.67)
Egypt	IS	273254(254591.7 to 293398.1)	801.9(740.7 to 863.4)	737060.4(702530.8 to 773597.5)	975.3(921.5 to 1031.2)	0.67(0.65 to 0.69)	30076.7(26448 to 34628.9)	114.1(99.5 to 130.6)	83090(73098.3 to 95678.3)	132.1(117.8 to 151)	0.56(0.47 to 0.65)	37238.1(27094.7 to 47755.7)	110.4(80.1 to 141.9)	98470.3(72001.9 to 125255.6)	131.9(96.5 to 168)	0.61(0.59 to 0.64)
El Salvador	IS	18298.1(17345.1 to 19364.1)	561.3(531.5 to 592.9)	29410.3(27945.2 to 30946.5)	464.7(440.8 to 489.3)	-0.7(-0.75 to -0.66)	1897.8(1653.4 to 2172.7)	60.5(52.2 to 69.2)	3005.6(2630.7 to 3409)	45.6(40 to 51.9)	-1.13(-1.26 to -1)	1657(1249.5 to 2089)	51.7(39 to 65.2)	2758.2(2042.4 to 3499.4)	42.9(31.8 to 54.4)	-0.68(-0.72 to -0.64)
Equatorial Guinea	IS	2397.7(2268.1 to 2524.9)	1198.9(1128.9 to 1271.3)	6168.3(5879.7 to 6497.2)	1060.2(1012.9 to 1114.1)	-0.47(-0.52 to -0.42)	256.4(218.9 to 302.5)	135.9(116.1 to 158.7)	549.6(469.8 to 636.6)	105.4(89 to 120.9)	-0.86(-0.91 to -0.82)	312.7(234.1 to 393.9)	162.4(122.2 to 203.8)	810(608.7 to 1020.2)	147.1(109.6 to 183.3)	-0.38(-0.44 to -0.33)
Eritrea	IS	14176.6(13404.4 to 15021.4)	1156.6(1089.4 to 1227.4)	32508.6(30979.2 to 34009.4)	1074.6(1020.6 to 1125.3)	-0.28(-0.3 to -0.27)	1414(1206.8 to 1673.3)	129.8(110.5 to 150.6)	3074.4(2661.1 to 3597.3)	110.5(94.9 to 128.2)	-0.64(-0.7 to -0.58)	1846.3(1368.6 to 2297)	160.1(119.3 to 198.8)	4291.2(3174.4 to 5342.1)	149.8(110.1 to 186)	-0.25(-0.26 to -0.23)
Estonia	IS	17917.5(16343 to 19504)	890.4(814.3 to 966.9)	15107.8(14187.9 to 16052.3)	640.7(603.7 to 675.5)	-1.22(-1.33 to -1.1)	3383.5(3055.5 to 3759.8)	170(154.2 to 187.8)	1888.5(1639.9 to 2158.7)	71.5(62.1 to 81.8)	-3.2(-3.42 to -2.97)	3204(2340.7 to 4040.9)	159.1(116 to 201.2)	2786.2(2014.9 to 3565.8)	116.5(84 to 148.6)	-1.16(-1.28 to -1.03)
Eswatini	IS	3218.9(3032.4 to 3404.5)	1050.6(983.9 to 1118.3)	6050(5763.3 to 6353.6)	1064.5(1009.6 to 1122.1)	0.01(-0.01 to 0.04)	341(290 to 397.8)	124.1(106 to 146.7)	662.2(565.9 to 760.5)	138(118.9 to 157.7)	0.41(0.27 to 0.55)	438.5(323.5 to 551.6)	150.2(109.5 to 186.8)	805(598.5 to 1005)	149.8(111.3 to 185.8)	-0.04(-0.06 to -0.02)
Ethiopia	IS	185294.7(162389.2 to 207340)	857(755.1 to 960.4)	338613.5(304513.4 to 374421.2)	687.2(614.1 to 767)	-0.84(-0.9 to -0.77)	18702.6(15513 to 22347.3)	92.5(76.7 to 111.3)	31926.3(26736.6 to 37066.2)	71.3(59.4 to 83.4)	-0.97(-1.12 to -0.83)	24094.9(17545.2 to 30897.1)	116.3(85.9 to 148.6)	45222.6(32742.9 to 57437.1)	95.2(69.6 to 119.9)	-0.75(-0.81 to -0.68)
Fiji	IS	4649.8(4416.2 to 4907.3)	1146.1(1087.9 to 1209.8)	7860.1(7534.7 to 8189.4)	1029.4(987.3 to 1074.1)	-0.4(-0.42 to -0.38)	407.5(352.8 to 468.4)	113.3(97.5 to 130)	696(608.5 to 799.1)	98(87 to 112)	-0.68(-0.76 to -0.6)	863.9(620.6 to 1093.6)	216.8(157.3 to 275.5)	1449.3(1039.8 to 1853.8)	193(139.5 to 243.9)	-0.42(-0.44 to -0.4)
Finland	IS	75820.2(73220 to 78328.4)	1069.9(1034.3 to 1104.8)	96832.9(93436.5 to 100115.9)	805.3(775.8 to 830.5)	-0.87(-0.98 to -0.76)	9292.9(8364.1 to 10288.3)	130.5(118.3 to 143.8)	9250.8(8181 to 10433.2)	72.6(64.2 to 81.6)	-1.93(-2.16 to -1.7)	10718.1(7846.2 to 13512.4)	150.1(110 to 189)	14281.3(10508.8 to 17994.3)	113.4(83.6 to 143.6)	-0.84(-0.95 to -0.73)
France	IS	460947.9(436161.7 to 487237.3)	565.7(536 to 594.6)	740251.5(720510.2 to 762133)	559(544.4 to 575.9)	-0.02(-0.07 to 0.02)	53998.8(49850.3 to 58582.2)	63.9(59.4 to 68.6)	70226.6(64648.9 to 76088.6)	49.9(46.1 to 54.1)	-0.87(-0.94 to -0.81)	66325.2(47761.3 to 83754.7)	79.6(57.4 to 100.2)	110227.5(80473 to 137630.7)	79.3(57.8 to 99.4)	0(-0.04 to 0.05)
Gabon	IS	7820.3(7392.9 to 8218.8)	1365.5(1289.9 to 1438.6)	12645.6(12128.4 to 13213.9)	1200.1(1151.1 to 1251.3)	-0.48(-0.5 to -0.46)	757(647 to 874)	135(115.3 to 154.9)	1209(1036.8 to 1381)	121.9(104.8 to 139.6)	-0.44(-0.5 to -0.38)	1055(779.5 to 1317.1)	189.4(140.5 to 236.9)	1681.2(1251.1 to 2120.9)	166.3(123 to 208.1)	-0.48(-0.5 to -0.46)
Gambia	IS	5421.4(5144.8 to 5714)	1250.4(1191.5 to 1316.6)	13513.6(12926.9 to 14066)	1148.8(1100.1 to 1199.8)	-0.31(-0.33 to -0.29)	451.5(392.9 to 520.5)	110.4(96.2 to 127.3)	1156.2(1015.2 to 1315.5)	105(92.5 to 118.6)	-0.14(-0.17 to -0.1)	714.8(525.5 to 897.9)	172.8(126.5 to 214.5)	1801.7(1323.8 to 2254.4)	159.5(118.5 to 198.9)	-0.29(-0.31 to -0.27)
Georgia	IS	52538.9(49429 to 55865.3)	858.1(807.2 to 911.1)	50797.8(47962 to 53894.1)	929.5(877.7 to 981.8)	0.31(0.26 to 0.37)	6934.9(6020.2 to 7997)	119.2(104.3 to 136.6)	7502.5(6565.2 to 8390.7)	125.2(110.2 to 139.5)	0.43(0.27 to 0.59)	9704.5(6992.3 to 12466.4)	158.9(114.9 to 203.7)	9337.8(6719.7 to 11859.7)	169.6(121.7 to 215)	0.26(0.21 to 0.31)
Germany	IS	1365189.9(1315358.3 to 1415987.7)	1098.8(1060.5 to 1137.9)	1660910(1606056.7 to 1709638)	890(857.8 to 918)	-0.68(-0.7 to -0.67)	164978.1(147734.4 to 182136.6)	131(118.4 to 143.4)	151528.1(134230.4 to 167830.8)	79.3(70.8 to 87.8)	-1.74(-1.82 to -1.65)	192970.6(140718.9 to 244507.1)	152.7(111.5 to 193.8)	243363(177244.9 to 303731)	124.4(90.4 to 156.8)	-0.67(-0.69 to -0.66)
Ghana	IS	115887.8(110068 to 121771.8)	1568.4(1483.2 to 1647)	313624.9(301340.9 to 327552.7)	1603.9(1542 to 1675.7)	0.06(0.05 to 0.07)	9760.8(8574.2 to 11214)	139.8(121.8 to 161.1)	26183.6(23176.2 to 29265.7)	143.1(125.6 to 160.4)	0.21(0.15 to 0.27)	15402.9(11394.5 to 19347.3)	217.4(160.9 to 273.6)	41869.5(30791.9 to 52566.8)	223.7(166.8 to 281)	0.09(0.08 to 0.1)
Greece	IS	120564.5(113429.6 to 128527.8)	809.6(761.2 to 860)	140778.4(134169.2 to 148220.3)	617.4(589.7 to 646.7)	-1(-1.06 to -0.95)	18569.8(16314.8 to 21347.9)	126(111.7 to 142.9)	19139.1(16786.7 to 21384)	70.3(61.5 to 79.3)	-2.2(-2.38 to -2.03)	16797.6(12279.4 to 21287.2)	112.4(82 to 142.4)	20820.6(15516.5 to 26510.7)	86.3(63.8 to 109.8)	-0.99(-1.05 to -0.93)
Greenland	IS	577.6(547.2 to 612.8)	1668.4(1573.2 to 1782.8)	645.3(615 to 672.8)	977.2(933.7 to 1018.6)	-1.94(-2.08 to -1.8)	58.3(50.7 to 67.1)	200.3(176.5 to 227.9)	56.4(48.7 to 65.5)	93.8(82.8 to 106)	-2.99(-3.23 to -2.76)	76.7(55.5 to 97.8)	230.6(167.8 to 293.1)	85.3(63.1 to 108.5)	133.5(99.2 to 169)	-1.99(-2.14 to -1.85)
Grenada	IS	589(540.8 to 643.4)	826.1(761.1 to 899)	784.5(720.2 to 851.3)	685.8(629.1 to 745.3)	-0.74(-0.8 to -0.68)	82.8(73.4 to 93.9)	108.5(96.5 to 123.4)	87.5(78 to 97.6)	88.4(79.9 to 97.8)	-0.8(-0.95 to -0.64)	54.4(40.1 to 70.2)	74.2(55.2 to 95.6)	67.5(50 to 86.6)	60.8(45 to 78)	-0.79(-0.85 to -0.74)
Guam	IS	782.9(745.3 to 821.5)	969.4(924.5 to 1018.9)	1950.1(1869.2 to 2023.7)	966.7(927.2 to 1003.5)	-0.01(-0.03 to 0.01)	67.5(58.3 to 77.5)	93.2(80.8 to 106.8)	156.9(137.9 to 178.1)	76.4(67.4 to 86.5)	-0.87(-0.99 to -0.74)	147.3(106 to 189)	186.6(134.8 to 238.2)	373.7(270.5 to 473.9)	184.5(133 to 233.8)	-0.04(-0.05 to -0.02)
Guatemala	IS	20849.5(19648.4 to 22140)	526.5(498.2 to 558)	52158.7(49606.7 to 54918.2)	441.6(419.7 to 464.8)	-0.68(-0.72 to -0.64)	2031(1745.6 to 2348.7)	58.8(51.2 to 66.7)	5069.6(4441.3 to 5755.3)	46.9(41.2 to 52.9)	-0.91(-1.03 to -0.8)	1802.2(1351.1 to 2291.2)	47.7(36.1 to 61.1)	4625.2(3448.4 to 5880.2)	40(29.9 to 50.4)	-0.7(-0.74 to -0.65)
Guinea	IS	39243.5(37208 to 41228)	1099.7(1042 to 1154.7)	72860.4(69721.3 to 76038.8)	1096.7(1045 to 1144.1)	-0.03(-0.03 to -0.02)	3513.4(3038.3 to 4050.9)	101.9(88.3 to 117.4)	6410.2(5655.2 to 7214)	102.1(90.2 to 114.9)	0.06(0.04 to 0.08)	5327.2(3936.8 to 6637.1)	153(113.5 to 190.8)	9783.6(7196.4 to 12249.5)	152.6(113.3 to 190.2)	-0.02(-0.03 to -0.02)
Guinea-Bissau	IS	6131.3(5828.7 to 6441.6)	1302.8(1230.8 to 1373.3)	11030.2(10545 to 11515.3)	1199.5(1143.8 to 1256.9)	-0.32(-0.34 to -0.3)	526.3(452.1 to 613.6)	117.7(101.8 to 137)	935(810.6 to 1071.4)	110.6(96.1 to 127.2)	-0.21(-0.25 to -0.16)	805.9(591.5 to 1003.8)	178.6(132.8 to 222.7)	1446.9(1069.8 to 1816.3)	166.6(123.9 to 207)	-0.28(-0.3 to -0.26)
Guyana	IS	4230.6(3974.3 to 4495.3)	957.5(893.2 to 1030.4)	5073.3(4835.9 to 5346.2)	751.6(715.5 to 793.5)	-0.97(-1.03 to -0.91)	522(462.7 to 592.2)	136.1(120 to 153.8)	587.8(516.9 to 666.1)	98.5(87.1 to 110.2)	-1.16(-1.23 to -1.09)	355.2(265.6 to 450.6)	82.3(61.7 to 104.2)	424.4(316.2 to 537)	64.8(48.5 to 82)	-0.96(-1.03 to -0.9)
Haiti	IS	25642.1(24153.1 to 27309.5)	695.2(647.5 to 745.9)	52967.7(50225.8 to 55758.9)	633.2(596.1 to 669)	-0.33(-0.36 to -0.3)	3022.6(2624.5 to 3500.1)	100.3(87.6 to 115.5)	6037.7(5298.5 to 6899)	89.6(78.9 to 102.5)	-0.37(-0.41 to -0.33)	2159.6(1602.9 to 2770.8)	61(45.1 to 77.6)	4448.2(3311.5 to 5642.4)	55.4(41.2 to 70.4)	-0.34(-0.37 to -0.31)
Honduras	IS	13480.7(12610.6 to 14312.4)	559.7(523.8 to 592.3)	35479.8(33693.4 to 37425.5)	514.1(486.7 to 542)	-0.32(-0.34 to -0.3)	1269.3(1083.2 to 1474.5)	60(51.7 to 68.8)	3689.2(3173.8 to 4231.3)	63.2(55 to 72.3)	0.13(0.07 to 0.18)	1176.7(879.5 to 1485.2)	50.7(37.8 to 63.7)	3105(2300.8 to 3946.5)	46.7(34.8 to 58.9)	-0.31(-0.33 to -0.29)
Hungary	IS	183680.7(173472.9 to 195225.3)	1272.8(1205.1 to 1351.6)	141661.5(134387.8 to 149052.2)	778.9(742.4 to 817.2)	-1.78(-1.88 to -1.69)	26163.1(23032.4 to 29287.9)	186.2(166.1 to 208.3)	18241.6(16024.2 to 20713.6)	94.2(82.1 to 105.8)	-2.48(-2.6 to -2.36)	32600.2(23524.7 to 41430.8)	226(162.9 to 287.1)	25703.2(18673.9 to 32821.8)	140.3(101.3 to 178.8)	-1.73(-1.82 to -1.63)
Iceland	IS	2486.5(2349.2 to 2626)	866.8(820.7 to 915.2)	3291.9(3150.3 to 3423.5)	593.3(567.3 to 617.4)	-1.27(-1.31 to -1.23)	309.9(268.4 to 360.6)	106.6(92.5 to 123.6)	305.2(263.1 to 354)	52.6(45.1 to 60.9)	-2.56(-2.69 to -2.44)	352.9(256.8 to 448.5)	121.5(88.6 to 154.2)	475.1(348.3 to 601.5)	83.4(60.7 to 105.8)	-1.25(-1.29 to -1.21)
India	IS	2590086.1(2262622.5 to 2903481.9)	487.9(424.7 to 547.6)	5797618.4(5134722.4 to 6439022.6)	460.3(408.7 to 512.1)	-0.24(-0.27 to -0.2)	302344(249878.4 to 362046.6)	68.8(57 to 82.8)	626032.7(529532.3 to 730397.4)	55.8(47.5 to 65)	-0.9(-0.99 to -0.8)	356296.4(259898.9 to 457008.8)	68.8(50.2 to 88.5)	813153.1(599910.1 to 1046618.4)	65.7(48.6 to 84.2)	-0.18(-0.21 to -0.15)
Indonesia	IS	1179831(1041615.6 to 1324265.9)	1085.9(961.6 to 1226)	2721699.1(2432157.9 to 3036174.9)	1108.8(989 to 1233.7)	0.07(0.06 to 0.07)	116745.9(97656.7 to 138207)	121.5(100.6 to 146.6)	306627.9(258912.7 to 363145)	142.6(120.4 to 168.4)	0.6(0.56 to 0.63)	220749.9(157098.4 to 285392)	205.4(147.2 to 265.9)	508032.1(364339.1 to 661103.7)	210(149.8 to 274.6)	0.07(0.07 to 0.08)
Iran (Islamic Republic of)	IS	303360.5(269901.7 to 336543.3)	899.2(795.7 to 1001.6)	653015.7(591272.4 to 718461)	784.9(710.5 to 863.9)	-0.5(-0.53 to -0.46)	31636.5(26816 to 37594.6)	118.3(99.5 to 139.5)	62281.5(53414.3 to 72121)	82.8(70.5 to 95.7)	-1.01(-1.12 to -0.9)	40954.2(30123 to 52779.9)	123.1(90.3 to 158.5)	88655.7(65264.9 to 113224.2)	107.8(79.3 to 136.7)	-0.48(-0.51 to -0.44)
Iraq	IS	98961.9(93086.4 to 104839.4)	1015.9(951.1 to 1080.9)	284908.3(271715.4 to 297853)	1012.9(962 to 1063.3)	0.02(0.01 to 0.03)	11862.6(10477.3 to 13409.8)	135.9(119 to 155.1)	32005.9(28248.4 to 36542.5)	139(122.1 to 158.5)	0.16(0.13 to 0.19)	13409.6(9734.8 to 16983.7)	138.1(101.3 to 174.8)	37993.3(27871.7 to 47875.6)	136.9(100.4 to 172.7)	0.02(0 to 0.03)
Ireland	IS	30263.4(28451.9 to 32269)	737.1(693.7 to 783.6)	29124(27487.2 to 30762.3)	392.9(370.5 to 414.8)	-2.39(-2.52 to -2.26)	4303.6(3698 to 4967.4)	106(91.8 to 121.9)	3085.9(2688.3 to 3582)	39.6(34.4 to 45.7)	-4.04(-4.47 to -3.62)	4209.4(3074 to 5365.9)	102.3(74.6 to 129.9)	4086.1(3009.9 to 5244.1)	54.2(39.9 to 69.6)	-2.42(-2.55 to -2.29)
Israel	IS	43495.8(41714.9 to 45180)	886.5(851.4 to 920.9)	74015.1(71007 to 77110.9)	612.3(585.9 to 638.3)	-1.16(-1.24 to -1.07)	4730(4046.4 to 5490.9)	98.8(85 to 114)	5772.5(4961.2 to 6692.8)	46.9(40.4 to 54.8)	-2.23(-2.4 to -2.06)	6121(4475.5 to 7677.7)	125.2(91.5 to 157.1)	10628.3(7746.5 to 13445.1)	86.2(62.8 to 109.3)	-1.16(-1.24 to -1.08)

Italy	IS	516037.4(465750 to 573957.6)	609.3(552.7 to 672.1)	569281.8(512598.1 to 629687.6)	429.8(388.2 to 471.2)	-1.25(-1.32 to -1.18)	88027.6(69503.4 to 109473)	100.3(80.5 to 123.2)	67740.6(59293.9 to 77123.2)	43.7(38.7 to 49.2)	-2.78(-2.97 to -2.58)	70531.1(50296.4 to 91473.5)	82.6(58.4 to 106.9)	82868(59245 to 106662.5)	59.2(42.3 to 75.6)	-1.18(-1.25 to -1.12)
Jamaica	IS	11134.4(10454.7 to 11799.1)	595.4(560.5 to 629.3)	16218.2(15403.5 to 17149.8)	529.8(503.1 to 559.9)	-0.43(-0.46 to -0.4)	1538.3(1351.1 to 1743.1)	82(71.7 to 93.2)	2229.6(1952.4 to 2465.8)	69.2(60.4 to 76.8)	-0.64(-0.77 to -0.52)	1028.7(757.2 to 1310.7)	54.6(40.2 to 69.4)	1474.6(1109.4 to 1871.6)	47.8(36 to 60.8)	-0.48(-0.51 to -0.45)
Japan	IS	1506699.9(1353537.5 to 1651487.9)	903.8(815.2 to 987.9)	2455644.5(2232199.1 to 2694573)	746.6(682 to 817.6)	-0.71(-0.74 to -0.67)	171957.7(137000.1 to 211473.5)	105.2(85.2 to 127.6)	212866.3(184984.6 to 243881.9)	62(54.5 to 71.5)	-2.16(-2.37 to -1.96)	247422.5(178171.4 to 318244.4)	149.4(107 to 192)	427384.1(304766 to 551006.5)	122.4(87.7 to 158.1)	-0.75(-0.79 to -0.71)
Jordan	IS	20526.4(19316.6 to 21643)	1182.3(1103.5 to 1252.3)	97941.2(93498.6 to 102010.5)	1190.7(1132.6 to 1246.8)	0.05(-0.04 to 0.14)	2292.1(1994.2 to 2627.5)	163.2(142.7 to 186.2)	9791.2(8603.8 to 10978.8)	134.2(115.8 to 150.5)	-0.67(-0.83 to -0.51)	2773.9(2009 to 3523.7)	161.8(119.3 to 203.6)	13268.3(9759.2 to 16790.8)	165.5(121.2 to 209.7)	0.12(0.02 to 0.22)
Kazakhstan	IS	201465.8(190474.4 to 213302.8)	1516.3(1429.9 to 1609.9)	223075.5(213058.8 to 233878.6)	1216(1157.3 to 1275.1)	-0.81(-0.88 to -0.73)	23701.4(20962.9 to 26930.4)	192.7(168.9 to 219)	25492.6(22255.9 to 28888)	154.8(135.2 to 173.9)	-0.81(-0.97 to -0.64)	36479.6(26655.7 to 46466.4)	277.1(202.9 to 352.1)	40479.4(29273.9 to 51114.9)	223.1(160.2 to 282.3)	-0.79(-0.85 to -0.72)
Kenya	IS	98182.4(87494.1 to 108911.5)	1041.9(923.9 to 1167.2)	242424.3(218340 to 267619.1)	978.7(881 to 1083.6)	-0.25(-0.27 to -0.24)	9012.4(7472.5 to 10779.6)	101.1(83.2 to 121.7)	22369.9(19008.9 to 26101.4)	100.6(85 to 117.9)	-0.02(-0.06 to 0.03)	12978.3(9523.6 to 16458)	142.6(105.2 to 181)	32092.4(23415.9 to 40607.8)	136(99.5 to 170.3)	-0.19(-0.2 to -0.17)
Kiribati	IS	535(509.1 to 562.3)	1272.6(1209.7 to 1344.2)	924.6(886.2 to 965.6)	1156.3(1107.6 to 1211.5)	-0.35(-0.37 to -0.33)	49.7(43.7 to 57.3)	127.5(110.8 to 146.5)	85.3(75.3 to 97.2)	119.1(104.1 to 136.2)	-0.31(-0.35 to -0.27)	98.2(69.9 to 125.6)	237.4(170.3 to 303.3)	169.6(123.1 to 214.8)	215.9(158 to 272.2)	-0.35(-0.36 to -0.33)
Kuwait	IS	8485.8(7998.6 to 8999.9)	937.6(890.8 to 984.6)	32360.5(30771.4 to 33928.2)	865.7(828.7 to 903.2)	-0.29(-0.4 to -0.18)	642.6(558.5 to 741.8)	86.9(76.2 to 97.2)	2494.3(2188.1 to 2826)	78.6(68.8 to 87.6)	-0.28(-0.56 to 0)	1145.2(840.6 to 1465.3)	129.5(95.4 to 164.7)	4365.4(3168.2 to 5576.7)	119.4(87.5 to 152.1)	-0.3(-0.41 to -0.19)
Kyrgyzstan	IS	35165.9(33139.8 to 37089.7)	1121.5(1057.7 to 1188.2)	41747.1(39665.6 to 43805.9)	797.2(753.7 to 841.3)	-1.22(-1.27 to -1.17)	4721.5(4179.1 to 5352.2)	160.6(141.6 to 182.6)	5085(4471.1 to 5827.2)	107.4(94 to 122.2)	-1.52(-1.65 to -1.39)	6430.9(4543.6 to 8106.4)	206.2(146.2 to 260.7)	7582.5(5454.8 to 9650)	147(105.4 to 187.2)	-1.2(-1.26 to -1.15)
Lao People's Democratic Republic	IS	20123.8(18914 to 21344.3)	890.8(833.1 to 946.4)	42189.4(40150 to 44221.2)	852.3(808.1 to 900.1)	-0.17(-0.18 to -0.16)	2301.4(1970.8 to 2693.6)	117.1(101.4 to 134.8)	4718.8(4107.7 to 5383.2)	108(93.9 to 124)	-0.33(-0.38 to -0.28)	3739.3(2675 to 4826.1)	167.4(121.8 to 215.7)	7878(5615.3 to 10073.1)	161.1(115.8 to 206)	-0.14(-0.15 to -0.13)
Latvia	IS	35007.1(31530.2 to 38080.2)	998.2(903.6 to 1084)	34613(32364 to 36543.2)	934.8(885.2 to 980.5)	-0.09(-0.23 to 0.04)	7222.8(6350.8 to 8311.6)	205.6(181.2 to 235.8)	5679.3(5047.4 to 6349.4)	137.5(122.1 to 153.3)	-1.24(-1.29 to -1.19)	6304.7(4549.2 to 8019.8)	179.7(129.6 to 229)	6401.4(4624.6 to 8098.1)	170.4(123.6 to 216)	-0.02(-0.17 to 0.12)
Lebanon	IS	19250.3(18126.8 to 20336.2)	818.8(770.8 to 868.7)	52502.3(49630.9 to 55544.4)	880.7(833.5 to 927.8)	0.33(0.3 to 0.36)	2248.5(1957.5 to 2550.6)	108.9(94.7 to 123.6)	5676.2(4933.5 to 6390.1)	93(80.9 to 104.8)	-0.44(-0.48 to -0.4)	2614.2(1916.2 to 3308.4)	112.5(82.8 to 141.9)	7187.2(5280.4 to 9072.8)	119.7(87.9 to 151.5)	0.3(0.27 to 0.32)
Lesotho	IS	7134.1(6677 to 7563.4)	822(767.6 to 876.6)	10558.8(9987.3 to 11121.5)	989.6(932.5 to 1052)	0.65(0.62 to 0.68)	816(683.4 to 953.1)	100.3(83.7 to 117.6)	1311.4(1105.2 to 1530.1)	141(118.9 to 163.1)	1.39(1.23 to 1.55)	998.7(735.5 to 1248.2)	118.4(87.3 to 148.3)	1428.5(1061.4 to 1791.3)	140(103.4 to 174)	0.58(0.55 to 0.62)
Liberia	IS	14635.4(13926.7 to 15427.2)	1116.2(1054.6 to 1177)	25788.2(24578.8 to 26941.4)	953.4(910.4 to 995.2)	-0.55(-0.58 to -0.52)	1323.3(1129.3 to 1528.6)	105.8(90.4 to 122.2)	2131.1(1847.9 to 2434.8)	85.5(74.7 to 96.7)	-0.62(-0.68 to -0.57)	1915.4(1421.6 to 2404.3)	150.8(112.5 to 187.2)	3336.7(2424.3 to 4172)	130(96.2 to 161.1)	-0.51(-0.54 to -0.49)
Libya	IS	19089.9(18084.3 to 20146.2)	761.6(721.2 to 806.5)	52232.3(49804.1 to 54508.6)	837.4(799 to 875)	0.4(0.35 to 0.45)	1690.3(1502.5 to 1920.1)	77.1(68.1 to 87.2)	4784.7(4255.5 to 5402)	88.6(78.8 to 100.1)	0.48(0.44 to 0.51)	2606.7(1904.9 to 3328.4)	104.8(77.2 to 133.8)	7041.9(5261.5 to 8955.1)	114(85.3 to 143.9)	0.36(0.3 to 0.42)
Lithuania	IS	40396.1(36159.5 to 44683.2)	915.4(818.2 to 1010.3)	43580.7(37885 to 49795.2)	866.5(766.9 to 970.2)	1.59(0.95 to 2.23)	9460.2(8283.4 to 10533.2)	211(184.7 to 235.1)	8869.3(7638.8 to 10147.6)	151.8(132.6 to 172.2)	-0.94(-1.24 to -0.64)	7257(5305.8 to 9336.6)	164.4(119.2 to 211)	7971.3(5562.9 to 10484.3)	156.5(109.8 to 204.1)	1.71(1.03 to 2.39)
Luxembourg	IS	3744.6(3333.8 to 4151.2)	704.4(630 to 783.4)	4080.8(3906.4 to 4239.6)	408.8(391.1 to 425.7)	-2.17(-2.34 to -1.99)	528.5(466 to 596.7)	99.7(88.9 to 111.4)	440.8(399 to 483.3)	41.1(37 to 45.2)	-3.13(-3.53 to -2.73)	520.3(372.7 to 677.2)	97.3(69.9 to 126.5)	584.2(424.5 to 743.9)	57.2(41.8 to 72.8)	-2.15(-2.33 to -1.98)
Madagascar	IS	76990.5(73202.1 to 80750)	1413(1336.7 to 1491.2)	171926.9(163837.2 to 179172.9)	1365.6(1302.4 to 1428.9)	-0.12(-0.13 to -0.11)	7272.1(6258.3 to 8309.6)	138.8(118.6 to 160.4)	16080.3(14009.7 to 18497.8)	137.3(120.1 to 157.6)	-0.07(-0.12 to -0.02)	10207.2(7535.3 to 12691.1)	193.6(143 to 240.2)	22571(16500.9 to 28433.1)	188.8(141.5 to 236.4)	-0.07(-0.08 to -0.06)
Malawi	IS	47295.6(44720.3 to 50018)	1119.3(1055.2 to 1182.6)	87602.6(83266.9 to 91842.3)	1040.3(987 to 1091.8)	-0.27(-0.29 to -0.25)	4573.7(3915.4 to 5278.1)	117.5(100.9 to 135.6)	8415(7318 to 9673.1)	111.2(96.5 to 125.9)	-0.22(-0.28 to -0.16)	6225(4601 to 7738.7)	153.5(114 to 190.9)	11596.2(8568.2 to 14715.5)	144.5(107.7 to 182)	-0.21(-0.22 to -0.19)
Malaysia	IS	99495.7(94125.3 to 104831.2)	998.6(940.6 to 1058.7)	280508.6(268072.3 to 293768.4)	979.8(937.2 to 1027.7)	-0.07(-0.08 to -0.05)	11395.9(9844.4 to 13000.2)	121.7(104.3 to 140.1)	26668.7(23127.6 to 30695.9)	97.7(84.2 to 111.4)	-0.66(-0.76 to -0.57)	18575(13291.4 to 23498.7)	187.7(135 to 237.8)	52500.8(38094.5 to 66592.4)	185.2(134.9 to 235.3)	-0.04(-0.05 to -0.03)
Maldives	IS	1097.9(1042 to 1161.9)	1113.8(1054.2 to 1175.6)	2664.4(2534.1 to 2801.5)	701.1(665.8 to 736.1)	-1.68(-1.76 to -1.6)	115.4(98.7 to 134.2)	128.6(111.2 to 149)	263.4(227.8 to 301.3)	77.7(67.7 to 88.5)	-2(-2.17 to -1.84)	203(147.4 to 261.7)	209.6(152 to 269.2)	494.4(358 to 631.6)	132.8(96.7 to 168.7)	-1.64(-1.72 to -1.57)
Mali	IS	42390.9(40252.7 to 44714.9)	941.8(890.2 to 996.1)	90828.2(86420.9 to 94967.9)	822.7(785.5 to 860.4)	-0.51(-0.54 to -0.48)	3816.6(3309.7 to 4403.7)	91.8(80.1 to 105.4)	7566.2(6538.7 to 8776.6)	76.9(67.3 to 87.1)	-0.61(-0.66 to -0.56)	5614.9(4133.1 to 7061.4)	129.8(96.4 to 161.2)	12006.5(8648.7 to 15084.4)	113.7(83.3 to 141.6)	-0.49(-0.52 to -0.46)
Malta	IS	2715.1(2522.1 to 2906.2)	641(596.4 to 687.2)	3323.7(3150.5 to 3501.4)	383.7(363.2 to 403.8)	-2(-2.15 to -1.86)	400.1(343.2 to 461.4)	96.3(83.2 to 110.5)	377.3(326.6 to 433.4)	38.9(33.9 to 44.5)	-3.24(-3.61 to -2.87)	373.2(264.9 to 484.8)	88.7(63.2 to 114.9)	470.6(343.3 to 594.2)	52.6(37.7 to 66.8)	-2.04(-2.19 to -1.9)
Marshall Islands	IS	198.6(188.4 to 210.8)	1013.2(954.9 to 1076.1)	366.1(348.8 to 383.4)	975.1(926.6 to 1024.7)	-0.14(-0.15 to -0.13)	18.6(16.1 to 21.5)	108.1(92.9 to 125.2)	33.5(29.3 to 38.7)	104.3(91.1 to 119.9)	-0.2(-0.29 to -0.11)	37.1(26.5 to 47.2)	192(139.6 to 242)	66.9(48.4 to 85.6)	181.8(131.5 to 231.5)	-0.19(-0.21 to -0.18)
Mauritania	IS	15459.8(14699 to 16212.9)	1393.6(1324.1 to 1463.7)	26222.4(25043.8 to 27418.4)	1073.3(1025.4 to 1121.9)	-0.96(-1.01 to -0.91)	1394.1(1210.3 to 1611.1)	129.2(112.6 to 149.3)	2176.2(1905 to 2477.7)	93.6(81.5 to 106.3)	-1.12(-1.24 to -0.99)	2096.9(1543.2 to 2635.1)	194.9(144.1 to 245.3)	3576.7(2640.7 to 4496.5)	151.5(112.4 to 188.7)	-0.92(-0.97 to -0.87)
Mauritius	IS	8953.4(8470 to 9404.9)	1168.4(1102.6 to 1235.5)	12789.3(12208.3 to 13374.4)	728.3(695.4 to 760.8)	-1.74(-1.84 to -1.65)	1067.9(922.3 to 1243.5)	155.4(134.2 to 178.2)	1384(1182.6 to 1588)	80.3(69.2 to 91.5)	-2.85(-3.12 to -2.58)	1662.8(1191.5 to 2146.1)	219.2(157.7 to 282.5)	2397.9(1769.9 to 3014.7)	137.3(101.1 to 173.6)	-1.73(-1.82 to -1.64)
Mexico	IS	376710.3(340892.7 to 413921)	773.2(699.4 to 849.4)	757765.3(687567 to 832271)	596.4(542 to 655.3)	-1(-1.07 to -0.92)	37406.6(31440 to 43861)	84.8(71.1 to 100.5)	65180.8(55026.3 to 75805.3)	53.4(45.3 to 62.3)	-1.79(-1.98 to -1.61)	32851.5(24055.9 to 42285.8)	69.7(51.1 to 88.4)	67610.5(49746.6 to 86886.6)	54.2(39.9 to 69.4)	-0.98(-1.05 to -0.91)
Micronesia (Federated States of)	IS	584.9(555.1 to 619.7)	1075.7(1018.3 to 1142)	758.7(724.4 to 792.7)	1001.7(955.5 to 1052.2)	-0.26(-0.28 to -0.25)	56.8(49.3 to 66.2)	114.4(98.7 to 132.9)	72(62.4 to 82.6)	107.1(93.2 to 122.3)	-0.27(-0.3 to -0.25)	109.3(78.8 to 139.4)	203.6(147.4 to 260.7)	140.5(101.4 to 178.1)	189(137.8 to 239)	-0.28(-0.3 to -0.26)
Monaco	IS	613.2(578.4 to 651.6)	904.2(852.8 to 955.2)	573(544.3 to 604.8)	640.6(609.3 to 674)	-1.28(-1.35 to -1.21)	82.5(70.2 to 96)	113.4(97.2 to 131.1)	62.2(53.7 to 72.3)	62.6(54.2 to 72.2)	-2.25(-2.45 to -2.05)	91.4(66.4 to 116.9)	129.2(93 to 165.5)	84.3(61.7 to 107.3)	89.9(65.2 to 114.1)	-1.33(-1.4 to -1.26)
Mongolia	IS	9816(9284.4 to 10387.5)	764.3(717.6 to 813.9)	21810.1(20689.5 to 22849.8)	842.4(798 to 887)	0.35(0.28 to 0.41)	783.5(666.6 to 915.6)	64.5(55.2 to 74.9)	1800.4(1570.4 to 2082.2)	73.7(64.2 to 84.4)	0.53(0.35 to 0.71)	1766.7(1277.8 to 2259.2)	138.6(99.8 to 178.9)	3905.6(2760.6 to 4958.8)	154.1(110 to 195.6)	0.39(0.32 to 0.45)
Montenegro	IS	3829(3621.3 to 4055.6)	617.3(581.2 to 655)	5642.6(5335.3 to 5956.7)	607.2(574.5 to 640.4)	-0.03(-0.05 to -0.01)	466(401.6 to 532.7)	78.1(66.9 to 89.4)	716.3(619.7 to 825)	79.8(70 to 90.5)	0.37(0.24 to 0.5)	691.1(498.1 to 885)	111.9(80.5 to 143.8)	1012.3(734.4 to 1276.3)	109.3(79 to 137.6)	-0.04(-0.07 to -0.01)
Morocco	IS	146380(137503.9 to 155247.9)	883.1(828.5 to 940.3)	333629.1(317092.2 to 351288.1)	924.9(877.8 to 978)	0.16(0.14 to 0.17)	16785(14872.5 to 19317.7)	115.8(101.7 to 132.3)	40538(35616 to 46052.6)	123.8(109.5 to 139.5)	0.23(0.2 to 0.27)	19825.2(14438.5 to 25208)	120.1(88.2 to 152.2)	44686.2(32692.6 to 56221.5)	124.9(91.8 to 157.3)	0.14(0.12 to 0.15)
Mozambique	IS	72100.9(67914.2 to 76284.3)	1114.1(1043.5 to 1186.2)	158534.3(151209.5 to 166119.7)	1250.5(1187.9 to 1316.4)	0.41(0.39 to 0.44)	7507.7(6468.5 to 8785.5)	124.6(106.1 to 145.6)	16509.3(14381.1 to 18823.1)	140.7(121.9 to 161.7)	0.57(0.48 to 0.66)	9421.9(6953.3 to 11708.3)	150.9(112.9 to 187.5)	20504.7(15217.3 to 25407.8)	169.3(126 to 210.3)	0.43(0.39 to 0.46)
Myanmar	IS	219298.1(206730.8 to 232477.5)	875.2(820.4 to 929.1)	397616.6(379245.9 to 417558.2)	801.6(762.9 to 843.4)	-0.29(-0.31 to -0.28)	24793.9(21396 to 28806.1)	113.1(97.6 to 131.4)	45301.6(39623.1 to 51752.7)	99.1(86.7 to 112.2)	-0.49(-0.59 to -0.4)	41061.8(29839.9 to 52820.1)	165.7(121.2 to 213.4)	74816.5(54138.2 to 94831.7)	152.4(110.9 to 193.2)	-0.26(-0.28 to -0.24)
Namibia	IS	8247.3(7813.7 to 8696.9)	1266.6(1195.7 to 1341.6)	14987.7(14256 to 15710.2)	1096.7(1036.4 to 1158.5)	-0.52(-0.55 to -0.5)	918.5(780.9 to 1076.6)	153.2(129.4 to 177.1)	1726.2(1481 to 1989.9)	139.5(119.5 to 160.3)	-0.45(-0.51 to -0.39)	1124.1(827.3 to 1412.7)	180.9(134 to 225.3)	2057.1(1524.9 to 2566.9)	157.5(116.9 to 197.1)	-0.49(-0.51 to -0.47)
Nauru	IS	76(72.3 to 79.8)	1441.4(1366.5 to 1520)	91.2(87.7 to 94.9)	1369(1315.8 to 1425)	-0.18(-0.21 to -0.16)	6.2(5.4 to 7.1)	132.8(114.5 to 152.4)	7(6.2 to 8)	104.4(92.2 to 118.5)	-1.02(-1.13 to -0.91)	14(10 to 18)	271.5(195.8 to 344.5)	16.9(12.2 to 21.4)	257.3(187 to 324.8)	-0.18(-0.21 to -0.16)
Nepal	IS	53617.2(50429.5 to 57277.7)	502.6(468.7 to 539.7)	112477.8(105957.8 to 119183.9)	454.3(426 to 484.3)	-0.36(-0.37 to -0.34)	6045.1(5121.5 to 7064.6)	66.7(56.7 to 77.5)	13137.7(11288.8 to 15164)	60.7(52.5 to 69)	-0.36(-0.44 to -0.27)	7447.3(5423.1 to 9525.4)	71.7(52.3 to 91.5)	16003.9(11591.5 to 20418.9)	65.9(47.7 to 83.5)	-0.3(-0.31 to -0.28)
Netherlands	IS	201182.7(192377.9 to 210664.8)	1019.1(974.9 to 1066.6)	199639(189617.3 to 208839.9)	614(583.1 to 641.7)	-1.82(-1.91 to -1.73)	22329.1(19821.4 to 24994.8)	112.1(99.5 to 125.3)	19396.3(16696.3 to 22566.4)	56.8(49.4 to 65.6)	-2.83(-3.08 to -2.58)	28762.3(20814.5 to 36516.5)	144.2(104.3 to 183.1)	28423.4(20919.7 to 36084.4)	85.1(62.7 to 108.1)	-1.89(-1.98 to -1.79)
New Zealand	IS	27663.3(24556.1 to 30787)	710.3(633.6 to 787.2)	44625.1(40088.4 to 49773.5)	558.4(502.9 to 618)	-0.89(-0.98 to -0.81)	3406.9(2789.2 to 4110)	88(72.7 to 105.6)	4329.5(3709.3 to 4973.1)	51.8(44.5 to 58.9)	-1.8(-1.92 to -1.68)	3990.1(2859.8 to 5261.4)	102.5(73.5 to 134.6)	6592.9(4667.7 to 8501.7)	81(57.4 to 104.2)	-0.88(-0.96 to -0.8)
Nicaragua	IS	12127.4(11407.2 to 12838.6)	689.9(650.9 to 731.1)	27968.6(26569.9 to 29525.8)	547.8(521.7 to 577.6)	-0.84(-0.88 to -0.8)	1315.5(1134.8 to 1517.1)	82.6(71.8 to 94.2)	2774.4(2409.3 to 3150.3)	57.6(50.4 to 65.4)	-1.37(-1.49 to -1.26)	1076.6(795.1 to 1367.2)	63.4(47 to 80.5)	2506.2(1851.7 to 3171.1)	50.3(37.3 to 63.5)	-0.83(-0.88 to -0.79)
Niger	IS	36321.8(34465 to 38301.1)	1069.9(1012.5 to 1131.6)	93307(88199.8 to 97757)	906.9(860.8 to 953.3)	-0.62(-0.65 to -0.58)	3287.9(2848.1 to 3842.4)	103(88.3 to 119.7)	8182.5(6968.4 to 9515.6)	85.7(73.9 to 98.1)	-0.69(-0.76 to -0.62)	4791.5(3529.5 to 6099.9)	148.3(110.5 to 186.2)	12442.8(9184.9 to 15634.2)	127.1(94.9 to 158.9)	-0.56(-0.6 to -0.53)
Nigeria	IS	512496(456906.7 to 568534.7)	1039.2(917.9 to 1162.5)	1089161.3(987483.9 to 1197105.6)	987.4(883 to 1096)	-0.19(-0.21 to -0.17)	47010.6(39274.6 to 56143.8)	101.3(83.9 to 122.3)	91564.3(77814.4 to 107150.2)	90.3(76.5 to 105.6)	-0.36(-0.4 to -0.32)	68192.5(50030.5 to 86457.5)	143.2(105.6 to 182.5)	145988.9(106243.9 to 185167.8)	138.7(101.8 to 175.3)	-0.11(-0.14 to -0.08)
Niue	IS	26.3(25 to 27.7)	1172.7(1113.1 to 1235.1)	21.9(21 to 22.9)	1068.6(1023.7 to 1114.8)	-0.33(-0.35 to -0.31)	2.6(2.2 to 3)	112.4(97.6 to 129.8)	2(1.7 to 2.3)	96.4(83.3 to 109.9)	-0.65(-0.72 to -0.58)	5.1(3.7 to 6.4)	224.7(162.7 to 285.5)	4.1(3 to 5.2)	202.2(147 to 255.6)	-0.36(-0.37 to -0.35)
North Macedonia	IS	22673.7(20544.5 to 24884.6)	1212.9(1099.3 to 1334)	32571(29511.2 to 35983.8)	1012.9(918.8 to 1116.7)	-0.68(-0.73 to -0.63)	3783.1(3324.5 to 4339.6)	223.5(196.6 to 255.3)	5666(4899.7 to 6427.5)	214.4(190.5 to 239.9)	-0.11(-0.16 to -0.07)	4063.3(2912.8 to 5236.5)	218.8(155.5 to 281.2)	5804.8(4131.9 to 7451.3)	182.1(129.5 to 233.3)	-0.67(-0.72 to -0.63)
Northern Mariana Islands	IS	195.5(184.5 to 207.8)	919.5(876.9 to 966.7)	397.1(380.8 to 414.8)	827.5(792.4 to 865.2)	-0.4(-0.42 to -0.38)	15(12.8 to 17.4)	82.8(70.3 to 95.1)	35(29.9 to 40.6)	76(65.8 to 86.4)	-0.41(-0.45 to -0.36)	35.9(25.4 to 46.1)	174.2(126.6 to 221.5)	73.5(53.3 to 93.9)	156.6(113.7 to 198.2)	-0.4(-0.43 to -0.38)
Norway	IS	73172.4(64760.4 to 82024.6)	1097.6(980.9 to 1217.9)	74867(67523.5 to 82605.6)	759.4(688.4 to 834)	-1.34(-1.43 to -1.25)	9247.5(7267.8 to 11546)	134.6(107.6 to 165.6)	7596.7(6370.4 to 8922.7)	73.8(62.5 to 86.2)	-2.36(-2.59 to -2.12)	10283.3(7262.6 to 13402.1)	150.6(107.1 to 193.7)	10862.2(7683.1 to 13906.1)	106.8(76 to 136.3)	-1.27(-1.37 to -1.18)

Oman	IS	8986.8(8479.3 to 9529.4)	902(844.1 to 953.8)	29346.5(28018.1 to 30706.6)	924.1(883.3 to 966.3)	0.1(0.04 to 0.15)	875.9(761.1 to 998.4)	113.5(98.8 to 131.6)	2337.5(2057.7 to 2634.9)	110(96.3 to 123.5)	-0.02(-0.11 to 0.08)	1191.8(877.4 to 1517.7)	120.7(90 to 154.2)	3875.5(2826.4 to 4964.8)	124.4(91.2 to 157.9)	0.13(0.07 to 0.19)
Pakistan	IS	465948(409883.9 to 523235.5)	744(651.7 to 838.9)	1049105.7(930005.4 to 1169892.4)	740.3(660 to 829.3)	0(-0.01 to 0.01)	50475.7(42201.9 to 60354.7)	89.3(73.9 to 106.7)	106744.2(91890.9 to 124114.9)	86.5(73.6 to 100.9)	-0.2(-0.27 to -0.12)	65376.1(47313.9 to 84216.3)	106.1(76.8 to 136.3)	145907.9(106213.9 to 188400.9)	105.6(77.4 to 135.8)	0.01(0 to 0.03)
Palau	IS	123(117.1 to 129.2)	1182(1125.3 to 1243.1)	257.1(246.1 to 267.9)	1194.3(1138.2 to 1246.8)	0.03(0.02 to 0.04)	11(9.5 to 12.7)	114.4(98.6 to 131.8)	22.4(19.3 to 25.9)	110.7(96.4 to 126.7)	-0.19(-0.25 to -0.14)	23.1(16.6 to 29.8)	225.2(163.5 to 288.1)	47.2(34.2 to 59.6)	223.3(162.2 to 281.4)	-0.02(-0.04 to -0.01)
Palestine	IS	8087.9(7628.8 to 8609.1)	747.1(702.3 to 796.3)	22022.2(20849.4 to 23087.6)	685.3(647.8 to 718.6)	-0.36(-0.43 to -0.3)	1090.4(952.7 to 1243.6)	125.7(110.4 to 143.2)	2771.4(2482.6 to 3125.9)	123.1(111 to 135.9)	0.05(-0.1 to 0.21)	1107.9(824.7 to 1405)	103.1(76.2 to 131.1)	2975.2(2149.1 to 3797.7)	93.8(68.9 to 119.8)	-0.39(-0.45 to -0.32)
Panama	IS	10441.7(9911.7 to 11032.4)	648.8(616.1 to 684.4)	23976.9(22890.9 to 25269.2)	540.9(516.7 to 569.6)	-0.69(-0.73 to -0.65)	1166.5(1003 to 1333.1)	78.6(67.6 to 89.7)	2534.9(2174.8 to 2897.1)	56.3(48.2 to 64.6)	-1.26(-1.35 to -1.17)	940.3(705.7 to 1188.2)	59.8(45.3 to 75.9)	2232.3(1677.4 to 2834.4)	50.1(37.6 to 63.6)	-0.68(-0.72 to -0.63)
Papua New Guinea	IS	13078.7(12307.8 to 13863.1)	647.1(610 to 688.8)	34606.1(33021.5 to 36305.1)	616.2(587.1 to 645.1)	-0.19(-0.2 to -0.18)	1138.5(962.5 to 1354.3)	64.7(54.7 to 75.7)	3046.9(2614.2 to 3501.6)	61.2(52.9 to 69.7)	-0.24(-0.28 to -0.19)	2388.7(1715.3 to 3064.8)	120.5(88 to 152.4)	6301.7(4598.6 to 7972.2)	114.5(84.9 to 145.4)	-0.19(-0.2 to -0.18)
Paraguay	IS	16621.3(15654.9 to 17583.9)	711.2(669.2 to 753.7)	35992.7(34227.2 to 37844.3)	607.1(576.3 to 638.9)	-0.6(-0.63 to -0.57)	1964.1(1697.8 to 2251.3)	88.4(76.8 to 100.9)	4037.8(3525.8 to 4522.7)	70.6(61.5 to 79.3)	-0.85(-0.91 to -0.79)	1457.4(1087.7 to 1845.3)	63.6(47.7 to 80.7)	3139.1(2341.2 to 3980.9)	53.9(40.3 to 68.8)	-0.62(-0.65 to -0.59)
Peru	IS	75342.6(71193.3 to 79807.3)	564.6(534.3 to 597.8)	169715.8(161504.7 to 177970.1)	492.3(467.9 to 515.9)	-0.52(-0.57 to -0.48)	7544.2(6545.7 to 8643.8)	60.3(52.3 to 69.1)	14364.5(12519.3 to 16440.6)	41.8(36.6 to 48)	-1.31(-1.46 to -1.16)	6763.3(5028.9 to 8563.1)	52.1(39 to 66.4)	15636(11571.3 to 19802.6)	45.6(33.7 to 57.7)	-0.5(-0.55 to -0.46)
Philippines	IS	230229.1(207451 to 254570.8)	692(615.6 to 774.2)	691806.6(621854 to 768552.1)	800.9(714.4 to 885.9)	0.55(0.52 to 0.59)	20012.5(16475.1 to 23884.8)	67.5(54.9 to 82)	68843.1(58883.6 to 80269.3)	85.6(72.8 to 99.8)	0.87(0.67 to 1.06)	42629.7(30001.9 to 54974.1)	129.4(92 to 166.3)	129143.3(92410.3 to 166859.9)	151.2(107.7 to 194.7)	0.6(0.56 to 0.64)
Poland	IS	286014(257497.7 to 316470.9)	661.4(595.7 to 728.2)	409060.9(364813.9 to 458233.6)	603.4(542.8 to 666.4)	-0.44(-0.53 to -0.34)	46955.4(38144.6 to 56294.9)	113(93.2 to 134.4)	58610(48782.9 to 69316.9)	81.1(67.9 to 94.7)	-1.19(-1.27 to -1.11)	51027.1(36473.3 to 65514.2)	118.3(84.4 to 152)	75221.7(52569.5 to 97389)	110.1(77.9 to 140.5)	-0.37(-0.47 to -0.28)
Portugal	IS	136947.6(124539.8 to 150837.5)	986.9(899.3 to 1081.1)	91609.2(86448.1 to 96823.6)	411.7(390.9 to 433.8)	-3.08(-3.22 to -2.94)	21443.9(18753.4 to 24300.8)	161.9(144.2 to 182)	12969.9(11437.1 to 14480.4)	48(42.2 to 53.5)	-4.74(-5.12 to -4.37)	18772.7(13580.2 to 24294.9)	135.9(98.1 to 175.9)	13110.1(9519.3 to 16595.6)	56.3(40.9 to 71.5)	-3.11(-3.25 to -2.97)
Puerto Rico	IS	19479.8(18366.8 to 20643.4)	536.3(506.5 to 568.5)	31353.7(29892.4 to 32780.5)	496.6(470.4 to 519.8)	-0.25(-0.33 to -0.17)	1959.3(1690.5 to 2264.3)	56.5(49.1 to 64.9)	2800.4(2433.9 to 3203.7)	39.2(33.6 to 45.5)	-1.45(-1.55 to -1.35)	1767.1(1313.3 to 2273)	48.9(36.3 to 63.1)	3039.8(2244.3 to 3839.2)	45.2(33.5 to 57.5)	-0.28(-0.36 to -0.2)
Qatar	IS	2423.8(2291.1 to 2566.1)	1221.6(1162.4 to 1279)	14421.5(13555.5 to 15333.1)	747.7(708.1 to 787.4)	-1.68(-1.87 to -1.49)	188.7(161.9 to 217.7)	119(105.2 to 136.1)	928.8(753 to 1130.1)	77.1(67.2 to 88.5)	-1.62(-1.96 to -1.29)	317.5(229.2 to 408.7)	165.9(121.6 to 210.1)	1868.1(1367.7 to 2379.7)	100.3(75.1 to 126.6)	-1.71(-1.9 to -1.52)
Republic of Korea	IS	519259.2(493918.9 to 545058.2)	1716.4(1632.6 to 1807.9)	780867.5(748702.7 to 815511.8)	864.8(828.3 to 903)	-2.45(-2.57 to -2.32)	59462.6(51463.8 to 68651.1)	207.5(181.2 to 236.8)	66826.7(56987.2 to 76616.5)	74.9(64.4 to 85.3)	-3.91(-4.15 to -3.66)	81620.7(58683.3 to 104080.7)	276.9(199.5 to 352.4)	126748.8(91510.3 to 163145.7)	140.4(101.1 to 181.4)	-2.43(-2.55 to -2.3)
Republic of Moldova	IS	34234.3(31961 to 36389.9)	752.8(701.3 to 802.1)	43662.9(41334.8 to 46122)	771.3(731.8 to 813.9)	0.15(0.12 to 0.18)	5155(4481.3 to 5911.9)	128.9(113.6 to 146.8)	6404.9(5551.6 to 7299.6)	109.7(95.4 to 124.4)	-0.29(-0.38 to -0.2)	6138.9(4434.6 to 7883.3)	135.6(98.2 to 174.3)	7905.2(5755.3 to 10153.5)	139.4(101.3 to 179.2)	0.17(0.14 to 0.21)
Romania	IS	292407.5(269077.7 to 313392.6)	1055.5(973.2 to 1130.7)	321912.2(304643 to 339513.4)	902.8(860.2 to 946.8)	-0.57(-0.61 to -0.53)	46842.2(40144.8 to 54374.2)	183.6(160.9 to 209.6)	49448.6(43255.6 to 55128.1)	129.3(114.5 to 143.4)	-1.28(-1.37 to -1.19)	52156(38565.1 to 67175.4)	189.3(139.2 to 244.2)	59148.2(43310.7 to 74877.7)	164.5(120.4 to 207.8)	-0.51(-0.54 to -0.47)
Russian Federation	IS	1778849.6(1573976.6 to 1981910.2)	978.1(868.9 to 1085.1)	2048668.2(1836872.9 to 2268685.1)	899.7(810.3 to 990.4)	-0.36(-0.44 to -0.28)	325936(266419.6 to 396726.4)	196.7(163.2 to 232.2)	337709.1(285475.2 to 395209.6)	144.2(122.8 to 167.4)	-1.01(-1.15 to -0.88)	317912.4(230213.2 to 410126.9)	175.4(127.2 to 225.6)	368850.9(268660.6 to 473440.5)	161.5(117.3 to 207.5)	-0.35(-0.44 to -0.26)
Rwanda	IS	38914.7(36692.8 to 41271.2)	1277.1(1195.3 to 1363)	62427.6(59258.3 to 65458.5)	928.1(874.6 to 977.3)	-1.17(-1.23 to -1.1)	4102.6(3504.8 to 4824.5)	144(123.5 to 170)	6104.9(5230 to 7092.3)	100.7(86.1 to 117.6)	-1.5(-1.68 to -1.32)	5176.5(3803.1 to 6562.4)	176.9(131.2 to 225.1)	8389.1(6277.9 to 10431.4)	130.8(97.3 to 162.2)	-1.07(-1.13 to -1.02)
Saint Kitts and Nevis	IS	417.5(381.7 to 456.7)	1109.6(1020.2 to 1211)	511.2(483.8 to 541.1)	734(694.7 to 777)	-1.51(-1.6 to -1.42)	53.1(46.4 to 61.6)	147.5(131.4 to 166.9)	58.2(50.7 to 66.6)	96.5(85.1 to 108.1)	-1.63(-1.78 to -1.48)	37.9(28.2 to 48.8)	99.9(74.9 to 127.7)	43.6(32.9 to 55)	65.5(49.4 to 82.3)	-1.54(-1.62 to -1.45)
Saint Lucia	IS	796.2(737.1 to 857.4)	848.2(784.9 to 912.8)	1495.9(1428.6 to 1568.3)	641.6(611.8 to 672.3)	-0.99(-1.05 to -0.93)	98.3(85.7 to 111.1)	118.2(104.2 to 133)	167.8(148.5 to 188.2)	72.9(64.7 to 81.7)	-1.84(-2 to -1.67)	70.9(52.9 to 90)	76.7(57.4 to 97.4)	132.4(99 to 166.7)	57.1(42.7 to 72)	-1.03(-1.09 to -0.97)
Saint Vincent and the Grenadines	IS	598.4(560.3 to 641.2)	778.3(727.2 to 834.5)	852.3(809.2 to 897.4)	607.9(576.9 to 639.8)	-0.91(-0.98 to -0.85)	71.1(62 to 81.9)	99.9(88 to 114)	96.4(85.3 to 108.9)	71.7(63.5 to 80.4)	-1.13(-1.29 to -0.96)	53.4(39.6 to 68.4)	69.9(51.6 to 89.6)	74.2(55.4 to 93.4)	53.4(39.9 to 67.3)	-0.98(-1.04 to -0.92)
Samoa	IS	976(925.4 to 1030)	1050.2(994.6 to 1116)	1550.5(1487.1 to 1615.1)	1032.8(989.7 to 1077.9)	-0.05(-0.07 to -0.04)	89(75.9 to 104.3)	103.1(88.2 to 120.4)	137.2(120.1 to 154.8)	94.3(82.4 to 107.1)	-0.33(-0.38 to -0.28)	183.1(132.5 to 233.5)	199.6(144.6 to 254.8)	290.3(211.6 to 368.5)	195.4(143.3 to 248)	-0.07(-0.08 to -0.05)
San Marino	IS	265.5(251.8 to 280.5)	765.9(727.7 to 808.9)	392.5(368.9 to 414.4)	565.1(534.5 to 593.6)	-1.11(-1.17 to -1.06)	31.8(26.6 to 37.4)	89.7(75.8 to 104.8)	41.5(35.3 to 48.5)	53.7(45.7 to 63)	-1.94(-2.06 to -1.82)	38.1(27.8 to 48.5)	108.2(78.9 to 138)	58.3(42.3 to 73.7)	79.4(57.8 to 100.3)	-1.13(-1.18 to -1.07)
Sao Tome and Principe	IS	994.3(946.5 to 1045.2)	1427.5(1354.5 to 1498.2)	1837(1762.6 to 1915.4)	1422.2(1361.8 to 1485.1)	-0.02(-0.04 to 0.01)	81.8(70.5 to 93.6)	120.5(103.8 to 137.8)	151.9(134.3 to 170.9)	124.7(109.8 to 140.1)	0.1(0.03 to 0.16)	136.6(101.1 to 171.6)	200.4(149.5 to 250.2)	246.3(181.4 to 308.9)	198(146.4 to 247.7)	-0.03(-0.05 to -0.02)
Saudi Arabia	IS	58944.4(55258.8 to 62775.6)	740.9(693.4 to 791.1)	202682.5(193391.9 to 212307.3)	763.4(724.1 to 803.1)	0.04(0.02 to 0.07)	6659.5(5852 to 7602.8)	110.7(96.7 to 127.1)	19020.9(16690.6 to 21461.1)	96(85.3 to 106.8)	-0.52(-0.65 to -0.39)	7835.8(5724.1 to 9962.8)	98.9(73.2 to 125.2)	26400.1(19296.1 to 33776.2)	101.4(74.8 to 128.9)	0.02(0 to 0.05)
Senegal	IS	48799.9(46354.3 to 51367)	1297.9(1231.2 to 1365.8)	99786.3(95383 to 104070.6)	1116.3(1068.7 to 1164.8)	-0.54(-0.57 to -0.52)	4190.9(3635.3 to 4874.4)	114.7(99.3 to 131.6)	8114.7(7084.8 to 9235.7)	96.1(84.8 to 109.6)	-0.55(-0.6 to -0.51)	6504(4826.8 to 8239.4)	179.2(133.5 to 225)	13445.2(9868.5 to 16788.9)	156(116.7 to 194.4)	-0.5(-0.52 to -0.47)
Serbia	IS	119112.4(108291.1 to 130744)	1073.6(974.9 to 1181.4)	131245.9(119625.4 to 143085.5)	834.5(769.2 to 906.5)	-0.94(-1 to -0.89)	19070.1(16325.2 to 22068.2)	192.2(166.7 to 218.3)	25510(22729.6 to 28366.3)	153.3(137.4 to 168.8)	-0.88(-0.98 to -0.77)	21272.7(15574.2 to 27830.6)	193.3(141 to 252.2)	23664.8(16804.8 to 29988.3)	149.9(106.9 to 190.2)	-0.95(-1.01 to -0.89)
Seychelles	IS	556.8(525.5 to 587.4)	957.2(902.2 to 1009.5)	965.4(921.8 to 1009.6)	834.6(792.7 to 873.2)	-0.5(-0.52 to -0.48)	60.7(52.7 to 69.2)	106.2(92.3 to 120.8)	94.2(81.3 to 107.9)	85.5(74.1 to 97.5)	-0.82(-0.91 to -0.74)	105.3(76.3 to 135.3)	181(130.5 to 231.9)	177.6(128.7 to 225.6)	155(112.2 to 196.9)	-0.55(-0.57 to -0.53)
Sierra Leone	IS	29539.2(28103.9 to 31137.1)	1265.2(1202.7 to 1331)	52972(50763.2 to 55199.4)	1148.2(1098.9 to 1202.8)	-0.36(-0.38 to -0.34)	2577.1(2228.8 to 2949.1)	114.3(99.7 to 131.4)	4392.4(3872.7 to 5003)	101.6(89 to 116.1)	-0.36(-0.39 to -0.34)	3931.7(2908.2 to 4928.9)	173(128.3 to 215.7)	7044.4(5264.2 to 8766.1)	159(118.4 to 198.2)	-0.31(-0.33 to -0.29)
Singapore	IS	30753.9(29290.1 to 32317.2)	1381.1(1313 to 1451.7)	56077.3(53411.8 to 58841.6)	668.2(637.8 to 701.3)	-2.55(-2.67 to -2.43)	3339.7(2880.3 to 3833.3)	151.3(131.6 to 173.4)	4434.1(3706.2 to 5271.6)	52.9(44.6 to 61.9)	-4.09(-4.37 to -3.81)	4980.1(3583.8 to 6363.8)	229.3(164.9 to 291.8)	9282.5(6680.9 to 11789.4)	111.4(80.3 to 141.3)	-2.54(-2.66 to -2.43)
Slovakia	IS	83583.1(80293 to 87265.5)	1404.8(1348.2 to 1468)	95162.4(90419 to 99499)	1033.1(982.2 to 1078.4)	-0.99(-1.12 to -0.87)	10282.5(8727.7 to 11757.9)	173.6(148.1 to 197.7)	10730.5(9258.8 to 12349.5)	114.6(99.4 to 130.5)	-1.4(-1.65 to -1.14)	15334.3(10974.7 to 19319.2)	258.2(185.3 to 324.6)	17589.2(12817.5 to 22189.1)	190.5(139.2 to 239.9)	-0.97(-1.1 to -0.84)
Slovenia	IS	16809(15202.5 to 18351.5)	697.2(633.3 to 760.9)	20346.5(19472.3 to 21263.3)	490.3(468.5 to 511.7)	-1.18(-1.3 to -1.05)	3294.9(2860.3 to 3768.2)	137.3(119.7 to 156)	2737.1(2438.4 to 3043.2)	59.1(53 to 65.5)	-2.76(-3.08 to -2.43)	3005.5(2158.7 to 3858.6)	124.8(89.5 to 159.9)	3740.3(2723.2 to 4769.8)	89.1(64.9 to 113.1)	-1.13(-1.26 to -1)
Solomon Islands	IS	1760.1(1669.6 to 1858.5)	1143.1(1073 to 1214.8)	4326.9(4113 to 4515.2)	1135.4(1076.8 to 1192.4)	-0.04(-0.06 to -0.03)	172.7(149.4 to 201.7)	129.6(110.9 to 152.3)	445.7(390.2 to 508.5)	132.7(114.9 to 151.6)	0(-0.04 to 0.04)	329.9(239.4 to 418.3)	218.8(158.5 to 278.9)	812.7(585 to 1029.8)	217.2(158.1 to 276.1)	-0.05(-0.06 to -0.03)
Somalia	IS	29990.3(28402 to 31819.8)	1030.4(964 to 1104.4)	71934.4(68304.5 to 75821.1)	927.4(875.4 to 978.1)	-0.37(-0.38 to -0.35)	2976.9(2516.6 to 3520.1)	110.6(93.5 to 130.5)	6892.3(5919.7 to 8049.5)	99.3(86.6 to 112.9)	-0.36(-0.4 to -0.33)	3871(2892 to 4886.5)	139.8(104.4 to 176.8)	9310.6(6851.8 to 11755.2)	126.9(94.8 to 157.7)	-0.32(-0.34 to -0.31)
South Africa	IS	295631(265225 to 327268.6)	1355.1(1206.9 to 1511.4)	510187.4(458126.6 to 560993.7)	1123.2(1006 to 1240.1)	-0.71(-0.76 to -0.65)	26247.9(21881 to 31358.2)	125.3(103.3 to 151.5)	50938(42146.3 to 60031.8)	119.4(99.3 to 141.1)	-0.31(-0.6 to -0.01)	40550.4(29327.3 to 51699)	190.8(137.2 to 241.2)	69492.6(50238.1 to 87215.9)	157.6(112.9 to 198.2)	-0.71(-0.77 to -0.66)
South Sudan	IS	28667.3(27098.3 to 30404.2)	1055.3(999.2 to 1119.9)	39578.2(37542.9 to 41673.8)	932.3(887.4 to 978.3)	-0.4(-0.42 to -0.38)	2776.2(2355.3 to 3257.7)	106.9(92.1 to 125.4)	3722.1(3181.9 to 4283.8)	95.8(82.4 to 110.2)	-0.26(-0.3 to -0.21)	3690.6(2767.2 to 4585.1)	140.1(105.4 to 173.1)	5085(3748.4 to 6341.1)	124.5(93.4 to 154.2)	-0.37(-0.39 to -0.35)
Spain	IS	384187.6(350615 to 418178.2)	714.9(656.5 to 777.2)	566959.2(552727.3 to 580911.4)	606.8(590.1 to 623.6)	-0.41(-0.44 to -0.37)	60528.2(52351.6 to 69468.2)	112.2(97.7 to 127.5)	53332.8(49629.9 to 56839.8)	55(51.3 to 58.9)	-2.49(-2.71 to -2.28)	53956.8(39077 to 70538.6)	100(72.4 to 130)	83353.1(61882.5 to 103951.5)	85.4(63.3 to 106.5)	-0.4(-0.43 to -0.36)
Sri Lanka	IS	100000.8(94724.9 to 105459.7)	869.1(823.8 to 916.2)	204024.6(194395.6 to 213415.8)	770.3(732.7 to 806.4)	-0.48(-0.51 to -0.45)	10481(9045.3 to 11978.8)	106.8(92.1 to 121.4)	21902.4(18948.1 to 25221.3)	88.2(77.4 to 99.8)	-0.74(-0.86 to -0.63)	18658.2(13486.8 to 24190.7)	163.9(118.5 to 212)	38382(28120.5 to 48656.2)	145.8(106.4 to 184.5)	-0.46(-0.49 to -0.43)
Sudan	IS	95665.9(89863.2 to 102102.4)	831.8(775.9 to 889.5)	225083.2(214044.6 to 236472)	898.5(852.1 to 949.6)	0.29(0.26 to 0.31)	11138.3(9735.5 to 12761.6)	116.5(102 to 132.5)	24581(21643.7 to 27805.8)	118(104 to 132.4)	0.06(0 to 0.13)	12864.8(9645.7 to 16273.8)	112.7(84.8 to 143.5)	30259.9(22171.4 to 38745.4)	121.6(89.3 to 156.4)	0.29(0.27 to 0.31)
Suriname	IS	1987.2(1876.5 to 2100.3)	723.8(681.5 to 766.8)	4158.3(3957.8 to 4370.7)	660.7(628.4 to 695.7)	-0.32(-0.35 to -0.3)	214.6(188.5 to 244.6)	84.5(73.8 to 96.2)	469.5(410.8 to 529)	77.4(67.7 to 87.1)	-0.44(-0.55 to -0.34)	172.7(130.7 to 217.1)	64.4(48.5 to 80.9)	362.4(270.2 to 457)	58.5(43.7 to 73.7)	-0.34(-0.36 to -0.31)
Sweden	IS	127515.4(113785.8 to 144527.6)	858.8(770.1 to 956.1)	140796.3(125528.7 to 156341.1)	699.5(627.3 to 766.7)	-0.79(-0.85 to -0.73)	15774(12773.8 to 19035)	100.6(83.1 to 120.4)	14224.4(11663.9 to 17150)	62.9(52.3 to 74.3)	-1.91(-2.08 to -1.73)	18585.2(13043.8 to 24109.5)	121.6(86.2 to 156.1)	20858.3(14688.3 to 26712.3)	98.3(69.7 to 125.4)	-0.83(-0.89 to -0.77)
Switzerland	IS	58966.2(55745.7 to 62974.6)	575.2(544.1 to 612.1)	79394.6(75994 to 82783.4)	462.2(442.4 to 482.2)	-0.75(-0.78 to -0.71)	7895.9(6912.8 to 8948.2)	73.7(65 to 82.9)	7834.7(6796.2 to 8925.2)	42.6(36.9 to 48.6)	-1.8(-1.95 to -1.66)	8202.6(5967.7 to 10438.8)	78.4(57 to 99.7)	11508.5(8325.8 to 14530.5)	64.4(46.8 to 81)	-0.68(-0.71 to -0.64)
Syrian Arab Republic	IS	65943(62311.6 to 69496.4)	928.7(874.2 to 977.9)	106037.3(100688.8 to 111409.3)	754.9(712.8 to 797.3)	-0.74(-0.78 to -0.71)	6551.5(5841.1 to 7390.5)	108(95.1 to 121.4)	10526.4(9257 to 11912.9)	88.7(78.9 to 99.5)	-0.82(-0.92 to -0.71)	8871.6(6446.3 to 11307.5)	125.8(91.8 to 158.9)	14198.9(10508.7 to 18143.7)	102.6(75.7 to 130.3)	-0.74(-0.78 to -0.7)
Taiwan (Province of China)	IS	172144.2(164681.8 to 179582.3)	1163(1112.5 to 1212.3)	353147(338416.7 to 367800.3)	871.4(836.6 to 906.2)	-1.08(-1.14 to -1.01)	19367.2(16804.7 to 22190)	133.9(116.4 to 152.2)	34127.6(29303.1 to 39373.8)	82.6(71.6 to 94.9)	-2.12(-2.31 to -1.94)	33764.5(24182.4 to 42946.5)	233.2(168.9 to 294.3)	70999.2(51131.7 to 90029.2)	174(125.6 to 220.3)	-1.09(-1.16 to -1.03)
Tajikistan	IS	24659.4(23127.8 to 26213.7)	822.1(770.6 to 877.5)	51652.5(48943.2 to 54270.3)	801.4(751.4 to 850.4)	-0.11(-0.17 to -0.05)	3110.2(2697.8 to 3556.1)	110.4(94.7 to 126.8)	6863.4(5963.9 to 7811.8)	132.7(115.5 to 150.9)	0.89(0.78 to 1)	4561.7(3223.1 to 5801.3)	153.6(108.8 to 194.9)	9432.3(6761.3 to 12018)	149.6(108.5 to 190.4)	-0.1(-0.16 to -0.04)

Thailand	IS	317283.5(301806.9 to 334503.3)	848.9(804.3 to 898.3)	754917.1(723050.3 to 787757.9)	724.7(694.2 to 754.1)	-0.57(-0.61 to -0.54)	31360.6(27122.7 to 36331.6)	89.7(77.1 to 103.8)	68521.8(59605.2 to 78339.7)	65.5(57.2 to 74.3)	-1.32(-1.43 to -1.2)	60266.4(43347 to 76775.1)	163.7(118.3 to 208.7)	146412.9(107194.4 to 185883.9)	140.5(102.2 to 178.4)	-0.55(-0.59 to -0.51)
Timor-Leste	IS	2281.7(2149.2 to 2412.7)	694.1(650.7 to 739.1)	6744(6392.4 to 7076)	735.5(696.7 to 773.5)	0.21(0.2 to 0.23)	236.9(202.3 to 278.1)	86.3(73.5 to 100.3)	811.4(695.4 to 941.2)	98.1(84.7 to 112.9)	0.46(0.4 to 0.53)	416.9(301.7 to 529.6)	128.1(94.4 to 165.2)	1237.4(897.6 to 1583.8)	135.7(98.3 to 172.5)	0.23(0.21 to 0.25)
Togo	IS	19491.9(18526.2 to 20541.6)	1257.6(1193.1 to 1325.8)	49934.2(47555.4 to 52355.9)	1104.8(1054.5 to 1156.9)	-0.49(-0.52 to -0.46)	1683.5(1458.7 to 1940.9)	115.3(99.9 to 131.4)	4216.8(3690.9 to 4814.7)	101.5(88.1 to 115.4)	-0.45(-0.5 to -0.4)	2571.9(1900.3 to 3211)	173.8(128.3 to 216)	6645.4(4841.9 to 8331.9)	154.9(113.5 to 195.1)	-0.44(-0.46 to -0.41)
Tokelau	IS	11.9(11.3 to 12.6)	890.5(843.3 to 943)	12.8(12.3 to 13.4)	870.2(834.1 to 908.7)	-0.08(-0.1 to -0.07)	1.2(1 to 1.4)	90.5(77.7 to 105.2)	1.1(1 to 1.3)	76.2(66.2 to 86.8)	-0.78(-0.87 to -0.69)	2.3(1.6 to 2.9)	171.6(123.3 to 219)	2.4(1.8 to 3.1)	165.6(120.6 to 209.1)	-0.11(-0.13 to -0.1)
Tonga	IS	482.6(456.2 to 509.9)	814.7(768.8 to 857.7)	648.3(619.2 to 678.9)	780.7(746.4 to 814.8)	-0.17(-0.19 to -0.16)	41.9(35.7 to 48.5)	74.9(64.1 to 86.3)	59.3(51.7 to 66.6)	73(63.7 to 82.4)	-0.16(-0.2 to -0.11)	91.3(65.7 to 116.5)	156.3(113.9 to 199.1)	123.4(87.8 to 157.5)	149.3(106.5 to 190.1)	-0.18(-0.2 to -0.17)
Trinidad and Tobago	IS	8538.2(8093.9 to 8994.2)	938.8(888.2 to 988.7)	12843.3(12229.4 to 13448.1)	695.2(661.7 to 728.6)	-1.12(-1.18 to -1.06)	945.2(826.1 to 1073.7)	113.8(99.9 to 129.6)	1328.9(1146.2 to 1509.3)	72.7(63.3 to 82)	-1.7(-1.81 to -1.6)	736(541.8 to 932.9)	82.1(60.6 to 103.9)	1124.5(839.8 to 1424.2)	61.3(45.8 to 77.9)	-1.09(-1.15 to -1.03)
Tunisia	IS	38685.6(36360.6 to 40929.7)	674.5(634.6 to 714.2)	97243.5(92888.2 to 102139.8)	728.2(693.7 to 764.5)	0.29(0.24 to 0.34)	4343.6(3754.2 to 4973.3)	91.1(78.6 to 103.1)	11480.1(9981.1 to 13302.2)	91.3(79.8 to 104.8)	-0.01(-0.1 to 0.09)	5296.7(3839.2 to 6724.7)	93.5(68.2 to 119.4)	13222.1(9711.4 to 16879)	99.8(73.5 to 127.9)	0.27(0.22 to 0.32)
Turkmenistan	IS	405280.3(382127.3 to 427278.9)	1041.5(978 to 1099.2)	701854.2(671660.1 to 733949.3)	758.6(724.7 to 794.8)	-1.18(-1.24 to -1.11)	40404.1(35163.8 to 46703.2)	116.4(101 to 134.2)	69523.4(60002.1 to 79849.3)	78.5(68.4 to 90.2)	-1.5(-1.58 to -1.41)	56538.7(41545.2 to 72194.5)	147.5(108.5 to 187.2)	98373(71949.1 to 126110.2)	107.4(78.5 to 137.4)	-1.18(-1.25 to -1.11)
Tuvalu	IS	21746.7(20484 to 23025.9)	1013.4(953.2 to 1079.1)	53871.7(51405.4 to 56406.9)	1240.6(1169.6 to 1309)	0.82(0.76 to 0.87)	2516.5(2201.1 to 2865.1)	129.9(113.3 to 148.4)	5979.7(5235.5 to 6764.7)	147.1(129.7 to 165.9)	0.71(0.61 to 0.81)	3946.6(2793.4 to 5032.8)	186(131.7 to 238.7)	9744.6(7056.5 to 12452.5)	227.7(165.8 to 290.6)	0.84(0.78 to 0.9)
T 眉 rkiye	IS	64.5(61.1 to 67.9)	952.6(899.4 to 1005.8)	97.9(93.9 to 102.3)	934.9(895 to 979.7)	-0.09(-0.11 to -0.08)	6.2(5.3 to 7.3)	98.7(84.8 to 114.1)	9.5(8.3 to 10.9)	95.5(83.9 to 109.3)	-0.25(-0.32 to -0.18)	12.2(8.9 to 15.7)	183.6(135.1 to 234)	18.4(13.5 to 23.3)	177.9(129.7 to 224.2)	-0.11(-0.12 to -0.1)
Uganda	IS	84849.6(79892.7 to 90147.3)	1210.5(1144.6 to 1282.1)	179088.3(170916.5 to 188243.2)	1094.9(1041.9 to 1144.4)	-0.38(-0.43 to -0.33)	7981.1(6693.1 to 9407.2)	119(101 to 139.6)	16918.2(14455.4 to 19391.2)	111.1(94.3 to 127.4)	-0.38(-0.5 to -0.25)	11190.9(8270.7 to 14010.3)	166.5(124.7 to 207.5)	23999.5(17800.1 to 29867.2)	154.5(115 to 192.6)	-0.28(-0.33 to -0.23)
Ukraine	IS	822813.1(724980.7 to 916588.5)	1170.6(1037.3 to 1300.5)	712633.4(634548.3 to 793791.3)	991.1(885.5 to 1097.6)	-0.5(-0.56 to -0.45)	141423.1(115122.7 to 172948.9)	209.5(172.6 to 249.7)	107459.3(89950.7 to 126619.4)	143.5(121.3 to 167)	-1.51(-1.63 to -1.39)	148214.4(107337.4 to 191969)	211(152.2 to 272.2)	128677.5(93394.3 to 168476.2)	177.9(129.2 to 231.2)	-0.49(-0.54 to -0.43)
United Arab Emirates	IS	11369.7(10744.6 to 12062.9)	1378.6(1299.8 to 1468.3)	88362.9(84296.8 to 92704.7)	1201.6(1134.5 to 1263.3)	-0.51(-0.57 to -0.46)	1041.8(898.9 to 1197.9)	182.9(161.2 to 209.2)	7841.6(6528.5 to 9342.7)	138.8(123.3 to 156.8)	-1.07(-1.23 to -0.92)	1490.9(1103.1 to 1894.4)	184.5(136.5 to 235.7)	11257.3(8245.4 to 14292.9)	158.1(116.8 to 200)	-0.56(-0.61 to -0.5)
United Kingdom	IS	695085.6(641356.5 to 754317)	797.3(737.3 to 858.9)	714518.3(661107.7 to 769754.7)	581.4(538.5 to 622.9)	-1.15(-1.2 to -1.09)	94637.5(78247.4 to 112105.8)	104.2(87.4 to 121.9)	72318.6(62977.3 to 82443.2)	55.3(48.3 to 63)	-2.36(-2.47 to -2.25)	99119.3(70684 to 128045.7)	111.5(79.3 to 143.2)	104201.4(74584.5 to 132514)	81.8(58.5 to 103.7)	-1.14(-1.19 to -1.08)
United Republic of Tanzania	IS	119029.6(112545 to 125116.7)	1003.3(948.2 to 1060.3)	328680.4(314012.4 to 343890.5)	1164.8(1107.1 to 1220.7)	0.51(0.47 to 0.56)	11538.9(9881.8 to 13441.1)	102.5(87.8 to 119.2)	31679(27730.8 to 36056.3)	119.8(102.6 to 137)	0.8(0.68 to 0.91)	15641.5(11533.7 to 19570.7)	136.6(101.2 to 169)	43869.8(32484.2 to 54482)	161.5(120.7 to 201.4)	0.59(0.54 to 0.64)
United States of America	IS	3100388.7(2812947.1 to 3417383.5)	996.3(901.9 to 1095.5)	5136253.5(4700312.2 to 5584576.9)	962.1(885.8 to 1044.7)	-0.39(-0.59 to -0.2)	282791.3(230222.1 to 345341)	88.8(72.5 to 107.7)	311586.8(259142.2 to 370269)	56.3(47.5 to 66.3)	-1.62(-1.78 to -1.45)	438406.8(311557.6 to 565663.6)	139.6(99.6 to 178.5)	711180.9(514467.4 to 902154.8)	131.6(95.5 to 167.3)	-0.49(-0.69 to -0.29)
United States Virgin Islands	IS	486.1(457.9 to 514.3)	542.7(511.1 to 574)	881.9(836.5 to 930.2)	533.8(507.4 to 562.7)	-0.04(-0.1 to 0.03)	47.6(40.9 to 55.1)	60.2(52.1 to 68.9)	100.2(87.1 to 116.2)	59(51.6 to 67.6)	-0.04(-0.09 to 0.01)	42.5(31.3 to 53.6)	49.2(36.2 to 62)	80(59.8 to 101.4)	47.6(35.5 to 60.5)	-0.1(-0.17 to -0.03)
Uruguay	IS	44332.8(42135.9 to 46596.3)	1157.4(1100.7 to 1214.5)	36573(34930.7 to 38305.3)	700.2(670.4 to 733.4)	-1.87(-1.98 to -1.77)	4921.2(4315.3 to 5556.2)	130.9(115.4 to 147)	3919.4(3451.3 to 4436.3)	69.8(61.7 to 79.1)	-2.33(-2.48 to -2.17)	6418(4651 to 8107)	166.9(120.8 to 210.6)	5409.6(4011.3 to 6825)	100.8(74.5 to 127.4)	-1.87(-1.97 to -1.77)
Uzbekistan	IS	135724.1(127161.5 to 143791.5)	1095.5(1023.3 to 1164.9)	307473.9(292279.1 to 322147.3)	1087.9(1032.4 to 1146.3)	-0.04(-0.07 to -0.02)	16066.6(14133.7 to 18284.4)	134.6(118 to 152.7)	37227.7(32987.8 to 42472.4)	150.7(133.6 to 170.3)	0.31(0.1 to 0.53)	24756.9(17982.8 to 31538.5)	201.2(146.2 to 256.9)	55458.6(40011.5 to 70425.9)	199.4(144.6 to 251.9)	-0.04(-0.06 to -0.02)
Vanuatu	IS	882.4(834.8 to 931.5)	1209(1144.9 to 1280.5)	2344(2251.5 to 2453.3)	1213.8(1156.1 to 1271.6)	0.01(0 to 0.01)	76.9(66.8 to 89.7)	117.6(101.8 to 137.4)	208.7(182.5 to 236.5)	118.2(104.1 to 133.5)	-0.05(-0.08 to -0.02)	163.9(118 to 211.7)	228.7(165.6 to 290.9)	436.5(317.6 to 555.1)	229.9(167.1 to 291.1)	0.02(0.01 to 0.03)
Venezuela (Bolivarian Republic of)	IS	70789.7(66932.5 to 74879.6)	662(626.9 to 699.6)	157922(150264.8 to 165808.5)	540.5(515.4 to 568.4)	-0.71(-0.74 to -0.68)	7215.5(6209.9 to 8374.2)	74.2(64.4 to 86.1)	16234.9(14039.2 to 18534.9)	56.9(49.4 to 64.9)	-0.88(-0.97 to -0.8)	6248.5(4696.3 to 7932.3)	60.3(45.4 to 76.7)	14206.5(10558.4 to 18012.5)	49.4(36.6 to 62.5)	-0.69(-0.72 to -0.66)
Viet Nam	IS	338956.4(319206.3 to 359443.6)	790.1(741.3 to 837.9)	888433.3(852548.3 to 926825.7)	881.8(844.4 to 920.6)	0.36(0.34 to 0.39)	42679.4(36936.4 to 49114.9)	108.9(94 to 125.5)	110010.3(100112.7 to 120836.5)	116.1(105.6 to 127.4)	0.11(0.03 to 0.2)	64423.1(47043.6 to 82708.9)	151(110.4 to 194.7)	167151.8(120105.5 to 212163.6)	167.5(120.8 to 212.8)	0.34(0.32 to 0.36)
Yemen	IS	48196.9(45481.5 to 51338.5)	745.7(696.3 to 792.6)	137862.9(130780.4 to 145321.6)	751(707.9 to 794.9)	0.08(0.04 to 0.12)	5774.5(4988.1 to 6649.9)	113.4(99.3 to 128.2)	16560.2(14634.6 to 18601.1)	113.2(100.4 to 126.6)	0.07(0.02 to 0.13)	6441.5(4786.5 to 8263.8)	100.7(75.2 to 129.6)	18489.6(13795.7 to 23292.7)	101.8(75.9 to 129.9)	0.1(0.06 to 0.14)
Zambia	IS	34578.2(32790.3 to 36585.6)	1113.1(1052.3 to 1178.4)	87264.1(83128.3 to 91212.1)	1155.1(1097.6 to 1214.9)	0.15(0.13 to 0.17)	3381.9(2876.9 to 3950.1)	116.1(98.6 to 135.1)	9001.7(7766.5 to 10263.4)	131.6(112.6 to 150.5)	0.51(0.44 to 0.57)	4594.7(3395.9 to 5750)	153.8(113.7 to 190.3)	11621.9(8562.6 to 14557.5)	161.8(120.4 to 201.6)	0.23(0.2 to 0.26)
Zimbabwe	IS	42457.3(40157.6 to 44860.1)	954.4(901.8 to 1009.4)	79105.5(75099.5 to 82799.8)	1059(1007.2 to 1112.1)	0.34(0.32 to 0.36)	4172(3625.4 to 4807.8)	103.7(89.9 to 119.5)	7956.7(6833.6 to 9148.7)	122(106.5 to 139.6)	0.69(0.57 to 0.81)	5772.8(4198.5 to 7244.1)	135.4(98.9 to 169.4)	10644.4(7791.6 to 13453.9)	149.3(111.4 to 187)	0.33(0.31 to 0.35)

ASR, Agestandardised rates; EAPC, estimated annual percentage change; ICH, intracerebral hemorrhage; SAH, subarachnoid hemorrhage; IS, ischemic stroke; YLDs, years lived with disability

Table S4 Age-Standardized QCI in global and 5 sociodemographic index regions

		**Age-Standardized QCI**									**Age-Standardized QCI for percent change**		
		**both**			**female**			**male**					
**Location**	**cause**	**1990**	**2021**	**changes**	**1990**	**2021**	**changes**	**1990**	**2021**	**changes**	**both**	**female**	**male**
Andean Latin America	ICH	58.5	70.1	11.6	63.4	73.8	10.5	53.6	66.3	12.7	46.5	80.0	76.0
Australasia	ICH	74.6	84.1	9.5	76.5	85.6	9.0	72.9	82.7	9.8	46.6	77.0	75.1
Caribbean	ICH	51.2	56.8	5.6	57.0	63.5	6.5	45.2	49.5	4.3	46.5	79.6	76.5
Central Asia	ICH	55.3	60.2	4.9	59.9	64.9	4.9	50.6	55.2	4.6	46.3	77.3	77.1
Central Europe	ICH	50.9	62.4	11.5	57.5	70.5	13.1	44.2	53.3	9.1	46.5	79.6	76.6
Central Latin America	ICH	67.9	74.2	6.3	71.9	79.7	7.9	63.3	67.3	4.1	46.5	79.8	76.5
Central Sub-Saharan Africa	ICH	43.9	42.0	-1.9	46.3	46.2	-0.1	41.7	38.2	-3.5	46.5	81.8	77.3
East Asia	ICH	42.1	54.6	12.5	51.7	63.3	11.6	30.8	44.5	13.7	46.5	79.7	74.8
Eastern Europe	ICH	58.2	63.6	5.4	62.8	70.4	7.5	53.4	56.2	2.8	46.5	78.8	76.5
Eastern Sub-Saharan Africa	ICH	40.2	44.3	4.1	48.1	50.2	2.1	32.5	38.6	6.1	46.5	81.3	76.6
Global	ICH	55.7	62.1	6.4	61.7	67.4	5.7	49.3	56.5	7.2	46.5	79.7	75.2
High SDI	ICH	76.2	86.9	10.7	80.2	90.0	9.9	71.8	83.7	11.9	46.6	77.8	74.2

High-income Asia Pacific	ICH	81.4	95.2	13.7	84.8	98.6	13.9	77.7	92.1	14.4	46.5	79.2	75.4
High-income North America	ICH	83.3	91.2	7.9	86.1	92.9	6.8	80.1	89.8	9.7	46.3	100.0	85.1
High-middle SDI	ICH	54.3	62.6	8.2	61.7	69.4	7.7	45.9	55.0	9.1	46.5	79.7	75.6
Low SDI	ICH	47.3	52.7	5.4	51.1	54.9	3.8	43.8	50.8	7.0	46.5	80.7	75.9
Low-middle SDI	ICH	51.1	57.1	6.0	55.4	60.7	5.3	47.0	53.8	6.8	46.5	79.7	74.6
Middle SDI	ICH	47.2	55.8	8.6	54.2	63.0	8.8	40.3	48.1	7.8	46.5	79.7	75.2
North Africa and Middle East	ICH	48.6	63.2	14.6	54.1	65.4	11.3	43.7	61.6	17.9	46.5	79.6	75.7
Oceania	ICH	33.4	35.5	2.1	34.0	34.7	0.7	31.9	35.1	3.3	46.5	81.5	76.6
South Asia	ICH	56.8	62.8	6.1	61.7	66.0	4.3	51.7	59.6	8.0	46.6	79.7	71.7
Southeast Asia	ICH	47.5	50.9	3.4	52.2	56.5	4.3	43.5	45.6	2.1	46.5	80.4	76.6
Southern Latin America	ICH	72.0	82.7	10.7	77.9	87.8	9.9	64.9	76.3	11.4	46.5	79.2	76.1
Southern Sub-Saharan Africa	ICH	58.6	42.9	-15.7	61.3	45.9	-15.4	56.9	42.1	-14.8	46.5	86.0	78.5
Tropical Latin America	ICH	53.1	63.8	10.7	60.9	71.0	10.1	44.6	55.7	11.1	46.5	80.7	76.6
Western Europe	ICH	72.6	83.4	10.8	76.2	86.0	9.8	68.7	80.6	11.9	46.6	77.9	75.1
Western Sub-Saharan Africa	ICH	53.4	60.2	6.8	50.0	57.3	7.3	57.2	63.7	6.5	46.5	78.8	75.7
Andean Latin America	IS	68.5	80.3	11.8	75.7	85.4	9.7	58.9	73.2	14.3	87.5	72.5	77.8
Australasia	IS	69.4	87.7	18.3	71.7	87.1	15.5	67.4	87.6	20.2	87.5	72.5	77.8
Caribbean	IS	53.9	64.5	10.6	61.0	71.9	10.9	46.0	55.1	9.2	87.0	71.8	77.1
Central Asia	IS	61.5	63.8	2.3	70.9	73.3	2.3	46.7	49.6	2.9	87.4	72.9	75.3
Central Europe	IS	38.7	60.1	21.4	48.5	67.3	18.7	28.3	52.2	24.0	87.5	72.5	77.7
Central Latin America	IS	69.2	80.0	10.8	75.9	85.9	10.0	59.8	71.3	11.5	87.7	72.8	77.8
Central Sub-Saharan Africa	IS	68.0	68.1	0.1	75.6	75.9	0.3	56.4	55.1	-1.4	87.8	73.5	77.9
East Asia	IS	54.1	67.7	13.6	65.0	77.3	12.3	40.2	54.2	14.0	88.3	74.9	78.1
Eastern Europe	IS	33.1	53.6	20.5	46.3	63.7	17.5	16.7	41.0	24.3	87.3	72.3	78.0
Eastern Sub-Saharan Africa	IS	73.1	74.1	1.0	78.8	80.2	1.4	64.8	64.8	0.0	87.6	73.0	77.2
Global	IS	57.6	70.9	13.3	64.9	77.5	12.5	49.7	62.6	12.9	84.3	71.6	77.2
High SDI	IS	70.3	89.1	18.8	74.8	91.5	16.8	65.0	85.9	20.9	87.2	72.1	78.0
High-income Asia Pacific	IS	69.5	90.5	21.0	72.8	92.8	20.0	64.8	87.1	22.3	87.6	72.5	77.8
High-income North America	IS	84.4	99.6	15.2	87.3	99.6	12.3	80.5	99.5	19.0	86.6	72.1	78.5
High-middle SDI	IS	45.5	65.9	20.4	54.8	73.2	18.4	35.9	56.8	20.9	90.7	71.4	77.4
Low SDI	IS	63.3	65.2	1.9	71.1	73.7	2.6	53.2	53.6	0.5	87.8	72.9	77.9
Low-middle SDI	IS	55.3	60.2	4.9	61.8	68.1	6.3	47.9	50.6	2.6	86.4	71.8	79.1
Middle SDI	IS	56.7	67.4	10.7	66.3	76.2	9.9	45.0	55.8	10.7	88.6	23.9	78.1
North Africa and Middle East	IS	42.0	56.2	14.3	48.1	60.7	12.7	36.1	51.5	15.4	86.7	71.8	77.9
Oceania	IS	69.7	72.2	2.5	72.3	74.5	2.2	67.2	69.8	2.6	87.7	72.9	77.4
South Asia	IS	56.6	60.3	3.7	60.1	65.9	5.8	52.1	53.6	1.4	87.7	72.4	78.6
Southeast Asia	IS	61.2	63.3	2.1	69.6	72.4	2.8	50.7	51.7	1.0	0.0	71.6	77.9
Southern Latin America	IS	66.1	82.5	16.4	70.7	85.7	15.0	60.8	78.1	17.3	87.6	72.5	77.6
Southern Sub-Saharan Africa	IS	79.6	71.3	-8.3	86.0	78.4	-7.5	67.0	59.3	-7.7	77.5	100.0	71.1
Tropical Latin America	IS	46.7	70.1	23.4	57.6	77.1	19.5	33.7	61.3	27.6	87.7	72.7	77.8
Western Europe	IS	62.4	85.9	23.6	67.8	87.6	19.8	55.9	83.6	27.7	87.5	72.5	77.9
Western Sub-Saharan Africa	IS	64.3	66.7	2.4	77.3	79.2	1.9	45.9	47.8	2.0	87.6	72.8	77.8
Andean Latin America	SAH	70.6	76.6	6.0	67.1	71.3	4.2	71.6	79.7	8.1	84.9	12.9	80.5
Australasia	SAH	69.3	84.2	14.9	66.2	82.6	16.4	69.6	82.1	12.5	84.2	10.5	80.4
Caribbean	SAH	66.7	71.7	5.0	63.3	67.7	4.4	66.8	72.4	5.7	84.6	12.1	80.1
Central Asia	SAH	70.6	66.8	-3.7	70.0	67.4	-2.7	68.6	65.0	-3.6	85.2	12.3	75.9
Central Europe	SAH	62.9	72.9	10.0	61.7	72.2	10.5	61.7	71.4	9.7	84.8	12.3	80.3
Central Latin America	SAH	82.0	79.3	-2.7	78.5	77.2	-1.3	82.2	77.9	-4.3	85.2	12.3	80.2

Central Sub-Saharan Africa	SAH	77.8	81.0	3.1	76.7	79.8	3.1	77.3	79.7	2.4	84.2	10.8	80.5
East Asia	SAH	2.3	67.4	65.1	2.4	69.3	66.9	2.2	63.8	61.5	84.9	12.6	80.5
Eastern Europe	SAH	65.0	64.6	-0.4	66.3	66.1	-0.2	60.3	61.4	1.1	84.9	12.9	80.3
Eastern Sub-Saharan Africa	SAH	75.5	82.1	6.6	78.6	82.8	4.2	72.3	80.1	7.8	84.8	12.6	80.4
Global	SAH	51.7	74.3	22.5	51.8	73.6	21.8	50.0	72.7	22.7	84.7	12.1	80.5
High SDI	SAH	73.5	88.2	14.7	70.5	86.3	15.8	74.1	86.6	12.5	83.5	4.5	80.6
High-income Asia Pacific	SAH	73.1	91.0	18.0	69.8	88.1	18.4	74.3	90.8	16.5	100.0	49.7	80.9
High-income North America	SAH	75.9	84.5	8.6	73.4	84.8	11.4	74.4	79.2	4.8	87.8	26.3	77.0
High-middle SDI	SAH	49.8	75.0	25.1	52.1	75.3	23.3	45.2	72.3	27.1	84.8	12.2	80.5
Low SDI	SAH	66.7	73.1	6.3	67.0	71.2	4.2	65.9	73.4	7.6	84.8	12.7	80.5
Low-middle SDI	SAH	56.3	66.5	10.2	57.0	63.9	6.9	55.1	67.6	12.5	84.8	12.6	80.5
Middle SDI	SAH	26.8	68.6	41.8	25.6	68.4	42.8	28.3	66.9	38.6	84.8	12.3	80.5
North Africa and Middle East	SAH	59.7	78.8	19.2	63.1	77.8	14.7	54.0	77.3	23.3	84.7	12.3	80.4
Oceania	SAH	56.7	66.1	9.4	47.9	56.9	9.0	64.7	73.5	8.8	84.7	12.5	80.4
South Asia	SAH	54.0	65.1	11.1	55.6	61.9	6.3	53.2	67.1	13.9	84.8	12.9	80.5
Southeast Asia	SAH	60.6	70.2	9.6	60.3	69.3	9.0	58.9	69.2	10.3	84.8	12.4	80.5
Southern Latin America	SAH	59.4	79.9	20.5	57.7	77.2	19.5	59.0	79.6	20.6	84.7	12.3	80.4
Southern Sub-Saharan Africa	SAH	88.7	85.0	-3.8	86.4	82.3	-4.0	87.6	84.5	-3.1	85.5	13.8	80.3
Tropical Latin America	SAH	57.7	63.2	5.5	54.2	58.2	4.0	58.3	65.9	7.6	84.9	12.5	80.4
Western Europe	SAH	76.9	87.3	10.4	72.5	84.3	11.8	79.3	87.2	7.8	84.6	11.6	80.4
Western Sub-Saharan Africa	SAH	81.6	87.2	5.6	80.3	85.2	4.9	81.4	86.8	5.4	84.8	12.4	80.4
Andean Latin America	Stroke	61.4	76.8	15.3	65.8	79.6	13.9	55.1	72.4	17.3	28.1	2.2	33.9
Australasia	Stroke	72.0	88.5	16.5	71.5	87.1	15.5	72.0	89.3	17.2	28.0	2.3	34.0
Caribbean	Stroke	48.0	59.4	11.4	51.6	64.1	12.5	42.7	52.6	9.9	28.0	2.3	34.3
Central Asia	Stroke	55.4	60.5	5.1	61.0	66.5	5.5	46.3	51.7	5.3	27.9	2.2	33.7
Central Europe	Stroke	43.1	63.0	19.9	47.1	67.1	20.0	37.9	58.0	20.1	28.1	2.2	33.9
Central Latin America	Stroke	68.5	78.2	9.7	71.9	82.7	10.7	62.8	71.4	8.5	28.1	2.2	33.7
Central Sub-Saharan Africa	Stroke	51.4	54.4	3.0	57.6	60.9	3.3	42.0	43.4	1.4	28.2	2.1	33.0
East Asia	Stroke	32.3	59.9	27.6	40.5	68.1	27.6	21.2	48.8	27.6	25.9	2.8	50.1
Eastern Europe	Stroke	43.4	58.4	15.0	48.7	64.2	15.5	35.7	50.9	15.1	28.1	2.3	34.1
Eastern Sub-Saharan Africa	Stroke	48.7	58.8	10.0	57.5	65.6	8.1	37.0	48.8	11.8	28.1	2.2	33.8
Global	Stroke	51.6	65.7	14.2	55.9	70.4	14.5	45.9	59.6	13.7	28.1	2.3	33.8
High SDI	Stroke	72.4	90.2	17.8	74.5	91.9	17.4	69.4	88.0	18.6	28.1	2.3	34.0
High-income Asia Pacific	Stroke	72.2	92.6	20.5	73.7	94.6	20.9	69.6	90.4	20.7	28.0	2.3	34.0
High-income North America	Stroke	85.5	99.8	14.3	86.8	99.7	12.9	83.3	99.8	16.5	29.1	2.0	32.8
High-middle SDI	Stroke	44.9	64.3	19.4	50.4	69.6	19.2	38.0	57.4	19.4	28.2	2.3	33.5
Low SDI	Stroke	46.6	54.4	7.8	51.4	59.0	7.5	40.0	48.0	8.0	28.1	2.2	33.6
Low-middle SDI	Stroke	44.2	53.5	9.3	46.9	57.1	10.2	40.2	48.6	8.4	28.2	2.3	33.1
Middle SDI	Stroke	41.1	59.6	18.5	47.2	66.5	19.3	33.3	50.5	17.2	28.9	2.4	43.0
North Africa and Middle East	Stroke	42.6	60.6	17.9	44.1	60.6	16.6	40.2	59.5	19.3	28.1	2.3	34.0
Oceania	Stroke	37.7	44.1	6.4	38.9	44.7	5.8	34.9	42.0	7.1	28.1	2.2	33.8
South Asia	Stroke	46.9	55.0	8.1	48.2	56.8	8.6	43.9	51.8	7.9	28.2	2.2	33.3
Southeast Asia	Stroke	45.0	52.4	7.4	49.9	58.9	9.1	38.6	44.4	5.7	28.1	2.3	33.7
Southern Latin America	Stroke	64.1	81.6	17.5	67.3	84.4	17.0	59.7	77.7	18.0	28.1	2.2	33.8
Southern Sub-Saharan Africa	Stroke	71.5	63.8	-7.7	76.4	68.9	-7.5	62.0	54.7	-7.3	29.2	1.4	30.1
Tropical Latin America	Stroke	46.1	67.6	21.5	51.6	71.5	19.9	39.0	62.4	23.4	28.1	2.2	33.7
Western Europe	Stroke	66.8	87.0	20.2	68.7	87.5	18.8	63.7	85.9	22.2	28.1	2.3	34.0
Western Sub-Saharan Africa	Stroke	53.1	60.9	7.9	57.4	65.6	8.2	47.4	54.2	6.8	28.1	2.3	33.7

QCI, quality of care index; SDI, sociodemographic index; ICH, intracerebral hemorrhage; SAH, subarachnoid hemorrhage; IS, ischemic stroke. Changes, it represents the difference between the QCI of 2021 and that of 1990

Table S5 A world map depicting the QCI and its changes in each country for stroke, ICH, SAH, and IS in both sexes, females, and males in 1990 and 2021

		Age-Standardized QCI									Age-Standardized QCI for percent change		
		both			female			male			both	female	male
**Location**	**cause**	**1990**	**2021**	**changes**	**1990**	**2021**	**changes**	**1990**	**2021**	**changes**	**2021**	**2021**	**2021**
Singapore	ICH	84.9	100.0	15.1	85.9	100.0	14.1	84.0	100.0	16.0	46.5	72.9	76.1
Canada	ICH	87.4	95.6	8.2	89.6	95.9	6.3	84.7	95.2	10.5	46.9	72.2	69.0
Japan	ICH	84.1	94.9	10.8	87.5	98.9	11.4	80.2	91.8	11.5	46.5	72.4	74.2
Republic of Korea	ICH	71.6	93.5	21.9	76.2	96.2	20.0	65.9	90.1	24.2	46.5	73.0	76.4
Sweden	ICH	81.7	93.3	11.6	82.9	92.6	9.7	80.9	94.2	13.3	46.8	72.3	65.3
Austria	ICH	83.1	92.8	9.8	84.4	93.1	8.7	82.1	92.5	10.4	46.5	69.4	76.3
Switzerland	ICH	76.7	90.9	14.2	78.5	91.2	12.8	75.4	90.7	15.2	46.6	72.3	74.5
United States of America	ICH	82.9	90.7	7.8	85.7	92.5	6.8	79.5	89.1	9.6	46.4	70.6	82.9
Ireland	ICH	78.7	90.6	11.9	80.1	91.6	11.5	77.7	89.4	11.7	46.5	72.9	75.4
Finland	ICH	79.6	90.1	10.6	81.4	90.8	9.4	78.4	89.8	11.4	46.8	72.3	71.9
Iceland	ICH	80.3	89.7	9.4	81.7	91.4	9.7	78.9	88.2	9.3	46.5	72.8	75.7
San Marino	ICH	74.0	88.5	14.5	75.2	87.9	12.7	74.1	89.5	15.4	46.6	72.4	73.8
Andorra	ICH	81.6	88.5	6.9	81.3	87.7	6.4	82.3	89.6	7.3	46.6	72.4	73.6
Chile	ICH	73.7	88.4	14.8	79.3	92.2	12.8	66.1	83.3	17.2	46.6	72.4	74.5
Germany	ICH	75.5	88.2	12.7	79.2	90.4	11.2	70.9	85.8	14.9	46.6	72.2	72.8
Malta	ICH	71.2	87.3	16.1	73.0	88.2	15.2	69.7	86.2	16.6	46.6	72.9	74.3
Puerto Rico	ICH	77.3	86.9	9.6	83.3	93.1	9.8	70.3	78.9	8.6	46.6	71.0	75.9
Kuwait	ICH	79.9	86.4	6.5	81.8	91.6	9.8	77.6	82.5	4.9	46.3	72.0	81.6
Norway	ICH	77.4	86.3	8.9	81.8	87.8	5.9	71.9	84.9	13.0	46.6	72.6	72.0
Costa Rica	ICH	77.0	85.9	9.0	80.1	90.3	10.2	73.1	80.3	7.2	46.5	72.6	75.3
Bermuda	ICH	71.5	85.4	13.8	79.0	94.4	15.4	61.8	74.5	12.6	46.6	72.5	75.5
France	ICH	69.7	85.2	15.5	75.3	87.2	11.8	63.7	83.2	19.5	46.8	70.5	68.5
Netherlands	ICH	75.9	84.6	8.7	79.7	86.5	6.8	71.5	82.2	10.8	46.6	73.1	75.0
Australia	ICH	74.9	84.4	9.5	76.5	85.6	9.1	73.4	83.3	9.9	46.6	72.5	75.1
Monaco	ICH	68.6	82.8	14.2	72.9	85.3	12.4	64.1	80.4	16.3	46.6	72.7	74.6
New Zealand	ICH	73.2	82.2	9.0	76.3	85.1	8.9	69.5	78.9	9.4	46.6	72.6	75.4
Uruguay	ICH	73.3	82.0	8.8	79.3	87.4	8.1	65.6	74.5	8.9	46.5	73.1	76.2
Taiwan (Province of China)	ICH	62.2	82.0	19.8	67.0	88.1	21.2	57.5	76.1	18.6	46.5	72.3	75.2
United Kingdom	ICH	76.5	81.7	5.2	78.6	83.5	4.9	74.8	80.0	5.2	46.5	72.5	75.8
Denmark	ICH	71.1	81.4	10.3	73.9	85.1	11.2	68.5	77.4	8.8	46.6	72.9	75.7
Spain	ICH	71.4	81.2	9.8	74.2	85.1	10.9	68.6	76.8	8.2	46.5	72.6	76.5
Cyprus	ICH	60.8	80.4	19.6	65.1	82.4	17.3	56.9	78.5	21.7	46.5	72.9	75.0
Argentina	ICH	71.5	80.3	8.8	77.4	85.9	8.5	64.6	73.6	8.9	46.5	72.4	76.5
Czechia	ICH	59.6	80.2	20.5	67.3	85.4	18.2	51.3	73.9	22.7	46.5	72.4	75.7
Brunei Darussalam	ICH	76.2	80.2	4.0	77.3	81.9	4.6	75.4	78.3	3.0	46.5	73.2	76.8
Belgium	ICH	70.1	80.1	10.0	74.1	84.0	9.9	65.5	75.5	10.0	46.5	72.3	75.8
Israel	ICH	64.8	79.6	14.8	67.0	83.6	16.6	63.0	75.5	12.5	46.5	72.6	75.6

Colombia	ICH	64.7	79.6	14.8	68.6	84.3	15.7	60.6	73.1	12.5	46.5	72.8	76.1
Luxembourg	ICH	63.9	79.4	15.5	68.3	83.9	15.6	59.2	74.4	15.2	46.5	72.8	76.0
Slovenia	ICH	65.7	79.4	13.7	72.9	85.1	12.2	57.6	72.2	14.6	46.5	72.7	76.1
Qatar	ICH	59.6	78.7	19.1	66.8	81.0	14.2	54.0	77.4	23.5	46.5	73.0	75.8
Italy	ICH	70.3	78.4	8.1	75.0	82.4	7.4	65.2	74.1	8.9	46.6	72.7	75.3
Mexico	ICH	72.1	78.3	6.2	75.0	83.1	8.1	68.2	72.3	4.2	46.5	72.7	76.3
Estonia	ICH	60.4	77.2	16.8	64.7	84.9	20.1	56.2	67.2	10.9	46.5	72.3	76.6
Guam	ICH	66.2	77.0	10.7	68.6	80.9	12.3	63.8	72.6	8.8	46.6	69.6	68.8
Lebanon	ICH	44.6	76.7	32.1	59.5	81.2	21.7	27.5	71.6	44.1	46.5	70.1	74.4
Iran (Islamic Republic of)	ICH	65.1	76.6	11.5	68.9	77.9	9.0	61.5	75.3	13.8	46.5	72.7	74.8
Hungary	ICH	56.1	74.8	18.7	61.8	82.2	20.4	50.4	66.1	15.7	46.5	72.8	76.6
Panama	ICH	67.4	74.6	7.2	73.3	82.7	9.4	61.1	65.9	4.8	46.5	72.6	76.2
Peru	ICH	63.9	74.2	10.3	69.2	77.9	8.7	58.3	70.5	12.2	46.5	72.5	76.0
Oman	ICH	58.9	74.2	15.3	66.8	78.4	11.6	49.3	68.9	19.6	46.5	69.2	75.1
Greece	ICH	62.5	73.9	11.4	63.8	76.6	12.8	62.8	71.2	8.4	46.5	72.6	76.1
Nicaragua	ICH	65.5	73.7	8.2	70.4	78.2	7.8	59.1	67.8	8.7	46.5	72.9	76.0
United States Virgin Islands	ICH	55.6	73.7	18.0	64.8	84.2	19.4	44.9	61.5	16.5	46.4	72.0	75.4
Armenia	ICH	56.5	73.4	16.9	60.4	79.2	18.8	53.3	66.4	13.2	46.5	72.9	75.9
Ecuador	ICH	59.3	73.2	13.9	64.6	77.8	13.3	54.0	68.1	14.1	46.5	72.5	75.7
Portugal	ICH	56.1	73.0	16.9	60.6	78.3	17.8	51.8	67.5	15.7	46.5	73.1	76.5
Lithuania	ICH	65.6	72.5	6.9	68.8	78.2	9.3	62.9	66.3	3.5	46.6	72.5	75.2
Cabo Verde	ICH	72.3	72.4	0.0	70.2	72.6	2.3	75.2	72.4	-2.8	46.5	66.1	77.8
Turkey	ICH	61.5	72.3	10.9	65.7	76.0	10.3	57.6	68.7	11.2	46.5	73.0	76.3
Jordan	ICH	56.8	72.2	15.4	58.1	72.7	14.6	57.4	72.0	14.5	46.5	71.9	75.3
United Arab Emirates	ICH	65.4	72.1	6.6	69.5	51.8	-17.6	62.8	76.4	13.7	46.5	76.6	75.4
Cook Islands	ICH	57.3	71.6	14.3	64.9	78.3	13.4	49.9	64.9	14.9	46.6	70.7	73.0
El Salvador	ICH	66.0	71.5	5.6	70.5	77.0	6.6	60.8	63.8	3.0	46.5	72.8	76.9
Greenland	ICH	62.6	70.8	8.2	67.1	76.0	8.9	59.7	66.5	6.7	46.5	73.0	76.4
Mauritius	ICH	57.4	69.9	12.5	65.4	76.4	11.0	49.3	63.3	14.0	46.5	72.9	76.1
Poland	ICH	55.5	69.4	13.9	62.4	78.5	16.0	48.3	59.1	10.9	46.5	71.9	76.5
Ukraine	ICH	64.2	69.4	5.2	69.1	76.1	7.1	58.8	61.7	3.0	46.5	72.4	75.2
Thailand	ICH	62.2	68.8	6.5	65.0	73.3	8.3	59.6	64.7	5.1	46.5	72.6	76.4
Guatemala	ICH	66.8	68.3	1.6	69.4	72.1	2.7	64.4	64.2	-0.2	46.5	72.6	77.1
Cuba	ICH	63.4	68.3	4.9	68.4	75.9	7.6	58.0	59.8	1.8	46.5	72.6	76.8
Tunisia	ICH	56.1	68.2	12.2	58.9	70.6	11.7	53.6	66.0	12.4	46.5	91.9	75.0
Bahrain	ICH	49.7	68.0	18.4	56.9	72.4	15.5	43.5	64.5	21.0	46.5	72.7	75.3
Belarus	ICH	66.8	68.0	1.2	69.0	74.2	5.2	64.9	60.8	-4.1	46.5	72.4	77.7
Maldives	ICH	48.4	67.9	19.5	44.2	65.7	21.4	50.4	67.7	17.3	46.5	73.1	76.3
Seychelles	ICH	59.4	67.9	8.5	65.0	70.9	5.9	53.7	65.3	11.6	46.5	72.8	75.9
Slovakia	ICH	50.9	67.4	16.4	59.4	75.2	15.7	42.2	58.8	16.6	46.5	72.8	76.0
Sao Tome and Principe	ICH	66.7	67.3	0.7	63.9	64.4	0.5	72.2	72.9	0.7	46.5	67.6	77.0
Croatia	ICH	51.8	67.1	15.2	61.2	76.7	15.5	40.5	55.8	15.3	46.5	72.3	76.5
Malaysia	ICH	60.5	66.3	5.8	62.9	68.1	5.1	58.2	64.6	6.4	46.5	71.6	75.9
Bhutan	ICH	57.2	65.8	8.7	59.4	67.5	8.1	55.5	64.4	8.9	46.5	71.6	75.4
Nigeria	ICH	51.7	65.6	13.9	48.7	63.7	15.0	54.9	68.2	13.3	46.5	71.3	75.2
Algeria	ICH	59.6	65.5	5.9	56.6	61.5	4.9	61.3	69.0	7.7	46.5	86.6	76.3
Latvia	ICH	51.4	65.5	14.1	56.8	73.1	16.3	46.2	56.8	10.6	46.5	72.5	76.4
Barbados	ICH	57.5	65.4	7.9	65.3	72.6	7.3	49.2	57.2	8.0	46.5	72.2	76.0

Uzbekistan	ICH	52.7	65.4	12.7	56.8	68.9	12.1	48.8	61.2	12.4	47.3	72.6	68.0
Venezuela (Bolivarian Republic of)	ICH	62.5	65.4	2.9	68.4	74.5	6.2	55.8	53.7	-2.1	46.5	73.1	77.3
Bosnia and Herzegovina	ICH	52.4	65.3	12.9	57.0	71.0	14.0	48.7	59.3	10.6	46.5	71.9	75.8
Bahamas	ICH	53.6	65.2	11.5	61.9	72.2	10.3	44.8	57.4	12.6	46.5	72.6	75.5
Libya	ICH	65.7	65.0	-0.7	66.6	65.0	-1.5	66.0	66.8	0.8	46.5	73.4	77.1
Pakistan	ICH	65.1	64.5	-0.6	66.3	65.1	-1.2	63.7	64.3	0.6	46.5	70.8	76.9
Trinidad and Tobago	ICH	53.3	64.1	10.7	60.1	72.5	12.4	45.9	54.4	8.6	46.5	72.8	76.1
Brazil	ICH	53.1	64.0	10.8	61.0	71.1	10.1	44.5	55.8	11.3	46.5	72.7	76.6
India	ICH	57.7	63.8	6.1	62.9	67.1	4.2	52.2	60.5	8.3	46.6	72.5	64.1
Kyrgyzstan	ICH	54.8	63.5	8.7	57.6	69.0	11.4	51.6	57.3	5.7	46.5	72.9	76.9
Tonga	ICH	61.8	63.2	1.3	66.5	66.9	0.4	57.0	59.6	2.6	46.5	74.7	75.9
Syrian Arab Republic	ICH	60.1	63.0	2.9	62.0	65.1	3.1	58.5	60.7	2.2	46.5	74.1	76.8
Palau	ICH	57.4	62.0	4.6	68.7	69.9	1.2	46.5	55.0	8.5	46.5	74.1	75.4
Georgia	ICH	55.9	61.3	5.4	61.3	67.4	6.1	49.3	54.2	4.9	46.5	64.9	76.7
Russian Federation	ICH	55.6	61.1	5.5	60.5	68.0	7.5	50.5	53.8	3.3	46.5	72.2	76.5
Northern Mariana Islands	ICH	56.3	61.0	4.7	53.0	63.2	10.1	56.1	57.9	1.8	46.5	72.4	76.8
Kiribati	ICH	64.4	60.9	-3.5	68.0	65.2	-2.8	59.7	55.5	-4.1	46.5	75.4	77.8
Coted'Ivoire	ICH	60.7	60.8	0.1	58.4	57.5	-0.9	61.7	63.7	2.0	46.5	74.0	76.7
Saint Lucia	ICH	46.1	60.5	14.3	55.9	72.2	16.3	35.3	48.4	13.1	46.5	72.7	76.2
Palestine	ICH	46.7	60.2	13.5	53.7	66.0	12.3	39.7	54.3	14.6	46.5	67.8	75.9
Dominican Republic	ICH	52.5	60.2	7.7	59.0	67.4	8.3	45.7	52.7	7.0	47.4	74.7	100.0
Belize	ICH	53.0	59.8	6.8	58.7	65.0	6.2	47.4	54.7	7.3	46.5	73.1	76.0
Mauritania	ICH	52.8	59.8	7.0	43.2	50.9	7.7	64.8	69.7	4.9	46.5	73.5	76.4
Mongolia	ICH	45.4	59.8	14.3	54.7	66.8	12.1	33.9	51.0	17.1	45.9	74.2	55.6
Saudi Arabia	ICH	43.3	59.6	16.3	46.4	63.6	17.2	39.9	55.9	16.0	46.5	71.4	74.6
Nepal	ICH	51.6	59.6	8.0	53.9	64.6	10.7	49.9	54.1	4.2	46.5	72.1	76.3
Botswana	ICH	42.2	59.1	16.9	40.8	60.5	19.7	43.8	58.9	15.0	46.4	76.8	76.0
Iraq	ICH	51.0	58.7	7.7	57.6	63.8	6.1	44.5	53.6	9.1	46.5	70.3	75.7
Antigua and Barbuda	ICH	47.4	58.5	11.1	59.2	69.6	10.4	34.9	46.4	11.5	46.5	72.3	76.2
Bulgaria	ICH	43.6	58.3	14.7	49.0	65.8	16.8	38.4	50.3	11.9	46.5	64.1	76.7
Morocco	ICH	51.8	58.1	6.3	50.5	56.5	6.0	55.2	61.1	5.9	46.5	74.4	76.2
Guinea	ICH	55.6	57.8	2.2	48.2	51.1	2.8	63.7	63.9	0.1	46.4	74.3	79.0
Mali	ICH	52.3	57.3	5.0	43.4	45.4	2.0	61.8	66.0	4.2	46.5	73.3	75.7
American Samoa	ICH	56.9	57.3	0.4	61.5	61.3	-0.2	52.2	53.4	1.1	46.5	73.1	76.9
Burkina Faso	ICH	54.7	57.1	2.4	59.3	61.0	1.7	50.5	53.7	3.2	46.4	75.4	76.9
Benin	ICH	51.5	57.1	5.6	42.5	51.4	8.8	60.3	64.3	4.1	46.5	73.2	76.1
Paraguay	ICH	53.6	57.1	3.5	58.1	64.1	6.0	49.2	50.0	0.7	46.5	72.7	76.9
Niue	ICH	55.9	56.9	1.1	62.4	64.2	1.8	48.4	48.7	0.2	46.5	72.6	76.9
Jamaica	ICH	51.9	56.8	5.0	58.0	64.4	6.5	45.7	48.6	3.0	46.5	72.5	76.6
Sierra Leone	ICH	56.0	56.7	0.7	51.6	50.9	-0.7	60.2	64.1	3.9	46.5	74.5	76.2
Serbia	ICH	46.4	56.7	10.3	52.8	64.8	12.1	40.2	46.8	6.6	46.5	72.9	76.9
Ghana	ICH	53.4	56.7	3.2	49.5	53.6	4.1	59.9	61.6	1.6	46.5	73.8	76.2
Grenada	ICH	43.5	56.5	13.0	52.3	65.1	12.8	33.0	47.6	14.5	46.5	72.6	76.2
Senegal	ICH	54.7	56.5	1.8	50.3	51.3	1.0	59.2	62.1	2.9	46.5	73.7	76.1
Cameroon	ICH	54.0	56.4	2.4	53.6	54.3	0.8	55.1	59.0	3.9	46.5	78.0	75.3
Solomon Islands	ICH	55.4	56.2	0.9	61.3	62.3	1.0	49.3	49.8	0.5	46.4	83.6	77.4
Bangladesh	ICH	46.1	56.1	10.0	50.6	59.6	9.0	41.5	52.8	11.4	46.5	74.6	75.6
Sri Lanka	ICH	47.6	55.9	8.3	55.6	63.0	7.4	40.1	49.0	8.9	46.5	72.2	76.1

Azerbaijan	ICH	51.8	55.9	4.1	57.4	58.8	1.5	45.2	53.5	8.2	47.2	74.1	30.8
Republic of Moldova	ICH	44.7	55.6	10.9	48.7	61.3	12.6	41.6	49.9	8.4	46.5	71.5	76.6
Kazakhstan	ICH	61.8	55.4	-6.3	65.6	61.8	-3.8	58.2	48.8	-9.3	46.5	73.4	78.4
Philippines	ICH	40.7	55.3	14.7	48.0	62.9	14.8	32.9	47.5	14.6	46.5	74.2	78.5
Chad	ICH	56.0	54.6	-1.3	52.3	50.2	-2.1	60.1	57.6	-2.5	46.5	76.6	78.5
Saint Vincent and the Grenadines	ICH	39.7	54.6	14.9	48.9	64.4	15.6	29.5	44.7	15.2	46.3	73.0	77.2
China	ICH	41.6	54.4	12.8	51.3	63.2	11.9	29.9	44.0	14.1	46.5	74.8	74.7
Equatorial Guinea	ICH	33.9	54.2	20.3	39.7	55.7	16.0	28.5	53.7	25.2	46.5	73.0	76.0
Sudan	ICH	34.9	54.1	19.2	36.5	50.6	14.1	34.5	58.0	23.5	46.5	76.1	75.8
Samoa	ICH	50.9	54.1	3.2	61.5	60.8	-0.7	39.1	47.3	8.1	46.5	71.5	75.6
Vanuatu	ICH	48.6	53.9	5.3	58.2	61.1	2.8	39.5	46.8	7.3	46.5	69.1	68.4
Tajikistan	ICH	51.7	53.8	2.1	55.4	55.5	0.1	48.9	52.3	3.4	100.0	75.4	72.2
United Republic of Tanzania	ICH	43.9	53.6	9.6	51.5	56.6	5.1	36.5	50.8	14.3	46.5	73.1	75.6
Niger	ICH	54.9	53.5	-1.5	53.6	50.7	-3.0	56.7	57.1	0.4	46.5	73.8	77.0
Turkmenistan	ICH	59.9	53.3	-6.5	63.6	61.5	-2.1	56.0	44.8	-11.2	48.1	74.9	89.3
Saint Kitts and Nevis	ICH	45.8	53.3	7.5	53.0	63.1	10.1	37.2	43.7	6.5	46.5	73.3	76.7
Romania	ICH	45.2	52.8	7.6	49.8	62.6	12.8	41.5	42.4	0.9	46.5	72.5	76.9
Bolivia (Plurinational State of)	ICH	40.0	52.4	12.3	44.4	56.1	11.8	37.0	49.4	12.4	46.5	72.9	76.4
Gabon	ICH	49.3	52.4	3.1	54.5	55.6	1.1	43.8	49.3	5.4	46.5	73.8	76.6
Tokelau	ICH	45.7	52.0	6.3	56.4	60.8	4.3	31.8	42.0	10.2	46.5	71.4	75.9
Egypt	ICH	13.3	52.0	38.7	25.8	52.1	26.3	0.0	49.5	49.5	46.4	74.0	73.4
Liberia	ICH	56.3	51.9	-4.5	48.3	44.0	-4.3	63.3	60.5	-2.9	46.5	73.7	77.4
Suriname	ICH	47.6	51.7	4.0	53.1	60.4	7.3	42.5	42.5	0.0	46.4	72.3	77.7
Gambia	ICH	55.6	51.6	-4.0	51.1	47.5	-3.6	59.6	56.8	-2.8	46.5	78.4	77.8
Kenya	ICH	56.0	51.3	-4.7	59.2	54.8	-4.3	52.7	47.5	-5.2	46.5	74.7	78.5
North Macedonia	ICH	48.2	51.2	3.1	54.6	56.6	2.1	41.6	46.4	4.8	46.4	0.0	76.8
Ethiopia	ICH	43.1	51.0	8.0	51.3	56.2	4.9	35.0	45.9	10.9	46.5	73.2	76.6
Togo	ICH	54.1	50.9	-3.3	48.2	48.3	0.1	61.0	55.7	-5.3	46.5	73.7	78.5
Micronesia (Federated States of)	ICH	46.4	50.7	4.3	52.7	57.1	4.4	39.8	43.9	4.1	46.5	72.3	76.7
Tuvalu	ICH	39.9	49.8	9.9	47.9	55.9	8.0	31.4	43.8	12.4	46.5	65.3	75.4
South Africa	ICH	64.3	48.7	-15.6	66.3	51.9	-14.5	63.3	47.3	-16.0	46.5	80.3	78.3
Comoros	ICH	42.4	48.4	6.0	46.2	51.6	5.4	39.2	46.0	6.8	46.5	72.9	76.6
Djibouti	ICH	43.9	48.1	4.2	48.6	51.7	3.1	39.0	44.5	5.5	46.5	72.2	76.6
Uganda	ICH	37.0	47.9	10.9	50.1	57.0	7.0	24.9	37.9	13.0	46.5	72.4	76.0
Fiji	ICH	49.4	47.8	-1.6	57.4	55.3	-2.1	40.0	38.8	-1.2	46.5	73.8	76.9
Marshall Islands	ICH	44.5	47.8	3.2	55.1	54.8	-0.2	34.0	41.0	6.9	46.5	72.7	75.5
Dominica	ICH	40.8	46.9	6.1	52.5	58.1	5.6	26.7	36.1	9.4	46.5	73.0	76.4
Lao People's Democratic Republic	ICH	31.0	46.3	15.3	34.1	49.1	15.0	28.7	43.8	15.1	46.5	71.0	75.5
Indonesia	ICH	50.7	46.0	-4.7	54.3	50.3	-4.0	48.5	42.8	-5.7	46.5	71.2	78.2
Guyana	ICH	30.6	45.2	14.6	38.3	53.3	15.1	22.8	36.6	13.8	46.5	72.9	76.6
Viet Nam	ICH	40.4	44.8	4.4	47.5	55.3	7.8	33.1	34.1	1.0	46.5	73.3	76.8
Angola	ICH	40.7	44.6	3.9	46.6	48.6	2.0	35.0	41.3	6.3	46.5	73.6	76.4
Cambodia	ICH	34.1	43.9	9.7	35.6	48.1	12.5	35.5	40.6	5.1	46.5	74.6	76.0
Nauru	ICH	48.8	42.8	-6.0	54.7	49.5	-5.3	43.5	36.2	-7.3	46.5	72.7	77.8
Congo	ICH	40.2	42.6	2.3	41.1	41.7	0.6	40.7	45.7	4.9	46.5	73.3	76.8
Guinea-Bissau	ICH	42.9	42.4	-0.4	41.9	39.5	-2.4	44.2	47.8	3.6	46.5	75.9	76.8
Democratic People's Republic of Korea	ICH	47.3	42.1	-5.2	54.5	50.6	-3.9	38.4	32.7	-5.7	46.5	75.0	78.2
Rwanda	ICH	31.3	42.0	10.7	41.6	52.4	10.8	19.8	29.0	9.2	46.5	73.0	76.8

Myanmar	ICH	30.6	41.8	11.2	33.2	48.2	15.0	28.3	35.3	7.0	46.5	72.3	76.2
Democratic Republic of the Congo	ICH	46.1	41.6	-4.5	47.2	46.1	-1.1	45.1	37.2	-7.9	46.5	73.5	77.8
Afghanistan	ICH	35.5	39.8	4.3	50.2	44.6	-5.6	17.6	36.2	18.6	46.5	73.6	76.1
Namibia	ICH	40.5	39.2	-1.4	41.9	43.8	1.9	40.8	35.7	-5.1	46.5	73.9	77.6
Yemen	ICH	29.3	39.1	9.8	38.4	41.4	3.0	19.6	38.2	18.6	46.5	75.0	76.1
Timor-Leste	ICH	33.4	39.0	5.6	31.9	41.1	9.2	36.4	37.7	1.4	46.4	73.4	76.8
Albania	ICH	33.2	38.9	5.8	43.4	43.8	0.4	19.6	35.3	15.7	46.4	75.1	76.0
Burundi	ICH	32.8	38.6	5.8	45.3	47.8	2.5	19.4	29.8	10.4	46.5	73.1	76.7
Madagascar	ICH	39.7	37.1	-2.6	46.9	41.8	-5.1	33.2	33.0	-0.2	46.5	92.9	77.1
Zambia	ICH	35.1	36.6	1.5	39.4	39.8	0.4	30.9	32.8	1.8	46.4	71.4	77.7
Eritrea	ICH	28.2	35.7	7.5	35.2	43.0	7.8	20.6	27.6	7.0	46.5	72.5	76.5
Eswatini	ICH	31.0	34.8	3.8	38.0	36.9	-1.1	24.5	34.3	9.8	46.4	71.6	86.8
South Sudan	ICH	35.0	32.2	-2.8	44.1	39.9	-4.2	27.6	25.3	-2.2	46.5	73.2	77.3
Montenegro	ICH	43.9	30.9	-13.0	51.4	41.1	-10.3	35.8	21.1	-14.7	46.5	58.3	78.5
Mozambique	ICH	33.0	29.5	-3.5	41.1	41.7	0.6	25.3	17.9	-7.4	46.6	73.4	78.1
Honduras	ICH	44.8	29.4	-15.4	51.1	35.1	-16.0	38.4	25.3	-13.1	46.5	66.0	78.8
Malawi	ICH	37.3	29.0	-8.2	39.6	33.4	-6.2	35.9	25.7	-10.2	46.5	74.1	78.2
Central African Republic	ICH	31.1	28.9	-2.2	37.8	36.0	-1.8	24.7	21.8	-2.8	46.5	73.5	77.3
Somalia	ICH	26.8	28.6	1.8	36.6	38.2	1.6	16.9	17.9	1.0	46.5	72.8	77.0
Haiti	ICH	22.8	27.9	5.2	31.4	36.2	4.8	14.1	19.4	5.3	46.5	72.3	76.7
Papua New Guinea	ICH	16.7	23.0	6.3	6.2	12.4	6.2	21.8	27.7	6.0	46.5	72.7	76.0
Lesotho	ICH	31.6	22.3	-9.4	41.7	31.4	-10.3	19.4	14.0	-5.4	46.8	72.5	75.0
Zimbabwe	ICH	29.1	0.0	-29.1	28.6	0.0	-28.6	30.7	5.7	-25.0	0.0	72.4	0.0
United States of America	IS	84.9	100.0	15.1	87.8	100.0	12.2	80.9	100.0	19.1	86.5	11.4	78.5
Canada	IS	79.9	96.4	16.5	82.6	96.3	13.7	76.5	95.9	19.4	87.0	12.3	78.1
Singapore	IS	76.5	95.9	19.3	76.1	94.8	18.7	76.8	96.4	19.6	87.8	12.2	77.6
Guam	IS	77.3	94.0	16.8	80.2	97.4	17.2	73.8	88.5	14.7	62.8	10.6	78.7
Israel	IS	77.4	93.6	16.2	76.1	92.6	16.5	78.0	94.1	16.2	87.7	11.3	77.8
Austria	IS	57.7	92.5	34.8	61.9	93.7	31.8	54.4	90.5	36.1	89.8	11.5	78.3
Puerto Rico	IS	68.4	91.2	22.8	76.2	95.7	19.5	57.6	83.3	25.7	86.3	12.0	77.8
Japan	IS	69.5	91.2	21.7	73.0	93.9	20.9	64.5	87.4	22.9	87.4	24.5	77.9
Germany	IS	68.5	89.3	20.8	73.8	91.4	17.6	61.4	86.2	24.8	87.4	12.2	77.7
Taiwan (Province of China)	IS	73.2	89.3	16.1	77.0	92.9	16.0	69.2	84.0	14.8	87.5	9.9	77.5
Iceland	IS	74.3	88.8	14.4	77.6	89.4	11.7	70.1	87.6	17.5	87.7	12.2	77.8
Norway	IS	72.5	88.5	16.0	74.9	89.4	14.5	69.2	86.7	17.5	87.6	11.5	77.6
Sweden	IS	71.7	88.5	16.8	74.1	88.9	14.9	68.9	87.2	18.2	87.4	11.9	77.8
Australia	IS	69.8	88.4	18.7	71.7	87.6	15.9	68.1	88.5	20.4	87.5	8.5	77.8
Spain	IS	55.9	88.1	32.1	61.6	88.1	26.5	49.5	87.3	37.8	87.1	3.2	78.7
France	IS	63.5	87.9	24.4	68.6	89.0	20.4	57.2	86.1	28.9	59.1	16.3	78.0
Finland	IS	70.0	87.7	17.6	72.4	89.3	16.9	68.4	85.4	17.0	87.6	11.2	77.6
Switzerland	IS	64.0	87.1	23.1	68.5	87.9	19.4	58.6	85.5	26.9	87.4	9.6	77.8
Cook Islands	IS	75.4	87.0	11.6	81.8	90.0	8.2	67.5	82.7	15.2	94.0	12.1	77.6
United Kingdom	IS	62.4	85.9	23.5	68.2	88.0	19.8	56.0	82.9	26.9	87.6	12.0	77.7
Republic of Korea	IS	65.7	85.9	20.1	69.0	87.4	18.4	60.9	83.2	22.4	87.8	12.2	77.6
San Marino	IS	68.5	85.7	17.2	71.6	86.5	14.9	64.7	84.1	19.4	87.6	9.9	77.5
Mongolia	IS	84.3	85.1	0.7	91.0	91.8	0.8	72.1	73.6	1.6	88.3	20.0	77.9
Andorra	IS	75.5	85.0	9.5	76.4	85.2	8.9	74.3	84.5	10.2	87.6	9.9	77.6
Belgium	IS	58.8	84.9	26.1	65.1	87.3	22.2	51.5	81.9	30.4	87.2	11.4	78.1

Argentina	IS	66.9	83.8	16.9	71.5	86.7	15.2	61.5	79.8	18.3	87.6	12.4	77.6
Kuwait	IS	78.5	83.6	5.2	78.2	89.7	11.4	77.9	77.3	-0.6	87.1	9.7	69.8
Peru	IS	72.1	83.5	11.5	79.0	88.2	9.2	62.5	76.8	14.3	87.5	12.9	78.1
New Zealand	IS	67.6	83.5	15.8	71.6	84.5	12.9	63.8	82.2	18.4	87.6	12.6	77.9
Netherlands	IS	75.5	83.4	7.9	78.9	85.6	6.6	71.4	80.6	9.2	87.8	12.3	77.7
Costa Rica	IS	75.0	83.1	8.1	80.7	87.9	7.2	66.8	75.7	8.9	87.6	13.3	77.9
Mexico	IS	69.0	82.8	13.8	74.4	87.8	13.4	60.1	75.4	15.3	87.7	10.8	77.9
Denmark	IS	70.6	82.6	12.0	74.4	85.7	11.4	66.1	78.6	12.5	87.8	12.3	77.7
Colombia	IS	68.5	82.3	13.9	75.0	87.9	12.9	59.7	73.6	13.9	87.7	11.5	77.7
Chile	IS	63.3	81.4	18.1	67.9	85.2	17.3	57.8	76.1	18.4	87.5	5.8	77.7
Ecuador	IS	69.6	81.2	11.7	76.4	86.5	10.1	60.3	73.6	13.3	87.4	13.6	77.3
Ireland	IS	59.6	81.1	21.5	64.3	83.9	19.6	54.8	77.6	22.9	87.8	12.5	77.7
Greenland	IS	68.3	80.9	12.7	71.1	83.4	12.3	66.2	77.2	11.0	87.8	6.1	77.5
Thailand	IS	72.2	80.9	8.7	78.9	85.8	6.9	63.1	74.2	11.1	87.6	12.8	78.0
Lebanon	IS	53.3	80.9	27.5	58.5	83.2	24.8	47.4	77.3	29.9	90.8	11.6	78.2
Tonga	IS	80.0	80.3	0.2	85.8	84.9	-0.9	71.9	74.2	2.3	87.2	12.9	58.4
Luxembourg	IS	43.1	80.2	37.1	51.6	84.1	32.6	33.7	74.8	41.2	87.7	11.4	77.7
Nicaragua	IS	72.3	79.8	7.5	80.5	85.3	4.8	59.1	71.5	12.4	87.7	12.2	77.8
Northern Mariana Islands	IS	73.6	79.6	6.0	76.8	84.4	7.6	70.3	74.1	3.8	87.0	11.9	87.8
Uganda	IS	76.7	79.5	2.9	82.3	84.5	2.2	68.1	70.5	2.4	87.0	11.5	76.4
Bermuda	IS	60.1	79.5	19.5	70.3	87.7	17.4	45.8	67.8	22.0	87.6	11.5	77.6
Malta	IS	54.7	79.4	24.7	61.8	81.7	19.9	46.5	76.8	30.3	87.8	11.9	77.8
El Salvador	IS	71.9	79.1	7.3	80.0	85.2	5.2	59.7	68.9	9.1	87.6	12.6	77.6
Brunei Darussalam	IS	74.6	79.1	4.6	75.5	79.7	4.2	72.8	77.3	4.6	87.8	12.6	77.6
Samoa	IS	74.6	78.9	4.2	81.1	84.3	3.2	66.3	71.8	5.5	86.4	0.0	77.8
American Samoa	IS	76.1	78.7	2.5	80.6	81.7	1.0	70.8	75.2	4.4	87.7	13.4	77.5
Fiji	IS	78.3	78.5	0.2	84.4	83.8	-0.6	68.3	69.9	1.6	88.0	12.8	77.7
Czechia	IS	37.9	78.0	40.1	47.8	81.5	33.7	27.0	74.0	47.1	87.5	12.2	77.9
Italy	IS	51.9	78.0	26.1	58.6	80.2	21.6	44.0	75.2	31.2	87.7	11.9	78.0
Niue	IS	74.9	77.6	2.7	80.7	83.7	3.0	67.1	69.2	2.2	87.8	14.4	77.6
Ethiopia	IS	76.0	77.6	1.6	80.1	82.0	1.9	70.5	70.9	0.5	87.8	12.8	77.9
United States Virgin Islands	IS	56.2	77.0	20.8	65.5	84.7	19.2	44.9	64.9	20.0	81.1	21.3	79.6
Malaysia	IS	70.3	76.9	6.6	74.4	79.0	4.6	65.3	74.0	8.7	84.7	12.9	78.6
Estonia	IS	35.2	76.9	41.7	44.4	83.9	39.5	25.3	67.0	41.7	87.6	12.6	77.9
Kenya	IS	80.4	76.7	-3.7	83.7	82.1	-1.6	75.3	68.1	-7.3	85.0	13.6	78.2
Vanuatu	IS	72.0	76.6	4.6	79.8	83.0	3.2	62.2	67.8	5.7	100.0	2.8	80.5
Comoros	IS	72.6	76.2	3.6	79.1	82.1	3.0	62.6	66.3	3.7	87.6	12.1	77.5
Palau	IS	71.8	76.1	4.3	79.9	79.3	-0.6	61.4	71.7	10.3	87.2	13.1	72.1
Monaco	IS	59.9	76.1	16.2	64.0	77.8	13.8	55.9	74.5	18.6	87.7	12.0	77.7
Tokelau	IS	65.9	75.9	10.0	74.0	82.1	8.1	54.0	66.7	12.7	86.1	13.2	78.2
Uruguay	IS	67.2	75.8	8.6	71.9	79.7	7.8	61.8	71.3	9.5	87.8	12.2	77.6
Djibouti	IS	76.0	75.6	-0.3	82.4	83.7	1.3	65.8	63.6	-2.1	87.2	12.1	88.4
Greece	IS	46.6	75.5	28.9	49.7	75.0	25.2	45.0	75.9	30.9	87.5	22.7	77.8
Qatar	IS	64.8	75.3	10.5	69.3	78.3	9.0	59.6	71.6	12.0	87.6	10.9	77.3
Panama	IS	66.2	75.3	9.1	73.9	82.7	8.8	56.2	64.9	8.7	87.7	13.2	77.9
United Republic of Tanzania	IS	76.5	75.2	-1.3	81.7	81.2	-0.4	68.7	66.1	-2.6	87.8	11.9	77.1
Guatemala	IS	65.8	75.1	9.3	73.3	81.6	8.3	55.6	65.4	9.8	87.5	14.1	77.4
Slovakia	IS	64.5	74.7	10.2	70.2	79.4	9.1	58.2	69.1	10.9	87.7	12.7	77.4

Botswana	IS	66.4	74.7	8.3	74.9	81.4	6.5	52.2	62.0	9.8	97.7	9.6	83.3
Kiribati	IS	77.3	74.5	-2.8	86.0	83.7	-2.2	64.2	60.1	-4.1	88.0	9.6	78.1
Jordan	IS	51.7	74.0	22.3	52.4	72.2	19.8	52.1	74.3	22.3	98.8	12.2	78.6
Gabon	IS	74.8	73.9	-0.9	81.4	80.7	-0.8	63.3	62.7	-0.6	87.8	12.8	77.0
Somalia	IS	71.4	73.9	2.5	76.6	79.2	2.7	64.3	65.8	1.5	87.5	9.8	77.3
Solomon Islands	IS	72.8	73.7	0.9	75.7	77.0	1.3	68.8	69.7	1.0	86.6	21.1	81.3
Slovenia	IS	47.3	73.7	26.4	57.5	79.4	22.0	34.7	65.9	31.2	87.7	11.8	78.0
South Sudan	IS	72.5	73.6	1.1	79.1	80.0	0.9	63.9	64.8	0.9	87.6	13.1	75.9
Mauritius	IS	56.3	73.2	16.9	66.9	80.4	13.5	43.6	63.8	20.1	87.7	12.4	77.6
Seychelles	IS	66.2	73.2	7.0	77.0	79.7	2.7	51.5	65.6	14.1	87.6	12.0	77.7
South Africa	IS	82.2	72.7	-9.5	88.0	79.5	-8.5	70.2	61.5	-8.7	87.6	13.2	76.3
Nauru	IS	68.9	72.5	3.7	75.6	79.9	4.3	60.2	62.9	2.7	88.0	13.1	78.2
Bahamas	IS	64.6	72.4	7.8	71.0	76.7	5.8	57.9	67.6	9.7	87.5	11.7	77.8
Sao Tome and Principe	IS	76.1	72.4	-3.8	83.7	80.9	-2.9	58.9	58.9	0.0	93.2	13.5	78.4
Eritrea	IS	69.8	72.4	2.5	75.5	78.9	3.4	62.1	60.6	-1.5	87.8	10.4	78.5
Rwanda	IS	63.8	72.0	8.3	70.1	78.3	8.2	55.5	61.6	6.1	87.7	12.3	77.5
Azerbaijan	IS	68.6	72.0	3.4	77.7	78.4	0.7	51.5	62.6	11.1	73.7	12.0	80.9
Madagascar	IS	70.8	71.8	1.0	79.4	79.1	-0.3	58.0	60.3	2.4	85.9	12.4	77.2
Maldives	IS	63.6	71.6	8.0	66.3	77.0	10.7	58.8	65.3	6.5	87.7	12.4	77.4
Equatorial Guinea	IS	66.9	71.5	4.7	75.9	78.5	2.6	51.0	59.4	8.3	87.7	12.3	78.1
Micronesia (Federated States of)	IS	68.6	71.4	2.8	74.7	77.6	2.8	61.4	63.7	2.3	87.4	10.8	76.8
Marshall Islands	IS	68.6	71.1	2.5	76.5	76.6	0.0	58.7	64.1	5.4	87.2	10.8	77.5
United Arab Emirates	IS	62.8	71.1	8.3	65.8	40.1	-25.7	59.2	75.2	16.1	87.6	13.5	77.3
Dominican Republic	IS	64.7	70.9	6.2	70.5	77.9	7.5	57.6	61.7	4.1	88.6	19.3	78.6
Angola	IS	70.6	70.8	0.1	79.1	78.6	-0.5	56.9	57.0	0.1	88.0	12.5	78.3
Hungary	IS	51.1	70.7	19.6	60.1	77.6	17.4	40.4	62.2	21.8	87.7	12.1	77.5
Venezuela (Bolivarian Republic of)	IS	69.7	70.6	0.9	76.5	79.0	2.6	60.7	58.7	-2.1	87.9	14.0	78.0
Burundi	IS	64.6	70.5	5.9	71.2	76.5	5.3	55.6	62.4	6.8	87.7	12.0	77.5
Brazil	IS	46.5	70.2	23.8	57.4	77.2	19.8	33.4	61.5	28.1	87.7	12.5	77.8
Philippines	IS	62.3	70.0	7.7	68.4	77.7	9.4	53.6	59.1	5.5	88.5	13.6	78.6
Tuvalu	IS	63.2	70.0	6.8	71.5	76.6	5.1	52.6	61.7	9.1	85.7	11.8	79.5
Belize	IS	66.8	69.7	2.9	73.2	74.8	1.6	59.0	63.4	4.4	87.6	12.3	71.9
Zambia	IS	73.0	69.6	-3.4	78.2	76.5	-1.7	65.2	59.2	-6.1	87.6	12.5	78.0
Congo	IS	68.2	69.6	1.4	76.0	76.6	0.6	55.2	58.9	3.7	87.8	12.8	77.6
Burkina Faso	IS	71.7	69.2	-2.5	86.4	83.6	-2.9	48.5	46.2	-2.2	88.0	12.7	77.1
Nigeria	IS	61.5	69.0	7.4	75.6	81.2	5.6	41.7	49.8	8.1	86.8	12.1	77.7
Armenia	IS	60.8	69.0	8.2	68.2	74.4	6.2	51.1	63.2	12.1	87.8	12.3	78.7
Turkey	IS	59.2	68.9	9.7	66.9	73.9	7.0	50.8	63.7	13.0	87.7	12.3	77.5
Croatia	IS	36.1	68.0	32.0	45.3	70.8	25.6	24.6	65.5	40.9	87.3	12.3	78.0
Uzbekistan	IS	67.7	67.8	0.1	76.1	76.4	0.3	54.7	54.8	0.0	86.3	100.0	79.2
Cabo Verde	IS	76.2	67.7	-8.4	85.4	82.5	-2.9	60.6	42.4	-18.2	95.2	11.0	77.8
Portugal	IS	33.0	67.7	34.7	41.7	70.6	29.0	23.2	64.6	41.4	87.8	12.6	77.6
China	IS	53.1	67.3	14.2	64.4	77.1	12.7	38.6	53.3	14.7	88.3	12.7	78.1
Niger	IS	70.8	67.1	-3.7	80.4	78.1	-2.3	56.7	50.7	-6.0	88.1	12.1	78.0
Ghana	IS	67.4	67.1	-0.4	79.7	79.3	-0.4	47.6	46.7	-0.9	87.8	12.2	77.5
Democratic Republic of the Congo	IS	67.4	67.0	-0.3	74.5	74.9	0.4	56.7	54.0	-2.7	87.8	3.8	77.7
Democratic People's Republic of Korea	IS	66.2	67.0	0.9	72.9	74.8	1.9	57.3	56.3	-1.0	87.8	12.5	79.4
Oman	IS	52.6	66.8	14.2	64.0	74.5	10.5	37.7	55.8	18.1	91.4	16.3	78.2

Papua New Guinea	IS	64.2	66.6	2.4	62.1	65.0	2.9	67.2	67.9	0.7	87.5	11.9	77.6
Mauritania	IS	66.5	66.4	-0.2	77.4	76.9	-0.5	49.6	50.6	1.0	87.9	12.4	77.8
Malawi	IS	70.9	66.3	-4.6	76.5	74.1	-2.4	63.2	55.2	-8.0	88.5	12.7	73.7
Lithuania	IS	56.8	66.3	9.4	64.9	73.5	8.6	46.2	56.7	10.5	86.8	10.2	79.1
Mali	IS	67.5	65.8	-1.7	74.5	74.6	0.1	58.1	54.4	-3.8	88.1	12.8	77.9
Cuba	IS	60.8	65.5	4.7	67.8	74.2	6.4	52.0	54.4	2.4	87.7	12.3	77.5
Trinidad and Tobago	IS	49.8	64.8	15.0	61.5	74.0	12.5	36.3	53.8	17.5	87.7	12.8	77.7
Pakistan	IS	65.5	64.8	-0.7	66.6	68.4	1.8	62.9	60.4	-2.6	90.0	13.9	78.9
Iran (Islamic Republic of)	IS	44.4	64.7	20.3	52.9	67.1	14.1	35.7	61.7	26.0	87.4	11.8	78.3
Poland	IS	25.2	64.6	39.4	39.0	71.8	32.8	10.1	56.9	46.8	86.3	12.6	77.6
Albania	IS	60.7	64.6	3.9	71.4	70.7	-0.7	42.9	57.5	14.5	87.5	9.4	77.5
Zimbabwe	IS	70.8	64.4	-6.4	78.8	73.5	-5.3	57.1	47.4	-9.7	87.3	11.1	77.5
Senegal	IS	66.1	64.4	-1.7	79.2	77.7	-1.5	47.5	44.0	-3.6	88.0	12.8	77.9
Antigua and Barbuda	IS	53.6	64.4	10.7	64.9	72.7	7.9	38.1	53.7	15.6	87.5	11.9	77.7
Cameroon	IS	68.1	64.2	-3.9	79.7	77.5	-2.2	51.0	45.0	-6.1	95.8	13.0	78.4
Coted'Ivoire	IS	66.4	63.9	-2.4	79.9	78.5	-1.5	50.4	44.8	-5.5	87.9	12.9	77.5
Bhutan	IS	57.7	63.5	5.8	60.3	68.5	8.2	55.4	57.5	2.1	85.8	12.5	78.3
Paraguay	IS	55.4	63.5	8.1	63.0	71.0	8.0	46.5	54.5	8.0	87.7	12.2	77.5
Republic of Moldova	IS	49.7	63.5	13.8	57.1	70.7	13.6	42.0	54.8	12.8	91.0	12.8	79.3
Bolivia (Plurinational State of)	IS	51.5	63.3	11.9	61.3	70.4	9.1	39.2	54.8	15.6	87.6	12.4	77.4
Sierra Leone	IS	63.2	63.2	0.0	78.4	76.0	-2.4	43.0	45.0	2.0	87.9	15.4	77.7
Eswatini	IS	63.9	62.8	-1.1	73.7	70.9	-2.8	45.9	51.2	5.3	90.9	13.5	78.5
Namibia	IS	63.6	62.5	-1.1	73.7	72.3	-1.4	45.7	45.3	-0.3	87.6	12.6	75.4
Guinea	IS	66.5	62.5	-4.0	76.6	75.4	-1.2	52.3	45.2	-7.0	97.3	13.1	77.3
Benin	IS	61.9	62.4	0.6	73.9	74.5	0.6	45.0	44.4	-0.6	87.9	12.1	77.7
Libya	IS	63.1	62.3	-0.8	67.9	69.1	1.2	58.1	54.1	-4.0	87.9	13.3	77.9
India	IS	58.2	62.1	3.9	62.2	67.8	5.6	53.4	55.3	2.0	87.7	12.9	78.7
Barbados	IS	47.6	62.1	14.5	55.6	67.8	12.3	41.3	56.2	14.9	87.5	12.5	77.8
Suriname	IS	54.3	62.1	7.8	61.1	69.8	8.6	46.9	53.0	6.1	87.2	12.2	72.9
Belarus	IS	52.9	61.7	8.8	60.0	70.1	10.1	46.4	51.2	4.8	87.5	11.8	78.1
Togo	IS	65.4	61.5	-3.9	77.3	75.6	-1.6	48.4	38.0	-10.4	88.1	12.8	77.9
Central African Republic	IS	60.5	61.3	0.7	69.9	70.6	0.7	46.2	46.3	0.1	87.6	10.6	77.4
Mozambique	IS	64.2	61.2	-3.0	72.9	72.9	0.0	51.7	43.3	-8.4	87.6	13.5	77.4
Chad	IS	67.6	61.1	-6.5	79.3	76.7	-2.6	50.2	41.1	-9.1	88.9	14.4	78.7
Turkmenistan	IS	59.4	60.9	1.5	69.1	72.0	2.9	44.7	44.2	-0.5	87.5	12.8	76.9
Bosnia and Herzegovina	IS	49.6	60.7	11.1	54.9	65.5	10.7	45.6	55.9	10.3	86.1	11.7	78.2
Algeria	IS	48.0	60.4	12.4	44.8	59.4	14.6	47.3	59.5	12.1	86.3	12.6	78.1
Kazakhstan	IS	60.0	60.3	0.2	69.4	70.7	1.3	44.8	44.1	-0.7	87.8	10.5	77.2
Liberia	IS	63.2	59.9	-3.3	75.5	73.0	-2.5	47.1	42.1	-5.0	88.1	13.4	78.1
Ukraine	IS	43.5	59.7	16.3	52.6	68.3	15.7	33.8	48.4	14.5	87.6	9.2	77.7
Gambia	IS	66.3	59.5	-6.8	80.0	74.3	-5.7	47.8	37.8	-10.0	88.2	14.9	77.9
Kyrgyzstan	IS	51.3	59.5	8.2	63.3	72.8	9.5	31.3	38.2	6.8	87.8	13.0	77.4
Sri Lanka	IS	48.7	59.2	10.5	59.2	68.2	9.0	36.9	47.5	10.6	87.5	12.5	77.7
Tunisia	IS	43.5	58.4	14.9	49.4	65.3	16.0	36.4	49.8	13.4	79.0	12.1	76.9
Indonesia	IS	66.1	57.9	-8.2	72.5	67.7	-4.8	58.4	45.8	-12.7	85.5	12.5	77.9
Grenada	IS	38.6	57.7	19.1	54.4	67.5	13.1	12.9	45.4	32.5	87.4	11.6	0.0
Saint Lucia	IS	31.5	56.9	25.3	43.5	65.6	22.1	19.6	46.5	26.9	87.7	11.9	77.8
Latvia	IS	38.3	56.8	18.5	48.8	63.1	14.3	26.6	52.0	25.4	86.6	12.9	78.9

Myanmar	IS	45.9	56.3	10.4	58.1	68.0	9.9	31.2	40.5	9.3	87.3	12.4	78.0
Saint Vincent and the Grenadines	IS	47.2	56.2	9.0	56.4	65.4	9.0	37.0	45.2	8.2	87.8	12.8	77.6
Lao People's Democratic Republic	IS	43.3	56.2	12.9	51.8	65.8	14.0	34.5	44.7	10.1	86.3	11.7	77.6
Tajikistan	IS	50.1	56.0	5.9	64.0	66.1	2.2	29.4	42.4	12.9	86.9	12.1	80.1
Cyprus	IS	18.1	55.5	37.5	22.6	60.6	38.0	17.3	52.8	35.5	87.7	12.1	77.5
Romania	IS	39.0	55.4	16.4	46.9	63.1	16.2	31.4	47.0	15.7	87.5	11.5	78.0
Montenegro	IS	68.2	54.9	-13.3	74.9	63.0	-11.9	59.6	46.1	-13.5	92.4	12.1	69.2
Timor-Leste	IS	56.0	54.9	-1.1	61.8	64.3	2.5	49.7	43.7	-6.1	88.0	10.6	78.0
Syrian Arab Republic	IS	52.7	54.8	2.1	55.1	55.1	-0.1	49.9	52.0	2.0	87.7	12.0	77.5
Guinea-Bissau	IS	56.4	54.7	-1.7	75.7	71.8	-3.9	28.8	27.7	-1.1	87.8	13.0	77.5
Nepal	IS	46.2	54.6	8.3	46.9	62.2	15.3	45.8	45.4	-0.4	87.2	11.6	74.8
Lesotho	IS	64.0	54.4	-9.6	73.8	65.6	-8.2	41.1	33.9	-7.3	87.5	6.1	77.9
Saudi Arabia	IS	35.4	53.7	18.2	39.1	55.2	16.1	30.2	50.0	19.8	91.3	11.2	78.6
Cambodia	IS	50.6	53.0	2.4	58.5	61.9	3.4	41.8	41.9	0.1	90.5	11.1	78.8
Georgia	IS	57.2	52.6	-4.6	69.1	66.5	-2.6	33.9	30.1	-3.8	86.8	2.0	77.0
Sudan	IS	37.1	52.5	15.5	35.3	53.7	18.4	37.9	50.0	12.1	87.4	12.4	79.2
Jamaica	IS	41.5	52.1	10.6	49.7	60.3	10.6	33.9	43.1	9.1	87.5	11.3	77.3
Viet Nam	IS	48.9	51.0	2.0	61.5	64.1	2.5	31.3	33.6	2.2	87.6	12.0	77.1
Bahrain	IS	32.2	50.7	18.6	43.8	58.3	14.5	19.7	43.8	24.1	87.6	12.0	77.9
Russian Federation	IS	25.3	49.9	24.6	41.8	61.2	19.4	0.0	35.7	35.7	87.0	21.0	78.3
Morocco	IS	44.4	49.3	4.9	45.1	52.0	6.9	44.0	46.9	2.8	88.8	12.4	78.2
Dominica	IS	42.8	47.4	4.7	56.0	58.8	2.8	24.6	37.4	12.8	87.5	12.8	75.8
Saint Kitts and Nevis	IS	36.7	47.3	10.6	49.7	57.4	7.8	18.7	37.6	18.9	87.7	12.4	77.3
Guyana	IS	31.8	46.6	14.8	46.2	56.8	10.5	14.1	34.5	20.4	87.7	12.9	77.6
Bangladesh	IS	40.7	45.4	4.7	41.1	49.5	8.4	38.0	40.8	2.9	84.3	11.1	77.6
Honduras	IS	57.2	43.5	-13.8	69.3	55.8	-13.5	39.8	27.5	-12.2	89.6	26.0	79.1
Palestine	IS	18.5	42.0	23.5	33.6	53.1	19.5	0.1	27.6	27.6	84.6	11.2	100.0
Iraq	IS	36.4	41.1	4.7	49.4	54.9	5.5	21.2	24.7	3.4	82.3	12.9	78.4
Bulgaria	IS	31.9	40.3	8.4	41.6	52.8	11.2	21.9	25.3	3.4	86.4	14.9	76.2
Serbia	IS	18.1	39.3	21.2	29.5	47.3	17.8	8.0	31.6	23.6	87.5	11.4	77.4
Afghanistan	IS	31.6	34.2	2.6	37.4	41.0	3.7	24.5	27.7	3.3	87.8	11.3	79.1
Egypt	IS	0.0	31.9	31.9	0.0	22.4	22.4	0.4	31.3	30.9	88.3	11.7	77.2
Yemen	IS	25.9	31.6	5.7	32.2	41.4	9.2	20.1	20.7	0.6	91.0	12.3	78.3
Haiti	IS	15.3	28.4	13.1	21.7	34.0	12.3	14.5	24.6	10.2	87.4	10.2	77.5
North Macedonia	IS	34.2	25.5	-8.7	43.7	33.6	-10.0	23.1	20.6	-2.5	86.8	15.7	76.7
Kuwait	SAH	88.8	100.0	11.2	84.5	100.0	15.5	89.8	97.2	7.5	83.6	68.8	80.3
Jordan	SAH	93.0	99.7	6.7	90.3	97.5	7.2	92.6	98.7	6.1	84.6	79.5	80.5
San Marino	SAH	89.7	99.2	9.5	86.1	95.2	9.1	90.4	99.7	9.4	84.0	71.4	80.4
Singapore	SAH	85.2	98.5	13.4	79.8	93.6	13.8	88.2	100.0	11.8	84.7	79.6	80.4
Austria	SAH	90.1	98.3	8.2	85.5	94.6	9.1	91.8	98.1	6.3	84.7	79.9	80.5
Malta	SAH	89.9	95.9	6.0	84.9	92.6	7.7	91.9	95.5	3.6	84.7	76.7	80.4
Palestine	SAH	87.6	95.7	8.0	85.9	94.1	8.3	85.2	93.0	7.8	84.3	79.3	80.4
Bermuda	SAH	84.7	95.2	10.5	86.5	97.7	11.2	76.8	87.0	10.3	84.6	76.8	80.4
Cabo Verde	SAH	90.2	95.1	4.9	89.9	94.4	4.5	86.8	92.7	5.9	84.6	80.3	80.4
Puerto Rico	SAH	88.5	94.9	6.4	86.6	93.7	7.1	85.3	90.6	5.3	84.7	76.5	80.4
Israel	SAH	89.1	94.4	5.2	84.9	93.1	8.2	90.4	91.9	1.5	84.6	78.1	80.4
Cook Islands	SAH	74.5	93.2	18.8	69.1	88.4	19.2	78.5	95.3	16.7	84.5	76.2	80.5
Botswana	SAH	83.7	92.4	8.7	82.8	90.1	7.3	81.8	91.3	9.6	84.1	72.2	80.3

Nigeria	SAH	82.7	92.4	9.7	83.0	90.8	7.8	81.8	91.0	9.2	84.8	76.1	80.4
Iceland	SAH	82.1	92.3	10.2	75.4	88.2	12.8	86.7	93.4	6.7	84.7	79.3	80.4
Finland	SAH	80.5	92.1	11.6	75.7	88.4	12.6	83.5	92.7	9.1	84.6	63.0	80.3
Norway	SAH	74.4	92.0	17.7	68.1	89.8	21.7	79.5	91.1	11.5	84.6	78.3	80.4
Sweden	SAH	77.0	91.7	14.7	69.8	86.7	16.9	82.9	93.9	11.0	84.6	70.6	80.4
Japan	SAH	76.9	91.6	14.7	73.6	88.9	15.3	78.0	91.1	13.1	86.7	78.4	83.3
Azerbaijan	SAH	86.6	91.3	4.6	85.6	89.1	3.5	84.5	90.6	6.1	84.7	88.1	80.5
Guam	SAH	79.2	91.2	12.0	70.7	87.2	16.6	86.5	92.8	6.3	83.6	68.4	80.1
Switzerland	SAH	74.9	90.4	15.5	69.3	87.1	17.9	79.0	90.5	11.5	84.1	75.1	80.5
Slovenia	SAH	75.8	90.3	14.5	74.4	88.7	14.4	74.2	88.9	14.7	84.6	78.6	80.4
Andorra	SAH	78.9	90.3	11.4	69.5	82.8	13.3	86.1	95.5	9.3	84.0	68.4	80.4
Canada	SAH	81.9	90.2	8.3	79.8	88.8	9.0	79.4	87.0	7.6	84.7	70.1	80.4
Equatorial Guinea	SAH	71.1	89.8	18.7	73.7	86.0	12.3	66.9	90.5	23.6	84.7	79.8	80.5
Germany	SAH	78.2	89.8	11.6	73.9	85.6	11.7	80.1	90.7	10.6	84.7	75.3	80.5
Sri Lanka	SAH	83.5	89.7	6.2	83.7	88.8	5.0	80.8	87.2	6.4	84.8	79.9	80.5
South Africa	SAH	91.7	89.6	-2.1	88.6	86.3	-2.3	91.2	89.6	-1.6	85.3	85.3	80.4
Cuba	SAH	81.5	89.4	7.9	78.5	87.2	8.7	81.0	87.7	6.7	84.8	80.0	80.4
Syrian Arab Republic	SAH	78.1	89.4	11.3	76.9	87.2	10.2	77.2	88.9	11.7	84.6	80.7	80.4
Seychelles	SAH	77.0	89.4	12.4	77.9	87.7	9.8	73.4	88.5	15.1	84.7	78.8	80.5
Taiwan (Province of China)	SAH	82.7	89.4	6.7	79.5	88.7	9.2	83.4	86.7	3.3	83.7	78.3	80.6
Sao Tome and Principe	SAH	89.6	89.4	-0.3	85.6	84.5	-1.1	90.3	91.3	1.0	85.2	81.3	80.5
Iran (Islamic Republic of)	SAH	71.9	89.3	17.4	71.8	87.6	15.7	69.7	87.9	18.1	84.6	79.0	80.5
Oman	SAH	79.4	89.2	9.8	79.9	87.5	7.6	76.0	88.2	12.3	86.4	77.7	100.0
Luxembourg	SAH	73.9	89.1	15.2	68.0	85.5	17.5	78.0	89.4	11.4	84.5	79.0	80.4
Mauritania	SAH	81.2	88.6	7.4	75.9	82.4	6.5	83.9	91.5	7.5	84.7	80.1	80.4
Netherlands	SAH	76.8	88.6	11.8	73.0	85.7	12.6	77.7	88.0	10.3	84.7	77.0	80.5
United States Virgin Islands	SAH	73.3	88.6	15.3	72.7	90.3	17.6	68.0	80.9	12.9	84.4	95.6	79.9
Nicaragua	SAH	82.5	88.6	6.1	79.1	85.0	5.9	82.1	88.6	6.5	84.8	79.3	80.5
Gabon	SAH	83.9	88.4	4.5	84.2	87.2	3.0	80.9	86.7	5.8	84.9	81.5	80.5
France	SAH	81.9	88.2	6.3	78.7	85.9	7.2	83.3	87.3	4.0	82.4	73.8	80.3
Monaco	SAH	70.1	88.1	18.0	63.5	82.2	18.7	75.3	91.5	16.2	84.6	75.3	80.5
Guatemala	SAH	91.7	88.1	-3.7	88.8	85.9	-2.8	90.9	86.5	-4.4	90.7	80.3	80.3
Lebanon	SAH	52.9	88.0	35.0	59.6	88.5	28.9	41.7	83.1	41.3	84.6	77.3	80.5
Saudi Arabia	SAH	79.1	87.8	8.7	76.3	84.5	8.2	79.6	88.4	8.8	84.6	75.6	80.5
Ireland	SAH	74.6	87.6	13.0	69.8	84.7	14.8	77.3	87.5	10.2	84.9	78.0	80.4
Costa Rica	SAH	87.5	87.5	0.0	83.9	86.0	2.1	87.6	84.8	-2.8	85.3	78.0	80.6
Kenya	SAH	87.0	87.5	0.5	85.9	86.1	0.2	86.3	86.2	0.0	85.2	83.6	80.5
Namibia	SAH	85.4	87.4	2.0	84.1	86.6	2.5	83.7	85.1	1.4	84.9	81.4	80.4
Greece	SAH	81.3	87.4	6.1	77.1	85.3	8.3	83.2	86.3	3.1	86.6	79.0	82.3
United Republic of Tanzania	SAH	79.7	87.0	7.2	82.4	85.3	3.0	76.5	86.2	9.7	84.5	79.0	80.4
Denmark	SAH	73.8	86.8	13.0	68.6	83.4	14.8	77.0	87.3	10.3	84.7	77.3	80.4
Burkina Faso	SAH	84.5	86.8	2.3	88.0	89.4	1.3	79.4	82.5	3.1	84.9	80.0	80.4
Maldives	SAH	64.9	86.7	21.8	59.2	82.9	23.8	68.6	88.3	19.7	84.8	80.3	80.5
Spain	SAH	82.1	86.6	4.5	80.6	85.1	4.6	80.5	84.3	3.8	78.5	79.5	80.7
Tunisia	SAH	75.0	86.4	11.4	74.6	84.8	10.2	72.6	84.8	12.2	84.7	78.1	80.5
Ethiopia	SAH	76.9	86.3	9.4	80.4	86.8	6.4	73.6	85.0	11.4	84.9	81.2	80.4
Comoros	SAH	79.2	86.2	7.1	78.1	84.6	6.5	78.5	85.5	7.1	84.7	80.8	80.4
Republic of Korea	SAH	51.5	86.0	34.5	49.4	82.8	33.4	52.6	86.9	34.3	84.7	80.4	80.5

Australia	SAH	70.8	85.5	14.7	68.2	83.9	15.7	70.4	83.2	12.7	83.7	77.0	80.4
Cyprus	SAH	60.1	85.3	25.2	53.8	80.8	27.0	65.2	87.9	22.7	84.7	77.8	80.4
Tonga	SAH	80.9	85.3	4.5	74.2	78.6	4.4	86.5	91.0	4.5	84.9	81.6	80.5
Belize	SAH	78.4	85.2	6.9	78.7	83.2	4.5	73.9	83.8	9.9	84.5	79.9	80.3
Italy	SAH	77.2	85.2	8.0	72.8	82.9	10.1	79.5	84.1	4.6	84.8	79.4	80.4
Uganda	SAH	76.5	85.1	8.7	82.9	87.4	4.5	71.0	81.0	10.1	84.4	79.1	80.4
Benin	SAH	79.6	84.8	5.2	74.8	80.4	5.6	81.9	86.6	4.7	84.7	78.5	80.4
Qatar	SAH	55.3	84.8	29.5	75.0	88.8	13.8	37.9	80.1	42.2	83.7	78.0	80.3
Djibouti	SAH	79.6	84.7	5.1	82.3	86.1	3.8	76.6	83.0	6.4	84.6	81.0	80.4
Mali	SAH	79.6	84.5	4.9	70.4	75.4	5.0	85.1	89.2	4.2	84.8	81.3	80.4
Coted'Ivoire	SAH	81.5	84.2	2.7	81.4	82.6	1.2	80.8	84.3	3.5	84.9	82.5	80.4
Bahrain	SAH	64.3	84.2	19.9	62.6	82.4	19.9	64.2	84.1	19.8	84.7	79.0	80.5
Malaysia	SAH	75.3	84.1	8.8	72.8	79.7	6.9	75.4	86.0	10.6	84.9	80.1	80.5
El Salvador	SAH	75.8	84.0	8.2	74.0	81.2	7.2	73.8	82.5	8.7	84.9	80.4	80.4
Rwanda	SAH	67.1	83.9	16.8	71.0	85.3	14.3	61.1	79.8	18.8	84.7	80.8	80.4
Ghana	SAH	79.5	83.8	4.3	77.4	81.7	4.3	79.7	83.9	4.3	84.5	80.5	0.0
United States of America	SAH	75.1	83.7	8.6	72.5	84.3	11.8	73.6	78.2	4.5	86.8	71.9	79.7
Niger	SAH	80.5	83.7	3.1	78.7	81.4	2.6	80.6	83.9	3.3	84.6	82.9	80.2
Senegal	SAH	80.4	83.5	3.1	78.4	80.2	1.8	80.5	84.5	4.0	84.9	81.2	80.4
Czechia	SAH	63.5	83.2	19.7	61.9	81.4	19.5	62.7	82.1	19.3	84.7	76.9	80.4
Peru	SAH	73.4	83.2	9.8	70.7	77.7	6.9	73.3	86.0	12.7	84.9	80.4	80.5
Algeria	SAH	73.6	83.1	9.6	70.4	78.3	7.9	74.2	84.7	10.6	84.9	80.9	80.4
Antigua and Barbuda	SAH	71.6	82.9	11.3	74.4	81.4	6.9	63.4	80.5	17.1	84.6	78.2	80.4
Saint Lucia	SAH	68.1	82.7	14.6	63.5	79.9	16.3	69.5	81.9	12.4	84.6	79.2	80.4
Guinea	SAH	81.8	82.7	0.9	76.4	77.5	1.1	84.5	84.9	0.5	84.9	80.0	79.4
Armenia	SAH	71.9	82.7	10.8	71.0	83.9	12.9	70.8	79.5	8.7	84.8	77.4	80.5
Belgium	SAH	78.2	82.6	4.4	74.0	80.0	6.1	80.2	81.8	1.6	84.6	78.5	80.3
Slovakia	SAH	70.9	82.3	11.5	68.8	80.5	11.6	70.5	81.3	10.9	84.9	78.5	80.4
Cameroon	SAH	80.3	82.3	2.0	79.5	81.1	1.6	79.4	81.7	2.4	84.9	81.5	80.3
Angola	SAH	75.9	82.3	6.3	76.5	81.0	4.5	74.0	80.9	6.9	84.8	81.5	80.5
Sierra Leone	SAH	81.2	82.3	1.1	79.1	77.1	-2.0	81.6	84.7	3.2	84.9	82.6	80.3
Bahamas	SAH	76.7	82.1	5.3	75.0	81.0	6.0	73.8	78.7	4.9	84.5	77.9	80.4
Eswatini	SAH	81.3	82.0	0.7	82.1	80.9	-1.1	77.1	80.4	3.3	84.8	81.7	76.9
United Kingdom	SAH	67.7	82.0	14.3	62.0	78.6	16.6	72.2	82.6	10.4	84.7	78.1	80.4
Saint Vincent and the Grenadines	SAH	78.3	81.8	3.5	74.1	79.3	5.2	78.7	80.7	2.0	85.0	76.7	80.5
Congo	SAH	75.3	81.7	6.4	73.1	76.7	3.6	75.3	83.9	8.6	84.8	81.6	80.5
Liberia	SAH	82.0	81.7	-0.3	76.9	76.4	-0.5	84.2	84.2	0.1	85.1	82.8	80.4
Libya	SAH	78.8	81.3	2.5	75.7	76.3	0.6	79.3	83.7	4.4	85.1	82.2	80.5
Hungary	SAH	68.4	81.2	12.8	67.2	79.4	12.2	65.7	79.8	14.1	84.6	79.1	80.4
Montenegro	SAH	78.7	81.2	2.5	75.4	76.4	1.0	79.6	84.0	4.3	84.7	91.0	80.3
Democratic Republic of the Congo	SAH	79.2	81.0	1.8	77.4	79.9	2.5	79.3	79.6	0.3	89.3	82.2	80.4
Jamaica	SAH	73.7	81.0	7.2	70.1	77.3	7.2	74.2	81.2	7.1	84.5	77.1	80.3
Mexico	SAH	86.7	80.7	-6.1	82.8	79.4	-3.4	87.2	78.2	-8.9	86.3	79.4	80.7
Panama	SAH	81.9	80.7	-1.2	77.4	79.1	1.6	83.5	78.8	-4.8	85.4	78.9	80.5
Estonia	SAH	74.9	80.7	5.8	80.3	84.9	4.7	63.9	72.4	8.4	84.9	78.6	80.4
Argentina	SAH	56.7	80.6	23.9	55.3	77.8	22.4	56.1	80.5	24.5	84.8	79.8	80.4
Gambia	SAH	82.3	80.5	-1.8	81.0	77.5	-3.6	82.0	81.4	-0.6	85.3	84.6	80.4
Albania	SAH	71.5	80.4	8.9	71.2	77.3	6.2	68.8	81.3	12.5	83.8	79.7	80.2

Togo	SAH	80.2	80.2	0.0	76.5	78.3	1.8	81.6	80.3	-1.3	85.1	81.6	80.4
Chile	SAH	70.7	80.2	9.4	66.9	77.9	11.0	72.2	79.1	6.8	83.5	77.1	80.6
Iraq	SAH	69.1	80.0	10.9	70.7	79.7	9.0	63.5	77.1	13.7	85.0	79.7	80.5
South Sudan	SAH	76.4	79.7	3.3	79.3	81.1	1.8	74.8	78.3	3.5	84.8	82.5	80.3
Dominican Republic	SAH	70.7	79.7	8.9	68.7	76.6	7.9	68.9	78.9	10.0	87.4	70.5	81.2
Eritrea	SAH	67.7	79.1	11.4	74.1	81.6	7.4	59.3	73.9	14.6	84.2	80.5	80.5
Palau	SAH	72.9	79.1	6.2	71.2	74.3	3.1	73.7	82.5	8.8	84.9	81.0	80.6
Barbados	SAH	72.9	79.1	6.2	73.2	77.9	4.7	68.5	76.9	8.4	84.8	77.9	80.5
Republic of Moldova	SAH	74.3	79.0	4.7	77.2	81.7	4.5	68.0	74.0	6.0	84.9	80.5	80.5
Venezuela (Bolivarian Republic of)	SAH	80.1	78.3	-1.7	77.0	76.4	-0.6	79.3	75.6	-3.7	85.4	79.6	80.3
Burundi	SAH	65.3	78.3	13.0	70.6	81.0	10.4	59.2	75.7	16.6	84.5	81.4	80.3
Saint Kitts and Nevis	SAH	65.1	78.2	13.1	61.5	77.4	15.9	63.9	75.2	11.3	84.8	80.8	80.4
American Samoa	SAH	76.1	78.2	2.1	69.2	70.2	1.0	81.8	84.6	2.8	85.0	81.9	80.5
Northern Mariana Islands	SAH	70.3	78.2	7.9	52.0	68.8	16.8	83.6	85.1	1.5	84.6	78.8	80.4
Chad	SAH	80.2	78.2	-2.0	78.3	75.4	-2.9	80.1	79.0	-1.0	85.5	84.0	80.4
Colombia	SAH	76.8	78.1	1.3	73.1	75.2	2.1	77.4	77.7	0.3	84.7	79.4	80.1
Poland	SAH	66.2	78.1	11.9	67.2	79.3	12.1	61.9	74.4	12.4	84.9	79.3	80.4
Zambia	SAH	73.4	77.8	4.4	73.7	76.9	3.3	72.9	77.3	4.5	84.5	81.4	84.3
Grenada	SAH	65.0	77.2	12.2	63.5	75.1	11.6	61.1	74.2	13.1	84.7	79.1	80.3
Dominica	SAH	72.6	77.2	4.5	74.7	78.9	4.2	63.6	71.5	7.9	84.9	80.3	80.4
New Zealand	SAH	62.6	76.9	14.4	57.4	74.7	17.4	65.7	76.0	10.4	84.9	77.1	80.5
Turkey	SAH	50.5	76.7	26.2	56.8	78.5	21.7	41.5	71.7	30.2	84.8	79.4	80.4
Brunei Darussalam	SAH	63.6	76.4	12.8	55.5	71.5	16.0	70.9	79.7	8.7	84.9	80.7	80.5
Kiribati	SAH	71.8	76.3	4.5	76.4	77.9	1.5	64.9	74.3	9.4	85.6	83.5	80.4
Philippines	SAH	73.2	76.1	3.0	72.7	74.8	2.1	70.8	74.7	4.0	85.2	90.0	80.3
Malawi	SAH	75.5	76.1	0.6	74.1	76.2	2.1	75.3	74.6	-0.7	85.1	82.8	80.5
Samoa	SAH	67.0	76.0	9.0	64.8	69.9	5.0	69.2	81.2	12.1	83.4	83.1	80.5
Mauritius	SAH	67.5	75.8	8.3	72.9	77.9	5.0	57.7	70.3	12.6	84.6	79.7	80.5
Fiji	SAH	72.6	75.8	3.2	68.6	71.3	2.7	75.8	81.8	6.1	84.9	82.4	80.5
Bosnia and Herzegovina	SAH	53.0	75.7	22.7	52.1	73.5	21.4	52.3	75.5	23.2	84.6	75.2	80.4
Somalia	SAH	66.4	75.7	9.2	74.1	79.4	5.3	60.1	70.3	10.3	82.4	81.3	82.9
Portugal	SAH	63.8	75.6	11.8	60.2	72.0	11.8	65.7	76.8	11.1	84.9	80.1	80.4
Tajikistan	SAH	71.3	75.6	4.3	65.4	70.7	5.3	74.5	78.3	3.8	84.7	73.0	80.9
Lithuania	SAH	71.1	75.5	4.5	74.2	78.6	4.4	63.8	68.2	4.4	84.7	76.9	80.6
Latvia	SAH	69.9	75.5	5.6	72.8	78.1	5.3	62.6	69.4	6.8	85.0	79.4	80.5
Suriname	SAH	70.1	75.4	5.2	68.2	73.9	5.7	69.0	73.2	4.3	84.7	79.4	80.1
Egypt	SAH	42.5	75.0	32.5	54.2	74.5	20.3	28.3	72.3	44.0	84.5	76.6	80.5
Morocco	SAH	65.8	75.0	9.2	61.8	69.9	8.1	67.8	77.9	10.0	84.8	80.5	80.4
Ecuador	SAH	81.0	74.9	-6.1	78.6	72.2	-6.4	80.1	74.7	-5.4	85.6	78.3	80.5
Madagascar	SAH	69.0	74.8	5.9	73.4	74.3	0.9	65.7	74.3	8.6	83.9	82.8	80.8
United Arab Emirates	SAH	58.2	74.1	15.9	53.6	46.0	-7.6	63.0	83.2	20.2	84.7	87.2	80.4
Viet Nam	SAH	63.1	74.0	11.0	66.9	76.6	9.7	56.4	69.5	13.1	84.7	79.1	80.4
Lesotho	SAH	81.2	73.4	-7.8	84.0	74.9	-9.0	71.7	67.5	-4.2	82.0	76.7	80.9
Niue	SAH	70.2	73.4	3.2	67.2	67.4	0.2	73.6	78.7	5.0	85.2	81.1	80.5
Croatia	SAH	55.2	72.9	17.6	54.2	72.6	18.4	54.4	70.6	16.3	84.8	79.6	80.5
Uruguay	SAH	60.1	72.2	12.1	57.8	69.5	11.7	60.1	72.1	12.0	84.8	78.9	80.4
Bhutan	SAH	58.8	71.8	13.0	54.1	66.8	12.8	61.8	74.8	13.0	84.8	79.6	80.5
Trinidad and Tobago	SAH	67.2	71.4	4.1	66.4	72.2	5.7	64.5	67.0	2.6	84.9	79.5	80.3

Tokelau	SAH	60.6	71.3	10.7	54.9	64.0	9.1	65.1	77.3	12.2	84.9	80.7	80.4
Solomon Islands	SAH	63.2	71.2	7.9	59.7	67.7	8.0	65.0	73.1	8.1	86.2	80.4	80.6
Vanuatu	SAH	60.5	70.2	9.7	58.6	67.0	8.4	61.4	72.3	10.9	86.6	78.8	80.4
Cambodia	SAH	54.7	69.7	15.0	51.4	66.8	15.4	55.9	70.6	14.7	84.5	77.5	80.5
Central African Republic	SAH	65.4	69.3	3.9	68.0	71.7	3.7	61.5	65.3	3.8	83.8	82.2	79.9
Guinea-Bissau	SAH	65.3	69.1	3.8	66.6	66.9	0.3	63.3	70.0	6.7	84.9	82.3	80.3
Sudan	SAH	51.0	68.9	17.9	48.7	63.6	14.9	52.5	72.3	19.8	84.8	80.3	80.4
Belarus	SAH	67.2	68.8	1.6	68.5	73.7	5.2	62.2	60.5	-1.6	85.1	78.4	80.7
Paraguay	SAH	61.1	68.1	7.0	54.2	60.5	6.4	66.1	74.0	7.9	84.8	81.0	80.4
Zimbabwe	SAH	78.0	68.0	-10.0	75.2	65.2	-10.0	78.4	69.1	-9.3	84.4	0.0	80.4
Guyana	SAH	60.9	67.7	6.8	64.7	70.1	5.4	54.2	61.2	7.0	84.9	80.1	80.4
Ukraine	SAH	57.8	67.5	9.6	56.9	68.9	12.0	56.0	64.5	8.5	87.8	76.0	89.4
Mozambique	SAH	69.9	67.3	-2.6	75.0	75.3	0.3	65.4	60.6	-4.8	0.0	81.6	80.3
Lao People's Democratic Republic	SAH	38.6	67.2	28.6	34.8	64.4	29.6	41.4	68.3	26.9	84.6	78.5	80.5
Nepal	SAH	53.0	67.1	14.1	50.2	66.5	16.3	54.8	65.7	10.9	84.6	78.3	80.4
Tuvalu	SAH	47.0	67.0	20.0	43.9	62.1	18.1	50.1	71.9	21.8	84.4	79.5	80.4
China	SAH	0.0	67.0	67.0	0.0	68.9	68.9	0.0	63.2	63.2	84.9	79.6	80.5
Indonesia	SAH	63.1	67.0	3.9	59.2	63.0	3.8	65.1	69.5	4.3	84.9	82.6	80.3
Uzbekistan	SAH	81.5	66.7	-14.9	81.1	70.1	-11.0	79.1	62.2	-16.9	97.3	97.8	80.3
India	SAH	55.7	66.0	10.3	56.8	62.9	6.1	54.8	67.8	13.0	84.9	79.8	80.6
Micronesia (Federated States of)	SAH	52.5	65.8	13.3	51.9	63.5	11.6	53.8	68.3	14.5	83.2	79.5	75.5
Pakistan	SAH	64.7	65.8	1.1	61.8	60.6	-1.2	66.4	69.2	2.8	85.2	83.0	80.6
Georgia	SAH	73.2	65.7	-7.5	73.9	66.4	-7.4	70.3	64.4	-5.9	84.3	80.4	80.5
Thailand	SAH	50.0	65.5	15.5	54.3	69.3	15.0	43.9	60.8	16.9	84.9	79.8	80.6
Myanmar	SAH	43.7	65.4	21.7	44.9	66.8	21.8	41.3	62.2	20.9	84.8	79.7	80.5
Serbia	SAH	48.0	64.8	16.8	42.7	59.1	16.4	52.5	69.3	16.8	84.5	79.8	80.1
Marshall Islands	SAH	54.9	64.7	9.8	56.0	60.8	4.7	54.7	67.4	12.7	84.1	81.6	80.6
Timor-Leste	SAH	55.7	63.7	8.0	50.0	60.2	10.2	60.2	65.8	5.6	84.4	75.7	80.6
Brazil	SAH	57.7	63.1	5.4	54.2	58.1	3.9	58.2	65.6	7.5	84.9	80.7	80.3
Russian Federation	SAH	67.2	62.8	-4.4	69.2	64.0	-5.2	61.8	59.9	-1.9	86.2	79.2	78.6
Kyrgyzstan	SAH	62.9	62.8	-0.2	66.0	66.1	0.1	56.2	57.9	1.7	85.0	80.0	80.3
Bulgaria	SAH	65.0	62.7	-2.3	65.5	63.6	-2.0	63.0	60.0	-3.0	85.2	79.5	83.7
Kazakhstan	SAH	62.8	62.7	-0.2	62.3	61.7	-0.5	61.3	62.4	1.1	85.4	83.8	80.8
Bangladesh	SAH	37.1	61.1	23.9	42.5	57.5	15.0	36.7	64.0	27.2	84.0	77.8	80.2
Nauru	SAH	55.7	60.8	5.1	46.6	52.6	6.0	62.0	68.9	6.9	87.9	83.6	79.7
North Macedonia	SAH	51.1	60.8	9.8	45.2	53.1	8.0	55.6	68.3	12.7	85.9	80.5	84.3
Romania	SAH	60.4	60.8	0.4	57.0	60.7	3.7	62.2	59.6	-2.6	84.4	79.7	82.6
Papua New Guinea	SAH	47.3	60.4	13.1	31.2	46.0	14.8	60.7	71.1	10.4	84.6	80.0	80.5
Bolivia (Plurinational State of)	SAH	46.4	60.3	13.9	39.4	51.5	12.1	52.2	68.5	16.3	84.9	80.3	80.4
Democratic People's Republic of Korea	SAH	48.5	59.9	11.5	47.6	60.1	12.4	45.5	57.0	11.5	84.8	83.3	80.5
Greenland	SAH	8.0	59.7	51.6	0.7	56.4	55.7	18.6	61.5	42.9	88.6	80.2	81.1
Yemen	SAH	48.5	59.4	10.9	54.3	60.9	6.6	37.9	55.9	18.1	84.7	81.4	80.4
Turkmenistan	SAH	63.2	55.4	-7.8	62.9	59.7	-3.3	61.5	51.5	-10.1	84.8	85.5	80.4
Afghanistan	SAH	44.0	54.9	10.9	56.8	57.8	1.1	31.2	50.0	18.8	84.6	82.2	80.2
Honduras	SAH	55.8	43.2	-12.6	51.8	33.2	-18.6	57.3	53.2	-4.1	87.1	89.3	77.7
Mongolia	SAH	36.8	38.5	1.6	40.3	38.9	-1.4	31.4	37.9	6.5	85.6	73.8	80.8
Haiti	SAH	16.1	35.0	18.9	6.5	25.7	19.2	24.6	43.5	18.9	83.6	80.9	79.8
United States of America	Stroke	85.8	100.0	14.2	87.1	100.0	12.9	83.5	100.0	16.5	29.8	2.5	32.6

Singapore	Stroke	78.9	98.4	19.5	77.0	97.1	20.0	80.2	99.6	19.3	28.1	2.2	33.6
Canada	Stroke	83.1	98.2	15.1	84.1	97.5	13.4	81.4	98.6	17.2	28.2	2.3	33.8
Austria	Stroke	68.9	95.4	26.5	69.8	95.3	25.6	67.8	94.9	27.1	36.2	2.9	33.7
Japan	Stroke	74.1	93.1	19.0	75.9	95.5	19.6	71.1	90.4	19.3	28.1	2.3	34.2
Israel	Stroke	75.2	92.3	17.1	72.0	91.8	19.7	77.3	92.5	15.2	28.1	2.2	33.7
Puerto Rico	Stroke	73.0	91.8	18.8	78.9	96.7	17.8	64.3	83.5	19.2	28.1	2.4	34.2
Germany	Stroke	72.6	91.4	18.8	74.6	92.1	17.5	69.0	90.0	21.0	28.0	2.3	34.2
Sweden	Stroke	75.6	91.2	15.6	75.2	90.3	15.1	75.4	91.5	16.1	28.1	2.3	34.1
Iceland	Stroke	77.2	91.1	14.0	77.6	90.8	13.3	75.8	91.0	15.2	28.1	2.2	33.8
Finland	Stroke	74.7	90.4	15.7	74.4	90.7	16.3	75.3	89.8	14.4	28.1	2.3	33.8
Norway	Stroke	75.7	90.2	14.5	75.7	90.1	14.4	74.7	89.7	15.0	28.0	2.2	33.9
Switzerland	Stroke	68.8	89.9	21.1	69.5	89.3	19.8	67.1	89.9	22.8	28.1	2.3	34.1
Australia	Stroke	72.4	89.1	16.8	71.8	87.6	15.8	72.6	90.1	17.4	28.0	2.3	34.0
San Marino	Stroke	72.4	89.0	16.6	72.0	87.8	15.9	72.0	89.4	17.4	28.0	2.3	34.1
France	Stroke	67.0	88.9	21.9	69.5	89.0	19.5	63.4	88.3	24.8	29.2	2.6	31.7
Republic of Korea	Stroke	62.8	88.6	25.8	63.8	89.4	25.6	60.6	87.1	26.5	28.1	2.2	33.5
Spain	Stroke	61.3	87.9	26.7	63.2	87.6	24.3	58.0	87.9	29.8	28.1	2.2	32.6
Andorra	Stroke	78.6	87.9	9.2	76.6	86.0	9.5	80.0	89.4	9.4	28.0	2.3	33.9
Kuwait	Stroke	81.4	87.3	5.9	79.7	92.1	12.4	82.0	83.1	1.1	27.7	2.4	34.1
Guam	Stroke	70.6	87.1	16.5	71.8	91.0	19.1	68.4	81.8	13.4	29.8	2.4	7.4
Taiwan (Province of China)	Stroke	64.6	86.6	22.0	67.3	91.5	24.2	61.3	80.7	19.4	28.0	2.3	34.1
United Kingdom	Stroke	67.8	86.0	18.2	69.3	86.5	17.2	65.5	84.8	19.2	28.0	2.3	33.9
Netherlands	Stroke	77.4	85.8	8.5	78.6	86.4	7.8	75.3	84.6	9.3	28.1	2.2	33.3
Costa Rica	Stroke	76.6	85.6	8.9	79.8	89.4	9.6	71.5	79.4	7.9	28.1	2.2	33.9
Ireland	Stroke	66.8	85.4	18.6	67.6	86.4	18.8	65.3	83.7	18.4	28.1	2.2	33.5
Belgium	Stroke	63.0	84.8	21.8	65.5	86.0	20.6	59.3	82.8	23.5	28.1	2.3	33.9
New Zealand	Stroke	69.9	84.6	14.7	70.5	84.4	13.9	68.8	84.5	15.7	28.1	2.2	33.8
Bermuda	Stroke	66.2	84.5	18.4	73.3	92.2	18.9	55.7	74.1	18.4	28.1	2.3	33.9
Denmark	Stroke	71.9	84.0	12.2	72.7	85.8	13.2	70.3	81.5	11.2	28.1	2.2	33.4
Malta	Stroke	61.6	83.7	22.0	63.6	84.4	20.8	58.2	82.2	24.0	28.1	2.2	33.8
Chile	Stroke	64.8	83.3	18.5	67.5	85.9	18.4	60.7	79.4	18.6	28.1	2.3	33.9
Mexico	Stroke	71.2	82.1	10.9	73.3	85.9	12.6	66.3	76.2	9.9	28.0	2.2	34.1
Colombia	Stroke	66.6	81.8	15.2	69.4	85.6	16.1	61.8	75.4	13.6	28.1	2.2	33.6
Lebanon	Stroke	47.7	81.6	33.9	53.2	82.9	29.7	40.7	79.3	38.6	32.7	2.2	54.8
Luxembourg	Stroke	51.0	81.4	30.4	53.8	83.8	30.1	46.9	77.8	31.0	28.1	2.2	33.7
Argentina	Stroke	63.4	81.3	18.0	66.9	84.2	17.3	58.7	77.4	18.7	28.0	2.2	33.8
Peru	Stroke	65.6	81.1	15.5	70.5	84.0	13.6	58.7	76.8	18.1	28.1	2.2	33.9
Czechia	Stroke	47.2	80.7	33.6	51.3	82.5	31.3	41.7	78.4	36.6	28.1	2.3	34.0
Monaco	Stroke	64.1	80.2	16.1	64.8	79.9	15.1	62.6	80.1	17.4	28.1	2.2	33.8
Estonia	Stroke	46.2	79.5	33.3	49.4	84.9	35.6	42.0	72.1	30.2	28.1	2.3	33.7
Cook Islands	Stroke	60.5	79.5	19.0	67.8	84.2	16.4	52.0	73.5	21.5	28.5	2.4	89.2
Italy	Stroke	58.7	79.1	20.4	61.1	80.4	19.2	54.8	76.8	22.0	28.1	2.2	33.8
Ecuador	Stroke	66.0	78.6	12.6	70.5	82.2	11.7	59.4	73.1	13.7	28.0	2.2	33.9
Nicaragua	Stroke	68.1	78.4	10.3	73.2	82.1	8.9	59.4	72.3	12.9	28.1	2.2	33.9
Brunei Darussalam	Stroke	72.2	78.4	6.2	69.9	77.3	7.3	73.3	78.5	5.2	28.1	2.1	33.1
Slovenia	Stroke	54.7	77.7	23.1	60.2	81.5	21.4	47.2	72.3	25.1	28.1	2.2	33.7
Uruguay	Stroke	67.8	77.5	9.7	70.2	79.8	9.6	64.3	74.1	9.7	28.1	2.2	33.4
Qatar	Stroke	61.0	77.4	16.4	63.8	78.6	14.8	58.0	76.1	18.1	28.0	2.2	33.5

Jordan	Stroke	55.9	77.3	21.4	51.6	73.0	21.4	59.6	79.6	20.0	28.7	2.3	44.2
El Salvador	Stroke	65.5	76.8	11.3	70.7	81.3	10.6	57.5	68.5	11.0	28.1	2.2	33.5
United States Virgin Islands	Stroke	55.1	76.6	21.5	62.6	85.2	22.6	44.0	64.5	20.5	27.1	0.9	0.0
Slovakia	Stroke	63.8	76.4	12.6	66.7	79.2	12.5	60.3	72.8	12.5	28.1	2.2	33.6
Greenland	Stroke	63.5	76.1	12.6	62.7	77.8	15.1	64.8	73.7	8.9	28.1	2.2	33.4
Panama	Stroke	65.7	75.1	9.4	70.8	81.8	11.0	58.8	66.2	7.4	28.1	2.2	33.6
Greece	Stroke	51.4	75.1	23.7	49.6	74.1	24.5	53.2	75.4	22.1	28.0	2.2	34.1
Hungary	Stroke	53.8	74.2	20.5	58.0	78.7	20.7	48.2	68.5	20.3	28.1	2.2	33.5
Armenia	Stroke	60.1	73.2	13.1	63.2	76.0	12.8	55.2	69.8	14.6	28.2	2.2	33.5
United Arab Emirates	Stroke	63.7	73.2	9.5	62.9	40.7	-22.2	63.5	78.8	15.2	28.2	1.6	34.2
Thailand	Stroke	60.5	72.5	11.9	65.7	78.4	12.7	53.5	65.3	11.8	28.1	2.2	34.0
Iran (Islamic Republic of)	Stroke	53.9	71.8	17.9	56.6	70.7	14.2	50.4	71.6	21.2	28.1	2.2	33.4
Oman	Stroke	54.7	71.6	16.9	61.4	75.7	14.3	45.8	65.4	19.6	28.2	2.5	25.2
Mauritius	Stroke	53.8	70.8	17.0	61.8	77.5	15.7	44.5	62.5	18.0	28.1	2.2	33.5
Turkey	Stroke	57.0	70.7	13.7	61.0	73.2	12.2	52.1	67.5	15.4	28.1	2.2	33.4
Tonga	Stroke	67.5	70.6	3.1	71.4	73.7	2.4	61.9	66.3	4.4	28.1	2.1	34.4
Guatemala	Stroke	62.7	70.5	7.8	66.1	74.6	8.6	57.3	63.9	6.6	28.0	2.2	33.6
Seychelles	Stroke	59.3	70.3	11.0	67.9	74.6	6.7	49.1	65.5	16.3	28.1	2.2	33.9
Malaysia	Stroke	59.4	70.1	10.7	61.5	71.1	9.5	56.1	67.9	11.8	28.3	2.2	33.7
Croatia	Stroke	41.5	70.1	28.6	45.8	71.5	25.7	34.9	68.4	33.6	28.1	2.3	34.0
Lithuania	Stroke	61.3	69.9	8.6	64.9	74.2	9.3	55.9	64.0	8.1	28.4	2.3	55.6
Botswana	Stroke	53.7	69.6	16.0	59.3	74.0	14.6	44.6	61.6	17.1	26.7	9.0	36.1
Portugal	Stroke	40.6	69.4	28.9	42.8	70.9	28.1	36.6	67.1	30.5	28.1	2.1	33.4
Cuba	Stroke	61.1	69.0	8.0	65.0	75.3	10.3	55.3	61.0	5.7	28.1	2.2	33.7
Sao Tome and Principe	Stroke	68.8	68.8	-0.1	70.6	70.8	0.2	63.8	65.6	1.8	28.4	2.0	35.2
Cabo Verde	Stroke	70.9	68.6	-2.3	73.9	76.5	2.6	65.6	57.0	-8.6	29.5	2.1	45.6
Bahamas	Stroke	55.9	68.5	12.6	62.2	73.2	11.0	47.4	61.9	14.5	28.0	2.3	34.1
Poland	Stroke	37.3	68.3	31.0	43.5	73.3	29.8	29.5	62.8	33.2	28.0	2.3	34.3
Maldives	Stroke	46.1	67.9	21.8	42.1	69.7	27.6	46.3	65.0	18.7	28.1	2.2	33.4
Brazil	Stroke	46.0	67.8	21.8	51.6	71.7	20.1	38.8	62.5	23.7	28.1	2.2	33.7
Venezuela (Bolivarian Republic of)	Stroke	63.9	67.3	3.4	68.7	74.8	6.1	56.7	56.3	-0.4	28.2	2.2	32.8
South Africa	Stroke	75.4	66.9	-8.5	79.7	71.7	-8.0	66.8	58.5	-8.3	29.0	1.5	30.3
United Republic of Tanzania	Stroke	55.9	66.7	10.8	63.8	70.4	6.6	45.5	60.9	15.4	25.4	0.0	41.1
Northern Mariana Islands	Stroke	57.2	66.5	9.3	56.6	70.9	14.3	56.1	61.7	5.6	27.9	2.3	34.2
Cyprus	Stroke	34.7	66.4	31.7	33.7	67.6	33.9	35.6	65.7	30.1	28.0	2.2	33.7
Uganda	Stroke	55.3	66.2	10.9	67.2	74.0	6.9	40.0	53.0	13.0	27.9	2.3	34.1
Nigeria	Stroke	50.8	66.1	15.3	56.1	71.0	14.9	44.3	58.6	14.3	28.1	2.4	33.8
Bosnia and Herzegovina	Stroke	53.9	66.0	12.1	54.5	67.2	12.7	53.4	64.3	10.9	28.3	2.3	33.5
Gabon	Stroke	60.4	65.9	5.5	68.1	72.1	4.0	48.2	56.2	8.0	28.1	2.1	33.6
Libya	Stroke	64.0	65.9	1.9	64.2	67.3	3.2	63.0	63.5	0.5	27.7	1.5	32.5
Trinidad and Tobago	Stroke	50.7	65.2	14.6	55.9	71.7	15.8	44.2	57.6	13.4	28.1	2.2	33.7
Belarus	Stroke	58.5	65.2	6.7	60.6	70.2	9.7	56.4	58.8	2.4	28.2	2.3	33.4
Djibouti	Stroke	57.9	65.1	7.3	66.0	73.0	7.0	46.8	55.4	8.6	28.1	2.3	33.9
Palau	Stroke	57.1	65.1	8.0	68.6	71.2	2.6	44.1	58.5	14.4	28.3	2.1	33.8
Equatorial Guinea	Stroke	44.4	65.1	20.7	53.8	69.4	15.6	29.3	57.1	27.8	28.1	2.3	33.7
Uzbekistan	Stroke	58.3	64.9	6.6	63.3	70.2	6.9	50.6	56.9	6.4	29.7	1.8	29.9
Kenya	Stroke	66.6	64.8	-1.7	70.5	70.3	-0.2	60.3	56.0	-4.3	28.4	2.0	28.0
Comoros	Stroke	54.3	64.8	10.5	60.4	69.6	9.2	45.1	56.8	11.7	28.1	2.2	33.6

Belize	Stroke	56.5	64.7	8.2	61.6	68.9	7.3	49.6	59.1	9.5	28.1	2.2	35.2
Algeria	Stroke	51.8	64.6	12.8	43.7	59.0	15.3	54.7	67.0	12.4	28.1	2.4	33.7
Barbados	Stroke	51.2	64.5	13.2	56.5	68.5	12.0	45.1	59.0	13.9	28.1	2.3	33.8
Dominican Republic	Stroke	54.8	64.5	9.7	59.1	70.8	11.6	48.6	56.4	7.8	28.6	1.2	32.9
Tunisia	Stroke	48.2	64.0	15.8	48.4	66.2	17.8	46.1	60.2	14.2	16.7	5.3	52.9
Ukraine	Stroke	51.5	64.0	12.4	54.7	69.0	14.3	47.4	56.9	9.5	28.1	2.3	34.1
Niue	Stroke	58.5	63.5	5.0	65.0	69.9	4.9	49.6	54.8	5.2	28.1	2.2	33.6
American Samoa	Stroke	59.5	63.2	3.7	64.9	66.7	1.7	53.1	58.5	5.4	28.1	2.2	33.5
Fiji	Stroke	59.1	63.1	4.0	65.2	68.0	2.8	49.4	54.8	5.4	28.2	2.1	33.4
Latvia	Stroke	44.7	62.7	18.0	48.8	65.4	16.6	39.2	60.5	21.3	28.3	2.2	42.2
Antigua and Barbuda	Stroke	46.5	61.8	15.3	57.8	70.0	12.3	31.1	50.7	19.5	28.1	2.3	33.7
Ethiopia	Stroke	45.0	61.7	16.7	54.5	67.9	13.5	33.0	53.2	20.2	28.1	2.2	33.6
Republic of Moldova	Stroke	40.5	61.6	21.0	43.2	65.8	22.6	37.0	56.3	19.3	28.2	2.3	32.3
Mauritania	Stroke	55.4	61.5	6.1	56.0	62.3	6.4	54.5	59.7	5.2	28.2	2.1	33.1
Bhutan	Stroke	49.3	61.3	12.0	47.5	61.9	14.4	50.0	59.5	9.5	28.1	2.3	33.7
Ghana	Stroke	56.7	61.1	4.4	60.5	66.3	5.8	51.1	53.3	2.2	28.4	2.2	32.3
Paraguay	Stroke	50.6	61.0	10.4	52.5	64.7	12.2	47.4	56.1	8.7	28.1	2.2	33.7
Azerbaijan	Stroke	53.8	60.5	6.7	60.4	64.1	3.6	41.7	55.1	13.3	29.5	2.1	9.0
Kyrgyzstan	Stroke	48.9	60.5	11.6	55.2	69.2	14.0	37.9	47.6	9.6	28.1	2.2	33.5
Sri Lanka	Stroke	47.9	60.3	12.4	54.7	66.2	11.5	39.8	52.0	12.1	28.1	2.3	33.7
Samoa	Stroke	52.8	60.3	7.5	62.2	65.6	3.4	40.7	53.3	12.6	28.2	2.1	34.1
Pakistan	Stroke	58.6	59.9	1.3	56.5	58.9	2.4	58.5	59.5	1.1	28.2	2.2	33.6
China	Stroke	31.0	59.5	28.5	39.5	67.9	28.4	19.5	48.1	28.6	25.8	2.9	53.3
Angola	Stroke	51.2	59.5	8.3	60.0	66.0	6.0	38.5	48.3	9.8	28.2	2.2	33.5
Tokelau	Stroke	44.4	59.4	14.9	52.9	65.6	12.8	30.8	49.5	18.8	28.2	2.3	33.9
Coted'Ivoire	Stroke	59.0	59.0	0.0	64.9	64.5	-0.4	52.6	52.4	-0.3	28.2	2.0	32.8
Saint Lucia	Stroke	34.9	58.7	23.8	42.7	67.0	24.3	24.2	47.9	23.7	28.1	2.2	33.8
Congo	Stroke	50.4	58.5	8.1	55.9	61.9	6.0	41.3	52.7	11.4	28.1	2.2	33.4
Kazakhstan	Stroke	60.1	58.2	-1.9	64.8	64.8	0.0	51.7	48.0	-3.8	28.4	2.0	31.9
Kiribati	Stroke	58.8	58.2	-0.6	67.9	67.2	-0.7	47.8	46.9	-0.9	28.3	2.0	32.9
Bahrain	Stroke	36.1	58.0	21.9	40.8	60.8	20.0	30.2	55.2	25.0	28.1	2.2	33.8
Burkina Faso	Stroke	56.8	58.0	1.2	67.6	68.1	0.5	42.9	44.9	2.0	28.2	2.0	32.8
Senegal	Stroke	56.7	57.9	1.2	61.5	62.8	1.3	50.5	51.1	0.7	28.3	2.1	32.7
Syrian Arab Republic	Stroke	51.5	57.8	6.3	50.3	55.2	4.8	51.2	57.3	6.2	28.2	2.0	33.0
Vanuatu	Stroke	49.7	57.7	8.0	59.6	65.3	5.7	38.6	48.2	9.7	28.9	2.4	57.4
Philippines	Stroke	46.5	57.7	11.2	52.1	65.8	13.6	37.9	47.1	9.2	28.5	1.8	32.4
Grenada	Stroke	37.9	57.5	19.6	47.4	64.6	17.2	21.9	48.2	26.3	28.0	2.2	35.0
Mongolia	Stroke	38.4	57.0	18.6	48.1	65.7	17.6	23.0	44.4	21.3	87.7	2.7	28.9
Bolivia (Plurinational State of)	Stroke	38.6	56.9	18.3	41.9	59.1	17.2	33.6	53.5	19.9	28.1	2.2	33.7
Saudi Arabia	Stroke	36.2	56.8	20.6	33.6	54.8	21.2	36.0	56.4	20.4	30.1	2.3	25.3
Mali	Stroke	52.2	56.5	4.3	49.2	54.3	5.2	54.8	57.3	2.5	28.2	2.1	33.2
Sierra Leone	Stroke	55.1	56.4	1.3	60.2	58.8	-1.4	48.8	53.0	4.2	28.2	2.0	33.2
India	Stroke	48.5	56.4	7.9	50.3	58.5	8.2	45.2	53.1	7.9	28.3	2.2	33.0
Turkmenistan	Stroke	57.0	56.3	-0.6	62.2	65.5	3.3	48.5	43.5	-5.0	27.9	17.1	33.7
Romania	Stroke	40.6	56.2	15.7	42.2	60.8	18.5	38.3	51.0	12.7	28.1	2.2	33.8
Rwanda	Stroke	33.7	55.7	22.0	42.4	63.2	20.8	19.8	42.2	22.4	28.1	2.2	33.4
Benin	Stroke	50.2	55.6	5.5	50.7	58.2	7.5	48.6	52.1	3.5	28.1	2.2	33.2
Russian Federation	Stroke	37.9	55.3	17.4	45.0	61.7	16.7	25.7	47.0	21.3	28.1	2.3	33.7

Guinea	Stroke	54.2	55.1	0.9	53.4	56.8	3.4	54.2	52.1	-2.0	28.4	2.2	36.7
Cameroon	Stroke	53.0	54.9	1.9	58.0	60.1	2.1	46.2	48.1	1.9	28.2	2.1	34.7
Morocco	Stroke	46.0	54.7	8.6	40.1	51.3	11.2	50.5	56.8	6.3	28.1	2.4	33.8
Niger	Stroke	55.5	54.6	-1.0	59.5	57.9	-1.6	50.1	49.4	-0.6	28.3	2.0	32.5
Saint Vincent and the Grenadines	Stroke	42.6	54.4	11.8	48.7	62.5	13.8	33.1	44.2	11.0	28.0	2.2	33.9
Sudan	Stroke	29.0	54.3	25.3	20.4	48.7	28.3	34.3	57.1	22.8	28.5	2.4	33.1
Georgia	Stroke	47.7	54.0	6.3	56.2	63.1	6.9	32.5	40.2	7.7	28.3	2.2	51.0
Namibia	Stroke	51.4	53.7	2.3	57.8	61.0	3.2	40.7	41.5	0.8	28.2	2.1	32.9
Suriname	Stroke	44.4	53.4	9.0	48.0	60.4	12.4	39.4	44.7	5.3	27.9	2.3	34.0
Jamaica	Stroke	43.6	53.3	9.7	48.2	58.8	10.6	37.6	46.2	8.5	28.1	2.2	33.5
Tajikistan	Stroke	46.1	53.2	7.1	52.0	57.2	5.3	37.2	47.2	10.0	28.8	2.1	32.5
South Sudan	Stroke	47.8	53.1	5.4	58.7	62.0	3.3	36.7	42.7	6.0	28.1	2.1	33.5
Burundi	Stroke	38.8	52.7	13.9	49.9	60.8	10.9	22.1	42.3	20.2	28.1	2.1	33.5
Democratic Republic of the Congo	Stroke	52.2	52.6	0.4	57.4	59.2	1.8	44.5	41.4	-3.1	28.3	2.1	32.5
Eritrea	Stroke	38.2	52.6	14.4	47.9	60.7	12.8	22.9	37.9	14.9	28.2	2.3	33.5
Togo	Stroke	55.1	52.6	-2.6	57.2	58.0	0.8	51.9	44.6	-7.3	28.4	2.1	30.8
Solomon Islands	Stroke	48.8	52.4	3.6	54.3	57.7	3.5	41.5	44.8	3.4	27.6	2.3	35.3
Gambia	Stroke	57.0	52.3	-4.6	62.3	57.6	-4.8	50.7	45.4	-5.3	28.7	1.5	31.3
Zambia	Stroke	47.1	52.3	5.2	54.5	60.1	5.6	37.0	40.9	3.9	25.9	2.2	33.6
Chad	Stroke	55.6	51.9	-3.7	59.0	57.2	-1.8	50.5	45.4	-5.2	28.7	1.9	30.5
Liberia	Stroke	53.7	51.7	-1.9	54.2	53.1	-1.1	51.8	49.5	-2.3	28.3	2.1	32.7
Tuvalu	Stroke	34.5	51.3	16.8	42.4	57.4	15.1	23.1	43.4	20.3	28.1	2.3	33.9
Madagascar	Stroke	45.4	51.3	5.8	56.0	58.4	2.5	32.1	40.7	8.6	28.1	2.1	33.6
Palestine	Stroke	30.2	51.2	21.0	36.0	56.3	20.2	21.6	43.6	22.0	27.7	2.4	35.9
Nepal	Stroke	38.3	50.9	12.6	34.3	54.1	19.8	40.4	45.9	5.4	28.0	2.3	33.3
Malawi	Stroke	51.3	50.2	-1.1	55.0	56.4	1.4	45.7	41.5	-4.1	28.3	2.1	32.6
Micronesia (Federated States of)	Stroke	41.5	50.0	8.5	48.9	57.3	8.4	32.5	40.9	8.4	28.0	2.3	34.0
Eswatini	Stroke	46.4	49.8	3.4	56.0	56.3	0.4	29.4	39.9	10.5	30.6	1.2	29.3
Nauru	Stroke	47.1	49.5	2.3	52.9	56.3	3.5	40.0	40.4	0.3	28.2	2.2	33.4
Saint Kitts and Nevis	Stroke	35.2	49.2	14.0	41.9	56.9	15.1	24.0	40.6	16.6	28.1	2.1	33.6
Democratic People's Republic of Korea	Stroke	47.0	48.9	1.8	52.3	55.5	3.2	39.0	40.1	1.1	28.0	2.2	33.7
Dominica	Stroke	40.0	48.1	8.0	50.1	56.9	6.9	23.9	38.8	14.9	28.0	2.2	34.4
Zimbabwe	Stroke	59.2	48.0	-11.2	65.1	54.8	-10.3	49.6	35.4	-14.2	27.9	2.3	34.6
Iraq	Stroke	39.4	47.8	8.3	46.4	54.9	8.5	30.9	39.1	8.2	28.2	2.2	32.9
Serbia	Stroke	28.5	47.5	19.1	31.5	50.1	18.6	25.0	44.1	19.1	28.0	2.2	33.7
Marshall Islands	Stroke	41.1	47.5	6.4	53.1	54.8	1.7	27.2	38.4	11.3	28.0	2.3	34.2
Bulgaria	Stroke	31.4	47.4	16.0	34.5	53.9	19.4	27.2	39.2	12.0	28.0	2.3	33.8
Somalia	Stroke	37.3	47.3	10.0	48.5	56.3	7.7	22.3	32.8	10.5	28.1	2.2	33.6
Indonesia	Stroke	48.8	46.5	-2.3	51.1	51.5	0.4	45.6	40.1	-5.5	100.0	1.7	41.7
Lao People's Democratic Republic	Stroke	20.4	43.4	23.0	22.0	47.1	25.1	17.5	38.2	20.7	28.1	2.3	33.9
Albania	Stroke	33.0	43.0	10.0	41.6	45.9	4.4	18.3	38.9	20.6	28.1	1.9	34.0
Bangladesh	Stroke	27.9	42.9	15.0	27.4	42.7	15.3	25.6	41.3	15.7	28.1	2.3	33.6
Viet Nam	Stroke	34.0	42.9	8.9	43.6	54.1	10.5	21.5	30.0	8.5	26.4	2.3	49.2
Egypt	Stroke	4.7	42.6	37.9	1.3	29.3	28.0	4.4	45.4	41.0	32.4	1.1	16.2
Mozambique	Stroke	42.3	42.0	-0.3	51.6	55.6	4.0	30.2	24.7	-5.5	28.2	2.6	33.1
Guinea-Bissau	Stroke	38.9	41.9	3.0	47.8	48.1	0.4	28.1	33.5	5.4	28.2	2.0	33.2
Guyana	Stroke	17.7	41.5	23.8	25.2	48.0	22.8	8.1	33.0	24.9	28.1	2.2	33.5
Myanmar	Stroke	21.3	40.8	19.6	26.2	50.0	23.8	14.7	29.5	14.9	28.1	2.2	33.6

Cambodia	Stroke	26.5	39.6	13.1	27.1	43.8	16.7	25.5	33.7	8.1	28.1	2.4	33.4
Central African Republic	Stroke	35.9	39.3	3.4	46.2	49.3	3.1	20.5	23.3	2.8	28.2	2.1	33.2
Timor-Leste	Stroke	32.7	39.2	6.5	30.2	42.0	11.8	33.9	35.0	1.1	28.2	7.0	33.9
North Macedonia	Stroke	38.6	36.3	-2.3	41.1	37.1	-3.9	34.1	36.1	1.9	28.8	1.0	32.0
Lesotho	Stroke	45.5	35.4	-10.1	56.3	46.2	-10.1	20.9	14.2	-6.7	28.2	2.2	32.9
Yemen	Stroke	19.7	34.8	15.1	20.9	36.0	15.1	16.7	32.0	15.3	28.1	2.3	33.7
Honduras	Stroke	47.0	34.1	-12.9	52.8	37.8	-15.0	38.0	28.6	-9.5	0.0	100.0	100.0
Afghanistan	Stroke	21.5	33.3	11.8	27.2	33.1	5.9	13.6	32.6	18.9	28.1	2.1	33.8
Papua New Guinea	Stroke	21.9	31.8	9.9	14.4	24.8	10.3	25.9	34.9	9.1	28.1	2.2	33.9
Montenegro	Stroke	38.3	28.3	-10.0	43.0	32.3	-10.7	32.1	24.5	-7.7	29.8	1.0	27.8
Haiti	Stroke	0.0	17.6	17.6	0.0	17.9	17.9	0.0	15.9	15.9	28.1	2.2	33.6

ICH, intracerebral hemorrhage; SAH, subarachnoid hemorrhage; IS, ischemic stroke; QCI, quality of care index

Table S6 The number of QCI growing countries stroke, ICH, SAH, and IS in women and men from 1990 to 2021

	**No. of QCI growing countries**		
**cause**	**both sexes**	**women**	**men**
**stroke**	184	188	182
**ICH**	176	171	176
**SAH**	183	181	181
**IS**	165	164	160

ICH, intracerebral hemorrhage; SAH, subarachnoid hemorrhage; IS, ischemic stroke; QCI, quality of care index

Table S7 QCI values by sex, age group and SDI regions for stroke, ICH, SAH, and IS in 1990 and 2021

		**QCI**																							
		**stroke**						**ICH**						**SAH**						**IS**					
		**both**	**both**	**female**	**female**	**male**	**male**	**both**	**both**	**female**	**female**	**male**	**male**	**both**	**both**	**female**	**female**	**male**	**male**	**both**	**both**	**female**	**female**	**male**	**male**
**location**	**age**	**1990**	**2021**	**1990**	**2021**	**1990**	**2021**	**1990**	**2021**	**1990**	**2021**	**1990**	**2021**	**1990**	**2021**	**2021**	**1990**	**2021**	**1990**	**1990**	**2021**	**1990**	**2021**	**1990**	**2021**
Global	<5	72.5	86.0	76.1	88.0	68.1	83.6	79.4	90.1	81.9	90.8	76.9	89.6	73.2	89.9	76.6	92.0	70.9	88.1	78.4	87.6	84.1	92.5	85.3	89.3
High SDI	<5	90.7	96.5	91.4	96.3	89.8	96.6	93.2	97.7	94.4	97.8	91.7	97.6	92.5	97.2	93.9	98.1	91.3	96.2	90.1	97.5	92.7	98.1	93.1	97.9
High-middle SDI	<5	80.0	92.4	81.9	92.4	77.8	92.5	86.2	95.7	88.0	95.8	84.5	95.5	76.9	95.6	78.5	96.2	76.4	95.1	86.0	93.8	88.7	95.4	91.1	95.4
Low SDI	<5	64.9	81.1	70.1	84.6	58.9	76.5	74.1	86.9	73.5	86.8	74.7	87.2	73.0	85.5	77.2	88.5	70.1	83.1	70.2	81.7	83.0	90.9	73.6	80.8
Low-middle SDI	<5	58.0	83.5	64.4	86.0	50.1	80.4	67.1	88.6	71.0	89.2	63.1	88.0	61.6	85.8	69.9	89.0	54.1	82.8	65.6	85.1	73.4	90.7	78.0	87.4
Middle SDI	<5	76.1	89.2	79.0	90.0	72.8	88.4	83.5	92.9	85.8	93.6	81.0	92.3	74.3	93.2	75.9	94.2	74.0	92.3	82.3	91.4	86.5	93.9	88.1	93.2
Global	5 to 9	86.4	94.5	86.2	94.9	86.3	93.7	89.4	95.2	88.9	94.5	88.7	94.6	74.8	90.1	70.6	89.8	80.6	91.7	88.0	92.9	89.5	95.1	82.3	85.3
High SDI	5 to 9	95.2	98.4	94.9	98.5	95.5	98.5	96.1	99.0	95.5	98.2	95.6	98.8	88.4	94.6	86.2	95.1	91.2	95.4	94.3	97.6	95.2	98.1	92.8	96.9
High-middle SDI	5 to 9	90.2	97.3	90.5	97.1	89.6	96.8	93.2	97.5	93.1	96.6	92.1	96.8	78.4	95.4	75.5	95.2	82.9	96.0	91.0	95.8	92.2	95.7	86.6	92.0
Low SDI	5 to 9	86.9	92.6	87.5	94.3	86.4	91.1	88.6	93.7	87.3	93.3	88.8	93.1	83.0	89.0	82.0	90.6	86.5	90.5	89.1	90.2	93.1	96.0	78.1	75.5
Low-middle SDI	5 to 9	78.1	92.3	76.6	92.5	78.7	91.5	81.6	93.5	80.0	92.3	81.7	93.3	66.9	85.3	63.0	84.6	73.6	87.8	81.3	90.3	82.3	92.9	74.9	81.2
Middle SDI	5 to 9	86.7	95.6	86.7	95.5	86.3	95.0	90.8	96.3	90.7	95.4	89.7	95.5	72.0	90.9	65.7	89.5	79.2	92.7	88.0	94.8	89.5	95.1	82.2	89.6
Global	10 to 14	84.4	90.4	83.9	90.3	85.9	91.0	86.8	90.0	84.2	87.6	88.2	91.2	82.4	89.7	88.7	93.4	74.9	85.2	87.2	90.9	88.5	92.2	79.7	82.5
High SDI	10 to 14	92.1	97.1	91.1	96.1	93.0	97.5	93.6	97.7	92.8	97.4	93.5	97.3	87.8	94.2	92.3	96.1	82.1	92.0	90.4	95.6	92.0	96.0	85.0	93.3
High-middle SDI	10 to 14	87.7	94.8	88.4	95.6	87.8	94.4	90.3	93.9	89.4	93.5	90.4	93.5	84.1	94.9	90.4	96.9	75.9	92.2	88.5	93.2	90.7	93.8	79.0	86.0
Low SDI	10 to 14	83.1	87.9	78.5	84.8	87.5	90.7	83.3	87.3	74.1	79.9	88.4	91.3	87.1	89.3	89.7	91.4	84.6	87.2	90.2	90.1	90.5	91.7	84.2	80.9

Low-middle SDI	10 to 14	76.5	87.1	75.7	87.6	78.9	87.4	79.3	87.2	74.9	84.5	81.8	88.6	77.2	85.5	85.5	91.0	67.1	78.8	82.2	87.9	82.4	89.9	76.1	77.1
Middle SDI	10 to 14	85.4	91.3	85.6	92.5	86.1	90.9	88.8	91.2	87.3	90.6	89.3	91.1	81.9	90.0	88.3	94.0	74.4	84.6	87.3	91.6	89.1	92.8	78.5	83.0
Global	15 to 19	66.8	74.3	77.7	83.2	63.2	72.3	66.5	70.3	71.2	73.4	68.4	73.3	66.5	80.1	75.9	84.8	58.1	76.6	70.0	74.8	78.0	82.1	68.6	73.2
High SDI	15 to 19	84.2	93.6	89.9	96.6	82.2	92.4	84.2	93.4	87.1	94.2	83.8	92.9	77.3	91.1	83.4	93.3	71.6	89.4	81.8	92.7	87.1	94.4	79.4	91.6
High-middle SDI	15 to 19	68.8	80.9	82.4	90.5	61.3	77.0	70.3	78.7	78.0	83.9	68.4	77.6	65.5	86.6	78.0	91.1	53.0	82.8	67.4	77.9	79.3	85.8	59.5	74.0
Low SDI	15 to 19	58.9	68.1	67.1	74.6	59.6	69.2	52.5	61.4	51.5	58.5	62.2	70.9	74.1	78.4	79.0	81.5	70.9	76.5	74.4	74.0	78.8	80.2	77.5	74.6
Low-middle SDI	15 to 19	59.9	69.9	68.3	78.8	61.1	69.1	57.2	66.3	58.5	68.1	64.5	71.2	66.8	75.2	73.1	79.8	62.6	72.5	64.1	69.3	69.4	77.1	70.6	69.3
Middle SDI	15 to 19	66.2	74.5	78.8	86.0	60.2	69.7	68.5	71.9	74.6	78.3	68.2	71.2	62.3	79.4	73.4	85.4	52.1	74.5	69.5	73.5	78.7	82.8	65.7	68.7
Global	20 to 24	68.8	73.9	77.8	82.8	78.2	81.2	69.7	71.3	67.6	70.6	78.3	79.1	66.1	77.0	73.6	82.8	74.5	82.4	75.3	78.2	82.4	85.8	66.9	68.1
High SDI	20 to 24	84.1	92.4	88.6	95.2	89.4	94.7	85.2	92.1	83.3	90.7	89.4	93.8	72.7	88.7	78.2	91.1	79.4	91.3	84.8	91.8	89.0	94.4	79.4	87.3
High-middle SDI	20 to 24	71.8	79.7	82.3	88.6	78.3	84.2	73.7	78.4	74.5	80.9	79.4	82.1	67.8	85.0	76.6	89.1	74.6	88.3	73.3	79.4	83.2	87.8	58.6	66.9
Low SDI	20 to 24	63.9	69.1	70.8	76.4	77.8	80.1	60.3	64.2	52.0	56.9	76.0	78.1	73.7	75.4	77.8	80.0	81.9	82.8	80.4	78.9	84.7	85.4	77.2	71.6
Low-middle SDI	20 to 24	59.5	68.7	68.7	78.7	73.4	77.6	59.3	66.9	55.1	65.6	72.3	76.0	61.4	69.5	69.8	77.6	71.4	76.8	70.3	74.2	76.4	82.8	66.9	63.1
Middle SDI	20 to 24	68.0	73.4	77.9	83.8	76.7	79.5	70.5	71.8	69.4	73.8	78.1	77.5	64.0	76.8	71.6	82.8	73.0	82.1	74.3	76.5	82.2	85.5	63.6	63.3
Global	25 to 29	68.1	75.3	76.7	84.0	70.0	75.0	61.3	65.8	69.7	75.0	64.0	66.6	64.7	76.6	75.1	84.8	71.0	80.0	73.0	77.9	79.7	85.4	74.0	75.6
High SDI	25 to 29	82.7	91.9	87.0	94.9	84.3	91.7	79.4	87.4	83.2	90.3	80.4	86.5	67.0	86.0	75.4	90.4	73.6	87.9	83.8	90.2	87.7	93.3	84.5	89.3
High-middle SDI	25 to 29	71.7	80.0	81.9	89.3	70.4	77.7	66.5	72.4	76.0	83.4	65.9	69.8	67.5	83.2	78.9	89.5	71.8	85.3	71.2	78.9	81.1	87.9	68.1	74.1
Low SDI	25 to 29	67.1	72.6	71.6	79.3	74.3	75.7	55.9	60.3	59.8	65.9	66.8	67.2	74.6	77.3	79.9	84.1	81.4	82.0	80.7	80.0	82.8	85.1	84.9	80.2
Low-middle SDI	25 to 29	57.5	69.7	67.1	79.1	62.2	70.5	48.3	60.3	58.8	69.8	53.9	62.6	59.8	69.6	72.2	79.9	67.1	74.8	66.7	73.7	72.0	81.7	72.7	72.4
Middle SDI	25 to 29	65.8	73.8	75.6	84.4	66.6	71.6	60.3	64.7	69.7	76.5	61.8	63.1	62.9	75.7	73.4	84.6	69.6	78.7	70.7	75.5	78.8	85.0	70.5	71.0
Global	30 to 34	60.5	68.6	70.4	79.4	59.5	65.5	53.7	57.7	66.3	71.9	51.4	53.9	62.0	74.5	68.9	81.2	57.8	70.5	69.7	74.5	78.7	84.6	68.7	70.5
High SDI	30 to 34	76.9	86.9	82.3	91.4	76.9	85.5	73.3	80.6	81.0	88.2	71.0	77.2	63.8	83.1	68.6	86.7	60.8	81.0	81.7	87.2	87.0	92.0	81.6	85.8
High-middle SDI	30 to 34	64.3	72.9	76.3	85.8	60.5	67.5	58.9	63.4	72.4	80.2	54.3	57.3	65.3	80.4	73.7	86.8	60.0	76.4	66.4	73.6	79.9	86.8	60.1	66.2
Low SDI	30 to 34	57.2	63.9	62.5	71.4	62.2	65.0	46.4	49.7	55.0	60.2	51.4	51.9	70.3	73.3	73.6	77.5	69.7	72.0	76.6	76.4	80.4	83.6	80.6	75.8
Low-middle SDI	30 to 34	49.8	61.6	61.1	73.0	50.0	59.4	41.3	51.3	56.7	66.1	39.5	48.0	57.5	66.1	67.0	74.5	51.8	61.7	64.7	70.4	72.0	80.4	67.6	67.9
Middle SDI	30 to 34	56.6	67.3	67.8	80.2	54.7	62.4	51.5	56.9	65.2	73.3	48.4	51.4	59.7	74.9	66.1	82.0	55.9	70.3	66.5	72.5	76.7	84.4	64.6	66.6
Global	35 to 39	55.9	66.1	65.5	76.0	46.0	56.3	56.0	60.9	68.5	73.6	53.4	57.7	59.3	73.3	64.4	78.4	56.3	70.1	62.1	70.3	72.4	81.3	59.7	65.2
High SDI	35 to 39	73.0	83.8	79.8	89.5	66.3	78.4	72.4	79.6	82.1	89.0	68.7	75.5	62.3	81.5	67.3	85.4	58.4	78.3	78.1	84.4	85.0	89.8	75.9	82.4
High-middle SDI	35 to 39	58.0	68.7	70.8	82.6	44.8	55.7	58.6	63.8	72.8	80.1	53.8	58.2	61.6	77.6	68.6	84.4	57.1	72.7	55.5	67.3	71.8	82.7	47.2	58.2
Low SDI	35 to 39	55.8	63.2	60.3	69.6	52.2	57.2	53.4	56.7	61.7	66.0	56.2	57.8	69.6	73.2	71.5	76.0	69.6	72.4	73.5	74.2	78.2	82.9	75.6	70.9
Low-middle SDI	35 to 39	49.5	60.7	57.1	69.1	42.2	52.7	49.0	57.2	60.9	68.3	49.0	56.2	59.6	66.8	64.5	70.8	57.4	65.3	59.5	67.5	65.4	77.0	62.9	64.7
Middle SDI	35 to 39	49.9	63.5	61.1	75.9	37.6	51.0	52.9	58.8	66.8	73.8	49.3	54.3	54.3	72.8	59.0	78.5	51.6	68.8	56.5	66.8	68.2	79.8	53.2	59.9
Global	40 to 44	52.9	64.2	60.7	72.9	45.8	56.7	57.2	62.0	69.8	74.5	48.1	53.1	59.0	72.6	60.8	75.1	58.8	71.8	60.3	69.1	72.5	80.5	50.8	59.7
High SDI	40 to 44	72.1	83.9	77.8	89.1	67.3	79.5	73.3	80.9	83.0	89.8	65.3	73.7	63.9	80.0	65.1	82.0	63.6	79.0	77.7	84.6	85.6	90.4	72.7	80.9
High-middle SDI	40 to 44	52.9	67.3	64.1	79.5	42.7	57.2	58.0	65.0	72.0	79.8	47.2	54.4	60.1	76.8	63.6	80.9	58.5	74.5	49.7	64.6	70.1	81.8	30.0	48.7
Low SDI	40 to 44	50.5	58.9	56.3	66.0	45.6	52.6	53.3	56.2	65.1	68.3	45.8	48.1	66.7	70.7	68.4	73.0	67.4	70.9	70.6	72.2	76.4	80.9	69.3	65.8
Low-middle SDI	40 to 44	48.2	58.8	53.9	65.6	43.3	52.9	53.1	59.0	65.2	70.5	45.2	51.2	60.0	66.8	61.9	67.9	60.4	67.8	61.3	67.2	68.1	76.3	59.6	61.3
Middle SDI	40 to 44	45.4	61.2	54.4	71.8	36.8	51.9	54.0	60.2	67.4	74.0	44.2	50.4	53.4	72.1	54.4	74.8	53.6	71.0	53.1	65.3	67.7	78.9	40.1	53.1
Global	45 to 49	53.2	65.3	62.6	73.4	49.3	60.8	55.6	61.1	69.2	73.2	49.2	55.3	57.5	71.2	64.3	76.3	50.1	66.1	58.9	69.0	68.8	78.1	57.9	67.0
High SDI	45 to 49	72.8	85.7	78.3	89.1	69.9	82.6	71.3	81.1	81.6	89.2	64.7	75.7	61.3	76.5	66.7	81.0	55.4	71.7	76.7	86.3	83.6	90.7	77.1	86.6
High-middle SDI	45 to 49	53.8	69.3	65.4	79.2	47.5	62.6	57.3	65.2	71.5	78.4	49.8	57.9	57.9	75.0	66.0	81.1	49.2	68.6	49.1	66.2	65.1	79.3	43.1	61.5
Low SDI	45 to 49	47.5	57.5	57.1	65.9	44.3	53.8	49.4	53.0	63.9	66.3	43.6	47.8	63.4	68.9	69.8	73.9	57.6	64.5	66.7	69.0	72.4	77.0	68.1	67.0
Low-middle SDI	45 to 49	47.9	58.8	55.7	66.0	46.8	56.3	51.7	57.5	64.8	69.1	47.1	53.1	59.5	66.4	65.8	70.4	53.3	62.9	58.3	65.4	63.2	72.9	62.3	65.4
Middle SDI	45 to 49	46.3	62.3	58.1	72.2	40.8	56.4	53.1	59.4	67.9	72.6	45.8	53.0	52.3	70.6	59.9	76.0	43.9	65.0	52.2	65.8	64.6	76.5	48.2	62.6
Global	50 to 54	50.6	64.0	61.0	73.0	46.8	60.3	63.5	68.9	68.6	73.4	56.5	62.8	53.2	71.6	55.4	73.5	53.0	71.4	52.8	65.9	63.2	74.6	53.2	66.0
High SDI	50 to 54	72.5	86.5	78.5	90.9	71.4	85.5	78.4	87.6	82.2	90.7	72.5	83.1	64.9	79.5	65.3	80.6	65.8	79.5	74.0	86.5	79.5	90.1	78.1	89.7
High-middle SDI	50 to 54	52.4	67.8	64.0	77.6	47.0	63.3	65.1	73.0	70.3	78.0	58.2	66.6	54.8	75.9	58.0	78.6	53.7	74.6	43.8	63.1	59.3	74.5	39.7	61.7
Low SDI	50 to 54	43.5	55.0	54.5	64.3	39.6	51.6	57.6	61.4	62.6	65.5	51.0	55.8	60.9	68.5	64.1	70.1	61.0	69.6	61.2	65.8	67.6	73.4	62.9	64.6

Low-middle SDI	50 to 54	43.5	55.1	54.0	63.9	40.7	52.6	59.9	64.7	65.3	68.8	52.8	58.9	54.9	64.3	58.9	64.8	54.1	66.0	51.7	59.9	58.4	68.1	56.1	60.8
Middle SDI	50 to 54	41.2	60.7	53.9	71.7	35.6	55.2	60.6	67.6	66.3	73.1	53.1	60.5	41.5	70.4	42.8	72.7	42.2	69.6	44.3	62.3	58.1	72.9	41.3	60.9
Global	55 to 59	50.5	64.4	57.2	70.2	48.2	62.0	63.7	70.1	67.6	72.7	55.9	63.9	48.7	71.5	53.2	73.0	47.1	71.7	48.4	62.2	58.1	70.5	50.5	63.2
High SDI	55 to 59	73.3	88.0	76.9	90.8	72.6	87.2	80.5	90.9	82.5	91.0	74.7	87.8	67.6	83.3	68.6	83.4	68.6	83.8	69.8	85.3	75.1	88.7	74.1	88.3
High-middle SDI	55 to 59	51.0	68.0	59.5	74.7	46.7	64.6	64.0	74.4	68.5	77.4	55.5	68.0	48.3	75.7	54.8	78.2	44.5	74.5	37.9	58.3	52.5	69.3	36.2	57.7
Low SDI	55 to 59	42.5	52.8	49.0	58.0	41.1	51.5	56.9	60.3	59.9	62.0	50.6	55.6	58.8	66.4	62.3	65.9	59.5	69.6	56.7	61.5	63.0	69.1	59.7	61.0
Low-middle SDI	55 to 59	43.7	52.6	48.7	57.7	43.7	51.8	61.1	64.3	64.4	66.9	54.4	58.5	51.1	59.8	55.6	59.6	50.6	62.8	48.6	53.1	54.3	61.6	54.9	55.0
Middle SDI	55 to 59	39.7	61.1	48.9	68.8	35.6	57.0	59.9	68.8	65.0	72.7	50.8	61.2	31.8	69.6	36.6	71.6	30.3	69.3	39.8	59.1	52.9	68.9	39.2	58.8
Global	60 to 64	51.4	64.4	57.5	69.9	52.0	63.9	60.4	68.0	67.0	73.2	50.5	60.0	51.6	75.9	50.7	73.4	49.4	73.6	54.3	66.6	57.2	69.7	63.5	73.4
High SDI	60 to 64	73.7	88.2	77.1	90.9	73.9	87.6	79.5	90.4	83.5	92.7	73.1	86.9	75.6	88.8	70.6	85.4	75.5	85.5	73.0	88.0	73.2	88.3	81.4	92.8
High-middle SDI	60 to 64	52.0	67.2	59.7	73.9	50.7	65.3	61.0	71.2	68.0	77.3	50.5	62.4	51.2	76.6	53.3	76.3	46.1	72.5	47.2	65.1	52.2	69.2	55.5	71.8
Low SDI	60 to 64	39.4	49.7	46.7	56.3	40.5	49.8	50.6	56.8	56.9	61.7	40.4	48.8	64.6	71.6	63.3	67.2	63.9	71.9	53.9	58.6	58.2	64.8	60.8	61.7
Low-middle SDI	60 to 64	39.9	51.7	43.8	56.0	43.9	53.7	56.1	63.1	62.0	67.6	46.9	55.8	54.7	65.9	53.0	60.3	54.5	67.6	45.8	53.7	46.7	57.5	57.9	61.6
Middle SDI	60 to 64	40.9	60.4	48.7	67.8	41.1	58.8	55.9	65.7	63.5	72.3	44.6	56.1	26.9	72.1	25.5	69.8	26.7	70.1	49.7	63.9	54.9	68.3	57.6	70.1
Global	65 to 69	52.0	64.5	56.8	69.1	47.2	60.1	57.1	64.1	60.9	67.1	53.4	61.2	52.4	74.5	54.6	76.4	50.7	73.0	58.2	68.4	60.0	70.9	57.2	67.3
High SDI	65 to 69	74.3	88.9	76.8	90.9	71.8	87.3	76.2	87.7	78.3	89.1	73.8	86.3	77.0	88.9	76.0	89.8	77.9	87.1	73.5	87.8	74.1	88.3	74.3	88.7
High-middle SDI	65 to 69	50.4	67.2	56.2	72.6	44.3	61.9	55.8	66.4	60.4	70.6	51.2	62.1	49.4	74.5	54.0	78.1	44.1	71.5	50.3	66.8	52.4	69.7	48.6	65.3
Low SDI	65 to 69	38.4	47.6	45.3	53.6	31.7	41.5	46.2	53.1	48.3	54.4	43.7	51.7	65.6	70.6	68.8	70.9	65.2	71.6	57.6	59.9	61.6	65.4	53.8	53.9
Low-middle SDI	65 to 69	39.8	48.7	43.1	51.9	36.8	45.9	53.7	58.9	55.9	60.2	51.6	57.8	55.7	63.6	58.6	62.0	55.3	66.2	50.4	54.8	52.1	57.8	49.8	52.7
Middle SDI	65 to 69	40.4	60.3	46.7	66.6	34.2	54.2	51.3	60.9	55.6	65.2	47.2	56.6	25.7	68.9	24.9	70.7	27.6	68.1	54.0	66.2	58.3	69.9	50.5	64.0
Global	70 to 74	52.6	65.9	65.5	76.6	46.5	59.4	52.1	59.9	67.6	72.6	53.4	60.9	57.6	79.2	74.5	87.9	55.2	76.8	63.2	73.1	71.6	80.4	58.3	67.8
High SDI	70 to 74	74.9	90.3	82.2	94.5	70.4	86.3	73.5	84.7	81.7	89.3	73.5	84.4	82.6	91.4	88.6	94.7	82.3	89.4	76.6	91.6	81.7	93.8	73.1	88.8
High-middle SDI	70 to 74	48.4	66.9	62.4	77.7	41.6	60.0	49.2	59.6	66.3	72.9	49.8	59.9	52.3	77.0	72.5	86.9	45.9	73.8	55.9	71.8	65.6	79.5	51.0	66.2
Low SDI	70 to 74	42.7	48.8	59.3	63.5	34.4	41.2	43.0	49.7	59.7	63.8	46.5	54.0	75.0	76.9	85.9	85.6	73.4	76.6	60.4	61.1	71.1	72.5	52.6	51.7
Low-middle SDI	70 to 74	42.0	49.0	55.2	62.0	37.9	43.7	50.3	55.2	64.6	68.4	53.7	57.9	66.6	71.9	80.1	82.0	65.3	72.2	53.6	57.1	63.0	67.4	49.4	50.8
Middle SDI	70 to 74	40.0	60.5	56.3	73.8	32.7	52.2	42.2	54.1	60.7	69.4	44.7	54.6	28.3	71.7	53.9	83.0	30.4	70.2	59.6	70.4	70.5	79.5	52.1	63.3
Global	75 to 79	61.5	72.3	69.5	79.1	50.8	62.5	58.1	64.5	71.8	75.4	56.7	63.6	58.2	77.0	75.0	87.4	53.3	71.9	67.0	76.8	74.2	82.8	58.9	68.8
High SDI	75 to 79	76.5	90.3	81.7	93.2	68.6	84.2	74.1	84.1	82.7	89.6	71.8	81.5	80.2	88.8	88.1	94.6	77.7	83.6	76.3	90.5	81.6	92.6	69.5	86.2
High-middle SDI	75 to 79	54.1	72.1	63.0	78.7	43.4	62.6	53.9	63.4	68.8	74.3	52.8	62.7	52.1	73.2	72.0	84.8	43.5	68.1	57.4	75.0	66.0	81.1	49.2	67.1
Low SDI	75 to 79	56.2	60.6	66.2	69.6	42.9	48.6	50.4	55.7	65.1	68.2	51.0	57.3	73.8	76.6	86.2	86.5	68.8	73.2	68.2	69.0	76.2	77.3	57.8	57.7
Low-middle SDI	75 to 79	53.3	59.2	61.9	67.5	42.4	48.3	53.7	58.2	67.7	70.8	53.8	58.2	64.9	70.5	80.1	82.0	60.1	67.8	62.1	65.4	69.7	73.4	53.4	55.6
Middle SDI	75 to 79	52.7	68.5	62.6	76.9	40.1	56.8	48.7	60.1	64.4	72.1	49.2	59.5	27.6	69.5	51.8	82.1	29.3	65.4	66.9	74.9	75.2	82.3	56.5	65.1
Global	All ages	49.6	60.8	53.6	65.5	44.8	55.1	49.2	53.2	56.7	60.4	48.6	52.4	49.4	70.1	55.1	73.4	42.0	65.7	49.1	60.2	51.8	65.4	53.1	60.8
High SDI	All ages	66.7	81.8	67.2	81.8	65.7	81.0	68.0	76.2	72.9	79.9	66.8	75.1	69.3	83.5	70.6	84.3	66.8	81.1	57.4	78.0	57.0	78.3	64.0	81.5
High-middle SDI	All ages	43.2	58.1	46.8	62.3	39.0	52.9	46.8	51.4	54.7	59.4	46.2	50.1	47.3	68.5	54.3	72.3	37.9	63.6	33.6	51.9	34.8	55.9	41.0	54.5
Low SDI	All ages	47.7	57.8	53.7	63.2	40.6	51.1	43.5	51.6	49.6	55.8	45.1	53.7	63.3	71.3	67.7	73.7	59.0	69.0	62.4	65.4	69.6	73.5	60.3	62.0
Low-middle SDI	All ages	45.1	52.8	49.4	57.2	39.8	47.5	46.1	51.6	53.2	57.2	46.4	52.4	53.4	64.1	59.9	66.7	46.0	61.4	52.3	53.7	56.9	60.0	54.1	53.8
Middle SDI	All ages	43.7	56.2	50.1	63.5	36.0	47.8	45.1	47.8	53.5	56.8	44.4	46.2	32.7	65.3	38.8	69.4	25.1	60.1	53.5	58.3	61.2	66.8	52.0	56.1
Global	Age-standardized	51.6	65.7	55.9	70.4	45.9	59.6	55.7	62.1	61.7	67.4	49.3	56.5	51.7	74.3	51.8	73.6	50.0	72.7	57.6	70.9	64.9	77.5	49.7	62.6
High SDI	Age-standardized	72.4	90.2	74.5	91.9	69.4	88.0	76.2	86.9	80.2	90.0	71.8	83.7	73.5	88.2	70.5	86.3	74.1	86.6	70.3	89.1	74.8	91.5	65.0	85.9
High-middle SDI	Age-standardized	44.9	64.3	50.4	69.6	38.0	57.4	54.3	62.6	61.7	69.4	45.9	55.0	49.8	75.0	52.1	75.3	45.2	72.3	45.5	65.9	54.8	73.2	35.9	56.8
Low SDI	Age-standardized	46.6	54.4	51.4	59.0	40.0	48.0	47.3	52.7	51.1	54.9	43.8	50.8	66.7	73.1	67.0	71.2	65.9	73.4	63.3	65.2	71.1	73.7	53.2	53.6
Low-middle SDI	Age-standardized	44.2	53.5	46.9	57.1	40.2	48.6	51.1	57.1	55.4	60.7	47.0	53.8	56.3	66.5	57.0	63.9	55.1	67.6	55.3	60.2	61.8	68.1	47.9	50.6
Middle SDI	Age-standardized	41.1	59.6	47.2	66.5	33.3	50.5	47.2	55.8	54.2	63.0	40.3	48.1	26.8	68.6	25.6	68.4	28.3	66.9	56.7	67.4	66.3	76.2	45.0	55.8
Global	80 to 84	65.9	75.7	73.0	81.5	59.2	69.1	56.4	61.5	60.2	64.7	53.8	59.3	62.9	77.6	66.5	81.1	61.5	74.9	69.9	79.8	77.1	85.3	68.5	77.2
High SDI	80 to 84	78.0	91.7	83.2	94.5	72.6	87.9	74.2	83.3	76.4	85.1	72.4	81.6	82.2	87.9	83.9	90.6	81.8	84.7	78.1	92.5	83.9	94.7	76.1	90.7
High-middle SDI	80 to 84	55.1	72.1	63.0	77.9	49.4	66.3	47.0	55.5	52.2	59.0	42.9	53.0	53.6	70.9	58.2	74.1	50.6	69.0	58.4	75.8	66.9	81.4	60.0	74.4
Low SDI	80 to 84	66.5	69.1	75.3	77.3	56.2	59.5	54.4	58.0	55.9	58.2	53.9	59.1	76.9	79.8	83.9	84.2	72.7	77.2	72.8	73.8	81.6	82.6	67.3	67.8

Low-middle SDI	80 to 84	62.3	64.9	69.4	71.9	55.3	58.0	56.7	58.5	57.6	59.4	57.1	59.0	70.1	73.6	75.7	76.7	67.2	72.7	66.4	68.2	74.3	76.2	64.2	65.5
Middle SDI	80 to 84	58.7	71.7	67.1	79.3	50.7	62.9	40.9	52.7	44.7	56.5	39.4	49.8	33.2	69.3	33.5	72.7	39.7	68.0	70.0	78.4	77.7	85.3	67.4	74.0
Global	85 to 89	76.3	83.0	84.3	89.6	67.9	73.7	61.9	63.9	72.5	75.6	61.9	60.5	69.7	79.8	69.8	81.1	71.5	79.1	78.8	85.8	85.8	90.9	75.5	81.8
High SDI	85 to 89	84.5	94.0	89.6	96.1	79.1	90.5	78.1	84.0	83.5	88.1	79.4	84.4	84.5	87.7	84.0	88.6	85.8	86.1	84.4	93.6	89.4	95.1	82.4	93.4
High-middle SDI	85 to 89	65.4	78.6	76.9	86.8	55.0	66.9	49.9	54.3	65.4	70.8	44.5	45.4	56.4	71.2	56.2	72.0	59.3	72.2	68.6	81.8	79.3	88.4	65.0	76.6
Low SDI	85 to 89	79.0	80.6	86.7	88.1	69.5	70.5	62.8	65.2	69.8	71.8	67.4	69.4	81.0	83.0	84.7	85.4	79.9	82.5	82.7	83.5	89.2	90.1	77.2	76.9
Low-middle SDI	85 to 89	74.0	76.7	81.8	84.3	66.6	68.6	62.9	65.4	69.8	72.6	68.0	69.3	74.9	78.0	76.3	77.7	76.4	80.0	76.7	78.9	84.0	86.1	73.3	74.4
Middle SDI	85 to 89	70.6	78.2	81.0	87.7	58.8	62.8	43.9	49.3	59.6	68.0	44.1	39.4	45.2	70.7	41.6	72.3	55.0	71.4	78.1	83.5	85.9	90.4	72.8	75.5
Global	95 plus	75.5	83.0	88.9	92.4	80.7	87.0	68.5	68.1	82.3	82.5	74.8	73.7	71.1	77.1	80.0	85.0	77.8	80.0	75.3	82.5	90.1	93.2	77.8	84.3
High SDI	95 plus	81.4	90.1	91.7	95.6	87.0	94.1	81.3	80.7	89.3	89.3	83.5	83.0	77.5	80.8	84.8	87.8	83.0	82.6	79.4	87.1	91.8	94.9	82.5	89.5
High-middle SDI	95 plus	58.8	73.5	80.6	88.3	68.2	76.6	54.2	52.3	74.4	74.7	66.3	60.0	53.4	62.0	65.5	73.2	68.7	70.9	61.0	75.2	83.7	90.3	66.6	76.1
Low SDI	95 plus	77.7	80.6	90.7	92.0	78.4	81.3	65.8	68.4	79.9	80.7	73.0	75.8	77.3	80.4	88.4	89.1	75.2	80.1	78.5	80.8	92.3	93.3	76.2	78.5
Low-middle SDI	95 plus	73.7	78.0	88.1	90.3	76.8	80.3	65.3	69.2	79.6	82.6	72.3	74.3	73.7	78.6	84.3	87.0	74.9	79.4	74.0	77.6	89.9	91.6	74.3	77.4
Middle SDI	95 plus	71.2	78.9	86.6	90.6	76.6	82.0	50.9	54.0	70.5	74.2	67.5	65.2	60.2	71.9	69.0	80.1	75.6	78.8	74.0	80.7	89.7	92.7	75.2	81.1

ICH, intracerebral hemorrhage; SAH, subarachnoid hemorrhage; IS, ischemic stroke; QCI, quality of care index

Table S8 QCI values by sex, age group and 21 GBD regions for stroke, ICH, SAH, and IS in 1990 and 2021

		QCI																							
		stroke						ICH						SAH						IS					
		both	both	female	female	male	male	both	both	female	female	male	male	both	both	female	female	male	male	both	both	female	female	male	male
**location**	**age**	**1990**	**2021**	**1990**	**2021**	**1990**	**2021**	**1990**	**2021**	**1990**	**2021**	**1990**	**2021**	**1990**	**2021**	**1990**	**2021**	**1990**	**2021**	**1990**	**2021**	**1990**	**2021**	**1990**	**2021**
Andean Latin America	<5	66.1	84.5	72.5	86.5	58.6	82.3	78.4	90.8	82.7	91.6	72.8	89.9	65.4	85.9	71.4	89.2	61.3	83.3	64.5	87.9	77.4	92.0	75.4	90.5
Australasia	<5	91.6	94.6	92.1	94.2	91.2	95.2	96.0	97.8	96.0	97.3	96.1	98.4	94.6	97.9	96.2	98.4	92.7	97.1	90.5	95.5	94.1	96.8	92.1	96.0
Caribbean	<5	64.0	74.4	66.1	76.3	62.2	72.3	75.1	83.3	77.2	85.0	73.9	81.6	61.3	70.0	61.6	70.4	63.6	71.5	65.9	77.5	72.6	82.1	79.3	85.8
Central Asia	<5	87.1	89.7	87.9	90.3	86.3	89.3	90.4	94.1	91.7	94.1	88.6	94.0	91.5	93.5	92.5	94.2	90.8	93.0	90.2	91.9	92.5	94.3	93.2	93.0
Central Europe	<5	85.2	92.2	87.7	92.5	81.9	91.8	90.5	95.6	92.1	95.5	88.7	95.8	88.8	96.3	91.2	96.8	86.4	95.9	89.1	94.2	92.4	95.9	90.9	94.6
Central Latin America	<5	82.5	89.4	84.6	90.3	80.3	88.5	89.1	94.0	90.7	94.7	87.5	93.3	86.8	92.0	88.6	93.9	85.4	90.3	84.5	92.8	88.7	94.6	89.0	94.7
Central Sub-Saharan Africa	<5	58.4	85.6	59.3	86.5	57.5	84.8	70.0	90.2	67.0	89.0	72.5	91.1	70.0	90.1	70.5	91.8	70.9	89.0	61.6	87.3	74.7	92.2	72.5	89.0
East Asia	<5	78.1	93.7	80.5	93.2	75.6	94.2	86.0	96.9	88.0	96.8	83.8	96.8	70.1	96.5	70.1	96.8	71.7	96.3	87.6	94.8	90.2	95.8	91.9	96.4
Eastern Europe	<5	88.9	91.7	90.4	92.3	86.7	90.3	92.8	95.4	93.6	95.4	91.9	95.3	93.6	95.4	95.1	96.5	92.1	94.3	91.0	93.5	94.3	95.7	90.9	93.4
Eastern Sub-Saharan Africa	<5	71.0	85.5	74.4	87.1	67.2	83.7	76.7	88.9	77.5	89.2	76.3	89.0	80.4	90.0	84.4	92.8	77.6	87.8	82.0	89.2	87.1	92.4	86.1	91.0
High-income Asia Pacific	<5	89.9	94.5	91.0	94.4	88.6	94.4	94.3	97.8	95.1	97.9	93.5	97.8	93.8	98.6	95.0	98.9	92.6	98.2	92.5	94.8	95.1	96.4	93.4	95.1
High-income North America	<5	94.6	99.7	94.5	99.6	94.7	99.5	97.0	99.1	97.6	99.6	96.3	98.6	93.1	96.6	95.0	98.6	91.0	94.3	91.5	99.7	93.4	99.7	94.5	99.7
North Africa and Middle East	<5	51.5	85.1	55.5	86.2	46.8	84.2	56.2	88.1	59.5	88.6	52.7	87.7	62.2	89.7	68.5	91.5	55.7	87.9	55.6	86.0	58.2	89.2	78.5	90.6
Oceania	<5	79.3	81.4	79.2	81.5	80.0	81.8	84.0	86.3	81.7	84.2	86.3	88.4	75.6	80.4	74.3	78.4	78.5	83.4	80.5	81.5	82.7	85.4	89.2	88.5
South Asia	<5	60.2	80.9	66.7	83.5	52.2	77.7	68.4	86.6	73.7	88.4	62.3	84.5	56.7	81.6	67.6	85.8	47.8	78.3	71.3	84.2	77.9	89.0	81.1	87.8
Southeast Asia	<5	70.9	84.2	74.6	86.0	67.0	82.1	77.9	88.5	80.6	90.0	75.2	86.7	80.3	90.3	82.0	91.7	79.4	89.2	79.2	88.1	83.6	91.3	86.5	91.4
Southern Latin America	<5	85.3	92.9	87.0	93.0	83.3	92.9	92.4	96.3	93.3	96.2	91.3	96.7	85.4	96.6	88.3	97.2	83.2	96.0	86.2	94.5	89.5	96.1	90.1	95.0
Southern Sub-Saharan Africa	<5	86.1	89.3	86.8	90.2	85.2	88.0	90.1	92.0	87.9	91.5	92.3	92.6	91.7	93.4	91.6	93.6	92.2	93.5	86.5	91.0	90.8	93.9	87.7	91.6
Tropical Latin America	<5	81.4	88.7	83.1	89.8	80.0	87.7	88.4	93.9	89.5	94.3	87.4	93.5	84.0	91.9	84.9	92.6	84.0	91.6	85.1	92.1	89.4	95.0	88.5	92.4
Western Europe	<5	89.7	94.6	90.5	94.5	88.9	94.7	93.9	97.4	94.6	97.2	93.3	97.7	91.8	97.3	92.3	97.2	91.5	97.3	89.0	95.6	92.0	96.9	91.4	95.7
Western Sub-Saharan Africa	<5	73.6	82.4	80.8	87.0	64.1	75.8	82.0	88.4	81.7	88.0	82.1	88.9	85.8	89.4	90.6	92.8	81.2	86.0	72.5	80.5	90.4	92.9	60.1	70.4
Andean Latin America	5 to 9	81.1	90.3	79.1	90.0	82.7	90.1	88.3	94.3	87.2	93.2	88.4	93.8	63.5	75.9	54.2	71.7	73.6	81.5	83.4	93.3	85.6	93.9	77.8	88.7
Australasia	5 to 9	97.1	99.4	96.1	98.9	97.7	99.6	97.9	99.6	96.3	98.3	98.1	99.7	92.5	97.7	90.4	97.6	94.6	98.2	95.3	98.7	95.1	98.3	92.9	97.1
Caribbean	5 to 9	85.5	86.8	84.5	86.2	86.4	87.3	90.7	91.8	90.7	92.2	89.4	90.3	69.8	68.9	63.8	62.5	77.9	77.4	88.9	90.6	87.5	89.8	91.2	92.1

Central Asia	5 to 9	96.5	98.4	95.3	97.6	96.7	98.2	97.1	98.6	95.5	97.0	96.9	98.4	93.5	95.8	88.3	93.4	96.7	97.2	92.7	95.9	92.4	95.2	88.7	91.6
Central Europe	5 to 9	93.9	98.8	93.4	98.3	93.7	98.7	95.6	99.1	94.8	97.8	95.1	98.9	88.3	96.7	85.5	96.1	91.3	97.3	94.0	97.6	93.7	96.8	89.4	94.5
Central Latin America	5 to 9	91.4	96.2	90.2	95.8	91.9	96.1	95.2	98.4	94.3	97.4	94.9	98.0	80.3	87.7	74.6	85.7	85.7	90.1	88.1	97.1	88.8	96.6	83.3	94.2
Central Sub-Saharan Africa	5 to 9	84.5	93.9	80.9	93.1	87.3	94.2	85.3	93.4	80.2	91.1	88.4	94.3	81.1	90.4	78.7	91.1	85.3	91.8	89.2	95.1	90.3	95.5	84.3	90.5
East Asia	5 to 9	88.8	97.1	89.7	97.1	87.8	96.4	93.7	97.4	94.4	96.7	92.2	96.5	68.4	94.4	62.3	94.2	76.2	95.0	92.2	95.9	93.6	95.7	88.5	91.9
Eastern Europe	5 to 9	97.6	99.1	96.6	98.3	97.5	98.8	97.3	98.9	95.2	97.0	97.7	98.8	95.1	97.6	93.8	96.9	95.9	97.8	96.2	98.0	95.4	96.8	91.0	94.0
Eastern Sub-Saharan Africa	5 to 9	90.9	95.9	90.8	95.6	90.7	95.8	90.9	95.5	89.6	94.1	91.0	95.7	89.3	93.2	90.9	94.6	90.3	93.7	95.5	96.8	95.4	96.8	91.2	92.7
High-income Asia Pacific	5 to 9	92.2	98.6	91.5	98.1	92.5	98.5	95.4	99.1	94.6	97.9	94.9	99.0	84.6	95.3	81.8	95.3	88.0	95.9	92.6	98.2	93.5	97.4	87.6	95.4
High-income North America	5 to 9	97.5	98.0	97.8	98.6	98.0	98.3	98.7	99.2	98.5	99.2	98.3	98.8	89.6	91.1	88.3	92.6	92.1	92.2	95.8	97.3	97.3	98.9	97.4	98.7
North Africa and Middle East	5 to 9	65.6	91.0	63.1	90.7	68.1	91.2	65.4	90.8	63.8	90.0	66.2	90.5	66.9	89.0	63.9	89.6	73.3	90.8	65.4	86.5	65.7	87.6	62.9	82.2
Oceania	5 to 9	91.9	91.8	90.6	90.4	93.4	93.7	94.4	94.1	84.5	83.7	99.9	99.8	73.0	73.4	85.6	85.1	73.5	74.8	94.3	93.9	96.9	96.9	93.2	93.8
South Asia	5 to 9	83.2	93.6	80.9	92.5	84.1	93.3	85.9	94.2	83.3	92.1	86.7	94.4	68.3	85.7	66.3	84.0	73.7	88.2	87.7	94.2	86.8	93.6	82.1	88.3
Southeast Asia	5 to 9	84.9	92.4	83.6	92.6	85.5	91.4	89.4	94.2	88.2	93.8	89.2	93.1	70.2	84.8	60.2	82.0	79.2	88.1	88.7	93.0	90.0	93.8	82.7	87.2
Southern Latin America	5 to 9	92.3	97.8	91.3	97.4	92.8	97.8	96.5	98.9	95.9	97.6	96.2	98.8	78.5	92.8	73.5	92.1	83.7	93.9	88.4	96.9	88.2	96.5	84.4	94.1
Southern Sub-Saharan Africa	5 to 9	92.6	94.8	93.2	94.9	90.4	93.6	88.4	91.9	88.4	91.3	86.6	91.0	94.5	93.2	93.6	92.6	95.8	94.6	94.2	95.1	94.1	94.6	88.8	90.8
Tropical Latin America	5 to 9	91.7	95.4	90.5	94.4	91.9	95.5	94.9	97.1	93.4	95.4	94.6	97.3	79.6	87.2	72.6	83.0	85.6	90.5	93.0	98.0	93.8	97.7	87.4	94.3
Western Europe	5 to 9	94.6	99.1	93.6	98.6	95.1	99.2	96.0	99.3	94.7	97.9	96.0	99.3	84.0	96.1	77.6	95.2	89.3	96.9	92.4	98.8	93.1	98.3	87.5	96.4
Western Sub-Saharan Africa	5 to 9	90.2	92.7	95.6	96.8	86.4	89.5	92.5	94.8	94.7	95.8	90.5	93.5	91.5	92.1	96.4	96.5	91.4	92.0	86.6	85.8	98.1	98.2	59.6	54.5
Andean Latin America	10 to 14	70.6	80.9	70.9	80.8	72.8	82.6	81.8	87.9	78.6	85.8	83.6	89.0	65.9	73.7	80.1	82.8	48.4	63.1	81.0	91.4	79.6	89.6	79.2	89.7
Australasia	10 to 14	92.2	97.9	91.3	96.7	93.3	98.4	94.7	98.3	93.6	97.8	95.0	98.1	85.0	95.6	91.4	96.6	75.9	94.8	92.0	97.7	93.0	96.7	86.0	95.2
Caribbean	10 to 14	79.5	81.5	74.4	76.3	84.9	86.8	87.4	89.0	82.3	84.2	90.8	92.2	74.4	74.7	80.9	80.1	68.9	71.5	87.5	89.7	88.4	90.3	84.5	87.4
Central Asia	10 to 14	94.0	96.7	94.3	97.4	94.3	96.4	91.7	94.7	88.9	92.9	93.1	95.4	94.5	96.3	95.7	97.4	93.3	94.8	91.2	94.1	94.4	95.2	75.9	83.8
Central Europe	10 to 14	88.5	96.1	89.5	96.3	88.1	96.0	91.0	96.4	89.7	95.3	91.3	96.5	86.6	95.3	92.6	97.0	78.1	93.0	88.8	94.7	91.4	94.6	75.9	88.7
Central Latin America	10 to 14	86.5	90.8	86.2	92.4	87.5	90.0	91.7	95.6	90.1	94.8	92.1	95.4	82.6	84.7	88.1	92.0	76.2	74.0	84.8	94.3	88.2	95.9	71.8	85.8
Central Sub-Saharan Africa	10 to 14	80.1	88.8	73.8	86.2	85.9	91.3	79.3	86.1	66.7	79.0	86.2	90.1	86.3	91.2	89.6	93.4	82.8	88.5	89.4	93.2	89.6	93.2	84.5	88.2
East Asia	10 to 14	88.2	95.0	88.5	95.8	88.4	94.4	92.7	94.1	92.7	94.4	92.3	93.5	81.8	95.1	88.5	97.2	73.6	92.1	90.0	92.6	92.5	93.9	80.8	84.3
Eastern Europe	10 to 14	94.9	96.8	95.5	97.6	94.6	96.0	92.3	94.6	90.7	93.7	92.7	94.4	94.7	96.1	96.9	97.8	91.4	93.4	93.9	95.9	93.5	95.2	87.8	90.8
Eastern Sub-Saharan Africa	10 to 14	84.2	90.9	81.5	89.5	87.1	92.2	82.2	87.9	74.9	83.3	86.5	90.5	89.9	92.8	93.2	95.1	85.6	89.6	94.0	95.0	93.3	94.6	90.3	90.3
High-income Asia Pacific	10 to 14	88.6	96.4	88.3	95.7	89.4	96.9	92.5	97.0	91.4	96.2	92.7	97.0	86.5	94.4	92.3	96.0	78.3	92.8	86.5	95.4	88.6	95.1	77.0	91.5
High-income North America	10 to 14	95.8	97.0	93.5	95.0	97.1	97.8	98.1	99.5	98.2	99.8	97.6	98.7	88.7	91.1	92.5	94.7	84.1	86.4	92.7	95.2	94.5	96.5	91.2	94.6
North Africa and Middle East	10 to 14	64.8	86.5	63.3	87.1	68.9	87.3	65.5	85.1	60.9	84.2	68.8	85.5	72.9	88.8	81.8	92.2	62.2	84.8	63.9	80.1	62.7	81.0	60.9	71.8
Oceania	10 to 14	82.1	81.9	74.9	73.9	88.4	88.7	84.6	84.5	76.6	75.7	88.9	89.2	76.0	76.3	77.4	77.2	79.8	81.1	81.8	80.7	81.6	80.7	83.1	81.9
South Asia	10 to 14	84.0	88.8	83.5	88.9	85.7	89.5	84.6	87.8	80.6	84.8	86.9	89.3	81.8	86.7	88.8	91.2	73.2	81.3	91.1	92.8	90.3	92.6	86.0	86.4
Southeast Asia	10 to 14	80.8	85.4	80.2	87.8	82.8	84.5	84.9	87.1	81.0	86.5	87.1	87.0	78.4	84.1	86.2	91.2	69.0	74.7	87.3	88.8	88.9	90.9	78.0	77.4
Southern Latin America	10 to 14	84.6	94.7	85.0	94.7	85.4	94.8	91.5	95.6	90.5	94.9	91.6	95.5	74.1	91.6	85.1	95.0	59.4	86.9	88.0	95.9	88.4	95.3	81.5	92.6
Southern Sub-Saharan Africa	10 to 14	93.6	90.1	94.6	90.7	92.4	89.5	88.0	83.4	86.2	80.1	88.8	85.4	96.8	93.6	97.5	95.1	95.5	91.3	95.2	91.7	96.0	93.4	87.6	81.4
Tropical Latin America	10 to 14	84.3	86.8	84.8	87.2	85.1	87.2	89.1	91.0	87.2	89.5	89.8	91.4	79.5	80.7	86.7	87.8	70.3	71.6	88.3	95.5	92.1	96.0	74.9	89.9
Western Europe	10 to 14	88.4	97.6	87.5	96.8	89.8	98.0	90.5	97.4	88.8	96.8	91.2	97.3	81.3	95.6	86.9	96.5	75.4	95.0	86.8	97.5	89.3	96.9	76.7	95.1
Western Sub-Saharan Africa	10 to 14	87.0	88.9	82.7	85.5	90.0	91.3	88.7	90.1	80.2	81.7	92.7	94.0	91.5	91.2	93.4	93.0	89.6	89.3	89.2	87.7	93.2	93.1	75.4	69.2
Andean Latin America	15 to 19	28.1	53.3	47.2	64.7	21.7	52.8	39.5	59.4	49.0	64.2	41.4	62.8	32.5	50.0	46.5	55.5	21.6	48.2	47.6	71.1	61.9	77.7	44.0	72.3
Australasia	15 to 19	81.8	90.6	88.8	95.8	80.4	89.7	86.6	94.9	89.6	95.0	86.8	95.3	69.2	89.7	79.6	92.7	58.0	87.1	81.1	91.9	87.0	94.4	79.4	92.0
Caribbean	15 to 19	62.2	61.0	66.3	64.5	66.1	66.1	66.0	65.2	64.5	63.7	74.7	74.2	63.4	60.1	67.7	61.6	61.4	63.1	60.9	59.8	67.2	66.9	65.6	63.5
Central Asia	15 to 19	69.4	77.7	80.2	88.8	67.7	73.7	64.8	75.2	68.7	79.2	67.4	75.0	80.4	83.1	86.7	90.5	75.0	76.1	57.0	68.8	69.9	80.4	53.1	61.4
Central Europe	15 to 19	74.9	85.4	85.3	93.1	70.7	83.1	78.2	88.8	81.3	89.4	78.6	89.2	73.5	86.8	81.4	91.1	66.2	83.2	70.7	83.3	82.7	89.5	61.8	79.9
Central Latin America	15 to 19	71.5	77.4	79.2	86.0	70.8	74.5	77.6	85.0	77.9	85.2	80.9	86.7	68.4	71.2	74.9	80.2	63.3	62.1	64.6	80.0	74.9	87.3	60.1	75.1
Central Sub-Saharan Africa	15 to 19	60.2	68.5	69.0	75.7	60.3	69.2	52.8	59.7	51.2	57.2	62.7	69.2	74.6	79.3	81.2	84.1	70.1	76.0	75.6	78.6	80.4	83.0	77.2	80.3
East Asia	15 to 19	66.1	78.9	82.3	90.7	54.6	72.7	71.2	76.2	81.9	85.0	66.5	73.1	54.2	85.2	70.7	91.0	38.1	80.0	74.0	78.0	84.8	87.0	64.6	72.2
Eastern Europe	15 to 19	75.1	82.0	86.3	91.5	70.0	77.9	71.5	79.0	76.8	83.5	70.2	76.3	77.7	84.5	86.6	89.6	68.9	80.2	64.7	80.6	76.6	87.5	58.1	76.9

Eastern Sub-Saharan Africa	15 to 19	43.4	59.1	59.7	71.0	38.6	56.5	35.0	48.1	40.0	49.9	43.4	56.5	63.7	71.3	77.0	80.8	53.8	64.3	74.4	75.2	78.9	81.1	77.5	75.9
High-income Asia Pacific	15 to 19	78.5	90.2	87.7	95.2	74.4	88.6	83.2	92.8	86.3	93.1	82.5	92.6	74.1	89.3	82.6	91.9	65.0	87.1	73.9	90.0	82.5	92.5	70.0	90.0
High-income North America	15 to 19	97.1	99.8	96.6	99.3	97.0	98.7	96.6	99.8	96.4	99.4	96.2	98.8	84.8	91.5	87.2	93.3	83.4	89.6	95.3	99.3	95.5	98.8	93.9	97.7
North Africa and Middle East	15 to 19	60.1	71.6	72.3	82.0	56.7	69.1	51.7	64.6	59.1	70.9	54.1	65.9	82.2	90.7	87.8	93.2	75.4	88.1	41.7	49.8	52.8	62.2	46.0	49.3
Oceania	15 to 19	73.2	71.7	81.4	76.7	70.1	72.0	60.7	57.9	67.0	60.5	63.1	63.8	84.0	84.2	83.6	80.4	84.7	88.5	85.3	81.9	83.9	79.4	91.8	91.6
South Asia	15 to 19	66.9	74.6	73.2	81.3	70.7	76.6	64.9	71.5	63.9	71.2	73.4	77.8	69.6	77.4	74.1	79.8	67.6	77.1	74.9	79.5	76.6	82.7	83.4	84.1
Southeast Asia	15 to 19	58.9	61.4	68.4	77.2	58.8	53.7	59.1	59.3	60.9	68.5	65.5	58.7	66.2	70.9	72.8	80.5	61.5	62.1	63.7	63.4	72.1	76.6	64.5	55.6
Southern Latin America	15 to 19	62.0	79.9	73.3	89.4	59.9	76.6	74.3	83.9	76.7	86.3	76.9	84.2	40.4	73.8	56.8	83.4	25.4	64.9	70.9	86.5	79.3	91.7	69.0	83.9
Southern Sub-Saharan Africa	15 to 19	75.4	69.7	85.3	85.7	69.5	57.0	58.7	47.8	63.5	63.8	62.1	44.0	90.4	82.5	93.6	91.2	87.7	75.2	84.8	80.9	89.2	88.4	83.3	74.5
Tropical Latin America	15 to 19	60.7	67.8	71.9	78.7	58.0	63.7	64.5	71.0	69.0	76.5	66.0	70.1	62.2	64.8	68.8	71.7	57.9	59.7	62.1	76.0	75.6	83.9	53.5	72.1
Western Europe	15 to 19	77.3	91.5	85.8	95.7	74.5	90.6	78.8	92.6	83.0	93.3	79.0	92.7	67.2	88.6	74.5	91.0	62.0	87.9	73.2	94.2	83.5	95.8	67.2	93.5
Western Sub-Saharan Africa	15 to 19	75.6	79.7	76.6	82.1	78.9	81.6	72.2	77.0	61.9	67.5	84.8	88.3	89.8	90.3	88.6	89.2	91.5	91.8	79.0	78.5	86.9	87.6	75.3	73.4
Andean Latin America	20 to 24	37.6	60.2	53.0	72.0	57.2	72.2	49.7	67.2	47.4	66.9	63.9	75.6	39.7	56.2	49.5	63.0	57.1	69.5	53.6	75.8	68.2	84.1	35.1	64.6
Australasia	20 to 24	80.7	90.5	85.8	93.4	87.9	94.5	85.7	96.1	83.1	92.7	90.4	98.1	68.0	89.1	73.9	91.6	76.0	91.6	84.6	92.7	90.4	94.6	76.5	90.7
Caribbean	20 to 24	57.5	58.4	63.9	63.2	74.1	76.2	61.2	61.2	54.0	53.8	76.2	76.4	60.3	60.4	66.4	61.1	70.7	75.7	60.0	60.6	67.1	67.0	59.9	61.9
Central Asia	20 to 24	66.0	75.3	72.8	83.6	79.2	82.2	63.7	74.1	58.7	72.8	76.0	80.5	77.1	81.3	79.5	86.2	84.7	85.6	59.3	69.7	70.9	81.4	46.6	51.0
Central Europe	20 to 24	73.0	84.0	82.2	89.8	80.1	88.3	76.8	88.3	76.7	86.6	81.6	90.3	69.9	84.1	75.8	87.5	77.6	88.1	73.1	83.3	83.3	89.2	58.3	74.5
Central Latin America	20 to 24	71.7	77.9	78.0	84.8	81.1	83.6	77.4	85.0	73.0	82.0	84.7	88.9	67.8	70.4	72.5	78.0	76.6	75.9	70.0	83.1	79.8	89.1	55.0	72.8
Central Sub-Saharan Africa	20 to 24	67.6	71.5	73.4	77.2	80.5	82.7	63.1	65.6	52.1	55.1	78.9	80.7	78.0	80.1	82.0	83.8	84.9	86.3	83.7	84.4	87.1	88.0	80.9	80.8
East Asia	20 to 24	71.1	75.7	83.3	88.7	76.2	79.4	74.7	73.4	77.0	80.5	79.1	76.9	61.4	83.7	71.8	90.0	69.8	86.4	78.8	77.0	87.6	88.1	63.5	60.3
Eastern Europe	20 to 24	74.1	78.8	83.8	88.1	80.0	82.6	71.7	76.3	73.0	79.8	76.6	77.9	74.9	80.1	83.0	85.7	79.7	84.4	67.2	78.7	79.6	87.4	48.3	64.5
Eastern Sub-Saharan Africa	20 to 24	54.8	64.2	67.4	76.6	69.1	74.4	51.1	56.8	45.7	53.5	67.7	71.0	68.5	72.4	79.2	83.2	76.2	78.7	82.6	82.6	86.3	88.2	79.8	75.8
High-income Asia Pacific	20 to 24	80.3	90.6	86.6	94.1	86.2	93.3	85.8	94.3	83.1	91.6	90.2	95.9	71.4	87.4	79.1	89.9	77.0	90.5	78.6	90.8	84.4	93.4	72.4	87.4
High-income North America	20 to 24	96.6	99.6	96.1	99.7	98.9	99.8	95.8	98.2	91.7	95.3	98.2	99.0	79.0	88.0	81.7	90.7	84.9	90.0	97.2	99.4	97.8	99.9	94.5	96.8
North Africa and Middle East	20 to 24	64.8	73.5	72.3	80.7	77.3	82.2	58.7	67.5	55.7	66.6	71.2	76.4	80.0	88.7	83.9	90.7	84.7	91.5	57.0	61.4	64.5	71.6	54.9	51.2
Oceania	20 to 24	61.9	63.2	67.6	67.0	77.1	79.0	52.6	51.4	39.8	35.2	73.6	74.6	69.8	72.9	75.7	76.6	78.1	81.3	90.3	90.3	93.8	93.4	83.3	84.5
South Asia	20 to 24	64.1	71.7	72.3	80.1	76.8	80.4	64.7	70.8	60.3	68.1	76.3	79.4	62.6	70.3	71.3	77.3	72.5	78.1	78.3	81.6	82.0	86.5	77.2	76.6
Southeast Asia	20 to 24	59.1	63.3	67.5	76.7	73.7	72.2	60.8	63.2	55.3	65.0	74.7	71.7	65.6	70.4	70.7	78.7	75.5	76.8	67.1	68.8	75.9	80.6	57.9	51.7
Southern Latin America	20 to 24	63.1	80.9	72.0	86.4	75.9	87.4	76.3	86.5	73.1	83.7	83.8	90.8	43.7	74.3	52.4	78.3	59.8	81.9	77.9	89.8	83.1	92.2	72.9	86.9
Southern Sub-Saharan Africa	20 to 24	71.8	71.7	79.0	85.0	80.5	75.3	58.0	54.7	51.7	60.9	73.1	63.2	88.3	82.3	89.1	89.4	92.5	86.0	82.5	82.5	86.7	89.4	79.0	70.6
Tropical Latin America	20 to 24	54.8	67.6	66.4	76.3	68.6	77.4	61.7	71.2	60.7	70.8	70.9	77.5	52.4	64.1	58.8	68.2	65.9	75.3	64.7	77.8	76.4	84.8	49.0	68.6
Western Europe	20 to 24	75.6	91.4	83.6	94.4	82.9	94.5	78.7	93.5	79.0	91.2	84.0	95.6	61.6	86.8	68.3	89.0	72.8	90.8	77.0	95.5	84.6	96.6	67.5	93.3
Western Sub-Saharan Africa	20 to 24	77.2	79.1	82.9	85.2	85.2	86.0	73.3	75.7	64.4	66.8	85.3	86.9	87.4	86.2	91.4	90.8	90.1	89.0	83.4	80.9	92.8	92.5	70.7	64.7
Andean Latin America	25 to 29	44.4	65.5	56.7	76.4	50.4	66.2	42.8	63.1	54.3	73.1	49.3	63.7	47.0	61.0	59.6	72.2	59.1	68.7	57.7	80.5	69.2	87.5	58.4	77.7
Australasia	25 to 29	78.0	90.7	82.2	91.5	81.9	93.8	80.9	94.8	81.8	91.8	84.9	97.5	58.2	86.3	71.8	90.8	63.0	87.8	82.3	93.3	86.5	93.7	82.7	93.9
Caribbean	25 to 29	54.0	57.0	61.7	63.2	61.6	65.5	47.6	49.5	56.2	57.7	56.1	57.7	56.1	57.2	64.3	61.3	67.4	73.0	56.8	60.1	67.3	69.5	59.4	63.0
Central Asia	25 to 29	66.3	75.4	74.7	84.2	68.7	73.7	55.8	67.8	66.0	77.7	57.9	65.7	73.9	78.6	82.3	88.5	78.0	79.8	60.2	69.9	72.2	81.7	58.0	62.7
Central Europe	25 to 29	69.9	83.7	79.7	89.1	69.4	83.0	66.7	83.6	76.5	87.9	65.1	80.7	64.4	80.9	75.6	87.0	69.7	83.7	69.6	83.3	79.9	88.9	66.1	80.3
Central Latin America	25 to 29	70.2	78.6	76.6	85.1	72.7	77.6	68.5	79.4	73.6	82.8	71.0	79.0	65.0	69.4	73.6	80.2	71.5	71.6	67.7	83.2	76.0	88.7	68.4	80.6
Central Sub-Saharan Africa	25 to 29	73.1	75.9	77.5	81.8	78.3	78.8	62.0	63.5	64.1	67.7	71.7	70.9	81.1	81.9	85.6	87.9	86.1	85.9	85.2	86.4	86.6	88.9	89.0	87.7
East Asia	25 to 29	71.3	76.3	82.3	90.5	68.9	71.5	67.4	66.8	77.0	83.2	66.0	62.6	63.5	84.0	75.3	92.2	69.2	84.5	77.2	76.9	85.0	88.2	74.5	70.4
Eastern Europe	25 to 29	73.8	74.7	84.5	86.0	70.8	71.1	64.0	64.3	77.2	79.4	59.4	57.7	71.4	72.3	84.2	81.6	73.7	76.9	67.2	74.5	79.8	86.3	60.4	65.5
Eastern Sub-Saharan Africa	25 to 29	62.8	71.6	70.4	81.4	68.2	72.2	51.1	56.9	57.4	66.2	60.8	61.9	75.1	78.4	83.5	88.7	80.2	81.4	84.4	86.2	85.8	89.7	88.4	85.9
High-income Asia Pacific	25 to 29	76.3	90.4	84.4	94.7	76.0	89.3	79.0	92.3	82.9	93.3	79.6	90.7	58.7	81.9	74.3	88.0	62.6	84.5	74.6	91.2	81.5	94.0	74.8	89.9
High-income North America	25 to 29	94.3	99.3	93.4	99.3	97.3	99.5	89.9	93.6	88.7	92.6	92.2	94.1	70.4	85.6	75.8	90.5	77.7	85.9	96.8	98.6	96.7	98.6	97.7	98.6
North Africa and Middle East	25 to 29	64.4	76.1	72.2	82.5	68.5	77.9	48.3	61.7	59.7	70.6	53.0	64.4	78.1	88.7	86.3	92.9	79.8	89.5	50.4	59.2	57.7	69.9	61.3	61.1
Oceania	25 to 29	42.1	46.0	52.9	57.3	50.8	52.7	22.4	21.8	33.2	33.8	38.5	36.7	48.4	54.9	67.8	72.8	55.5	60.9	76.6	78.6	75.4	77.9	88.7	89.1
South Asia	25 to 29	62.9	72.6	71.1	80.4	67.4	74.1	55.7	65.1	64.1	72.2	60.8	68.0	61.5	70.8	74.2	80.2	68.6	76.4	76.2	81.7	78.7	85.6	81.6	82.5

Southeast Asia	25 to 29	56.0	63.1	66.6	76.7	59.5	61.4	48.6	54.6	59.1	68.4	54.0	54.4	64.2	70.0	74.4	81.2	70.4	73.7	63.5	66.7	74.0	79.8	62.3	60.4
Southern Latin America	25 to 29	63.4	82.1	72.0	86.9	67.2	83.3	71.1	84.3	76.0	86.6	74.0	84.7	46.3	74.1	61.2	80.9	55.8	79.2	76.1	90.2	81.2	92.7	78.2	89.9
Southern Sub-Saharan Africa	25 to 29	58.4	71.4	67.7	83.4	62.0	67.2	30.6	45.2	41.8	62.2	43.2	45.3	81.6	81.5	83.6	89.9	87.8	83.6	72.1	81.3	79.3	89.0	71.9	75.3
Tropical Latin America	25 to 29	41.8	67.3	56.1	76.1	45.9	68.7	41.1	65.0	56.8	74.5	41.8	63.7	36.1	58.4	49.9	67.9	50.5	67.3	55.5	76.4	69.6	83.5	53.4	75.3
Western Europe	25 to 29	75.0	91.6	81.7	93.5	77.2	92.9	73.4	91.5	79.4	91.4	75.2	92.5	60.3	84.5	68.9	88.0	70.4	88.5	75.2	95.7	82.7	96.4	74.5	95.6
Western Sub-Saharan Africa	25 to 29	78.1	82.0	82.4	87.4	81.8	83.2	66.7	71.6	68.2	73.5	75.7	78.2	87.1	87.3	92.4	93.8	88.5	87.6	83.5	82.6	90.6	92.1	80.1	76.3
Andean Latin America	30 to 34	49.0	67.4	57.4	75.3	51.8	66.7	45.0	61.4	57.4	73.7	45.4	58.0	58.0	67.9	60.3	71.0	57.8	67.0	58.8	80.6	67.4	87.1	62.3	79.2
Australasia	30 to 34	77.5	89.5	80.3	89.6	80.1	91.8	80.0	92.1	82.6	91.6	81.1	93.1	62.4	83.0	67.6	86.0	58.2	80.8	83.6	93.7	88.2	95.1	84.0	94.4
Caribbean	30 to 34	45.5	53.4	52.6	59.4	50.2	57.3	38.1	44.1	50.4	55.8	40.0	44.5	56.4	61.9	57.7	60.7	56.8	65.7	50.3	58.5	62.0	68.7	51.3	58.9
Central Asia	30 to 34	64.8	72.9	72.8	82.2	64.3	68.9	54.7	64.4	66.6	77.0	52.5	59.1	74.1	78.2	80.3	86.6	70.6	73.2	61.4	69.8	72.4	82.4	60.6	62.6
Central Europe	30 to 34	62.2	80.1	73.1	87.1	59.6	76.4	56.4	74.2	70.7	85.3	51.3	67.1	60.7	78.6	66.1	83.4	57.3	75.6	63.3	81.1	77.9	89.5	56.6	75.9
Central Latin America	30 to 34	67.7	75.8	72.9	82.3	68.4	72.2	63.5	72.4	70.9	80.7	62.6	67.0	68.2	71.6	71.4	78.0	65.7	65.1	70.1	82.7	78.5	89.9	68.9	78.3
Central Sub-Saharan Africa	30 to 34	63.8	67.1	68.8	74.4	67.6	67.7	53.0	53.3	58.8	62.3	59.0	56.2	78.1	78.8	79.5	82.8	78.9	78.0	80.8	82.5	84.2	87.6	84.2	82.6
East Asia	30 to 34	60.9	68.7	74.3	86.6	56.1	61.2	58.5	59.3	72.4	79.7	53.4	52.1	59.4	82.2	67.6	91.0	54.8	76.5	70.1	71.2	81.5	87.3	64.6	61.5
Eastern Europe	30 to 34	68.8	65.4	81.1	80.4	63.5	58.7	57.8	51.6	74.1	72.8	50.5	41.6	69.3	65.2	81.2	75.0	62.4	60.4	64.6	65.8	81.6	83.6	53.7	53.3
Eastern Sub-Saharan Africa	30 to 34	50.1	59.7	58.3	71.2	53.9	58.4	39.2	41.4	49.7	56.1	44.2	42.5	70.0	73.8	74.4	81.4	69.3	71.1	79.4	81.3	82.3	87.0	84.3	81.1
High-income Asia Pacific	30 to 34	68.0	82.2	78.9	89.8	65.5	79.8	73.2	86.7	82.1	94.0	69.2	81.5	50.3	73.7	63.8	79.6	41.1	70.9	73.7	88.1	81.1	93.0	73.3	86.0
High-income North America	30 to 34	89.5	96.2	89.7	96.6	91.3	96.0	83.0	88.4	85.9	90.3	82.9	87.8	65.7	83.2	69.4	88.0	62.5	78.3	95.4	97.0	96.0	97.0	96.1	97.9
North Africa and Middle East	30 to 34	62.3	75.7	68.0	79.4	65.3	77.6	48.4	61.5	59.1	69.0	50.4	63.5	74.0	87.1	78.8	88.9	70.6	86.5	49.6	59.8	55.2	67.4	59.7	63.9
Oceania	30 to 34	45.9	48.1	53.0	56.8	51.7	51.6	26.5	23.7	38.0	37.6	33.9	29.4	69.3	73.0	68.3	73.0	72.5	75.3	79.7	80.9	83.9	85.5	81.2	81.3
South Asia	30 to 34	53.7	64.0	66.6	75.5	52.0	61.3	46.9	55.8	63.4	70.0	41.5	51.3	57.4	65.9	69.7	74.8	50.6	61.7	72.4	77.8	79.1	85.6	73.6	75.4
Southeast Asia	30 to 34	47.7	54.9	59.6	70.1	47.3	50.3	40.8	45.5	55.2	63.4	40.3	40.9	64.7	70.2	71.9	78.5	59.5	64.7	63.5	65.7	74.7	79.8	60.8	58.9
Southern Latin America	30 to 34	59.3	80.3	65.8	83.3	62.4	81.5	66.9	83.1	74.1	86.6	66.8	82.1	50.1	76.2	54.2	77.7	47.6	76.0	74.9	89.0	79.2	92.0	78.6	89.4
Southern Sub-Saharan Africa	30 to 34	47.7	63.7	59.6	75.0	45.9	59.7	23.0	36.4	39.4	52.7	25.9	36.4	75.4	78.8	73.8	82.0	78.5	77.8	62.3	74.8	72.8	83.1	59.9	71.6
Tropical Latin America	30 to 34	28.6	61.2	43.3	68.4	29.2	62.3	28.7	56.4	49.7	68.9	22.1	52.3	31.0	57.6	34.7	59.5	28.1	57.2	47.5	74.3	66.2	80.8	41.3	74.9
Western Europe	30 to 34	72.2	88.8	77.5	90.2	73.6	89.8	70.2	89.9	77.4	91.0	70.1	89.9	63.8	83.9	65.0	84.4	65.0	84.7	74.9	93.5	83.2	96.1	73.4	92.6
Western Sub-Saharan Africa	30 to 34	69.3	75.1	71.6	79.9	74.6	76.2	56.1	61.7	60.0	67.6	63.7	65.5	82.5	84.0	86.3	90.0	80.9	80.1	79.5	78.9	87.3	90.3	78.0	72.5
Andean Latin America	35 to 39	52.5	70.8	58.4	76.1	46.1	64.9	52.4	67.2	62.8	77.1	52.9	64.7	63.5	72.8	64.7	73.9	63.3	72.4	58.4	81.6	65.8	87.3	60.7	79.7
Australasia	35 to 39	78.3	88.8	80.9	89.5	76.2	88.3	83.9	91.2	87.6	92.8	83.1	90.5	61.5	78.8	62.7	81.9	61.4	75.8	85.5	95.3	90.5	97.0	83.3	94.6
Caribbean	35 to 39	43.8	50.6	49.2	56.1	37.7	44.3	43.4	47.0	54.6	59.1	45.1	46.0	54.6	59.7	55.8	58.3	53.9	62.7	50.7	58.5	62.7	68.7	47.5	55.6
Central Asia	35 to 39	63.8	71.2	72.1	81.5	54.7	60.0	59.2	67.2	69.6	78.8	57.8	62.9	72.7	76.4	80.4	85.4	67.8	71.0	58.4	66.5	71.9	81.3	51.9	55.5
Central Europe	35 to 39	57.7	78.5	70.4	88.6	44.3	66.5	57.1	73.2	72.5	87.7	51.4	64.8	57.0	76.6	62.2	83.0	53.1	71.3	55.3	77.6	75.4	89.0	42.5	68.5
Central Latin America	35 to 39	65.7	73.5	70.1	80.2	59.5	63.2	64.4	71.0	72.7	81.6	62.7	64.2	69.0	71.7	70.2	76.3	68.2	66.3	65.6	79.8	74.7	87.8	62.3	73.8
Central Sub-Saharan Africa	35 to 39	60.8	64.8	66.6	72.2	55.7	57.7	57.3	57.8	64.4	66.9	60.4	58.9	76.9	78.4	78.4	81.8	77.0	77.2	78.8	81.3	84.0	88.1	78.7	78.2
East Asia	35 to 39	50.2	63.8	64.1	81.9	35.0	48.2	56.6	60.8	71.3	79.5	51.3	54.8	52.4	80.1	57.7	87.9	49.7	74.3	50.7	62.5	65.5	81.1	44.7	51.7
Eastern Europe	35 to 39	65.3	62.0	78.3	78.2	52.1	46.2	59.6	54.3	74.8	74.0	53.8	46.4	66.7	61.9	78.1	73.2	60.2	56.0	56.5	59.1	77.5	79.9	42.0	44.2
Eastern Sub-Saharan Africa	35 to 39	52.6	62.5	59.9	71.2	45.7	54.4	51.0	53.5	60.9	64.5	52.7	54.0	72.1	77.3	75.8	81.9	71.1	75.5	80.5	82.5	84.1	88.6	82.3	80.3
High-income Asia Pacific	35 to 39	65.0	78.3	76.6	86.9	54.8	71.6	72.9	84.6	84.1	95.1	67.6	79.1	49.9	71.2	61.8	77.8	42.7	68.0	75.9	89.7	82.9	94.2	73.9	86.7
High-income North America	35 to 39	86.4	94.7	88.1	95.4	84.1	93.0	80.4	87.1	86.8	91.4	77.5	84.9	66.1	84.3	69.6	87.9	61.5	78.9	92.2	95.0	94.2	95.2	91.9	96.1
North Africa and Middle East	35 to 39	58.8	74.2	63.1	77.0	54.6	71.7	52.4	65.0	62.7	72.1	53.5	66.3	71.0	85.4	75.4	86.6	67.3	84.8	33.9	51.2	35.9	57.7	47.8	56.1
Oceania	35 to 39	35.7	40.0	46.0	51.5	25.9	28.6	30.6	29.5	42.3	43.2	35.6	33.0	56.2	63.1	61.1	67.3	53.9	61.0	64.0	67.5	80.9	83.4	53.8	56.6
South Asia	35 to 39	55.4	64.3	63.9	72.5	47.7	56.8	55.6	62.7	67.4	72.7	53.6	61.2	61.0	67.3	67.8	72.0	58.1	65.8	71.4	77.6	77.5	84.7	71.8	74.9
Southeast Asia	35 to 39	41.6	49.6	52.7	64.2	29.6	34.6	43.2	47.3	57.2	64.0	42.5	43.7	60.9	67.8	67.3	74.6	56.3	63.1	56.2	59.7	67.2	74.4	53.6	52.1
Southern Latin America	35 to 39	54.6	78.1	60.5	81.7	49.1	74.4	65.4	82.7	74.4	88.3	64.1	80.0	48.2	75.9	49.2	77.2	49.1	75.7	74.3	89.7	79.1	93.3	75.6	88.5
Southern Sub-Saharan Africa	35 to 39	52.1	62.3	63.3	72.0	38.1	51.2	39.7	42.2	53.9	53.9	39.8	44.6	75.5	77.0	75.3	78.7	76.7	76.3	67.1	75.0	77.5	82.3	60.5	71.6
Tropical Latin America	35 to 39	23.4	55.9	36.2	61.9	9.1	49.6	32.9	56.0	52.8	68.4	26.8	52.8	26.0	51.4	29.7	51.7	22.4	52.7	35.1	68.5	55.1	76.5	27.6	67.0
Western Europe	35 to 39	71.4	87.2	76.1	89.2	67.0	85.3	72.4	90.7	79.3	92.8	72.0	89.7	64.8	83.6	65.8	84.6	65.4	83.3	73.6	92.7	82.9	96.3	69.8	90.3
Western Sub-Saharan Africa	35 to 39	65.3	72.4	67.5	76.8	64.4	68.4	58.5	64.3	63.7	71.0	63.4	66.2	79.9	83.0	82.5	87.3	78.6	79.7	74.8	75.4	86.4	90.3	70.1	64.9

Andean Latin America	40 to 44	56.0	72.7	59.9	75.4	51.7	69.7	58.9	70.4	68.9	78.5	52.4	64.3	65.4	72.8	63.3	70.8	68.2	75.5	65.8	82.6	75.3	88.3	57.6	77.6
Australasia	40 to 44	79.2	88.5	81.8	89.3	77.1	87.9	83.8	90.6	89.5	94.8	78.8	85.8	64.0	77.9	64.8	78.6	63.9	77.7	84.6	93.6	89.8	95.6	82.3	93.6
Caribbean	40 to 44	44.3	49.4	48.1	54.4	39.7	43.3	48.6	49.3	60.9	63.2	40.0	37.8	58.0	61.0	56.0	58.3	59.6	63.9	50.1	56.8	60.1	66.9	44.3	49.8
Central Asia	40 to 44	58.9	69.6	67.6	79.5	50.6	60.0	59.0	69.1	70.8	80.6	50.5	59.7	69.3	74.2	73.9	81.2	67.4	71.1	53.9	66.0	70.9	80.7	37.6	50.9
Central Europe	40 to 44	56.0	76.2	66.3	87.0	46.7	65.8	58.9	72.3	72.5	87.1	48.6	59.5	58.8	76.4	59.7	79.8	58.5	74.2	52.7	74.8	73.1	87.9	33.7	61.4
Central Latin America	40 to 44	67.3	74.3	70.5	79.6	62.4	66.0	67.3	71.6	75.5	82.2	60.0	59.8	71.9	72.6	71.0	74.0	73.0	70.8	69.6	81.7	78.0	87.7	61.8	74.8
Central Sub-Saharan Africa	40 to 44	54.5	59.0	61.2	66.8	48.1	51.7	55.5	55.0	65.7	67.0	49.5	47.5	72.8	75.0	73.8	76.6	74.0	75.9	74.9	77.9	81.1	84.6	72.3	74.0
East Asia	40 to 44	39.1	60.3	51.4	75.9	27.4	48.5	53.5	61.2	68.5	77.6	42.1	50.4	48.1	78.2	49.1	83.7	48.7	75.2	37.1	57.4	61.3	78.9	10.5	37.4
Eastern Europe	40 to 44	61.8	61.7	72.5	76.4	52.2	48.8	60.3	57.1	73.6	75.2	50.4	43.4	65.1	62.9	71.0	70.0	62.8	60.1	49.5	56.3	72.7	79.8	26.3	31.9
Eastern Sub-Saharan Africa	40 to 44	47.2	58.6	57.3	68.7	37.7	49.6	50.6	52.9	65.0	67.7	40.4	43.0	69.3	75.2	73.6	79.0	68.8	74.7	78.3	80.8	82.6	86.9	78.3	77.1
High-income Asia Pacific	40 to 44	64.6	78.6	72.8	85.5	58.8	74.3	73.4	84.4	84.4	93.7	64.1	77.3	53.6	72.4	56.1	75.4	53.8	72.3	76.4	88.4	83.7	93.4	74.0	87.4
High-income North America	40 to 44	86.4	96.4	87.3	96.5	85.1	95.1	81.5	88.5	87.3	92.3	76.1	84.4	68.5	83.2	69.0	85.3	66.9	79.6	92.7	97.0	94.5	96.8	91.3	96.6
North Africa and Middle East	40 to 44	59.1	72.8	61.1	73.5	57.6	72.4	57.3	66.2	67.1	72.7	51.7	63.3	70.1	83.5	72.1	82.9	68.8	84.7	40.4	52.8	45.3	59.0	43.0	52.1
Oceania	40 to 44	28.6	35.3	34.1	41.3	24.4	30.4	30.7	32.2	42.2	43.6	26.6	27.9	56.8	64.3	60.9	67.6	55.3	63.3	73.6	76.6	70.7	75.0	83.1	84.1
South Asia	40 to 44	54.1	63.1	60.4	69.8	49.3	57.8	58.9	64.7	70.5	74.9	50.5	57.3	61.6	67.3	64.9	69.3	61.6	68.0	71.2	76.9	75.9	82.9	71.0	74.2
Southeast Asia	40 to 44	41.5	48.6	50.7	60.7	32.3	37.6	48.7	51.0	62.5	66.2	39.4	40.6	62.5	69.1	66.5	72.9	59.9	67.1	58.3	60.6	70.4	75.3	48.1	47.0
Southern Latin America	40 to 44	54.0	77.9	60.1	81.4	48.6	74.6	66.0	82.3	76.5	88.4	57.8	76.3	51.5	74.9	52.3	75.1	50.7	75.7	74.2	88.4	80.3	91.7	73.0	88.2
Southern Sub-Saharan Africa	40 to 44	59.3	62.4	67.4	70.4	50.2	53.9	53.1	45.7	64.4	57.3	46.3	40.0	76.8	75.9	76.1	75.7	78.9	77.5	75.3	78.0	83.9	85.0	66.4	71.7
Tropical Latin America	40 to 44	26.3	55.5	34.6	58.9	17.9	52.3	40.8	58.7	58.4	70.9	27.0	48.7	30.2	50.8	30.3	46.8	27.7	55.4	39.6	70.1	58.1	78.2	25.7	65.3
Western Europe	40 to 44	73.9	87.7	77.9	89.8	70.6	85.6	75.8	90.9	82.8	94.3	70.6	86.8	69.4	83.2	68.0	82.4	71.8	85.0	76.6	91.1	85.9	95.0	70.2	89.4
Western Sub-Saharan Africa	40 to 44	58.3	67.0	61.6	72.8	56.4	61.6	55.9	61.5	64.1	71.1	52.5	55.6	74.5	78.7	76.2	82.1	74.9	77.2	71.1	72.5	83.7	87.7	60.1	54.3
Andean Latin America	45 to 49	55.9	74.4	62.4	76.4	53.7	73.7	52.5	65.4	65.8	75.2	47.2	60.9	64.9	71.5	67.4	72.1	63.5	72.8	66.4	82.7	71.4	85.9	67.0	82.1
Australasia	45 to 49	79.6	89.0	80.7	88.2	79.6	89.2	80.7	89.2	86.3	92.7	77.8	86.0	58.6	72.5	63.3	77.1	53.4	67.0	84.0	92.7	87.4	93.5	85.9	95.0
Caribbean	45 to 49	42.9	52.3	52.5	61.3	39.3	47.2	43.0	45.9	59.6	63.4	35.4	35.9	55.8	61.5	60.8	65.0	49.0	57.5	55.2	62.5	62.1	69.1	56.0	62.8
Central Asia	45 to 49	60.7	68.9	69.4	78.1	55.8	62.0	60.6	66.6	71.7	77.9	56.0	60.3	70.7	72.2	77.1	80.6	64.6	64.8	54.4	63.5	68.3	76.5	49.0	57.4
Central Europe	45 to 49	55.9	73.6	67.7	84.0	49.4	65.3	55.9	66.5	70.6	83.0	48.4	55.0	58.3	71.7	63.9	77.2	51.5	65.7	48.2	68.6	67.7	82.1	40.7	63.0
Central Latin America	45 to 49	67.2	74.9	72.1	80.2	63.3	67.9	63.2	67.1	73.6	79.3	57.4	56.9	71.2	70.9	74.1	75.6	68.3	64.6	67.8	80.4	75.4	86.2	63.7	75.6
Central Sub-Saharan Africa	45 to 49	51.9	57.1	60.7	66.0	48.2	52.5	50.4	49.4	62.5	63.1	47.6	44.8	70.4	73.0	74.5	76.9	66.8	69.8	72.4	75.5	77.1	80.6	73.7	75.9
East Asia	45 to 49	39.9	64.4	54.9	76.4	32.4	57.2	53.6	63.5	69.3	77.4	45.0	56.2	44.6	75.5	53.2	82.7	35.3	68.3	38.8	63.4	57.0	77.4	30.0	58.7
Eastern Europe	45 to 49	61.6	65.2	71.3	77.8	55.4	56.2	59.2	57.6	71.6	74.5	53.4	47.6	64.1	62.9	72.2	71.8	56.2	54.4	45.2	57.7	64.3	77.0	36.2	47.8
Eastern Sub-Saharan Africa	45 to 49	46.1	57.6	59.6	68.6	38.4	51.1	47.2	48.1	64.5	64.7	38.4	40.4	68.3	73.6	76.7	79.7	61.3	68.8	76.9	79.0	80.1	83.8	79.4	79.0
High-income Asia Pacific	45 to 49	67.4	81.0	74.0	85.7	65.1	78.7	72.6	83.5	83.3	91.8	65.5	78.5	53.6	72.7	60.4	78.2	46.9	67.7	77.6	88.4	82.7	92.7	81.1	91.0
High-income North America	45 to 49	86.2	98.5	86.3	96.1	85.4	97.6	78.2	86.8	84.9	91.0	73.8	83.1	63.5	77.9	68.0	82.4	57.3	70.5	90.9	98.9	92.9	97.8	91.0	98.4
North Africa and Middle East	45 to 49	56.0	71.1	60.9	73.2	56.3	71.7	53.1	62.5	65.6	70.7	48.9	61.3	65.5	80.0	72.1	82.6	57.5	77.2	36.2	52.4	38.8	56.3	45.3	57.8
Oceania	45 to 49	26.2	34.6	34.9	42.7	28.2	35.7	29.2	32.1	40.7	42.7	31.3	34.2	51.0	60.0	61.8	68.9	40.3	51.4	68.3	72.9	67.3	72.8	76.0	78.9
South Asia	45 to 49	52.5	63.4	58.7	68.1	52.5	63.0	56.6	63.9	68.3	72.7	52.4	61.4	59.4	66.6	65.7	70.0	53.7	63.8	66.5	74.5	68.4	77.6	72.3	77.5
Southeast Asia	45 to 49	42.5	49.2	54.8	62.5	36.6	42.0	47.2	48.2	62.9	64.3	40.4	40.9	62.1	68.4	70.3	75.4	52.2	60.9	59.9	61.3	69.9	73.9	56.6	56.4
Southern Latin America	45 to 49	57.8	79.2	66.3	82.7	54.2	76.4	64.6	79.4	76.8	86.7	57.2	74.0	53.7	73.3	61.1	77.5	43.7	68.5	74.0	88.3	80.8	91.3	75.9	90.0
Southern Sub-Saharan Africa	45 to 49	64.2	61.0	71.6	69.3	58.7	55.3	55.0	38.0	65.9	51.3	52.5	36.8	77.3	73.9	80.7	77.0	74.5	71.5	77.5	76.2	84.6	82.8	72.7	72.4
Tropical Latin America	45 to 49	31.6	55.9	46.3	62.6	25.3	53.7	38.8	51.8	59.0	67.4	27.6	43.1	43.0	52.1	50.6	54.9	31.8	50.0	27.6	63.5	52.8	74.0	17.5	60.8
Western Europe	45 to 49	75.9	88.8	80.1	89.9	73.5	87.0	73.4	88.5	82.1	93.1	68.5	84.2	68.5	79.4	71.4	81.8	66.0	77.7	76.9	90.5	84.8	93.5	76.6	91.7
Western Sub-Saharan Africa	45 to 49	55.4	65.3	64.8	74.6	51.7	58.2	52.9	59.1	65.0	71.4	49.2	53.5	70.9	76.3	78.3	83.1	64.9	69.7	66.0	68.2	82.4	86.3	54.9	51.5
Andean Latin America	50 to 54	57.3	74.4	65.9	78.6	52.4	72.7	63.0	72.7	68.2	76.2	55.5	66.9	65.6	71.9	64.5	69.2	68.1	76.5	65.5	80.5	72.0	84.2	64.3	80.3
Australasia	50 to 54	75.9	88.3	79.3	88.8	77.2	90.7	80.7	92.0	81.5	91.8	78.2	90.1	59.4	77.4	57.4	76.6	63.1	79.1	81.6	91.3	84.6	90.3	88.0	97.6
Caribbean	50 to 54	43.0	55.4	52.8	65.2	39.4	49.9	54.9	58.6	60.3	65.2	47.5	49.5	57.6	65.4	55.6	63.9	60.4	67.7	50.6	61.1	58.5	69.4	51.2	59.8
Central Asia	50 to 54	59.4	67.2	68.1	76.8	56.0	62.1	67.6	72.0	70.7	76.3	62.8	65.9	69.4	70.2	70.3	74.1	70.3	69.2	53.8	63.0	64.8	74.0	51.9	60.0
Central Europe	50 to 54	55.5	69.9	67.3	81.8	50.3	63.7	64.3	70.9	69.9	80.0	57.0	60.9	58.5	69.2	59.4	71.1	58.4	68.4	47.9	63.8	64.7	77.7	44.0	61.6
Central Latin America	50 to 54	67.7	75.3	73.9	82.3	63.7	68.5	71.6	74.8	75.1	80.9	65.0	64.4	73.3	72.5	72.9	73.8	74.1	71.1	68.8	80.3	74.9	85.4	67.0	77.7

Central Sub-Saharan Africa	50 to 54	51.2	56.6	61.4	66.6	46.0	51.4	59.7	58.7	63.2	63.2	54.9	52.7	71.1	74.2	71.8	75.2	72.4	75.5	70.6	74.5	74.5	78.0	72.3	76.3
East Asia	50 to 54	31.1	61.8	46.6	72.9	24.2	56.7	59.2	70.2	65.5	75.4	51.1	63.6	28.0	73.8	28.1	76.9	30.5	72.5	25.4	59.4	46.0	71.2	18.0	58.4
Eastern Europe	50 to 54	60.5	66.6	69.8	78.2	56.3	60.1	68.0	68.6	71.1	74.9	63.2	61.0	64.4	65.2	67.5	69.6	63.6	63.6	42.5	56.5	59.1	73.5	36.3	49.7
Eastern Sub-Saharan Africa	50 to 54	44.9	57.5	58.0	68.2	37.6	51.8	56.8	58.1	63.1	63.9	48.7	50.6	69.1	75.2	73.3	77.5	68.4	75.7	74.8	77.7	76.3	80.5	79.8	80.2
High-income Asia Pacific	50 to 54	69.5	83.6	75.3	88.0	69.5	83.8	80.2	89.8	83.9	92.9	74.6	86.1	60.4	78.5	61.4	80.2	61.7	78.8	76.0	88.6	79.3	91.3	83.1	94.6
High-income North America	50 to 54	83.9	99.2	86.0	99.2	84.6	99.3	83.7	91.9	85.6	92.8	79.4	88.3	65.0	78.3	64.7	79.9	65.0	75.1	87.4	99.2	88.4	99.1	91.7	99.7
North Africa and Middle East	50 to 54	53.4	68.8	59.6	72.4	53.7	70.1	62.2	69.9	66.1	71.0	56.7	67.3	63.9	80.2	66.3	79.7	62.2	81.1	29.4	48.2	34.8	53.4	38.5	54.5
Oceania	50 to 54	25.8	34.2	35.2	42.7	26.0	34.4	45.2	47.2	45.6	46.7	42.4	44.9	50.8	60.9	47.6	58.0	55.6	65.2	64.9	69.7	62.0	67.6	77.6	80.6
South Asia	50 to 54	47.2	59.9	57.7	67.6	44.4	58.3	63.6	70.4	69.8	74.1	55.5	64.9	54.0	64.7	60.0	66.3	52.9	65.9	59.7	69.3	64.2	74.6	65.8	72.6
Southeast Asia	50 to 54	43.1	50.4	54.9	63.7	37.8	43.5	59.1	59.9	64.0	65.8	52.6	52.4	61.2	69.6	64.6	72.3	59.0	68.3	56.9	59.6	66.7	71.8	55.1	55.9
Southern Latin America	50 to 54	60.4	79.0	69.7	84.5	56.9	76.9	72.7	83.8	78.7	88.0	64.5	77.0	56.7	75.0	60.6	76.4	52.4	74.2	74.5	87.1	79.4	88.9	79.6	92.0
Southern Sub-Saharan Africa	50 to 54	66.4	56.9	73.9	67.6	61.7	49.2	66.2	45.2	68.5	48.9	62.5	40.3	78.4	73.2	79.8	73.8	78.7	74.2	79.7	74.0	83.7	79.9	78.1	70.4
Tropical Latin America	50 to 54	37.7	59.0	51.9	67.7	30.8	55.1	55.0	62.9	63.6	70.6	43.6	52.5	41.9	51.6	44.0	50.8	38.3	52.7	35.7	66.5	55.6	76.4	29.4	64.8
Western Europe	50 to 54	75.9	86.6	81.2	90.4	74.9	86.1	79.9	90.2	83.2	92.7	74.6	85.5	72.1	81.7	71.3	81.1	74.4	83.7	76.9	88.1	82.3	90.6	80.6	93.0
Western Sub-Saharan Africa	50 to 54	47.6	59.4	57.7	69.1	44.7	53.8	57.2	63.2	58.1	65.3	54.6	59.8	66.7	74.1	68.6	75.7	68.1	74.4	57.6	62.1	75.8	80.2	45.0	43.1
Andean Latin America	55 to 59	60.1	75.6	66.4	78.4	55.6	74.0	65.3	74.8	69.2	76.6	56.9	68.9	66.5	72.8	66.1	69.4	67.7	76.9	66.7	79.8	73.9	83.5	64.9	80.1
Australasia	55 to 59	75.9	88.6	78.6	88.4	76.4	90.2	81.1	93.8	80.4	91.5	78.8	93.1	64.4	81.7	65.4	81.4	64.4	82.4	76.4	88.6	80.2	87.7	82.9	94.7
Caribbean	55 to 59	45.1	57.4	52.5	65.2	41.3	52.0	56.4	60.3	61.9	66.3	46.6	49.6	60.5	68.1	61.3	68.8	59.6	67.4	45.8	56.6	54.4	66.1	47.2	55.0
Central Asia	55 to 59	58.5	66.1	65.2	73.2	55.0	61.7	65.9	70.2	68.5	72.9	59.9	64.0	66.2	65.4	69.2	70.3	65.9	63.9	52.2	59.7	63.8	70.5	49.6	57.3
Central Europe	55 to 59	54.7	69.6	62.8	78.5	51.3	65.2	61.2	70.1	66.3	77.3	52.3	59.5	52.9	66.6	55.1	68.7	52.1	65.8	42.2	59.0	57.7	72.4	41.2	58.5
Central Latin America	55 to 59	68.7	76.8	73.4	82.2	64.3	70.6	72.9	77.3	76.1	81.9	64.1	66.3	75.6	74.1	75.5	75.2	75.2	72.0	66.1	79.0	72.6	84.1	64.7	76.8
Central Sub-Saharan Africa	55 to 59	49.6	55.1	57.4	62.9	44.6	49.5	56.7	55.8	59.4	59.7	50.8	48.4	71.8	74.9	72.5	75.6	73.7	76.9	66.4	70.3	71.2	74.8	67.3	70.4
East Asia	55 to 59	28.8	62.8	40.3	70.3	23.5	58.9	57.8	71.9	63.6	75.5	47.9	64.8	15.3	74.0	20.8	76.8	14.2	73.1	20.7	56.0	39.1	66.6	17.9	56.2
Eastern Europe	55 to 59	58.9	67.1	65.4	76.0	55.4	61.0	66.3	69.9	68.5	74.6	60.9	61.5	58.0	62.9	62.8	68.1	56.3	60.4	33.4	49.0	50.4	67.3	28.2	41.4
Eastern Sub-Saharan Africa	55 to 59	42.7	56.3	52.5	64.3	36.9	51.3	53.3	55.8	58.2	60.3	44.6	47.5	69.2	76.1	73.5	77.9	68.9	77.4	70.5	73.9	72.4	77.2	75.4	75.6
High-income Asia Pacific	55 to 59	73.4	87.7	76.1	89.8	73.9	88.2	84.9	94.7	85.7	93.8	80.5	93.4	67.4	85.2	69.0	85.5	69.4	87.6	72.7	87.7	76.0	90.5	79.0	92.6
High-income North America	55 to 59	82.8	99.5	83.6	99.6	83.5	99.5	84.6	94.2	84.9	93.3	80.3	91.1	65.3	78.9	66.0	80.5	63.6	73.7	83.7	99.4	84.4	99.6	88.5	99.8
North Africa and Middle East	55 to 59	52.3	67.5	56.7	69.5	52.1	68.4	59.9	68.6	64.1	69.3	51.9	64.8	60.6	79.4	67.4	79.8	54.4	78.8	27.0	44.2	35.4	51.0	34.1	49.7
Oceania	55 to 59	27.0	36.1	33.0	40.6	27.3	37.0	47.4	50.2	47.2	49.0	42.6	46.6	55.1	65.4	55.2	63.3	57.5	69.5	63.7	67.9	66.0	69.9	70.9	74.6
South Asia	55 to 59	46.1	53.9	49.0	55.6	47.9	56.4	65.2	68.9	67.9	69.6	59.1	65.2	48.3	57.2	53.3	55.2	48.9	62.4	53.4	58.1	54.2	61.4	63.3	64.8
Southeast Asia	55 to 59	43.8	52.6	52.6	63.0	39.3	46.4	59.3	61.6	63.9	67.0	50.8	52.2	59.1	69.6	64.3	72.8	55.3	68.1	56.2	59.1	65.7	71.1	55.7	56.1
Southern Latin America	55 to 59	63.7	79.8	69.8	83.9	61.2	77.9	75.5	85.2	80.1	88.3	66.5	78.0	55.1	75.8	60.7	77.4	49.0	75.1	73.1	85.0	77.7	87.0	77.5	89.3
Southern Sub-Saharan Africa	55 to 59	70.2	60.2	75.0	67.2	65.6	53.6	68.3	48.6	69.9	51.7	63.5	42.5	82.0	75.7	83.0	75.6	81.9	76.7	81.4	75.1	83.8	79.4	80.6	72.0
Tropical Latin America	55 to 59	44.8	64.1	55.1	70.5	39.3	60.6	57.2	65.6	66.1	72.9	42.3	52.8	40.2	49.7	45.7	51.3	32.6	48.5	42.4	68.9	59.8	78.5	37.9	67.0
Western Europe	55 to 59	75.4	86.5	79.0	89.4	74.5	85.9	79.9	91.0	82.5	91.4	73.5	87.5	73.7	85.0	73.6	83.9	75.5	87.0	73.1	85.6	78.2	88.2	77.1	90.6
Western Sub-Saharan Africa	55 to 59	51.1	61.2	57.3	66.8	49.6	57.7	60.7	65.6	58.7	64.9	58.5	63.9	70.7	78.2	72.0	77.4	71.7	79.1	56.6	61.0	74.0	77.2	46.2	44.1
Andean Latin America	60 to 64	59.1	75.2	65.5	79.2	57.3	73.9	59.8	70.7	66.1	75.7	50.0	62.7	74.5	77.9	69.3	70.6	73.7	78.5	64.5	79.0	71.1	82.9	66.3	80.7
Australasia	60 to 64	76.0	89.5	78.0	90.7	77.1	89.8	79.5	91.7	80.7	92.6	76.4	89.6	71.1	87.7	67.3	85.0	68.8	83.0	77.9	91.5	76.8	89.2	87.0	97.6
Caribbean	60 to 64	44.7	56.5	51.1	63.6	44.8	54.6	51.5	57.0	59.3	64.5	39.6	45.6	69.9	75.2	66.1	70.7	67.4	73.4	49.1	60.0	51.9	64.5	57.6	65.2
Central Asia	60 to 64	55.7	62.5	62.8	69.3	53.8	60.5	60.7	65.6	65.5	70.0	53.0	58.4	71.6	67.2	69.9	66.9	69.7	64.8	54.2	61.0	61.0	66.6	57.5	65.8
Central Europe	60 to 64	53.2	67.3	59.2	74.7	53.8	65.6	56.0	65.7	62.3	73.7	46.2	54.6	65.2	71.5	61.7	69.4	64.3	69.4	45.8	62.3	50.7	67.9	55.7	69.4
Central Latin America	60 to 64	67.6	76.1	72.9	81.8	65.1	72.2	69.8	75.2	75.7	81.6	59.7	64.3	84.1	79.4	79.5	75.1	82.2	77.1	66.9	78.0	70.4	82.1	70.8	79.5
Central Sub-Saharan Africa	60 to 64	47.2	51.9	55.5	60.5	44.3	48.1	49.3	48.8	54.8	55.6	40.1	38.3	78.9	80.9	76.1	78.4	77.1	78.8	64.1	67.0	66.9	70.4	68.0	69.4
East Asia	60 to 64	33.2	62.3	42.5	69.9	33.2	60.4	54.0	67.6	62.7	75.1	41.4	57.2	2.4	70.6	2.5	71.1	2.2	67.1	44.2	65.4	49.0	68.4	54.0	73.2
Eastern Europe	60 to 64	56.1	66.3	63.2	75.2	53.7	62.0	62.7	68.5	68.0	75.3	54.7	58.5	66.3	67.7	67.2	69.9	61.2	62.1	39.8	56.8	47.3	66.2	45.2	59.4
Eastern Sub-Saharan Africa	60 to 64	39.3	52.6	52.2	62.6	34.4	48.6	42.6	48.1	53.5	57.3	26.9	34.6	75.6	81.5	77.5	80.5	71.7	78.8	68.0	70.2	69.5	73.3	73.5	73.4
High-income Asia Pacific	60 to 64	75.7	90.4	77.8	91.9	77.0	91.0	86.4	98.0	88.8	98.5	81.9	97.1	75.1	91.6	69.9	87.5	76.0	89.9	76.4	90.8	75.0	90.1	85.5	96.2
High-income North America	60 to 64	81.9	99.3	83.9	99.5	82.0	99.3	82.1	92.4	84.8	94.0	77.2	89.4	75.4	85.0	70.7	83.2	73.6	78.4	83.5	99.3	82.4	99.3	90.2	99.5

North Africa and Middle East	60 to 64	48.5	64.8	51.9	67.1	51.8	67.0	54.7	67.0	61.6	70.3	44.3	61.3	61.4	79.8	64.2	78.0	55.7	76.5	34.0	52.0	32.3	51.0	50.0	64.3
Oceania	60 to 64	27.2	37.7	27.7	38.9	34.8	43.4	43.6	48.9	44.1	49.6	37.3	42.8	61.9	71.9	46.9	58.2	71.3	79.2	58.5	64.2	53.7	60.6	73.1	76.9
South Asia	60 to 64	40.5	52.4	41.0	53.1	46.8	57.4	60.6	68.2	65.9	71.3	52.2	62.9	49.7	62.4	47.4	54.9	51.5	66.0	45.9	54.0	40.1	52.5	62.2	65.8
Southeast Asia	60 to 64	44.7	53.7	51.9	62.8	44.5	51.2	55.1	59.4	61.5	66.2	45.3	49.3	66.2	75.6	65.0	73.5	63.4	73.3	58.7	62.1	65.3	70.7	62.9	64.9
Southern Latin America	60 to 64	65.4	79.9	70.8	83.9	65.1	79.0	74.5	83.2	81.4	88.7	64.0	75.1	64.7	82.0	62.3	78.4	61.5	79.9	69.6	83.9	71.6	84.1	77.0	89.5
Southern Sub-Saharan Africa	60 to 64	66.6	56.6	72.6	64.3	62.9	51.9	62.9	46.7	67.0	51.1	56.7	39.9	91.3	87.5	89.1	84.0	87.4	84.8	73.1	62.2	77.1	68.7	72.1	59.8
Tropical Latin America	60 to 64	50.5	66.5	58.3	72.6	49.8	65.1	57.9	65.6	66.2	73.3	45.2	53.8	69.8	65.9	63.3	57.3	71.4	70.4	43.2	66.7	54.3	73.7	48.2	70.7
Western Europe	60 to 64	73.3	86.1	77.3	89.1	73.1	85.5	77.2	88.6	81.9	91.6	69.9	84.2	81.5	89.9	75.9	85.7	81.6	87.4	72.8	87.6	73.7	87.0	81.1	94.0
Western Sub-Saharan Africa	60 to 64	48.4	58.3	58.2	67.0	47.0	54.3	56.7	63.1	57.4	65.1	51.5	59.0	79.6	86.4	78.4	82.9	76.5	83.0	53.3	58.1	72.5	75.5	46.9	45.4
Andean Latin America	65 to 69	57.9	74.6	63.3	77.4	51.9	71.4	55.4	66.2	58.8	68.6	52.3	63.9	73.2	76.5	70.9	71.9	75.1	81.1	67.5	80.2	72.7	83.1	62.2	77.8
Australasia	65 to 69	76.9	91.6	77.9	92.8	76.2	90.9	76.4	88.2	76.5	88.9	76.1	87.4	72.4	87.6	71.7	89.4	72.5	84.1	77.7	92.3	77.0	91.5	79.7	94.5
Caribbean	65 to 69	47.6	55.9	52.4	61.6	42.4	49.8	51.4	54.6	54.5	57.5	48.4	51.8	74.0	76.2	73.7	75.9	73.4	75.3	55.7	61.4	57.6	65.4	54.2	57.9
Central Asia	65 to 69	54.3	61.5	60.2	67.6	46.3	54.9	53.0	59.6	54.6	60.4	52.4	58.4	68.3	62.8	69.7	65.4	67.0	61.9	58.8	63.7	63.0	68.3	51.5	59.0
Central Europe	65 to 69	51.7	67.7	55.0	72.9	48.5	63.1	46.9	58.8	48.9	63.5	45.4	54.1	62.4	68.1	61.1	69.1	64.1	67.7	48.0	65.0	50.2	68.7	46.8	63.3
Central Latin America	65 to 69	66.9	75.6	71.8	81.0	61.0	69.0	65.6	71.2	69.0	75.6	61.7	65.8	83.9	79.6	82.9	78.7	84.0	79.6	68.9	78.6	72.8	83.2	64.8	74.3
Central Sub-Saharan Africa	65 to 69	45.6	49.3	53.8	57.9	35.3	37.9	42.5	41.0	43.6	44.1	41.5	38.4	78.4	79.9	80.2	81.7	77.3	78.6	64.9	66.6	68.6	70.9	59.0	59.0
East Asia	65 to 69	34.5	63.4	41.9	69.9	27.3	57.2	49.5	62.8	55.5	68.6	43.4	56.9	2.2	67.9	2.4	72.1	2.0	65.2	50.2	67.6	53.9	70.4	47.7	66.3
Eastern Europe	65 to 69	52.2	65.2	57.4	72.1	44.7	57.1	56.3	64.0	58.8	67.6	55.3	60.0	65.3	66.0	68.5	71.2	58.7	60.5	37.8	57.1	42.4	63.7	29.5	49.7
Eastern Sub-Saharan Africa	65 to 69	39.2	51.9	54.1	62.9	23.3	39.1	35.1	42.1	44.8	49.9	25.6	34.3	75.9	81.3	83.4	85.4	72.1	78.7	69.8	70.7	72.8	75.0	66.0	64.8
High-income Asia Pacific	65 to 69	77.0	92.9	78.2	93.3	76.0	93.2	85.2	99.1	86.1	98.7	83.6	99.2	79.4	93.2	78.2	93.0	81.5	93.6	75.8	90.5	75.1	90.0	77.8	92.3
High-income North America	65 to 69	82.7	99.3	85.0	99.4	80.2	99.4	79.2	90.0	80.2	90.9	78.0	89.1	76.2	84.0	76.2	86.1	74.7	79.5	83.5	99.2	84.1	99.0	84.2	99.5
North Africa and Middle East	65 to 69	45.3	62.7	47.5	63.4	43.2	62.0	48.3	63.0	52.9	64.0	43.7	62.1	61.9	77.0	68.6	79.3	57.0	75.2	36.7	53.4	34.0	50.9	39.7	56.5
Oceania	65 to 69	30.1	39.1	28.6	38.3	30.6	39.1	41.6	45.9	36.2	40.2	44.3	48.4	64.5	72.5	58.6	67.1	70.8	78.2	67.0	70.2	63.4	67.2	71.7	74.3
South Asia	65 to 69	39.3	47.5	39.7	47.1	38.6	47.8	59.0	64.1	61.4	64.6	56.5	63.6	49.2	58.6	52.0	54.9	50.3	63.3	48.7	52.4	45.3	50.7	52.3	54.6
Southeast Asia	65 to 69	44.0	53.1	48.9	60.4	38.9	45.7	49.7	54.0	51.2	56.5	48.8	51.6	65.8	74.3	67.7	75.6	64.5	73.2	62.8	65.2	67.9	72.3	58.1	58.9
Southern Latin America	65 to 69	66.7	80.0	70.9	83.4	62.7	76.9	71.7	79.3	76.4	83.6	66.2	74.5	66.5	81.2	67.2	81.7	65.6	80.3	72.3	83.9	74.3	84.8	71.6	84.2
Southern Sub-Saharan Africa	65 to 69	67.2	59.3	72.9	66.3	58.8	48.3	56.7	42.7	57.6	44.4	56.8	42.6	89.0	85.4	91.3	86.2	86.2	84.0	76.1	68.1	79.5	73.0	69.3	58.3
Tropical Latin America	65 to 69	52.6	67.4	58.9	72.6	46.7	62.5	55.8	62.6	59.1	66.5	52.5	58.6	72.2	66.5	68.4	61.2	75.8	72.5	51.4	70.1	61.0	76.2	43.8	66.3
Western Europe	65 to 69	72.8	87.7	75.9	89.5	69.8	86.3	73.2	85.4	75.5	87.0	70.5	83.9	80.3	89.4	78.4	90.1	82.2	87.7	72.2	88.2	73.6	87.8	72.4	90.0
Western Sub-Saharan Africa	65 to 69	47.8	56.7	52.6	62.0	42.8	50.7	52.7	59.0	45.8	54.2	58.0	63.9	80.2	85.8	80.0	84.1	80.3	86.1	59.0	61.5	71.9	74.3	43.3	43.9
Andean Latin America	70 to 74	60.4	74.8	72.9	83.1	52.6	69.1	51.8	61.6	67.4	73.6	53.3	62.9	82.3	81.4	87.7	86.5	83.5	84.5	68.9	81.2	78.2	87.1	61.0	75.6
Australasia	70 to 74	76.4	92.1	82.5	95.1	72.9	89.1	73.1	82.5	80.3	87.0	74.6	83.0	80.2	90.0	86.7	94.1	81.1	87.7	78.3	94.7	82.4	95.4	75.7	92.9
Caribbean	70 to 74	48.8	58.6	62.7	71.4	42.3	51.2	48.5	53.7	64.9	68.5	50.5	55.0	81.4	83.7	88.8	90.2	79.2	81.5	56.3	64.5	66.1	73.8	50.9	57.9
Central Asia	70 to 74	54.7	59.4	68.6	72.8	43.8	49.7	47.3	52.6	62.6	65.5	52.0	56.2	75.0	67.8	84.7	80.3	72.1	66.4	62.7	65.6	73.8	76.7	50.1	54.7
Central Europe	70 to 74	49.7	67.1	61.6	76.6	45.6	61.6	41.4	52.9	59.2	68.2	44.9	53.4	73.2	74.8	83.2	84.9	71.5	72.2	50.9	68.9	61.1	76.3	46.5	64.0
Central Latin America	70 to 74	67.6	75.7	78.3	85.5	60.1	67.4	62.0	67.3	74.0	78.5	62.8	66.0	88.6	84.2	92.4	89.6	87.9	83.7	71.8	80.2	80.4	87.6	64.0	72.5
Central Sub-Saharan Africa	70 to 74	47.7	50.6	64.6	67.0	35.8	36.4	36.2	34.2	55.1	56.2	40.5	36.1	82.7	83.7	90.0	90.5	81.3	81.8	65.2	66.5	75.2	76.4	55.7	54.9
East Asia	70 to 74	34.9	63.2	52.2	76.2	27.6	54.9	38.9	54.0	59.4	70.0	40.1	53.7	2.0	67.7	37.6	81.2	1.6	65.0	59.9	72.9	70.4	81.6	53.0	66.1
Eastern Europe	70 to 74	46.3	62.8	61.0	75.1	37.5	53.4	52.1	58.3	66.8	71.6	55.4	59.0	72.4	72.2	84.0	84.6	63.8	65.1	41.0	61.8	56.2	73.1	28.8	51.3
Eastern Sub-Saharan Africa	70 to 74	45.8	56.4	64.7	71.3	32.9	45.3	30.2	39.0	54.2	59.4	30.7	40.0	81.8	86.0	91.1	92.2	78.9	84.1	71.4	72.8	78.6	80.6	65.9	65.2
High-income Asia Pacific	70 to 74	77.0	96.3	83.3	98.8	73.5	93.3	82.2	98.8	87.4	98.8	81.8	97.7	83.7	95.2	88.6	96.1	85.7	95.8	77.8	95.2	82.3	96.4	74.9	92.8
High-income North America	70 to 74	83.3	97.5	89.2	99.6	78.3	94.4	77.4	86.4	83.7	90.2	77.8	86.4	84.7	87.3	90.6	93.0	82.3	83.4	85.1	100.0	88.9	99.9	81.4	98.0
North Africa and Middle East	70 to 74	40.2	58.7	52.1	67.2	37.9	56.6	39.5	57.0	59.2	69.7	41.6	59.8	65.7	77.2	81.4	86.4	61.3	75.5	42.1	58.1	50.9	64.8	40.3	56.2
Oceania	70 to 74	35.5	42.0	49.6	54.2	31.8	39.0	33.9	36.5	51.7	52.2	39.6	43.1	68.6	74.4	74.4	78.8	78.1	82.7	67.6	70.0	71.3	73.4	68.0	70.3
South Asia	70 to 74	43.1	48.2	53.9	58.9	40.9	45.5	57.7	61.3	70.4	72.3	59.4	63.5	62.9	69.2	78.0	79.7	62.0	70.6	52.3	54.2	57.9	61.9	52.0	51.7
Southeast Asia	70 to 74	45.1	53.5	59.3	68.3	39.1	44.7	42.5	47.4	58.6	63.4	48.1	50.4	70.6	77.3	81.9	85.9	69.4	76.3	62.6	65.9	72.3	76.8	56.0	56.8
Southern Latin America	70 to 74	66.9	81.2	77.0	88.6	60.9	75.2	69.5	77.2	80.7	86.1	66.8	74.0	74.8	85.2	84.0	90.5	74.1	84.2	71.3	85.4	78.2	89.7	66.6	81.2
Southern Sub-Saharan Africa	70 to 74	72.2	66.1	82.2	77.4	62.0	55.7	57.1	46.8	69.0	62.0	61.1	52.5	89.6	86.7	94.1	91.7	86.7	85.2	79.5	72.9	86.2	81.0	70.0	62.9

Tropical Latin America	70 to 74	51.7	69.4	65.8	79.6	44.4	62.9	53.4	61.8	68.4	74.3	54.8	62.1	84.8	78.8	89.0	85.5	86.7	80.1	52.9	72.2	65.9	80.6	43.6	65.8
Western Europe	70 to 74	72.1	89.2	79.8	92.9	67.7	86.0	71.2	81.2	79.8	86.3	71.9	81.5	85.9	90.8	90.4	94.5	86.2	88.9	72.6	91.5	78.5	92.9	69.0	89.7
Western Sub-Saharan Africa	70 to 74	47.8	55.9	64.1	70.8	38.1	45.8	49.0	56.5	58.9	64.9	58.3	64.6	84.3	88.7	90.5	92.6	83.6	88.7	60.2	63.3	77.4	79.7	40.3	43.3
Andean Latin America	75 to 79	67.0	78.4	75.7	84.3	54.7	69.4	54.5	64.9	69.6	76.3	52.9	63.4	81.5	81.0	88.6	87.4	79.7	81.2	73.1	82.7	80.7	87.7	62.7	75.3
Australasia	75 to 79	78.2	91.0	82.4	92.6	71.5	86.5	72.6	79.9	81.0	86.0	71.5	78.2	76.1	85.3	85.3	92.5	74.5	79.8	78.4	92.5	82.7	93.2	72.7	89.7
Caribbean	75 to 79	58.8	66.7	67.8	74.9	47.2	55.4	54.6	59.1	69.7	73.0	53.1	57.0	82.1	83.5	90.2	91.0	77.9	79.2	63.6	70.4	71.3	77.4	54.7	61.4
Central Asia	75 to 79	59.4	65.6	69.8	74.9	42.6	51.1	47.4	56.6	63.8	68.8	48.4	57.6	70.3	59.9	83.0	76.6	63.0	53.9	65.3	69.6	75.4	78.9	49.1	55.3
Central Europe	75 to 79	52.0	70.3	61.1	76.4	41.2	61.6	37.3	51.4	58.0	67.4	36.5	49.6	67.0	68.7	79.9	81.2	62.1	63.6	52.6	71.1	62.9	77.4	42.0	63.5
Central Latin America	75 to 79	71.5	79.0	79.1	85.5	60.2	68.6	63.4	69.6	75.0	80.0	62.4	66.8	87.6	82.7	93.0	89.7	84.5	80.0	74.1	81.7	81.8	87.5	63.2	72.9
Central Sub-Saharan Africa	75 to 79	61.7	63.3	72.6	73.7	45.3	45.2	46.8	46.3	64.4	65.2	45.1	42.9	82.2	82.9	91.4	91.5	77.3	77.6	72.8	73.1	80.6	80.8	61.3	60.1
East Asia	75 to 79	50.1	70.9	59.6	79.1	38.3	59.7	48.3	61.2	64.0	72.5	48.6	60.7	1.7	65.1	34.0	79.7	3.9	60.0	67.7	76.8	75.4	84.2	58.4	67.3
Eastern Europe	75 to 79	43.9	61.3	55.9	70.7	29.3	47.3	51.1	55.4	66.8	70.2	52.0	53.8	65.5	64.0	80.4	79.4	51.2	52.8	38.0	59.1	53.5	70.0	20.3	44.2
Eastern Sub-Saharan Africa	75 to 79	59.9	66.6	71.0	75.5	44.1	53.1	42.4	49.4	62.3	66.4	39.4	47.6	81.0	85.0	91.7	92.6	75.1	80.3	76.1	76.9	81.9	83.0	68.6	68.2
High-income Asia Pacific	75 to 79	78.1	95.3	83.1	97.7	70.2	89.7	82.5	96.6	88.6	98.6	79.6	92.7	82.9	95.6	89.4	97.8	82.8	94.6	76.9	93.3	82.2	94.9	69.8	89.2
High-income North America	75 to 79	84.8	94.5	88.9	96.5	77.3	89.6	78.4	86.1	85.5	90.7	75.9	83.8	82.0	82.7	90.0	91.4	77.1	76.1	84.8	95.9	88.6	96.4	78.9	93.4
North Africa and Middle East	75 to 79	47.1	63.0	55.2	69.1	37.2	54.7	42.0	57.2	61.3	70.6	40.6	56.8	60.0	72.4	79.8	84.9	49.9	66.4	49.6	62.6	57.9	69.5	41.0	54.6
Oceania	75 to 79	54.3	57.2	62.0	64.2	44.4	48.3	41.1	41.7	58.2	57.8	42.0	43.6	68.1	73.0	76.3	79.8	71.8	76.3	75.6	76.5	78.6	79.4	72.2	73.3
South Asia	75 to 79	53.4	58.4	60.5	65.1	43.4	49.1	60.2	63.8	73.1	74.9	58.2	62.9	60.2	68.6	77.7	80.4	55.6	66.7	60.7	63.2	66.2	69.4	54.4	56.0
Southeast Asia	75 to 79	56.1	61.6	65.3	71.1	44.4	48.5	44.6	48.0	60.9	64.4	46.5	47.9	66.4	72.7	79.7	83.7	62.5	69.2	69.1	70.8	76.7	79.0	59.5	59.8
Southern Latin America	75 to 79	69.4	83.1	76.8	88.2	58.7	74.5	69.0	77.9	81.2	87.1	62.9	72.2	69.3	80.9	80.1	88.8	68.4	77.9	72.1	85.5	78.7	89.5	63.7	79.1
Southern Sub-Saharan Africa	75 to 79	79.4	75.2	85.8	82.1	66.8	62.9	61.7	53.0	73.9	68.2	61.0	53.7	89.8	86.8	95.6	93.0	83.9	82.6	83.6	79.4	88.8	85.2	73.2	69.2
Tropical Latin America	75 to 79	58.6	74.2	68.2	80.7	45.8	64.6	52.7	61.8	69.5	75.4	49.4	58.5	83.3	76.5	88.8	85.0	83.4	75.5	60.2	75.9	70.2	82.3	47.6	67.3
Western Europe	75 to 79	73.4	89.3	79.1	91.4	65.1	84.2	70.6	79.8	80.2	86.0	68.8	78.1	81.5	85.9	88.6	92.8	80.3	81.4	72.7	90.5	78.9	91.8	65.0	87.3
Western Sub-Saharan Africa	75 to 79	59.8	64.9	68.4	73.1	48.8	53.7	55.0	60.8	63.2	67.7	62.8	67.4	83.6	87.2	90.0	91.7	81.8	86.0	68.9	70.3	79.5	80.8	52.3	53.8
Andean Latin America	All ages	59.2	73.2	64.7	76.6	51.9	68.3	55.1	64.3	62.3	70.0	53.6	62.9	63.5	73.4	66.0	72.6	59.5	74.3	63.0	73.8	70.2	79.5	60.6	72.4
Australasia	All ages	68.1	80.9	65.8	77.8	69.7	82.9	67.3	73.3	69.7	75.6	68.8	74.0	65.1	78.2	66.6	80.2	62.2	74.0	59.1	77.4	54.8	72.9	68.2	84.4
Caribbean	All ages	44.9	52.9	49.3	57.9	39.2	46.4	43.4	46.3	52.0	55.6	42.4	44.0	62.0	69.2	64.1	70.5	57.7	66.2	42.6	49.0	46.6	53.9	45.7	50.7
Central Asia	All ages	54.2	61.0	58.5	66.4	48.6	53.9	50.0	56.1	54.5	61.4	52.6	56.2	68.3	66.0	71.2	70.3	64.8	61.3	52.8	58.1	58.8	65.6	52.5	55.9
Central Europe	All ages	38.3	52.8	40.0	54.4	36.4	51.0	38.6	45.0	45.4	54.0	40.7	43.5	57.8	62.7	61.5	65.4	52.3	59.2	20.4	38.6	20.5	38.8	29.8	46.6
Central Latin America	All ages	69.8	74.9	74.2	80.0	62.9	67.2	68.0	68.4	73.7	76.0	65.2	63.4	76.8	76.2	77.4	78.0	74.8	72.2	66.3	73.3	73.5	80.5	63.5	70.1
Central Sub-Saharan Africa	All ages	51.7	59.4	58.1	65.0	43.2	51.6	40.9	45.6	44.3	49.5	45.3	49.1	72.3	78.1	74.3	80.1	70.3	76.4	68.2	70.8	75.5	76.3	64.5	69.2
East Asia	All ages	35.4	55.4	42.9	63.5	26.8	46.6	40.4	44.1	50.4	54.3	38.9	42.1	12.7	61.2	20.0	67.2	3.7	54.3	49.3	57.1	57.8	66.6	47.5	54.6
Eastern Europe	All ages	39.5	50.0	40.4	52.9	40.0	47.0	48.2	51.5	51.8	58.5	52.6	51.0	60.1	56.1	64.6	60.8	54.0	50.1	13.3	31.7	13.3	34.0	25.7	38.6
Eastern Sub-Saharan Africa	All ages	49.3	61.4	58.9	68.7	37.7	52.1	38.7	45.9	48.7	53.1	37.5	46.2	71.2	78.4	77.4	82.5	65.9	75.2	72.7	74.6	78.3	80.5	71.3	72.5
High-income Asia Pacific	All ages	69.4	83.4	70.1	82.7	67.6	83.0	76.1	83.8	80.7	86.7	73.7	82.2	68.4	89.6	70.1	88.5	65.6	90.4	61.4	77.4	60.5	76.0	66.9	81.8
High-income North America	All ages	80.1	91.4	80.4	89.9	79.0	92.5	75.8	80.9	79.4	83.7	74.5	79.9	72.1	78.3	73.8	82.0	67.4	70.4	75.4	92.0	76.6	91.4	79.0	95.5
North Africa and Middle East	All ages	46.7	63.6	49.3	64.7	43.1	61.5	44.6	61.5	52.3	65.2	44.8	62.8	57.7	79.5	65.0	81.4	47.6	76.7	40.5	52.3	42.1	54.0	45.9	56.6
Oceania	All ages	41.5	46.2	44.4	48.6	37.8	42.7	32.2	33.8	34.5	35.2	38.0	39.9	56.7	64.8	56.1	63.1	57.3	66.6	73.3	73.7	76.0	76.6	75.5	75.6
South Asia	All ages	48.9	54.3	52.6	56.9	44.4	50.7	52.4	57.2	59.9	62.4	51.2	57.4	52.3	62.6	59.6	64.7	45.1	60.7	55.5	54.3	57.5	57.5	59.3	57.2
Southeast Asia	All ages	46.3	50.3	51.9	57.4	39.2	42.0	44.5	43.9	51.2	51.3	45.6	44.4	60.1	68.0	65.0	71.6	53.1	63.3	58.9	56.7	66.1	65.0	57.1	54.4
Southern Latin America	All ages	60.2	76.0	63.5	78.2	55.9	72.6	65.4	75.4	73.2	81.9	61.2	70.6	55.2	75.3	59.3	76.1	48.7	73.6	56.1	72.9	57.1	74.6	60.9	75.8
Southern Sub-Saharan Africa	All ages	70.1	62.3	75.6	67.8	60.9	53.5	54.3	35.8	58.3	39.5	56.7	42.2	84.7	81.8	85.3	82.7	83.8	80.5	76.8	67.2	83.9	73.4	71.3	64.1
Tropical Latin America	All ages	46.1	63.0	52.7	67.1	38.3	57.8	48.0	55.0	58.4	64.4	44.2	51.1	52.0	59.0	55.5	59.9	45.8	57.3	39.2	59.3	47.6	64.9	38.7	59.7
Western Europe	All ages	59.3	77.2	59.2	75.9	59.5	78.2	62.0	70.6	66.5	73.7	62.5	70.9	73.1	80.1	72.5	80.2	74.0	79.6	44.6	72.4	43.1	70.5	54.0	78.8
Western Sub-Saharan Africa	All ages	54.9	66.0	59.8	71.7	49.1	58.6	51.0	61.4	49.4	60.8	57.7	66.3	78.0	83.4	81.0	85.5	75.3	81.3	62.6	68.8	75.9	82.7	53.3	57.1
Andean Latin America	Age-standardized	61.4	76.8	65.8	79.6	55.1	72.4	58.5	70.1	63.4	73.8	53.6	66.3	70.6	76.6	67.1	71.3	71.6	79.7	68.5	80.3	75.7	85.4	58.9	73.2
Australasia	Age-standardized	72.0	88.5	71.5	87.1	72.0	89.3	74.6	84.1	76.5	85.6	72.9	82.7	69.3	84.2	66.2	82.6	69.6	82.1	69.4	87.7	71.7	87.1	67.4	87.6
Caribbean	Age-standardized	48.0	59.4	51.6	64.1	42.7	52.6	51.2	56.8	57.0	63.5	45.2	49.5	66.7	71.7	63.3	67.7	66.8	72.4	53.9	64.5	61.0	71.9	46.0	55.1

Central Asia	Age-standardized	55.4	60.5	61.0	66.5	46.3	51.7	55.3	60.2	59.9	64.9	50.6	55.2	70.6	66.8	70.0	67.4	68.6	65.0	61.5	63.8	70.9	73.3	46.7	49.6
Central Europe	Age-standardized	43.1	63.0	47.1	67.1	37.9	58.0	50.9	62.4	57.5	70.5	44.2	53.3	62.9	72.9	61.7	72.2	61.7	71.4	38.7	60.1	48.5	67.3	28.3	52.2
Central Latin America	Age-standardized	68.5	78.2	71.9	82.7	62.8	71.4	67.9	74.2	71.9	79.7	63.3	67.3	82.0	79.3	78.5	77.2	82.2	77.9	69.2	80.0	75.9	85.9	59.8	71.3
Central Sub-Saharan Africa	Age-standardized	51.4	54.4	57.6	60.9	42.0	43.4	43.9	42.0	46.3	46.2	41.7	38.2	77.8	81.0	76.7	79.8	77.3	79.7	68.0	68.1	75.6	75.9	56.4	55.1
East Asia	Age-standardized	32.3	59.9	40.5	68.1	21.2	48.8	42.1	54.6	51.7	63.3	30.8	44.5	2.3	67.4	2.4	69.3	2.2	63.8	54.1	67.7	65.0	77.3	40.2	54.2
Eastern Europe	Age-standardized	43.4	58.4	48.7	64.2	35.7	50.9	58.2	63.6	62.8	70.4	53.4	56.2	65.0	64.6	66.3	66.1	60.3	61.4	33.1	53.6	46.3	63.7	16.7	41.0
Eastern Sub-Saharan Africa	Age-standardized	48.7	58.8	57.5	65.6	37.0	48.8	40.2	44.3	48.1	50.2	32.5	38.6	75.5	82.1	78.6	82.8	72.3	80.1	73.1	74.1	78.8	80.2	64.8	64.8
High-income Asia Pacific	Age-standardized	72.2	92.6	73.7	94.6	69.6	90.4	81.4	95.2	84.8	98.6	77.7	92.1	73.1	91.0	69.8	88.1	74.3	90.8	69.5	90.5	72.8	92.8	64.8	87.1
High-income North America	Age-standardized	85.5	99.8	86.8	99.7	83.3	99.8	83.3	91.2	86.1	92.9	80.1	89.8	75.9	84.5	73.4	84.8	74.4	79.2	84.4	99.6	87.3	99.6	80.5	99.5
North Africa and Middle East	Age-standardized	42.6	60.6	44.1	60.6	40.2	59.5	48.6	63.2	54.1	65.4	43.7	61.6	59.7	78.8	63.1	77.8	54.0	77.3	42.0	56.2	48.1	60.7	36.1	51.5
Oceania	Age-standardized	37.7	44.1	38.9	44.7	34.9	42.0	33.4	35.5	34.0	34.7	31.9	35.1	56.7	66.1	47.9	56.9	64.7	73.5	69.7	72.2	72.3	74.5	67.2	69.8
South Asia	Age-standardized	46.9	55.0	48.2	56.8	43.9	51.8	56.8	62.8	61.7	66.0	51.7	59.6	54.0	65.1	55.6	61.9	53.2	67.1	56.6	60.3	60.1	65.9	52.1	53.6
Southeast Asia	Age-standardized	45.0	52.4	49.9	58.9	38.6	44.4	47.5	50.9	52.2	56.5	43.5	45.6	60.6	70.2	60.3	69.3	58.9	69.2	61.2	63.3	69.6	72.4	50.7	51.7
Southern Latin America	Age-standardized	64.1	81.6	67.3	84.4	59.7	77.7	72.0	82.7	77.9	87.8	64.9	76.3	59.4	79.9	57.7	77.2	59.0	79.6	66.1	82.5	70.7	85.7	60.8	78.1
Southern Sub-Saharan Africa	Age-standardized	71.5	63.8	76.4	68.9	62.0	54.7	58.6	42.9	61.3	45.9	56.9	42.1	88.7	85.0	86.4	82.3	87.6	84.5	79.6	71.3	86.0	78.4	67.0	59.3
Tropical Latin America	Age-standardized	46.1	67.6	51.6	71.5	39.0	62.4	53.1	63.8	60.9	71.0	44.6	55.7	57.7	63.2	54.2	58.2	58.3	65.9	46.7	70.1	57.6	77.1	33.7	61.3
Western Europe	Age-standardized	66.8	87.0	68.7	87.5	63.7	85.9	72.6	83.4	76.2	86.0	68.7	80.6	76.9	87.3	72.5	84.3	79.3	87.2	62.4	85.9	67.8	87.6	55.9	83.6
Western Sub-Saharan Africa	Age-standardized	53.1	60.9	57.4	65.6	47.4	54.2	53.4	60.2	50.0	57.3	57.2	63.7	81.6	87.2	80.3	85.2	81.4	86.8	64.3	66.7	77.3	79.2	45.9	47.8
Andean Latin America	80 to 84	73.5	80.7	80.4	86.2	65.5	74.1	61.1	68.8	63.0	70.1	60.4	68.2	84.5	83.3	86.9	84.9	83.0	83.1	75.2	82.2	82.6	87.9	71.0	78.4
Australasia	80 to 84	79.6	90.5	83.2	91.7	76.5	88.5	73.4	78.0	74.5	78.7	73.3	77.6	80.2	82.2	82.7	86.1	77.9	78.3	79.7	92.1	83.9	93.0	79.3	91.6
Caribbean	80 to 84	65.0	70.7	71.9	77.3	58.2	63.7	59.7	62.9	61.7	65.6	58.9	61.2	84.8	85.8	88.5	89.2	81.3	82.1	65.5	71.5	73.2	78.5	64.1	69.2
Central Asia	80 to 84	61.4	66.1	71.4	75.1	47.8	53.9	47.4	54.7	50.7	56.7	47.0	54.9	70.4	60.1	74.7	62.8	65.5	59.9	65.8	69.2	76.6	79.4	57.2	60.9
Central Europe	80 to 84	52.5	70.1	61.9	76.2	44.3	64.4	37.0	48.8	42.3	53.0	34.1	46.0	69.2	68.2	72.5	71.3	65.8	65.5	53.3	71.5	64.6	78.5	51.5	70.0
Central Latin America	80 to 84	77.2	81.7	83.3	87.1	69.9	74.9	68.2	71.8	69.4	73.5	67.9	70.5	90.3	84.4	92.1	86.7	88.4	82.8	78.1	83.0	85.0	88.6	73.6	79.0
Central Sub-Saharan Africa	80 to 84	70.0	70.3	79.7	79.3	57.6	56.6	47.1	44.6	49.3	48.4	46.0	43.7	83.0	83.8	89.1	88.4	79.4	79.5	77.3	76.9	85.9	85.1	69.9	68.5
East Asia	80 to 84	55.0	73.4	63.2	80.9	47.5	64.8	32.9	49.5	39.1	53.5	28.7	46.6	1.7	62.3	2.0	65.8	10.2	61.4	72.4	81.1	78.6	87.9	71.1	76.4
Eastern Europe	80 to 84	34.6	56.5	47.1	66.6	28.3	45.3	48.4	52.8	52.1	57.5	48.5	48.4	63.4	62.3	67.8	66.5	52.0	53.8	29.5	55.3	46.4	67.7	31.8	50.1
Eastern Sub-Saharan Africa	80 to 84	70.4	74.6	80.0	82.6	57.9	63.9	43.7	50.5	49.9	54.0	38.5	48.3	81.8	85.8	90.2	91.1	76.3	81.8	80.5	81.4	87.4	88.1	75.1	75.8
High-income Asia Pacific	80 to 84	78.2	95.1	83.6	98.1	72.2	90.5	79.8	92.1	82.7	95.5	76.7	88.0	85.2	95.4	86.0	95.6	87.8	97.5	77.5	94.0	83.3	96.1	75.2	91.7
High-income North America	80 to 84	87.4	95.8	91.3	97.7	82.6	93.0	81.1	88.5	82.7	89.0	79.7	88.3	84.6	82.1	87.2	86.4	81.4	77.1	87.5	97.0	91.6	97.9	84.9	96.0
North Africa and Middle East	80 to 84	46.8	64.4	53.9	69.8	41.2	59.7	38.6	56.1	42.7	57.8	36.7	55.9	59.5	71.8	68.7	76.1	51.6	68.8	49.1	65.1	56.9	71.6	51.1	65.1
Oceania	80 to 84	65.1	66.3	71.4	72.2	58.9	60.5	37.4	36.2	39.7	37.6	36.6	36.5	69.2	73.3	69.3	73.0	71.6	75.8	79.8	80.3	83.6	84.1	79.1	79.6
South Asia	80 to 84	65.1	66.2	70.6	71.7	58.9	60.7	65.3	66.0	67.6	67.5	63.3	65.2	68.4	74.0	75.9	77.0	65.2	73.4	67.6	67.9	73.6	74.4	66.9	67.1
Southeast Asia	80 to 84	62.2	65.1	69.5	73.0	55.6	57.0	44.1	46.3	44.4	48.1	46.9	47.3	64.7	70.9	68.0	73.6	63.3	69.5	69.6	70.7	77.2	78.9	67.1	66.9
Southern Latin America	80 to 84	72.0	84.8	78.5	89.3	65.4	79.2	70.2	79.2	74.6	83.3	64.5	73.5	70.5	80.3	68.8	82.3	75.5	80.0	73.3	86.5	80.0	90.7	71.2	83.8
Southern Sub-Saharan Africa	80 to 84	83.5	79.4	89.5	85.7	73.9	70.5	62.6	55.4	63.6	57.1	64.6	58.2	90.4	87.9	92.9	90.0	87.6	85.9	86.9	82.3	92.7	88.5	79.5	76.3
Tropical Latin America	80 to 84	59.7	77.7	68.1	82.8	51.2	72.0	58.3	66.9	62.0	70.0	55.5	63.9	86.5	79.8	88.0	83.2	85.9	77.4	57.9	78.3	68.1	83.9	54.9	75.7
Western Europe	80 to 84	74.1	90.2	80.1	92.7	67.9	87.0	71.8	79.1	73.9	80.2	70.7	78.3	82.7	84.2	83.9	87.3	83.3	81.6	73.6	91.7	80.7	93.8	71.1	90.5
Western Sub-Saharan Africa	80 to 84	68.6	72.5	76.8	80.2	59.2	63.4	58.6	64.6	53.1	59.0	66.3	71.2	85.8	89.0	88.0	90.1	84.9	88.7	73.7	75.8	84.2	85.8	64.3	66.9
Andean Latin America	85 to 89	83.8	88.2	88.9	92.4	78.5	82.4	70.0	75.7	75.9	81.0	74.1	78.0	90.1	87.3	90.8	87.4	89.3	87.5	84.5	88.5	89.2	92.3	82.5	85.9
Australasia	85 to 89	84.5	91.9	88.4	93.3	82.9	90.4	75.6	78.5	80.6	82.9	79.6	81.2	83.1	82.6	83.3	84.5	82.6	80.6	84.5	92.1	88.3	93.2	85.8	93.5
Caribbean	85 to 89	74.3	80.8	81.9	86.9	67.7	74.3	66.1	71.4	73.3	78.4	70.0	73.1	88.8	89.5	89.7	91.1	87.8	87.5	74.2	80.8	82.1	86.8	71.9	78.4
Central Asia	85 to 89	71.2	74.6	82.3	84.3	53.8	60.0	56.4	64.4	67.8	73.7	57.5	64.6	72.7	65.0	72.9	63.5	73.1	69.0	74.9	76.8	85.0	86.1	62.5	66.2
Central Europe	85 to 89	63.7	77.3	76.1	84.7	51.5	69.9	47.3	56.9	61.5	68.5	48.5	57.3	71.6	70.7	71.3	69.5	73.2	73.5	65.7	78.8	78.1	85.9	58.4	75.4
Central Latin America	85 to 89	82.6	89.1	87.3	92.8	79.0	84.2	71.5	78.3	75.6	83.0	78.1	80.4	90.8	87.3	90.4	87.4	91.0	87.3	83.0	89.3	87.6	92.6	82.1	87.5
Central Sub-Saharan Africa	85 to 89	82.7	82.2	90.2	89.7	71.8	68.7	56.8	52.5	64.4	63.2	62.6	58.2	87.1	87.5	91.5	91.1	84.8	83.9	87.1	86.2	92.7	91.9	80.5	77.6
East Asia	85 to 89	65.3	78.5	79.0	89.1	46.4	58.8	24.2	39.0	51.8	65.1	2.9	16.6	2.3	59.0	2.3	64.0	7.8	56.7	78.6	85.1	86.9	92.4	70.9	74.3
Eastern Europe	85 to 89	42.0	65.0	61.3	76.7	33.7	56.5	53.9	61.4	65.9	71.1	55.8	64.0	59.5	60.4	57.6	57.2	59.5	65.7	39.7	65.4	62.0	78.1	38.5	61.2

Eastern Sub-Saharan Africa	85 to 89	83.4	85.3	90.9	92.1	71.7	73.7	53.6	58.2	66.0	68.9	54.9	59.9	85.5	88.1	92.3	93.1	81.8	84.9	89.3	89.4	93.7	93.9	84.2	83.4
High-income Asia Pacific	85 to 89	83.4	96.5	89.0	98.3	77.0	92.2	80.9	89.4	86.4	93.7	80.3	86.6	89.6	95.6	88.7	95.0	92.0	96.2	82.5	94.6	88.0	96.0	80.1	93.7
High-income North America	85 to 89	92.4	97.7	95.0	98.3	88.9	96.1	85.7	90.7	88.8	92.0	87.1	93.3	85.7	82.0	85.7	83.1	85.7	81.3	91.5	97.0	93.9	96.9	90.9	98.5
North Africa and Middle East	85 to 89	59.1	70.2	69.1	76.9	52.7	65.2	51.4	61.7	61.5	68.3	57.4	67.7	64.1	71.4	70.0	72.8	61.7	72.9	60.9	71.3	70.8	78.3	60.1	70.3
Oceania	85 to 89	77.3	78.7	84.7	85.3	69.4	71.9	45.4	44.9	61.6	60.3	44.8	46.0	68.9	73.6	55.0	61.1	84.3	86.3	86.5	87.7	89.8	90.3	86.0	87.9
South Asia	85 to 89	75.8	77.9	82.5	84.7	69.2	70.8	70.4	71.8	76.8	78.5	72.7	73.5	72.8	78.0	74.8	77.7	75.4	80.3	77.4	79.1	83.7	85.7	75.1	75.9
Southeast Asia	85 to 89	72.7	75.5	81.1	83.5	65.3	67.4	47.4	51.1	58.1	62.1	55.0	56.9	66.5	72.7	64.7	71.0	71.0	76.5	78.1	79.6	85.1	86.5	75.0	75.8
Southern Latin America	85 to 89	81.8	88.8	87.6	92.5	75.8	84.1	76.9	82.6	83.7	87.9	75.0	80.2	73.3	79.8	63.9	78.5	82.0	82.5	82.3	89.1	87.8	92.2	80.1	88.0
Southern Sub-Saharan Africa	85 to 89	90.0	86.9	94.7	92.0	79.7	78.4	69.2	63.0	76.1	71.2	73.0	69.4	93.1	90.8	94.6	91.4	90.5	89.3	91.9	88.6	95.6	92.8	84.6	83.5
Tropical Latin America	85 to 89	73.5	85.1	82.0	89.9	65.1	79.6	67.7	72.6	75.8	79.7	69.4	73.1	89.5	82.7	89.4	83.2	89.5	83.1	73.0	85.3	82.0	89.9	68.4	83.1
Western Europe	85 to 89	80.4	91.8	86.9	94.2	73.5	88.8	75.0	79.8	80.9	84.3	77.3	81.8	83.2	82.9	82.1	83.6	85.9	82.7	80.4	92.1	87.0	93.9	76.9	92.3
Western Sub-Saharan Africa	85 to 89	80.1	82.5	86.9	89.0	72.4	74.3	66.7	71.6	68.8	73.0	77.2	80.0	88.6	91.0	88.9	90.9	88.6	91.0	83.2	84.3	90.0	91.0	75.9	77.0
Andean Latin America	95 plus	81.0	88.0	90.8	94.5	84.6	90.4	72.9	80.4	82.8	87.4	77.7	83.3	86.1	86.6	91.5	90.8	87.3	88.8	79.2	85.1	91.4	94.3	80.5	85.7
Australasia	95 plus	72.2	82.7	86.7	91.1	83.4	90.8	70.2	71.7	83.4	83.8	76.7	77.8	70.0	71.8	80.2	82.0	73.8	73.5	72.3	81.4	88.1	91.7	80.0	86.8
Caribbean	95 plus	69.7	80.2	85.5	90.9	76.6	84.0	68.2	78.9	81.5	87.5	75.8	81.8	84.6	89.4	91.1	94.4	84.5	87.8	67.9	77.6	86.4	91.1	72.2	79.3
Central Asia	95 plus	60.7	69.8	82.7	86.7	59.0	67.8	53.7	68.1	74.4	82.4	63.7	68.8	58.9	62.3	72.5	74.5	63.1	64.7	62.0	68.8	85.4	88.1	55.9	65.1
Central Europe	95 plus	59.8	73.2	81.8	87.9	65.1	77.6	55.4	64.1	76.3	80.7	60.8	67.6	66.0	67.5	76.9	77.4	70.5	73.3	61.9	73.6	84.5	89.6	64.5	75.5
Central Latin America	95 plus	86.0	91.5	93.5	96.1	88.3	93.3	80.0	86.6	87.5	91.4	82.0	86.5	90.9	88.4	95.3	93.1	90.5	88.4	83.3	87.8	93.5	95.4	83.8	88.2
Central Sub-Saharan Africa	95 plus	83.3	82.5	94.3	93.3	80.1	79.8	62.6	58.4	78.8	76.2	69.6	68.9	86.0	87.4	96.1	95.5	80.5	81.5	84.6	83.5	95.6	94.5	79.2	78.6
East Asia	95 plus	66.3	78.5	84.8	91.3	70.9	73.7	11.9	29.4	53.3	65.6	23.3	18.3	5.1	52.6	31.4	68.4	28.3	55.1	75.5	82.8	90.3	94.1	78.2	78.6
Eastern Europe	95 plus	28.2	58.2	66.1	80.5	41.9	67.1	48.5	59.9	72.7	78.1	54.3	66.2	38.2	47.0	58.2	62.2	40.4	59.9	31.6	60.3	71.2	83.8	43.4	66.0
Eastern Sub-Saharan Africa	95 plus	85.2	88.6	94.8	96.0	82.8	86.7	60.5	68.3	78.9	81.5	66.4	74.2	84.0	88.1	95.2	96.0	77.5	83.8	87.4	88.7	96.3	96.7	83.8	85.4
High-income Asia Pacific	95 plus	79.1	93.3	90.7	97.4	84.0	95.9	80.1	81.6	89.7	90.5	79.4	82.6	91.1	91.7	94.4	94.4	91.8	94.5	76.6	88.9	90.4	95.8	79.8	90.2
High-income North America	95 plus	89.5	92.1	95.5	96.6	93.3	96.4	90.5	89.1	94.0	93.1	89.4	91.6	78.2	69.3	85.3	79.1	82.6	75.3	85.8	88.6	94.6	95.6	88.0	91.3
North Africa and Middle East	95 plus	60.8	70.3	80.9	83.7	67.6	78.9	57.5	68.2	75.9	79.9	67.3	76.5	59.8	66.0	74.3	75.9	63.1	72.7	61.6	70.4	83.4	85.7	65.5	75.8
Oceania	95 plus	76.1	83.5	90.0	93.1	76.7	85.4	41.3	50.3	71.4	75.2	43.3	56.5	58.6	71.1	69.2	77.9	70.7	79.8	83.0	89.3	93.5	96.0	84.4	90.1
South Asia	95 plus	72.7	75.4	86.8	88.7	76.9	79.1	69.3	71.1	82.3	84.3	73.6	74.0	65.7	71.6	78.8	81.4	70.1	75.5	72.0	74.9	88.2	89.9	74.1	76.5
Southeast Asia	95 plus	70.9	75.3	87.0	88.2	73.9	81.7	46.6	50.2	70.5	70.7	59.1	65.2	57.1	64.5	70.2	74.4	64.7	73.0	74.3	77.5	90.0	90.7	74.6	81.1
Southern Latin America	95 plus	73.0	83.3	87.5	91.7	80.2	89.4	75.4	82.6	87.0	89.7	76.7	84.3	60.4	71.0	57.2	73.9	80.4	82.6	72.1	81.2	88.3	92.1	76.6	84.9
Southern Sub-Saharan Africa	95 plus	85.5	85.9	94.4	94.2	82.9	86.8	67.9	67.1	81.9	81.0	75.4	77.4	91.8	90.2	96.0	94.3	89.3	89.3	85.3	85.0	95.3	94.8	79.9	83.3
Tropical Latin America	95 plus	70.2	83.9	86.2	92.2	75.5	88.6	70.8	77.8	83.7	87.0	75.4	80.8	86.2	81.1	91.1	87.0	87.9	84.9	68.8	81.7	87.1	92.5	71.4	84.1
Western Europe	95 plus	72.0	83.6	87.4	92.5	78.1	88.7	72.3	72.8	84.6	84.8	77.7	78.1	71.2	70.1	79.7	79.9	80.6	75.5	71.8	82.4	88.6	93.0	74.7	85.2
Western Sub-Saharan Africa	95 plus	78.2	81.1	90.7	92.1	80.0	82.8	70.7	74.6	81.0	83.0	80.0	82.4	86.5	89.5	93.0	94.4	85.3	89.0	77.9	80.3	92.2	93.2	74.7	77.8

		QCI																							
		stroke						ICH						SAH						IS					
		both	both	female	female	male	male	both	both	female	female	male	male	both	both	female	female	male	male	both	both	female	female	male	male
location	age	1990	2021	1990	2021	1990	2021	1990	2021	1990	2021	1990	2021	1990	2021	2021	1990	2021	1990	1990	2021	1990	2021	1990	2021
Afghanistan	<5	41.5	75.1	49.4	79.1	32.5	71.2	45.0	80.3	52.7	83.0	36.2	77.7	71.4	81.7	78.3	86.7	63.6	77.1	59.5	76.8	62.8	83.6	78.0	84.2
Albania	<5	82.9	86.1	83.3	85.9	82.9	86.9	88.2	90.4	89.2	90.7	87.1	90.6	84.3	91.6	86.2	92.3	82.9	91.2	93.0	92.4	93.2	92.5	96.4	95.8
Algeria	<5	69.2	88.7	72.0	88.7	66.2	89.2	71.1	91.3	72.6	91.1	69.5	91.9	79.3	93.4	82.0	93.9	76.9	93.2	71.7	91.5	76.3	93.1	84.4	94.2
American Samoa	<5	90.0	94.1	90.0	93.5	90.5	95.0	93.1	95.8	92.5	95.3	94.1	96.5	93.1	97.2	92.6	96.8	93.9	97.7	92.6	95.9	94.0	96.6	94.7	97.2
Andorra	<5	87.2	94.4	86.5	94.3	88.6	94.7	92.3	97.4	91.9	97.1	93.7	97.9	87.9	98.0	84.8	97.7	91.4	98.2	84.2	95.2	85.4	96.6	90.9	95.4
Angola	<5	51.3	85.9	55.5	87.1	46.3	84.6	63.7	89.8	63.1	88.9	63.7	90.5	64.4	88.8	66.5	90.4	64.0	87.8	57.0	87.3	75.0	92.5	66.6	89.0
Antigua and Barbuda	<5	90.1	92.1	90.2	91.7	90.3	92.9	93.8	95.2	93.4	94.6	94.6	96.2	94.5	97.3	94.9	97.2	94.2	97.3	93.1	94.5	94.4	95.6	94.6	95.7
Argentina	<5	84.9	92.9	86.8	93.0	82.6	92.9	92.3	96.3	93.2	96.1	91.2	96.6	84.5	96.6	87.7	97.2	82.1	96.0	86.3	94.8	89.8	96.4	89.9	95.1
Armenia	<5	88.0	90.8	89.1	90.9	86.8	90.9	92.4	95.3	93.4	94.7	91.1	96.2	93.1	94.8	94.3	95.6	92.2	94.3	90.3	93.1	93.2	95.0	92.0	94.1
Australia	<5	92.4	95.2	92.9	94.8	92.1	95.8	96.3	98.0	96.3	97.4	96.4	98.6	95.0	98.1	96.7	98.7	93.0	97.4	91.8	96.3	95.1	97.4	93.2	96.5
Austria	<5	91.6	94.8	92.3	94.4	90.9	95.4	94.9	97.2	95.3	96.7	94.4	97.9	96.2	99.1	96.8	98.9	95.5	99.2	91.6	95.5	94.6	96.8	92.6	95.8
Azerbaijan	<5	85.1	90.1	86.9	90.6	82.9	89.7	89.4	95.7	91.3	95.2	86.4	96.1	92.8	94.3	93.5	94.9	92.4	94.1	89.1	92.2	92.6	94.7	90.2	92.5
Bahamas	<5	89.2	92.3	89.5	92.2	89.2	92.7	92.9	95.2	92.8	94.8	93.4	96.0	94.5	97.5	94.9	97.8	94.3	97.1	92.7	95.0	94.2	96.2	94.3	95.8
Bahrain	<5	80.3	90.7	81.4	91.1	79.5	90.5	83.8	93.9	85.7	94.3	82.0	93.9	86.4	94.8	88.8	95.5	83.9	94.2	85.5	93.3	87.3	95.0	90.8	94.8
Bangladesh	<5	32.2	82.7	47.8	85.8	11.3	78.4	47.4	88.7	60.4	90.5	30.2	86.0	25.7	83.2	49.9	89.4	4.0	77.3	50.1	85.0	63.5	90.1	68.3	88.0
Barbados	<5	90.3	92.2	90.3	91.7	90.7	93.0	93.6	95.1	93.2	94.5	94.5	96.1	95.1	97.3	95.2	97.3	95.2	97.3	93.8	94.7	94.8	95.6	95.6	96.1
Belarus	<5	88.2	91.8	90.2	92.4	85.2	90.8	92.1	95.0	93.2	95.0	90.8	95.0	93.0	95.4	94.0	96.5	92.2	94.3	90.4	94.4	94.3	96.1	89.8	94.5
Belgium	<5	88.9	94.6	90.4	94.6	87.3	94.6	93.0	97.8	94.4	97.6	91.8	98.0	90.5	96.9	92.1	96.9	89.2	97.0	89.6	95.4	92.9	97.0	91.4	95.3
Belize	<5	82.0	89.1	84.3	89.4	79.2	89.1	87.4	93.0	88.9	92.7	85.7	93.7	86.7	94.9	89.0	95.6	84.7	94.3	88.0	92.5	91.1	94.4	91.0	93.8
Benin	<5	79.2	85.1	83.6	88.1	73.8	81.1	86.4	90.3	84.4	88.7	87.9	91.8	87.5	90.1	90.8	92.6	84.7	88.2	79.7	85.6	92.0	93.6	68.9	77.8
Bermuda	<5	92.1	94.2	91.9	93.5	92.7	95.2	95.0	96.6	94.6	96.0	95.9	97.6	98.5	100.0	98.5	99.8	98.4	100.0	94.0	95.7	95.4	96.6	95.1	96.5
Bhutan	<5	54.1	83.9	62.6	86.1	43.3	81.1	63.0	89.3	69.9	90.5	54.8	87.8	47.0	85.6	61.8	89.2	34.1	82.4	62.9	85.9	72.9	91.1	76.1	88.3
Bolivia (Plurinational State of)	<5	39.1	76.9	50.6	81.8	25.0	70.7	59.9	86.4	67.4	88.9	51.2	83.1	26.8	72.0	38.5	79.2	18.8	66.5	31.8	79.0	56.4	87.4	54.5	83.8
Bosnia and Herzegovina	<5	90.1	93.8	91.6	94.2	88.7	93.3	93.9	96.3	94.2	96.1	94.3	96.7	93.7	97.7	95.1	98.4	91.9	97.0	93.6	95.5	95.8	97.0	93.5	95.2
Botswana	<5	89.2	88.9	89.4	89.4	88.9	88.3	93.7	92.7	91.5	90.0	95.5	94.7	93.6	93.8	94.0	94.3	93.4	93.5	89.6	88.2	93.1	92.8	90.1	88.6
Brazil	<5	81.3	88.7	83.1	89.8	79.8	87.6	88.2	93.8	89.5	94.3	87.1	93.5	84.1	91.9	85.0	92.5	83.9	91.6	85.0	92.1	89.4	95.0	88.4	92.3
Brunei Darussalam	<5	88.9	92.0	90.5	92.9	87.2	90.8	93.2	95.1	94.2	95.7	92.4	94.3	92.6	95.9	94.7	97.4	90.7	94.3	92.0	94.6	94.9	96.5	92.6	94.7
Bulgaria	<5	85.9	93.2	88.3	93.8	82.9	92.2	91.1	95.7	92.4	96.5	89.9	94.9	87.1	94.6	90.6	95.6	83.7	93.6	90.4	96.0	92.8	96.8	93.0	97.0
Burkina Faso	<5	77.2	81.9	83.4	86.3	69.0	75.6	85.2	88.3	86.2	88.7	84.4	88.2	88.0	89.7	92.5	93.2	83.0	85.7	79.3	84.3	91.7	92.9	70.7	77.0
Burundi	<5	62.7	83.9	65.8	85.6	59.6	82.1	69.7	87.5	69.5	87.7	70.7	87.7	72.3	89.0	78.6	92.4	67.4	86.1	74.8	87.6	80.1	90.7	83.1	90.4
Cabo Verde	<5	86.1	93.1	89.3	93.8	82.4	92.4	91.7	96.5	90.1	95.1	92.8	97.6	89.9	96.0	93.1	97.5	86.7	94.4	82.9	93.6	94.4	96.8	70.5	90.5
Cambodia	<5	64.5	83.8	68.5	85.3	60.1	82.3	72.8	88.7	76.2	89.5	69.5	88.2	78.4	91.1	80.2	92.5	77.6	90.1	78.5	89.2	81.7	91.6	86.6	92.2
Cameroon	<5	80.0	84.0	86.4	88.2	71.2	78.2	86.0	89.4	86.6	88.7	85.5	90.0	87.6	89.7	92.6	93.5	82.4	85.8	83.1	84.1	94.5	94.0	72.2	74.8
Canada	<5	93.0	96.7	92.3	96.1	93.9	97.3	95.8	97.8	95.7	97.5	96.1	98.2	94.2	98.6	95.5	99.6	92.8	97.2	90.8	96.5	92.3	97.2	94.5	97.5
Central African Republic	<5	54.3	75.3	55.3	77.8	53.8	72.6	65.7	82.1	63.3	81.4	68.5	82.3	64.4	80.5	66.6	84.6	64.1	77.9	63.5	79.4	73.8	87.4	75.1	83.3
Chad	<5	73.1	77.0	80.7	83.6	63.4	68.0	82.7	84.8	82.8	84.5	82.5	85.1	83.6	84.7	90.0	90.3	77.2	79.0	71.1	75.2	90.3	91.7	56.4	60.3
Chile	<5	86.8	92.8	87.8	92.9	85.7	92.8	92.8	96.5	93.5	96.4	91.8	96.8	88.9	96.5	90.7	97.2	87.3	95.8	86.5	93.9	89.0	95.6	90.9	94.7
China	<5	77.8	93.7	80.1	93.3	75.1	94.3	85.7	97.0	87.8	96.9	83.4	96.9	69.2	96.5	69.1	96.8	70.9	96.2	87.4	94.8	90.0	95.8	91.8	96.5
Colombia	<5	84.9	90.7	86.2	91.1	83.8	90.4	91.3	94.8	92.1	94.9	91.0	94.7	88.9	94.4	90.4	95.8	87.6	92.9	87.7	93.6	91.0	95.2	90.8	95.0
Comoros	<5	72.4	85.9	76.3	87.5	67.6	84.1	76.3	88.3	77.1	88.3	76.0	88.8	76.5	89.3	82.4	92.2	72.0	87.0	80.5	89.3	85.9	92.4	85.7	91.4
Congo	<5	75.6	89.5	77.6	90.0	73.3	88.9	82.1	92.7	80.7	91.6	83.2	93.6	79.7	92.4	82.8	94.0	77.8	91.2	77.8	91.0	87.4	94.5	81.6	91.9
Cook Islands	<5	89.4	93.0	89.3	92.4	89.9	93.9	92.8	95.5	91.8	94.8	93.9	96.5	88.9	96.8	87.5	96.3	91.0	97.3	90.9	94.4	92.3	95.2	94.3	96.2
Costa Rica	<5	90.6	94.1	91.0	93.6	90.4	94.9	94.6	97.0	94.7	96.7	94.7	97.6	96.6	98.7	97.2	99.1	96.0	98.1	92.7	95.7	94.7	96.5	93.9	96.9
Coted'Ivoire	<5	78.1	83.8	85.1	88.1	68.9	78.2	85.4	89.6	84.5	88.3	85.9	90.7	84.9	88.5	90.1	92.2	80.0	85.1	75.8	81.9	92.8	93.5	56.5	69.4
Croatia	<5	89.1	93.5	90.2	93.4	87.9	93.6	94.2	97.1	94.5	96.7	94.0	97.6	92.9	97.3	95.0	97.8	90.8	96.8	90.9	94.5	93.8	96.1	91.9	94.9
Cuba	<5	89.0	93.1	89.8	93.0	88.1	93.2	92.9	95.6	93.1	95.4	92.9	96.0	95.6	98.7	96.7	99.1	94.2	98.0	90.9	95.3	93.4	96.5	92.5	95.9
Cyprus	<5	88.0	93.8	89.1	93.9	86.9	93.8	94.3	97.2	94.9	96.8	93.6	97.8	86.7	97.8	86.3	97.6	87.5	97.9	87.7	94.9	90.4	96.9	91.0	94.5
Czechia	<5	88.4	92.9	89.6	93.1	87.0	92.8	94.0	96.5	94.5	96.2	93.8	97.0	92.2	97.4	94.2	98.0	90.1	96.8	89.5	94.4	92.8	96.2	90.8	94.5
Democratic People's Republic of Korea	<5	85.2	90.6	86.4	90.8	84.1	90.5	90.1	93.6	90.8	93.8	89.8	93.6	87.2	94.8	88.6	95.5	86.2	94.1	91.1	93.5	93.0	95.1	93.6	94.9

Democratic Republic of the Congo	<5	59.1	86.0	59.1	86.6	59.4	85.5	71.0	90.9	67.3	89.5	74.2	92.0	70.9	91.0	70.5	92.6	72.3	89.9	61.1	87.5	73.4	92.2	73.0	89.1
Denmark	<5	86.0	93.7	87.5	93.6	84.3	94.0	90.8	96.6	92.3	96.3	89.1	97.1	88.1	97.1	88.5	97.1	88.2	97.0	85.8	95.2	89.9	96.6	89.0	95.5
Djibouti	<5	79.7	89.1	83.6	90.2	75.1	87.9	83.6	91.7	85.0	91.5	82.2	92.2	82.2	92.1	88.8	94.6	77.1	90.1	87.4	92.0	91.8	94.7	89.5	93.0
Dominica	<5	86.9	90.0	87.3	90.0	87.1	90.4	91.7	93.6	91.3	93.2	92.8	94.5	93.2	94.3	93.6	94.7	92.9	94.0	90.3	92.9	92.4	94.0	92.3	94.9
Dominican Republic	<5	71.8	86.0	74.9	86.8	68.1	85.3	81.8	91.0	84.1	91.3	79.2	90.9	64.7	88.8	67.7	90.4	63.0	87.5	78.3	88.7	84.6	90.7	84.7	92.4
Ecuador	<5	75.1	86.2	81.4	87.9	67.1	84.3	82.9	92.2	86.9	92.6	77.3	91.7	78.3	87.0	86.6	90.8	71.4	83.8	71.6	90.3	84.0	93.1	78.6	92.6
Egypt	<5	15.3	86.4	26.3	86.8	0.6	86.2	32.7	92.1	42.7	90.7	20.0	92.7	32.1	88.4	52.6	91.0	0.0	85.7	0.0	82.5	0.0	86.0	58.7	89.0
El Salvador	<5	75.7	90.0	79.0	90.4	72.1	89.9	85.3	94.2	87.8	94.3	82.5	94.2	81.2	95.2	82.7	96.1	80.7	94.3	81.2	92.9	86.8	94.8	86.5	94.4
Equatorial Guinea	<5	66.2	88.6	67.0	89.1	65.7	88.1	75.8	91.8	73.7	90.8	77.8	92.6	72.1	92.0	74.5	92.7	71.2	91.7	71.4	89.5	80.9	93.3	79.2	91.5
Eritrea	<5	73.7	86.4	77.9	88.3	68.7	84.1	79.2	89.4	80.5	90.0	77.7	88.9	81.6	90.5	88.4	93.9	76.0	87.7	83.8	90.1	89.1	93.5	86.8	91.4
Estonia	<5	86.9	90.5	89.2	91.2	83.9	89.7	91.9	94.3	92.8	94.2	91.0	94.7	92.8	96.0	94.7	96.7	91.0	95.3	89.3	93.2	93.8	95.4	88.5	93.2
Eswatini	<5	84.6	88.5	86.5	90.0	81.4	86.1	87.7	91.0	86.9	91.3	87.9	90.6	91.2	93.5	92.1	94.2	90.7	93.0	87.2	90.4	91.7	93.7	87.8	90.8
Ethiopia	<5	69.1	85.4	71.3	86.5	67.2	84.4	76.9	90.0	76.8	89.9	77.8	90.6	80.6	90.3	83.5	92.2	79.0	89.0	83.0	89.0	87.1	91.8	86.8	91.1
Fiji	<5	89.3	89.6	90.1	90.0	88.8	89.5	91.6	92.4	91.5	92.6	92.4	92.6	90.1	88.7	91.1	88.8	89.4	89.3	92.1	91.4	94.4	94.2	94.0	93.8
Finland	<5	88.9	95.1	89.1	94.5	89.0	95.8	93.7	97.3	93.8	96.8	93.7	98.0	92.7	98.6	92.3	98.3	93.3	98.9	87.7	95.9	90.6	96.9	90.8	96.3
France	<5	92.8	95.0	93.7	95.1	91.7	94.8	95.4	97.3	96.1	97.3	94.8	97.4	94.4	97.3	94.8	97.1	94.2	97.5	94.3	97.0	96.3	98.2	94.7	96.5
Gabon	<5	83.8	90.8	85.1	91.7	82.6	89.6	88.6	93.6	88.1	93.3	89.1	93.9	88.0	93.9	90.0	95.5	86.7	92.6	86.6	92.5	91.1	95.6	89.4	92.8
Gambia	<5	81.2	87.6	86.5	90.0	74.2	84.3	87.8	92.2	87.3	91.0	88.3	93.3	88.4	92.3	92.7	94.7	84.3	89.8	78.7	86.8	92.9	94.3	67.4	82.1
Georgia	<5	89.7	92.0	89.9	91.8	89.9	92.5	92.7	95.6	93.1	94.9	92.4	96.7	95.3	97.3	95.5	97.4	95.2	97.2	92.2	93.2	93.9	95.2	93.8	93.9
Germany	<5	89.8	94.7	90.6	94.4	88.9	95.1	94.0	97.2	94.8	96.8	93.0	97.9	91.9	97.3	92.6	97.2	91.6	97.4	89.1	95.5	92.4	96.7	91.1	95.8
Ghana	<5	82.4	90.7	88.5	93.3	73.5	86.8	88.2	93.8	87.6	93.4	88.8	94.6	88.3	93.9	93.2	96.5	83.0	91.3	79.0	91.6	94.9	96.7	58.4	84.5
Greece	<5	88.8	93.3	90.2	93.3	87.0	93.4	91.0	96.3	92.9	96.2	88.6	96.5	94.2	96.5	94.6	96.5	94.0	96.5	89.0	94.6	92.1	96.1	91.6	95.2
Greenland	<5	87.8	97.9	83.9	95.9	90.5	100.0	94.9	97.9	95.6	97.9	94.4	98.2	28.3	94.3	18.9	90.8	46.6	98.6	86.9	97.8	96.5	98.3	91.8	99.1
Grenada	<5	87.7	91.7	87.7	91.2	88.1	92.6	91.9	94.4	91.7	93.9	92.7	95.3	92.5	96.4	92.7	96.6	92.7	96.4	89.7	95.0	91.0	95.6	93.6	97.0
Guam	<5	91.5	92.6	92.0	92.9	91.3	92.5	93.7	94.5	93.3	94.1	94.6	95.3	93.8	95.2	93.6	94.6	94.2	95.8	95.2	94.4	96.6	96.2	96.0	95.2
Guatemala	<5	78.4	85.1	80.2	86.8	76.9	83.1	85.4	90.2	87.1	91.7	84.1	88.6	90.6	89.9	91.6	92.3	89.8	87.8	80.6	90.6	84.3	93.2	87.8	93.0
Guinea	<5	65.7	78.9	73.9	84.6	55.4	70.9	77.7	86.2	75.8	85.2	79.0	87.0	79.9	85.7	86.6	90.5	73.2	81.1	59.9	76.2	87.3	91.5	36.6	59.4
Guinea-Bissau	<5	70.2	84.6	80.5	88.3	56.1	79.7	80.1	89.9	80.9	88.8	79.1	90.9	81.0	89.2	89.3	93.4	73.1	85.4	65.0	84.5	90.9	93.8	41.2	76.6
Guyana	<5	79.1	89.3	82.0	90.0	75.5	88.3	85.6	92.9	86.9	92.9	84.6	93.1	81.7	91.6	86.5	93.2	78.0	90.2	81.7	92.4	85.1	93.7	87.8	94.5
Haiti	<5	21.5	57.7	26.0	61.2	17.8	53.7	50.1	74.3	56.5	77.8	45.0	70.7	0.0	41.8	0.0	40.2	7.9	47.4	14.3	59.2	28.0	67.5	53.1	75.3
Honduras	<5	77.3	88.9	79.4	89.7	75.3	88.3	85.7	93.4	87.4	93.9	84.1	93.3	76.9	91.9	79.6	93.9	75.2	90.4	81.0	91.7	85.0	93.9	88.4	94.1
Hungary	<5	88.6	92.6	89.8	92.6	86.9	92.8	93.4	96.1	94.0	95.7	92.8	96.7	93.0	96.9	94.7	97.4	91.4	96.4	90.4	94.4	93.4	95.9	91.5	95.1
Iceland	<5	88.8	93.5	89.8	93.4	87.9	93.7	93.6	96.7	94.4	96.4	93.0	97.1	90.5	97.5	90.8	97.4	90.6	97.6	88.8	94.4	91.9	96.2	91.2	94.7
India	<5	65.9	83.5	70.7	85.6	60.0	80.8	73.4	89.1	77.2	90.6	69.3	87.2	64.2	86.0	72.7	89.5	57.6	83.2	75.2	86.8	80.9	90.8	83.2	89.2
Indonesia	<5	66.6	80.2	70.8	83.5	61.8	75.6	74.1	84.2	77.8	87.7	70.3	79.2	74.6	85.9	75.6	88.1	74.8	84.3	76.7	84.7	81.7	89.2	84.7	88.8
Iran (Islamic Republic of)	<5	80.4	92.7	81.6	92.3	79.1	93.5	83.7	94.9	84.9	94.5	82.8	95.7	81.4	95.9	82.3	96.2	81.6	95.7	76.3	95.6	80.1	96.4	86.4	96.9
Iraq	<5	61.2	85.7	64.8	87.3	57.5	84.1	69.4	87.9	72.3	89.4	66.5	86.6	74.0	91.0	76.0	92.5	73.2	89.3	54.9	87.0	59.3	90.7	76.4	90.6
Ireland	<5	91.3	93.9	92.3	93.9	90.0	94.0	95.3	97.0	95.9	96.7	94.6	97.6	92.7	98.2	94.8	98.4	90.6	97.8	91.5	94.9	94.3	96.6	92.7	94.8
Israel	<5	88.9	94.5	89.6	94.4	88.3	94.9	90.1	97.6	92.0	97.3	87.4	98.0	90.9	97.0	91.5	96.8	90.7	97.2	90.7	95.7	92.6	97.1	93.1	95.8
Italy	<5	88.9	94.2	89.2	94.3	88.8	94.1	93.5	97.4	94.0	97.4	93.0	97.5	90.6	96.9	91.0	97.2	90.5	96.6	89.2	94.9	91.1	96.5	92.8	95.1
Jamaica	<5	84.1	91.1	85.1	91.2	83.5	91.3	89.9	94.5	89.6	94.0	90.9	95.3	91.0	96.9	92.5	97.5	89.9	96.2	81.6	93.8	82.6	95.0	90.1	95.2
Japan	<5	90.7	94.1	91.5	94.1	89.7	94.0	95.0	98.0	95.9	98.2	94.1	97.8	95.9	98.6	96.6	98.8	95.1	98.3	92.3	94.1	94.7	95.9	93.2	94.4
Jordan	<5	69.3	88.7	73.1	88.8	64.9	88.7	68.8	90.7	71.5	90.2	65.1	90.8	87.3	95.2	90.7	96.2	83.7	94.1	66.8	87.4	73.1	90.1	81.2	91.9
Kazakhstan	<5	92.9	90.9	90.9	90.9	95.5	91.3	95.0	94.5	94.7	94.3	95.5	94.9	94.6	94.8	95.0	95.0	94.5	94.8	95.8	93.2	94.7	94.9	99.8	94.5
Kenya	<5	83.5	90.2	84.5	90.7	83.0	90.0	87.4	93.5	86.6	93.0	88.8	94.3	90.4	94.2	91.7	95.4	89.7	93.4	88.5	92.3	91.5	94.4	90.7	93.5
Kiribati	<5	78.1	85.7	80.8	87.3	75.1	84.3	81.9	89.5	80.8	89.7	83.0	89.6	74.7	86.4	78.1	87.3	71.8	86.1	76.2	86.1	83.5	91.2	83.6	90.0
Kuwait	<5	84.4	90.6	86.6	91.8	81.7	89.1	84.9	92.3	88.2	93.5	81.0	90.8	88.8	96.0	90.1	97.5	87.6	94.1	85.7	90.4	88.6	94.0	91.5	92.9
Kyrgyzstan	<5	84.7	89.9	86.0	90.1	83.4	90.0	88.7	94.6	90.0	94.0	86.9	95.6	91.2	94.6	92.1	94.9	90.6	94.5	87.5	92.2	90.4	94.4	90.4	93.1

Lao People's Democratic Republic	<5	35.8	76.7	39.8	79.9	31.4	73.1	51.6	83.6	54.2	85.5	49.2	81.7	56.8	84.8	58.4	87.3	57.6	82.7	52.6	83.7	56.7	87.9	75.7	88.8
Latvia	<5	89.3	91.9	91.1	92.4	86.8	91.2	92.5	95.5	93.9	95.4	90.8	96.0	93.0	95.8	94.8	96.6	91.2	95.1	92.7	94.7	95.4	96.4	92.7	94.8
Lebanon	<5	79.9	90.8	80.3	90.6	79.8	91.3	84.3	93.5	84.3	93.1	84.4	94.1	84.7	95.0	85.8	96.0	84.4	93.9	84.6	93.4	85.0	94.9	91.1	95.0
Lesotho	<5	87.1	88.1	88.5	89.8	85.1	85.3	91.2	91.2	91.2	91.6	91.3	90.5	93.2	93.4	94.0	94.6	92.6	92.3	89.2	90.3	92.7	93.7	89.6	90.5
Liberia	<5	61.3	81.8	71.5	86.0	48.4	76.2	75.4	88.0	73.6	87.1	76.1	88.8	79.1	88.9	84.8	92.5	74.5	85.2	48.2	80.5	84.0	92.0	15.3	72.9
Libya	<5	66.1	68.4	72.5	75.5	57.6	59.0	65.3	65.7	70.8	72.5	59.2	57.7	68.9	77.3	75.7	81.1	60.7	73.9	73.6	64.6	79.7	78.3	83.9	75.5
Lithuania	<5	88.8	92.9	90.1	93.2	87.1	92.4	92.0	95.1	92.9	94.9	91.4	95.6	92.2	95.8	93.9	96.6	90.5	95.0	91.5	95.7	94.3	96.8	92.0	95.9
Luxembourg	<5	87.6	93.7	89.5	93.6	85.6	93.9	92.1	97.4	93.3	97.0	91.0	97.9	90.7	96.9	91.7	96.6	90.1	97.2	87.5	94.5	91.6	96.3	89.9	94.5
Madagascar	<5	69.6	84.1	75.4	87.2	62.7	80.1	76.7	87.4	78.7	88.8	74.6	86.1	74.3	86.2	82.9	92.4	67.7	81.2	79.8	88.4	87.8	92.6	83.2	90.2
Malawi	<5	63.5	86.3	65.8	87.5	61.4	85.0	69.4	89.1	69.6	89.2	70.8	89.6	73.8	90.5	75.9	92.7	73.0	88.7	74.5	89.3	81.3	92.5	82.0	91.2
Malaysia	<5	88.5	93.3	88.8	92.8	88.6	94.2	93.5	96.5	93.5	95.9	93.8	97.2	96.1	98.3	96.4	98.4	95.9	98.1	90.1	94.9	92.6	96.0	92.2	96.0
Maldives	<5	79.8	90.6	80.9	91.0	79.1	90.7	87.4	94.5	87.7	94.6	87.4	94.9	87.7	95.7	88.6	96.3	87.3	95.2	84.9	94.0	86.8	95.3	91.2	95.8
Mali	<5	71.3	81.5	78.4	85.9	61.9	75.9	80.8	88.1	80.8	87.3	80.7	88.9	83.5	87.6	88.7	91.4	78.8	84.8	73.4	82.6	89.3	92.5	61.7	74.2
Malta	<5	88.5	92.8	90.3	92.9	86.8	92.8	93.5	96.3	94.3	96.0	93.0	96.7	93.5	96.5	95.0	96.7	92.1	96.3	87.4	94.3	92.0	96.2	89.4	94.5
Marshall Islands	<5	89.0	89.8	90.0	90.4	88.2	89.4	91.6	93.1	91.7	93.4	92.0	93.3	88.9	90.1	90.1	90.2	88.0	90.5	92.3	92.8	94.3	94.7	94.4	94.8
Mauritania	<5	83.9	89.6	87.9	91.8	78.5	86.6	89.8	93.4	88.9	92.4	90.7	94.4	89.0	93.4	93.1	95.5	84.8	91.4	83.1	90.1	93.6	95.8	73.6	85.1
Mauritius	<5	88.0	90.9	88.1	90.8	88.4	91.3	92.2	94.5	92.0	94.3	92.7	95.0	93.7	96.1	94.3	96.4	93.3	95.8	90.7	93.8	92.3	95.1	93.7	95.5
Mexico	<5	82.7	89.7	84.8	90.7	80.4	88.5	89.4	94.4	91.0	95.3	87.7	93.5	86.0	91.1	88.0	93.1	84.4	89.1	83.7	92.9	88.3	94.7	88.2	94.8
Micronesia (Federated States of)	<5	85.1	91.0	85.7	91.3	84.8	90.8	89.8	93.7	89.5	93.9	90.7	94.1	82.9	92.8	83.4	93.1	83.3	92.7	87.8	93.7	90.6	95.4	91.7	95.0
Monaco	<5	89.0	94.3	88.4	94.0	90.3	94.7	93.5	97.4	93.3	97.0	94.2	98.0	90.0	96.7	86.4	96.0	93.9	97.5	88.1	94.8	88.5	96.0	93.0	95.5
Mongolia	<5	89.3	95.4	90.8	95.3	86.3	95.2	89.1	95.5	91.0	95.4	85.4	95.7	86.2	96.2	87.6	95.7	85.5	96.8	97.0	98.1	97.3	98.5	98.1	98.1
Montenegro	<5	86.9	93.8	87.8	94.1	85.8	93.2	91.8	98.7	92.7	99.4	91.0	98.0	94.0	97.0	93.8	97.2	94.5	97.0	91.9	93.5	94.3	95.6	92.6	93.7
Morocco	<5	53.3	87.0	55.9	87.0	50.7	87.6	54.6	89.6	55.7	89.2	54.0	90.6	65.6	91.5	69.8	92.4	61.9	90.9	56.0	89.2	61.0	91.0	77.1	92.8
Mozambique	<5	71.4	86.6	78.4	88.2	62.0	84.7	76.6	90.4	81.4	90.9	70.7	90.2	82.1	91.6	88.1	94.5	77.4	89.1	78.6	89.5	88.5	93.2	81.2	90.8
Myanmar	<5	45.9	78.2	54.2	81.7	36.1	73.9	59.3	84.0	65.3	86.7	52.7	80.7	58.9	84.8	65.1	87.8	54.1	82.1	59.8	84.3	67.9	88.7	76.1	89.0
Namibia	<5	87.4	90.9	88.1	90.8	86.5	91.0	93.4	94.9	91.2	92.7	94.8	96.7	92.9	95.1	93.4	95.5	92.6	94.8	86.8	91.0	91.9	94.0	86.9	91.6
Nauru	<5	87.2	87.2	87.9	87.7	86.6	87.0	89.0	90.0	88.8	89.7	89.6	90.6	83.3	83.5	82.9	82.5	85.1	86.1	87.5	87.7	91.2	91.3	91.6	91.6
Nepal	<5	48.3	83.8	55.2	86.1	40.1	80.9	59.1	89.7	64.1	90.8	54.2	88.3	46.0	86.9	58.3	91.2	36.1	83.0	61.1	86.7	69.3	91.5	76.1	88.7
Netherlands	<5	91.3	93.8	92.1	93.7	90.3	94.0	93.9	96.6	94.6	96.3	93.2	97.0	91.6	96.8	93.0	97.0	90.4	96.6	92.7	95.0	95.0	96.5	93.8	95.3
New Zealand	<5	88.3	92.6	89.2	92.5	87.7	93.0	94.8	97.1	94.8	96.8	95.0	97.5	92.5	96.7	94.1	97.3	90.8	96.0	86.1	92.8	91.5	94.9	88.3	93.7
Nicaragua	<5	71.0	89.0	77.2	89.6	63.3	88.6	78.8	93.3	84.4	93.6	72.3	93.2	78.0	94.5	80.8	95.3	75.9	93.6	77.7	92.2	84.4	94.3	84.4	93.7
Niger	<5	64.7	80.6	70.8	83.9	57.7	76.7	76.1	87.5	72.5	86.4	78.4	88.9	82.3	88.6	87.2	92.1	77.9	85.2	68.1	83.5	87.1	91.9	56.8	79.0
Nigeria	<5	72.2	81.4	79.7	86.5	62.3	73.2	80.3	87.6	81.4	87.9	79.5	87.5	86.9	89.9	91.3	92.9	82.9	86.8	72.3	76.9	89.3	92.2	65.3	66.6
Niue	<5	91.7	82.9	93.4	86.0	89.7	79.3	91.8	88.7	92.3	88.2	91.6	89.2	90.4	70.2	92.5	71.0	88.2	71.4	93.3	77.2	96.7	89.3	94.7	81.6
North Macedonia	<5	73.6	94.5	77.6	94.8	68.2	94.1	82.4	95.9	85.3	95.6	79.3	96.3	61.7	97.8	63.1	98.6	62.5	96.8	81.0	97.2	85.3	98.1	87.1	97.0
Northern Mariana Islands	<5	90.3	94.7	90.5	94.1	90.5	95.7	93.9	96.3	93.6	95.6	94.8	97.1	95.1	98.7	95.4	98.6	94.9	98.6	93.1	96.5	95.1	97.1	94.2	97.5
Norway	<5	90.1	93.9	90.8	94.0	89.3	94.1	94.5	97.2	94.9	97.3	94.2	97.4	94.2	97.6	96.2	97.7	92.6	97.5	89.8	95.0	93.5	96.6	90.9	95.2
Oman	<5	75.6	91.8	77.0	91.3	74.4	92.6	76.2	92.9	81.1	93.6	70.1	92.6	84.8	97.1	87.6	97.7	81.6	96.2	81.1	93.6	77.1	94.2	91.4	96.3
Pakistan	<5	58.9	72.7	64.9	75.9	51.6	68.9	67.1	79.0	72.2	81.0	61.6	77.3	49.4	65.3	60.5	70.3	40.3	62.0	69.7	76.4	75.3	82.8	80.9	84.0
Palau	<5	86.8	91.5	87.8	91.8	85.9	91.3	91.0	93.8	90.4	93.4	91.8	94.5	84.3	90.8	84.4	90.1	85.2	92.0	87.4	92.7	90.5	94.8	91.6	94.6
Palestine	<5	52.4	84.6	57.9	86.4	45.8	82.4	56.1	85.9	60.5	87.4	51.6	83.9	81.8	94.0	83.1	94.9	81.3	93.1	47.2	85.7	55.6	89.9	71.1	89.8
Panama	<5	89.2	92.8	88.7	91.7	90.1	94.2	92.7	95.3	93.4	95.5	92.0	95.2	93.7	95.8	95.1	96.8	92.1	94.7	92.7	96.3	92.4	95.2	95.5	99.2
Papua New Guinea	<5	75.0	79.5	74.2	79.4	76.5	80.2	80.6	84.9	76.3	81.8	83.8	87.5	70.1	78.4	67.7	76.0	74.3	81.9	75.2	78.8	76.9	83.2	87.2	87.2
Paraguay	<5	83.9	89.8	83.7	90.0	84.6	90.0	91.3	94.4	90.6	94.0	92.5	95.1	83.2	93.5	81.2	93.7	86.2	93.5	88.5	93.2	91.1	95.2	91.7	94.1
Peru	<5	71.4	86.3	76.1	87.5	66.1	85.2	82.3	91.6	85.5	92.0	78.0	91.3	72.9	90.3	76.9	91.8	70.4	89.2	72.2	89.8	81.7	93.0	80.6	91.6
Philippines	<5	74.9	86.0	80.6	87.6	67.1	84.3	79.0	90.8	84.9	91.9	70.9	89.6	83.3	91.3	85.9	92.2	81.3	90.7	80.8	89.2	86.4	92.1	86.2	91.5
Poland	<5	84.8	92.4	87.5	92.8	81.2	91.8	90.7	96.6	92.6	96.6	88.6	96.6	90.5	97.4	93.1	97.8	88.0	96.9	89.0	93.3	92.4	95.5	90.7	93.3
Portugal	<5	81.0	95.2	83.4	94.8	78.6	95.7	86.9	98.9	88.5	98.5	85.6	99.2	80.7	96.9	81.4	96.8	80.9	96.9	74.6	95.0	79.0	96.3	85.3	95.6

Puerto Rico	<5	91.4	94.5	91.3	94.1	91.7	95.3	94.9	96.7	94.4	96.0	95.6	97.6	96.7	99.3	97.2	99.7	96.3	98.7	93.8	96.8	95.1	97.4	95.2	97.5
Qatar	<5	87.0	92.4	87.1	92.2	87.2	92.8	90.4	95.2	89.8	94.7	90.9	95.9	89.3	97.2	93.6	98.4	83.2	95.6	92.0	94.4	93.0	95.8	94.6	95.5
Republic of Korea	<5	89.2	95.3	90.6	95.2	87.7	95.4	93.8	97.6	94.6	97.5	93.1	97.7	89.2	98.6	91.0	99.3	87.8	97.8	93.1	96.4	95.7	97.3	93.9	96.8
Republic of Moldova	<5	83.0	89.6	86.5	90.6	77.6	88.1	85.0	93.6	88.9	94.1	79.1	93.0	87.9	92.7	91.1	93.9	84.7	91.7	90.1	93.1	93.7	95.4	90.5	93.2
Romania	<5	82.9	90.7	86.5	91.1	77.7	90.3	87.2	94.0	90.2	93.9	83.2	94.4	86.4	92.8	88.4	92.6	84.7	93.3	87.0	93.8	91.1	95.6	89.4	94.2
Russian Federation	<5	88.9	91.3	90.4	92.0	86.6	89.8	92.6	95.2	93.4	95.2	91.7	95.0	93.1	95.2	94.9	96.4	91.3	94.0	91.1	93.1	94.2	95.4	91.4	93.0
Rwanda	<5	66.0	87.1	70.0	88.5	61.3	85.7	72.4	90.3	73.8	90.6	71.4	90.4	76.4	92.2	82.1	94.5	71.9	90.4	78.2	90.5	84.1	93.2	84.0	91.9
Saint Kitts and Nevis	<5	86.6	91.3	87.4	91.3	85.7	91.5	90.8	94.5	91.2	94.1	90.7	95.4	89.3	95.3	90.2	95.7	88.8	95.0	89.2	94.0	90.7	95.2	93.3	95.3
Saint Lucia	<5	88.1	91.8	88.6	91.7	87.7	92.3	92.4	94.8	92.3	94.4	92.8	95.6	93.9	96.8	94.1	97.0	94.0	96.8	89.9	94.6	92.3	95.5	92.0	95.9
Saint Vincent and the Grenadines	<5	85.6	91.4	86.4	91.5	84.8	91.4	89.8	94.5	90.5	94.2	89.1	95.1	90.6	96.0	90.3	96.4	91.4	95.6	88.4	94.3	90.3	95.5	92.0	95.4
Samoa	<5	86.2	90.1	87.2	90.5	85.2	90.1	90.3	93.2	90.9	93.3	90.1	93.5	85.4	92.2	85.8	92.3	85.8	92.5	88.5	92.3	91.7	94.5	91.5	93.9
San Marino	<5	89.2	94.5	89.6	94.3	88.8	94.9	93.1	97.5	93.6	97.2	92.9	98.0	92.0	97.8	91.9	97.4	92.3	98.1	88.4	95.4	91.0	96.8	91.6	95.6
Sao Tome and Principe	<5	83.8	92.1	89.2	93.7	76.8	89.8	90.0	95.2	90.6	94.6	89.8	96.0	89.0	95.2	93.5	97.3	84.8	93.2	82.0	93.6	94.0	97.0	68.9	89.4
Saudi Arabia	<5	58.4	90.7	67.2	91.0	47.2	90.8	56.3	93.4	66.5	93.3	44.0	93.9	83.1	96.0	86.7	96.6	78.9	95.3	57.3	93.5	69.2	95.4	74.1	94.5
Senegal	<5	75.8	87.4	82.7	90.3	65.7	83.4	83.3	91.6	81.9	90.6	84.3	92.5	84.7	90.9	89.7	93.9	79.5	87.9	71.3	88.8	91.3	95.0	53.9	83.1
Serbia	<5	84.2	94.0	88.6	94.4	75.6	93.3	89.7	95.7	92.5	95.4	85.6	96.3	82.8	98.1	88.5	98.6	75.7	97.6	87.6	97.0	92.7	98.2	86.7	96.0
Seychelles	<5	90.1	91.1	90.0	90.8	90.6	91.9	93.9	94.7	93.3	94.1	94.9	95.7	95.9	96.6	95.9	96.5	95.9	96.8	93.4	93.5	94.9	94.8	94.8	95.1
Sierra Leone	<5	58.4	77.0	69.3	83.2	43.8	67.9	73.5	85.0	71.0	83.5	74.8	86.1	75.2	83.4	80.8	87.8	70.6	79.4	39.8	71.1	81.6	89.6	0.0	46.4
Singapore	<5	87.3	94.8	88.6	94.7	86.2	94.9	92.7	97.8	93.6	97.6	91.8	98.2	93.9	98.8	94.7	99.0	93.2	98.6	89.3	95.7	92.5	97.1	90.8	95.5
Slovakia	<5	87.7	91.9	89.1	92.2	86.2	91.7	92.2	95.4	93.0	95.3	91.7	95.6	91.9	95.8	93.2	96.4	90.8	95.2	90.6	94.0	93.3	95.6	92.0	94.8
Slovenia	<5	90.4	93.4	90.9	93.4	90.0	93.4	95.6	97.2	95.8	96.8	95.7	97.9	96.9	98.0	97.7	98.4	96.0	97.6	90.5	94.6	93.4	96.3	91.4	94.7
Solomon Islands	<5	88.7	91.0	89.0	91.0	88.9	91.3	91.0	93.2	89.9	92.9	92.7	94.1	90.5	94.4	90.1	94.0	91.5	95.2	91.9	94.4	93.0	95.5	94.4	95.7
Somalia	<5	67.1	81.2	71.7	83.4	61.7	78.7	72.1	85.2	74.3	86.3	69.9	84.5	78.9	88.1	85.2	92.0	74.2	85.1	82.0	87.1	86.9	90.7	86.3	89.4
South Sudan	<5	85.6	90.5	65.6	74.7	49.1	65.4	90.5	93.5	68.7	75.8	61.6	72.8	90.6	94.4	78.8	84.0	65.8	76.9	85.0	91.8	82.4	85.3	80.6	85.1
Spain	<5	58.1	70.5	90.1	94.7	88.5	95.2	65.5	74.2	95.3	98.4	94.9	98.5	71.2	79.8	93.0	96.9	90.9	97.3	73.7	79.4	91.2	96.7	89.6	95.7
Sri Lanka	<5	89.1	94.9	90.1	92.0	89.5	92.7	94.9	98.4	93.0	94.6	93.1	95.6	91.9	97.1	96.8	98.2	96.4	98.1	87.4	95.3	93.9	95.8	94.3	95.7
Sudan	<5	89.7	92.2	0.0	79.5	0.0	74.9	92.9	94.9	0.0	82.6	0.0	77.7	96.6	98.2	50.7	83.8	49.3	80.1	92.1	94.5	25.4	83.3	67.3	85.9
Suriname	<5	0.0	77.2	84.1	88.9	78.3	87.2	0.0	80.1	89.4	92.3	85.1	91.8	45.8	81.7	85.9	92.5	82.6	90.6	26.7	77.8	90.1	93.6	90.2	93.8
Sweden	<5	81.7	88.0	92.7	95.5	92.5	95.3	87.7	92.0	98.1	100.0	97.4	100.0	84.0	91.4	90.7	97.5	95.7	97.3	86.7	91.8	95.4	96.9	93.1	95.5
Switzerland	<5	92.5	95.4	89.2	93.9	86.7	94.0	97.8	100.0	93.6	97.2	92.0	97.9	93.4	97.3	90.0	96.6	89.2	96.9	92.4	95.4	91.3	96.8	90.5	95.0
Syrian Arab Republic	<5	88.0	93.9	62.6	87.5	52.2	84.3	92.8	97.5	63.3	89.0	53.0	84.7	89.4	96.7	81.9	95.9	74.6	93.0	87.7	95.1	67.1	92.5	82.3	92.2
Taiwan (Province of China)	<5	57.7	86.0	92.4	94.5	92.2	95.4	58.5	86.9	95.2	97.0	95.7	97.5	78.2	94.6	98.3	100.0	98.0	99.7	64.6	89.6	96.4	97.0	95.2	96.7
Tajikistan	<5	92.2	94.9	80.1	86.8	70.8	82.6	95.3	97.2	84.6	91.0	72.0	86.4	98.1	99.9	86.1	92.2	83.6	89.9	94.8	96.1	84.4	91.2	82.9	89.1
Thailand	<5	76.2	85.0	86.9	91.2	84.7	91.2	79.7	89.3	91.7	94.4	91.7	94.8	84.5	90.9	90.7	96.7	86.8	95.4	77.5	87.2	92.7	95.4	92.1	95.2
Timor-Leste	<5	85.6	91.1	53.8	80.1	36.4	71.7	91.5	94.5	66.9	85.9	50.9	79.7	88.6	96.1	66.6	86.9	63.9	81.4	90.1	94.0	67.1	88.0	77.3	87.9
Togo	<5	45.3	76.2	84.8	89.7	70.3	82.2	59.5	83.0	85.2	90.2	86.6	92.3	64.4	83.9	91.6	94.4	81.8	87.9	60.6	83.3	91.9	94.4	60.1	78.4
Tokelau	<5	78.5	86.4	87.6	84.4	90.2	84.2	86.0	91.4	91.3	90.6	94.6	92.2	86.5	91.0	82.8	70.5	91.5	79.6	74.6	85.6	92.3	89.4	95.5	88.4
Tonga	<5	88.5	84.2	89.1	91.2	88.2	91.0	92.7	91.1	91.4	93.2	92.6	94.2	86.1	73.6	89.3	92.6	91.8	94.8	92.0	83.7	93.5	95.1	92.5	94.2
Trinidad and Tobago	<5	88.5	90.9	90.6	91.5	89.0	91.9	91.7	93.5	93.2	94.1	93.1	95.1	90.2	93.5	93.4	95.4	91.3	94.1	90.7	93.0	94.9	95.7	95.6	96.1
Tropical Latin America	<5	89.9	91.6	83.1	89.8	80.0	87.7	93.0	94.4	89.5	94.3	87.4	93.5	92.3	94.8	84.9	92.6	84.0	91.6	93.6	94.7	89.4	95.0	88.5	92.4
Tunisia	<5	70.4	90.0	70.8	90.0	70.5	90.2	73.4	92.8	73.1	92.6	74.2	93.3	80.5	95.3	81.6	96.0	80.5	94.7	73.5	92.7	74.9	94.7	86.3	94.3
Turkey	<5	61.3	91.1	63.1	91.1	59.6	91.3	71.2	93.5	71.3	93.3	71.5	93.9	44.8	92.7	53.9	94.0	35.1	91.2	70.3	94.6	72.3	95.8	85.2	96.0
Turkmenistan	<5	84.2	89.9	86.5	90.0	81.7	90.1	86.9	93.4	89.8	93.3	83.4	93.5	90.7	94.0	92.2	94.6	89.5	93.5	86.6	92.3	91.0	94.2	89.7	94.0
Tuvalu	<5	69.3	87.1	73.8	88.0	63.9	86.5	77.7	91.8	78.8	92.1	76.9	92.2	60.5	86.8	64.8	87.0	57.7	87.2	63.4	88.9	77.1	92.4	74.7	91.5
Uganda	<5	71.0	85.7	75.1	87.6	66.0	83.4	74.1	88.0	75.1	88.4	73.4	87.9	77.1	89.2	82.7	92.4	72.7	86.5	82.1	90.1	86.5	93.0	87.0	91.8
Ukraine	<5	89.7	93.3	90.8	93.6	88.1	92.3	94.4	96.5	94.7	96.3	94.1	96.7	96.0	96.8	96.5	97.5	95.5	96.1	90.9	94.7	94.4	96.4	90.1	94.4
United Arab Emirates	<5	86.1	94.5	88.2	94.3	83.8	94.8	89.5	95.9	91.5	95.9	87.2	96.1	90.1	97.8	92.8	98.6	86.7	96.6	87.9	96.4	91.9	97.2	90.4	97.1
United Kingdom	<5	89.1	94.7	89.7	94.6	88.6	94.7	93.7	97.1	94.0	96.9	93.6	97.4	90.4	97.3	91.0	97.5	90.3	97.1	86.6	95.8	90.2	97.0	89.9	96.0

United Republic of Tanzania	<5	73.3	86.2	76.1	87.4	70.3	84.9	78.5	89.3	79.2	89.3	78.3	89.8	81.2	89.3	84.4	92.4	79.1	86.9	83.7	88.7	87.8	92.4	88.0	90.8
United States Virgin Islands	<5	89.7	92.9	90.3	92.6	89.2	93.5	93.5	95.6	93.6	95.2	93.8	96.4	95.0	98.8	95.8	98.9	94.3	98.6	93.0	95.1	94.5	96.3	94.2	95.8
United States of America	<5	94.7	100.0	94.7	100.0	94.7	99.7	97.1	99.2	97.8	99.8	96.3	98.6	92.9	96.4	95.0	98.4	90.7	94.0	91.6	100.0	93.5	100.0	94.5	100.0
Uruguay	<5	84.2	92.8	86.2	93.0	81.6	92.7	91.7	96.2	92.6	95.9	90.6	96.6	82.6	96.8	84.6	97.4	81.6	96.3	84.0	94.4	88.4	96.1	88.0	94.8
Uzbekistan	<5	84.7	89.2	86.7	89.9	82.0	88.4	88.5	94.7	90.9	94.6	84.3	94.7	91.2	92.6	92.3	93.6	90.3	91.8	87.6	91.1	91.5	93.9	90.0	92.0
Vanuatu	<5	85.4	89.3	86.5	89.8	84.6	88.9	89.1	91.9	88.6	91.7	90.3	92.6	81.1	88.6	82.0	88.6	81.3	89.2	87.5	91.4	90.6	93.7	91.1	93.7
Venezuela (Bolivarian Republic of)	<5	83.9	88.1	86.3	89.2	81.1	87.0	89.3	93.1	91.2	93.8	86.9	92.4	90.2	90.6	91.9	92.9	88.9	88.3	88.0	92.0	91.7	94.0	90.8	94.0
Viet Nam	<5	83.3	89.8	84.4	89.4	82.6	90.6	88.3	93.2	88.3	92.9	88.3	93.7	90.2	95.4	91.7	95.9	89.2	95.1	88.9	93.1	91.0	94.2	93.0	95.8
Yemen	<5	36.0	78.5	35.4	78.6	37.9	79.1	38.3	82.3	37.7	81.8	40.9	83.8	63.3	83.9	65.5	84.6	62.9	83.9	49.1	79.8	47.2	82.3	77.3	88.1
Zambia	<5	73.9	87.9	77.2	89.1	70.2	86.6	79.2	90.7	79.6	90.5	79.3	91.3	83.1	92.5	86.2	94.1	81.0	91.2	85.0	91.3	89.6	94.0	87.5	92.3
Zimbabwe	<5	87.5	86.9	89.4	88.2	84.5	85.0	89.0	88.4	89.9	88.4	88.8	89.1	93.7	91.6	94.0	91.3	93.7	92.3	90.9	89.9	94.1	93.0	90.3	90.5
Afghanistan	5 to 9	77.8	86.7	79.5	89.4	77.1	85.0	77.5	86.6	79.5	88.6	75.3	84.4	77.2	81.3	82.5	87.1	79.0	82.2	86.9	86.4	86.6	89.6	84.2	79.0
Albania	5 to 9	88.6	97.1	87.6	96.7	89.1	97.0	91.5	97.6	91.1	96.9	90.6	97.4	84.8	95.6	81.8	95.7	88.4	96.1	95.8	97.7	95.3	96.7	95.2	95.2
Algeria	5 to 9	75.0	94.9	70.8	93.3	78.5	95.8	75.2	94.8	70.6	92.4	77.9	95.6	76.4	92.7	72.5	91.9	82.7	94.8	76.1	92.5	75.9	91.7	74.2	90.8
American Samoa	5 to 9	95.9	97.0	96.0	97.2	96.3	97.2	97.8	98.5	93.1	95.3	99.9	99.8	85.9	88.3	94.1	95.1	85.4	88.0	94.9	96.3	97.7	97.5	93.5	96.0
Andorra	5 to 9	95.5	99.4	92.6	98.6	97.5	99.8	96.7	99.5	93.9	97.9	98.1	99.8	85.3	97.1	72.4	95.6	93.5	98.2	94.7	98.9	93.5	98.2	92.3	96.7
Angola	5 to 9	82.9	94.4	80.5	93.8	84.7	94.5	83.2	94.1	79.6	92.2	85.3	94.6	78.8	90.7	76.7	91.1	83.3	92.0	89.0	94.7	90.7	95.5	83.3	90.2
Antigua and Barbuda	5 to 9	97.4	99.0	97.0	99.0	97.9	99.1	98.7	99.6	97.2	98.5	99.0	99.5	91.4	95.3	91.8	97.1	92.8	95.5	98.3	99.1	97.7	98.7	98.8	99.5
Argentina	5 to 9	92.0	97.6	91.1	97.1	92.7	97.6	96.5	98.7	96.1	97.4	96.1	98.7	77.4	92.5	71.8	91.5	83.2	93.7	89.6	97.0	89.0	96.5	86.1	94.4
Armenia	5 to 9	98.0	99.8	97.3	99.0	97.6	99.6	98.6	99.6	96.9	97.6	98.4	99.8	97.6	99.4	95.8	98.9	98.3	99.3	91.4	98.0	92.3	96.9	83.8	94.3
Australia	5 to 9	96.9	99.3	95.9	98.9	97.5	99.5	97.8	99.5	96.2	98.3	98.1	99.6	92.4	97.6	91.4	97.9	94.1	98.0	95.1	98.6	95.1	98.6	93.2	97.3
Austria	5 to 9	97.0	99.6	97.0	99.1	96.7	99.8	97.8	99.6	96.6	98.1	97.3	99.7	90.8	97.5	90.6	97.6	92.3	97.7	94.9	99.1	95.9	98.5	89.1	96.9
Azerbaijan	5 to 9	96.4	98.2	94.9	97.0	96.6	98.4	95.6	97.7	94.1	96.1	95.1	97.5	96.1	98.1	89.7	93.9	99.5	99.8	95.5	97.0	93.9	95.5	91.7	93.6
Bahamas	5 to 9	97.4	99.1	98.1	99.3	96.5	98.9	98.3	99.5	97.9	98.6	97.4	99.2	92.4	95.7	94.9	98.0	92.4	95.7	98.5	99.4	98.6	99.4	97.8	99.3
Bahrain	5 to 9	91.3	95.7	93.9	95.7	89.7	95.6	91.9	96.3	93.3	95.4	89.5	95.8	87.1	93.8	92.3	94.7	87.7	94.8	93.6	95.0	95.1	95.0	91.3	92.8
Bangladesh	5 to 9	45.7	78.6	47.1	78.5	43.4	77.2	59.3	83.7	61.7	83.0	54.5	82.1	9.1	56.7	18.2	57.3	18.4	62.4	61.9	82.8	66.3	84.8	50.3	73.6
Barbados	5 to 9	97.2	99.1	95.8	99.1	98.5	99.2	98.6	99.7	96.7	98.6	99.3	99.6	91.8	95.3	89.3	96.5	94.8	96.0	96.4	99.3	95.2	99.1	98.9	99.5
Belarus	5 to 9	97.3	99.6	96.0	98.9	97.4	99.5	97.2	99.5	95.0	97.5	97.6	99.7	93.7	98.7	90.2	98.3	96.0	98.7	95.4	98.2	94.6	97.1	90.2	95.2
Belgium	5 to 9	94.0	98.8	92.4	98.5	94.8	98.7	94.7	99.1	92.5	98.1	95.3	98.9	83.7	94.5	78.7	94.6	88.4	95.1	92.7	98.6	93.0	98.3	87.8	95.8
Belize	5 to 9	92.5	99.1	94.8	99.5	90.1	98.4	94.4	99.3	96.0	98.3	91.1	98.8	86.2	96.2	87.8	98.9	87.2	95.5	96.1	99.6	96.6	99.5	94.1	98.5
Benin	5 to 9	93.6	94.9	96.6	97.0	92.3	93.8	95.6	96.6	96.0	96.3	94.5	95.8	94.4	94.4	96.5	96.0	95.1	95.3	91.0	91.4	99.0	98.7	73.6	73.5
Bermuda	5 to 9	99.2	99.6	99.2	99.9	99.2	99.6	99.7	100.0	98.5	99.0	99.7	99.8	95.4	96.4	97.8	99.6	95.5	96.2	99.2	99.8	99.1	99.8	99.1	99.7
Bhutan	5 to 9	90.7	94.9	89.2	95.0	91.3	93.8	92.4	95.8	90.0	94.4	93.0	95.2	80.9	87.5	79.9	89.1	83.9	88.1	91.9	94.4	91.3	95.0	87.9	87.3
Bolivia (Plurinational State of)	5 to 9	65.0	87.1	59.1	87.0	69.7	86.7	78.2	92.4	74.4	91.6	80.7	91.7	30.1	68.5	8.8	63.4	51.1	75.8	72.6	91.1	75.8	92.6	64.9	84.9
Bosnia and Herzegovina	5 to 9	95.6	98.7	96.2	98.4	94.8	98.7	97.8	99.2	97.0	97.9	97.1	98.9	86.0	96.0	88.5	96.3	86.6	96.5	94.7	97.2	95.2	96.7	89.1	93.2
Botswana	5 to 9	94.1	95.8	94.4	95.9	92.7	95.0	91.2	94.2	90.3	93.2	90.6	93.7	94.9	95.2	95.4	95.9	95.6	95.9	95.8	95.7	95.6	95.7	91.9	91.4
Brazil	5 to 9	91.8	95.3	90.7	94.4	91.9	95.5	94.9	97.1	93.5	95.3	94.6	97.2	80.0	87.1	73.2	83.0	85.8	90.4	93.0	98.0	93.8	97.7	87.3	94.2
Brunei Darussalam	5 to 9	92.8	98.5	95.0	98.0	90.6	98.8	95.3	99.0	95.9	97.7	93.2	99.0	82.9	95.8	88.4	95.6	82.3	96.7	93.8	97.7	95.8	97.4	87.5	95.9
Bulgaria	5 to 9	89.9	96.7	89.1	96.8	90.7	96.4	93.0	97.6	92.6	97.1	92.9	97.0	86.8	93.1	84.9	93.1	89.7	94.1	90.3	96.0	91.2	96.0	86.4	93.3
Burkina Faso	5 to 9	94.0	94.8	97.8	97.7	91.6	92.9	95.5	96.3	97.3	97.7	93.6	95.0	94.8	94.5	98.2	97.7	94.9	94.8	93.4	92.9	99.1	99.0	79.0	76.8
Burundi	5 to 9	82.1	93.7	80.5	92.0	83.1	94.9	82.5	93.1	79.9	90.2	83.8	94.8	77.6	90.3	79.5	90.5	80.8	92.0	90.0	95.5	89.7	94.7	86.5	92.6
Cabo Verde	5 to 9	96.0	96.6	97.5	98.5	95.8	95.5	98.0	98.2	97.4	97.6	97.4	97.6	95.0	95.0	97.2	98.0	95.5	95.3	92.8	91.3	99.0	98.6	78.3	72.7
Cambodia	5 to 9	85.4	94.5	83.9	94.4	86.3	94.3	89.8	96.2	88.8	95.3	89.8	95.8	73.4	88.9	66.4	87.6	81.1	91.1	90.8	95.3	91.1	95.1	86.9	92.0
Cameroon	5 to 9	89.9	91.8	96.1	96.0	86.0	89.1	92.6	94.6	95.3	94.9	90.3	93.5	90.2	89.7	96.7	95.3	90.0	89.9	88.8	86.7	99.1	98.1	66.6	58.5
Canada	5 to 9	97.1	98.7	96.8	98.9	97.5	98.8	98.5	99.4	97.4	98.5	98.4	99.2	88.1	94.5	86.5	95.8	91.0	94.9	95.1	97.8	95.7	98.4	95.1	98.5
Central African Republic	5 to 9	80.6	88.4	78.9	88.7	81.7	88.0	80.7	88.5	77.7	87.4	82.5	88.8	75.1	81.0	76.1	85.4	79.2	82.7	88.7	92.1	90.3	93.9	82.0	85.4
Chad	5 to 9	89.1	87.7	95.2	93.8	85.3	83.7	91.9	91.5	94.6	92.9	89.8	90.3	88.7	85.6	95.4	92.9	88.8	86.1	86.1	79.9	98.3	97.5	58.2	38.9
Chile	5 to 9	92.9	98.4	92.1	98.3	93.1	98.1	96.5	99.3	95.5	98.1	96.1	99.1	82.8	93.7	80.5	94.3	85.9	94.0	85.8	96.9	86.4	96.9	81.0	93.2

China	5 to 9	88.5	97.0	89.4	97.0	87.4	96.4	93.6	97.3	94.3	96.7	92.0	96.4	67.4	94.3	61.0	94.0	75.5	95.0	92.1	95.9	93.5	95.7	88.2	91.7
Colombia	5 to 9	91.5	95.6	90.1	95.3	92.5	95.5	96.6	98.7	95.6	97.6	96.4	98.4	75.3	84.1	68.6	81.9	82.3	87.4	89.6	97.4	90.0	97.1	85.6	94.5
Comoros	5 to 9	88.7	95.3	88.4	94.2	89.0	95.9	88.5	94.9	86.4	92.4	89.4	96.1	86.3	92.1	87.7	92.2	88.2	93.5	92.7	95.9	94.0	96.1	88.0	92.4
Congo	5 to 9	90.1	95.9	90.8	95.8	89.3	95.6	90.2	95.7	89.3	94.2	90.0	95.9	86.0	92.4	88.9	93.8	87.1	93.0	92.3	95.6	94.9	96.6	84.9	90.8
Cook Islands	5 to 9	90.8	96.6	89.8	95.7	92.3	97.6	94.5	97.8	85.2	93.5	99.9	99.8	73.4	88.8	82.7	93.3	74.8	89.8	92.2	96.0	96.3	96.8	89.3	96.5
Costa Rica	5 to 9	98.3	99.5	97.6	99.3	98.5	99.3	99.2	99.8	97.8	98.7	99.2	99.6	94.4	97.0	93.4	98.0	95.6	96.8	97.6	99.8	96.7	98.9	95.5	98.1
Coted'Ivoire	5 to 9	90.4	91.4	96.4	96.2	87.1	88.5	93.7	94.9	95.6	95.1	92.2	94.0	90.3	89.0	96.0	94.8	90.4	89.4	83.5	81.4	99.2	98.1	48.8	40.3
Croatia	5 to 9	95.5	99.1	95.6	98.6	95.3	99.3	97.6	99.6	98.0	98.6	96.7	99.4	88.8	96.9	86.3	96.1	91.7	97.9	94.8	97.9	94.4	96.9	90.5	95.3
Cuba	5 to 9	97.1	98.8	97.3	99.4	96.9	98.5	98.4	99.5	97.3	98.8	98.0	99.1	91.2	94.7	92.9	98.1	92.3	94.6	97.5	99.1	97.2	99.3	96.5	98.6
Cyprus	5 to 9	94.3	99.2	93.8	98.9	94.9	99.3	97.6	99.5	97.8	98.2	97.2	99.5	81.2	96.8	74.6	97.1	87.1	97.1	94.2	98.7	94.0	98.4	90.4	96.2
Czechia	5 to 9	96.8	99.3	96.6	98.8	96.2	99.2	98.2	99.6	97.1	98.2	97.7	99.5	91.1	97.6	90.7	97.3	92.5	98.1	94.5	97.8	94.5	97.0	89.3	94.9
Democratic People's Republic of Korea	5 to 9	94.2	97.6	94.0	97.5	94.8	97.4	96.6	98.1	96.3	97.1	96.4	97.9	85.5	94.5	83.8	95.1	89.3	95.1	94.4	96.6	95.4	96.6	94.7	94.7
Democratic Republic of the Congo	5 to 9	84.7	93.8	80.2	92.7	88.1	94.3	85.6	93.3	79.7	90.6	89.3	94.5	81.5	90.7	78.4	91.2	85.9	92.1	89.0	95.4	89.7	95.5	84.5	91.0
Denmark	5 to 9	92.8	98.8	91.2	98.7	93.6	98.6	94.7	99.3	93.3	98.0	94.5	99.1	77.3	94.4	66.1	95.6	86.1	94.7	91.2	98.7	91.9	98.4	85.3	96.2
Djibouti	5 to 9	91.9	96.9	92.1	97.0	91.8	96.6	91.7	96.7	90.4	95.4	92.0	96.7	89.0	93.4	91.3	95.8	90.2	93.8	95.4	97.2	96.2	97.6	91.7	93.2
Dominica	5 to 9	97.6	97.0	97.5	97.5	97.5	96.4	98.5	98.3	97.1	97.4	98.4	97.5	92.3	91.1	93.6	93.1	93.0	91.5	98.4	97.7	97.7	97.5	97.8	97.2
Dominican Republic	5 to 9	86.8	93.6	89.2	95.2	84.3	91.6	91.2	95.6	93.6	96.1	87.0	93.1	70.2	83.4	69.3	85.2	74.6	84.6	92.4	95.7	93.4	96.2	90.8	93.4
Ecuador	5 to 9	84.7	92.8	85.0	92.5	84.0	92.6	88.8	96.7	88.8	95.2	87.1	96.7	75.6	79.3	71.2	77.0	81.1	83.5	81.8	95.3	86.5	95.7	71.0	91.3
Egypt	5 to 9	0.0	87.5	0.0	88.1	0.0	87.1	0.0	87.8	0.0	88.2	0.0	86.7	23.1	85.8	24.9	88.2	29.1	86.8	0.0	76.3	0.0	78.9	0.0	70.1
El Salvador	5 to 9	84.4	95.6	82.5	95.3	85.9	95.4	92.6	97.8	92.3	96.9	92.1	97.4	66.1	87.9	54.5	86.1	76.6	90.4	81.8	95.8	84.1	95.9	75.2	91.9
Equatorial Guinea	5 to 9	83.8	95.2	82.2	94.2	84.9	95.8	84.2	95.2	80.9	92.6	85.9	96.0	78.3	93.3	79.5	92.1	81.8	94.9	89.9	93.8	91.6	94.7	83.7	89.5
Eritrea	5 to 9	88.4	95.1	89.7	95.3	87.3	94.7	88.1	94.6	87.7	93.4	87.5	94.7	85.6	91.8	90.7	94.4	86.2	92.2	95.1	96.7	95.4	97.0	90.5	92.4
Estonia	5 to 9	97.5	100.0	96.4	99.1	97.5	100.0	97.6	99.7	95.4	97.5	97.9	100.0	94.5	100.0	93.9	99.5	95.0	99.9	95.8	98.4	94.8	97.2	90.5	95.2
Eswatini	5 to 9	91.2	92.5	92.9	94.1	88.0	90.0	87.4	89.6	88.6	90.7	84.6	87.5	93.1	92.5	94.3	94.1	93.7	93.3	94.9	94.0	94.9	94.4	90.3	88.5
Ethiopia	5 to 9	91.1	95.8	91.0	94.8	90.8	96.0	91.6	95.3	90.4	93.5	91.7	95.9	90.5	94.9	91.9	94.7	91.3	95.5	97.2	97.3	95.9	96.4	92.9	93.7
Fiji	5 to 9	92.6	91.1	91.9	90.1	93.6	92.3	95.4	94.3	88.3	86.1	100.0	99.9	73.1	68.9	83.6	80.1	74.1	69.6	94.0	92.5	96.9	96.2	91.0	88.3
Finland	5 to 9	93.4	98.5	92.1	98.0	94.2	98.8	96.2	99.3	94.9	97.8	96.1	99.3	78.6	92.3	68.7	91.0	86.6	94.2	89.5	98.5	91.1	98.1	83.8	96.5
France	5 to 9	97.0	98.8	96.7	98.5	97.1	99.1	97.8	99.3	96.8	98.3	97.5	99.4	91.6	95.8	89.6	95.1	93.8	96.6	96.2	98.5	96.7	98.6	92.8	96.5
Gabon	5 to 9	93.2	96.9	92.6	97.0	93.5	96.4	93.4	96.8	91.3	95.7	94.0	96.5	91.7	94.8	91.9	96.1	92.8	94.9	94.6	96.3	95.3	97.1	91.0	92.0
Gambia	5 to 9	91.6	93.1	96.5	96.6	88.5	90.4	93.9	95.1	95.4	95.2	92.2	94.3	91.4	89.7	96.3	95.6	91.6	89.8	86.9	87.2	98.7	97.7	60.9	60.9
Georgia	5 to 9	97.4	99.8	95.5	99.0	98.2	99.7	97.6	99.7	95.1	97.6	98.2	99.9	93.9	98.6	88.8	98.4	97.1	98.7	95.4	98.3	93.5	97.0	93.4	94.9
Germany	5 to 9	93.9	99.2	93.0	98.9	94.2	99.2	95.1	99.3	94.0	98.0	94.6	99.2	80.2	95.6	71.5	95.7	87.2	96.1	90.5	98.8	91.7	98.4	84.5	96.4
Ghana	5 to 9	90.2	96.5	96.4	98.8	86.2	95.4	93.7	98.1	95.5	97.7	92.2	97.5	89.2	94.5	96.0	97.8	89.1	95.0	81.7	92.2	99.2	99.7	41.6	74.1
Greece	5 to 9	92.5	98.2	90.5	97.8	93.7	98.3	92.4	98.3	90.5	97.2	92.9	98.2	89.3	95.8	83.1	95.9	93.5	96.2	90.4	98.1	90.9	97.6	85.1	95.5
Greenland	5 to 9	89.5	96.4	92.4	95.7	89.0	97.6	98.8	99.7	100.0	99.6	97.4	99.3	34.7	72.8	28.4	57.9	46.3	86.3	77.4	96.2	96.7	98.6	70.9	98.0
Grenada	5 to 9	95.2	98.6	93.7	99.1	96.4	98.1	97.3	99.2	95.5	98.3	97.8	98.6	87.0	94.8	84.1	97.1	90.6	94.8	94.1	98.9	92.3	99.1	97.0	98.8
Guam	5 to 9	94.3	98.8	96.1	99.4	93.5	98.6	98.1	99.7	93.8	97.7	99.9	99.9	80.6	92.2	92.3	98.9	79.9	91.5	92.6	97.1	97.6	98.3	88.1	97.6
Guatemala	5 to 9	82.0	94.7	78.9	93.5	84.2	95.2	87.1	97.1	85.9	95.8	87.1	97.0	84.7	86.9	79.2	82.3	89.7	90.8	72.6	95.5	74.0	94.8	67.5	92.9
Guinea	5 to 9	84.4	89.4	92.9	94.7	79.6	86.0	89.4	93.3	93.2	93.9	86.8	92.3	86.2	88.1	93.8	93.8	86.5	88.7	77.2	79.5	97.5	97.7	35.0	35.0
Guinea-Bissau	5 to 9	84.5	90.5	94.1	96.5	78.4	86.1	89.3	93.7	93.6	95.5	86.6	92.2	83.6	85.9	93.9	95.0	83.7	85.8	76.7	82.6	98.1	98.2	30.3	43.1
Guyana	5 to 9	86.8	95.2	87.2	96.1	86.5	94.3	90.5	97.1	90.9	96.8	89.4	96.2	73.9	85.7	74.1	88.5	77.8	86.5	89.1	96.4	89.9	96.8	89.9	96.1
Haiti	5 to 9	64.5	71.7	58.6	68.8	69.7	74.6	78.7	83.3	79.3	84.8	77.1	81.5	25.1	33.6	0.0	10.6	49.9	56.1	71.6	79.3	64.9	76.9	82.9	85.5
Honduras	5 to 9	71.0	94.7	63.9	95.4	76.6	94.0	83.5	97.4	80.8	96.8	84.9	96.5	41.2	84.3	16.4	85.3	62.1	86.3	68.5	96.0	68.8	96.6	66.3	92.6
Hungary	5 to 9	96.8	99.4	96.4	99.0	96.5	99.1	97.7	99.5	96.5	98.2	97.4	99.3	93.7	98.5	91.6	98.6	95.5	98.4	95.0	97.9	95.0	97.2	90.5	95.0
Iceland	5 to 9	91.8	99.9	87.8	99.4	94.7	100.0	95.0	99.8	92.2	98.3	96.4	99.9	74.2	98.4	54.7	99.0	87.2	98.3	90.0	99.3	88.9	98.7	87.4	97.1
India	5 to 9	87.3	97.8	84.7	96.8	88.4	97.5	89.1	97.6	86.0	95.5	90.4	97.8	75.9	94.7	72.8	94.0	80.6	95.3	90.2	97.9	88.8	96.3	85.1	93.1
Indonesia	5 to 9	84.6	90.5	82.0	91.1	86.3	88.8	88.8	92.0	87.0	92.2	89.3	90.1	67.2	80.8	51.3	77.1	79.5	84.9	89.1	91.9	89.8	93.2	83.5	84.4
Iran (Islamic Republic of)	5 to 9	88.2	96.2	87.8	95.9	88.1	95.9	89.6	96.3	89.3	95.0	88.8	96.0	83.1	95.7	79.0	95.8	87.4	96.2	73.3	90.0	77.5	91.0	63.9	84.2

Iraq	5 to 9	58.9	92.6	55.4	92.9	62.6	92.3	64.5	91.9	61.5	91.6	66.3	91.1	63.2	91.3	54.6	92.0	73.5	92.7	50.2	89.9	53.5	91.2	42.4	85.3
Ireland	5 to 9	95.5	99.4	95.0	99.3	95.5	99.2	97.8	99.7	96.4	98.3	97.7	99.6	81.5	96.0	77.7	97.9	86.2	95.6	94.0	99.1	94.7	98.6	88.5	96.3
Israel	5 to 9	94.5	99.2	92.6	98.6	95.9	99.4	93.4	99.1	91.8	97.8	94.1	99.1	88.6	97.1	84.1	96.5	92.1	97.4	94.5	99.0	93.6	98.4	93.5	96.9
Italy	5 to 9	95.1	99.1	93.8	98.1	95.6	99.4	96.6	99.2	95.1	97.5	96.5	99.4	85.1	96.0	76.0	93.3	91.3	97.6	92.6	98.9	92.0	97.7	88.8	96.1
Jamaica	5 to 9	92.0	97.9	90.3	98.7	93.5	97.2	95.2	98.9	93.2	98.4	95.5	98.1	83.0	92.2	81.3	96.2	87.9	92.6	91.1	98.5	89.0	98.6	93.3	97.6
Japan	5 to 9	96.6	98.5	95.9	97.9	96.6	98.4	97.9	99.0	96.7	97.8	97.8	99.0	92.3	95.0	91.1	94.9	93.6	95.6	96.1	98.4	95.5	97.3	92.2	95.2
Jordan	5 to 9	67.6	87.6	66.9	89.2	68.7	86.3	60.1	84.0	60.4	86.3	59.6	81.2	86.2	93.4	87.1	95.1	88.3	94.3	64.3	81.0	68.0	84.6	56.7	73.7
Kazakhstan	5 to 9	98.1	99.5	97.4	98.7	98.1	99.3	98.8	99.4	97.6	97.6	98.6	99.5	95.7	98.3	92.2	97.8	97.7	98.4	95.0	97.7	94.7	96.8	93.1	94.6
Kenya	5 to 9	97.3	98.5	96.3	97.9	97.5	98.5	96.7	98.1	94.6	96.4	97.3	98.3	97.1	97.8	96.6	97.9	97.5	97.8	98.1	98.6	97.1	97.8	94.6	95.1
Kiribati	5 to 9	89.1	88.9	92.7	93.3	86.9	86.0	95.1	95.6	85.6	88.6	100.0	99.9	57.0	60.2	89.8	90.1	52.1	59.3	89.6	88.9	97.4	97.0	80.3	77.5
Kuwait	5 to 9	84.0	91.7	86.3	94.4	82.4	89.5	82.8	90.8	86.3	93.1	78.9	87.8	80.9	92.8	78.7	96.4	85.7	92.6	81.2	86.0	84.6	90.3	75.0	77.9
Kyrgyzstan	5 to 9	95.8	99.1	93.8	98.2	96.5	99.1	96.7	99.4	94.6	97.4	96.9	99.5	90.9	96.7	83.6	94.6	95.6	97.8	90.3	97.0	89.0	96.0	86.5	92.9
Lao People's Democratic Republic	5 to 9	69.7	88.1	68.7	89.2	70.7	87.0	80.1	92.3	79.6	92.8	79.8	90.8	40.5	75.1	27.0	72.7	56.1	79.9	77.0	89.2	81.3	92.0	67.3	81.8
Latvia	5 to 9	96.3	99.9	95.5	99.1	96.0	99.9	95.8	99.6	93.8	97.7	95.9	99.9	93.8	100.0	94.3	99.2	93.8	100.0	94.8	98.3	94.5	97.3	90.7	95.5
Lebanon	5 to 9	89.7	96.8	89.7	97.0	89.9	96.4	89.9	96.4	89.2	95.7	89.3	95.8	86.3	93.5	87.1	95.5	88.7	94.1	93.1	97.1	92.3	96.7	91.8	94.9
Lesotho	5 to 9	94.0	89.4	94.1	92.0	92.8	85.2	91.1	85.6	90.1	88.2	90.6	81.8	94.8	90.3	95.5	92.4	95.5	91.1	96.6	93.1	95.7	93.5	93.3	87.3
Liberia	5 to 9	83.5	90.2	93.1	94.5	77.9	86.8	89.1	93.3	93.3	93.1	86.2	92.5	86.0	86.7	93.5	93.4	86.4	86.8	72.1	82.3	97.4	96.7	15.0	44.6
Libya	5 to 9	81.2	81.6	81.3	83.3	82.1	80.6	82.1	80.5	81.8	82.4	82.0	78.2	79.2	76.8	78.8	76.0	83.5	81.3	81.3	72.4	85.3	80.5	79.7	59.7
Lithuania	5 to 9	98.5	99.8	97.4	99.0	98.6	99.7	98.1	99.6	95.7	97.6	98.8	99.8	95.5	99.5	93.9	98.8	96.5	99.6	97.0	98.2	95.9	97.3	93.5	95.7
Luxembourg	5 to 9	91.4	99.3	86.2	99.5	95.1	98.8	93.7	99.4	89.8	98.6	96.1	98.8	81.7	96.9	66.2	99.4	91.3	96.1	86.6	98.9	83.5	98.7	86.7	96.0
Madagascar	5 to 9	82.6	92.1	84.4	92.6	81.4	91.4	84.1	92.0	84.0	91.2	83.6	91.8	79.4	85.7	83.7	89.4	81.6	86.8	90.3	94.7	93.2	95.9	82.6	89.2
Malawi	5 to 9	88.9	95.8	87.1	94.8	90.1	96.3	88.6	95.1	86.1	92.8	90.0	96.1	86.2	92.4	85.5	92.8	88.9	93.5	93.0	96.4	93.7	96.3	88.7	92.5
Malaysia	5 to 9	93.5	98.0	92.6	97.3	94.1	98.2	95.7	98.6	94.5	97.2	95.7	98.6	90.7	96.1	88.7	96.0	93.2	96.9	93.9	96.9	93.6	96.4	90.3	95.0
Maldives	5 to 9	85.0	96.8	78.7	96.6	89.2	96.9	91.4	98.1	86.7	97.0	93.6	97.7	72.9	94.9	59.6	94.1	83.8	96.0	86.0	95.7	85.0	95.6	85.1	93.1
Mali	5 to 9	89.2	92.0	94.9	96.2	85.7	89.8	91.6	94.1	94.7	96.4	89.4	92.8	90.5	91.5	95.4	95.0	91.1	92.5	88.9	88.7	98.3	98.5	67.6	66.0
Malta	5 to 9	92.0	97.2	90.3	97.3	93.0	96.7	94.2	97.8	92.4	96.9	94.4	97.1	87.6	93.4	82.8	94.4	91.5	93.7	87.2	97.1	87.9	97.2	81.6	93.4
Marshall Islands	5 to 9	94.1	91.9	94.9	93.5	94.1	91.0	97.0	96.3	91.7	90.5	99.9	99.9	78.3	71.3	90.8	88.2	77.9	70.4	95.2	93.8	97.7	97.0	94.5	90.6
Mauritania	5 to 9	92.4	95.5	96.5	97.8	89.7	93.9	94.6	97.0	95.0	96.4	93.5	96.4	91.2	93.2	96.3	97.1	91.3	93.4	88.0	91.3	98.8	98.6	62.4	72.4
Mauritius	5 to 9	96.1	98.0	95.3	98.0	96.2	97.6	97.5	98.9	95.8	97.6	97.5	98.6	92.0	94.9	90.5	95.3	93.7	95.3	93.0	96.3	92.8	96.2	90.6	93.7
Mexico	5 to 9	93.3	96.7	92.5	96.2	93.4	96.6	96.2	98.5	95.4	97.7	95.8	98.2	84.2	89.0	79.9	87.0	88.1	91.1	89.4	97.4	90.1	96.6	84.2	94.3
Micronesia (Federated States of)	5 to 9	90.7	94.3	92.2	95.1	90.1	93.8	95.5	97.4	87.7	92.9	100.0	99.9	65.7	78.2	86.4	91.0	63.5	77.0	93.3	95.1	97.1	97.2	89.9	93.1
Monaco	5 to 9	94.8	98.2	92.8	97.0	96.1	98.9	96.2	98.7	94.5	97.1	96.4	99.0	85.7	93.9	74.0	89.5	93.0	96.9	92.5	97.1	92.0	96.2	89.5	95.4
Mongolia	5 to 9	97.5	99.1	97.9	98.9	97.7	98.9	98.6	99.3	98.3	97.9	98.4	99.2	90.3	95.8	84.1	95.7	94.1	96.0	96.9	97.8	98.5	97.7	98.9	96.0
Montenegro	5 to 9	90.7	99.0	89.4	98.9	91.5	98.9	91.8	99.2	91.5	99.0	91.5	98.6	94.5	99.1	91.9	99.3	96.4	98.9	96.8	98.2	95.9	97.3	93.5	95.4
Morocco	5 to 9	73.4	94.0	63.3	91.9	81.5	95.2	72.9	93.9	62.9	91.0	80.4	95.0	71.2	90.6	61.4	89.2	82.0	93.2	73.8	90.5	70.4	89.4	76.6	89.0
Mozambique	5 to 9	93.2	97.0	95.0	97.9	91.5	95.9	93.0	97.0	93.9	96.9	91.3	96.1	90.4	93.0	94.8	97.2	90.3	92.5	95.4	97.0	96.8	97.8	89.1	91.4
Myanmar	5 to 9	74.3	88.2	75.0	90.0	73.8	86.7	82.5	91.8	82.9	93.3	81.2	89.6	47.9	75.9	39.4	75.1	59.9	79.9	82.6	90.2	86.7	92.8	73.3	83.8
Namibia	5 to 9	94.2	95.4	94.6	95.9	92.7	94.2	91.3	93.5	90.5	93.0	90.6	92.6	95.1	95.3	95.7	96.3	95.8	95.9	95.8	95.7	95.6	95.7	91.1	91.2
Nauru	5 to 9	90.9	89.6	92.4	90.6	90.6	89.5	95.6	94.3	87.4	84.7	99.9	99.9	63.8	59.4	85.4	78.8	61.1	59.9	89.5	89.7	97.2	96.4	84.1	83.2
Nepal	5 to 9	87.3	95.2	86.0	95.8	87.5	93.6	90.0	96.1	87.7	95.0	90.5	95.2	74.6	88.2	75.1	91.9	77.8	87.8	90.6	95.7	90.2	96.0	85.3	88.8
Netherlands	5 to 9	95.9	99.1	95.4	98.5	95.9	99.4	97.0	99.4	95.8	97.9	96.5	99.5	82.7	95.2	78.1	93.5	87.3	96.7	95.1	98.9	95.6	98.3	90.8	96.9
New Zealand	5 to 9	98.0	99.8	96.5	98.9	98.6	100.0	98.4	99.7	96.8	98.1	98.5	99.9	92.6	98.1	85.8	96.4	97.2	99.2	96.0	99.0	95.1	97.8	92.1	96.3
Nicaragua	5 to 9	86.7	98.0	85.1	97.9	87.6	97.6	92.1	98.9	91.0	97.8	91.9	98.5	72.3	94.1	63.4	94.2	80.4	94.6	84.1	97.9	85.6	97.5	77.7	94.7
Niger	5 to 9	85.0	93.3	92.2	95.8	80.9	91.8	88.7	95.0	91.8	95.3	86.5	94.0	86.6	92.4	93.9	95.8	87.1	93.0	85.8	92.2	97.7	98.2	60.0	77.5
Nigeria	5 to 9	91.3	92.8	96.1	97.3	87.4	88.5	92.5	94.4	94.7	96.2	90.2	92.5	93.7	93.4	97.3	97.6	93.3	92.8	89.0	84.3	97.7	98.0	66.8	48.7
Niue	5 to 9	92.1	72.1	92.5	64.6	92.6	78.7	95.9	81.3	88.2	48.7	99.9	99.7	69.5	0.0	85.5	29.6	67.6	0.0	92.9	79.1	97.4	90.3	90.9	67.9
North Macedonia	5 to 9	90.8	97.8	90.4	97.7	90.3	97.4	93.6	98.6	92.3	97.2	92.9	98.3	77.8	93.3	72.0	93.4	83.9	94.2	91.2	96.7	91.7	96.5	85.3	93.7
Northern Mariana Islands	5 to 9	96.9	97.7	97.6	98.1	96.8	97.7	98.8	99.1	95.9	96.5	99.9	99.8	87.9	89.8	95.3	96.4	87.6	89.8	95.8	96.8	97.9	98.0	95.1	96.9

Norway	5 to 9	93.9	99.8	93.3	99.4	93.7	99.8	97.0	99.7	94.5	98.1	97.8	100.0	74.5	98.7	71.8	99.1	79.5	98.3	90.5	99.2	92.4	98.6	82.2	96.7
Oman	5 to 9	83.9	97.3	84.5	97.2	83.9	97.3	82.7	96.8	85.8	95.6	77.9	96.3	86.6	93.3	87.6	97.0	89.0	95.1	84.6	95.6	84.2	95.3	83.4	93.7
Pakistan	5 to 9	89.8	85.7	86.9	83.6	91.4	86.4	91.5	88.3	88.2	85.1	93.0	89.6	76.8	65.3	72.3	59.2	81.9	73.2	91.7	86.4	90.1	87.6	87.8	78.4
Palau	5 to 9	93.0	95.9	93.3	95.0	93.5	96.9	96.4	97.4	88.8	92.4	99.9	99.9	76.6	86.1	88.0	90.0	77.0	88.0	91.5	94.9	97.2	96.9	87.8	93.7
Palestine	5 to 9	69.1	88.1	71.5	89.2	67.2	86.9	66.3	84.8	70.3	86.2	62.2	82.3	87.7	94.4	87.4	95.6	89.9	95.1	68.2	85.9	74.2	88.1	56.5	80.0
Panama	5 to 9	94.3	96.8	95.1	96.3	93.9	97.0	97.2	98.7	98.0	97.8	95.6	98.4	86.7	89.7	87.5	88.3	88.8	92.0	92.2	97.2	93.1	96.3	94.4	97.8
Papua New Guinea	5 to 9	90.9	91.2	88.6	89.3	93.1	93.5	93.3	93.5	80.6	81.3	99.9	99.7	71.5	72.2	84.2	84.2	72.2	73.8	94.3	93.7	96.5	96.8	93.9	94.4
Paraguay	5 to 9	87.8	96.3	84.3	95.5	90.4	96.7	94.5	98.2	91.8	96.7	95.2	98.3	65.9	88.4	50.3	84.3	78.5	91.8	92.9	98.3	92.6	97.8	90.6	96.3
Peru	5 to 9	84.5	90.1	82.7	89.7	86.0	90.0	91.0	93.6	90.2	92.6	91.1	93.0	68.6	76.6	61.2	71.6	77.0	82.4	87.4	93.0	88.4	93.3	84.6	88.5
Philippines	5 to 9	78.9	90.2	81.6	90.2	75.4	89.3	82.9	92.5	86.1	92.3	77.4	91.1	65.7	79.9	56.6	72.6	74.8	86.1	83.6	91.6	88.0	92.6	71.8	84.5
Poland	5 to 9	95.9	99.4	95.2	98.6	95.7	99.3	97.0	99.3	96.0	97.8	96.7	99.3	92.0	98.3	90.0	97.4	93.7	98.6	96.4	98.3	94.7	96.9	92.6	94.6
Portugal	5 to 9	80.9	96.7	76.8	95.7	84.3	97.4	86.7	97.6	83.9	96.7	88.1	97.6	62.6	90.2	45.4	87.0	76.5	93.4	69.6	96.9	72.8	96.4	62.4	94.7
Puerto Rico	5 to 9	98.6	99.7	98.9	100.0	98.4	99.6	99.5	100.0	98.4	98.9	99.1	99.7	94.8	97.3	96.7	99.9	95.2	97.2	98.8	99.8	99.0	100.0	99.0	100.0
Qatar	5 to 9	89.4	97.5	91.8	97.6	88.5	97.4	91.4	97.9	91.5	96.7	90.5	97.6	80.1	93.5	89.7	96.4	77.6	93.9	93.9	97.6	95.1	97.2	93.7	96.1
Republic of Korea	5 to 9	86.9	98.7	86.1	98.4	87.6	98.7	93.1	99.2	92.8	98.0	92.3	99.0	66.2	95.9	57.7	96.2	75.6	96.5	87.9	97.8	90.8	97.6	81.6	96.0
Republic of Moldova	5 to 9	94.4	99.0	92.7	98.0	94.9	99.0	92.8	98.5	90.5	96.6	93.6	98.8	94.6	98.7	93.7	97.5	95.0	98.8	95.7	98.1	94.9	96.9	91.8	94.9
Romania	5 to 9	91.4	98.0	90.9	97.5	91.0	98.1	93.2	98.8	92.4	97.6	92.3	98.7	83.3	92.8	78.6	91.4	88.4	94.7	91.4	97.1	92.0	96.4	85.6	94.2
Russian Federation	5 to 9	97.5	99.0	96.4	98.2	97.4	98.6	97.6	98.8	95.3	97.0	98.1	98.5	93.7	97.0	92.4	96.2	94.7	97.3	95.9	97.9	94.9	96.6	91.5	94.0
Rwanda	5 to 9	80.0	96.8	78.6	96.2	80.7	96.9	80.6	96.4	78.4	94.6	81.6	96.8	75.7	94.3	79.1	95.5	78.3	94.8	90.5	97.7	90.5	97.2	86.2	94.4
Saint Kitts and Nevis	5 to 9	93.2	96.7	93.2	97.7	93.2	95.7	95.9	98.1	95.4	97.7	95.1	97.1	83.0	90.1	80.5	93.2	87.0	90.0	93.5	97.5	93.6	97.7	94.8	96.7
Saint Lucia	5 to 9	97.6	98.4	97.5	98.7	97.4	98.2	98.6	99.2	97.4	98.4	98.1	98.6	91.7	94.3	91.9	95.6	93.2	94.9	97.7	98.6	97.2	98.6	96.9	98.5
Saint Vincent and the Grenadines	5 to 9	94.2	96.8	94.5	95.1	94.0	98.4	95.7	98.1	96.0	96.3	94.3	98.9	87.8	91.3	85.6	87.7	91.3	95.2	94.9	96.2	95.0	94.7	95.8	98.6
Samoa	5 to 9	93.2	95.9	93.6	95.9	93.4	96.0	96.6	97.8	90.4	93.8	100.0	99.9	73.7	83.4	88.4	92.4	73.0	82.9	94.6	95.8	97.4	97.4	92.7	94.5
San Marino	5 to 9	96.4	99.5	94.9	98.8	97.3	99.7	96.6	99.4	94.6	97.9	97.4	99.6	92.9	98.1	89.4	98.0	95.3	98.2	94.6	98.8	94.6	98.2	90.6	96.4
Sao Tome and Principe	5 to 9	87.6	97.8	97.9	99.3	81.3	97.0	92.2	98.9	97.3	98.6	89.5	98.2	87.8	96.1	97.4	98.9	86.9	96.3	75.5	95.2	99.6	99.4	21.0	83.9
Saudi Arabia	5 to 9	76.9	98.0	75.9	97.9	77.7	98.0	73.7	97.9	73.5	96.7	73.2	97.7	88.9	97.5	88.3	98.7	91.1	98.0	77.9	96.7	80.4	96.7	71.9	94.4
Senegal	5 to 9	89.6	94.3	94.5	97.1	86.1	92.5	92.2	96.2	93.3	95.9	90.8	95.6	88.6	90.6	94.1	95.5	88.8	90.9	84.2	90.0	97.7	98.5	53.3	68.8
Serbia	5 to 9	89.6	98.9	89.9	98.7	88.9	98.9	93.4	99.4	93.4	98.1	92.2	99.2	75.4	96.7	67.0	96.8	83.4	97.3	89.0	97.4	91.5	97.2	81.6	94.9
Seychelles	5 to 9	97.5	98.8	96.0	97.3	98.2	99.7	98.4	99.2	96.1	96.9	98.9	99.8	94.7	97.3	92.0	95.3	96.6	98.5	95.8	97.1	95.2	95.8	94.0	96.4
Sierra Leone	5 to 9	83.8	87.9	92.2	93.7	79.0	84.3	89.1	92.6	92.7	93.1	86.7	91.6	85.3	85.5	91.5	91.2	86.5	86.4	71.8	75.4	96.7	97.1	17.5	18.8
Singapore	5 to 9	95.5	99.3	94.1	99.0	96.4	99.4	97.1	99.6	95.6	98.4	97.3	99.5	91.6	97.9	90.3	98.6	93.6	98.0	94.2	98.2	93.3	97.8	93.3	96.3
Slovakia	5 to 9	95.9	98.8	95.3	98.3	95.7	98.6	96.8	99.0	95.4	97.8	96.4	98.8	91.7	96.9	89.0	96.2	94.1	97.5	95.0	97.5	94.5	96.8	90.8	94.7
Slovenia	5 to 9	98.4	99.6	98.2	99.0	98.2	99.8	98.9	99.8	98.0	98.4	98.7	99.7	96.6	99.1	95.8	98.7	97.3	99.3	97.0	98.2	96.7	97.3	93.1	95.5
Solomon Islands	5 to 9	96.1	96.3	96.1	96.4	96.9	97.0	97.7	97.7	92.8	93.1	99.9	99.9	83.8	83.6	93.1	93.3	84.5	84.1	96.2	96.4	98.2	98.1	97.8	97.8
Somalia	5 to 9	92.6	96.4	92.8	96.7	92.2	95.7	92.0	95.7	90.9	95.3	91.9	95.2	89.4	92.9	93.1	96.4	90.1	92.9	97.2	98.3	96.4	97.5	93.5	94.2
South Sudan	5 to 9	91.6	95.8	89.1	90.6	89.6	91.2	85.8	93.0	88.0	88.7	89.6	90.7	94.5	96.5	89.7	89.1	90.5	89.6	92.9	95.2	94.7	94.6	90.0	89.5
Spain	5 to 9	89.4	91.0	92.6	98.0	94.9	99.0	89.4	90.2	95.9	98.0	96.1	99.1	88.8	87.8	78.7	94.0	87.8	96.3	94.2	94.1	91.6	97.9	85.5	96.3
Sri Lanka	5 to 9	94.0	98.7	87.9	96.7	91.1	97.7	96.4	99.1	90.2	96.4	92.9	98.3	83.2	95.2	84.5	96.1	92.1	96.7	91.6	98.6	86.0	94.4	79.4	91.9
Sudan	5 to 9	89.8	97.5	46.5	80.1	67.5	85.1	92.2	98.1	49.1	80.2	63.0	83.3	88.4	96.0	50.6	71.8	74.3	82.5	84.4	94.6	65.8	79.3	73.9	74.0
Suriname	5 to 9	58.2	82.8	91.6	94.6	89.6	94.9	56.9	82.2	94.9	95.7	91.7	96.0	61.9	76.0	77.7	84.8	82.3	89.5	70.5	78.1	94.0	95.8	93.7	96.5
Sweden	5 to 9	90.6	94.8	96.0	99.1	96.8	99.9	93.9	96.5	97.4	98.9	97.4	99.7	78.3	86.4	85.2	97.1	92.9	99.2	93.6	96.3	95.5	98.3	92.4	96.4
Switzerland	5 to 9	96.6	99.8	92.0	98.0	95.3	99.5	97.9	99.7	94.2	97.9	96.9	99.6	89.5	98.6	71.6	92.5	88.5	97.3	95.5	99.3	91.7	98.0	87.7	96.5
Syrian Arab Republic	5 to 9	94.1	99.0	45.1	89.6	50.6	89.9	96.3	99.4	44.6	85.7	38.0	83.7	81.1	95.4	73.7	95.1	82.6	97.7	91.9	98.8	57.5	89.6	57.8	87.1
Taiwan (Province of China)	5 to 9	47.1	90.0	97.7	98.9	97.5	98.8	40.8	85.2	97.5	98.8	98.0	98.9	77.6	96.6	97.0	98.6	97.0	97.4	58.7	90.1	96.6	97.3	94.4	96.1
Tajikistan	5 to 9	97.7	98.8	90.6	93.8	93.0	95.2	98.4	99.3	92.6	94.8	93.6	95.6	96.4	97.2	73.4	81.2	92.6	93.8	96.4	97.5	86.4	90.8	77.2	84.2
Thailand	5 to 9	92.5	95.1	80.5	95.4	84.5	95.3	94.1	96.2	88.7	96.0	92.1	97.3	84.9	88.7	43.6	87.8	66.8	89.8	85.5	90.7	90.0	95.8	84.0	93.4
Timor-Leste	5 to 9	82.8	95.6	80.3	90.4	80.5	88.1	91.2	97.4	87.0	93.1	85.9	91.3	54.6	87.9	55.7	76.4	73.4	81.7	89.1	96.1	88.8	93.2	80.3	85.0
Togo	5 to 9	80.4	89.3	95.1	96.7	87.1	91.1	87.0	92.8	93.9	95.4	91.6	94.9	63.9	77.8	94.9	95.6	90.6	91.4	86.7	91.5	98.1	98.2	53.8	61.2

Tokelau	5 to 9	90.0	93.3	93.9	73.4	93.0	81.2	92.9	95.6	90.6	63.5	99.9	99.8	90.0	90.9	89.6	52.3	77.5	39.4	84.1	87.5	97.5	92.1	92.2	77.1
Tonga	5 to 9	93.1	77.3	97.1	96.8	96.7	97.6	96.3	84.5	94.8	94.6	99.9	99.9	78.5	35.9	94.8	94.1	86.8	89.8	94.3	85.6	97.9	97.6	94.4	95.5
Trinidad and Tobago	5 to 9	96.7	97.1	93.9	98.3	92.2	97.3	98.4	98.3	95.2	97.9	94.6	98.8	87.2	89.3	84.2	93.6	83.4	90.5	95.8	96.3	94.7	98.8	91.3	98.0
Tropical Latin America	5 to 9	93.2	97.8	90.5	94.4	91.9	95.5	95.6	99.0	93.4	95.4	94.6	97.3	81.8	90.6	72.6	83.0	85.6	90.5	93.6	98.7	93.8	97.7	87.4	94.3
Tunisia	5 to 9	79.6	96.6	74.5	95.4	83.6	97.1	79.7	96.6	74.6	94.3	82.8	97.1	80.6	94.7	76.4	94.8	86.4	96.1	80.5	94.5	78.7	93.7	79.9	92.4
Turkey	5 to 9	79.2	96.5	79.7	96.3	79.6	96.5	83.6	97.0	83.4	95.5	83.1	96.9	58.6	91.9	60.9	93.1	63.2	93.0	84.5	96.3	86.8	96.1	81.1	94.8
Turkmenistan	5 to 9	96.2	98.9	95.0	98.1	96.4	98.6	97.3	99.0	95.5	97.2	97.1	99.0	91.4	95.6	85.6	94.2	95.4	96.5	90.5	96.8	90.6	95.8	86.1	92.8
Tuvalu	5 to 9	83.7	91.7	89.8	93.4	79.8	90.7	94.0	96.6	84.0	90.8	99.9	99.9	42.6	68.0	83.7	87.0	35.5	66.5	86.7	92.6	96.8	96.5	74.7	87.2
Uganda	5 to 9	91.9	96.2	92.3	96.0	91.4	96.1	91.0	95.7	89.9	94.2	90.9	95.9	88.8	93.1	91.3	94.7	89.6	93.6	95.6	97.0	95.8	97.0	92.0	93.7
Ukraine	5 to 9	98.4	99.3	97.5	98.5	97.9	99.2	97.2	98.8	95.4	96.9	97.0	99.0	99.6	99.5	98.8	99.1	99.5	99.3	97.1	98.1	96.8	97.2	89.8	93.6
United Arab Emirates	5 to 9	88.4	98.2	91.4	97.8	86.7	98.3	89.0	98.3	91.9	96.9	85.8	98.3	80.2	94.3	83.3	95.6	82.6	95.7	88.5	97.6	92.1	97.1	82.7	96.4
United Kingdom	5 to 9	95.7	99.4	94.9	98.9	96.0	99.6	96.6	99.4	94.8	97.7	96.9	99.6	82.7	97.6	76.3	96.7	88.1	98.1	94.4	99.0	95.6	98.6	89.7	96.7
United Republic of Tanzania	5 to 9	91.6	95.8	91.4	95.7	91.6	95.6	91.6	95.5	90.5	94.3	91.8	95.5	89.2	91.5	90.9	93.4	90.4	92.2	95.6	95.8	95.7	96.6	92.0	90.9
United States Virgin Islands	5 to 9	97.7	99.9	98.1	100.0	97.2	99.9	98.9	100.0	98.0	99.0	98.5	99.9	92.0	97.2	94.2	100.0	92.6	96.9	98.7	100.0	98.7	99.9	97.8	99.6
United States of America	5 to 9	97.6	97.9	97.9	98.6	98.0	98.3	98.7	99.1	98.7	99.3	98.3	98.8	89.8	90.7	88.6	92.2	92.2	91.8	95.9	97.3	97.4	98.9	97.5	98.8
Uruguay	5 to 9	92.1	98.0	90.6	97.1	92.9	98.4	97.0	99.2	95.3	97.7	97.2	99.4	73.2	92.5	64.3	90.1	81.2	94.6	86.2	96.4	86.4	95.5	81.1	94.0
Uzbekistan	5 to 9	95.5	98.1	94.3	97.4	95.5	97.7	95.5	98.4	93.9	97.1	95.1	98.1	94.4	95.7	90.0	93.5	96.7	96.9	90.9	94.8	90.9	94.4	85.0	89.7
Vanuatu	5 to 9	93.1	93.0	93.5	93.3	93.2	93.1	96.1	95.7	89.4	89.2	100.0	99.9	71.0	71.1	86.7	85.6	69.9	71.3	93.7	93.8	97.2	96.9	90.6	90.3
Venezuela (Bolivarian Republic of)	5 to 9	93.5	95.4	93.0	95.2	93.4	95.2	96.2	98.1	95.3	97.2	95.7	97.6	85.9	84.8	81.9	82.4	89.6	88.0	91.6	96.5	92.0	96.1	87.4	93.7
Viet Nam	5 to 9	96.2	97.7	96.4	97.8	95.6	97.1	97.5	98.4	95.9	97.2	96.9	97.9	92.2	95.0	93.2	96.1	92.3	94.9	96.0	96.5	96.0	96.4	94.0	93.9
Yemen	5 to 9	73.2	86.3	64.3	83.1	80.2	89.0	72.4	86.5	65.1	83.2	77.7	88.5	75.3	82.2	69.0	79.5	83.5	87.2	81.1	83.5	76.4	82.8	84.3	81.7
Zambia	5 to 9	92.8	96.5	91.7	96.2	93.6	96.4	92.8	96.1	90.3	94.5	93.9	96.4	91.6	94.2	91.8	95.5	92.9	94.7	96.6	97.3	96.4	97.2	93.5	93.6
Zimbabwe	5 to 9	97.8	92.8	97.5	93.3	97.5	91.1	96.7	89.5	94.8	88.9	97.0	88.6	94.1	85.8	95.3	86.1	95.2	88.7	98.1	94.9	97.4	94.7	94.6	90.3
Afghanistan	10 to 14	71.5	81.0	68.5	80.1	76.2	83.5	71.3	80.2	64.4	76.1	75.6	82.7	79.0	82.3	85.3	87.5	72.0	77.2	80.6	79.9	76.8	79.1	82.4	74.7
Albania	10 to 14	85.0	92.0	85.0	92.1	86.2	92.5	87.8	92.0	85.4	90.9	88.9	92.6	87.4	93.9	93.7	96.8	78.4	89.8	93.6	95.0	94.4	94.9	87.2	89.3
Algeria	10 to 14	68.6	91.5	64.6	90.6	74.3	92.9	69.6	90.1	61.9	87.6	74.6	91.3	77.1	92.0	82.6	93.4	72.4	91.3	68.8	87.5	67.1	86.6	64.9	83.6
American Samoa	10 to 14	89.8	91.1	87.3	87.7	91.8	94.0	90.1	91.6	87.5	88.2	91.5	93.4	86.9	87.3	88.3	87.8	87.7	89.4	85.1	86.8	87.0	86.8	82.2	86.3
Andorra	10 to 14	92.4	98.2	89.5	96.9	94.9	98.9	93.6	97.8	90.8	96.6	95.1	98.1	86.2	96.7	87.6	96.6	86.5	97.7	92.9	97.7	92.4	96.8	89.1	95.6
Angola	10 to 14	78.3	89.2	73.2	86.9	83.5	91.4	77.1	86.5	66.2	80.4	83.5	90.1	84.7	90.7	88.8	93.1	80.1	87.7	89.0	92.3	89.9	92.7	83.2	86.7
Antigua and Barbuda	10 to 14	94.0	96.8	93.0	95.2	94.7	98.1	95.8	98.2	94.1	97.2	96.5	98.5	90.7	94.9	94.0	96.0	86.9	94.6	97.1	93.7	96.9	91.8	96.7	98.8
Argentina	10 to 14	84.0	94.3	84.3	94.5	84.9	94.2	91.4	95.0	90.3	94.3	91.6	95.0	72.9	91.8	84.3	95.5	57.9	86.6	89.0	96.0	89.2	95.5	82.9	92.7
Armenia	10 to 14	96.9	98.7	98.2	99.4	96.1	98.4	94.9	96.5	93.5	94.8	95.3	97.1	96.8	99.2	98.6	99.7	93.6	98.1	93.3	96.1	95.0	95.7	81.3	89.9
Australia	10 to 14	92.3	98.1	90.8	97.0	93.7	98.5	94.7	98.4	93.5	98.1	95.1	98.2	86.4	96.3	92.0	97.4	78.6	95.2	92.0	97.8	92.4	96.7	87.1	95.3
Austria	10 to 14	94.9	99.0	95.3	98.7	94.9	98.9	94.8	98.1	94.3	97.4	94.7	98.0	91.6	98.0	95.0	98.8	87.6	97.2	92.5	98.2	94.1	97.7	84.5	95.7
Azerbaijan	10 to 14	94.3	96.5	94.5	96.9	94.8	96.6	90.1	93.2	86.8	91.0	91.9	94.3	98.6	99.5	98.9	99.5	98.0	99.0	94.7	95.6	95.3	95.5	85.2	88.2
Bahamas	10 to 14	94.2	98.3	92.6	98.2	95.4	97.9	95.5	98.6	93.9	98.4	96.1	98.1	91.6	96.1	93.9	97.7	89.6	94.1	96.9	99.3	96.6	99.2	97.1	98.6
Bahrain	10 to 14	84.0	92.5	90.9	94.0	80.2	92.0	83.8	92.1	87.9	92.2	80.3	91.5	86.1	93.4	92.5	95.3	76.2	91.3	89.1	91.6	91.7	91.7	79.6	86.5
Bangladesh	10 to 14	52.3	67.0	66.9	77.0	42.8	59.0	64.7	73.5	70.6	77.7	58.0	67.6	47.8	63.3	79.5	84.0	1.9	32.3	77.5	80.8	83.8	87.0	56.6	58.0
Barbados	10 to 14	91.7	98.3	90.1	97.4	93.2	98.8	94.9	99.2	93.1	98.4	95.7	99.3	87.8	95.5	91.2	96.6	84.7	95.2	93.2	99.0	93.6	98.7	91.7	99.3
Belarus	10 to 14	93.9	98.1	94.3	98.3	93.8	98.2	92.4	96.4	91.3	95.0	92.2	96.8	90.6	97.5	92.1	97.7	89.9	97.2	92.2	96.1	92.5	95.1	83.8	92.0
Belgium	10 to 14	89.4	97.4	88.2	96.1	91.0	98.2	90.0	97.9	87.4	96.9	91.3	98.1	83.4	94.7	88.2	95.1	78.6	95.2	88.6	97.4	89.8	96.8	80.7	95.3
Belize	10 to 14	88.2	97.5	87.8	97.5	88.7	97.2	91.9	97.2	90.4	96.7	92.4	96.9	85.3	96.2	91.1	97.7	77.6	94.3	94.8	98.9	95.8	98.8	91.2	97.4
Benin	10 to 14	90.5	91.0	84.6	85.5	93.9	94.4	92.9	92.5	83.8	83.0	96.5	96.7	92.9	92.4	93.4	92.6	93.0	93.0	92.1	90.5	94.1	92.9	83.6	80.6
Bermuda	10 to 14	98.5	100.0	97.9	99.5	98.8	100.0	98.8	99.9	97.9	99.2	98.9	99.8	96.4	98.4	97.4	98.8	95.8	98.7	98.9	100.0	98.6	99.8	98.7	99.8
Bhutan	10 to 14	90.9	92.7	88.5	93.9	93.4	92.2	90.2	91.7	85.3	90.1	93.1	92.1	90.1	91.1	92.7	95.2	87.4	85.3	93.4	93.8	91.9	94.4	90.6	86.0
Bolivia (Plurinational State of)	10 to 14	54.5	79.7	47.3	79.6	63.7	81.3	73.7	87.6	63.1	84.8	80.6	89.1	44.8	72.2	62.8	82.6	24.3	59.8	72.7	90.5	69.3	89.0	75.6	88.7
Bosnia and Herzegovina	10 to 14	90.6	95.6	91.1	95.8	90.8	95.5	95.2	96.6	92.7	94.8	96.2	97.3	82.6	92.5	90.4	95.5	70.7	88.5	90.3	93.2	91.7	93.4	79.6	83.9
Botswana	10 to 14	89.8	93.1	91.1	94.2	88.3	92.1	83.7	88.9	81.5	87.9	85.2	89.5	95.2	96.1	96.7	97.2	93.1	94.5	93.5	93.5	95.2	95.5	84.0	83.3

Brazil	10 to 14	84.4	86.7	85.0	87.1	85.0	87.1	89.1	90.9	87.3	89.3	89.7	91.3	79.7	80.6	86.9	87.8	70.4	71.3	88.3	95.6	92.1	96.0	74.6	89.9
Brunei Darussalam	10 to 14	78.0	95.8	85.8	94.7	73.3	96.6	85.1	96.7	88.6	95.4	81.9	97.0	72.2	94.2	89.9	96.1	44.3	92.0	78.5	94.0	87.3	93.7	54.2	91.2
Bulgaria	10 to 14	79.4	92.9	81.0	93.5	79.2	92.6	84.8	93.9	83.1	92.9	85.6	94.0	83.2	91.9	91.9	95.6	70.7	86.7	80.4	91.9	87.4	93.1	57.9	83.2
Burkina Faso	10 to 14	92.7	91.3	90.8	88.0	93.6	93.3	94.3	92.8	91.0	87.1	95.5	95.3	94.5	93.1	95.5	94.1	93.5	92.1	94.8	92.6	96.2	94.8	87.3	82.8
Burundi	10 to 14	67.7	86.7	59.4	81.2	75.9	91.5	67.7	83.6	51.8	73.3	77.2	89.7	78.3	90.2	84.8	91.4	70.7	88.7	85.1	92.5	83.4	90.9	82.9	90.7
Cabo Verde	10 to 14	93.6	93.5	90.8	92.1	95.1	94.1	96.9	95.4	91.2	91.1	98.8	96.8	94.2	93.6	95.5	95.2	93.0	91.9	91.7	89.2	95.0	94.6	80.0	71.2
Cambodia	10 to 14	83.5	91.6	81.6	92.6	86.1	91.4	87.0	92.2	83.0	91.1	89.3	92.5	83.5	91.5	88.8	95.2	77.5	86.4	89.9	93.0	90.2	93.2	84.5	86.8
Cameroon	10 to 14	84.6	85.4	80.5	80.7	87.6	88.9	87.1	87.7	78.6	77.5	91.1	92.4	89.8	87.8	92.6	90.5	86.6	85.1	89.3	85.9	93.3	91.2	76.5	67.2
Canada	10 to 14	94.8	97.4	93.1	96.7	96.2	97.5	96.8	98.7	95.6	98.4	97.1	98.3	88.5	93.4	92.1	97.2	84.4	87.3	92.6	95.7	93.6	96.4	89.3	94.7
Central African Republic	10 to 14	72.9	81.9	68.0	79.4	78.3	84.8	71.8	80.2	58.7	71.8	79.3	85.1	80.2	84.5	87.1	90.1	72.6	77.8	87.8	90.8	88.7	91.7	80.8	83.5
Chad	10 to 14	85.0	80.7	79.4	72.0	88.9	87.2	87.9	84.0	77.4	68.5	92.7	91.3	88.8	83.9	91.5	86.5	86.1	81.9	88.8	82.7	93.1	89.1	74.5	62.1
Chile	10 to 14	86.5	95.6	86.8	95.3	87.1	96.0	91.6	96.8	90.9	96.1	91.3	96.6	79.5	91.1	88.7	93.8	66.6	87.9	85.8	95.8	86.3	95.2	78.8	92.3
China	10 to 14	88.0	94.9	88.3	95.8	88.2	94.3	92.6	94.1	92.6	94.3	92.2	93.5	81.3	95.0	88.2	97.2	73.0	92.0	90.0	92.6	92.5	93.9	80.7	84.2
Colombia	10 to 14	85.0	89.0	82.5	91.5	88.1	87.6	93.4	96.1	90.9	95.7	94.5	95.6	76.6	80.4	82.1	89.9	71.9	66.5	85.3	94.7	88.1	96.6	74.1	85.9
Comoros	10 to 14	80.5	90.3	75.4	86.7	85.4	93.3	78.3	87.4	66.7	79.7	85.0	91.7	86.9	92.3	90.4	93.4	82.8	90.9	90.1	93.7	89.7	92.9	86.3	90.6
Congo	10 to 14	86.1	91.1	84.1	88.8	88.3	93.1	84.0	88.7	77.9	83.1	87.7	91.9	89.7	92.1	93.2	94.0	85.3	89.7	91.7	93.0	93.0	93.4	84.7	87.7
Cook Islands	10 to 14	74.8	91.6	64.9	86.1	84.0	96.1	82.3	93.0	73.6	88.2	87.2	95.5	68.0	88.2	69.8	87.7	73.2	92.7	66.2	83.4	66.8	81.2	66.6	88.2
Costa Rica	10 to 14	95.7	97.5	96.2	98.5	95.6	96.8	97.2	99.0	96.1	98.2	97.3	98.9	93.6	94.9	96.2	97.9	90.0	90.1	95.4	99.1	95.9	98.7	89.9	96.0
Coted'Ivoire	10 to 14	85.3	86.1	82.1	82.1	87.2	88.8	88.3	89.1	80.3	78.8	91.6	93.3	88.5	87.1	92.1	90.2	84.5	84.0	84.3	81.6	92.6	90.8	60.1	53.3
Croatia	10 to 14	91.5	97.8	92.2	97.2	91.2	98.3	95.5	98.7	95.6	97.7	94.8	99.0	87.8	96.8	93.2	97.0	80.0	97.1	91.0	95.7	92.7	94.9	80.8	91.7
Cuba	10 to 14	93.3	97.4	93.7	98.0	92.8	96.3	95.2	98.6	94.8	98.8	94.8	97.7	90.3	94.8	94.2	97.4	85.1	91.0	94.8	98.3	96.4	99.0	89.1	95.7
Cyprus	10 to 14	92.9	95.3	92.0	92.2	93.7	97.6	97.4	96.5	97.8	94.6	96.7	97.4	87.4	92.6	91.6	92.0	82.7	94.8	93.7	95.1	93.7	94.0	89.2	93.9
Czechia	10 to 14	93.0	97.9	94.7	98.5	91.9	97.3	95.2	97.9	94.4	97.0	95.0	97.9	89.2	97.2	94.8	98.6	80.5	94.8	89.9	95.4	92.3	95.4	77.3	89.8
Democratic People's Republic of Korea	10 to 14	90.8	93.9	89.0	93.9	92.4	93.9	93.6	93.3	92.1	92.7	94.2	93.3	87.4	93.3	91.1	95.8	83.5	90.0	90.3	92.4	92.0	93.6	84.4	85.0
Democratic Republic of the Congo	10 to 14	80.5	88.9	73.2	86.0	86.8	91.6	79.9	86.0	66.2	78.4	87.2	90.3	86.8	91.7	89.6	93.6	83.9	89.3	89.3	93.8	89.3	93.5	85.0	89.3
Denmark	10 to 14	86.5	97.0	85.7	96.6	88.0	97.2	90.0	97.5	89.0	96.8	90.1	97.4	75.7	93.7	82.9	95.9	68.1	91.3	86.1	97.3	89.1	96.9	75.4	94.6
Djibouti	10 to 14	86.9	92.7	84.6	92.3	89.1	93.2	84.5	90.0	78.0	87.1	88.0	91.6	90.5	92.8	93.9	95.9	86.3	89.0	94.3	95.5	93.9	95.5	90.6	90.3
Dominica	10 to 14	95.0	94.1	94.3	94.1	95.5	94.0	96.0	96.2	94.3	94.9	96.7	96.5	92.1	90.9	95.0	94.7	88.6	85.9	97.4	96.4	97.1	96.7	96.5	94.3
Dominican Republic	10 to 14	81.7	88.5	82.4	90.4	81.5	87.0	88.5	91.7	87.6	91.6	88.0	90.7	74.9	83.2	84.7	90.5	61.2	72.7	91.3	93.5	94.1	95.8	82.8	84.9
Ecuador	10 to 14	72.4	83.4	75.2	81.0	72.5	86.5	79.3	91.0	78.3	88.4	79.4	92.3	72.9	74.7	85.2	81.1	56.7	68.4	76.2	93.4	76.1	91.4	70.9	92.2
Egypt	10 to 14	0.0	78.4	6.6	83.6	0.0	76.6	0.0	77.2	0.0	82.4	0.0	74.5	37.3	83.0	63.4	91.6	0.0	70.4	0.0	62.0	0.0	68.5	0.0	41.4
El Salvador	10 to 14	71.2	84.6	69.8	85.3	74.3	85.1	85.0	91.2	82.3	90.6	86.1	90.9	65.6	79.8	76.8	86.8	52.2	71.2	72.0	86.9	79.6	90.8	49.9	73.5
Equatorial Guinea	10 to 14	76.7	89.3	72.6	84.8	81.2	92.6	75.5	87.2	64.7	78.6	81.9	91.4	82.7	92.2	88.8	92.4	75.6	91.7	88.9	89.7	89.9	89.6	81.9	84.7
Eritrea	10 to 14	78.1	89.3	76.5	87.9	80.9	90.7	76.3	86.2	68.0	80.9	81.2	89.2	85.8	91.5	92.1	94.6	78.1	87.2	92.9	94.9	92.4	94.5	88.5	90.2
Estonia	10 to 14	95.1	99.1	95.9	99.6	94.7	98.8	93.2	96.6	92.0	95.3	93.4	97.0	95.0	100.0	98.0	100.0	89.8	99.4	93.4	96.7	92.5	95.7	87.4	92.5
Eswatini	10 to 14	86.9	87.4	90.1	90.6	83.8	85.0	80.3	81.8	80.4	81.8	80.2	82.4	94.3	93.1	96.4	95.8	91.4	89.6	93.2	90.8	95.1	94.2	82.6	76.4
Ethiopia	10 to 14	82.5	91.4	78.9	89.0	86.3	93.5	82.0	88.9	73.5	83.0	86.8	92.2	90.1	94.8	93.3	95.9	86.2	93.1	95.5	95.9	93.9	94.7	92.8	93.2
Fiji	10 to 14	81.3	78.4	76.7	75.9	85.9	81.8	84.1	81.2	80.2	79.4	86.4	82.4	72.7	70.1	75.7	75.6	74.5	68.0	81.6	77.5	83.2	80.4	77.1	68.9
Finland	10 to 14	87.6	96.4	87.7	95.3	88.4	97.2	91.9	97.3	91.9	96.0	91.4	97.6	76.9	91.3	84.6	93.6	68.4	89.5	84.9	97.0	88.8	96.5	72.1	94.6
France	10 to 14	93.4	97.9	92.1	96.7	94.5	98.4	94.7	98.4	93.3	98.7	95.1	97.8	90.4	96.4	92.5	97.0	88.9	96.2	92.9	97.5	93.7	97.1	87.6	95.3
Gabon	10 to 14	89.8	93.7	88.3	93.8	91.3	93.6	88.0	91.4	83.6	90.2	90.4	92.1	93.5	94.8	95.6	96.9	90.7	91.8	93.7	94.5	94.0	95.1	89.2	88.6
Gambia	10 to 14	85.4	85.1	81.7	80.4	88.0	88.6	87.5	86.3	78.6	75.0	91.5	91.7	88.9	87.2	91.8	90.1	85.9	84.1	85.8	83.5	92.4	90.4	65.8	60.7
Georgia	10 to 14	95.7	98.7	96.2	99.6	95.8	98.1	92.4	96.3	89.7	95.0	93.7	96.6	95.5	98.4	96.5	99.3	94.6	96.9	94.6	96.5	95.3	95.9	85.7	90.4
Germany	10 to 14	88.0	98.2	89.5	97.5	87.7	98.4	89.9	97.7	90.0	96.8	89.2	97.8	76.5	95.7	85.1	96.5	66.5	95.2	84.1	97.7	89.6	97.2	67.0	95.2
Ghana	10 to 14	82.9	92.0	78.7	90.3	86.0	92.6	86.1	92.8	75.9	87.0	90.9	95.1	86.1	91.2	89.8	93.4	82.1	88.7	81.9	89.0	90.8	94.1	55.0	69.2
Greece	10 to 14	84.7	96.0	84.5	96.0	86.0	96.0	83.5	95.5	81.8	95.1	84.4	95.3	88.9	95.7	92.4	97.9	84.8	92.9	82.6	96.4	86.4	96.3	69.5	92.8
Greenland	10 to 14	80.1	94.8	68.3	89.3	89.6	98.4	96.7	99.6	95.9	99.4	96.4	99.1	20.1	79.7	31.7	79.3	26.7	88.7	84.5	95.6	93.2	96.8	76.5	95.8
Grenada	10 to 14	90.2	96.7	86.6	97.7	93.8	95.5	94.0	97.5	89.8	97.6	96.8	96.7	84.9	94.1	88.6	97.2	82.1	89.8	91.4	97.8	90.3	98.9	94.3	94.5

Guam	10 to 14	82.2	89.9	82.0	98.3	83.5	83.5	86.7	89.7	85.9	95.1	87.3	86.1	76.2	85.8	82.1	97.0	71.7	70.6	73.7	86.2	81.5	95.3	56.5	62.0
Guatemala	10 to 14	75.5	83.6	75.4	86.1	77.5	82.6	80.9	89.8	77.7	88.8	82.4	89.6	86.4	79.4	91.0	88.8	80.7	66.5	69.8	88.9	76.8	91.8	48.3	77.1
Guinea	10 to 14	81.6	81.7	71.8	73.0	88.3	88.1	86.4	85.2	72.6	70.5	92.6	92.2	87.7	85.3	89.4	86.7	86.5	84.6	85.0	80.2	89.4	87.2	70.6	56.5
Guinea-Bissau	10 to 14	73.9	79.3	68.5	73.3	78.6	84.0	79.2	82.1	66.6	67.2	85.4	89.1	80.1	81.4	86.7	86.5	73.4	76.1	78.1	79.4	89.0	88.9	47.6	49.1
Guyana	10 to 14	77.2	88.6	77.2	89.6	77.3	87.6	83.7	92.3	80.9	91.0	84.8	92.4	74.5	82.8	86.3	90.9	58.8	71.5	83.5	92.6	89.1	94.7	69.1	86.0
Haiti	10 to 14	50.4	61.1	29.5	46.4	72.6	76.6	72.3	78.9	57.2	67.6	85.1	87.9	37.2	47.2	47.1	54.2	38.0	49.0	70.1	78.4	67.5	78.1	78.5	80.3
Honduras	10 to 14	58.5	88.0	47.2	89.6	70.5	87.6	76.6	93.7	66.2	92.8	83.5	93.7	51.6	81.7	61.9	89.4	43.8	72.2	62.4	92.4	66.0	95.0	50.5	82.6
Hungary	10 to 14	93.1	98.4	95.0	98.2	91.6	98.6	93.9	97.9	93.6	96.5	93.4	98.4	92.3	98.8	96.4	98.8	85.7	98.8	90.5	95.8	93.4	95.2	76.1	91.7
Iceland	10 to 14	82.9	94.5	82.2	89.5	84.8	98.3	88.6	96.0	87.5	92.8	89.0	97.8	70.0	88.4	79.0	85.6	60.5	95.3	82.4	96.5	86.8	95.1	68.2	95.6
India	10 to 14	87.2	92.9	85.3	92.7	89.9	93.7	86.9	91.0	81.7	88.0	90.1	92.6	86.0	92.0	90.1	94.2	81.6	89.3	92.3	95.4	90.9	94.3	88.4	90.7
Indonesia	10 to 14	80.0	82.2	77.6	84.6	83.5	81.3	83.9	83.5	78.6	82.5	87.1	83.6	75.3	79.5	82.4	88.1	67.9	68.2	87.6	87.6	88.5	90.0	79.7	74.7
Iran (Islamic Republic of)	10 to 14	87.4	93.1	86.9	93.8	88.5	93.1	87.7	91.5	87.2	91.1	87.8	91.5	86.3	94.4	90.0	96.2	82.4	92.1	71.2	82.9	73.5	84.3	60.8	73.0
Iraq	10 to 14	66.8	89.5	71.8	92.0	66.9	88.6	69.8	86.8	71.0	88.0	70.5	86.3	77.4	91.7	84.8	94.4	68.9	88.4	63.4	86.2	67.2	88.0	48.6	77.1
Ireland	10 to 14	89.7	98.5	89.1	98.2	90.8	98.5	94.0	98.2	92.7	97.6	94.3	98.1	77.5	96.5	84.7	97.6	69.5	95.4	88.9	98.1	90.8	97.5	79.9	95.6
Israel	10 to 14	91.7	97.7	90.0	97.2	93.3	97.9	89.8	97.4	87.9	96.8	90.7	97.2	89.5	96.7	93.6	97.9	84.4	95.3	92.7	97.9	92.1	97.2	89.5	95.3
Italy	10 to 14	87.8	96.0	86.7	94.3	89.5	97.3	89.6	95.5	87.4	93.7	90.7	96.2	80.8	92.9	85.4	93.1	76.7	94.0	87.2	97.2	89.1	96.1	77.9	95.0
Jamaica	10 to 14	84.9	96.0	83.6	96.0	86.6	95.6	90.8	97.5	88.0	97.2	92.2	97.0	82.9	93.5	88.0	95.7	77.6	90.8	88.0	97.5	90.2	97.9	81.0	95.0
Japan	10 to 14	93.8	96.4	93.7	95.6	94.2	97.0	95.5	97.0	94.7	96.2	95.5	97.1	92.6	94.3	95.7	95.8	88.2	92.9	91.2	95.9	91.5	95.4	85.5	92.0
Jordan	10 to 14	68.0	88.3	68.1	92.0	70.8	86.5	60.0	83.5	57.4	87.4	61.9	80.8	89.6	95.7	92.8	97.0	85.9	93.9	63.3	82.7	63.8	85.9	55.7	70.9
Kazakhstan	10 to 14	96.9	98.4	97.0	98.9	97.1	98.2	95.2	96.3	93.3	94.6	96.0	97.0	95.9	98.1	96.2	98.8	96.1	97.0	94.1	95.9	95.1	95.7	85.5	89.4
Kenya	10 to 14	94.5	95.6	93.0	95.4	95.7	95.8	91.8	92.8	87.7	90.7	93.9	94.0	96.9	96.9	97.4	97.9	95.8	95.1	97.1	97.3	95.9	96.6	94.4	93.6
Kiribati	10 to 14	80.6	78.5	82.3	80.6	80.8	79.0	80.5	79.2	78.2	78.1	82.7	80.5	76.1	77.3	83.3	82.8	67.0	70.7	79.7	76.5	85.0	82.7	67.2	58.8
Kuwait	10 to 14	78.4	89.6	85.7	95.9	74.7	85.8	75.5	86.6	83.9	92.8	70.2	82.7	82.4	94.2	88.5	97.3	74.6	89.4	77.8	84.8	82.3	91.0	63.4	66.9
Kyrgyzstan	10 to 14	92.5	97.4	92.9	97.8	92.9	97.3	90.4	95.9	87.4	94.3	91.9	96.5	91.6	96.1	93.1	96.8	90.6	95.5	89.1	94.8	93.4	95.2	69.6	86.2
Lao People's Democratic Republic	10 to 14	63.0	79.7	61.0	81.5	67.6	79.9	74.0	84.9	67.1	83.2	78.5	85.7	58.3	77.4	74.1	87.2	38.6	64.4	77.4	84.8	81.0	88.3	63.6	70.7
Latvia	10 to 14	93.0	98.8	94.1	98.9	92.1	98.9	89.7	97.0	88.8	95.6	89.1	97.5	94.6	99.9	97.9	99.7	88.7	99.8	91.2	96.3	91.8	95.4	83.7	92.6
Lebanon	10 to 14	87.2	95.2	88.8	96.6	87.4	94.6	86.0	92.8	85.8	93.2	86.3	92.2	88.0	94.5	92.0	96.2	83.1	92.2	92.0	96.2	90.7	95.4	88.8	92.0
Lesotho	10 to 14	90.3	82.4	92.1	86.5	88.2	78.9	84.5	76.4	83.7	76.2	84.9	76.7	95.4	90.9	97.0	94.7	93.4	86.2	95.6	89.3	96.0	92.9	88.9	73.6
Liberia	10 to 14	81.3	81.5	73.6	72.7	86.9	88.5	86.7	83.8	73.9	66.9	92.4	92.2	86.9	85.5	89.0	87.0	85.1	84.5	82.8	82.1	89.5	87.5	62.7	62.5
Libya	10 to 14	84.5	85.1	82.4	86.4	86.9	85.4	85.0	83.4	81.8	83.0	86.8	83.5	86.4	85.2	89.5	89.6	83.8	80.7	82.9	78.1	83.4	81.6	81.0	65.1
Lithuania	10 to 14	96.8	99.0	97.2	99.4	96.6	98.7	93.9	96.9	92.3	95.6	94.4	97.2	95.7	99.7	97.6	99.8	92.4	98.9	94.8	96.4	94.2	95.6	89.9	92.6
Luxembourg	10 to 14	88.2	98.3	83.3	98.4	92.2	98.0	90.4	98.1	86.3	98.4	92.8	97.3	84.5	98.1	86.9	98.8	83.3	97.3	81.6	97.7	79.1	97.5	80.2	94.3
Madagascar	10 to 14	69.1	81.8	66.5	78.9	73.4	85.0	70.8	79.7	60.4	70.8	77.2	84.9	78.3	84.1	86.3	89.3	68.6	77.5	85.6	90.9	88.2	91.5	74.5	83.7
Malawi	10 to 14	83.4	89.8	77.5	87.5	88.5	91.8	80.9	86.0	70.6	80.1	87.1	89.6	88.3	91.2	90.5	93.2	85.7	88.4	91.9	93.8	91.4	93.7	88.7	88.6
Malaysia	10 to 14	87.7	94.5	89.2	94.8	87.6	94.6	89.5	94.0	88.5	92.9	90.0	94.4	91.3	95.9	95.2	97.3	85.8	94.5	90.1	93.7	91.6	93.9	81.0	87.9
Maldives	10 to 14	77.6	94.7	72.4	96.0	83.2	94.3	84.9	94.9	77.3	94.6	89.0	94.9	79.5	96.1	85.6	98.0	73.2	93.2	82.3	93.2	83.4	93.9	74.8	86.3
Mali	10 to 14	83.4	84.7	73.4	74.7	90.0	91.3	86.2	87.5	71.6	71.0	92.9	94.5	89.2	88.3	90.1	88.4	89.0	89.0	89.8	87.2	91.2	90.2	82.1	76.0
Malta	10 to 14	88.6	87.0	82.6	94.0	93.5	82.6	91.2	89.5	85.1	93.6	94.5	85.7	87.2	84.2	87.9	95.9	88.6	66.7	84.5	90.1	82.8	94.9	81.9	76.4
Marshall Islands	10 to 14	86.5	77.1	80.5	71.8	91.4	82.6	88.3	82.0	82.9	77.0	91.3	85.1	79.9	69.6	80.2	72.4	83.6	70.1	85.8	77.5	85.8	78.8	86.0	73.5
Mauritania	10 to 14	84.7	90.5	76.7	85.4	90.4	94.0	86.4	91.4	71.6	81.3	93.3	96.1	88.9	92.0	90.3	92.4	87.9	91.8	86.3	89.3	90.5	92.3	70.4	76.8
Mauritius	10 to 14	90.1	93.6	92.5	95.4	89.0	92.6	91.4	94.8	90.7	94.3	91.2	94.4	89.7	91.9	95.0	96.1	81.8	85.6	86.5	91.4	90.3	92.7	72.9	83.1
Mexico	10 to 14	90.0	92.8	90.5	93.9	90.1	92.1	93.7	96.7	92.8	95.8	93.6	96.7	87.1	87.4	91.7	93.5	81.0	78.2	86.8	95.3	90.0	96.6	74.4	87.2
Micronesia (Federated States of)	10 to 14	78.3	82.1	74.3	78.5	82.6	86.0	82.4	85.5	77.3	81.5	85.4	87.8	69.7	75.4	74.6	78.6	67.5	74.3	79.3	80.9	81.2	82.2	75.2	75.8
Monaco	10 to 14	88.1	95.5	85.1	93.0	91.1	97.3	89.9	95.7	87.8	93.9	91.0	96.5	82.0	92.8	83.5	92.4	82.6	95.0	85.6	93.8	86.0	92.7	79.3	92.1
Mongolia	10 to 14	96.6	97.0	96.3	97.7	96.6	96.5	94.9	94.8	93.3	93.3	95.4	95.1	93.4	93.7	95.4	95.7	91.1	91.4	95.2	96.0	96.0	95.9	91.9	90.6
Montenegro	10 to 14	82.9	91.3	83.0	90.5	83.6	92.4	84.3	90.9	82.9	89.1	84.6	91.6	93.7	97.0	96.3	97.8	90.0	95.9	94.0	95.2	94.3	94.9	87.2	90.2
Morocco	10 to 14	71.1	90.2	58.0	86.7	83.2	93.6	72.0	89.2	55.4	84.0	82.4	92.1	74.9	89.5	77.5	90.6	77.7	90.5	68.5	83.9	62.4	80.6	75.9	84.5
Mozambique	10 to 14	90.8	93.1	91.9	95.3	90.3	91.7	88.2	90.5	87.5	91.8	88.7	90.2	93.0	93.1	96.4	97.2	88.2	87.3	95.2	95.5	95.7	96.5	89.4	87.6

Myanmar	10 to 14	63.6	80.0	63.4	83.6	66.7	78.6	73.8	84.2	68.2	84.6	77.4	83.9	57.8	77.9	74.9	88.5	35.6	63.4	79.4	85.6	84.0	89.6	63.6	70.8
Namibia	10 to 14	89.4	91.5	90.6	93.3	88.2	89.8	83.2	86.8	80.3	86.3	84.9	87.1	95.5	95.8	96.9	97.3	93.6	93.8	92.8	92.4	94.9	94.9	81.6	80.0
Nauru	10 to 14	75.8	70.7	71.4	65.4	80.6	76.8	79.9	76.2	75.3	70.1	82.8	80.1	64.3	56.8	69.7	60.6	62.2	57.4	68.4	63.6	74.7	68.7	56.5	50.8
Nepal	10 to 14	86.4	92.1	84.1	94.4	89.3	90.7	86.9	91.4	81.5	90.7	90.2	91.1	85.8	91.0	90.4	96.0	80.4	83.6	92.0	94.8	91.0	95.3	88.2	86.9
Netherlands	10 to 14	90.4	97.9	89.1	97.8	92.0	97.8	91.8	97.7	90.2	97.2	92.4	97.4	79.7	95.2	85.1	97.1	74.4	92.9	90.3	97.8	91.1	97.3	84.1	95.4
New Zealand	10 to 14	91.8	97.2	92.7	96.0	91.7	98.1	94.5	97.6	93.9	96.8	94.5	97.7	78.4	92.0	88.3	93.0	63.7	92.6	92.1	97.4	94.5	96.5	82.0	94.4
Nicaragua	10 to 14	83.3	94.3	83.2	93.9	84.4	95.0	89.2	96.2	86.9	94.8	90.1	96.5	80.9	92.4	87.4	94.6	72.8	89.8	83.4	95.1	87.5	95.1	68.7	90.9
Niger	10 to 14	82.8	87.4	74.9	79.3	88.5	93.1	86.2	88.9	73.7	76.5	92.1	94.9	88.4	90.2	90.6	90.5	86.8	90.6	90.3	91.6	92.6	92.6	81.0	84.5
Nigeria	10 to 14	90.5	91.5	87.7	90.4	92.5	92.1	90.7	92.0	84.8	87.3	93.6	94.2	94.5	93.9	95.6	95.6	93.3	91.6	92.7	89.2	94.8	94.8	83.2	69.6
Niue	10 to 14	83.5	34.0	80.2	0.0	86.8	60.9	86.6	56.0	83.0	27.3	88.6	70.5	75.0	0.0	79.4	0.0	72.1	15.7	80.2	23.5	82.9	15.3	76.5	31.7
North Macedonia	10 to 14	82.8	89.9	83.0	91.7	83.6	88.7	86.9	91.8	83.4	90.6	88.7	91.7	75.3	84.7	84.2	91.8	63.9	74.4	84.7	90.1	88.1	92.5	70.2	78.3
Northern Mariana Islands	10 to 14	90.5	93.6	87.1	91.5	93.3	95.0	92.0	94.1	89.6	92.9	93.2	94.6	86.3	89.6	87.2	90.5	89.4	90.4	86.5	90.7	88.5	92.0	83.7	88.2
Norway	10 to 14	90.9	98.6	92.2	97.4	90.5	99.2	92.8	97.4	92.7	96.4	92.2	97.7	78.4	97.3	87.4	97.2	67.1	98.1	92.5	98.0	93.6	97.3	84.6	96.4
Oman	10 to 14	84.1	95.1	89.3	95.8	82.5	95.1	81.8	91.7	86.3	91.0	77.6	91.6	90.7	95.1	93.7	94.9	86.3	95.8	85.0	92.9	86.7	91.8	76.5	88.1
Pakistan	10 to 14	89.8	83.1	86.3	79.5	93.2	87.5	89.2	83.8	83.2	76.3	92.8	88.7	87.1	77.3	89.5	82.1	84.9	73.1	93.4	88.1	91.5	87.9	91.1	81.8
Palau	10 to 14	85.4	92.1	82.1	89.9	88.2	93.8	87.2	91.8	84.4	89.7	88.9	92.9	81.8	89.2	82.5	88.7	82.9	90.6	77.6	85.8	81.0	86.7	72.1	82.1
Palestine	10 to 14	68.4	83.2	74.2	87.2	66.2	81.1	64.6	77.6	68.9	81.0	61.6	74.9	90.8	95.1	94.0	96.6	86.7	93.2	68.5	80.7	73.0	83.4	53.1	69.4
Panama	10 to 14	88.2	93.4	83.9	94.4	92.3	93.1	93.7	96.6	91.7	96.3	94.6	96.2	85.9	89.4	88.9	94.1	83.8	82.8	84.2	95.2	82.6	95.9	84.4	91.1
Papua New Guinea	10 to 14	80.6	80.8	70.9	70.8	88.8	89.0	83.3	84.0	71.9	72.7	89.3	89.7	75.4	75.4	75.9	75.9	81.0	81.6	81.0	79.9	79.3	79.2	85.7	83.7
Paraguay	10 to 14	81.4	89.2	76.8	89.1	86.5	90.0	90.4	93.7	84.9	92.4	93.4	94.0	72.7	83.6	78.9	89.0	67.6	77.3	90.6	95.3	91.0	96.1	85.9	90.1
Peru	10 to 14	74.5	80.0	76.2	81.2	75.4	80.8	84.9	86.4	83.0	84.8	85.8	87.1	69.1	73.6	83.1	83.9	51.7	61.2	85.7	90.5	84.5	88.8	84.1	88.6
Philippines	10 to 14	77.1	82.3	80.8	85.6	75.1	80.7	80.6	84.7	81.4	84.9	78.6	83.8	77.0	80.0	86.7	88.1	64.0	69.4	83.7	87.0	87.8	89.7	68.5	73.6
Poland	10 to 14	89.9	97.1	91.1	96.8	89.3	97.6	92.4	96.7	91.2	95.3	92.6	97.2	88.5	97.1	94.5	97.5	79.3	96.8	90.7	95.7	91.9	94.9	80.4	91.5
Portugal	10 to 14	63.8	94.6	57.9	95.0	71.0	94.2	73.8	96.6	66.7	97.4	78.5	95.4	60.0	92.2	70.0	95.8	50.9	87.7	48.3	95.1	58.3	95.7	21.6	90.3
Puerto Rico	10 to 14	97.4	100.0	97.1	99.5	97.6	100.0	98.6	100.0	97.7	99.2	98.7	100.0	94.9	98.6	96.6	98.9	93.1	98.7	98.2	99.9	98.6	99.9	97.0	100.0
Qatar	10 to 14	86.0	96.5	91.7	99.2	84.1	94.7	87.6	95.4	88.4	96.0	87.1	94.3	81.5	94.6	92.9	97.8	61.3	88.9	92.4	97.0	93.4	97.2	87.3	92.3
Republic of Korea	10 to 14	81.8	96.3	81.0	95.9	83.4	96.5	89.6	96.9	88.2	96.2	90.1	96.8	70.7	94.3	82.7	96.3	54.8	91.9	79.5	94.4	84.2	94.6	65.0	90.4
Republic of Moldova	10 to 14	91.4	97.1	90.5	97.3	92.7	97.2	87.2	94.6	83.4	92.9	89.5	95.3	96.0	98.6	98.1	99.3	92.2	97.1	93.1	96.1	92.3	95.1	88.7	91.9
Romania	10 to 14	87.1	94.1	88.0	94.7	86.7	93.7	88.9	95.8	87.6	94.9	88.9	95.8	84.8	90.1	90.5	94.1	77.9	85.0	87.4	93.3	90.8	93.8	72.5	85.9
Russian Federation	10 to 14	94.8	96.3	95.6	97.4	94.3	95.3	92.8	94.4	91.3	93.8	93.2	93.7	94.0	95.3	96.7	97.5	89.9	91.7	93.1	95.5	92.8	94.9	86.8	90.2
Rwanda	10 to 14	66.2	92.8	57.3	91.5	75.1	93.9	67.0	89.9	50.9	86.0	76.9	92.1	77.4	94.5	84.2	95.9	69.4	92.4	87.0	96.3	85.4	95.4	85.0	93.2
Saint Kitts and Nevis	10 to 14	80.8	93.8	81.2	94.2	80.5	93.3	87.1	95.6	85.6	95.0	87.0	95.5	75.0	89.3	84.5	94.4	62.5	82.1	84.7	96.1	88.7	96.5	73.7	93.6
Saint Lucia	10 to 14	95.2	95.2	96.7	97.0	93.4	93.1	96.1	97.5	96.2	97.9	95.0	96.3	91.6	90.9	95.9	96.6	85.0	82.0	96.7	97.3	97.9	98.2	91.6	93.9
Saint Vincent and the Grenadines	10 to 14	86.8	92.8	79.5	89.8	94.0	95.5	90.2	94.9	83.4	92.5	95.6	96.1	83.6	90.1	85.1	91.2	85.9	91.1	89.8	94.3	88.3	93.7	95.2	95.1
Samoa	10 to 14	84.2	87.5	82.1	84.6	86.7	90.5	86.6	88.9	84.2	85.4	88.1	90.7	76.7	81.4	81.0	83.2	74.1	82.1	84.3	85.8	86.0	86.7	80.8	82.4
San Marino	10 to 14	93.4	98.7	91.7	97.9	94.4	99.0	92.8	97.7	90.6	96.6	93.8	98.0	92.7	98.7	94.8	99.0	90.6	98.6	91.0	97.5	92.3	96.8	83.7	95.0
Sao Tome and Principe	10 to 14	84.9	95.2	87.5	94.8	83.6	94.8	89.0	95.4	87.3	92.7	89.9	96.3	88.4	94.3	94.1	96.2	81.7	92.0	81.1	92.7	93.7	95.8	47.3	78.6
Saudi Arabia	10 to 14	75.9	95.4	73.2	95.3	79.9	95.7	72.8	93.8	66.6	92.4	76.7	94.3	90.5	97.2	92.4	97.4	88.9	97.1	75.3	92.5	74.9	92.0	69.9	88.5
Senegal	10 to 14	81.8	88.4	74.3	83.2	87.1	91.7	84.0	90.0	70.5	79.0	90.8	94.5	86.0	88.8	88.7	90.6	83.6	86.9	83.2	87.6	89.1	91.8	63.8	72.3
Serbia	10 to 14	83.2	97.1	83.1	97.7	83.7	96.3	88.8	97.6	87.0	97.2	89.5	97.3	75.9	96.0	84.3	98.0	65.7	93.0	83.2	94.4	88.1	94.9	65.8	88.2
Seychelles	10 to 14	94.7	98.3	92.4	97.4	95.7	99.0	93.8	96.6	90.6	94.8	95.4	97.4	94.0	98.8	95.1	98.5	93.6	99.7	91.7	95.4	91.4	94.2	86.9	92.4
Sierra Leone	10 to 14	78.3	78.0	69.4	66.1	84.4	87.2	84.3	83.3	69.9	63.3	90.8	93.2	84.2	81.7	87.2	82.9	81.9	82.1	77.1	74.1	86.8	82.5	49.7	46.0
Singapore	10 to 14	89.3	97.6	87.3	96.4	91.5	98.4	92.5	97.8	90.5	96.8	93.4	98.1	90.1	97.5	93.5	97.8	86.2	97.8	83.9	95.3	83.3	94.7	80.4	93.1
Slovakia	10 to 14	91.4	97.1	91.7	97.1	91.5	97.1	92.0	96.8	90.3	95.7	92.6	97.0	90.2	96.8	93.8	97.6	85.7	95.7	90.6	95.0	92.0	94.7	80.6	89.8
Slovenia	10 to 14	96.4	99.0	97.4	98.8	95.4	99.2	96.8	98.7	96.7	97.5	96.3	99.0	96.3	99.6	98.3	99.4	92.9	100.0	93.9	96.3	94.8	95.5	86.3	92.8
Solomon Islands	10 to 14	90.9	90.5	86.2	85.6	94.6	94.6	91.3	90.5	86.1	84.7	94.0	93.6	84.8	85.1	85.0	84.8	89.1	89.8	88.5	88.3	89.1	88.1	90.3	90.1
Somalia	10 to 14	84.8	90.3	82.2	89.3	87.6	91.5	81.5	86.4	73.7	81.7	85.9	89.3	89.8	92.3	93.4	95.1	85.4	88.5	95.6	96.7	94.2	95.7	92.6	93.0
South Sudan	10 to 14	93.6	91.2	80.9	80.0	88.0	87.7	87.7	84.4	74.4	70.9	86.9	86.3	97.4	95.7	93.5	91.6	88.2	85.1	95.0	91.5	92.6	91.6	90.0	88.0

Spain	10 to 14	84.6	83.9	82.3	95.3	88.7	97.4	82.4	80.8	87.4	96.9	92.4	97.2	91.2	88.7	86.7	95.4	74.1	94.0	93.3	92.2	84.2	95.4	75.4	94.2
Sri Lanka	10 to 14	85.5	96.6	85.7	94.3	88.1	94.6	90.7	97.4	83.8	92.0	89.3	94.2	80.6	94.5	94.6	97.4	89.1	95.5	83.7	96.3	85.8	91.0	71.7	82.5
Sudan	10 to 14	86.5	94.3	41.7	74.2	75.0	85.1	87.3	93.7	39.7	71.6	73.4	83.1	91.9	96.4	71.8	82.2	71.0	77.8	83.1	90.1	58.5	70.5	76.9	70.1
Suriname	10 to 14	59.1	79.5	77.4	79.7	88.2	95.8	60.2	78.7	83.5	84.3	92.0	96.4	68.6	78.8	82.6	83.1	76.2	90.5	66.8	72.8	90.6	90.9	88.8	96.1
Sweden	10 to 14	82.8	88.1	90.9	98.4	93.8	98.5	88.5	91.3	96.5	99.7	95.5	97.9	78.3	84.2	89.0	97.6	82.0	95.1	90.1	92.6	91.1	97.5	88.4	95.7
Switzerland	10 to 14	92.5	98.6	87.8	97.0	90.0	98.4	96.1	98.9	90.6	97.3	92.3	98.1	85.5	96.5	85.6	96.5	73.6	95.5	91.7	98.4	89.7	97.0	76.3	95.5
Syrian Arab Republic	10 to 14	88.6	97.9	53.0	84.5	52.3	78.5	91.9	98.1	49.5	76.1	49.2	69.7	79.7	95.8	88.0	96.3	74.5	91.7	87.0	97.7	57.8	82.5	50.7	70.8
Taiwan (Province of China)	10 to 14	48.8	79.6	95.6	97.5	95.3	98.0	47.9	71.7	95.0	98.5	95.1	97.6	81.9	94.9	97.6	98.2	96.1	97.7	58.9	81.1	93.5	94.8	86.7	90.7
Tajikistan	10 to 14	95.4	98.0	88.3	92.7	89.4	93.4	95.3	98.2	83.0	88.4	89.6	93.2	96.8	97.8	90.1	93.3	87.4	91.1	92.6	94.6	92.7	94.1	54.1	72.7
Thailand	10 to 14	88.4	92.8	83.5	92.6	85.4	89.0	87.1	91.5	86.1	91.2	91.3	91.4	88.2	91.9	86.3	93.7	63.8	77.3	84.3	90.5	91.2	93.4	83.5	85.9
Timor-Leste	10 to 14	83.8	90.2	78.3	83.7	82.8	81.5	89.5	91.5	81.3	84.4	87.8	86.4	76.5	87.0	86.5	88.9	70.2	67.2	90.5	92.8	88.5	89.8	78.0	73.6
Togo	10 to 14	79.9	81.7	76.9	81.5	88.5	89.8	85.4	85.8	73.1	76.8	92.2	93.1	79.0	79.6	89.9	90.4	86.0	86.2	86.8	86.8	90.6	91.3	66.9	64.5
Tokelau	10 to 14	83.8	86.4	77.2	13.4	85.9	60.8	86.4	88.2	82.2	40.7	88.5	71.8	87.7	88.2	79.1	20.9	76.5	28.6	84.7	85.0	81.9	29.3	75.7	35.9
Tonga	10 to 14	81.7	37.5	89.8	89.2	95.0	95.0	86.2	59.7	90.0	89.7	94.6	94.4	76.2	16.9	89.7	89.0	90.5	90.7	79.1	31.1	90.0	88.6	87.1	86.0
Trinidad and Tobago	10 to 14	92.7	92.4	86.7	94.8	86.8	95.3	93.1	92.9	89.9	96.1	91.9	97.7	88.9	88.8	87.0	93.0	68.9	84.0	88.9	88.0	90.6	98.0	78.5	95.1
Tropical Latin America	10 to 14	86.7	95.1	84.8	87.2	85.1	87.2	91.6	97.4	87.2	89.5	89.8	91.4	78.9	88.9	86.7	87.8	70.3	71.6	87.6	97.5	92.1	96.0	74.9	89.9
Tunisia	10 to 14	80.2	94.5	78.3	94.4	83.4	95.0	80.0	92.9	75.5	91.3	82.8	93.5	85.6	94.8	89.1	95.8	82.6	94.1	80.8	91.4	79.8	90.8	77.1	86.8
Turkey	10 to 14	79.2	94.1	81.4	94.9	79.4	93.8	83.8	93.4	83.0	92.6	84.2	93.4	69.5	91.4	83.1	94.7	49.6	86.9	84.6	93.9	85.9	93.8	78.3	90.0
Turkmenistan	10 to 14	95.0	95.7	95.3	96.6	95.3	95.4	93.1	93.4	90.5	91.1	94.5	94.5	93.4	92.3	94.7	94.3	92.3	90.0	90.9	93.4	94.2	95.0	76.6	81.0
Tuvalu	10 to 14	69.6	79.5	70.0	78.6	71.5	82.0	76.1	84.0	74.7	82.0	77.5	85.3	61.8	71.3	71.6	77.0	52.1	66.6	72.2	78.2	79.0	81.9	57.5	67.1
Uganda	10 to 14	87.7	91.0	87.2	89.9	88.6	92.1	84.3	87.7	80.1	83.8	86.9	90.1	90.7	92.5	94.7	95.1	85.5	89.0	94.8	95.0	94.4	94.5	91.0	91.1
Ukraine	10 to 14	95.4	97.5	95.6	97.7	95.4	97.4	91.6	94.6	89.9	93.2	91.9	95.0	97.3	98.2	97.9	98.7	96.4	97.1	96.4	96.8	95.6	95.9	91.1	92.1
United Arab Emirates	10 to 14	82.8	95.9	90.3	95.9	79.0	96.2	82.5	95.0	88.9	94.0	78.3	95.1	79.3	92.9	89.0	94.1	64.1	92.7	85.0	95.2	89.9	94.4	70.7	92.5
United Kingdom	10 to 14	91.0	98.4	89.7	98.0	92.3	98.4	92.2	97.3	90.2	96.8	93.1	97.2	80.1	97.4	86.3	98.2	73.2	96.2	91.3	98.0	92.8	97.6	85.0	95.8
United Republic of Tanzania	10 to 14	87.4	91.5	85.6	90.8	89.2	92.3	85.3	88.5	80.4	85.1	88.2	90.5	91.3	91.5	94.2	94.5	87.3	87.4	94.9	94.1	94.3	94.5	91.6	88.1
United States Virgin Islands	10 to 14	88.0	99.4	93.7	100.0	82.4	98.4	93.5	99.6	95.7	99.4	90.4	99.0	82.7	97.6	93.8	99.4	63.6	95.0	94.3	99.9	97.6	100.0	84.1	98.6
United States of America	10 to 14	95.9	97.0	93.5	94.9	97.1	97.8	98.3	99.5	98.5	100.0	97.7	98.7	88.8	90.8	92.6	94.4	84.1	86.3	92.7	95.2	94.6	96.5	91.4	94.6
Uruguay	10 to 14	84.0	94.8	85.4	94.3	84.0	95.3	91.8	96.4	91.0	95.6	91.6	96.4	68.3	90.2	81.3	93.6	51.5	86.1	86.8	95.1	88.1	94.0	77.7	92.3
Uzbekistan	10 to 14	91.0	96.6	91.7	97.6	91.1	95.8	87.9	94.7	84.4	93.4	89.9	95.0	94.4	96.7	96.1	98.0	91.9	94.4	87.2	93.0	93.6	95.0	62.6	80.2
Vanuatu	10 to 14	83.1	83.1	80.3	79.4	86.1	86.9	85.0	84.6	81.6	80.2	87.0	87.2	72.3	73.6	77.1	75.4	70.3	74.5	81.8	81.8	84.2	83.0	76.8	77.1
Venezuela (Bolivarian Republic of)	10 to 14	86.0	87.7	87.4	90.9	85.7	85.7	91.0	94.3	90.3	93.5	90.7	93.9	83.5	79.7	89.7	90.5	75.1	62.4	85.8	92.4	90.0	94.5	71.4	82.9
Viet Nam	10 to 14	93.3	94.5	95.3	97.1	92.6	93.1	94.0	94.5	92.6	94.7	94.3	93.8	93.1	94.5	97.1	98.1	87.5	89.1	93.5	93.4	94.1	94.4	87.5	85.6
Yemen	10 to 14	70.2	81.1	58.4	75.8	81.0	86.4	70.0	81.2	55.1	74.2	79.3	85.4	78.4	82.7	80.6	85.5	79.7	82.0	76.3	76.1	69.2	73.3	83.7	75.5
Zambia	10 to 14	86.4	91.7	81.9	90.3	90.3	92.8	84.4	88.7	75.1	84.6	89.6	91.1	91.6	93.7	93.3	95.4	89.4	91.0	94.8	95.3	93.8	94.9	92.4	91.1
Zimbabwe	10 to 14	95.7	87.4	95.9	87.7	95.1	87.3	91.6	80.6	89.7	75.0	92.7	84.2	95.6	89.0	96.3	91.3	94.2	84.9	96.7	91.9	96.7	93.5	91.7	81.6
Afghanistan	15 to 19	46.1	50.4	57.6	59.5	46.0	52.3	36.0	39.5	42.5	42.3	41.5	48.8	80.6	79.5	85.6	83.3	74.8	76.0	45.9	26.7	47.3	34.7	65.4	40.9
Albania	15 to 19	75.0	85.4	86.3	92.5	70.3	84.7	75.4	85.2	79.9	86.3	75.0	87.0	80.1	91.2	89.0	94.3	70.9	89.2	84.5	87.5	89.9	91.2	82.1	87.5
Algeria	15 to 19	66.7	80.0	76.2	86.4	66.4	81.1	60.1	75.4	63.2	76.1	65.2	79.5	88.4	94.5	90.6	94.4	86.0	95.1	55.1	68.0	62.6	73.2	60.1	72.5
American Samoa	15 to 19	73.5	75.1	87.6	85.5	64.7	70.8	63.1	66.6	75.3	74.2	61.4	67.0	87.0	85.2	89.6	86.4	84.3	84.0	85.2	82.9	85.8	82.7	88.0	89.3
Andorra	15 to 19	83.4	91.9	86.8	94.8	85.4	92.8	85.8	93.4	85.0	92.5	89.0	95.0	74.6	90.1	73.6	89.3	78.8	92.9	84.4	93.4	87.2	94.3	85.9	94.3
Angola	15 to 19	58.9	69.8	69.3	76.5	57.4	70.7	50.7	61.0	51.8	58.8	59.1	70.3	73.3	78.6	80.4	82.7	68.4	75.9	76.9	77.1	81.8	81.5	77.9	79.5
Antigua and Barbuda	15 to 19	75.5	85.4	83.4	91.0	71.6	82.5	78.6	87.7	79.2	88.6	81.6	87.9	72.3	84.2	83.2	89.3	59.8	79.0	80.9	86.5	83.9	88.0	83.1	89.0
Argentina	15 to 19	58.3	78.4	71.1	88.7	55.3	74.7	72.3	82.2	75.0	85.0	75.1	82.6	34.8	73.5	54.1	83.8	16.7	63.7	70.8	86.7	79.8	91.8	67.9	83.9
Armenia	15 to 19	77.3	86.2	87.4	94.5	75.8	85.0	75.4	86.1	77.8	86.1	77.3	88.4	83.3	91.4	91.6	95.9	74.8	87.6	67.7	81.6	76.3	88.6	68.1	78.8
Australia	15 to 19	82.2	90.2	88.9	95.8	80.9	89.2	86.9	95.0	89.9	95.2	87.0	95.2	70.9	89.7	81.2	93.2	59.7	86.5	81.6	91.7	86.8	94.4	80.5	91.9
Austria	15 to 19	81.5	93.2	90.5	96.9	78.3	92.8	82.1	93.6	86.7	93.2	81.1	94.6	77.0	92.3	84.5	94.0	70.8	92.0	76.2	95.0	87.2	96.4	69.1	94.5
Azerbaijan	15 to 19	68.5	80.4	81.1	89.5	65.1	79.0	59.7	74.6	66.0	77.4	60.9	76.5	90.3	93.7	93.9	96.1	87.8	92.4	71.5	81.3	80.2	86.9	69.5	80.8
Bahamas	15 to 19	77.3	88.0	84.7	92.5	73.4	86.0	78.0	89.4	80.0	89.5	79.1	90.1	77.5	86.1	85.3	90.2	68.4	81.6	81.0	91.0	84.8	92.3	81.1	91.8

Bahrain	15 to 19	65.8	82.3	74.0	91.0	67.0	80.3	60.7	79.7	63.9	83.3	64.6	80.2	87.0	95.8	87.6	96.6	86.5	95.0	59.4	75.6	64.0	82.5	68.6	74.3
Bangladesh	15 to 19	33.1	57.1	58.0	71.4	16.4	50.1	38.1	57.7	52.7	64.7	34.4	58.1	32.3	58.8	63.6	71.2	0.0	46.5	56.6	66.4	67.4	74.9	55.8	66.3
Barbados	15 to 19	76.6	88.5	81.3	91.9	77.0	88.2	81.2	92.6	79.7	90.7	86.3	95.1	72.8	84.7	78.8	87.7	67.7	82.7	71.9	88.9	74.8	90.2	78.7	90.2
Belarus	15 to 19	77.4	86.6	87.4	93.6	74.3	86.5	76.5	87.1	80.5	87.2	75.8	88.8	81.8	89.9	86.8	93.0	77.6	87.6	61.1	83.7	72.5	87.5	57.2	85.9
Belgium	15 to 19	74.1	92.4	85.1	95.8	69.7	92.2	73.1	94.7	79.3	93.9	72.6	95.5	66.9	90.1	76.0	91.8	59.9	90.1	70.4	94.1	82.7	95.6	62.3	93.8
Belize	15 to 19	70.7	85.4	86.0	91.0	57.3	83.1	73.4	85.2	80.5	86.7	69.8	85.4	68.8	86.6	86.8	90.2	45.3	83.3	80.6	89.3	89.8	92.2	69.3	87.4
Benin	15 to 19	80.1	81.3	78.3	80.5	84.9	86.1	78.3	79.2	64.8	66.5	92.0	93.0	91.8	91.1	87.9	86.5	96.3	96.6	83.4	81.0	89.0	86.3	81.8	80.8
Bermuda	15 to 19	90.9	96.0	95.1	98.5	89.9	96.0	92.5	97.1	91.0	94.2	94.9	100.0	91.6	97.4	94.4	98.1	88.9	97.2	90.5	97.3	92.7	97.9	89.6	96.1
Bhutan	15 to 19	72.2	78.3	75.1	87.0	78.1	76.7	69.7	76.1	65.8	78.2	79.8	78.4	75.9	81.1	76.7	86.6	77.3	76.8	76.5	79.7	76.9	85.5	85.2	78.8
Bolivia (Plurinational State of)	15 to 19	0.0	39.7	5.0	48.5	14.1	43.4	19.2	50.4	15.5	52.2	38.9	58.1	0.0	33.1	3.6	36.2	4.9	35.4	30.4	61.8	38.8	68.9	45.1	65.8
Bosnia and Herzegovina	15 to 19	79.9	83.8	89.8	93.0	77.1	82.5	89.7	91.9	87.8	90.0	93.0	94.8	72.5	87.1	83.5	91.2	60.4	83.6	76.5	79.5	86.1	87.8	72.3	77.5
Botswana	15 to 19	59.8	77.5	81.5	88.7	42.2	71.3	39.4	65.1	57.2	72.3	38.2	66.8	88.4	93.4	93.9	95.7	83.2	91.3	80.5	84.5	89.1	90.6	71.1	79.6
Brazil	15 to 19	60.6	67.6	71.9	78.7	57.8	63.4	64.3	70.8	69.0	76.5	65.6	69.6	62.2	64.6	68.8	71.7	57.8	59.3	61.9	75.9	75.5	83.9	53.0	71.8
Brunei Darussalam	15 to 19	69.4	91.1	83.9	95.0	61.9	90.8	74.5	92.6	81.9	91.7	72.6	94.1	60.0	91.0	74.9	92.4	44.4	90.5	66.7	89.9	80.0	91.8	58.5	90.7
Bulgaria	15 to 19	58.0	73.8	72.2	86.0	51.6	67.6	61.8	75.6	67.8	81.2	63.5	73.6	64.4	73.5	75.8	82.3	53.4	65.4	50.0	68.2	69.5	82.4	35.1	55.6
Burkina Faso	15 to 19	82.7	80.9	84.4	82.0	84.2	83.6	81.6	78.6	73.9	69.5	90.5	89.4	95.0	93.4	93.5	91.3	96.6	95.5	87.1	83.6	92.3	89.5	85.2	81.6
Burundi	15 to 19	3.7	48.9	20.9	54.2	3.0	54.2	0.0	37.6	0.8	29.9	16.7	54.7	33.6	66.1	51.7	70.6	20.0	62.8	45.0	66.1	48.7	67.9	60.3	77.4
Cabo Verde	15 to 19	80.2	81.7	79.5	89.2	84.6	79.2	82.0	83.0	68.5	79.4	96.3	88.1	89.3	91.5	87.5	93.2	91.9	90.4	76.7	72.1	85.6	89.1	72.0	58.6
Cambodia	15 to 19	57.7	68.0	63.2	78.8	62.6	65.5	57.5	66.9	56.0	70.8	68.0	70.1	69.7	78.8	72.5	83.9	69.4	74.9	68.0	73.5	72.1	80.0	75.6	74.1
Cameroon	15 to 19	69.6	71.4	70.3	71.9	74.0	76.3	65.7	69.4	55.8	57.7	79.7	83.3	86.2	84.5	86.3	82.8	86.6	86.7	79.4	72.4	86.9	81.5	76.2	69.5
Canada	15 to 19	91.6	97.3	94.1	98.2	92.1	96.9	93.7	97.2	92.8	95.6	94.9	98.2	84.6	91.7	87.9	94.2	81.8	89.4	90.1	97.9	92.0	97.8	89.8	96.8
Central African Republic	15 to 19	43.6	52.4	63.3	64.9	35.1	50.0	34.7	43.6	42.2	43.2	41.5	54.0	62.3	65.6	78.0	77.0	50.0	57.1	69.8	71.4	79.1	79.0	66.0	70.4
Chad	15 to 19	74.7	70.6	78.0	72.4	75.7	74.1	72.4	68.5	64.0	58.0	82.9	81.3	87.4	83.0	88.7	82.9	86.8	83.7	79.4	72.0	89.3	83.7	72.9	65.5
Chile	15 to 19	72.2	84.2	80.6	91.7	71.7	82.1	80.1	88.5	81.8	89.5	81.9	88.9	59.5	77.0	69.0	83.9	51.5	71.3	73.1	87.1	80.3	91.9	72.3	84.8
China	15 to 19	65.9	79.1	82.2	90.9	54.3	73.0	71.1	76.4	82.0	85.2	66.3	73.3	53.4	85.3	70.2	91.1	37.1	80.3	74.1	78.1	84.8	87.0	64.5	72.5
Colombia	15 to 19	64.7	77.3	73.9	87.3	63.3	72.8	75.5	87.4	76.1	88.0	79.0	88.0	58.8	69.7	66.9	80.4	52.1	58.7	54.3	81.6	71.3	89.6	41.8	74.7
Comoros	15 to 19	42.5	62.6	49.5	66.0	47.2	68.1	33.7	51.7	27.1	44.0	50.7	67.0	65.4	74.9	71.5	76.7	61.4	74.2	63.9	72.6	67.2	74.9	73.0	80.0
Congo	15 to 19	60.9	69.8	75.2	75.3	55.5	71.4	52.8	60.2	57.9	56.7	58.4	70.6	74.9	78.8	84.4	82.0	67.4	76.8	74.9	75.2	83.7	80.3	70.2	76.8
Cook Islands	15 to 19	65.4	84.2	78.5	91.5	59.1	80.9	57.2	76.0	67.2	80.7	57.4	76.0	77.7	94.0	79.4	94.2	76.4	93.1	74.2	85.1	73.9	85.2	84.4	90.7
Costa Rica	15 to 19	83.2	88.8	89.2	93.9	83.2	89.0	88.9	94.8	87.3	92.3	92.2	97.7	80.6	86.8	85.2	90.6	77.1	83.8	82.0	91.0	87.0	93.3	80.9	91.2
Coted'Ivoire	15 to 19	71.7	74.1	76.2	75.9	72.1	77.3	69.7	73.9	60.7	61.3	80.2	86.2	84.7	84.6	86.3	82.9	83.7	86.6	71.5	68.8	86.7	82.1	61.9	62.0
Croatia	15 to 19	78.9	89.3	87.3	94.9	76.3	88.9	86.6	95.2	88.5	93.2	86.0	97.5	74.1	91.1	80.4	93.3	68.7	89.7	74.3	86.7	83.4	90.8	69.9	86.1
Cuba	15 to 19	79.9	88.2	86.1	94.0	77.9	84.1	81.4	90.4	82.5	91.7	83.1	89.1	81.7	88.4	86.0	92.5	77.7	83.8	76.3	87.8	83.1	92.7	72.5	82.0
Cyprus	15 to 19	83.7	95.4	90.2	99.3	81.6	94.2	92.3	95.8	93.9	95.5	91.6	96.2	74.5	96.7	81.9	98.6	69.1	96.0	85.6	96.7	89.9	98.0	84.3	95.6
Czechia	15 to 19	80.0	89.0	89.1	95.1	77.5	88.5	85.6	92.5	86.4	90.9	86.8	95.2	77.4	90.8	83.9	93.6	71.5	89.0	70.8	86.3	82.4	90.8	63.6	85.0
Democratic People's Republic of Korea	15 to 19	65.6	64.7	79.9	83.0	54.5	53.6	66.8	62.1	76.4	75.6	62.6	57.5	64.8	72.3	76.1	84.0	50.8	60.7	70.4	68.6	81.1	82.7	61.7	57.9
Democratic Republic of the Congo	15 to 19	61.7	68.8	68.5	75.9	63.3	69.7	54.7	60.1	50.8	57.1	65.6	69.8	75.6	80.2	81.2	85.0	72.1	76.9	75.6	80.0	79.7	84.1	78.4	81.6
Denmark	15 to 19	84.2	92.3	88.0	96.3	83.5	91.7	86.2	94.6	87.5	94.3	87.2	95.2	73.1	90.4	76.5	92.7	72.1	89.5	82.7	94.4	86.9	95.9	81.6	94.0
Djibouti	15 to 19	58.9	70.5	75.8	84.4	52.9	66.1	50.5	61.9	58.0	68.9	55.5	65.5	72.4	76.8	86.2	88.8	62.7	69.1	80.5	80.8	86.2	88.4	79.5	77.7
Dominica	15 to 19	75.2	80.2	83.9	86.0	70.4	78.8	77.3	82.9	79.4	82.4	78.4	86.1	74.4	79.7	84.3	85.5	63.4	74.3	77.3	79.0	83.1	83.4	75.2	79.2
Dominican Republic	15 to 19	64.9	63.3	75.0	80.7	59.4	48.1	67.2	63.9	71.4	75.5	67.4	55.2	63.6	64.9	72.7	77.8	53.4	49.6	71.1	60.4	81.1	81.4	62.4	33.7
Ecuador	15 to 19	34.0	67.2	57.1	79.1	22.1	64.3	36.6	73.2	51.1	78.0	33.8	73.7	46.9	63.4	63.2	71.3	31.7	57.7	38.5	78.6	58.6	85.4	28.3	76.9
Egypt	15 to 19	32.2	64.6	56.4	79.5	12.8	58.2	12.2	56.5	38.0	70.6	0.0	52.7	72.5	87.9	84.8	93.7	56.0	80.9	0.0	23.3	19.5	44.7	6.8	17.9
El Salvador	15 to 19	50.8	70.6	66.2	82.6	44.4	64.3	62.9	76.2	68.5	81.2	63.6	74.4	51.3	69.8	63.7	78.9	39.3	60.9	44.3	69.9	66.7	83.8	26.3	56.6
Equatorial Guinea	15 to 19	50.7	75.5	67.1	81.3	44.1	76.3	42.8	68.6	48.0	66.5	49.7	75.6	65.8	84.6	80.2	87.0	54.2	83.4	71.2	75.4	80.3	80.4	67.3	76.5
Eritrea	15 to 19	19.8	48.0	55.8	66.9	4.2	41.5	14.6	37.5	34.0	44.4	18.9	44.5	48.9	64.5	78.5	80.4	27.2	52.9	69.1	71.9	78.2	80.5	67.0	69.8
Estonia	15 to 19	75.5	87.8	87.9	94.8	70.7	87.8	74.6	87.2	80.2	87.2	72.6	89.3	80.8	93.1	91.3	95.5	69.3	91.4	62.8	86.8	75.8	90.3	57.5	87.8
Eswatini	15 to 19	58.6	54.2	78.9	75.9	40.8	39.1	34.6	32.5	53.0	49.1	31.6	33.7	89.5	85.9	93.4	90.8	85.8	81.7	82.1	73.1	88.6	83.5	76.7	64.8

Ethiopia	15 to 19	36.8	64.1	55.1	71.3	31.3	65.4	33.0	54.6	39.2	51.2	40.8	65.4	62.5	78.8	76.2	83.6	53.0	75.7	77.3	79.8	80.2	82.0	82.6	85.2
Fiji	15 to 19	62.6	66.1	76.2	76.6	56.5	63.3	52.8	55.6	64.4	64.0	51.5	56.9	72.1	73.9	74.5	75.1	71.4	75.0	79.0	76.8	79.5	76.9	85.7	85.8
Finland	15 to 19	73.3	90.9	81.0	94.8	72.6	90.6	78.9	92.9	82.4	92.5	79.5	94.0	61.2	85.0	66.0	87.9	59.5	84.1	63.4	93.6	75.4	95.1	59.3	93.1
France	15 to 19	82.8	92.3	88.9	96.4	80.6	90.9	83.4	95.4	86.5	97.7	83.3	94.1	78.5	88.5	81.6	91.4	77.5	87.2	83.1	95.3	89.3	96.7	78.9	94.0
Gabon	15 to 19	70.3	77.4	81.2	87.1	66.8	73.7	63.5	70.4	66.9	75.3	68.6	73.5	82.7	85.9	89.2	91.2	77.3	81.4	81.0	81.0	86.0	86.9	80.2	79.0
Gambia	15 to 19	71.9	65.8	75.8	69.3	72.9	68.6	68.2	60.9	60.0	50.7	79.3	74.9	85.3	77.7	86.4	79.5	85.0	76.8	73.5	61.3	86.1	77.0	65.4	52.5
Georgia	15 to 19	71.3	82.5	82.1	93.1	70.5	78.9	67.9	82.4	70.5	85.1	71.6	82.9	81.4	83.3	86.4	92.0	77.9	74.9	72.0	80.1	78.6	88.2	73.7	74.9
Germany	15 to 19	75.2	91.8	85.3	96.6	71.4	90.3	78.0	92.5	83.0	93.2	77.4	92.4	56.9	87.9	67.8	91.4	48.4	85.9	65.6	93.7	80.3	96.0	55.6	92.2
Ghana	15 to 19	64.5	75.8	65.3	81.6	70.1	76.5	62.0	74.2	49.0	66.2	78.8	84.3	79.6	85.6	78.7	85.9	81.0	85.8	64.5	71.3	78.2	84.9	57.4	64.3
Greece	15 to 19	71.3	86.7	81.2	91.4	68.9	86.3	67.4	86.5	72.2	87.7	69.8	87.8	77.7	86.3	84.3	89.4	72.9	84.8	66.8	90.2	77.8	92.4	62.0	90.1
Greenland	15 to 19	77.9	96.6	63.7	93.8	97.5	100.0	80.5	96.1	74.7	93.9	91.9	97.9	18.5	86.3	0.0	81.6	61.4	95.7	96.9	100.0	91.6	98.2	99.7	100.0
Grenada	15 to 19	68.7	87.7	81.4	91.6	59.3	87.7	75.1	89.1	78.2	88.7	75.2	90.7	60.6	86.3	79.2	88.2	38.5	85.9	62.0	89.5	73.9	91.6	53.7	89.2
Guam	15 to 19	73.4	80.8	90.5	93.0	62.5	72.0	63.7	69.9	79.0	81.4	59.2	65.3	83.0	89.1	90.1	93.2	76.8	85.3	81.8	86.3	86.5	89.2	79.0	85.7
Guatemala	15 to 19	49.5	65.6	56.1	76.2	55.6	63.1	52.7	68.6	51.6	70.6	63.5	72.2	74.9	71.3	79.4	80.0	72.3	63.3	46.2	71.1	54.0	80.3	55.7	66.7
Guinea	15 to 19	70.1	68.4	66.7	66.6	77.6	76.1	69.2	67.1	55.0	52.4	85.5	83.9	86.1	82.6	82.9	79.2	89.7	86.4	73.3	66.0	81.1	76.5	71.5	62.6
Guinea-Bissau	15 to 19	46.8	56.2	50.5	57.7	51.1	62.0	44.9	51.9	34.3	37.8	61.4	70.1	66.0	68.3	70.7	69.3	63.4	68.8	54.2	55.2	73.9	72.7	41.8	45.3
Guyana	15 to 19	58.0	71.0	70.6	79.9	49.9	67.0	58.4	72.0	63.5	73.6	60.2	75.3	64.8	70.1	78.4	80.0	51.0	60.3	55.4	68.9	71.8	77.8	39.8	64.1
Haiti	15 to 19	9.3	30.7	0.0	22.2	44.4	59.7	26.7	43.0	16.5	32.2	60.7	72.9	6.8	24.4	1.3	13.4	28.2	49.1	2.1	28.3	0.0	28.0	56.2	65.5
Honduras	15 to 19	45.1	64.8	49.3	70.8	52.9	66.9	57.4	75.1	54.8	73.7	69.6	81.1	37.5	53.6	42.7	58.1	37.7	53.1	43.9	70.5	53.1	77.7	50.3	69.6
Hungary	15 to 19	79.3	89.2	90.1	95.3	73.9	88.6	81.9	92.4	85.4	91.0	81.0	94.6	81.0	92.1	88.0	94.7	74.2	90.5	71.1	86.6	85.1	91.2	56.2	84.7
Iceland	15 to 19	74.6	92.8	84.3	98.9	71.6	89.9	78.6	93.6	83.9	94.5	78.5	93.0	61.7	91.5	70.7	98.5	55.2	85.1	71.2	95.3	82.7	97.5	64.8	93.5
India	15 to 19	70.3	78.9	74.8	85.7	76.2	80.3	68.0	75.8	65.2	75.6	78.2	80.9	74.4	82.7	75.8	84.9	75.6	82.4	76.3	83.3	77.3	86.2	85.6	86.8
Indonesia	15 to 19	57.0	56.0	62.6	71.2	63.1	49.5	56.8	51.8	55.2	60.5	68.3	52.8	60.9	64.2	63.4	73.2	62.8	56.9	62.8	59.2	69.1	72.9	69.0	52.1
Iran (Islamic Republic of)	15 to 19	75.8	76.7	85.5	87.4	72.5	73.3	71.2	71.8	78.0	79.1	70.3	71.2	89.3	93.5	92.5	95.6	85.9	91.3	44.4	48.6	60.5	65.5	38.8	41.3
Iraq	15 to 19	57.7	66.6	72.7	83.4	52.7	58.1	53.9	59.2	62.5	72.3	55.8	57.8	84.2	90.4	87.3	93.0	81.6	87.5	34.3	45.2	48.7	66.0	35.7	31.2
Ireland	15 to 19	79.5	92.5	85.1	95.7	80.0	92.7	85.7	94.5	86.1	93.8	87.8	95.6	64.1	89.7	68.3	90.5	62.8	90.7	77.8	94.8	83.9	95.8	76.5	95.1
Israel	15 to 19	80.2	93.2	88.5	97.2	77.9	92.6	75.3	95.4	82.1	95.3	74.0	95.5	78.3	93.6	85.3	95.7	72.9	93.0	83.3	95.1	88.1	96.3	82.5	95.0
Italy	15 to 19	78.3	88.2	85.9	92.0	76.6	88.7	78.2	87.5	80.9	87.5	79.8	89.5	73.3	85.1	77.0	84.4	72.3	88.3	75.9	92.7	85.2	94.2	70.0	93.0
Jamaica	15 to 19	70.5	87.4	78.7	91.5	67.3	86.9	75.4	89.1	75.2	88.9	80.0	90.2	73.0	89.7	81.1	90.5	63.1	89.9	65.9	86.7	73.7	89.4	64.9	86.8
Japan	15 to 19	85.4	90.1	91.8	95.4	83.3	88.3	89.3	93.2	90.8	93.9	89.0	92.6	85.6	89.7	89.8	92.5	81.5	87.3	79.9	90.5	85.5	93.0	79.0	90.5
Jordan	15 to 19	64.3	83.9	72.6	91.1	64.8	82.8	50.0	78.0	55.9	82.0	53.8	78.1	95.5	99.6	95.9	99.1	95.2	99.9	40.6	71.2	48.3	78.3	50.1	70.9
Kazakhstan	15 to 19	75.4	83.5	85.3	92.7	74.2	80.5	74.0	82.5	76.6	84.1	76.2	83.6	78.5	85.6	86.3	93.5	71.3	77.3	61.1	77.6	74.0	86.3	56.6	72.1
Kenya	15 to 19	71.5	71.7	79.0	83.3	71.2	67.3	62.7	61.5	62.3	66.4	70.2	66.0	83.1	81.4	87.4	89.0	80.0	75.7	84.3	83.0	86.3	87.8	87.7	82.5
Kiribati	15 to 19	56.9	50.4	83.0	78.6	43.1	36.4	46.6	40.2	66.3	63.5	46.0	36.8	75.5	70.8	88.1	82.9	62.8	61.6	79.9	71.9	84.4	78.5	79.6	72.5
Kuwait	15 to 19	77.5	85.6	89.2	94.3	71.5	82.4	69.4	81.3	79.5	86.1	64.7	80.2	93.0	99.8	93.8	99.3	91.5	100.0	67.1	74.3	77.8	85.3	61.9	66.8
Kyrgyzstan	15 to 19	66.6	80.8	80.0	89.6	62.3	79.6	63.4	81.9	69.4	83.2	63.5	83.8	75.4	82.8	84.5	87.5	66.9	79.4	49.1	70.2	64.3	79.2	44.1	67.8
Lao People's Democratic Republic	15 to 19	27.0	49.6	39.4	61.3	27.7	48.4	32.1	51.7	34.2	54.8	44.0	58.6	36.3	60.0	50.3	68.3	23.9	53.6	33.6	49.7	47.0	63.1	38.2	47.4
Latvia	15 to 19	71.6	86.9	85.0	94.2	64.4	85.8	65.6	87.0	73.7	87.8	62.1	87.7	77.3	91.2	90.6	94.9	62.7	88.2	57.2	85.0	72.6	89.4	47.1	84.6
Lebanon	15 to 19	67.3	81.9	82.4	92.5	61.3	78.3	59.0	75.8	70.9	83.5	57.4	74.2	86.2	94.8	90.9	96.5	80.4	92.3	70.1	81.8	75.6	87.6	73.0	80.9
Lesotho	15 to 19	68.0	43.6	85.4	69.4	51.1	23.4	47.9	20.0	65.5	41.3	41.2	18.1	91.8	82.9	95.8	88.9	87.7	77.7	86.8	70.6	91.6	81.4	83.0	62.0
Liberia	15 to 19	71.1	67.0	69.1	63.4	77.5	77.3	70.9	63.4	56.5	45.9	87.3	84.3	85.2	81.2	82.7	76.5	88.4	86.3	72.5	66.3	81.5	73.6	68.9	67.7
Libya	15 to 19	72.2	68.9	78.8	80.9	71.9	64.8	64.6	60.2	66.9	67.9	69.1	60.3	91.1	88.5	91.6	90.3	90.0	87.2	59.3	43.4	65.5	62.5	64.4	32.6
Lithuania	15 to 19	81.3	88.9	90.1	95.9	79.0	87.8	77.0	88.0	80.0	88.6	77.7	88.8	81.2	92.4	89.1	96.4	73.0	88.8	75.0	88.2	82.1	91.9	73.8	87.6
Luxembourg	15 to 19	64.6	92.2	83.9	96.1	54.2	91.5	66.2	94.1	80.8	94.4	62.3	94.2	61.4	91.2	77.0	92.9	47.5	91.1	50.0	93.8	78.4	95.4	27.2	93.4
Madagascar	15 to 19	7.6	20.6	33.2	34.7	0.0	20.8	10.4	12.0	17.4	7.8	21.3	29.8	31.0	35.3	55.4	50.7	13.5	24.1	47.8	50.9	62.1	62.0	44.8	52.1
Malawi	15 to 19	48.8	54.9	56.9	66.9	51.0	52.6	37.3	41.1	34.9	42.5	50.8	50.9	65.4	65.6	72.7	75.1	60.0	58.3	71.4	69.1	75.3	76.7	76.2	69.1
Malaysia	15 to 19	67.7	78.6	82.3	89.6	62.0	74.2	69.0	76.7	75.5	82.1	70.0	76.7	83.4	90.7	89.5	93.6	77.2	88.5	68.9	78.6	79.7	86.5	63.7	74.5
Maldives	15 to 19	60.7	77.5	69.7	89.6	61.8	73.7	66.3	77.4	66.3	82.9	72.9	78.7	72.7	88.6	78.9	93.6	67.7	84.3	60.9	75.7	68.8	85.9	63.5	70.0

Mali	15 to 19	64.3	69.8	55.3	60.7	78.1	82.3	60.1	65.5	37.8	39.6	84.2	88.7	83.6	84.2	76.4	75.2	91.0	93.1	75.4	73.5	78.3	77.2	80.0	77.5
Malta	15 to 19	74.5	88.9	82.6	94.8	73.3	87.3	76.4	89.0	78.8	90.2	79.0	89.5	77.4	89.2	82.4	93.6	74.3	86.0	63.7	91.6	75.8	94.2	58.7	90.6
Marshall Islands	15 to 19	66.4	50.2	81.0	71.3	58.9	38.4	56.0	40.8	68.2	59.2	55.3	37.6	76.2	63.5	79.9	69.4	72.7	58.9	83.2	72.0	83.9	74.0	87.5	80.5
Mauritania	15 to 19	68.5	80.5	67.9	81.2	75.0	84.2	64.9	78.0	50.1	66.4	82.5	91.6	83.3	90.0	81.8	87.5	85.6	92.9	69.7	78.1	80.8	85.2	64.5	75.8
Mauritius	15 to 19	73.0	70.2	82.7	82.4	71.4	65.0	73.6	73.3	76.0	79.0	76.3	72.5	81.2	74.3	86.8	82.5	76.4	66.5	60.0	58.2	71.6	70.5	56.6	54.2
Mexico	15 to 19	78.1	81.0	84.7	88.9	77.8	78.0	83.9	89.6	83.5	89.5	86.7	90.6	75.3	73.3	80.4	82.5	71.6	63.7	69.7	83.1	78.0	89.4	66.9	78.6
Micronesia (Federated States of)	15 to 19	52.5	53.1	72.6	73.6	40.7	42.9	42.9	46.5	59.6	62.8	40.8	44.3	64.3	64.4	71.4	71.8	57.6	58.8	75.6	71.9	76.4	74.1	83.4	79.5
Monaco	15 to 19	71.6	86.5	77.2	89.6	73.1	88.3	73.2	87.4	76.6	87.5	75.3	89.4	60.1	83.3	58.7	81.0	66.3	88.9	64.2	84.6	71.6	86.3	65.7	87.5
Mongolia	15 to 19	79.3	81.0	89.2	90.5	74.6	78.1	74.8	76.9	77.8	79.6	75.5	78.2	75.9	79.0	84.7	84.0	68.4	75.6	89.4	86.6	92.7	91.1	87.6	85.4
Montenegro	15 to 19	73.2	78.2	82.6	88.1	70.5	73.6	72.0	76.1	76.0	81.0	73.0	73.4	87.8	90.5	90.9	93.3	85.8	88.8	85.3	86.2	90.0	90.9	84.2	84.5
Morocco	15 to 19	63.8	76.1	65.6	81.7	72.5	78.7	55.4	71.0	50.2	71.0	69.6	76.6	86.2	91.7	85.5	91.7	87.6	92.4	45.5	56.1	46.2	61.0	66.7	65.7
Mozambique	15 to 19	62.2	56.5	79.7	81.0	52.3	42.6	51.8	47.2	63.8	65.6	51.8	45.6	74.0	63.5	88.0	86.3	62.1	45.1	79.7	71.2	87.2	85.9	75.4	59.6
Myanmar	15 to 19	24.2	45.4	41.4	69.9	19.7	30.5	27.3	45.8	33.9	62.0	35.8	42.6	32.1	56.8	49.6	74.9	16.0	38.7	37.8	50.1	55.4	71.3	32.3	33.3
Namibia	15 to 19	63.7	70.8	81.4	86.9	49.8	58.6	42.6	54.1	57.1	68.7	41.9	51.9	90.3	91.8	94.0	95.4	87.0	88.7	80.9	81.2	88.8	89.5	73.4	73.0
Nauru	15 to 19	48.1	42.2	69.2	65.4	36.8	29.5	35.1	28.6	55.5	50.9	32.5	24.8	60.8	53.5	66.0	58.2	55.7	50.0	63.6	55.9	66.0	59.8	71.9	67.6
Nepal	15 to 19	68.9	74.8	73.7	86.3	73.9	69.9	66.7	72.8	64.5	77.7	76.5	72.6	73.9	78.5	77.0	87.2	73.1	70.3	76.2	81.0	77.9	87.4	84.7	78.4
Netherlands	15 to 19	84.7	93.6	90.0	97.3	84.5	93.0	85.7	94.5	87.2	94.1	86.6	95.2	73.2	91.6	76.6	93.8	72.5	90.8	85.9	95.5	89.7	96.5	85.1	95.3
New Zealand	15 to 19	80.6	92.1	88.2	96.0	78.7	92.0	85.4	94.8	88.1	94.3	85.4	95.6	61.6	89.5	72.2	90.6	51.2	89.7	79.6	92.5	87.5	94.6	75.6	92.4
Nicaragua	15 to 19	70.5	81.2	75.4	87.4	74.4	81.0	75.4	86.2	72.7	85.6	83.4	88.7	72.4	81.1	75.5	85.2	72.3	78.7	66.5	80.7	73.8	86.0	67.7	79.3
Niger	15 to 19	71.8	76.9	70.6	75.0	77.8	83.6	69.6	73.7	57.0	60.9	84.8	88.9	87.9	90.4	86.4	86.5	90.2	94.6	81.6	82.4	87.4	86.5	80.1	83.5
Nigeria	15 to 19	82.7	87.0	84.2	90.7	84.4	86.0	79.3	85.7	71.8	79.8	88.7	92.8	94.5	95.1	93.2	94.8	96.2	95.4	85.8	84.6	90.8	92.4	83.8	78.3
Niue	15 to 19	67.8	54.8	83.9	60.3	60.2	57.9	57.8	45.5	71.5	47.4	57.3	54.2	75.2	60.5	82.2	52.4	68.7	66.8	84.3	66.0	83.7	58.2	88.1	85.6
North Macedonia	15 to 19	69.8	78.0	81.8	87.2	64.7	75.6	72.7	80.2	75.7	81.7	73.9	82.1	62.4	73.1	72.1	79.6	53.5	68.1	67.3	76.5	79.8	85.2	57.4	71.1
Northern Mariana Islands	15 to 19	78.3	82.5	88.6	92.8	70.4	76.4	68.2	73.7	78.9	83.6	64.9	72.4	88.1	89.8	88.1	92.4	87.4	86.6	85.8	89.9	86.6	90.6	88.6	90.7
Norway	15 to 19	82.7	94.5	89.2	97.3	81.9	94.6	85.2	93.0	86.9	92.7	86.1	94.2	66.6	93.4	73.3	93.9	62.1	94.5	87.8	97.3	89.8	97.5	89.6	97.2
Oman	15 to 19	65.3	81.5	83.5	90.7	57.7	80.8	57.6	75.5	73.5	80.2	50.0	75.5	92.8	100.0	95.5	98.8	89.2	99.9	53.0	73.4	68.6	80.0	47.8	74.4
Pakistan	15 to 19	74.2	65.5	75.9	67.1	81.4	75.0	71.3	63.1	66.1	56.6	82.5	77.5	75.3	64.0	73.5	59.8	79.7	71.5	79.4	69.0	79.1	70.5	88.5	81.0
Palau	15 to 19	62.9	52.7	76.4	45.6	57.6	67.6	53.3	48.6	63.7	35.9	56.3	65.7	76.7	62.1	74.8	37.5	77.3	81.0	71.3	37.3	70.2	22.1	81.7	82.7
Palestine	15 to 19	57.3	76.2	77.2	87.4	43.8	71.2	43.8	66.7	64.6	76.0	33.1	63.1	94.4	98.2	96.2	98.3	92.2	98.2	38.0	64.1	59.9	75.2	24.9	59.5
Panama	15 to 19	76.5	78.8	80.0	85.0	80.1	79.1	81.8	85.7	82.1	85.9	84.4	87.3	75.0	74.0	79.0	79.5	72.2	69.8	68.6	79.0	70.6	82.5	75.5	81.2
Papua New Guinea	15 to 19	77.1	73.0	80.8	74.1	77.3	75.8	63.3	57.0	64.8	55.7	68.9	66.0	87.7	86.0	85.0	79.8	90.7	92.8	87.0	82.4	84.1	78.4	94.9	93.7
Paraguay	15 to 19	64.8	71.3	70.6	78.0	68.5	72.1	72.0	76.7	69.7	77.9	79.8	79.7	61.5	68.9	65.8	71.6	60.6	69.4	73.4	78.9	78.7	84.3	75.1	78.2
Peru	15 to 19	33.4	50.5	55.3	62.5	23.6	49.9	45.8	55.8	57.4	61.4	44.7	59.1	35.6	48.6	52.2	53.5	21.8	47.6	57.2	70.2	70.8	76.5	51.3	71.9
Philippines	15 to 19	68.6	65.2	80.5	79.7	62.4	58.5	65.7	63.2	73.2	71.4	63.1	62.3	76.8	72.1	82.9	80.1	71.6	65.6	69.7	67.2	79.3	78.1	65.0	62.1
Poland	15 to 19	74.7	87.1	85.7	94.4	69.9	84.4	80.4	90.4	84.0	91.3	79.8	89.4	72.1	89.2	81.5	93.3	63.1	85.7	71.9	86.4	84.1	91.2	62.0	84.2
Portugal	15 to 19	50.1	88.1	68.1	91.4	42.6	87.6	55.8	92.8	67.1	93.2	55.9	92.7	44.3	85.1	59.7	87.2	31.6	84.6	21.1	88.5	54.6	90.8	0.0	88.5
Puerto Rico	15 to 19	88.3	96.1	92.8	99.2	87.0	94.9	90.8	97.0	89.9	94.9	92.7	98.8	87.1	96.5	90.3	98.4	84.3	94.5	88.6	98.9	92.8	99.7	84.0	96.2
Qatar	15 to 19	69.8	83.6	87.3	94.4	61.9	78.3	65.8	79.8	75.7	85.5	63.6	77.0	80.9	93.4	94.5	98.0	62.3	85.7	76.1	83.1	84.3	90.3	72.6	78.3
Republic of Korea	15 to 19	69.1	90.1	81.8	94.7	62.7	88.9	77.7	91.9	82.4	92.0	76.6	92.4	43.7	87.3	61.9	89.9	25.1	85.2	64.1	88.9	77.7	91.6	55.9	88.7
Republic of Moldova	15 to 19	71.5	82.9	81.1	91.0	70.3	81.8	62.4	79.7	66.3	81.8	65.7	80.6	85.9	89.5	91.7	93.7	80.6	86.1	71.3	84.0	78.2	88.3	72.8	84.7
Romania	15 to 19	76.5	82.6	85.3	91.7	74.0	78.3	77.9	87.6	79.9	89.1	79.5	87.0	76.8	79.4	82.2	87.3	72.5	71.7	72.6	80.1	82.7	88.1	66.1	74.3
Russian Federation	15 to 19	73.4	80.6	85.4	90.8	67.5	75.3	70.2	76.8	76.1	82.9	68.8	71.9	74.9	82.8	85.7	88.5	64.0	77.6	59.1	78.5	72.5	86.2	52.1	73.5
Rwanda	15 to 19	2.7	63.4	15.8	73.5	6.5	61.7	1.8	53.0	0.0	55.3	20.7	60.3	36.2	76.7	50.6	83.7	25.5	71.2	50.4	79.7	53.1	83.0	66.0	83.0
Saint Kitts and Nevis	15 to 19	69.0	77.8	76.0	86.7	68.2	73.1	71.2	78.8	71.9	82.7	75.9	79.6	67.1	77.2	74.3	86.0	60.9	67.5	64.1	75.9	71.1	82.7	66.0	72.5
Saint Lucia	15 to 19	76.0	85.9	82.6	93.7	73.8	79.8	78.3	87.9	80.1	91.5	79.5	84.5	76.0	85.5	81.0	91.1	71.6	79.6	66.1	85.3	72.2	91.6	69.1	77.8
Saint Vincent and the Grenadines	15 to 19	63.4	75.9	71.9	82.2	60.3	74.3	61.5	77.4	65.7	79.7	63.1	78.4	70.4	77.7	74.9	80.6	67.8	76.8	59.0	71.6	68.2	77.1	58.4	73.0
Samoa	15 to 19	68.5	69.6	84.3	83.0	59.5	63.4	58.6	61.5	72.7	71.5	56.1	60.1	77.0	77.7	82.6	81.2	72.1	75.0	85.9	82.6	85.4	83.3	90.4	88.1
San Marino	15 to 19	86.1	92.3	91.9	96.0	84.9	92.4	85.5	92.5	87.0	91.7	86.5	94.1	86.8	93.7	90.8	94.9	84.3	94.2	84.0	92.9	90.0	94.6	80.5	92.8

Sao Tome and Principe	15 to 19	83.6	73.7	82.4	82.3	87.1	72.5	83.4	72.2	70.4	69.6	95.4	79.5	92.8	82.0	90.1	86.2	95.9	78.8	82.5	67.4	88.3	83.1	81.1	58.5
Saudi Arabia	15 to 19	60.1	79.8	65.3	86.8	64.8	80.1	49.0	76.0	48.3	77.8	59.5	78.9	92.1	96.9	92.3	97.0	92.2	97.0	39.8	64.2	45.0	72.2	53.2	65.0
Senegal	15 to 19	65.8	75.1	65.5	77.0	71.5	77.8	61.1	72.8	47.5	60.8	78.6	85.2	80.1	83.1	79.1	82.5	82.1	84.5	66.9	73.4	78.3	83.6	61.4	69.0
Serbia	15 to 19	70.5	88.8	81.2	94.0	65.5	88.2	75.6	92.4	79.3	91.1	76.1	94.6	61.0	89.7	68.7	90.8	55.4	90.1	63.7	85.2	79.0	90.0	50.1	83.4
Seychelles	15 to 19	72.0	90.3	77.9	96.6	75.2	90.2	72.5	88.3	71.4	88.9	79.2	90.3	80.6	98.1	82.0	98.6	81.7	98.5	64.0	89.6	70.9	92.4	67.6	90.5
Sierra Leone	15 to 19	73.3	71.0	75.6	68.1	74.4	79.3	73.6	70.3	63.8	53.3	85.0	90.1	85.6	82.3	85.3	77.3	86.8	88.1	69.7	65.5	83.6	74.7	59.6	63.6
Singapore	15 to 19	86.8	91.5	90.8	93.3	87.5	93.5	89.0	93.3	88.4	91.1	91.1	96.2	87.9	93.2	89.5	91.1	87.8	97.2	79.7	90.0	81.3	90.5	84.0	92.7
Slovakia	15 to 19	77.3	85.8	87.5	92.4	73.7	85.5	78.7	88.8	82.1	88.4	78.7	90.7	78.6	87.6	84.4	89.8	73.8	87.1	72.9	83.4	84.0	88.6	64.9	82.3
Slovenia	15 to 19	85.7	91.1	93.5	96.5	82.7	91.2	89.2	94.7	89.8	92.3	89.8	97.8	87.0	95.5	91.7	97.3	82.9	94.8	82.0	89.1	89.5	92.3	76.1	89.2
Solomon Islands	15 to 19	85.8	82.1	92.5	90.7	82.2	78.7	75.1	72.4	79.0	77.1	76.7	73.7	95.3	92.5	94.8	93.0	94.4	91.6	93.5	90.4	92.4	90.2	96.0	94.1
Somalia	15 to 19	33.8	48.7	55.5	64.5	27.6	44.6	23.5	34.5	31.4	38.6	32.2	43.6	56.3	63.7	76.0	77.6	43.4	54.1	76.3	78.0	79.5	82.1	80.2	80.6
South Sudan	15 to 19	76.7	81.7	59.8	59.5	45.1	49.3	60.5	66.6	38.9	32.6	47.7	47.4	92.9	94.2	79.0	75.6	62.1	59.9	84.6	86.6	77.5	75.0	77.7	75.4
Spain	15 to 19	46.8	49.2	81.8	95.0	67.9	87.3	37.5	34.5	82.1	94.5	77.1	90.1	69.0	66.3	76.0	91.0	57.1	84.9	73.7	70.8	76.5	94.2	60.8	91.8
Sri Lanka	15 to 19	71.2	89.4	74.7	86.0	63.0	74.5	76.4	91.9	64.8	78.1	65.9	76.0	65.7	87.2	88.7	93.6	85.4	91.5	64.9	92.2	61.4	74.7	44.3	58.9
Sudan	15 to 19	64.6	76.9	50.5	72.1	63.2	72.7	61.5	74.6	33.6	58.0	57.4	68.9	86.4	92.1	81.2	86.7	84.1	87.7	47.4	63.5	36.7	49.7	69.6	59.1
Suriname	15 to 19	51.0	67.9	65.0	79.9	55.2	62.5	39.2	59.6	61.8	74.9	64.5	69.8	82.2	86.8	67.0	78.8	54.8	60.7	40.7	45.6	69.0	81.1	54.9	62.0
Sweden	15 to 19	56.5	69.0	93.1	99.0	83.5	93.6	59.4	70.1	96.4	100.0	89.6	97.2	59.8	69.5	84.7	96.5	70.0	92.6	58.4	70.7	89.9	97.0	83.9	95.2
Switzerland	15 to 19	86.4	94.9	83.5	96.1	72.9	93.5	92.3	98.6	83.1	94.3	79.6	96.3	76.7	94.0	70.3	92.3	58.5	93.2	85.6	95.6	81.2	95.7	63.3	95.1
Syrian Arab Republic	15 to 19	74.8	93.1	66.7	80.5	37.9	55.1	79.3	95.2	53.6	65.5	36.5	48.0	63.2	92.0	92.1	96.3	85.4	91.8	69.5	94.8	47.4	65.5	29.5	42.2
Taiwan (Province of China)	15 to 19	45.5	62.5	88.5	96.3	77.4	88.8	37.2	50.5	82.7	93.6	80.5	91.1	89.1	94.4	93.4	97.1	91.4	96.3	30.3	48.5	85.1	91.0	72.6	84.2
Tajikistan	15 to 19	80.3	91.0	74.9	83.5	62.9	74.9	79.9	92.1	63.1	73.0	62.8	75.4	92.1	96.5	82.4	87.6	76.0	82.2	77.1	86.5	61.7	73.8	36.4	63.4
Thailand	15 to 19	64.0	75.0	79.3	86.6	56.5	52.0	59.3	71.5	73.6	79.1	69.4	62.2	78.3	84.1	78.9	86.9	49.2	51.5	44.3	64.2	81.8	85.4	66.8	60.6
Timor-Leste	15 to 19	63.7	65.7	64.9	65.8	58.9	47.4	67.8	66.1	59.2	58.5	66.6	56.3	64.7	70.5	72.5	72.0	61.5	52.9	72.1	72.3	71.2	70.0	65.6	50.5
Togo	15 to 19	57.1	51.5	65.7	72.6	76.4	76.1	58.8	52.3	47.7	55.8	83.4	83.2	65.9	61.7	79.8	81.2	88.1	86.6	63.6	56.0	79.5	80.7	68.8	65.6
Tokelau	15 to 19	68.9	71.7	78.7	51.2	51.0	32.8	65.2	69.2	67.5	42.7	47.6	26.4	83.6	83.6	78.9	44.0	69.0	53.3	70.9	69.7	79.5	47.9	84.6	75.7
Tonga	15 to 19	62.6	37.0	93.6	91.8	84.2	77.7	52.5	27.7	82.8	81.0	78.9	73.4	74.2	47.8	94.0	92.0	91.7	85.6	78.7	50.2	92.0	89.4	93.2	90.1
Trinidad and Tobago	15 to 19	87.5	82.7	86.0	90.4	63.4	75.7	78.7	74.1	80.3	87.6	73.6	87.9	93.0	88.8	81.2	83.7	53.5	55.0	91.9	88.3	85.5	90.3	43.1	73.3
Tropical Latin America	15 to 19	73.7	81.9	71.9	78.7	58.0	63.7	76.1	87.2	69.0	76.5	66.0	70.1	68.2	70.6	68.8	71.7	57.9	59.7	67.6	82.5	75.6	83.9	53.5	72.1
Tunisia	15 to 19	74.9	83.2	83.3	90.5	74.1	82.7	68.9	79.8	72.0	81.5	72.0	81.9	92.8	96.4	94.2	96.8	91.1	95.9	66.8	73.4	73.0	80.5	69.7	73.0
Turkey	15 to 19	65.7	81.3	79.7	90.0	58.8	78.6	63.9	78.1	70.8	81.5	63.9	78.7	68.9	88.6	82.7	93.1	51.6	83.1	56.5	74.2	71.2	82.2	48.2	71.1
Turkmenistan	15 to 19	67.9	56.1	76.8	73.9	69.0	47.5	65.9	54.1	67.6	63.5	71.2	52.3	71.8	48.2	78.6	69.7	66.4	27.0	42.5	29.6	55.6	52.9	44.1	17.2
Tuvalu	15 to 19	47.8	53.5	74.2	76.1	28.5	41.6	37.6	46.3	61.3	65.4	30.1	41.5	62.3	63.3	73.9	72.6	50.3	56.1	76.1	72.2	78.7	75.4	80.1	77.6
Uganda	15 to 19	55.7	64.8	73.3	77.2	47.4	61.0	42.5	54.2	51.9	58.1	46.9	60.5	68.9	74.6	83.3	84.4	57.2	67.1	79.5	79.3	84.0	83.8	80.4	80.5
Ukraine	15 to 19	79.1	84.6	88.7	92.9	75.5	82.2	73.9	81.8	78.3	84.1	72.8	82.4	84.4	88.4	88.5	91.8	81.4	85.9	78.7	85.7	87.9	91.4	71.4	83.0
United Arab Emirates	15 to 19	65.3	86.0	82.3	95.1	56.5	82.0	57.7	82.5	72.9	88.0	50.7	79.2	79.5	96.1	86.0	98.0	71.7	92.6	52.5	83.2	69.6	90.4	43.2	77.6
United Kingdom	15 to 19	81.6	92.8	88.0	96.8	79.0	91.4	84.5	92.5	86.9	93.1	84.9	92.6	59.0	89.3	68.9	92.1	51.2	87.8	81.8	96.1	88.5	97.3	76.9	94.9
United Republic of Tanzania	15 to 19	55.2	69.7	70.3	79.8	49.5	67.4	45.8	59.9	52.1	62.7	51.5	65.7	70.2	76.0	82.9	84.2	59.8	69.6	79.7	77.7	83.9	84.2	81.1	76.4
United States Virgin Islands	15 to 19	75.2	89.5	87.0	100.0	65.4	77.1	79.7	91.3	85.4	95.3	75.8	84.5	72.7	90.1	83.7	100.0	59.0	75.0	79.9	91.8	88.8	100.0	69.2	75.3
United States of America	15 to 19	97.7	100.0	96.8	99.4	97.5	98.9	97.0	100.0	96.8	99.7	96.4	98.8	84.9	91.5	87.1	93.2	83.6	89.7	95.8	99.4	95.8	98.9	94.2	97.8
Uruguay	15 to 19	57.2	77.0	64.8	86.5	60.1	73.8	73.2	85.1	73.0	86.9	78.8	85.4	23.7	63.8	33.7	75.4	18.7	52.8	62.3	81.5	70.0	88.2	64.9	78.5
Uzbekistan	15 to 19	62.2	77.6	74.0	90.1	59.9	71.3	52.8	74.6	58.5	80.8	55.1	71.8	81.9	85.6	87.7	93.3	77.2	77.7	45.1	66.9	61.9	81.5	38.4	54.2
Vanuatu	15 to 19	63.7	60.5	80.6	77.3	52.0	52.1	50.9	50.1	67.4	64.0	45.7	48.2	69.6	67.5	76.5	71.7	62.8	64.1	80.5	76.4	81.9	78.3	84.8	82.8
Venezuela (Bolivarian Republic of)	15 to 19	65.1	71.0	74.9	81.6	62.8	66.9	74.0	79.6	75.5	80.5	76.7	81.9	59.7	66.0	69.6	76.4	50.4	54.0	62.3	70.5	73.6	81.4	57.0	61.6
Viet Nam	15 to 19	74.4	73.6	86.5	90.4	69.4	64.0	74.0	73.0	78.7	84.1	74.3	68.5	82.6	84.0	90.1	93.2	75.1	74.2	77.6	72.4	85.1	85.9	74.2	61.7
Yemen	15 to 19	78.3	76.5	85.4	83.7	77.7	76.7	71.4	70.7	74.1	72.6	74.2	74.5	95.9	93.5	96.8	94.4	94.1	92.3	77.5	64.2	79.4	70.8	83.2	67.7
Zambia	15 to 19	44.8	57.0	52.0	65.4	48.8	57.8	36.8	47.2	30.4	43.0	53.1	59.5	67.1	72.2	73.3	77.8	62.6	67.9	75.2	72.7	77.4	77.3	82.1	76.4
Zimbabwe	15 to 19	75.6	41.4	89.2	67.5	64.3	21.4	60.0	4.7	71.1	25.8	58.4	5.6	83.6	59.3	93.1	77.1	75.4	45.9	86.8	65.7	91.6	77.7	82.6	57.1
Afghanistan	20 to 24	46.3	49.9	49.5	52.8	73.1	73.4	42.3	42.5	31.6	28.8	66.1	66.8	76.5	73.7	76.0	73.2	86.1	84.3	47.0	36.3	48.0	40.4	72.2	48.0

Albania	20 to 24	73.4	83.9	82.9	89.3	80.0	89.4	75.0	84.9	75.2	82.6	80.2	89.7	77.9	89.2	84.8	91.9	81.6	91.8	86.0	88.3	90.4	91.5	79.5	84.7
Algeria	20 to 24	71.0	79.7	75.2	84.4	83.6	87.5	67.3	75.6	60.4	72.2	80.1	83.7	86.7	92.3	87.0	92.4	91.6	95.1	67.2	72.9	70.8	78.9	69.4	68.1
American Samoa	20 to 24	61.9	60.2	82.4	76.7	66.1	68.3	58.0	57.1	64.1	58.9	65.8	67.5	68.5	64.8	87.3	79.9	70.5	70.7	86.3	84.9	95.4	92.5	66.4	69.9
Andorra	20 to 24	83.8	91.0	85.3	92.5	91.8	95.5	88.0	94.2	82.9	90.3	93.6	97.1	73.6	86.7	69.3	85.3	86.9	93.2	87.5	94.2	88.6	94.7	87.7	93.4
Angola	20 to 24	68.4	72.5	74.8	77.9	80.6	83.3	63.8	66.4	54.5	56.7	78.7	81.0	78.0	78.7	82.1	82.2	84.8	85.4	85.4	83.0	88.1	86.9	83.2	79.4
Antigua and Barbuda	20 to 24	68.0	83.9	79.1	91.5	74.1	85.5	72.9	87.0	70.0	86.6	80.4	88.4	63.0	81.0	81.2	91.1	62.6	80.6	77.3	90.2	81.5	92.6	75.9	86.7
Argentina	20 to 24	59.0	79.7	67.9	85.6	73.8	86.5	74.1	85.0	70.1	82.2	82.9	89.8	38.3	74.6	46.0	78.8	57.5	81.9	77.7	90.5	82.2	92.7	74.4	87.8
Armenia	20 to 24	75.5	84.8	81.9	90.4	84.4	89.6	73.6	87.2	70.7	83.0	81.2	91.7	84.0	88.9	88.1	94.0	87.3	90.0	69.5	81.1	78.1	88.6	60.9	70.0
Australia	20 to 24	80.5	89.8	85.5	92.9	88.0	94.2	85.5	95.7	82.6	92.2	90.4	97.9	69.5	89.3	75.8	92.1	76.7	91.6	84.5	92.3	89.5	94.0	78.1	90.7
Austria	20 to 24	81.8	93.5	87.2	96.1	88.2	96.0	83.6	95.4	82.4	92.3	88.1	97.3	76.2	91.4	80.4	94.1	83.2	93.4	82.3	96.3	87.4	97.4	76.1	93.8
Azerbaijan	20 to 24	67.3	80.3	75.3	85.7	78.4	87.5	62.1	77.0	58.4	73.1	73.7	84.7	89.8	93.8	91.7	94.9	92.6	95.7	73.3	83.1	81.8	88.0	61.8	77.0
Bahamas	20 to 24	65.6	82.2	79.5	89.9	70.0	84.3	67.6	84.2	70.4	84.3	71.5	86.4	64.7	78.7	80.7	88.6	64.7	78.6	74.7	87.6	82.2	90.9	64.6	83.1
Bahrain	20 to 24	76.1	81.9	79.7	88.5	86.7	87.8	73.4	79.5	68.7	79.9	82.6	84.7	88.2	94.1	87.3	95.1	93.2	96.0	76.5	79.3	78.5	84.6	78.6	73.3
Bangladesh	20 to 24	26.6	48.7	64.8	77.0	34.5	51.4	38.1	54.3	53.7	67.1	43.6	55.8	16.2	42.5	65.7	74.6	22.0	46.0	61.6	68.6	78.7	84.8	35.3	38.8
Barbados	20 to 24	73.7	87.0	82.8	89.7	79.3	91.8	78.8	91.6	77.1	86.8	83.8	95.4	69.3	82.9	80.7	85.7	72.7	87.4	74.0	87.9	80.4	89.0	68.0	89.4
Belarus	20 to 24	77.1	84.9	84.7	90.2	83.1	89.5	76.0	86.4	75.6	84.4	80.9	89.5	80.1	85.9	81.6	89.4	87.0	89.2	71.5	85.4	81.9	89.7	54.5	79.8
Belgium	20 to 24	68.8	92.9	80.4	94.8	76.6	96.0	69.4	96.0	72.2	92.4	75.4	97.9	57.4	89.5	67.3	90.4	68.5	93.3	70.9	95.5	82.3	96.3	55.1	93.7
Belize	20 to 24	56.0	82.5	75.8	93.3	60.4	81.5	61.3	81.8	64.5	86.4	68.0	80.6	50.5	85.4	80.1	94.8	45.1	82.7	75.2	89.6	83.4	95.8	62.8	74.9
Benin	20 to 24	81.6	80.5	85.0	83.0	88.9	89.1	78.4	77.6	67.6	64.5	90.3	90.8	90.1	87.5	91.0	87.3	93.4	92.6	87.3	83.7	94.3	91.5	77.7	73.5
Bermuda	20 to 24	81.4	94.4	88.1	96.4	85.1	96.1	86.1	96.9	83.1	91.0	89.6	100.0	81.5	95.1	89.6	98.0	82.0	94.5	76.0	95.6	83.7	97.3	64.6	90.9
Bhutan	20 to 24	72.2	78.4	77.1	86.3	83.7	84.4	71.5	77.9	64.9	76.5	82.9	83.7	73.3	78.5	76.6	85.5	82.2	83.1	82.6	84.7	84.7	89.7	82.5	78.3
Bolivia (Plurinational State of)	20 to 24	18.9	52.4	21.8	57.9	58.7	72.9	36.2	62.4	19.5	54.3	66.5	77.5	18.3	45.2	13.3	42.3	55.2	69.2	42.2	71.4	50.5	76.9	46.4	68.5
Bosnia and Herzegovina	20 to 24	76.9	82.6	86.2	88.1	83.3	89.0	85.7	90.4	84.1	86.8	89.0	93.4	64.3	80.8	79.3	86.2	67.9	84.6	77.1	79.8	85.8	85.6	67.0	74.5
Botswana	20 to 24	67.6	82.3	83.1	89.0	71.8	87.0	55.3	74.3	57.9	71.5	67.1	83.0	88.7	94.6	93.7	96.2	90.3	95.8	84.7	88.8	90.7	92.5	73.3	83.0
Brazil	20 to 24	54.7	67.3	66.5	76.2	68.4	77.1	61.5	70.8	60.7	70.5	70.6	77.2	52.4	63.8	59.0	68.0	65.7	74.9	64.5	77.5	76.4	84.6	48.5	68.2
Brunei Darussalam	20 to 24	60.0	86.6	75.2	89.7	70.0	91.8	71.1	90.4	73.3	86.3	77.3	94.3	38.8	80.0	55.9	82.0	52.1	86.5	58.1	87.1	73.5	88.8	38.7	86.6
Bulgaria	20 to 24	61.4	67.3	73.2	82.1	72.2	72.1	65.2	70.6	64.2	75.8	74.6	73.0	67.2	64.2	73.8	75.9	75.7	70.1	60.8	58.6	75.5	81.2	39.5	17.7
Burkina Faso	20 to 24	84.0	80.9	88.8	86.2	89.2	87.2	81.7	78.0	74.2	69.9	90.4	88.3	93.7	90.6	96.4	93.7	94.3	92.0	90.0	86.4	95.6	93.8	82.1	75.3
Burundi	20 to 24	32.3	57.6	48.4	66.2	55.1	73.4	34.0	51.4	26.7	41.0	56.4	70.5	48.5	69.2	63.0	76.0	62.4	78.4	67.8	77.8	72.7	81.5	68.3	77.2
Cabo Verde	20 to 24	74.7	82.0	78.0	91.2	85.6	86.4	75.1	82.9	62.0	78.8	89.1	89.5	82.2	88.3	85.0	95.0	87.7	89.4	76.3	76.9	88.4	93.6	60.4	54.9
Cambodia	20 to 24	57.7	71.1	60.7	78.9	77.9	80.7	58.5	71.6	46.8	68.0	78.1	81.2	69.2	80.2	69.0	83.9	81.6	85.6	70.7	78.5	74.4	83.4	74.7	74.7
Cameroon	20 to 24	69.7	70.5	77.2	76.9	79.9	81.6	66.0	68.7	58.5	58.2	79.4	82.8	81.6	77.9	88.4	84.5	84.6	83.0	83.5	76.6	92.7	88.9	70.5	61.3
Canada	20 to 24	89.7	95.2	91.6	96.0	94.5	97.7	94.4	96.9	89.6	92.2	97.5	99.2	79.8	87.6	84.2	89.7	84.1	90.8	91.0	97.3	92.6	98.0	88.7	95.2
Central African Republic	20 to 24	57.7	62.1	70.5	71.4	70.5	75.2	53.4	57.1	46.9	47.0	69.9	74.2	69.3	70.6	80.2	79.1	76.2	78.3	81.2	81.5	86.7	86.8	74.0	75.1
Chad	20 to 24	75.4	70.7	82.4	77.3	83.4	81.5	72.0	68.7	63.6	57.9	83.9	82.9	83.9	77.4	90.8	85.1	86.5	82.1	83.6	76.8	93.5	90.0	70.1	59.4
Chile	20 to 24	72.6	84.5	81.5	88.5	80.5	90.2	82.0	90.6	80.6	87.0	86.1	93.8	58.9	75.8	69.6	78.0	67.0	84.1	78.5	89.0	85.4	91.6	69.8	85.9
China	20 to 24	70.9	75.9	83.3	88.9	76.1	79.5	74.7	73.5	77.1	80.7	79.0	76.9	60.8	83.8	71.4	90.0	69.4	86.5	78.9	77.1	87.7	88.2	63.5	60.3
Colombia	20 to 24	67.1	79.4	75.6	85.4	76.9	85.7	76.6	89.9	72.7	85.1	83.8	94.1	61.4	71.0	68.9	78.1	70.1	77.1	62.8	85.5	78.6	90.1	35.7	78.2
Comoros	20 to 24	50.3	65.8	54.9	70.6	72.4	80.3	46.5	58.2	29.6	45.9	70.9	76.7	66.6	74.7	67.5	76.8	79.3	83.7	74.0	80.4	77.0	83.7	76.3	79.1
Congo	20 to 24	65.3	71.5	76.3	74.7	76.1	84.2	61.1	63.6	55.5	50.0	75.1	81.3	75.8	78.4	83.3	79.2	82.0	86.5	82.3	81.4	88.4	85.4	73.9	78.3
Cook Islands	20 to 24	62.9	75.8	75.8	87.3	72.1	79.2	61.5	70.6	58.5	72.3	73.4	76.2	66.1	83.0	78.8	92.4	71.9	83.4	86.5	88.1	93.3	93.6	72.0	77.2
Costa Rica	20 to 24	83.4	87.1	87.6	91.3	89.2	91.0	89.9	95.2	84.2	89.7	94.9	98.3	79.8	82.0	84.3	88.0	84.1	84.0	84.3	90.6	89.6	93.7	75.4	85.0
Coted'Ivoire	20 to 24	72.2	73.3	83.0	80.6	80.5	82.8	69.5	72.5	63.4	61.8	81.0	85.1	79.1	78.1	88.9	84.5	82.3	83.6	76.5	72.3	93.2	89.5	57.4	51.8
Croatia	20 to 24	76.7	88.9	86.4	92.0	81.3	93.3	83.3	96.1	85.4	91.2	84.0	98.5	72.4	90.3	79.6	91.0	77.6	93.8	75.9	88.0	86.1	91.3	60.4	83.9
Cuba	20 to 24	75.8	87.5	83.0	93.7	82.3	88.7	78.2	89.8	75.9	89.7	84.0	89.6	78.1	88.7	84.6	93.7	81.2	89.1	74.3	89.2	81.5	93.8	65.6	79.8
Cyprus	20 to 24	81.9	90.0	88.9	95.8	86.9	91.7	90.5	93.5	89.4	92.6	92.7	94.5	71.6	85.9	80.3	92.9	78.0	87.7	87.4	94.7	90.9	97.5	83.1	89.4
Czechia	20 to 24	78.8	87.4	86.5	90.8	84.4	92.4	85.5	93.2	83.9	87.7	88.4	97.1	75.3	87.1	81.6	88.4	80.3	91.5	73.8	86.5	84.5	90.4	57.9	81.5
Democratic People's Republic of Korea	20 to 24	67.6	61.4	80.8	79.9	72.8	68.2	69.0	60.9	71.9	69.0	73.6	66.7	68.4	70.5	77.2	81.1	73.2	75.5	74.8	69.6	84.8	83.9	58.1	48.0

Democratic Republic of the Congo	20 to 24	68.2	71.4	72.7	77.1	81.6	82.7	63.7	65.8	51.1	54.9	80.1	80.9	78.6	80.9	81.8	84.7	85.7	86.9	83.5	85.3	86.6	88.7	81.3	82.0
Denmark	20 to 24	83.0	92.0	87.9	94.7	88.6	95.1	86.4	96.1	86.3	92.8	89.6	97.9	70.0	89.0	72.2	91.0	80.8	92.2	85.8	95.5	90.8	96.4	79.0	93.7
Djibouti	20 to 24	67.4	73.5	82.5	86.7	75.7	80.3	63.2	67.9	64.4	70.2	74.1	77.4	75.6	76.7	89.7	90.7	80.1	81.2	87.2	85.9	91.8	92.3	81.3	77.5
Dominica	20 to 24	75.4	79.8	84.3	85.9	80.2	85.0	77.4	82.6	75.9	79.4	82.1	87.4	75.0	79.2	86.5	86.7	75.7	81.6	81.5	82.0	85.8	85.7	77.3	79.2
Dominican Republic	20 to 24	56.3	59.3	69.3	77.2	66.2	63.2	58.4	59.2	59.0	67.2	65.9	60.1	57.2	62.9	68.4	76.1	63.6	66.3	68.1	63.9	78.3	80.6	53.8	32.3
Ecuador	20 to 24	43.7	68.2	65.2	81.3	55.0	75.4	48.2	76.0	53.9	77.4	56.9	80.8	53.7	63.1	70.5	75.7	60.1	69.6	47.0	78.6	68.6	88.4	13.3	63.2
Egypt	20 to 24	47.0	67.8	64.7	80.4	56.8	74.9	32.5	59.8	43.2	68.6	39.7	64.6	71.4	86.0	82.6	92.3	73.6	86.8	35.1	43.9	49.6	63.9	26.9	17.5
El Salvador	20 to 24	52.3	73.1	69.7	82.2	61.3	79.2	64.9	78.2	66.4	77.9	71.1	82.0	51.8	71.5	67.3	78.3	58.1	77.0	53.8	77.4	76.7	87.6	11.5	58.2
Equatorial Guinea	20 to 24	63.1	78.8	74.2	83.6	74.3	87.2	59.0	73.5	53.2	65.5	73.7	84.8	72.5	85.1	82.5	87.4	78.4	90.3	83.0	83.6	88.1	88.3	76.1	78.2
Eritrea	20 to 24	39.1	53.9	66.6	74.6	53.7	65.2	39.7	49.1	44.1	51.9	56.1	63.4	55.4	64.2	81.6	83.5	62.3	70.9	79.6	79.9	86.2	87.9	71.5	69.1
Estonia	20 to 24	76.2	87.2	85.4	91.1	82.9	92.3	77.3	88.8	77.7	84.2	81.9	93.3	79.6	93.1	88.7	94.5	81.4	95.1	72.5	88.0	82.0	90.9	61.7	85.4
Eswatini	20 to 24	69.7	62.9	81.5	80.4	74.3	68.3	54.2	48.6	55.0	53.7	65.8	61.2	90.8	85.8	93.1	91.1	92.9	88.5	86.8	80.1	90.5	88.0	81.5	66.8
Ethiopia	20 to 24	51.4	68.9	61.7	77.3	68.4	79.4	50.7	62.4	42.8	55.4	68.9	76.6	69.8	80.1	77.6	86.1	78.3	85.4	83.9	86.3	85.9	88.9	85.4	84.9
Fiji	20 to 24	47.9	58.1	64.5	70.6	61.4	70.2	43.9	51.7	43.6	48.7	58.8	66.6	53.2	60.9	68.6	73.1	61.2	68.2	82.7	83.9	91.9	91.4	65.4	70.9
Finland	20 to 24	75.5	89.5	82.0	92.0	84.2	94.0	81.9	92.9	81.3	89.3	86.5	96.1	64.4	83.0	65.7	84.1	77.8	89.2	73.8	94.0	80.8	94.9	66.9	92.7
France	20 to 24	79.7	89.5	87.0	94.5	85.1	92.3	80.9	94.7	81.7	95.2	84.6	95.4	75.0	84.5	79.0	90.7	82.6	87.3	83.4	94.6	89.5	96.1	73.6	91.7
Gabon	20 to 24	73.7	80.5	82.9	88.4	81.6	85.7	69.6	75.8	65.7	74.4	80.5	83.9	83.7	86.5	89.4	91.5	87.4	89.1	86.7	87.0	90.7	91.8	81.4	79.8
Gambia	20 to 24	71.7	65.2	83.6	76.5	78.8	76.0	67.2	60.1	64.5	53.8	77.8	74.6	79.4	70.2	90.1	82.5	81.5	75.1	76.6	66.7	92.9	87.0	54.4	40.4
Georgia	20 to 24	65.3	79.4	74.3	88.0	76.6	84.2	66.5	81.6	63.3	81.2	76.5	85.9	76.5	78.4	79.6	86.1	83.8	82.5	71.5	79.3	78.7	87.9	64.8	64.6
Germany	20 to 24	72.1	92.0	81.1	94.8	80.5	95.0	77.9	93.9	77.7	91.0	83.6	96.2	45.0	86.7	57.7	89.2	59.3	90.8	70.6	95.2	80.4	96.4	58.5	92.9
Ghana	20 to 24	61.5	70.3	70.1	80.8	75.3	79.6	58.7	67.6	50.0	62.5	75.0	79.2	68.8	71.8	78.9	83.6	75.2	76.7	69.1	70.2	85.3	88.5	47.4	48.0
Greece	20 to 24	64.2	84.1	75.1	89.9	75.2	88.7	63.3	83.7	63.7	82.9	73.0	87.8	73.4	84.4	76.6	89.2	82.1	87.7	62.7	90.4	74.4	93.1	48.7	86.4
Greenland	20 to 24	74.8	92.7	69.2	90.2	92.2	98.9	73.9	91.3	66.8	84.9	85.0	96.5	34.2	81.3	24.9	76.3	68.7	93.5	97.9	100.0	94.9	98.6	90.0	100.0
Grenada	20 to 24	47.3	85.5	66.3	89.1	56.3	90.7	50.5	87.6	58.2	83.9	53.7	91.9	54.4	83.9	68.4	86.7	61.4	88.4	32.3	88.3	55.2	90.0	0.0	87.3
Guam	20 to 24	68.3	78.3	80.7	92.9	76.9	78.9	65.8	73.2	63.1	78.7	76.8	76.5	67.2	77.6	79.7	96.1	75.1	77.8	84.0	88.5	94.1	95.6	66.8	73.4
Guatemala	20 to 24	53.3	67.2	64.2	77.9	68.5	75.4	59.3	70.2	55.4	68.5	71.6	77.5	76.1	73.7	82.5	82.9	80.3	77.2	58.1	78.0	70.0	85.6	44.1	65.9
Guinea	20 to 24	73.9	68.0	76.9	71.6	85.3	82.0	72.3	67.2	60.0	53.1	87.0	84.2	84.8	76.6	87.5	79.4	89.0	84.0	81.3	71.1	90.3	84.9	69.6	53.2
Guinea-Bissau	20 to 24	51.1	54.8	59.7	63.6	69.8	71.9	49.4	50.9	37.1	39.1	70.1	70.8	58.6	58.0	71.0	70.0	68.3	68.4	66.5	62.1	83.4	82.4	45.1	37.3
Guyana	20 to 24	47.1	56.1	68.6	71.0	54.6	65.4	50.1	59.9	55.2	59.7	57.2	68.8	56.3	55.6	78.6	73.7	58.1	60.3	53.2	56.5	73.7	70.5	17.2	37.9
Haiti	20 to 24	6.9	27.2	0.0	20.6	67.1	74.2	23.3	37.8	0.0	16.1	70.8	77.1	12.1	26.3	0.0	10.0	59.2	69.3	0.0	25.3	0.0	25.4	64.7	69.9
Honduras	20 to 24	26.5	65.5	34.1	69.3	57.7	80.6	40.9	74.7	30.7	66.6	65.1	86.7	15.6	51.8	19.6	51.4	45.9	71.5	31.3	74.5	47.6	80.8	18.7	67.8
Hungary	20 to 24	77.3	88.3	85.6	92.3	83.0	92.1	81.5	93.2	80.8	89.1	85.4	95.7	78.0	90.7	82.6	92.7	83.4	93.1	73.5	87.2	84.5	91.4	55.7	80.6
Iceland	20 to 24	77.5	92.6	85.3	94.6	84.3	95.9	82.4	95.0	83.2	91.0	86.4	97.6	64.6	89.8	69.5	90.9	75.8	93.5	81.6	96.6	89.4	97.0	70.5	95.2
India	20 to 24	68.0	76.7	72.8	83.5	81.7	84.2	68.0	75.3	60.8	72.1	81.0	82.9	68.7	77.3	72.0	82.4	79.4	83.5	79.5	85.0	82.0	88.7	80.8	81.3
Indonesia	20 to 24	56.8	60.7	62.3	72.9	75.5	71.4	58.6	59.3	50.1	59.1	75.9	69.8	60.1	66.1	61.0	72.8	75.3	75.3	65.9	66.8	73.4	78.8	61.9	49.9
Iran (Islamic Republic of)	20 to 24	80.3	79.9	85.0	87.6	87.4	85.1	75.8	74.6	74.2	77.9	82.5	79.4	89.9	93.4	91.1	95.3	92.7	94.3	63.0	63.8	72.3	77.5	54.1	45.1
Iraq	20 to 24	55.4	72.7	69.4	84.2	67.7	79.0	52.9	67.9	56.1	71.8	63.9	75.6	79.0	90.0	80.7	91.7	86.1	92.6	43.9	66.5	56.6	77.3	34.0	52.1
Ireland	20 to 24	78.8	93.4	83.6	95.4	86.9	96.2	86.6	96.7	83.5	92.9	91.2	98.7	64.6	90.3	66.7	92.3	77.5	93.0	80.8	96.9	86.0	97.3	74.7	95.6
Israel	20 to 24	76.3	92.5	84.7	94.4	83.3	96.0	73.6	96.0	76.7	92.9	78.7	97.9	72.2	92.5	81.4	93.1	77.9	95.3	82.4	95.6	86.2	96.2	79.0	94.6
Italy	20 to 24	80.6	90.1	86.2	92.7	87.0	94.0	81.8	89.8	79.9	86.5	87.2	93.6	72.7	85.8	73.7	85.5	83.1	91.8	83.0	95.5	88.5	96.6	75.9	93.5
Jamaica	20 to 24	66.5	82.2	79.9	86.0	71.2	88.7	73.8	85.2	72.4	81.5	80.0	89.7	63.7	85.1	81.5	85.4	62.4	90.7	69.4	81.2	78.8	84.5	56.7	80.1
Japan	20 to 24	84.8	90.3	90.7	94.3	88.6	92.8	90.1	94.7	88.8	93.0	92.2	95.5	80.6	87.7	87.5	90.6	82.8	90.4	82.4	91.3	87.2	94.1	77.0	87.4
Jordan	20 to 24	66.6	84.7	71.9	89.7	80.2	89.8	52.9	79.1	50.8	79.6	66.3	83.2	94.4	99.3	94.5	99.4	96.5	99.5	52.6	75.8	58.8	82.9	52.8	67.2
Kazakhstan	20 to 24	72.0	81.3	78.6	88.0	82.5	86.3	72.8	81.3	69.1	79.8	81.6	85.3	71.9	84.7	76.2	89.2	80.1	87.7	60.2	77.1	74.1	86.3	42.6	61.6
Kenya	20 to 24	76.5	76.5	83.9	86.8	83.8	82.0	70.0	68.9	65.7	68.5	80.6	78.5	85.0	83.4	89.9	91.2	88.6	86.5	89.7	88.8	91.9	92.7	87.7	83.1
Kiribati	20 to 24	50.5	42.3	74.2	72.4	61.0	53.5	52.8	45.4	54.2	54.0	66.2	57.4	54.2	50.6	85.5	81.8	54.9	57.5	80.8	73.1	92.2	90.2	61.5	45.9
Kuwait	20 to 24	81.3	88.1	88.3	93.0	86.5	91.4	74.6	85.1	77.4	84.5	78.5	88.0	91.6	99.8	92.4	100.0	94.1	99.5	78.3	81.9	84.5	89.3	70.5	70.5
Kyrgyzstan	20 to 24	61.8	78.4	72.6	85.1	73.3	85.3	61.6	81.8	60.2	78.7	71.4	87.4	70.0	79.5	75.6	83.3	78.1	85.3	50.2	69.1	67.6	80.4	25.9	52.2

Lao People's Democratic Republic	20 to 24	37.0	52.5	45.5	61.4	62.4	70.2	42.4	55.9	31.6	49.0	65.7	72.2	44.3	60.4	51.1	64.6	61.1	73.3	48.5	58.6	58.5	69.0	45.8	49.3
Latvia	20 to 24	75.7	83.8	84.0	90.4	82.5	87.8	71.9	84.7	72.1	84.5	78.0	85.9	81.3	87.8	88.0	92.8	84.3	89.2	71.7	84.0	80.8	89.8	60.4	75.3
Lebanon	20 to 24	66.7	82.4	80.5	90.4	74.0	87.0	59.5	76.7	67.3	80.7	65.3	80.8	81.2	92.4	86.9	95.4	83.9	93.0	74.9	85.5	79.4	89.4	72.5	81.4
Lesotho	20 to 24	77.3	58.0	87.1	75.4	79.4	65.1	64.7	44.8	67.1	48.6	71.1	58.0	94.2	84.3	96.7	89.2	94.7	87.8	90.5	78.7	93.2	86.4	86.2	66.4
Liberia	20 to 24	72.8	64.7	76.3	65.2	84.4	82.1	71.6	60.7	58.4	41.7	86.8	82.6	82.1	74.2	84.9	74.0	87.7	84.3	79.3	70.4	89.4	81.1	66.3	58.5
Libya	20 to 24	74.4	70.5	79.5	77.7	83.9	80.4	67.5	62.5	63.6	60.8	78.2	72.9	88.8	85.2	89.0	84.5	92.8	91.5	69.2	54.5	74.6	68.3	66.5	37.2
Lithuania	20 to 24	80.8	87.9	87.9	92.0	85.9	92.3	78.4	89.0	77.2	85.6	83.2	92.5	79.5	91.4	88.5	93.9	81.3	93.1	79.6	88.7	86.4	91.6	70.5	85.3
Luxembourg	20 to 24	67.4	93.0	80.4	95.2	75.4	95.8	70.7	95.3	74.9	93.1	77.1	96.9	59.2	91.3	69.4	91.6	69.3	94.8	64.7	95.7	79.0	96.5	45.9	94.0
Madagascar	20 to 24	23.5	31.4	47.2	49.5	46.3	53.9	31.4	29.3	28.0	20.5	52.8	53.7	34.6	38.7	58.2	55.7	51.2	56.4	63.0	65.1	75.8	76.7	46.4	51.2
Malawi	20 to 24	61.4	59.5	68.2	74.0	76.4	70.5	54.5	49.5	44.5	47.5	72.8	65.1	70.9	65.3	76.4	78.4	79.9	73.4	82.5	78.3	85.6	86.0	81.2	68.2
Malaysia	20 to 24	69.0	80.4	80.7	87.8	77.2	86.0	73.0	80.4	72.6	79.2	80.4	86.1	81.2	90.5	87.4	92.5	84.0	92.9	71.9	81.9	82.2	87.9	58.0	74.0
Maldives	20 to 24	60.2	78.6	65.2	88.5	77.4	85.2	67.7	79.9	58.5	80.8	81.8	86.6	70.9	88.6	73.8	93.4	80.5	91.0	63.0	79.8	69.8	88.3	60.6	69.7
Mali	20 to 24	63.6	66.5	62.1	64.6	83.4	84.3	58.8	61.3	38.3	35.6	83.4	85.3	78.3	76.8	76.0	73.8	88.2	87.4	79.9	76.0	85.4	84.5	75.2	66.5
Malta	20 to 24	73.4	88.5	79.5	93.4	83.4	91.7	76.2	90.1	72.8	88.2	84.7	92.8	76.1	87.4	78.9	92.8	84.1	89.1	68.7	93.8	76.1	95.6	62.1	90.7
Marshall Islands	20 to 24	0.0	31.1	68.2	52.7	0.0	50.0	0.0	33.1	47.1	28.8	0.0	54.1	0.0	34.9	71.9	56.6	0.0	49.9	69.3	77.3	92.4	87.8	24.5	57.5
Mauritania	20 to 24	69.3	80.8	76.2	85.5	80.5	87.7	66.4	78.3	56.1	68.1	81.3	89.8	78.9	87.0	85.3	90.1	83.7	90.3	74.5	80.8	89.1	91.8	54.4	64.6
Mauritius	20 to 24	75.1	71.2	80.6	85.1	84.6	75.4	77.6	75.2	72.6	79.7	86.1	77.6	79.9	73.0	83.7	84.3	85.2	75.5	68.0	66.1	75.9	81.4	60.8	41.3
Mexico	20 to 24	78.0	81.0	82.0	87.9	86.6	85.1	83.0	88.7	77.7	87.2	89.6	90.5	74.2	71.7	76.0	80.3	82.9	76.0	73.9	84.7	81.2	90.5	64.3	74.5
Micronesia (Federated States of)	20 to 24	37.1	36.2	57.7	59.9	53.0	52.0	39.1	40.8	35.4	41.0	57.9	57.0	35.3	36.8	61.9	62.7	45.4	49.4	81.1	75.8	90.4	88.2	63.1	53.8
Monaco	20 to 24	70.1	82.8	75.6	86.0	81.7	90.0	73.0	84.6	72.4	83.2	80.4	89.1	56.5	77.0	53.8	73.8	76.0	88.5	67.7	82.9	73.5	85.3	64.8	82.1
Mongolia	20 to 24	75.1	79.4	80.4	86.0	84.3	85.5	73.9	78.0	68.3	75.1	83.5	84.5	70.6	75.1	71.2	76.8	81.9	84.0	89.4	88.0	92.1	91.4	85.9	83.4
Montenegro	20 to 24	59.3	80.1	71.6	87.4	70.8	84.8	59.2	79.4	60.1	79.2	68.9	81.9	80.6	91.5	82.5	92.9	87.0	94.0	83.4	88.4	88.7	91.7	76.5	84.0
Morocco	20 to 24	63.0	78.8	63.4	81.2	82.8	88.8	56.2	74.5	43.6	67.9	77.0	84.9	81.1	90.3	78.5	89.2	90.3	94.8	52.0	67.3	54.5	71.5	64.9	69.1
Mozambique	20 to 24	69.4	59.8	77.2	77.2	79.7	69.1	62.1	53.4	55.6	54.9	76.3	66.6	79.0	64.0	85.5	81.6	83.8	70.4	85.6	77.7	89.0	87.6	82.3	63.3
Myanmar	20 to 24	25.4	48.5	35.5	69.7	55.0	59.0	30.9	51.0	19.2	57.2	58.0	61.0	30.9	56.4	37.9	72.6	53.3	63.5	43.1	59.2	56.4	76.4	33.2	33.0
Namibia	20 to 24	72.1	76.8	82.4	88.4	78.1	79.8	59.2	65.8	57.7	70.3	71.1	73.1	91.4	92.6	93.6	95.9	93.7	93.7	85.9	86.5	90.3	92.1	79.3	76.3
Nauru	20 to 24	33.7	28.2	51.1	46.0	53.7	50.9	31.2	23.6	23.7	14.4	54.7	50.2	29.9	27.1	49.8	41.2	45.6	48.0	71.8	69.2	87.2	84.4	44.3	41.7
Nepal	20 to 24	66.6	73.0	73.4	84.8	78.8	77.0	65.5	73.0	61.0	74.8	78.4	76.4	68.8	73.3	75.6	85.7	76.4	75.2	79.5	82.8	83.2	89.8	78.7	70.4
Netherlands	20 to 24	83.2	92.8	87.2	94.3	89.7	96.4	86.4	95.6	84.0	91.7	90.7	98.0	68.4	87.9	72.2	87.7	78.7	93.2	88.1	96.2	90.4	96.4	85.7	95.6
New Zealand	20 to 24	81.4	93.4	87.2	95.7	87.5	95.9	87.0	97.8	86.1	94.4	90.2	99.0	61.4	87.9	66.0	89.8	73.3	91.3	84.9	94.6	94.4	96.8	70.7	90.9
Nicaragua	20 to 24	71.8	82.5	73.8	85.7	86.1	89.8	75.5	87.8	65.9	81.9	89.1	93.6	73.9	81.9	73.4	83.5	85.0	88.1	73.4	84.6	78.5	88.5	71.7	79.6
Niger	20 to 24	77.1	77.6	80.2	80.1	86.9	87.7	74.6	74.4	63.0	62.1	88.1	88.3	87.9	87.7	90.2	89.4	91.3	91.6	87.7	85.9	93.2	91.8	81.0	78.9
Nigeria	20 to 24	86.1	88.6	91.0	94.3	90.4	90.8	82.3	86.3	75.8	80.7	90.2	92.4	94.9	94.7	97.1	98.0	95.7	94.6	90.1	87.7	96.3	96.7	81.3	73.0
Niue	20 to 24	59.0	51.0	75.7	54.2	69.2	73.1	57.7	46.9	57.1	27.0	70.1	72.5	56.2	49.3	77.2	49.0	63.3	68.5	85.9	83.1	93.4	88.9	72.6	73.7
North Macedonia	20 to 24	66.5	79.5	70.7	84.9	81.8	86.7	73.6	83.7	66.9	79.7	84.7	89.2	54.5	74.9	49.1	76.6	77.3	83.8	66.1	80.6	73.5	86.6	62.1	72.7
Northern Mariana Islands	20 to 24	67.7	75.9	78.7	88.9	75.7	80.2	63.3	71.8	61.6	73.1	74.1	79.5	71.4	77.7	75.6	90.4	79.6	80.3	88.9	91.2	94.9	96.0	75.1	81.5
Norway	20 to 24	86.2	96.2	90.8	97.3	90.9	98.2	90.2	96.2	87.5	92.3	93.4	98.5	73.5	94.1	81.1	94.1	79.3	96.6	90.3	98.9	91.0	98.8	91.0	98.0
Oman	20 to 24	73.8	85.0	83.0	89.8	82.5	90.8	69.1	80.2	73.1	80.5	75.2	84.9	93.4	100.0	94.5	99.7	95.4	100.0	70.7	81.4	76.0	85.6	65.4	77.1
Pakistan	20 to 24	72.5	63.2	75.4	66.3	85.4	80.6	71.6	63.6	62.6	52.2	84.8	81.1	70.7	57.1	71.2	53.8	82.1	76.0	82.7	73.0	84.2	77.0	84.2	74.0
Palau	20 to 24	51.6	44.4	68.4	42.8	65.8	71.4	50.2	43.7	45.2	12.0	67.1	71.9	58.0	49.4	69.2	33.1	69.4	73.8	78.3	73.0	90.4	82.3	58.1	62.1
Palestine	20 to 24	64.8	75.9	77.2	85.6	72.8	80.7	53.8	65.6	61.1	72.5	59.6	68.2	94.3	97.6	95.4	98.0	95.6	98.2	55.6	68.9	69.0	79.6	39.7	53.7
Panama	20 to 24	72.6	81.4	74.9	85.0	85.7	88.9	77.9	87.4	72.2	82.9	86.6	92.2	72.5	77.1	74.1	80.0	81.9	84.0	67.2	85.1	71.3	88.0	67.0	82.0
Papua New Guinea	20 to 24	67.0	65.1	63.8	63.4	85.9	83.4	51.8	47.4	25.4	20.9	81.3	77.6	77.0	76.4	75.7	76.0	86.4	85.5	94.1	93.2	94.5	94.5	93.1	90.3
Paraguay	20 to 24	58.7	74.6	60.4	80.5	79.5	83.8	68.5	79.9	57.5	77.1	84.2	86.0	53.6	72.5	49.8	73.1	75.5	83.2	72.8	85.0	76.0	89.3	75.2	79.6
Peru	20 to 24	40.0	58.3	56.5	71.6	57.8	70.3	53.6	64.5	52.7	66.0	65.8	72.7	39.6	56.0	50.8	62.8	56.3	69.5	60.1	75.6	73.1	84.1	42.9	64.2
Philippines	20 to 24	66.2	62.9	79.0	76.4	72.2	71.7	61.9	62.8	67.3	65.2	65.4	70.5	75.4	69.5	81.3	75.8	80.9	77.7	71.8	69.0	82.6	80.1	54.4	53.3
Poland	20 to 24	70.7	87.0	82.4	91.8	76.6	90.4	76.6	91.0	79.9	89.9	79.1	91.5	64.9	88.0	74.2	90.4	72.1	91.0	72.7	87.4	83.8	91.1	55.3	82.2
Portugal	20 to 24	47.7	88.8	64.7	91.4	63.0	93.0	56.5	93.5	59.7	92.2	67.5	94.8	38.8	83.6	51.6	84.2	55.7	89.9	30.6	90.8	54.3	92.3	3.0	88.8

Puerto Rico	20 to 24	84.7	94.1	91.0	96.5	86.9	95.3	88.7	96.4	86.2	92.1	91.0	97.9	81.6	93.4	89.6	96.4	80.7	92.9	86.8	97.3	92.0	98.4	76.5	92.7
Qatar	20 to 24	77.5	89.2	85.2	93.4	85.9	93.8	75.7	87.5	74.1	85.1	83.6	92.4	80.1	94.7	92.7	98.8	82.8	95.8	86.0	90.9	87.5	92.8	83.4	88.2
Republic of Korea	20 to 24	75.0	90.9	81.6	93.6	83.6	94.2	82.8	93.8	79.2	89.9	88.8	96.4	50.0	86.0	57.9	87.5	64.6	90.4	73.3	89.8	80.3	92.1	66.1	87.3
Republic of Moldova	20 to 24	67.4	81.4	78.0	87.9	75.8	86.8	58.8	78.6	60.6	78.6	66.5	82.3	82.0	88.7	86.1	92.2	86.4	90.9	74.4	84.8	83.0	89.0	62.8	80.5
Romania	20 to 24	75.2	79.4	82.7	87.7	82.4	83.6	76.4	84.8	75.2	85.3	81.9	85.9	76.1	74.5	78.5	81.9	83.9	79.6	74.3	79.7	83.7	87.0	60.6	68.6
Russian Federation	20 to 24	72.5	77.0	82.4	87.3	78.9	80.3	70.0	72.8	71.2	79.1	75.8	73.1	72.1	77.8	81.2	84.0	77.4	82.5	63.1	76.6	75.9	86.0	44.9	60.4
Rwanda	20 to 24	28.7	67.8	39.3	79.1	56.9	76.7	33.4	61.0	20.9	59.8	59.1	72.5	48.7	77.0	56.8	86.5	65.3	81.8	68.7	86.2	71.8	89.6	73.1	82.8
Saint Kitts and Nevis	20 to 24	63.4	81.1	72.2	88.8	74.8	85.1	66.2	83.7	62.4	82.6	76.1	88.7	66.1	78.5	74.1	89.3	73.2	78.9	62.7	84.0	71.1	87.9	57.4	80.1
Saint Lucia	20 to 24	68.0	80.7	80.8	91.8	72.1	80.5	71.5	82.4	75.9	88.3	72.6	80.2	70.6	81.4	81.4	90.3	72.3	81.3	56.9	82.5	67.6	90.7	47.7	66.3
Saint Vincent and the Grenadines	20 to 24	63.6	75.9	69.1	82.6	78.4	82.8	62.7	76.4	55.3	75.0	77.5	81.8	69.5	80.4	74.4	84.2	77.9	85.3	64.2	75.3	68.5	80.0	72.5	73.3
Samoa	20 to 24	60.6	58.3	76.4	72.5	70.0	69.1	58.5	57.0	58.2	55.5	70.1	69.0	59.0	59.8	78.3	74.6	66.1	68.0	89.8	85.4	94.2	91.3	81.2	74.6
San Marino	20 to 24	85.5	91.5	89.7	93.8	90.6	95.2	85.8	92.8	83.5	88.9	90.0	95.9	85.0	91.8	87.9	92.8	89.3	94.7	86.2	93.5	90.4	94.8	80.8	91.4
Sao Tome and Principe	20 to 24	82.9	70.0	81.8	81.7	92.7	78.9	81.4	69.0	64.6	66.8	95.5	78.4	90.8	70.0	88.7	84.7	95.5	74.4	86.2	69.0	90.6	87.3	82.2	47.6
Saudi Arabia	20 to 24	59.8	73.5	60.5	80.6	79.7	82.6	49.9	67.7	36.2	67.9	71.8	76.1	88.9	93.4	87.3	93.5	94.2	95.8	46.4	57.6	49.4	69.3	53.3	45.0
Senegal	20 to 24	67.9	74.5	75.6	81.8	79.0	83.2	63.2	71.4	53.5	61.3	78.7	84.2	75.2	77.2	82.7	84.5	80.6	82.6	73.5	76.8	88.2	90.3	53.4	59.7
Serbia	20 to 24	70.4	86.9	78.0	90.8	79.8	91.4	76.4	92.2	74.1	88.3	83.1	95.0	59.2	86.0	60.0	86.3	75.0	91.5	68.2	85.3	79.5	90.0	52.6	78.9
Seychelles	20 to 24	62.5	77.1	74.7	77.7	72.8	89.2	65.9	77.8	65.5	67.9	75.0	89.4	71.9	87.7	78.0	83.0	78.7	95.6	57.1	72.4	74.5	75.6	31.6	73.6
Sierra Leone	20 to 24	76.5	69.6	83.0	71.0	84.0	84.2	74.5	68.0	65.5	50.4	86.2	87.5	83.7	76.4	88.8	76.6	87.2	85.5	78.5	70.8	91.4	83.0	59.4	55.4
Singapore	20 to 24	85.9	93.4	88.5	95.8	92.0	95.6	89.1	95.2	84.9	91.4	93.5	97.1	86.0	95.5	86.4	96.4	91.2	96.6	79.4	92.9	81.2	94.2	81.7	91.3
Slovakia	20 to 24	77.4	86.1	84.7	90.2	84.1	91.3	79.8	90.6	78.7	86.9	84.4	93.8	78.2	87.9	81.5	88.7	84.5	92.3	76.8	85.7	85.0	89.9	65.3	80.5
Slovenia	20 to 24	82.9	90.8	90.0	93.3	86.5	95.0	87.2	96.0	86.3	90.0	89.4	99.6	83.7	95.5	88.4	96.3	86.7	96.9	82.0	90.2	89.7	92.5	68.5	87.5
Solomon Islands	20 to 24	79.1	73.4	85.3	82.0	85.8	81.3	74.0	70.3	68.4	66.0	84.0	80.3	86.3	81.4	92.9	89.5	87.5	84.1	94.5	90.7	95.4	92.9	92.3	87.1
Somalia	20 to 24	47.5	57.8	65.2	73.3	63.4	69.9	43.7	49.8	40.9	48.1	61.4	65.4	60.7	68.2	78.2	82.3	70.0	75.9	83.7	85.3	86.7	89.1	81.7	81.4
South Sudan	20 to 24	70.5	77.5	71.1	72.0	72.5	73.8	56.3	61.1	48.7	44.8	70.1	68.4	89.5	92.0	82.4	81.2	79.9	79.3	80.9	85.1	87.1	86.3	80.1	77.8
Spain	20 to 24	59.0	61.4	78.7	92.6	75.2	92.9	54.2	51.2	74.3	91.0	78.7	94.7	73.0	72.4	70.6	87.4	67.9	89.5	83.4	81.9	78.1	94.8	54.5	89.3
Sri Lanka	20 to 24	65.8	89.1	74.8	83.9	70.6	79.3	71.4	92.7	61.2	73.7	68.5	76.8	58.8	84.9	87.8	92.6	87.5	92.3	67.1	92.6	70.3	78.7	17.4	37.1
Sudan	20 to 24	61.7	73.7	50.1	69.1	78.8	82.9	59.7	71.7	27.8	48.7	71.2	76.4	83.9	90.2	73.2	79.6	88.6	90.3	50.2	63.0	47.0	58.1	71.7	57.9
Suriname	20 to 24	51.8	67.1	66.5	70.8	64.0	69.6	43.7	59.0	55.8	60.1	65.8	70.2	77.1	81.9	70.0	71.3	64.9	69.1	48.3	53.7	73.8	75.3	47.4	55.2
Sweden	20 to 24	51.9	59.1	92.1	98.7	92.8	97.8	55.6	60.8	94.7	100.0	94.8	99.6	56.5	61.2	82.9	93.8	87.2	95.9	62.2	65.9	88.9	97.9	84.6	95.4
Switzerland	20 to 24	88.7	96.6	81.0	95.8	82.2	96.6	94.1	100.0	79.2	93.7	84.4	98.8	80.8	93.4	64.5	93.2	72.0	94.4	86.8	97.1	81.9	97.1	62.5	95.4
Syrian Arab Republic	20 to 24	73.5	93.9	63.5	76.8	64.6	75.3	79.2	97.1	48.7	61.0	59.4	68.6	59.0	91.8	88.8	96.4	88.5	96.1	73.2	96.7	55.2	70.1	30.7	46.6
Taiwan (Province of China)	20 to 24	48.3	66.1	87.7	94.2	87.1	91.6	45.9	59.7	80.2	90.0	87.3	91.9	85.4	95.8	92.5	96.9	95.0	95.4	41.9	58.6	86.9	91.3	71.1	78.6
Tajikistan	20 to 24	81.7	89.3	63.1	79.2	79.1	82.5	82.3	90.6	46.4	66.1	76.3	79.2	92.2	94.9	70.3	83.0	87.5	88.0	80.2	86.3	58.8	77.3	38.9	55.3
Thailand	20 to 24	59.4	72.8	84.7	84.1	77.6	69.6	56.5	69.8	76.3	75.7	81.9	73.4	74.3	81.5	85.4	84.2	73.7	67.9	46.5	67.4	87.4	86.1	68.5	54.0
Timor-Leste	20 to 24	71.6	65.2	68.7	66.9	75.5	68.3	75.6	68.5	56.7	54.2	76.3	69.4	71.6	66.7	74.3	70.2	77.3	71.4	79.8	74.0	78.1	75.7	65.0	44.8
Togo	20 to 24	61.1	54.4	72.6	75.3	82.7	81.6	62.6	56.4	50.0	54.0	82.8	82.0	68.6	61.8	81.1	81.0	85.6	83.6	71.2	62.5	87.8	87.2	64.3	57.4
Tokelau	20 to 24	69.3	69.5	64.7	40.5	62.1	58.3	65.1	66.5	44.3	15.0	64.1	56.6	78.5	76.5	68.6	37.2	61.1	57.9	77.2	73.5	91.4	85.0	68.5	59.7
Tonga	20 to 24	49.5	30.9	87.7	86.2	87.6	80.8	48.3	27.4	70.8	69.4	85.0	78.9	52.5	34.1	91.1	88.9	86.2	79.6	84.3	75.9	96.1	94.7	87.3	79.2
Trinidad and Tobago	20 to 24	82.3	75.9	81.4	86.2	73.9	78.1	76.1	70.8	72.8	81.6	77.5	85.1	84.1	77.0	76.6	78.2	64.6	59.3	93.5	89.7	83.5	87.1	35.9	61.2
Tropical Latin America	20 to 24	69.4	75.5	66.4	76.3	68.6	77.4	73.3	82.5	60.7	70.8	70.9	77.5	61.4	58.6	58.8	68.2	65.9	75.3	67.0	77.8	76.4	84.8	49.0	68.6
Tunisia	20 to 24	77.7	81.1	81.7	86.9	87.0	87.3	73.5	78.1	68.3	76.5	83.3	84.1	91.9	94.2	92.3	94.9	94.5	95.7	74.5	73.6	78.4	81.9	73.4	63.3
Turkey	20 to 24	73.1	84.3	81.4	90.1	80.8	88.6	73.1	82.5	70.9	81.2	80.7	86.6	68.7	87.2	79.1	92.3	73.2	88.2	72.3	82.9	80.3	88.5	62.3	74.6
Turkmenistan	20 to 24	68.0	57.0	74.1	72.5	80.8	66.9	68.0	56.0	62.5	60.2	79.5	63.6	70.1	50.0	72.7	63.8	80.2	61.7	53.4	43.8	66.0	65.4	40.5	11.9
Tuvalu	20 to 24	34.7	38.9	53.5	61.6	52.4	53.9	36.8	42.1	30.4	43.0	58.0	57.4	36.4	37.3	59.1	61.1	47.6	51.2	80.7	75.0	90.0	88.3	60.7	51.3
Uganda	20 to 24	66.3	69.2	81.0	82.1	73.7	77.0	57.8	61.9	59.3	61.3	69.5	73.7	73.9	75.6	87.2	87.4	78.0	80.2	87.6	86.1	91.1	90.5	83.3	80.6
Ukraine	20 to 24	77.9	81.5	87.3	89.5	82.1	85.9	75.4	80.9	76.9	80.3	78.1	85.1	81.4	84.5	87.4	89.3	84.7	88.0	75.5	82.2	88.2	90.7	52.5	68.9
United Arab Emirates	20 to 24	82.8	78.7	86.8	89.3	90.0	81.1	79.0	71.1	76.7	80.4	85.4	68.9	86.5	87.1	87.9	91.7	91.1	88.2	80.3	77.3	83.8	87.0	76.4	60.4
United Kingdom	20 to 24	82.6	92.6	87.3	95.2	88.3	95.1	87.8	93.8	86.0	91.5	91.4	95.8	57.5	86.5	62.7	88.1	70.9	90.9	88.0	97.9	91.7	98.5	82.7	95.9

United Republic of Tanzania	20 to 24	63.5	73.0	78.0	83.8	72.2	79.9	57.9	65.9	58.2	65.7	69.7	75.7	73.0	75.9	86.0	87.1	77.4	80.3	86.6	84.2	90.4	90.2	81.8	75.8
United States Virgin Islands	20 to 24	71.5	68.3	85.7	90.2	72.3	58.8	76.6	70.4	80.9	86.3	76.3	53.9	68.2	69.6	83.5	88.7	67.0	62.0	79.9	73.2	88.7	91.2	62.5	27.1
United States of America	20 to 24	97.4	100.0	96.6	100.0	99.4	100.0	96.0	98.4	92.0	95.6	98.2	99.0	79.0	88.1	81.5	90.9	85.0	90.0	97.8	99.6	98.3	100.0	95.1	96.9
Uruguay	20 to 24	65.2	77.5	72.7	85.2	78.1	83.8	79.8	87.3	75.3	85.1	87.2	90.5	37.7	63.2	46.1	73.4	56.7	70.3	76.9	85.8	81.4	89.9	74.3	80.9
Uzbekistan	20 to 24	58.9	74.2	65.3	83.2	76.0	81.0	50.7	72.4	44.3	71.6	68.2	79.1	80.3	83.6	81.4	88.8	87.5	86.7	50.5	65.9	62.3	80.3	42.0	42.2
Vanuatu	20 to 24	53.6	49.1	71.5	69.8	62.9	59.5	49.1	47.9	49.7	50.1	61.9	60.3	47.3	43.3	70.3	67.3	54.6	53.7	84.3	80.6	92.8	90.9	66.3	60.9
Venezuela (Bolivarian Republic of)	20 to 24	67.9	68.0	77.3	78.1	76.3	74.0	75.9	78.1	73.3	75.4	82.1	82.2	62.5	59.7	71.5	71.0	69.4	62.1	71.9	73.2	83.0	84.5	52.6	49.6
Viet Nam	20 to 24	71.2	74.0	83.4	88.2	77.0	77.6	71.9	74.7	73.6	81.2	77.5	77.3	79.1	83.3	88.3	93.0	80.5	83.5	77.5	76.0	86.1	87.7	63.9	57.3
Yemen	20 to 24	74.3	74.0	78.8	78.1	84.7	85.0	67.1	68.2	62.0	62.4	78.6	80.1	92.3	89.8	94.0	90.2	93.7	93.3	76.2	66.2	77.8	71.2	80.6	66.6
Zambia	20 to 24	54.1	61.9	60.2	73.0	73.3	74.0	50.5	56.1	36.0	48.6	72.4	72.3	69.8	72.3	73.8	80.8	79.7	79.4	82.5	81.5	84.7	86.6	83.7	75.7
Zimbabwe	20 to 24	79.7	55.0	89.2	72.8	81.8	63.3	68.8	32.3	70.1	34.5	76.0	50.0	83.5	61.5	92.2	73.5	86.1	72.2	89.1	73.4	92.6	81.9	82.7	62.0
Afghanistan	25 to 29	44.2	48.1	49.1	54.1	63.2	60.1	30.5	28.9	41.4	38.9	46.9	43.0	76.7	72.9	80.6	79.9	82.7	78.6	38.1	30.6	40.1	34.7	76.9	52.6
Albania	25 to 29	73.4	84.5	80.0	88.0	75.4	86.7	68.2	80.7	74.6	83.4	70.5	83.6	76.4	87.8	83.4	90.6	80.3	90.7	86.6	89.6	89.4	91.5	86.9	89.8
Algeria	25 to 29	67.8	80.5	71.0	84.5	76.1	83.5	55.7	69.2	60.2	74.1	65.7	73.5	83.9	91.7	87.0	93.5	88.0	93.4	58.1	69.6	59.8	75.4	72.1	73.4
American Samoa	25 to 29	55.3	51.9	79.3	71.0	46.0	48.5	45.6	41.8	67.9	60.7	39.9	40.3	57.1	53.3	85.9	76.1	56.2	57.8	81.8	77.4	86.9	80.6	81.3	82.1
Andorra	25 to 29	83.0	91.0	82.9	91.4	89.4	94.2	84.8	92.5	83.4	90.6	90.2	95.2	69.9	84.1	67.6	84.4	84.7	90.8	87.5	94.9	87.8	94.9	90.9	95.9
Angola	25 to 29	71.0	76.8	74.8	81.6	77.5	80.1	59.0	63.7	61.1	67.0	70.2	71.8	79.2	80.7	83.6	86.1	85.0	85.1	85.3	85.3	86.3	87.7	89.8	87.3
Antigua and Barbuda	25 to 29	66.0	87.5	75.7	91.3	65.5	87.0	59.4	86.0	68.4	87.8	60.8	86.0	66.6	86.0	78.1	90.9	69.2	87.1	73.3	92.3	80.5	94.2	72.8	91.2
Argentina	25 to 29	58.9	80.3	68.2	85.7	63.7	81.6	68.1	82.0	73.4	85.2	72.0	82.6	41.5	73.8	57.0	80.5	53.0	79.2	76.3	90.8	80.4	92.9	79.5	90.6
Armenia	25 to 29	75.8	85.5	83.1	89.9	76.4	85.7	66.8	84.6	76.8	85.4	63.5	85.6	83.1	87.2	90.5	94.5	83.6	86.3	70.3	82.7	80.5	89.6	66.7	77.7
Australia	25 to 29	77.6	89.9	81.8	90.6	81.6	93.4	80.6	94.0	81.4	91.0	84.8	97.0	58.8	86.8	72.7	91.0	62.9	88.4	82.0	92.8	86.0	92.9	82.9	93.7
Austria	25 to 29	80.9	94.3	84.9	95.0	84.1	96.0	78.9	94.8	82.7	92.3	80.8	96.9	73.7	90.2	78.7	92.5	81.0	92.7	80.6	97.8	85.2	97.8	81.6	97.7
Azerbaijan	25 to 29	66.6	82.4	75.2	87.4	67.8	82.7	53.3	75.0	64.2	80.5	55.2	74.7	87.4	92.4	91.6	95.0	89.1	93.6	73.0	85.3	81.4	89.8	69.2	82.6
Bahamas	25 to 29	59.9	78.9	70.5	88.2	60.5	74.1	51.2	73.6	63.1	83.1	52.5	69.1	60.8	77.3	71.3	86.8	67.1	76.9	67.0	83.4	75.8	90.6	67.1	77.3
Bahrain	25 to 29	80.9	84.1	88.1	91.5	81.4	84.5	70.7	75.8	80.1	85.0	69.7	75.2	88.9	93.1	94.1	96.7	89.8	94.0	79.9	78.2	85.1	86.8	81.0	77.1
Bangladesh	25 to 29	21.8	48.8	56.2	77.1	17.5	39.2	22.4	44.7	50.8	70.1	19.3	36.1	17.3	46.7	62.6	77.1	25.5	50.8	52.8	65.6	67.8	82.5	51.5	54.2
Barbados	25 to 29	70.6	86.0	80.9	91.5	68.1	83.4	68.8	86.9	77.0	89.7	67.9	84.2	64.9	80.4	78.5	88.3	66.8	80.7	70.0	88.1	80.5	92.9	65.7	83.9
Belarus	25 to 29	75.2	82.7	85.8	89.1	71.0	81.3	65.3	77.4	78.1	84.3	60.5	75.1	77.5	80.9	86.7	88.8	79.5	82.5	71.6	84.4	85.1	90.0	59.4	80.5
Belgium	25 to 29	71.7	94.1	79.2	94.9	74.2	95.5	66.2	95.6	74.0	93.1	68.3	97.2	61.2	87.8	68.2	90.7	72.2	90.9	73.1	96.7	82.3	96.7	71.1	97.0
Belize	25 to 29	51.3	73.7	81.6	83.9	30.5	69.5	42.8	65.9	74.3	77.4	24.7	61.4	51.3	76.5	83.4	84.4	37.7	78.1	67.9	80.1	90.6	89.6	43.1	71.6
Benin	25 to 29	83.1	83.7	85.4	86.4	86.8	87.0	73.4	74.7	71.8	73.1	83.4	83.7	90.3	88.7	92.8	91.4	92.1	91.4	88.4	85.7	92.9	91.6	85.6	82.8
Bermuda	25 to 29	83.3	94.1	88.8	95.4	81.7	94.1	83.0	94.9	84.8	90.8	82.8	98.5	83.7	94.9	90.5	97.4	83.0	94.0	77.0	91.2	87.2	97.6	69.3	82.1
Bhutan	25 to 29	70.1	79.9	73.9	85.9	76.9	81.4	61.7	73.7	65.4	78.5	70.4	76.1	71.4	79.0	77.0	86.0	79.5	83.1	79.7	85.3	79.9	89.0	86.2	85.1
Bolivia (Plurinational State of)	25 to 29	28.1	61.7	34.4	67.1	48.5	69.7	28.5	60.8	35.9	65.0	49.3	69.1	28.6	54.1	36.7	59.3	53.8	69.9	49.8	79.4	55.2	83.3	65.4	81.7
Bosnia and Herzegovina	25 to 29	74.5	84.1	82.5	86.9	76.0	87.4	75.3	86.1	81.5	87.2	74.7	86.3	57.4	77.1	74.7	85.1	60.9	79.9	73.4	81.7	81.4	85.5	72.5	82.8
Botswana	25 to 29	68.4	83.5	80.0	88.2	66.0	84.3	47.4	69.4	57.4	73.1	54.5	75.0	86.5	93.7	92.6	96.2	87.4	94.6	84.0	89.5	89.7	93.1	79.7	87.0
Brazil	25 to 29	41.6	67.1	56.0	75.9	45.5	68.4	40.9	64.6	56.7	74.3	41.3	63.1	35.9	57.9	49.9	67.6	50.1	66.8	55.3	76.1	69.5	83.3	52.9	74.9
Brunei Darussalam	25 to 29	62.6	82.3	76.3	96.0	62.9	73.9	67.7	82.2	77.2	91.5	68.3	73.3	36.9	69.1	58.2	96.1	47.9	61.8	62.7	87.3	74.0	95.2	62.4	81.0
Bulgaria	25 to 29	60.5	70.6	73.8	81.8	59.7	68.5	55.8	65.5	68.4	78.4	55.7	60.5	63.8	64.1	76.4	76.0	68.1	69.0	59.0	68.2	72.9	78.8	55.2	65.2
Burkina Faso	25 to 29	83.1	83.1	85.8	87.2	86.3	84.8	73.3	73.6	72.9	74.4	82.5	80.7	92.3	91.3	95.6	95.6	92.4	91.0	89.8	87.8	93.6	93.3	87.3	83.7
Burundi	25 to 29	50.4	66.1	60.7	73.9	57.5	70.5	42.8	52.0	51.8	58.6	52.7	60.8	61.2	75.5	73.1	84.6	70.0	80.4	71.8	82.6	72.8	84.2	82.7	86.7
Cabo Verde	25 to 29	66.7	86.7	80.4	92.3	65.0	87.2	59.8	82.3	70.0	82.9	61.9	86.6	73.5	90.4	88.6	96.3	72.6	90.2	64.3	81.5	87.0	93.6	48.8	74.8
Cambodia	25 to 29	49.3	69.8	54.3	77.2	62.6	72.6	39.3	62.8	45.2	69.1	56.4	68.1	64.4	78.8	69.7	84.8	75.0	82.9	64.6	77.3	68.8	82.0	74.9	79.2
Cameroon	25 to 29	72.2	74.7	78.1	80.9	76.1	77.9	60.2	65.0	64.5	67.8	68.8	73.0	82.4	80.4	90.1	89.4	83.5	82.1	84.3	79.1	90.8	88.7	81.0	74.8
Canada	25 to 29	87.5	93.3	88.3	93.7	91.7	95.5	90.7	94.1	88.4	91.3	94.2	96.6	75.1	84.0	80.2	88.2	81.0	86.7	90.8	95.9	92.3	96.3	91.4	96.0
Central African Republic	25 to 29	66.6	67.6	75.4	76.5	69.6	69.9	54.5	53.9	60.8	60.7	62.8	61.9	74.9	74.6	84.3	84.6	79.3	78.7	83.8	83.9	86.3	87.4	86.5	84.8
Chad	25 to 29	77.5	75.2	83.6	82.1	79.6	77.5	66.8	65.8	70.2	69.3	73.8	72.9	83.6	79.0	92.0	89.2	84.1	80.3	84.1	79.1	91.7	89.5	79.7	73.5
Chile	25 to 29	73.9	86.5	80.7	89.8	75.9	87.8	79.1	89.8	82.5	89.5	79.8	90.4	60.7	76.6	73.0	82.8	65.5	81.0	76.2	89.7	83.2	92.6	76.0	89.1

China	25 to 29	71.3	76.7	82.3	90.9	68.8	71.7	67.3	67.1	76.9	83.6	65.9	62.9	62.9	84.2	74.8	92.4	68.7	84.6	77.3	77.0	85.0	88.4	74.6	70.5
Colombia	25 to 29	65.5	80.6	74.0	86.4	67.2	80.3	67.5	86.4	72.8	86.3	70.1	87.8	57.4	72.0	69.6	82.4	63.3	74.0	63.8	85.1	77.2	89.8	57.4	83.4
Comoros	25 to 29	61.7	74.0	68.2	79.6	68.7	77.7	50.0	60.0	55.4	65.2	61.1	67.5	74.2	81.0	80.5	87.2	80.9	85.2	79.2	85.3	81.1	87.5	84.9	87.4
Congo	25 to 29	68.1	74.6	77.2	78.1	70.7	80.2	56.5	58.6	62.9	60.7	64.1	70.1	77.6	80.3	85.8	84.2	82.0	85.8	82.8	82.5	87.3	85.3	82.6	85.4
Cook Islands	25 to 29	54.1	71.0	68.2	83.9	55.1	67.0	47.7	60.3	59.8	73.7	51.8	57.5	53.4	76.0	73.6	90.3	58.7	75.5	75.7	83.6	77.1	86.3	84.7	85.7
Costa Rica	25 to 29	83.6	88.1	87.8	90.6	84.0	89.2	86.5	93.8	85.9	90.3	88.8	96.9	77.7	80.5	85.7	86.8	78.6	82.1	84.7	91.4	89.2	93.3	83.3	91.2
Coted'Ivoire	25 to 29	77.5	78.2	87.1	85.3	78.5	80.0	67.5	69.1	73.1	71.4	72.6	76.1	81.5	81.5	92.3	90.8	82.2	82.8	79.6	75.3	92.3	90.3	74.4	67.9
Croatia	25 to 29	74.8	89.0	84.5	91.4	72.5	90.4	75.5	94.4	84.3	92.0	70.5	95.6	68.6	89.1	80.4	92.1	70.5	91.0	74.2	88.3	83.6	91.3	70.1	87.3
Cuba	25 to 29	72.7	85.4	81.0	91.1	72.1	83.3	68.4	84.3	75.9	88.2	68.1	80.6	74.5	85.3	82.5	90.8	77.3	85.9	71.4	85.8	81.8	92.4	66.6	80.4
Cyprus	25 to 29	81.8	91.3	85.5	94.1	84.7	91.9	86.9	92.9	86.6	92.2	89.8	94.1	70.9	85.8	76.8	89.9	78.8	88.9	86.5	95.9	88.0	96.8	89.1	95.4
Czechia	25 to 29	79.3	87.8	86.0	90.0	79.1	90.0	81.3	91.6	85.5	89.1	79.9	94.6	74.9	85.7	83.7	88.0	77.5	89.9	74.9	87.4	84.0	90.7	70.8	86.2
Democratic People's Republic of Korea	25 to 29	66.2	57.8	79.9	76.9	62.9	54.3	60.2	48.2	73.7	69.5	57.1	43.8	68.6	69.2	79.0	80.6	72.6	73.7	72.3	65.4	81.1	79.0	70.4	60.8
Democratic Republic of the Congo	25 to 29	74.6	75.9	78.4	82.2	79.9	78.5	63.9	64.1	65.3	68.6	73.6	71.0	82.2	82.6	86.1	88.9	87.1	86.3	85.4	87.2	86.5	89.6	89.4	88.2
Denmark	25 to 29	81.1	91.5	83.9	92.1	85.1	94.3	82.0	94.7	85.3	91.8	83.3	97.4	67.9	85.8	69.7	86.9	80.6	91.2	82.1	95.8	86.2	95.7	83.7	96.4
Djibouti	25 to 29	70.8	78.0	82.7	88.1	71.4	77.8	59.5	65.9	69.4	76.0	64.1	68.8	78.0	80.3	90.8	92.7	80.7	82.5	88.0	87.7	91.0	92.7	87.8	85.4
Dominica	25 to 29	79.0	80.7	83.9	85.1	80.8	82.0	74.3	77.3	77.5	80.0	77.5	79.6	80.1	80.3	86.2	85.7	82.9	83.6	84.3	82.1	88.1	86.9	84.2	81.6
Dominican Republic	25 to 29	53.3	60.6	68.1	74.8	50.5	56.4	44.9	50.9	61.6	68.3	40.7	43.6	54.8	64.5	67.7	73.3	60.4	71.0	62.6	61.9	79.3	81.3	50.7	46.3
Ecuador	25 to 29	48.6	71.5	65.5	81.3	47.5	71.5	40.1	73.1	57.4	79.7	40.3	73.6	58.2	63.6	73.7	76.7	62.4	68.5	48.5	83.2	68.4	90.1	40.0	79.5
Egypt	25 to 29	46.1	71.3	64.3	81.8	40.8	69.6	16.4	54.8	47.8	71.9	5.1	50.2	71.4	87.3	86.3	94.0	68.8	86.1	22.8	42.2	38.3	62.6	35.4	37.3
El Salvador	25 to 29	52.4	74.5	72.5	83.4	44.5	71.5	56.4	72.7	72.1	81.0	50.1	68.3	52.1	70.9	73.2	80.6	52.0	73.0	53.9	78.4	76.4	87.4	36.7	71.9
Equatorial Guinea	25 to 29	66.7	84.0	74.2	87.4	70.4	86.8	54.0	72.8	59.4	74.1	63.7	79.5	75.7	87.0	84.5	90.6	79.7	90.5	83.7	87.2	86.8	90.2	85.2	87.5
Eritrea	25 to 29	52.6	63.0	70.4	79.1	53.3	62.5	42.7	49.6	56.8	64.5	48.6	53.4	67.2	72.1	85.8	88.6	70.1	75.1	82.5	83.7	86.0	89.0	83.9	82.2
Estonia	25 to 29	75.6	85.6	84.0	89.9	76.2	86.2	70.7	83.7	79.0	85.7	70.4	83.5	76.7	88.5	87.5	94.0	77.5	89.0	72.6	87.9	80.6	91.3	72.0	86.2
Eswatini	25 to 29	72.9	67.6	80.2	81.6	72.4	62.9	49.3	44.2	58.1	60.1	56.8	47.3	90.4	85.5	93.0	92.3	91.9	86.4	88.1	82.5	90.8	89.2	87.4	77.5
Ethiopia	25 to 29	59.9	74.7	65.2	81.8	69.0	76.7	51.8	61.5	56.1	67.2	64.4	68.1	76.8	83.6	82.2	90.3	82.9	86.4	85.0	89.1	85.0	90.2	91.7	91.0
Fiji	25 to 29	47.0	55.2	62.7	67.3	48.1	57.8	35.3	39.9	52.6	53.9	37.4	44.8	50.1	55.8	70.3	72.8	53.8	60.3	75.8	77.9	78.4	79.7	82.0	84.1
Finland	25 to 29	76.4	88.6	82.7	89.9	79.5	91.7	79.8	89.8	83.3	88.9	81.8	92.7	65.0	82.0	69.8	83.2	76.1	88.8	74.7	93.9	80.2	94.3	76.9	94.6
France	25 to 29	77.3	87.8	83.9	92.6	78.2	87.7	73.7	91.5	80.4	93.4	73.7	90.9	71.0	82.0	77.5	90.9	78.4	83.0	80.5	93.2	86.7	95.2	79.5	92.4
Gabon	25 to 29	77.2	84.7	84.3	90.1	78.9	84.5	66.7	74.3	71.5	79.1	72.7	77.6	85.1	87.8	91.1	93.3	87.6	89.1	88.5	89.7	90.9	92.8	89.1	88.3
Gambia	25 to 29	75.0	72.3	85.1	82.2	74.6	73.4	62.3	58.7	70.4	66.7	66.4	64.2	81.0	75.6	92.1	88.8	80.5	76.1	78.1	72.0	91.4	87.9	70.1	64.3
Georgia	25 to 29	67.3	79.2	78.5	88.3	65.6	76.5	63.4	77.3	73.4	85.2	63.6	76.0	71.0	75.0	81.5	88.2	74.5	76.6	74.0	78.6	81.3	87.4	72.1	72.2
Germany	25 to 29	73.2	92.5	80.3	93.7	75.7	94.4	73.7	92.5	79.0	91.1	76.2	94.5	51.2	85.7	63.6	87.9	62.4	90.2	70.3	95.2	80.6	95.9	68.1	95.1
Ghana	25 to 29	62.9	75.5	71.2	84.0	67.7	76.7	50.0	64.7	57.7	71.6	58.7	69.0	68.4	75.7	82.6	89.6	70.7	76.2	69.4	74.8	83.2	88.8	63.0	68.0
Greece	25 to 29	64.4	84.0	72.7	89.6	68.7	84.0	57.8	79.4	66.5	83.9	62.3	80.0	70.9	81.9	76.0	88.7	79.4	84.2	62.4	90.6	70.6	93.3	66.5	89.9
Greenland	25 to 29	61.5	85.0	58.4	81.9	78.6	94.0	59.2	81.1	64.2	79.1	67.7	88.5	0.0	61.7	10.5	62.1	35.3	79.6	92.6	98.3	90.3	96.8	97.5	100.0
Grenada	25 to 29	34.2	81.0	56.3	88.5	29.3	78.8	24.6	77.4	51.7	84.5	13.7	74.3	41.1	78.1	59.0	86.2	49.1	80.1	12.5	83.1	44.8	90.9	0.0	77.7
Guam	25 to 29	73.4	75.3	84.0	91.2	72.3	67.9	65.2	64.7	73.3	79.8	67.2	59.3	67.5	70.1	85.2	93.8	70.1	68.8	84.6	88.2	87.3	93.2	86.9	84.8
Guatemala	25 to 29	51.7	68.7	64.5	77.2	53.6	69.8	49.2	64.3	61.1	71.7	51.5	66.2	75.9	73.9	84.5	81.6	77.3	78.0	57.0	79.1	67.0	85.1	60.6	77.9
Guinea	25 to 29	73.5	71.9	76.0	76.4	80.5	77.4	63.8	62.9	64.0	64.0	76.5	74.0	83.7	79.1	88.7	86.2	86.3	82.3	80.5	73.8	87.2	85.4	78.3	68.5
Guinea-Bissau	25 to 29	57.2	60.1	62.7	69.9	66.8	64.9	44.7	45.7	49.3	54.5	58.7	54.7	63.8	61.3	75.5	77.9	70.3	65.7	70.1	64.4	80.1	81.8	68.7	57.7
Guyana	25 to 29	43.1	49.7	63.8	67.8	39.0	44.9	35.5	42.2	55.1	60.0	33.6	39.1	54.8	52.0	73.4	70.7	57.7	54.9	45.6	47.4	72.4	71.0	26.8	31.5
Haiti	25 to 29	0.0	25.3	0.0	27.0	49.3	56.0	1.9	21.8	12.1	29.6	43.5	50.0	1.4	20.7	0.0	18.5	54.6	61.7	0.0	30.0	0.0	33.7	61.5	61.8
Honduras	25 to 29	17.2	61.4	28.8	65.3	34.2	71.6	14.4	60.8	29.3	63.4	31.2	72.5	7.9	44.5	24.0	49.9	33.5	64.6	17.7	69.9	32.0	75.0	35.8	76.0
Hungary	25 to 29	72.3	87.4	82.9	91.3	69.5	87.0	70.4	89.7	81.0	90.4	65.9	88.2	69.8	87.5	80.2	91.6	72.7	89.2	67.6	85.9	81.9	91.2	57.9	81.8
Iceland	25 to 29	77.7	89.6	83.8	93.1	79.7	90.1	77.7	90.9	82.6	91.0	78.8	91.5	67.0	81.4	75.3	89.3	74.5	83.3	79.8	96.1	87.3	97.2	77.6	95.3
India	25 to 29	67.5	77.3	72.4	82.9	74.2	79.5	60.2	69.7	65.4	74.6	67.9	73.4	67.8	76.8	75.4	83.4	75.7	81.8	78.0	84.9	79.4	87.7	84.4	86.1
Indonesia	25 to 29	53.0	60.6	61.6	72.8	59.9	61.1	46.1	50.7	55.6	63.4	54.3	52.9	58.3	66.0	66.7	76.1	68.7	72.5	62.1	64.4	71.1	77.3	64.5	59.5
Iran (Islamic Republic of)	25 to 29	81.5	85.4	84.3	91.2	85.1	84.5	69.6	73.5	74.0	82.2	74.0	72.3	89.8	94.6	91.8	96.9	92.1	94.3	55.6	64.8	63.9	78.6	62.9	60.5

Iraq	25 to 29	58.1	76.5	70.8	84.9	59.0	76.6	45.8	65.2	61.6	74.5	47.5	68.0	79.0	89.9	84.4	92.6	82.9	91.6	41.2	65.2	53.8	74.3	48.5	66.6
Ireland	25 to 29	74.8	92.4	77.5	92.7	81.5	95.2	81.4	95.3	82.0	92.1	85.5	98.0	58.2	85.8	60.9	87.6	74.8	90.7	76.6	97.3	80.8	96.6	79.8	98.3
Israel	25 to 29	77.0	92.8	83.3	93.3	79.2	95.3	70.7	96.1	79.0	93.7	71.0	97.8	74.3	91.2	82.0	92.5	79.1	94.1	82.0	96.3	85.2	96.1	83.7	96.6
Italy	25 to 29	78.9	92.9	85.0	94.8	80.1	93.7	75.1	88.4	80.5	89.4	76.6	89.7	68.2	86.1	74.6	87.8	76.6	90.7	80.6	97.1	86.7	98.3	79.5	96.0
Jamaica	25 to 29	64.3	77.1	76.2	80.0	61.1	82.2	64.5	74.4	72.2	76.8	65.7	78.3	59.3	79.1	75.5	79.3	59.3	89.1	64.3	75.9	78.3	81.3	55.9	77.5
Japan	25 to 29	81.9	90.2	89.3	95.0	80.3	88.8	84.4	93.1	88.2	95.3	83.3	90.2	70.4	82.2	83.7	87.8	70.8	85.0	79.5	92.0	85.0	94.8	79.5	90.2
Jordan	25 to 29	71.9	87.1	77.5	91.6	76.0	87.5	48.8	75.8	62.2	83.5	50.3	73.7	94.2	98.5	96.0	99.2	95.1	98.5	54.9	74.7	62.5	83.7	62.7	72.9
Kazakhstan	25 to 29	73.0	82.4	81.0	88.2	73.9	81.6	67.2	77.5	76.1	83.7	66.5	75.1	67.5	82.7	80.6	91.0	70.6	83.3	61.4	79.4	76.4	86.9	55.3	74.7
Kenya	25 to 29	79.8	82.3	85.0	88.0	81.9	82.9	67.2	69.1	71.3	73.7	73.3	74.3	87.1	87.6	92.2	93.3	89.3	89.2	91.4	91.6	92.1	93.1	92.9	91.7
Kiribati	25 to 29	42.0	35.0	62.2	63.0	43.3	32.8	40.8	32.8	54.1	55.5	47.4	33.8	42.5	40.5	78.8	77.2	40.1	44.3	74.7	68.5	80.0	78.6	76.7	66.6
Kuwait	25 to 29	87.1	91.6	88.9	94.4	90.3	91.7	75.9	85.5	80.1	88.0	78.7	85.0	92.2	99.0	92.6	99.5	95.0	98.6	81.1	83.4	82.6	90.7	85.6	79.4
Kyrgyzstan	25 to 29	57.7	73.4	69.5	84.7	59.2	69.3	48.3	70.8	61.2	81.1	50.7	66.4	63.2	70.7	75.4	84.7	68.8	72.2	43.3	59.2	60.8	80.1	40.0	41.8
Lao People's Democratic Republic	25 to 29	33.2	52.1	43.8	63.8	45.2	56.6	27.3	44.7	38.9	55.8	40.9	51.6	41.9	61.9	52.9	72.0	57.3	70.1	39.2	56.6	48.9	69.9	52.4	55.2
Latvia	25 to 29	72.4	84.3	82.9	90.7	70.2	82.4	58.4	80.6	73.8	87.8	52.9	73.6	77.5	86.5	87.2	93.8	79.2	86.4	67.2	85.7	79.9	91.5	60.6	81.1
Lebanon	25 to 29	67.1	87.3	80.1	93.4	64.2	86.5	50.0	76.5	69.8	85.7	42.9	74.1	78.3	92.1	87.7	96.8	77.3	91.0	70.8	88.0	75.3	91.8	76.1	87.0
Lesotho	25 to 29	79.7	60.6	85.2	74.4	78.5	58.2	60.4	38.4	67.6	53.5	64.5	43.1	94.1	83.1	96.3	89.9	94.3	85.0	91.4	79.6	93.1	86.4	90.9	76.1
Liberia	25 to 29	74.1	69.8	78.3	73.8	79.1	76.6	65.3	57.4	66.2	58.6	75.9	70.2	82.1	76.7	88.5	83.7	84.8	81.4	79.4	73.2	87.8	83.4	75.6	70.3
Libya	25 to 29	74.4	73.3	80.5	79.4	77.1	75.7	58.6	56.5	67.5	65.8	62.3	60.1	87.4	86.0	90.6	87.7	89.6	90.4	63.2	49.8	70.2	64.7	68.7	48.7
Lithuania	25 to 29	78.1	88.0	85.9	91.3	77.0	89.0	69.4	86.3	78.4	87.6	66.7	86.9	73.4	90.2	85.9	94.0	74.2	91.4	76.7	89.5	85.4	92.4	72.0	87.9
Luxembourg	25 to 29	71.6	94.5	80.0	93.8	73.8	97.5	69.6	95.3	76.5	92.3	72.7	98.2	63.5	90.5	73.4	89.2	71.4	95.7	67.8	97.1	77.4	96.1	67.6	98.5
Madagascar	25 to 29	37.8	45.5	53.8	61.6	44.4	49.5	33.3	31.9	45.0	45.8	43.7	40.7	50.9	54.2	67.9	71.4	61.9	64.1	67.0	71.6	74.4	79.3	69.7	71.8
Malawi	25 to 29	69.5	68.0	71.8	78.8	77.5	68.8	54.1	49.2	55.7	60.6	67.8	54.7	77.9	73.4	82.0	85.3	84.4	77.5	85.2	82.3	85.4	87.4	90.4	81.5
Malaysia	25 to 29	70.1	80.9	80.5	87.4	70.2	81.4	68.2	75.9	75.9	81.2	70.1	77.8	80.3	88.7	88.3	92.3	81.6	90.6	70.4	81.2	81.0	87.7	66.9	78.9
Maldives	25 to 29	50.8	75.0	58.0	87.5	60.8	76.8	51.7	70.9	56.9	81.4	62.5	74.5	64.3	85.0	72.3	92.8	72.7	87.6	52.3	74.0	60.8	87.4	60.0	70.9
Mali	25 to 29	63.8	71.0	62.3	71.6	79.6	80.6	50.5	57.6	48.0	53.3	73.8	75.6	76.0	78.2	75.6	79.6	86.2	86.0	77.7	77.0	79.7	82.7	83.7	77.9
Malta	25 to 29	73.9	92.5	78.1	93.3	79.1	94.8	71.7	91.1	74.4	89.5	77.8	94.2	75.4	91.3	79.1	92.8	83.1	94.1	66.5	96.9	72.2	96.6	71.8	97.6
Marshall Islands	25 to 29	10.6	14.9	50.5	40.8	0.0	17.5	6.1	10.4	41.7	33.1	0.0	17.1	0.0	16.0	58.8	50.1	0.0	29.6	66.3	60.0	72.6	64.5	71.1	71.8
Mauritania	25 to 29	70.5	82.7	78.2	86.8	73.4	84.8	59.5	73.9	64.7	73.8	67.3	81.9	77.8	87.0	88.6	92.5	79.3	88.2	74.1	81.3	87.3	90.5	66.7	75.5
Mauritius	25 to 29	71.5	68.0	82.5	83.5	69.9	61.9	67.9	64.9	77.4	80.5	66.5	58.2	76.6	68.1	86.8	82.9	77.4	68.6	59.6	59.8	75.9	77.8	53.4	49.4
Mexico	25 to 29	77.2	81.8	80.9	88.8	81.0	78.7	75.7	83.3	77.8	87.6	79.2	80.1	71.9	70.1	77.2	82.8	78.8	69.9	70.9	85.1	77.2	91.0	73.9	80.9
Micronesia (Federated States of)	25 to 29	12.9	23.4	43.5	52.2	10.6	21.0	11.1	21.8	37.1	46.8	13.1	21.8	3.3	21.8	53.4	60.7	9.1	30.7	61.6	62.4	66.0	68.8	72.1	68.9
Monaco	25 to 29	71.1	82.0	76.9	86.2	75.5	84.2	68.8	79.5	76.5	85.3	70.6	79.0	56.0	73.4	60.5	75.6	71.6	83.4	69.3	83.1	75.8	87.4	72.5	83.3
Mongolia	25 to 29	73.8	79.5	78.8	87.6	77.1	77.2	66.7	73.3	71.2	80.6	71.5	72.3	69.0	73.1	75.4	83.9	77.0	76.2	90.1	89.1	92.2	92.4	89.7	87.0
Montenegro	25 to 29	60.6	75.7	76.4	81.3	57.0	77.9	52.5	67.5	69.6	74.5	48.2	67.8	79.6	87.3	86.3	88.7	82.5	91.7	84.7	87.9	89.8	90.6	82.0	87.5
Morocco	25 to 29	62.9	81.8	66.2	82.3	72.9	88.0	45.5	69.8	52.0	70.5	58.1	78.9	80.6	90.6	84.2	91.3	85.5	93.8	46.0	65.0	49.5	67.4	65.0	76.4
Mozambique	25 to 29	73.8	66.0	80.5	84.4	76.1	61.8	59.1	50.8	65.3	69.9	65.9	51.3	81.1	69.2	89.0	89.6	83.8	70.7	87.3	80.7	89.6	90.4	88.4	74.6
Myanmar	25 to 29	22.0	48.9	34.6	71.2	35.8	43.9	13.2	39.7	27.3	61.9	29.7	37.2	34.3	57.7	49.0	76.8	49.9	61.0	37.7	58.1	52.0	77.1	43.8	47.1
Namibia	25 to 29	74.7	78.9	81.5	87.8	75.5	75.9	54.8	60.8	61.8	72.2	62.3	61.9	90.9	91.4	93.7	95.7	92.6	91.8	86.8	87.4	90.5	92.6	85.2	82.7
Nauru	25 to 29	18.4	19.8	39.5	39.3	24.4	26.5	10.7	7.6	30.9	27.9	20.1	18.0	7.6	16.2	42.3	41.0	19.9	33.8	50.5	49.1	55.3	54.1	65.9	65.9
Nepal	25 to 29	62.5	76.2	67.7	83.7	70.6	75.0	54.1	69.3	60.3	76.2	63.7	68.6	65.6	76.2	73.6	85.5	73.6	77.9	73.6	83.8	74.9	88.5	82.9	82.1
Netherlands	25 to 29	80.6	93.1	82.9	93.0	85.4	96.1	81.0	94.2	83.0	91.5	83.9	96.9	62.9	86.7	67.1	87.2	76.0	92.3	86.4	96.7	88.2	96.0	88.6	98.0
New Zealand	25 to 29	80.2	94.8	84.6	96.1	82.9	95.6	83.3	98.9	85.1	95.5	85.2	100.0	56.1	84.1	68.1	90.1	63.4	85.1	83.9	96.0	90.0	97.2	81.7	95.0
Nicaragua	25 to 29	72.6	82.1	75.9	85.7	79.3	84.2	68.5	82.4	70.4	83.1	77.3	84.6	74.1	79.5	78.3	84.1	81.6	84.0	73.0	82.4	77.6	86.9	78.2	82.3
Niger	25 to 29	79.1	80.2	81.5	83.1	84.3	84.5	70.3	70.3	70.0	70.3	80.4	79.4	87.6	88.5	91.4	92.9	89.8	90.1	88.3	87.5	91.3	91.7	87.7	85.8
Nigeria	25 to 29	86.4	90.6	89.5	94.7	88.6	89.5	75.8	81.8	75.6	82.4	83.0	85.8	94.7	95.2	97.5	99.2	94.8	93.6	90.2	88.6	94.8	96.3	87.5	81.2
Niue	25 to 29	49.7	46.8	69.9	53.2	48.1	58.7	34.4	34.8	60.8	41.4	44.6	51.0	42.6	43.4	74.3	54.7	46.6	58.5	77.5	68.6	80.6	66.8	81.8	85.6
North Macedonia	25 to 29	64.6	82.1	69.9	85.9	71.6	84.2	65.3	82.9	70.3	84.1	70.3	84.6	51.3	77.5	55.5	81.7	69.3	83.6	62.8	82.9	69.1	87.0	69.9	82.8
Northern Mariana Islands	25 to 29	55.4	68.0	70.7	83.0	55.7	66.2	46.3	58.6	60.9	70.4	49.0	61.2	55.9	65.5	68.4	84.8	65.3	69.0	79.2	87.3	82.2	89.5	83.5	88.8

Norway	25 to 29	84.0	97.1	87.2	97.4	86.7	98.1	85.5	96.2	86.8	93.7	87.4	97.9	65.4	91.4	69.9	93.0	76.7	93.9	88.0	100.0	92.3	100.0	86.5	99.2
Oman	25 to 29	78.5	92.2	83.9	94.1	82.4	94.3	66.6	80.5	77.7	86.4	67.9	81.7	93.1	100.0	95.1	100.0	94.9	100.0	69.3	82.9	73.9	86.8	74.2	84.6
Pakistan	25 to 29	72.6	67.6	75.7	71.7	79.5	75.2	65.0	60.7	67.6	63.6	73.9	71.2	71.4	63.2	77.0	67.6	79.5	75.5	82.6	75.4	82.8	78.2	88.2	81.1
Palau	25 to 29	37.0	26.9	70.8	72.8	28.9	22.1	30.1	17.7	61.6	64.6	25.3	12.0	37.4	29.0	76.5	75.5	40.3	39.6	67.2	61.3	77.5	77.9	66.2	57.9
Palestine	25 to 29	65.5	78.7	75.8	87.2	65.9	76.3	42.5	60.5	61.2	76.5	39.4	52.7	93.5	96.9	95.6	97.9	94.2	97.2	48.4	66.8	62.2	79.1	50.7	62.4
Panama	25 to 29	71.3	82.6	72.9	84.3	80.5	87.2	70.4	85.1	72.7	84.7	77.2	88.2	69.3	74.8	73.3	78.0	79.1	83.0	61.8	85.4	63.0	86.1	78.1	90.9
Papua New Guinea	25 to 29	36.5	43.4	39.6	50.9	54.8	54.8	0.0	5.3	0.0	11.5	36.1	32.2	48.6	56.0	63.1	71.6	59.2	62.9	76.0	79.3	71.0	76.3	94.1	93.0
Paraguay	25 to 29	52.6	74.6	57.7	81.0	64.5	76.9	54.4	74.3	58.2	79.6	66.4	75.8	45.8	70.0	52.8	75.8	63.8	78.1	69.7	85.6	73.6	89.7	76.6	84.8
Peru	25 to 29	46.9	63.6	59.2	76.8	52.2	62.7	47.7	59.3	58.5	72.6	52.9	58.2	47.2	61.8	60.1	74.0	59.1	68.5	65.0	79.4	73.9	87.4	66.6	75.6
Philippines	25 to 29	59.2	59.9	76.1	75.6	52.2	56.8	39.6	51.1	64.7	67.9	26.9	48.6	72.0	67.7	81.5	78.4	75.3	73.0	65.5	65.0	80.1	78.7	56.2	58.1
Poland	25 to 29	67.1	85.3	79.4	91.8	64.5	81.8	65.5	84.7	77.8	91.6	61.6	77.5	59.6	81.8	74.3	89.3	63.8	82.6	69.2	85.1	80.4	91.2	64.3	79.8
Portugal	25 to 29	47.9	89.1	62.4	91.3	52.6	90.7	50.2	90.7	62.4	92.1	54.8	90.2	37.6	81.5	52.3	83.7	52.8	87.7	22.8	90.9	46.5	92.1	24.2	92.3
Puerto Rico	25 to 29	81.0	93.8	87.6	95.9	78.0	92.5	80.2	94.5	84.8	92.7	76.8	93.6	78.3	91.8	85.7	95.6	78.3	90.2	80.0	96.2	89.8	98.7	70.8	91.9
Qatar	25 to 29	82.7	93.0	87.4	95.7	86.3	94.9	75.7	89.3	80.4	90.2	79.6	92.4	80.5	94.2	94.1	99.0	82.1	94.6	87.8	92.6	88.4	94.7	90.1	92.6
Republic of Korea	25 to 29	70.3	90.6	79.3	94.2	71.1	90.1	75.6	91.4	80.0	91.1	76.9	91.6	34.0	80.0	53.7	87.3	45.7	82.7	68.3	89.8	77.2	92.5	68.5	89.2
Republic of Moldova	25 to 29	69.6	80.4	80.7	88.2	67.4	78.6	53.1	70.0	69.0	82.3	48.8	62.6	82.3	85.9	90.0	92.4	83.4	86.8	77.4	85.4	84.9	90.2	74.1	82.8
Romania	25 to 29	69.2	79.2	78.1	86.6	69.9	77.7	61.6	78.6	71.9	85.5	61.3	74.4	68.2	73.9	75.8	82.4	75.2	77.7	66.6	79.4	77.3	86.9	63.9	75.1
Russian Federation	25 to 29	72.0	72.9	83.3	85.0	68.8	68.4	60.8	59.4	75.3	77.5	56.3	51.6	68.7	70.4	82.8	79.8	71.3	75.8	63.8	73.2	76.8	85.1	58.2	64.0
Rwanda	25 to 29	41.6	74.2	48.8	82.6	54.9	74.9	35.0	60.4	42.3	69.7	50.0	63.4	59.5	82.0	68.7	91.2	70.4	84.0	71.6	88.9	71.4	90.7	84.3	90.0
Saint Kitts and Nevis	25 to 29	32.2	85.0	36.8	90.1	55.3	84.8	22.7	83.4	30.1	86.1	49.4	85.6	46.6	82.5	53.2	89.5	64.9	83.2	18.7	86.3	28.4	90.4	46.4	84.8
Saint Lucia	25 to 29	66.8	79.1	73.9	88.6	69.9	74.4	63.4	74.7	70.5	86.8	66.4	67.2	64.4	79.8	71.5	85.4	72.8	82.9	61.2	77.9	68.2	88.3	68.0	69.9
Saint Vincent and the Grenadines	25 to 29	55.9	70.9	67.4	77.9	56.3	72.8	42.3	63.3	59.2	71.8	37.2	64.2	67.1	75.2	70.5	78.7	79.1	83.0	58.8	69.5	70.7	77.1	56.4	70.6
Samoa	25 to 29	51.4	49.4	69.7	66.7	49.8	47.7	44.1	42.4	60.5	59.2	44.8	41.7	45.4	47.6	74.2	71.3	50.3	53.2	82.7	78.3	83.5	81.3	88.4	82.9
San Marino	25 to 29	83.8	91.7	87.3	93.0	86.0	93.6	79.8	90.4	82.3	89.1	82.0	92.8	81.2	89.7	85.3	91.7	86.1	92.7	83.7	94.0	88.6	95.1	82.8	93.9
Sao Tome and Principe	25 to 29	79.0	76.1	81.1	83.9	84.5	77.9	70.6	67.8	69.5	74.4	81.3	71.1	84.7	75.6	89.1	89.1	87.8	76.7	82.6	75.1	87.9	87.9	81.6	70.1
Saudi Arabia	25 to 29	59.4	70.5	62.1	79.1	70.4	72.7	36.9	53.4	43.9	66.8	50.6	54.8	87.2	90.5	89.3	92.8	91.0	92.7	40.5	44.6	42.2	60.7	59.4	46.8
Senegal	25 to 29	71.1	79.8	78.0	85.9	74.5	81.6	57.5	69.7	62.0	71.6	66.7	76.9	76.6	80.7	86.0	89.5	79.2	82.5	74.3	80.8	86.0	90.5	69.0	75.9
Serbia	25 to 29	68.2	85.3	76.6	88.9	69.9	86.3	67.4	87.8	75.1	88.8	67.9	87.7	55.2	81.1	63.5	84.0	67.1	86.8	65.5	83.4	75.6	88.0	64.1	82.2
Seychelles	25 to 29	63.7	70.0	73.0	79.4	67.0	72.2	59.0	63.6	67.2	72.0	63.7	67.4	72.6	80.8	79.4	86.6	78.5	84.5	56.7	62.6	70.7	76.8	54.5	59.4
Sierra Leone	25 to 29	77.5	72.6	82.7	75.7	80.0	79.2	67.6	62.6	69.1	61.6	76.5	76.5	84.0	78.1	90.3	83.5	85.5	83.3	78.4	71.1	88.9	82.6	71.8	66.8
Singapore	25 to 29	82.5	93.2	86.8	95.3	84.6	92.9	82.4	92.6	84.9	91.3	84.0	92.5	78.7	92.4	83.9	94.8	83.5	93.3	76.5	93.2	78.6	94.4	81.9	93.1
Slovakia	25 to 29	76.0	85.5	82.7	89.1	77.1	86.6	72.1	86.7	79.1	88.0	71.3	86.1	74.8	84.4	81.0	88.2	80.2	87.8	75.4	85.4	82.9	89.5	73.3	83.9
Slovenia	25 to 29	82.3	90.7	89.0	92.4	80.6	92.5	82.2	94.4	87.3	91.0	79.9	97.3	81.5	93.3	88.5	95.5	82.8	94.2	81.7	90.7	88.8	93.0	76.7	89.7
Solomon Islands	25 to 29	62.5	60.7	77.2	75.1	58.3	57.0	55.3	54.6	68.0	67.6	54.1	52.7	64.7	65.9	87.7	85.2	62.9	66.1	87.5	84.2	88.4	86.1	91.7	88.6
Somalia	25 to 29	59.7	68.0	69.6	79.2	64.6	69.2	47.2	52.9	55.5	64.0	56.2	57.8	72.2	76.8	83.6	88.2	77.5	80.8	86.0	88.9	86.6	90.7	90.0	89.7
South Sudan	25 to 29	53.8	73.8	75.9	79.6	72.5	75.5	24.9	47.7	61.6	62.7	64.4	64.1	79.6	88.6	87.0	87.9	83.9	84.4	68.2	82.1	87.9	88.9	89.4	89.1
Spain	25 to 29	68.3	72.8	77.1	92.2	67.5	91.1	56.0	56.4	74.8	91.4	68.1	91.1	79.6	81.0	70.9	86.9	64.4	87.9	86.8	87.2	75.2	94.2	65.8	93.1
Sri Lanka	25 to 29	65.8	89.8	78.6	85.6	65.0	72.6	65.4	90.7	69.8	77.4	55.9	63.1	57.1	83.5	89.7	93.9	86.1	90.4	65.3	92.9	72.4	80.9	40.1	48.8
Sudan	25 to 29	67.1	75.9	48.5	71.6	62.3	73.6	57.4	66.6	34.4	54.6	42.7	56.9	84.3	89.9	78.5	85.6	80.9	85.8	52.1	62.7	35.9	54.9	68.5	58.7
Suriname	25 to 29	45.4	66.8	68.7	75.4	52.2	63.7	24.6	47.1	62.3	68.3	45.0	56.5	73.9	81.6	71.5	75.1	65.6	72.7	37.6	47.6	78.7	82.6	52.2	64.2
Sweden	25 to 29	53.5	64.7	87.1	100.0	89.5	99.1	46.6	56.9	89.5	100.0	89.7	99.4	58.9	66.4	74.8	94.1	83.3	94.3	61.9	71.2	84.3	99.3	83.0	97.9
Switzerland	25 to 29	85.6	99.0	81.0	94.3	77.7	95.5	88.3	100.0	81.0	93.1	76.5	97.8	73.2	92.4	68.7	90.3	72.5	91.5	80.6	98.8	82.4	97.1	72.3	97.8
Syrian Arab Republic	25 to 29	74.8	93.6	65.2	80.0	54.4	69.9	75.2	95.7	56.7	68.6	40.9	55.0	61.6	88.1	93.5	99.7	87.7	96.8	73.7	97.4	48.8	69.1	42.6	58.1
Taiwan (Province of China)	25 to 29	50.8	71.5	88.1	95.0	79.4	89.8	38.8	57.2	82.6	90.5	75.2	86.3	87.7	98.9	92.3	98.0	92.6	95.5	34.8	57.8	85.8	92.6	75.1	84.7
Tajikistan	25 to 29	80.6	91.1	65.3	79.3	66.4	72.1	76.0	87.9	54.4	70.0	55.2	58.9	90.4	95.8	74.8	85.0	81.8	82.0	77.9	87.8	58.7	76.3	47.3	62.0
Thailand	25 to 29	58.6	71.5	85.6	81.8	71.7	50.6	45.6	59.7	79.8	76.1	72.7	49.5	72.0	78.2	88.1	82.4	72.9	57.2	44.9	65.6	88.3	83.0	75.7	53.6
Timor-Leste	25 to 29	74.3	59.5	68.0	70.6	58.8	57.0	71.9	55.1	58.9	61.5	51.4	50.1	74.2	59.4	77.2	77.0	70.4	70.0	80.5	66.0	78.1	78.2	67.1	57.4
Togo	25 to 29	56.3	57.0	74.6	79.7	77.6	77.7	46.9	47.8	59.1	64.9	70.8	71.4	65.4	65.1	84.9	87.5	82.9	81.8	68.5	64.2	85.5	87.3	75.1	71.9

Tokelau	25 to 29	71.0	73.7	52.1	40.8	34.9	36.1	58.1	62.3	45.6	32.5	32.7	24.5	78.7	79.1	60.9	46.5	40.7	44.8	77.3	76.2	68.0	57.7	80.2	77.0
Tonga	25 to 29	33.8	26.7	82.1	81.9	76.9	70.6	28.5	14.3	70.1	69.6	68.1	62.1	33.2	28.6	87.6	86.6	76.7	72.8	65.5	55.4	89.9	87.8	92.3	88.7
Trinidad and Tobago	25 to 29	75.6	72.1	82.1	83.7	62.8	66.7	63.9	60.3	77.1	81.9	59.7	68.0	75.5	71.6	75.3	71.7	58.4	52.2	89.1	85.9	86.3	85.9	48.7	57.0
Tropical Latin America	25 to 29	68.4	72.0	56.1	76.1	45.9	68.7	65.5	73.1	56.8	74.5	41.8	63.7	57.7	51.8	49.9	67.9	50.5	67.3	65.9	70.0	69.6	83.5	53.4	75.3
Tunisia	25 to 29	74.5	82.2	79.2	88.1	78.6	82.1	61.5	72.8	68.1	79.9	66.7	72.4	88.6	93.6	91.8	95.8	90.2	93.9	65.9	70.8	70.9	81.3	72.9	67.3
Turkey	25 to 29	72.6	87.7	81.7	92.2	72.2	86.9	65.5	81.5	73.7	85.2	66.8	80.9	65.3	86.7	81.9	94.4	65.6	85.0	69.1	84.5	77.9	90.1	69.1	81.9
Turkmenistan	25 to 29	70.5	58.5	75.1	77.2	76.4	52.4	62.8	46.2	67.7	69.8	69.6	39.0	70.2	48.7	77.4	75.1	77.0	52.5	58.2	50.4	65.7	70.8	65.5	39.5
Tuvalu	25 to 29	12.0	26.7	33.4	51.9	17.1	27.1	8.6	22.4	28.2	45.3	19.1	24.5	10.8	24.2	45.6	58.0	20.5	35.8	55.1	61.3	58.7	67.1	74.2	69.0
Uganda	25 to 29	75.3	77.9	84.6	87.0	75.2	77.2	60.3	63.8	69.2	73.0	64.8	67.2	81.4	82.7	91.4	92.2	83.3	84.3	90.8	90.3	92.2	92.3	91.7	90.3
Ukraine	25 to 29	79.2	76.3	88.2	87.4	76.5	73.7	73.2	70.6	82.5	81.7	68.6	66.4	77.9	73.3	87.1	83.8	79.9	77.4	74.4	73.8	87.3	88.1	64.6	63.5
United Arab Emirates	25 to 29	90.9	85.7	89.7	86.9	95.6	90.1	83.2	75.6	81.7	78.4	89.2	79.1	90.0	87.7	91.0	88.0	93.4	92.5	85.0	79.6	85.7	82.2	88.6	84.1
United Kingdom	25 to 29	81.5	91.0	85.3	93.1	84.2	91.8	84.8	90.3	86.4	90.9	86.7	90.8	55.4	80.6	64.0	84.7	67.5	85.5	85.0	97.7	88.2	97.6	86.1	97.8
United Republic of Tanzania	25 to 29	70.0	79.5	79.2	86.9	71.5	79.5	56.9	66.4	64.6	74.1	63.1	69.2	78.4	82.4	88.2	91.2	81.2	84.3	88.2	87.9	90.0	91.9	89.7	86.2
United States Virgin Islands	25 to 29	69.2	76.1	79.1	93.6	66.4	53.3	66.9	71.9	76.6	90.3	62.9	38.4	65.1	76.0	75.1	94.1	69.5	61.0	74.9	77.5	84.8	96.3	67.9	47.8
United States of America	25 to 29	95.2	100.0	94.2	99.9	98.0	100.0	89.9	93.5	88.8	92.7	92.0	93.9	69.7	85.9	75.2	90.9	77.3	85.9	97.6	98.8	97.3	98.8	98.4	98.9
Uruguay	25 to 29	64.7	79.4	72.8	85.7	68.5	79.7	75.3	86.1	78.6	87.8	78.2	85.7	39.0	65.0	55.6	77.0	50.3	68.9	74.2	86.0	79.6	89.9	76.4	85.5
Uzbekistan	25 to 29	58.6	73.3	67.7	82.6	62.9	72.1	38.5	65.1	53.4	75.3	41.4	64.0	78.5	80.4	84.3	89.4	83.0	81.4	52.4	63.9	64.3	78.2	54.5	55.9
Vanuatu	25 to 29	30.7	36.4	62.5	64.5	17.8	28.0	19.6	27.8	51.6	55.0	8.4	21.7	15.2	25.9	64.3	66.5	15.7	30.9	71.3	72.5	78.8	79.8	71.9	72.3
Venezuela (Bolivarian Republic of)	25 to 29	66.0	69.3	74.2	76.7	67.9	68.8	65.9	71.9	72.7	76.1	67.0	72.0	61.8	61.3	71.5	73.1	68.6	61.5	70.2	73.4	77.1	80.9	72.5	71.6
Viet Nam	25 to 29	65.8	76.5	80.7	88.2	61.3	72.9	58.4	71.2	73.3	83.2	55.1	67.4	75.1	84.2	88.1	93.6	74.2	83.1	72.2	76.6	84.5	88.1	64.4	68.9
Yemen	25 to 29	61.2	67.6	68.9	73.9	66.4	72.3	41.6	50.1	54.2	59.0	47.2	57.3	85.0	85.4	91.0	90.2	85.2	87.0	60.6	52.7	63.0	61.0	74.6	60.8
Zambia	25 to 29	59.7	69.5	63.3	78.4	70.9	71.5	46.6	55.5	48.5	62.5	63.3	62.8	74.2	77.7	78.4	86.7	82.0	81.7	83.2	84.7	83.3	87.7	90.2	85.9
Zimbabwe	25 to 29	83.6	60.0	88.9	74.2	82.4	57.3	67.5	25.9	72.6	43.5	72.1	32.5	85.4	61.4	93.1	76.7	86.8	69.1	91.2	75.5	93.6	84.0	89.5	70.5
Afghanistan	30 to 34	43.2	47.1	46.0	49.6	56.5	57.3	36.3	32.1	47.5	40.6	40.8	41.3	71.9	70.3	71.8	71.2	74.1	71.3	37.1	32.1	36.5	33.6	71.8	51.4
Albania	30 to 34	71.7	81.3	75.9	85.3	73.7	81.7	63.7	73.7	69.7	79.4	66.0	74.3	78.2	86.3	80.4	88.1	77.7	86.1	87.6	89.8	91.0	93.2	88.0	89.4
Algeria	30 to 34	63.8	77.6	62.7	78.2	73.6	82.4	53.3	66.0	56.3	68.7	62.9	72.0	79.4	88.5	77.6	87.5	83.1	90.7	53.2	66.5	51.4	69.1	70.1	74.5
American Samoa	30 to 34	53.1	54.4	72.1	67.5	47.1	52.9	42.5	42.4	64.5	60.1	35.9	39.2	75.3	73.4	80.0	72.3	74.5	76.3	76.3	77.2	88.5	86.1	68.3	73.3
Andorra	30 to 34	79.8	89.9	78.3	89.4	85.9	92.5	82.2	92.6	81.6	91.3	86.5	94.5	69.7	85.0	61.4	82.7	79.3	88.7	85.9	93.9	88.1	95.6	87.9	94.1
Angola	30 to 34	61.3	68.5	65.7	74.6	66.3	69.3	49.8	53.2	55.7	61.2	56.6	56.9	75.8	78.1	76.7	81.4	77.0	77.2	81.0	81.6	83.9	86.7	85.1	82.0
Antigua and Barbuda	30 to 34	53.0	85.3	67.6	87.5	44.8	85.4	41.8	82.0	61.0	84.2	32.2	82.6	62.1	87.6	74.4	89.9	47.7	86.1	65.6	90.5	76.3	93.0	61.7	90.5
Argentina	30 to 34	55.2	78.7	62.4	82.1	58.7	80.1	64.2	80.8	71.7	84.8	64.8	80.2	47.0	76.7	51.6	78.1	44.4	76.8	74.7	89.3	78.5	92.1	79.0	89.9
Armenia	30 to 34	75.6	85.2	82.1	89.3	74.4	83.4	65.0	83.2	77.8	87.4	57.7	78.8	83.6	88.0	90.7	95.1	78.7	82.9	72.8	82.7	81.8	90.1	71.1	78.6
Australia	30 to 34	77.9	88.9	81.0	88.6	80.1	91.9	80.3	91.2	82.9	90.6	81.3	92.5	64.1	83.6	70.9	85.9	58.2	82.2	84.0	93.4	88.4	94.6	84.6	94.5
Austria	30 to 34	78.2	91.8	80.1	92.1	81.4	93.2	75.2	93.7	79.8	92.3	76.2	95.2	72.0	89.0	70.3	89.7	75.8	89.6	78.7	95.6	84.0	97.2	79.9	95.3
Azerbaijan	30 to 34	65.0	79.5	73.3	85.9	63.3	76.4	53.8	69.1	65.8	79.6	51.1	64.1	86.9	91.9	90.6	95.3	84.9	90.1	70.8	83.6	80.3	90.4	67.6	79.7
Bahamas	30 to 34	56.4	66.6	64.2	77.0	57.4	62.0	47.7	57.7	58.2	71.0	48.9	52.9	65.1	72.3	69.4	78.1	61.1	66.9	66.5	73.7	73.9	83.5	67.9	68.2
Bahrain	30 to 34	85.5	85.3	89.9	92.3	86.1	85.1	76.1	75.9	82.7	86.5	76.4	75.0	89.1	92.0	91.7	95.5	88.5	90.9	84.6	79.7	87.1	88.2	86.6	79.4
Bangladesh	30 to 34	8.4	35.7	49.9	70.8	0.0	23.0	10.6	30.3	49.6	66.6	0.0	15.3	18.1	44.6	56.2	71.4	3.9	34.5	45.8	57.5	67.5	81.5	39.8	42.5
Barbados	30 to 34	66.0	77.2	75.5	85.1	62.7	72.0	61.5	73.7	72.5	83.7	57.6	66.0	68.3	75.9	75.5	82.7	61.5	69.5	66.9	80.2	78.3	89.0	62.1	74.0
Belarus	30 to 34	68.9	74.4	81.7	85.5	62.9	68.2	56.2	61.5	73.6	78.4	48.7	51.9	76.2	75.3	83.1	84.3	72.4	69.8	69.5	79.9	83.7	89.2	59.6	73.4
Belgium	30 to 34	69.0	91.0	73.5	90.2	71.9	93.8	63.6	94.1	70.6	91.5	65.3	97.0	64.8	84.9	64.2	84.2	67.7	87.0	72.1	94.6	81.0	95.4	70.9	96.0
Belize	30 to 34	63.9	74.4	76.7	83.9	57.5	68.6	54.2	65.7	69.6	78.5	48.7	59.0	72.3	82.3	82.2	87.6	61.3	76.3	77.3	82.4	89.2	91.6	68.3	74.8
Benin	30 to 34	74.6	76.1	77.1	79.2	78.1	78.7	63.6	64.5	66.4	67.8	70.1	70.1	85.5	85.0	88.7	88.0	83.7	83.7	84.5	82.0	91.0	90.2	81.8	78.4
Bermuda	30 to 34	80.9	92.3	86.2	93.6	77.9	91.2	79.9	93.1	84.2	91.6	77.0	93.8	84.4	95.8	90.2	99.1	77.6	91.9	78.0	92.3	87.9	97.2	71.0	87.5
Bhutan	30 to 34	59.7	73.2	66.9	80.6	62.5	72.5	51.1	64.6	62.4	75.0	51.1	61.9	65.5	75.5	70.4	81.0	64.3	73.2	74.8	81.4	78.2	87.8	79.0	80.0
Bolivia (Plurinational State of)	30 to 34	32.3	61.9	37.1	65.9	43.9	66.8	30.6	56.8	41.8	64.9	37.9	59.2	39.9	59.6	37.1	57.9	46.5	63.7	49.3	78.1	53.9	82.3	63.1	80.6
Bosnia and Herzegovina	30 to 34	74.1	87.3	82.1	87.6	72.9	89.9	66.2	84.3	79.4	87.4	60.5	83.0	60.3	83.1	72.5	84.9	51.0	82.9	74.6	85.7	85.6	89.0	70.9	87.1
Botswana	30 to 34	61.8	78.1	73.5	83.8	58.2	77.0	43.6	64.9	54.3	71.9	47.3	66.4	82.7	91.0	88.0	93.8	79.5	89.8	79.8	86.4	87.0	91.9	75.9	83.5

Brazil	30 to 34	28.2	61.0	43.2	68.3	28.6	62.0	28.3	56.1	49.6	68.8	21.5	51.8	30.7	57.3	34.6	59.3	27.5	56.7	47.1	74.0	66.1	80.6	40.7	74.7
Brunei Darussalam	30 to 34	56.9	69.4	65.1	82.0	60.2	65.4	64.7	71.7	73.3	84.9	65.0	63.9	34.8	53.5	38.3	67.2	36.2	45.5	62.9	80.4	67.0	86.2	69.2	79.8
Bulgaria	30 to 34	53.3	66.1	67.5	78.2	50.2	61.6	49.4	57.0	65.1	74.9	45.3	48.3	62.4	66.4	69.8	73.5	57.3	61.4	51.7	64.0	69.0	77.0	45.6	59.6
Burkina Faso	30 to 34	70.4	73.1	72.3	78.3	75.7	74.0	56.4	60.2	60.6	67.2	64.6	63.2	85.5	85.9	89.1	91.8	83.1	81.7	84.8	83.4	89.8	91.2	83.9	79.2
Burundi	30 to 34	38.1	54.4	50.5	64.3	40.5	56.0	32.0	37.6	47.5	51.0	33.6	40.6	56.2	71.5	62.8	77.7	54.6	69.9	66.5	78.3	69.3	81.8	76.7	82.2
Cabo Verde	30 to 34	61.1	84.1	67.7	86.7	63.3	85.9	53.2	77.1	60.4	78.7	58.0	81.3	73.2	89.8	79.7	94.2	68.2	87.3	63.6	81.1	80.9	92.2	53.5	76.7
Cambodia	30 to 34	38.4	60.7	42.6	68.2	49.4	62.3	29.7	53.6	37.8	62.7	42.7	55.8	63.7	77.1	65.4	80.4	63.8	75.7	62.8	75.6	67.6	80.9	71.8	77.6
Cameroon	30 to 34	63.2	66.8	67.1	72.3	68.3	69.6	51.3	55.4	57.6	62.0	57.7	60.2	78.3	77.2	83.8	84.4	75.1	73.2	80.6	75.8	87.7	87.1	79.2	71.4
Canada	30 to 34	84.5	89.1	85.2	89.7	87.4	91.0	88.3	90.3	88.2	89.8	89.9	91.8	73.1	82.5	76.7	85.9	69.6	79.4	89.5	92.4	92.8	94.3	89.7	92.8
Central African Republic	30 to 34	52.7	54.4	63.2	66.8	53.8	52.6	41.5	39.5	52.8	53.9	45.3	41.0	69.3	69.5	74.9	76.9	67.6	66.3	77.0	78.4	81.8	84.8	79.7	77.7
Chad	30 to 34	69.1	68.2	72.0	73.5	73.6	70.7	57.3	57.2	61.6	63.4	64.3	62.0	79.7	77.0	84.2	83.6	77.3	73.1	80.7	76.5	88.0	87.5	78.7	71.6
Chile	30 to 34	71.3	84.6	75.5	86.7	73.5	85.7	76.5	89.1	81.6	90.8	74.8	87.6	62.2	76.8	65.1	78.3	60.2	76.6	76.7	88.9	82.3	92.5	78.5	88.8
China	30 to 34	60.6	68.9	74.1	86.9	55.7	61.3	58.3	59.5	72.2	80.0	53.1	52.3	58.5	82.3	66.9	91.1	53.9	76.5	70.0	71.2	81.5	87.4	64.4	61.3
Colombia	30 to 34	63.8	78.8	69.2	83.3	65.4	77.7	62.5	83.0	69.1	85.9	63.4	81.5	63.6	73.0	67.6	78.5	60.0	68.3	66.0	86.0	77.6	91.4	61.6	83.8
Comoros	30 to 34	51.8	64.2	58.9	71.2	56.0	65.7	40.3	46.9	49.8	58.1	46.1	49.1	71.1	77.1	72.8	81.0	72.0	76.4	74.7	81.1	78.9	86.0	80.0	82.4
Congo	30 to 34	56.3	65.4	66.6	69.3	56.9	69.4	45.2	46.6	56.2	53.1	48.5	54.1	73.2	76.9	77.9	77.4	72.0	78.2	77.1	78.3	84.3	83.3	76.3	80.4
Cook Islands	30 to 34	57.7	72.5	66.7	82.6	58.2	68.9	49.3	61.5	62.1	74.9	48.0	56.7	73.0	87.1	72.6	90.5	75.5	86.1	77.1	83.8	84.4	90.4	75.0	80.2
Costa Rica	30 to 34	80.2	85.8	83.1	89.0	80.8	84.2	80.3	89.8	83.0	90.7	80.2	88.4	78.8	80.7	81.6	87.0	76.6	73.9	82.7	89.6	87.8	93.3	82.2	88.5
Coted'Ivoire	30 to 34	71.0	72.0	77.4	77.9	73.3	73.5	59.4	59.6	66.5	65.7	63.1	64.0	79.1	79.5	87.2	87.2	75.3	75.1	77.0	72.3	89.0	88.6	73.9	64.9
Croatia	30 to 34	68.7	89.9	79.8	92.9	64.1	88.3	66.4	91.8	80.3	94.4	58.9	88.2	66.1	89.3	74.3	93.0	58.6	86.7	71.0	89.5	83.1	93.9	65.0	87.2
Cuba	30 to 34	61.9	83.5	69.3	89.0	62.0	80.4	55.1	79.0	66.0	86.4	53.4	73.7	71.0	90.2	73.1	93.2	69.9	87.5	63.3	82.7	74.6	92.1	60.4	75.3
Cyprus	30 to 34	79.4	91.7	80.7	92.4	82.9	92.7	83.4	93.3	83.7	91.7	86.5	95.3	73.3	88.3	72.7	88.5	75.7	89.4	85.7	95.9	87.8	97.0	88.2	95.7
Czechia	30 to 34	74.1	87.6	81.1	89.2	72.4	88.2	73.3	89.7	82.3	90.1	68.8	89.4	71.1	85.4	75.9	86.1	67.9	86.4	69.7	87.2	81.5	91.7	64.9	85.8
Democratic People's Republic of Korea	30 to 34	59.1	50.7	72.6	69.8	55.5	46.3	54.8	41.9	69.8	64.5	50.1	35.5	68.1	70.0	74.3	77.3	64.6	66.7	69.3	61.6	79.4	77.3	66.8	56.4
Democratic Republic of the Congo	30 to 34	66.0	67.2	70.2	75.0	70.1	67.7	55.7	54.5	60.4	63.8	62.0	56.8	79.7	79.4	80.5	83.9	80.7	78.5	81.2	83.4	84.3	88.5	85.0	83.2
Denmark	30 to 34	72.3	90.0	75.4	91.0	75.8	91.1	72.7	94.6	79.8	93.8	72.1	95.3	64.3	87.4	60.9	88.5	70.3	87.7	75.5	94.2	82.6	96.7	75.1	93.3
Djibouti	30 to 34	58.4	67.5	71.4	79.4	58.1	65.1	45.9	50.7	59.4	66.0	47.9	50.2	73.6	76.3	83.3	86.7	70.6	72.3	82.5	82.4	87.8	90.7	82.8	79.1
Dominica	30 to 34	73.2	73.2	77.5	79.1	74.6	72.0	66.4	64.9	72.3	73.0	67.6	63.3	80.5	78.7	83.6	83.0	78.4	75.4	79.8	76.0	85.0	83.5	80.3	73.6
Dominican Republic	30 to 34	45.8	55.8	59.1	67.2	41.9	53.0	35.0	44.6	54.3	62.1	27.4	38.0	57.2	67.5	61.9	68.8	52.3	67.5	57.6	60.8	75.3	77.9	46.4	50.8
Ecuador	30 to 34	48.9	71.0	62.2	78.5	46.3	70.0	37.7	68.0	57.2	79.1	32.3	63.6	65.6	68.6	70.8	73.0	61.5	66.1	45.7	83.4	62.4	90.0	42.3	81.3
Egypt	30 to 34	51.6	70.3	63.9	77.1	47.3	70.1	28.8	54.3	52.6	68.4	18.4	50.8	73.3	87.0	81.4	90.6	65.1	84.2	32.3	43.3	43.3	58.1	41.9	44.2
El Salvador	30 to 34	54.0	71.7	69.3	80.2	46.5	65.8	55.8	66.4	70.5	78.4	49.0	57.8	59.9	72.6	71.3	78.5	48.2	65.8	58.2	77.3	78.3	88.1	42.6	68.0
Equatorial Guinea	30 to 34	54.9	76.7	63.2	80.7	56.3	78.5	42.4	59.9	52.1	65.8	47.3	64.4	71.6	85.2	76.9	86.4	69.2	85.8	77.7	83.1	83.5	88.2	78.7	83.1
Eritrea	30 to 34	36.4	47.0	55.8	66.8	34.2	43.5	26.7	31.3	46.2	53.7	27.7	30.0	62.0	66.4	76.0	79.9	55.1	61.1	76.2	76.8	81.8	85.7	78.4	74.5
Estonia	30 to 34	71.1	85.9	81.3	89.1	69.0	85.6	64.5	83.4	76.8	87.3	59.7	80.8	75.3	90.2	85.6	94.2	67.6	88.4	70.3	88.3	83.3	93.4	65.4	86.1
Eswatini	30 to 34	66.3	60.2	74.9	75.9	62.1	52.5	44.0	38.0	56.6	56.8	44.6	36.4	86.4	81.8	89.2	88.0	84.8	77.8	85.0	78.6	89.2	87.2	84.2	73.0
Ethiopia	30 to 34	48.1	64.1	54.9	72.8	53.6	64.1	42.5	47.6	51.8	58.7	47.8	50.1	72.0	79.5	74.7	85.0	71.9	77.5	81.0	85.5	82.8	88.2	88.1	87.8
Fiji	30 to 34	58.9	64.1	66.2	67.3	60.9	69.6	46.4	48.2	58.8	55.7	46.6	54.1	75.3	76.7	74.4	73.6	78.3	81.9	80.2	81.5	87.3	86.4	77.8	81.6
Finland	30 to 34	67.4	87.0	79.0	88.6	66.1	88.6	68.1	89.9	82.6	90.5	63.0	90.8	62.6	82.8	68.4	83.6	61.1	83.7	64.5	93.3	81.4	95.9	58.7	92.5
France	30 to 34	72.8	83.3	80.2	87.7	71.3	83.0	68.1	89.6	78.3	92.6	65.1	87.8	71.8	79.8	73.5	84.3	72.4	77.2	79.7	89.0	88.1	94.3	75.7	86.6
Gabon	30 to 34	66.8	76.3	75.7	83.2	67.3	74.8	56.3	62.6	65.6	72.0	58.9	63.1	81.6	84.7	86.1	89.3	80.3	82.5	83.7	85.4	88.5	91.1	84.0	83.6
Gambia	30 to 34	69.0	66.7	76.4	74.5	69.9	67.6	54.4	51.1	63.2	61.0	57.6	54.0	79.6	75.0	87.8	84.6	74.4	68.9	76.0	70.2	89.2	86.5	69.8	62.6
Georgia	30 to 34	66.7	75.2	80.5	88.2	59.5	68.6	63.4	71.7	77.7	86.4	57.8	66.2	75.7	73.7	85.1	87.9	69.4	66.7	74.3	76.9	83.6	89.9	69.3	65.8
Germany	30 to 34	70.7	89.3	75.1	89.6	73.3	91.4	69.5	90.9	76.4	90.7	69.9	92.1	57.6	84.0	57.4	82.4	60.4	86.9	70.4	92.6	79.5	95.7	69.8	91.7
Ghana	30 to 34	56.6	68.4	59.4	74.1	64.5	70.7	42.1	53.3	48.1	61.5	51.8	57.0	66.7	72.9	71.1	80.9	65.1	68.7	67.1	70.9	79.6	85.8	63.9	64.8
Greece	30 to 34	68.1	82.8	71.2	86.1	72.7	83.4	63.8	78.7	67.9	81.6	68.5	80.2	75.1	83.7	74.8	87.4	77.2	81.4	68.7	89.4	74.2	93.1	72.5	88.4
Greenland	30 to 34	54.4	75.2	55.0	72.3	64.4	83.9	55.9	72.9	66.6	75.0	55.3	77.1	0.0	50.1	3.8	43.8	0.0	61.8	81.6	92.9	84.1	93.5	84.9	94.9
Grenada	30 to 34	28.2	70.9	45.4	77.1	25.4	70.7	17.4	62.9	44.8	72.2	5.5	61.3	47.5	74.5	47.3	77.2	50.7	73.1	8.0	73.3	36.7	81.2	0.0	72.1

Guam	30 to 34	76.2	71.8	81.6	84.1	76.6	67.6	66.2	58.8	74.2	74.4	66.1	53.4	83.4	82.3	83.4	87.1	84.7	80.9	84.3	82.7	90.5	92.5	81.8	76.0
Guatemala	30 to 34	51.0	63.9	57.7	71.3	55.0	63.3	49.6	56.6	58.3	66.5	53.1	55.4	77.2	76.4	80.3	79.4	74.6	74.4	58.2	78.8	65.7	86.0	64.4	76.1
Guinea	30 to 34	64.0	65.9	63.5	69.1	73.9	70.9	53.7	55.7	55.1	60.0	66.4	63.3	78.9	76.9	80.7	81.0	78.9	75.2	76.2	71.7	83.1	84.5	76.2	65.7
Guinea-Bissau	30 to 34	46.9	51.3	48.1	58.6	58.9	56.0	34.5	35.7	39.2	46.4	48.1	41.8	60.0	59.7	62.1	68.0	60.5	55.2	65.5	60.9	75.1	79.2	65.5	53.4
Guyana	30 to 34	34.6	43.1	56.1	62.2	28.0	34.2	27.3	33.9	50.5	57.5	20.6	22.0	59.0	54.6	72.1	66.0	47.6	43.1	37.0	42.9	67.9	69.4	17.6	25.0
Haiti	30 to 34	0.0	22.9	0.0	26.7	30.7	39.1	5.0	19.6	18.6	33.4	21.5	26.9	12.2	30.1	0.0	21.4	35.0	45.3	0.0	28.6	0.0	34.6	44.9	48.9
Honduras	30 to 34	17.3	55.0	26.6	56.4	26.5	65.2	9.3	45.8	27.0	51.7	15.3	57.2	19.6	44.7	22.8	40.6	19.3	53.3	26.0	68.4	39.4	73.2	34.5	74.2
Hungary	30 to 34	59.3	86.1	71.2	90.4	56.2	83.4	53.5	84.8	69.1	91.4	47.9	78.2	57.8	85.5	62.8	87.1	54.0	85.3	54.8	84.6	74.5	92.1	44.1	78.9
Iceland	30 to 34	77.8	89.1	79.0	90.3	82.0	90.4	78.8	91.1	80.8	90.8	81.5	92.4	71.6	83.7	69.8	85.6	75.4	83.4	81.9	95.5	86.7	97.3	82.7	95.0
India	30 to 34	59.1	68.4	68.2	77.5	59.6	66.9	51.9	60.0	64.8	71.8	48.8	56.9	63.2	70.7	71.3	77.2	59.0	67.7	74.7	81.0	80.0	87.2	77.0	79.6
Indonesia	30 to 34	45.9	52.8	55.5	65.6	48.0	50.5	40.4	42.7	53.4	58.7	41.1	40.1	60.5	67.3	65.4	73.3	57.8	64.0	63.2	63.7	72.9	77.3	62.8	57.8
Iran (Islamic Republic of)	30 to 34	79.4	87.1	81.4	91.5	81.7	85.4	67.3	75.3	72.6	82.9	69.7	73.6	88.0	94.7	89.1	97.3	88.0	92.7	53.8	67.3	61.0	78.2	60.7	65.0
Iraq	30 to 34	55.7	75.1	65.7	80.7	55.6	75.4	44.7	64.9	59.4	72.2	44.4	66.7	75.7	86.8	77.1	87.3	75.5	87.4	40.1	63.7	49.8	70.2	48.9	68.2
Ireland	30 to 34	74.2	90.8	76.5	90.8	77.9	92.7	80.2	96.0	82.4	93.6	81.8	97.6	64.9	85.3	64.1	85.7	68.3	86.5	79.1	95.7	85.2	97.1	78.9	95.7
Israel	30 to 34	74.0	90.6	79.1	91.6	75.3	91.8	67.0	94.2	76.0	94.5	65.7	93.9	77.4	91.3	80.6	92.8	75.8	91.1	79.9	94.4	85.7	96.6	80.1	94.1
Italy	30 to 34	75.0	93.1	80.9	95.7	75.0	91.9	70.9	88.0	78.0	91.2	70.3	86.7	70.4	88.1	73.1	90.6	69.6	86.7	78.2	97.8	86.1	99.3	75.9	96.5
Jamaica	30 to 34	59.2	71.2	67.4	75.7	58.5	71.9	54.6	64.6	64.2	73.6	55.0	60.6	63.1	79.2	69.9	77.2	55.6	83.9	60.9	71.2	71.3	79.2	59.6	69.7
Japan	30 to 34	74.1	81.1	84.1	89.7	70.8	78.3	77.9	85.8	86.6	95.4	73.6	79.6	61.1	73.3	74.2	79.5	51.8	70.2	79.3	88.6	85.1	93.7	78.9	86.2
Jordan	30 to 34	71.4	88.1	73.4	91.6	76.1	87.5	49.7	75.9	60.5	83.3	50.9	73.7	92.7	98.2	93.6	99.7	92.8	97.5	54.5	75.8	58.9	83.7	64.7	75.0
Kazakhstan	30 to 34	70.9	76.5	79.1	84.2	69.2	72.6	63.0	67.5	75.3	79.3	57.9	61.3	66.8	77.0	78.6	86.0	59.2	71.7	63.8	75.5	76.5	85.0	60.5	70.6
Kenya	30 to 34	71.3	72.1	76.1	77.7	73.4	73.2	57.7	55.9	64.4	63.0	61.7	60.0	83.5	83.0	86.5	86.4	83.0	82.1	88.2	87.1	89.9	90.0	90.8	88.7
Kiribati	30 to 34	51.4	46.5	66.0	66.9	50.4	43.5	51.6	44.7	64.4	64.6	51.6	41.2	72.0	71.7	80.6	78.7	66.4	69.4	74.7	67.5	87.4	87.0	67.7	56.0
Kuwait	30 to 34	88.8	92.6	89.1	94.7	91.2	91.3	77.5	85.4	81.4	88.9	79.6	83.4	91.4	98.3	90.3	100.0	93.2	97.2	83.9	85.3	86.2	92.0	86.7	81.9
Kyrgyzstan	30 to 34	58.0	64.3	67.9	81.0	57.4	54.5	50.3	58.3	62.8	78.6	49.4	46.6	65.4	65.4	71.0	79.9	62.6	55.6	52.5	49.6	65.8	78.0	51.1	26.0
Lao People's Democratic Republic	30 to 34	21.9	45.1	32.4	57.1	29.8	46.3	18.5	37.7	33.1	52.0	25.1	39.0	44.0	65.4	48.6	70.9	42.8	62.8	38.3	58.1	46.9	71.2	49.2	55.8
Latvia	30 to 34	68.0	80.8	79.7	89.9	63.3	75.2	52.4	68.6	70.8	87.0	43.4	53.1	76.5	85.2	84.5	93.1	71.4	80.4	66.2	83.5	81.9	93.0	56.8	76.5
Lebanon	30 to 34	61.3	88.8	71.8	92.9	60.3	87.8	44.4	78.2	63.9	85.7	36.2	75.7	71.0	91.1	76.5	94.7	65.4	88.2	64.0	88.7	67.1	92.1	71.8	88.3
Lesotho	30 to 34	73.5	50.1	80.4	67.5	68.7	43.1	53.9	30.8	64.9	51.7	52.4	28.2	90.6	76.8	93.8	84.3	88.2	72.3	89.0	74.3	92.3	83.6	88.1	69.4
Liberia	30 to 34	68.5	66.2	71.5	69.9	73.3	70.8	59.3	52.7	63.5	58.1	66.0	59.7	80.6	76.4	85.1	81.4	78.5	74.4	77.1	72.4	86.7	84.5	74.1	68.1
Libya	30 to 34	75.4	74.1	74.1	76.5	82.5	77.5	60.9	57.1	63.1	62.5	69.1	62.8	85.7	85.0	83.6	82.7	89.0	89.2	63.6	51.5	63.1	62.0	75.7	54.6
Lithuania	30 to 34	74.1	83.1	82.9	89.0	70.6	80.5	63.1	76.0	76.2	85.8	56.8	70.0	72.8	83.0	83.1	90.8	65.2	77.8	74.1	85.8	86.0	93.0	67.1	81.2
Luxembourg	30 to 34	67.4	94.1	71.5	93.2	71.4	96.1	65.6	95.3	70.1	93.7	69.4	96.8	64.7	90.6	63.9	89.1	67.8	93.0	64.7	96.9	73.2	97.2	66.5	98.0
Madagascar	30 to 34	29.3	34.8	42.9	49.5	33.9	36.9	26.1	20.3	39.0	36.7	32.3	25.0	51.7	54.2	57.1	59.6	51.8	54.5	63.2	67.1	72.0	76.4	65.5	67.3
Malawi	30 to 34	56.4	53.5	58.1	67.1	65.4	51.4	39.7	26.8	44.3	46.0	51.2	27.7	72.3	66.4	70.8	75.2	75.2	63.2	79.9	75.7	80.9	83.5	86.7	74.5
Malaysia	30 to 34	64.4	73.7	75.4	82.8	62.5	71.9	62.3	66.6	73.3	77.4	60.6	64.9	79.9	87.1	86.4	90.9	74.9	85.0	70.0	78.9	81.3	87.4	66.3	75.9
Maldives	30 to 34	36.5	70.7	46.7	84.4	42.3	71.8	37.5	65.0	48.4	78.5	42.6	66.6	62.3	86.2	68.1	92.9	59.1	84.9	49.6	75.4	60.5	88.4	53.4	73.6
Mali	30 to 34	57.1	65.3	51.4	62.7	76.1	76.8	43.6	49.6	41.6	46.8	66.7	67.5	73.6	77.1	66.2	72.8	81.9	82.2	75.5	75.9	76.8	80.9	84.4	78.4
Malta	30 to 34	76.0	86.6	75.8	85.2	81.8	91.1	74.3	85.6	73.8	83.8	80.7	90.3	80.3	85.0	78.2	82.1	84.2	89.4	72.8	92.2	75.9	92.1	78.3	95.2
Marshall Islands	30 to 34	28.8	29.4	52.1	46.2	24.2	30.5	23.4	23.1	49.9	43.9	17.0	21.9	53.6	57.2	60.6	54.7	50.9	61.8	68.7	65.2	81.9	78.2	62.2	59.8
Mauritania	30 to 34	65.6	77.2	71.5	79.8	68.1	79.9	53.9	65.1	61.6	68.1	58.0	71.4	76.9	84.9	84.0	89.2	72.7	82.5	72.3	78.8	86.4	89.1	65.3	73.5
Mauritius	30 to 34	58.1	53.0	75.2	77.3	52.0	40.2	54.0	45.5	72.9	74.7	45.5	31.2	71.4	61.8	82.4	79.0	62.0	47.1	47.7	53.2	70.2	76.1	38.6	39.4
Mexico	30 to 34	75.6	79.1	78.8	86.9	76.7	73.1	71.6	75.5	76.5	86.2	71.1	67.0	74.8	72.7	77.2	81.4	73.1	63.5	74.1	84.1	81.2	92.2	73.6	77.5
Micronesia (Federated States of)	30 to 34	23.2	33.6	44.7	53.1	21.6	31.6	19.7	28.2	44.5	51.1	15.4	23.9	49.6	60.2	54.8	61.4	49.0	62.2	62.7	64.0	77.3	79.7	56.7	55.7
Monaco	30 to 34	66.6	79.5	71.3	84.0	69.8	79.7	63.5	75.5	72.6	84.6	63.3	72.2	56.3	74.7	53.9	73.9	61.8	77.5	67.1	81.7	74.5	87.9	69.1	80.5
Mongolia	30 to 34	70.5	73.1	74.4	82.9	72.8	68.9	65.4	68.5	71.1	78.3	66.8	64.8	71.1	66.9	72.7	75.4	71.7	62.2	90.4	88.3	93.0	93.6	90.0	84.6
Montenegro	30 to 34	54.9	72.6	68.2	79.7	52.4	70.2	48.2	61.6	63.3	73.1	45.0	55.3	79.8	88.3	82.1	89.5	79.3	88.6	84.0	88.5	90.3	92.8	81.0	86.7
Morocco	30 to 34	59.9	77.6	60.2	74.6	69.5	86.6	45.0	64.4	50.4	63.7	55.0	76.3	76.6	86.4	75.5	83.5	79.9	91.2	42.9	59.6	43.7	57.5	61.3	77.1
Mozambique	30 to 34	61.1	48.4	69.2	74.1	62.0	40.3	44.6	29.6	55.5	59.2	47.6	24.6	75.2	61.8	81.5	82.2	72.7	52.7	82.0	71.5	86.4	87.5	83.4	62.3

Myanmar	30 to 34	15.1	42.3	27.3	65.1	23.0	35.4	9.0	32.8	24.7	56.3	17.1	27.8	41.7	62.5	48.8	75.8	38.6	54.2	40.1	60.6	53.9	78.4	42.8	50.8
Namibia	30 to 34	68.7	71.8	76.2	82.9	67.2	65.8	51.0	52.3	60.6	68.3	53.5	48.9	87.8	88.6	90.4	93.5	86.7	85.6	83.7	83.9	89.1	91.5	81.6	78.2
Nauru	30 to 34	29.5	31.2	40.8	41.1	35.2	37.7	23.8	20.5	39.2	35.1	27.8	26.2	48.9	53.6	40.8	42.0	59.4	65.9	55.8	56.5	70.5	69.6	51.6	53.6
Nepal	30 to 34	52.1	68.0	60.7	77.4	54.2	62.7	44.7	58.9	57.9	72.0	43.1	49.9	60.4	72.3	68.0	80.4	55.8	65.0	68.7	79.9	72.7	87.3	74.7	76.0
Netherlands	30 to 34	76.9	91.2	78.6	90.3	80.6	94.0	78.0	93.9	81.4	91.7	79.2	96.2	62.7	86.5	62.3	84.6	65.5	89.8	84.6	95.1	88.4	96.1	85.5	95.9
New Zealand	30 to 34	75.8	92.7	76.7	94.7	80.6	91.8	78.2	97.4	80.8	96.7	80.2	96.2	55.6	79.9	55.2	86.5	58.6	73.3	81.9	95.3	87.4	98.0	81.6	93.7
Nicaragua	30 to 34	64.6	80.7	69.8	84.3	66.1	80.0	56.9	77.7	65.5	82.4	57.1	75.2	71.0	81.7	73.9	84.8	68.7	79.2	68.5	83.2	77.0	89.3	67.9	80.6
Niger	30 to 34	70.8	73.5	71.4	76.0	77.6	77.5	61.5	61.9	63.7	65.8	69.8	67.7	82.9	84.9	84.6	89.1	82.7	82.9	85.0	85.3	88.7	90.9	86.6	84.3
Nigeria	30 to 34	76.0	84.1	79.1	88.9	79.0	82.2	62.3	71.4	66.6	77.5	67.6	71.9	89.0	91.9	93.5	98.2	86.5	86.9	85.0	84.5	91.5	95.1	83.3	76.1
Niue	30 to 34	54.7	57.7	67.7	60.9	53.8	64.6	47.0	44.9	63.0	52.4	44.4	51.8	70.3	71.1	72.8	64.2	70.7	78.8	76.8	77.5	86.2	82.3	73.0	79.4
North Macedonia	30 to 34	67.4	79.4	72.4	81.9	69.1	81.3	65.6	77.7	73.0	81.7	64.2	77.4	61.6	74.6	60.5	72.2	65.8	79.4	68.1	81.2	76.2	86.0	68.2	81.5
Northern Mariana Islands	30 to 34	52.5	67.0	60.9	77.8	56.8	66.8	43.5	55.5	54.0	67.4	47.2	56.3	70.6	79.4	61.1	80.8	78.5	80.7	74.7	84.1	84.4	91.4	71.7	80.9
Norway	30 to 34	79.0	95.1	82.2	95.0	81.1	96.1	81.2	96.9	85.8	95.4	80.3	97.5	63.6	90.2	61.9	89.9	67.8	91.7	84.4	98.5	91.6	99.7	81.5	97.6
Oman	30 to 34	79.0	95.4	81.5	96.6	82.5	96.4	67.1	81.2	77.6	90.2	67.5	81.1	91.9	100.0	90.8	100.0	93.0	100.0	71.5	86.6	73.7	88.5	77.9	89.6
Pakistan	30 to 34	64.3	62.9	70.5	69.4	66.9	64.9	58.0	56.2	66.8	64.6	58.8	58.0	66.5	62.9	72.1	65.9	64.4	62.8	78.8	74.0	82.4	80.6	81.9	74.6
Palau	30 to 34	43.8	40.4	68.2	71.3	36.2	36.7	36.1	30.4	64.3	66.9	27.1	23.5	68.4	68.8	74.2	74.0	66.5	68.9	64.3	58.3	83.6	84.2	52.6	49.2
Palestine	30 to 34	66.8	82.1	73.2	86.5	67.3	80.9	43.6	64.3	59.2	75.6	40.7	59.8	92.4	96.7	93.6	97.9	92.0	96.2	49.8	70.4	59.9	78.1	54.3	70.7
Panama	30 to 34	72.6	75.5	75.2	80.2	76.1	75.6	69.4	74.2	75.0	81.5	69.9	70.9	76.0	71.8	77.3	74.7	76.4	70.5	71.4	81.8	75.9	86.3	77.6	83.3
Papua New Guinea	30 to 34	36.9	43.7	38.0	50.5	50.2	50.2	0.0	4.6	0.0	14.1	23.2	19.1	66.7	73.2	62.5	72.9	72.3	75.8	81.0	82.7	81.0	84.9	87.6	85.6
Paraguay	30 to 34	45.7	66.6	49.1	72.6	55.5	68.5	46.1	64.0	52.8	72.9	53.2	63.3	45.7	65.6	41.7	65.2	53.2	68.3	68.6	82.2	74.2	88.6	72.0	80.3
Peru	30 to 34	54.1	67.1	61.6	76.7	56.9	65.2	52.3	59.8	62.6	73.9	53.1	55.4	60.1	70.1	62.8	74.2	59.3	68.4	69.1	79.8	74.5	87.0	73.6	77.7
Philippines	30 to 34	47.4	51.8	68.6	69.3	34.8	45.9	19.1	42.8	53.7	63.7	0.6	36.3	71.6	68.8	79.2	75.6	65.5	65.0	65.2	65.0	79.5	78.9	56.8	58.3
Poland	30 to 34	57.0	79.0	70.2	89.1	53.2	71.6	53.4	71.5	69.9	86.5	46.9	61.3	54.3	77.3	61.9	85.4	48.9	71.1	60.3	81.1	76.7	92.0	52.1	70.8
Portugal	30 to 34	48.9	82.4	59.8	85.1	51.8	83.6	51.4	80.9	62.2	85.5	53.4	80.2	50.0	76.4	52.3	76.1	50.9	78.7	31.1	86.7	54.9	89.4	27.1	87.9
Puerto Rico	30 to 34	76.5	88.7	85.3	92.5	68.6	84.3	72.7	86.8	83.6	91.2	63.1	80.3	80.2	89.8	86.7	94.6	69.9	83.1	76.8	92.2	90.0	97.7	64.0	86.1
Qatar	30 to 34	83.1	93.3	85.9	95.5	85.9	94.5	76.8	88.9	81.6	91.2	79.1	91.0	81.8	93.1	89.0	96.3	78.9	92.2	88.4	93.6	89.9	96.1	90.4	93.9
Republic of Korea	30 to 34	61.9	83.6	73.8	89.9	59.8	81.9	70.1	87.8	79.6	92.4	66.1	84.2	28.5	72.6	43.4	78.1	19.3	70.3	67.4	86.9	76.7	91.7	67.1	85.4
Republic of Moldova	30 to 34	64.4	77.6	76.0	86.1	60.6	74.1	47.8	61.2	64.2	77.5	42.7	52.2	81.9	86.4	89.9	92.2	76.5	83.3	75.8	85.4	85.9	92.3	70.3	81.6
Romania	30 to 34	62.7	75.1	73.4	84.1	60.2	70.4	52.7	67.6	67.5	82.1	47.9	58.7	66.8	71.7	69.7	77.4	66.3	68.6	62.1	77.9	77.4	87.3	54.5	72.4
Russian Federation	30 to 34	66.9	63.0	80.1	78.8	61.2	55.5	55.0	47.1	72.2	69.8	47.9	37.0	67.0	63.2	80.4	72.4	59.4	58.5	61.4	63.2	79.8	82.0	49.5	49.3
Rwanda	30 to 34	26.9	63.0	34.5	72.7	37.0	61.9	22.8	45.8	34.9	60.7	30.8	44.3	54.8	77.5	56.1	84.7	56.8	74.4	64.9	85.1	65.6	88.4	78.8	86.9
Saint Kitts and Nevis	30 to 34	31.4	80.9	33.9	85.1	47.0	80.8	23.5	77.7	31.5	80.5	38.4	79.9	52.4	82.0	48.5	87.9	60.4	76.9	23.1	83.1	31.8	88.1	41.3	82.3
Saint Lucia	30 to 34	48.6	72.1	66.4	82.4	38.1	66.3	41.3	64.6	64.2	80.7	27.8	55.4	58.1	78.5	67.1	81.2	46.1	76.6	44.2	73.0	65.2	84.8	31.8	65.4
Saint Vincent and the Grenadines	30 to 34	49.5	68.9	61.5	77.2	45.9	65.4	33.7	58.9	55.1	72.0	22.0	53.1	69.9	79.3	68.3	81.1	74.2	78.4	54.1	71.6	67.4	81.0	50.4	67.8
Samoa	30 to 34	53.4	54.2	66.2	65.0	51.6	53.3	45.2	46.1	62.2	61.8	40.3	41.3	69.2	70.8	71.7	69.7	69.5	73.9	81.2	78.6	87.6	86.8	79.3	75.0
San Marino	30 to 34	79.6	90.7	83.3	91.5	80.6	91.8	73.7	90.2	79.1	89.7	73.8	91.2	80.8	90.5	82.5	91.1	80.7	91.1	80.6	93.1	88.0	95.8	78.4	92.2
Sao Tome and Principe	30 to 34	70.4	76.4	74.4	78.1	73.5	80.8	60.5	67.5	66.7	71.9	64.3	71.3	79.8	79.8	84.6	84.7	77.2	77.5	77.5	78.8	86.6	87.3	74.6	77.9
Saudi Arabia	30 to 34	56.1	64.9	55.9	72.1	65.9	67.1	35.9	47.4	42.1	62.3	45.8	46.9	84.1	86.8	82.7	86.4	86.5	88.3	38.0	38.8	35.2	52.5	56.4	43.6
Senegal	30 to 34	66.0	74.6	70.6	79.3	69.5	76.1	50.9	61.0	57.3	66.0	57.3	66.0	76.2	79.6	82.0	86.2	72.9	75.8	73.4	78.9	84.7	89.1	69.1	74.2
Serbia	30 to 34	61.9	82.8	70.0	87.5	61.9	81.2	58.3	80.7	69.9	88.0	55.4	75.9	54.3	79.2	54.7	80.2	56.6	80.0	62.4	81.4	74.6	88.4	58.8	78.0
Seychelles	30 to 34	57.1	73.2	68.0	82.3	57.9	72.9	52.1	66.5	63.5	76.3	52.7	66.5	73.0	86.7	78.5	91.3	70.1	84.6	59.1	74.1	73.2	85.0	56.4	71.2
Sierra Leone	30 to 34	69.6	67.2	73.3	69.6	73.1	72.7	57.5	55.8	62.3	59.2	64.2	64.7	80.6	76.0	85.2	78.7	77.9	75.5	74.5	69.2	86.0	82.5	69.0	63.7
Singapore	30 to 34	73.2	88.6	79.2	92.3	74.0	87.4	75.6	92.0	82.4	93.4	73.4	89.7	70.5	89.2	74.3	91.6	69.4	88.2	72.5	90.9	74.7	94.7	78.2	89.3
Slovakia	30 to 34	69.6	85.0	79.2	89.5	66.1	82.7	60.0	81.3	75.5	89.1	52.3	74.9	71.7	85.3	76.3	88.5	68.8	83.4	70.1	85.1	82.9	91.9	63.6	81.1
Slovenia	30 to 34	77.9	91.7	86.0	93.2	74.1	91.5	74.9	93.8	85.2	93.4	69.5	93.3	79.8	93.6	84.4	96.7	76.3	91.5	79.2	91.7	89.3	95.2	72.3	90.1
Solomon Islands	30 to 34	58.8	56.8	73.2	70.5	53.3	51.1	54.4	52.8	70.2	68.8	47.3	44.4	77.1	75.8	84.8	80.0	73.0	73.9	84.7	81.8	90.9	88.9	81.2	77.6
Somalia	30 to 34	44.5	54.4	56.1	68.9	46.9	52.4	32.2	36.0	46.0	55.3	35.9	34.6	65.9	71.1	73.4	80.6	63.7	68.3	80.7	84.8	83.0	89.0	85.7	85.5
South Sudan	30 to 34	42.0	64.1	64.3	69.6	59.6	60.4	17.4	36.2	52.1	50.5	48.6	40.8	71.2	82.6	79.5	81.6	75.0	74.1	57.0	74.3	84.7	86.8	85.3	84.4

Spain	30 to 34	56.2	60.9	74.8	89.2	66.3	86.4	43.2	37.7	73.6	90.8	65.3	85.5	75.5	76.4	71.2	83.4	62.4	84.6	81.9	82.8	78.8	93.4	66.3	89.7
Sri Lanka	30 to 34	65.9	86.3	76.6	83.3	60.8	69.3	64.6	87.4	68.9	75.2	49.0	55.0	65.7	83.2	89.3	93.7	81.3	87.3	68.1	90.4	75.2	82.6	47.7	57.1
Sudan	30 to 34	65.4	74.3	42.4	66.3	57.3	72.2	54.2	61.7	35.8	51.3	39.0	55.4	84.8	90.2	67.9	78.8	73.0	81.5	57.7	67.5	28.3	50.1	63.9	59.2
Suriname	30 to 34	42.7	65.1	60.2	65.0	41.3	45.4	27.0	46.2	56.3	58.8	30.8	31.1	69.2	79.3	67.2	68.1	55.9	59.9	34.3	46.3	73.8	76.4	45.4	49.2
Sweden	30 to 34	45.1	50.9	83.6	100.0	85.3	100.0	37.2	39.2	87.8	100.0	84.4	100.0	60.9	63.6	70.0	93.5	77.1	94.3	55.4	59.9	85.8	100.0	83.5	100.0
Switzerland	30 to 34	82.4	100.0	78.9	92.1	80.3	94.6	84.4	100.0	81.3	93.8	78.7	98.6	72.6	93.4	65.6	87.8	74.6	91.7	81.9	100.0	85.5	97.3	78.7	96.1
Syrian Arab Republic	30 to 34	76.9	92.6	63.2	79.1	60.5	75.3	77.3	96.4	58.9	69.7	48.5	60.8	69.0	89.2	88.5	99.1	86.7	97.4	79.0	96.2	49.3	69.1	53.5	68.0
Taiwan (Province of China)	30 to 34	56.3	75.1	85.5	93.8	72.1	81.7	46.9	61.5	81.7	90.6	67.2	74.6	87.0	97.8	92.6	98.3	87.9	89.6	43.2	63.1	84.3	92.7	72.3	85.7
Tajikistan	30 to 34	75.9	86.2	61.8	75.7	62.0	66.1	71.4	80.3	55.7	67.3	51.2	49.1	89.5	93.2	70.7	81.6	76.5	75.5	75.4	87.8	56.8	74.3	45.5	58.5
Thailand	30 to 34	56.9	68.2	80.0	76.0	65.4	32.3	46.9	54.2	76.7	71.3	65.4	28.1	72.5	77.4	84.3	78.1	63.5	38.1	43.5	62.5	87.3	81.3	75.6	45.7
Timor-Leste	30 to 34	69.0	46.4	59.4	66.3	45.6	46.7	67.0	39.8	51.2	58.7	33.9	36.6	72.9	55.3	74.6	77.1	60.3	62.1	79.3	60.3	78.2	80.7	66.8	56.2
Togo	30 to 34	46.1	51.2	62.8	71.0	71.9	70.6	34.5	40.6	50.3	58.4	61.2	59.5	65.8	68.4	75.8	81.7	75.8	73.2	68.5	65.5	81.3	85.4	73.3	68.5
Tokelau	30 to 34	62.8	66.8	54.5	47.6	41.3	48.5	48.6	52.8	53.5	42.1	29.0	29.5	74.9	76.2	62.8	53.2	64.1	72.1	73.3	73.4	79.8	74.9	66.8	68.6
Tonga	30 to 34	42.7	41.3	72.0	76.3	75.5	74.2	35.9	26.9	63.3	65.7	62.2	61.0	62.1	61.5	79.9	82.5	85.6	85.5	70.6	67.1	89.3	89.6	86.6	84.2
Trinidad and Tobago	30 to 34	70.2	72.3	74.1	79.6	51.3	62.7	57.3	58.4	69.7	77.4	40.7	54.7	81.9	83.2	68.4	69.4	41.4	47.9	86.0	85.2	81.9	84.1	30.7	56.5
Tropical Latin America	30 to 34	59.0	68.5	43.3	68.4	29.2	62.3	52.2	63.4	49.7	68.9	22.1	52.3	55.3	58.8	34.7	59.5	28.1	57.2	52.6	67.7	66.2	80.8	41.3	74.9
Tunisia	30 to 34	68.9	79.8	71.1	84.0	74.1	79.7	55.2	68.9	61.6	76.1	60.2	68.3	84.0	91.2	84.4	92.4	85.0	90.9	58.2	67.0	61.1	76.6	68.7	66.0
Turkey	30 to 34	66.0	87.0	78.1	90.5	62.1	85.8	59.6	80.0	72.9	84.7	55.8	78.7	57.6	83.4	71.8	89.1	46.4	78.9	63.7	85.3	76.3	89.8	60.4	84.5
Turkmenistan	30 to 34	66.9	64.1	71.7	78.1	69.5	59.1	59.6	53.8	67.2	72.8	61.1	47.8	68.1	62.8	72.1	75.5	66.9	57.1	55.8	61.3	63.2	75.3	62.8	56.3
Tuvalu	30 to 34	25.9	37.9	39.6	52.8	27.0	39.6	21.6	31.2	41.2	50.4	18.7	29.8	50.3	61.6	49.2	58.7	54.6	66.1	63.5	63.8	74.8	78.5	58.7	57.8
Uganda	30 to 34	63.3	66.9	75.1	78.9	61.6	63.4	45.4	47.4	59.8	63.2	46.3	46.9	76.2	77.9	84.8	87.2	72.8	73.6	87.0	86.3	89.7	90.3	88.7	86.5
Ukraine	30 to 34	74.6	68.2	84.6	83.2	70.6	62.9	67.2	59.4	80.2	77.9	59.6	50.0	74.3	67.0	82.3	79.2	70.0	61.7	71.8	67.4	86.4	86.1	62.6	56.3
United Arab Emirates	30 to 34	86.1	87.7	87.0	87.8	89.1	90.8	77.3	80.7	81.0	82.7	80.3	84.3	86.8	91.1	84.9	88.4	88.7	92.5	82.6	87.4	86.2	89.5	84.6	88.8
United Kingdom	30 to 34	77.7	88.1	81.1	89.4	79.3	89.1	82.7	88.7	85.4	90.2	83.1	88.2	56.8	80.6	59.4	80.9	56.6	81.8	84.3	95.4	89.7	97.2	82.9	94.8
United Republic of Tanzania	30 to 34	56.7	70.3	66.2	78.7	58.0	69.2	42.1	52.5	52.7	65.1	46.3	52.3	73.1	78.5	79.2	84.8	71.1	76.0	83.1	83.5	86.0	89.5	86.0	81.9
United States Virgin Islands	30 to 34	59.0	72.8	73.7	88.2	47.3	54.0	51.7	63.5	71.8	87.0	35.9	32.1	63.8	78.8	72.4	89.8	51.9	62.1	65.6	76.4	82.1	93.4	50.2	55.0
United States of America	30 to 34	90.2	97.1	90.3	97.4	91.8	96.7	82.5	88.2	85.6	90.4	82.2	87.4	64.7	83.4	68.4	88.4	61.5	78.2	96.1	97.6	96.5	97.3	96.8	98.5
Uruguay	30 to 34	57.6	75.5	62.2	79.9	63.1	76.2	66.3	82.7	73.1	87.0	66.5	79.8	41.6	64.8	43.0	69.7	42.9	60.3	69.9	84.8	74.1	88.5	75.6	85.8
Uzbekistan	30 to 34	56.4	72.2	63.8	81.2	58.8	69.0	38.3	64.2	52.2	76.1	38.6	60.0	78.1	82.3	80.8	89.6	77.9	77.9	52.6	65.4	62.8	79.8	55.9	57.8
Vanuatu	30 to 34	41.5	45.5	64.2	64.2	31.4	39.9	31.2	36.5	58.8	59.2	17.7	28.7	59.4	63.6	67.1	66.1	56.2	64.5	72.1	73.0	86.8	86.6	60.6	63.4
Venezuela (Bolivarian Republic of)	30 to 34	58.0	67.1	66.0	74.5	57.5	62.3	55.1	64.8	67.1	74.5	50.4	56.4	61.1	66.6	63.1	72.6	60.3	56.4	67.9	76.1	75.2	83.2	69.4	73.5
Viet Nam	30 to 34	49.0	65.2	68.4	81.4	42.4	58.8	40.7	57.0	61.8	76.4	34.3	49.7	69.2	80.2	81.8	89.9	59.6	73.2	64.9	71.9	80.4	86.6	56.4	63.6
Yemen	30 to 34	50.2	61.9	56.5	67.9	55.4	64.7	34.6	46.0	48.6	56.8	36.4	48.6	76.2	80.4	80.2	83.4	73.0	78.4	47.4	46.7	48.4	56.7	64.0	52.2
Zambia	30 to 34	45.4	53.6	47.9	64.2	56.6	54.4	32.2	34.9	37.5	47.6	45.4	39.4	67.3	70.7	64.7	75.3	71.3	70.0	77.5	78.4	78.6	83.5	86.8	80.3
Zimbabwe	30 to 34	81.8	59.5	86.3	72.2	79.9	53.7	66.3	26.2	73.0	47.5	67.4	23.4	86.3	65.5	90.0	71.2	85.0	64.5	89.5	73.4	93.2	83.0	87.9	67.9
Afghanistan	35 to 39	40.5	46.0	45.5	48.5	35.0	44.8	44.5	41.7	58.2	51.5	38.6	46.3	66.8	67.2	70.1	70.1	62.6	66.0	20.8	15.6	16.9	16.5	53.3	33.7
Albania	35 to 39	72.9	80.5	77.1	86.6	68.2	73.3	67.9	72.1	74.6	81.2	68.6	69.8	80.9	87.2	83.3	90.3	79.0	84.0	89.1	90.4	92.6	94.5	88.2	88.2
Algeria	35 to 39	63.5	75.0	60.2	73.7	69.1	77.8	60.2	67.7	62.5	69.9	68.7	74.2	77.1	85.7	75.2	83.9	81.2	89.0	44.6	57.7	36.8	56.4	65.7	69.7
American Samoa	35 to 39	52.3	53.2	71.7	66.6	35.4	40.4	53.2	51.0	71.5	65.8	48.0	48.6	70.8	69.2	77.7	71.3	67.2	68.8	61.2	65.6	88.4	86.1	41.0	50.4
Andorra	35 to 39	78.9	89.6	78.2	89.1	80.3	90.5	83.3	94.6	84.7	93.7	85.4	95.6	69.9	85.9	62.8	83.2	77.6	89.4	86.0	94.1	89.4	96.2	85.5	93.2
Angola	35 to 39	58.5	66.5	64.6	73.1	53.0	59.3	54.5	57.6	62.8	66.3	57.1	58.9	74.7	77.9	76.6	81.1	74.6	76.5	79.1	80.8	84.4	87.7	79.0	77.3
Antigua and Barbuda	35 to 39	53.0	82.2	65.1	84.8	35.7	78.7	46.7	80.3	64.8	83.3	37.4	80.9	67.6	86.4	72.2	88.4	62.0	83.9	64.8	90.9	77.9	93.6	55.0	89.7
Argentina	35 to 39	50.3	76.9	56.3	80.5	44.9	73.5	63.4	80.8	72.2	86.4	62.9	78.9	44.5	76.7	45.0	77.5	46.0	77.1	73.5	90.2	77.8	93.3	75.4	89.3
Armenia	35 to 39	75.4	85.7	82.0	91.4	68.4	78.6	67.6	84.8	78.3	91.1	63.0	78.6	81.5	88.2	90.9	97.5	74.4	80.9	72.6	82.4	82.0	91.5	68.4	75.2
Australia	35 to 39	79.2	88.7	82.6	89.3	76.2	88.4	84.5	90.6	88.9	92.5	83.0	89.9	63.7	79.8	65.8	82.8	62.5	76.9	86.2	95.4	91.1	96.9	83.8	94.9
Austria	35 to 39	77.6	90.0	80.0	91.1	75.3	89.1	76.4	94.6	81.6	94.9	76.5	94.2	72.4	88.0	72.3	88.7	73.9	87.8	73.7	94.5	80.0	97.1	73.6	93.0
Azerbaijan	35 to 39	64.2	79.0	73.6	86.3	53.1	70.2	59.5	72.3	70.9	81.7	56.5	68.5	86.0	92.7	91.8	97.5	82.0	88.9	69.3	84.4	81.1	91.7	60.7	78.1
Bahamas	35 to 39	46.1	61.4	56.0	72.8	33.9	47.6	42.4	56.7	56.5	71.2	40.8	51.2	61.2	69.7	64.3	74.7	57.8	64.4	60.3	72.4	70.4	83.6	56.6	64.2

Bahrain	35 to 39	84.3	87.2	88.0	92.6	82.5	84.5	78.4	81.2	84.3	88.7	78.9	80.4	87.2	91.6	88.5	94.0	86.6	90.1	79.7	78.1	80.9	84.3	82.6	78.4
Bangladesh	35 to 39	29.0	47.6	40.4	64.3	21.1	34.7	38.1	49.1	49.9	66.0	41.3	46.2	41.0	57.8	49.6	66.3	40.3	55.9	49.8	61.1	59.0	77.2	51.9	53.2
Barbados	35 to 39	57.7	74.2	71.6	79.1	39.8	67.7	55.2	73.3	73.0	79.5	45.0	71.8	62.0	72.4	70.7	76.4	53.0	68.3	58.4	80.4	77.8	85.7	42.5	78.2
Belarus	35 to 39	62.8	67.3	78.1	81.9	47.7	51.4	56.1	57.5	72.9	76.2	50.7	48.7	69.6	66.5	77.4	76.5	65.1	60.3	64.2	75.3	82.2	88.2	50.0	64.4
Belgium	35 to 39	66.7	87.9	69.4	86.9	65.0	89.8	64.2	93.2	70.5	91.3	66.6	96.2	66.3	80.8	63.4	78.7	70.7	84.1	71.6	94.3	79.1	96.4	70.2	93.9
Belize	35 to 39	64.8	67.6	68.4	76.7	61.2	56.1	60.4	60.5	65.7	71.9	65.1	57.5	72.6	79.0	76.6	83.2	68.2	73.6	80.4	77.0	86.7	87.9	77.5	67.8
Benin	35 to 39	68.1	72.4	69.9	75.3	67.5	70.1	62.5	66.0	65.8	70.3	68.6	69.9	81.4	83.2	83.6	85.3	80.0	81.9	79.3	78.6	88.9	89.6	74.2	71.7
Bermuda	35 to 39	77.7	89.9	84.1	92.7	69.1	84.9	79.3	92.4	85.0	93.6	75.9	89.7	81.4	95.1	88.7	99.5	72.3	88.7	77.7	92.2	88.4	98.0	69.0	86.1
Bhutan	35 to 39	59.5	72.0	63.5	76.4	56.6	68.4	57.4	67.2	65.8	74.7	58.9	67.1	66.3	75.1	68.1	76.6	66.9	75.3	74.0	81.0	75.8	86.1	78.2	80.0
Bolivia (Plurinational State of)	35 to 39	37.1	62.7	40.7	64.9	34.1	60.8	40.7	59.4	51.3	67.2	44.8	61.4	46.9	62.6	44.8	59.7	50.8	66.9	48.4	77.1	52.1	80.8	58.7	79.1
Bosnia and Herzegovina	35 to 39	74.1	88.3	83.0	91.1	65.9	85.5	65.8	82.4	80.1	90.0	59.9	78.0	58.2	82.5	69.7	85.8	48.9	79.3	73.4	84.5	85.9	91.0	67.5	81.9
Botswana	35 to 39	57.3	74.1	69.3	81.4	45.0	66.6	48.6	66.7	58.4	74.9	51.2	66.5	79.1	88.3	84.5	91.6	75.3	86.2	78.4	86.1	86.8	93.0	72.5	80.8
Brazil	35 to 39	23.0	55.8	36.1	61.9	8.4	49.3	32.6	55.9	52.7	68.4	26.3	52.4	25.6	51.1	29.5	51.6	21.7	52.2	34.5	68.1	54.8	76.3	26.9	66.7
Brunei Darussalam	35 to 39	53.3	59.0	54.2	69.3	53.3	50.6	64.9	62.2	69.7	76.8	68.1	57.1	32.5	42.7	24.3	49.4	41.8	41.0	65.1	77.5	62.2	80.5	73.2	78.4
Bulgaria	35 to 39	48.8	64.6	65.9	77.3	32.0	52.0	51.9	60.5	68.7	76.1	46.7	54.5	58.6	67.0	68.3	73.7	51.3	62.2	46.0	56.1	70.1	75.5	31.1	45.2
Burkina Faso	35 to 39	65.1	68.9	69.4	76.3	61.0	61.0	57.8	61.8	66.2	72.4	59.1	59.7	81.9	83.6	86.4	90.1	77.9	78.4	81.6	80.2	90.0	92.1	76.5	71.4
Burundi	35 to 39	42.7	58.4	54.0	65.8	31.0	52.1	45.8	51.5	59.8	61.7	44.2	53.0	61.2	75.1	66.2	79.1	59.8	74.0	68.1	80.3	72.1	84.0	73.3	81.9
Cabo Verde	35 to 39	61.7	81.6	70.1	83.2	50.5	81.4	59.7	77.1	69.9	79.4	57.7	80.3	75.2	89.0	81.6	91.6	68.1	87.1	59.7	77.4	83.6	91.3	35.5	70.8
Cambodia	35 to 39	32.3	54.7	35.8	61.4	30.0	48.3	35.7	55.4	44.3	64.0	44.5	57.3	59.9	74.4	61.2	76.7	60.0	73.4	54.7	70.3	58.5	74.9	63.4	72.7
Cameroon	35 to 39	59.1	63.7	62.5	68.9	56.9	59.5	54.6	58.7	61.9	66.4	58.4	61.1	76.1	76.1	79.6	81.0	73.8	73.1	75.9	71.4	86.3	86.6	71.3	62.9
Canada	35 to 39	83.7	88.6	85.3	90.0	82.5	87.6	87.6	90.3	91.1	92.2	85.5	89.3	73.4	87.0	76.8	90.1	69.5	82.7	90.2	93.5	93.9	96.0	89.0	92.5
Central African Republic	35 to 39	48.4	49.4	61.6	62.4	35.2	36.0	46.7	43.7	60.9	58.1	45.7	43.3	67.3	68.3	74.0	75.6	64.1	64.7	73.2	74.6	81.4	84.2	70.3	69.2
Chad	35 to 39	65.3	65.8	68.2	71.0	63.5	61.0	60.0	61.0	65.7	68.4	64.3	62.9	77.7	76.4	80.9	81.1	75.4	73.1	76.6	73.1	87.0	87.8	70.9	63.1
Chile	35 to 39	68.5	82.3	73.3	86.1	63.5	77.7	74.3	88.3	82.2	93.6	70.3	83.3	64.5	76.0	66.3	78.9	63.6	73.4	77.2	89.4	83.1	94.1	76.7	87.1
China	35 to 39	49.7	64.0	63.8	82.3	34.4	48.3	56.3	61.0	71.1	79.8	50.9	54.9	51.6	80.2	57.0	88.1	48.9	74.3	50.1	62.5	65.2	81.3	44.0	51.4
Colombia	35 to 39	58.3	75.9	62.9	80.1	52.9	69.8	60.3	81.0	68.4	86.5	60.4	77.3	59.2	70.9	61.2	74.0	57.6	68.0	60.5	82.9	72.4	89.8	54.8	78.1
Comoros	35 to 39	54.1	66.9	58.5	71.4	50.5	63.1	50.8	58.3	57.8	66.0	56.0	60.6	73.0	80.0	72.8	81.5	74.4	79.9	75.7	83.0	79.9	87.9	78.5	82.5
Congo	35 to 39	53.4	62.6	62.4	65.6	44.5	60.4	50.4	50.6	60.3	56.9	52.5	57.0	71.7	76.2	74.8	74.7	70.8	78.4	73.8	76.3	82.7	82.3	69.5	75.8
Cook Islands	35 to 39	55.0	72.0	66.6	82.7	43.4	61.1	56.4	67.9	69.0	79.9	54.1	63.4	67.3	85.7	71.0	89.1	65.4	82.9	64.6	78.8	84.8	91.4	49.6	68.8
Costa Rica	35 to 39	76.5	83.5	79.1	87.6	73.0	76.6	77.6	87.2	82.4	92.0	76.1	82.1	78.0	80.3	78.9	85.9	77.2	73.1	77.0	88.2	82.0	92.6	77.1	85.7
Coted'Ivoire	35 to 39	67.7	68.9	73.5	74.4	64.3	64.8	62.4	61.9	70.1	69.2	64.3	64.1	77.8	78.9	83.7	84.0	74.8	75.9	71.3	66.7	88.1	88.2	65.7	55.3
Croatia	35 to 39	63.7	91.2	78.7	97.6	47.9	82.8	64.5	89.6	81.8	98.1	55.9	82.0	63.5	89.0	72.6	95.6	54.9	82.4	64.5	90.3	82.1	95.8	52.7	85.5
Cuba	35 to 39	58.0	75.8	64.6	82.4	50.4	67.7	56.6	68.9	66.0	79.9	56.7	63.6	67.1	87.7	70.2	88.8	63.7	86.3	63.5	79.6	76.2	89.3	56.1	72.3
Cyprus	35 to 39	72.3	91.0	70.4	92.2	75.5	89.4	79.6	94.1	80.5	93.2	84.1	95.2	65.3	88.5	57.3	89.6	73.7	87.7	79.5	94.6	79.2	96.8	84.6	92.9
Czechia	35 to 39	71.5	89.5	80.9	92.2	61.2	85.8	72.6	91.0	84.4	94.0	66.4	88.1	67.7	86.9	73.2	87.8	62.7	85.9	65.5	89.0	81.2	93.1	55.5	86.3
Democratic People's Republic of Korea	35 to 39	52.8	44.3	63.8	60.9	41.6	30.1	56.6	43.8	69.4	63.3	53.7	39.7	65.1	68.3	67.7	72.3	63.9	66.6	57.6	49.2	66.1	64.4	58.3	46.2
Democratic Republic of the Congo	35 to 39	63.1	65.2	67.8	73.0	59.2	58.1	59.8	59.4	65.5	68.6	63.5	60.1	78.5	79.0	79.3	82.9	79.0	77.8	79.5	82.3	84.1	89.0	80.0	79.2
Denmark	35 to 39	69.8	87.6	73.0	89.1	67.2	86.4	72.5	94.5	80.4	95.7	71.2	93.3	64.8	85.8	63.0	85.5	69.2	86.8	71.1	93.6	79.5	97.1	68.7	91.3
Djibouti	35 to 39	59.5	69.2	69.7	77.9	51.9	61.5	55.2	59.8	65.6	70.1	56.4	60.1	75.0	79.4	82.1	86.0	72.6	76.5	82.3	83.5	88.7	91.9	80.6	78.5
Dominica	35 to 39	67.3	67.8	72.2	74.2	62.0	59.8	64.4	61.5	71.5	70.7	64.8	60.2	76.1	75.5	79.5	79.3	73.1	71.9	77.4	72.9	83.9	82.3	75.2	67.1
Dominican Republic	35 to 39	47.9	57.0	56.6	64.4	36.7	49.0	44.6	53.2	58.7	64.7	41.8	51.8	59.7	67.0	61.6	66.2	57.3	69.4	59.5	64.3	75.9	78.6	48.0	55.5
Ecuador	35 to 39	52.8	74.3	60.5	79.0	44.1	69.1	46.7	73.9	59.5	81.9	46.4	71.3	70.0	73.2	72.4	75.8	68.4	71.3	45.3	84.3	57.4	89.8	44.6	82.4
Egypt	35 to 39	45.4	70.5	54.9	76.2	33.0	64.6	31.1	60.5	52.6	72.8	23.6	57.1	69.0	86.1	75.9	89.2	61.4	83.2	4.9	36.4	9.4	51.1	25.1	36.2
El Salvador	35 to 39	57.5	69.4	66.5	78.4	45.6	53.0	62.2	64.4	72.7	79.0	59.0	52.7	65.2	73.8	69.6	78.6	60.5	66.5	58.8	72.9	74.6	86.6	47.5	57.1
Equatorial Guinea	35 to 39	51.7	73.6	61.3	77.3	40.6	70.1	47.6	60.3	59.1	66.7	48.4	63.7	70.2	85.0	75.9	85.1	67.2	85.5	75.5	80.8	83.7	87.4	71.2	78.3
Eritrea	35 to 39	35.4	52.2	49.4	65.2	22.1	41.1	35.9	47.0	50.0	60.8	37.7	47.2	61.8	71.3	71.6	79.0	56.9	68.2	73.2	78.2	79.3	86.7	73.7	74.4
Estonia	35 to 39	67.5	84.7	77.4	90.9	59.3	77.6	66.7	80.4	76.2	91.0	64.8	71.0	69.5	88.6	83.4	97.4	59.6	82.4	64.4	88.9	76.8	95.3	59.9	83.8
Eswatini	35 to 39	59.2	52.0	71.2	70.3	42.5	32.4	45.0	38.0	60.9	56.6	41.8	36.5	82.2	77.2	86.2	83.9	78.9	72.5	83.3	74.9	89.1	85.7	79.2	66.1

Ethiopia	35 to 39	51.5	68.6	58.0	74.2	45.1	63.3	54.3	60.8	63.4	68.4	55.6	62.8	74.1	83.1	77.2	86.3	73.3	81.7	83.0	87.7	85.2	90.3	86.7	88.5
Fiji	35 to 39	54.2	62.1	64.5	67.4	42.9	56.9	49.5	52.9	63.5	62.1	47.3	55.2	70.2	74.1	73.5	74.3	67.4	75.4	66.5	73.2	86.4	86.9	52.4	64.7
Finland	35 to 39	64.0	85.4	73.0	88.0	57.0	83.3	67.7	91.2	81.9	93.1	63.6	90.8	60.3	80.1	63.1	83.1	60.2	78.4	57.7	93.9	75.2	96.9	51.6	92.0
France	35 to 39	69.7	83.1	79.1	86.7	60.0	80.0	68.3	92.4	79.2	94.1	65.0	91.5	70.7	80.6	74.8	83.0	68.3	79.3	75.8	89.8	87.7	95.5	67.2	85.9
Gabon	35 to 39	63.7	72.3	73.1	79.3	56.4	65.3	60.2	63.2	69.8	72.2	60.9	63.4	80.0	83.4	84.5	86.8	78.3	81.7	81.6	83.1	88.6	90.4	79.2	79.1
Gambia	35 to 39	65.1	64.3	70.9	69.5	60.6	60.0	56.8	54.9	64.7	62.8	59.5	58.0	77.8	75.3	83.3	80.1	74.2	72.2	69.4	65.3	87.5	85.3	59.5	53.2
Georgia	35 to 39	69.0	71.9	82.9	88.5	53.7	57.1	70.0	72.1	82.0	87.8	65.4	66.8	78.0	73.2	89.2	86.8	70.3	66.9	77.1	68.3	87.2	88.5	67.5	49.2
Germany	35 to 39	70.1	87.5	74.0	88.6	66.8	86.7	70.2	91.4	76.9	93.1	70.5	90.7	60.7	83.5	60.2	83.1	62.9	84.7	68.5	91.4	79.5	95.8	64.3	88.8
Ghana	35 to 39	56.2	67.5	59.1	72.8	55.0	63.0	49.7	58.5	56.8	66.4	55.4	60.6	66.6	73.6	69.6	79.1	65.3	70.3	62.6	68.1	81.0	87.5	51.6	55.2
Greece	35 to 39	71.7	82.2	75.6	86.1	68.4	78.3	71.6	81.5	76.8	85.7	73.0	81.0	78.3	83.8	78.3	86.9	79.3	81.3	71.1	88.4	78.2	93.3	70.3	85.5
Greenland	35 to 39	50.2	70.4	54.2	71.7	47.6	70.0	55.1	69.7	71.7	78.4	49.4	67.8	0.0	50.1	2.0	48.1	0.0	54.4	75.5	90.5	82.4	93.0	75.3	90.4
Grenada	35 to 39	37.9	63.0	48.8	69.1	25.7	56.4	37.3	57.1	53.8	68.3	34.5	55.0	49.6	68.3	50.1	68.4	50.5	69.0	25.5	66.6	46.1	75.5	17.8	64.2
Guam	35 to 39	73.3	67.8	82.7	73.4	64.5	63.0	71.0	62.7	80.9	68.6	67.9	65.3	80.0	76.9	83.6	73.5	77.9	80.0	72.5	74.8	91.1	87.4	59.6	66.3
Guatemala	35 to 39	54.5	64.3	57.8	69.3	51.8	57.8	59.2	62.2	65.2	69.8	63.3	62.5	79.3	75.7	80.7	77.3	78.4	74.5	53.4	76.5	62.0	83.3	55.5	73.4
Guinea	35 to 39	62.8	65.6	62.7	68.8	65.3	63.4	59.6	61.6	62.9	67.1	67.4	65.8	78.5	77.4	79.0	80.0	78.6	75.8	73.4	69.6	83.7	85.9	68.9	57.5
Guinea-Bissau	35 to 39	44.0	50.4	46.8	56.4	42.8	44.8	41.0	43.8	48.7	54.2	48.7	47.0	58.7	61.7	60.4	65.4	58.5	59.7	58.7	55.8	74.6	78.7	50.2	39.5
Guyana	35 to 39	38.1	45.9	53.7	61.2	21.9	26.9	38.6	43.0	55.4	61.8	36.2	35.0	61.0	54.9	71.4	66.1	51.8	43.1	43.0	45.0	70.1	70.3	24.9	25.9
Haiti	35 to 39	0.0	22.3	0.0	26.9	4.8	17.9	16.2	28.0	30.5	43.6	23.0	26.7	10.3	30.1	0.0	23.8	26.2	40.2	0.0	28.7	1.1	36.6	28.4	37.8
Honduras	35 to 39	20.4	48.9	23.6	48.9	18.0	51.3	18.6	39.3	33.8	49.2	24.8	47.2	27.3	43.0	22.9	35.7	33.9	54.1	19.0	60.5	27.1	64.9	31.6	66.7
Hungary	35 to 39	55.7	85.8	69.3	91.8	41.6	77.0	56.5	83.2	73.3	94.3	50.1	74.7	52.9	84.0	56.8	86.1	49.7	82.1	44.1	81.5	70.5	89.9	27.5	74.4
Iceland	35 to 39	77.6	91.3	77.2	91.8	79.3	91.1	81.4	95.5	82.9	94.3	84.0	96.8	72.7	89.2	68.1	89.9	79.0	88.9	81.9	96.9	86.1	98.9	82.0	95.4
India	35 to 39	58.1	66.6	65.9	74.2	50.8	59.9	57.4	64.7	69.0	74.0	54.9	63.5	63.4	69.5	69.5	73.7	60.7	68.2	73.0	80.0	78.8	86.1	73.4	77.8
Indonesia	35 to 39	41.5	47.9	50.2	59.0	31.9	35.8	44.5	45.2	57.4	59.9	44.2	43.3	58.4	65.3	62.4	68.9	56.2	63.5	57.8	57.8	66.7	71.2	57.6	51.7
Iran (Islamic Republic of)	35 to 39	76.1	86.7	79.0	91.0	72.8	81.9	68.3	78.4	75.0	85.6	68.8	76.3	85.7	94.7	87.2	97.0	84.4	91.8	39.5	61.0	48.3	72.3	44.5	58.6
Iraq	35 to 39	54.8	71.0	64.0	75.5	44.9	66.5	52.0	66.6	65.6	73.2	50.4	68.2	73.2	83.0	75.9	83.1	71.1	83.7	24.2	46.5	32.8	52.6	33.7	53.7
Ireland	35 to 39	74.7	90.4	76.3	90.5	73.7	90.3	82.1	98.6	84.9	96.4	82.8	99.4	68.4	86.0	66.9	86.4	71.6	86.2	78.6	95.9	85.1	98.0	76.5	94.6
Israel	35 to 39	74.6	89.9	78.1	91.8	71.9	88.5	71.5	93.9	78.2	96.5	71.6	91.7	80.7	92.7	82.1	94.7	80.1	91.0	81.9	95.9	86.3	98.4	81.6	94.3
Italy	35 to 39	74.0	92.0	78.8	94.4	69.3	88.8	74.1	88.5	80.5	92.3	73.5	86.8	69.7	88.5	71.4	90.4	69.3	86.3	78.2	96.1	85.4	97.9	75.2	94.8
Jamaica	35 to 39	60.2	65.0	64.7	72.4	55.0	54.4	59.9	61.2	66.8	74.4	62.6	52.6	65.9	75.4	69.6	75.1	61.8	76.3	63.3	67.9	70.4	78.1	63.8	61.7
Japan	35 to 39	68.9	77.6	79.9	86.8	58.9	70.6	75.3	83.7	86.4	95.7	69.9	78.1	56.3	71.8	67.7	78.6	49.3	68.4	78.5	90.6	84.4	95.0	76.8	87.4
Jordan	35 to 39	69.4	88.7	70.1	92.8	69.7	84.7	54.8	78.8	65.0	86.4	54.7	75.2	91.6	98.8	92.0	100.0	91.5	97.3	42.4	72.1	45.1	81.0	54.7	70.0
Kazakhstan	35 to 39	70.4	72.8	78.6	82.1	61.7	62.1	66.4	66.0	76.4	78.5	63.5	60.7	65.7	74.9	77.3	83.3	58.2	70.4	60.3	72.6	75.6	82.5	52.4	66.4
Kenya	35 to 39	72.3	72.2	75.4	75.5	70.3	69.7	66.2	63.6	70.8	68.2	69.5	67.6	84.8	84.2	85.8	84.6	84.7	84.4	89.2	87.6	91.1	90.6	90.7	88.3
Kiribati	35 to 39	51.1	47.5	68.9	69.8	38.0	31.6	61.8	56.6	73.4	72.9	60.6	53.5	64.7	66.5	81.2	80.3	51.9	59.1	58.6	47.0	88.9	89.2	38.0	19.8
Kuwait	35 to 39	89.7	92.0	88.7	95.5	91.0	87.8	82.4	85.7	84.5	91.4	84.7	82.3	90.9	98.3	88.6	99.8	92.8	96.3	81.0	81.9	81.4	89.0	84.9	78.6
Kyrgyzstan	35 to 39	57.7	60.9	65.9	78.7	49.6	42.1	57.8	60.7	65.7	77.7	59.5	53.9	63.1	63.1	69.7	77.4	59.1	54.1	50.7	36.8	63.2	75.3	47.3	3.7
Lao People's Democratic Republic	35 to 39	10.6	40.4	18.8	51.9	2.7	29.2	20.7	42.2	35.2	56.4	26.0	42.1	36.9	64.0	39.1	68.1	37.3	62.0	18.3	49.9	25.0	64.8	32.0	45.1
Latvia	35 to 39	65.8	78.5	76.3	89.1	55.0	66.8	58.9	67.1	71.2	84.2	55.7	56.6	72.0	80.9	81.8	91.8	65.6	74.1	60.2	79.0	76.7	91.5	50.0	68.6
Lebanon	35 to 39	54.3	88.2	63.8	91.7	43.3	84.7	42.8	79.8	63.7	86.6	32.4	77.0	62.7	90.1	69.0	93.0	54.5	86.7	47.0	86.3	48.0	89.2	58.1	86.1
Lesotho	35 to 39	65.8	38.0	77.4	61.4	47.8	12.8	51.1	28.8	67.2	54.2	45.3	23.0	85.7	69.4	91.0	79.0	80.4	63.3	88.3	67.7	92.9	81.9	84.0	56.3
Liberia	35 to 39	65.0	63.8	67.8	66.7	63.8	62.2	61.4	55.6	66.8	61.9	65.6	60.4	78.9	76.2	81.6	78.6	77.7	75.1	72.3	67.8	85.9	84.0	65.3	58.7
Libya	35 to 39	73.1	73.6	70.0	74.9	77.6	72.4	63.4	61.1	66.9	66.4	69.9	65.9	83.4	84.3	80.4	81.2	87.1	88.4	51.1	40.3	48.6	50.5	66.4	43.8
Lithuania	35 to 39	68.7	79.6	80.3	88.6	56.3	69.6	60.6	72.1	75.3	86.3	54.8	63.9	65.1	77.0	78.8	88.2	55.2	68.9	66.6	84.0	81.9	94.0	56.0	75.5
Luxembourg	35 to 39	65.6	94.2	71.1	94.3	61.0	93.9	66.0	97.2	73.8	97.4	66.8	96.6	65.3	91.0	64.0	91.9	67.8	90.5	61.5	97.0	73.6	98.6	58.1	96.5
Madagascar	35 to 39	39.4	45.9	50.4	55.6	29.2	37.0	44.6	42.1	56.0	53.8	46.5	44.5	58.8	64.0	63.0	66.3	58.4	64.5	67.6	72.2	77.2	81.2	64.5	69.0
Malawi	35 to 39	58.4	57.1	59.6	66.5	59.0	47.8	51.3	40.9	56.6	54.1	58.5	42.5	74.1	72.0	72.6	76.5	76.1	70.2	80.4	76.4	82.4	84.6	84.5	73.2
Malaysia	35 to 39	63.3	69.7	71.3	79.1	55.9	61.7	66.0	67.1	74.5	77.5	65.6	65.6	79.1	85.3	83.7	88.4	75.4	83.0	67.8	75.0	76.3	83.6	66.5	71.9
Maldives	35 to 39	34.3	70.4	40.5	81.6	29.5	66.0	42.3	68.7	50.5	78.3	47.5	69.9	61.9	87.7	64.5	92.2	61.2	86.1	46.5	76.2	52.8	86.7	52.5	74.6

Mali	35 to 39	55.7	65.4	48.3	61.8	70.0	72.6	49.2	56.0	48.5	55.6	68.6	70.3	72.7	78.6	62.6	72.4	81.7	83.9	73.5	75.1	74.6	81.2	81.8	75.6
Malta	35 to 39	77.5	90.1	77.1	91.5	79.2	88.7	78.5	91.1	78.5	92.6	84.1	90.7	83.0	90.8	81.0	92.1	86.0	89.9	74.0	94.5	78.0	96.8	76.3	93.4
Marshall Islands	35 to 39	42.2	30.7	51.7	49.4	33.9	13.9	50.2	38.6	59.6	56.2	52.7	36.6	53.7	50.5	58.1	57.1	52.3	48.4	63.3	49.7	81.5	79.9	51.5	26.7
Mauritania	35 to 39	64.8	75.3	69.7	76.5	60.6	75.4	59.2	67.1	66.6	70.3	61.8	72.8	77.1	84.6	82.1	86.5	74.0	83.4	69.5	77.1	87.3	88.9	56.9	69.2
Mauritius	35 to 39	45.8	49.5	67.5	72.5	25.2	26.0	49.3	47.0	71.6	73.9	39.8	36.4	61.1	63.6	76.1	74.7	47.6	54.5	21.8	45.7	55.3	71.0	6.5	29.5
Mexico	35 to 39	73.8	77.3	77.2	85.2	68.7	65.1	71.5	74.2	78.4	86.3	68.5	65.4	77.2	73.9	77.6	81.1	77.0	65.4	69.5	81.0	77.6	90.0	66.2	73.2
Micronesia (Federated States of)	35 to 39	22.7	32.5	44.4	53.2	4.4	14.2	36.3	39.0	54.9	57.8	34.2	36.2	37.3	53.2	51.9	61.4	29.8	49.6	39.0	43.8	76.4	79.7	14.6	17.1
Monaco	35 to 39	65.6	79.6	71.9	83.5	59.4	75.0	64.2	77.2	76.2	86.4	61.7	73.0	56.3	75.6	56.1	73.9	58.1	78.4	64.8	81.1	75.0	87.4	62.1	78.3
Mongolia	35 to 39	59.1	65.4	62.2	78.0	56.5	52.4	61.4	68.0	67.9	78.6	64.0	64.1	62.0	57.4	62.2	68.7	63.5	50.8	88.0	86.7	90.7	94.1	87.4	79.5
Montenegro	35 to 39	53.0	71.7	66.5	78.2	39.7	63.2	54.9	65.6	68.6	75.7	51.8	60.9	79.9	90.3	82.3	90.7	77.9	89.5	84.3	90.4	91.8	94.3	78.7	87.5
Morocco	35 to 39	57.0	71.8	55.8	67.3	60.0	79.6	50.6	62.3	56.3	62.7	58.4	74.4	73.1	82.4	72.1	78.6	76.1	88.6	25.7	42.9	21.7	36.2	48.0	67.1
Mozambique	35 to 39	56.4	45.0	66.0	70.6	46.4	24.0	48.7	36.2	61.0	63.3	48.6	30.5	72.5	61.5	79.3	80.0	68.9	52.9	78.9	66.2	85.7	87.0	76.5	52.5
Myanmar	35 to 39	8.9	37.6	18.1	58.7	0.0	18.4	17.4	37.4	31.2	57.7	23.9	34.2	39.3	62.2	44.1	72.2	37.5	55.7	25.0	53.8	38.1	72.4	28.6	43.7
Namibia	35 to 39	63.2	65.4	72.7	78.8	52.2	50.0	53.0	51.0	64.2	67.6	53.0	47.6	84.4	85.4	87.9	90.9	81.9	81.2	82.1	81.5	89.0	90.8	77.1	73.1
Nauru	35 to 39	28.8	31.0	42.3	43.0	15.8	18.7	38.5	34.5	52.3	47.9	39.7	37.1	40.0	47.7	39.8	43.8	42.3	53.4	29.4	35.5	70.3	69.4	0.0	8.9
Nepal	35 to 39	50.9	65.0	56.5	71.9	45.8	55.8	51.3	61.1	61.9	71.9	51.6	56.4	60.5	70.8	64.5	75.4	59.1	66.9	65.1	77.5	68.0	84.7	70.8	73.5
Netherlands	35 to 39	76.9	90.5	78.6	91.6	76.0	89.4	80.7	95.4	84.2	95.8	81.1	94.5	65.0	87.3	65.1	88.7	66.6	86.7	83.6	95.4	89.0	98.0	81.8	93.7
New Zealand	35 to 39	73.5	89.5	72.2	90.8	76.1	87.8	79.7	95.2	80.4	95.0	83.8	95.0	52.4	72.9	50.9	76.6	56.4	69.1	82.0	94.7	87.3	97.3	80.8	92.9
Nicaragua	35 to 39	59.6	75.4	63.6	80.6	54.5	67.0	54.7	70.2	64.5	80.9	54.2	61.6	69.6	80.0	69.6	82.2	70.5	77.3	61.2	77.2	69.4	86.2	60.7	70.7
Niger	35 to 39	67.5	70.8	69.1	73.9	67.8	68.9	64.1	64.6	68.6	70.6	68.5	67.3	80.5	83.5	82.0	87.2	80.0	81.1	83.6	84.0	89.2	91.7	82.9	80.3
Nigeria	35 to 39	70.1	80.5	73.5	85.1	68.0	75.3	62.0	71.9	67.8	78.6	65.4	71.5	85.3	90.5	89.3	95.2	82.9	86.0	79.7	80.7	89.9	94.5	75.9	69.3
Niue	35 to 39	53.4	60.8	66.7	69.4	41.7	52.3	56.1	57.5	69.1	68.2	54.0	56.9	64.6	71.9	70.6	73.0	61.4	72.0	65.2	70.7	85.9	86.4	51.6	59.9
North Macedonia	35 to 39	58.0	77.8	66.8	83.0	46.6	71.3	58.5	75.8	71.3	83.9	53.2	72.2	51.9	72.5	50.7	72.2	54.3	73.8	54.6	79.0	69.1	86.0	46.2	75.0
Northern Mariana Islands	35 to 39	46.9	62.6	56.5	74.9	40.4	53.1	50.2	60.3	58.9	71.0	53.1	60.2	63.2	73.8	55.6	76.7	68.7	73.0	53.6	72.4	82.1	90.5	39.6	60.0
Norway	35 to 39	76.7	92.6	81.2	94.3	73.2	91.1	82.5	97.6	89.5	99.0	79.2	95.6	61.5	87.6	62.8	89.9	61.8	85.9	83.0	97.4	91.2	99.7	78.7	95.5
Oman	35 to 39	75.6	92.4	79.5	96.1	74.0	91.2	69.2	80.7	79.4	93.0	68.9	79.1	90.1	100.0	88.5	98.7	91.4	100.0	59.8	79.7	66.0	85.4	65.2	81.7
Pakistan	35 to 39	64.7	64.0	69.8	69.7	60.4	58.7	65.0	63.1	72.6	70.9	64.9	63.5	68.4	65.2	72.6	67.8	66.7	64.8	78.4	75.1	82.3	81.8	79.8	73.3
Palau	35 to 39	43.4	45.5	69.0	73.9	20.2	28.7	48.8	48.7	72.2	74.9	40.3	43.2	61.2	65.3	73.5	76.0	53.5	61.9	40.7	36.5	84.2	86.6	7.2	14.5
Palestine	35 to 39	66.9	82.7	71.8	86.0	60.4	77.8	48.9	66.7	63.3	77.1	45.8	63.4	91.4	96.7	92.6	97.9	90.5	95.3	37.5	62.5	48.3	69.8	40.9	63.5
Panama	35 to 39	67.2	73.0	72.9	78.5	60.5	65.7	66.7	72.7	76.2	83.5	63.8	66.2	75.2	71.8	77.4	72.7	73.3	71.4	63.4	79.3	72.9	84.6	62.1	78.6
Papua New Guinea	35 to 39	19.1	31.3	22.8	41.1	15.3	21.5	0.0	8.6	0.0	18.5	18.9	19.4	47.9	60.7	48.4	64.0	49.3	59.3	63.8	67.5	75.2	81.1	60.2	59.4
Paraguay	35 to 39	42.4	60.7	45.0	63.8	40.9	58.6	48.9	61.0	57.0	68.8	53.1	62.3	43.2	59.9	38.9	55.1	49.9	66.1	64.9	79.2	70.5	84.5	66.6	78.0
Peru	35 to 39	57.3	71.4	63.3	78.1	50.8	63.9	58.6	66.4	68.2	78.0	58.2	62.5	65.8	75.9	67.7	77.7	64.8	74.7	69.2	81.4	75.2	87.9	70.6	78.5
Philippines	35 to 39	36.9	47.1	59.0	64.3	9.4	29.8	14.0	45.8	48.0	65.1	0.0	40.2	68.4	67.5	74.8	72.6	62.7	64.6	56.0	60.3	71.5	74.3	47.1	53.1
Poland	35 to 39	53.1	76.1	67.1	90.4	37.9	59.4	55.0	71.1	71.5	90.0	48.4	59.9	52.1	74.5	59.2	84.8	46.6	66.2	51.6	75.5	74.4	89.8	36.1	62.1
Portugal	35 to 39	51.8	81.1	59.7	84.1	45.3	77.9	58.1	80.7	67.9	86.5	59.0	79.1	51.7	77.8	51.5	76.5	53.7	79.7	35.5	84.8	52.9	88.8	33.2	83.8
Puerto Rico	35 to 39	72.4	87.2	83.1	92.5	55.8	77.1	72.1	86.1	84.6	93.2	62.1	76.9	77.3	91.4	84.7	96.8	64.5	81.8	74.1	92.6	91.0	99.4	55.7	84.1
Qatar	35 to 39	81.1	91.8	83.9	95.8	80.7	91.3	79.2	88.6	84.0	93.3	80.3	89.2	80.0	91.0	85.9	95.2	78.1	89.2	86.6	92.6	88.3	97.3	87.6	92.1
Republic of Korea	35 to 39	59.0	79.5	71.7	87.0	48.4	73.3	70.4	86.2	82.1	94.6	65.0	81.0	30.5	68.1	45.2	74.0	22.2	65.4	71.8	88.0	80.6	92.8	69.2	85.2
Republic of Moldova	35 to 39	59.6	75.2	72.6	86.2	46.2	64.3	51.1	61.0	66.7	78.1	46.7	53.2	80.7	85.9	91.4	95.1	73.2	80.0	72.9	85.5	84.4	93.6	65.3	79.5
Romania	35 to 39	58.5	73.1	70.5	85.9	45.7	57.6	54.0	66.8	69.0	84.8	50.1	56.5	63.4	69.2	65.9	77.4	62.4	63.1	55.6	74.4	74.9	88.6	43.0	62.1
Russian Federation	35 to 39	63.6	59.5	77.4	76.3	49.5	42.2	57.8	50.4	73.5	71.2	52.3	42.7	64.9	59.7	77.4	70.7	58.1	53.8	52.0	55.2	75.2	77.2	35.4	38.5
Rwanda	35 to 39	34.3	64.7	41.9	72.8	27.1	56.5	40.3	56.2	52.5	68.6	41.9	53.9	59.4	80.2	61.7	85.2	60.0	77.8	67.4	86.4	69.4	90.1	75.7	86.2
Saint Kitts and Nevis	35 to 39	29.2	73.8	41.8	79.7	13.1	67.6	30.0	70.8	47.8	77.3	26.8	71.2	47.6	78.9	50.2	83.6	45.8	74.5	24.0	76.8	46.6	85.6	10.5	72.0
Saint Lucia	35 to 39	44.5	71.1	59.9	79.7	24.5	60.1	42.2	67.2	63.0	80.3	33.0	60.7	57.8	77.6	59.9	79.4	55.6	75.7	40.2	75.1	62.5	85.6	24.1	67.4
Saint Vincent and the Grenadines	35 to 39	49.3	55.6	62.3	61.9	32.1	47.6	38.8	48.4	59.3	60.9	29.1	47.1	69.6	67.5	71.1	64.2	68.6	72.4	57.4	59.7	71.3	67.9	48.2	59.2
Samoa	35 to 39	50.8	53.4	65.3	64.6	35.3	40.9	53.8	54.3	69.2	67.9	48.4	50.3	61.9	66.7	69.8	69.4	57.0	66.1	70.5	70.4	87.4	87.8	56.8	55.3
San Marino	35 to 39	79.1	90.8	83.3	91.7	75.2	89.6	75.2	92.3	82.0	92.5	73.7	92.2	81.8	92.1	83.8	92.7	80.7	91.9	80.0	93.8	88.8	96.8	75.6	91.6

Sao Tome and Principe	35 to 39	66.3	74.8	71.4	73.6	61.0	79.3	61.5	69.6	70.0	72.0	61.5	75.5	76.6	79.7	80.8	78.9	73.6	81.2	72.3	77.1	85.9	86.0	62.4	75.6
Saudi Arabia	35 to 39	53.2	59.9	51.2	65.5	55.4	55.9	43.2	51.0	49.4	64.8	50.6	50.0	81.1	83.6	79.6	82.4	83.4	85.7	19.5	17.7	11.2	28.8	41.6	26.4
Senegal	35 to 39	62.7	72.1	66.0	75.3	60.4	69.5	53.7	62.9	59.7	67.5	59.4	67.3	75.0	79.5	77.8	83.0	73.4	77.2	68.3	75.9	83.4	88.5	59.5	68.4
Serbia	35 to 39	53.3	80.0	63.8	86.9	41.2	71.2	54.5	76.4	69.2	87.9	49.6	69.9	46.1	74.3	46.1	75.1	48.0	74.1	49.6	76.3	68.7	86.5	38.2	69.0
Seychelles	35 to 39	45.2	67.7	63.5	70.7	32.1	66.0	47.5	65.2	64.7	66.5	45.4	69.6	66.6	83.8	75.5	84.0	61.2	84.0	43.8	68.1	67.6	72.3	35.6	71.0
Sierra Leone	35 to 39	65.0	65.6	68.7	68.0	62.3	64.5	58.6	59.6	64.8	64.5	62.8	65.3	77.6	76.4	81.0	77.3	75.6	76.5	67.3	65.0	84.5	83.0	56.7	52.9
Singapore	35 to 39	70.8	83.0	76.3	89.0	66.4	77.9	76.1	88.1	83.1	94.3	73.9	83.0	68.1	83.5	71.0	87.5	67.5	81.4	76.1	91.8	77.2	95.5	79.4	89.2
Slovakia	35 to 39	64.5	85.4	77.1	91.3	50.6	78.1	57.4	79.8	75.4	90.4	49.0	73.3	67.8	86.2	72.9	88.7	63.5	83.6	63.2	85.3	81.1	92.1	50.9	80.2
Slovenia	35 to 39	72.5	94.5	83.2	96.6	60.9	91.2	72.2	95.6	84.7	97.3	66.4	93.1	74.3	95.8	79.2	100.0	70.2	91.4	72.6	94.4	87.5	97.4	61.0	92.0
Solomon Islands	35 to 39	50.6	50.6	69.1	67.0	32.2	31.9	58.5	57.6	74.1	72.6	52.2	50.6	63.6	65.9	78.0	75.4	54.1	59.2	73.0	72.5	89.9	88.8	57.8	55.5
Somalia	35 to 39	46.5	56.8	57.9	68.6	35.8	46.0	44.7	48.9	58.3	63.9	44.5	46.9	67.7	74.3	75.0	81.0	64.9	72.0	81.3	85.9	84.7	90.7	83.3	84.6
South Sudan	35 to 39	48.3	63.6	63.7	69.6	49.9	52.7	37.1	43.5	60.6	58.9	53.8	48.1	72.6	81.2	79.7	82.0	75.4	76.5	62.9	74.8	85.7	88.5	82.8	82.6
Spain	35 to 39	56.1	61.7	75.9	89.0	65.1	83.0	51.3	47.0	78.0	92.7	73.1	88.5	76.2	78.3	74.7	85.3	68.1	84.6	81.8	83.7	81.1	94.4	68.7	88.6
Sri Lanka	35 to 39	70.1	85.9	74.9	83.0	48.1	61.7	72.0	90.0	71.3	78.1	49.5	57.0	70.8	84.7	89.0	93.7	78.5	86.6	71.8	90.9	69.5	81.2	41.7	56.1
Sudan	35 to 39	62.5	73.7	37.4	61.3	43.4	64.7	55.4	63.9	44.4	54.4	43.1	58.9	83.5	90.3	64.7	74.8	67.3	79.1	51.7	66.6	0.0	32.7	49.0	48.4
Suriname	35 to 39	39.3	62.3	49.5	56.5	14.0	37.5	35.0	49.7	53.0	56.3	25.1	42.0	65.2	76.4	59.6	61.8	46.8	61.7	13.8	31.9	67.7	73.1	27.4	51.1
Sweden	35 to 39	33.6	47.6	81.3	100.0	77.4	100.0	32.1	42.6	87.5	100.0	82.8	100.0	53.2	61.2	67.8	94.7	69.5	93.3	44.3	59.1	85.5	100.0	80.0	100.0
Switzerland	35 to 39	79.0	100.0	79.5	92.2	77.3	91.7	83.2	100.0	84.6	96.3	82.4	98.8	68.0	94.0	68.1	89.7	76.0	90.7	80.5	100.0	86.4	98.4	78.5	94.8
Syrian Arab Republic	35 to 39	78.1	91.8	64.0	80.2	56.5	72.6	81.4	97.9	67.6	76.2	58.0	65.6	71.4	90.0	86.2	97.7	87.2	99.2	80.3	96.3	34.9	60.2	48.1	63.2
Taiwan (Province of China)	35 to 39	59.9	77.0	83.2	92.2	58.2	69.0	57.5	67.4	83.4	92.0	65.2	71.4	86.9	98.9	91.3	94.4	85.5	85.2	33.4	55.9	80.6	90.8	65.0	80.1
Tajikistan	35 to 39	70.6	80.7	56.4	75.2	48.5	60.8	70.8	77.9	57.3	70.1	53.9	58.4	88.3	89.6	68.4	82.8	73.0	76.3	70.0	84.3	47.3	74.2	25.9	55.7
Thailand	35 to 39	52.2	68.9	71.2	73.9	48.5	20.3	49.2	60.2	74.2	73.6	63.1	38.2	70.1	78.7	74.8	75.5	54.2	39.0	30.7	62.5	79.3	79.0	67.9	44.0
Timor-Leste	35 to 39	59.9	45.7	52.2	60.1	26.6	29.4	64.4	46.6	53.4	60.3	36.1	39.1	63.5	54.1	70.8	73.6	57.6	61.2	70.8	58.4	72.0	76.0	59.5	48.6
Togo	35 to 39	39.5	45.5	62.0	69.4	62.9	61.4	37.0	42.9	58.4	64.4	62.8	60.4	62.9	66.5	75.3	79.8	74.6	73.1	61.7	59.3	82.3	86.3	64.9	60.0
Tokelau	35 to 39	61.5	65.1	53.0	61.0	21.9	39.2	54.6	57.1	61.6	62.7	38.8	45.4	74.5	75.8	58.0	66.0	50.2	66.8	70.3	70.7	78.7	82.6	31.8	45.2
Tonga	35 to 39	40.8	51.8	72.8	77.5	61.4	64.7	46.2	49.0	71.7	73.1	63.2	64.9	53.6	65.7	79.2	83.0	77.9	82.3	58.0	64.9	89.9	90.6	65.8	68.4
Trinidad and Tobago	35 to 39	67.8	71.8	69.7	73.1	50.5	44.9	63.8	65.2	68.4	71.7	52.0	40.1	78.3	82.4	64.9	60.2	49.3	41.8	77.8	79.3	81.8	82.5	40.8	48.4
Tropical Latin America	35 to 39	60.7	60.4	36.2	61.9	9.1	49.6	56.3	51.7	52.8	68.4	26.8	52.8	57.5	51.2	29.7	51.7	22.4	52.7	57.9	62.9	55.1	76.5	27.6	67.0
Tunisia	35 to 39	68.8	79.1	68.5	81.6	70.1	76.4	61.8	71.6	66.4	77.2	67.0	72.4	82.5	90.0	82.3	90.5	84.0	89.8	50.3	60.3	48.0	67.6	64.7	61.8
Turkey	35 to 39	64.7	85.3	76.1	89.7	53.7	79.8	63.9	80.1	76.0	86.4	60.7	77.4	57.6	81.5	69.7	87.0	48.3	76.5	57.3	82.1	69.2	87.1	54.4	80.5
Turkmenistan	35 to 39	60.6	60.5	68.8	76.4	51.1	45.6	57.8	54.9	68.4	73.3	56.5	49.5	62.7	59.9	69.6	72.6	58.7	54.5	42.3	53.8	56.3	71.3	39.2	44.4
Tuvalu	35 to 39	24.6	38.2	40.8	54.1	3.0	24.7	35.4	44.2	53.3	59.8	30.4	42.7	40.4	55.8	47.9	59.4	34.5	55.7	44.6	45.5	74.5	79.3	11.4	22.5
Uganda	35 to 39	62.0	68.5	73.5	78.5	51.6	58.6	52.3	57.4	65.5	69.5	51.8	56.7	76.0	80.8	83.8	87.4	72.5	77.3	86.6	87.6	90.3	92.2	86.4	85.9
Ukraine	35 to 39	71.2	66.2	81.7	82.0	61.0	53.2	66.4	62.4	79.5	79.6	60.0	54.5	71.6	65.7	79.4	78.9	67.0	59.6	66.9	63.7	83.4	84.6	56.1	51.1
United Arab Emirates	35 to 39	80.2	86.7	85.0	88.4	78.7	86.8	76.1	84.9	84.0	87.5	76.0	86.2	81.9	91.2	82.1	89.4	82.6	92.2	74.7	89.5	83.3	92.9	74.7	88.7
United Kingdom	35 to 39	74.3	85.6	77.4	87.7	71.4	83.1	81.8	88.5	85.5	91.3	81.6	87.0	56.0	79.4	57.4	81.2	56.1	78.3	81.1	93.8	88.2	97.1	77.7	91.5
United Republic of Tanzania	35 to 39	59.0	72.8	66.7	78.2	52.5	68.0	53.8	63.0	62.4	70.9	56.3	64.1	75.5	82.1	80.1	84.7	73.8	81.1	84.1	85.3	87.5	90.9	84.8	83.1
United States Virgin Islands	35 to 39	55.0	70.7	67.0	85.8	37.2	46.0	52.1	63.9	68.7	86.7	43.4	41.4	61.0	76.7	66.4	87.7	53.0	60.8	63.1	77.0	79.1	93.8	48.6	57.2
United States of America	35 to 39	86.8	95.6	88.5	96.2	84.4	93.7	79.7	86.8	86.4	91.3	76.8	84.5	65.1	84.0	68.7	87.6	60.4	78.4	92.4	95.3	94.3	95.2	92.3	96.6
Uruguay	35 to 39	55.0	73.1	59.1	75.4	51.7	71.3	63.7	80.9	72.9	86.0	62.4	78.6	41.6	64.6	40.9	65.4	44.7	65.2	71.5	84.5	76.3	88.2	73.6	84.6
Uzbekistan	35 to 39	56.1	72.0	64.5	81.4	47.1	62.1	45.4	69.5	58.5	79.7	44.7	66.0	78.7	80.7	84.6	89.1	75.3	75.5	51.0	62.0	65.0	79.1	45.9	50.2
Vanuatu	35 to 39	39.8	45.1	63.7	63.7	16.4	26.9	44.0	47.9	66.3	65.7	35.7	43.0	51.0	58.0	65.7	65.6	42.2	54.5	53.4	59.1	86.9	87.4	26.0	34.6
Venezuela (Bolivarian Republic of)	35 to 39	58.2	63.0	65.1	72.2	48.8	47.3	59.2	61.0	70.4	76.2	55.1	48.5	65.2	67.3	66.5	70.9	64.5	61.7	67.7	75.3	75.7	81.5	65.7	72.9
Viet Nam	35 to 39	40.3	55.4	58.2	74.7	22.4	38.4	41.5	52.7	59.2	73.1	38.4	46.1	64.5	75.9	75.9	85.7	55.7	68.9	55.9	61.9	72.9	81.4	45.9	50.2
Yemen	35 to 39	38.9	53.7	44.7	59.3	33.5	48.6	34.5	44.4	50.0	56.1	34.0	46.3	67.5	73.3	72.5	77.0	62.9	70.4	16.5	22.3	12.2	33.8	40.6	28.7
Zambia	35 to 39	48.7	55.8	50.9	63.6	48.6	48.5	45.2	46.8	51.5	56.3	53.0	50.1	69.6	73.1	66.5	74.7	73.2	73.3	79.3	79.1	81.0	84.7	85.1	78.7
Zimbabwe	35 to 39	78.6	56.4	83.9	68.8	71.8	40.1	64.8	26.8	72.4	46.3	65.4	25.9	83.8	63.8	86.1	68.5	82.7	62.6	89.4	73.1	93.6	83.3	86.2	64.8
Afghanistan	40 to 44	36.6	43.2	41.5	41.3	29.9	45.9	47.8	43.9	64.0	54.3	28.3	40.1	61.7	63.3	65.6	64.2	55.4	63.7	22.0	16.6	22.0	18.6	39.8	25.4

Albania	40 to 44	71.4	83.1	73.5	88.4	69.5	76.3	68.9	75.2	74.4	83.6	66.6	68.1	81.0	89.6	80.1	90.7	82.1	88.2	87.6	91.3	91.3	94.9	85.2	86.6
Algeria	40 to 44	66.1	75.6	61.1	71.4	72.4	80.6	66.1	70.4	69.2	71.6	68.8	74.7	77.3	84.6	74.1	80.9	82.3	89.7	51.1	60.0	47.7	58.7	63.9	69.7
American Samoa	40 to 44	50.5	54.3	65.2	62.1	40.2	47.7	54.2	53.6	70.9	66.2	43.6	45.7	71.2	73.0	75.7	73.4	70.1	74.0	78.7	79.6	81.6	80.2	79.5	83.9
Andorra	40 to 44	79.5	91.3	78.6	90.1	80.5	92.4	83.3	96.6	86.0	95.4	81.8	96.9	71.1	86.0	63.0	81.5	79.3	91.4	84.8	93.4	89.6	95.4	83.2	93.3
Angola	40 to 44	51.8	61.6	59.3	68.4	44.8	54.3	52.3	55.2	64.3	66.4	44.9	48.2	70.8	75.0	71.8	76.5	72.1	75.5	75.4	77.6	81.7	84.3	73.1	73.2
Antigua and Barbuda	40 to 44	49.3	78.3	60.4	79.9	35.0	75.9	47.0	75.5	65.8	80.1	29.6	72.4	68.3	82.1	67.5	81.0	68.9	83.5	61.8	85.7	73.7	88.3	49.2	85.6
Argentina	40 to 44	50.4	77.4	56.9	80.8	44.9	74.4	64.6	81.2	75.3	87.2	56.8	75.9	48.9	76.2	49.7	76.1	48.2	77.5	74.6	89.3	80.3	92.2	73.5	89.5
Armenia	40 to 44	73.2	82.1	80.1	92.5	66.2	71.4	66.6	79.4	78.5	92.5	56.2	64.0	78.2	84.1	86.9	96.0	72.9	77.1	69.1	74.0	80.2	89.5	60.4	57.2
Australia	40 to 44	80.4	88.6	84.0	89.3	77.5	88.2	84.5	90.2	90.8	94.5	79.1	85.5	66.2	79.2	68.2	79.9	64.9	79.1	85.8	93.7	91.0	95.6	83.4	94.0
Austria	40 to 44	75.7	90.0	79.2	90.2	72.9	90.1	76.3	96.6	83.3	96.0	71.2	96.1	74.3	87.8	72.7	85.5	77.0	91.1	71.0	92.2	81.3	94.9	62.6	92.2
Azerbaijan	40 to 44	60.3	78.0	70.5	84.9	49.4	70.7	60.2	74.3	72.7	82.3	50.1	67.6	84.0	92.2	89.0	96.5	81.4	89.6	64.2	82.4	79.8	90.3	46.2	74.0
Bahamas	40 to 44	43.3	59.5	52.7	69.6	32.2	48.0	44.1	57.9	60.8	72.5	31.1	45.8	64.1	67.1	62.2	70.2	65.9	63.4	56.1	70.3	66.7	81.0	48.1	59.6
Bahrain	40 to 44	81.8	90.8	85.8	92.2	80.4	90.9	77.9	85.4	85.1	88.8	73.6	84.2	84.4	91.7	85.3	91.4	85.1	92.4	77.7	81.0	81.7	82.5	76.0	80.8
Bangladesh	40 to 44	25.7	54.7	38.0	63.8	19.1	48.6	41.1	59.4	57.4	71.5	32.0	51.6	42.3	65.9	48.7	66.7	43.3	67.9	51.4	68.5	61.0	78.3	47.7	62.4
Barbados	40 to 44	55.6	72.0	61.7	77.1	48.6	65.4	55.8	70.2	67.5	79.6	47.2	61.4	61.0	71.8	61.5	73.5	60.6	70.3	56.8	76.9	66.5	82.9	50.5	72.9
Belarus	40 to 44	57.7	64.9	69.3	79.2	48.1	52.2	59.3	61.8	68.6	75.3	53.9	51.7	61.7	60.8	75.0	78.8	54.0	50.4	50.4	67.1	75.8	86.3	25.1	47.0
Belgium	40 to 44	68.7	86.1	71.4	86.5	66.6	85.7	68.0	90.4	75.0	91.6	64.0	88.8	70.8	78.5	68.1	75.7	74.5	82.5	73.1	91.2	81.3	94.9	68.9	89.2
Belize	40 to 44	61.3	62.8	60.7	70.6	62.4	53.7	60.0	56.9	64.2	69.7	61.3	47.1	71.0	74.9	71.0	75.6	71.0	73.9	77.1	74.0	80.7	82.9	77.1	65.3
Benin	40 to 44	61.5	68.0	63.7	71.8	60.0	64.5	60.3	64.7	66.0	71.3	59.1	61.6	75.9	79.5	76.9	81.0	76.1	79.3	75.3	76.3	85.4	87.3	65.0	63.3
Bermuda	40 to 44	71.0	89.2	80.3	93.8	59.7	82.3	71.8	90.0	83.9	95.8	58.2	80.8	77.2	90.7	82.3	95.6	71.3	85.0	66.2	89.1	82.6	94.9	47.4	82.8
Bhutan	40 to 44	57.4	70.9	56.6	72.5	59.2	70.1	60.3	68.2	67.4	74.7	57.6	64.6	65.5	74.1	61.1	71.7	70.4	77.4	72.3	80.0	72.2	83.3	78.5	80.4
Bolivia (Plurinational State of)	40 to 44	38.2	61.3	41.0	61.4	35.2	61.6	46.9	60.2	58.5	68.3	40.3	56.2	48.6	61.6	44.2	55.9	53.1	68.4	55.5	77.7	64.9	82.6	49.3	75.0
Bosnia and Herzegovina	40 to 44	71.5	87.8	76.4	91.6	67.4	84.3	64.6	83.6	76.2	92.5	55.9	75.2	56.9	84.3	57.1	85.7	57.2	83.0	68.4	82.0	80.1	89.9	60.2	76.2
Botswana	40 to 44	53.1	71.9	63.1	79.1	44.7	65.3	50.4	68.4	59.7	77.2	46.2	62.6	74.5	84.5	77.9	86.7	73.6	84.3	76.3	83.7	85.1	91.1	68.8	77.5
Brazil	40 to 44	26.0	55.5	34.6	58.9	17.4	52.1	40.6	58.7	58.4	71.0	26.7	48.6	30.0	50.7	30.3	46.8	27.2	55.1	39.2	69.9	57.9	78.0	25.0	65.0
Brunei Darussalam	40 to 44	57.3	65.1	55.5	69.6	59.2	62.6	68.7	70.0	73.5	79.8	66.6	62.9	43.6	56.0	32.3	53.6	53.6	60.0	71.6	80.7	73.3	84.6	74.8	81.3
Bulgaria	40 to 44	47.5	64.7	62.7	76.2	35.5	55.0	55.9	64.6	71.6	79.5	44.5	53.3	59.9	70.1	64.9	72.6	56.8	68.9	46.0	53.5	69.8	76.2	24.1	32.7
Burkina Faso	40 to 44	56.0	61.8	60.5	70.6	51.3	53.1	53.1	58.4	63.8	71.3	46.8	48.6	75.2	78.2	78.6	83.8	73.0	75.1	77.1	77.4	85.9	88.7	67.7	64.6
Burundi	40 to 44	36.9	54.4	51.0	64.2	21.4	46.3	45.3	51.0	63.9	66.3	29.5	40.5	58.4	73.0	64.3	76.9	56.6	73.0	66.0	78.9	71.6	83.2	67.0	78.8
Cabo Verde	40 to 44	63.8	75.5	70.6	81.9	54.0	70.8	64.3	72.7	74.0	80.5	56.1	67.7	75.9	84.5	80.4	88.1	70.7	82.9	65.8	72.0	84.4	89.5	33.8	54.7
Cambodia	40 to 44	29.4	51.8	31.2	56.6	28.2	47.4	42.3	58.2	51.8	66.9	40.3	53.5	59.6	73.5	59.4	74.1	60.3	74.1	54.2	68.4	61.5	74.7	53.6	66.8
Cameroon	40 to 44	52.4	57.6	57.4	64.8	48.3	51.7	53.5	56.6	64.3	67.8	47.1	49.7	71.3	71.8	74.2	75.8	70.4	70.5	72.7	68.8	84.2	84.5	60.9	52.5
Canada	40 to 44	86.1	90.5	87.0	91.2	85.3	90.0	90.1	93.6	92.5	94.9	87.2	91.0	75.4	88.1	77.2	89.6	73.2	86.0	90.3	93.0	93.3	95.0	89.8	93.7
Central African Republic	40 to 44	35.3	37.9	49.1	52.9	22.1	24.0	41.3	38.1	58.3	56.9	30.1	25.9	58.8	62.1	63.8	67.6	57.6	60.7	65.5	69.3	74.7	79.3	59.2	61.3
Chad	40 to 44	59.5	58.9	63.2	65.8	56.4	52.5	59.1	57.7	67.4	68.5	55.1	50.8	72.7	70.7	74.6	74.1	72.1	69.4	73.5	69.6	84.4	85.1	61.4	51.3
Chile	40 to 44	65.1	80.7	69.3	84.7	61.1	76.2	72.1	86.2	81.1	92.4	63.5	78.5	63.9	73.8	63.1	75.8	65.3	71.9	74.8	87.6	80.7	91.6	73.5	86.2
China	40 to 44	38.4	60.5	50.9	76.3	26.6	48.7	53.2	61.4	68.3	77.9	41.5	50.5	47.3	78.3	48.2	84.0	47.9	75.1	36.1	57.2	60.9	79.0	8.6	37.0
Colombia	40 to 44	60.8	76.6	64.0	80.1	56.7	71.0	64.0	80.0	72.9	87.4	56.4	70.3	64.6	72.3	64.2	73.1	65.2	71.6	65.8	82.6	74.8	88.1	59.3	76.9
Comoros	40 to 44	52.6	66.4	58.0	71.6	47.0	61.4	52.9	60.3	63.5	71.0	47.0	52.8	72.0	79.4	71.6	80.3	74.0	80.4	75.5	82.5	80.3	87.0	76.0	81.6
Congo	40 to 44	45.2	57.1	53.6	59.0	36.9	55.9	46.9	48.5	58.9	56.6	40.3	47.1	66.2	72.7	66.6	68.6	67.8	77.4	69.1	73.4	78.4	79.3	61.9	71.8
Cook Islands	40 to 44	55.6	74.0	63.3	81.7	48.2	66.7	58.1	69.6	70.1	81.3	49.7	60.2	70.5	87.0	73.1	89.1	69.5	85.8	77.9	87.0	78.7	88.0	82.4	89.1
Costa Rica	40 to 44	78.7	82.6	80.9	86.0	75.4	76.6	80.0	84.7	85.3	92.0	74.0	74.8	78.3	76.7	78.2	78.1	78.8	74.7	81.6	88.4	85.6	91.4	79.7	85.9
Coted'Ivoire	40 to 44	60.0	61.8	67.9	69.7	55.8	56.8	59.3	58.3	69.9	69.1	53.6	52.3	71.1	73.8	76.2	77.9	70.0	73.0	66.6	63.3	84.6	85.7	52.4	42.7
Croatia	40 to 44	60.5	88.0	73.1	96.4	49.8	80.0	64.8	86.7	79.4	97.8	53.1	76.0	64.5	86.5	67.6	88.4	61.9	84.8	62.5	87.5	79.3	94.8	48.1	81.0
Cuba	40 to 44	57.2	71.6	61.3	79.6	52.5	62.5	59.1	65.3	69.8	79.7	51.1	52.0	67.6	82.5	65.7	83.2	70.0	81.7	60.3	72.5	69.5	83.2	54.6	62.6
Cyprus	40 to 44	73.4	92.8	73.6	93.5	73.9	91.1	81.2	95.1	84.7	95.2	78.9	93.4	68.9	87.2	63.7	86.5	74.7	88.6	81.0	95.2	84.7	97.1	81.2	92.9
Czechia	40 to 44	65.3	90.4	76.5	95.4	55.3	84.8	67.0	90.9	82.3	97.9	54.2	83.0	64.0	88.0	66.3	88.2	61.9	87.8	57.4	88.2	77.4	93.5	39.8	83.5
Democratic People's Republic of Korea	40 to 44	45.0	38.1	53.0	50.8	38.1	28.5	55.5	44.5	67.3	61.5	47.9	33.7	62.2	66.9	62.0	68.4	63.4	67.4	49.5	43.1	63.3	63.4	39.2	27.0

Democratic Republic of the Congo	40 to 44	58.1	59.5	63.4	67.8	52.9	52.2	58.9	56.9	67.5	69.0	54.3	49.0	75.0	75.8	75.4	77.7	76.3	76.6	75.9	79.1	81.6	85.6	74.1	75.4
Denmark	40 to 44	69.4	88.1	73.7	89.3	65.7	86.9	72.7	93.7	83.1	95.9	64.5	90.5	66.4	85.4	64.2	83.6	70.0	88.4	71.9	92.0	83.7	94.9	63.8	91.6
Djibouti	40 to 44	54.4	65.2	64.0	74.5	47.3	58.2	53.3	57.9	65.3	70.5	46.7	50.5	71.8	76.9	76.7	81.8	71.4	76.3	79.3	81.1	85.1	88.9	77.1	75.8
Dominica	40 to 44	59.9	63.3	64.9	70.5	54.2	54.7	58.4	56.8	68.5	70.4	51.3	45.5	70.8	72.8	72.7	74.9	69.4	71.1	68.3	67.9	75.4	78.1	64.2	58.0
Dominican Republic	40 to 44	48.2	55.8	54.0	63.4	41.2	47.8	49.7	55.1	63.1	68.7	39.6	44.6	61.7	67.9	60.2	66.1	62.4	70.4	58.1	62.2	70.2	75.4	47.2	50.7
Ecuador	40 to 44	55.0	74.6	61.6	78.8	47.6	69.8	52.9	74.8	66.2	83.0	43.4	67.7	69.9	72.1	69.0	73.3	71.6	71.6	54.0	83.9	69.3	91.0	37.8	76.5
Egypt	40 to 44	53.2	69.8	60.6	73.9	44.3	66.2	46.6	62.8	65.1	74.2	29.4	54.2	70.5	84.7	76.3	86.4	64.3	83.6	27.9	41.2	37.4	54.5	27.0	33.4
El Salvador	40 to 44	57.2	69.2	63.0	76.6	49.6	54.8	63.2	64.1	72.7	78.7	55.8	45.3	66.9	73.6	67.3	75.1	66.2	70.0	63.3	74.9	76.0	86.1	48.0	55.5
Equatorial Guinea	40 to 44	41.9	68.3	52.9	72.3	28.3	64.0	42.9	56.3	58.4	66.2	32.1	50.8	64.3	82.1	69.2	80.8	61.9	84.4	70.3	78.1	79.6	84.3	61.6	73.6
Eritrea	40 to 44	24.7	48.0	40.4	61.4	10.0	36.7	31.8	46.3	50.9	63.1	20.0	35.8	56.9	69.1	65.7	75.6	53.0	67.5	69.4	76.6	75.9	84.1	68.1	72.3
Estonia	40 to 44	64.3	84.3	73.8	92.0	57.3	75.7	64.8	78.3	76.1	92.3	56.6	63.4	70.2	81.4	81.4	93.0	62.7	74.5	57.6	86.3	72.8	93.0	47.8	80.0
Eswatini	40 to 44	51.7	44.4	65.2	63.0	35.1	28.1	44.4	36.5	62.3	55.2	31.3	26.2	76.2	72.0	79.8	76.6	74.3	70.2	80.5	72.6	87.3	84.2	73.3	60.7
Ethiopia	40 to 44	44.4	66.8	55.3	73.4	33.8	60.6	53.1	61.8	67.5	72.5	41.7	54.7	71.2	81.9	76.0	84.6	70.4	81.9	80.4	87.1	83.4	89.2	82.9	88.3
Fiji	40 to 44	55.9	65.2	62.4	68.0	49.4	62.8	53.0	56.1	66.4	66.2	43.6	50.7	73.9	78.6	76.7	79.3	71.3	79.1	80.7	83.4	81.8	83.4	83.8	87.4
Finland	40 to 44	66.5	87.2	74.0	89.6	61.7	85.4	71.2	91.4	83.6	94.7	62.9	88.2	64.1	82.3	66.8	83.3	63.7	82.8	63.5	92.4	79.7	95.9	53.8	91.2
France	40 to 44	73.2	85.7	82.0	88.0	65.3	83.9	70.6	92.7	82.5	95.7	61.4	88.9	76.0	80.9	77.5	80.7	76.0	82.3	78.6	90.2	90.0	94.6	67.7	88.8
Gabon	40 to 44	57.2	66.8	68.1	74.1	48.8	60.0	57.1	59.9	70.0	70.9	49.2	53.1	76.0	80.1	79.7	81.7	75.6	80.5	77.8	79.6	85.1	86.5	73.7	74.8
Gambia	40 to 44	57.9	57.4	64.1	63.2	54.1	52.6	53.5	51.2	63.4	62.0	48.9	45.6	72.8	70.9	76.1	73.1	72.4	70.4	65.5	62.1	84.1	82.0	48.8	39.7
Georgia	40 to 44	63.8	68.3	79.4	86.9	48.7	54.1	68.6	71.4	81.8	87.7	58.3	61.1	74.0	69.5	85.1	81.6	68.1	65.5	73.3	63.7	85.6	87.7	55.2	34.7
Germany	40 to 44	75.1	87.6	78.0	90.2	72.7	84.9	76.6	91.7	82.6	95.6	72.5	86.7	68.3	83.6	65.1	82.5	72.6	85.7	75.1	88.8	85.5	94.0	67.8	85.4
Ghana	40 to 44	51.1	60.7	56.1	68.9	46.7	53.0	48.5	53.5	59.7	65.5	42.7	46.3	63.7	69.3	65.8	73.7	63.4	67.3	61.3	63.6	80.4	84.9	38.0	37.4
Greece	40 to 44	73.2	82.2	76.6	85.3	70.4	79.3	74.6	81.6	80.6	87.1	70.8	76.9	79.2	82.9	76.7	82.0	82.3	84.7	73.3	88.6	81.5	92.0	67.8	87.1
Greenland	40 to 44	51.5	70.4	53.8	72.7	50.9	69.0	58.0	69.8	73.4	80.3	47.8	62.1	0.0	51.9	0.0	51.2	0.0	52.8	74.0	89.0	84.1	93.0	70.7	87.8
Grenada	40 to 44	46.8	67.3	55.0	71.1	37.9	63.5	50.7	65.2	64.7	73.9	40.3	59.0	59.3	70.9	59.6	69.9	58.9	72.4	39.2	72.8	55.3	79.6	25.5	69.1
Guam	40 to 44	71.5	71.9	80.1	82.4	64.5	62.5	69.7	66.3	81.8	81.5	60.0	53.8	78.7	80.3	82.9	86.3	77.2	77.3	83.9	86.7	86.6	89.4	84.0	86.1
Guatemala	40 to 44	61.1	70.3	61.1	74.5	61.8	63.9	67.3	69.6	72.0	77.6	67.0	62.4	81.5	79.8	80.3	80.4	83.9	79.5	62.6	81.1	69.4	86.3	59.1	75.3
Guinea	40 to 44	57.1	60.8	55.8	64.6	60.3	57.3	58.2	60.2	63.4	68.1	59.7	56.5	74.3	74.4	71.9	75.0	77.0	75.0	70.7	67.7	80.7	83.9	61.2	47.2
Guinea-Bissau	40 to 44	33.4	40.9	39.2	48.3	27.2	33.1	38.0	39.5	51.0	53.7	31.8	31.4	51.4	56.2	52.8	57.4	50.9	56.2	53.1	52.3	73.0	76.0	26.9	20.8
Guyana	40 to 44	23.4	45.4	32.1	55.3	15.5	34.2	32.7	46.9	47.4	63.0	25.0	33.4	53.4	58.2	57.8	63.4	49.7	52.6	29.9	47.5	51.8	66.6	10.7	27.8
Haiti	40 to 44	0.0	21.1	0.0	25.0	0.0	15.4	25.8	32.4	42.8	50.6	13.7	15.3	17.6	35.5	7.1	28.0	27.6	43.1	0.0	28.6	5.7	38.5	12.3	28.1
Honduras	40 to 44	24.8	41.4	24.2	37.8	25.4	47.2	24.8	32.5	40.3	44.2	18.9	32.1	37.8	40.8	29.0	28.6	46.0	55.0	33.9	59.9	42.9	64.2	32.0	62.1
Hungary	40 to 44	55.8	83.0	66.3	91.2	46.7	73.8	59.5	81.4	73.5	93.7	49.5	69.9	53.8	80.7	52.0	81.3	55.3	80.4	44.1	78.2	70.4	90.8	20.2	63.4
Iceland	40 to 44	79.5	90.4	78.5	91.1	80.9	89.7	84.2	95.6	85.5	96.0	84.3	93.9	75.7	86.1	69.1	85.4	83.2	87.5	82.7	94.2	87.2	96.6	82.0	93.9
India	40 to 44	56.7	63.7	62.0	70.7	52.6	58.2	60.6	65.0	71.5	75.4	52.5	57.4	63.7	67.2	66.2	69.8	64.0	67.5	72.6	78.1	76.9	83.6	72.8	75.6
Indonesia	40 to 44	43.4	47.3	50.1	55.4	36.0	39.1	51.3	49.3	64.0	62.8	42.2	40.4	62.6	67.4	64.1	67.9	62.0	68.3	61.4	59.4	71.5	72.7	53.7	47.4
Iran (Islamic Republic of)	40 to 44	73.7	87.1	75.6	90.4	71.4	83.8	68.6	80.9	76.0	87.4	63.8	75.8	81.8	93.2	82.4	94.8	81.5	91.6	37.7	63.8	48.9	74.2	32.0	55.7
Iraq	40 to 44	53.6	64.6	61.8	68.8	45.9	61.1	56.3	64.2	69.6	72.0	47.2	60.2	72.8	79.9	75.4	78.8	70.1	81.5	30.1	41.8	43.0	51.9	23.2	37.5
Ireland	40 to 44	76.2	91.0	78.0	90.7	74.8	90.9	84.6	100.0	88.6	98.7	80.8	99.3	70.2	84.1	68.2	82.4	73.4	86.6	79.2	94.5	87.0	96.2	74.2	94.3
Israel	40 to 44	75.0	91.0	75.9	92.7	74.8	89.6	73.4	92.4	77.7	96.4	71.5	87.9	82.0	92.4	79.3	92.7	85.8	93.1	80.2	94.3	84.1	96.4	81.3	94.7
Italy	40 to 44	74.2	91.2	78.2	94.8	70.7	87.4	75.6	88.0	82.7	94.1	70.2	82.1	70.3	86.4	70.5	86.0	71.2	87.4	78.8	95.7	85.9	97.9	74.8	94.0
Jamaica	40 to 44	51.2	61.6	54.7	67.3	47.4	53.8	55.6	61.2	65.7	74.2	49.4	47.7	61.8	72.8	60.7	70.7	62.8	75.4	48.6	65.0	56.8	72.8	45.6	59.3
Japan	40 to 44	67.7	77.2	75.6	84.6	62.1	72.8	75.3	82.7	86.1	92.8	66.2	75.7	57.9	72.3	59.9	75.8	58.4	71.9	78.8	88.6	85.0	93.8	76.8	87.6
Jordan	40 to 44	68.8	86.4	66.0	89.7	72.6	84.2	59.3	80.0	66.3	86.0	58.1	75.1	91.1	100.0	89.9	100.0	93.1	100.0	46.3	73.2	49.0	79.0	53.0	70.9
Kazakhstan	40 to 44	66.4	70.2	74.4	80.2	58.7	59.9	65.0	65.6	75.7	79.6	56.7	53.5	64.8	72.4	70.1	77.0	62.6	71.0	57.4	70.0	75.7	81.6	39.4	58.3
Kenya	40 to 44	70.3	68.9	73.8	73.8	67.6	64.8	67.0	62.9	74.3	71.5	62.9	58.2	82.2	81.4	82.8	81.9	83.4	82.6	87.8	85.7	89.4	88.8	89.7	85.8
Kiribati	40 to 44	51.9	50.9	68.9	69.0	41.9	39.6	65.1	60.6	77.8	76.0	57.5	51.2	66.8	71.2	82.9	82.7	55.6	65.4	79.9	77.7	85.3	84.9	77.6	73.4
Kuwait	40 to 44	89.0	92.3	88.5	98.4	90.1	87.6	81.3	85.7	85.1	94.2	79.6	78.6	90.2	98.3	87.0	99.6	93.0	97.4	82.0	81.8	82.6	90.1	83.0	75.2
Kyrgyzstan	40 to 44	51.0	59.9	60.7	75.5	42.0	45.9	56.3	64.7	67.4	80.3	49.3	52.8	58.6	63.9	63.1	72.8	56.4	59.6	47.2	38.4	66.1	71.7	28.6	0.0

Lao People's Democratic Republic	40 to 44	5.2	39.0	9.1	48.3	2.0	31.1	26.2	47.0	40.8	60.6	20.0	38.7	37.3	65.5	34.5	67.2	40.7	65.5	18.0	50.0	30.1	67.0	15.8	35.2
Latvia	40 to 44	61.7	73.5	71.8	85.3	52.4	62.7	59.5	63.8	72.8	80.7	49.1	48.8	70.8	75.5	77.7	85.2	67.0	70.2	54.5	71.7	74.3	87.3	36.0	56.4
Lebanon	40 to 44	51.2	89.0	58.3	92.0	44.6	86.0	43.7	82.9	65.4	89.1	23.9	76.5	57.7	89.6	62.2	91.5	51.6	87.2	45.1	86.2	47.7	89.6	48.8	84.8
Lesotho	40 to 44	57.6	29.1	71.2	52.2	38.9	6.0	48.8	28.4	66.4	54.1	34.7	11.0	78.5	62.8	84.4	69.7	73.7	59.2	85.5	65.9	90.5	80.0	79.5	49.7
Liberia	40 to 44	60.8	57.7	63.4	61.7	59.6	55.0	61.3	52.5	68.4	62.6	58.4	47.6	75.7	72.3	76.2	72.9	76.9	73.5	70.7	65.1	83.6	81.8	58.1	48.1
Libya	40 to 44	78.2	75.0	71.3	72.2	86.7	78.4	71.7	65.0	72.8	67.7	76.7	68.8	84.3	83.5	79.6	77.6	89.4	90.2	62.6	48.3	59.8	55.0	72.2	47.7
Lithuania	40 to 44	64.3	76.1	73.1	86.3	55.7	66.1	60.0	71.5	72.5	85.6	50.7	59.5	62.9	69.9	69.6	80.7	58.8	62.8	58.3	76.6	75.3	90.7	42.8	62.4
Luxembourg	40 to 44	67.8	94.0	70.1	94.3	66.5	93.4	69.7	96.4	75.4	97.3	67.3	94.4	68.1	89.1	61.9	87.4	74.4	91.5	66.0	95.6	76.9	97.1	59.6	94.8
Madagascar	40 to 44	41.0	48.1	51.9	56.0	30.7	40.8	49.4	47.7	63.2	60.7	40.3	39.9	61.8	67.5	65.6	66.9	61.8	70.2	70.4	74.7	78.7	81.7	64.8	71.0
Malawi	40 to 44	53.1	48.7	57.5	62.5	49.4	37.2	50.4	35.1	61.7	55.8	44.1	22.4	70.1	67.6	69.8	72.8	72.2	66.8	78.4	73.7	82.1	83.2	79.3	66.1
Malaysia	40 to 44	61.6	68.6	67.5	76.6	57.0	62.4	67.6	68.8	75.6	78.5	62.6	62.5	77.7	84.2	80.3	85.9	76.3	83.5	66.8	73.0	75.2	82.2	62.8	66.8
Maldives	40 to 44	38.7	73.3	37.9	79.6	40.3	71.7	49.3	73.5	54.7	77.9	49.1	71.6	65.6	88.5	63.8	90.0	68.7	88.9	53.1	78.3	58.2	85.6	55.6	76.0
Mali	40 to 44	53.9	63.1	46.3	58.5	65.5	69.6	51.9	56.3	54.3	57.7	60.9	63.0	71.8	76.9	61.0	68.5	80.6	83.4	74.2	75.2	76.5	80.6	78.3	72.0
Malta	40 to 44	79.0	88.7	77.3	88.1	81.4	89.1	81.1	88.4	80.8	89.5	84.4	87.9	84.5	86.9	80.6	84.8	89.7	89.6	76.4	90.8	80.1	91.3	77.4	91.9
Marshall Islands	40 to 44	32.5	35.2	47.9	48.0	20.9	24.6	45.9	45.0	63.3	60.9	34.6	35.0	52.3	59.1	62.7	65.2	47.2	56.7	73.3	71.7	74.3	73.6	77.6	75.9
Mauritania	40 to 44	57.1	70.0	59.6	69.8	55.4	71.4	54.7	62.6	61.7	67.2	53.4	63.8	71.6	80.6	72.1	79.4	72.5	82.3	66.3	74.6	81.9	84.5	48.4	63.4
Mauritius	40 to 44	43.7	58.9	62.4	69.7	27.8	49.2	51.5	62.0	71.7	74.9	36.0	53.4	60.9	68.6	73.6	72.0	49.8	66.5	27.7	55.5	58.0	70.8	0.1	41.2
Mexico	40 to 44	75.4	78.4	78.1	84.7	70.6	68.9	73.6	74.9	80.6	86.0	65.8	62.5	79.3	74.5	78.7	78.1	80.1	70.3	72.5	83.1	80.7	89.7	63.9	74.8
Micronesia (Federated States of)	40 to 44	24.0	34.6	39.7	48.6	13.6	23.9	41.7	43.4	58.8	59.9	31.6	33.5	44.5	59.6	57.7	66.7	38.6	56.7	66.9	69.8	67.5	72.1	72.0	72.9
Monaco	40 to 44	67.3	82.6	72.7	86.4	62.8	77.9	65.9	80.0	78.2	89.6	57.1	70.5	60.3	78.9	57.2	76.5	64.0	82.2	68.3	83.3	79.1	89.9	61.3	77.8
Mongolia	40 to 44	48.2	58.4	50.5	71.8	46.1	46.2	59.3	67.2	67.6	79.3	54.8	57.0	53.8	50.3	49.9	57.7	58.0	47.0	85.0	82.7	89.4	92.6	81.0	71.9
Montenegro	40 to 44	56.1	70.4	63.8	77.8	48.8	60.9	62.8	67.5	72.6	79.2	56.1	54.5	81.3	90.2	80.2	89.9	82.3	90.1	84.2	89.8	90.9	94.3	77.5	84.4
Morocco	40 to 44	56.4	67.4	51.7	60.4	62.6	76.6	55.0	61.2	60.9	62.0	56.6	69.7	71.5	78.8	68.2	72.6	76.1	87.1	30.9	41.0	30.8	36.8	42.4	60.1
Mozambique	40 to 44	44.5	32.8	55.0	58.8	34.7	14.1	43.1	29.5	58.4	58.2	33.4	12.6	65.0	54.8	70.5	69.6	63.6	49.7	72.4	60.9	80.1	80.7	67.7	42.9
Myanmar	40 to 44	7.3	36.3	15.3	54.7	0.4	22.0	26.4	42.7	41.2	61.0	19.5	31.4	42.3	64.0	45.6	71.0	41.1	60.3	28.0	54.6	46.5	74.1	14.1	35.7
Namibia	40 to 44	57.6	59.8	66.0	73.5	49.2	46.1	53.7	49.4	64.6	66.5	47.3	38.2	79.0	81.0	80.9	85.3	79.0	78.8	78.9	79.2	86.3	88.6	72.7	69.3
Nauru	40 to 44	30.1	33.8	39.7	42.7	22.8	25.7	43.1	39.5	58.4	55.0	34.1	30.4	47.3	56.7	49.5	56.4	47.1	58.1	60.2	61.5	61.1	62.4	65.9	68.9
Nepal	40 to 44	48.5	62.7	48.0	66.7	50.1	58.0	54.7	62.4	62.9	72.4	51.6	53.8	60.5	69.9	58.2	70.5	64.1	70.5	62.4	75.8	62.1	80.9	71.1	74.0
Netherlands	40 to 44	79.4	91.3	80.9	91.7	78.4	90.7	83.2	98.2	87.1	97.0	80.1	98.1	70.6	86.0	68.2	85.5	74.2	87.9	84.0	92.9	89.8	95.5	81.4	92.1
New Zealand	40 to 44	72.7	88.3	71.0	89.7	75.0	86.3	79.6	93.7	83.6	97.1	77.1	88.4	54.9	70.3	51.7	71.0	59.0	69.6	78.2	92.9	83.4	95.5	76.4	91.2
Nicaragua	40 to 44	62.5	77.7	65.9	80.8	57.2	72.4	60.6	73.7	70.7	82.4	51.7	62.9	72.3	80.6	71.1	80.2	74.1	81.5	69.0	82.1	76.7	87.6	63.0	76.4
Niger	40 to 44	61.5	65.3	63.2	68.1	61.0	63.0	62.1	61.9	68.8	69.5	59.6	58.1	75.4	78.9	74.9	80.4	77.0	78.8	80.4	81.6	85.8	88.3	77.9	75.6
Nigeria	40 to 44	61.6	76.1	66.8	81.9	59.1	70.0	57.9	69.5	66.6	78.6	53.7	62.5	78.6	86.1	83.0	90.6	78.0	83.3	74.5	78.2	86.4	91.8	66.1	60.7
Niue	40 to 44	54.0	63.2	63.3	71.3	46.8	55.7	58.2	60.8	70.7	73.7	50.0	51.2	68.0	76.9	73.1	80.0	65.6	75.6	79.2	83.1	79.7	83.7	83.2	86.6
North Macedonia	40 to 44	59.7	75.1	66.0	81.0	52.4	69.0	63.2	74.3	74.1	83.4	53.8	66.4	58.6	72.6	53.6	70.0	63.1	75.5	58.0	76.0	71.8	85.4	44.8	67.3
Northern Mariana Islands	40 to 44	49.0	61.1	48.7	67.9	49.7	58.8	54.9	61.8	58.4	68.3	53.0	58.3	67.4	74.8	56.0	74.2	74.1	77.0	76.3	84.4	73.7	84.3	81.3	86.5
Norway	40 to 44	75.9	91.8	79.0	93.3	74.1	90.7	81.2	97.6	89.2	98.8	74.3	95.4	63.3	85.7	59.3	85.3	67.9	87.2	79.8	94.6	88.7	97.4	75.7	93.9
Oman	40 to 44	72.4	91.9	75.4	96.2	71.8	90.7	69.1	82.7	78.3	92.9	64.4	78.1	86.5	96.5	84.6	95.2	89.0	98.0	59.2	80.6	63.3	85.0	59.0	78.6
Pakistan	40 to 44	66.1	66.4	68.2	70.0	64.7	63.3	69.7	67.6	76.2	75.1	65.5	62.6	70.3	68.7	70.6	68.8	71.8	70.3	79.0	76.8	81.3	82.3	81.4	74.8
Palau	40 to 44	47.9	55.1	68.1	76.4	31.4	43.0	54.3	56.8	75.7	80.2	38.4	43.3	66.8	72.4	76.8	81.7	60.9	69.6	72.9	77.7	78.6	84.4	70.8	74.7
Palestine	40 to 44	65.8	82.7	68.2	87.3	62.7	77.1	54.2	70.6	65.6	81.7	46.5	60.4	91.0	97.2	90.9	97.8	91.7	96.7	42.0	67.3	52.5	77.4	36.5	57.9
Panama	40 to 44	66.0	74.0	70.3	78.8	61.2	67.6	66.7	73.5	75.9	84.7	59.5	62.3	75.7	73.5	74.7	71.9	77.2	75.6	64.9	81.9	74.4	86.0	56.7	79.3
Papua New Guinea	40 to 44	5.2	22.8	0.8	24.5	8.4	21.3	0.0	12.7	0.0	18.4	6.3	14.3	46.9	60.3	45.5	61.7	49.7	60.9	67.8	74.1	59.6	70.1	84.7	84.9
Paraguay	40 to 44	38.7	57.1	36.2	58.5	42.0	56.3	49.9	59.5	58.1	69.3	47.5	53.4	41.8	56.4	31.9	48.8	52.2	64.7	64.2	77.1	69.5	83.2	64.4	75.1
Peru	40 to 44	62.3	75.2	65.4	78.1	59.0	71.9	65.7	71.4	73.7	79.9	60.4	65.0	68.9	77.1	67.2	74.7	71.4	79.9	76.0	83.3	82.2	88.5	71.5	78.8
Philippines	40 to 44	35.3	46.5	55.8	61.5	13.9	33.3	22.8	49.7	52.2	67.4	0.0	37.2	70.0	69.8	74.5	72.1	66.3	69.2	59.2	61.6	75.1	75.8	40.7	48.4
Poland	40 to 44	53.0	74.2	64.0	89.2	42.5	60.3	57.7	70.3	71.8	88.7	46.8	55.8	56.5	75.3	59.5	82.2	54.2	70.5	47.3	72.0	70.6	88.9	24.5	53.2
Portugal	40 to 44	58.4	82.4	62.0	83.1	56.1	81.5	64.9	83.1	72.4	87.2	61.0	79.8	60.2	77.2	54.6	71.4	66.1	82.9	53.1	87.4	66.1	89.7	45.5	86.9

Puerto Rico	40 to 44	74.1	88.2	82.9	92.8	61.1	79.4	74.2	85.7	86.3	94.2	60.7	72.6	77.8	88.9	81.3	92.0	71.0	83.7	75.9	91.6	88.7	96.6	57.4	84.0
Qatar	40 to 44	80.6	94.4	84.7	99.4	79.9	94.0	80.6	91.2	86.9	96.7	77.2	89.2	78.8	90.2	85.6	95.5	77.2	89.0	85.3	93.5	88.6	98.0	85.4	92.5
Republic of Korea	40 to 44	58.0	80.8	67.1	87.0	52.1	76.6	70.6	87.0	82.3	95.2	60.7	79.8	34.6	70.9	39.6	72.7	33.4	71.6	71.6	87.8	81.3	92.8	68.3	86.6
Republic of Moldova	40 to 44	51.6	68.0	59.3	81.5	44.6	56.7	51.0	56.5	62.4	75.2	44.4	42.2	77.6	81.4	85.1	91.8	73.3	76.1	67.5	76.3	78.2	89.0	59.2	65.0
Romania	40 to 44	55.1	68.7	63.4	81.7	47.6	56.3	56.2	64.4	67.7	82.6	48.6	48.7	62.6	68.5	61.5	71.7	64.5	67.1	57.2	73.6	74.0	85.8	41.8	61.5
Russian Federation	40 to 44	60.4	59.3	71.9	74.8	49.9	45.1	59.3	53.8	73.1	73.2	49.2	39.6	64.0	61.1	69.8	67.5	62.1	58.6	44.4	52.0	70.3	77.5	17.1	23.4
Rwanda	40 to 44	32.0	62.2	42.8	71.2	18.6	52.3	43.8	57.2	60.8	72.3	28.8	44.0	59.4	78.4	64.0	82.6	57.7	77.2	67.3	84.8	72.2	88.3	69.9	84.5
Saint Kitts and Nevis	40 to 44	33.9	64.9	43.0	73.7	23.7	56.8	40.4	62.8	56.9	75.7	28.3	53.6	53.7	75.7	51.6	76.9	56.0	75.2	32.2	66.8	50.2	79.4	16.1	55.8
Saint Lucia	40 to 44	41.3	63.7	49.0	72.4	32.4	53.9	44.3	60.0	59.5	75.4	33.9	46.9	57.7	72.7	53.3	71.5	62.7	74.2	35.8	67.6	50.8	77.6	23.9	58.9
Saint Vincent and the Grenadines	40 to 44	49.3	52.1	56.4	58.2	40.5	44.6	43.7	48.6	59.1	61.9	32.0	39.3	69.4	68.8	68.6	66.1	70.4	71.9	57.5	55.1	66.2	64.1	52.9	50.1
Samoa	40 to 44	50.0	55.0	61.9	62.3	38.3	47.9	56.0	56.9	71.3	69.7	43.6	47.3	65.0	71.8	72.1	73.5	60.8	71.9	81.0	81.7	82.1	83.2	84.8	85.0
San Marino	40 to 44	79.8	92.5	84.2	93.5	76.5	90.7	77.2	94.3	84.1	94.5	71.7	92.4	83.1	92.1	83.4	91.9	84.0	93.3	80.1	92.8	89.0	95.8	74.7	91.0
Sao Tome and Principe	40 to 44	70.7	73.0	70.9	72.5	71.8	75.2	68.9	69.6	73.5	74.1	68.5	68.9	80.0	78.9	78.7	76.7	81.8	81.6	78.7	75.7	85.5	84.4	72.1	68.4
Saudi Arabia	40 to 44	49.0	57.2	43.9	59.0	52.8	56.7	45.3	52.6	52.7	65.4	43.3	45.3	79.1	82.5	75.7	79.6	82.6	85.9	23.3	21.2	18.3	29.7	30.8	19.0
Senegal	40 to 44	56.6	65.9	60.4	69.3	53.5	63.0	51.3	58.5	60.0	65.4	48.4	56.3	70.2	75.1	71.1	76.6	70.4	75.1	65.5	72.9	80.8	85.1	48.3	59.3
Serbia	40 to 44	53.6	76.3	61.2	84.0	45.8	68.0	58.3	73.2	71.5	85.6	48.1	61.7	50.2	72.4	45.5	70.9	55.2	74.2	50.1	72.7	68.0	84.8	32.9	60.3
Seychelles	40 to 44	45.7	58.9	62.6	75.7	35.9	48.5	52.1	57.5	68.0	75.5	43.1	47.0	68.5	81.2	75.2	87.1	65.3	78.0	47.3	60.3	71.8	80.8	29.1	44.4
Sierra Leone	40 to 44	58.5	60.2	63.3	63.0	54.7	58.1	56.1	56.8	65.1	64.8	51.9	53.8	72.6	72.9	74.7	71.7	72.6	74.8	63.2	63.0	82.1	80.5	42.6	41.9
Singapore	40 to 44	69.1	85.5	71.9	89.4	67.5	83.0	75.0	90.2	81.5	94.8	70.1	85.1	67.7	85.1	66.0	85.1	71.0	86.7	75.5	91.2	77.4	94.7	78.9	91.3
Slovakia	40 to 44	58.9	82.7	72.5	91.7	46.6	74.6	53.8	77.3	73.6	90.5	37.5	66.1	66.8	84.8	68.9	86.2	65.2	83.5	56.3	81.6	78.3	91.3	35.3	73.2
Slovenia	40 to 44	69.6	94.1	79.8	99.2	60.6	87.8	72.4	94.2	83.9	100.0	63.4	87.3	73.6	92.0	74.7	96.5	73.3	88.4	67.9	92.5	84.4	97.0	52.2	87.1
Solomon Islands	40 to 44	46.8	47.8	61.6	61.3	33.6	34.6	60.6	59.4	74.8	73.5	49.1	47.6	62.0	66.8	73.7	74.7	55.0	61.8	80.0	79.1	82.3	81.9	82.9	81.7
Somalia	40 to 44	37.0	49.2	51.9	64.0	23.8	36.4	41.8	45.4	60.4	65.3	29.0	31.2	62.1	70.4	71.0	77.5	59.4	69.0	77.3	82.6	81.8	87.5	77.7	81.0
South Sudan	40 to 44	57.8	65.1	61.4	67.4	41.4	44.6	52.6	48.1	64.8	62.7	40.6	31.8	76.0	80.5	76.8	79.1	72.4	74.4	73.4	78.8	84.3	87.1	77.8	79.2
Spain	40 to 44	50.6	56.3	78.4	89.7	67.2	84.3	50.0	44.6	81.9	95.1	69.8	86.6	72.7	75.3	77.6	84.1	75.5	86.2	79.2	82.0	84.6	94.5	65.9	88.9
Sri Lanka	40 to 44	72.1	86.5	76.5	84.5	51.3	65.7	74.5	91.1	76.3	82.1	46.1	56.2	76.1	84.7	88.3	92.8	79.2	86.7	73.5	90.0	76.4	84.1	42.3	60.2
Sudan	40 to 44	63.7	75.6	31.0	57.1	46.7	67.7	59.9	68.3	50.7	57.6	41.5	57.9	83.3	89.5	57.5	69.8	66.4	79.5	59.5	72.3	10.0	39.9	43.1	46.0
Suriname	40 to 44	38.6	61.9	54.4	53.3	32.0	44.9	42.0	53.6	63.7	59.6	35.1	43.4	61.6	74.3	65.0	62.1	60.7	68.5	21.0	37.9	71.7	70.1	40.6	54.6
Sweden	40 to 44	44.0	49.2	81.4	100.0	77.9	100.0	47.8	49.1	89.9	99.6	79.4	100.0	63.0	65.1	64.9	89.2	72.3	90.8	56.7	60.8	88.4	100.0	75.7	100.0
Switzerland	40 to 44	79.2	100.0	80.2	94.4	77.2	90.3	84.1	100.0	86.8	98.3	80.1	98.5	68.2	89.6	67.5	90.4	74.3	88.7	80.4	100.0	87.1	97.5	75.2	93.3
Syrian Arab Republic	40 to 44	78.5	92.3	64.1	80.4	64.1	76.0	82.8	99.0	72.4	79.6	61.1	66.1	70.3	88.9	82.2	94.6	87.9	99.5	79.8	94.4	48.0	67.4	46.5	59.8
Taiwan (Province of China)	40 to 44	63.7	78.2	79.3	91.4	57.7	69.5	64.9	71.6	83.9	93.7	61.9	69.8	85.0	97.3	87.7	89.4	86.2	84.7	44.2	62.0	80.3	91.1	61.1	76.0
Tajikistan	40 to 44	67.7	80.0	50.0	72.1	46.4	61.4	71.7	79.7	60.8	72.3	49.6	56.3	86.6	86.6	62.1	79.2	73.6	78.2	70.0	84.0	47.7	74.3	13.3	53.2
Thailand	40 to 44	47.9	67.1	63.8	73.7	48.7	36.8	52.4	63.0	72.8	76.4	59.6	44.5	68.0	77.9	67.3	74.6	54.0	52.5	29.2	63.4	73.9	79.8	62.1	48.5
Timor-Leste	40 to 44	56.0	52.9	43.1	53.3	28.3	29.7	64.5	56.5	55.2	61.6	32.6	33.2	59.6	60.5	65.3	69.8	61.0	64.0	66.1	64.0	70.3	75.1	52.7	38.5
Togo	40 to 44	35.3	41.2	55.5	64.0	56.0	51.3	40.8	44.9	59.0	63.7	53.2	46.5	62.3	66.0	68.1	73.0	71.5	69.2	59.9	57.1	79.6	83.5	52.8	44.6
Tokelau	40 to 44	55.2	57.3	51.3	64.1	28.1	48.3	52.8	52.7	66.2	69.3	34.2	42.8	69.5	70.1	63.8	75.1	56.0	73.6	66.7	65.4	72.2	79.9	77.0	83.4
Tonga	40 to 44	42.2	57.5	70.2	74.8	59.9	67.9	50.8	55.6	74.1	74.0	53.9	59.7	59.9	73.8	79.3	83.4	78.1	84.6	69.8	78.3	85.8	86.8	87.6	89.8
Trinidad and Tobago	40 to 44	65.8	71.7	67.0	68.9	59.7	58.3	63.6	65.5	70.8	69.3	53.7	47.5	78.3	83.7	63.6	57.5	60.7	56.9	84.9	86.5	77.8	77.7	48.7	53.9
Tropical Latin America	40 to 44	63.3	63.8	34.6	58.9	17.9	52.3	61.2	57.1	58.4	70.9	27.0	48.7	62.2	57.3	30.3	46.8	27.7	55.4	62.3	65.2	58.1	78.2	25.7	65.3
Tunisia	40 to 44	68.5	78.6	66.1	79.2	71.1	77.9	64.6	72.9	69.4	77.7	64.6	70.7	81.3	88.6	79.9	87.6	83.7	90.1	52.0	60.8	52.2	67.3	58.6	58.7
Turkey	40 to 44	66.9	86.5	73.9	90.1	61.1	82.2	68.9	82.0	77.7	88.0	62.8	76.3	60.5	81.3	66.5	84.3	56.4	79.0	64.4	84.1	74.1	88.1	57.5	80.7
Turkmenistan	40 to 44	53.4	58.0	62.8	74.8	44.0	44.0	57.1	56.1	70.1	76.2	47.3	42.0	57.2	58.8	61.3	69.9	56.0	54.9	36.2	53.7	55.3	73.0	18.6	35.3
Tuvalu	40 to 44	25.5	41.2	37.6	51.9	9.2	33.1	40.6	48.2	58.3	63.1	25.4	39.0	48.4	62.3	55.4	66.3	40.6	61.9	63.2	70.6	66.3	72.6	69.6	74.4
Uganda	40 to 44	55.4	64.9	70.6	77.1	42.7	53.7	49.8	56.8	68.5	72.7	37.9	46.5	72.2	77.9	80.4	84.7	69.7	75.3	83.8	85.7	88.3	90.6	82.0	82.9
Ukraine	40 to 44	67.2	66.2	75.4	80.0	59.8	56.2	64.5	63.9	76.8	80.1	53.8	51.2	68.6	67.4	73.0	75.2	66.8	64.9	62.3	63.7	78.9	84.5	47.8	46.2
United Arab Emirates	40 to 44	77.0	87.0	81.7	88.6	76.3	87.2	76.1	87.2	84.8	89.3	71.8	86.3	77.9	89.5	76.7	86.7	80.4	92.0	71.7	87.7	80.2	90.2	69.8	88.6
United Kingdom	40 to 44	75.8	86.0	77.5	87.8	74.3	83.7	83.4	88.9	86.8	92.3	80.8	84.9	62.1	79.2	60.7	78.8	64.3	80.6	81.0	91.7	88.5	95.4	76.2	89.3

United Republic of Tanzania	40 to 44	54.6	71.5	64.6	77.2	46.1	66.6	53.6	64.3	66.9	74.6	45.1	57.2	72.4	80.5	77.3	83.0	71.5	80.5	82.1	84.4	86.3	89.6	81.3	81.4
United States Virgin Islands	40 to 44	57.9	77.7	66.0	82.0	47.3	72.1	57.8	75.5	71.5	83.4	45.8	67.9	65.7	81.4	66.9	80.3	63.2	82.6	65.8	84.0	76.7	88.6	54.7	80.9
United States of America	40 to 44	86.5	97.3	87.4	97.3	85.1	95.9	80.7	87.9	86.8	92.0	75.1	83.8	67.5	82.7	67.9	84.8	66.0	78.8	93.0	97.8	94.7	97.2	91.6	97.2
Uruguay	40 to 44	57.2	71.5	62.6	73.0	52.2	70.1	64.8	79.5	75.5	85.8	56.0	72.9	47.9	63.3	50.5	61.5	44.1	65.9	70.8	81.3	78.8	85.1	67.9	81.6
Uzbekistan	40 to 44	48.1	72.1	56.4	80.2	40.6	64.9	43.3	73.5	58.3	82.3	33.5	66.6	75.4	79.6	78.5	87.6	74.7	75.8	40.3	64.3	57.8	79.3	26.7	50.5
Vanuatu	40 to 44	39.1	48.1	57.8	61.7	24.1	36.8	46.8	52.6	67.6	68.6	32.0	41.7	54.9	64.4	66.3	70.7	49.5	62.1	75.6	79.1	80.0	82.8	74.8	79.1
Venezuela (Bolivarian Republic of)	40 to 44	56.8	63.1	61.1	70.9	50.9	51.1	60.8	62.2	71.0	77.2	52.5	45.4	66.2	69.4	63.8	69.2	69.2	68.6	69.4	79.3	77.3	84.2	62.4	75.2
Viet Nam	40 to 44	37.8	48.9	54.9	70.2	23.5	34.1	46.5	51.6	63.5	73.1	35.5	37.4	64.3	74.0	73.9	82.7	57.8	69.1	55.1	57.8	74.1	80.1	36.4	39.2
Yemen	40 to 44	29.1	48.0	26.3	46.5	32.6	50.5	33.9	43.3	47.8	51.5	27.3	42.5	58.3	67.6	59.3	67.1	58.5	68.9	8.3	16.2	0.0	24.7	27.8	18.2
Zambia	40 to 44	44.3	50.1	50.4	61.1	37.5	40.4	45.9	45.7	58.7	60.4	38.1	37.0	65.8	69.5	65.0	71.1	68.3	70.8	77.7	77.1	81.2	83.7	80.3	73.8
Zimbabwe	40 to 44	76.0	54.8	80.1	63.5	71.3	44.3	63.4	28.4	70.2	43.5	60.3	21.6	80.3	64.2	79.8	63.5	82.4	67.0	87.7	74.7	91.6	83.2	85.3	66.3
Afghanistan	45 to 49	33.6	41.4	46.8	45.9	27.0	45.3	44.4	39.1	65.8	55.0	23.6	35.2	55.3	59.5	68.2	67.0	36.2	51.2	20.1	16.2	18.8	16.2	36.9	31.0
Albania	45 to 49	68.9	81.9	73.7	86.2	66.9	76.3	64.5	71.2	73.3	80.1	62.0	66.2	77.8	86.1	80.4	88.0	75.1	83.7	83.9	89.1	88.9	93.3	83.2	85.6
Algeria	45 to 49	65.7	77.3	63.0	74.4	73.4	82.5	63.7	69.4	68.5	71.5	69.2	76.2	75.3	83.6	76.1	83.4	75.6	84.9	50.4	64.2	43.5	60.8	67.1	75.8
American Samoa	45 to 49	54.7	57.1	67.0	64.7	49.3	54.4	57.4	54.2	70.4	65.2	52.1	51.6	69.2	71.0	76.8	76.3	62.9	66.2	75.2	77.5	80.5	79.4	75.4	80.2
Andorra	45 to 49	80.7	92.6	80.3	89.8	82.0	94.0	80.2	95.3	84.0	93.7	79.3	96.7	67.8	82.0	65.3	80.5	71.7	85.9	83.9	93.1	87.8	94.1	85.9	95.0
Angola	45 to 49	49.5	60.7	60.3	68.6	44.0	55.6	47.2	50.0	62.1	63.0	41.5	46.0	68.2	73.0	73.7	77.2	63.6	69.2	73.0	76.1	78.3	81.3	73.8	75.5
Antigua and Barbuda	45 to 49	52.9	73.4	66.3	77.0	42.3	69.8	49.7	64.8	67.5	74.8	38.8	59.5	65.4	75.8	72.9	77.8	55.2	74.3	68.5	83.6	76.7	85.3	64.8	84.9
Argentina	45 to 49	55.2	78.2	64.5	81.8	51.5	75.6	63.7	77.7	76.1	85.1	56.7	72.8	51.9	74.1	59.9	78.0	41.5	70.0	74.1	89.1	81.5	91.6	75.6	91.3
Armenia	45 to 49	74.9	81.4	80.1	90.5	71.0	72.8	68.5	76.8	78.2	89.7	63.1	64.6	76.7	81.3	85.8	93.8	67.8	71.3	68.7	69.4	76.2	84.4	68.6	61.6
Australia	45 to 49	81.0	89.0	82.6	88.1	80.4	89.4	81.9	89.0	87.9	92.5	78.6	86.1	61.0	73.8	65.9	78.2	55.6	68.5	85.1	92.5	88.7	93.2	86.7	95.1
Austria	45 to 49	77.3	91.3	81.6	90.0	74.6	91.7	76.5	95.3	84.3	94.8	72.0	95.1	74.7	85.0	76.6	85.3	73.7	86.2	72.4	92.5	83.7	93.8	68.8	95.2
Azerbaijan	45 to 49	61.2	76.0	71.0	81.7	54.5	70.8	61.2	70.9	72.8	78.8	55.5	67.2	83.4	90.0	89.0	94.5	78.0	85.7	63.1	78.9	76.1	86.2	56.0	75.3
Bahamas	45 to 49	46.8	62.0	56.9	71.0	41.6	55.3	43.6	56.0	59.3	70.4	37.7	48.2	62.2	66.0	66.7	72.0	56.6	58.0	63.1	75.5	69.4	80.3	62.8	75.1
Bahrain	45 to 49	73.9	92.1	76.2	91.9	74.3	93.2	69.8	86.3	78.3	88.7	66.3	87.0	76.5	89.3	78.6	91.2	74.6	88.2	65.3	79.6	67.3	81.7	69.1	81.5
Bangladesh	45 to 49	40.1	58.7	46.6	67.5	41.8	55.6	51.3	61.1	61.7	73.4	49.3	55.2	51.9	68.2	58.2	72.3	47.1	65.1	57.2	68.6	59.4	77.0	63.9	68.4
Barbados	45 to 49	55.9	69.4	67.5	76.9	47.4	62.4	50.5	61.1	68.7	74.9	39.2	51.7	60.7	66.1	67.1	74.1	52.8	55.6	61.1	76.5	69.9	81.1	58.9	76.1
Belarus	45 to 49	57.5	66.8	67.1	80.0	53.4	57.4	56.7	59.3	67.4	76.1	53.6	48.8	59.2	62.4	71.1	79.7	46.9	45.4	47.9	65.9	64.6	82.7	44.1	58.7
Belgium	45 to 49	71.9	86.8	76.5	87.1	69.6	85.8	68.8	88.2	77.0	91.0	65.4	85.8	71.3	73.5	74.4	76.1	68.6	71.7	74.0	90.1	81.9	92.3	74.3	91.5
Belize	45 to 49	55.3	61.4	59.5	73.6	56.6	50.5	50.0	51.0	60.4	70.4	49.8	38.0	67.6	72.5	71.0	78.4	64.0	64.2	76.1	74.4	78.3	83.6	78.8	68.8
Benin	45 to 49	56.6	65.2	62.6	72.0	55.7	61.3	55.0	61.0	63.1	69.8	56.2	59.4	70.9	76.2	75.9	80.9	66.3	72.0	69.1	71.7	81.9	84.9	58.7	59.7
Bermuda	45 to 49	70.1	87.8	81.0	92.8	58.8	80.0	67.2	83.6	82.7	94.3	52.9	72.5	72.0	83.4	81.0	90.9	58.7	73.4	67.7	87.5	81.7	92.8	58.4	84.2
Bhutan	45 to 49	54.7	70.2	55.4	70.7	60.4	72.7	56.8	65.8	65.4	71.9	57.6	66.5	62.1	71.5	62.5	71.5	62.1	71.7	68.3	77.7	64.5	78.3	79.3	82.6
Bolivia (Plurinational State of)	45 to 49	37.3	59.0	45.2	61.8	37.2	61.2	38.1	49.8	53.9	62.4	34.2	46.7	49.2	59.8	51.3	59.6	48.5	62.8	53.3	73.5	59.7	76.8	54.1	74.5
Bosnia and Herzegovina	45 to 49	69.2	85.3	74.1	87.7	67.1	82.9	56.2	77.4	70.1	86.7	49.4	71.1	51.3	77.2	55.6	78.8	46.5	75.7	62.1	77.0	71.0	83.8	62.4	76.5
Botswana	45 to 49	52.2	71.8	62.2	78.2	48.2	67.3	47.1	65.6	56.9	74.6	46.6	62.4	73.0	82.2	79.1	86.2	67.4	79.1	73.8	81.8	81.8	88.2	71.1	79.9
Brazil	45 to 49	31.4	55.9	46.3	62.6	24.9	53.6	38.6	51.8	59.0	67.4	27.3	42.9	42.9	51.9	50.6	54.8	31.4	49.7	27.0	63.4	52.5	73.8	16.7	60.6
Brunei Darussalam	45 to 49	63.7	67.1	63.6	71.5	67.2	66.7	69.9	66.9	75.2	77.8	69.9	61.3	49.1	57.0	48.6	61.4	50.1	53.1	74.5	80.6	73.7	82.7	82.2	85.5
Bulgaria	45 to 49	48.5	61.6	64.5	75.1	40.4	52.8	54.5	59.2	70.9	77.1	46.0	48.2	61.2	68.0	68.7	74.0	53.0	61.6	42.8	42.1	64.8	66.2	34.8	31.3
Burkina Faso	45 to 49	51.9	58.3	64.2	73.0	42.7	46.4	49.2	54.6	65.9	72.5	39.8	43.0	70.4	74.2	80.0	84.4	60.0	64.5	71.9	72.0	84.4	87.5	59.3	57.5
Burundi	45 to 49	35.3	53.7	53.0	65.1	22.2	47.8	40.2	45.9	61.9	63.6	25.9	37.3	57.7	71.4	68.6	78.6	46.7	66.1	64.3	77.1	68.4	80.4	68.0	79.8
Cabo Verde	45 to 49	66.9	71.7	75.6	82.8	56.2	64.0	65.8	69.1	76.1	80.2	59.1	63.8	76.3	81.0	83.8	88.2	65.5	74.3	66.8	64.0	85.3	88.2	39.0	46.7
Cambodia	45 to 49	24.9	46.7	35.3	56.1	25.1	43.5	37.2	51.0	52.1	63.6	35.7	47.4	56.6	70.3	63.4	75.3	47.8	64.7	52.5	65.4	59.5	71.2	54.6	66.9
Cameroon	45 to 49	51.8	56.4	62.5	68.6	46.0	49.3	52.6	54.6	66.7	68.7	46.1	48.0	68.9	70.2	77.3	79.0	60.7	62.3	69.7	64.3	83.5	83.6	57.5	49.9
Canada	45 to 49	88.4	92.6	88.2	91.1	87.8	92.9	88.9	94.3	92.1	94.1	85.6	93.8	72.0	83.2	76.6	87.2	65.7	77.5	90.8	93.4	92.8	93.6	92.4	96.3
Central African Republic	45 to 49	30.2	33.4	47.0	51.8	21.1	23.4	35.1	30.0	54.3	52.0	27.4	20.5	56.1	59.7	64.7	68.6	47.7	52.1	60.7	65.4	68.7	74.4	59.0	63.0
Chad	45 to 49	56.2	55.4	63.3	66.0	53.5	49.3	55.3	53.5	65.6	66.6	53.7	48.5	68.8	67.7	74.7	74.5	62.9	61.4	69.2	64.3	81.4	82.1	57.9	49.1
Chile	45 to 49	64.3	82.6	70.7	85.6	61.5	79.1	68.1	84.3	79.3	90.5	59.8	77.7	61.0	73.3	66.3	78.1	54.3	67.2	73.7	87.8	78.9	91.6	76.7	88.4

China	45 to 49	39.4	64.9	54.6	76.8	31.8	57.7	53.5	64.1	69.3	77.9	44.7	56.7	43.7	75.7	52.4	83.0	34.3	68.4	37.9	63.6	56.6	77.6	28.5	58.8
Colombia	45 to 49	62.3	78.7	66.1	80.8	62.1	75.5	60.7	76.9	70.1	84.7	57.9	68.7	65.0	71.3	67.5	74.2	63.2	68.5	65.8	81.8	72.5	86.4	65.2	78.6
Comoros	45 to 49	54.1	66.9	62.5	72.9	49.7	63.0	50.4	56.2	63.7	68.6	45.9	51.1	71.7	77.9	75.7	81.8	68.5	74.9	76.1	82.1	79.4	85.0	78.6	83.6
Congo	45 to 49	44.3	56.1	54.6	60.8	40.4	56.9	42.8	42.9	56.1	53.7	40.6	45.1	65.0	70.7	68.7	70.7	61.7	71.3	67.8	72.5	74.9	76.7	66.5	74.6
Cook Islands	45 to 49	58.7	79.1	67.9	83.9	54.2	74.7	59.5	73.0	71.0	82.3	55.0	67.5	68.8	86.2	77.2	90.1	60.6	82.3	76.4	87.9	79.5	88.5	78.1	89.9
Costa Rica	45 to 49	78.3	83.9	79.9	86.3	76.7	78.9	76.0	81.9	81.5	90.1	73.2	73.0	75.5	73.5	78.1	77.7	73.3	68.1	79.0	87.6	82.6	90.2	78.2	85.7
Coted'Ivoire	45 to 49	57.7	59.2	71.4	72.7	51.7	52.6	57.1	55.7	71.7	70.2	50.7	49.4	68.7	71.6	79.4	80.9	60.5	64.7	60.8	56.9	84.1	85.0	49.1	40.4
Croatia	45 to 49	54.6	83.0	68.0	91.4	47.5	75.8	57.0	77.4	73.8	92.9	47.0	65.0	54.6	78.4	60.6	82.7	47.3	73.3	55.8	81.9	71.7	90.1	52.3	80.4
Cuba	45 to 49	56.1	70.6	64.3	79.4	51.3	62.6	53.5	60.9	67.8	78.1	45.9	48.1	63.8	77.1	68.4	79.6	58.0	74.3	64.8	69.5	73.2	78.1	62.0	66.5
Cyprus	45 to 49	72.2	91.6	71.8	92.2	75.7	88.2	78.1	91.5	81.9	94.1	78.1	86.7	63.5	81.0	61.8	83.8	67.1	78.3	78.6	94.0	78.9	96.7	84.7	91.5
Czechia	45 to 49	62.7	88.9	75.6	93.8	54.7	83.4	61.4	85.8	78.4	96.6	50.4	76.4	60.6	82.7	66.8	83.6	52.7	81.7	49.8	85.0	71.3	91.7	42.0	83.0
Democratic People's Republic of Korea	45 to 49	42.9	36.2	53.7	51.7	39.3	29.9	52.0	40.0	65.6	59.5	46.7	31.0	58.4	62.7	63.9	68.7	52.3	56.5	48.5	42.9	58.2	58.2	48.9	40.2
Democratic Republic of the Congo	45 to 49	55.5	57.5	62.4	66.4	53.3	53.1	54.0	51.3	64.0	64.8	53.0	46.5	72.5	73.8	75.8	77.7	69.7	70.8	73.5	76.3	77.4	81.1	75.6	77.2
Denmark	45 to 49	72.2	89.1	77.2	89.7	69.7	87.5	71.6	89.4	82.1	93.5	65.2	85.4	64.4	82.0	68.6	83.6	60.0	81.7	73.3	91.8	83.4	94.2	72.6	93.3
Djibouti	45 to 49	55.2	64.7	65.7	74.2	50.6	59.4	50.6	52.8	64.1	67.1	46.1	47.3	71.2	75.5	78.3	82.1	66.0	70.8	78.3	78.8	82.7	85.9	79.8	77.4
Dominica	45 to 49	53.3	60.0	63.3	70.3	45.6	51.0	45.8	45.2	62.6	65.2	36.9	32.5	64.4	68.2	70.9	74.7	55.6	60.3	66.7	68.8	72.2	76.5	65.6	64.8
Dominican Republic	45 to 49	48.6	56.2	58.3	66.9	43.6	49.1	45.5	50.8	62.1	67.4	37.4	41.7	60.0	66.6	64.4	70.2	54.4	62.7	63.8	66.8	71.1	76.3	62.5	63.7
Ecuador	45 to 49	57.7	76.4	64.1	78.8	55.1	74.8	49.2	70.3	63.2	79.3	44.2	64.9	70.3	70.5	73.0	72.8	68.4	69.1	60.4	85.3	65.0	89.0	63.1	83.9
Egypt	45 to 49	50.2	69.1	59.8	75.0	45.1	65.9	41.5	60.4	62.7	73.5	27.0	53.0	65.7	82.0	75.8	86.9	51.6	76.0	23.4	42.6	28.5	54.2	32.3	42.9
El Salvador	45 to 49	58.2	71.5	63.9	78.2	55.6	59.8	59.9	61.7	69.4	76.9	57.7	45.1	66.4	72.5	70.0	76.8	62.2	65.8	64.4	76.3	73.2	84.7	58.6	64.4
Equatorial Guinea	45 to 49	38.7	65.9	53.1	70.8	30.0	63.2	37.4	51.6	55.0	63.1	31.1	49.8	62.2	79.2	70.6	80.7	53.8	78.6	67.3	75.4	75.6	80.5	63.8	73.2
Eritrea	45 to 49	22.9	48.2	42.7	62.3	11.9	39.5	25.7	40.9	48.4	59.9	16.5	32.3	55.3	68.9	68.8	77.2	43.0	61.8	67.7	75.7	72.9	81.3	69.7	75.4
Estonia	45 to 49	61.9	85.9	72.6	92.7	56.4	77.0	60.1	74.5	73.3	93.1	53.5	58.4	64.4	79.0	79.0	91.6	47.3	66.6	49.0	85.1	65.4	92.4	46.5	80.3
Eswatini	45 to 49	45.6	39.4	62.5	59.3	32.4	27.8	37.4	27.4	58.2	47.2	27.2	20.7	72.2	69.5	79.7	76.3	64.4	63.2	75.9	67.3	83.5	79.7	70.5	60.4
Ethiopia	45 to 49	43.0	65.3	58.0	72.0	33.8	61.6	50.4	57.6	67.6	69.4	40.2	53.2	70.4	79.8	78.9	84.1	63.4	76.7	79.1	85.9	80.7	86.1	83.6	89.5
Fiji	45 to 49	53.4	65.9	62.6	70.5	48.7	64.4	48.6	53.2	63.9	65.2	42.0	49.9	69.3	76.1	77.1	81.4	57.5	69.4	75.5	81.7	79.4	83.1	76.6	84.5
Finland	45 to 49	73.2	90.0	79.5	90.1	70.4	89.2	74.8	92.0	84.6	93.7	69.1	90.7	66.0	80.0	72.9	83.2	59.0	77.3	68.7	92.8	81.6	94.9	68.0	94.2
France	45 to 49	77.0	88.1	84.3	88.7	71.2	87.3	68.6	89.8	81.5	94.2	60.2	85.4	76.6	77.8	80.8	80.7	72.6	75.4	81.0	90.5	90.1	93.4	76.9	92.0
Gabon	45 to 49	58.2	65.8	68.8	73.4	51.8	60.8	55.2	55.0	68.5	67.0	49.6	51.0	74.9	77.9	80.3	81.6	70.1	75.0	77.2	78.1	82.4	83.3	77.4	77.8
Gambia	45 to 49	55.4	55.0	67.9	66.6	49.9	48.2	51.4	47.9	65.2	63.0	46.5	42.1	69.3	68.5	78.6	75.2	62.3	62.5	58.9	57.2	83.5	80.9	43.7	37.6
Georgia	45 to 49	62.5	68.9	75.4	85.4	52.5	57.5	66.8	70.0	78.3	85.1	60.4	61.8	75.4	69.2	84.9	82.2	66.9	59.5	69.7	62.6	80.8	85.5	59.1	47.0
Germany	45 to 49	76.7	89.0	80.8	90.0	74.3	87.1	72.1	90.3	82.0	93.7	65.9	86.7	68.8	80.2	70.2	81.2	68.7	80.6	75.0	87.9	84.2	91.7	73.8	88.8
Ghana	45 to 49	52.5	60.2	63.2	72.9	45.8	50.4	48.3	52.3	62.3	67.4	43.5	45.2	64.0	69.5	72.7	78.2	55.3	61.2	58.8	59.3	80.8	84.3	37.1	36.3
Greece	45 to 49	79.3	82.7	82.3	87.4	77.6	78.5	79.5	78.9	83.9	87.7	78.0	72.7	78.6	79.3	82.3	83.6	75.0	74.9	80.6	88.9	85.3	92.4	81.7	89.3
Greenland	45 to 49	58.5	73.7	61.0	76.3	60.8	73.6	57.0	66.9	72.9	79.6	48.8	59.8	0.0	53.3	0.0	56.8	0.0	49.7	74.4	88.3	81.6	91.4	77.2	89.7
Grenada	45 to 49	44.8	65.6	51.3	74.2	45.2	59.3	42.7	57.6	54.9	73.4	41.8	47.8	57.9	67.2	61.1	72.4	54.6	61.1	42.4	73.4	45.9	80.3	49.7	71.8
Guam	45 to 49	74.4	72.4	78.8	78.8	72.2	67.2	72.1	62.6	79.5	75.6	69.1	55.0	76.7	77.0	82.0	83.3	72.1	70.8	81.6	83.9	84.2	86.4	83.2	83.7
Guatemala	45 to 49	62.1	72.3	64.8	75.1	63.4	69.9	65.0	67.5	71.5	75.2	66.3	65.0	81.7	79.4	83.6	81.8	81.0	77.2	65.6	81.3	69.7	84.6	65.7	78.8
Guinea	45 to 49	55.8	59.8	59.6	67.7	58.2	55.6	56.4	58.5	64.3	68.6	58.7	56.0	71.5	72.5	74.4	77.0	69.0	68.4	67.5	64.0	79.5	82.5	58.5	47.4
Guinea-Bissau	45 to 49	31.5	38.6	49.2	54.8	19.8	27.8	36.1	36.2	55.7	55.5	27.0	27.6	49.5	54.9	62.5	64.1	34.6	45.1	46.9	46.7	74.1	75.6	17.2	16.3
Guyana	45 to 49	15.6	42.0	39.1	54.7	3.7	34.9	23.8	37.6	50.0	58.2	10.4	25.7	50.7	56.2	65.0	65.7	31.7	43.1	30.5	52.7	55.8	63.4	17.4	49.8
Haiti	45 to 49	0.3	22.2	13.0	34.0	2.0	19.4	22.1	26.7	44.6	49.8	12.0	12.8	16.9	35.3	21.1	38.4	12.0	32.3	12.5	39.4	12.6	42.7	30.0	48.3
Honduras	45 to 49	34.5	34.0	44.2	39.6	31.5	38.4	24.8	13.5	46.2	35.4	16.9	11.6	47.2	36.5	51.4	36.8	41.7	39.2	44.1	53.3	54.3	59.5	39.6	52.8
Hungary	45 to 49	58.2	79.1	70.0	87.7	51.6	70.6	57.6	73.1	71.8	88.3	50.5	62.0	56.3	74.2	62.4	79.2	47.5	68.1	43.9	72.5	66.3	84.8	35.3	66.1
Iceland	45 to 49	80.4	92.6	80.3	92.0	81.5	91.7	81.7	96.4	84.2	97.0	82.0	94.3	72.4	84.0	70.4	86.6	77.2	81.6	81.5	93.9	85.2	95.1	84.5	95.9
India	45 to 49	52.2	63.5	58.7	67.8	52.1	63.4	55.7	64.1	68.0	72.5	50.9	61.9	59.3	66.0	65.8	69.4	53.4	63.2	66.2	75.2	68.1	77.4	71.8	78.6
Indonesia	45 to 49	46.0	47.3	55.2	57.8	42.6	42.5	51.2	46.0	65.0	61.3	45.5	40.4	64.0	67.2	69.6	71.5	57.5	62.6	64.5	60.2	71.6	71.2	63.2	55.7
Iran (Islamic Republic of)	45 to 49	71.1	86.9	74.9	88.7	69.0	84.7	64.9	79.5	74.5	85.7	60.9	76.2	78.1	90.7	82.5	93.6	72.6	87.0	36.6	65.2	45.2	72.3	39.0	64.8

Iraq	45 to 49	36.9	60.0	52.1	68.4	29.7	56.4	40.9	58.4	61.0	69.9	30.8	54.5	62.6	75.9	70.2	79.0	52.0	72.3	0.0	35.7	16.8	47.7	0.0	35.9
Ireland	45 to 49	77.5	92.8	78.7	91.6	77.8	92.0	82.9	100.0	86.2	98.7	81.7	99.2	66.8	80.9	70.0	84.0	64.2	77.9	79.0	94.4	83.6	95.4	80.9	95.8
Israel	45 to 49	76.0	92.5	78.9	93.4	75.2	90.5	68.8	87.7	78.3	95.2	64.0	80.9	80.0	89.0	82.1	91.7	79.1	86.6	81.1	94.6	83.7	95.8	85.4	96.6
Italy	45 to 49	74.4	90.7	78.7	93.1	72.0	87.2	71.8	85.4	80.8	92.6	66.5	79.3	68.9	81.6	72.9	83.3	64.8	80.6	78.1	94.0	84.1	97.1	78.7	93.7
Jamaica	45 to 49	50.0	60.0	55.8	67.3	50.0	55.2	50.1	57.0	62.1	71.2	47.5	47.8	62.6	68.6	64.9	70.7	61.0	67.2	54.9	66.9	57.6	71.7	60.5	67.3
Japan	45 to 49	69.6	79.9	76.3	85.0	66.9	77.5	73.8	82.2	84.8	91.1	66.4	77.1	56.8	72.9	62.8	78.7	51.0	67.8	79.6	88.3	84.4	93.0	82.5	91.0
Jordan	45 to 49	68.0	84.7	68.1	86.1	71.8	84.2	58.2	77.2	67.1	82.3	58.8	75.7	89.6	100.0	90.0	100.0	90.1	100.0	47.5	72.3	49.3	74.7	56.4	76.8
Kazakhstan	45 to 49	65.7	68.4	73.8	78.1	60.5	60.3	62.9	59.9	73.6	74.7	58.1	50.6	64.2	69.2	70.5	76.8	58.0	62.3	55.4	67.1	71.9	78.4	47.3	61.4
Kenya	45 to 49	69.7	67.0	73.0	72.2	69.0	65.0	64.3	57.6	72.1	67.1	63.0	55.8	79.9	78.7	82.6	81.1	78.1	77.0	86.8	83.8	86.6	85.3	91.0	86.6
Kiribati	45 to 49	55.9	55.2	67.3	68.9	51.9	48.8	67.3	62.4	75.2	73.5	65.0	58.1	65.4	70.8	82.5	83.8	46.4	58.4	74.3	72.5	82.0	83.2	72.2	66.6
Kuwait	45 to 49	86.3	93.7	85.5	99.0	87.6	88.9	78.8	87.8	83.4	96.3	78.0	82.1	85.5	97.0	83.6	99.0	87.0	94.8	71.4	81.6	71.4	91.1	76.0	78.3
Kyrgyzstan	45 to 49	52.4	59.5	62.9	72.9	47.6	50.3	57.5	62.6	67.1	75.8	55.4	55.3	59.9	65.1	71.5	71.4	48.1	59.1	44.3	34.4	63.8	64.7	34.2	14.7
Lao People's Democratic Republic	45 to 49	7.1	41.6	20.5	53.4	7.7	37.5	24.9	45.8	44.0	60.7	20.9	40.7	38.7	66.1	45.1	72.0	31.3	60.0	24.5	54.5	33.7	67.8	29.6	50.4
Latvia	45 to 49	59.1	73.3	69.8	83.9	52.7	65.3	57.3	61.5	69.5	78.8	52.1	49.2	66.1	73.1	77.3	84.1	53.8	62.1	45.2	67.2	66.6	83.1	35.6	60.7
Lebanon	45 to 49	51.1	88.6	63.2	90.9	44.7	85.4	39.9	80.6	66.8	88.5	17.0	73.2	52.1	86.8	64.3	91.1	33.8	80.2	44.4	84.1	51.0	88.2	48.4	84.5
Lesotho	45 to 49	51.9	21.6	67.1	49.3	34.1	0.0	42.0	16.6	61.7	47.3	30.4	0.0	74.4	60.2	82.8	71.3	62.7	47.8	82.1	58.9	87.0	74.7	75.9	44.8
Liberia	45 to 49	56.9	55.3	63.0	63.5	56.0	52.3	56.7	49.1	65.7	61.4	56.2	46.5	72.0	70.1	76.3	74.7	68.6	66.2	64.7	60.2	80.3	79.7	53.7	45.1
Libya	45 to 49	77.7	74.4	72.4	73.7	86.3	77.7	69.5	61.6	72.0	66.7	76.5	67.6	82.1	81.7	80.9	79.9	84.0	85.2	62.1	50.5	58.1	55.6	72.1	54.0
Lithuania	45 to 49	62.8	75.2	71.3	84.7	57.7	67.1	54.8	66.7	66.5	82.8	51.1	55.4	60.8	69.3	72.1	78.9	47.9	58.5	54.7	69.7	69.5	85.6	50.0	61.5
Luxembourg	45 to 49	67.6	93.9	75.6	92.9	63.5	92.9	64.6	93.9	77.9	94.9	56.8	92.3	66.8	84.2	69.0	85.3	65.4	84.0	65.2	94.7	78.2	96.2	62.7	95.0
Madagascar	45 to 49	43.7	50.2	57.9	60.3	34.8	45.3	47.0	44.2	64.0	59.9	38.5	38.7	63.5	67.9	72.5	72.3	55.6	64.4	71.7	75.2	78.7	80.2	69.1	75.3
Malawi	45 to 49	51.6	45.6	59.7	61.3	49.1	38.0	46.0	26.5	60.3	49.7	41.5	17.1	68.0	65.4	73.5	73.2	63.4	59.4	76.8	70.0	79.7	79.4	79.6	67.8
Malaysia	45 to 49	60.7	65.5	67.4	75.5	58.8	64.9	64.3	65.9	73.2	75.5	61.7	62.1	75.5	80.9	80.4	84.5	70.1	77.1	67.1	72.3	73.1	79.0	69.5	72.6
Maldives	45 to 49	35.1	76.3	30.7	80.1	49.0	76.1	40.3	74.0	42.1	76.2	52.2	74.7	63.1	86.5	62.9	89.2	64.5	84.7	52.7	79.7	48.4	84.5	67.9	82.1
Mali	45 to 49	51.6	61.7	51.1	61.9	61.1	66.6	47.9	53.0	54.1	56.9	57.3	60.5	69.6	75.4	66.8	73.2	72.2	77.2	72.5	73.7	75.4	79.9	74.2	70.1
Malta	45 to 49	78.0	93.1	78.7	91.8	78.7	92.5	77.1	90.4	79.9	91.3	78.8	90.1	82.1	86.5	82.2	87.7	83.9	86.1	75.2	93.9	79.4	94.7	78.1	94.4
Marshall Islands	45 to 49	35.4	39.3	51.7	53.2	28.5	33.2	47.0	45.3	63.0	60.8	40.4	39.7	50.0	59.1	66.6	70.6	33.7	47.3	68.6	69.9	73.3	73.9	70.1	71.5
Mauritania	45 to 49	56.0	67.8	62.6	70.6	54.2	68.2	53.0	58.7	62.0	65.7	54.2	61.9	69.6	77.5	74.5	79.5	65.2	75.8	63.5	71.6	80.2	82.4	48.3	60.1
Mauritius	45 to 49	47.3	58.9	64.8	73.9	36.3	47.8	51.1	56.8	70.0	75.0	40.1	46.1	63.2	68.9	76.5	77.3	45.9	58.6	37.0	58.1	60.4	73.1	26.0	49.7
Mexico	45 to 49	74.0	78.8	78.0	84.6	69.1	70.3	68.3	70.7	77.8	83.1	60.9	59.2	77.2	72.4	79.6	78.7	74.8	63.6	69.5	81.2	77.4	87.9	63.8	75.1
Micronesia (Federated States of)	45 to 49	31.1	39.9	45.6	54.0	27.0	34.2	45.4	46.7	59.5	61.1	41.2	41.8	45.4	59.2	63.5	71.7	27.2	47.1	61.9	66.3	66.9	72.6	64.5	66.2
Monaco	45 to 49	70.9	86.2	76.0	88.4	68.4	83.3	64.0	80.8	77.1	89.3	56.6	74.1	59.5	78.3	61.9	79.4	58.1	78.4	71.1	85.5	78.5	90.1	72.0	85.3
Mongolia	45 to 49	44.9	56.0	55.2	70.1	40.9	46.6	57.4	64.6	70.0	77.9	51.1	55.8	53.5	46.4	62.1	56.8	44.6	36.0	81.0	78.2	87.3	89.7	77.8	71.5
Montenegro	45 to 49	50.9	64.1	59.2	72.0	48.4	57.5	57.8	59.9	69.0	74.2	54.1	48.2	75.2	83.9	75.3	83.8	75.6	84.1	80.0	86.0	86.7	90.8	77.3	83.1
Morocco	45 to 49	55.7	64.7	53.5	61.0	65.8	74.6	53.4	55.3	60.7	59.3	60.1	66.5	69.0	75.1	69.7	73.6	69.7	79.2	31.1	40.3	24.8	32.0	51.1	62.8
Mozambique	45 to 49	40.9	32.1	55.4	59.0	33.9	16.8	38.0	24.1	56.7	55.5	30.3	7.9	62.0	54.2	72.5	71.0	53.0	40.5	68.6	58.2	76.2	76.5	67.4	49.4
Myanmar	45 to 49	9.9	38.2	27.2	56.8	6.0	29.2	26.1	41.1	44.9	59.4	21.1	33.4	45.1	64.7	56.9	73.8	31.7	55.4	30.7	56.6	49.3	72.8	24.4	49.3
Namibia	45 to 49	53.9	55.8	64.3	70.3	48.3	45.4	48.6	41.4	61.6	60.1	45.0	33.3	76.0	77.8	81.3	83.9	71.1	72.0	74.7	74.5	82.6	84.8	71.4	68.8
Nauru	45 to 49	34.2	37.2	46.1	49.2	31.9	33.1	45.2	39.9	59.0	55.4	41.5	35.9	46.5	56.1	56.7	63.4	36.0	47.9	53.7	58.5	62.1	65.0	53.5	58.6
Nepal	45 to 49	45.9	60.1	47.4	64.7	52.6	59.7	52.0	58.8	61.9	70.0	52.8	54.1	57.9	67.2	60.0	71.1	56.1	63.4	56.8	71.4	51.3	74.6	71.5	74.3
Netherlands	45 to 49	81.4	91.9	82.7	91.0	81.0	91.3	80.1	96.1	85.1	95.4	77.7	95.8	69.1	81.9	71.0	84.0	68.2	80.7	83.6	91.2	88.1	93.6	85.4	92.2
New Zealand	45 to 49	71.8	88.9	71.5	89.0	75.1	87.7	73.6	90.5	78.9	94.6	72.8	85.6	48.1	65.6	52.8	70.6	42.7	59.2	78.1	93.6	80.6	95.3	81.3	94.2
Nicaragua	45 to 49	63.1	77.9	67.9	81.2	60.6	72.8	58.2	70.1	69.1	80.3	53.7	60.2	71.3	77.8	74.1	80.7	68.6	74.9	69.9	81.7	74.8	86.2	69.5	78.7
Niger	45 to 49	58.6	63.2	65.7	69.0	56.0	60.0	58.3	58.1	68.9	68.5	55.0	55.4	71.1	76.2	76.7	81.1	66.3	71.2	77.1	79.5	83.8	86.2	74.0	73.4
Nigeria	45 to 49	56.8	74.1	67.6	81.5	52.8	66.7	53.5	67.2	66.3	77.9	49.0	60.8	73.5	82.6	82.7	89.1	67.2	76.0	67.4	74.2	84.3	89.9	59.0	57.9
Niue	45 to 49	57.6	65.2	66.8	74.0	53.2	59.3	59.5	61.0	70.9	74.0	55.1	54.1	66.8	75.0	76.4	82.6	56.8	67.3	77.4	81.4	80.0	84.2	79.6	82.2
North Macedonia	45 to 49	57.9	72.0	66.5	76.7	53.3	69.4	59.0	68.1	71.5	77.2	52.3	63.8	56.1	67.8	57.4	66.4	55.0	70.5	52.1	70.0	65.2	77.9	47.8	69.4
Northern Mariana Islands	45 to 49	48.0	66.6	50.6	73.7	51.0	61.3	52.8	62.7	55.6	71.9	53.5	60.0	62.9	74.0	60.1	79.5	63.2	67.9	68.9	82.8	71.4	85.6	72.9	82.0

Norway	45 to 49	78.9	92.8	81.5	93.4	78.2	91.2	79.8	95.8	87.8	97.6	74.2	93.1	64.2	83.1	65.1	86.5	64.3	80.0	80.7	93.6	87.9	96.7	82.3	94.8
Oman	45 to 49	67.1	90.9	74.2	95.5	65.1	89.1	63.4	82.4	75.4	92.0	58.2	78.6	81.1	92.1	83.7	93.6	79.0	91.5	47.7	77.4	59.2	86.4	48.6	76.9
Pakistan	45 to 49	66.0	67.3	68.3	70.2	67.7	67.8	68.6	66.1	75.4	73.4	67.6	65.0	68.3	68.2	71.8	71.0	65.1	65.8	76.9	75.7	76.8	78.8	82.7	78.6
Palau	45 to 49	53.5	63.5	71.2	79.8	41.5	53.7	58.2	62.9	76.0	81.4	47.3	52.4	66.7	74.0	80.0	85.3	53.0	64.6	68.6	75.1	78.9	85.3	62.9	70.6
Palestine	45 to 49	62.6	80.0	69.1	84.8	57.9	75.0	52.2	67.5	67.0	79.2	43.8	59.5	89.1	95.5	90.8	96.7	87.1	94.2	39.0	63.9	51.7	74.2	34.8	60.2
Panama	45 to 49	63.1	74.7	70.5	83.6	57.9	64.3	60.9	69.6	73.4	86.0	53.7	54.9	72.6	72.6	76.0	78.4	69.3	65.3	60.0	82.1	70.3	87.5	54.8	78.3
Papua New Guinea	45 to 49	0.0	20.7	0.0	24.6	12.7	27.4	0.0	14.7	0.0	18.7	14.0	24.0	39.6	54.8	45.9	62.0	33.5	48.2	61.4	69.2	53.5	66.4	76.7	78.6
Paraguay	45 to 49	42.1	57.6	46.9	63.5	45.6	56.8	47.5	53.3	59.4	67.1	45.9	47.3	48.9	58.5	51.1	59.5	48.0	59.6	57.3	70.3	66.1	78.9	58.4	69.8
Peru	45 to 49	61.3	78.2	67.6	80.0	58.2	77.0	59.1	68.3	71.4	77.9	52.8	63.3	67.7	76.6	70.4	76.6	65.9	78.3	74.1	83.9	79.3	86.8	73.1	83.2
Philippines	45 to 49	33.5	47.2	57.4	63.8	16.5	37.1	21.2	47.0	51.9	66.0	3.3	37.1	68.7	69.8	76.8	75.7	58.7	63.6	58.5	62.1	75.1	75.1	46.2	56.3
Poland	45 to 49	51.9	73.0	66.4	86.6	42.8	62.6	54.2	67.6	70.5	86.1	45.2	55.3	56.4	70.4	65.4	79.3	45.2	60.7	34.8	67.1	61.9	83.1	21.0	59.7
Portugal	45 to 49	61.2	80.2	66.2	84.0	60.8	77.4	61.4	76.3	70.1	86.0	59.8	69.8	61.6	70.6	62.8	70.7	61.1	70.8	62.3	87.5	70.9	90.3	63.6	88.3
Puerto Rico	45 to 49	77.6	89.6	85.1	92.1	67.1	81.9	73.4	83.1	86.2	92.8	62.1	71.0	77.8	83.7	82.5	87.9	69.5	76.4	81.6	92.7	88.7	95.4	74.5	89.1
Qatar	45 to 49	83.2	96.6	83.1	100.0	84.7	96.3	81.7	91.8	84.9	97.7	81.6	90.6	77.4	88.1	85.3	96.2	72.3	83.5	84.9	92.4	84.0	97.4	89.4	92.8
Republic of Korea	45 to 49	62.9	83.2	69.6	87.1	61.3	80.9	70.7	86.3	81.3	93.4	63.9	81.4	38.7	70.9	49.6	76.0	26.6	66.2	74.0	88.4	79.4	92.1	78.4	90.9
Republic of Moldova	45 to 49	50.8	69.6	60.4	79.0	47.0	63.8	52.3	60.2	65.1	72.9	47.9	54.2	72.7	78.7	83.1	88.0	62.0	70.5	66.0	72.9	73.9	83.2	65.4	70.3
Romania	45 to 49	54.1	66.1	63.4	79.6	50.3	56.3	53.1	57.0	65.7	77.7	48.6	43.2	61.3	66.3	63.5	72.2	59.2	60.3	55.8	67.7	69.7	81.8	51.8	61.8
Russian Federation	45 to 49	60.0	63.3	70.9	76.8	52.7	53.3	58.7	54.9	71.1	72.8	53.0	44.6	64.1	61.0	72.2	69.6	56.3	52.6	37.8	54.2	60.9	75.3	24.6	41.6
Rwanda	45 to 49	30.4	59.9	46.8	70.7	19.2	50.9	39.2	51.2	60.0	69.5	25.4	38.4	59.1	76.0	69.5	82.8	47.7	70.0	65.9	82.9	69.4	85.3	70.5	84.6
Saint Kitts and Nevis	45 to 49	40.8	59.0	53.6	72.2	33.6	50.1	42.9	52.7	61.1	71.7	33.2	41.9	55.8	68.6	58.6	74.7	53.4	61.5	48.4	65.4	58.1	76.4	46.4	61.9
Saint Lucia	45 to 49	38.8	60.2	53.7	73.2	28.7	48.8	36.4	50.3	58.5	73.1	24.0	34.3	55.8	68.9	58.7	72.0	53.4	65.7	41.0	69.1	52.0	75.7	38.1	68.0
Saint Vincent and the Grenadines	45 to 49	45.3	51.4	58.8	65.9	34.7	40.2	32.0	42.9	56.5	65.3	16.3	28.9	67.8	67.1	70.1	71.4	66.1	62.0	59.3	62.5	67.0	70.0	57.8	61.5
Samoa	45 to 49	53.1	57.6	65.2	65.6	45.1	54.0	57.2	56.6	71.5	69.1	49.0	51.5	63.8	71.0	75.7	77.5	51.0	64.5	78.9	80.4	81.4	82.6	80.5	82.1
San Marino	45 to 49	81.0	94.0	84.5	93.2	79.0	92.4	75.2	93.5	82.6	93.4	71.2	92.4	81.1	89.4	83.9	90.9	78.9	88.7	79.9	92.8	86.8	94.8	81.0	93.5
Sao Tome and Principe	45 to 49	71.9	70.6	73.4	73.7	73.3	70.0	68.4	65.4	73.5	72.3	70.9	65.5	78.8	76.3	80.6	78.4	77.4	74.4	78.6	71.3	84.8	82.6	72.8	62.1
Saudi Arabia	45 to 49	47.8	57.3	48.1	61.6	52.9	58.5	45.0	52.8	55.1	65.6	44.9	48.5	77.0	81.4	77.6	81.4	76.2	81.3	22.3	26.0	16.6	31.9	37.1	33.3
Senegal	45 to 49	55.1	62.8	65.3	70.7	49.0	57.8	49.2	54.1	62.6	65.0	45.1	51.9	67.6	71.8	75.1	77.4	60.4	66.4	61.4	68.2	80.7	83.3	43.9	53.8
Serbia	45 to 49	59.5	71.8	67.3	79.8	55.7	65.3	61.0	64.6	72.6	79.3	55.6	54.1	54.4	65.1	56.8	66.6	52.5	64.2	53.9	64.7	68.1	78.0	49.2	58.7
Seychelles	45 to 49	50.9	66.4	66.2	78.6	44.3	59.9	53.2	62.4	67.4	76.7	47.5	55.7	70.3	83.3	78.5	88.4	61.6	78.3	54.0	68.4	73.0	82.7	47.5	63.9
Sierra Leone	45 to 49	55.7	59.5	66.5	67.3	50.1	55.5	53.1	55.2	65.8	66.2	48.8	52.5	70.2	71.7	77.7	76.0	64.0	67.9	56.5	59.1	81.1	80.4	38.0	40.2
Singapore	45 to 49	74.9	87.1	78.1	88.3	74.4	86.5	78.1	88.2	84.3	91.8	74.8	85.8	70.8	83.0	75.1	84.3	67.1	82.5	80.0	91.6	80.9	93.2	86.1	95.0
Slovakia	45 to 49	56.7	81.1	70.9	89.8	47.6	74.4	46.5	71.8	69.0	87.1	31.9	61.2	63.1	79.8	68.2	83.4	57.1	75.4	51.4	78.0	72.2	88.5	43.2	75.3
Slovenia	45 to 49	65.3	95.6	77.9	98.0	56.7	90.8	66.1	95.4	80.9	100.0	56.7	90.8	70.6	89.8	74.8	92.5	65.8	86.9	59.0	91.5	77.7	96.2	51.0	89.3
Solomon Islands	45 to 49	46.6	48.4	61.1	62.2	38.0	39.8	60.5	58.8	73.7	72.4	53.0	51.3	55.5	63.7	72.4	76.5	37.5	49.7	74.8	76.0	78.3	79.6	77.0	77.9
Somalia	45 to 49	34.5	48.9	53.1	63.8	23.7	39.0	36.5	40.9	58.2	61.9	25.5	29.3	60.5	69.9	72.9	78.7	50.1	62.8	75.6	81.2	78.6	84.3	78.9	83.1
South Sudan	45 to 49	64.5	65.5	62.3	66.6	40.8	43.8	56.6	43.4	63.1	58.0	36.8	25.0	78.2	78.7	78.1	79.5	64.6	67.8	76.6	78.1	81.2	84.0	78.8	80.1
Spain	45 to 49	47.6	53.0	80.9	88.7	69.8	85.2	44.9	35.9	82.3	93.3	67.8	82.7	70.3	73.1	80.0	82.6	71.6	77.8	76.9	80.0	83.7	92.4	76.8	91.3
Sri Lanka	45 to 49	73.9	86.8	75.6	83.9	52.2	67.7	72.7	87.5	73.2	79.6	41.7	54.3	75.9	80.0	88.0	91.7	72.6	80.4	75.9	89.0	75.6	83.4	56.4	71.0
Sudan	45 to 49	62.3	75.7	35.2	60.0	50.6	69.9	54.2	64.6	51.7	57.0	44.5	59.3	80.9	86.6	59.2	71.8	57.6	73.3	62.2	74.6	3.9	39.7	49.7	56.8
Suriname	45 to 49	37.8	62.4	56.3	64.9	39.0	36.4	40.5	51.4	61.9	65.9	37.6	28.4	58.1	72.0	68.2	71.6	56.0	56.7	20.5	42.7	70.9	76.7	60.2	59.9
Sweden	45 to 49	45.0	48.5	83.9	97.6	82.2	100.0	45.5	43.2	89.3	98.0	83.9	100.0	62.8	65.1	69.6	86.7	64.7	84.3	62.8	65.5	89.4	100.0	83.2	100.0
Switzerland	45 to 49	82.6	100.0	80.5	93.9	80.6	92.2	85.6	99.2	84.3	97.3	80.9	98.3	66.9	85.1	68.8	89.3	68.8	84.0	83.1	99.8	85.0	96.3	82.2	95.1
Syrian Arab Republic	45 to 49	80.1	93.8	65.2	76.9	64.2	71.5	81.2	98.5	71.3	75.3	63.0	63.0	68.0	86.4	80.8	90.2	79.3	91.3	80.6	93.7	48.3	63.2	47.4	55.8
Taiwan (Province of China)	45 to 49	62.5	73.1	72.9	89.7	52.3	72.7	63.6	66.3	78.1	92.7	54.9	72.4	79.8	90.6	82.0	87.0	78.8	78.9	42.9	55.4	74.6	89.4	65.6	81.2
Tajikistan	45 to 49	60.4	81.1	56.2	70.7	49.2	63.6	63.7	80.0	64.5	69.3	54.3	59.2	80.2	83.2	71.3	81.2	71.1	75.5	66.7	84.0	47.8	68.8	23.2	57.4
Thailand	45 to 49	49.4	65.8	64.9	76.5	48.7	50.7	54.9	60.4	70.5	76.4	56.0	51.7	71.4	78.2	67.9	76.8	39.8	47.8	29.8	60.1	71.3	80.0	64.6	64.9
Timor-Leste	45 to 49	54.5	61.2	38.6	51.8	32.7	33.9	59.7	60.0	49.1	56.8	34.1	33.1	55.0	62.2	63.5	70.7	53.9	57.9	64.6	69.5	64.4	70.9	58.4	49.5
Togo	45 to 49	30.5	38.8	61.7	67.9	51.9	46.7	34.7	39.4	62.1	64.7	50.5	42.9	58.7	64.3	73.3	76.4	61.6	59.5	57.1	56.0	79.7	82.8	47.7	40.0

Tokelau	45 to 49	54.4	54.8	54.9	66.7	35.0	51.2	51.3	49.4	65.8	68.4	40.1	44.0	67.5	67.5	67.4	77.9	44.3	64.0	62.8	59.0	70.9	79.4	68.9	76.9
Tonga	45 to 49	44.1	58.3	71.9	75.9	63.8	71.2	50.4	53.5	73.9	73.4	58.5	62.5	58.2	71.4	80.2	84.1	70.2	77.8	67.5	76.7	84.7	86.2	82.1	86.6
Trinidad and Tobago	45 to 49	66.9	72.8	60.9	70.2	57.3	59.7	63.6	64.8	60.8	65.2	42.1	38.6	75.4	80.8	62.1	60.7	50.8	48.0	82.4	85.2	69.2	78.1	59.1	65.9
Tropical Latin America	45 to 49	56.5	63.4	46.3	62.6	25.3	53.7	46.8	48.1	59.0	67.4	27.6	43.1	57.0	55.0	50.6	54.9	31.8	50.0	60.2	69.2	52.8	74.0	17.5	60.8
Tunisia	45 to 49	68.5	79.1	69.9	80.3	70.1	78.8	63.5	71.6	71.4	77.3	62.7	71.0	79.3	87.2	82.1	88.6	76.2	85.7	51.8	62.8	52.8	67.8	59.3	65.4
Turkey	45 to 49	66.6	85.8	73.6	88.5	63.1	82.0	66.8	78.8	76.0	85.6	62.9	74.2	56.7	77.4	66.7	82.5	45.0	71.3	61.7	81.5	71.2	85.8	60.3	79.9
Turkmenistan	45 to 49	57.5	57.0	67.7	71.8	51.5	47.7	61.6	53.0	73.2	71.2	56.1	43.2	61.5	60.3	70.0	70.9	53.3	51.5	40.5	48.1	57.4	64.7	33.2	41.6
Tuvalu	45 to 49	27.2	45.6	41.4	56.6	20.3	41.7	40.2	48.8	56.9	62.4	34.5	44.7	46.3	62.7	59.2	71.7	28.1	54.4	59.1	67.7	64.8	73.0	58.7	68.4
Uganda	45 to 49	54.3	64.5	71.6	77.0	43.2	55.3	46.5	53.1	68.1	71.1	35.2	43.7	70.1	76.4	82.0	84.7	61.0	69.8	82.2	84.5	86.2	88.2	82.9	84.8
Ukraine	45 to 49	66.4	68.8	73.7	79.7	62.3	62.0	62.0	63.2	74.2	78.1	55.1	54.1	64.5	67.6	70.8	76.4	58.1	60.0	58.4	63.8	70.7	79.7	55.5	58.4
United Arab Emirates	45 to 49	73.7	88.4	76.4	89.6	74.6	89.1	71.2	87.5	80.1	89.7	68.4	87.9	68.4	86.8	68.4	87.9	67.6	86.1	64.7	87.2	69.1	89.3	70.1	91.2
United Kingdom	45 to 49	76.5	87.8	79.1	88.7	75.3	85.7	80.1	88.1	85.6	91.9	76.8	84.3	59.1	75.6	63.4	79.2	54.6	72.0	79.2	91.2	86.5	94.1	78.7	91.5
United Republic of Tanzania	45 to 49	54.8	72.1	66.1	76.5	48.7	69.9	51.3	61.8	66.5	71.9	44.5	58.2	71.7	79.5	79.4	83.2	65.5	76.7	80.8	83.4	83.7	87.0	83.1	84.3
United States Virgin Islands	45 to 49	53.8	68.3	63.8	87.8	46.2	47.6	48.2	57.9	65.1	86.9	38.7	32.4	60.4	73.9	65.8	86.3	52.5	54.6	65.1	78.3	71.9	92.1	63.1	67.2
United States of America	45 to 49	85.9	99.6	86.1	97.0	85.2	98.4	77.2	86.0	84.2	90.6	72.7	82.0	62.3	77.2	66.7	81.9	56.0	69.7	91.0	100.0	93.0	98.6	90.9	98.9
Uruguay	45 to 49	63.8	76.1	69.8	79.4	61.6	74.0	63.8	79.7	75.9	86.8	56.1	73.4	53.4	64.5	60.1	70.7	44.2	56.1	73.9	81.7	80.4	85.8	76.2	83.4
Uzbekistan	45 to 49	58.4	72.9	66.2	80.2	55.5	67.7	56.4	73.3	68.1	81.6	52.1	68.9	78.7	77.5	82.8	87.7	75.1	68.8	50.9	63.9	61.2	76.0	51.3	60.0
Vanuatu	45 to 49	43.5	52.8	60.1	65.1	34.6	46.0	49.4	54.5	66.8	68.5	40.5	48.4	54.1	64.4	69.3	74.9	39.7	54.5	71.0	76.8	78.4	82.1	68.6	75.6
Venezuela (Bolivarian Republic of)	45 to 49	56.8	62.3	66.2	71.5	49.6	53.3	57.7	57.1	71.4	72.9	49.0	45.0	66.7	67.9	71.0	72.4	61.4	61.6	68.0	79.4	76.5	83.9	62.8	77.0
Viet Nam	45 to 49	39.6	47.5	57.3	69.3	30.3	37.2	46.2	47.8	63.3	69.8	38.5	36.8	64.6	72.0	75.9	82.4	51.5	62.0	56.4	55.9	72.3	77.6	48.9	49.3
Yemen	45 to 49	27.0	46.6	34.0	50.6	30.9	50.2	31.2	37.5	51.3	51.2	23.4	37.7	52.8	63.7	62.3	69.3	41.3	57.4	6.7	19.1	0.0	24.3	28.5	29.1
Zambia	45 to 49	48.0	49.8	58.4	63.8	42.0	42.1	46.6	41.7	61.6	58.4	40.8	35.5	66.8	68.7	72.0	74.3	62.3	64.4	79.1	75.8	81.2	81.4	82.5	75.8
Zimbabwe	45 to 49	70.8	47.3	74.7	57.8	69.0	41.4	49.4	4.2	57.2	21.2	51.6	7.6	75.5	61.7	77.7	65.1	74.4	59.2	84.4	69.6	87.6	78.2	84.7	64.2
Afghanistan	50 to 54	26.4	41.2	38.2	45.0	23.6	46.5	51.7	51.4	62.4	55.4	35.1	47.3	47.7	58.7	55.3	60.3	40.5	58.4	5.3	14.2	1.4	15.5	28.6	30.5
Albania	50 to 54	62.2	78.4	69.8	84.3	60.3	75.0	68.1	77.3	70.6	79.4	63.8	73.2	75.6	86.8	75.6	86.1	76.7	87.6	77.0	84.4	83.0	89.1	79.5	84.3
Algeria	50 to 54	67.8	76.6	66.9	76.3	74.8	81.4	74.7	76.8	72.7	74.1	77.5	79.5	78.1	85.3	75.9	82.7	81.1	88.2	53.1	63.9	48.9	62.1	69.5	75.4
American Samoa	50 to 54	54.2	58.2	69.2	67.7	47.4	54.0	66.2	64.7	73.2	68.8	58.6	59.0	71.8	73.7	70.5	70.4	74.7	78.0	74.4	77.4	78.1	78.0	77.8	82.6
Andorra	50 to 54	79.7	89.8	81.6	89.9	81.9	92.3	84.2	95.2	84.3	92.8	82.2	95.4	70.1	83.7	63.2	79.1	78.3	89.9	82.6	90.9	85.1	90.9	88.5	96.7
Angola	50 to 54	49.2	60.8	62.1	70.0	41.2	54.9	57.3	59.6	63.5	63.8	49.4	53.9	69.1	74.9	71.6	75.9	69.5	75.7	71.6	75.0	76.6	79.3	71.8	74.8
Antigua and Barbuda	50 to 54	44.7	67.5	66.7	75.6	25.6	61.1	54.7	67.2	68.8	73.8	38.3	57.5	57.6	74.6	68.7	71.8	45.6	78.8	58.4	77.1	72.3	80.2	49.3	78.4
Argentina	50 to 54	58.5	77.2	68.6	83.0	54.8	75.3	72.5	81.6	78.6	86.1	64.5	75.0	55.1	75.0	59.5	76.1	50.4	74.9	74.7	87.5	79.9	88.7	79.6	93.2
Armenia	50 to 54	71.2	78.3	75.9	89.0	70.9	71.6	71.6	78.9	73.5	88.0	68.1	67.0	75.3	81.7	74.7	86.9	76.8	79.2	64.9	64.6	70.0	79.4	68.5	59.7
Australia	50 to 54	76.6	88.4	80.8	88.8	77.3	90.9	81.4	92.0	82.6	91.7	78.7	90.3	61.1	78.9	59.9	78.5	63.7	80.3	82.1	91.3	85.5	90.0	88.4	97.8
Austria	50 to 54	77.9	89.5	84.0	90.7	76.4	91.1	83.8	95.4	85.6	93.7	80.3	94.9	81.3	88.6	80.4	87.7	83.3	90.7	71.6	90.5	83.6	90.3	71.4	97.1
Azerbaijan	50 to 54	58.0	72.4	67.7	78.4	53.4	70.0	67.8	74.9	71.2	76.4	62.7	71.8	82.1	90.0	84.2	91.0	81.3	89.5	59.8	76.2	70.7	81.3	56.7	77.0
Bahamas	50 to 54	50.4	59.5	62.5	70.5	42.2	51.8	58.2	61.3	64.9	68.8	49.2	51.7	66.4	68.0	66.4	67.2	66.8	69.5	63.0	70.2	69.1	75.6	62.8	70.0
Bahrain	50 to 54	59.7	88.3	57.8	88.9	67.1	90.8	67.8	89.8	67.1	87.6	68.1	90.4	68.2	90.0	58.9	89.2	76.5	91.3	43.9	73.6	38.9	76.4	58.2	77.1
Bangladesh	50 to 54	26.1	50.2	43.4	56.7	20.8	50.9	53.3	63.7	62.4	67.0	43.4	58.8	38.2	63.2	48.4	58.2	37.6	69.0	43.2	59.1	52.6	64.1	47.7	64.6
Barbados	50 to 54	60.3	71.8	67.9	78.5	57.1	67.8	65.6	70.9	67.9	75.7	61.5	63.5	63.3	71.2	64.6	72.2	62.6	70.7	62.0	76.5	66.5	78.7	65.7	80.0
Belarus	50 to 54	58.9	67.4	66.3	77.6	57.7	62.7	69.8	71.1	70.9	76.6	67.3	64.0	54.9	59.7	57.3	67.3	54.5	55.3	41.8	59.9	55.4	74.1	41.6	57.3
Belgium	50 to 54	74.0	85.7	80.2	89.3	72.8	84.9	80.5	90.0	82.6	92.4	76.4	84.9	76.0	78.2	76.4	76.7	77.0	81.2	75.3	88.1	80.5	90.2	80.7	92.8
Belize	50 to 54	53.1	65.1	58.0	70.5	54.8	64.5	57.9	65.9	58.8	66.6	56.1	63.5	71.4	75.5	68.5	72.8	75.7	79.4	72.7	75.9	74.6	78.1	77.2	80.3
Benin	50 to 54	47.7	58.1	54.1	66.5	48.5	54.8	57.9	64.2	55.5	64.1	59.8	62.9	65.7	73.2	64.0	73.0	68.9	75.0	60.6	63.8	74.6	79.0	48.5	49.3
Bermuda	50 to 54	68.2	85.3	78.9	92.6	59.7	78.9	73.2	85.5	80.6	94.1	62.6	75.4	72.1	83.7	76.4	88.6	67.1	78.9	62.8	83.3	75.0	88.5	56.4	82.0
Bhutan	50 to 54	52.7	66.9	57.3	71.1	55.1	68.0	66.2	72.2	69.2	74.0	61.8	68.8	59.3	70.4	57.4	68.4	63.2	73.8	64.3	73.3	63.1	76.0	75.1	78.9
Bolivia (Plurinational State of)	50 to 54	34.1	56.5	45.5	62.3	28.9	55.8	47.4	57.2	53.3	61.4	40.1	51.4	45.0	57.2	41.7	51.7	49.9	65.3	46.3	68.3	57.6	74.3	40.9	66.9
Bosnia and Herzegovina	50 to 54	67.1	81.4	73.4	86.9	65.8	79.5	63.8	77.5	67.5	82.9	58.4	70.6	49.1	74.4	48.4	74.0	50.9	75.4	56.9	71.8	65.8	79.8	58.6	72.5
Botswana	50 to 54	50.9	70.2	60.5	77.4	47.9	66.4	56.5	70.1	56.0	72.8	54.7	65.9	73.2	83.3	76.1	85.2	72.7	83.1	73.3	81.5	78.1	84.7	73.9	82.1

Brazil	50 to 54	37.6	59.1	51.9	67.8	30.6	55.1	54.9	63.0	63.5	70.6	43.5	52.5	41.7	51.5	43.9	50.7	38.0	52.5	35.3	66.4	55.4	76.4	28.9	64.6
Brunei Darussalam	50 to 54	67.8	71.1	66.8	74.0	73.2	73.3	79.1	77.4	76.9	79.1	79.7	74.0	57.4	64.8	50.5	63.7	66.1	67.9	75.9	81.0	73.3	81.4	86.9	89.0
Bulgaria	50 to 54	49.4	61.2	63.4	75.8	43.7	53.2	63.4	67.8	69.3	77.1	56.2	57.8	62.4	65.6	65.2	68.5	61.3	64.4	45.7	41.0	62.8	65.0	42.8	31.1
Burkina Faso	50 to 54	46.0	52.9	61.1	69.6	35.0	39.9	55.9	60.3	62.8	68.8	46.9	49.8	67.8	71.9	74.9	79.7	63.9	67.8	65.2	66.2	80.4	83.3	48.7	48.5
Burundi	50 to 54	35.3	53.8	52.6	65.0	21.2	48.3	50.9	55.7	61.0	62.7	37.5	47.1	58.9	73.0	64.6	76.8	55.9	73.0	63.7	76.6	66.8	77.9	68.8	81.6
Cabo Verde	50 to 54	66.3	67.1	77.7	80.3	53.2	59.1	73.7	73.3	78.1	76.9	66.0	68.4	78.1	80.5	83.1	84.1	72.4	79.0	62.7	56.6	84.0	83.6	27.6	33.7
Cambodia	50 to 54	22.9	44.7	32.3	54.9	24.3	41.0	49.1	59.4	51.7	63.2	47.1	54.4	54.2	70.0	54.6	71.3	54.2	69.6	47.2	62.0	53.8	67.6	51.8	64.9
Cameroon	50 to 54	46.4	51.4	56.9	63.0	42.0	46.0	58.8	60.2	62.0	63.0	54.5	55.8	66.0	68.7	68.1	70.7	66.3	69.2	63.7	59.1	78.3	78.2	49.3	43.3
Canada	50 to 54	86.4	94.2	88.6	94.3	87.0	95.7	90.9	97.7	91.9	95.8	87.3	96.7	73.6	84.0	73.4	85.3	74.1	82.3	88.3	94.6	89.3	93.2	93.7	100.0
Central African Republic	50 to 54	31.2	36.0	49.3	54.8	18.6	23.9	48.7	45.1	56.7	54.5	39.0	34.3	57.2	62.0	61.8	68.1	55.5	60.2	59.7	65.8	67.5	73.5	57.2	63.9
Chad	50 to 54	49.1	47.3	56.6	57.5	48.1	43.9	59.4	57.3	59.2	58.1	59.4	55.0	64.7	62.6	63.4	60.9	67.5	66.1	61.9	55.0	75.2	74.7	48.5	38.2
Chile	50 to 54	63.9	83.0	71.5	88.0	61.5	80.6	73.8	88.7	79.3	92.2	64.9	81.7	63.0	76.1	65.1	78.3	60.6	73.9	73.7	87.5	77.8	90.2	78.9	90.9
China	50 to 54	30.2	62.2	45.9	73.3	23.1	57.2	58.9	70.5	65.3	75.8	50.8	64.0	26.5	73.9	26.4	77.1	29.2	72.6	23.7	59.6	45.1	71.4	15.6	58.5
Colombia	50 to 54	63.1	78.9	68.7	82.9	61.5	75.5	68.8	82.1	71.9	85.5	63.2	74.0	67.4	72.6	66.5	72.0	69.1	74.1	65.7	81.4	71.2	85.3	66.7	80.0
Comoros	50 to 54	53.4	67.2	63.1	73.7	48.1	63.9	59.0	64.8	63.4	68.3	53.2	59.7	73.0	80.0	73.7	80.5	74.5	81.4	74.9	81.6	77.0	82.5	78.3	85.0
Congo	50 to 54	47.1	58.4	57.8	64.4	41.7	57.8	55.9	56.2	59.2	57.3	51.5	54.8	67.0	73.0	67.0	69.7	68.9	77.5	68.7	73.3	74.0	75.8	68.4	76.4
Cook Islands	50 to 54	59.9	78.6	72.1	86.1	52.7	74.1	69.2	79.0	75.6	84.8	61.1	71.8	72.0	89.4	73.6	88.8	72.2	91.1	75.9	87.3	78.4	87.0	79.8	91.6
Costa Rica	50 to 54	77.3	85.0	80.8	89.8	75.8	79.4	80.0	86.8	81.6	92.4	75.5	77.3	79.2	78.2	78.5	80.0	81.2	76.1	77.7	87.9	79.7	89.5	80.8	88.2
Coted'Ivoire	50 to 54	51.7	53.6	65.0	66.5	46.8	48.2	62.2	61.0	65.4	63.8	57.2	56.5	65.6	69.6	69.4	72.3	66.1	70.4	52.7	49.9	77.3	78.6	39.1	30.6
Croatia	50 to 54	54.0	76.9	68.0	87.6	48.2	72.5	64.6	78.1	74.0	89.7	54.1	66.1	51.2	71.4	54.2	74.3	49.1	68.8	56.7	78.5	69.5	84.8	58.8	83.3
Cuba	50 to 54	58.8	70.8	66.9	80.7	54.9	63.7	65.6	70.7	70.0	78.2	58.9	61.0	69.2	79.5	67.9	79.6	71.3	79.6	61.7	65.3	70.1	77.3	59.5	60.0
Cyprus	50 to 54	67.0	89.1	72.4	91.9	66.7	87.9	79.0	93.0	82.3	94.1	72.6	88.7	61.2	83.1	58.2	82.0	66.2	85.5	72.9	91.2	75.9	94.2	79.6	93.0
Czechia	50 to 54	62.3	82.9	74.0	91.6	57.4	78.9	70.1	86.3	77.4	94.1	61.7	78.2	58.5	78.8	58.7	80.4	58.8	77.9	49.8	79.6	67.0	88.2	47.6	80.8
Democratic People's Republic of Korea	50 to 54	41.3	37.8	52.5	51.6	37.9	32.2	61.2	53.1	65.6	60.2	55.1	44.4	54.2	61.6	54.5	62.5	55.4	62.4	44.7	42.4	54.5	55.7	47.4	42.3
Democratic Republic of the Congo	50 to 54	53.9	56.3	62.2	66.1	50.7	51.5	62.0	59.6	64.1	64.0	58.9	53.6	73.0	74.7	72.8	75.6	74.8	76.1	71.2	75.0	74.2	77.9	74.0	77.7
Denmark	50 to 54	70.5	87.1	75.3	90.5	70.8	86.8	76.1	91.6	78.5	93.4	71.9	87.9	64.9	82.9	64.2	82.1	67.0	85.0	71.9	89.4	77.3	91.2	77.6	95.1
Djibouti	50 to 54	55.5	64.5	67.2	74.7	49.6	60.1	60.3	61.9	65.3	66.7	53.9	55.7	72.7	77.2	76.7	80.7	72.8	77.7	77.5	78.0	80.0	82.8	80.7	79.1
Dominica	50 to 54	49.6	56.1	62.1	71.2	36.8	43.0	52.8	52.2	60.6	64.4	41.4	37.1	63.8	68.1	65.9	71.5	59.8	65.4	60.3	62.0	68.2	73.4	54.1	54.5
Dominican Republic	50 to 54	46.9	58.6	57.2	70.3	42.0	50.9	55.2	62.4	61.3	69.8	47.1	53.1	59.9	69.5	57.4	68.5	63.4	71.4	57.2	66.3	65.2	75.7	56.0	63.2
Ecuador	50 to 54	61.2	77.3	69.0	81.6	56.7	75.1	62.5	77.1	67.6	80.2	55.3	71.6	72.4	71.7	71.3	71.8	74.6	72.7	62.2	83.5	67.6	86.9	63.2	83.4
Egypt	50 to 54	41.8	62.9	53.9	70.7	35.7	60.5	46.8	64.1	59.0	70.1	30.4	56.2	61.1	80.3	68.7	82.5	53.0	78.5	4.9	31.5	15.9	45.3	11.7	31.3
El Salvador	50 to 54	59.5	72.1	68.4	79.9	52.8	60.8	68.9	70.6	73.0	77.8	61.9	55.8	68.4	75.1	69.5	75.8	67.1	72.9	65.5	76.2	75.0	83.5	58.6	66.5
Equatorial Guinea	50 to 54	38.1	65.4	54.3	71.9	27.0	61.8	49.4	62.6	56.3	65.3	41.0	58.9	62.2	80.8	67.6	79.4	60.1	83.5	65.6	73.9	73.1	78.2	62.6	71.7
Eritrea	50 to 54	27.0	51.8	46.7	65.3	14.8	41.7	43.5	54.0	52.1	62.0	33.4	44.0	57.9	72.6	67.2	77.1	53.6	71.0	68.9	77.1	72.1	79.9	72.5	78.4
Estonia	50 to 54	62.0	81.6	73.5	91.9	57.0	73.1	69.5	81.1	75.6	91.8	62.1	69.5	69.6	76.8	80.2	88.9	59.9	68.1	45.7	75.1	62.6	88.7	43.5	66.5
Eswatini	50 to 54	42.0	41.4	59.9	59.4	29.0	29.8	48.0	42.9	56.2	47.5	38.4	36.0	71.1	70.6	76.6	74.2	68.2	69.1	73.9	69.0	79.6	77.5	71.8	65.2
Ethiopia	50 to 54	40.7	62.8	54.2	70.4	33.5	59.8	59.4	65.0	65.2	68.1	51.6	60.3	71.3	80.9	75.4	82.0	70.7	82.0	76.1	83.3	75.7	82.2	84.1	89.2
Fiji	50 to 54	54.4	63.2	63.4	70.6	50.3	59.8	60.5	60.0	65.8	65.9	53.4	52.1	70.5	76.5	71.0	76.3	70.8	78.3	75.1	79.7	76.2	79.9	80.6	85.1
Finland	50 to 54	73.0	88.5	78.7	90.4	72.7	89.3	79.2	93.2	82.8	93.8	74.2	90.9	72.6	82.1	71.0	81.9	75.7	83.8	67.5	90.2	75.6	91.2	72.0	95.4
France	50 to 54	77.7	85.9	87.3	89.1	73.0	86.2	77.3	90.4	83.8	93.6	69.0	85.3	79.3	81.7	80.7	81.6	79.3	83.1	80.3	87.6	90.1	89.1	80.2	93.3
Gabon	50 to 54	59.3	66.7	70.2	75.8	51.9	61.2	64.5	65.2	69.4	69.5	57.9	59.5	76.2	79.8	79.1	81.3	75.4	80.3	77.2	78.1	80.2	81.7	78.7	79.1
Gambia	50 to 54	48.8	48.4	60.0	58.7	45.5	44.7	56.9	53.7	56.7	54.3	54.3	51.8	66.3	64.9	67.0	62.6	68.6	68.7	51.5	49.6	76.2	73.2	35.8	29.3
Georgia	50 to 54	58.0	68.6	71.0	84.0	50.0	59.5	71.5	76.6	75.8	84.1	65.5	69.3	73.9	68.5	78.6	78.1	72.2	65.1	67.9	62.5	75.9	80.4	64.5	52.7
Germany	50 to 54	78.5	87.1	82.9	90.7	78.2	86.7	81.7	91.0	85.1	92.8	76.2	86.9	74.2	82.4	72.9	79.6	77.1	86.9	77.3	86.2	82.5	89.4	80.7	90.6
Ghana	50 to 54	49.3	57.5	60.2	69.4	43.2	49.4	55.4	59.4	57.6	62.0	52.6	55.4	62.9	68.7	64.3	69.9	63.7	69.2	54.9	56.3	76.4	79.4	30.2	32.0
Greece	50 to 54	75.8	80.7	80.8	88.0	75.1	77.4	82.4	82.8	83.9	88.2	78.9	76.0	78.1	80.5	78.4	82.7	79.2	79.3	78.9	87.3	82.4	90.2	83.9	91.5
Greenland	50 to 54	63.3	73.4	70.1	79.6	63.2	72.2	67.1	73.0	76.9	81.0	56.4	64.2	0.0	52.4	0.0	53.2	0.0	51.7	77.9	87.6	84.0	90.4	81.9	91.8
Grenada	50 to 54	31.5	63.1	44.4	72.3	25.5	58.4	42.0	63.0	48.9	69.2	33.6	55.1	58.5	68.2	51.8	68.7	67.7	68.9	20.3	69.0	35.4	75.1	15.8	69.7

Guam	50 to 54	72.1	77.0	78.2	79.2	70.1	78.3	76.8	76.3	79.6	76.0	72.1	74.9	77.2	81.9	75.4	76.9	80.3	87.6	78.6	84.7	80.2	83.2	83.2	90.4
Guatemala	50 to 54	65.1	74.1	69.2	77.9	65.1	72.3	75.2	76.4	74.8	77.3	74.7	73.5	83.8	82.3	83.3	82.2	85.1	83.0	69.5	82.6	74.0	84.8	69.2	82.8
Guinea	50 to 54	48.3	53.3	51.0	60.1	54.1	52.8	59.8	62.1	56.6	61.1	64.1	62.5	68.0	70.0	62.3	66.3	74.0	74.3	59.9	57.0	72.1	75.7	50.9	40.6
Guinea-Bissau	50 to 54	26.2	35.5	44.2	50.4	14.6	26.5	45.5	46.4	51.0	50.2	38.8	41.9	45.6	51.6	50.8	52.2	43.1	53.0	39.7	42.5	69.8	71.1	5.0	9.7
Guyana	50 to 54	12.5	42.7	26.2	55.5	9.7	35.6	38.3	51.1	42.2	58.9	33.3	41.0	49.0	57.9	53.9	61.9	45.2	53.9	26.6	52.1	44.6	64.9	21.5	46.4
Haiti	50 to 54	2.3	22.3	15.1	34.2	1.3	18.6	38.9	39.7	46.9	49.0	28.8	27.0	15.7	34.6	6.9	27.3	26.5	44.0	9.5	35.9	14.4	41.6	23.4	43.0
Honduras	50 to 54	40.4	30.6	51.9	41.1	33.4	26.9	44.1	28.0	51.5	37.1	34.3	17.5	50.2	33.1	49.6	27.2	50.9	40.4	48.9	49.0	60.4	59.0	43.2	45.3
Hungary	50 to 54	59.7	74.1	71.6	85.5	54.0	67.3	67.1	76.7	73.1	87.2	59.7	66.1	61.2	72.9	62.7	72.3	58.4	74.1	44.2	63.4	63.0	77.9	39.3	59.7
Iceland	50 to 54	80.7	93.0	84.2	94.2	81.1	93.9	87.5	100.0	87.9	99.3	85.1	98.0	78.2	90.4	75.7	90.2	82.3	91.4	81.6	93.3	84.7	92.6	87.7	99.4
India	50 to 54	47.5	60.4	57.6	68.6	45.0	58.3	63.5	71.1	69.7	75.1	55.5	65.4	54.4	64.4	60.2	67.3	53.4	64.8	59.5	70.2	64.0	75.8	65.8	73.1
Indonesia	50 to 54	47.5	48.2	56.0	59.2	45.3	43.1	63.0	58.5	66.7	63.3	57.9	52.0	64.1	67.8	63.9	67.4	65.2	69.3	62.6	58.3	69.1	69.4	63.5	54.3
Iran (Islamic Republic of)	50 to 54	65.5	83.3	72.2	87.1	63.4	82.5	70.4	82.9	74.1	84.9	64.6	79.0	76.0	91.0	78.0	92.2	74.6	89.7	28.5	61.5	41.1	69.2	29.4	62.5
Iraq	50 to 54	40.4	57.5	53.4	67.7	34.8	53.3	57.8	66.1	64.3	70.4	49.7	60.5	64.0	74.7	66.4	74.5	61.0	75.1	0.0	29.8	17.6	45.1	0.0	28.2
Ireland	50 to 54	77.2	90.7	81.3	92.6	77.1	90.7	86.1	99.6	86.8	98.9	83.3	97.4	71.6	83.9	72.4	85.5	72.2	83.3	78.1	91.5	82.3	92.2	83.4	96.1
Israel	50 to 54	74.2	88.6	76.9	92.2	76.3	88.1	73.9	84.2	76.4	91.2	69.7	75.8	81.7	88.5	79.8	89.5	85.5	88.8	79.8	92.9	78.9	92.3	88.9	98.6
Italy	50 to 54	72.7	87.0	78.4	92.8	71.4	84.9	77.6	88.1	81.4	92.4	71.5	82.3	70.9	81.7	70.6	81.6	72.6	83.2	76.0	90.7	80.1	94.8	80.6	94.5
Jamaica	50 to 54	43.7	61.4	53.2	69.9	40.6	56.6	56.5	67.7	61.2	73.0	50.2	59.9	61.7	72.4	58.0	70.8	67.3	75.2	44.9	64.7	52.1	71.5	47.4	64.6
Japan	50 to 54	72.8	82.6	78.6	87.4	72.4	82.9	82.6	88.6	86.6	92.0	77.1	84.9	64.3	78.8	65.0	80.6	65.8	79.1	78.4	88.6	81.7	91.8	85.1	94.8
Jordan	50 to 54	66.3	81.7	68.5	83.6	69.7	83.7	69.0	82.2	70.1	81.9	67.1	80.7	91.5	100.0	90.2	99.8	93.4	100.0	45.1	71.4	48.6	72.3	54.0	79.1
Kazakhstan	50 to 54	65.7	67.1	73.1	77.5	62.9	60.8	70.9	67.2	73.5	73.5	66.5	58.9	63.3	66.2	62.4	68.2	65.7	66.7	55.1	65.0	66.9	76.0	52.2	61.3
Kenya	50 to 54	67.5	66.2	71.6	72.9	68.2	63.9	70.6	65.6	71.1	67.4	68.9	62.2	80.8	80.3	80.0	79.9	83.0	82.2	84.3	82.5	82.3	82.7	91.6	87.3
Kiribati	50 to 54	56.0	56.0	69.7	71.1	50.3	48.3	74.3	70.3	77.6	75.5	69.8	64.2	69.2	75.3	79.9	81.8	60.4	71.8	74.2	73.2	80.2	81.4	74.3	69.9
Kuwait	50 to 54	84.3	92.2	85.5	100.0	86.8	89.8	86.6	93.8	87.4	100.0	84.8	89.2	87.3	98.5	83.5	100.0	90.4	97.2	68.4	80.0	66.9	90.2	76.3	79.5
Kyrgyzstan	50 to 54	52.9	59.0	62.3	72.4	49.7	51.2	66.4	69.9	67.8	75.8	63.4	62.7	57.4	63.4	63.9	67.0	53.9	62.8	46.4	35.6	58.6	60.1	43.7	22.4
Lao People's Democratic Republic	50 to 54	11.9	43.0	23.8	53.7	11.3	39.7	43.0	57.4	47.6	61.0	37.8	52.3	37.8	66.9	35.9	67.9	41.1	67.4	25.6	54.2	35.3	66.1	30.9	52.1
Latvia	50 to 54	56.7	70.2	67.9	80.7	51.5	65.2	64.9	69.0	69.6	75.6	58.5	60.7	60.4	70.0	69.5	78.8	53.3	64.2	43.6	62.0	61.0	77.5	38.8	58.5
Lebanon	50 to 54	52.9	86.5	64.5	89.9	47.4	85.6	55.6	85.3	69.4	88.3	36.6	79.1	52.7	88.5	58.2	90.2	46.3	85.7	41.9	82.1	49.6	84.7	46.7	85.9
Lesotho	50 to 54	45.1	23.9	62.0	49.1	25.8	0.0	48.9	33.8	57.6	47.2	37.8	18.4	72.3	60.7	78.8	68.5	64.2	54.0	78.3	61.3	81.9	72.8	74.1	49.0
Liberia	50 to 54	47.8	49.7	52.3	55.8	51.0	50.6	58.6	55.3	55.6	53.2	61.5	56.7	67.6	69.0	62.2	64.4	73.3	73.9	56.6	54.3	71.5	72.9	45.8	39.3
Libya	50 to 54	75.0	75.8	73.6	76.7	81.3	79.0	77.6	74.0	75.0	71.5	80.7	77.6	82.9	84.4	79.2	79.8	86.7	89.3	57.9	54.3	57.2	59.6	67.7	57.6
Lithuania	50 to 54	63.9	74.1	72.0	83.1	60.7	69.5	68.4	74.8	70.3	81.7	65.2	66.5	65.3	70.0	73.4	76.2	58.2	65.0	55.5	70.2	66.5	81.1	54.9	69.1
Luxembourg	50 to 54	67.7	88.4	73.7	92.9	67.1	87.0	71.9	91.9	75.3	93.4	66.7	87.9	68.2	84.6	63.4	83.7	74.4	86.9	66.5	90.3	73.3	94.1	70.6	94.2
Madagascar	50 to 54	44.7	51.5	59.0	61.3	34.8	46.7	57.1	54.7	63.9	59.3	48.2	48.8	65.2	70.0	69.2	69.9	64.7	72.5	72.0	75.7	77.1	78.2	70.8	77.8
Malawi	50 to 54	49.3	45.9	57.5	59.9	47.5	39.2	54.1	40.0	57.5	47.1	49.6	31.3	68.3	67.1	68.8	69.6	70.2	68.0	74.1	68.9	75.5	75.8	79.1	69.2
Malaysia	50 to 54	55.8	67.8	64.6	75.1	53.2	65.2	68.3	72.9	71.5	75.4	63.5	68.7	73.1	81.3	75.1	81.1	72.2	82.1	61.5	69.8	67.4	75.5	65.3	72.0
Maldives	50 to 54	38.9	75.6	39.2	79.6	46.9	76.6	54.6	79.6	49.3	75.9	58.0	79.7	64.0	87.3	60.2	87.2	69.3	88.6	52.9	78.2	51.8	81.4	65.4	83.0
Mali	50 to 54	43.1	53.2	43.1	54.2	54.2	59.7	52.2	57.0	46.6	49.1	61.3	63.9	65.3	72.4	53.7	62.9	75.3	80.2	64.6	64.4	68.4	72.6	66.4	60.1
Malta	50 to 54	74.3	91.3	78.6	93.6	74.0	91.0	80.8	94.0	81.3	93.1	78.3	92.6	83.3	89.5	81.4	89.1	86.9	90.9	72.3	91.5	76.9	93.9	77.1	94.7
Marshall Islands	50 to 54	37.4	42.5	57.9	58.5	24.4	32.2	59.1	58.3	68.9	66.3	47.9	48.5	52.7	62.6	60.4	65.2	48.6	62.3	68.8	70.7	73.1	73.3	71.1	74.1
Mauritania	50 to 54	46.6	60.7	50.6	63.6	51.3	64.3	54.8	61.4	49.1	57.3	62.1	67.7	64.9	74.6	58.6	68.5	71.7	80.6	55.7	64.3	70.7	75.2	41.6	51.9
Mauritius	50 to 54	47.8	67.9	65.9	78.8	36.4	61.0	61.3	73.9	71.3	79.0	49.8	67.6	63.6	75.3	71.4	80.0	56.1	71.1	36.9	64.5	59.9	75.6	26.5	59.8
Mexico	50 to 54	73.4	79.4	78.7	86.4	69.3	72.1	75.3	78.0	78.4	84.3	68.4	67.3	78.4	74.0	78.2	77.1	79.0	70.6	70.1	81.3	76.3	87.1	67.3	77.9
Micronesia (Federated States of)	50 to 54	34.6	43.4	50.4	58.8	26.3	34.8	58.2	59.9	64.5	66.8	50.2	51.4	50.1	63.9	55.5	66.6	47.2	63.7	62.5	67.1	65.8	71.3	67.8	70.0
Monaco	50 to 54	73.0	86.1	78.0	89.2	72.7	85.8	73.7	87.0	77.9	89.5	67.7	82.6	63.7	81.4	60.1	78.6	69.2	85.5	73.3	85.8	77.6	88.1	78.3	89.9
Mongolia	50 to 54	37.7	55.3	48.2	69.1	35.0	47.2	63.1	71.7	67.2	77.7	57.3	63.4	43.0	44.1	47.5	46.9	42.1	44.4	77.2	77.7	82.5	87.1	76.6	73.8
Montenegro	50 to 54	52.5	62.9	65.2	72.5	45.5	57.4	68.8	69.5	74.5	75.8	60.7	59.5	76.7	83.7	76.3	81.0	77.4	86.6	75.8	81.7	84.4	87.5	75.1	82.7
Morocco	50 to 54	54.0	63.2	52.9	61.6	64.0	72.3	63.1	64.5	62.2	60.8	66.5	70.7	68.3	74.9	63.1	68.8	74.5	82.1	26.7	38.8	22.1	33.1	48.4	60.7
Mozambique	50 to 54	40.3	36.7	56.1	61.2	31.4	21.3	50.0	42.3	57.9	57.8	40.7	27.0	61.8	57.1	69.6	69.3	59.4	52.3	66.7	59.7	73.4	75.0	66.4	52.5

Myanmar	50 to 54	10.2	36.8	24.5	53.7	7.1	28.8	41.4	51.7	44.6	57.3	36.6	44.3	42.4	63.8	45.7	67.7	41.3	62.0	25.1	51.9	42.7	67.3	21.4	46.8
Namibia	50 to 54	49.6	52.7	60.7	67.8	44.4	42.4	55.6	49.9	58.4	57.2	51.2	41.2	74.8	77.3	77.7	81.2	74.0	75.5	71.9	72.9	77.9	80.8	70.8	69.8
Nauru	50 to 54	37.3	39.5	52.1	54.7	31.4	30.2	58.3	53.2	64.6	61.2	50.6	43.4	49.4	57.9	47.2	55.6	53.7	61.7	55.8	58.8	62.6	64.3	58.6	61.3
Nepal	50 to 54	42.6	57.1	47.3	65.1	46.3	54.8	61.4	67.2	64.6	72.3	57.1	59.3	54.6	66.6	53.9	68.4	57.6	66.6	50.8	66.0	47.6	71.7	65.3	69.6
Netherlands	50 to 54	81.5	90.0	85.1	91.9	81.7	90.5	83.6	96.1	85.3	95.5	79.7	93.9	71.9	83.9	72.2	82.5	72.5	86.9	84.0	89.6	87.3	91.5	89.1	93.8
New Zealand	50 to 54	71.7	87.7	72.2	88.6	76.1	89.4	76.8	92.0	76.6	92.4	75.5	89.1	52.1	69.2	46.9	66.5	60.0	73.0	79.0	91.6	80.5	91.9	85.4	96.6
Nicaragua	50 to 54	64.5	76.1	72.4	81.4	58.7	71.0	68.7	75.6	73.6	79.8	60.4	66.9	73.7	78.7	74.4	78.7	73.3	78.9	70.9	80.5	76.9	84.0	69.2	79.7
Niger	50 to 54	50.2	54.0	58.3	60.0	48.6	54.0	60.7	59.3	62.1	59.5	58.2	59.4	66.7	72.2	66.2	70.7	69.3	74.9	70.3	72.6	77.4	79.1	67.2	66.8
Nigeria	50 to 54	46.9	67.1	58.7	75.5	43.7	60.8	57.0	69.0	58.0	71.6	53.2	64.8	68.2	80.3	73.5	83.0	68.4	78.9	56.8	67.1	76.5	83.2	47.3	47.6
Niue	50 to 54	58.3	63.8	69.7	76.2	51.3	55.5	68.4	68.1	74.2	76.5	60.7	58.0	69.3	77.4	71.5	79.0	68.5	77.5	76.5	79.7	78.1	82.3	80.7	82.2
North Macedonia	50 to 54	58.0	67.8	66.7	75.0	55.1	65.7	67.0	70.5	70.9	74.3	61.1	65.0	56.6	64.8	52.2	59.1	61.9	72.0	53.1	65.2	64.8	74.1	53.1	66.8
Northern Mariana Islands	50 to 54	52.6	62.0	56.0	70.6	54.9	61.6	66.5	70.0	62.4	70.1	64.8	66.7	67.6	74.6	52.1	68.7	76.8	79.2	70.5	78.5	70.1	80.2	77.6	84.2
Norway	50 to 54	79.8	90.3	82.6	92.9	81.4	90.9	85.6	95.2	87.1	95.3	82.1	93.1	70.6	86.4	67.9	87.6	74.7	86.3	81.7	91.3	83.9	92.3	88.9	97.3
Oman	50 to 54	62.9	85.7	70.2	90.1	61.9	86.6	70.2	86.0	73.0	86.7	65.9	84.4	79.8	91.4	80.0	91.7	80.6	92.3	38.6	69.7	50.1	77.5	41.3	72.7
Pakistan	50 to 54	63.7	65.4	68.5	70.6	64.5	65.5	74.7	72.6	76.8	74.7	70.8	68.7	66.9	67.5	68.5	67.9	67.7	69.3	73.2	72.6	73.9	76.6	80.3	76.6
Palau	50 to 54	54.2	65.0	72.0	81.6	40.7	53.7	67.0	70.9	76.9	82.5	55.5	59.4	70.6	79.2	76.1	84.2	66.8	77.3	68.8	76.5	76.5	83.9	65.7	73.5
Palestine	50 to 54	57.0	72.6	65.6	79.7	52.0	69.1	61.0	70.5	67.0	76.1	51.9	62.7	89.5	95.9	89.1	95.7	89.2	96.0	28.7	53.4	43.9	65.1	23.2	51.1
Panama	50 to 54	68.8	74.0	76.8	83.8	63.4	65.2	74.7	76.4	80.1	84.7	66.5	65.6	76.6	73.3	75.1	76.9	79.6	70.0	69.1	80.8	76.4	85.0	67.0	80.3
Papua New Guinea	50 to 54	0.0	19.8	0.0	22.6	9.6	25.8	25.0	35.4	11.6	25.4	29.2	37.9	38.1	54.5	23.6	45.9	50.2	62.3	55.3	64.4	45.3	58.5	76.8	80.0
Paraguay	50 to 54	45.6	57.1	54.8	65.3	43.5	54.8	60.3	61.2	65.6	67.4	52.8	53.2	48.9	56.1	49.1	54.4	49.4	59.3	59.0	70.2	69.2	77.5	58.7	71.3
Peru	50 to 54	62.6	78.3	71.2	82.3	57.1	76.3	68.5	75.8	73.9	79.7	60.4	69.6	69.0	77.4	69.0	74.1	70.5	82.6	73.2	82.2	79.3	85.4	71.4	82.1
Philippines	50 to 54	37.9	50.3	57.6	66.2	24.6	40.6	44.1	60.4	54.6	68.5	31.8	51.0	68.9	71.9	72.2	73.7	66.3	71.2	57.1	62.6	72.9	74.1	48.1	58.9
Poland	50 to 54	52.7	70.7	68.3	85.6	43.1	62.5	64.7	74.1	72.6	85.4	54.9	63.5	59.5	71.1	64.6	76.7	54.8	67.1	33.8	61.6	60.2	80.1	20.7	56.5
Portugal	50 to 54	61.6	78.9	69.5	87.1	59.7	75.4	68.4	80.3	72.5	87.6	62.7	71.7	61.8	72.2	61.0	71.2	64.0	74.0	66.1	86.0	73.6	90.5	69.0	89.2
Puerto Rico	50 to 54	77.9	87.8	85.5	92.4	70.2	80.9	80.4	85.8	86.0	93.0	72.2	74.2	80.0	83.5	80.2	84.7	79.3	81.1	79.5	90.7	86.0	92.2	73.8	89.1
Qatar	50 to 54	76.5	94.8	79.0	98.2	79.0	97.0	80.6	95.3	81.6	96.2	78.4	95.1	70.6	89.9	81.2	98.0	67.7	88.0	76.6	89.4	75.3	92.8	84.3	92.4
Republic of Korea	50 to 54	63.6	85.2	69.4	89.2	64.1	85.4	77.0	92.0	80.9	94.6	70.8	88.3	43.9	76.7	47.4	78.6	42.3	76.8	71.8	88.4	75.2	90.6	79.4	94.0
Republic of Moldova	50 to 54	51.4	66.0	58.6	76.3	51.1	61.5	63.7	66.8	65.6	71.4	60.6	60.6	72.8	76.3	78.0	83.4	69.6	72.3	67.3	65.2	71.8	75.8	71.3	64.4
Romania	50 to 54	53.6	63.9	62.7	77.2	51.2	57.3	61.6	63.1	64.9	73.5	56.8	51.8	59.7	61.8	57.0	63.7	63.6	62.0	56.6	66.2	67.7	77.9	57.0	65.8
Russian Federation	50 to 54	59.4	65.6	69.8	77.9	53.9	58.1	67.8	67.5	71.2	74.0	62.9	59.8	64.9	64.0	68.7	67.8	63.7	62.8	37.7	53.7	57.3	72.6	27.6	44.5
Rwanda	50 to 54	30.8	57.7	46.0	68.5	20.3	49.7	50.9	59.0	59.2	67.0	39.4	47.8	61.0	77.3	66.1	80.8	57.8	76.2	65.2	80.2	67.0	80.9	72.1	84.6
Saint Kitts and Nevis	50 to 54	47.6	54.1	59.5	72.8	39.1	41.3	60.4	57.6	66.8	72.2	51.1	42.9	61.9	69.8	58.2	72.0	67.7	68.8	50.8	57.3	61.0	73.1	46.0	50.1
Saint Lucia	50 to 54	39.6	63.2	48.5	75.0	37.9	54.7	51.7	63.9	54.3	73.0	48.3	53.5	53.4	72.5	48.5	71.1	60.9	75.0	36.4	68.4	41.9	74.7	43.1	68.4
Saint Vincent and the Grenadines	50 to 54	55.1	54.8	61.3	70.2	54.1	43.5	56.0	56.8	58.6	67.9	52.8	44.3	72.0	71.2	70.3	71.5	75.0	71.5	64.9	64.1	66.4	72.1	71.3	62.7
Samoa	50 to 54	51.6	58.1	66.7	68.1	41.1	52.4	65.2	65.7	73.5	71.5	54.3	57.7	65.4	73.7	69.6	72.9	63.5	76.2	76.3	79.2	78.5	80.8	80.2	82.4
San Marino	50 to 54	79.2	90.8	84.3	92.6	79.0	91.1	80.5	94.1	82.5	92.9	76.6	92.3	83.0	91.0	83.2	90.5	84.2	92.4	79.2	90.6	84.9	91.8	84.2	95.4
Sao Tome and Principe	50 to 54	66.1	67.8	72.3	71.9	63.0	68.1	70.5	70.6	71.8	69.5	68.2	71.4	76.9	76.8	75.7	72.7	79.2	81.2	71.0	67.6	81.4	78.7	57.7	56.7
Saudi Arabia	50 to 54	46.4	58.4	47.9	63.1	51.0	59.9	57.0	65.0	57.6	68.6	54.2	59.9	76.6	82.9	73.2	79.6	79.8	85.6	19.0	29.5	15.4	34.7	34.2	37.9
Senegal	50 to 54	48.9	56.4	59.1	64.4	44.4	53.4	54.9	58.5	55.8	57.5	53.2	58.6	64.6	69.1	64.6	67.0	66.6	72.4	54.0	61.4	74.1	76.7	34.7	44.8
Serbia	50 to 54	55.8	66.0	65.2	75.7	52.5	61.5	65.8	67.1	70.0	74.4	59.8	57.8	50.8	59.8	46.5	54.4	56.5	66.5	47.3	55.4	63.0	69.1	44.2	53.6
Seychelles	50 to 54	54.4	68.3	69.1	78.7	47.5	64.1	65.2	72.0	70.2	76.1	59.0	66.8	72.3	84.9	76.6	86.1	68.9	84.5	55.5	68.8	72.8	79.8	49.5	67.2
Sierra Leone	50 to 54	48.5	53.3	60.1	61.1	43.4	51.3	57.2	59.1	58.8	59.2	54.2	58.8	66.2	68.8	67.4	65.4	67.8	72.9	48.4	52.0	74.6	74.1	26.7	31.4
Singapore	50 to 54	72.1	89.3	75.4	90.0	73.8	91.8	80.8	94.2	82.9	93.0	76.6	93.6	71.5	87.3	70.8	86.2	74.3	89.9	76.6	92.4	74.8	91.1	87.2	99.5
Slovakia	50 to 54	57.8	76.6	69.9	85.5	52.3	73.4	58.6	74.3	68.0	82.6	47.4	65.4	64.2	76.7	63.9	76.2	65.2	77.5	52.3	73.1	68.3	81.4	49.6	75.7
Slovenia	50 to 54	61.0	88.0	75.0	94.8	53.7	84.9	70.2	93.8	78.6	97.3	60.9	89.7	68.6	88.6	70.2	89.4	67.6	88.3	54.0	84.0	71.2	90.9	50.3	85.8
Solomon Islands	50 to 54	47.7	49.0	61.9	63.3	39.6	39.5	69.6	67.6	75.2	74.0	61.9	58.9	58.4	66.7	64.9	71.8	54.6	63.5	73.0	74.1	74.2	75.7	79.3	79.3
Somalia	50 to 54	33.8	47.0	50.9	61.1	23.4	37.8	48.1	49.6	55.9	57.9	38.6	39.4	60.9	70.6	68.8	75.4	57.9	69.3	73.5	79.4	74.8	80.2	79.6	84.4
South Sudan	50 to 54	70.0	62.4	60.1	63.9	37.7	42.4	70.7	51.8	60.5	54.4	45.4	36.9	81.8	78.6	74.0	75.5	69.9	73.9	80.4	75.7	77.3	79.8	78.0	80.0

Spain	50 to 54	44.5	50.3	79.8	89.0	69.7	83.8	53.5	46.3	82.2	93.1	73.7	83.0	69.6	73.2	79.9	81.1	78.4	82.9	74.0	77.2	80.3	89.4	79.6	93.2
Sri Lanka	50 to 54	72.0	84.0	75.7	83.8	52.4	68.3	78.7	88.8	73.4	79.1	50.9	61.7	78.7	81.4	87.2	91.3	78.3	85.3	74.5	86.7	74.6	81.1	58.4	73.1
Sudan	50 to 54	61.5	74.7	36.1	58.7	49.0	69.0	63.0	71.4	54.2	57.1	53.6	66.1	82.3	88.2	51.4	65.1	61.5	77.5	61.9	73.8	4.2	36.3	45.1	55.9
Suriname	50 to 54	37.9	60.7	51.4	63.9	34.4	36.1	53.5	61.2	58.7	64.4	45.5	41.5	56.1	71.1	59.2	67.7	65.1	66.7	17.0	39.8	62.7	72.7	53.4	56.1
Sweden	50 to 54	39.7	47.6	84.4	96.3	82.1	98.4	53.0	53.9	89.0	96.5	86.1	100.0	61.6	66.9	66.3	83.5	75.8	88.1	54.2	61.3	86.5	97.2	84.6	99.8
Switzerland	50 to 54	81.2	96.3	83.0	92.8	78.7	90.4	88.3	99.1	86.5	96.0	82.5	97.1	70.5	85.1	70.1	87.5	75.4	86.0	80.9	96.5	84.3	92.4	82.7	96.6
Syrian Arab Republic	50 to 54	78.7	90.1	65.8	75.3	63.5	68.8	85.4	97.8	73.1	75.5	71.9	69.7	71.8	86.0	77.7	87.1	82.4	92.4	78.6	90.8	47.7	59.5	42.1	49.7
Taiwan (Province of China)	50 to 54	61.8	69.6	71.3	89.4	54.1	75.2	73.1	73.4	77.4	94.4	64.7	80.6	79.8	89.8	79.1	88.0	80.9	82.4	39.2	49.4	72.9	86.1	68.3	84.7
Tajikistan	50 to 54	59.9	81.0	59.1	69.1	46.9	61.6	71.9	87.0	68.0	68.8	60.8	64.6	79.5	84.9	68.8	77.2	77.0	80.5	66.3	83.1	51.4	66.0	24.7	59.2
Thailand	50 to 54	49.8	62.9	67.1	79.4	48.5	58.2	65.0	67.2	72.5	79.2	63.7	65.8	72.8	78.4	63.1	75.4	47.2	61.1	33.2	58.6	69.2	78.9	59.1	65.9
Timor-Leste	50 to 54	55.5	66.6	32.3	49.5	31.6	34.6	68.9	72.9	45.9	55.2	45.8	45.9	54.1	66.3	50.8	63.3	59.3	64.8	60.7	69.5	58.0	66.6	55.6	47.3
Togo	50 to 54	26.7	37.9	55.4	60.8	48.3	43.1	45.8	51.3	55.5	56.6	57.6	50.9	54.3	63.1	62.7	66.2	67.1	65.8	51.4	51.7	73.1	75.9	40.5	33.1
Tokelau	50 to 54	48.4	48.7	57.3	69.5	30.2	49.9	56.6	54.6	68.8	71.6	45.9	52.2	64.1	64.7	58.5	73.6	57.6	76.0	56.0	52.5	68.4	77.8	68.8	78.1
Tonga	50 to 54	44.0	59.2	70.0	76.4	63.8	68.4	60.6	64.1	73.1	74.5	66.2	66.8	57.9	74.1	73.1	79.8	80.3	86.0	65.5	75.9	79.8	83.2	83.2	85.6
Trinidad and Tobago	50 to 54	64.8	70.8	63.8	78.1	51.1	61.4	70.5	71.6	65.1	74.1	45.3	50.9	75.9	82.3	56.5	66.0	56.4	59.4	79.0	82.2	69.8	80.5	49.3	65.7
Tropical Latin America	50 to 54	54.8	68.1	51.9	67.7	30.8	55.1	57.1	64.1	63.6	70.6	43.6	52.5	56.1	62.4	44.0	50.8	38.3	52.7	55.5	70.1	55.6	76.4	29.4	64.8
Tunisia	50 to 54	63.4	77.0	65.9	80.1	66.6	77.7	68.9	78.2	69.9	78.6	67.0	76.2	78.4	88.5	78.0	87.9	79.0	89.0	43.3	61.6	43.6	66.3	55.3	66.3
Turkey	50 to 54	64.5	82.0	72.2	87.3	62.1	79.2	73.1	82.1	75.7	85.3	68.8	76.6	55.7	77.6	60.2	79.8	52.6	76.0	57.5	76.8	67.1	82.4	57.9	76.9
Turkmenistan	50 to 54	52.6	57.9	62.1	71.6	49.4	49.8	65.9	63.1	69.0	71.0	61.4	54.0	58.0	58.5	58.4	64.1	59.8	57.4	34.8	51.2	48.2	63.9	33.3	48.6
Tuvalu	50 to 54	29.1	47.4	45.8	60.6	16.6	40.0	53.7	59.8	61.6	66.4	43.0	51.6	47.2	65.8	49.5	66.1	45.1	67.7	58.0	67.9	63.1	71.7	60.4	70.6
Uganda	50 to 54	51.2	62.8	68.8	75.7	40.3	54.3	54.6	61.0	65.3	69.8	43.4	51.4	69.6	77.5	78.2	82.8	67.0	76.0	78.9	82.4	81.3	84.2	82.3	85.5
Ukraine	50 to 54	63.8	68.5	70.9	78.8	62.0	63.8	68.5	70.6	71.5	76.5	63.7	63.1	63.8	69.1	64.2	74.5	65.0	67.1	52.6	61.3	63.5	75.1	52.6	58.5
United Arab Emirates	50 to 54	71.2	86.6	74.1	90.5	73.7	88.9	76.7	89.5	79.1	90.0	73.3	88.7	65.0	86.9	58.2	87.9	70.5	88.1	60.9	85.2	62.3	86.6	69.4	92.5
United Kingdom	50 to 54	75.2	86.3	79.3	89.6	75.3	85.5	82.6	90.8	84.3	91.9	78.7	87.1	61.5	78.8	59.8	79.2	64.7	79.4	77.1	89.0	82.6	91.1	80.9	93.3
United Republic of Tanzania	50 to 54	53.3	70.7	64.2	74.7	48.4	71.2	59.8	68.7	65.0	70.4	53.1	65.9	72.8	81.2	76.5	81.3	72.5	82.9	78.9	81.6	79.1	82.4	85.0	86.4
United States Virgin Islands	50 to 54	56.0	80.3	67.0	88.6	47.2	75.0	60.8	81.1	68.4	87.3	50.5	74.2	63.8	83.5	64.3	85.7	63.2	81.0	63.3	85.9	70.9	89.5	60.1	88.1
United States of America	50 to 54	83.6	100.0	85.7	100.0	84.3	100.0	83.0	91.3	85.0	92.5	78.6	87.4	63.7	77.7	63.5	79.3	63.6	74.3	87.3	100.0	88.3	100.0	91.4	99.7
Uruguay	50 to 54	67.0	79.0	73.9	82.9	65.0	78.3	71.5	86.0	77.8	88.5	62.0	80.6	58.9	70.0	61.0	71.6	56.6	68.4	75.5	81.3	79.7	84.6	80.6	85.4
Uzbekistan	50 to 54	55.6	70.3	64.4	78.8	53.1	66.5	62.2	75.8	65.7	79.0	57.2	70.9	78.3	73.0	79.1	79.3	79.2	70.5	50.1	64.2	60.1	74.2	50.9	62.7
Vanuatu	50 to 54	45.8	54.4	63.4	68.2	35.2	45.3	61.4	64.3	70.7	71.6	50.7	55.3	57.3	68.2	62.2	70.3	56.2	68.5	71.0	77.0	76.4	80.3	71.5	78.3
Venezuela (Bolivarian Republic of)	50 to 54	59.4	62.8	68.9	74.9	52.7	51.7	68.3	66.4	73.4	75.8	60.2	53.1	71.2	70.3	71.1	71.4	71.7	68.6	70.9	78.7	77.1	83.4	69.7	78.1
Viet Nam	50 to 54	38.6	47.3	56.7	68.6	28.8	38.1	57.0	58.3	63.9	69.6	48.5	47.7	63.4	72.5	71.2	79.9	57.2	68.2	52.1	53.4	68.3	74.1	45.7	49.5
Yemen	50 to 54	26.4	46.1	34.5	51.3	28.1	48.8	45.5	49.7	53.7	52.3	35.5	47.3	49.0	62.8	54.4	63.7	45.7	62.9	1.3	17.2	0.0	25.3	20.3	25.4
Zambia	50 to 54	48.7	50.9	58.8	64.4	43.5	43.7	57.4	53.9	61.0	58.2	52.6	47.7	69.1	70.4	69.9	71.9	70.8	71.9	78.0	75.0	78.1	78.4	83.7	77.6
Zimbabwe	50 to 54	56.3	35.2	63.1	49.5	55.2	25.1	37.9	0.0	33.0	0.0	40.0	0.0	68.5	57.2	66.9	57.0	72.3	60.1	78.8	66.3	80.7	74.3	81.8	59.5
Afghanistan	55 to 59	21.3	39.2	27.7	39.6	21.9	46.2	44.0	45.8	55.9	50.5	24.7	40.5	37.7	54.4	51.9	57.5	25.2	50.3	2.5	11.5	0.0	16.1	24.8	28.2
Albania	55 to 59	56.8	72.1	64.7	77.7	53.7	68.7	63.7	72.3	67.9	74.7	55.8	66.1	73.1	85.1	75.1	83.6	72.7	86.4	69.8	77.0	77.5	82.9	72.2	77.4
Algeria	55 to 59	66.9	76.5	66.1	75.3	70.9	79.9	73.8	77.1	72.6	73.6	73.1	78.7	78.4	86.4	78.7	84.4	78.5	88.2	50.6	62.6	51.0	62.5	62.1	72.1
American Samoa	55 to 59	57.7	62.8	68.6	69.2	52.5	59.8	69.6	69.3	73.3	70.6	62.8	64.6	78.4	82.2	75.3	76.4	82.3	88.1	73.3	77.9	79.8	81.5	74.5	79.6
Andorra	55 to 59	80.5	88.7	81.3	88.2	82.0	90.5	86.3	95.0	85.5	91.4	84.4	95.6	73.9	86.2	68.7	81.4	81.2	93.0	79.8	88.0	81.9	87.9	85.9	94.0
Angola	55 to 59	47.2	60.5	58.6	67.6	38.7	54.2	53.6	57.8	59.7	61.5	43.4	50.7	69.3	75.8	72.8	76.8	69.4	76.6	67.4	71.8	74.1	76.8	65.9	70.1
Antigua and Barbuda	55 to 59	46.9	65.4	63.8	75.2	31.3	55.4	56.2	66.3	67.5	74.9	39.6	52.2	63.6	74.6	72.1	75.9	52.6	73.2	56.1	71.6	68.1	76.5	49.7	71.4
Argentina	55 to 59	62.2	77.9	69.0	82.1	59.4	76.1	75.5	82.7	80.2	86.1	66.6	75.5	53.2	75.8	59.7	77.1	46.0	75.7	73.6	85.0	78.3	86.6	78.0	90.0
Armenia	55 to 59	69.7	77.0	71.9	85.6	70.0	71.6	69.7	77.8	69.4	85.0	67.7	65.7	71.8	81.8	73.3	85.0	72.6	78.8	61.3	59.6	68.7	74.1	62.4	56.3
Australia	55 to 59	76.4	88.8	79.9	88.4	76.5	90.5	81.7	94.0	81.4	91.5	79.3	93.5	66.1	83.4	67.9	82.7	64.6	84.5	76.8	88.7	81.0	87.5	83.4	95.0
Austria	55 to 59	77.2	89.3	82.6	90.1	75.2	90.0	85.8	95.9	86.1	92.5	82.4	96.1	85.8	91.4	84.8	90.0	87.7	93.8	65.0	87.8	77.6	88.2	65.6	93.7
Azerbaijan	55 to 59	54.9	68.7	62.4	72.8	50.7	66.6	64.8	71.7	68.0	72.3	58.1	68.0	81.1	90.0	82.2	88.3	81.9	91.6	57.7	72.5	68.4	77.6	55.6	73.5
Bahamas	55 to 59	53.8	66.9	65.9	76.7	42.7	57.6	60.6	68.0	69.4	76.1	46.4	55.0	70.2	74.9	72.8	77.0	66.9	71.9	62.2	72.8	69.6	77.9	60.3	72.3

Bahrain	55 to 59	54.6	81.3	50.6	78.1	62.5	85.9	64.9	85.2	64.0	80.1	64.2	88.2	64.0	87.9	56.0	84.3	72.0	90.5	37.0	63.9	34.2	62.9	52.2	71.3
Bangladesh	55 to 59	46.0	57.8	46.5	59.2	49.3	60.2	68.0	71.7	68.2	72.4	64.5	68.0	51.2	67.9	53.5	63.7	53.6	73.8	55.1	62.2	52.7	64.7	66.0	68.9
Barbados	55 to 59	59.6	70.8	71.0	77.5	49.4	64.8	64.1	69.8	72.8	76.1	50.7	58.5	65.8	72.9	70.5	74.7	60.5	71.0	56.3	69.7	65.8	74.4	54.2	70.6
Belarus	55 to 59	61.2	67.6	63.8	74.2	62.0	64.1	71.0	71.3	69.4	74.6	70.4	64.3	60.0	63.9	63.5	71.8	59.0	57.9	37.8	51.8	48.9	66.9	40.2	48.4
Belgium	55 to 59	73.6	85.7	78.8	89.4	71.5	83.9	81.0	89.8	83.7	91.4	73.8	84.2	76.5	81.8	76.2	82.4	78.1	82.8	71.2	85.9	76.9	88.9	75.9	90.1
Belize	55 to 59	51.1	63.5	61.2	71.3	44.1	58.9	55.7	65.0	63.0	69.1	43.7	57.4	73.7	77.2	75.8	78.3	72.1	76.4	67.4	69.5	75.9	76.1	64.7	70.0
Benin	55 to 59	49.2	58.4	51.6	62.7	51.3	57.3	59.3	65.7	54.7	62.7	62.3	66.1	68.5	76.0	65.9	73.6	72.3	79.1	57.5	60.2	71.3	75.2	46.3	46.6
Bermuda	55 to 59	70.2	84.8	78.2	92.3	62.9	77.3	76.6	86.5	81.7	95.6	66.4	74.6	74.2	85.6	79.6	91.2	66.0	78.0	59.5	80.3	71.8	86.7	53.3	77.8
Bhutan	55 to 59	49.6	64.6	47.8	65.3	55.7	67.1	66.4	73.5	66.6	73.2	64.0	70.8	53.0	66.9	50.3	63.3	59.1	72.5	56.4	66.8	52.5	68.7	70.7	73.9
Bolivia (Plurinational State of)	55 to 59	34.3	56.9	42.0	61.4	30.5	55.0	46.5	57.9	52.3	62.5	36.9	49.3	41.0	55.5	39.4	50.8	44.4	63.0	46.8	66.8	58.1	74.4	43.0	64.3
Bosnia and Herzegovina	55 to 59	65.7	79.2	69.2	84.0	65.3	77.0	59.9	73.5	62.9	78.8	53.4	64.3	41.7	71.2	42.2	71.5	43.9	70.9	50.0	65.2	58.3	74.0	53.8	66.2
Botswana	55 to 59	52.4	71.0	58.9	75.6	49.6	67.1	56.3	69.5	55.1	70.6	53.4	65.2	75.8	86.2	78.1	86.8	75.7	86.8	73.4	81.2	76.3	83.2	75.2	81.3
Brazil	55 to 59	44.7	64.2	55.1	70.6	39.1	60.7	57.0	65.7	66.1	73.0	42.0	52.9	40.0	49.6	45.6	51.2	32.1	48.3	42.0	68.9	59.6	78.6	37.5	66.9
Brunei Darussalam	55 to 59	68.6	78.8	67.0	77.8	72.3	81.6	81.0	86.5	79.2	83.8	80.1	86.6	59.5	75.3	56.4	74.3	67.9	80.0	69.0	80.6	69.0	80.4	78.1	87.9
Bulgaria	55 to 59	49.1	60.4	57.4	70.8	46.4	55.2	60.4	66.2	64.2	71.6	52.9	57.4	55.7	54.8	57.6	57.5	56.5	54.1	43.7	35.7	57.7	58.0	44.1	29.6
Burkina Faso	55 to 59	51.6	55.4	63.9	69.3	41.9	44.1	60.3	62.9	65.4	69.0	51.3	52.9	73.4	76.7	80.4	83.1	68.1	71.8	65.1	64.5	80.2	81.9	50.9	47.6
Burundi	55 to 59	32.2	51.2	46.3	60.5	19.7	45.7	45.2	51.1	54.9	57.7	30.1	40.5	57.8	73.0	64.8	77.0	53.5	73.3	58.9	71.5	62.6	74.3	63.9	75.1
Cabo Verde	55 to 59	69.8	69.7	77.6	79.6	60.9	62.4	78.1	77.0	79.3	78.2	72.7	72.8	83.4	85.3	86.0	86.9	79.2	83.7	63.7	56.9	82.8	82.1	36.4	33.8
Cambodia	55 to 59	22.8	44.6	27.3	52.5	25.6	40.8	47.9	59.3	50.3	63.0	44.3	52.0	50.5	68.3	52.6	70.3	50.0	67.9	46.4	59.3	53.1	66.5	51.5	61.3
Cameroon	55 to 59	50.4	53.9	56.3	60.8	48.4	50.9	61.5	62.7	61.7	62.5	58.8	59.7	71.0	72.9	71.7	73.0	71.5	73.9	63.5	57.9	76.7	75.8	52.0	43.3
Canada	55 to 59	84.5	96.4	86.8	97.1	84.1	96.7	91.8	100.0	92.1	96.5	87.7	99.8	74.3	85.5	75.9	85.8	71.6	84.2	84.3	95.7	85.5	96.3	90.0	99.0
Central African Republic	55 to 59	28.8	34.0	44.0	50.1	17.2	21.8	44.4	41.0	51.9	49.8	32.7	27.7	56.5	60.4	62.8	67.5	53.7	57.7	55.4	60.4	64.2	69.5	53.1	56.9
Chad	55 to 59	53.2	49.1	57.4	55.3	52.9	47.5	62.6	59.4	60.6	57.7	62.6	57.5	69.9	67.0	69.0	64.2	71.6	70.5	61.6	52.5	74.3	72.0	51.2	37.8
Chile	55 to 59	66.0	83.8	70.2	87.5	64.8	81.6	76.2	90.4	79.8	92.6	67.2	83.7	62.8	77.3	65.0	79.1	61.6	75.5	71.9	85.8	75.7	88.3	76.8	89.2
China	55 to 59	27.9	63.0	39.5	70.5	22.6	59.1	57.5	72.2	63.4	75.7	47.6	65.1	13.5	74.0	18.9	76.8	12.6	73.1	19.1	56.0	38.1	66.6	16.2	56.2
Colombia	55 to 59	65.5	80.5	69.9	84.0	62.2	75.8	70.7	84.4	74.1	86.5	61.8	75.5	70.4	75.0	70.8	75.7	69.8	73.8	64.1	80.4	70.1	84.4	64.2	79.3
Comoros	55 to 59	52.1	66.2	59.1	70.3	48.0	63.4	56.1	62.2	58.9	64.0	50.2	57.7	74.0	81.6	74.3	80.7	76.2	84.1	71.2	78.1	73.7	79.6	74.6	80.7
Congo	55 to 59	46.6	58.4	54.0	61.7	41.8	58.0	52.6	54.4	54.7	53.8	48.1	53.2	67.3	74.4	67.1	71.2	70.2	79.5	65.3	70.0	70.9	73.3	64.2	72.1
Cook Islands	55 to 59	61.7	79.9	72.6	86.8	55.2	75.6	71.6	82.0	76.5	85.6	63.5	75.6	78.4	96.4	78.7	91.5	78.7	100.0	74.1	85.9	80.6	88.2	74.7	88.0
Costa Rica	55 to 59	76.8	85.6	79.0	90.0	75.2	79.7	80.8	88.4	81.0	91.8	76.3	79.8	80.9	82.2	78.9	84.6	84.2	77.7	74.7	85.8	78.3	87.7	76.0	86.2
Coted'Ivoire	55 to 59	55.7	56.7	64.4	64.2	52.8	53.7	65.6	64.3	65.8	63.7	61.4	61.2	70.8	74.3	73.1	74.6	71.4	75.7	53.6	50.9	75.1	75.4	43.1	34.3
Croatia	55 to 59	53.6	75.1	62.2	83.4	50.8	72.0	59.8	75.2	69.2	85.9	46.2	61.5	36.8	63.2	40.0	69.8	35.8	56.7	54.4	74.6	64.8	80.9	58.6	79.6
Cuba	55 to 59	62.2	70.4	67.5	78.3	58.9	64.3	69.4	72.0	72.5	77.6	62.0	62.0	74.1	80.6	72.8	81.0	75.3	79.2	56.8	59.2	65.0	71.6	56.0	54.8
Cyprus	55 to 59	70.5	86.1	74.3	88.7	69.5	85.0	82.8	92.4	84.9	92.7	76.4	88.7	67.3	84.1	67.3	82.2	70.5	88.1	71.8	86.1	74.6	89.5	78.6	88.9
Czechia	55 to 59	60.1	82.4	67.7	88.0	57.1	80.3	65.9	88.1	72.7	92.3	55.4	82.0	47.7	82.2	49.3	82.2	47.0	82.4	43.5	76.0	58.1	82.5	44.3	79.5
Democratic People's Republic of Korea	55 to 59	42.5	39.9	51.1	50.2	37.7	34.8	60.9	53.7	66.0	61.2	51.4	41.8	52.7	60.2	55.7	63.2	51.0	60.0	41.8	38.9	52.2	52.4	43.1	38.8
Democratic Republic of the Congo	55 to 59	52.2	54.1	58.1	61.8	49.0	49.0	59.0	56.2	60.3	60.0	55.2	48.8	73.7	75.3	73.3	75.8	76.6	77.8	66.8	70.3	70.8	74.4	68.9	71.3
Denmark	55 to 59	71.6	85.8	74.1	89.8	72.1	84.4	77.1	89.6	78.8	92.0	72.1	84.5	66.5	83.9	66.8	83.2	67.9	85.8	70.4	85.8	74.0	88.9	76.8	90.6
Djibouti	55 to 59	54.8	63.8	63.7	72.0	49.8	59.7	58.0	60.2	61.2	63.7	51.1	53.1	73.7	78.4	77.5	80.9	74.3	79.9	74.3	74.2	77.1	80.0	77.1	74.2
Dominica	55 to 59	48.0	55.1	58.8	72.6	29.6	40.9	51.0	53.0	60.2	67.1	31.9	34.2	63.6	68.4	67.5	76.4	52.2	60.9	52.4	55.7	62.0	71.5	41.6	47.2
Dominican Republic	55 to 59	55.9	60.9	62.4	71.2	52.1	52.9	62.7	64.6	67.1	71.5	54.1	53.8	67.4	73.0	67.4	73.7	67.0	71.8	60.7	63.3	66.9	73.7	61.7	59.3
Ecuador	55 to 59	64.2	78.8	70.5	81.4	59.2	77.0	64.7	79.5	68.9	80.4	56.2	74.5	74.3	71.6	73.3	71.1	75.5	71.9	63.6	82.7	72.4	85.8	60.3	83.3
Egypt	55 to 59	39.6	59.4	51.9	65.1	30.6	57.2	40.1	58.6	56.5	64.5	14.9	48.7	55.2	76.0	70.1	79.4	37.9	72.1	1.0	23.1	19.8	39.1	0.0	22.5
El Salvador	55 to 59	62.8	73.2	68.4	78.9	58.0	63.5	71.7	72.9	74.2	78.3	64.5	57.4	71.0	77.0	71.3	76.8	70.2	75.1	67.0	75.1	74.6	81.8	63.3	67.4
Equatorial Guinea	55 to 59	37.3	64.8	50.8	69.3	26.7	60.7	46.3	63.1	53.0	64.6	35.6	59.0	62.5	82.2	69.0	80.1	60.1	86.3	61.7	69.5	70.0	74.9	58.3	65.8
Eritrea	55 to 59	25.8	52.0	40.8	61.6	15.5	42.9	39.4	51.0	46.3	57.3	28.3	40.6	58.4	74.4	67.7	77.7	52.6	72.8	65.3	74.8	68.6	77.7	69.4	75.2
Estonia	55 to 59	60.1	80.6	68.3	89.2	56.2	73.7	68.7	81.8	71.8	89.1	62.1	71.0	69.1	76.5	79.5	84.8	56.7	69.9	33.0	71.5	52.3	85.2	29.8	65.4
Eswatini	55 to 59	41.7	44.4	57.9	57.4	28.4	33.8	47.1	44.3	55.0	47.4	35.1	36.9	71.6	73.7	77.9	76.2	67.7	72.3	71.4	69.3	77.9	76.7	68.0	64.6

Ethiopia	55 to 59	37.3	59.5	46.5	65.4	32.9	56.5	56.3	62.0	59.9	64.2	49.3	56.5	71.5	81.4	75.1	81.7	72.0	83.6	72.0	79.2	71.6	78.5	80.4	85.0
Fiji	55 to 59	56.0	64.9	65.2	70.1	49.4	61.8	62.1	62.8	68.1	66.9	51.5	54.8	75.8	81.5	76.3	79.6	74.8	85.1	73.9	78.9	80.0	82.0	74.1	81.3
Finland	55 to 59	74.1	89.7	77.6	90.5	73.9	90.4	82.1	95.4	84.5	93.8	76.7	94.4	77.4	88.1	74.0	86.9	82.0	90.1	65.2	88.8	72.4	88.8	69.9	94.3
France	55 to 59	77.8	85.7	86.8	88.0	73.8	85.7	79.0	91.5	84.4	92.2	69.6	88.0	81.7	86.0	82.7	85.6	81.5	87.1	77.6	84.7	88.3	86.0	79.0	90.8
Gabon	55 to 59	58.8	66.5	68.1	74.1	50.4	61.0	62.5	64.6	67.2	67.6	53.8	58.1	77.1	81.1	79.7	82.2	76.7	82.5	73.9	74.7	77.7	79.6	73.7	74.4
Gambia	55 to 59	53.1	51.0	59.9	56.8	51.4	49.3	61.2	57.1	58.1	54.8	59.3	56.1	71.3	69.1	71.0	65.2	73.5	73.4	51.8	48.0	74.3	70.5	38.6	29.9
Georgia	55 to 59	54.3	66.0	65.5	79.5	46.3	56.4	69.1	74.3	72.6	80.9	62.1	65.3	71.9	66.9	75.5	73.4	70.8	64.4	67.5	56.1	75.6	75.5	64.3	44.3
Germany	55 to 59	78.8	87.4	81.1	90.2	78.7	86.9	82.3	91.8	85.2	91.5	75.3	88.9	77.4	86.2	76.9	83.1	79.0	90.2	74.0	84.4	78.4	87.7	77.4	88.8
Ghana	55 to 59	55.1	61.5	60.2	69.3	52.9	55.3	59.3	63.1	57.2	63.1	59.8	60.3	70.2	75.6	68.8	74.2	71.5	76.4	57.0	57.3	75.2	78.0	37.2	34.4
Greece	55 to 59	73.8	80.5	76.2	86.3	74.0	77.8	81.2	83.8	81.4	87.6	77.7	77.3	80.0	83.6	80.8	84.6	80.3	82.8	75.9	84.6	78.1	87.4	81.8	89.1
Greenland	55 to 59	68.4	75.7	69.5	81.9	70.3	73.8	71.5	76.4	75.2	82.9	64.1	68.1	0.0	56.1	0.0	62.2	0.0	49.6	76.6	84.3	81.2	88.3	80.4	88.2
Grenada	55 to 59	39.7	63.1	42.1	72.6	42.9	56.7	49.6	62.7	50.6	70.9	47.3	50.9	57.2	68.0	54.6	70.9	62.3	65.6	27.1	64.1	33.0	72.9	36.6	62.3
Guam	55 to 59	70.0	79.4	75.0	84.4	67.7	76.0	75.8	80.3	76.9	81.9	71.3	75.3	79.8	90.7	76.1	85.8	84.9	95.0	73.9	83.4	79.4	87.3	75.4	84.0
Guatemala	55 to 59	65.8	74.2	67.9	76.4	65.9	72.3	76.4	77.7	75.2	77.4	75.1	74.4	87.7	84.4	85.5	83.6	90.0	84.1	69.3	80.8	72.2	82.9	72.6	81.8
Guinea	55 to 59	51.6	54.3	50.1	56.4	58.4	56.3	61.8	63.2	55.9	58.8	67.5	65.4	73.0	74.0	66.7	67.8	79.3	79.7	59.6	54.0	70.4	71.6	53.1	40.0
Guinea-Bissau	55 to 59	30.1	38.6	43.6	48.2	21.3	33.1	47.3	48.6	50.2	49.2	40.8	45.5	50.1	56.2	55.7	55.5	46.1	57.7	38.2	40.6	68.5	68.8	7.4	11.2
Guyana	55 to 59	8.8	41.9	25.1	53.2	0.0	33.9	36.7	50.3	46.7	58.2	21.8	37.3	48.9	60.3	60.3	66.5	37.3	52.8	13.5	46.3	40.9	62.9	0.3	37.2
Haiti	55 to 59	0.0	21.5	8.3	29.1	0.2	19.6	35.8	37.7	44.6	47.2	22.3	22.5	13.5	32.6	13.3	29.8	16.5	38.7	1.8	27.8	7.6	34.4	17.7	36.9
Honduras	55 to 59	45.9	27.8	56.2	36.3	37.3	23.5	50.1	29.2	59.1	39.9	34.4	13.0	53.8	26.0	56.8	22.7	49.5	31.8	50.7	41.3	63.8	54.6	43.9	36.4
Hungary	55 to 59	60.4	72.6	69.1	82.1	56.2	67.2	67.4	76.7	71.2	85.2	60.2	65.4	63.7	72.4	66.7	72.5	57.9	72.6	37.1	56.6	57.0	71.7	32.9	54.2
Iceland	55 to 59	79.9	91.0	81.9	92.3	80.1	90.9	88.0	97.5	86.8	96.7	85.8	94.8	80.1	90.7	77.4	89.5	84.9	92.6	77.3	90.3	80.4	90.2	83.2	95.7
India	55 to 59	44.4	51.8	47.7	53.6	45.4	54.3	63.6	68.0	67.1	68.8	56.5	64.1	46.1	54.1	52.1	52.4	45.9	59.1	51.4	55.9	53.0	59.4	61.0	62.6
Indonesia	55 to 59	47.1	49.7	52.0	57.8	46.3	45.3	62.5	59.9	65.4	64.2	56.5	51.9	60.9	66.3	61.1	66.3	62.5	68.4	61.1	57.0	66.9	68.3	63.6	53.9
Iran (Islamic Republic of)	55 to 59	61.8	80.2	67.1	82.7	60.0	79.7	67.5	80.1	71.2	80.7	60.1	76.0	71.8	90.5	75.7	90.8	69.2	89.0	21.8	56.1	36.4	64.3	23.4	57.8
Iraq	55 to 59	41.3	55.2	49.8	63.5	37.7	50.8	55.8	62.3	61.0	66.5	46.8	54.5	64.7	73.5	69.9	75.2	57.9	71.4	2.3	24.4	20.8	42.7	2.6	20.8
Ireland	55 to 59	75.4	89.5	78.2	90.9	75.4	89.0	85.1	98.3	84.9	96.6	81.9	96.1	70.6	87.1	69.8	87.9	73.8	86.9	72.4	88.6	78.2	89.0	77.3	93.6
Israel	55 to 59	74.8	88.8	74.7	90.6	77.1	88.8	73.4	84.7	75.3	88.3	67.9	78.3	84.2	89.7	81.8	89.9	88.6	90.3	78.1	91.1	76.0	90.3	87.2	96.5
Italy	55 to 59	70.5	85.4	74.5	90.1	69.5	83.8	75.3	88.4	79.2	90.1	67.1	83.7	70.3	83.0	70.7	83.0	72.0	84.5	69.9	87.1	74.7	91.5	74.3	91.4
Jamaica	55 to 59	42.6	60.0	52.0	69.4	36.7	53.0	57.0	67.1	63.7	73.5	45.5	55.9	62.1	74.0	61.9	74.2	63.5	74.4	38.7	59.7	47.3	69.7	41.9	57.7
Japan	55 to 59	76.1	87.3	79.4	89.6	76.0	87.7	87.1	93.6	88.5	92.9	82.6	92.2	70.8	86.0	72.0	86.1	72.9	88.3	74.5	88.1	78.2	91.5	80.5	93.0
Jordan	55 to 59	62.8	80.1	61.0	78.7	68.3	83.2	64.8	79.9	64.0	76.8	64.5	80.5	92.5	97.9	90.3	97.3	94.8	98.4	37.3	68.2	37.6	66.9	51.7	77.6
Kazakhstan	55 to 59	65.7	67.0	71.1	75.4	62.7	60.7	69.6	67.0	71.7	72.0	64.1	58.0	56.5	61.1	59.8	66.6	56.3	59.6	52.1	59.9	64.3	72.5	48.8	54.7
Kenya	55 to 59	66.4	66.3	68.8	71.7	66.4	63.0	69.1	65.0	69.0	66.3	66.7	60.4	81.7	82.1	80.8	81.8	85.2	84.7	80.9	80.0	79.1	81.2	88.1	83.4
Kiribati	55 to 59	54.4	55.9	64.9	66.8	49.4	50.2	73.8	70.8	74.4	72.4	70.0	65.9	72.7	80.7	81.9	84.1	61.8	78.1	70.6	70.1	81.2	83.1	67.3	64.0
Kuwait	55 to 59	80.5	91.7	78.0	96.9	84.4	90.6	86.1	97.1	82.7	100.0	86.2	94.8	85.6	100.0	81.3	100.0	89.4	99.0	61.8	79.4	55.8	86.5	73.5	81.1
Kyrgyzstan	55 to 59	52.5	58.2	59.0	68.0	49.1	52.1	64.1	67.2	65.0	71.4	60.3	59.6	52.5	56.4	62.6	61.9	44.3	54.8	44.1	36.3	55.7	56.2	42.8	28.8
Lao People's Democratic Republic	55 to 59	13.3	44.1	20.6	51.7	13.5	41.5	42.2	57.9	47.3	61.2	34.0	50.9	32.8	65.9	34.9	67.4	33.1	66.5	26.3	52.4	36.9	65.2	31.3	50.7
Latvia	55 to 59	55.4	70.1	63.4	77.6	51.1	66.4	61.8	71.3	66.5	77.7	53.1	61.2	56.0	68.4	64.8	73.4	48.4	66.0	35.4	55.1	52.7	69.1	31.7	53.8
Lebanon	55 to 59	53.5	85.1	62.4	86.6	49.5	85.0	54.9	84.8	68.6	85.3	33.7	79.6	49.9	89.6	59.8	90.3	38.4	85.7	42.3	79.7	50.0	81.4	47.7	84.2
Lesotho	55 to 59	43.3	26.6	58.7	45.8	23.7	3.1	47.1	34.0	56.1	45.4	33.2	17.3	72.5	62.9	79.8	69.7	62.6	54.2	75.4	62.0	79.2	71.5	71.6	48.9
Liberia	55 to 59	51.9	50.2	52.0	51.7	56.3	53.4	62.3	56.0	56.0	50.9	66.3	60.2	73.2	72.0	66.9	65.2	79.1	78.1	55.5	51.1	69.8	68.7	47.2	36.1
Libya	55 to 59	71.8	74.3	70.3	74.4	76.2	76.2	76.2	72.6	73.1	69.7	78.1	74.9	83.1	84.7	80.1	80.3	86.7	88.7	53.8	50.5	55.0	57.7	63.6	52.7
Lithuania	55 to 59	63.6	74.0	68.7	81.1	61.2	69.9	69.2	78.3	69.5	83.4	66.3	70.2	62.9	70.9	69.0	77.5	58.6	65.1	48.4	64.9	59.8	76.8	48.3	63.4
Luxembourg	55 to 59	69.5	85.7	74.0	90.7	68.5	84.2	73.5	90.6	77.1	91.1	66.2	86.4	70.8	85.2	68.3	83.4	76.2	89.0	64.8	86.4	72.1	91.7	68.1	91.2
Madagascar	55 to 59	43.1	50.3	54.2	56.6	35.1	47.1	53.8	51.5	59.0	54.4	44.6	45.7	65.4	71.4	69.9	70.1	64.2	74.7	69.1	72.3	74.5	75.2	67.9	74.0
Malawi	55 to 59	47.3	46.7	52.1	56.3	46.9	41.5	49.4	39.1	51.4	45.2	44.7	28.7	68.0	68.0	69.2	70.6	70.2	69.2	69.4	64.6	71.1	71.8	74.5	64.3
Malaysia	55 to 59	55.8	68.7	61.9	73.6	53.2	66.5	67.9	74.2	70.5	75.6	61.9	69.5	72.1	81.5	73.9	80.5	71.8	83.4	60.7	69.0	66.1	74.0	64.7	71.4
Maldives	55 to 59	45.1	76.7	42.3	77.8	51.2	78.2	58.8	81.4	53.1	75.9	59.5	82.5	65.3	88.7	62.7	86.7	70.9	91.6	56.4	77.6	55.5	79.1	67.4	82.8

Mali	55 to 59	48.1	55.4	45.8	53.4	56.6	61.8	57.2	61.2	50.6	52.2	64.3	67.6	71.1	76.7	61.7	66.8	79.7	85.0	63.3	62.1	68.5	70.4	63.6	58.7
Malta	55 to 59	71.1	87.6	73.7	90.2	70.9	86.8	79.2	92.9	79.4	91.1	75.3	91.5	83.9	90.5	81.5	89.1	88.7	92.9	65.7	86.2	68.7	88.9	72.8	90.5
Marshall Islands	55 to 59	41.5	45.6	55.7	57.3	32.2	37.4	62.0	60.7	67.7	66.0	52.4	51.4	59.4	69.5	65.3	70.9	54.9	69.1	68.7	70.7	75.4	76.3	68.4	70.5
Mauritania	55 to 59	51.0	61.9	50.9	61.8	56.4	66.1	58.5	63.9	50.0	57.2	67.3	71.9	70.0	78.8	63.0	71.1	77.2	85.8	55.0	61.7	69.5	73.1	42.2	50.0
Mauritius	55 to 59	50.0	72.5	63.7	79.4	41.8	67.5	62.0	78.4	70.0	80.3	50.0	73.2	58.0	78.0	70.0	81.3	44.3	74.1	39.7	67.8	59.4	76.7	34.8	65.2
Mexico	55 to 59	73.5	80.9	77.5	85.9	69.1	75.1	75.9	80.5	78.7	84.6	66.6	70.0	80.4	75.3	79.8	77.4	80.2	72.1	66.8	80.6	73.4	85.9	64.5	77.8
Micronesia (Federated States of)	55 to 59	36.8	46.9	47.9	57.9	31.2	40.1	59.8	62.6	63.7	67.2	52.2	54.3	55.8	70.2	61.0	71.9	51.7	69.8	62.1	66.8	69.3	74.4	63.1	66.1
Monaco	55 to 59	75.5	86.4	77.8	88.0	75.7	86.6	77.3	89.3	79.3	89.1	72.0	86.4	68.0	84.8	65.1	81.5	73.5	89.6	71.8	83.7	75.3	85.4	77.2	88.5
Mongolia	55 to 59	33.7	54.9	44.6	66.9	27.2	46.4	61.2	71.4	66.6	76.5	51.2	62.3	35.6	38.6	43.7	46.9	31.2	35.0	77.1	78.0	81.5	85.6	76.1	74.2
Montenegro	55 to 59	47.6	53.2	58.9	61.2	41.5	49.3	65.2	62.0	71.3	68.3	54.9	50.7	74.3	78.4	74.2	73.9	75.0	83.5	69.5	71.8	78.8	79.5	70.0	72.9
Morocco	55 to 59	53.1	61.5	48.5	57.8	62.8	69.1	60.4	61.4	58.1	57.6	63.5	65.4	66.9	72.3	63.9	67.1	71.2	78.6	25.1	34.2	21.4	30.4	46.6	54.6
Mozambique	55 to 59	34.8	34.6	48.7	55.7	26.6	21.2	43.1	38.4	50.8	52.0	31.0	21.0	58.5	55.6	68.4	68.6	55.0	50.0	58.9	52.7	67.9	70.3	57.5	43.9
Myanmar	55 to 59	13.1	39.3	23.3	52.7	10.5	31.9	41.5	53.0	45.8	59.0	33.1	42.9	39.1	62.8	46.5	67.9	34.4	59.7	29.5	52.0	45.4	66.3	28.3	48.2
Namibia	55 to 59	48.8	53.1	56.8	65.1	44.1	43.1	53.7	50.1	55.1	56.4	48.8	39.7	76.3	79.3	78.8	82.6	75.9	77.1	69.6	71.4	75.0	78.8	68.7	67.3
Nauru	55 to 59	39.7	43.5	50.6	54.6	35.3	35.9	59.1	55.3	63.3	61.3	51.0	45.1	55.0	64.4	53.9	61.2	58.0	68.5	55.5	60.1	68.7	70.5	53.9	57.1
Nepal	55 to 59	38.2	53.6	35.4	57.7	46.2	53.6	61.0	67.6	62.0	70.8	57.9	60.6	46.4	61.9	45.6	64.5	51.0	61.9	40.5	57.1	33.4	62.7	59.6	62.9
Netherlands	55 to 59	81.4	89.5	83.9	91.3	81.2	89.2	83.0	95.6	84.3	93.9	77.8	93.7	73.0	86.1	73.8	85.8	72.4	87.0	80.8	87.7	84.2	89.5	85.3	92.2
New Zealand	55 to 59	73.2	87.7	72.6	88.3	76.3	88.5	77.6	93.0	76.3	91.4	76.2	91.0	57.1	72.8	55.2	74.3	63.2	71.0	74.2	88.0	76.8	88.5	79.9	92.7
Nicaragua	55 to 59	64.8	75.3	70.7	80.5	59.7	69.3	69.4	76.1	73.5	80.2	59.9	65.5	75.0	79.8	75.2	79.9	75.2	79.6	68.6	77.8	74.8	82.3	67.9	76.7
Niger	55 to 59	53.9	54.4	58.6	57.0	53.0	55.8	63.6	60.6	62.6	58.4	61.6	61.6	70.9	75.3	70.7	72.8	72.7	78.3	68.7	68.8	75.8	76.0	66.4	63.9
Nigeria	55 to 59	49.5	67.9	57.6	72.3	47.6	64.3	60.4	70.9	58.8	70.4	56.6	69.5	70.6	83.7	75.4	83.5	70.6	83.3	54.4	66.1	74.2	79.6	46.5	49.1
Niue	55 to 59	59.5	65.5	68.8	75.8	53.6	58.6	69.9	70.7	73.8	76.3	62.3	61.5	74.5	84.3	75.3	82.3	74.5	86.1	74.6	77.9	79.8	84.2	75.5	77.9
North Macedonia	55 to 59	53.5	63.6	59.1	67.8	52.2	63.1	59.7	64.4	64.2	67.2	51.4	58.1	44.3	55.3	41.4	48.9	49.7	65.5	44.5	56.2	56.5	65.6	46.2	58.9
Northern Mariana Islands	55 to 59	52.8	66.0	55.0	72.7	54.5	62.2	67.9	72.8	63.0	73.8	65.4	68.5	71.9	79.9	58.1	75.3	81.6	85.2	67.1	77.9	72.7	82.2	71.0	78.5
Norway	55 to 59	80.9	89.9	81.4	91.4	82.6	90.5	86.4	94.8	86.5	93.0	82.7	93.8	74.7	91.0	72.5	91.3	78.3	90.0	79.3	88.8	80.1	89.0	86.2	94.8
Oman	55 to 59	54.5	80.6	59.5	83.3	54.1	81.4	61.0	81.7	64.6	81.6	53.7	79.3	74.6	90.0	76.4	91.3	74.6	90.8	26.1	62.5	37.5	70.5	31.1	65.3
Pakistan	55 to 59	61.3	63.1	60.9	64.1	65.0	65.5	74.3	72.6	73.7	72.5	72.2	69.9	62.6	63.1	62.6	61.8	66.1	67.2	67.2	66.6	65.4	69.3	77.5	72.7
Palau	55 to 59	55.6	68.0	72.7	83.5	41.8	55.2	68.5	73.9	77.8	83.6	55.3	60.5	75.7	87.2	79.3	88.1	72.3	85.8	66.6	75.5	78.5	86.5	60.0	68.5
Palestine	55 to 59	51.7	67.8	57.8	73.1	48.3	65.0	56.4	66.3	62.5	71.3	45.2	57.3	89.4	96.1	88.5	94.9	88.2	96.0	17.0	43.9	33.4	56.8	14.6	42.3
Panama	55 to 59	67.4	74.8	72.4	83.2	64.3	66.9	75.0	78.9	78.0	85.4	67.8	68.2	75.8	74.5	74.0	77.3	79.2	71.1	65.9	78.8	70.5	83.4	68.6	78.8
Papua New Guinea	55 to 59	3.9	21.1	0.0	18.4	12.9	28.1	31.2	40.1	19.9	30.8	31.6	40.2	43.3	57.8	36.9	51.5	51.7	65.4	55.5	61.7	52.4	60.4	69.6	73.0
Paraguay	55 to 59	51.1	58.5	56.1	65.6	50.4	55.3	62.8	61.1	67.1	68.7	54.4	49.2	48.3	52.0	49.2	52.8	49.1	53.3	62.1	69.0	69.9	76.8	63.7	69.2
Peru	55 to 59	66.1	79.6	72.4	82.1	61.1	78.1	71.7	78.4	75.4	80.0	63.3	72.8	71.2	80.0	71.9	75.3	70.9	85.0	74.2	81.7	79.7	84.8	73.3	82.4
Philippines	55 to 59	46.6	54.0	61.5	66.9	35.9	45.0	53.4	63.3	62.9	70.5	39.3	52.3	70.9	73.2	75.0	75.4	67.2	71.8	61.1	63.4	74.4	74.5	55.2	60.2
Poland	55 to 59	52.6	71.1	63.2	82.5	46.6	65.2	63.4	74.9	69.4	83.7	53.4	64.0	58.5	71.9	63.5	75.6	53.6	69.0	28.1	58.0	50.8	75.6	21.2	54.9
Portugal	55 to 59	61.3	79.3	67.7	87.1	58.9	75.8	66.4	81.3	71.5	87.5	57.2	72.3	58.5	75.9	63.0	76.1	55.5	75.9	63.1	83.9	70.5	90.6	66.1	86.2
Puerto Rico	55 to 59	78.0	88.5	84.9	91.8	69.8	82.4	82.2	88.6	86.7	92.8	73.2	78.8	82.9	85.4	83.0	86.6	80.7	80.9	74.2	88.7	82.4	89.9	67.3	87.4
Qatar	55 to 59	74.6	92.0	72.5	90.7	78.0	95.3	79.1	95.4	75.7	89.5	78.6	98.8	65.2	90.9	79.4	97.8	60.1	88.4	72.3	85.3	68.9	84.8	80.8	89.9
Republic of Korea	55 to 59	67.7	88.2	69.7	90.0	68.8	88.7	81.6	96.7	82.5	95.5	76.7	95.4	51.4	82.5	56.0	83.4	49.1	84.7	68.5	86.7	71.5	89.1	75.4	91.4
Republic of Moldova	55 to 59	47.9	64.9	50.9	71.4	49.5	61.8	59.8	66.8	60.7	68.8	56.5	61.5	65.8	70.3	70.6	77.4	63.9	66.2	62.3	57.3	66.3	68.8	67.8	56.1
Romania	55 to 59	53.0	62.9	59.3	73.3	51.2	57.7	57.4	58.9	61.5	69.2	49.6	44.8	51.4	51.4	52.4	57.1	53.3	49.2	52.5	62.3	62.8	73.9	54.9	62.9
Russian Federation	55 to 59	57.1	66.3	64.7	76.0	52.0	59.4	65.4	69.3	68.1	74.1	59.3	60.9	58.2	61.4	63.2	66.2	56.2	59.2	27.2	46.4	47.5	66.6	17.9	36.5
Rwanda	55 to 59	27.2	55.7	38.0	64.2	19.3	47.9	45.4	55.7	53.0	63.3	33.3	42.7	61.5	78.6	67.2	81.3	57.2	78.1	60.0	75.7	62.4	77.2	67.2	79.1
Saint Kitts and Nevis	55 to 59	41.4	54.5	50.3	70.5	33.8	42.8	55.2	58.6	60.9	71.4	44.6	42.2	58.6	69.5	58.7	73.7	58.5	65.9	35.8	52.3	48.4	68.3	30.8	45.5
Saint Lucia	55 to 59	46.4	65.9	58.9	77.0	33.3	56.8	56.4	66.7	65.6	76.0	41.3	54.4	60.7	76.5	60.4	76.8	61.4	75.7	40.1	66.3	54.1	73.2	30.9	65.9
Saint Vincent and the Grenadines	55 to 59	60.3	55.0	63.1	67.7	59.9	45.7	61.3	56.6	63.1	66.2	57.0	43.2	74.2	72.7	73.3	73.4	76.7	72.1	61.9	58.5	63.3	66.1	68.8	59.2
Samoa	55 to 59	53.2	60.2	66.5	67.6	43.3	55.3	66.9	67.7	73.8	71.1	55.4	60.5	70.8	80.6	74.4	77.4	68.1	84.4	74.5	78.5	80.4	83.9	74.7	77.7
San Marino	55 to 59	79.7	89.5	82.9	90.5	79.7	89.7	83.6	94.7	84.1	92.0	79.6	93.4	85.4	93.2	85.3	91.5	87.6	96.3	76.7	87.5	81.7	88.5	82.0	92.6

Sao Tome and Principe	55 to 59	72.0	68.8	71.5	70.4	75.2	69.5	75.9	72.1	71.0	68.7	80.8	74.5	84.6	81.2	79.1	74.8	89.1	86.3	74.6	65.4	79.9	76.5	70.1	54.4
Saudi Arabia	55 to 59	44.6	58.0	42.5	59.4	49.6	60.2	53.7	64.1	54.0	66.5	48.9	58.3	75.5	82.1	73.5	79.1	78.8	85.4	16.3	28.6	15.0	34.4	31.0	37.3
Senegal	55 to 59	52.9	58.0	58.4	62.7	51.2	56.3	59.0	61.6	56.4	58.5	59.0	62.4	69.8	73.4	68.3	70.0	72.2	77.1	53.4	58.2	72.1	73.9	37.8	42.0
Serbia	55 to 59	51.5	63.8	59.5	71.9	47.9	60.0	59.0	63.4	65.0	71.1	48.9	51.4	38.8	54.3	37.4	51.8	42.7	59.7	36.7	47.3	54.7	61.9	33.5	47.0
Seychelles	55 to 59	53.8	69.4	71.5	79.4	43.0	63.8	63.8	72.8	73.6	78.0	51.3	64.9	72.1	85.4	79.1	87.0	65.0	84.0	52.2	67.3	74.4	80.1	42.8	64.0
Sierra Leone	55 to 59	52.9	55.1	59.8	58.3	50.0	55.8	60.7	60.7	59.2	57.4	59.2	62.6	71.8	73.2	71.1	67.9	73.5	78.0	49.9	51.2	73.3	71.3	31.2	34.3
Singapore	55 to 59	73.2	91.9	72.8	90.3	75.9	94.6	83.4	98.2	82.5	93.8	81.1	99.8	74.4	90.8	72.8	88.9	80.2	95.5	72.2	92.0	69.9	89.7	82.3	98.3
Slovakia	55 to 59	61.0	75.5	67.7	81.3	58.3	73.5	58.6	72.6	66.4	79.0	46.6	62.9	62.6	74.9	63.6	74.7	63.2	75.3	52.8	68.4	64.1	76.1	54.2	71.4
Slovenia	55 to 59	60.2	84.4	71.9	90.8	53.2	81.6	68.4	92.6	77.1	94.9	55.9	88.8	66.4	89.0	70.2	90.0	61.8	87.8	47.8	77.7	66.0	86.0	43.8	79.8
Solomon Islands	55 to 59	46.6	50.4	57.7	60.8	39.5	42.9	69.1	68.9	73.0	73.1	61.3	60.7	61.5	71.2	68.4	74.7	56.0	69.1	70.5	72.8	74.6	77.0	73.3	74.7
Somalia	55 to 59	29.8	43.3	43.5	53.7	20.8	34.7	41.6	42.2	48.3	49.5	30.7	31.0	58.6	69.4	67.5	74.2	54.3	67.3	69.2	75.1	70.9	76.3	75.3	79.4
South Sudan	55 to 59	75.1	65.0	54.8	58.2	36.3	41.1	74.2	54.8	55.7	49.4	40.1	31.8	86.5	80.5	73.7	75.1	70.4	74.9	83.1	76.6	73.3	75.7	73.7	75.0
Spain	55 to 59	41.6	47.3	77.7	88.3	70.7	83.9	50.0	42.8	83.4	91.6	76.2	85.5	69.1	73.3	81.9	84.8	79.1	86.5	69.6	72.4	75.9	87.1	76.6	90.2
Sri Lanka	55 to 59	72.4	84.2	74.9	83.3	57.3	70.4	81.5	90.0	74.4	80.1	52.8	63.0	80.1	84.9	88.3	92.5	81.4	89.1	71.0	83.9	74.4	80.6	62.0	72.9
Sudan	55 to 59	64.2	76.1	29.8	55.3	48.3	67.5	65.7	73.8	49.3	53.7	47.9	62.3	84.4	90.9	51.1	64.0	55.6	74.6	63.5	73.6	4.8	35.4	42.8	52.1
Suriname	55 to 59	37.0	60.0	51.4	60.4	34.8	32.2	49.4	58.8	60.8	63.7	43.7	34.9	51.9	68.7	66.0	69.4	62.7	63.2	16.3	37.3	60.9	67.7	47.4	46.4
Sweden	55 to 59	41.0	44.4	82.8	94.0	79.9	95.5	54.4	51.8	87.6	94.6	84.4	100.0	64.2	66.3	68.7	85.0	76.0	90.7	50.0	53.2	82.4	94.5	80.2	97.3
Switzerland	55 to 59	79.9	94.1	82.0	90.4	78.1	89.7	87.3	98.4	87.1	94.3	82.8	99.3	71.0	86.8	73.6	87.6	78.1	90.6	76.5	93.7	81.4	88.8	81.2	93.6
Syrian Arab Republic	55 to 59	78.6	89.1	61.8	70.7	61.2	65.8	86.6	98.4	71.1	73.0	69.4	65.8	74.3	88.2	79.6	87.8	78.4	89.5	76.3	87.6	44.8	55.2	41.2	47.1
Taiwan (Province of China)	55 to 59	59.6	66.6	67.7	87.1	53.1	78.0	71.7	71.1	76.0	93.7	62.2	85.7	79.5	90.0	78.9	89.2	79.8	86.7	36.7	45.4	68.9	83.4	65.2	84.3
Tajikistan	55 to 59	58.2	82.1	55.8	63.4	44.9	59.7	70.8	90.6	65.9	64.9	57.0	62.1	78.4	88.3	68.4	73.8	76.5	81.2	62.2	81.6	50.9	61.3	23.8	56.7
Thailand	55 to 59	48.6	59.9	69.5	80.9	54.5	67.0	63.0	64.7	75.2	81.8	67.6	74.0	71.4	76.1	66.6	77.9	45.6	64.9	32.1	54.1	70.8	79.5	62.3	70.6
Timor-Leste	55 to 59	60.7	72.9	26.8	45.6	34.0	37.2	73.2	79.4	44.2	54.1	44.3	44.8	55.0	70.1	47.3	60.8	57.0	63.4	62.6	72.3	56.2	64.8	55.0	47.8
Togo	55 to 59	26.9	38.6	54.3	58.6	54.2	46.5	44.9	51.3	55.1	56.6	62.0	53.2	50.3	60.7	66.2	68.7	73.3	69.9	49.9	50.4	70.8	72.8	42.8	30.2
Tokelau	55 to 59	52.1	50.6	55.1	68.6	32.5	52.8	59.2	56.5	67.4	71.2	46.0	56.2	69.5	68.8	63.4	77.6	61.1	82.6	55.2	49.7	71.8	79.8	62.0	72.4
Tonga	55 to 59	44.9	60.5	69.8	76.8	62.3	67.9	60.6	66.2	73.6	75.5	66.6	69.0	62.6	79.8	77.6	83.9	84.8	93.0	65.7	74.5	81.2	85.0	76.0	79.8
Trinidad and Tobago	55 to 59	65.0	71.4	59.3	79.8	49.5	59.0	71.9	74.0	62.4	76.0	41.9	47.1	80.5	88.6	57.8	73.5	50.3	56.6	76.4	80.0	63.1	81.4	39.3	58.5
Tropical Latin America	55 to 59	52.6	68.2	55.1	70.5	39.3	60.6	55.1	64.5	66.1	72.9	42.3	52.8	54.1	65.5	45.7	51.3	32.6	48.5	46.3	66.8	59.8	78.5	37.9	67.0
Tunisia	55 to 59	61.2	73.3	61.4	75.0	64.3	74.1	67.2	75.0	67.7	74.7	63.9	72.4	77.1	87.6	78.4	86.7	76.3	87.5	39.8	54.8	41.6	61.0	51.1	59.1
Turkey	55 to 59	62.7	79.2	69.1	83.2	59.8	76.8	71.5	81.3	74.1	82.9	65.4	75.7	52.5	78.0	62.8	81.0	42.9	73.9	53.6	72.6	64.4	78.2	53.5	73.3
Turkmenistan	55 to 59	53.2	54.6	59.5	64.9	50.5	47.7	64.7	59.0	66.6	65.2	59.6	49.0	50.9	47.6	54.7	54.6	50.7	45.1	36.7	44.8	49.4	57.1	36.1	43.6
Tuvalu	55 to 59	29.2	50.1	42.2	59.5	19.6	44.1	53.7	62.0	60.0	65.9	43.0	54.3	51.1	72.2	55.3	71.0	46.1	74.4	57.6	67.4	67.3	75.2	54.1	65.2
Uganda	55 to 59	50.5	62.5	64.9	72.4	40.4	54.4	51.8	59.6	61.8	67.2	38.2	48.2	69.9	78.9	78.5	83.2	67.3	78.3	75.4	79.4	77.1	80.7	79.6	82.5
Ukraine	55 to 59	63.8	68.6	68.1	76.4	62.4	63.9	68.7	71.0	70.4	75.7	63.9	62.3	55.9	66.9	59.3	72.2	56.0	64.3	45.6	53.6	56.9	68.4	46.1	49.9
United Arab Emirates	55 to 59	68.0	85.7	65.1	89.6	71.8	87.2	73.1	88.1	71.7	88.2	70.8	87.1	53.7	86.2	43.9	90.0	62.4	86.7	52.9	81.4	50.5	84.4	63.4	88.1
United Kingdom	55 to 59	75.2	86.4	77.6	88.5	75.3	85.8	81.6	92.2	82.4	91.3	77.0	89.5	61.7	81.7	60.6	80.7	65.4	83.3	73.4	86.6	78.7	88.4	77.2	91.3
United Republic of Tanzania	55 to 59	53.2	70.6	61.4	71.5	48.8	72.4	58.1	68.6	62.7	68.2	49.8	66.7	74.9	83.3	78.6	81.8	75.2	87.2	75.3	78.3	76.0	79.0	80.7	82.9
United States Virgin Islands	55 to 59	58.1	75.5	67.8	84.0	48.0	68.7	62.8	75.9	70.6	82.5	49.1	66.1	67.2	82.0	70.0	83.9	61.8	79.2	59.3	77.6	68.1	83.4	54.8	77.5
United States of America	55 to 59	82.6	100.0	83.3	100.0	83.5	100.0	83.7	93.4	84.2	92.9	79.4	90.1	63.9	78.1	64.5	79.8	62.3	72.3	83.7	100.0	84.3	100.0	88.4	100.0
Uruguay	55 to 59	69.8	79.9	74.8	83.6	67.6	77.9	74.3	85.8	80.0	88.9	62.6	77.4	57.2	70.1	60.7	72.7	53.2	67.1	72.5	80.4	78.3	84.6	75.6	83.3
Uzbekistan	55 to 59	58.1	69.4	64.6	75.2	55.1	66.4	62.9	73.6	65.6	74.7	56.9	69.3	75.5	65.3	78.4	72.5	75.2	61.8	51.0	62.2	63.0	71.6	48.9	61.8
Vanuatu	55 to 59	47.3	57.2	61.6	67.9	38.0	49.4	62.4	66.6	69.6	71.4	51.4	57.8	62.4	75.4	67.1	75.5	59.8	75.9	68.0	75.5	77.9	82.3	64.9	73.3
Venezuela (Bolivarian Republic of)	55 to 59	60.5	65.2	68.1	75.3	54.1	54.8	69.8	69.7	74.0	78.1	60.4	54.5	73.6	72.6	74.0	72.7	72.1	71.2	67.2	76.2	74.5	82.3	65.5	74.1
Viet Nam	55 to 59	36.9	48.2	54.8	67.4	26.3	39.0	55.2	57.7	64.2	70.1	42.5	43.4	59.7	71.3	71.5	80.0	49.5	65.3	49.6	52.3	67.1	72.7	44.0	48.7
Yemen	55 to 59	24.9	45.4	28.3	47.6	27.9	48.2	40.2	45.4	49.0	48.1	27.0	40.9	42.1	58.2	53.1	62.2	34.3	55.2	0.0	15.0	1.1	25.2	18.0	22.3
Zambia	55 to 59	44.2	50.5	52.0	60.4	39.8	44.1	51.5	51.6	53.9	54.1	46.1	44.9	67.7	70.9	68.8	71.5	70.0	73.9	73.2	71.4	73.8	75.0	79.0	73.6
Zimbabwe	55 to 59	54.9	37.6	60.6	47.7	53.0	27.6	36.4	0.0	31.4	0.0	34.7	0.0	68.3	57.2	67.5	57.0	71.8	58.9	76.7	67.8	79.1	74.2	79.1	60.1
Afghanistan	60 to 64	18.8	37.1	22.1	37.0	25.7	46.2	34.2	41.0	49.8	48.0	11.9	32.6	35.3	53.9	47.7	53.2	26.1	48.6	11.9	21.8	0.0	17.1	38.2	43.5

Albania	60 to 64	46.0	65.5	54.2	69.6	46.0	65.9	51.2	63.7	59.3	67.9	38.8	56.5	75.0	87.1	74.6	83.1	71.8	85.2	64.2	76.4	68.7	76.9	71.5	83.3
Algeria	60 to 64	65.2	73.9	65.1	73.4	69.2	77.2	69.8	74.0	71.8	73.7	66.4	73.0	80.0	86.5	78.3	83.4	76.9	84.0	56.7	67.1	52.1	62.1	71.0	79.6
American Samoa	60 to 64	56.0	60.4	64.6	66.6	54.2	59.6	64.9	65.1	70.7	70.0	56.1	57.2	82.8	86.4	71.0	73.7	87.5	91.7	70.9	74.4	74.2	75.4	76.4	80.6
Andorra	60 to 64	79.4	87.7	80.6	87.9	80.6	89.0	84.1	90.9	84.8	90.5	81.9	90.1	80.9	91.0	71.3	83.3	85.2	92.7	80.4	89.1	78.6	86.4	89.3	96.2
Angola	60 to 64	45.4	56.9	57.1	65.0	40.0	52.5	46.1	51.5	54.8	58.0	32.9	41.4	76.8	82.3	76.6	79.5	73.6	79.7	65.1	68.3	70.5	72.2	66.7	69.3
Antigua and Barbuda	60 to 64	46.1	63.0	59.2	73.6	39.1	55.9	52.2	62.0	63.3	73.6	36.3	45.5	72.0	79.7	70.6	77.6	67.0	75.5	56.9	70.9	62.6	74.3	60.6	73.9
Argentina	60 to 64	64.4	77.9	70.3	82.0	63.9	77.2	74.2	80.4	81.4	86.5	63.6	71.5	62.5	81.8	60.8	78.0	58.7	79.9	70.0	83.1	72.3	83.1	77.2	89.2
Armenia	60 to 64	63.5	76.2	66.6	81.5	64.8	74.5	62.9	76.6	66.0	83.2	58.0	67.0	68.9	86.2	65.1	84.3	68.9	82.2	61.0	68.8	62.4	72.4	69.3	74.9
Australia	60 to 64	76.5	89.7	78.6	91.0	77.5	90.1	80.1	92.3	81.1	93.2	77.2	90.3	72.8	89.6	69.8	86.9	69.1	84.9	78.4	91.7	77.1	89.3	87.5	97.8
Austria	60 to 64	75.6	90.5	80.3	92.3	74.8	90.3	85.5	95.2	87.8	95.5	81.4	93.5	92.7	97.5	88.8	93.4	90.3	94.3	67.8	90.8	70.4	89.1	76.2	97.0
Azerbaijan	60 to 64	51.4	62.8	58.7	66.9	50.3	63.2	58.9	65.2	64.4	67.7	50.4	60.5	86.3	92.3	84.1	88.6	83.9	89.8	59.9	71.5	65.7	73.3	64.0	77.2
Bahamas	60 to 64	48.5	68.1	58.0	75.5	44.4	64.0	52.3	66.7	61.9	74.6	37.9	55.2	73.4	82.4	69.7	78.6	70.7	79.3	58.2	75.1	60.1	76.2	65.5	80.3
Bahrain	60 to 64	42.6	75.5	43.6	72.5	49.0	81.0	54.2	79.9	60.4	79.3	44.9	79.6	56.9	88.3	46.6	82.7	62.2	86.9	28.8	64.8	26.4	58.3	45.5	76.5
Bangladesh	60 to 64	21.9	49.6	21.2	49.6	30.8	55.3	51.4	66.0	57.6	69.7	41.2	59.7	37.1	65.8	33.3	55.8	40.5	70.3	28.8	52.5	20.6	49.4	48.2	65.6
Barbados	60 to 64	57.1	69.4	65.7	74.4	53.0	67.7	59.8	68.5	67.4	74.4	48.3	59.4	73.3	78.6	72.6	74.1	67.8	76.6	56.4	70.0	60.5	70.0	62.0	77.4
Belarus	60 to 64	62.4	67.8	65.8	75.3	63.8	65.0	70.1	71.2	71.8	77.0	67.2	62.4	68.0	70.7	66.9	73.7	63.8	62.8	50.5	59.3	52.2	67.6	60.6	63.5
Belgium	60 to 64	70.9	84.2	76.2	88.6	69.9	82.7	77.8	85.4	82.9	90.3	69.8	78.2	83.3	85.8	78.7	82.1	82.5	83.7	70.2	86.5	71.9	87.3	78.9	92.5
Belize	60 to 64	49.1	57.6	58.0	60.6	47.0	59.8	50.6	56.0	59.6	57.1	37.3	51.0	77.3	81.1	74.4	75.4	74.6	80.8	65.3	66.8	70.2	66.9	69.4	75.3
Benin	60 to 64	45.5	54.0	50.9	61.0	47.0	52.7	53.8	61.6	51.2	60.9	53.8	59.9	76.8	83.2	71.0	77.7	76.5	81.9	53.3	55.3	68.5	72.0	44.9	44.9
Bermuda	60 to 64	66.6	84.8	75.9	94.1	61.0	77.5	71.5	84.4	79.8	95.9	58.8	71.6	80.5	92.2	81.5	94.7	71.6	81.9	60.0	82.8	68.6	88.8	61.0	83.2
Bhutan	60 to 64	45.5	60.7	40.4	60.2	55.8	65.4	63.0	71.8	65.1	73.4	59.5	68.2	55.4	69.3	45.0	60.2	61.9	73.4	50.6	62.0	38.7	58.6	70.8	74.4
Bolivia (Plurinational State of)	60 to 64	37.1	57.5	43.1	63.0	37.9	56.4	42.1	54.8	49.1	61.6	31.6	44.2	52.4	63.5	43.1	53.6	57.1	68.5	46.2	64.4	55.5	72.1	47.1	64.7
Bosnia and Herzegovina	60 to 64	60.8	74.1	63.6	78.6	62.9	73.4	52.9	68.4	58.6	74.9	44.1	58.9	51.5	76.3	46.2	73.4	53.5	74.4	50.1	66.0	48.9	68.3	63.6	73.8
Botswana	60 to 64	46.4	64.2	54.7	71.0	44.6	60.3	48.4	63.6	49.6	66.8	43.0	58.0	86.1	92.7	84.2	90.0	83.1	89.7	58.4	70.1	65.1	74.8	59.2	70.1
Brazil	60 to 64	50.5	66.7	58.4	72.8	49.7	65.3	57.9	65.8	66.2	73.4	45.1	54.0	69.8	65.8	63.3	57.2	71.4	70.3	43.0	66.8	54.1	73.8	48.0	70.8
Brunei Darussalam	60 to 64	70.2	80.0	72.0	79.7	72.1	82.9	81.7	87.8	84.4	87.9	77.2	86.5	68.8	80.7	62.1	75.2	72.1	81.7	72.4	82.4	70.4	78.6	83.1	90.8
Bulgaria	60 to 64	45.5	58.1	50.7	67.8	47.7	55.7	52.9	63.5	57.8	70.4	43.9	53.5	68.2	62.8	64.3	64.0	68.8	58.7	43.3	43.5	47.5	55.0	54.3	49.4
Burkina Faso	60 to 64	52.6	54.8	69.4	72.5	43.3	44.4	57.4	60.8	66.8	70.5	44.5	47.9	84.2	86.5	89.0	89.9	75.6	78.3	64.0	61.9	82.6	83.1	52.4	48.1
Burundi	60 to 64	30.0	46.7	46.2	57.8	19.4	43.4	33.2	40.6	49.4	52.4	9.7	23.9	67.2	78.1	71.2	79.2	59.8	75.1	57.2	67.8	59.6	69.3	62.5	74.3
Cabo Verde	60 to 64	73.6	68.4	78.5	81.5	71.1	59.8	79.5	76.4	80.5	80.7	77.2	69.9	93.9	94.3	91.7	92.5	88.6	88.3	71.9	57.9	82.7	83.2	62.7	40.5
Cambodia	60 to 64	24.9	44.4	26.9	50.7	32.4	44.5	42.2	54.7	45.8	60.5	37.1	46.0	58.7	73.9	54.0	70.0	59.3	72.8	50.2	61.4	53.0	64.6	58.8	67.5
Cameroon	60 to 64	48.5	51.1	58.1	61.1	46.0	48.3	56.6	59.5	60.1	62.0	50.0	53.9	80.1	82.3	78.6	79.4	76.2	79.1	60.2	53.6	75.6	73.8	51.4	43.0
Canada	60 to 64	81.2	95.0	84.6	95.9	80.5	94.9	88.5	100.0	91.3	100.0	83.8	98.9	80.4	90.2	77.1	86.2	76.9	87.4	82.3	95.2	81.6	94.0	90.1	99.3
Central African Republic	60 to 64	27.3	34.4	43.2	49.9	19.8	25.7	35.0	34.6	45.9	46.2	18.7	17.5	66.1	70.4	67.8	71.7	62.1	66.0	52.4	57.7	59.7	65.0	53.5	56.9
Chad	60 to 64	50.9	46.1	57.9	56.3	49.6	44.7	57.1	56.2	58.0	56.6	53.9	51.3	79.7	77.3	76.2	72.0	77.0	76.4	59.3	47.1	72.8	70.3	50.8	37.3
Chile	60 to 64	65.1	84.2	69.2	87.5	65.6	83.1	74.7	89.3	80.8	92.9	64.6	83.3	72.7	83.7	66.6	80.5	74.7	80.7	66.5	86.1	67.4	86.7	75.3	91.0
China	60 to 64	32.3	62.4	41.7	70.0	32.3	60.5	53.7	67.9	62.4	75.3	41.0	57.4	0.0	70.3	0.0	70.8	0.0	66.9	43.3	65.3	48.3	68.3	53.0	73.1
Colombia	60 to 64	63.6	79.0	68.7	83.7	62.2	75.3	66.9	81.3	73.0	86.3	56.7	71.7	79.4	76.8	74.0	72.3	78.7	74.9	61.5	78.7	65.6	82.6	66.1	80.2
Comoros	60 to 64	47.1	61.8	56.3	67.8	43.2	58.9	44.7	54.1	52.1	60.1	33.2	45.1	79.3	86.2	77.2	83.2	77.0	83.6	67.2	74.8	69.6	75.8	70.7	77.9
Congo	60 to 64	46.5	56.5	54.3	61.0	43.2	56.4	45.9	49.6	50.7	51.4	38.6	45.8	76.6	81.9	72.8	75.6	75.7	82.4	64.6	67.6	68.4	69.8	65.4	71.5
Cook Islands	60 to 64	57.2	75.7	67.4	83.4	54.3	72.6	65.3	77.2	73.4	84.7	54.4	68.0	82.5	99.6	74.0	90.7	84.1	99.3	69.5	82.7	73.4	84.1	75.2	87.6
Costa Rica	60 to 64	75.5	85.2	77.8	90.0	75.5	80.9	78.3	85.8	80.2	90.9	74.5	77.8	86.5	88.7	79.9	86.2	87.0	83.5	75.7	85.8	76.2	87.3	81.7	88.1
Coted'Ivoire	60 to 64	53.4	54.1	65.5	64.8	51.1	51.4	62.2	62.4	65.1	64.1	55.4	57.4	81.2	84.5	79.8	80.7	78.3	82.2	51.2	48.3	74.2	73.5	46.1	38.3
Croatia	60 to 64	55.4	73.5	59.8	79.5	57.0	72.7	55.5	69.1	63.5	80.3	43.4	54.7	57.7	67.9	51.9	65.9	59.6	65.4	55.4	75.5	57.4	76.9	66.8	84.1
Cuba	60 to 64	62.0	69.0	66.7	77.3	61.7	64.9	66.6	69.2	71.8	77.2	58.3	57.6	82.6	85.9	78.4	82.2	79.6	82.3	59.2	63.4	60.6	71.0	67.1	66.1
Cyprus	60 to 64	66.2	83.7	71.9	87.5	65.4	82.7	77.0	88.3	83.3	91.5	67.3	83.7	72.1	89.2	67.8	84.2	71.8	88.3	67.5	84.5	68.2	86.2	77.2	89.2
Czechia	60 to 64	57.0	80.0	63.9	85.0	56.6	78.8	61.6	83.7	69.4	89.4	50.3	76.7	63.0	83.0	58.8	81.9	62.3	78.8	44.7	78.2	50.9	79.5	54.4	85.7
Democratic People's Republic of Korea	60 to 64	43.4	43.6	51.4	52.8	41.9	42.1	54.7	50.0	63.9	60.8	40.8	34.2	48.4	58.1	47.4	58.2	44.0	54.7	55.6	55.0	57.1	58.3	65.1	64.1

Democratic Republic of the Congo	60 to 64	49.1	50.5	55.8	59.1	47.8	47.1	51.6	48.5	55.6	55.4	44.5	37.9	80.3	80.8	76.6	78.4	79.2	79.0	64.4	67.0	66.0	70.0	69.6	70.0
Denmark	60 to 64	72.8	85.5	75.3	90.6	73.8	83.6	76.3	85.4	79.4	90.8	71.1	78.5	74.3	88.2	68.0	84.8	75.5	85.3	74.1	87.3	73.2	89.4	83.8	92.4
Djibouti	60 to 64	50.6	60.3	62.0	70.4	46.5	57.3	48.1	53.6	56.7	60.3	35.4	42.9	78.9	84.3	80.0	83.4	75.7	81.8	71.2	71.3	73.8	77.1	75.3	73.6
Dominica	60 to 64	44.0	52.8	57.0	68.4	32.0	45.5	44.2	50.1	57.6	64.4	22.2	31.8	68.4	74.9	69.9	78.7	57.6	67.6	51.2	55.4	59.9	66.7	47.4	56.9
Dominican Republic	60 to 64	51.4	60.9	60.8	70.4	48.0	56.7	54.1	62.9	64.2	70.5	39.3	51.8	74.9	81.3	71.9	77.1	70.7	79.1	60.0	65.9	65.4	72.2	64.0	69.2
Ecuador	60 to 64	64.3	78.0	71.4	83.0	61.1	75.1	61.6	74.0	67.8	80.2	52.0	64.1	84.1	74.9	81.8	71.3	79.6	71.7	63.5	82.8	71.7	86.4	64.0	84.2
Egypt	60 to 64	28.1	56.4	36.6	60.5	28.8	58.1	26.6	57.8	44.1	63.8	1.7	48.6	50.6	76.0	61.5	75.9	36.9	71.8	0.0	34.6	2.1	36.7	18.6	47.7
El Salvador	60 to 64	60.8	74.2	66.8	79.5	58.2	68.7	66.2	72.2	72.2	78.6	56.2	59.6	78.8	84.9	74.4	79.9	77.1	83.0	65.0	76.1	70.4	80.5	67.1	75.1
Equatorial Guinea	60 to 64	36.5	61.8	50.1	67.3	29.3	58.9	38.4	60.6	48.1	64.6	23.8	54.6	72.1	88.9	73.2	83.9	68.1	87.9	59.7	66.8	66.7	70.2	59.3	67.0
Eritrea	60 to 64	26.0	48.8	42.2	60.1	17.2	39.4	28.4	40.2	41.0	52.3	9.8	22.3	67.8	80.6	73.2	80.9	59.6	74.3	63.4	71.3	65.6	73.9	67.9	71.1
Estonia	60 to 64	56.1	78.8	61.9	87.8	56.1	73.4	64.2	80.3	69.4	88.1	56.0	69.9	77.2	78.7	80.0	82.1	66.8	70.6	37.4	74.5	41.3	82.9	48.8	76.5
Eswatini	60 to 64	34.8	39.9	50.7	52.8	25.4	31.8	36.4	39.2	45.3	44.0	22.4	30.0	82.7	85.5	83.3	83.9	77.9	81.4	53.8	51.5	64.6	62.4	49.2	45.5
Ethiopia	60 to 64	32.1	55.6	47.2	64.1	26.4	52.5	44.6	55.1	55.8	62.4	28.7	44.0	76.4	85.4	79.6	84.0	72.1	82.9	70.3	75.7	70.2	75.0	77.6	81.8
Fiji	60 to 64	51.0	59.4	57.0	64.3	51.1	59.3	54.3	55.5	61.2	62.7	43.9	44.9	76.4	82.5	67.9	73.9	81.9	89.0	69.1	74.0	70.9	74.3	75.8	80.8
Finland	60 to 64	75.1	89.2	77.6	92.0	76.2	88.5	83.5	92.9	85.7	95.3	79.6	89.7	86.4	93.1	78.9	89.4	87.5	89.3	69.7	89.4	68.4	88.7	80.2	95.1
France	60 to 64	74.9	86.5	83.1	89.3	72.5	86.2	75.6	90.0	83.3	93.3	64.8	85.9	87.8	91.2	84.5	88.0	84.9	87.6	75.8	87.8	82.2	86.6	82.3	94.6
Gabon	60 to 64	56.0	63.4	66.4	72.3	49.3	59.5	55.3	60.2	63.8	65.9	42.9	51.1	83.4	87.3	82.7	85.4	79.3	84.2	71.6	71.8	74.7	76.0	73.2	74.6
Gambia	60 to 64	51.6	49.1	62.2	59.7	49.8	45.9	58.6	55.9	58.2	56.8	54.0	51.5	81.7	80.6	78.6	75.2	79.4	79.4	50.4	45.1	73.8	69.8	42.5	31.7
Georgia	60 to 64	46.0	62.8	56.9	73.4	41.4	57.6	59.8	69.9	66.1	76.9	50.2	60.3	75.4	70.0	74.0	68.4	73.1	68.5	58.0	59.6	65.9	69.1	57.8	60.5
Germany	60 to 64	77.1	86.2	80.0	89.0	77.2	85.8	80.2	90.3	84.7	92.9	72.8	86.4	85.6	91.8	80.3	86.1	84.7	90.2	75.4	86.2	75.3	85.6	83.1	92.9
Ghana	60 to 64	53.4	58.7	61.0	68.9	50.7	52.7	54.5	59.9	54.2	61.3	53.3	55.9	81.8	85.4	77.2	80.2	79.2	82.7	55.9	55.3	74.1	76.2	40.4	38.7
Greece	60 to 64	68.4	80.9	70.4	86.1	70.5	79.3	74.6	82.3	77.8	87.8	69.1	75.2	85.6	89.2	81.0	86.5	84.4	84.9	71.9	86.3	70.8	86.8	81.4	92.2
Greenland	60 to 64	69.2	76.6	69.9	81.6	72.3	76.6	69.0	74.8	73.3	81.1	62.1	67.3	22.5	62.2	9.4	58.9	32.1	61.7	75.4	84.6	75.1	86.3	82.9	90.6
Grenada	60 to 64	32.4	60.5	38.9	67.3	33.4	59.4	41.3	58.3	49.6	65.8	28.7	47.3	63.4	75.0	56.6	70.2	65.1	74.2	24.1	64.3	27.6	66.4	35.2	71.1
Guam	60 to 64	66.5	79.9	70.6	83.8	66.9	78.6	71.8	79.6	74.9	82.6	66.2	74.7	83.3	97.3	71.0	85.9	88.9	100.0	70.4	84.4	72.3	85.5	77.8	89.1
Guatemala	60 to 64	63.1	71.8	66.4	75.2	63.7	70.7	70.6	72.6	73.5	76.4	65.7	66.0	95.2	90.7	91.6	86.8	91.7	87.2	66.7	78.4	68.6	79.8	72.8	81.8
Guinea	60 to 64	50.2	51.4	52.2	57.0	54.5	52.5	57.6	60.2	53.8	57.3	59.6	59.6	82.4	83.2	74.7	75.5	82.6	83.1	57.4	50.4	69.4	70.3	52.5	41.5
Guinea-Bissau	60 to 64	29.2	37.3	46.0	50.2	22.6	32.2	42.8	46.5	47.2	48.4	33.8	41.2	66.0	70.6	67.4	66.8	60.9	68.8	33.5	36.5	67.1	67.4	11.9	14.3
Guyana	60 to 64	0.0	38.8	13.3	50.9	0.0	34.3	27.5	46.8	38.5	57.0	10.6	31.4	57.1	67.5	58.6	67.8	51.0	61.3	4.4	43.7	31.1	57.2	0.0	43.3
Haiti	60 to 64	1.2	21.0	4.4	25.3	9.9	25.9	27.6	31.4	37.9	41.8	11.8	15.9	27.2	41.3	18.6	30.4	32.4	48.5	13.2	31.7	7.5	30.2	36.4	47.6
Honduras	60 to 64	45.7	33.1	53.7	40.3	43.5	32.9	46.4	32.2	56.0	42.7	31.7	16.2	64.8	42.5	60.6	30.0	63.3	51.2	48.0	39.0	58.6	51.3	48.7	39.4
Hungary	60 to 64	60.0	72.5	66.6	80.4	59.2	69.4	62.4	73.8	68.3	82.2	53.4	62.9	71.4	76.5	70.1	74.5	65.6	72.6	49.8	65.5	55.9	72.0	58.2	71.0
Iceland	60 to 64	77.7	90.2	79.4	91.9	78.7	90.0	85.1	94.4	85.6	95.4	83.2	92.7	85.8	95.1	78.0	88.4	88.1	94.6	77.9	91.6	75.4	89.8	88.2	98.1
India	60 to 64	40.7	51.8	41.7	53.0	46.7	56.7	60.7	68.5	66.3	71.6	51.9	63.1	49.6	61.3	48.0	54.3	51.0	64.5	45.7	53.0	40.4	51.9	61.8	64.6
Indonesia	60 to 64	47.5	49.8	51.5	57.2	49.9	48.9	57.6	57.1	62.9	62.8	50.1	48.3	67.8	72.3	62.1	66.7	69.1	73.4	63.6	59.3	67.0	67.6	69.0	62.1
Iran (Islamic Republic of)	60 to 64	58.7	76.9	63.7	79.1	59.5	77.7	66.6	78.3	71.9	80.7	58.4	73.9	72.0	90.0	73.0	88.8	68.2	85.2	35.2	63.4	38.4	63.7	47.6	72.1
Iraq	60 to 64	37.7	52.7	46.2	61.2	37.6	51.2	50.3	60.4	59.2	67.2	37.3	50.5	69.8	79.3	71.2	78.1	63.0	75.1	13.5	34.8	21.6	43.7	24.7	42.5
Ireland	60 to 64	72.6	89.9	76.2	91.7	72.7	89.4	81.2	94.1	83.3	95.6	77.2	91.2	78.1	91.2	71.4	87.8	80.0	88.1	70.6	90.8	72.5	89.7	79.6	96.0
Israel	60 to 64	74.5	90.1	74.6	93.0	77.3	89.5	71.8	82.6	76.0	88.6	65.0	75.1	90.2	94.7	85.1	92.5	89.5	90.2	79.7	93.8	74.5	92.6	90.3	98.1
Italy	60 to 64	67.6	84.0	72.4	87.9	67.6	83.3	72.2	85.2	78.4	88.8	63.0	80.2	79.3	86.8	73.7	83.7	79.9	84.2	67.3	87.5	68.9	88.0	76.4	93.7
Jamaica	60 to 64	39.9	54.1	46.7	59.5	40.1	54.1	51.0	59.5	58.1	65.2	39.9	50.5	72.8	79.1	65.8	73.5	74.5	78.6	44.0	60.5	46.1	62.2	54.1	68.4
Japan	60 to 64	78.3	90.6	81.2	92.4	78.8	91.1	88.7	97.6	91.7	98.4	84.2	96.5	77.4	92.1	72.4	88.2	77.8	90.2	78.3	91.3	77.9	91.2	86.7	96.6
Jordan	60 to 64	59.9	77.4	58.5	76.9	65.5	80.4	62.3	75.2	63.6	75.9	59.8	73.0	93.3	97.9	90.0	95.8	91.1	94.2	45.9	72.8	38.9	67.2	64.2	84.6
Kazakhstan	60 to 64	62.3	64.7	68.5	72.7	60.1	60.8	64.4	63.8	69.1	70.1	57.2	54.3	61.5	62.1	60.5	60.6	58.7	61.2	55.4	62.5	61.6	69.7	58.1	64.7
Kenya	60 to 64	61.3	61.4	68.1	70.5	58.4	56.7	59.9	56.6	66.7	63.9	49.7	45.8	85.1	86.1	83.5	84.5	82.7	83.2	78.4	76.5	77.0	78.5	84.4	79.7
Kiribati	60 to 64	51.7	54.0	63.3	65.5	48.6	50.3	68.0	66.5	73.1	72.2	60.3	58.1	79.9	86.4	79.0	81.4	74.4	85.1	67.7	66.7	76.3	77.8	69.8	66.7
Kuwait	60 to 64	77.7	86.8	77.5	93.6	80.3	85.4	82.6	91.5	83.9	96.5	79.8	87.8	88.2	100.0	83.2	100.0	87.3	94.5	69.3	81.9	62.1	86.0	81.5	87.8
Kyrgyzstan	60 to 64	51.1	57.4	58.5	66.4	49.6	54.2	60.0	65.9	63.1	70.5	54.5	58.4	68.1	62.5	70.8	66.2	61.2	56.7	45.7	44.8	54.4	55.9	48.5	47.9

Lao People's Democratic Republic	60 to 64	17.8	44.6	21.2	49.9	24.5	46.7	38.1	54.6	43.1	58.7	29.0	47.0	45.4	72.0	38.8	67.8	48.9	72.0	33.4	55.8	38.5	62.5	44.6	62.0
Latvia	60 to 64	55.3	69.0	61.9	75.1	54.0	67.7	59.7	70.6	66.1	77.4	50.1	60.9	70.0	72.5	71.3	71.4	62.4	69.6	43.8	61.3	48.9	65.6	52.0	69.1
Lebanon	60 to 64	51.9	82.2	59.9	84.6	51.9	82.2	50.1	80.5	67.3	84.4	26.7	73.8	49.4	89.5	53.8	88.2	42.1	83.3	49.3	81.9	49.3	80.4	61.9	89.2
Lesotho	60 to 64	34.4	23.3	49.8	42.0	20.4	4.1	34.6	27.8	45.1	40.2	18.9	9.6	82.0	78.1	83.7	78.8	74.0	70.6	57.8	41.3	65.1	56.3	51.6	21.4
Liberia	60 to 64	50.3	49.3	54.3	54.5	52.7	50.8	58.2	55.3	54.7	51.8	58.9	56.0	82.4	82.4	75.1	74.7	82.5	82.6	53.8	48.4	68.9	68.0	48.2	38.4
Libya	60 to 64	64.7	67.6	63.7	67.9	69.8	71.3	68.3	67.1	68.9	66.5	67.4	68.4	82.3	84.0	76.5	76.0	82.9	85.5	53.5	53.0	48.2	51.9	69.3	64.6
Lithuania	60 to 64	63.0	72.7	68.9	79.5	61.0	69.9	68.8	78.1	73.1	83.2	62.0	70.6	71.3	74.9	74.1	76.3	62.2	67.7	56.1	70.0	58.5	74.1	65.1	76.5
Luxembourg	60 to 64	67.8	83.9	73.6	88.4	67.1	83.0	71.7	85.8	78.2	90.4	62.1	79.7	79.6	89.7	73.5	84.9	80.8	88.6	64.7	86.5	69.3	87.7	71.9	93.1
Madagascar	60 to 64	39.0	47.1	52.0	55.3	33.5	45.1	41.9	42.2	52.0	48.9	27.2	31.3	74.0	78.5	74.8	74.5	70.2	77.5	66.1	69.1	70.9	71.5	67.3	72.4
Malawi	60 to 64	44.8	45.2	50.6	54.2	45.6	43.2	38.9	32.8	44.8	40.6	29.3	19.9	75.7	76.2	73.2	74.0	74.4	74.7	66.8	61.2	66.6	66.6	73.9	64.2
Malaysia	60 to 64	53.3	68.3	59.7	72.9	53.1	68.0	62.1	71.9	67.7	75.3	53.5	66.2	76.0	85.4	72.9	81.0	74.5	84.1	62.8	72.8	65.1	74.4	70.4	78.8
Maldives	60 to 64	45.0	74.3	44.1	77.2	51.3	75.3	53.5	76.8	51.3	75.6	49.4	75.0	71.1	92.1	64.9	87.5	73.4	90.4	59.7	78.7	58.5	79.3	71.2	85.2
Mali	60 to 64	47.9	52.8	48.1	54.3	54.1	57.1	54.4	59.1	49.6	52.9	57.2	61.4	80.4	84.8	71.0	74.9	82.0	85.9	61.6	57.8	66.9	68.1	63.1	55.5
Malta	60 to 64	66.7	87.5	70.2	89.1	67.4	87.6	75.3	91.3	78.8	91.4	69.3	90.1	89.4	96.0	84.1	91.3	89.1	94.1	63.5	88.6	62.4	86.7	75.0	95.5
Marshall Islands	60 to 64	31.7	44.2	52.9	54.5	21.8	41.2	49.8	55.9	64.9	64.2	30.7	43.8	63.5	75.1	59.8	66.1	62.1	78.5	59.4	66.2	69.0	69.3	60.1	71.3
Mauritania	60 to 64	50.8	60.7	54.1	64.4	53.6	61.5	55.5	63.3	48.7	59.4	60.9	66.7	80.7	87.4	72.1	79.1	81.8	87.3	55.3	59.8	69.6	72.9	46.2	50.7
Mauritius	60 to 64	48.2	70.5	62.8	79.1	42.8	66.4	58.4	74.7	69.2	79.5	43.8	67.5	70.6	84.1	75.3	85.2	59.3	76.1	39.4	70.0	58.6	77.6	40.1	72.4
Mexico	60 to 64	72.8	80.4	77.2	85.3	70.2	76.8	73.9	79.0	79.0	84.4	64.4	69.4	88.6	81.2	84.0	77.3	86.3	78.5	69.6	80.3	72.4	84.1	73.4	81.8
Micronesia (Federated States of)	60 to 64	35.8	46.0	46.2	56.8	34.9	43.1	54.2	58.4	62.0	66.9	42.7	46.4	64.2	76.4	56.3	68.4	67.1	78.9	58.4	63.5	62.8	68.6	64.9	68.1
Monaco	60 to 64	73.9	85.4	76.5	87.0	74.9	85.9	76.1	87.7	79.8	89.1	70.3	84.9	77.0	91.4	69.0	85.0	80.3	91.4	72.3	84.8	70.9	82.9	81.9	91.8
Mongolia	60 to 64	21.5	51.1	32.8	64.1	20.3	44.6	45.9	64.3	55.7	73.1	31.5	51.6	37.3	37.3	41.0	39.2	34.2	35.4	72.1	77.5	75.3	83.4	75.1	77.2
Montenegro	60 to 64	43.7	49.2	49.3	56.5	45.4	48.9	56.5	55.1	63.5	64.2	45.9	41.6	78.5	81.7	73.5	75.6	78.3	82.1	70.4	69.9	72.7	73.7	77.9	76.3
Morocco	60 to 64	51.0	59.8	45.0	56.9	61.7	66.5	56.9	60.8	56.2	59.7	57.4	60.5	68.3	73.4	62.0	66.5	70.4	75.6	35.7	45.4	21.6	35.4	61.4	66.3
Mozambique	60 to 64	32.7	33.6	50.1	56.9	26.1	23.5	34.1	32.6	48.8	51.9	14.1	8.8	67.8	66.6	74.3	74.3	62.0	60.3	55.2	48.6	65.1	67.6	55.8	44.1
Myanmar	60 to 64	18.1	41.4	24.5	52.6	22.5	39.0	38.3	50.6	42.6	58.0	29.1	39.3	51.0	70.7	49.7	69.2	49.6	67.8	34.8	54.6	45.3	65.3	41.5	56.5
Namibia	60 to 64	41.1	46.0	50.7	58.3	37.8	38.8	44.4	44.6	47.1	51.4	38.2	33.6	86.0	88.7	84.4	87.3	82.7	84.5	51.2	52.7	61.7	64.7	47.7	47.4
Nauru	60 to 64	40.2	44.6	51.2	55.4	39.0	39.2	52.7	49.4	61.6	60.7	40.1	32.8	64.6	71.6	50.9	59.3	72.3	79.5	53.8	58.8	64.7	65.9	57.1	59.0
Nepal	60 to 64	33.5	50.4	24.5	52.1	48.0	54.6	57.4	66.0	59.6	70.6	53.7	58.7	49.7	65.1	40.7	61.4	55.6	65.2	32.6	52.4	11.7	51.0	61.3	65.3
Netherlands	60 to 64	80.0	88.8	83.6	90.9	79.4	88.4	80.0	91.0	84.3	92.3	73.4	88.1	81.1	90.4	77.3	86.9	78.5	86.9	81.8	89.4	81.8	88.6	89.2	95.1
New Zealand	60 to 64	73.2	87.9	75.3	89.0	74.5	88.4	76.3	88.1	79.1	89.5	71.4	85.2	64.0	77.3	56.5	75.0	67.7	72.5	74.9	90.4	75.0	88.9	83.4	96.0
Nicaragua	60 to 64	65.2	72.8	71.0	79.0	62.7	68.7	68.7	73.4	74.4	79.2	59.3	63.5	83.5	85.6	79.3	81.1	81.9	84.1	69.2	75.6	72.4	79.3	74.7	79.0
Niger	60 to 64	51.0	51.2	59.7	57.9	49.5	50.6	57.4	56.2	60.2	57.6	50.9	52.0	79.4	81.8	77.8	78.5	76.6	79.3	65.7	63.8	75.2	74.7	64.2	58.5
Nigeria	60 to 64	45.7	64.6	57.3	71.8	44.6	60.0	56.5	68.7	57.6	70.8	49.8	65.2	78.0	90.6	79.6	87.5	74.7	86.0	49.6	63.5	71.7	77.6	45.9	50.3
Niue	60 to 64	55.2	61.1	65.0	71.5	52.3	57.4	63.4	65.4	71.4	74.4	52.1	53.6	79.1	88.3	71.0	79.3	81.4	90.1	69.3	72.4	73.4	77.5	74.0	76.8
North Macedonia	60 to 64	51.7	58.1	53.3	61.7	55.8	60.0	54.0	57.3	57.9	61.9	47.7	49.3	56.4	61.5	47.7	51.4	61.5	68.0	46.0	52.4	45.5	54.2	59.8	63.4
Northern Mariana Islands	60 to 64	53.8	62.4	55.0	69.2	57.9	62.9	65.6	69.8	64.2	72.5	61.2	63.7	78.0	85.2	54.9	69.9	88.1	89.5	65.6	72.9	66.5	76.6	74.5	80.0
Norway	60 to 64	77.9	88.4	79.5	91.4	79.1	87.8	81.1	91.3	84.9	92.7	75.0	88.9	78.0	95.4	69.7	94.1	80.4	88.9	78.4	89.7	76.2	88.5	87.8	96.5
Oman	60 to 64	52.3	74.3	58.1	75.5	53.7	76.7	57.7	76.7	65.9	78.6	45.9	74.0	76.0	88.5	77.0	86.7	72.2	86.7	38.3	65.6	41.9	63.8	50.7	76.0
Pakistan	60 to 64	56.4	58.8	53.7	57.5	63.2	64.5	69.7	69.5	71.3	70.9	66.2	66.4	63.5	65.5	56.7	56.4	67.0	69.9	61.8	61.2	53.8	58.2	76.8	73.1
Palau	60 to 64	51.3	62.8	71.5	82.2	40.2	50.5	62.0	67.4	77.6	83.8	43.3	47.8	82.4	92.7	77.6	87.5	81.3	90.3	62.0	70.7	74.6	84.0	60.4	66.7
Palestine	60 to 64	45.9	61.4	50.5	67.5	47.7	60.3	53.5	62.4	60.6	69.7	43.0	51.4	89.1	95.8	86.3	94.0	85.0	91.0	23.9	47.3	27.1	52.2	36.9	55.6
Panama	60 to 64	64.0	75.4	72.5	82.3	60.2	71.4	70.6	78.0	78.4	84.7	59.3	68.7	80.5	80.7	75.6	77.0	79.7	78.0	64.9	78.5	71.0	81.8	68.9	81.9
Papua New Guinea	60 to 64	10.1	24.9	0.0	16.9	25.9	37.1	31.9	41.1	23.2	33.6	29.8	38.3	53.5	65.4	30.7	45.1	68.2	76.5	50.5	57.7	37.3	47.3	72.6	75.9
Paraguay	60 to 64	50.7	60.5	55.7	67.2	52.0	59.7	57.0	59.0	63.9	67.0	46.3	47.3	68.6	69.5	60.3	59.7	72.3	74.9	54.4	64.1	61.3	71.7	60.5	68.6
Peru	60 to 64	63.7	79.7	70.4	82.6	61.2	79.0	65.1	75.3	71.7	79.0	55.0	69.2	77.4	85.6	72.9	77.1	75.9	86.8	70.7	81.2	75.8	84.1	73.0	83.2
Philippines	60 to 64	48.8	55.8	60.0	67.4	44.9	50.6	52.6	61.8	62.1	70.7	39.0	49.4	78.0	79.9	76.4	77.2	74.4	77.0	62.6	66.1	72.2	74.5	63.6	67.6
Poland	60 to 64	51.3	69.6	59.5	78.9	49.8	66.4	60.0	71.6	67.0	80.8	49.4	60.4	70.8	75.8	69.9	75.1	66.2	71.7	33.9	63.1	43.5	71.4	41.3	69.0
Portugal	60 to 64	60.6	78.0	67.0	83.5	60.1	77.0	65.3	78.4	72.3	85.1	55.0	69.8	73.8	80.6	70.5	78.1	72.4	76.9	60.3	82.6	64.7	84.8	68.3	87.9

Puerto Rico	60 to 64	75.4	89.4	82.8	94.9	69.7	82.6	78.0	85.6	84.2	92.8	68.6	75.1	87.3	92.4	84.1	90.2	82.5	85.8	75.0	91.6	80.5	93.9	75.3	90.5
Qatar	60 to 64	65.8	88.5	67.7	86.5	68.9	92.0	66.9	89.3	70.7	86.5	60.3	91.4	51.9	91.9	70.9	95.6	42.9	85.7	66.5	85.7	64.9	81.9	76.9	91.8
Republic of Korea	60 to 64	68.9	89.9	70.5	91.1	71.2	90.7	82.2	98.7	85.0	98.7	76.4	97.9	62.1	89.6	57.1	85.4	62.6	88.6	71.3	89.7	68.9	88.7	81.7	95.3
Republic of Moldova	60 to 64	45.9	60.9	50.3	68.3	48.2	59.1	52.7	62.2	58.1	67.8	44.6	53.6	73.3	73.4	73.8	75.1	68.9	68.5	60.7	57.9	61.6	64.3	69.8	63.7
Romania	60 to 64	51.7	61.6	56.0	69.9	53.7	59.7	51.2	54.7	56.1	65.0	42.7	40.6	63.3	60.6	59.5	60.1	64.2	58.9	51.5	60.8	54.4	67.2	62.2	67.4
Russian Federation	60 to 64	53.5	65.6	61.9	75.0	49.7	60.6	61.8	67.8	67.4	74.8	53.3	57.7	67.9	66.2	68.8	68.2	62.9	60.8	33.9	55.0	44.1	65.6	36.6	56.4
Rwanda	60 to 64	23.1	50.5	35.9	61.0	17.3	44.0	31.9	46.3	45.3	59.2	12.1	26.7	69.2	83.0	71.8	83.1	62.2	78.5	56.2	70.7	57.3	72.0	63.3	74.7
Saint Kitts and Nevis	60 to 64	41.9	53.0	47.7	69.2	41.9	44.1	51.0	55.2	57.4	70.4	41.2	35.7	67.7	76.7	62.4	75.3	66.8	72.1	44.6	54.0	48.1	66.6	51.1	53.4
Saint Lucia	60 to 64	35.8	63.1	48.5	74.7	27.9	56.6	44.1	62.7	57.9	74.7	23.8	47.9	62.0	82.2	52.3	79.6	67.7	77.8	33.8	66.5	39.6	70.2	38.8	71.1
Saint Vincent and the Grenadines	60 to 64	48.8	58.9	53.3	66.9	49.2	56.7	44.6	58.7	50.7	65.7	35.9	48.1	77.9	83.2	70.9	79.4	79.9	80.7	55.3	64.5	54.5	66.0	65.7	71.9
Samoa	60 to 64	49.2	57.0	62.0	64.3	44.1	55.0	60.0	62.3	70.7	69.0	45.6	52.2	75.8	84.5	69.4	73.7	76.5	89.1	69.2	73.8	73.8	77.6	73.8	77.2
San Marino	60 to 64	78.6	88.4	80.9	90.1	79.4	88.3	82.0	91.5	83.7	91.8	78.6	89.8	91.0	97.9	86.8	93.7	89.6	95.6	78.3	88.8	78.4	87.7	86.6	95.1
Sao Tome and Principe	60 to 64	67.4	67.4	70.8	71.7	67.5	66.4	69.7	70.5	69.2	70.0	70.0	70.5	90.6	91.0	84.5	83.4	88.6	89.7	70.3	65.0	78.2	76.0	64.3	57.6
Saudi Arabia	60 to 64	41.2	57.3	37.8	58.1	48.9	61.2	49.5	63.5	52.4	67.6	41.5	56.3	78.0	85.6	73.4	81.2	78.4	84.9	25.5	43.4	13.9	38.5	47.8	59.8
Senegal	60 to 64	52.1	55.6	61.4	64.3	49.8	52.2	57.0	59.7	57.3	59.7	53.4	57.0	81.0	83.4	77.5	77.8	78.6	81.7	52.1	54.4	72.0	73.1	42.0	40.8
Serbia	60 to 64	50.8	61.4	56.0	66.7	52.1	61.6	54.4	58.9	60.3	66.4	45.2	47.9	51.7	61.2	44.0	51.4	56.1	67.7	41.8	51.5	46.3	55.3	52.5	61.3
Seychelles	60 to 64	58.0	74.0	70.3	79.6	54.0	73.0	64.2	76.0	71.9	78.8	53.8	71.0	80.7	92.7	80.7	89.9	75.4	89.0	59.8	75.7	73.5	80.3	61.0	80.2
Sierra Leone	60 to 64	51.0	53.1	61.3	59.4	48.0	53.0	57.6	58.7	57.7	57.0	53.9	58.3	82.3	83.8	79.2	76.5	79.6	83.3	48.1	48.9	72.6	69.6	36.2	38.3
Singapore	60 to 64	74.2	93.2	73.2	91.8	78.2	95.5	85.2	99.1	85.9	97.7	83.3	100.0	82.7	96.3	75.5	90.4	85.9	96.0	72.9	94.1	65.6	89.4	85.4	100.0
Slovakia	60 to 64	61.9	73.6	67.7	78.9	61.7	72.8	55.6	68.4	65.2	76.5	41.9	57.5	73.2	81.6	69.6	78.5	72.2	78.9	58.7	70.9	62.2	73.0	67.3	78.9
Slovenia	60 to 64	58.2	81.5	67.1	87.3	55.3	79.8	64.7	85.5	73.2	90.8	52.5	79.7	73.6	90.6	70.4	87.2	70.5	87.9	50.0	80.9	57.8	83.1	56.8	88.0
Solomon Islands	60 to 64	45.0	49.9	54.4	58.9	43.5	47.2	63.6	65.2	70.5	72.3	53.4	54.6	69.1	77.4	62.5	70.6	70.9	79.5	68.8	71.1	69.3	71.8	76.8	77.9
Somalia	60 to 64	26.5	38.7	43.2	51.9	18.6	30.2	26.6	27.5	40.3	41.7	6.9	6.7	66.2	74.4	72.6	77.1	59.0	68.1	66.5	71.2	67.4	72.3	72.9	75.1
South Sudan	60 to 64	72.0	60.4	53.2	56.5	34.0	39.9	69.7	52.7	50.6	46.4	21.7	18.0	94.9	90.4	77.0	78.4	72.8	76.6	76.2	64.0	69.4	70.8	70.8	72.1
Spain	60 to 64	36.6	43.6	76.0	88.8	70.0	84.4	38.3	34.8	83.0	91.7	71.7	81.7	74.8	78.9	83.9	87.8	82.6	86.6	64.9	67.4	72.2	86.6	81.6	94.7
Sri Lanka	60 to 64	70.8	84.9	70.7	79.1	59.5	68.8	78.5	87.4	71.1	76.8	49.7	58.3	86.2	90.5	87.5	92.0	83.3	88.1	71.9	87.2	71.1	77.3	69.6	76.8
Sudan	60 to 64	61.9	72.1	26.8	54.3	48.7	66.1	62.1	69.2	45.3	55.2	37.7	57.7	87.3	92.9	49.7	61.7	55.8	72.9	64.7	73.1	5.9	37.8	55.5	65.6
Suriname	60 to 64	36.0	58.7	46.8	61.6	44.6	41.5	43.4	57.8	56.3	64.7	43.3	36.2	53.5	69.7	67.0	74.5	71.1	70.7	26.9	47.1	55.9	67.0	60.3	58.2
Sweden	60 to 64	42.1	47.8	81.7	93.6	78.3	92.0	51.5	52.7	87.4	95.7	80.8	96.5	71.8	75.6	72.0	84.5	81.8	90.6	52.6	57.1	78.6	93.0	86.5	97.6
Switzerland	60 to 64	78.3	91.9	80.1	90.5	76.0	89.1	84.8	96.3	86.2	95.6	78.1	93.0	79.5	90.5	74.8	86.9	79.2	89.9	78.3	93.0	77.6	88.0	84.6	96.1
Syrian Arab Republic	60 to 64	76.2	88.8	56.4	64.0	57.6	61.7	83.2	94.9	67.4	69.8	59.2	57.7	79.4	91.4	77.3	85.6	76.2	83.6	76.5	89.6	41.4	48.9	54.7	58.4
Taiwan (Province of China)	60 to 64	54.0	60.0	65.9	88.0	58.0	77.2	64.6	65.2	73.8	92.0	58.8	79.9	79.3	88.2	78.7	90.6	80.0	86.1	41.6	47.5	68.2	85.7	74.5	89.6
Tajikistan	60 to 64	58.3	81.2	52.1	60.6	46.9	58.7	67.5	86.2	62.0	63.7	54.1	59.2	81.3	91.8	70.1	73.5	80.2	82.7	65.3	84.6	47.7	57.7	40.6	64.3
Thailand	60 to 64	46.1	57.0	69.3	80.9	57.8	70.8	59.3	62.6	74.1	81.5	61.8	71.5	77.6	80.2	65.7	76.3	53.3	69.4	36.7	55.4	71.8	80.4	70.9	79.2
Timor-Leste	60 to 64	60.9	74.0	28.3	45.0	42.2	44.6	69.3	77.5	41.3	51.5	40.5	42.8	60.4	74.5	49.7	61.3	65.6	71.0	66.6	76.4	55.7	63.3	65.1	61.3
Togo	60 to 64	31.2	41.1	57.8	59.8	52.0	45.2	41.9	48.7	55.3	56.5	55.4	49.1	59.5	68.4	75.9	76.5	79.0	77.6	54.9	56.0	70.6	71.3	46.1	33.5
Tokelau	60 to 64	51.7	49.3	49.9	67.2	33.3	54.0	56.8	54.3	63.4	70.7	33.3	50.5	80.6	80.3	56.7	75.7	71.4	87.6	53.8	48.3	63.0	75.0	59.9	73.0
Tonga	60 to 64	37.6	59.0	68.9	74.3	62.4	65.7	51.0	62.7	73.8	75.8	62.0	63.7	67.0	84.7	76.3	82.2	88.8	94.2	56.6	71.1	76.9	79.6	77.5	80.2
Trinidad and Tobago	60 to 64	63.4	67.9	57.8	78.0	50.1	59.9	69.2	71.0	60.7	75.7	40.7	46.1	85.4	91.8	61.6	75.7	62.4	68.4	73.3	75.9	60.9	78.0	47.9	65.1
Tropical Latin America	60 to 64	50.7	66.1	58.3	72.6	49.8	65.1	52.9	63.3	66.2	73.3	45.2	53.8	64.8	75.1	63.3	57.3	71.4	70.4	46.8	65.6	54.3	73.7	48.2	70.7
Tunisia	60 to 64	58.8	70.4	58.7	72.5	63.4	72.0	64.2	72.9	67.4	75.3	58.7	68.6	77.8	87.2	76.6	84.9	75.0	83.8	47.3	61.0	41.5	60.4	63.8	71.5
Turkey	60 to 64	60.7	74.7	66.3	79.5	60.8	73.4	67.4	77.4	72.6	81.8	59.5	70.3	55.6	77.3	59.9	77.9	47.8	70.9	57.4	72.4	61.2	74.5	65.1	78.2
Turkmenistan	60 to 64	50.9	51.1	56.9	59.8	50.9	48.6	60.4	55.2	63.8	61.9	54.5	44.8	63.4	55.4	61.2	55.6	62.3	53.6	40.6	44.1	46.6	50.5	47.6	50.5
Tuvalu	60 to 64	28.4	48.8	39.2	57.3	26.3	46.4	48.3	57.7	57.5	65.0	34.8	46.9	59.2	77.7	49.2	67.0	64.6	82.7	52.3	63.5	58.4	68.0	57.1	67.2
Uganda	60 to 64	47.5	59.5	63.7	70.5	39.5	52.8	41.6	52.8	58.3	65.2	20.8	35.9	76.1	84.5	80.8	84.7	70.8	80.5	72.6	76.3	74.2	77.3	77.4	80.2
Ukraine	60 to 64	61.4	68.1	66.4	75.6	60.9	64.9	64.8	69.7	69.8	76.2	57.2	59.9	60.3	71.8	61.3	74.7	55.5	65.6	50.3	60.6	53.5	67.4	58.9	65.1
United Arab Emirates	60 to 64	59.1	82.3	57.5	85.0	64.1	83.5	62.0	81.7	65.7	82.3	55.1	78.8	43.5	81.2	26.2	78.0	54.3	80.4	47.9	81.0	41.2	78.6	63.9	89.9
United Kingdom	60 to 64	73.4	86.5	76.1	88.8	74.0	86.0	78.6	88.7	81.2	90.2	74.0	85.7	70.1	85.7	61.2	80.2	74.9	84.9	71.4	88.8	72.7	88.2	79.6	94.9

United Republic of Tanzania	60 to 64	50.5	64.1	58.2	67.0	49.7	66.0	49.6	60.0	56.8	63.5	38.4	54.1	80.9	86.6	79.6	81.9	78.7	86.0	72.7	73.3	71.7	73.4	80.2	79.7
United States Virgin Islands	60 to 64	55.2	73.0	65.1	83.3	48.8	67.0	56.1	71.3	67.0	81.4	39.4	58.7	72.8	87.0	70.3	86.4	67.4	80.9	58.9	77.3	64.2	82.1	61.3	81.0
United States of America	60 to 64	82.0	100.0	83.9	100.0	82.2	100.0	81.3	91.5	84.0	93.2	76.4	88.2	74.6	84.3	69.8	82.8	73.1	77.2	83.6	100.0	82.4	100.0	90.2	99.6
Uruguay	60 to 64	73.2	80.3	77.3	84.1	72.9	79.2	77.1	83.8	83.6	89.9	66.6	74.2	68.7	77.6	66.3	73.3	64.9	76.1	72.8	80.3	74.6	81.8	79.4	85.4
Uzbekistan	60 to 64	56.9	64.2	63.6	70.5	55.7	62.8	60.2	67.8	64.3	71.1	53.4	61.8	83.0	61.7	81.8	65.4	80.1	56.8	54.5	61.9	62.2	66.7	58.1	67.8
Vanuatu	60 to 64	45.6	55.1	59.0	65.4	41.5	50.8	57.1	61.4	67.5	69.9	43.1	49.5	70.6	80.9	62.9	72.1	72.6	83.3	63.0	71.6	71.6	77.0	65.4	74.2
Venezuela (Bolivarian Republic of)	60 to 64	60.4	65.8	68.3	75.4	56.4	59.3	66.0	67.4	73.6	77.5	53.7	51.6	82.9	80.1	79.5	76.0	78.9	76.8	67.3	73.8	72.1	79.7	70.7	75.3
Viet Nam	60 to 64	37.6	50.5	52.6	66.4	33.2	46.1	51.1	56.6	61.5	69.0	36.8	41.4	67.6	78.1	70.9	80.3	60.5	72.0	51.2	57.0	65.3	71.8	52.5	60.2
Yemen	60 to 64	23.4	42.3	24.3	43.5	31.4	48.1	33.6	42.3	44.7	46.1	17.3	35.8	42.3	57.3	49.9	56.9	35.9	54.6	11.2	24.1	1.7	23.1	36.2	41.4
Zambia	60 to 64	40.2	48.3	51.1	58.9	36.4	43.5	39.9	44.5	47.0	50.3	28.0	34.1	74.3	78.1	73.2	74.7	72.6	77.5	70.1	68.3	70.5	70.9	75.7	71.8
Zimbabwe	60 to 64	51.2	39.3	58.7	49.1	50.1	30.9	30.4	0.0	25.2	0.0	26.1	0.0	80.9	76.2	77.3	72.4	79.8	73.8	64.4	54.3	70.1	63.1	66.6	43.3
Afghanistan	65 to 69	14.8	31.3	15.6	29.7	13.5	34.2	23.2	32.1	34.8	35.5	11.5	30.6	37.2	52.7	53.8	56.3	29.0	48.7	18.9	22.7	7.0	17.5	27.3	29.6
Albania	65 to 69	38.6	60.7	46.1	64.3	31.4	57.4	41.7	55.0	48.4	56.9	34.9	52.9	70.3	82.7	72.8	83.1	68.9	82.6	63.6	76.0	67.6	76.5	60.8	76.7
Algeria	65 to 69	67.3	73.6	66.6	72.5	67.8	74.4	66.8	70.8	66.0	68.3	67.9	73.1	80.7	84.4	83.7	86.0	78.4	83.2	63.1	68.6	59.4	64.6	67.5	73.2
American Samoa	65 to 69	56.8	60.3	62.2	64.2	51.7	56.3	60.7	60.5	61.7	61.4	59.3	59.6	81.0	83.9	76.5	78.9	85.8	89.7	75.3	77.1	76.3	76.9	75.4	78.3
Andorra	65 to 69	79.5	88.5	79.8	87.6	79.1	89.4	80.7	86.7	79.4	85.0	81.8	88.0	81.2	90.1	76.0	86.5	86.4	93.4	79.7	88.8	78.6	86.7	82.5	91.8
Angola	65 to 69	44.8	54.6	56.0	62.9	31.4	43.1	39.9	44.0	43.5	47.5	36.0	41.2	76.9	81.5	80.9	83.1	74.5	79.8	66.6	67.9	72.2	72.8	58.4	59.3
Antigua and Barbuda	65 to 69	46.1	58.0	58.8	69.9	32.4	44.3	50.0	53.8	56.6	64.7	43.3	42.7	71.7	79.2	77.1	80.4	64.8	77.2	60.3	68.2	66.7	73.5	53.1	61.8
Argentina	65 to 69	66.1	78.6	70.5	82.3	62.0	75.3	71.3	76.9	76.1	81.7	65.8	71.6	64.4	81.6	65.3	82.3	63.4	80.5	73.4	83.8	75.4	84.4	72.8	84.4
Armenia	65 to 69	57.9	75.1	61.3	78.4	54.1	72.0	54.8	70.8	55.4	73.3	55.6	68.0	62.7	78.5	61.5	81.2	64.7	76.3	55.3	69.8	57.2	71.6	53.1	68.8
Australia	65 to 69	77.5	91.9	78.0	93.1	77.2	91.2	76.8	88.9	76.5	89.5	76.9	88.2	73.8	89.1	73.2	91.0	73.8	85.5	78.3	92.5	77.2	91.6	80.7	94.8
Austria	65 to 69	74.2	93.6	76.7	94.4	71.7	93.1	82.8	93.3	83.0	93.2	82.0	93.1	90.5	100.0	90.3	99.9	90.3	97.6	67.1	91.7	67.3	90.6	68.2	93.8
Azerbaijan	65 to 69	51.4	58.8	57.5	62.2	43.3	55.1	53.7	57.6	55.3	55.8	52.9	59.3	84.5	87.9	86.7	89.2	82.2	86.4	64.7	70.8	68.3	72.6	58.6	69.1
Bahamas	65 to 69	52.5	68.0	62.1	72.9	40.9	62.3	53.7	63.9	60.3	66.9	46.8	60.8	77.4	83.8	78.8	84.1	74.0	82.2	63.9	75.3	67.7	75.7	59.2	75.7
Bahrain	65 to 69	42.8	68.4	43.3	66.3	42.3	70.6	51.6	70.8	54.6	70.9	48.9	70.7	57.6	83.0	53.2	81.5	62.7	83.7	37.2	60.5	35.3	56.0	39.5	64.6
Bangladesh	65 to 69	23.6	44.4	24.6	42.7	22.2	45.4	51.8	61.9	55.7	62.9	47.6	60.8	39.2	60.5	44.4	55.5	40.6	65.9	36.4	50.6	31.5	46.9	40.2	54.4
Barbados	65 to 69	55.0	66.2	65.5	70.2	43.0	61.8	56.0	63.1	63.0	64.9	48.8	61.3	74.8	76.7	78.4	75.0	70.1	77.7	57.5	69.3	63.9	70.3	49.9	69.0
Belarus	65 to 69	62.0	68.3	63.9	74.6	60.5	61.5	65.6	67.4	65.9	70.9	66.9	63.5	69.5	70.5	72.8	78.3	62.5	60.9	50.8	60.7	50.2	66.4	52.4	55.5
Belgium	65 to 69	69.7	85.1	73.5	88.2	66.1	82.6	72.9	81.1	75.6	84.7	69.9	77.5	81.7	83.1	80.3	84.7	83.2	80.8	69.4	87.1	71.3	87.8	69.3	88.2
Belize	65 to 69	54.6	64.2	60.9	71.6	48.3	57.5	55.3	60.7	58.3	63.6	52.3	57.6	79.0	86.4	81.6	87.5	75.9	84.5	70.0	73.0	72.6	77.8	68.1	69.9
Benin	65 to 69	43.1	50.5	45.4	54.4	40.7	46.2	49.9	56.6	38.2	48.2	59.3	64.1	76.8	82.6	72.6	78.9	79.8	84.6	54.9	56.4	69.4	70.8	36.3	36.6
Bermuda	65 to 69	66.5	84.3	74.5	94.3	56.9	74.7	68.2	79.9	73.4	88.7	62.1	72.6	81.6	92.3	85.8	98.7	74.6	83.3	62.8	82.3	70.0	89.7	54.7	77.0
Bhutan	65 to 69	43.9	57.9	39.9	57.8	47.3	58.0	60.8	68.6	60.9	68.4	61.0	68.7	54.4	66.0	51.0	63.1	59.2	69.8	52.8	61.0	44.8	59.5	60.5	63.4
Bolivia (Plurinational State of)	65 to 69	35.3	55.0	38.6	58.2	31.4	51.2	37.5	50.3	39.2	52.6	37.4	48.7	50.9	59.6	43.5	51.4	59.0	69.2	52.3	66.9	58.0	71.7	45.3	61.8
Bosnia and Herzegovina	65 to 69	57.8	71.3	57.7	74.3	58.9	68.5	41.8	59.2	41.6	63.0	45.5	55.5	46.5	70.0	42.2	71.1	51.5	69.7	49.5	64.5	45.7	65.6	55.5	64.7
Botswana	65 to 69	48.9	64.7	57.2	71.3	39.0	55.8	43.3	58.5	40.8	58.1	45.0	59.1	84.1	90.4	86.4	91.8	82.2	88.7	63.4	71.4	70.6	76.8	52.9	62.4
Brazil	65 to 69	52.6	67.5	58.9	72.7	46.7	62.6	55.8	62.9	59.2	66.7	52.5	58.8	72.2	66.4	68.5	61.2	75.8	72.3	51.2	70.2	60.8	76.2	43.5	66.4
Brunei Darussalam	65 to 69	70.4	82.4	69.9	80.1	70.3	85.1	79.0	87.0	79.6	85.2	78.4	88.7	70.5	83.8	65.7	81.3	76.6	87.8	71.8	83.2	68.9	78.6	75.5	87.9
Bulgaria	65 to 69	42.0	58.0	45.0	65.5	39.3	51.1	42.8	56.0	44.1	59.5	41.1	52.2	65.0	56.4	64.8	58.9	66.2	54.8	45.3	49.7	47.4	57.3	44.6	43.7
Burkina Faso	65 to 69	51.1	53.5	61.6	63.6	40.6	43.5	51.8	56.6	50.8	54.9	51.9	57.1	84.6	86.5	90.4	90.2	80.0	83.0	68.1	65.9	81.3	80.3	52.3	49.0
Burundi	65 to 69	30.1	45.0	46.8	57.9	7.4	31.8	22.9	32.0	36.6	42.7	8.3	21.6	69.4	77.6	77.7	84.1	62.9	74.8	60.8	67.9	63.9	71.3	55.3	64.4
Cabo Verde	65 to 69	68.3	68.3	66.7	78.4	70.6	56.5	71.0	72.2	65.1	72.4	78.7	72.2	91.9	95.0	91.1	96.3	91.2	91.4	72.6	64.4	76.6	82.7	66.2	40.6
Cambodia	65 to 69	26.4	43.5	27.1	48.3	26.3	37.9	38.1	48.6	35.5	49.9	42.5	48.8	60.2	72.7	59.2	72.6	61.3	72.6	56.5	64.0	59.4	67.2	53.5	59.8
Cameroon	65 to 69	47.9	50.3	52.9	55.5	42.6	44.9	52.5	55.6	48.7	50.2	56.0	59.9	80.1	82.7	79.0	81.3	80.6	83.0	65.1	59.0	74.2	72.8	52.5	42.6
Canada	65 to 69	81.5	95.2	84.3	95.0	78.9	95.6	84.4	97.6	85.8	98.2	82.6	96.9	81.2	90.0	82.0	90.6	78.7	88.2	81.9	94.1	82.2	92.4	83.3	96.5
Central African Republic	65 to 69	27.6	33.3	43.1	47.4	9.3	13.3	28.1	25.7	33.8	32.8	21.8	18.5	68.1	71.8	73.9	75.8	64.7	68.5	55.8	59.3	63.2	66.2	44.9	46.3
Chad	65 to 69	48.1	43.8	51.5	49.9	44.7	38.5	52.8	51.8	44.0	42.3	59.2	56.4	79.4	77.6	77.8	73.8	80.3	79.9	59.9	51.0	71.8	69.5	45.8	33.2
Chile	65 to 69	65.5	83.5	69.7	86.2	61.5	80.9	72.6	85.1	76.9	87.6	67.0	81.8	75.1	81.8	74.4	82.1	75.9	80.8	67.2	85.1	69.6	86.6	66.2	85.0

China	65 to 69	33.7	63.4	41.2	70.0	26.3	57.2	49.2	62.8	55.4	68.7	43.0	56.9	0.0	67.6	0.0	71.7	0.0	65.0	49.4	67.5	53.3	70.3	46.7	66.2
Colombia	65 to 69	64.4	79.0	68.5	83.4	59.7	73.3	64.2	76.9	67.2	80.5	61.0	72.0	79.7	77.1	78.0	75.8	80.9	78.0	66.1	80.3	69.5	84.5	62.7	76.2
Comoros	65 to 69	46.8	59.4	56.9	66.4	34.3	48.9	37.8	45.4	41.4	49.1	34.1	42.5	79.4	85.1	82.7	87.1	77.5	83.0	68.5	73.7	72.3	76.3	62.6	67.8
Congo	65 to 69	46.1	55.2	53.6	60.2	35.2	48.4	38.5	43.2	37.8	40.6	40.4	47.2	77.2	81.9	77.6	80.5	77.0	82.9	65.5	67.5	70.2	71.2	56.0	60.8
Cook Islands	65 to 69	56.3	75.2	64.9	81.2	49.5	69.6	60.1	72.4	64.5	77.2	56.1	67.9	80.7	95.9	79.3	93.7	82.3	97.1	73.4	83.8	76.2	84.5	72.8	84.4
Costa Rica	65 to 69	74.0	84.3	78.6	88.6	68.6	78.6	72.7	80.3	75.5	83.8	69.2	76.3	87.4	87.1	87.2	86.0	86.3	86.8	76.0	84.9	78.8	87.4	73.6	82.4
Coted'Ivoire	65 to 69	53.9	53.8	60.4	59.8	48.8	48.5	59.2	59.1	53.4	53.0	61.3	63.1	82.3	84.9	81.5	82.9	82.6	85.5	58.1	55.2	73.5	72.7	46.2	38.7
Croatia	65 to 69	54.9	74.3	56.2	77.7	53.7	71.6	46.3	61.2	49.3	69.1	44.5	53.2	49.4	61.3	43.5	62.1	57.0	60.9	57.0	76.1	57.0	76.9	58.2	77.6
Cuba	65 to 69	62.7	66.5	67.2	73.5	57.9	59.4	63.7	63.9	66.5	68.2	60.7	59.6	85.6	84.6	84.9	85.4	85.0	82.0	63.3	62.5	65.0	69.1	62.1	56.8
Cyprus	65 to 69	68.1	83.3	71.3	85.9	65.1	81.2	75.7	85.1	78.4	85.8	72.7	84.2	74.3	89.8	73.8	88.8	75.0	89.9	70.3	83.9	69.9	85.0	72.3	84.3
Czechia	65 to 69	54.0	81.0	58.3	84.5	49.9	78.2	50.9	77.0	55.1	80.5	46.9	73.7	53.4	79.3	49.1	82.3	58.5	76.3	44.2	79.8	47.6	80.9	42.1	80.7
Democratic People's Republic of Korea	65 to 69	45.2	47.4	51.5	54.9	37.2	39.4	50.0	46.1	56.0	52.8	43.3	39.1	49.1	59.1	51.1	62.9	42.8	54.7	60.0	60.2	61.1	63.4	59.1	57.7
Democratic Republic of the Congo	65 to 69	46.8	47.4	53.6	56.1	38.2	35.9	44.5	40.2	44.4	43.4	44.8	37.4	79.4	79.6	80.5	81.4	78.8	78.4	64.8	66.3	67.5	70.2	60.2	59.2
Denmark	65 to 69	75.0	87.0	76.6	90.2	73.6	84.5	74.0	81.7	74.7	84.5	73.1	79.0	77.9	87.4	75.5	87.2	80.5	86.5	75.9	88.0	75.8	89.8	77.7	88.5
Djibouti	65 to 69	50.5	58.5	62.5	70.4	37.6	47.9	41.3	46.0	47.6	51.7	35.1	40.4	79.5	83.5	85.0	88.1	76.7	81.5	72.4	71.1	75.9	78.3	68.0	64.6
Dominica	65 to 69	38.6	50.3	51.4	61.5	21.1	39.6	37.6	47.7	46.1	55.1	28.8	40.6	68.0	74.2	73.5	79.2	60.1	70.1	50.5	56.1	58.9	63.2	37.5	49.4
Dominican Republic	65 to 69	57.6	60.8	64.6	68.9	50.4	52.8	57.8	60.5	62.1	63.9	53.3	56.9	79.3	82.2	79.9	82.2	77.4	80.8	68.2	68.1	72.0	73.8	65.2	63.7
Ecuador	65 to 69	64.5	78.8	70.5	82.4	57.9	74.5	58.6	71.3	62.1	74.5	55.4	67.7	84.3	75.3	84.0	73.3	83.6	76.6	68.0	83.5	74.0	86.5	62.3	81.1
Egypt	65 to 69	19.5	51.1	27.4	50.8	11.6	51.4	15.8	51.8	31.2	53.4	0.0	49.9	48.9	71.7	64.6	75.0	34.5	69.7	0.0	32.3	0.0	27.6	0.0	37.0
El Salvador	65 to 69	60.9	74.9	66.2	79.6	54.5	67.6	62.4	69.2	65.8	73.4	58.7	63.3	79.6	84.7	78.6	83.3	80.0	85.4	68.5	78.5	73.3	82.5	63.4	73.0
Equatorial Guinea	65 to 69	37.5	59.6	50.2	65.3	20.5	51.2	32.7	54.7	37.4	55.2	28.2	55.5	73.4	88.5	78.5	88.1	69.6	88.0	62.9	66.9	69.5	71.2	51.4	58.0
Eritrea	65 to 69	30.9	48.4	45.5	60.2	9.2	27.6	22.7	31.4	31.7	42.2	13.2	19.0	71.7	80.4	80.1	85.6	63.4	74.1	67.6	71.7	70.1	75.2	62.1	62.1
Estonia	65 to 69	52.7	79.9	55.9	87.0	49.5	72.8	59.1	76.4	60.5	80.0	59.0	72.3	77.6	76.2	83.8	84.2	65.0	66.5	35.2	76.8	36.3	83.1	34.4	72.3
Eswatini	65 to 69	40.0	45.4	52.0	54.5	22.5	32.1	31.0	36.4	33.6	35.6	28.4	38.0	81.2	83.3	84.9	84.9	77.7	81.4	62.4	61.0	68.8	67.1	50.1	48.9
Ethiopia	65 to 69	30.4	53.5	51.3	64.9	9.7	40.6	36.2	48.9	48.8	56.5	24.5	41.4	76.0	84.3	86.5	88.8	71.4	81.8	71.9	75.1	75.1	77.1	68.5	72.5
Fiji	65 to 69	54.5	59.9	59.2	63.8	49.7	55.7	52.8	51.5	55.5	54.4	50.4	49.4	77.5	81.5	75.7	79.5	80.9	86.9	75.4	77.1	76.4	77.1	75.5	78.0
Finland	65 to 69	76.9	90.3	77.5	93.4	76.4	88.0	81.8	90.1	81.2	92.0	82.2	88.8	84.2	93.4	80.3	93.2	89.1	91.9	72.3	88.6	71.3	90.1	74.6	89.3
France	65 to 69	75.4	89.5	80.7	91.1	71.6	88.4	73.1	87.5	77.5	89.3	68.5	86.1	85.5	90.8	84.9	92.5	85.4	88.0	76.8	89.4	81.1	88.9	76.7	91.3
Gabon	65 to 69	54.7	61.0	64.6	69.8	41.9	51.9	49.0	53.0	53.2	55.0	44.9	50.4	82.8	86.5	86.5	89.0	79.3	84.4	71.9	71.2	75.5	76.0	66.0	65.9
Gambia	65 to 69	50.7	48.3	56.8	54.5	45.5	41.6	54.0	51.3	44.4	44.3	58.7	57.7	82.3	81.0	80.6	77.4	83.0	82.9	56.6	52.2	73.4	69.6	40.8	30.2
Georgia	65 to 69	43.3	59.4	52.7	68.4	29.3	49.2	51.1	62.1	54.5	65.5	48.1	58.6	70.5	66.9	72.7	67.6	68.6	67.5	61.6	60.3	67.1	67.3	50.0	51.6
Germany	65 to 69	76.7	87.3	79.2	88.9	74.0	86.0	75.3	87.7	77.6	89.2	72.0	86.0	83.6	90.9	82.5	90.4	83.9	90.0	75.2	86.4	75.8	85.8	74.9	88.3
Ghana	65 to 69	52.6	57.8	56.3	63.8	48.2	50.3	49.3	53.8	40.9	47.9	59.4	60.6	82.6	85.1	79.6	81.5	84.0	86.7	61.6	61.0	74.0	74.9	41.8	40.1
Greece	65 to 69	67.3	81.7	67.8	84.8	66.9	79.4	70.6	78.9	71.3	81.1	70.1	76.6	82.7	89.7	80.9	91.1	84.5	87.0	71.7	86.6	70.9	87.0	73.8	88.0
Greenland	65 to 69	68.2	79.1	69.4	82.3	67.3	77.2	64.0	73.6	66.3	76.4	62.0	71.4	25.8	65.9	17.2	65.9	35.3	66.4	74.7	86.1	75.7	87.4	74.5	87.3
Grenada	65 to 69	32.8	58.4	38.4	63.4	26.1	53.4	39.8	53.7	42.5	55.4	37.4	51.6	62.2	74.7	61.8	73.9	61.7	75.1	32.0	64.5	35.4	66.4	27.3	63.4
Guam	65 to 69	65.7	80.2	70.1	81.7	61.6	78.7	66.0	74.8	67.3	73.9	64.5	75.9	81.8	95.0	77.6	89.4	86.1	100.0	74.0	86.1	76.5	86.0	73.2	87.4
Guatemala	65 to 69	61.0	70.9	64.5	75.5	57.1	65.1	63.6	67.1	64.8	70.1	63.0	63.7	94.2	89.5	94.8	90.4	92.3	87.2	68.9	79.8	71.4	82.7	67.1	76.4
Guinea	65 to 69	48.1	49.4	46.7	51.2	50.1	47.7	52.1	56.0	40.0	44.3	63.1	63.5	81.7	82.9	76.8	76.9	84.7	85.7	61.5	54.7	69.6	69.6	50.5	39.5
Guinea-Bissau	65 to 69	28.9	36.5	39.8	43.6	18.5	28.4	39.5	41.6	31.6	33.2	43.7	49.6	69.1	73.1	70.1	69.0	69.1	76.2	41.4	45.0	67.3	66.8	12.4	15.5
Guyana	65 to 69	14.4	39.5	20.6	50.2	8.2	27.9	36.7	45.7	39.6	51.2	33.9	39.8	63.6	69.0	65.9	72.5	61.3	64.3	25.6	48.4	36.5	59.2	14.0	36.3
Haiti	65 to 69	0.0	17.7	0.0	18.7	0.0	16.8	23.5	27.7	26.2	29.4	21.5	27.4	34.5	44.4	28.9	37.0	40.4	52.3	21.6	33.7	16.0	31.2	27.9	37.0
Honduras	65 to 69	45.7	32.6	51.8	38.1	38.8	26.0	43.2	29.6	48.2	34.8	39.0	25.7	66.3	45.4	63.9	35.2	68.4	56.2	53.7	44.3	62.9	55.9	43.9	31.5
Hungary	65 to 69	60.0	74.8	63.9	79.9	56.2	69.9	54.5	67.6	56.7	72.1	52.5	63.0	71.1	77.4	72.4	79.0	68.3	75.0	53.5	70.1	55.9	74.0	52.1	67.8
Iceland	65 to 69	77.5	91.1	79.3	93.5	75.8	89.5	80.4	90.1	80.9	92.1	79.6	88.6	84.3	94.5	81.6	94.2	87.1	93.5	77.3	91.0	77.1	91.0	79.3	92.9
India	65 to 69	38.8	47.0	39.6	46.6	37.9	47.3	58.9	64.3	61.5	64.8	56.1	63.8	48.5	57.7	51.7	54.1	49.3	62.4	48.0	51.6	44.9	49.9	51.4	53.9
Indonesia	65 to 69	45.9	48.8	48.1	54.7	43.9	42.8	51.0	51.4	52.0	52.3	51.7	50.8	67.0	70.6	64.9	68.8	69.8	73.0	66.5	62.4	69.3	69.6	63.8	55.6
Iran (Islamic Republic of)	65 to 69	55.6	74.8	59.3	75.4	52.4	74.3	61.4	73.6	64.8	73.9	58.1	73.3	70.0	86.4	75.0	88.7	67.2	84.1	37.2	64.0	38.5	62.2	37.0	66.4

Iraq	65 to 69	36.2	50.4	43.0	57.9	29.5	43.6	45.0	55.4	50.3	59.9	39.6	50.8	70.9	77.6	75.9	80.6	65.1	74.7	21.7	37.9	27.2	45.2	16.4	32.0
Ireland	65 to 69	71.7	91.6	74.3	92.7	69.4	90.7	77.3	90.2	77.1	90.7	77.4	89.5	75.6	90.5	73.5	90.2	77.9	89.8	70.0	91.6	72.3	91.4	70.1	92.9
Israel	65 to 69	75.3	93.2	74.7	95.8	76.0	91.5	69.5	81.2	71.0	84.4	67.9	78.3	90.5	95.6	90.4	98.2	90.2	91.5	79.8	95.3	76.5	95.8	83.9	96.3
Italy	65 to 69	66.4	84.5	69.7	86.9	63.3	82.8	68.2	81.6	71.3	83.6	64.8	79.8	79.2	86.2	76.9	87.4	81.9	84.6	66.0	87.2	67.5	87.2	66.5	88.9
Jamaica	65 to 69	41.1	52.2	47.3	58.4	34.4	45.9	50.4	56.2	51.6	58.6	48.8	53.5	74.0	79.2	70.8	78.5	77.2	79.2	51.1	62.4	54.6	65.4	47.3	60.1
Japan	65 to 69	80.4	93.3	81.8	94.0	79.2	93.5	88.9	100.0	89.4	100.0	87.6	100.0	81.7	93.5	80.6	93.4	83.6	93.8	78.0	90.8	77.6	90.7	80.0	92.5
Jordan	65 to 69	58.2	77.5	55.3	76.7	60.8	78.4	58.3	71.2	56.4	71.2	61.2	71.1	90.4	95.1	91.5	96.5	89.5	93.3	47.6	73.7	40.4	69.6	55.2	78.3
Kazakhstan	65 to 69	60.2	63.0	64.9	70.4	53.2	53.8	56.7	56.1	58.2	59.5	56.7	52.7	58.5	57.7	58.4	58.0	58.0	58.5	57.1	64.1	61.0	70.6	49.5	55.8
Kenya	65 to 69	61.2	60.3	68.3	69.6	52.2	49.0	55.0	50.9	59.4	55.8	50.4	46.1	85.2	85.6	88.3	88.7	83.2	83.3	78.7	76.4	78.7	79.3	78.5	72.5
Kiribati	65 to 69	50.3	52.8	59.2	61.2	41.7	43.7	61.4	59.7	62.4	61.4	59.8	57.8	79.2	83.7	83.0	84.7	74.1	82.5	72.5	71.9	77.6	77.8	67.9	65.2
Kuwait	65 to 69	77.0	85.0	71.6	90.1	79.2	82.7	76.7	85.3	72.7	86.6	78.8	84.6	86.2	96.3	82.9	100.0	88.0	93.1	71.0	81.3	58.3	84.1	78.6	82.5
Kyrgyzstan	65 to 69	50.4	56.0	56.7	64.8	41.6	46.2	51.4	58.6	51.6	60.2	52.7	56.7	66.9	56.4	72.1	63.7	59.4	50.9	51.7	49.6	57.2	59.9	41.9	37.7
Lao People's Democratic Republic	65 to 69	19.9	43.7	21.4	47.7	18.3	40.0	34.1	48.9	31.8	47.6	35.4	49.5	48.3	71.0	45.7	70.1	52.1	72.4	43.3	58.7	47.7	65.6	40.3	53.7
Latvia	65 to 69	53.7	69.5	57.7	74.3	49.1	64.9	53.1	67.9	55.8	71.6	51.6	63.9	70.5	72.9	73.8	77.4	62.7	67.2	44.9	62.0	46.7	65.6	42.7	59.7
Lebanon	65 to 69	52.1	81.0	57.5	83.1	47.6	79.1	46.7	75.4	59.1	78.1	33.3	71.4	54.9	86.2	62.5	89.6	48.2	81.3	53.8	81.0	52.7	80.1	55.8	83.0
Lesotho	65 to 69	37.1	29.3	51.9	44.9	11.5	0.3	31.5	22.7	38.7	28.4	23.9	17.4	79.4	76.6	85.4	80.8	72.4	71.0	62.9	52.4	69.6	62.9	46.4	25.0
Liberia	65 to 69	49.0	46.5	48.5	47.2	49.3	45.9	53.8	49.3	40.4	36.3	63.2	60.4	82.8	82.3	78.1	76.4	85.2	85.4	58.3	53.2	69.2	66.7	46.9	36.5
Libya	65 to 69	61.9	64.0	59.6	63.6	64.2	64.9	61.8	60.9	60.1	58.4	64.6	66.2	80.1	80.8	78.9	77.5	81.6	83.9	54.9	53.3	48.8	51.5	61.8	56.2
Lithuania	65 to 69	61.7	71.4	66.6	77.6	55.7	65.1	65.2	74.0	67.0	75.6	64.0	71.9	73.8	75.5	78.0	81.8	66.7	66.1	54.1	67.2	57.2	72.4	50.2	63.1
Luxembourg	65 to 69	65.2	84.1	69.1	86.7	61.2	82.4	66.3	81.1	70.1	84.6	62.2	78.6	77.9	88.4	75.2	88.6	81.1	87.7	63.2	86.1	66.4	86.3	61.1	87.8
Madagascar	65 to 69	36.6	45.4	52.0	55.9	19.9	32.5	30.6	32.4	38.7	38.1	22.5	26.8	72.9	78.0	79.8	80.3	69.6	76.8	67.8	69.7	73.4	73.9	60.3	63.2
Malawi	65 to 69	45.0	45.8	52.6	55.8	35.8	34.1	32.0	27.6	35.2	33.9	29.1	21.6	76.3	77.0	79.6	80.1	74.4	75.2	68.3	63.4	71.0	69.9	64.3	54.5
Malaysia	65 to 69	53.4	68.9	56.2	73.0	50.8	64.9	57.4	67.3	57.1	68.3	57.8	66.2	73.9	83.7	73.5	83.9	74.7	83.2	67.4	76.3	67.8	77.7	68.0	75.8
Maldives	65 to 69	43.2	72.1	39.9	74.5	44.4	70.1	47.2	70.0	38.6	66.0	50.1	71.7	70.0	89.8	67.4	89.5	73.3	89.5	63.2	78.6	62.1	80.0	65.4	79.0
Mali	65 to 69	45.3	49.8	41.7	46.6	49.3	52.7	50.0	54.8	35.1	38.6	61.9	65.6	80.2	83.6	73.3	75.6	84.3	87.5	63.2	59.9	66.6	66.0	58.6	52.8
Malta	65 to 69	65.6	88.2	68.1	90.5	63.3	86.3	72.2	88.0	72.8	88.6	71.7	87.3	89.3	96.6	88.5	97.5	89.7	94.4	62.3	88.5	62.9	88.4	63.2	90.1
Marshall Islands	65 to 69	31.0	44.3	49.4	52.6	11.4	36.0	43.2	50.7	53.4	54.1	33.4	47.3	64.9	75.0	67.4	73.2	61.9	77.4	66.0	71.1	72.5	72.8	58.6	69.9
Mauritania	65 to 69	50.6	58.5	50.3	59.2	51.2	57.8	51.3	58.0	36.0	46.7	65.0	69.2	81.7	87.1	76.0	81.5	85.2	89.8	60.8	62.8	70.9	72.1	46.3	49.4
Mauritius	65 to 69	51.7	69.1	59.4	77.2	44.5	61.4	55.1	67.8	57.2	70.7	52.9	64.9	67.4	80.3	73.6	84.9	58.9	73.3	52.8	72.3	61.4	78.3	46.0	68.2
Mexico	65 to 69	71.3	79.7	75.8	84.6	65.5	73.7	69.0	75.4	72.2	78.9	65.2	70.8	87.9	81.0	87.1	80.8	87.8	80.3	70.3	80.5	74.1	84.9	65.9	76.6
Micronesia (Federated States of)	65 to 69	36.5	47.0	44.1	54.9	29.4	38.9	49.1	53.9	51.7	56.9	46.2	50.8	66.1	76.0	64.8	74.7	67.8	77.8	66.6	70.4	68.6	73.2	65.5	68.0
Monaco	65 to 69	73.2	85.7	75.3	86.4	71.3	85.3	72.1	83.7	74.4	84.0	69.8	83.3	76.7	91.5	73.2	89.1	80.6	92.8	71.7	84.4	71.3	82.9	73.5	86.9
Mongolia	65 to 69	21.0	50.5	28.1	61.5	13.3	38.0	37.8	57.4	40.1	62.7	35.9	51.6	28.5	38.5	31.2	38.3	28.8	40.6	73.3	79.3	75.4	83.4	70.8	74.4
Montenegro	65 to 69	37.8	44.1	40.5	49.6	35.3	38.9	46.8	46.4	50.9	52.5	43.0	39.9	74.3	76.8	72.3	74.4	76.7	79.4	70.4	68.6	71.5	71.5	70.4	67.2
Morocco	65 to 69	48.1	57.7	40.1	53.8	54.7	60.3	50.6	56.9	46.2	52.2	56.1	60.9	67.3	71.9	65.9	70.4	69.6	73.9	38.5	46.8	24.4	38.0	51.5	55.0
Mozambique	65 to 69	33.8	35.3	48.4	54.3	18.7	16.0	30.9	29.2	40.9	44.1	20.6	14.2	69.1	68.8	77.5	77.7	64.7	63.8	58.9	53.8	66.8	68.0	49.7	37.9
Myanmar	65 to 69	20.9	41.3	26.2	50.6	15.8	31.9	35.1	45.3	32.9	48.2	36.1	42.3	54.2	70.0	56.8	72.0	52.4	68.1	45.0	59.0	54.3	67.4	36.7	50.7
Namibia	65 to 69	46.1	50.3	54.5	59.9	35.1	37.2	40.2	42.1	39.1	45.0	41.8	39.7	84.4	86.3	86.7	88.6	82.4	83.7	61.4	61.5	68.7	69.2	49.0	48.6
Nauru	65 to 69	39.6	43.9	47.6	53.2	32.8	30.1	43.1	34.2	48.5	46.3	38.5	21.4	66.3	71.3	61.1	66.5	71.9	78.5	63.1	66.4	69.7	70.1	58.4	59.2
Nepal	65 to 69	31.7	47.4	24.1	50.2	37.9	44.9	55.2	63.4	55.3	66.0	55.8	60.7	48.7	62.3	46.8	64.6	52.5	61.3	38.5	52.4	25.0	54.1	50.1	52.1
Netherlands	65 to 69	79.6	89.6	82.8	90.9	76.8	88.6	75.7	86.0	78.5	86.8	73.0	84.9	80.2	89.5	80.3	90.0	79.1	87.6	80.8	89.3	81.7	89.2	81.9	90.8
New Zealand	65 to 69	73.7	89.7	77.0	91.1	70.7	88.7	74.0	84.1	76.4	85.7	71.2	82.1	65.5	79.2	64.5	80.9	66.2	76.2	74.3	91.3	76.4	91.3	74.1	92.7
Nicaragua	65 to 69	65.9	73.5	71.6	78.2	59.7	68.1	66.4	71.4	69.3	73.7	63.0	68.6	84.1	86.1	83.8	84.6	83.8	87.2	71.5	77.3	75.7	80.6	68.5	75.6
Niger	65 to 69	49.0	48.2	54.2	51.8	44.0	44.3	52.5	50.8	46.9	45.0	55.7	56.4	79.2	81.5	79.0	80.6	79.4	81.8	67.0	65.3	75.1	74.3	58.1	52.9
Nigeria	65 to 69	45.8	62.9	52.1	68.1	39.5	56.5	53.1	65.4	47.8	62.5	57.0	69.3	79.1	89.5	80.6	88.8	78.2	88.7	56.8	65.2	71.2	76.4	39.7	48.0
Niue	65 to 69	54.0	60.5	61.6	68.3	46.4	52.4	57.2	59.7	61.4	64.8	53.2	54.8	77.4	84.7	75.9	82.6	79.3	87.2	72.9	75.5	75.3	78.3	71.4	73.5
North Macedonia	65 to 69	46.3	54.5	46.9	56.4	45.7	52.8	40.0	47.9	38.7	46.6	41.3	48.3	45.7	54.3	38.7	46.0	54.1	64.1	43.6	50.9	43.5	51.5	44.8	51.6
Northern Mariana Islands	65 to 69	54.6	62.4	54.3	68.3	54.0	57.2	60.9	64.0	56.7	65.4	62.0	62.2	76.6	81.5	64.3	76.5	86.7	86.6	72.0	76.9	71.5	79.3	73.9	76.4

Norway	65 to 69	78.3	89.3	80.1	91.8	76.7	87.8	77.4	88.2	80.1	88.2	74.2	88.1	79.0	94.6	74.7	96.4	83.4	91.1	78.5	89.5	78.1	89.5	80.7	91.7
Oman	65 to 69	47.1	68.9	51.0	69.5	43.9	68.7	49.9	69.4	56.6	70.6	42.5	68.0	73.3	85.0	78.0	86.9	70.7	84.0	37.9	61.3	40.1	59.4	37.3	64.0
Pakistan	65 to 69	53.7	54.7	51.4	54.0	55.1	55.4	66.3	65.2	65.7	64.6	66.6	66.1	61.3	61.9	60.1	58.0	64.3	66.7	61.2	58.9	57.0	58.6	65.5	60.1
Palau	65 to 69	51.7	63.0	65.9	70.2	37.9	55.8	57.3	63.0	66.1	66.9	49.1	59.1	80.4	86.8	79.3	82.5	82.0	91.8	68.0	74.3	74.7	77.0	61.9	72.7
Palestine	65 to 69	40.0	59.3	41.0	63.5	39.4	55.1	50.2	60.1	52.9	64.0	47.8	55.7	84.7	91.9	86.1	94.3	83.0	89.0	21.9	48.5	19.1	50.4	25.7	47.4
Panama	65 to 69	61.7	73.3	69.6	80.3	54.0	66.2	65.5	71.8	70.7	77.3	60.2	66.5	80.1	80.5	78.9	80.6	81.1	79.8	66.4	78.5	72.0	82.5	62.4	75.6
Papua New Guinea	65 to 69	15.0	27.5	3.6	16.9	22.0	32.8	32.6	40.1	18.1	25.1	39.5	45.8	57.9	67.3	46.8	56.9	68.2	75.8	62.0	65.6	52.4	57.6	71.5	73.1
Paraguay	65 to 69	53.6	61.1	57.3	66.2	49.9	56.7	54.3	54.9	56.6	58.8	52.3	51.1	70.6	69.3	65.6	61.1	75.5	78.1	61.2	67.1	67.5	74.3	56.5	63.0
Peru	65 to 69	61.8	79.1	68.0	81.4	54.9	76.6	59.9	69.8	64.1	71.9	55.7	67.8	75.4	84.2	74.4	79.3	76.0	88.9	71.8	82.5	76.7	84.8	66.9	80.6
Philippines	65 to 69	52.1	56.6	60.0	66.3	43.9	46.3	52.1	58.2	55.8	62.9	48.8	53.4	78.6	79.0	80.7	80.1	76.3	77.4	68.9	69.8	75.6	76.5	62.8	63.2
Poland	65 to 69	48.1	70.4	53.3	77.5	42.5	64.3	51.7	65.5	55.6	72.0	48.4	59.1	70.9	73.7	72.6	75.7	68.5	72.0	34.9	66.4	40.2	72.4	29.5	63.1
Portugal	65 to 69	59.0	78.1	63.7	81.4	54.8	75.6	62.6	76.1	66.2	79.7	59.1	72.7	72.5	78.4	71.4	80.0	73.8	76.8	59.6	81.7	63.5	82.7	57.8	82.3
Puerto Rico	65 to 69	74.8	89.4	81.2	96.7	67.2	79.6	73.8	80.5	77.6	86.3	69.6	74.6	87.1	93.0	87.3	94.7	85.1	88.6	77.0	91.6	80.7	97.0	73.5	85.7
Qatar	65 to 69	70.2	83.7	67.9	80.0	70.9	87.0	66.6	79.7	64.7	75.8	66.8	82.9	59.4	86.1	73.8	89.7	52.7	83.2	73.2	81.9	68.7	77.0	76.9	85.8
Republic of Korea	65 to 69	68.5	91.8	70.1	91.9	67.2	92.3	79.1	97.3	81.4	96.6	75.5	97.5	66.7	91.4	65.2	91.2	67.9	92.0	70.1	89.6	69.7	88.7	71.6	91.5
Republic of Moldova	65 to 69	46.2	57.8	48.4	62.3	44.3	53.3	47.0	53.9	47.5	55.7	48.1	52.1	72.6	69.5	77.9	74.6	66.2	64.6	61.1	56.5	61.5	59.0	61.5	55.0
Romania	65 to 69	50.5	63.4	52.4	68.9	48.7	58.3	40.1	48.1	39.0	53.0	41.4	43.0	58.3	57.0	55.7	59.0	61.9	56.3	53.1	64.5	54.3	68.6	53.0	62.2
Russian Federation	65 to 69	48.4	64.4	55.0	71.8	37.6	55.5	54.3	63.3	57.1	67.0	53.2	59.5	67.4	64.9	70.7	69.8	60.4	59.4	31.3	55.7	38.4	63.2	16.6	47.0
Rwanda	65 to 69	24.5	50.5	38.9	61.6	5.6	34.4	22.7	40.6	33.5	51.3	11.3	27.9	71.4	83.0	79.2	87.7	64.8	79.0	61.0	71.9	64.0	74.7	55.9	66.3
Saint Kitts and Nevis	65 to 69	43.7	53.1	47.3	65.1	39.5	40.8	50.6	53.5	52.6	61.1	49.2	46.1	69.9	77.3	67.8	77.7	71.7	76.1	49.8	56.5	51.1	65.6	48.1	47.0
Saint Lucia	65 to 69	47.0	62.5	54.2	68.0	37.6	56.9	52.2	61.1	55.6	63.5	48.7	58.5	71.8	81.7	69.3	79.7	74.2	83.0	51.1	66.9	53.5	66.4	47.3	68.1
Saint Vincent and the Grenadines	65 to 69	49.9	62.3	51.3	72.2	48.2	52.9	43.8	60.2	41.6	66.1	47.9	54.6	79.7	86.6	76.9	89.2	82.6	83.0	59.5	68.8	56.6	73.7	64.4	64.5
Samoa	65 to 69	48.2	56.0	59.0	61.5	38.1	50.0	54.4	56.4	61.0	59.5	48.2	53.6	74.7	82.2	74.9	78.7	74.9	86.6	73.2	76.4	76.1	78.3	71.6	74.8
San Marino	65 to 69	78.8	89.4	79.6	90.1	78.2	88.9	78.1	87.6	78.1	86.7	78.2	88.3	90.5	97.9	90.3	98.2	90.2	96.4	78.1	88.6	78.3	88.1	79.7	90.6
Sao Tome and Principe	65 to 69	63.3	65.3	65.3	67.5	61.2	62.7	62.1	65.1	57.4	59.3	69.1	72.1	88.4	89.9	85.4	85.5	89.5	91.9	70.5	67.4	76.9	75.0	59.0	55.5
Saudi Arabia	65 to 69	38.4	58.2	32.6	57.0	40.8	58.1	44.5	59.8	43.6	60.4	43.5	58.2	77.2	84.8	76.9	85.1	78.4	84.7	30.4	51.1	17.8	44.8	38.5	55.8
Senegal	65 to 69	51.0	53.8	56.3	59.3	45.9	47.7	52.7	54.8	44.1	47.3	58.6	61.5	81.7	83.7	79.6	80.1	82.6	85.4	56.9	58.5	71.7	72.5	40.5	39.3
Serbia	65 to 69	50.6	57.4	52.4	61.2	48.8	54.0	47.4	47.3	45.9	51.7	48.0	43.1	48.5	55.8	41.9	49.4	56.3	63.6	45.8	49.3	46.9	52.0	45.9	47.7
Seychelles	65 to 69	59.3	72.1	68.1	78.1	51.3	67.1	59.2	68.9	62.7	71.0	55.9	66.9	80.6	91.2	83.9	93.2	76.5	88.0	65.6	75.3	74.8	81.1	58.4	72.0
Sierra Leone	65 to 69	51.2	51.0	56.3	52.6	46.3	49.4	53.8	52.5	44.8	42.2	60.0	62.2	82.9	83.3	81.5	78.1	83.4	85.9	56.2	55.4	72.3	68.5	39.6	39.7
Singapore	65 to 69	76.5	95.7	74.1	93.6	79.7	98.1	85.0	98.8	83.0	97.3	87.4	100.0	86.6	97.9	83.9	95.9	91.1	99.5	74.1	94.0	68.5	90.0	79.9	97.9
Slovakia	65 to 69	64.6	75.1	67.6	79.0	61.9	71.7	48.3	62.4	52.1	67.0	44.8	57.8	71.4	79.0	70.6	80.5	72.5	77.2	63.7	73.4	65.1	75.1	63.5	73.6
Slovenia	65 to 69	58.4	80.6	64.7	85.8	51.5	76.8	58.1	76.8	62.0	82.0	54.0	72.6	71.3	84.8	71.1	85.9	70.5	83.3	53.1	80.0	58.6	82.7	47.4	80.3
Solomon Islands	65 to 69	42.5	48.1	48.5	54.1	37.0	41.6	57.4	58.8	59.0	61.3	55.2	56.1	68.2	76.2	67.4	75.0	70.0	78.4	72.1	73.9	72.0	73.8	73.4	74.7
Somalia	65 to 69	27.0	38.1	44.1	52.6	6.0	16.3	15.4	16.3	25.7	29.8	4.7	1.5	68.1	74.7	78.7	82.8	61.4	67.9	68.6	71.7	70.6	74.5	65.6	65.7
South Sudan	65 to 69	72.6	62.6	54.3	57.8	21.8	28.7	63.9	48.5	41.2	39.8	18.0	19.3	93.2	88.6	82.7	83.7	73.6	76.4	78.8	69.5	72.3	73.2	63.4	62.7
Spain	65 to 69	34.9	42.8	74.8	89.9	67.1	86.7	28.6	29.2	76.8	87.0	71.5	81.9	75.0	78.4	86.4	93.7	83.4	87.2	66.7	67.9	72.8	88.3	72.4	91.4
Sri Lanka	65 to 69	70.8	87.9	64.7	74.1	51.1	62.1	74.3	84.3	57.6	63.4	48.0	55.4	85.2	91.2	88.5	92.4	82.3	86.4	71.5	89.0	69.6	75.1	60.1	68.2
Sudan	65 to 69	57.5	68.3	21.5	50.2	39.7	60.2	52.6	59.2	34.5	46.9	37.6	57.8	84.7	89.4	55.8	65.0	56.3	71.5	63.9	71.5	10.9	38.9	44.5	55.9
Suriname	65 to 69	33.1	56.3	45.7	56.7	32.3	38.3	36.8	52.8	49.2	54.8	42.8	46.2	54.3	67.9	73.0	76.4	71.7	73.3	31.0	48.2	58.8	65.7	47.1	53.1
Sweden	65 to 69	39.1	47.7	80.7	93.7	76.4	91.1	45.9	50.6	82.5	92.3	81.9	95.0	72.6	75.3	75.7	90.1	83.9	90.8	53.0	59.4	78.4	92.1	79.1	93.2
Switzerland	65 to 69	78.4	91.9	78.2	91.6	72.7	91.1	82.3	93.8	80.2	92.2	76.6	90.4	79.8	90.9	75.9	91.6	78.6	90.7	77.6	91.5	77.7	89.1	75.7	92.8
Syrian Arab Republic	65 to 69	75.2	91.2	52.6	59.8	50.3	56.8	78.6	91.4	58.4	61.9	57.3	59.0	77.1	91.6	79.3	85.5	75.6	82.5	75.5	90.4	43.9	49.7	49.1	54.5
Taiwan (Province of China)	65 to 69	51.5	58.3	63.1	89.4	56.1	76.1	58.2	60.6	64.5	84.4	59.4	76.9	76.9	84.1	76.6	91.5	77.9	83.4	46.3	51.8	70.0	88.9	69.7	85.2
Tajikistan	65 to 69	59.3	82.4	54.3	56.8	40.8	51.2	62.0	80.2	53.7	51.4	55.7	59.5	76.9	88.0	73.6	73.7	77.4	79.7	68.6	85.9	56.9	58.4	36.8	54.2
Thailand	65 to 69	48.3	54.0	66.1	79.4	53.8	68.4	54.2	56.2	64.9	73.3	63.4	70.3	75.1	76.4	66.0	78.5	53.7	70.4	49.1	56.0	73.4	81.8	66.8	76.9
Timor-Leste	65 to 69	60.0	73.9	28.8	43.9	36.4	39.6	64.2	71.8	31.7	41.8	45.0	47.4	59.1	73.8	55.7	64.9	67.1	71.6	69.8	79.0	61.6	66.6	59.9	55.9
Togo	65 to 69	32.7	41.7	52.0	53.8	47.9	40.0	38.4	44.4	41.6	42.8	60.1	55.4	61.0	67.9	78.3	78.3	82.7	81.8	60.2	60.9	70.5	70.8	44.2	29.8

Tokelau	65 to 69	49.9	47.5	47.8	63.0	27.0	47.8	51.6	48.5	54.0	58.7	39.5	50.6	81.1	80.6	66.1	78.9	71.2	84.7	58.5	54.1	68.2	76.0	60.1	70.8
Tonga	65 to 69	38.8	56.4	64.5	70.0	57.3	60.6	46.9	54.4	63.2	66.1	62.5	63.7	68.5	81.6	79.9	84.7	85.7	90.6	65.2	73.8	77.8	79.2	74.3	75.8
Trinidad and Tobago	65 to 69	60.9	65.4	56.7	75.1	45.6	57.9	62.8	64.9	54.5	68.7	47.3	56.3	82.5	87.4	67.9	77.0	69.7	73.3	75.4	76.9	61.5	77.2	43.8	60.4
Tropical Latin America	65 to 69	51.1	66.1	58.9	72.6	46.7	62.5	50.9	62.7	59.1	66.5	52.5	58.6	69.0	75.5	68.4	61.2	75.8	72.5	52.0	67.5	61.0	76.2	43.8	66.3
Tunisia	65 to 69	54.3	67.8	52.9	69.1	55.1	66.7	57.7	68.1	58.2	68.3	57.3	67.9	74.9	83.6	77.6	85.5	73.5	82.0	45.9	60.3	40.3	59.5	51.1	62.1
Turkey	65 to 69	59.0	72.9	64.1	76.6	54.2	69.1	61.6	73.1	65.5	75.7	57.6	70.1	57.5	75.5	65.3	79.6	49.8	71.0	59.0	72.0	62.3	73.4	56.9	71.5
Turkmenistan	65 to 69	51.8	51.2	57.3	59.9	44.5	40.9	53.7	48.0	53.9	51.7	54.3	43.9	62.3	49.3	63.5	54.1	60.9	46.8	49.6	51.0	53.9	56.8	42.4	43.5
Tuvalu	65 to 69	29.0	48.5	36.3	54.8	21.4	41.2	43.6	51.9	45.9	54.9	41.4	49.5	61.5	76.7	59.1	73.5	64.4	80.7	61.8	69.2	65.1	71.8	58.7	66.2
Uganda	65 to 69	47.5	59.3	64.3	70.8	29.9	44.2	34.5	46.7	49.7	58.0	20.6	34.7	76.7	84.2	85.9	89.3	71.9	80.2	73.5	76.2	76.3	78.6	70.3	72.1
Ukraine	65 to 69	58.2	66.7	61.7	72.4	54.0	60.2	60.5	65.5	62.8	69.2	58.8	61.1	58.2	68.4	60.6	73.8	53.2	63.1	46.6	59.5	48.3	64.1	44.1	54.7
United Arab Emirates	65 to 69	59.1	84.8	52.2	84.8	63.3	84.7	57.3	80.0	54.6	75.6	59.8	81.0	42.1	82.7	27.1	81.7	56.8	83.5	53.0	84.2	41.1	80.7	62.0	87.7
United Kingdom	65 to 69	71.6	88.4	74.0	90.0	69.3	87.1	72.9	84.8	73.6	85.1	72.5	84.2	69.6	85.3	64.2	84.9	76.2	85.1	69.0	89.7	71.2	90.0	68.3	91.1
United Republic of Tanzania	65 to 69	48.9	61.4	59.2	67.0	38.4	55.6	41.8	52.2	48.9	56.3	34.6	47.8	80.0	84.9	85.1	86.4	77.4	84.0	73.4	71.9	74.3	74.9	72.7	69.0
United States Virgin Islands	65 to 69	54.9	78.0	64.5	86.6	42.7	69.7	53.1	74.2	60.9	79.5	44.5	69.2	74.1	90.5	75.4	94.5	70.4	84.8	61.3	81.9	66.7	86.1	54.2	79.7
United States of America	65 to 69	82.8	100.0	85.1	100.0	80.4	100.0	78.6	89.1	79.6	90.0	77.4	88.1	75.4	83.2	75.3	85.5	74.1	78.3	83.7	100.0	84.3	100.0	84.4	100.0
Uruguay	65 to 69	73.5	79.6	76.2	82.7	71.0	76.6	73.4	79.4	78.0	84.0	67.7	73.6	69.6	76.1	70.0	75.5	68.7	76.3	74.2	80.2	75.6	82.2	73.8	79.2
Uzbekistan	65 to 69	58.2	64.5	63.3	69.7	51.7	59.4	53.1	63.5	53.5	63.1	53.6	63.0	83.0	58.0	86.1	65.1	79.7	54.2	63.7	66.3	67.5	69.6	58.0	64.0
Vanuatu	65 to 69	45.1	54.3	55.6	62.6	36.1	45.8	51.4	55.4	56.7	59.9	46.2	51.0	70.3	79.0	69.5	77.2	71.7	81.1	68.6	74.5	74.3	78.1	64.6	71.5
Venezuela (Bolivarian Republic of)	65 to 69	60.2	66.1	66.6	74.8	52.8	55.9	61.6	63.5	66.2	71.0	56.4	54.5	81.9	82.1	81.8	81.4	80.6	81.4	70.3	74.7	74.3	80.4	66.5	69.3
Viet Nam	65 to 69	36.6	48.7	47.0	61.2	26.4	38.7	45.9	49.7	50.2	56.8	41.6	43.0	66.7	75.5	71.8	80.1	61.1	70.5	56.6	59.7	66.1	71.5	47.6	51.7
Yemen	65 to 69	19.7	37.4	18.9	36.8	20.2	38.0	27.0	36.4	34.7	35.1	19.2	38.5	45.8	56.2	55.9	59.7	38.0	54.1	16.1	25.2	6.6	23.2	24.8	27.6
Zambia	65 to 69	41.0	49.2	52.8	60.2	28.1	35.2	33.3	39.8	35.4	42.3	30.0	36.7	76.0	79.2	80.1	80.9	74.6	78.5	71.6	69.4	73.7	73.5	68.6	62.6
Zimbabwe	65 to 69	54.5	45.2	62.7	54.6	44.7	27.4	29.6	0.0	27.0	0.0	30.8	6.2	77.9	73.8	79.7	74.7	77.1	72.5	69.7	63.0	74.9	70.0	62.4	44.9
Afghanistan	70 to 74	9.3	24.1	25.4	35.9	7.3	26.2	9.3	19.9	44.1	44.9	6.5	25.6	42.5	56.6	73.8	74.8	33.8	52.6	24.1	28.0	30.2	37.3	24.7	29.1
Albania	70 to 74	35.9	54.2	53.7	65.2	27.8	49.9	29.6	42.4	53.7	58.8	30.1	46.4	73.8	81.7	85.3	88.1	70.9	81.5	65.6	75.8	75.5	81.0	58.3	72.6
Algeria	70 to 74	63.4	71.1	71.1	76.6	61.1	69.7	60.4	65.3	71.0	72.8	63.4	70.1	81.1	84.2	89.1	90.0	78.8	83.6	65.7	72.1	71.3	75.7	63.7	71.3
American Samoa	70 to 74	58.8	62.2	71.1	73.7	52.1	55.6	54.1	54.4	67.8	68.7	56.3	56.0	81.1	82.9	83.8	85.4	88.6	90.4	76.4	78.3	81.7	82.8	73.0	75.5
Andorra	70 to 74	80.2	88.9	83.8	90.6	78.5	87.9	78.6	82.8	82.7	85.6	81.2	85.1	86.9	91.7	90.0	92.9	89.4	94.1	81.1	90.6	82.8	90.6	80.2	90.3
Angola	70 to 74	47.3	55.8	66.5	71.3	31.8	41.8	32.6	38.1	54.8	58.6	34.6	40.1	81.5	84.9	90.4	91.3	78.5	82.9	67.1	68.7	78.1	78.7	54.8	56.3
Antigua and Barbuda	70 to 74	43.9	56.9	65.3	74.6	28.4	42.3	43.0	47.6	64.8	70.2	41.1	41.9	77.3	83.8	88.2	90.3	71.5	81.7	56.6	67.0	70.0	77.4	45.1	57.2
Argentina	70 to 74	66.8	80.6	76.9	88.1	60.8	74.6	69.1	74.9	80.5	84.9	66.4	71.6	73.1	86.0	83.0	91.1	72.3	85.0	72.3	85.8	78.8	89.6	68.0	82.0
Armenia	70 to 74	59.7	73.1	70.7	81.6	53.8	67.4	52.5	64.3	65.8	74.9	57.5	65.4	70.3	80.9	80.2	89.0	71.4	77.9	63.0	73.0	72.0	80.3	56.7	67.4
Australia	70 to 74	77.0	92.9	82.9	95.8	73.8	89.9	73.3	83.2	80.2	87.4	75.2	83.7	80.9	90.9	87.2	94.7	81.8	88.1	79.1	95.4	82.8	96.0	76.8	93.6
Austria	70 to 74	73.0	95.1	80.0	97.4	69.4	92.2	81.2	88.9	85.4	91.1	82.4	89.1	93.8	100.0	95.4	100.0	93.5	97.7	69.0	95.1	74.5	95.6	66.7	93.4
Azerbaijan	70 to 74	52.5	56.2	66.8	67.8	41.5	50.3	45.0	47.9	60.9	60.5	50.4	54.7	86.9	87.6	92.2	92.6	83.9	85.9	68.9	70.7	78.3	78.5	57.6	64.5
Bahamas	70 to 74	52.7	69.1	69.1	79.1	40.2	62.2	48.1	62.1	67.6	74.6	45.6	61.9	83.9	87.7	90.8	93.3	80.0	83.9	63.9	75.5	73.5	80.6	56.1	72.3
Bahrain	70 to 74	28.0	55.0	39.2	62.0	28.9	54.9	35.8	57.1	54.4	69.0	41.6	60.8	55.8	75.6	69.6	83.3	60.7	77.7	32.2	55.5	41.4	60.3	31.4	55.4
Bangladesh	70 to 74	28.3	45.0	42.9	57.1	24.8	41.3	48.5	58.3	65.5	71.6	49.4	59.3	53.5	69.0	74.7	79.9	51.8	70.2	41.9	52.4	49.3	60.6	40.2	49.6
Barbados	70 to 74	53.0	64.1	67.5	75.8	44.1	56.4	53.0	58.0	69.1	72.4	53.0	57.7	81.3	81.9	89.5	88.5	77.5	81.3	55.6	67.9	66.2	76.1	48.8	61.9
Belarus	70 to 74	60.9	69.0	70.4	79.2	57.9	61.6	60.1	61.9	71.2	73.4	64.0	63.0	76.6	76.4	86.8	88.2	67.8	66.7	58.2	68.4	66.6	77.3	55.4	60.9
Belgium	70 to 74	68.2	85.4	76.7	90.7	63.6	81.2	69.4	76.7	78.5	84.3	70.3	76.1	86.5	85.3	90.8	92.4	86.7	81.2	69.3	88.8	75.9	91.4	65.4	86.3
Belize	70 to 74	56.8	63.6	68.4	76.3	51.3	55.9	52.2	56.0	65.9	69.7	55.7	57.4	85.6	89.1	91.9	93.5	82.3	87.2	69.6	72.6	75.9	80.8	66.2	66.3
Benin	70 to 74	43.9	50.2	58.2	63.9	38.4	43.4	47.0	53.3	52.3	59.6	60.4	65.3	81.6	85.9	87.1	89.8	83.4	87.5	56.8	59.1	74.5	75.4	38.4	39.9
Bermuda	70 to 74	65.2	80.4	79.1	94.7	52.6	67.1	65.4	73.5	78.3	87.7	62.2	67.4	87.1	92.4	93.7	99.0	80.6	83.7	64.6	80.9	77.1	92.0	51.2	69.8
Bhutan	70 to 74	46.2	58.7	53.8	67.1	47.8	56.5	58.3	66.1	69.5	74.8	62.3	69.2	66.5	74.6	78.0	82.8	67.9	76.0	54.8	63.1	57.9	69.2	58.3	60.9
Bolivia (Plurinational State of)	70 to 74	36.6	54.8	51.4	66.3	32.3	50.1	32.5	45.3	52.0	61.5	39.5	49.9	64.8	69.8	74.6	77.4	71.3	77.1	50.3	64.9	62.0	73.8	43.2	58.5
Bosnia and Herzegovina	70 to 74	53.5	66.3	63.1	74.5	52.8	63.0	33.4	50.7	53.0	65.8	40.4	52.9	62.3	75.4	75.6	84.1	61.8	74.9	52.0	65.3	60.2	72.0	51.9	62.5
Botswana	70 to 74	54.2	67.9	68.7	78.7	43.7	58.7	41.5	56.1	56.7	67.0	49.0	61.7	84.4	90.4	90.8	93.9	82.5	88.8	65.6	74.1	76.3	82.3	53.8	63.9

Brazil	70 to 74	51.6	69.6	65.8	79.8	44.2	63.0	53.5	62.1	68.4	74.4	54.8	62.4	84.9	78.8	89.0	85.5	86.8	80.0	52.6	72.3	65.8	80.7	43.3	65.9
Brunei Darussalam	70 to 74	75.8	84.2	80.0	87.3	74.2	82.7	77.8	83.7	83.6	87.2	79.3	85.3	78.3	87.0	84.0	90.2	82.5	90.0	79.6	86.7	81.7	87.8	78.3	85.8
Bulgaria	70 to 74	39.7	56.1	53.1	70.4	35.9	47.0	34.7	50.1	55.0	65.8	38.0	51.2	75.8	67.1	85.6	82.5	73.8	59.9	46.7	55.4	57.7	68.9	42.0	44.7
Burkina Faso	70 to 74	49.1	51.1	74.0	72.8	28.4	33.8	45.9	51.0	64.0	65.2	46.9	53.9	86.5	88.2	95.6	95.3	81.0	84.1	65.8	64.2	87.0	84.6	39.2	38.8
Burundi	70 to 74	36.8	51.2	59.1	68.5	18.9	39.4	17.4	30.8	49.4	57.1	13.3	28.4	77.0	83.4	89.0	91.8	72.2	80.9	61.9	69.8	71.3	77.4	55.4	64.4
Cabo Verde	70 to 74	64.2	63.9	74.4	80.8	58.7	47.3	65.7	68.0	71.4	76.5	74.2	70.7	93.0	96.0	95.9	98.2	91.4	92.8	70.7	65.3	82.4	84.6	56.1	37.2
Cambodia	70 to 74	26.9	41.1	41.6	56.5	26.1	34.1	27.6	37.5	45.2	55.6	40.1	43.6	65.3	74.6	77.4	83.5	66.4	74.6	53.3	59.5	63.1	69.7	49.4	52.3
Cameroon	70 to 74	49.0	50.2	66.0	66.6	38.0	40.0	49.3	52.2	61.2	61.7	55.9	60.2	84.0	86.6	90.6	91.5	83.0	86.4	65.0	60.7	80.1	78.4	47.0	40.6
Canada	70 to 74	82.9	98.7	88.7	100.0	78.4	96.3	81.7	92.4	86.8	93.9	81.6	92.1	88.1	92.1	92.6	95.0	85.4	90.0	83.9	99.8	87.6	99.4	80.9	98.3
Central African Republic	70 to 74	32.6	36.9	55.8	58.6	13.0	15.9	21.0	19.6	48.0	48.3	22.2	19.0	75.6	77.6	86.9	87.7	72.1	74.1	56.6	58.9	69.8	71.6	42.2	42.8
Chad	70 to 74	49.0	44.4	65.2	63.0	40.0	34.4	50.7	49.4	58.6	56.7	59.4	58.0	84.0	83.1	90.3	88.6	83.8	84.0	60.7	53.5	78.6	76.1	42.1	31.6
Chile	70 to 74	64.7	83.1	75.5	90.2	58.2	77.1	70.6	82.7	81.1	88.7	67.9	80.4	82.3	84.8	87.8	90.4	83.4	83.2	66.1	85.6	74.9	90.9	60.0	80.4
China	70 to 74	34.0	63.0	51.6	76.2	26.6	54.7	38.6	54.0	59.2	70.0	39.8	53.6	0.0	67.4	36.1	81.0	0.0	64.8	59.3	72.7	70.1	81.5	52.3	66.0
Colombia	70 to 74	64.2	79.4	74.3	88.1	58.5	71.3	58.5	71.6	71.2	81.7	60.4	69.3	84.9	82.4	90.0	88.2	84.8	82.5	68.7	83.0	76.6	89.6	63.0	75.5
Comoros	70 to 74	51.1	61.7	66.8	73.8	40.8	52.5	32.1	39.7	51.1	56.8	38.2	46.3	84.4	88.4	91.0	92.9	83.1	87.2	70.0	75.0	78.5	82.0	62.3	67.3
Congo	70 to 74	48.6	56.4	64.5	69.2	36.8	48.0	30.8	37.1	49.6	52.9	39.6	47.3	82.1	85.6	88.9	90.3	81.4	86.0	66.3	68.5	76.5	77.4	54.7	59.1
Cook Islands	70 to 74	57.0	76.3	72.4	86.1	47.7	68.8	51.9	67.2	69.4	79.9	51.1	64.9	81.0	93.4	85.1	93.8	85.9	96.3	74.2	85.7	81.5	89.6	68.9	82.1
Costa Rica	70 to 74	74.7	83.3	83.9	91.3	67.4	75.5	69.6	75.6	79.0	83.8	69.7	74.6	91.1	90.3	94.5	93.7	89.0	89.1	77.8	85.2	84.7	91.0	71.3	78.9
Coted'Ivoire	70 to 74	53.9	54.2	71.1	70.0	43.9	44.5	56.6	57.3	65.2	64.9	61.7	64.4	86.6	88.5	91.9	92.2	86.0	88.6	61.2	59.8	80.5	78.9	43.9	40.4
Croatia	70 to 74	51.5	73.5	61.6	80.7	49.5	69.5	43.1	52.7	61.9	71.1	44.1	49.0	63.7	69.4	75.4	82.6	66.5	64.7	52.6	76.7	60.3	80.9	51.5	74.2
Cuba	70 to 74	61.9	67.6	73.6	79.7	54.8	58.8	59.0	62.0	72.0	75.0	60.0	61.5	87.9	89.6	92.5	93.8	86.2	87.0	66.0	68.8	74.8	79.0	59.6	60.0
Cyprus	70 to 74	66.5	77.2	72.3	85.0	65.5	72.0	72.4	79.2	79.3	85.3	74.6	79.4	79.5	89.7	85.6	93.2	81.6	89.1	68.8	78.7	71.4	84.1	69.9	74.6
Czechia	70 to 74	52.7	81.2	64.6	86.8	47.7	77.4	49.0	69.1	65.5	78.8	50.1	69.0	71.1	80.4	82.0	89.3	69.5	76.6	49.2	83.2	60.4	86.4	43.6	80.8
Democratic People's Republic of Korea	70 to 74	48.3	52.2	62.7	66.7	40.1	43.4	39.1	35.2	60.0	57.1	39.3	36.3	53.6	62.8	71.1	78.2	46.0	56.8	69.1	70.0	76.7	78.5	63.5	63.0
Democratic Republic of the Congo	70 to 74	48.4	48.4	64.4	65.4	38.0	33.6	38.4	32.9	56.1	55.8	43.3	34.0	83.4	83.4	90.2	90.3	82.3	81.5	64.8	65.6	74.4	75.6	56.5	54.1
Denmark	70 to 74	75.7	87.2	82.2	92.7	72.2	82.5	71.2	78.3	78.9	85.0	73.1	78.1	83.4	89.4	88.7	93.9	84.6	87.0	77.8	89.9	82.3	93.1	75.0	86.5
Djibouti	70 to 74	55.0	62.5	70.9	76.8	43.3	51.8	34.8	41.3	55.4	58.7	37.6	44.6	84.3	87.8	91.9	93.2	81.8	86.2	74.3	74.6	81.6	83.6	67.3	65.3
Dominica	70 to 74	35.2	46.2	57.2	64.1	17.8	35.0	30.4	41.3	55.7	62.4	29.9	41.1	75.3	79.3	87.6	89.4	67.7	75.1	46.4	52.8	62.2	65.6	31.0	43.3
Dominican Republic	70 to 74	55.4	64.0	69.8	76.8	46.9	55.3	49.1	59.0	66.0	72.0	50.3	59.9	83.9	87.8	90.7	92.4	81.1	85.9	67.1	71.1	76.6	80.1	59.8	63.8
Ecuador	70 to 74	67.2	79.9	78.8	87.9	59.2	73.3	56.2	67.1	70.7	78.1	57.0	66.7	91.7	79.7	94.1	86.4	91.3	80.7	71.7	85.7	80.6	91.1	64.0	80.1
Egypt	70 to 74	2.8	37.2	22.0	45.0	1.1	39.8	0.0	40.4	36.3	57.3	0.0	45.3	50.8	70.7	77.0	82.4	38.6	69.2	0.0	30.0	14.4	36.2	0.0	32.8
El Salvador	70 to 74	63.1	75.2	75.5	84.5	54.4	66.9	58.4	65.5	71.7	76.9	59.3	64.7	85.8	88.7	90.7	92.1	85.2	88.9	71.0	80.0	80.9	87.1	61.6	72.1
Equatorial Guinea	70 to 74	41.8	61.3	62.1	73.5	23.9	51.3	26.2	51.1	50.2	64.8	29.4	56.4	79.5	91.0	89.2	94.0	75.9	90.2	63.9	69.4	75.8	78.2	48.9	58.9
Eritrea	70 to 74	40.1	52.2	59.3	68.4	23.3	35.2	18.2	25.0	46.1	52.1	19.9	23.7	79.5	85.1	89.7	92.0	73.4	80.8	68.9	72.6	76.4	80.3	62.6	62.5
Estonia	70 to 74	46.1	81.4	60.2	90.0	39.5	73.1	51.7	68.2	66.7	78.6	53.9	67.5	80.4	80.3	89.9	90.8	69.7	69.8	39.8	82.7	54.2	89.6	31.2	75.0
Eswatini	70 to 74	47.8	52.4	65.4	66.7	30.9	41.3	30.7	36.8	52.7	54.0	34.7	45.7	81.8	83.8	90.2	90.1	77.6	82.1	63.6	63.5	74.8	73.5	49.4	52.5
Ethiopia	70 to 74	38.5	57.9	60.8	72.6	24.8	47.6	30.3	45.9	53.4	63.8	31.4	46.7	81.7	87.8	92.0	93.5	78.8	86.0	72.5	75.9	79.1	82.0	68.1	71.1
Fiji	70 to 74	55.7	60.3	69.9	72.7	47.4	52.5	43.6	41.8	63.0	62.0	44.0	42.4	78.5	80.8	83.9	85.8	84.0	87.3	76.0	77.4	82.7	82.9	70.9	73.4
Finland	70 to 74	76.3	90.4	81.3	94.8	74.9	86.1	78.4	86.4	83.3	89.6	80.8	86.6	88.0	94.0	90.9	95.9	91.7	93.0	75.2	91.0	78.7	93.7	74.5	87.8
France	70 to 74	74.1	93.1	81.5	95.8	70.0	90.3	68.4	83.6	79.0	87.2	68.2	84.6	87.3	93.0	91.6	96.2	87.2	90.5	76.6	94.9	81.8	95.6	73.1	93.1
Gabon	70 to 74	56.3	62.3	72.8	76.2	41.7	50.9	41.3	46.2	60.8	62.8	42.4	48.7	85.9	89.2	92.7	94.0	82.5	87.0	72.4	72.6	81.2	81.1	62.3	63.5
Gambia	70 to 74	51.6	47.2	68.8	65.4	41.0	35.0	51.2	48.2	59.0	58.0	59.6	58.4	86.3	85.5	91.5	90.3	86.1	86.3	60.8	54.6	80.2	75.8	40.6	29.2
Georgia	70 to 74	44.7	52.8	62.1	69.0	28.7	40.3	44.8	51.8	61.9	66.9	48.1	53.0	75.4	68.3	85.4	78.5	70.9	70.2	59.7	57.9	72.1	71.5	43.1	44.2
Germany	70 to 74	77.7	90.8	84.3	94.0	73.2	87.8	74.1	85.5	81.8	89.4	73.9	85.3	88.8	92.9	92.6	95.5	87.8	91.3	77.9	91.7	82.8	93.0	74.2	89.8
Ghana	70 to 74	53.1	57.7	67.7	72.3	43.7	46.4	46.5	51.0	55.4	60.3	60.5	62.3	87.3	89.2	91.4	92.0	87.8	90.6	64.0	64.6	80.1	80.3	42.3	42.7
Greece	70 to 74	62.0	81.0	68.7	86.6	61.8	77.3	64.9	74.2	74.1	81.8	69.0	74.7	86.6	90.7	90.6	95.0	87.5	87.6	65.3	86.4	69.7	88.4	65.5	84.6
Greenland	70 to 74	69.0	79.3	76.1	86.3	66.7	75.0	61.4	69.7	73.0	79.5	64.1	70.3	51.5	75.5	67.5	84.5	58.6	75.5	75.2	87.1	79.6	89.8	73.3	84.5
Grenada	70 to 74	36.7	58.8	56.7	72.3	23.4	50.2	41.7	50.6	62.7	67.1	40.2	51.2	75.7	80.3	86.9	87.9	69.7	78.9	38.1	64.8	55.4	74.0	24.0	57.8

Guam	70 to 74	68.5	82.1	79.4	89.8	60.5	75.5	62.3	71.1	75.0	80.6	61.8	70.5	83.2	93.5	86.6	93.5	88.8	97.3	77.3	89.2	83.9	93.2	71.3	84.7
Guatemala	70 to 74	60.8	70.7	73.5	81.0	52.5	63.0	57.5	61.4	71.1	73.6	58.6	62.1	95.6	91.0	97.5	95.1	93.3	88.1	68.1	80.2	77.1	86.4	60.8	74.2
Guinea	70 to 74	49.2	49.0	61.5	63.0	45.0	42.7	49.3	52.7	55.2	57.2	62.5	63.9	85.8	86.8	90.0	90.0	86.8	88.3	62.4	57.1	76.5	75.7	47.3	38.8
Guinea-Bissau	70 to 74	31.3	36.9	57.0	57.6	15.3	23.1	36.8	37.4	48.6	49.4	46.5	51.1	77.2	79.8	87.6	86.7	75.9	81.2	44.8	48.2	74.6	72.7	12.4	16.3
Guyana	70 to 74	11.9	40.2	36.8	57.3	0.0	31.3	31.2	44.0	55.9	62.5	30.4	45.1	73.9	78.7	85.1	88.2	70.4	74.7	21.5	46.5	43.5	60.2	5.6	37.1
Haiti	70 to 74	0.0	15.8	17.4	31.5	1.7	15.8	18.8	24.2	43.6	46.5	24.9	30.8	52.8	59.6	70.1	73.5	53.9	61.8	16.2	28.1	25.3	37.8	22.2	30.4
Honduras	70 to 74	48.5	33.3	64.0	49.9	39.9	27.2	39.3	24.1	59.4	47.7	41.7	30.7	77.1	60.2	85.4	71.2	76.9	67.8	58.5	46.0	72.3	61.7	46.8	34.1
Hungary	70 to 74	59.1	75.6	69.8	84.0	54.3	69.3	44.6	61.7	61.2	74.6	48.2	60.7	78.2	82.1	86.7	89.8	75.7	78.4	60.6	76.4	69.4	83.0	55.6	70.9
Iceland	70 to 74	77.4	92.3	83.4	96.3	73.9	88.5	77.0	85.2	82.8	90.0	78.4	84.9	88.1	93.8	91.4	95.7	89.5	93.0	78.4	93.7	82.7	95.5	75.5	91.4
India	70 to 74	43.3	48.0	54.5	58.8	40.8	45.2	58.3	61.7	70.9	72.6	59.6	64.0	63.1	68.9	78.1	79.5	61.8	70.3	52.0	53.7	58.1	61.5	51.6	51.2
Indonesia	70 to 74	48.4	49.6	59.8	64.0	45.9	42.2	44.7	44.8	59.4	60.2	52.0	49.9	72.5	74.5	81.4	82.9	75.1	76.4	66.7	62.9	74.0	74.0	62.8	53.9
Iran (Islamic Republic of)	70 to 74	50.0	70.7	61.7	76.7	45.9	69.0	55.6	68.5	69.5	76.7	57.1	71.1	72.2	85.0	84.0	90.6	70.0	83.8	44.3	68.1	55.3	72.7	39.4	67.1
Iraq	70 to 74	31.9	46.9	50.0	63.1	24.6	38.7	37.1	48.6	58.8	66.4	37.8	48.5	74.7	79.2	86.4	88.3	68.6	76.5	30.7	45.3	47.4	60.7	21.6	35.0
Ireland	70 to 74	70.6	88.2	78.0	93.0	66.9	83.9	76.2	84.4	81.9	88.2	78.3	84.6	83.2	89.7	89.0	93.7	83.0	87.7	68.8	90.3	75.0	93.0	65.5	87.1
Israel	70 to 74	77.9	94.7	80.5	97.7	78.0	91.3	67.6	77.4	75.5	84.5	71.3	77.0	93.3	94.8	95.2	98.2	92.7	90.4	82.8	99.3	82.7	99.1	83.9	97.6
Italy	70 to 74	64.3	82.7	73.5	87.8	59.9	79.1	66.7	76.2	77.3	83.1	66.8	76.7	86.0	88.1	90.4	93.0	86.4	85.7	64.1	86.9	71.2	88.7	60.5	85.2
Jamaica	70 to 74	42.6	46.8	59.7	62.6	33.1	38.4	50.1	48.4	66.2	65.3	51.3	49.4	83.1	81.4	89.4	89.2	81.7	78.4	45.9	53.7	58.5	64.2	38.3	47.6
Japan	70 to 74	80.0	96.8	85.9	99.6	76.0	93.4	86.3	100.0	89.9	100.0	85.8	98.5	85.5	95.6	89.8	96.3	87.3	96.1	79.4	95.4	83.9	97.0	76.3	92.8
Jordan	70 to 74	54.0	75.3	60.1	78.9	56.9	75.2	51.1	64.0	63.1	73.2	59.0	67.4	90.4	93.2	93.7	95.5	89.3	92.1	52.4	75.9	57.0	77.4	55.8	76.9
Kazakhstan	70 to 74	59.8	60.7	72.0	75.2	49.9	47.2	53.0	47.8	66.3	64.8	58.0	48.7	69.9	65.8	80.4	78.5	69.4	64.7	62.0	65.9	73.0	78.3	49.5	50.7
Kenya	70 to 74	63.1	62.3	74.7	75.4	55.2	52.2	48.8	46.5	64.2	62.8	51.5	49.3	88.1	88.5	93.2	93.4	86.5	86.8	79.2	76.7	83.5	83.1	76.3	70.4
Kiribati	70 to 74	52.4	54.7	71.2	71.2	37.6	40.9	52.3	50.5	68.7	67.0	51.8	51.0	81.6	83.9	88.3	88.7	79.7	85.7	73.4	72.7	83.7	82.7	62.4	60.9
Kuwait	70 to 74	78.2	83.2	79.5	91.3	78.1	78.0	72.1	77.3	77.5	85.3	75.5	76.8	86.3	94.4	89.7	97.9	87.7	91.4	78.1	83.6	77.0	89.9	78.9	79.3
Kyrgyzstan	70 to 74	49.8	59.0	65.2	73.8	37.2	46.4	44.9	56.8	60.2	68.8	51.5	59.7	74.3	67.2	85.1	81.3	69.1	63.5	54.4	59.7	68.2	74.4	38.0	43.3
Lao People's Democratic Republic	70 to 74	23.5	45.0	39.3	58.7	20.9	39.8	27.4	41.1	45.5	55.6	36.1	48.0	58.7	75.0	73.4	83.5	60.6	76.3	43.7	59.6	54.7	70.7	40.0	52.1
Latvia	70 to 74	51.4	69.2	64.2	78.1	45.0	64.0	48.8	62.5	64.2	74.8	52.4	62.1	76.0	76.7	86.3	87.7	67.1	68.2	49.9	68.1	61.9	75.5	42.3	63.3
Lebanon	70 to 74	47.9	79.6	61.5	85.7	42.3	75.3	34.7	68.3	63.5	79.4	25.2	66.3	58.6	84.1	78.2	91.7	50.5	79.3	56.9	82.6	63.6	86.0	55.2	80.2
Lesotho	70 to 74	44.6	38.6	65.1	59.3	19.1	14.4	33.2	23.9	57.7	48.9	30.5	27.4	79.4	77.5	90.1	87.4	71.5	72.3	62.9	54.6	75.1	69.1	43.0	31.1
Liberia	70 to 74	48.9	47.1	62.1	61.3	43.5	41.1	51.0	47.2	55.1	53.4	62.5	61.6	86.5	86.7	90.2	90.1	87.4	88.1	59.1	56.4	76.1	74.3	43.3	36.8
Libya	70 to 74	58.5	62.3	66.5	70.5	57.3	60.5	54.1	56.0	66.4	66.5	59.3	63.7	81.4	81.3	88.0	87.0	81.1	83.2	59.7	61.1	66.0	68.7	58.7	58.1
Lithuania	70 to 74	60.7	71.4	71.2	81.2	54.8	63.4	62.0	67.9	72.4	77.7	66.3	68.2	80.0	79.2	89.2	89.2	70.8	70.0	58.4	71.5	68.2	80.0	51.7	63.4
Luxembourg	70 to 74	59.7	83.2	70.5	89.9	54.7	78.3	62.7	76.2	74.7	84.8	63.6	75.2	83.9	90.0	88.8	94.0	85.0	88.3	59.2	86.4	68.8	90.4	53.5	82.7
Madagascar	70 to 74	41.3	49.2	63.0	66.3	26.2	36.4	21.7	25.7	48.1	50.4	23.3	28.3	78.5	82.9	89.7	90.2	75.4	81.6	68.0	70.2	78.4	79.2	58.2	60.8
Malawi	70 to 74	50.6	51.0	64.4	66.2	43.3	41.0	28.7	25.8	49.2	49.8	35.7	29.8	82.6	83.6	89.6	90.0	81.3	82.5	70.2	66.7	77.6	76.5	64.7	57.1
Malaysia	70 to 74	52.1	69.5	63.8	78.9	47.7	63.8	49.2	61.2	62.6	72.0	54.0	63.6	75.1	84.3	83.8	89.9	75.2	83.8	66.6	79.2	73.3	84.3	63.5	75.2
Maldives	70 to 74	43.7	67.5	53.8	76.0	41.5	63.5	37.0	60.0	50.2	67.6	43.8	64.9	73.2	86.9	82.1	90.9	74.8	87.3	63.7	76.5	68.8	82.1	61.8	73.0
Mali	70 to 74	46.6	49.4	58.0	60.1	44.4	47.1	47.6	51.8	52.6	53.8	60.9	64.9	83.9	87.0	88.4	89.8	85.5	89.0	62.1	60.2	73.6	73.4	52.1	48.5
Malta	70 to 74	63.6	84.0	73.5	88.7	58.4	80.5	69.1	82.2	77.7	86.2	70.8	83.3	92.6	96.4	94.9	97.9	91.8	94.1	62.6	85.6	71.6	87.7	56.9	84.0
Marshall Islands	70 to 74	41.5	46.3	62.3	63.9	27.0	36.1	39.9	41.6	61.7	61.8	38.8	41.6	70.8	77.1	78.9	82.1	75.2	82.3	69.0	70.3	77.9	77.5	61.8	65.7
Mauritania	70 to 74	50.3	56.5	63.2	68.5	45.1	50.7	46.7	55.4	51.7	59.8	64.3	68.4	85.7	89.8	89.5	91.7	87.7	91.5	62.3	63.3	77.3	77.8	43.2	46.6
Mauritius	70 to 74	56.0	70.8	69.7	82.3	48.0	62.2	55.6	64.4	69.1	75.9	57.2	64.5	75.6	79.9	87.8	89.8	67.5	73.5	59.1	75.4	70.1	83.3	51.6	68.9
Mexico	70 to 74	72.1	79.9	82.0	88.5	64.4	72.4	67.0	72.0	77.0	81.1	67.8	71.2	91.6	85.2	94.3	90.4	90.7	84.4	74.0	83.1	82.6	89.8	65.5	76.0
Micronesia (Federated States of)	70 to 74	40.6	50.2	58.9	66.2	30.1	39.7	40.5	46.6	60.9	64.3	40.8	47.2	70.0	77.9	77.7	83.3	76.0	82.9	67.6	71.3	75.4	78.8	62.5	65.5
Monaco	70 to 74	71.6	84.6	78.7	88.3	68.2	82.5	69.5	79.9	79.0	85.1	70.0	80.9	83.4	93.1	88.4	94.7	85.0	93.6	72.1	84.9	76.9	86.6	70.0	84.1
Mongolia	70 to 74	32.9	52.6	55.4	67.4	16.6	42.5	30.7	46.7	53.8	60.4	31.9	52.0	45.2	46.7	70.4	65.0	37.1	47.7	80.4	83.5	87.6	89.2	71.5	76.1
Montenegro	70 to 74	42.0	42.2	54.1	55.8	39.8	37.7	40.7	37.8	59.6	59.3	43.3	37.0	79.5	79.0	86.4	85.6	80.2	80.4	74.3	70.1	79.6	76.9	71.3	65.8
Morocco	70 to 74	43.6	54.8	46.6	60.5	49.0	55.6	42.8	51.3	54.8	61.4	52.9	58.9	70.7	74.7	80.5	83.3	72.2	75.6	44.3	53.6	45.0	57.7	50.0	54.7
Mozambique	70 to 74	41.2	42.0	59.7	63.2	29.4	25.0	32.3	30.3	56.0	57.4	32.3	25.8	77.6	78.3	88.2	88.7	74.6	74.8	62.0	58.8	72.8	73.5	52.4	42.5

Myanmar	70 to 74	25.0	42.4	43.0	60.8	19.3	31.6	28.1	36.8	45.4	56.5	37.4	39.8	64.1	73.7	78.6	84.2	61.7	71.7	46.5	59.2	60.3	72.1	37.9	48.6
Namibia	70 to 74	53.0	55.6	67.8	70.3	41.8	43.3	40.9	42.7	56.9	60.3	48.1	46.7	85.3	86.4	91.4	91.9	83.1	84.2	64.3	64.3	75.6	75.5	51.1	51.0
Nauru	70 to 74	43.2	44.9	61.5	64.1	33.8	29.5	38.0	17.1	61.3	52.1	37.1	9.5	71.5	70.7	77.1	78.0	80.5	84.4	64.8	68.0	74.8	78.2	58.0	58.1
Nepal	70 to 74	32.7	47.6	39.7	59.9	37.1	43.4	52.4	60.6	65.2	72.8	57.1	61.6	61.7	71.8	76.1	83.0	62.1	69.9	39.7	53.0	41.3	62.4	46.6	48.8
Netherlands	70 to 74	78.6	89.7	85.1	93.4	74.2	86.4	72.1	81.9	80.8	86.7	72.3	82.1	85.2	91.7	91.0	95.2	82.9	89.3	80.6	91.5	84.7	93.1	77.7	89.3
New Zealand	70 to 74	72.8	88.0	81.0	91.9	67.8	84.7	71.8	78.8	80.7	85.1	71.5	78.7	77.0	85.0	84.7	90.1	78.1	84.9	74.1	90.9	80.4	92.6	69.6	88.9
Nicaragua	70 to 74	68.9	75.2	79.4	83.9	61.7	68.6	64.4	69.1	75.2	78.6	65.1	69.0	89.1	90.6	92.8	93.4	88.2	90.2	74.9	79.7	82.7	85.7	68.3	74.4
Niger	70 to 74	49.9	49.2	66.6	64.4	39.9	40.5	48.2	47.7	59.7	58.8	54.5	56.9	83.2	85.1	90.7	90.8	82.0	84.7	66.9	65.0	80.7	79.0	52.4	48.0
Nigeria	70 to 74	45.7	61.3	62.6	74.8	35.4	51.3	48.8	63.0	59.6	70.5	57.7	69.5	83.2	91.2	90.1	94.2	82.2	90.7	58.3	66.2	76.0	81.4	37.0	47.1
Niue	70 to 74	55.4	61.0	70.8	75.4	44.3	49.3	48.8	51.0	67.6	69.8	47.1	47.8	78.1	83.5	83.7	87.6	83.5	88.5	73.8	76.2	81.1	83.5	67.5	69.3
North Macedonia	70 to 74	46.0	52.3	55.0	62.8	45.9	49.4	37.8	44.3	52.4	58.8	45.9	49.5	60.5	67.4	73.3	77.1	63.7	70.8	47.0	52.5	54.4	61.4	46.7	49.4
Northern Mariana Islands	70 to 74	56.9	65.0	66.7	76.7	53.5	58.7	54.9	59.6	66.2	71.9	58.9	60.8	76.8	83.2	77.8	84.6	88.9	89.1	73.8	79.1	78.3	84.8	71.8	75.3
Norway	70 to 74	77.2	89.2	83.4	92.9	73.5	85.8	74.6	83.8	83.2	87.4	73.2	84.6	83.6	93.2	88.3	96.0	86.0	91.1	77.7	90.9	81.7	92.4	75.3	89.1
Oman	70 to 74	45.2	65.8	59.3	74.3	39.6	62.0	44.0	64.1	64.5	75.1	41.9	64.2	76.9	86.3	87.0	91.2	73.7	84.6	46.4	65.1	59.1	72.2	39.5	61.5
Pakistan	70 to 74	53.6	54.2	59.6	62.1	53.8	53.7	62.9	61.8	71.7	71.0	66.7	66.6	70.0	71.2	80.0	79.8	71.7	73.9	61.4	59.9	64.0	66.1	62.5	58.4
Palau	70 to 74	55.3	64.1	72.9	74.7	43.1	58.3	52.8	57.4	71.1	70.4	50.4	59.0	81.8	85.0	85.8	86.6	86.5	92.8	70.7	75.5	80.1	80.7	63.1	72.6
Palestine	70 to 74	29.5	51.5	43.9	62.0	27.2	48.9	43.4	51.6	61.1	66.5	45.8	53.6	84.4	89.5	90.3	93.4	82.9	88.2	22.7	48.1	36.9	57.4	18.6	45.5
Panama	70 to 74	62.6	73.2	74.9	85.5	54.9	63.2	61.7	68.5	74.3	81.2	61.8	65.8	84.4	84.5	89.3	90.3	84.9	83.0	68.3	77.7	77.2	86.3	61.6	69.7
Papua New Guinea	70 to 74	22.1	30.7	32.3	38.1	24.1	32.9	25.4	30.0	40.7	41.5	35.4	40.4	62.9	70.0	67.4	72.7	76.3	81.1	61.9	64.8	61.5	64.3	67.6	68.8
Paraguay	70 to 74	56.6	61.4	68.7	72.3	50.7	56.0	51.3	50.6	66.7	66.8	52.9	51.9	81.7	79.8	87.1	84.3	83.4	85.0	63.7	66.5	73.3	75.1	56.9	60.9
Peru	70 to 74	64.4	79.1	77.1	86.9	55.3	73.1	56.3	64.9	71.2	76.2	56.1	65.5	83.6	87.5	89.2	90.3	83.5	90.4	72.9	83.9	82.1	89.5	64.4	78.1
Philippines	70 to 74	53.3	57.7	68.2	73.8	44.3	44.8	46.9	52.0	63.7	68.6	49.1	51.6	81.4	81.9	88.9	89.0	79.6	79.9	68.9	70.6	78.3	81.3	61.2	59.5
Poland	70 to 74	43.0	71.8	57.6	81.1	36.9	65.6	47.6	61.1	64.7	74.6	49.0	59.9	78.7	80.1	87.3	88.3	75.2	77.5	38.4	72.8	52.7	80.1	30.8	67.3
Portugal	70 to 74	55.2	77.1	67.2	83.7	49.8	73.1	61.1	72.5	73.8	81.2	61.7	72.7	81.0	82.9	87.9	89.6	80.6	81.7	55.5	81.5	65.8	84.8	49.8	79.2
Puerto Rico	70 to 74	72.8	88.7	84.0	98.4	63.7	77.1	69.9	76.0	80.0	85.8	69.2	73.1	89.8	93.1	93.3	95.9	88.6	90.4	75.0	93.0	83.7	99.6	66.9	83.8
Qatar	70 to 74	55.3	72.6	65.6	77.0	51.6	71.8	40.5	67.4	61.2	74.5	40.5	71.2	41.8	78.9	78.5	88.4	24.0	75.3	65.7	76.1	72.1	78.2	62.1	75.6
Republic of Korea	70 to 74	68.1	94.1	76.0	95.9	64.9	92.2	73.8	95.2	82.9	95.6	72.1	94.7	72.0	92.5	81.1	94.4	74.1	93.4	72.9	94.1	78.0	94.4	70.2	92.5
Republic of Moldova	70 to 74	44.6	55.3	57.4	67.9	40.9	48.9	39.3	43.0	57.8	60.3	43.6	46.4	76.9	74.0	88.4	86.3	67.9	67.3	57.3	59.2	65.7	69.9	54.2	51.9
Romania	70 to 74	48.7	63.0	60.1	73.7	45.8	57.0	33.4	42.6	51.6	61.1	40.5	43.9	67.3	65.5	78.9	79.4	66.2	62.0	52.7	66.3	62.1	74.7	49.2	60.6
Russian Federation	70 to 74	40.3	61.5	57.4	74.6	27.4	51.1	50.0	57.7	65.5	71.2	53.5	58.4	74.6	71.8	85.6	84.3	65.6	64.5	33.0	60.3	51.6	72.5	13.7	48.4
Rwanda	70 to 74	32.4	54.5	53.7	70.0	17.1	40.3	15.3	37.2	45.9	61.2	14.3	31.8	78.5	86.9	89.8	93.0	72.8	83.5	62.0	72.8	70.7	79.9	56.8	65.6
Saint Kitts and Nevis	70 to 74	37.4	50.9	55.0	67.6	27.9	39.9	43.1	49.4	62.7	67.0	43.1	49.1	77.4	83.3	85.9	88.6	76.0	84.1	44.2	55.5	58.2	68.7	34.7	44.6
Saint Lucia	70 to 74	41.5	60.8	59.8	75.8	30.1	50.2	44.1	55.6	64.2	73.2	43.5	53.4	79.5	87.2	86.5	92.1	79.2	85.3	47.7	66.0	59.8	74.8	40.4	59.6
Saint Vincent and the Grenadines	70 to 74	41.2	58.6	59.1	72.9	29.9	49.3	29.2	53.9	54.4	70.3	30.1	53.4	85.4	88.0	90.7	93.3	84.0	85.1	51.9	65.2	62.9	73.6	45.4	59.3
Samoa	70 to 74	49.3	57.7	68.6	71.5	36.5	48.7	44.7	48.4	66.5	66.1	41.9	48.7	76.5	81.4	82.8	85.1	80.2	88.2	73.7	77.2	81.7	84.0	67.3	71.2
San Marino	70 to 74	77.0	87.9	81.9	91.3	74.8	85.1	74.5	83.6	80.6	86.6	77.3	85.3	92.6	98.4	94.8	98.6	92.0	96.8	78.1	88.7	81.6	90.6	76.0	86.8
Sao Tome and Principe	70 to 74	67.1	64.3	73.7	75.8	67.1	55.9	61.9	62.2	66.4	68.9	74.3	70.7	91.9	92.2	93.3	94.0	93.5	93.1	76.4	69.4	82.4	81.7	71.2	52.9
Saudi Arabia	70 to 74	35.8	57.9	41.9	62.4	37.6	56.7	38.4	53.5	54.6	65.6	43.8	56.6	79.9	85.2	87.1	91.0	79.8	84.4	38.5	59.3	41.1	60.6	40.6	58.8
Senegal	70 to 74	51.2	52.0	67.9	69.0	40.7	40.0	50.0	51.5	58.6	60.5	59.3	61.0	85.9	86.8	91.3	91.2	85.6	87.3	60.4	59.8	78.6	78.6	40.1	36.2
Serbia	70 to 74	52.1	49.1	62.3	59.9	49.5	46.5	45.9	34.9	60.3	54.1	51.1	39.6	66.2	63.7	77.4	74.3	67.4	68.2	52.5	47.2	61.3	56.7	49.6	44.4
Seychelles	70 to 74	62.0	69.6	74.9	78.6	53.9	64.7	55.2	61.0	68.3	71.1	58.1	64.8	83.2	89.4	90.4	93.5	80.3	87.8	70.1	75.7	80.4	82.3	61.5	70.7
Sierra Leone	70 to 74	50.5	51.3	67.9	65.0	39.9	44.7	51.6	50.7	59.4	57.3	60.4	63.9	86.7	87.6	91.7	91.0	86.3	88.8	58.2	59.0	78.9	75.9	36.9	40.2
Singapore	70 to 74	78.8	100.0	82.2	99.1	78.9	100.0	85.1	99.5	87.8	97.6	87.6	100.0	90.4	98.8	92.4	97.9	94.2	100.0	78.0	99.4	79.3	96.8	79.0	100.0
Slovakia	70 to 74	68.0	76.1	76.1	83.3	64.4	71.5	43.8	56.1	61.1	70.7	46.6	56.2	79.2	82.2	86.9	89.7	78.0	79.3	70.8	77.9	76.7	82.8	67.6	74.2
Slovenia	70 to 74	55.0	78.6	67.2	86.9	48.8	72.5	54.5	67.5	67.9	79.7	57.1	65.1	78.1	85.0	86.4	91.6	76.0	81.9	52.4	81.0	63.8	86.7	45.3	76.3
Solomon Islands	70 to 74	44.2	49.5	60.3	63.9	36.4	41.5	48.6	49.5	65.3	65.7	49.1	50.2	71.3	77.4	77.7	82.6	77.2	82.7	71.8	73.3	76.7	78.3	69.8	71.2
Somalia	70 to 74	33.8	42.6	56.6	62.2	16.6	25.1	7.9	11.0	40.8	44.4	7.5	7.4	75.0	80.2	88.6	90.3	69.8	75.3	68.0	70.8	75.5	78.5	63.1	63.3
South Sudan	70 to 74	77.0	69.4	65.4	67.7	31.5	40.1	64.3	53.0	52.6	51.5	22.8	30.4	93.9	90.1	90.8	91.3	79.9	83.1	82.5	74.8	78.5	79.3	64.0	64.8

Spain	70 to 74	44.0	50.9	78.0	94.0	64.1	88.2	24.0	28.1	79.6	86.3	70.7	78.9	81.6	84.5	92.8	96.3	87.2	87.9	70.2	71.3	77.1	94.1	66.4	92.3
Sri Lanka	70 to 74	69.3	91.1	66.2	75.2	42.2	55.6	70.4	79.6	58.4	64.3	40.8	49.7	88.7	92.1	91.9	93.6	84.0	87.1	70.5	93.8	71.1	77.3	50.4	61.9
Sudan	70 to 74	50.5	63.2	31.1	56.1	33.2	54.1	38.2	47.0	45.3	56.0	33.8	54.5	86.1	89.4	75.9	79.4	58.3	72.6	58.9	68.5	34.1	56.0	41.1	54.2
Suriname	70 to 74	27.2	52.5	54.5	70.0	40.3	44.6	25.7	45.6	59.6	68.3	50.4	52.6	59.0	70.0	87.1	91.0	79.2	80.8	34.8	53.4	61.7	74.6	50.7	55.5
Sweden	70 to 74	42.5	54.9	83.5	92.1	74.6	85.7	44.4	52.4	84.3	90.8	81.6	90.8	79.8	84.2	88.8	94.0	87.7	92.0	53.3	64.2	82.3	91.1	76.1	88.2
Switzerland	70 to 74	77.9	88.7	79.8	93.6	70.0	88.9	79.8	89.6	81.2	89.6	76.1	86.6	85.1	91.9	87.8	94.8	85.0	89.7	78.5	89.8	79.6	93.0	71.9	91.5
Syrian Arab Republic	70 to 74	73.3	91.2	57.1	63.7	44.1	52.6	74.5	86.5	63.3	66.0	52.9	56.3	83.0	91.6	87.2	90.1	75.8	82.0	74.8	92.7	58.4	63.5	47.7	55.3
Taiwan (Province of China)	70 to 74	46.5	54.7	70.2	92.2	57.6	76.3	49.3	52.9	68.2	82.9	57.1	74.4	77.6	83.2	85.8	93.0	79.1	81.9	50.3	57.0	78.1	94.1	70.5	84.3
Tajikistan	70 to 74	61.3	83.7	62.2	65.0	37.9	47.5	54.8	74.9	60.0	61.1	54.7	59.1	78.5	86.7	85.4	85.0	79.2	81.3	73.1	89.0	67.4	69.1	38.1	50.6
Thailand	70 to 74	47.1	53.0	71.8	83.5	52.4	67.0	46.8	52.0	68.4	75.5	60.5	67.4	78.3	79.4	78.6	86.1	58.7	73.6	52.9	58.2	80.3	88.0	66.3	77.0
Timor-Leste	70 to 74	59.5	74.0	44.3	55.0	37.5	38.9	56.7	65.6	43.8	50.5	44.3	45.4	62.7	75.9	76.2	80.5	71.4	74.3	72.5	82.3	65.3	70.2	58.2	54.0
Togo	70 to 74	34.8	42.1	65.1	65.6	42.6	35.3	30.6	35.6	56.7	57.3	60.5	57.2	66.4	71.4	90.6	90.6	85.9	85.7	59.0	60.3	77.7	77.1	42.1	31.1
Tokelau	70 to 74	50.4	48.3	61.2	73.8	24.8	47.2	49.0	45.6	63.5	66.6	36.3	45.9	85.7	85.5	78.8	86.8	77.5	87.9	60.7	58.2	74.7	83.3	54.9	68.8
Tonga	70 to 74	40.9	58.9	76.9	78.2	53.7	58.7	40.7	47.3	72.7	73.1	57.3	59.6	71.3	82.9	88.5	89.3	87.8	91.4	64.4	75.9	85.5	85.0	69.3	72.3
Trinidad and Tobago	70 to 74	63.1	66.7	65.1	75.3	44.0	55.1	58.0	59.6	66.0	71.9	53.5	58.7	84.9	86.8	86.9	87.3	77.0	78.4	77.0	78.3	66.7	76.4	41.7	56.1
Tropical Latin America	70 to 74	51.0	62.7	65.8	79.6	44.4	62.9	51.0	58.2	68.4	74.3	54.8	62.1	78.6	79.6	89.0	85.5	86.7	80.1	52.3	65.0	65.9	80.6	43.6	65.8
Tunisia	70 to 74	50.9	64.0	59.4	72.0	49.4	61.1	51.7	61.0	65.2	71.4	55.3	64.6	76.8	82.6	86.5	89.2	75.2	81.6	51.8	63.6	58.1	70.3	50.3	60.6
Turkey	70 to 74	55.8	70.6	68.2	79.3	49.8	65.3	52.6	68.0	68.5	78.1	53.1	67.8	61.0	76.1	79.1	86.9	54.2	71.5	61.6	73.9	71.2	80.0	55.4	69.5
Turkmenistan	70 to 74	56.4	49.8	68.7	67.7	48.7	35.6	54.0	41.3	65.8	60.1	60.0	42.8	73.2	59.6	83.4	77.3	70.5	55.0	59.3	53.9	70.1	69.2	49.9	38.5
Tuvalu	70 to 74	33.4	50.7	53.5	66.0	20.8	40.7	33.9	44.1	56.5	63.3	34.9	44.4	65.8	77.5	74.8	82.6	73.8	84.6	62.3	69.7	72.3	77.6	54.7	63.5
Uganda	70 to 74	53.9	64.1	72.9	77.9	37.9	50.4	29.7	42.6	58.8	64.5	24.4	38.8	82.4	88.2	92.3	93.9	78.1	84.8	75.7	78.7	82.4	84.5	69.5	72.0
Ukraine	70 to 74	55.8	64.8	67.1	75.6	50.7	57.4	57.7	60.2	70.3	72.7	60.5	60.7	65.9	72.3	79.5	84.6	58.7	66.4	52.6	63.9	63.3	73.6	46.7	56.1
United Arab Emirates	70 to 74	61.5	58.4	64.2	0.0	63.0	77.7	54.8	43.4	64.5	0.0	60.8	71.0	52.8	35.5	65.8	0.0	61.2	77.7	64.4	60.9	65.1	0.0	66.1	81.0
United Kingdom	70 to 74	72.2	89.1	79.9	92.7	68.0	86.0	73.9	79.3	80.7	84.5	75.9	80.3	81.0	86.8	86.7	91.2	83.3	86.4	71.2	92.3	77.8	93.8	66.9	90.2
United Republic of Tanzania	70 to 74	54.3	64.2	70.4	75.3	43.1	56.9	35.8	47.2	58.8	63.5	35.0	49.7	84.6	88.6	92.4	92.9	81.8	87.5	75.9	74.9	81.8	82.1	71.2	68.2
United States Virgin Islands	70 to 74	55.5	75.2	70.9	87.4	44.0	65.2	49.8	67.5	68.3	80.0	47.8	65.1	81.1	89.8	89.1	95.3	76.9	84.4	61.8	80.2	72.3	87.8	53.1	73.1
United States of America	70 to 74	83.3	97.3	89.3	99.6	78.3	94.1	76.9	85.6	83.4	89.7	77.4	85.7	84.2	86.6	90.3	92.8	81.8	82.5	85.2	100.0	89.1	100.0	81.5	98.0
Uruguay	70 to 74	72.2	79.1	80.5	86.8	67.1	73.0	71.1	76.5	81.9	85.8	68.0	72.3	76.5	79.8	85.1	86.7	75.5	80.3	74.1	81.7	80.3	87.1	69.6	76.6
Uzbekistan	70 to 74	58.3	63.0	71.3	74.9	48.0	55.2	46.7	59.2	61.6	68.9	52.4	62.8	84.8	59.9	91.5	78.9	80.0	54.6	67.9	68.7	78.0	78.3	55.6	60.4
Vanuatu	70 to 74	47.6	55.2	66.5	71.0	36.1	44.8	42.4	46.0	64.0	65.4	40.8	45.2	74.3	79.8	80.7	84.4	78.9	84.7	70.4	74.6	79.8	82.6	63.0	67.8
Venezuela (Bolivarian Republic of)	70 to 74	61.7	65.7	74.6	79.7	52.7	54.2	57.2	59.6	71.9	75.1	56.6	56.1	88.5	86.1	92.6	91.3	87.0	84.2	70.2	72.1	79.2	82.2	62.2	62.1
Viet Nam	70 to 74	34.7	45.9	54.9	66.0	23.1	33.6	36.0	40.2	56.6	62.0	38.6	39.3	70.1	77.7	83.0	87.4	65.3	73.8	53.9	58.4	68.5	74.1	41.8	45.9
Yemen	70 to 74	13.3	30.4	28.9	43.3	13.4	29.0	16.3	27.1	45.8	46.3	17.1	35.7	52.9	60.3	75.8	77.2	42.0	57.3	21.3	30.7	31.5	43.3	23.0	26.4
Zambia	70 to 74	46.7	53.1	63.3	68.7	37.1	41.2	26.9	34.0	46.0	52.8	33.4	39.3	81.3	84.1	89.2	90.1	80.5	83.5	72.9	70.7	78.9	78.5	69.1	63.3
Zimbabwe	70 to 74	60.1	51.9	73.5	67.4	50.1	37.8	31.7	5.1	50.0	33.2	39.8	20.7	77.4	73.0	86.3	83.4	76.9	72.6	72.2	65.5	80.7	76.2	63.3	50.8
Afghanistan	75 to 79	22.6	33.9	33.7	40.8	7.3	24.3	18.5	24.8	51.3	49.7	10.0	23.9	35.7	51.1	73.9	74.9	19.9	40.7	35.6	38.2	42.2	46.2	25.0	28.7
Albania	75 to 79	31.7	49.7	44.5	58.3	17.0	39.0	19.0	37.1	44.8	56.2	19.6	37.9	62.6	73.3	75.9	82.4	61.4	72.0	63.9	72.6	72.8	78.4	53.2	65.5
Algeria	75 to 79	67.6	75.2	74.8	79.7	57.1	68.2	60.3	61.9	73.2	71.7	58.3	64.1	75.5	79.3	87.3	88.1	69.5	75.4	69.0	75.2	76.7	80.3	58.9	68.7
American Samoa	75 to 79	67.5	69.3	74.2	75.9	58.2	60.2	55.0	55.3	68.4	68.8	55.1	55.2	78.1	79.4	83.0	84.3	82.8	83.4	80.1	81.0	83.8	84.5	75.3	76.7
Andorra	75 to 79	81.9	88.7	83.6	89.3	77.6	85.7	79.0	82.6	84.1	86.4	79.1	82.7	83.2	88.5	88.5	91.9	83.9	89.3	81.2	89.0	83.1	89.3	77.8	87.3
Angola	75 to 79	62.6	66.8	74.3	76.6	43.5	49.5	45.1	48.0	64.9	66.1	40.9	45.3	81.7	84.2	91.7	92.1	75.6	79.1	74.8	74.8	82.7	82.4	61.9	61.7
Antigua and Barbuda	75 to 79	54.7	64.9	67.4	77.4	36.9	46.2	50.2	54.0	67.3	73.9	48.0	46.1	75.9	81.8	87.4	90.8	69.0	75.8	62.8	71.8	72.4	80.3	50.3	59.4
Argentina	75 to 79	69.1	83.2	76.4	88.2	58.6	74.6	68.0	75.9	80.5	86.0	62.1	69.9	66.7	81.8	78.2	89.2	66.1	79.0	72.8	86.3	79.0	89.8	65.0	80.6
Armenia	75 to 79	61.2	72.5	69.9	78.2	49.3	64.6	48.9	62.3	64.4	73.9	50.7	61.5	65.7	74.8	80.3	86.5	58.2	67.5	63.9	71.5	73.2	77.7	51.9	64.5
Australia	75 to 79	78.6	92.0	82.6	93.4	72.2	87.5	72.5	80.5	80.7	86.2	71.8	79.0	76.1	86.1	85.3	93.2	74.8	80.3	78.9	93.5	83.1	94.0	73.4	90.8
Austria	75 to 79	75.3	94.7	79.9	96.3	68.6	90.1	82.7	87.7	87.2	90.9	82.1	86.2	91.6	96.2	95.2	99.6	90.2	90.8	71.0	94.2	76.5	95.2	64.9	91.0
Azerbaijan	75 to 79	57.6	66.2	68.4	73.1	41.2	57.0	43.8	52.9	61.4	66.0	45.1	55.3	83.8	84.8	91.6	92.0	77.4	80.0	71.7	76.2	80.2	81.9	58.2	68.7
Bahamas	75 to 79	64.4	72.3	72.6	78.0	53.2	64.0	55.2	63.3	69.9	75.1	54.2	61.9	84.6	85.6	91.9	92.9	79.2	79.4	71.5	76.1	77.5	80.1	64.7	72.2

Bahrain	75 to 79	25.2	50.9	42.1	60.8	5.5	38.8	27.2	47.7	53.6	66.2	23.7	44.8	43.8	66.7	67.9	80.4	36.1	62.3	31.1	52.6	48.7	62.8	10.6	41.2
Bangladesh	75 to 79	41.2	57.3	52.8	64.8	26.5	47.2	50.2	61.3	68.6	74.3	46.2	59.1	48.0	66.8	73.1	79.9	41.9	64.2	54.0	63.9	61.4	70.1	45.0	56.9
Barbados	75 to 79	61.1	67.6	69.1	76.2	50.8	55.0	58.1	58.9	71.9	75.2	56.5	52.8	80.5	81.3	89.5	89.6	75.1	77.1	62.4	70.2	69.6	76.8	55.0	62.4
Belarus	75 to 79	63.3	70.7	70.0	77.6	56.4	60.3	59.3	59.9	71.8	72.6	60.5	59.2	73.4	72.5	85.9	86.3	57.7	56.3	60.2	69.2	68.5	77.0	53.4	59.0
Belgium	75 to 79	70.8	86.5	76.8	89.6	62.1	80.3	69.1	75.6	79.3	84.0	67.2	72.8	81.3	80.2	88.4	90.0	80.2	74.4	70.7	88.6	77.2	90.7	62.9	84.5
Belize	75 to 79	59.6	68.8	69.9	76.4	45.6	58.1	51.6	58.8	67.8	72.7	49.9	56.7	84.3	87.3	92.9	93.6	77.2	82.9	69.4	75.2	76.7	80.6	60.0	68.1
Benin	75 to 79	57.6	60.5	64.6	68.1	49.4	51.5	54.4	58.0	59.5	64.5	64.3	67.5	80.8	83.7	86.4	88.7	81.2	84.0	67.2	67.5	77.7	77.8	52.4	51.4
Bermuda	75 to 79	69.5	81.4	78.9	91.1	54.1	67.4	66.3	75.7	79.2	87.3	60.5	69.1	85.3	90.4	93.9	99.3	75.5	79.1	69.2	80.6	78.4	89.0	55.1	68.7
Bhutan	75 to 79	54.5	64.2	60.2	69.7	48.8	56.4	59.2	66.3	71.2	75.7	60.3	66.7	63.9	72.0	77.7	82.1	61.1	70.4	61.3	67.4	66.2	73.2	58.5	60.6
Bolivia (Plurinational State of)	75 to 79	50.4	62.3	59.7	69.5	39.8	53.3	40.7	50.1	58.4	65.2	43.4	51.5	65.3	68.6	76.0	77.4	68.7	73.5	60.5	69.5	69.1	76.2	51.0	61.5
Bosnia and Herzegovina	75 to 79	52.2	64.2	59.6	70.7	45.4	55.9	29.7	45.9	51.9	62.8	31.6	45.9	51.3	67.0	68.6	78.6	48.7	65.0	50.9	62.4	59.5	70.0	44.9	54.5
Botswana	75 to 79	66.6	75.9	75.5	82.3	52.3	64.4	47.7	58.5	64.5	71.1	48.3	59.7	84.6	89.5	92.1	94.7	79.7	85.0	74.8	79.7	82.1	85.5	63.0	69.4
Brazil	75 to 79	58.5	74.3	68.2	80.8	45.6	64.8	52.8	62.1	69.6	75.5	49.4	58.7	83.4	76.5	88.9	85.0	83.5	75.3	60.0	76.0	70.1	82.3	47.3	67.5
Brunei Darussalam	75 to 79	81.1	82.5	83.6	85.3	76.5	77.3	77.9	78.8	85.4	85.5	75.8	77.9	78.6	82.5	85.7	88.3	81.2	84.0	83.8	84.0	85.7	86.7	80.3	79.6
Bulgaria	75 to 79	46.5	58.9	54.5	68.9	36.8	44.8	34.7	48.6	56.6	66.0	32.9	45.7	70.5	56.8	82.3	74.4	66.5	48.9	52.5	58.0	61.1	69.3	43.5	43.4
Burkina Faso	75 to 79	62.7	62.5	74.6	73.2	45.6	47.4	54.9	57.4	67.2	68.2	56.8	60.3	86.9	87.1	95.1	94.0	80.9	82.7	74.7	72.3	86.0	83.3	55.7	54.2
Burundi	75 to 79	52.6	62.2	66.6	72.5	31.9	47.7	32.4	44.5	59.0	65.1	24.3	39.6	75.3	82.1	89.6	92.1	67.3	76.5	69.4	74.3	76.6	80.2	61.0	66.9
Cabo Verde	75 to 79	71.6	69.7	77.3	79.2	64.2	54.1	68.5	68.8	73.7	76.3	74.4	71.7	92.7	94.5	96.7	98.1	89.0	90.6	77.1	71.8	84.7	83.3	65.0	49.7
Cambodia	75 to 79	43.0	51.5	51.9	61.3	35.0	39.7	32.3	37.0	50.1	56.3	40.1	39.5	61.5	69.0	75.6	80.5	59.2	67.0	62.5	65.5	70.0	73.6	55.2	55.9
Cameroon	75 to 79	61.2	61.9	70.1	70.7	49.4	50.1	56.8	58.6	66.4	67.2	61.3	64.4	83.2	85.3	89.8	90.8	81.4	83.8	72.1	69.5	81.5	80.4	57.6	52.7
Canada	75 to 79	84.8	98.8	88.4	100.0	78.2	94.6	82.6	90.2	88.3	93.2	80.3	87.9	85.1	89.1	92.1	95.2	79.9	82.7	84.1	100.0	87.5	100.0	78.8	97.4
Central African Republic	75 to 79	53.1	54.2	66.7	67.2	29.5	29.8	37.6	36.9	60.2	60.0	32.6	31.1	76.5	77.0	88.1	88.1	69.9	70.4	67.9	68.0	77.1	77.2	53.0	51.6
Chad	75 to 79	61.9	59.2	69.9	68.7	51.4	46.6	57.3	57.3	64.8	64.4	63.7	62.6	83.7	83.0	90.0	88.5	81.9	82.1	70.6	66.1	80.7	79.0	55.7	48.1
Chile	75 to 79	68.5	83.5	76.5	88.9	56.7	74.7	71.7	82.7	82.9	89.8	65.1	77.8	80.3	80.2	87.8	88.8	78.4	75.8	68.6	84.2	76.7	89.5	58.2	76.6
China	75 to 79	49.4	70.9	59.0	79.1	37.4	59.6	48.2	61.4	63.9	72.6	48.4	60.7	0.0	64.9	32.6	79.6	2.8	59.9	67.2	76.6	75.0	84.1	57.8	67.1
Colombia	75 to 79	70.2	82.0	77.1	88.1	60.4	71.6	61.5	73.5	73.1	82.9	61.5	70.0	84.2	81.5	90.7	88.9	81.5	78.8	73.4	84.1	80.2	89.5	64.3	75.3
Comoros	75 to 79	63.4	69.8	73.2	77.5	49.4	58.3	42.7	48.7	60.4	64.1	43.3	50.8	83.6	87.1	92.1	93.4	79.0	83.1	75.9	78.5	82.5	84.3	66.6	69.9
Congo	75 to 79	61.6	66.7	71.7	75.1	46.2	53.8	40.7	46.1	58.7	61.9	43.1	49.5	81.2	84.5	89.7	91.1	77.2	81.8	73.1	74.3	80.9	81.4	60.8	63.5
Cook Islands	75 to 79	65.9	79.4	75.5	85.6	52.9	69.8	52.3	66.3	69.7	79.3	48.6	61.8	77.8	89.4	84.6	92.8	79.1	89.7	78.6	86.2	84.1	89.4	71.4	81.1
Costa Rica	75 to 79	75.0	84.4	81.6	90.6	65.1	74.4	68.7	77.4	78.9	85.5	66.8	74.2	88.2	86.8	93.4	92.9	84.8	82.8	76.4	85.0	82.7	90.5	67.7	76.6
Coted'Ivoire	75 to 79	64.8	64.8	72.9	73.2	53.7	53.1	60.7	62.2	67.3	68.9	65.2	67.5	85.7	87.0	90.9	91.2	84.1	86.3	70.8	69.2	81.3	80.4	56.3	52.6
Croatia	75 to 79	42.7	76.5	53.0	80.5	32.0	70.1	24.2	49.4	52.6	67.6	17.3	45.1	39.4	57.4	57.7	74.4	45.5	51.3	42.4	77.4	54.1	81.2	32.3	72.8
Cuba	75 to 79	64.7	70.7	73.2	78.8	53.0	58.7	60.1	63.4	73.3	76.4	58.4	60.1	86.7	87.6	93.0	93.2	82.4	83.9	67.4	71.4	75.4	79.3	57.4	60.8
Cyprus	75 to 79	70.5	77.5	75.1	82.1	64.1	70.2	71.6	79.6	80.0	85.4	70.9	78.4	70.8	84.8	80.2	90.7	71.6	83.0	71.7	77.7	76.5	81.9	67.2	72.0
Czechia	75 to 79	56.8	83.7	65.8	87.0	45.2	77.7	45.2	67.4	63.8	77.6	43.2	65.8	62.1	75.7	76.2	86.7	58.7	69.1	53.9	83.9	64.7	87.0	41.5	79.4
Democratic People's Republic of Korea	75 to 79	59.3	63.7	68.3	72.5	47.4	51.0	44.0	42.3	63.1	61.6	41.9	41.0	48.9	61.9	68.4	77.6	38.5	51.3	74.0	75.6	80.5	82.5	65.8	65.6
Democratic Republic of the Congo	75 to 79	61.8	61.7	72.4	72.5	46.3	42.4	48.5	45.9	65.1	65.3	47.1	41.5	82.6	82.6	91.6	91.4	77.9	77.0	72.3	72.3	80.0	80.2	61.4	59.0
Denmark	75 to 79	78.8	86.8	83.5	90.6	71.3	79.9	71.8	77.4	80.6	85.1	70.7	75.0	81.3	85.3	88.4	92.1	80.5	81.3	79.3	88.4	84.1	91.3	72.9	83.3
Djibouti	75 to 79	66.1	71.1	76.4	80.0	50.4	57.6	44.6	49.5	63.5	65.4	42.1	48.8	83.3	86.5	92.9	93.7	77.3	82.0	78.7	78.7	85.0	85.7	69.1	68.0
Dominica	75 to 79	45.6	54.2	61.5	64.7	20.8	40.5	36.2	45.9	60.7	64.2	29.6	44.4	74.2	77.6	87.5	88.3	63.4	71.7	54.4	59.0	67.2	67.9	36.3	49.4
Dominican Republic	75 to 79	68.7	71.5	77.1	79.6	56.8	59.8	57.3	62.6	71.6	75.1	55.5	60.4	85.9	87.7	93.2	93.4	80.5	83.5	76.9	76.3	83.1	82.8	68.4	67.2
Ecuador	75 to 79	72.6	82.3	80.7	88.5	60.7	72.5	58.0	68.5	72.3	79.8	55.9	65.1	91.2	79.9	95.3	87.8	87.7	77.7	76.0	86.2	83.2	91.2	65.8	78.3
Egypt	75 to 79	0.0	40.1	0.0	43.1	0.0	35.5	0.0	42.0	24.9	59.0	8.9	43.6	36.9	64.5	68.7	80.9	24.0	57.0	0.0	35.1	0.0	39.7	2.9	30.7
El Salvador	75 to 79	68.2	79.0	77.2	85.4	55.3	68.5	60.1	69.3	72.8	79.2	59.1	67.4	84.4	87.7	90.9	92.7	81.5	85.2	74.4	81.9	83.1	87.8	61.9	72.7
Equatorial Guinea	75 to 79	58.5	70.8	71.2	78.6	36.6	57.1	40.0	57.3	61.4	70.2	36.0	58.1	79.4	89.6	90.5	94.6	72.1	85.8	72.4	75.5	81.1	82.4	57.1	64.0
Eritrea	75 to 79	56.0	61.8	67.2	72.2	38.2	44.5	36.0	38.6	58.5	60.7	32.4	34.7	79.1	83.1	90.3	91.9	70.1	76.3	74.2	75.8	80.3	82.3	67.3	65.7
Estonia	75 to 79	49.1	82.8	59.0	87.7	38.2	73.9	53.6	66.9	68.1	77.7	55.2	64.7	76.5	74.9	88.4	88.4	59.9	58.4	43.4	82.7	56.3	87.4	31.0	75.1
Eswatini	75 to 79	62.6	64.6	73.5	73.2	42.8	51.0	42.0	42.7	62.5	61.1	39.0	46.0	82.0	83.1	91.0	90.4	75.5	79.5	73.6	72.6	81.4	79.8	59.9	61.6

Ethiopia	75 to 79	57.0	68.6	67.5	76.1	42.9	57.9	43.8	56.2	59.7	69.7	44.9	55.5	82.1	87.1	92.3	93.7	76.5	82.9	77.6	79.4	81.9	83.6	72.9	74.3
Fiji	75 to 79	68.3	69.4	76.4	77.2	56.8	58.0	49.3	45.3	67.3	65.2	46.0	41.2	77.7	78.7	85.0	86.0	77.8	79.8	81.2	81.2	86.4	85.8	74.3	74.8
Finland	75 to 79	78.9	90.8	82.4	93.1	74.0	85.6	78.3	86.5	84.2	89.7	78.4	85.6	85.9	92.3	91.2	95.9	86.6	89.6	77.2	90.4	81.3	92.4	73.1	86.4
France	75 to 79	77.2	94.3	81.2	95.5	70.9	90.2	69.7	82.8	79.8	87.4	67.9	81.8	82.6	89.7	89.1	95.8	81.8	84.1	78.1	95.6	82.1	95.8	72.8	93.3
Gabon	75 to 79	67.6	71.1	78.1	79.7	49.4	55.9	47.9	50.9	66.7	66.7	43.8	50.4	85.4	88.2	93.8	94.6	78.8	83.0	77.8	77.3	84.5	83.8	66.4	66.3
Gambia	75 to 79	63.2	60.0	71.8	69.5	51.8	47.4	56.3	54.8	63.5	63.1	64.3	63.6	85.5	84.3	91.0	89.3	84.0	84.4	70.9	66.2	81.6	78.2	54.2	46.7
Georgia	75 to 79	48.2	54.3	61.3	66.9	27.4	34.4	43.2	49.8	61.3	66.5	43.9	47.8	71.7	60.9	83.4	75.2	65.5	57.7	60.1	57.3	71.5	70.3	42.1	37.3
Germany	75 to 79	76.9	92.3	82.4	94.0	68.4	87.8	71.5	84.2	81.1	89.0	69.1	82.4	82.5	87.8	89.6	93.8	80.4	83.8	75.8	92.2	81.7	93.3	67.9	89.3
Ghana	75 to 79	63.0	66.1	71.1	73.9	52.8	55.4	51.4	53.8	58.2	61.8	64.0	65.8	86.7	87.5	91.3	91.0	86.0	88.6	71.1	71.8	81.4	81.2	54.0	55.4
Greece	75 to 79	62.4	81.9	68.1	84.9	55.8	76.4	61.5	72.8	73.0	80.6	62.7	72.3	79.7	86.4	87.7	93.0	78.3	82.5	64.9	85.0	71.2	87.0	58.8	81.8
Greenland	75 to 79	73.6	82.1	77.3	86.5	69.3	74.8	64.4	71.8	74.7	80.8	66.2	70.1	41.7	71.4	60.4	82.5	50.5	68.3	77.9	87.0	81.4	89.8	74.6	82.2
Grenada	75 to 79	52.5	56.6	66.8	72.6	30.5	31.7	50.9	44.8	70.3	70.0	43.7	32.7	77.7	75.9	90.3	89.0	65.9	64.8	54.2	61.5	67.6	74.6	35.3	41.7
Guam	75 to 79	76.8	87.0	83.1	91.5	66.6	78.8	65.7	74.5	77.5	83.4	62.5	71.0	82.1	92.7	87.7	95.2	83.3	92.4	81.9	91.0	86.8	93.9	74.6	85.5
Guatemala	75 to 79	69.3	74.9	77.1	82.4	57.7	63.7	59.0	65.1	72.0	76.2	58.0	63.6	95.7	91.4	99.0	96.4	90.7	86.5	74.7	81.1	81.2	86.9	65.9	72.5
Guinea	75 to 79	61.6	61.2	67.4	68.2	54.8	52.3	56.5	58.7	61.7	63.2	66.1	66.9	85.5	85.9	89.9	89.4	84.9	85.8	70.8	67.5	79.7	78.4	58.4	52.2
Guinea-Bissau	75 to 79	51.4	52.7	63.7	63.5	35.7	39.4	47.3	45.9	56.1	56.9	55.1	58.1	78.3	78.7	87.2	85.5	76.2	79.8	61.9	62.2	77.7	75.8	38.9	39.2
Guyana	75 to 79	30.5	52.0	45.3	61.4	12.6	40.4	37.7	48.4	60.6	64.9	33.2	48.3	74.6	79.6	86.0	89.5	69.4	73.0	40.7	56.9	54.8	66.0	24.0	47.1
Haiti	75 to 79	21.7	31.0	32.2	39.5	13.1	23.6	30.6	34.5	53.2	54.9	31.0	35.7	53.8	59.5	70.9	73.6	52.8	59.8	34.6	40.7	41.7	47.0	33.5	38.3
Honduras	75 to 79	56.6	45.3	67.1	56.2	43.2	32.7	44.7	33.4	62.1	53.1	45.4	37.7	75.0	58.7	84.7	70.5	73.1	64.2	65.2	56.0	75.7	67.7	51.2	41.1
Hungary	75 to 79	62.8	77.9	70.9	83.2	52.1	69.2	42.5	61.5	60.8	74.2	42.7	59.2	76.7	77.4	86.4	87.4	71.4	71.0	63.8	77.6	72.5	83.0	53.3	70.0
Iceland	75 to 79	80.4	91.4	84.9	94.2	72.9	85.6	77.7	83.4	84.9	89.6	75.2	80.7	84.9	88.5	90.7	93.5	83.4	85.7	80.1	92.0	84.8	94.0	73.5	87.8
India	75 to 79	54.0	58.3	61.2	64.9	44.0	48.9	61.4	64.4	73.9	75.2	59.4	63.5	61.1	69.0	78.1	80.5	56.2	67.2	60.5	62.7	66.2	69.0	53.9	55.3
Indonesia	75 to 79	59.3	59.6	66.2	68.4	51.4	47.9	47.3	45.9	61.6	61.7	51.3	48.1	69.8	70.0	80.3	80.5	69.8	69.8	72.2	69.3	78.1	77.4	65.3	58.6
Iran (Islamic Republic of)	75 to 79	51.7	68.4	60.0	72.1	41.5	62.6	55.3	66.4	69.4	75.6	54.9	67.0	67.4	79.8	81.6	88.3	61.1	75.9	47.0	65.4	57.3	70.0	36.1	60.2
Iraq	75 to 79	43.0	53.0	54.9	64.7	28.1	37.7	41.2	48.8	62.2	67.2	37.8	45.2	72.7	76.0	87.1	87.9	62.1	68.6	43.4	52.1	56.3	65.3	28.4	35.9
Ireland	75 to 79	73.7	86.2	78.5	90.1	66.6	79.1	77.2	83.5	83.7	88.1	76.7	82.0	79.1	83.9	87.3	91.4	77.4	79.4	71.2	86.7	76.9	90.5	64.4	80.6
Israel	75 to 79	80.5	93.6	82.3	94.9	76.6	89.4	66.5	76.8	76.4	83.9	66.7	75.2	90.8	90.7	95.1	97.8	87.7	83.0	83.8	97.0	85.0	96.4	81.7	95.6
Italy	75 to 79	66.1	81.8	72.6	85.3	57.1	75.4	66.3	75.0	78.0	82.9	63.5	73.2	82.6	83.3	89.2	92.0	81.1	76.9	64.6	84.2	71.9	86.5	56.1	80.2
Jamaica	75 to 79	50.8	57.8	62.3	67.2	35.9	45.3	50.7	52.9	68.0	69.0	48.0	50.8	83.0	81.2	90.1	90.3	80.1	75.4	52.9	62.2	62.9	69.9	42.2	53.1
Japan	75 to 79	79.8	96.3	84.8	99.1	71.7	90.3	86.0	98.1	90.9	100.0	82.9	94.0	84.6	96.6	90.6	98.4	84.3	95.6	77.4	93.9	82.8	95.8	70.1	89.5
Jordan	75 to 79	60.1	79.6	63.0	81.7	58.2	75.3	53.2	65.5	64.9	74.7	59.0	66.4	88.3	90.9	94.0	96.0	83.8	86.1	59.0	79.0	63.2	81.5	57.4	75.8
Kazakhstan	75 to 79	63.5	66.0	72.9	75.7	46.9	48.5	51.7	49.8	66.9	66.5	53.0	48.4	64.5	57.7	78.4	73.8	58.1	52.8	64.8	69.5	75.1	79.2	46.7	51.9
Kenya	75 to 79	70.1	69.5	77.5	77.8	59.4	56.6	53.7	52.1	67.3	66.9	54.0	52.2	86.5	86.6	93.7	93.5	81.8	81.9	80.9	78.7	84.9	84.2	75.7	70.7
Kiribati	75 to 79	63.3	63.5	74.5	73.8	45.9	48.0	51.2	48.5	68.5	65.8	47.5	46.5	79.1	80.6	88.0	87.9	73.4	78.7	77.9	76.5	85.2	84.1	66.6	65.0
Kuwait	75 to 79	80.9	84.7	81.2	89.7	77.9	76.7	72.0	76.8	78.6	85.8	72.9	73.4	84.6	92.4	90.7	98.5	82.1	85.9	80.3	84.0	80.5	88.7	78.6	77.2
Kyrgyzstan	75 to 79	54.6	65.9	66.4	76.5	35.6	48.1	43.1	58.8	60.7	71.8	45.6	57.3	67.7	61.1	82.6	79.6	56.9	51.7	57.7	66.1	70.2	77.7	37.4	46.7
Lao People's Democratic Republic	75 to 79	42.1	56.1	51.4	64.3	31.6	45.8	32.7	41.9	50.8	57.4	36.7	45.2	55.7	71.2	71.6	81.6	54.8	70.2	57.4	66.9	65.3	74.9	49.2	57.1
Latvia	75 to 79	51.1	72.4	60.9	77.6	39.8	65.3	45.7	61.4	63.6	73.6	45.3	60.6	72.0	72.9	85.3	86.4	54.6	57.2	49.3	70.4	60.9	76.5	37.5	64.0
Lebanon	75 to 79	52.0	79.6	61.7	83.8	39.6	72.4	36.0	65.1	63.4	77.8	23.8	61.0	50.0	78.1	75.4	89.9	32.9	68.8	59.6	80.9	66.4	84.7	52.2	75.4
Lesotho	75 to 79	60.4	56.5	72.7	67.9	32.8	33.7	46.5	36.8	67.1	59.0	37.3	34.9	80.5	78.5	91.3	88.2	69.7	71.5	72.5	67.5	80.9	76.5	54.9	50.7
Liberia	75 to 79	60.3	58.3	66.4	65.7	52.4	50.2	56.5	53.5	59.7	59.9	65.4	65.2	85.6	85.1	89.7	89.2	84.7	85.2	68.1	65.8	78.2	76.6	54.8	50.2
Libya	75 to 79	59.6	63.9	65.7	70.2	52.2	55.7	53.0	55.2	66.5	66.5	55.0	59.3	77.1	77.3	87.1	85.9	72.4	75.3	60.6	62.8	67.4	70.7	53.7	53.6
Lithuania	75 to 79	63.5	73.4	70.5	79.1	54.5	64.6	59.7	66.9	71.0	77.0	63.7	66.4	76.2	73.7	87.4	86.8	63.6	58.7	60.3	71.9	69.4	78.4	50.2	63.5
Luxembourg	75 to 79	61.5	84.5	71.0	89.3	48.1	76.2	63.0	75.5	75.9	85.1	60.4	71.2	79.0	86.4	86.8	93.5	77.9	81.3	60.5	86.5	71.4	90.1	46.3	80.5
Madagascar	75 to 79	56.9	61.0	70.1	72.0	38.3	44.3	36.5	39.1	58.8	60.4	33.2	36.5	77.5	81.3	90.3	90.5	71.4	76.8	73.8	74.9	81.8	82.1	62.3	63.9
Malawi	75 to 79	61.6	61.3	70.3	70.9	50.0	47.9	41.2	39.3	59.8	59.7	42.1	38.4	80.9	81.9	89.6	90.0	77.1	78.2	74.4	71.9	80.8	79.8	65.8	60.4
Malaysia	75 to 79	64.8	72.8	70.2	78.4	57.9	64.5	54.6	59.6	66.0	70.4	57.2	60.4	73.1	78.8	83.0	86.9	71.3	77.0	74.8	80.4	78.8	84.4	70.4	74.9
Maldives	75 to 79	57.6	71.5	62.0	76.7	49.0	63.6	38.9	58.8	52.4	68.1	41.1	60.7	70.2	82.0	80.8	88.5	68.9	80.3	72.4	78.4	75.4	82.9	66.6	72.2

Mali	75 to 79	60.2	61.0	64.4	65.2	56.3	56.4	56.0	58.3	60.3	60.9	66.1	68.1	83.1	85.8	87.3	88.2	83.6	86.5	70.6	68.7	77.0	76.4	63.1	59.2
Malta	75 to 79	66.0	84.7	74.3	87.6	54.0	79.0	69.4	83.5	79.5	87.5	67.1	82.6	90.4	95.3	95.3	99.0	86.3	90.1	64.2	84.3	73.7	86.9	51.9	80.2
Marshall Islands	75 to 79	55.0	59.3	67.8	68.9	37.4	46.5	42.2	43.7	63.8	63.1	37.1	40.7	67.4	74.1	78.2	81.6	66.6	75.0	74.5	75.9	80.8	80.6	66.5	70.1
Mauritania	75 to 79	61.6	65.3	68.3	71.4	55.0	58.2	52.8	61.0	57.4	64.1	67.7	71.1	85.3	88.8	89.7	91.4	85.8	89.1	70.6	70.5	79.8	79.4	56.3	57.5
Mauritius	75 to 79	63.5	75.6	72.8	82.5	50.2	65.0	51.6	62.6	67.7	75.4	49.7	59.7	70.7	74.3	85.2	86.4	58.9	66.8	66.8	78.7	75.7	84.3	55.3	71.0
Mexico	75 to 79	73.6	82.1	81.1	87.6	62.2	72.9	66.7	72.8	77.1	81.9	65.9	70.2	90.2	83.6	94.7	90.2	86.7	80.8	74.4	83.9	82.5	89.0	62.6	76.0
Micronesia (Federated States of)	75 to 79	55.5	61.7	65.9	70.8	41.6	48.7	43.5	48.2	63.1	66.0	40.3	45.2	67.1	74.6	77.2	82.5	69.0	75.9	74.4	76.3	79.8	81.5	68.4	70.0
Monaco	75 to 79	73.6	84.4	78.8	87.0	66.1	79.6	69.7	79.6	80.0	85.7	67.5	78.3	77.4	89.3	85.2	93.5	78.0	87.8	73.1	83.8	78.4	86.0	67.1	80.7
Mongolia	75 to 79	44.0	61.1	59.9	71.4	19.8	45.6	33.6	50.0	55.3	60.3	32.8	54.2	39.6	46.4	65.9	65.7	28.8	43.0	84.6	85.5	90.3	89.9	73.9	77.5
Montenegro	75 to 79	47.0	42.9	56.3	52.1	36.2	33.9	41.0	36.4	61.7	58.6	37.0	33.1	73.5	69.7	83.0	79.0	72.1	71.6	75.3	69.7	80.8	76.0	68.4	62.7
Morocco	75 to 79	49.9	57.9	51.6	62.0	46.1	52.3	45.4	50.2	57.9	62.5	51.0	54.5	66.1	70.0	80.2	81.9	62.3	66.6	51.1	56.9	54.3	62.4	46.9	51.3
Mozambique	75 to 79	54.2	55.3	66.2	68.5	37.1	33.4	45.0	43.5	64.9	65.2	40.9	35.9	76.6	77.7	88.6	88.8	70.6	71.5	68.2	66.5	76.9	77.1	56.2	48.7
Myanmar	75 to 79	43.4	55.3	54.7	66.4	29.9	40.5	32.3	39.1	50.1	58.4	36.7	38.8	61.5	70.5	77.5	82.8	55.7	65.8	59.9	67.3	69.8	76.5	48.1	55.3
Namibia	75 to 79	65.8	66.7	75.0	75.7	50.6	51.4	49.1	49.4	65.2	66.5	50.2	48.3	85.7	85.8	92.6	92.4	80.9	81.4	73.4	72.6	81.7	80.7	59.1	58.7
Nauru	75 to 79	58.7	53.7	68.5	63.4	45.7	43.8	45.7	18.6	65.1	43.1	42.2	25.4	70.4	64.3	77.2	73.8	74.6	77.6	73.5	74.0	79.4	81.5	65.8	66.2
Nepal	75 to 79	45.2	55.5	50.7	64.2	39.0	44.0	55.2	61.6	68.5	74.3	56.0	59.5	59.1	69.0	76.0	82.3	55.2	64.0	52.1	60.2	56.7	68.9	49.0	50.1
Netherlands	75 to 79	80.8	87.9	85.1	90.6	73.7	82.4	73.1	79.6	82.1	85.8	70.7	78.1	80.7	85.2	88.7	92.5	77.8	80.3	81.1	88.9	85.1	90.8	75.4	85.1
New Zealand	75 to 79	75.9	86.3	81.3	88.9	67.6	80.8	72.9	76.4	82.1	84.7	69.7	73.2	75.7	80.3	85.1	88.4	73.3	77.4	75.5	87.7	81.2	89.6	68.2	84.0
Nicaragua	75 to 79	75.0	80.4	81.3	85.1	65.4	72.3	67.5	72.7	77.4	81.0	66.4	71.2	88.4	90.1	93.6	94.4	84.7	87.0	78.5	82.4	84.6	86.7	69.9	75.9
Niger	75 to 79	61.9	59.6	70.2	68.5	50.9	48.1	55.7	55.1	65.2	65.4	60.1	60.5	82.5	83.2	89.9	89.9	80.0	81.1	73.2	70.3	81.6	80.1	61.1	55.2
Nigeria	75 to 79	57.9	67.9	67.3	75.9	45.7	56.3	54.2	65.2	63.4	71.5	62.1	70.4	82.5	89.4	89.5	93.5	80.4	87.6	67.2	71.4	78.6	82.0	48.6	54.6
Niue	75 to 79	64.5	68.1	73.6	77.0	50.7	54.2	49.4	50.9	67.8	69.5	44.7	44.7	74.1	79.4	82.7	86.4	76.2	81.5	77.7	79.3	83.2	84.9	70.4	71.4
North Macedonia	75 to 79	38.8	50.1	47.2	57.4	29.0	41.8	18.0	36.3	41.9	55.1	20.8	38.0	37.8	53.0	57.9	66.7	40.1	56.7	39.8	49.2	50.0	58.1	29.3	40.9
Northern Mariana Islands	75 to 79	66.3	70.7	71.3	77.6	59.3	61.0	56.6	58.7	67.1	71.1	58.9	58.1	73.2	76.8	76.8	82.2	82.7	80.8	78.7	81.1	81.9	85.4	74.6	75.4
Norway	75 to 79	79.3	89.8	83.1	91.7	72.9	84.8	75.2	83.4	84.6	88.2	70.9	81.7	78.0	88.7	85.4	94.4	79.7	84.5	78.3	89.8	82.2	91.3	73.2	86.3
Oman	75 to 79	53.0	69.3	64.0	75.5	38.6	60.0	47.8	64.5	67.4	76.8	42.0	61.1	75.6	84.3	88.0	91.8	66.4	78.4	54.3	68.1	66.0	75.0	40.2	59.2
Pakistan	75 to 79	61.0	61.4	64.4	66.4	54.6	54.8	63.7	62.8	73.5	72.9	64.0	64.1	67.1	68.9	79.7	79.8	65.0	67.9	67.2	66.0	69.9	71.3	63.2	60.3
Palau	75 to 79	66.3	72.2	76.0	77.0	52.7	65.2	55.3	59.7	72.2	70.9	50.6	59.9	79.2	82.1	85.3	85.8	80.7	87.1	76.8	79.6	82.6	82.7	69.0	75.7
Palestine	75 to 79	31.8	54.0	46.5	62.6	13.9	43.4	45.3	53.7	65.3	69.1	40.1	51.8	81.0	86.6	90.2	93.5	74.1	80.6	26.2	50.6	44.1	61.0	5.8	39.8
Panama	75 to 79	70.0	76.8	77.6	85.8	59.0	63.7	64.1	70.2	75.7	82.4	62.4	65.4	84.5	83.1	90.0	91.4	83.6	77.5	73.7	79.4	80.6	86.5	64.3	69.2
Papua New Guinea	75 to 79	44.8	49.8	49.7	53.3	38.2	43.8	34.5	36.4	49.8	49.9	39.2	41.6	62.8	69.1	69.8	74.5	70.0	74.9	71.9	73.5	72.1	73.8	72.0	72.5
Paraguay	75 to 79	61.1	68.6	68.4	76.7	51.6	57.2	48.8	53.3	65.6	70.8	47.9	48.8	78.0	78.7	85.2	85.6	79.0	79.7	67.3	72.1	74.3	79.8	58.6	61.9
Peru	75 to 79	69.4	81.7	78.5	87.4	56.0	72.7	57.4	68.0	72.4	78.5	54.5	66.2	82.3	86.6	89.7	91.1	79.3	86.8	75.3	85.1	83.1	89.7	64.3	77.7
Philippines	75 to 79	61.0	65.2	71.1	75.7	47.1	48.4	47.3	51.3	65.2	68.7	45.4	47.4	77.4	79.5	87.1	88.5	73.2	74.4	72.7	74.4	80.1	82.5	62.8	61.9
Poland	75 to 79	41.4	75.1	53.8	80.7	26.3	66.2	44.6	62.0	63.4	75.6	43.1	57.8	74.8	77.0	85.6	86.9	68.1	71.9	37.3	74.8	52.5	80.5	20.5	67.3
Portugal	75 to 79	57.6	76.9	66.6	81.3	46.1	69.7	59.8	70.2	73.6	79.7	57.6	68.8	73.3	75.8	83.9	85.0	70.9	73.9	57.6	79.7	67.4	83.4	46.2	74.6
Puerto Rico	75 to 79	75.5	88.8	83.4	95.5	63.6	77.5	71.2	77.8	81.8	86.4	67.7	73.8	89.2	90.2	93.9	95.5	86.4	84.8	76.4	91.6	83.4	96.6	66.8	83.1
Qatar	75 to 79	60.0	74.6	68.5	79.8	48.1	66.3	37.6	66.7	61.5	77.5	31.3	63.9	33.0	75.3	78.7	88.9	0.0	64.1	69.1	76.7	75.5	81.6	60.3	69.9
Republic of Korea	75 to 79	70.4	91.6	76.6	93.4	62.3	87.0	72.5	92.0	83.0	95.2	67.3	88.4	67.4	89.1	79.5	93.7	67.3	88.2	74.5	91.0	80.0	92.1	68.0	87.8
Republic of Moldova	75 to 79	49.1	64.2	57.9	70.6	39.4	56.1	40.8	46.5	61.0	63.0	38.6	47.6	70.4	73.4	86.4	86.8	54.2	62.6	58.0	66.8	65.9	73.6	51.0	59.3
Romania	75 to 79	55.0	66.7	62.7	73.7	45.8	56.9	33.4	39.3	53.5	59.0	35.8	38.5	61.7	55.2	76.1	72.5	57.0	49.0	57.3	68.4	66.0	75.7	48.1	59.6
Russian Federation	75 to 79	36.4	57.3	51.4	68.1	14.5	40.7	50.1	53.7	66.3	69.2	50.7	51.9	69.9	62.6	83.4	78.4	54.8	51.0	28.6	54.7	48.2	67.3	0.0	36.5
Rwanda	75 to 79	49.4	64.3	62.9	74.1	29.7	47.8	30.8	48.7	56.9	68.4	23.7	39.8	76.8	85.2	89.9	93.3	68.8	78.7	69.1	76.1	76.3	82.2	60.8	67.3
Saint Kitts and Nevis	75 to 79	47.5	56.2	60.6	66.3	29.1	42.9	47.7	50.9	67.1	67.9	42.0	48.2	74.5	78.7	85.4	86.7	69.3	77.1	53.0	59.8	65.2	68.9	37.3	49.6
Saint Lucia	75 to 79	55.5	68.0	65.0	77.8	43.4	53.7	52.6	59.4	69.1	76.1	49.8	53.6	81.0	85.3	88.6	91.6	78.5	82.2	59.5	71.1	66.5	78.0	53.5	62.1
Saint Vincent and the Grenadines	75 to 79	44.2	61.2	59.3	71.7	22.9	46.7	29.5	53.5	57.5	70.3	22.3	50.1	82.2	85.2	90.5	92.3	76.9	80.6	53.3	66.0	64.0	73.7	41.8	56.5
Samoa	75 to 79	60.9	66.0	72.0	74.0	44.7	55.1	47.0	49.5	67.6	66.3	40.5	47.9	72.7	78.1	81.8	84.3	72.6	81.3	78.0	80.2	83.4	85.2	70.7	73.5
San Marino	75 to 79	78.6	87.5	82.1	89.7	72.9	82.5	74.5	83.0	81.7	87.2	74.8	82.5	89.6	96.9	94.3	99.3	86.4	92.6	78.5	86.9	82.5	89.3	73.1	82.7

Sao Tome and Principe	75 to 79	69.3	70.9	76.2	77.7	59.7	61.0	61.4	63.7	68.6	70.3	68.7	71.3	89.7	91.0	93.7	94.0	88.2	90.7	75.6	74.9	83.5	82.9	62.1	60.6
Saudi Arabia	75 to 79	45.3	64.8	49.4	64.8	37.8	58.4	43.3	53.9	59.6	66.6	44.4	53.8	77.5	82.0	87.7	91.4	72.7	76.8	48.2	65.9	53.1	66.0	40.8	60.4
Senegal	75 to 79	63.1	62.6	71.6	71.8	51.7	50.0	57.1	57.5	63.3	65.1	64.3	65.1	85.5	85.7	91.1	90.4	83.7	85.4	70.1	68.8	80.8	80.0	54.2	50.5
Serbia	75 to 79	23.7	59.7	37.2	62.8	10.2	55.4	3.1	40.5	37.1	56.4	0.0	44.0	30.1	62.2	52.2	73.1	32.3	64.7	22.7	58.5	39.0	62.6	8.1	55.0
Seychelles	75 to 79	64.8	72.4	76.3	81.2	48.0	59.0	49.8	59.1	67.0	73.4	47.6	56.1	78.6	86.0	88.7	93.3	71.1	79.4	71.5	76.8	82.1	84.8	56.0	64.8
Sierra Leone	75 to 79	61.9	61.6	70.2	69.1	50.9	52.6	57.4	56.5	63.4	63.1	64.6	66.6	85.6	86.2	90.8	90.2	84.1	86.1	68.2	67.7	79.9	78.2	51.7	52.1
Singapore	75 to 79	82.7	100.0	86.1	98.5	77.2	100.0	87.1	100.0	91.6	98.4	84.5	100.0	91.9	100.0	95.3	100.0	92.2	100.0	80.9	98.5	84.2	96.2	77.1	100.0
Slovakia	75 to 79	73.1	80.1	78.6	84.5	65.0	72.7	40.9	54.5	61.0	69.7	38.6	52.2	74.0	77.0	84.5	86.7	69.4	71.9	74.4	80.2	80.0	84.5	67.6	74.3
Slovenia	75 to 79	59.3	80.2	69.1	84.8	45.0	72.3	52.4	64.6	68.8	76.6	49.0	61.6	73.5	80.7	84.5	89.2	67.9	76.0	56.8	81.1	68.2	85.5	42.0	74.6
Solomon Islands	75 to 79	57.5	61.1	66.5	69.2	45.2	50.1	50.2	51.2	67.3	67.2	47.0	49.0	67.9	74.6	77.1	82.1	69.3	75.8	76.7	77.4	80.1	81.2	72.3	73.6
Somalia	75 to 79	50.0	55.7	64.2	67.3	29.9	37.7	27.8	32.5	54.8	57.3	21.4	26.2	73.2	78.9	89.0	90.1	64.9	71.4	72.9	74.2	79.1	80.4	65.8	66.4
South Sudan	75 to 79	83.0	77.7	72.3	73.7	42.7	50.3	67.6	57.6	62.5	62.4	34.1	41.6	94.0	89.8	91.6	92.0	76.0	78.8	85.8	80.7	82.4	82.8	67.7	68.6
Spain	75 to 79	59.9	63.8	78.1	92.8	61.6	88.1	39.5	43.7	79.7	85.3	67.2	75.1	81.1	83.2	92.4	94.0	80.4	77.8	76.2	76.7	78.3	93.4	62.3	91.8
Sri Lanka	75 to 79	71.5	91.9	68.6	75.4	44.4	54.9	69.4	77.7	56.8	63.1	37.2	45.8	85.3	85.4	91.5	93.2	78.5	81.5	71.3	93.7	74.3	78.7	52.6	61.0
Sudan	75 to 79	58.0	66.8	38.9	59.3	32.0	52.3	37.1	45.5	52.3	59.2	33.8	51.9	83.6	86.8	76.6	78.9	45.9	64.2	64.6	71.0	45.5	61.9	40.1	52.4
Suriname	75 to 79	36.6	57.5	57.4	71.0	39.1	55.1	31.9	47.1	62.8	71.2	45.3	58.0	54.7	66.1	86.1	90.9	75.3	79.2	43.4	58.2	64.7	75.4	51.1	63.6
Sweden	75 to 79	49.0	64.1	82.3	90.5	72.9	83.7	45.5	58.3	84.6	90.9	78.5	86.7	77.4	83.6	85.6	93.3	82.5	86.2	57.8	69.7	81.9	89.8	72.7	84.6
Switzerland	75 to 79	78.9	88.7	79.6	92.1	67.9	86.9	78.9	88.0	81.3	89.5	73.7	84.3	79.8	88.7	84.9	91.8	77.9	83.2	77.9	88.1	80.3	91.9	68.3	88.9
Syrian Arab Republic	75 to 79	75.0	90.9	57.2	62.4	43.7	51.1	73.8	85.7	65.5	66.1	50.8	52.7	77.2	85.9	86.1	88.5	67.8	73.5	75.0	91.3	61.1	64.7	48.8	54.8
Taiwan (Province of China)	75 to 79	51.7	57.8	75.2	90.8	63.7	77.6	50.8	52.2	70.4	82.6	59.1	72.1	73.5	78.2	84.8	89.7	70.4	73.0	55.4	60.1	82.1	92.9	73.2	82.9
Tajikistan	75 to 79	70.7	85.8	65.7	70.0	34.4	49.3	58.6	74.4	61.8	65.8	46.5	57.9	74.5	80.2	83.3	83.1	68.3	73.9	78.4	88.9	71.9	74.5	37.6	52.4
Thailand	75 to 79	53.8	61.3	74.5	83.3	54.8	66.8	45.3	55.7	69.7	75.6	57.5	64.0	72.0	75.1	74.3	83.2	49.4	66.3	58.4	64.9	83.7	88.7	68.3	76.8
Timor-Leste	75 to 79	66.3	76.8	54.8	61.4	43.5	44.0	57.0	64.8	49.7	53.4	43.9	42.8	56.1	70.9	74.8	78.3	65.2	67.3	77.2	84.0	71.9	74.4	61.5	57.7
Togo	75 to 79	49.4	53.6	69.1	68.8	51.8	46.2	36.1	37.9	61.8	62.7	64.2	61.9	63.3	67.0	90.0	89.6	83.5	83.3	66.9	66.9	79.6	78.6	53.7	46.3
Tokelau	75 to 79	61.4	59.1	67.0	75.0	38.1	51.8	54.9	51.6	65.3	65.0	39.5	44.5	84.6	83.6	78.4	85.5	70.8	81.4	69.2	67.2	78.8	84.8	63.4	72.0
Tonga	75 to 79	55.0	65.6	78.4	79.1	61.6	64.5	44.9	46.6	73.5	73.3	57.6	59.1	69.1	78.7	87.5	88.4	82.5	85.7	71.9	79.3	86.4	85.8	74.7	75.6
Trinidad and Tobago	75 to 79	71.6	73.2	68.5	75.2	46.7	57.7	60.0	60.5	68.4	73.8	52.1	58.9	81.9	83.5	87.7	88.0	74.6	77.0	81.6	81.5	71.6	76.8	47.1	59.5
Tropical Latin America	75 to 79	59.2	67.9	68.2	80.7	45.8	64.6	53.0	60.7	69.5	75.4	49.4	58.5	78.6	79.8	88.8	85.0	83.4	75.5	60.9	69.3	70.2	82.3	47.6	67.3
Tunisia	75 to 79	52.8	67.4	55.7	73.0	45.9	59.2	50.0	59.6	64.0	71.7	51.1	59.6	69.5	78.4	83.5	88.1	64.4	73.4	53.8	67.0	57.4	73.5	47.3	58.9
Turkey	75 to 79	59.3	71.9	69.0	78.4	46.2	62.0	50.6	66.3	68.5	77.7	46.5	63.7	54.9	70.3	78.0	84.5	39.6	61.6	63.9	73.6	73.1	79.9	52.5	65.3
Turkmenistan	75 to 79	65.6	61.8	74.5	73.0	51.0	44.4	58.7	48.9	71.4	65.9	58.1	46.6	70.7	55.0	83.8	76.3	62.2	43.6	67.4	65.0	76.8	75.7	52.9	49.4
Tuvalu	75 to 79	50.0	61.7	61.8	70.6	34.1	49.6	37.3	45.7	59.3	64.2	34.3	43.6	62.7	74.6	74.2	82.1	66.1	77.6	70.6	75.2	77.4	80.9	63.1	68.3
Uganda	75 to 79	64.9	72.5	77.1	81.0	45.6	56.8	41.3	51.3	66.1	70.0	31.0	43.9	81.0	87.0	92.7	94.4	73.3	80.6	79.1	81.3	84.8	86.3	70.6	73.2
Ukraine	75 to 79	53.5	65.7	62.3	73.7	44.5	53.8	54.2	58.7	68.3	72.2	56.0	57.0	53.7	64.4	71.6	79.4	42.6	55.0	49.2	63.5	60.4	72.9	39.2	51.1
United Arab Emirates	75 to 79	65.3	64.1	69.8	11.0	58.4	72.6	55.4	44.1	67.9	0.0	56.2	63.2	47.7	34.1	66.9	0.0	46.2	65.1	67.2	66.7	72.4	19.4	60.6	75.0
United Kingdom	75 to 79	73.4	88.1	78.8	90.5	64.4	82.9	73.2	77.2	81.3	84.1	72.5	75.7	76.5	81.2	84.7	88.6	77.4	78.9	70.9	90.1	77.8	91.8	62.2	86.5
United Republic of Tanzania	75 to 79	64.5	70.9	75.9	78.7	47.8	58.7	44.6	52.0	66.6	68.1	37.4	50.0	83.5	86.9	93.8	93.8	76.7	82.1	79.4	78.0	85.0	84.4	71.3	68.3
United States Virgin Islands	75 to 79	62.3	79.9	72.1	87.4	48.5	68.4	53.8	70.9	70.1	82.2	50.8	66.2	79.8	89.4	88.9	95.9	74.1	82.0	66.7	82.1	74.7	87.8	56.9	73.8
United States of America	75 to 79	84.8	94.0	88.9	96.1	77.2	89.0	77.9	85.5	85.2	90.4	75.3	83.3	81.6	81.9	89.7	90.9	76.8	75.3	84.8	95.5	88.7	96.1	78.9	92.8
Uruguay	75 to 79	73.1	81.1	79.6	86.3	63.5	72.4	71.6	77.3	83.1	86.7	64.6	70.5	71.9	77.2	81.7	85.9	72.0	75.3	73.5	82.5	79.9	86.9	65.4	75.8
Uzbekistan	75 to 79	62.7	68.6	72.0	77.6	48.7	54.7	50.1	63.2	64.7	72.7	52.1	63.7	80.1	51.8	91.0	74.5	69.3	41.9	70.6	71.9	79.3	81.2	57.3	57.8
Vanuatu	75 to 79	59.6	64.9	70.7	73.9	44.8	52.0	43.5	46.7	65.0	65.8	38.1	42.7	70.7	76.4	79.5	83.8	71.7	77.6	76.0	78.6	82.3	84.1	67.8	71.1
Venezuela (Bolivarian Republic of)	75 to 79	68.5	70.9	77.2	80.1	55.5	56.7	59.4	62.7	73.4	76.6	56.7	58.1	88.2	83.3	93.4	90.5	85.1	79.5	74.4	74.7	81.9	82.6	63.6	63.1
Viet Nam	75 to 79	46.0	51.6	59.7	65.5	27.4	33.0	36.9	38.2	57.7	61.1	35.7	33.3	64.4	71.2	79.7	83.8	56.4	64.6	61.3	62.2	72.4	74.2	46.4	46.5
Yemen	75 to 79	25.2	38.1	37.2	47.2	13.4	26.9	24.3	29.3	51.6	50.0	20.1	32.6	50.5	55.6	76.2	76.1	29.5	46.9	33.1	39.7	44.1	51.1	24.6	26.7
Zambia	75 to 79	59.0	63.3	70.5	73.7	43.5	46.6	37.7	42.0	57.3	61.2	36.8	40.5	78.8	81.6	89.8	90.2	74.3	77.5	77.1	74.9	82.4	81.6	70.0	65.0
Zimbabwe	75 to 79	69.2	62.7	78.1	73.0	55.0	46.5	40.1	20.7	60.0	47.0	41.5	26.0	76.3	71.5	87.0	83.6	72.5	68.4	77.4	72.7	84.0	80.7	66.8	59.7
Afghanistan	All ages	18.3	47.1	28.2	47.8	7.7	45.5	22.6	47.0	44.3	51.2	8.9	50.4	41.0	61.0	59.7	66.5	14.8	53.9	19.9	42.1	22.6	43.5	25.9	47.7

Albania	All ages	38.5	32.9	43.3	33.9	32.9	31.6	33.9	18.5	42.9	23.9	34.9	25.6	71.7	73.6	74.1	73.9	68.8	73.6	57.8	49.5	63.7	51.7	57.9	54.2
Algeria	All ages	61.6	68.9	61.4	68.0	60.8	68.9	60.5	65.2	62.6	65.2	63.7	69.7	73.3	83.2	74.8	82.8	71.2	83.7	54.9	59.2	55.9	59.4	59.7	64.6
American Samoa	All ages	63.1	60.9	70.9	65.6	54.8	55.1	57.3	51.0	64.6	57.0	55.9	51.6	75.8	77.1	76.3	73.6	75.6	81.5	79.0	75.5	84.6	79.0	77.6	76.5
Andorra	All ages	77.7	80.5	75.6	76.9	78.1	83.4	77.5	80.1	78.3	79.8	78.6	82.2	76.1	86.0	71.2	80.9	82.7	93.5	70.9	73.5	69.7	69.4	75.7	81.3
Angola	All ages	50.8	64.0	59.6	70.1	39.6	55.1	38.7	48.9	45.5	53.5	40.4	51.4	69.6	78.4	73.1	80.4	66.4	76.4	70.9	73.4	79.3	80.2	65.7	69.4
Antigua and Barbuda	All ages	38.4	58.7	48.0	67.5	25.7	46.9	35.9	48.8	50.2	63.5	30.5	40.5	68.5	79.8	75.3	81.5	56.2	76.3	31.0	56.4	36.4	62.6	33.4	55.4
Argentina	All ages	59.2	76.1	62.7	78.6	54.8	72.4	64.4	72.9	72.2	80.0	60.6	68.2	52.4	76.1	57.0	76.8	45.1	74.8	56.9	75.2	57.8	77.0	61.8	77.9
Armenia	All ages	60.2	66.7	62.1	68.2	57.6	64.5	51.8	64.4	55.8	71.4	55.0	61.0	70.6	77.1	72.9	80.6	67.7	72.8	53.6	54.6	56.9	56.2	56.8	59.3
Australia	All ages	68.7	81.6	66.0	78.4	70.4	83.7	67.6	73.7	69.7	75.6	69.5	74.6	66.7	79.5	68.4	81.6	63.3	75.2	59.8	78.4	55.0	73.7	69.1	85.4
Austria	All ages	61.3	89.2	60.0	88.4	63.4	89.2	76.7	83.8	78.9	84.6	76.8	84.2	87.8	96.1	86.5	94.5	89.2	97.1	37.1	85.2	31.3	86.0	51.6	87.9
Azerbaijan	All ages	54.0	62.1	59.0	65.2	47.3	57.9	47.8	52.6	53.0	55.6	50.1	56.0	83.6	90.0	85.1	90.1	81.9	89.8	63.2	69.2	70.2	74.7	60.4	68.3
Bahamas	All ages	56.2	66.2	62.2	71.4	47.7	58.9	50.6	58.1	60.4	67.5	46.9	53.9	73.6	78.6	76.1	81.2	68.3	73.1	59.7	67.2	62.5	69.7	62.8	70.1
Bahrain	All ages	68.3	83.2	67.2	79.9	68.2	84.5	70.1	84.5	72.7	83.4	71.2	86.5	77.5	91.5	76.6	89.9	78.5	93.3	65.1	77.1	64.0	74.1	70.0	81.6
Bangladesh	All ages	26.5	41.9	31.8	44.6	21.2	38.4	38.8	49.5	47.8	56.2	38.6	49.6	31.4	58.5	47.1	62.0	19.1	55.7	34.1	36.0	34.2	37.3	40.4	41.9
Barbados	All ages	41.5	54.7	44.2	58.1	38.2	50.2	44.8	50.3	54.3	60.2	42.7	47.0	69.6	74.5	74.4	77.4	61.3	69.5	21.2	42.4	18.9	43.1	34.3	49.5
Belarus	All ages	53.8	58.0	53.9	60.3	54.3	55.3	58.4	56.7	60.2	63.3	62.2	55.7	61.7	60.8	66.8	68.7	54.0	49.0	36.1	43.7	35.3	45.6	46.4	49.8
Belgium	All ages	55.5	75.1	55.3	74.5	55.9	75.1	59.3	66.5	64.3	71.5	59.7	65.4	74.3	74.0	73.6	75.7	75.4	71.3	40.0	71.4	37.6	70.2	50.7	77.3
Belize	All ages	55.5	65.2	61.4	71.3	47.8	57.8	49.9	57.1	58.4	65.7	48.0	54.3	73.2	82.4	78.6	83.6	64.2	79.7	58.9	66.2	63.3	72.0	60.2	65.7
Benin	All ages	52.0	62.0	54.4	64.4	48.8	58.7	50.3	59.8	44.2	55.5	61.4	68.3	77.1	82.4	77.5	82.1	76.8	82.8	57.2	64.4	71.2	75.3	46.6	56.2
Bermuda	All ages	63.3	74.0	69.6	80.8	54.3	64.8	65.1	72.1	74.2	83.5	58.8	63.2	81.9	91.8	86.8	98.7	71.8	79.6	49.0	62.9	55.8	70.1	47.8	60.9
Bhutan	All ages	53.8	59.7	52.4	61.0	54.4	57.4	55.0	60.6	58.4	64.1	57.7	62.0	57.3	69.5	58.6	69.3	56.1	69.9	60.5	55.1	58.5	57.9	67.7	58.2
Bolivia (Plurinational State of)	All ages	36.5	56.0	40.4	58.4	31.1	52.6	34.7	46.5	41.4	52.0	37.8	48.7	37.5	57.6	39.9	55.2	33.7	61.8	46.4	58.5	53.5	64.0	45.1	58.7
Bosnia and Herzegovina	All ages	55.5	56.2	55.2	55.7	55.6	56.8	47.2	48.0	51.3	55.0	51.0	48.3	50.3	66.3	54.7	67.9	44.1	64.4	42.5	39.6	41.1	37.5	51.5	49.4
Botswana	All ages	55.1	70.5	61.8	75.2	46.0	63.2	39.6	58.0	39.7	59.8	46.6	61.6	81.5	89.6	83.8	89.8	78.8	89.3	65.4	72.7	73.4	79.4	60.6	68.7
Brazil	All ages	46.1	63.2	52.7	67.2	38.2	57.9	48.0	55.1	58.4	64.6	44.1	51.1	51.9	58.8	55.5	59.8	45.5	56.9	39.0	59.4	47.5	65.1	38.5	59.8
Brunei Darussalam	All ages	71.9	79.9	71.4	80.1	70.7	78.4	75.6	79.3	78.5	82.9	75.1	77.7	56.3	70.8	55.8	71.4	57.5	70.3	72.0	81.0	72.9	82.0	74.8	83.2
Bulgaria	All ages	30.5	35.5	32.5	39.4	27.8	31.3	29.7	40.5	35.8	48.4	33.4	40.8	60.4	50.3	65.8	55.5	53.7	43.5	21.5	10.2	22.9	13.7	28.5	16.9
Burkina Faso	All ages	58.7	62.5	70.4	73.0	44.4	49.7	52.5	57.9	59.6	63.7	51.8	57.5	80.7	83.4	86.4	88.2	74.2	78.2	71.7	70.7	88.7	86.4	55.7	56.5
Burundi	All ages	36.8	56.8	48.5	65.1	21.3	47.2	27.9	41.8	41.7	51.8	24.3	40.2	60.0	75.3	69.4	80.3	49.8	71.6	59.8	72.7	64.8	77.7	59.9	72.2
Cabo Verde	All ages	65.5	66.7	69.2	72.7	60.3	59.1	66.7	69.1	65.0	68.9	72.7	72.3	86.1	92.5	88.7	94.2	82.1	90.0	64.0	59.2	76.8	73.7	52.8	48.1
Cambodia	All ages	29.5	42.4	30.8	45.6	28.2	38.3	30.8	40.4	34.1	44.4	39.0	45.3	55.0	69.5	57.7	70.6	51.0	68.0	50.3	51.2	55.0	55.7	52.3	53.3
Cameroon	All ages	55.9	60.9	62.2	66.4	48.2	54.2	52.7	58.2	54.7	58.1	56.7	62.8	76.9	79.2	80.7	82.4	72.9	76.2	69.3	67.6	82.1	80.9	58.9	57.8
Canada	All ages	79.3	91.7	79.4	89.6	78.3	93.0	81.6	87.1	84.6	88.4	79.7	86.4	78.4	85.1	79.9	86.6	73.9	80.9	72.0	89.9	72.1	88.4	76.6	93.6
Central African Republic	All ages	36.4	45.0	47.1	55.1	22.8	32.2	28.3	33.0	36.0	40.4	30.7	34.9	60.0	66.0	67.1	72.9	52.4	59.2	62.0	67.2	69.5	73.6	58.2	64.6
Chad	All ages	54.7	57.3	60.0	64.9	48.0	48.6	51.6	56.0	50.5	56.2	58.5	60.1	75.4	73.6	78.7	77.2	71.9	70.5	62.3	63.4	76.7	80.9	50.6	49.8
Chile	All ages	63.0	77.8	66.4	80.1	58.4	74.3	69.3	81.9	76.8	87.0	64.1	77.3	66.6	75.6	68.1	76.9	64.1	72.7	54.8	71.7	57.0	74.4	58.6	73.7
China	All ages	34.5	55.2	42.2	63.4	25.7	46.3	39.9	44.0	50.1	54.4	38.4	41.9	10.9	60.8	18.3	66.9	1.8	53.9	48.5	56.7	57.2	66.3	46.6	54.2
Colombia	All ages	66.7	77.3	70.5	81.6	61.0	70.5	64.1	73.3	69.6	79.7	62.5	68.6	71.7	74.6	72.8	75.7	69.3	71.8	63.9	74.1	70.3	80.9	62.3	71.1
Comoros	All ages	53.9	64.3	60.7	69.2	44.7	57.0	40.0	45.1	45.9	49.2	42.5	49.0	73.8	82.8	76.5	84.2	71.4	81.6	71.1	73.3	77.4	79.2	68.2	70.7
Congo	All ages	51.0	61.8	56.9	64.9	42.8	57.3	38.1	44.3	39.6	44.6	45.1	52.2	71.3	78.4	73.7	77.0	68.7	79.9	67.2	71.2	74.5	76.7	62.8	69.5
Cook Islands	All ages	60.2	73.8	68.5	79.6	51.0	66.7	53.7	62.0	63.6	71.9	50.7	57.3	72.5	91.5	72.3	87.8	72.8	96.2	73.0	80.1	80.2	84.4	70.9	80.0
Costa Rica	All ages	76.4	81.1	80.2	85.3	70.5	74.4	76.5	80.2	81.0	86.5	73.7	75.0	83.8	84.7	83.6	86.3	83.2	80.6	69.9	74.9	76.5	80.6	68.0	73.2
Coted'Ivoire	All ages	65.4	65.4	73.3	71.5	57.8	58.6	63.3	63.2	64.3	62.2	65.3	67.1	76.7	80.2	81.4	83.1	73.0	77.9	71.6	67.9	87.1	83.2	62.5	57.8
Croatia	All ages	38.4	60.4	39.4	58.9	37.0	61.7	40.5	47.2	49.4	58.8	40.0	42.3	49.2	57.1	52.6	60.3	44.3	52.6	20.5	50.0	17.7	44.8	32.2	61.3
Cuba	All ages	57.4	58.8	62.5	64.4	50.7	51.6	55.8	54.4	63.4	64.2	53.7	50.5	78.7	85.9	79.5	86.8	76.4	83.0	48.7	45.1	54.6	51.1	49.2	46.0
Cyprus	All ages	47.1	64.4	43.3	64.2	51.3	64.3	59.7	74.4	63.8	77.4	61.3	73.9	61.7	82.5	59.7	80.4	65.0	85.2	25.9	46.3	14.8	43.8	45.5	56.1
Czechia	All ages	39.5	73.7	40.7	74.0	38.4	73.0	45.8	66.8	53.9	72.6	45.3	64.7	56.4	73.9	59.1	75.2	52.4	72.1	13.8	65.9	12.7	66.0	25.2	71.3
Democratic People's Republic of Korea	All ages	46.6	43.1	51.2	48.6	40.7	36.3	44.3	30.5	50.8	38.3	46.0	33.2	49.7	54.9	53.3	58.6	44.5	50.1	60.1	55.8	64.8	61.4	61.2	56.2

Democratic Republic of the Congo	All ages	53.0	58.1	58.4	63.4	45.9	50.8	43.0	45.3	45.1	48.9	48.2	49.0	73.7	78.4	75.0	80.4	72.6	76.9	68.0	69.9	74.9	74.8	64.8	69.3
Denmark	All ages	64.8	74.7	64.0	74.5	65.0	74.1	60.1	68.3	63.6	73.2	62.0	67.1	69.8	80.1	69.3	79.7	70.7	80.3	55.6	69.1	54.1	68.4	63.0	74.4
Djibouti	All ages	62.2	68.3	70.3	76.7	53.1	59.2	50.3	51.2	55.7	57.5	51.2	51.5	75.5	81.3	81.5	85.5	71.4	78.7	79.4	77.7	84.9	85.9	77.6	73.6
Dominica	All ages	33.5	41.5	41.1	46.3	22.4	35.5	28.5	32.4	41.0	45.8	25.7	28.6	69.9	73.7	75.6	79.5	59.1	64.7	22.6	30.5	28.1	29.2	25.1	41.4
Dominican Republic	All ages	55.9	60.6	62.6	67.5	47.2	51.9	50.1	52.7	60.2	62.4	46.2	49.3	63.9	75.9	67.3	77.0	56.9	72.9	63.0	61.0	69.8	67.7	61.2	60.0
Ecuador	All ages	64.3	75.9	70.4	79.9	56.0	70.1	55.8	68.0	63.9	74.4	53.3	65.1	74.2	71.9	76.9	73.5	69.7	68.9	64.4	76.1	71.9	82.0	61.6	74.2
Egypt	All ages	17.7	55.1	25.8	57.9	8.3	51.4	7.7	53.8	28.7	61.3	0.0	52.6	39.9	78.4	56.8	82.7	14.1	72.3	9.4	37.2	10.9	40.1	17.9	41.7
El Salvador	All ages	63.0	71.6	69.2	76.7	54.0	63.0	62.9	64.7	69.5	72.0	60.2	60.1	69.3	80.6	72.9	81.5	62.9	77.3	64.6	68.5	73.7	75.7	59.5	64.7
Equatorial Guinea	All ages	42.9	70.7	53.1	73.3	28.9	66.6	29.6	59.4	36.9	59.4	32.3	64.6	65.3	85.7	72.3	84.9	57.5	86.9	64.5	74.5	73.4	79.4	57.9	73.2
Eritrea	All ages	40.6	56.7	51.3	64.3	27.6	46.2	31.8	40.4	38.8	46.1	34.2	43.2	62.8	75.1	74.0	81.4	51.3	68.8	74.6	75.9	78.2	80.3	75.3	75.2
Estonia	All ages	39.2	68.8	37.9	71.3	42.0	65.6	49.5	63.2	52.8	70.8	53.8	59.3	70.0	69.0	78.7	75.3	55.6	59.5	10.2	58.8	4.7	61.6	28.0	62.3
Eswatini	All ages	49.4	50.7	59.5	58.3	33.8	39.7	30.9	31.2	39.6	33.2	32.3	38.4	79.9	80.1	83.2	82.8	75.6	76.8	64.2	62.0	71.8	67.9	58.7	59.9
Ethiopia	All ages	45.8	64.2	56.8	71.1	32.9	55.7	41.7	51.7	52.9	58.6	38.8	51.3	72.5	82.9	78.4	85.7	67.6	81.2	76.4	77.5	81.2	82.5	75.7	76.3
Fiji	All ages	62.6	63.7	69.2	67.9	54.3	58.0	51.2	43.7	60.3	51.5	48.8	44.3	71.7	75.7	72.8	74.4	69.7	78.8	80.8	78.5	87.9	83.0	77.8	78.1
Finland	All ages	69.5	82.0	67.9	80.8	71.2	82.8	71.7	80.1	73.9	80.9	73.0	81.0	77.7	89.8	76.8	88.2	78.7	91.3	57.3	75.8	54.4	74.8	66.8	81.1
France	All ages	57.6	78.3	55.7	75.7	59.5	80.7	58.2	72.0	64.3	73.9	57.7	73.5	77.0	81.3	75.8	81.5	78.7	80.8	43.7	73.7	38.2	70.1	56.0	81.3
Gabon	All ages	58.0	67.0	66.4	73.5	46.7	58.3	43.9	51.7	50.1	56.8	45.3	52.9	80.0	85.0	83.6	86.6	76.2	83.3	69.5	72.6	76.4	78.8	65.8	70.2
Gambia	All ages	61.9	57.2	68.3	62.2	54.9	51.2	57.5	52.5	56.2	50.3	62.4	60.1	78.4	77.9	82.2	80.2	75.3	75.6	67.8	59.4	82.3	73.3	57.8	49.3
Georgia	All ages	42.3	41.6	48.8	48.4	33.6	33.2	46.4	47.8	51.3	54.0	49.1	48.6	68.7	56.1	72.2	58.8	64.5	53.4	43.8	24.7	51.0	33.4	41.4	23.3
Germany	All ages	65.5	84.4	66.1	84.4	65.2	83.7	65.3	77.3	69.7	80.0	65.4	76.8	75.0	84.6	74.7	83.5	75.3	85.5	52.8	80.1	53.4	81.5	60.0	82.9
Ghana	All ages	60.0	65.4	64.6	70.6	54.0	58.3	53.7	57.5	51.8	55.8	61.9	64.1	74.9	80.4	78.2	83.1	71.3	77.6	68.3	68.4	81.8	81.0	56.3	57.9
Greece	All ages	45.0	60.7	42.0	58.8	48.4	63.0	51.5	58.4	54.1	62.5	56.7	59.9	77.7	79.8	77.0	80.9	78.4	77.6	27.6	52.0	21.1	45.8	42.5	64.9
Greenland	All ages	67.2	76.3	64.8	78.1	68.5	73.4	62.2	64.6	67.5	72.8	61.9	61.9	8.0	56.2	11.3	59.6	3.4	51.0	72.0	81.5	69.6	83.2	78.0	83.0
Grenada	All ages	27.7	55.8	38.1	60.3	13.3	50.1	31.6	46.8	43.9	57.5	28.3	43.3	61.8	73.7	67.1	75.6	51.9	70.0	6.3	50.4	18.7	51.4	0.0	56.3
Guam	All ages	74.8	82.5	78.7	86.8	69.6	76.4	67.7	69.6	72.6	75.0	66.7	67.4	80.0	88.6	78.3	86.1	82.1	91.7	80.4	89.4	85.9	95.5	79.2	86.4
Guatemala	All ages	65.6	70.3	69.3	75.2	59.9	63.0	66.8	65.5	70.9	71.1	66.4	63.5	84.7	84.0	84.7	85.1	84.0	81.4	67.8	71.9	74.3	79.3	65.6	68.4
Guinea	All ages	51.3	57.8	52.4	61.6	49.4	52.9	48.8	57.1	43.8	54.3	60.1	64.4	75.9	78.3	76.2	78.7	75.7	78.0	59.9	60.5	72.9	75.7	50.4	49.1
Guinea-Bissau	All ages	41.1	51.0	52.0	57.2	28.6	43.1	40.5	46.3	43.5	45.9	45.3	53.8	61.5	66.8	68.7	71.0	53.8	62.2	55.5	61.7	78.3	77.8	34.8	47.4
Guyana	All ages	21.5	40.9	30.9	48.6	10.6	31.2	26.9	36.8	37.6	47.9	26.6	34.1	57.6	64.0	67.1	71.1	44.3	52.1	24.2	39.2	36.4	46.7	20.1	38.6
Haiti	All ages	0.0	23.3	0.0	25.8	0.0	19.9	14.0	25.0	25.4	36.1	14.9	24.0	0.0	30.0	0.0	28.8	0.0	32.0	12.3	30.2	7.8	31.5	27.7	37.7
Honduras	All ages	47.0	35.8	52.5	40.1	39.4	30.0	43.2	24.4	51.4	32.9	42.9	28.1	46.9	42.7	49.6	40.8	42.5	46.2	54.1	37.8	64.9	48.6	48.3	33.2
Hungary	All ages	47.8	65.3	50.4	67.9	44.9	62.0	42.2	60.0	47.8	67.9	45.0	56.5	63.3	72.4	67.2	74.1	55.9	69.6	33.7	53.5	36.4	56.4	39.1	57.4
Iceland	All ages	71.7	83.6	71.7	81.0	70.9	85.0	73.4	80.2	76.0	82.0	73.6	80.1	78.0	87.5	75.3	85.3	82.4	90.2	62.3	78.5	62.6	75.4	67.3	84.5
India	All ages	50.7	54.9	54.4	57.7	46.2	51.3	53.5	57.8	61.0	63.0	52.0	57.9	54.5	63.2	60.9	65.2	48.1	61.4	57.5	55.4	59.6	58.5	61.0	58.3
Indonesia	All ages	50.7	48.0	54.2	53.9	46.0	40.8	48.8	41.5	54.5	47.8	50.6	43.6	61.4	66.2	63.4	67.6	58.6	64.5	66.1	56.4	72.2	65.2	65.0	53.2
Iran (Islamic Republic of)	All ages	64.6	72.5	68.8	73.7	59.3	70.2	67.6	74.4	73.5	78.0	65.4	73.5	73.2	88.7	76.1	90.0	69.1	86.5	49.8	58.9	56.2	61.0	50.0	62.3
Iraq	All ages	40.5	55.6	47.7	62.1	32.3	48.2	47.7	60.0	56.0	65.8	46.8	59.5	64.5	80.9	69.3	82.7	56.9	78.1	26.9	41.3	34.9	50.7	27.2	39.4
Ireland	All ages	62.3	78.1	61.7	78.1	62.5	77.5	71.7	82.4	74.0	84.3	72.8	81.7	70.7	82.4	70.2	82.2	71.5	82.1	45.5	68.9	43.2	68.6	54.3	74.1
Israel	All ages	72.1	86.1	70.0	84.8	73.0	86.5	56.3	70.4	60.4	75.3	58.1	68.8	86.1	90.1	85.2	91.4	87.0	87.6	70.4	86.8	67.5	84.5	76.6	91.4
Italy	All ages	51.1	63.6	51.3	61.5	50.7	65.5	58.4	62.1	64.7	66.4	57.7	62.6	73.2	74.7	72.7	76.0	73.9	72.3	31.4	54.2	28.4	48.5	42.4	65.9
Jamaica	All ages	37.6	45.2	41.6	49.4	32.6	39.9	43.3	47.0	51.8	56.9	42.7	44.0	71.1	77.6	72.7	77.8	67.8	76.4	17.8	29.1	17.5	29.3	28.2	37.8
Japan	All ages	70.6	82.8	71.5	82.4	68.7	82.3	78.5	82.1	83.2	85.1	75.7	80.8	72.5	91.0	73.6	89.8	70.6	91.7	60.1	76.6	59.1	75.3	65.8	80.9
Jordan	All ages	62.2	81.4	60.9	80.5	62.8	80.8	55.5	75.4	59.6	77.7	57.8	75.5	88.1	98.8	88.1	97.9	88.1	100.0	54.1	74.3	52.4	74.1	61.8	78.2
Kazakhstan	All ages	59.4	59.2	62.2	64.4	55.7	52.0	57.2	50.2	60.5	56.8	60.1	50.4	60.9	61.3	64.1	64.0	57.0	58.4	51.6	55.1	56.2	62.0	53.3	53.3
Kenya	All ages	69.2	68.4	73.5	73.6	63.1	61.2	57.7	53.2	61.8	57.1	58.7	55.2	83.4	84.7	84.7	86.1	82.7	83.9	80.5	78.5	84.3	83.1	80.4	77.7
Kiribati	All ages	59.6	59.9	69.4	70.0	49.7	49.5	62.7	59.7	67.4	65.6	62.3	59.1	65.9	73.0	75.9	80.0	52.6	65.8	77.3	76.3	87.0	86.4	71.9	70.4
Kuwait	All ages	89.0	92.6	88.1	100.0	88.4	85.8	86.8	91.4	88.9	100.0	85.4	85.2	87.9	100.0	85.2	100.0	90.9	99.6	86.0	87.6	86.0	99.5	88.2	82.7
Kyrgyzstan	All ages	47.3	60.6	51.4	69.4	41.9	49.6	48.5	60.0	50.8	66.8	53.5	58.0	60.4	61.9	67.2	69.3	51.2	53.9	39.9	52.1	45.9	65.1	40.6	44.0

Lao People's Democratic Republic	All ages	17.9	44.9	20.7	49.6	14.4	39.3	21.9	43.4	28.0	48.3	27.5	46.1	35.0	65.7	39.6	68.3	28.8	62.5	38.4	53.5	43.3	61.6	41.0	51.9
Latvia	All ages	37.5	49.5	38.3	47.4	37.6	53.8	38.6	49.9	43.1	57.3	43.9	49.3	63.6	63.7	70.2	68.7	52.9	56.1	14.3	28.8	13.3	22.5	27.1	46.0
Lebanon	All ages	49.0	75.9	55.2	77.8	42.1	72.9	40.1	68.5	57.9	75.4	30.2	65.1	53.9	83.6	64.5	87.4	37.2	76.8	47.5	70.6	48.4	72.5	52.9	73.4
Lesotho	All ages	44.1	33.2	55.2	44.9	23.2	12.5	23.9	13.6	35.9	23.1	22.8	17.5	79.2	71.7	84.3	76.9	70.4	63.8	58.1	48.7	66.1	56.7	50.0	41.5
Liberia	All ages	52.4	59.8	55.8	61.8	48.2	56.8	51.2	55.3	46.7	51.3	60.3	64.5	76.1	78.3	76.6	78.5	75.9	78.4	58.3	65.4	74.3	77.7	47.1	56.9
Libya	All ages	65.4	70.1	65.7	71.7	64.4	67.5	63.1	65.6	65.0	66.6	66.1	69.9	72.0	82.8	72.9	80.6	70.8	85.5	62.3	61.1	64.1	66.3	66.0	61.1
Lithuania	All ages	56.9	59.9	59.7	61.5	53.1	58.3	56.6	59.5	60.1	64.6	59.1	59.7	65.7	64.5	72.8	70.5	54.4	54.5	40.8	44.9	44.1	45.8	45.1	52.1
Luxembourg	All ages	42.9	72.0	42.2	71.8	44.1	71.6	52.3	67.6	57.6	72.6	54.1	66.6	69.9	83.2	68.1	81.9	72.8	84.7	19.9	65.6	16.5	64.4	33.3	71.5
Madagascar	All ages	44.1	54.5	55.8	61.7	30.4	45.3	36.8	40.4	46.7	46.2	36.1	42.8	63.2	69.6	71.8	73.5	55.6	66.6	68.4	75.0	77.7	81.2	62.5	72.1
Malawi	All ages	51.9	54.2	55.6	60.5	46.8	46.3	35.0	32.2	38.4	37.5	41.0	36.4	70.6	72.9	72.9	77.6	68.6	69.0	71.1	67.7	75.7	73.6	70.8	66.4
Malaysia	All ages	59.9	68.6	63.4	71.7	55.4	64.5	59.7	61.7	63.8	65.7	60.6	62.4	74.6	82.6	76.3	82.1	72.1	83.0	64.8	72.3	68.4	75.8	66.6	73.5
Maldives	All ages	49.6	71.4	49.9	72.3	47.7	69.1	49.5	70.2	50.1	66.6	54.0	73.0	64.8	86.7	65.6	85.6	64.1	88.2	64.6	70.8	69.8	74.1	65.2	72.1
Mali	All ages	53.5	62.2	52.1	61.9	54.6	61.4	48.4	57.7	42.5	49.3	60.9	68.4	74.7	80.0	71.5	76.6	77.9	83.2	68.2	69.8	75.7	79.6	64.4	63.6
Malta	All ages	57.6	73.4	58.9	72.1	55.6	74.2	63.7	76.9	66.6	78.4	65.6	77.7	87.0	94.0	85.0	93.2	89.6	94.2	40.1	62.3	41.7	59.0	46.0	71.0
Marshall Islands	All ages	45.1	49.9	58.0	59.8	31.0	38.9	42.9	46.4	55.5	56.0	39.2	44.6	55.3	63.0	63.6	66.8	45.9	58.7	71.5	75.6	78.3	82.5	69.1	72.8
Mauritania	All ages	56.0	64.0	56.8	66.1	54.9	61.0	50.1	58.8	41.6	52.5	64.8	70.2	77.6	85.6	77.7	83.9	77.5	87.5	62.3	64.5	73.8	76.7	53.4	54.6
Mauritius	All ages	53.2	66.1	62.0	73.0	43.1	57.6	52.9	60.9	63.2	69.1	49.3	58.1	66.4	72.3	75.4	78.1	52.6	63.0	48.4	63.7	57.4	70.6	46.3	62.2
Mexico	All ages	74.0	79.6	78.2	84.5	67.3	72.4	73.6	73.6	78.6	80.6	70.4	68.5	81.7	77.7	81.7	80.3	80.8	72.7	67.8	78.0	75.1	85.2	64.5	74.6
Micronesia (Federated States of)	All ages	41.3	49.9	49.5	58.6	32.1	40.2	41.1	47.0	49.2	55.1	41.4	46.3	50.6	64.3	57.0	68.0	43.1	60.2	66.3	71.6	70.9	77.3	66.9	70.5
Monaco	All ages	51.1	66.5	49.7	64.4	53.6	69.2	53.9	69.1	60.4	73.0	53.9	69.0	64.2	84.0	62.8	81.0	67.6	88.4	33.3	53.5	28.4	49.0	47.6	64.9
Mongolia	All ages	42.9	59.5	51.2	68.9	31.6	47.9	43.8	58.9	53.4	66.9	41.3	55.7	42.7	42.7	49.8	49.6	34.2	35.0	84.5	85.5	91.9	94.9	79.3	78.9
Montenegro	All ages	33.8	20.5	37.5	20.4	29.8	21.5	34.7	12.1	42.7	22.4	36.1	16.1	75.3	74.9	75.4	72.6	75.1	78.4	58.9	41.3	63.5	42.4	60.3	47.9
Morocco	All ages	46.5	54.6	42.5	51.3	49.9	57.0	46.3	52.0	47.5	51.5	53.8	59.6	62.6	73.4	63.7	72.5	60.9	74.8	36.3	39.4	32.2	35.7	47.4	49.6
Mozambique	All ages	43.0	45.2	54.0	60.3	30.0	28.9	29.5	30.8	41.0	45.4	28.4	27.7	67.1	64.5	75.9	77.2	58.6	53.2	62.2	60.9	70.6	72.1	58.2	54.4
Myanmar	All ages	19.4	38.3	25.4	47.9	12.8	27.9	22.4	34.0	27.6	41.9	28.0	35.3	40.6	62.8	49.1	69.1	29.4	54.7	40.5	48.7	50.5	58.9	37.9	45.1
Namibia	All ages	52.3	54.5	59.3	62.2	42.3	43.2	36.1	35.6	38.6	41.1	42.6	39.4	83.0	85.3	84.6	87.3	81.1	82.7	61.6	59.0	70.7	67.3	54.8	53.8
Nauru	All ages	49.4	50.8	58.7	58.2	39.9	41.5	47.8	42.5	56.7	50.1	46.5	43.2	52.3	57.4	54.1	57.4	50.1	57.4	72.5	74.1	83.3	81.5	67.2	70.3
Nepal	All ages	40.8	50.8	39.2	55.8	41.7	44.6	46.8	53.7	51.5	61.5	49.6	51.6	50.5	64.9	54.7	69.4	45.8	58.9	45.3	48.3	41.8	54.9	54.9	48.6
Netherlands	All ages	71.9	76.8	71.7	75.5	71.6	77.9	67.3	72.9	71.9	75.7	66.6	73.1	72.7	82.9	73.0	83.0	71.8	81.8	64.2	70.2	63.5	68.2	70.5	76.9
New Zealand	All ages	65.2	76.8	64.8	74.9	65.4	78.0	65.4	71.2	70.0	75.6	64.9	70.0	58.1	70.6	59.0	72.4	56.9	67.1	55.4	71.6	54.1	68.8	63.1	78.6
Nicaragua	All ages	67.9	77.7	73.2	81.8	59.9	71.1	63.6	71.1	70.4	77.0	60.3	67.4	75.4	85.1	76.3	84.7	73.3	84.7	69.2	76.1	76.9	82.6	65.6	73.5
Niger	All ages	58.3	61.8	63.2	65.3	52.1	56.9	54.4	56.4	55.0	55.2	58.9	62.9	75.6	80.7	78.2	82.1	73.4	79.4	73.4	72.1	83.5	82.7	66.3	64.0
Nigeria	All ages	52.4	71.3	57.2	77.5	46.9	62.6	48.5	66.9	46.5	67.4	55.3	70.3	80.0	88.2	83.7	90.1	77.1	85.8	58.4	71.3	71.1	85.7	51.0	57.9
Niue	All ages	50.1	57.2	54.9	63.4	44.2	49.4	43.8	46.0	50.9	55.0	44.9	44.7	64.1	72.5	63.5	70.2	66.3	76.2	60.8	68.3	63.2	73.3	64.9	68.4
North Macedonia	All ages	36.9	36.5	39.9	35.2	33.2	37.8	40.7	38.0	49.1	43.8	40.7	41.1	48.5	55.2	49.5	52.6	47.4	59.9	19.0	13.2	22.4	8.8	24.7	27.3
Northern Mariana Islands	All ages	66.0	66.0	69.6	71.9	62.0	60.1	62.6	57.5	63.7	61.4	64.2	58.2	73.2	78.6	67.4	74.3	77.8	82.7	84.1	78.5	89.1	85.4	83.1	76.9
Norway	All ages	68.0	83.8	66.4	82.0	68.9	84.2	67.3	76.2	73.4	77.7	64.5	77.1	69.3	86.7	66.7	86.4	74.1	86.5	56.8	79.8	53.4	78.1	65.4	84.5
Oman	All ages	64.8	86.6	66.2	86.0	62.5	85.9	64.1	84.3	69.2	85.0	63.4	84.5	79.7	93.3	79.5	90.8	80.2	96.3	58.7	80.8	59.5	81.7	62.5	82.5
Pakistan	All ages	58.3	63.0	59.1	64.3	56.3	60.7	60.4	62.7	64.3	65.5	61.2	64.4	60.2	63.4	63.4	64.1	57.0	62.9	61.3	65.3	62.8	69.2	65.0	66.7
Palau	All ages	56.5	64.6	70.0	75.4	42.2	54.0	52.8	57.1	67.0	69.7	46.6	52.4	70.3	79.8	74.1	81.0	65.9	78.7	69.6	75.2	78.9	82.3	65.1	73.2
Palestine	All ages	34.6	62.7	40.2	66.3	27.5	57.9	38.5	62.7	48.6	70.0	36.7	59.9	82.2	94.8	83.2	94.3	80.1	94.9	14.0	47.6	20.4	51.7	16.8	50.4
Panama	All ages	65.0	69.9	70.5	77.1	58.0	60.6	64.7	67.7	72.7	78.2	60.8	61.2	78.5	77.2	78.0	79.3	78.8	73.0	59.0	63.9	65.5	71.7	58.3	61.4
Papua New Guinea	All ages	26.7	37.0	22.2	34.5	28.9	37.3	13.5	22.4	3.9	15.2	28.1	34.1	48.2	60.6	43.2	56.5	52.7	64.2	69.5	71.0	67.9	71.6	75.7	75.0
Paraguay	All ages	49.1	58.1	50.9	61.7	46.4	53.5	49.9	51.1	55.3	60.0	51.3	48.9	56.3	64.6	55.6	62.7	57.4	67.5	45.3	53.6	49.3	58.3	48.4	55.4
Peru	All ages	63.5	77.0	69.4	80.6	55.6	71.9	61.2	68.4	68.5	74.2	58.3	66.4	66.6	80.0	69.4	78.5	62.3	81.6	66.9	76.7	74.2	82.6	64.3	74.9
Philippines	All ages	50.6	56.5	61.2	65.9	38.0	45.1	38.7	50.4	52.3	60.1	34.1	47.6	71.0	73.0	75.7	76.0	64.4	68.8	65.5	66.2	74.8	74.4	61.1	62.7
Poland	All ages	30.8	59.1	33.3	61.3	28.8	56.7	44.4	55.1	51.6	64.8	45.3	51.0	61.5	70.3	67.4	74.5	52.3	64.3	0.0	45.4	0.0	46.2	12.1	52.4
Portugal	All ages	35.6	53.2	35.4	51.9	35.6	54.7	44.1	56.9	50.6	63.3	45.7	56.3	60.7	63.6	62.4	64.2	58.3	63.2	13.1	39.0	9.3	32.7	26.4	52.9

Puerto Rico	All ages	69.8	81.3	76.3	86.2	60.6	73.3	71.0	73.9	79.5	81.6	65.4	68.1	86.0	92.0	87.0	93.6	81.9	86.4	59.0	77.7	66.5	83.6	56.7	75.3
Qatar	All ages	84.8	100.0	84.4	98.4	83.6	100.0	84.0	100.0	84.4	97.7	84.1	100.0	76.7	93.1	83.4	94.7	71.7	93.0	87.3	100.0	87.7	100.0	88.4	100.0
Republic of Korea	All ages	65.5	84.3	66.0	83.0	64.0	84.5	71.6	87.5	76.5	90.2	69.7	85.2	47.8	82.8	53.0	82.1	39.5	83.7	64.5	79.1	64.0	77.0	69.5	83.8
Republic of Moldova	All ages	40.1	54.0	41.4	56.8	38.7	50.6	35.5	41.6	39.5	47.2	41.2	44.2	71.5	72.0	77.5	77.7	63.5	64.9	43.1	46.7	43.9	49.5	50.4	51.0
Romania	All ages	38.5	44.4	39.3	46.2	37.2	42.6	32.5	31.4	37.3	41.7	37.6	31.1	55.4	48.5	57.4	52.6	53.0	43.7	26.2	30.1	26.6	29.7	34.6	39.3
Russian Federation	All ages	35.0	47.2	36.5	50.4	34.7	43.8	45.9	49.1	49.3	56.1	51.2	49.1	62.7	54.7	68.2	59.4	55.5	48.7	5.2	27.5	6.6	30.2	16.5	34.3
Rwanda	All ages	33.5	58.8	42.5	66.0	21.2	48.2	28.2	42.5	39.6	52.8	26.8	39.6	61.9	81.2	69.7	85.2	52.9	77.0	62.7	73.2	66.9	77.7	63.6	72.7
Saint Kitts and Nevis	All ages	27.3	49.7	32.1	57.2	20.9	40.6	30.1	44.1	39.8	57.3	30.2	39.5	62.2	75.5	64.3	78.3	57.8	70.6	12.9	42.5	16.6	45.9	18.9	46.2
Saint Lucia	All ages	36.6	52.2	42.6	58.9	28.6	43.8	39.8	49.2	52.1	63.6	35.5	42.8	66.9	79.3	67.4	79.9	65.4	77.0	21.6	40.6	22.4	42.2	31.1	46.6
Saint Vincent and the Grenadines	All ages	40.6	49.6	44.8	57.6	34.9	39.9	31.1	42.9	42.2	56.2	30.0	38.4	75.2	79.0	75.1	80.3	74.7	76.1	34.8	43.3	35.5	47.9	43.4	45.7
Samoa	All ages	53.9	59.0	63.4	64.8	42.8	51.7	48.1	49.8	60.1	58.0	43.4	48.6	65.5	73.5	68.2	72.3	62.5	75.4	73.7	75.9	79.2	81.7	72.6	74.3
San Marino	All ages	64.8	78.0	62.7	76.2	66.4	79.3	63.9	76.9	66.1	77.7	67.3	78.9	86.4	96.7	85.5	94.6	87.4	98.9	52.6	69.6	49.6	67.8	61.0	75.8
Sao Tome and Principe	All ages	67.7	72.0	68.9	73.4	66.1	69.7	63.5	67.8	60.7	65.6	72.4	74.4	85.0	85.5	85.3	84.8	84.7	86.3	71.2	72.9	78.5	80.6	66.4	68.2
Saudi Arabia	All ages	45.6	68.8	43.5	71.3	45.6	65.5	43.7	65.0	47.6	70.8	46.8	63.7	77.7	88.0	77.4	86.2	78.2	89.9	39.1	63.3	35.2	67.4	47.6	64.9
Senegal	All ages	57.7	61.4	64.1	66.3	49.8	55.1	52.3	56.2	50.9	52.5	59.1	64.4	75.2	80.7	78.1	81.7	72.4	79.6	63.2	63.9	79.3	77.8	50.3	52.5
Serbia	All ages	32.3	35.5	32.6	35.3	32.0	36.0	38.8	37.0	45.5	47.4	41.1	34.6	44.9	53.5	46.0	52.6	43.8	56.0	9.7	8.9	7.7	4.3	21.8	23.7
Seychelles	All ages	54.4	67.8	62.2	71.3	45.7	63.4	51.5	61.8	58.1	65.3	51.5	62.7	73.4	86.8	77.3	87.6	67.9	85.6	53.6	67.0	63.2	71.4	50.4	67.9
Sierra Leone	All ages	52.4	60.2	59.7	63.3	44.1	56.1	49.9	57.3	48.3	53.7	56.8	66.3	74.6	77.7	77.2	77.6	72.2	78.0	55.9	63.1	75.7	76.8	40.7	52.3
Singapore	All ages	77.3	95.3	76.4	93.9	76.9	95.6	81.5	95.8	84.4	97.0	80.0	93.7	79.7	95.8	78.7	93.1	81.2	99.1	71.6	93.2	69.2	92.2	77.3	95.8
Slovakia	All ages	58.7	70.4	61.5	72.0	55.2	67.9	38.1	52.7	47.8	61.0	37.4	50.6	66.0	75.7	68.3	76.7	62.8	74.1	51.4	62.9	54.2	65.0	55.3	66.7
Slovenia	All ages	48.2	66.4	52.6	67.3	43.0	65.1	55.5	64.7	63.2	71.0	53.6	62.3	70.5	80.9	72.5	80.8	66.9	81.0	26.7	54.5	30.5	54.5	31.7	61.1
Solomon Islands	All ages	54.3	56.2	63.6	63.8	44.3	46.5	56.1	56.4	65.0	64.5	52.9	53.7	66.6	70.9	70.9	73.7	61.5	67.5	76.9	75.4	82.2	79.6	75.8	75.6
Somalia	All ages	44.0	55.3	54.6	63.4	31.7	44.8	34.6	38.1	44.2	46.0	34.5	39.1	64.4	73.0	74.3	79.9	55.9	67.3	76.0	79.7	80.1	83.6	76.2	79.8
South Sudan	All ages	73.2	65.1	58.2	65.6	32.7	43.3	59.4	41.2	42.9	44.6	26.9	31.9	87.0	86.7	77.9	80.0	67.7	72.1	79.0	67.9	75.7	82.3	66.9	71.4
Spain	All ages	45.1	55.1	55.6	74.1	54.9	80.9	29.0	33.1	65.2	72.0	62.6	66.8	71.8	75.6	80.9	79.9	75.7	74.3	68.7	74.8	37.4	68.7	49.3	83.8
Sri Lanka	All ages	55.4	78.2	62.8	62.2	43.2	50.9	61.2	67.5	59.5	53.8	43.6	44.7	79.1	77.9	85.6	88.2	77.7	84.0	39.3	75.6	59.2	54.8	45.1	50.9
Sudan	All ages	53.4	57.2	18.4	59.0	30.4	60.7	47.5	45.1	25.0	56.2	26.8	61.2	82.1	86.7	46.7	70.8	41.4	70.7	48.4	49.6	23.2	53.6	44.8	57.6
Suriname	All ages	25.2	60.5	48.0	55.6	37.4	39.0	19.7	55.8	50.8	52.5	41.8	37.4	44.4	70.7	69.5	75.0	61.4	66.3	31.4	52.7	49.0	54.6	49.2	51.9
Sweden	All ages	43.3	48.2	64.9	78.9	69.1	83.1	42.4	40.9	73.3	81.2	73.2	83.2	66.4	71.9	68.2	82.3	78.0	89.3	45.6	50.0	51.4	72.9	64.5	81.8
Switzerland	All ages	67.3	81.5	58.1	77.8	62.0	82.5	71.6	81.5	68.8	81.1	69.5	81.6	72.1	85.3	67.9	82.1	73.0	83.9	55.3	75.7	40.5	70.4	55.4	81.0
Syrian Arab Republic	All ages	60.0	80.4	55.6	64.5	49.4	56.5	67.0	80.4	60.4	65.7	54.2	57.9	69.6	83.0	75.9	91.5	70.0	87.6	44.4	73.9	54.0	55.8	57.4	54.5
Taiwan (Province of China)	All ages	52.9	60.9	68.1	87.2	57.4	75.2	54.3	59.1	66.7	80.7	56.6	69.5	73.3	90.0	82.3	85.9	81.4	79.3	52.9	52.0	71.7	89.0	68.1	81.8
Tajikistan	All ages	63.2	81.9	50.6	63.2	39.1	52.0	58.8	73.5	51.0	56.7	50.4	55.1	81.9	83.4	67.3	75.7	70.7	76.1	67.2	83.3	50.5	64.1	34.2	51.9
Thailand	All ages	45.6	58.4	67.3	72.4	54.1	58.9	46.8	52.9	65.4	64.9	62.1	58.6	68.8	75.7	61.9	67.2	39.2	49.9	39.7	55.3	75.8	77.1	66.9	70.3
Timor-Leste	All ages	61.5	66.4	39.1	42.9	36.8	33.6	61.4	59.0	38.5	36.5	42.3	37.2	52.2	59.5	57.9	64.6	53.6	58.8	69.0	71.4	65.4	57.6	62.0	47.2
Togo	All ages	38.5	38.7	63.4	63.7	56.0	52.8	35.6	31.9	52.7	50.7	64.5	60.3	55.9	62.0	77.5	80.2	74.6	74.8	60.9	49.0	80.4	78.5	57.5	54.9
Tokelau	All ages	60.3	58.9	48.3	59.4	22.9	41.1	56.2	52.7	48.4	51.4	24.6	35.7	76.0	77.6	57.5	66.2	58.2	71.7	67.5	65.3	63.3	71.8	51.1	61.6
Tonga	All ages	37.8	51.8	72.6	70.6	61.7	63.3	33.0	39.7	65.5	62.4	57.7	57.9	57.6	68.3	76.6	78.5	82.5	87.3	55.1	64.7	85.7	78.7	75.0	74.2
Trinidad and Tobago	All ages	68.0	67.5	57.1	64.7	45.4	51.5	59.0	57.3	57.4	63.6	45.8	45.7	79.1	82.1	69.3	73.2	57.8	59.0	78.4	74.2	51.4	56.8	41.3	50.6
Tropical Latin America	All ages	52.0	58.7	52.7	67.1	38.3	57.8	48.6	51.8	58.4	64.4	44.2	51.1	65.1	68.0	55.5	59.9	45.8	57.3	42.7	50.4	47.6	64.9	38.7	59.7
Tunisia	All ages	53.8	61.8	55.2	63.6	51.2	59.0	54.2	61.6	59.0	65.3	55.5	62.8	74.5	84.0	77.2	85.2	70.7	81.9	41.8	47.4	44.0	50.5	46.5	51.0
Turkey	All ages	58.7	67.9	62.5	69.9	54.0	65.1	59.7	66.3	65.0	71.2	59.7	65.5	48.6	74.0	59.7	78.9	33.2	66.6	56.0	60.3	60.3	62.4	57.8	64.1
Turkmenistan	All ages	57.6	55.6	62.2	64.4	51.2	44.9	56.3	48.1	60.5	57.3	57.6	46.2	62.3	53.8	66.3	62.5	57.4	45.3	53.0	54.1	59.6	63.1	51.8	50.6
Tuvalu	All ages	32.1	48.4	41.3	54.8	20.0	40.8	32.5	42.0	43.2	49.1	31.5	43.2	43.3	64.4	49.0	65.0	35.0	64.2	60.9	65.1	68.3	70.4	57.9	64.9
Uganda	All ages	55.1	68.3	67.7	76.4	40.5	57.4	35.1	51.4	49.4	60.2	31.5	49.5	72.0	80.8	81.0	86.2	64.3	76.1	75.4	80.0	81.2	84.7	73.5	78.7
Ukraine	All ages	46.7	55.8	46.6	58.4	48.2	52.5	53.9	58.0	58.3	65.0	56.0	56.0	51.1	58.3	53.2	62.4	48.9	53.5	24.7	39.6	22.8	41.9	38.0	45.5
United Arab Emirates	All ages	81.1	92.4	79.9	93.2	80.2	90.5	80.5	90.0	81.5	90.8	80.6	89.1	76.0	90.8	73.5	87.2	78.5	93.9	78.6	90.2	78.2	93.8	81.0	90.4
United Kingdom	All ages	59.4	77.5	58.8	76.9	60.6	77.6	65.9	69.5	68.8	72.4	67.8	70.6	63.0	74.1	62.1	74.2	65.3	73.8	43.0	74.1	41.6	74.1	52.8	78.8

United Republic of Tanzania	All ages	56.2	69.1	64.9	73.2	46.1	63.5	41.7	55.1	51.0	59.3	40.8	56.6	75.3	82.9	80.8	84.7	70.6	81.5	75.7	74.5	81.1	80.5	74.4	72.6
United States Virgin Islands	All ages	57.1	70.4	64.5	78.3	46.2	60.5	51.3	61.3	62.8	73.7	45.5	53.7	70.2	86.1	73.7	90.5	62.0	77.0	55.0	66.0	60.4	72.8	55.0	64.3
United States of America	All ages	80.2	91.4	80.5	90.0	79.1	92.4	75.1	80.1	78.8	83.1	73.9	79.1	71.2	77.4	73.0	81.4	66.5	69.0	75.8	92.3	77.1	91.8	79.3	95.8
Uruguay	All ages	61.9	67.2	63.6	68.1	59.7	66.2	64.2	71.8	72.5	78.6	59.2	66.6	56.0	65.6	59.4	67.0	50.2	63.3	53.4	57.1	53.4	56.2	59.9	64.9
Uzbekistan	All ages	56.5	66.8	61.2	72.3	50.2	59.6	46.9	62.5	51.6	67.1	49.6	62.1	78.1	68.3	80.6	75.2	75.3	61.0	59.2	64.4	66.6	72.5	56.9	61.3
Vanuatu	All ages	53.8	59.6	66.5	69.1	40.4	48.6	48.6	52.7	61.6	62.2	43.7	49.8	57.8	67.4	64.6	71.1	50.3	63.4	75.9	78.5	85.7	86.4	70.6	74.4
Venezuela (Bolivarian Republic of)	All ages	64.3	62.9	70.0	70.8	56.1	51.6	61.5	56.9	69.1	68.9	57.8	48.6	74.5	75.4	75.8	77.5	71.3	69.9	66.1	61.3	72.8	69.1	64.0	58.4
Viet Nam	All ages	33.0	41.3	41.8	51.0	23.3	31.7	33.8	37.5	42.4	49.0	34.9	35.7	62.3	71.6	69.8	77.4	51.8	64.3	39.2	41.8	47.2	50.0	38.5	41.7
Yemen	All ages	25.4	46.3	24.2	48.4	26.1	43.3	23.0	43.9	31.5	47.7	26.0	48.4	47.4	65.3	54.2	69.3	37.8	59.8	28.5	34.5	23.0	39.3	41.2	37.5
Zambia	All ages	49.3	56.3	57.3	64.0	39.1	46.5	36.4	41.0	43.9	45.3	37.8	44.1	70.5	75.3	73.4	78.2	67.9	73.2	73.3	71.0	78.9	76.4	71.6	69.5
Zimbabwe	All ages	63.3	50.6	69.3	57.9	54.6	38.9	32.3	0.0	35.3	0.0	38.9	17.0	78.3	66.6	79.1	69.0	77.7	64.1	71.9	65.8	78.9	74.0	67.8	58.4
Afghanistan	Age-standardized	21.5	33.3	27.2	33.1	13.6	32.6	35.5	39.8	50.2	44.6	17.6	36.2	44.0	54.9	56.8	57.8	31.2	50.0	31.6	34.2	37.4	41.0	24.5	27.7
Albania	Age-standardized	33.0	43.0	41.6	45.9	18.3	38.9	33.2	38.9	43.4	43.8	19.6	35.3	71.5	80.4	71.2	77.3	68.8	81.3	60.7	64.6	71.4	70.7	42.9	57.5
Algeria	Age-standardized	51.8	64.6	43.7	59.0	54.7	67.0	59.6	65.5	56.6	61.5	61.3	69.0	73.6	83.1	70.4	78.3	74.2	84.7	48.0	60.4	44.8	59.4	47.3	59.5
American Samoa	Age-standardized	59.5	63.2	64.9	66.7	53.1	58.5	56.9	57.3	61.5	61.3	52.2	53.4	76.1	78.2	69.2	70.2	81.8	84.6	76.1	78.7	80.6	81.7	70.8	75.2
Andorra	Age-standardized	78.6	87.9	76.6	86.0	80.0	89.4	81.6	88.5	81.3	87.7	82.3	89.6	78.9	90.3	69.5	82.8	86.1	95.5	75.5	85.0	76.4	85.2	74.3	84.5
Angola	Age-standardized	51.2	59.5	60.0	66.0	38.5	48.3	40.7	44.6	46.6	48.6	35.0	41.3	75.9	82.3	76.5	81.0	74.0	80.9	70.6	70.8	79.1	78.6	56.9	57.0
Antigua and Barbuda	Age-standardized	46.5	61.8	57.8	70.0	31.1	50.7	47.4	58.5	59.2	69.6	34.9	46.4	71.6	82.9	74.4	81.4	63.4	80.5	53.6	64.4	64.9	72.7	38.1	53.7
Argentina	Age-standardized	63.4	81.3	66.9	84.2	58.7	77.4	71.5	80.3	77.4	85.9	64.6	73.6	56.7	80.6	55.3	77.8	56.1	80.5	66.9	83.8	71.5	86.7	61.5	79.8
Armenia	Age-standardized	60.1	73.2	63.2	76.0	55.2	69.8	56.5	73.4	60.4	79.2	53.3	66.4	71.9	82.7	71.0	83.9	70.8	79.5	60.8	69.0	68.2	74.4	51.1	63.2
Australia	Age-standardized	72.4	89.1	71.8	87.6	72.6	90.1	74.9	84.4	76.5	85.6	73.4	83.3	70.8	85.5	68.2	83.9	70.4	83.2	69.8	88.4	71.7	87.6	68.1	88.5
Austria	Age-standardized	68.9	95.4	69.8	95.3	67.8	94.9	83.1	92.8	84.4	93.1	82.1	92.5	90.1	98.3	85.5	94.6	91.8	98.1	57.7	92.5	61.9	93.7	54.4	90.5
Azerbaijan	Age-standardized	53.8	60.5	60.4	64.1	41.7	55.1	51.8	55.9	57.4	58.8	45.2	53.5	86.6	91.3	85.6	89.1	84.5	90.6	68.6	72.0	77.7	78.4	51.5	62.6
Bahamas	Age-standardized	55.9	68.5	62.2	73.2	47.4	61.9	53.6	65.2	61.9	72.2	44.8	57.4	76.7	82.1	75.0	81.0	73.8	78.7	64.6	72.4	71.0	76.7	57.9	67.6
Bahrain	Age-standardized	36.1	58.0	40.8	60.8	30.2	55.2	49.7	68.0	56.9	72.4	43.5	64.5	64.3	84.2	62.6	82.4	64.2	84.1	32.2	50.7	43.8	58.3	19.7	43.8
Bangladesh	Age-standardized	27.9	42.9	27.4	42.7	25.6	41.3	46.1	56.1	50.6	59.6	41.5	52.8	37.1	61.1	42.5	57.5	36.7	64.0	40.7	45.4	41.1	49.5	38.0	40.8
Barbados	Age-standardized	51.2	64.5	56.5	68.5	45.1	59.0	57.5	65.4	65.3	72.6	49.2	57.2	72.9	79.1	73.2	77.9	68.5	76.9	47.6	62.1	55.6	67.8	41.3	56.2
Belarus	Age-standardized	58.5	65.2	60.6	70.2	56.4	58.8	66.8	68.0	69.0	74.2	64.9	60.8	67.2	68.8	68.5	73.7	62.2	60.5	52.9	61.7	60.0	70.1	46.4	51.2
Belgium	Age-standardized	63.0	84.8	65.5	86.0	59.3	82.8	70.1	80.1	74.1	84.0	65.5	75.5	78.2	82.6	74.0	80.0	80.2	81.8	58.8	84.9	65.1	87.3	51.5	81.9
Belize	Age-standardized	56.5	64.7	61.6	68.9	49.6	59.1	53.0	59.8	58.7	65.0	47.4	54.7	78.4	85.2	78.7	83.2	73.9	83.8	66.8	69.7	73.2	74.8	59.0	63.4
Benin	Age-standardized	50.2	55.6	50.7	58.2	48.6	52.1	51.5	57.1	42.5	51.4	60.3	64.3	79.6	84.8	74.8	80.4	81.9	86.6	61.9	62.4	73.9	74.5	45.0	44.4
Bermuda	Age-standardized	66.2	84.5	73.3	92.2	55.7	74.1	71.5	85.4	79.0	94.4	61.8	74.5	84.7	95.2	86.5	97.7	76.8	87.0	60.1	79.5	70.3	87.7	45.8	67.8
Bhutan	Age-standardized	49.3	61.3	47.5	61.9	50.0	59.5	57.2	65.8	59.4	67.5	55.5	64.4	58.8	71.8	54.1	66.8	61.8	74.8	57.7	63.5	60.3	68.5	55.4	57.5
Bolivia (Plurinational State of)	Age-standardized	38.6	56.9	41.9	59.1	33.6	53.5	40.0	52.4	44.4	56.1	37.0	49.4	46.4	60.3	39.4	51.5	52.2	68.5	51.5	63.3	61.3	70.4	39.2	54.8
Bosnia and Herzegovina	Age-standardized	53.9	66.0	54.5	67.2	53.4	64.3	52.4	65.3	57.0	71.0	48.7	59.3	53.0	75.7	52.1	73.5	52.3	75.5	49.6	60.7	54.9	65.5	45.6	55.9
Botswana	Age-standardized	53.7	69.6	59.3	74.0	44.6	61.6	42.2	59.1	40.8	60.5	43.8	58.9	83.7	92.4	82.8	90.1	81.8	91.3	66.4	74.7	74.9	81.4	52.2	62.0
Brazil	Age-standardized	46.0	67.8	51.6	71.7	38.8	62.5	53.1	64.0	61.0	71.1	44.5	55.8	57.7	63.1	54.2	58.1	58.2	65.6	46.5	70.2	57.4	77.2	33.4	61.5
Brunei Darussalam	Age-standardized	72.2	78.4	69.9	77.3	73.3	78.5	76.2	80.2	77.3	81.9	75.4	78.3	63.6	76.4	55.5	71.5	70.9	79.7	74.6	79.1	75.5	79.7	72.8	77.3
Bulgaria	Age-standardized	31.4	47.4	34.5	53.9	27.2	39.2	43.6	58.3	49.0	65.8	38.4	50.3	65.0	62.7	65.5	63.6	63.0	60.0	31.9	40.3	41.6	52.8	21.9	25.3
Burkina Faso	Age-standardized	56.8	58.0	67.6	68.1	42.9	44.9	54.7	57.1	59.3	61.0	50.5	53.7	84.5	86.8	88.0	89.4	79.4	82.5	71.7	69.2	86.4	83.6	48.5	46.2
Burundi	Age-standardized	38.8	52.7	49.9	60.8	22.1	42.3	32.8	38.6	45.3	47.8	19.4	29.8	65.3	78.3	70.6	81.0	59.2	75.7	64.6	70.5	71.2	76.5	55.6	62.4
Cabo Verde	Age-standardized	70.9	68.6	73.9	76.5	65.6	57.0	72.3	72.4	70.2	72.6	75.2	72.4	90.2	95.1	89.9	94.4	86.8	92.7	76.2	67.7	85.4	82.5	60.6	42.4
Cambodia	Age-standardized	26.5	39.6	27.1	43.8	25.5	33.7	34.1	43.9	35.6	48.1	35.5	40.6	54.7	69.7	51.4	66.8	55.9	70.6	50.6	53.0	58.5	61.9	41.8	41.9
Cameroon	Age-standardized	53.0	54.9	58.0	60.1	46.2	48.1	54.0	56.4	53.6	54.3	55.1	59.0	80.3	82.3	79.5	81.1	79.4	81.7	68.1	64.2	79.7	77.5	51.0	45.0
Canada	Age-standardized	83.1	98.2	84.1	97.5	81.4	98.6	87.4	95.6	89.6	95.9	84.7	95.2	81.9	90.2	79.8	88.8	79.4	87.0	79.9	96.4	82.6	96.3	76.5	95.9
Central African Republic	Age-standardized	35.9	39.3	46.2	49.3	20.5	23.3	31.1	28.9	37.8	36.0	24.7	21.8	65.4	69.3	68.0	71.7	61.5	65.3	60.5	61.3	69.9	70.6	46.2	46.3
Chad	Age-standardized	55.6	51.9	59.0	57.2	50.5	45.4	56.0	54.6	52.3	50.2	60.1	57.6	80.2	78.2	78.3	75.4	80.1	79.0	67.6	61.1	79.3	76.7	50.2	41.1
Chile	Age-standardized	64.8	83.3	67.5	85.9	60.7	79.4	73.7	88.4	79.3	92.2	66.1	83.3	70.7	80.2	66.9	77.9	72.2	79.1	63.3	81.4	67.9	85.2	57.8	76.1

China	Age-standardized	31.0	59.5	39.5	67.9	19.5	48.1	41.6	54.4	51.3	63.2	29.9	44.0	0.0	67.0	0.0	68.9	0.0	63.2	53.1	67.3	64.4	77.1	38.6	53.3
Colombia	Age-standardized	66.6	81.8	69.4	85.6	61.8	75.4	64.7	79.6	68.6	84.3	60.6	73.1	76.8	78.1	73.1	75.2	77.4	77.7	68.5	82.3	75.0	87.9	59.7	73.6
Comoros	Age-standardized	54.3	64.8	60.4	69.6	45.1	56.8	42.4	48.4	46.2	51.6	39.2	46.0	79.2	86.2	78.1	84.6	78.5	85.5	72.6	76.2	79.1	82.1	62.6	66.3
Congo	Age-standardized	50.4	58.5	55.9	61.9	41.3	52.7	40.2	42.6	41.1	41.7	40.7	45.7	75.3	81.7	73.1	76.7	75.3	83.9	68.2	69.6	76.0	76.6	55.2	58.9
Cook Islands	Age-standardized	60.5	79.5	67.8	84.2	52.0	73.5	57.3	71.6	64.9	78.3	49.9	64.9	74.5	93.2	69.1	88.4	78.5	95.3	75.4	87.0	81.8	90.0	67.5	82.7
Costa Rica	Age-standardized	76.6	85.6	79.8	89.4	71.5	79.4	77.0	85.9	80.1	90.3	73.1	80.3	87.5	87.5	83.9	86.0	87.6	84.8	75.0	83.1	80.7	87.9	66.8	75.7
Coted'Ivoire	Age-standardized	59.0	59.0	64.9	64.5	52.6	52.4	60.7	60.8	58.4	57.5	61.7	63.7	81.5	84.2	81.4	82.6	80.8	84.3	66.4	63.9	79.9	78.5	50.4	44.8
Croatia	Age-standardized	41.5	70.1	45.8	71.5	34.9	68.4	51.8	67.1	61.2	76.7	40.5	55.8	55.2	72.9	54.2	72.6	54.4	70.6	36.1	68.0	45.3	70.8	24.6	65.5
Cuba	Age-standardized	61.1	69.0	65.0	75.3	55.3	61.0	63.4	68.3	68.4	75.9	58.0	59.8	81.5	89.4	78.5	87.2	81.0	87.7	60.8	65.5	67.8	74.2	52.0	54.4
Cyprus	Age-standardized	34.7	66.4	33.7	67.6	35.6	65.7	60.8	80.4	65.1	82.4	56.9	78.5	60.1	85.3	53.8	80.8	65.2	87.9	18.1	55.5	22.6	60.6	17.3	52.8
Czechia	Age-standardized	47.2	80.7	51.3	82.5	41.7	78.4	59.6	80.2	67.3	85.4	51.3	73.9	63.5	83.2	61.9	81.4	62.7	82.1	37.9	78.0	47.8	81.5	27.0	74.0
Democratic People's Republic of Korea	Age-standardized	47.0	48.9	52.3	55.5	39.0	40.1	47.3	42.1	54.5	50.6	38.4	32.7	48.5	59.9	47.6	60.1	45.5	57.0	66.2	67.0	72.9	74.8	57.3	56.3
Democratic Republic of the Congo	Age-standardized	52.2	52.6	57.4	59.2	44.5	41.4	46.1	41.6	47.2	46.1	45.1	37.2	79.2	81.0	77.4	79.9	79.3	79.6	67.4	67.0	74.5	74.9	56.7	54.0
Denmark	Age-standardized	71.9	84.0	72.7	85.8	70.3	81.5	71.1	81.4	73.9	85.1	68.5	77.4	73.8	86.8	68.6	83.4	77.0	87.3	70.6	82.6	74.4	85.7	66.1	78.6
Djibouti	Age-standardized	57.9	65.1	66.0	73.0	46.8	55.4	43.9	48.1	48.6	51.7	39.0	44.5	79.6	84.7	82.3	86.1	76.6	83.0	76.0	75.6	82.4	83.7	65.8	63.6
Dominica	Age-standardized	40.0	48.1	50.1	56.9	23.9	38.8	40.8	46.9	52.5	58.1	26.7	36.1	72.6	77.2	74.7	78.9	63.6	71.5	42.8	47.4	56.0	58.8	24.6	37.4
Dominican Republic	Age-standardized	54.8	64.5	59.1	70.8	48.6	56.4	52.5	60.2	59.0	67.4	45.7	52.7	70.7	79.7	68.7	76.6	68.9	78.9	64.7	70.9	70.5	77.9	57.6	61.7
Ecuador	Age-standardized	66.0	78.6	70.5	82.2	59.4	73.1	59.3	73.2	64.6	77.8	54.0	68.1	81.0	74.9	78.6	72.2	80.1	74.7	69.6	81.2	76.4	86.5	60.3	73.6
Egypt	Age-standardized	4.7	42.6	1.3	29.3	4.4	45.4	13.3	52.0	25.8	52.1	0.0	49.5	42.5	75.0	54.2	74.5	28.3	72.3	0.0	31.9	0.0	22.4	0.4	31.3
El Salvador	Age-standardized	65.5	76.8	70.7	81.3	57.5	68.5	66.0	71.5	70.5	77.0	60.8	63.8	75.8	84.0	74.0	81.2	73.8	82.5	71.9	79.1	80.0	85.2	59.7	68.9
Equatorial Guinea	Age-standardized	44.4	65.1	53.8	69.4	29.3	57.1	33.9	54.2	39.7	55.7	28.5	53.7	71.1	89.8	73.7	86.0	66.9	90.5	66.9	71.5	75.9	78.5	51.0	59.4
Eritrea	Age-standardized	38.2	52.6	47.9	60.7	22.9	37.9	28.2	35.7	35.2	43.0	20.6	27.6	67.7	79.1	74.1	81.6	59.3	73.9	69.8	72.4	75.5	78.9	62.1	60.6
Estonia	Age-standardized	46.2	79.5	49.4	84.9	42.0	72.1	60.4	77.2	64.7	84.9	56.2	67.2	74.9	80.7	80.3	84.9	63.9	72.4	35.2	76.9	44.4	83.9	25.3	67.0
Eswatini	Age-standardized	46.4	49.8	56.0	56.3	29.4	39.9	31.0	34.8	38.0	36.9	24.5	34.3	81.3	82.0	82.1	80.9	77.1	80.4	63.9	62.8	73.7	70.9	45.9	51.2
Ethiopia	Age-standardized	45.0	61.7	54.5	67.9	33.0	53.2	43.1	51.0	51.3	56.2	35.0	45.9	76.9	86.3	80.4	86.8	73.6	85.0	76.0	77.6	80.1	82.0	70.5	70.9
Fiji	Age-standardized	59.1	63.1	65.2	68.0	49.4	54.8	49.4	47.8	57.4	55.3	40.0	38.8	72.6	75.8	68.6	71.3	75.8	81.8	78.3	78.5	84.4	83.8	68.3	69.9
Finland	Age-standardized	74.7	90.4	74.4	90.7	75.3	89.8	79.6	90.1	81.4	90.8	78.4	89.8	80.5	92.1	75.7	88.4	83.5	92.7	70.0	87.7	72.4	89.3	68.4	85.4
France	Age-standardized	67.0	88.9	69.5	89.0	63.4	88.3	69.7	85.2	75.3	87.2	63.7	83.2	81.9	88.2	78.7	85.9	83.3	87.3	63.5	87.9	68.6	89.0	57.2	86.1
Gabon	Age-standardized	60.4	65.9	68.1	72.1	48.2	56.2	49.3	52.4	54.5	55.6	43.8	49.3	83.9	88.4	84.2	87.2	80.9	86.7	74.8	73.9	81.4	80.7	63.3	62.7
Gambia	Age-standardized	57.0	52.3	62.3	57.6	50.7	45.4	55.6	51.6	51.1	47.5	59.6	56.8	82.3	80.5	81.0	77.5	82.0	81.4	66.3	59.5	80.0	74.3	47.8	37.8
Georgia	Age-standardized	47.7	54.0	56.2	63.1	32.5	40.2	55.9	61.3	61.3	67.4	49.3	54.2	73.2	65.7	73.9	66.4	70.3	64.4	57.2	52.6	69.1	66.5	33.9	30.1
Germany	Age-standardized	72.6	91.4	74.6	92.1	69.0	90.0	75.5	88.2	79.2	90.4	70.9	85.8	78.2	89.8	73.9	85.6	80.1	90.7	68.5	89.3	73.8	91.4	61.4	86.2
Ghana	Age-standardized	56.7	61.1	60.5	66.3	51.1	53.3	53.4	56.7	49.5	53.6	59.9	61.6	79.5	83.8	77.4	81.7	79.7	83.9	67.4	67.1	79.7	79.3	47.6	46.7
Greece	Age-standardized	51.4	75.1	49.6	74.1	53.2	75.4	62.5	73.9	63.8	76.6	62.8	71.2	81.3	87.4	77.1	85.3	83.2	86.3	46.6	75.5	49.7	75.0	45.0	75.9
Greenland	Age-standardized	63.5	76.1	62.7	77.8	64.8	73.7	62.6	70.8	67.1	76.0	59.7	66.5	8.0	59.7	0.7	56.4	18.6	61.5	68.3	80.9	71.1	83.4	66.2	77.2
Grenada	Age-standardized	37.9	57.5	47.4	64.6	21.9	48.2	43.5	56.5	52.3	65.1	33.0	47.6	65.0	77.2	63.5	75.1	61.1	74.2	38.6	57.7	54.4	67.5	12.9	45.4
Guam	Age-standardized	70.6	87.1	71.8	91.0	68.4	81.8	66.2	77.0	68.6	80.9	63.8	72.6	79.2	91.2	70.7	87.2	86.5	92.8	77.3	94.0	80.2	97.4	73.8	88.5
Guatemala	Age-standardized	62.7	70.5	66.1	74.6	57.3	63.9	66.8	68.3	69.4	72.1	64.4	64.2	91.7	88.1	88.8	85.9	90.9	86.5	65.8	75.1	73.3	81.6	55.6	65.4
Guinea	Age-standardized	54.2	55.1	53.4	56.8	54.2	52.1	55.6	57.8	48.2	51.1	63.7	63.9	81.8	82.7	76.4	77.5	84.5	84.9	66.5	62.5	76.6	75.4	52.3	45.2
Guinea-Bissau	Age-standardized	38.9	41.9	47.8	48.1	28.1	33.5	42.9	42.4	41.9	39.5	44.2	47.8	65.3	69.1	66.6	66.9	63.3	70.0	56.4	54.7	75.7	71.8	28.8	27.7
Guyana	Age-standardized	17.7	41.5	25.2	48.0	8.1	33.0	30.6	45.2	38.3	53.3	22.8	36.6	60.9	67.7	64.7	70.1	54.2	61.2	31.8	46.6	46.2	56.8	14.1	34.5
Haiti	Age-standardized	0.0	17.6	0.0	17.9	0.0	15.9	22.8	27.9	31.4	36.2	14.1	19.4	16.1	35.0	6.5	25.7	24.6	43.5	15.3	28.4	21.7	34.0	14.5	24.6
Honduras	Age-standardized	47.0	34.1	52.8	37.8	38.0	28.6	44.8	29.4	51.1	35.1	38.4	25.3	55.8	43.2	51.8	33.2	57.3	53.2	57.2	43.5	69.3	55.8	39.8	27.5
Hungary	Age-standardized	53.8	74.2	58.0	78.7	48.2	68.5	56.1	74.8	61.8	82.2	50.4	66.1	68.4	81.2	67.2	79.4	65.7	79.8	51.1	70.7	60.1	77.6	40.4	62.2
Iceland	Age-standardized	77.2	91.1	77.6	90.8	75.8	91.0	80.3	89.7	81.7	91.4	78.9	88.2	82.1	92.3	75.4	88.2	86.7	93.4	74.3	88.8	77.6	89.4	70.1	87.6
India	Age-standardized	48.5	56.4	50.3	58.5	45.2	53.1	57.7	63.8	62.9	67.1	52.2	60.5	55.7	66.0	56.8	62.9	54.8	67.8	58.2	62.1	62.2	67.8	53.4	55.3
Indonesia	Age-standardized	48.8	46.5	51.1	51.5	45.6	40.1	50.7	46.0	54.3	50.3	48.5	42.8	63.1	67.0	59.2	63.0	65.1	69.5	66.1	57.9	72.5	67.7	58.4	45.8
Iran (Islamic Republic of)	Age-standardized	53.9	71.8	56.6	70.7	50.4	71.6	65.1	76.6	68.9	77.9	61.5	75.3	71.9	89.3	71.8	87.6	69.7	87.9	44.4	64.7	52.9	67.1	35.7	61.7

Iraq	Age-standardized	39.4	47.8	46.4	54.9	30.9	39.1	51.0	58.7	57.6	63.8	44.5	53.6	69.1	80.0	70.7	79.7	63.5	77.1	36.4	41.1	49.4	54.9	21.2	24.7
Ireland	Age-standardized	66.8	85.4	67.6	86.4	65.3	83.7	78.7	90.6	80.1	91.6	77.7	89.4	74.6	87.6	69.8	84.7	77.3	87.5	59.6	81.1	64.3	83.9	54.8	77.6
Israel	Age-standardized	75.2	92.3	72.0	91.8	77.3	92.5	64.8	79.6	67.0	83.6	63.0	75.5	89.1	94.4	84.9	93.1	90.4	91.9	77.4	93.6	76.1	92.6	78.0	94.1
Italy	Age-standardized	58.7	79.1	61.1	80.4	54.8	76.8	70.3	78.4	75.0	82.4	65.2	74.1	77.2	85.2	72.8	82.9	79.5	84.1	51.9	78.0	58.6	80.2	44.0	75.2
Jamaica	Age-standardized	43.6	53.3	48.2	58.8	37.6	46.2	51.9	56.8	58.0	64.4	45.7	48.6	73.7	81.0	70.1	77.3	74.2	81.2	41.5	52.1	49.7	60.3	33.9	43.1
Japan	Age-standardized	74.1	93.1	75.9	95.5	71.1	90.4	84.1	94.9	87.5	98.9	80.2	91.8	76.9	91.6	73.6	88.9	78.0	91.1	69.5	91.2	73.0	93.9	64.5	87.4
Jordan	Age-standardized	55.9	77.3	51.6	73.0	59.6	79.6	56.8	72.2	58.1	72.7	57.4	72.0	93.0	99.7	90.3	97.5	92.6	98.7	51.7	74.0	52.4	72.2	52.1	74.3
Kazakhstan	Age-standardized	60.0	58.2	64.8	64.8	51.7	48.0	61.8	55.4	65.6	61.8	58.2	48.8	62.8	62.7	62.3	61.7	61.3	62.4	60.0	60.3	69.4	70.7	44.8	44.1
Kenya	Age-standardized	66.6	64.8	70.5	70.3	60.3	56.0	56.0	51.3	59.2	54.8	52.7	47.5	87.0	87.5	85.9	86.1	86.3	86.2	80.4	76.7	83.7	82.1	75.3	68.1
Kiribati	Age-standardized	58.8	58.2	67.9	67.2	47.8	46.9	64.4	60.9	68.0	65.2	59.7	55.5	71.8	76.3	76.4	77.9	64.9	74.3	77.3	74.5	86.0	83.7	64.2	60.1
Kuwait	Age-standardized	81.4	87.3	79.7	92.1	82.0	83.1	79.9	86.4	81.8	91.6	77.6	82.5	88.8	100.0	84.5	100.0	89.8	97.2	78.5	83.6	78.2	89.7	77.9	77.3
Kyrgyzstan	Age-standardized	48.9	60.5	55.2	69.2	37.9	47.6	54.8	63.5	57.6	69.0	51.6	57.3	62.9	62.8	66.0	66.1	56.2	57.9	51.3	59.5	63.3	72.8	31.3	38.2
Lao People's Democratic Republic	Age-standardized	20.4	43.4	22.0	47.1	17.5	38.2	31.0	46.3	34.1	49.1	28.7	43.8	38.6	67.2	34.8	64.4	41.4	68.3	43.3	56.2	51.8	65.8	34.5	44.7
Latvia	Age-standardized	44.7	62.7	48.8	65.4	39.2	60.5	51.4	65.5	56.8	73.1	46.2	56.8	69.9	75.5	72.8	78.1	62.6	69.4	38.3	56.8	48.8	63.1	26.6	52.0
Lebanon	Age-standardized	47.7	81.6	53.2	82.9	40.7	79.3	44.6	76.7	59.5	81.2	27.5	71.6	52.9	88.0	59.6	88.5	41.7	83.1	53.3	80.9	58.5	83.2	47.4	77.3
Lesotho	Age-standardized	45.5	35.4	56.3	46.2	20.9	14.2	31.6	22.3	41.7	31.4	19.4	14.0	81.2	73.4	84.0	74.9	71.7	67.5	64.0	54.4	73.8	65.6	41.1	33.9
Liberia	Age-standardized	53.7	51.7	54.2	53.1	51.8	49.5	56.3	51.9	48.3	44.0	63.3	60.5	82.0	81.7	76.9	76.4	84.2	84.2	63.2	59.9	75.5	73.0	47.1	42.1
Libya	Age-standardized	64.0	65.9	64.2	67.3	63.0	63.5	65.7	65.0	66.6	65.0	66.0	66.8	78.8	81.3	75.7	76.3	79.3	83.7	63.1	62.3	67.9	69.1	58.1	54.1
Lithuania	Age-standardized	61.3	69.9	64.9	74.2	55.9	64.0	65.6	72.5	68.8	78.2	62.9	66.3	71.1	75.5	74.2	78.6	63.8	68.2	56.8	66.3	64.9	73.5	46.2	56.7
Luxembourg	Age-standardized	51.0	81.4	53.8	83.8	46.9	77.8	63.9	79.4	68.3	83.9	59.2	74.4	73.9	89.1	68.0	85.5	78.0	89.4	43.1	80.2	51.6	84.1	33.7	74.8
Madagascar	Age-standardized	45.4	51.3	56.0	58.4	32.1	40.7	39.7	37.1	46.9	41.8	33.2	33.0	69.0	74.8	73.4	74.3	65.7	74.3	70.8	71.8	79.4	79.1	58.0	60.3
Malawi	Age-standardized	51.1	50.2	55.0	56.4	45.7	41.5	37.3	29.0	39.6	33.4	35.9	25.7	75.5	76.1	74.1	76.2	75.3	74.6	70.9	66.3	76.5	74.1	63.2	55.2
Malaysia	Age-standardized	59.4	70.1	61.5	71.1	56.1	67.9	60.5	66.3	62.9	68.1	58.2	64.6	75.3	84.1	72.8	79.7	75.4	86.0	70.3	76.9	74.4	79.0	65.3	74.0
Maldives	Age-standardized	46.1	67.9	42.1	69.7	46.3	65.0	48.4	67.9	44.2	65.7	50.4	67.7	64.9	86.7	59.2	82.9	68.6	88.3	63.6	71.6	66.3	77.0	58.8	65.3
Mali	Age-standardized	52.2	56.5	49.2	54.3	54.8	57.3	52.3	57.3	43.4	45.4	61.8	66.0	79.6	84.5	70.4	75.4	85.1	89.2	67.5	65.8	74.5	74.6	58.1	54.4
Malta	Age-standardized	61.6	83.7	63.6	84.4	58.2	82.2	71.2	87.3	73.0	88.2	69.7	86.2	89.9	95.9	84.9	92.6	91.9	95.5	54.7	79.4	61.8	81.7	46.5	76.8
Marshall Islands	Age-standardized	41.1	47.5	53.1	54.8	27.2	38.4	44.5	47.8	55.1	54.8	34.0	41.0	54.9	64.7	56.0	60.8	54.7	67.4	68.6	71.1	76.5	76.6	58.7	64.1
Mauritania	Age-standardized	55.4	61.5	56.0	62.3	54.5	59.7	52.8	59.8	43.2	50.9	64.8	69.7	81.2	88.6	75.9	82.4	83.9	91.5	66.5	66.4	77.4	76.9	49.6	50.6
Mauritius	Age-standardized	53.8	70.8	61.8	77.5	44.5	62.5	57.4	69.9	65.4	76.4	49.3	63.3	67.5	75.8	72.9	77.9	57.7	70.3	56.3	73.2	66.9	80.4	43.6	63.8
Mexico	Age-standardized	71.2	82.1	73.3	85.9	66.3	76.2	72.1	78.3	75.0	83.1	68.2	72.3	86.7	80.7	82.8	79.4	87.2	78.2	69.0	82.8	74.4	87.8	60.1	75.4
Micronesia (Federated States of)	Age-standardized	41.5	50.0	48.9	57.3	32.5	40.9	46.4	50.7	52.7	57.1	39.8	43.9	52.5	65.8	51.9	63.5	53.8	68.3	68.6	71.4	74.7	77.6	61.4	63.7
Monaco	Age-standardized	64.1	80.2	64.8	79.9	62.6	80.1	68.6	82.8	72.9	85.3	64.1	80.4	70.1	88.1	63.5	82.2	75.3	91.5	59.9	76.1	64.0	77.8	55.9	74.5
Mongolia	Age-standardized	38.4	57.0	48.1	65.7	23.0	44.4	45.4	59.8	54.7	66.8	33.9	51.0	36.8	38.5	40.3	38.9	31.4	37.9	84.3	85.1	91.0	91.8	72.1	73.6
Montenegro	Age-standardized	38.3	28.3	43.0	32.3	32.1	24.5	43.9	30.9	51.4	41.1	35.8	21.1	78.7	81.2	75.4	76.4	79.6	84.0	68.2	54.9	74.9	63.0	59.6	46.1
Morocco	Age-standardized	46.0	54.7	40.1	51.3	50.5	56.8	51.8	58.1	50.5	56.5	55.2	61.1	65.8	75.0	61.8	69.9	67.8	77.9	44.4	49.3	45.1	52.0	44.0	46.9
Mozambique	Age-standardized	42.3	42.0	51.6	55.6	30.2	24.7	33.0	29.5	41.1	41.7	25.3	17.9	69.9	67.3	75.0	75.3	65.4	60.6	64.2	61.2	72.9	72.9	51.7	43.3
Myanmar	Age-standardized	21.3	40.8	26.2	50.0	14.7	29.5	30.6	41.8	33.2	48.2	28.3	35.3	43.7	65.4	44.9	66.8	41.3	62.2	45.9	56.3	58.1	68.0	31.2	40.5
Namibia	Age-standardized	51.4	53.7	57.8	61.0	40.7	41.5	40.5	39.2	41.9	43.8	40.8	35.7	85.4	87.4	84.1	86.6	83.7	85.1	63.6	62.5	73.7	72.3	45.7	45.3
Nauru	Age-standardized	47.1	49.5	52.9	56.3	40.0	40.4	48.8	42.8	54.7	49.5	43.5	36.2	55.7	60.8	46.6	52.6	62.0	68.9	68.9	72.5	75.6	79.9	60.2	62.9
Nepal	Age-standardized	38.3	50.9	34.3	54.1	40.4	45.9	51.6	59.6	53.9	64.6	49.9	54.1	53.0	67.1	50.2	66.5	54.8	65.7	46.2	54.6	46.9	62.2	45.8	45.4
Netherlands	Age-standardized	77.4	85.8	78.6	86.4	75.3	84.6	75.9	84.6	79.7	86.5	71.5	82.2	76.8	88.6	73.0	85.7	77.7	88.0	75.5	83.4	78.9	85.6	71.4	80.6
New Zealand	Age-standardized	69.9	84.6	70.5	84.4	68.8	84.5	73.2	82.2	76.3	85.1	69.5	78.9	62.6	76.9	57.4	74.7	65.7	76.0	67.6	83.5	71.6	84.5	63.8	82.2
Nicaragua	Age-standardized	68.1	78.4	73.2	82.1	59.4	72.3	65.5	73.7	70.4	78.2	59.1	67.8	82.5	88.6	79.1	85.0	82.1	88.6	72.3	79.8	80.5	85.3	59.1	71.5
Niger	Age-standardized	55.5	54.6	59.5	57.9	50.1	49.4	54.9	53.5	53.6	50.7	56.7	57.1	80.5	83.7	78.7	81.4	80.6	83.9	70.8	67.1	80.4	78.1	56.7	50.7
Nigeria	Age-standardized	50.8	66.1	56.1	71.0	44.3	58.6	51.7	65.6	48.7	63.7	54.9	68.2	82.7	92.4	83.0	90.8	81.8	91.0	61.5	69.0	75.6	81.2	41.7	49.8
Niue	Age-standardized	58.5	63.5	65.0	69.9	49.6	54.8	55.9	56.9	62.4	64.2	48.4	48.7	70.2	73.4	67.2	67.4	73.6	78.7	74.9	77.6	80.7	83.7	67.1	69.2
North Macedonia	Age-standardized	38.6	36.3	41.1	37.1	34.1	36.1	48.2	51.2	54.6	56.6	41.6	46.4	51.1	60.8	45.2	53.1	55.6	68.3	34.2	25.5	43.7	33.6	23.1	20.6
Northern Mariana Islands	Age-standardized	57.2	66.5	56.6	70.9	56.1	61.7	56.3	61.0	53.0	63.2	56.1	57.9	70.3	78.2	52.0	68.8	83.6	85.1	73.6	79.6	76.8	84.4	70.3	74.1

Norway	Age-standardized	75.7	90.2	75.7	90.1	74.7	89.7	77.4	86.3	81.8	87.8	71.9	84.9	74.4	92.0	68.1	89.8	79.5	91.1	72.5	88.5	74.9	89.4	69.2	86.7
Oman	Age-standardized	54.7	71.6	61.4	75.7	45.8	65.4	58.9	74.2	66.8	78.4	49.3	68.9	79.4	89.2	79.9	87.5	76.0	88.2	52.6	66.8	64.0	74.5	37.7	55.8
Pakistan	Age-standardized	58.6	59.9	56.5	58.9	58.5	59.5	65.1	64.5	66.3	65.1	63.7	64.3	64.7	65.8	61.8	60.6	66.4	69.2	65.5	64.8	66.6	68.4	62.9	60.4
Palau	Age-standardized	57.1	65.1	68.6	71.2	44.1	58.5	57.4	62.0	68.7	69.9	46.5	55.0	72.9	79.1	71.2	74.3	73.7	82.5	71.8	76.1	79.9	79.3	61.4	71.7
Palestine	Age-standardized	30.2	51.2	36.0	56.3	21.6	43.6	46.7	60.2	53.7	66.0	39.7	54.3	87.6	95.7	85.9	94.1	85.2	93.0	18.5	42.0	33.6	53.1	0.1	27.6
Panama	Age-standardized	65.7	75.1	70.8	81.8	58.8	66.2	67.4	74.6	73.3	82.7	61.1	65.9	81.9	80.7	77.4	79.1	83.5	78.8	66.2	75.3	73.9	82.7	56.2	64.9
Papua New Guinea	Age-standardized	21.9	31.8	14.4	24.8	25.9	34.9	16.7	23.0	6.2	12.4	21.8	27.7	47.3	60.4	31.2	46.0	60.7	71.1	64.2	66.6	62.1	65.0	67.2	67.9
Paraguay	Age-standardized	50.6	61.0	52.5	64.7	47.4	56.1	53.6	57.1	58.1	64.1	49.2	50.0	61.1	68.1	54.2	60.5	66.1	74.0	55.4	63.5	63.0	71.0	46.5	54.5
Peru	Age-standardized	65.6	81.1	70.5	84.0	58.7	76.8	63.9	74.2	69.2	77.9	58.3	70.5	73.4	83.2	70.7	77.7	73.3	86.0	72.1	83.5	79.0	88.2	62.5	76.8
Philippines	Age-standardized	46.5	57.7	52.1	65.8	37.9	47.1	40.7	55.3	48.0	62.9	32.9	47.5	73.2	76.1	72.7	74.8	70.8	74.7	62.3	70.0	68.4	77.7	53.6	59.1
Poland	Age-standardized	37.3	68.3	43.5	73.3	29.5	62.8	55.5	69.4	62.4	78.5	48.3	59.1	66.2	78.1	67.2	79.3	61.9	74.4	25.2	64.6	39.0	71.8	10.1	56.9
Portugal	Age-standardized	40.6	69.4	42.8	70.9	36.6	67.1	56.1	73.0	60.6	78.3	51.8	67.5	63.8	75.6	60.2	72.0	65.7	76.8	33.0	67.7	41.7	70.6	23.2	64.6
Puerto Rico	Age-standardized	73.0	91.8	78.9	96.7	64.3	83.5	77.3	86.9	83.3	93.1	70.3	78.9	88.5	94.9	86.6	93.7	85.3	90.6	68.4	91.2	76.2	95.7	57.6	83.3
Qatar	Age-standardized	61.0	77.4	63.8	78.6	58.0	76.1	59.6	78.7	66.8	81.0	54.0	77.4	55.3	84.8	75.0	88.8	37.9	80.1	64.8	75.3	69.3	78.3	59.6	71.6
Republic of Korea	Age-standardized	62.8	88.6	63.8	89.4	60.6	87.1	71.6	93.5	76.2	96.2	65.9	90.1	51.5	86.0	49.4	82.8	52.6	86.9	65.7	85.9	69.0	87.4	60.9	83.2
Republic of Moldova	Age-standardized	40.5	61.6	43.2	65.8	37.0	56.3	44.7	55.6	48.7	61.3	41.6	49.9	74.3	79.0	77.2	81.7	68.0	74.0	49.7	63.5	57.1	70.7	42.0	54.8
Romania	Age-standardized	40.6	56.2	42.2	60.8	38.3	51.0	45.2	52.8	49.8	62.6	41.5	42.4	60.4	60.8	57.0	60.7	62.2	59.6	39.0	55.4	46.9	63.1	31.4	47.0
Russian Federation	Age-standardized	37.9	55.3	45.0	61.7	25.7	47.0	55.6	61.1	60.5	68.0	50.5	53.8	67.2	62.8	69.2	64.0	61.8	59.9	25.3	49.9	41.8	61.2	0.0	35.7
Rwanda	Age-standardized	33.7	55.7	42.4	63.2	19.8	42.2	31.3	42.0	41.6	52.4	19.8	29.0	67.1	83.9	71.0	85.3	61.1	79.8	63.8	72.0	70.1	78.3	55.5	61.6
Saint Kitts and Nevis	Age-standardized	35.2	49.2	41.9	56.9	24.0	40.6	45.8	53.3	53.0	63.1	37.2	43.7	65.1	78.2	61.5	77.4	63.9	75.2	36.7	47.3	49.7	57.4	18.7	37.6
Saint Lucia	Age-standardized	34.9	58.7	42.7	67.0	24.2	47.9	46.1	60.5	55.9	72.2	35.3	48.4	68.1	82.7	63.5	79.9	69.5	81.9	31.5	56.9	43.5	65.6	19.6	46.5
Saint Vincent and the Grenadines	Age-standardized	42.6	54.4	48.7	62.5	33.1	44.2	39.7	54.6	48.9	64.4	29.5	44.7	78.3	81.8	74.1	79.3	78.7	80.7	47.2	56.2	56.4	65.4	37.0	45.2
Samoa	Age-standardized	52.8	60.3	62.2	65.6	40.7	53.3	50.9	54.1	61.5	60.8	39.1	47.3	67.0	76.0	64.8	69.9	69.2	81.2	74.6	78.9	81.1	84.3	66.3	71.8
San Marino	Age-standardized	72.4	89.0	72.0	87.8	72.0	89.4	74.0	88.5	75.2	87.9	74.1	89.5	89.7	99.2	86.1	95.2	90.4	99.7	68.5	85.7	71.6	86.5	64.7	84.1
Sao Tome and Principe	Age-standardized	68.8	68.8	70.6	70.8	63.8	65.6	66.7	67.3	63.9	64.4	72.2	72.9	89.6	89.4	85.6	84.5	90.3	91.3	76.1	72.4	83.7	80.9	58.9	58.9
Saudi Arabia	Age-standardized	36.2	56.8	33.6	54.8	36.0	56.4	43.3	59.6	46.4	63.6	39.9	55.9	79.1	87.8	76.3	84.5	79.6	88.4	35.4	53.7	39.1	55.2	30.2	50.0
Senegal	Age-standardized	56.7	57.9	61.5	62.8	50.5	51.1	54.7	56.5	50.3	51.3	59.2	62.1	80.4	83.5	78.4	80.2	80.5	84.5	66.1	64.4	79.2	77.7	47.5	44.0
Serbia	Age-standardized	28.5	47.5	31.5	50.1	25.0	44.1	46.4	56.7	52.8	64.8	40.2	46.8	48.0	64.8	42.7	59.1	52.5	69.3	18.1	39.3	29.5	47.3	8.0	31.6
Seychelles	Age-standardized	59.3	70.3	67.9	74.6	49.1	65.5	59.4	67.9	65.0	70.9	53.7	65.3	77.0	89.4	77.9	87.7	73.4	88.5	66.2	73.2	77.0	79.7	51.5	65.6
Sierra Leone	Age-standardized	55.1	56.4	60.2	58.8	48.8	53.0	56.0	56.7	51.6	50.9	60.2	64.1	81.2	82.3	79.1	77.1	81.6	84.7	63.2	63.2	78.4	76.0	43.0	45.0
Singapore	Age-standardized	78.9	98.4	77.0	97.1	80.2	99.6	84.9	100.0	85.9	100.0	84.0	100.0	85.2	98.5	79.8	93.6	88.2	100.0	76.5	95.9	76.1	94.8	76.8	96.4
Slovakia	Age-standardized	63.8	76.4	66.7	79.2	60.3	72.8	50.9	67.4	59.4	75.2	42.2	58.8	70.9	82.3	68.8	80.5	70.5	81.3	64.5	74.7	70.2	79.4	58.2	69.1
Slovenia	Age-standardized	54.7	77.7	60.2	81.5	47.2	72.3	65.7	79.4	72.9	85.1	57.6	72.2	75.8	90.3	74.4	88.7	74.2	88.9	47.3	73.7	57.5	79.4	34.7	65.9
Solomon Islands	Age-standardized	48.8	52.4	54.3	57.7	41.5	44.8	55.4	56.2	61.3	62.3	49.3	49.8	63.2	71.2	59.7	67.7	65.0	73.1	72.8	73.7	75.7	77.0	68.8	69.7
Somalia	Age-standardized	37.3	47.3	48.5	56.3	22.3	32.8	26.8	28.6	36.6	38.2	16.9	17.9	66.4	75.7	74.1	79.4	60.1	70.3	71.4	73.9	76.6	79.2	64.3	65.8
South Sudan	Age-standardized	75.4	66.9	58.7	62.0	36.7	42.7	64.3	48.7	44.1	39.9	27.6	25.3	91.7	89.6	79.3	81.1	74.8	78.3	82.2	72.7	79.1	80.0	63.9	64.8
Spain	Age-standardized	47.8	53.1	63.2	87.6	58.0	87.9	35.0	32.2	74.2	85.1	68.6	76.8	76.4	79.7	80.6	85.1	80.5	84.3	72.5	73.6	61.6	88.1	49.5	87.3
Sri Lanka	Age-standardized	61.3	87.9	54.7	66.2	39.8	52.0	71.4	81.2	55.6	63.0	40.1	49.0	82.1	86.6	83.7	88.8	80.8	87.2	55.9	88.1	59.2	68.2	36.9	47.5
Sudan	Age-standardized	47.9	60.3	20.4	48.7	34.3	57.1	47.6	55.9	36.5	50.6	34.5	58.0	83.5	89.7	48.7	63.6	52.5	72.3	48.7	59.2	35.3	53.7	37.9	50.0
Suriname	Age-standardized	29.0	54.3	48.0	60.4	39.4	44.7	34.9	54.1	53.1	60.4	42.5	42.5	51.0	68.9	68.2	73.9	69.0	73.2	37.1	52.5	61.1	69.8	46.9	53.0
Sweden	Age-standardized	44.4	53.4	75.2	90.3	75.4	91.5	47.6	51.7	82.9	92.6	80.9	94.2	70.1	75.4	69.8	86.7	82.9	93.9	54.3	62.1	74.1	88.9	68.9	87.2
Switzerland	Age-standardized	75.6	91.2	69.5	89.3	67.1	89.9	81.7	93.3	78.5	91.2	75.4	90.7	77.0	91.7	69.3	87.1	79.0	90.5	71.7	88.5	68.5	87.9	58.6	85.5
Syrian Arab Republic	Age-standardized	68.8	89.9	50.3	55.2	51.2	57.3	76.7	90.9	62.0	65.1	58.5	60.7	74.9	90.4	76.9	87.2	77.2	88.9	64.0	87.1	55.1	55.1	49.9	52.0
Taiwan (Province of China)	Age-standardized	51.5	57.8	67.3	91.5	61.3	80.7	60.1	63.0	67.0	88.1	57.5	76.1	78.1	89.4	79.5	88.7	83.4	86.7	52.7	54.8	77.0	92.9	69.2	84.0
Tajikistan	Age-standardized	64.6	86.6	52.0	57.2	37.2	47.2	62.2	82.0	55.4	55.5	48.9	52.3	82.7	89.4	65.4	70.7	74.5	78.3	73.2	89.3	64.0	66.1	29.4	42.4
Thailand	Age-standardized	46.1	53.2	65.7	78.4	53.5	65.3	51.7	53.8	65.0	73.3	59.6	64.7	71.3	75.6	54.3	69.3	43.9	60.8	50.1	56.0	78.9	85.8	63.1	74.2
Timor-Leste	Age-standardized	60.5	72.5	30.2	42.0	33.9	35.0	62.2	68.8	31.9	41.1	36.4	37.7	50.0	65.5	50.0	60.2	60.2	65.8	72.2	80.9	61.8	64.3	49.7	43.7
Togo	Age-standardized	32.7	39.2	57.2	58.0	51.9	44.6	33.4	39.0	48.2	48.3	61.0	55.7	55.7	63.7	76.5	78.3	81.6	80.3	56.0	54.9	77.3	75.6	48.4	38.0

Tokelau	Age-standardized	55.1	52.6	52.9	65.6	30.8	49.5	54.1	50.9	56.4	60.8	31.8	42.0	80.2	80.2	54.9	64.0	65.1	77.3	65.4	61.5	74.0	82.1	54.0	66.7
Tonga	Age-standardized	44.4	59.4	71.4	73.7	61.9	66.3	45.7	52.0	66.5	66.9	57.0	59.6	60.6	71.3	74.2	78.6	86.5	91.0	65.9	75.9	85.8	84.9	71.9	74.2
Trinidad and Tobago	Age-standardized	67.5	70.6	55.9	71.7	44.2	57.6	61.8	63.2	60.1	72.5	45.9	54.4	80.9	85.3	66.4	72.2	64.5	67.0	80.0	80.3	61.5	74.0	36.3	53.8
Tropical Latin America	Age-standardized	50.7	65.2	51.6	71.5	39.0	62.4	53.3	64.1	60.9	71.0	44.6	55.7	67.2	71.4	54.2	58.2	58.3	65.9	49.8	64.8	57.6	77.1	33.7	61.3
Tunisia	Age-standardized	48.2	64.0	48.4	66.2	46.1	60.2	56.1	68.2	58.9	70.6	53.6	66.0	75.0	86.4	74.6	84.8	72.6	84.8	43.5	58.4	49.4	65.3	36.4	49.8
Turkey	Age-standardized	57.0	70.7	61.0	73.2	52.1	67.5	61.5	72.3	65.7	76.0	57.6	68.7	50.5	76.7	56.8	78.5	41.5	71.7	59.2	68.9	66.9	73.9	50.8	63.7
Turkmenistan	Age-standardized	57.0	56.3	62.2	65.5	48.5	43.5	59.9	53.3	63.6	61.5	56.0	44.8	63.2	55.4	62.9	59.7	61.5	51.5	59.4	60.9	69.1	72.0	44.7	44.2
Tuvalu	Age-standardized	34.5	51.3	42.4	57.4	23.1	43.4	39.9	49.8	47.9	55.9	31.4	43.8	47.0	67.0	43.9	62.1	50.1	71.9	63.2	70.0	71.5	76.6	52.6	61.7
Uganda	Age-standardized	55.3	66.2	67.2	74.0	40.0	53.0	37.0	47.9	50.1	57.0	24.9	37.9	76.5	85.1	82.9	87.4	71.0	81.0	76.7	79.5	82.3	84.5	68.1	70.5
Ukraine	Age-standardized	51.5	64.0	54.7	69.0	47.4	56.9	64.2	69.4	69.1	76.1	58.8	61.7	57.8	67.5	56.9	68.9	56.0	64.5	43.5	59.7	52.6	68.3	33.8	48.4
United Arab Emirates	Age-standardized	63.7	73.2	62.9	40.7	63.5	78.8	65.4	72.1	69.5	51.8	62.8	76.4	58.2	74.1	53.6	46.0	63.0	83.2	62.8	71.1	65.8	40.1	59.2	75.2
United Kingdom	Age-standardized	67.8	86.0	69.3	86.5	65.5	84.8	76.5	81.7	78.6	83.5	74.8	80.0	67.7	82.0	62.0	78.6	72.2	82.6	62.4	85.9	68.2	88.0	56.0	82.9
United Republic of Tanzania	Age-standardized	55.9	66.7	63.8	70.4	45.5	60.9	43.9	53.6	51.5	56.6	36.5	50.8	79.7	87.0	82.4	85.3	76.5	86.2	76.5	75.2	81.7	81.2	68.7	66.1
United States Virgin Islands	Age-standardized	55.1	76.6	62.6	85.2	44.0	64.5	55.6	73.7	64.8	84.2	44.9	61.5	73.3	88.6	72.7	90.3	68.0	80.9	56.2	77.0	65.5	84.7	44.9	64.9
United States of America	Age-standardized	85.8	100.0	87.1	100.0	83.5	100.0	82.9	90.7	85.7	92.5	79.5	89.1	75.1	83.7	72.5	84.3	73.6	78.2	84.9	100.0	87.8	100.0	80.9	100.0
Uruguay	Age-standardized	67.8	77.5	70.2	79.8	64.3	74.1	73.3	82.0	79.3	87.4	65.6	74.5	60.1	72.2	57.8	69.5	60.1	72.1	67.2	75.8	71.9	79.7	61.8	71.3
Uzbekistan	Age-standardized	58.3	64.9	63.3	70.2	50.6	56.9	52.7	65.4	56.8	68.9	48.8	61.2	81.5	66.7	81.1	70.1	79.1	62.2	67.7	67.8	76.1	76.4	54.7	54.8
Vanuatu	Age-standardized	49.7	57.7	59.6	65.3	38.6	48.2	48.6	53.9	58.2	61.1	39.5	46.8	60.5	70.2	58.6	67.0	61.4	72.3	72.0	76.6	79.8	83.0	62.2	67.8
Venezuela (Bolivarian Republic of)	Age-standardized	63.9	67.3	68.7	74.8	56.7	56.3	62.5	65.4	68.4	74.5	55.8	53.7	80.1	78.3	77.0	76.4	79.3	75.6	69.7	70.6	76.5	79.0	60.7	58.7
Viet Nam	Age-standardized	34.0	42.9	43.6	54.1	21.5	30.0	40.4	44.8	47.5	55.3	33.1	34.1	63.1	74.0	66.9	76.6	56.4	69.5	48.9	51.0	61.5	64.1	31.3	33.6
Yemen	Age-standardized	19.7	34.8	20.9	36.0	16.7	32.0	29.3	39.1	38.4	41.4	19.6	38.2	48.5	59.4	54.3	60.9	37.9	55.9	25.9	31.6	32.2	41.4	20.1	20.7
Zambia	Age-standardized	47.1	52.3	54.5	60.1	37.0	40.9	35.1	36.6	39.4	39.8	30.9	32.8	73.4	77.8	73.7	76.9	72.9	77.3	73.0	69.6	78.2	76.5	65.2	59.2
Zimbabwe	Age-standardized	59.2	48.0	65.1	54.8	49.6	35.4	29.1	0.0	28.6	0.0	30.7	5.7	78.0	68.0	75.2	65.2	78.4	69.1	70.8	64.4	78.8	73.5	57.1	47.4
Afghanistan	80 to 84	26.8	36.8	38.5	42.9	12.6	29.0	20.5	28.5	37.7	34.6	6.2	24.1	37.9	53.0	66.3	66.3	23.0	44.4	34.9	39.4	42.6	46.5	34.1	40.3
Albania	80 to 84	35.0	46.7	48.9	56.3	21.3	38.0	3.5	18.6	10.8	22.1	0.0	18.3	60.1	68.4	64.0	68.8	57.6	70.1	61.8	68.4	72.5	76.1	57.2	66.3
Algeria	80 to 84	46.9	78.3	48.2	82.9	47.9	73.4	30.5	58.2	17.2	57.0	43.9	60.9	59.4	77.4	56.5	79.9	64.8	76.1	50.0	79.5	53.8	84.9	55.5	77.3
American Samoa	80 to 84	73.1	74.9	78.8	80.4	67.3	69.1	51.4	53.0	52.5	54.8	51.7	52.3	77.4	79.1	75.3	77.7	82.3	82.9	83.0	84.2	87.3	88.0	81.2	82.6
Andorra	80 to 84	83.8	88.3	85.7	90.1	81.6	86.6	80.9	83.9	79.8	83.2	82.2	85.3	84.4	87.4	84.6	87.6	86.0	88.5	83.5	88.4	85.8	90.5	83.5	88.3
Angola	80 to 84	71.6	73.9	81.5	82.5	57.2	60.7	46.1	47.3	50.9	50.3	42.5	47.1	82.9	85.5	89.6	89.8	78.0	81.8	79.5	79.5	88.0	87.3	70.7	71.0
Antigua and Barbuda	80 to 84	60.8	68.2	71.5	77.0	47.5	57.4	54.0	58.4	57.4	63.7	51.8	53.7	79.7	81.5	85.4	87.2	72.7	75.5	63.0	70.3	74.1	78.8	56.4	65.9
Argentina	80 to 84	71.7	85.5	78.1	89.8	65.2	80.0	68.5	76.9	72.9	81.4	63.2	71.0	67.8	81.0	65.3	83.0	73.6	80.5	73.7	87.9	80.3	91.6	71.7	85.6
Armenia	80 to 84	62.5	72.7	71.2	77.4	53.2	69.7	48.8	64.3	52.5	66.1	47.1	63.8	66.1	72.9	70.2	76.8	62.3	69.4	65.1	73.0	74.9	78.3	60.6	73.7
Australia	80 to 84	80.1	91.2	83.4	92.2	77.3	89.2	73.2	78.4	73.9	78.8	73.9	78.3	80.5	82.6	83.0	86.8	78.2	78.2	80.3	92.9	84.2	93.5	80.1	92.3
Austria	80 to 84	76.4	98.1	81.1	99.9	72.8	95.2	87.4	90.8	87.3	91.0	88.6	90.8	92.6	95.1	93.2	97.4	92.8	92.0	72.0	97.9	78.1	99.1	72.3	96.0
Azerbaijan	80 to 84	64.9	69.6	74.3	75.6	51.3	64.3	45.6	50.6	49.6	51.0	44.1	53.6	83.6	82.7	86.8	85.0	79.1	80.4	75.3	78.4	83.9	84.2	66.8	75.9
Bahamas	80 to 84	70.4	73.6	75.3	78.7	67.9	69.2	60.1	64.5	60.7	66.8	62.1	63.0	86.3	86.4	89.2	90.2	82.7	81.9	72.8	74.9	77.9	79.9	75.1	75.7
Bahrain	80 to 84	26.7	50.3	43.4	58.7	9.2	42.8	19.8	39.8	29.8	46.1	11.3	35.1	46.9	64.9	56.0	69.2	35.6	62.4	30.9	52.5	48.8	61.7	24.9	52.9
Bangladesh	80 to 84	49.6	50.9	58.2	60.3	40.6	41.6	51.5	52.6	55.0	56.7	49.1	49.8	51.0	61.5	62.9	66.4	47.9	60.7	54.1	53.7	62.7	64.0	53.1	51.7
Barbados	80 to 84	63.0	69.0	70.4	75.2	56.4	63.3	60.9	62.7	64.3	67.2	58.3	58.3	81.9	82.1	87.1	86.2	75.9	78.6	61.9	68.9	70.3	75.4	61.7	69.0
Belarus	80 to 84	60.7	68.6	66.8	75.2	59.2	62.9	57.8	56.7	60.9	60.8	56.6	53.0	71.2	69.9	75.9	74.9	59.7	56.7	59.4	68.5	67.5	76.6	63.1	67.3
Belgium	80 to 84	70.7	88.2	77.5	90.9	63.8	84.8	68.9	74.4	71.8	76.7	66.5	72.5	82.3	80.1	83.6	84.7	82.6	75.8	70.5	90.5	78.4	92.7	67.7	89.3
Belize	80 to 84	67.3	71.6	74.2	75.4	60.3	67.6	58.0	60.6	59.6	60.6	57.7	61.1	86.0	87.3	91.0	90.1	80.8	85.0	70.1	74.0	77.1	77.6	68.5	74.3
Benin	80 to 84	66.4	68.1	73.1	75.4	60.1	61.1	56.3	59.4	44.2	51.5	67.7	70.4	82.9	85.3	82.9	85.7	84.2	86.2	72.0	72.3	81.9	81.9	65.1	64.7
Bermuda	80 to 84	71.2	83.3	79.1	90.3	61.4	74.2	69.4	77.9	73.1	83.4	65.2	71.9	86.4	89.8	91.9	97.2	78.1	81.2	69.9	82.7	78.7	89.3	65.2	77.4
Bhutan	80 to 84	66.3	71.3	72.2	76.9	61.2	65.2	64.3	69.0	66.1	69.8	63.7	68.7	72.4	77.4	77.5	80.2	68.7	76.3	69.0	72.7	75.5	79.3	68.8	70.2
Bolivia (Plurinational State of)	80 to 84	58.3	66.8	66.5	73.2	50.8	61.0	48.0	55.4	48.3	55.3	50.8	57.9	71.3	73.1	73.0	72.8	72.6	76.8	60.6	69.0	70.1	76.4	58.0	67.0
Bosnia and Herzegovina	80 to 84	53.9	64.0	61.0	70.5	48.6	58.8	36.7	48.1	40.8	50.6	34.7	47.9	58.2	69.4	61.5	71.2	57.6	69.2	54.1	63.8	62.9	71.9	55.1	63.4
Botswana	80 to 84	73.6	81.3	81.3	87.1	62.8	72.8	49.1	61.0	51.1	61.7	51.6	64.3	85.9	90.3	89.0	92.5	83.1	87.6	78.9	84.1	86.5	90.1	71.4	77.5

Brazil	80 to 84	59.5	77.9	67.9	83.0	51.0	72.1	58.4	67.0	62.1	70.2	55.5	64.1	86.7	79.8	88.1	83.1	86.0	77.2	57.7	78.4	67.9	84.0	54.6	75.8
Brunei Darussalam	80 to 84	84.4	84.9	86.4	85.8	83.0	83.8	72.8	75.7	77.0	76.3	68.1	76.4	80.7	84.0	80.2	83.8	88.6	87.9	88.3	86.9	89.0	87.4	90.7	87.6
Bulgaria	80 to 84	48.5	54.5	56.4	64.9	42.1	43.3	31.8	49.6	37.6	53.2	28.3	47.6	72.8	53.1	76.2	56.3	70.0	51.2	52.9	53.8	62.5	66.7	52.5	48.2
Burkina Faso	80 to 84	72.3	72.0	83.8	81.9	56.1	58.5	61.1	62.4	62.6	61.8	60.2	64.0	87.6	88.4	94.4	92.9	82.6	85.0	78.8	77.4	91.6	88.7	64.4	64.9
Burundi	80 to 84	65.0	70.6	76.2	80.1	49.0	58.1	36.2	45.0	47.1	52.8	24.5	38.0	78.2	82.5	88.2	90.5	70.0	77.2	74.6	78.1	82.6	85.4	69.2	73.0
Cabo Verde	80 to 84	76.9	75.2	83.4	83.7	69.3	62.6	70.0	70.5	65.0	68.8	75.8	74.9	92.9	95.1	95.5	97.0	90.5	92.3	80.8	77.0	89.4	87.3	72.7	64.1
Cambodia	80 to 84	51.3	55.1	58.9	63.5	47.1	49.0	32.4	32.7	32.1	35.4	39.1	35.5	60.2	66.6	63.4	68.7	58.3	66.0	61.2	63.1	69.5	71.8	61.3	61.9
Cameroon	80 to 84	69.8	69.9	78.5	78.2	59.3	60.0	60.0	60.8	56.7	56.0	63.8	67.5	84.7	86.9	87.7	88.8	83.4	86.1	76.0	74.4	86.4	84.9	66.7	64.7
Canada	80 to 84	86.5	98.9	89.8	99.9	82.9	96.6	86.2	91.9	87.2	92.1	85.2	91.6	87.1	87.1	89.9	91.0	82.6	82.2	85.7	100.0	89.4	100.0	84.3	98.6
Central African Republic	80 to 84	63.4	63.2	74.6	73.9	46.7	45.3	38.2	34.9	43.5	40.8	34.8	31.6	78.1	79.2	84.9	84.5	72.9	73.6	72.2	71.6	81.9	81.1	63.1	61.4
Chad	80 to 84	71.0	68.5	78.7	77.5	62.0	58.2	60.8	61.2	55.4	54.3	67.4	66.7	86.0	85.3	88.4	86.6	85.0	85.3	76.2	72.2	86.1	84.3	67.4	62.4
Chile	80 to 84	71.9	84.4	78.7	89.2	64.5	78.3	74.5	84.3	79.0	87.6	67.8	79.7	80.2	79.8	79.3	81.9	84.1	79.2	71.4	84.7	78.8	89.7	68.8	81.2
China	80 to 84	54.0	73.2	62.4	80.8	46.5	64.5	32.4	49.4	38.7	53.4	28.1	46.4	0.0	62.0	0.0	65.4	9.2	61.3	71.8	80.9	78.1	87.7	70.5	76.1
Colombia	80 to 84	75.8	83.8	82.0	89.4	68.9	76.4	64.1	75.0	65.1	76.7	64.5	73.6	87.9	82.9	89.9	85.3	86.3	81.0	77.4	84.9	84.1	90.5	73.5	80.1
Comoros	80 to 84	72.8	77.1	81.1	84.5	62.2	67.4	43.9	49.6	45.2	49.8	44.8	51.4	84.9	87.8	90.7	92.3	80.8	84.4	80.9	83.1	87.8	89.6	75.1	77.1
Congo	80 to 84	69.7	73.6	78.4	81.1	58.6	64.3	40.0	45.9	39.9	44.3	44.5	52.0	82.7	85.8	86.4	88.5	80.2	83.9	77.5	79.0	85.6	86.2	70.4	73.0
Cook Islands	80 to 84	72.4	84.4	80.1	89.4	63.4	77.8	49.0	65.7	54.4	70.8	44.6	60.1	77.6	87.4	78.4	88.1	79.3	88.8	82.0	90.6	88.0	94.0	78.0	87.7
Costa Rica	80 to 84	79.5	85.0	85.5	90.2	72.2	78.4	72.8	80.4	75.0	82.2	70.8	78.7	89.5	87.8	91.7	90.5	87.5	85.3	79.5	84.5	86.1	89.8	75.3	80.4
Coted'Ivoire	80 to 84	72.4	72.4	80.4	80.5	63.1	62.6	64.3	65.7	59.4	61.2	68.7	71.3	87.6	88.6	89.4	89.4	86.8	88.5	76.1	75.2	86.5	85.6	68.0	65.9
Croatia	80 to 84	51.2	77.7	62.7	81.3	38.2	75.1	40.0	49.7	49.8	55.2	27.0	44.7	53.6	54.7	57.7	58.3	50.7	52.5	50.9	78.9	64.5	83.1	45.1	79.4
Cuba	80 to 84	68.9	72.0	75.7	78.8	61.7	64.5	62.9	63.8	64.8	67.0	61.9	61.3	87.6	88.0	90.9	90.7	84.5	85.2	69.4	72.1	76.8	79.4	66.8	69.0
Cyprus	80 to 84	35.8	72.9	45.1	77.8	33.9	68.2	38.7	77.7	40.1	77.5	43.4	78.1	42.2	81.0	37.8	81.9	56.3	81.8	37.2	71.0	48.2	77.7	43.9	69.5
Czechia	80 to 84	57.8	85.7	66.7	88.9	49.5	82.3	45.7	65.5	50.1	68.3	43.4	63.0	67.3	75.6	69.9	78.8	65.2	72.2	57.1	86.5	68.0	90.0	54.4	85.4
Democratic People's Republic of Korea	80 to 84	65.1	69.3	72.7	76.6	58.4	61.6	33.1	31.8	39.9	38.0	28.1	28.3	49.1	63.0	52.3	66.5	44.9	58.2	79.2	81.3	84.9	87.3	77.3	77.3
Democratic Republic of the Congo	80 to 84	69.4	68.5	78.9	78.0	58.0	53.6	48.9	43.7	50.2	48.2	47.9	41.6	83.1	83.2	89.3	88.0	80.0	78.4	76.2	75.6	84.9	84.1	69.7	66.6
Denmark	80 to 84	79.7	86.7	84.3	90.3	75.1	82.1	70.2	76.2	71.6	78.3	70.3	74.3	83.4	83.0	85.1	86.0	82.9	80.8	80.7	88.4	85.7	91.7	78.8	86.3
Djibouti	80 to 84	75.0	78.1	84.0	86.4	62.3	67.1	45.6	50.2	49.9	50.8	42.6	50.7	84.7	87.5	91.7	92.4	79.1	84.2	83.5	83.4	90.4	90.8	76.3	76.0
Dominica	80 to 84	50.0	55.2	62.4	64.2	35.5	49.0	39.9	46.6	45.5	48.9	36.6	48.4	77.3	80.6	84.2	85.1	67.7	74.5	52.0	55.6	65.1	65.8	46.4	56.6
Dominican Republic	80 to 84	69.8	75.3	75.8	81.0	63.8	69.1	55.9	65.2	57.9	67.1	55.4	64.2	85.4	88.9	89.4	91.5	82.0	85.8	73.9	77.2	79.5	82.9	72.7	74.9
Ecuador	80 to 84	77.9	83.0	83.9	88.6	70.8	75.9	64.4	70.3	66.2	72.8	63.6	68.2	90.9	81.9	92.9	85.0	88.9	79.7	78.7	84.7	85.0	90.2	75.0	80.4
Egypt	80 to 84	0.0	29.6	0.0	20.5	3.9	37.0	0.0	35.1	0.0	31.4	3.1	40.7	41.8	60.2	59.0	65.1	27.9	58.7	0.0	26.9	0.0	18.5	19.2	43.4
El Salvador	80 to 84	74.7	81.7	81.8	86.9	66.3	75.2	63.6	71.6	64.7	72.3	63.8	71.7	87.4	89.0	89.6	90.5	85.4	87.6	77.4	83.0	85.2	88.7	71.8	79.0
Equatorial Guinea	80 to 84	68.2	78.1	78.7	85.2	51.9	67.9	40.3	59.1	45.6	58.9	36.8	61.6	81.0	90.3	87.9	93.0	75.1	87.6	77.1	81.9	86.0	88.7	67.0	74.9
Eritrea	80 to 84	68.0	70.6	77.0	79.7	54.4	56.0	38.6	38.8	44.7	45.2	33.6	33.4	81.2	84.5	88.1	90.0	71.9	77.9	79.0	79.6	85.7	86.8	74.1	72.0
Estonia	80 to 84	38.7	80.3	50.5	84.8	31.8	75.7	44.6	63.9	48.6	67.8	45.0	59.4	74.1	71.7	80.0	78.2	56.2	56.8	34.9	81.2	50.4	86.2	36.2	80.2
Eswatini	80 to 84	70.5	71.4	79.4	78.7	55.8	62.2	44.6	46.7	48.8	48.8	43.2	51.7	83.8	84.7	87.7	86.7	79.4	82.9	77.0	76.0	85.4	83.5	67.4	70.2
Ethiopia	80 to 84	69.8	76.5	79.1	84.0	57.5	67.3	45.6	57.8	49.8	60.6	42.5	55.5	82.7	86.8	91.3	92.9	76.7	83.3	82.7	83.8	88.2	89.4	79.2	79.7
Fiji	80 to 84	74.6	74.7	80.8	81.6	67.6	66.5	45.2	40.6	50.3	47.7	41.4	34.3	76.8	77.9	77.9	79.1	77.2	78.9	85.0	84.4	90.2	89.8	81.3	80.4
Finland	80 to 84	82.7	92.7	85.9	94.9	81.6	89.9	79.3	87.6	79.1	86.9	81.9	88.9	87.4	91.5	87.9	92.0	90.3	92.1	82.1	92.7	86.0	94.9	83.2	91.0
France	80 to 84	74.6	93.1	79.5	94.7	70.5	90.6	67.9	81.0	70.4	81.5	66.3	80.9	81.1	87.0	81.7	89.7	83.0	84.3	75.3	95.1	80.7	96.1	74.8	94.4
Gabon	80 to 84	75.0	77.7	84.1	85.2	60.8	65.8	46.2	50.0	51.4	51.1	42.4	52.4	86.1	89.1	91.5	92.4	81.1	85.2	82.3	82.2	89.6	88.9	74.2	74.8
Gambia	80 to 84	72.0	68.9	79.9	77.5	62.3	58.6	60.2	58.8	54.8	53.0	68.7	68.2	87.6	86.9	89.2	87.7	86.8	87.0	77.1	72.6	86.8	83.4	67.1	62.3
Georgia	80 to 84	51.1	49.5	64.0	64.2	32.7	26.6	42.7	45.1	47.1	50.2	40.9	41.2	71.1	57.8	75.2	59.9	66.4	58.1	57.6	50.5	71.4	67.6	45.4	33.5
Germany	80 to 84	78.4	95.5	84.2	97.5	71.4	92.5	73.9	85.5	76.4	86.2	71.5	84.9	83.6	87.4	85.1	90.2	83.2	84.7	77.9	95.9	84.5	97.4	74.5	94.3
Ghana	80 to 84	70.6	71.9	78.3	79.6	61.8	62.7	57.0	57.1	49.5	50.9	68.2	69.7	88.6	89.3	89.2	88.9	88.8	90.5	75.3	75.8	85.4	85.1	65.9	66.4
Greece	80 to 84	64.6	81.3	70.4	84.3	60.3	78.9	62.6	70.4	63.7	71.3	63.9	70.5	79.1	83.5	81.7	85.7	78.5	82.5	65.5	83.6	72.7	86.6	65.4	84.1
Greenland	80 to 84	74.4	83.3	78.6	87.2	73.7	78.6	67.3	73.9	67.7	74.3	71.2	73.7	51.5	74.4	50.7	76.4	61.8	73.4	76.4	86.1	81.4	89.9	78.2	83.7
Grenada	80 to 84	53.8	64.1	73.1	74.1	19.6	49.4	54.2	57.3	67.1	62.0	36.8	51.9	79.6	83.1	91.3	87.1	62.3	75.8	52.1	63.9	73.1	75.4	27.0	54.8

Guam	80 to 84	82.3	92.3	87.0	96.8	76.9	85.3	65.6	77.4	66.8	81.0	65.4	72.7	81.7	91.5	81.3	92.9	85.2	90.8	87.2	96.6	91.7	99.9	83.9	91.6
Guatemala	80 to 84	74.7	77.0	81.1	83.0	67.7	70.2	62.6	67.4	62.8	67.6	63.3	67.7	94.6	91.3	97.5	93.7	91.3	88.9	77.2	79.8	84.0	86.2	73.3	75.6
Guinea	80 to 84	70.7	69.7	77.1	76.6	63.9	62.3	61.2	62.0	52.3	52.7	69.0	70.4	87.4	87.6	88.6	87.8	87.1	88.0	75.7	73.2	84.9	83.3	68.9	65.7
Guinea-Bissau	80 to 84	64.3	62.8	74.5	72.3	52.1	52.0	52.6	48.5	44.8	41.6	61.3	62.7	82.6	82.3	85.6	82.9	81.5	83.6	69.7	68.0	83.3	80.1	57.3	56.1
Guyana	80 to 84	48.2	55.4	59.2	62.8	37.0	50.2	47.5	54.5	53.5	55.3	42.0	56.8	80.6	83.0	86.7	87.6	74.7	77.8	50.8	54.7	62.9	63.8	47.0	55.2
Haiti	80 to 84	31.0	37.6	40.8	44.1	26.3	34.6	36.0	39.2	40.5	42.0	34.5	39.3	60.7	65.2	69.1	70.1	54.5	63.9	31.9	37.9	41.9	44.9	39.6	44.9
Honduras	80 to 84	66.8	54.0	75.7	63.1	56.5	45.4	55.7	41.7	57.5	40.8	55.2	46.3	80.7	67.4	84.6	67.8	78.4	70.8	69.6	57.7	79.7	68.8	63.3	53.6
Hungary	80 to 84	61.5	78.4	69.9	83.4	53.5	73.2	32.7	61.3	37.4	64.0	32.0	59.6	78.4	77.2	81.3	80.0	74.6	73.6	64.1	79.0	73.4	84.8	61.3	77.0
Iceland	80 to 84	84.0	92.9	87.8	94.1	79.8	90.7	79.7	85.4	81.3	86.0	78.4	85.0	85.3	87.5	86.2	88.4	85.9	87.1	84.2	93.6	88.4	94.4	82.2	92.6
India	80 to 84	67.4	69.2	72.6	73.9	61.7	64.4	68.0	69.0	70.1	70.1	66.0	68.4	71.2	76.7	78.0	79.2	67.8	76.0	69.4	70.5	75.0	76.2	68.7	69.9
Indonesia	80 to 84	64.2	62.8	69.8	70.2	60.2	56.2	44.5	42.3	43.5	42.2	49.5	46.4	68.0	68.0	69.4	68.8	69.1	69.9	72.0	68.8	78.0	77.0	71.4	65.9
Iran (Islamic Republic of)	80 to 84	48.9	67.6	56.6	69.6	44.3	65.5	54.8	66.6	56.6	65.9	55.3	67.6	66.8	77.8	70.5	79.3	63.8	77.5	46.2	66.5	56.3	69.9	48.4	68.6
Iraq	80 to 84	49.0	52.5	59.1	62.9	38.6	41.4	47.2	49.0	53.1	54.3	42.2	44.7	75.5	76.2	82.6	80.8	67.5	71.7	48.9	52.2	60.4	64.6	45.9	47.7
Ireland	80 to 84	73.8	84.9	78.8	88.5	69.5	80.4	78.1	83.5	78.9	83.6	78.3	83.5	80.8	81.1	82.6	84.1	80.7	79.0	72.2	85.0	78.3	89.0	71.6	82.7
Israel	80 to 84	84.8	93.5	86.1	94.6	83.3	91.8	67.8	75.4	68.6	76.6	67.9	74.5	90.5	85.8	92.6	90.9	88.7	81.1	87.3	96.7	88.4	96.5	88.2	97.3
Italy	80 to 84	66.1	81.9	73.0	85.6	59.4	77.7	68.9	73.4	71.4	75.1	67.1	72.2	83.7	81.1	84.7	85.4	84.0	77.1	64.5	83.8	73.2	87.3	62.3	82.7
Jamaica	80 to 84	57.2	61.1	65.3	68.8	50.1	53.5	60.0	57.9	62.2	61.0	59.1	56.0	86.5	83.4	89.1	87.3	84.1	79.6	53.7	59.9	63.8	68.7	53.3	58.5
Japan	80 to 84	79.4	96.5	84.9	99.7	73.1	91.5	83.4	93.3	86.4	96.9	79.8	89.2	86.9	96.8	87.7	96.8	89.1	98.9	77.8	95.1	83.7	97.3	75.3	92.3
Jordan	80 to 84	62.0	81.7	63.9	82.9	62.8	80.4	54.8	63.5	54.2	63.1	58.4	65.0	87.5	89.3	90.5	92.1	84.4	86.6	61.9	82.2	65.4	84.2	66.9	82.8
Kazakhstan	80 to 84	62.6	64.5	72.7	74.5	46.6	48.2	52.3	48.7	55.5	53.0	52.3	46.3	65.1	54.2	68.2	56.0	62.0	55.4	63.9	67.6	75.6	78.5	51.9	55.6
Kenya	80 to 84	76.9	76.3	83.9	83.5	68.4	66.4	54.0	52.1	55.2	53.7	53.4	52.9	86.4	87.3	92.1	91.4	82.8	83.5	84.7	82.6	89.8	88.6	81.0	77.4
Kiribati	80 to 84	70.7	69.5	80.2	78.4	57.8	58.3	45.9	42.1	53.0	47.1	39.0	38.6	78.7	79.4	82.7	81.6	73.8	78.1	81.6	79.4	89.5	87.4	74.2	72.5
Kuwait	80 to 84	82.2	85.4	83.1	89.5	81.2	80.0	72.3	75.9	74.1	79.8	70.8	72.9	85.2	90.2	88.0	95.0	82.8	86.5	82.8	85.9	83.6	89.9	85.0	82.8
Kyrgyzstan	80 to 84	55.5	69.8	67.5	79.5	37.2	53.6	41.5	59.6	45.4	63.1	41.8	56.4	66.9	60.0	73.0	66.1	58.0	55.0	57.8	71.0	71.3	81.9	44.8	58.7
Lao People's Democratic Republic	80 to 84	51.4	61.1	58.9	67.6	45.6	55.3	34.8	40.8	35.5	40.2	37.3	44.4	57.4	70.4	61.2	72.0	55.8	70.8	58.3	67.0	66.3	74.5	58.8	65.2
Latvia	80 to 84	44.5	70.1	54.4	74.8	39.0	68.5	41.5	57.8	45.2	61.4	42.3	54.9	70.8	71.2	76.5	77.3	56.0	55.1	43.4	69.8	56.3	75.7	44.7	72.3
Lebanon	80 to 84	50.5	78.4	60.1	82.2	41.0	74.2	28.9	58.5	44.4	64.2	11.0	53.1	47.8	74.1	60.5	80.2	34.2	68.2	57.2	80.7	65.1	84.6	57.5	79.5
Lesotho	80 to 84	68.7	65.6	78.5	74.7	47.9	49.4	48.8	40.4	55.6	45.8	38.9	38.5	82.7	81.3	88.2	84.5	73.9	75.7	75.6	71.5	84.4	80.5	62.6	61.3
Liberia	80 to 84	69.1	66.9	75.7	74.3	62.0	59.4	60.9	58.1	49.6	47.6	69.0	69.0	87.3	86.9	88.3	87.6	87.1	87.0	73.2	70.6	83.3	81.2	66.4	62.8
Libya	80 to 84	63.5	65.0	69.9	72.0	57.2	57.7	55.7	57.2	58.3	57.4	54.5	58.4	78.8	77.8	83.4	80.2	74.7	76.6	64.6	65.0	71.9	73.6	63.4	62.0
Lithuania	80 to 84	62.0	72.1	68.6	77.6	57.0	67.4	60.6	63.9	62.1	65.3	61.5	65.0	74.7	73.0	79.8	78.8	65.8	59.1	60.1	71.7	69.2	78.8	59.3	70.3
Luxembourg	80 to 84	59.0	83.3	68.4	87.8	49.4	77.6	63.3	72.2	66.2	75.3	61.8	69.3	79.4	82.7	80.6	86.1	80.4	80.2	57.4	85.6	68.9	89.9	53.2	82.8
Madagascar	80 to 84	67.4	69.7	78.7	79.5	53.1	56.0	37.8	38.7	43.2	44.3	33.7	34.7	78.3	82.1	88.4	88.4	72.9	77.9	77.9	78.5	86.9	86.6	70.2	71.0
Malawi	80 to 84	70.9	68.9	78.8	77.3	61.6	58.6	43.6	38.6	45.2	41.8	44.3	39.7	82.8	83.6	88.0	87.7	79.3	80.0	78.9	75.7	86.1	83.8	73.6	69.4
Malaysia	80 to 84	74.6	76.7	78.5	79.8	71.1	74.5	61.4	59.6	60.8	57.6	62.8	62.6	74.9	75.9	77.4	76.7	73.6	77.7	80.2	82.4	83.9	85.0	79.8	83.3
Maldives	80 to 84	66.4	72.7	67.0	77.4	61.9	67.4	44.4	58.5	36.7	55.9	46.0	60.0	67.3	78.4	69.4	79.5	68.4	78.7	74.6	77.3	75.4	82.4	74.0	75.2
Mali	80 to 84	70.7	70.9	75.9	76.2	66.0	65.8	61.5	64.7	50.9	51.5	69.5	72.2	85.5	87.5	86.4	87.1	85.5	87.8	76.3	74.5	83.6	82.9	72.0	69.3
Malta	80 to 84	68.6	81.7	76.1	85.9	60.5	77.0	69.3	82.4	71.4	83.4	68.3	81.4	90.6	91.5	92.6	94.9	88.3	87.6	67.6	80.8	76.6	85.3	63.7	79.5
Marshall Islands	80 to 84	63.9	67.0	73.0	74.8	53.2	58.3	37.9	39.8	43.7	45.7	33.7	35.1	66.6	73.6	69.0	74.3	65.4	74.6	76.9	78.4	83.8	84.1	73.2	75.7
Mauritania	80 to 84	70.8	72.6	77.4	79.0	64.6	65.7	57.0	65.2	46.2	54.4	71.0	74.2	87.8	90.0	88.2	89.5	88.4	90.8	76.4	75.6	85.2	84.7	68.9	68.7
Mauritius	80 to 84	69.6	77.8	76.5	83.7	62.6	70.8	57.5	63.5	60.4	66.6	56.2	60.9	69.9	71.6	74.8	76.1	62.3	66.5	71.2	79.9	78.9	85.9	68.5	76.6
Mexico	80 to 84	78.3	84.1	84.2	88.7	71.1	78.4	71.6	74.1	72.8	75.8	71.2	72.7	92.1	85.2	93.6	87.1	90.4	84.0	78.4	85.3	85.4	90.0	73.6	82.0
Micronesia (Federated States of)	80 to 84	64.7	68.4	73.1	76.1	55.4	59.9	39.7	43.5	46.1	49.0	34.3	39.3	67.6	74.3	69.0	75.2	68.3	75.6	77.8	79.1	83.9	85.1	75.2	76.2
Monaco	80 to 84	74.2	83.6	80.2	87.1	68.0	79.9	71.3	80.4	74.4	81.8	68.5	79.4	79.0	87.7	80.2	88.6	79.8	87.5	74.1	83.2	80.7	87.0	72.1	82.5
Mongolia	80 to 84	50.4	66.4	65.7	75.9	26.6	53.7	22.2	47.0	31.0	47.6	14.5	47.5	34.8	47.4	45.9	52.9	25.1	44.5	84.4	86.4	92.2	93.0	74.6	79.0
Montenegro	80 to 84	54.5	42.8	64.0	51.4	44.4	39.3	34.1	20.3	43.8	27.0	22.8	18.0	74.2	66.8	76.0	65.1	73.9	72.3	76.3	66.6	82.9	73.9	73.1	66.3
Morocco	80 to 84	49.9	57.1	51.9	60.7	48.3	54.9	44.5	50.1	41.7	48.7	48.9	53.5	66.1	70.3	71.4	73.0	63.1	69.1	50.9	57.2	54.6	62.5	54.9	59.8
Mozambique	80 to 84	66.4	66.1	75.8	76.2	54.5	50.6	48.5	44.2	53.2	50.5	44.8	38.2	80.5	82.1	87.4	87.2	75.8	76.9	74.1	72.7	82.8	82.3	67.8	63.1

Myanmar	80 to 84	51.7	60.0	61.4	69.2	42.3	50.1	33.4	36.8	32.6	39.9	36.9	36.1	61.1	69.9	66.6	73.8	56.5	66.8	59.5	66.7	70.3	76.2	55.3	62.2
Namibia	80 to 84	72.2	72.7	80.3	80.6	60.6	61.3	50.9	50.4	52.2	52.8	55.3	52.9	87.2	87.1	89.8	89.4	84.5	84.8	77.1	76.6	85.5	84.8	67.9	68.2
Nauru	80 to 84	68.1	62.5	75.6	68.6	59.4	57.9	46.2	19.9	51.1	11.8	42.3	30.5	72.6	68.7	70.2	65.6	76.1	79.0	77.7	77.2	84.2	82.6	74.3	75.2
Nepal	80 to 84	59.4	64.6	66.5	72.7	52.4	55.6	60.5	65.2	63.2	68.3	58.5	62.5	67.6	74.1	75.7	79.5	63.0	70.4	61.8	66.0	69.4	75.4	61.1	61.8
Netherlands	80 to 84	82.8	87.5	87.1	90.6	78.2	83.7	74.8	78.9	77.0	80.2	72.8	77.9	82.4	84.8	84.0	87.9	81.9	81.7	83.1	88.6	87.8	91.5	81.2	87.2
New Zealand	80 to 84	77.0	87.0	82.3	89.2	71.5	84.3	73.9	75.7	77.1	78.2	69.8	73.3	78.8	79.6	81.4	81.5	76.4	79.0	76.7	88.2	82.7	90.5	74.8	87.3
Nicaragua	80 to 84	79.6	84.7	86.0	89.2	71.4	78.9	71.0	75.1	73.2	76.6	69.1	73.9	90.3	92.0	92.8	93.7	87.2	89.8	81.3	85.9	88.3	90.7	75.6	82.4
Niger	80 to 84	70.6	67.8	78.6	76.6	60.9	57.5	58.4	57.4	55.1	52.5	62.9	63.2	84.2	84.1	87.8	87.2	82.5	82.8	77.3	73.5	86.5	84.4	69.0	63.9
Nigeria	80 to 84	66.9	75.0	75.6	82.6	56.7	65.7	57.3	69.0	52.7	64.6	65.4	73.9	85.1	91.2	87.4	92.3	83.9	90.6	72.0	77.4	83.1	87.3	61.4	68.5
Niue	80 to 84	70.6	73.5	78.3	81.1	61.3	64.2	45.4	47.1	51.5	54.1	39.4	39.9	74.2	79.1	75.2	79.9	76.3	81.5	80.8	82.6	86.9	88.6	76.8	78.2
North Macedonia	80 to 84	42.8	43.7	52.6	53.4	33.4	35.7	25.4	35.2	29.4	40.2	23.6	32.2	41.6	49.1	45.1	48.9	43.1	53.5	43.6	42.6	55.9	55.3	41.2	41.6
Northern Mariana Islands	80 to 84	72.2	77.9	76.8	83.0	68.0	72.1	52.6	57.9	49.6	57.3	56.2	57.8	72.6	76.9	66.9	74.4	82.0	81.3	82.5	86.8	86.3	90.7	81.4	84.0
Norway	80 to 84	80.5	92.5	84.5	94.1	76.7	89.9	77.3	82.6	80.9	83.1	72.8	82.3	80.7	86.4	81.1	88.6	83.7	84.8	80.0	93.0	84.5	94.6	79.2	91.3
Oman	80 to 84	56.8	71.7	68.4	77.3	42.6	65.1	53.5	67.5	62.8	73.0	39.4	60.4	77.9	84.6	83.9	88.1	69.0	80.8	56.9	71.2	69.5	77.5	50.6	68.8
Pakistan	80 to 84	69.6	69.6	73.4	74.0	64.9	65.4	67.4	66.6	67.9	66.6	66.8	67.6	72.8	75.4	78.1	78.0	71.1	74.9	72.3	71.5	76.5	77.1	71.7	70.8
Palau	80 to 84	72.9	77.1	80.4	79.9	63.7	74.4	54.3	58.0	60.5	56.3	48.2	59.9	78.9	81.3	79.2	78.2	81.0	87.2	80.3	83.4	86.4	85.6	76.6	83.4
Palestine	80 to 84	28.8	50.0	44.7	60.0	11.7	40.2	49.8	59.1	54.2	60.1	46.4	59.2	80.2	85.0	85.1	88.8	74.0	79.5	21.7	46.2	42.3	59.6	13.5	42.1
Panama	80 to 84	73.9	80.8	80.7	87.8	66.1	71.9	67.1	74.7	68.5	78.4	66.3	71.3	86.6	85.1	87.6	88.9	86.7	81.5	74.5	81.1	82.3	88.1	70.0	75.6
Papua New Guinea	80 to 84	57.9	59.7	61.7	62.7	54.2	56.7	30.0	28.5	26.7	23.5	33.0	32.7	64.3	69.2	61.7	66.0	69.3	74.0	76.2	76.7	77.1	77.7	78.5	78.6
Paraguay	80 to 84	66.3	72.6	72.5	77.8	60.5	67.3	56.1	60.2	58.8	62.6	55.1	59.0	81.5	82.3	84.1	83.6	80.4	83.0	67.7	73.3	74.9	79.2	66.1	71.6
Peru	80 to 84	75.2	83.1	82.3	88.6	66.5	76.4	63.4	71.7	65.9	72.9	61.7	70.9	85.0	87.7	87.9	88.7	82.8	87.7	77.0	84.1	84.5	89.7	71.9	80.0
Philippines	80 to 84	71.1	70.1	77.9	78.3	63.9	59.2	53.6	51.4	55.2	56.2	53.7	47.5	79.0	79.7	82.1	82.6	76.1	76.1	77.1	75.2	83.7	83.2	73.8	69.5
Poland	80 to 84	42.0	75.5	54.8	81.0	28.6	69.8	46.3	60.3	51.3	64.9	42.6	55.3	76.3	76.9	79.4	80.2	72.4	72.7	39.3	76.2	55.4	82.5	34.1	74.3
Portugal	80 to 84	55.8	75.0	65.0	79.9	47.3	70.0	61.0	68.4	63.5	70.8	60.1	66.7	74.7	74.6	76.0	77.0	76.1	74.1	54.4	76.5	65.8	81.8	51.5	75.4
Puerto Rico	80 to 84	77.0	89.2	83.4	94.5	69.2	81.8	75.8	81.0	77.6	83.9	74.3	78.0	90.1	91.4	92.2	94.5	87.8	86.9	75.6	89.9	82.9	94.5	71.3	85.2
Qatar	80 to 84	60.3	75.7	66.6	79.8	52.0	70.7	28.0	64.9	40.1	69.2	16.9	60.4	29.9	72.5	66.4	82.2	0.0	63.2	68.2	78.0	72.7	82.1	67.5	76.8
Republic of Korea	80 to 84	70.7	89.1	77.3	92.4	64.8	84.9	66.3	87.7	71.1	91.3	59.1	82.4	67.0	87.1	68.6	88.3	70.9	87.9	75.0	89.2	81.2	91.9	73.9	88.1
Republic of Moldova	80 to 84	39.9	67.6	47.6	71.3	37.2	66.3	32.8	46.1	38.0	49.0	31.1	46.3	64.6	74.0	74.1	79.4	48.6	66.5	45.4	70.1	54.6	74.9	49.7	72.4
Romania	80 to 84	54.5	66.9	62.4	73.4	47.8	61.2	34.5	40.1	37.2	43.2	35.7	40.2	62.8	56.9	66.5	60.1	60.2	54.7	55.7	68.5	65.7	76.1	54.4	67.1
Russian Federation	80 to 84	18.6	51.2	37.2	63.1	0.0	35.8	45.9	50.8	50.2	55.8	45.6	45.9	69.3	61.4	73.5	65.5	57.3	52.4	9.2	49.4	34.7	64.2	0.0	40.7
Rwanda	80 to 84	62.4	72.1	73.5	80.8	47.0	58.3	32.5	48.0	42.1	55.9	22.6	37.4	78.3	85.4	87.9	91.1	70.5	79.2	74.1	80.0	82.2	87.0	68.8	73.4
Saint Kitts and Nevis	80 to 84	49.2	57.1	60.5	66.4	38.0	48.4	49.7	54.1	54.5	58.1	45.9	51.2	76.7	79.8	81.5	83.5	70.7	75.3	49.3	56.8	62.3	67.5	45.8	55.1
Saint Lucia	80 to 84	53.5	67.7	64.2	77.3	42.2	55.6	54.1	59.0	59.9	67.2	48.2	51.3	83.1	86.4	86.2	89.5	79.1	82.9	52.0	68.3	64.0	77.7	49.5	63.2
Saint Vincent and the Grenadines	80 to 84	50.1	66.8	59.4	77.4	43.0	53.1	35.5	59.5	38.7	65.4	36.0	54.3	83.5	87.0	87.9	91.9	78.7	81.8	52.9	67.7	62.7	78.7	54.6	59.8
Samoa	80 to 84	67.6	71.8	76.5	78.3	56.4	65.0	40.4	42.1	49.4	46.6	30.3	39.7	71.4	76.9	73.2	76.5	71.4	80.6	80.7	83.6	86.7	88.6	77.1	80.6
San Marino	80 to 84	79.2	89.7	83.3	91.4	75.5	87.6	74.5	84.2	74.9	83.7	76.3	85.5	88.1	95.2	89.5	96.5	87.3	93.5	79.4	89.6	84.3	91.8	78.1	88.6
Sao Tome and Principe	80 to 84	76.3	76.5	82.4	83.0	69.5	68.3	64.2	66.3	61.1	62.7	72.8	74.0	91.4	92.2	92.3	92.5	90.8	92.5	81.0	79.5	88.1	87.3	74.0	71.7
Saudi Arabia	80 to 84	46.9	65.8	51.7	64.9	42.0	62.8	44.6	51.8	48.2	54.8	42.6	49.5	78.7	81.9	83.0	86.8	74.7	77.6	48.3	67.4	54.0	66.8	50.6	69.0
Senegal	80 to 84	71.6	69.9	79.9	78.6	61.8	59.4	62.0	60.7	54.9	53.8	68.5	68.7	87.6	87.3	89.8	88.2	86.6	87.5	75.8	73.6	86.4	84.5	66.7	63.4
Serbia	80 to 84	29.9	57.1	40.0	60.7	25.6	55.9	20.1	43.9	26.9	48.3	17.0	40.8	41.4	62.6	40.6	61.9	47.9	66.6	28.8	56.7	41.9	62.1	34.0	61.2
Seychelles	80 to 84	70.7	76.2	79.0	81.4	60.1	71.4	53.0	60.7	55.1	61.0	53.3	63.9	78.1	83.6	81.4	85.6	73.1	81.6	74.5	79.2	83.4	84.8	67.7	77.1
Sierra Leone	80 to 84	70.7	69.8	78.4	77.0	62.0	62.4	62.1	60.6	54.5	52.8	69.0	70.6	87.8	88.1	89.3	88.7	87.3	88.3	74.4	73.4	85.0	83.0	66.2	65.9
Singapore	80 to 84	86.4	100.0	88.3	100.0	85.6	100.0	90.2	100.0	91.5	100.0	89.6	100.0	95.1	100.0	94.8	100.0	98.3	100.0	85.2	98.8	86.8	98.1	86.9	100.0
Slovakia	80 to 84	77.2	84.1	82.4	88.4	72.2	79.3	37.7	53.2	42.6	56.6	35.5	51.4	73.9	77.0	76.1	79.5	72.2	74.2	79.3	85.1	84.7	89.5	77.8	83.0
Slovenia	80 to 84	60.5	80.3	69.9	85.4	49.8	74.1	56.2	62.7	59.3	67.9	54.4	57.2	74.8	77.6	77.6	80.2	71.5	75.8	59.3	81.6	70.9	86.9	53.7	78.7
Solomon Islands	80 to 84	65.8	67.9	72.7	74.3	58.6	61.4	45.0	45.2	50.2	49.6	40.9	42.2	66.7	73.4	68.1	74.3	68.2	74.3	79.8	80.4	83.7	84.5	79.0	79.9
Somalia	80 to 84	62.7	66.8	74.6	76.8	47.1	51.4	29.1	34.3	39.6	43.0	19.5	24.8	75.0	80.8	87.1	88.1	67.3	73.2	76.8	78.0	84.2	85.3	72.0	72.0
South Sudan	80 to 84	85.5	80.7	81.4	82.4	58.1	62.9	66.1	58.1	49.7	50.1	37.2	42.8	93.2	89.8	90.4	90.7	78.4	80.6	88.5	83.3	88.5	88.5	75.3	76.2

Spain	80 to 84	71.2	73.4	77.9	93.2	62.6	91.5	42.8	46.1	71.2	77.4	66.5	72.8	82.7	83.9	87.4	86.1	82.9	75.9	81.3	81.5	78.9	95.2	66.4	96.3
Sri Lanka	80 to 84	70.7	92.6	64.7	72.6	45.1	56.2	68.5	75.0	32.1	44.2	30.7	43.7	85.1	81.0	83.9	86.7	78.0	81.3	70.5	95.8	69.0	75.6	55.0	64.0
Sudan	80 to 84	55.1	64.5	41.8	59.5	35.0	56.1	30.7	42.3	36.3	45.0	28.8	52.2	80.7	84.2	68.2	69.4	47.0	67.2	58.6	66.9	45.8	62.7	47.9	61.5
Suriname	80 to 84	38.4	58.6	66.2	74.7	54.6	60.7	31.5	48.2	55.3	63.8	53.7	56.9	56.3	67.0	85.9	89.6	79.7	81.0	42.6	59.8	69.2	76.9	62.6	68.0
Sweden	80 to 84	59.8	67.8	84.4	92.6	78.9	88.4	53.4	60.0	80.3	87.0	82.2	87.3	82.4	85.6	83.4	87.9	87.7	87.1	62.2	69.7	84.7	92.8	80.6	89.7
Switzerland	80 to 84	81.5	91.0	80.7	93.4	71.5	89.6	80.7	87.2	74.7	86.4	77.7	87.5	84.0	87.1	80.5	86.3	80.5	82.3	80.9	91.0	81.5	93.9	74.0	91.5
Syrian Arab Republic	80 to 84	75.9	91.8	54.8	59.2	47.5	53.8	75.2	86.9	49.8	52.6	48.6	50.2	79.4	83.9	77.2	78.8	69.3	74.5	75.5	92.4	57.6	61.6	56.2	61.2
Taiwan (Province of China)	80 to 84	50.7	56.5	82.6	93.8	75.4	85.5	48.3	50.8	61.0	77.2	59.6	72.7	72.4	75.9	79.2	81.6	72.9	74.4	52.7	57.9	88.3	96.8	84.1	90.9
Tajikistan	80 to 84	78.9	90.3	65.2	72.2	40.1	52.7	59.6	74.9	42.1	51.4	42.6	56.2	76.1	78.2	66.2	67.7	62.4	71.1	85.2	94.0	71.0	77.0	49.2	59.2
Thailand	80 to 84	53.7	63.2	77.0	84.4	67.1	72.7	40.4	53.6	55.1	64.8	60.0	62.7	62.9	68.5	56.8	71.1	52.4	66.5	57.9	66.3	85.3	90.1	79.0	83.0
Timor-Leste	80 to 84	71.7	78.9	61.3	63.8	52.9	52.2	56.3	63.1	31.1	31.9	42.3	40.3	53.2	67.8	63.9	66.3	64.0	67.8	80.5	85.7	71.6	72.7	66.4	63.7
Togo	80 to 84	56.9	57.9	77.7	76.2	61.7	56.6	36.5	35.6	51.8	50.0	67.9	66.1	62.2	65.5	88.6	87.1	86.4	86.0	66.2	65.4	84.8	82.8	66.4	60.5
Tokelau	80 to 84	70.2	67.4	73.9	79.2	53.3	59.2	58.9	54.5	49.8	50.9	35.9	37.2	87.0	85.9	70.7	79.4	71.3	80.2	74.8	72.0	83.1	87.2	71.4	76.2
Tonga	80 to 84	64.6	70.5	82.7	83.1	70.6	73.5	42.7	43.9	61.3	60.8	56.1	57.9	69.8	78.5	81.2	82.5	83.1	85.9	75.7	80.7	90.6	89.8	81.5	83.1
Trinidad and Tobago	80 to 84	77.0	78.3	74.3	77.3	58.2	63.3	58.2	58.5	66.3	69.0	61.9	64.8	81.0	82.7	86.7	86.7	80.5	80.3	85.4	85.5	75.2	77.8	61.6	66.9
Tropical Latin America	80 to 84	66.6	70.6	68.1	82.8	51.2	72.0	63.6	66.5	62.0	70.0	55.5	63.9	83.6	83.5	88.0	83.2	85.9	77.4	65.9	70.0	68.1	83.9	54.9	75.7
Tunisia	80 to 84	48.3	63.4	55.1	68.6	40.0	58.2	44.0	54.8	49.8	56.0	39.5	55.1	66.6	75.4	74.6	78.1	61.7	73.9	49.2	63.8	56.8	70.5	48.3	63.1
Turkey	80 to 84	61.1	72.8	69.1	79.0	53.1	66.2	48.7	63.5	53.8	66.6	44.8	61.2	56.3	69.3	66.7	75.5	44.4	62.7	64.6	74.8	72.9	81.3	62.8	72.7
Turkmenistan	80 to 84	66.4	63.7	75.3	74.1	54.4	50.1	57.7	47.6	60.3	51.7	56.5	44.7	71.2	58.1	75.5	64.4	66.6	53.0	68.0	66.7	78.2	77.4	60.1	58.8
Tuvalu	80 to 84	59.6	68.4	69.2	75.8	49.2	60.6	31.5	41.7	38.3	46.1	27.0	39.4	64.2	74.8	65.3	74.9	65.9	77.4	73.6	78.4	81.2	84.6	70.1	75.4
Uganda	80 to 84	73.9	79.8	84.3	87.5	58.5	66.8	41.6	51.1	53.5	58.3	29.5	42.9	81.1	87.3	90.8	92.7	74.1	81.6	83.5	86.1	90.4	92.0	76.7	79.3
Ukraine	80 to 84	51.0	64.3	59.1	72.4	48.1	55.7	56.0	59.2	58.9	63.3	55.8	54.8	49.8	62.2	53.8	66.3	42.0	56.0	48.4	63.4	59.4	73.4	51.8	59.5
United Arab Emirates	80 to 84	68.2	72.7	72.2	38.3	64.6	74.6	58.8	57.6	60.5	11.3	58.2	64.5	54.4	52.8	57.7	5.3	54.1	67.4	69.9	74.7	75.0	43.8	69.9	78.6
United Kingdom	80 to 84	73.8	87.5	79.8	90.4	67.9	84.0	75.4	75.5	76.2	76.4	76.2	75.2	79.7	79.1	80.6	81.5	81.8	77.9	72.6	89.5	80.0	92.4	70.0	87.8
United Republic of Tanzania	80 to 84	72.0	76.5	82.5	84.2	58.0	66.0	43.6	51.5	54.3	55.2	33.4	48.9	83.1	87.4	92.4	92.1	76.6	83.4	82.1	81.7	89.4	88.9	75.8	75.1
United States Virgin Islands	80 to 84	63.7	81.4	72.3	88.0	54.7	72.3	55.0	72.5	58.5	77.2	52.9	67.1	81.0	89.6	85.3	93.4	75.4	84.0	64.5	82.1	73.4	88.6	62.1	76.8
United States of America	80 to 84	87.5	95.4	91.5	97.5	82.6	92.5	80.6	88.1	82.2	88.6	79.0	87.9	84.3	81.4	86.8	85.8	81.2	76.3	87.7	96.7	91.8	97.7	84.9	95.6
Uruguay	80 to 84	74.1	81.2	80.1	86.3	68.2	75.0	74.5	79.2	78.6	83.5	68.7	72.2	75.3	77.5	75.3	79.3	78.2	77.7	73.9	81.6	80.5	86.8	72.2	79.2
Uzbekistan	80 to 84	67.1	71.2	75.0	79.6	57.8	59.1	51.7	65.1	52.9	66.4	53.2	64.4	80.2	55.7	86.0	59.6	72.9	55.0	73.5	73.7	81.9	83.4	67.9	64.3
Vanuatu	80 to 84	67.1	70.7	75.9	78.1	57.0	62.4	39.7	42.0	47.8	47.9	32.5	37.3	70.3	75.9	71.3	76.7	71.5	77.1	78.7	81.3	85.6	87.2	74.6	77.6
Venezuela (Bolivarian Republic of)	80 to 84	76.8	74.7	82.5	82.3	70.4	65.0	64.8	65.0	65.8	67.9	65.2	62.6	91.3	84.9	93.4	88.4	88.7	80.9	79.3	76.0	85.4	83.9	75.9	70.4
Viet Nam	80 to 84	50.4	53.4	62.7	65.3	36.2	39.8	33.0	35.9	37.4	41.3	31.9	32.6	62.0	70.4	67.5	74.2	54.4	65.8	58.4	58.5	71.1	71.2	50.9	51.4
Yemen	80 to 84	28.4	37.3	41.4	46.4	18.7	28.4	25.5	29.3	35.2	31.9	15.5	29.4	54.4	56.3	67.4	65.4	32.4	49.4	32.4	38.5	45.6	50.0	35.7	37.3
Zambia	80 to 84	68.2	71.0	78.5	80.1	55.6	58.2	36.5	39.5	40.3	43.1	33.6	37.7	78.1	82.2	87.3	87.4	74.1	78.8	80.3	78.9	87.1	86.3	75.3	72.8
Zimbabwe	80 to 84	74.1	68.4	82.5	77.9	61.3	54.7	40.2	24.5	43.1	27.7	42.5	29.1	76.8	73.9	79.9	76.2	75.9	74.5	79.2	74.1	87.1	83.3	70.5	64.9
Afghanistan	85 to 89	45.2	48.6	62.1	62.9	26.3	33.3	39.6	45.4	59.4	58.6	38.1	48.6	44.6	56.5	68.5	68.6	41.4	55.3	48.9	49.6	62.0	63.4	40.5	41.0
Albania	85 to 89	61.2	58.8	78.1	72.7	32.4	45.3	28.0	23.5	52.9	40.6	16.2	31.8	65.9	68.1	69.9	64.7	62.1	74.4	77.3	75.9	87.5	84.5	62.5	70.1
Algeria	85 to 89	51.1	58.7	38.9	64.6	48.7	58.0	41.0	30.0	11.8	26.8	58.2	54.0	56.9	58.9	44.6	47.6	67.4	71.0	53.9	62.3	45.3	68.9	55.1	64.9
American Samoa	85 to 89	81.8	82.9	87.7	88.1	75.4	77.6	57.4	58.3	68.8	69.6	58.6	59.4	74.2	76.5	61.4	64.4	88.4	89.1	88.3	89.0	91.3	91.4	87.7	89.7
Andorra	85 to 89	88.3	90.6	90.9	92.9	87.0	88.4	83.9	86.1	85.8	87.7	88.7	90.0	85.2	86.3	83.5	84.7	87.9	88.5	87.5	89.9	90.1	92.2	88.6	90.0
Angola	85 to 89	84.6	85.4	91.6	92.4	73.2	72.5	57.0	55.9	66.1	65.5	61.7	61.1	87.8	89.2	92.3	92.5	84.7	86.1	89.3	88.8	94.1	93.8	82.5	80.3
Antigua and Barbuda	85 to 89	73.1	77.5	82.9	84.8	60.8	70.2	62.0	66.6	71.8	74.6	63.9	69.0	85.0	85.3	87.7	87.8	80.9	82.8	74.1	78.3	83.7	85.3	67.0	75.8
Argentina	85 to 89	82.0	89.5	87.8	92.9	75.9	85.3	75.3	80.0	82.5	86.0	73.8	77.8	71.2	80.2	60.2	78.7	80.8	83.0	83.0	90.3	88.3	93.0	80.7	89.9
Armenia	85 to 89	71.3	79.1	81.1	83.5	61.5	78.4	57.5	71.0	67.3	77.1	62.7	74.9	69.1	72.6	67.8	71.7	72.7	75.6	73.8	79.5	83.4	83.8	67.7	82.1
Australia	85 to 89	84.3	92.3	88.1	93.5	83.1	91.0	74.8	78.9	79.7	82.9	79.5	81.8	83.3	83.0	83.6	85.1	82.7	80.7	84.4	92.6	88.1	93.4	86.0	94.0
Austria	85 to 89	83.0	99.8	88.0	100.0	80.1	98.0	93.5	92.2	93.4	92.9	98.4	95.1	93.9	92.1	93.6	92.4	93.8	91.2	79.6	97.9	85.7	98.0	79.5	98.6
Azerbaijan	85 to 89	73.4	77.6	84.2	85.3	51.0	68.6	51.0	61.6	64.7	70.7	49.5	64.5	84.5	84.5	85.4	85.2	82.1	83.6	80.8	83.2	88.9	89.2	65.3	78.6
Bahamas	85 to 89	80.9	82.9	87.1	88.3	75.2	77.4	69.1	71.1	76.6	78.6	71.2	72.0	89.9	90.4	91.8	92.5	86.4	87.5	82.0	83.6	87.6	88.3	80.9	82.5

Bahrain	85 to 89	45.1	60.8	64.8	72.4	24.4	49.7	33.3	48.8	52.5	63.4	33.6	48.9	56.1	67.5	60.5	70.7	51.6	67.1	48.9	63.3	68.2	74.4	36.5	58.4
Bangladesh	85 to 89	65.4	64.6	75.4	77.4	55.7	50.2	60.3	60.1	68.8	71.3	63.8	60.4	58.8	66.1	61.1	66.8	64.5	70.2	67.8	66.3	77.2	79.0	63.6	57.1
Barbados	85 to 89	67.3	74.7	76.9	82.2	62.9	68.6	61.8	66.9	70.4	75.3	66.6	68.5	82.8	84.6	83.8	86.6	81.8	83.3	66.8	74.5	77.0	82.2	67.5	73.0
Belarus	85 to 89	64.4	71.0	73.8	80.2	63.5	65.0	65.9	65.1	74.2	74.6	68.4	63.2	65.3	66.5	65.0	66.4	63.3	62.2	64.2	71.5	74.6	81.2	67.9	70.0
Belgium	85 to 89	78.7	91.0	85.7	93.3	71.7	88.5	73.5	76.3	79.9	82.2	75.6	77.5	82.8	79.6	81.9	80.7	84.9	79.3	78.9	91.9	86.1	93.5	75.4	92.6
Belize	85 to 89	79.8	80.1	86.0	85.2	73.5	75.3	70.1	67.5	76.7	73.7	72.7	71.7	90.7	89.5	93.2	90.4	87.8	88.7	80.4	81.0	86.4	85.8	78.4	80.1
Benin	85 to 89	79.1	80.1	85.1	86.7	73.4	73.5	65.6	66.7	63.2	68.0	78.3	79.6	86.0	88.0	84.2	86.8	87.8	89.2	82.3	82.8	88.8	89.3	77.0	76.3
Bermuda	85 to 89	79.4	88.7	86.5	93.3	71.3	81.5	75.5	82.9	81.8	88.8	76.2	80.6	89.4	91.1	92.3	96.2	84.7	85.6	78.5	87.7	85.9	92.0	74.3	84.3
Bhutan	85 to 89	76.7	80.5	84.0	86.8	69.7	73.5	70.1	74.0	77.0	79.4	72.6	76.7	76.2	80.7	77.8	80.6	76.8	82.2	78.2	81.4	85.2	87.6	75.4	77.7
Bolivia (Plurinational State of)	85 to 89	75.6	78.8	83.3	85.6	68.1	71.9	61.7	65.6	69.0	72.3	67.6	70.2	81.4	81.0	80.8	79.3	83.1	84.0	76.9	79.9	84.5	86.5	73.1	76.4
Bosnia and Herzegovina	85 to 89	68.1	72.4	78.2	80.7	58.5	65.9	53.9	59.8	66.8	69.3	53.6	63.2	66.6	74.8	67.6	73.2	69.0	78.1	69.5	73.2	79.8	81.9	64.7	70.5
Botswana	85 to 89	83.5	89.1	90.1	93.7	71.9	79.2	57.0	68.5	66.6	75.4	63.6	73.5	89.4	92.6	90.7	93.7	87.6	90.2	87.1	90.6	92.3	94.4	79.1	83.6
Brazil	85 to 89	73.4	85.2	81.9	89.9	64.9	79.7	67.8	72.7	75.9	79.8	69.5	73.3	89.7	82.7	89.6	83.1	89.7	83.0	72.8	85.4	82.0	89.9	68.2	83.2
Brunei Darussalam	85 to 89	91.3	88.5	90.8	91.8	94.6	85.9	74.8	78.0	82.4	84.8	73.8	73.2	86.4	88.3	82.6	87.9	95.6	90.2	92.5	88.9	90.9	91.6	99.3	90.9
Bulgaria	85 to 89	53.6	63.6	69.1	76.6	38.4	49.5	30.8	56.5	50.6	67.3	30.3	59.2	68.9	57.2	69.1	53.6	72.0	64.7	58.7	64.6	73.6	78.3	49.6	54.6
Burkina Faso	85 to 89	81.7	81.6	90.0	89.2	69.5	70.4	69.5	69.3	74.9	74.0	73.4	75.1	88.7	89.6	93.4	92.1	86.0	87.7	85.2	84.7	93.3	92.0	74.3	74.3
Burundi	85 to 89	79.5	82.1	88.3	90.2	66.3	68.8	47.6	52.4	63.5	66.8	46.4	51.5	82.2	85.3	90.4	92.4	77.7	81.3	85.4	86.7	90.8	92.1	80.3	80.5
Cabo Verde	85 to 89	86.2	83.5	91.3	90.3	79.8	73.3	76.3	75.0	77.2	78.3	84.0	82.3	94.0	95.3	95.8	96.4	91.9	93.1	88.6	84.5	93.6	91.3	82.6	74.6
Cambodia	85 to 89	65.0	64.4	76.0	76.2	56.3	53.9	40.1	35.4	53.0	51.6	48.7	40.5	63.4	67.3	61.2	64.8	68.3	72.3	72.3	71.0	81.6	81.1	68.2	65.7
Cameroon	85 to 89	80.3	80.4	87.1	87.5	72.4	71.7	66.8	67.7	69.0	70.4	75.6	77.2	87.5	88.9	88.3	89.0	87.5	88.9	84.2	83.0	90.8	90.1	77.1	74.9
Canada	85 to 89	90.3	98.1	93.0	97.8	88.0	97.6	89.8	92.6	91.6	93.4	91.4	94.8	88.1	85.6	89.0	87.7	85.7	83.0	89.1	97.0	91.9	96.3	89.5	99.3
Central African Republic	85 to 89	78.7	77.0	86.9	86.1	65.8	61.9	47.4	42.4	59.4	55.7	54.4	50.0	84.6	84.1	87.3	86.9	81.4	81.2	84.1	82.5	90.0	89.2	77.0	73.3
Chad	85 to 89	81.2	80.3	87.7	87.5	73.9	71.6	68.4	69.2	70.5	70.1	78.0	77.6	88.4	87.9	89.0	87.7	88.2	88.6	84.3	82.4	90.7	90.1	77.5	74.3
Chile	85 to 89	82.2	88.5	87.8	92.5	76.6	83.1	81.5	88.1	87.3	91.9	78.6	85.7	82.8	80.0	77.9	79.1	88.3	82.0	81.5	87.9	87.0	91.7	79.9	85.8
China	85 to 89	64.0	78.1	78.3	88.9	43.7	57.7	22.8	38.2	51.3	65.0	0.0	14.8	0.0	58.3	0.0	63.5	5.6	55.9	77.7	84.8	86.5	92.3	69.3	73.5
Colombia	85 to 89	85.7	91.0	90.2	94.3	80.6	85.9	72.4	81.4	77.7	85.3	76.8	83.3	90.9	86.5	90.9	86.8	90.6	86.3	86.1	90.8	90.5	93.8	84.0	88.9
Comoros	85 to 89	84.9	86.8	91.4	93.3	74.6	75.9	53.7	57.6	62.5	65.7	59.7	62.4	88.3	89.4	92.9	94.3	85.0	86.4	89.7	90.2	93.9	94.7	84.5	83.9
Congo	85 to 89	81.9	84.0	89.1	90.5	71.7	74.5	49.0	54.5	57.0	60.5	60.2	65.1	86.5	88.8	88.8	90.8	84.9	87.4	86.9	87.2	92.1	92.3	80.5	81.3
Cook Islands	85 to 89	82.1	90.3	88.6	93.8	74.1	84.9	55.6	70.0	69.7	80.1	52.8	66.5	75.4	85.1	65.9	80.8	88.0	91.9	88.6	94.4	91.9	95.5	86.7	94.5
Costa Rica	85 to 89	88.6	90.8	92.3	94.2	83.7	85.9	80.1	85.2	83.9	88.5	82.6	86.3	91.9	89.9	93.1	91.8	90.2	87.5	88.1	89.8	91.7	93.0	86.1	87.9
Coted'Ivoire	85 to 89	82.4	82.2	88.9	89.0	74.3	73.2	71.2	70.7	73.4	72.9	78.6	79.7	89.4	90.4	89.4	90.2	89.6	90.5	84.6	84.1	91.3	91.1	77.8	75.7
Croatia	85 to 89	65.4	81.2	78.7	86.1	47.7	78.8	53.7	55.3	69.4	67.6	44.4	55.2	59.5	53.8	58.8	50.0	62.7	61.5	66.9	82.4	80.2	87.1	54.8	83.4
Cuba	85 to 89	76.8	80.2	83.9	86.6	69.5	73.2	68.7	70.9	75.5	78.4	71.7	72.1	91.5	91.1	92.6	92.3	90.2	89.4	76.5	80.0	83.9	86.4	73.1	76.9
Cyprus	85 to 89	23.8	78.8	49.8	86.1	0.3	70.4	18.6	79.7	42.0	83.7	21.4	82.3	27.2	80.6	19.7	79.2	43.9	83.0	27.5	77.7	52.9	86.1	13.2	71.1
Czechia	85 to 89	70.2	87.7	80.2	91.4	60.9	84.1	59.7	69.8	69.9	77.7	62.2	70.2	72.0	73.3	70.7	72.6	74.4	75.2	71.0	88.0	81.4	91.5	65.8	87.4
Democratic People's Republic of Korea	85 to 89	76.7	79.0	84.7	86.2	67.7	71.2	40.0	36.1	57.5	53.4	36.1	37.6	55.1	67.1	52.7	65.3	59.9	69.8	86.1	87.5	90.6	91.7	83.8	84.6
Democratic Republic of the Congo	85 to 89	81.8	80.8	89.6	88.8	71.3	66.2	58.1	51.3	64.7	62.6	63.6	56.4	86.8	86.9	91.7	90.7	84.9	83.0	85.9	85.1	91.9	91.2	79.7	75.7
Denmark	85 to 89	85.3	87.6	89.5	91.4	82.1	83.0	74.2	76.9	79.5	82.8	78.8	78.0	84.7	80.8	84.4	80.8	85.7	82.0	85.7	88.2	89.7	91.5	85.3	87.1
Djibouti	85 to 89	86.7	87.8	93.4	94.5	74.5	75.7	55.1	58.1	65.6	66.8	58.5	62.2	88.0	89.5	93.8	94.5	83.4	86.3	91.6	90.7	95.6	95.5	85.1	83.2
Dominica	85 to 89	64.2	66.4	76.4	77.4	52.7	60.4	52.0	55.0	64.3	65.7	55.5	62.1	82.7	85.7	86.2	87.2	78.0	81.8	65.1	66.5	77.4	77.9	59.9	65.9
Dominican Republic	85 to 89	72.0	82.9	79.1	87.8	67.2	78.3	54.1	72.5	63.0	78.8	61.2	74.7	85.2	91.6	85.2	92.2	85.8	90.4	74.7	83.4	80.9	87.9	74.5	82.6
Ecuador	85 to 89	84.7	86.9	89.6	91.9	79.0	79.4	69.5	72.9	76.5	80.2	71.9	73.1	94.3	83.0	95.3	84.1	92.5	82.5	84.8	87.5	89.4	92.0	82.7	83.8
Egypt	85 to 89	0.0	42.9	0.0	17.7	18.9	47.8	0.0	47.9	0.0	39.8	31.9	62.0	40.1	59.2	43.8	54.0	45.6	67.7	0.0	42.8	0.0	15.0	29.9	53.0
El Salvador	85 to 89	87.6	89.2	92.0	93.0	81.6	83.8	75.5	78.8	80.5	83.1	78.3	81.3	91.8	91.5	92.8	92.0	90.3	90.3	88.5	89.3	92.5	92.8	85.4	86.9
Equatorial Guinea	85 to 89	82.2	89.0	89.6	94.4	69.9	79.0	51.8	67.8	62.4	73.6	57.2	72.9	86.2	92.8	90.5	95.4	83.0	90.0	87.4	91.2	92.6	95.2	80.2	84.7
Eritrea	85 to 89	81.2	81.6	88.4	89.4	69.3	66.1	49.9	44.0	62.2	58.9	53.7	46.1	85.6	87.1	90.5	91.6	79.0	81.2	87.3	87.1	91.8	92.2	83.0	79.0
Estonia	85 to 89	45.3	78.9	63.9	83.8	33.1	77.7	51.5	69.4	63.7	77.4	56.1	69.5	69.7	63.0	72.0	63.9	59.9	59.7	43.9	79.8	65.1	84.8	37.9	82.1
Eswatini	85 to 89	81.5	81.6	88.8	87.6	67.8	77.6	53.7	57.8	65.1	66.1	58.2	71.5	88.0	88.2	89.5	87.8	85.5	88.9	85.9	84.5	91.4	89.7	76.9	83.1

Ethiopia	85 to 89	83.2	85.9	90.1	92.8	72.3	75.8	56.0	64.2	67.3	74.0	57.6	65.0	86.8	88.1	93.1	94.6	82.3	85.6	90.6	90.5	93.8	94.6	88.0	86.0
Fiji	85 to 89	80.6	80.7	89.5	88.9	66.3	66.6	44.3	42.9	68.6	64.4	27.1	27.0	73.9	72.1	66.9	65.6	84.5	85.4	88.6	88.0	93.6	92.8	82.6	81.9
Finland	85 to 89	90.5	94.8	93.0	96.2	90.1	93.1	86.4	89.6	88.4	90.2	91.1	94.4	90.9	91.7	90.1	91.3	92.8	92.1	89.2	93.5	92.0	94.9	91.1	94.0
France	85 to 89	77.2	92.5	84.7	94.6	69.9	89.7	69.2	81.5	77.0	85.4	70.9	83.5	79.4	85.5	77.1	86.1	84.1	85.1	77.8	92.9	85.1	94.4	74.3	93.2
Gabon	85 to 89	87.5	88.3	93.4	93.9	75.9	76.6	55.8	59.3	66.6	68.1	59.4	64.8	90.5	92.4	93.8	95.1	86.3	88.5	91.9	90.8	95.4	94.7	85.7	83.8
Gambia	85 to 89	82.2	78.9	88.6	86.6	74.5	69.5	68.2	65.4	70.8	68.3	79.2	77.3	89.8	88.7	89.8	87.9	89.6	89.6	85.3	81.1	91.3	88.8	77.8	71.9
Georgia	85 to 89	66.2	62.4	80.1	78.6	38.3	30.6	51.7	56.4	65.6	68.9	48.3	54.6	75.9	61.6	74.7	59.9	78.2	65.4	71.5	63.7	84.0	80.5	49.2	34.4
Germany	85 to 89	84.2	97.7	89.7	98.6	77.6	95.2	78.6	87.1	83.8	89.4	80.0	89.3	84.7	86.4	83.8	87.4	87.0	85.5	83.8	96.6	89.2	97.2	80.4	96.7
Ghana	85 to 89	80.1	80.3	86.9	87.5	72.5	71.1	65.4	64.8	67.2	68.0	78.4	78.1	90.2	90.6	89.6	89.4	90.7	91.4	82.8	82.5	89.8	89.6	75.3	73.7
Greece	85 to 89	70.4	84.0	79.2	88.3	63.5	80.6	63.0	71.4	70.6	77.9	68.8	74.1	78.6	83.3	77.5	83.3	81.4	84.1	71.8	85.3	80.8	89.2	68.5	85.6
Greenland	85 to 89	81.2	85.7	87.1	90.0	77.4	81.3	72.8	76.5	78.6	81.1	78.1	79.8	56.4	74.8	49.9	72.1	70.9	79.7	82.4	86.9	88.1	90.7	81.3	85.5
Grenada	85 to 89	68.6	75.3	83.2	83.9	41.0	69.8	64.6	68.4	78.8	76.3	54.4	69.9	85.0	88.0	92.6	88.0	74.2	82.7	67.7	74.9	82.8	84.1	46.3	74.3
Guam	85 to 89	82.8	97.7	85.2	99.3	85.8	93.4	60.2	83.7	63.8	88.0	74.0	83.4	66.4	91.5	47.9	90.4	90.4	93.1	87.5	100.0	88.3	100.0	93.0	99.2
Guatemala	85 to 89	81.6	84.4	88.0	89.8	73.9	77.7	68.9	73.2	74.8	78.2	72.9	76.4	93.6	92.7	96.7	93.9	90.4	91.0	82.7	85.8	88.8	90.8	78.4	82.2
Guinea	85 to 89	80.7	80.0	86.6	86.5	74.6	73.2	68.9	68.8	68.3	68.5	78.9	79.5	89.0	89.4	88.6	88.3	89.5	90.3	83.5	82.1	89.7	89.0	78.1	75.6
Guinea-Bissau	85 to 89	77.6	74.2	85.2	83.2	69.3	64.3	61.7	56.2	64.1	59.8	74.9	73.2	86.2	85.1	85.9	83.2	86.9	87.5	81.1	77.3	88.7	86.3	72.7	66.7
Guyana	85 to 89	66.6	70.3	76.4	79.1	59.9	63.8	61.5	64.5	70.6	71.8	65.4	69.5	86.9	86.8	88.7	89.1	85.3	84.5	66.7	70.0	76.9	79.4	65.1	67.7
Haiti	85 to 89	53.0	58.4	66.4	69.2	46.3	50.5	49.4	52.4	61.5	63.4	54.0	56.5	71.5	73.9	73.8	75.3	71.4	75.7	52.3	58.1	65.9	69.0	54.2	57.0
Honduras	85 to 89	80.1	73.3	87.2	81.6	71.4	65.6	69.1	60.4	75.7	67.5	72.3	67.2	86.2	76.8	86.8	74.4	86.1	81.1	81.2	75.3	88.3	83.6	75.7	70.8
Hungary	85 to 89	69.8	83.3	80.2	88.8	59.2	77.3	44.7	69.3	58.7	77.1	48.8	70.0	79.6	79.4	79.5	79.5	80.0	79.2	72.4	83.7	82.3	89.1	66.1	81.2
Iceland	85 to 89	88.7	93.5	91.9	94.3	85.0	92.4	83.6	85.4	87.1	87.5	85.4	88.6	85.6	84.8	84.5	83.6	87.6	86.6	88.1	93.0	91.4	93.6	87.3	94.5
India	85 to 89	78.1	80.4	84.1	86.2	72.2	74.5	73.3	74.7	79.1	80.5	75.4	76.4	76.1	80.5	77.8	80.1	77.9	82.3	79.2	81.4	85.0	87.0	77.4	79.1
Indonesia	85 to 89	74.3	71.1	81.5	80.1	68.5	63.3	47.3	41.3	56.9	53.3	56.3	50.8	69.6	68.0	67.0	63.7	74.6	75.4	79.9	76.2	85.8	84.1	78.0	72.1
Iran (Islamic Republic of)	85 to 89	59.9	76.5	69.5	80.1	53.7	73.5	64.3	74.1	70.4	77.6	69.7	78.3	67.0	78.8	67.0	77.7	70.0	81.2	59.0	76.5	69.9	80.6	57.4	76.6
Iraq	85 to 89	65.1	61.4	76.3	73.9	54.1	49.3	63.7	59.7	73.0	69.6	65.2	62.1	80.5	79.0	84.6	81.6	76.3	76.9	64.8	61.5	76.7	74.8	58.7	54.3
Ireland	85 to 89	79.8	86.6	85.9	90.8	75.1	81.5	80.5	86.1	84.4	88.3	84.0	88.9	81.8	80.7	81.3	80.7	83.4	81.7	79.0	85.8	85.6	90.4	77.6	83.4
Israel	85 to 89	88.8	94.7	89.9	95.3	88.5	94.2	69.0	77.3	74.1	82.2	74.5	79.4	90.0	85.9	89.8	87.9	90.1	84.4	89.9	95.5	90.7	95.3	92.4	98.4
Italy	85 to 89	74.3	85.5	82.9	90.1	65.6	80.2	72.5	73.5	79.1	80.0	74.6	75.3	83.5	78.9	82.1	79.9	86.1	78.8	74.2	86.5	83.3	90.5	69.1	85.2
Jamaica	85 to 89	71.0	73.1	79.2	81.3	66.6	66.1	67.9	64.8	74.6	72.9	72.4	68.4	90.5	88.1	91.1	89.6	89.2	86.2	69.2	72.5	78.5	81.1	69.2	70.1
Japan	85 to 89	83.9	97.7	89.5	99.4	77.2	93.1	83.5	90.0	88.6	94.3	82.0	87.2	90.9	96.7	90.2	96.1	92.7	97.0	82.5	95.6	88.0	96.8	79.9	94.4
Jordan	85 to 89	68.4	83.6	71.7	83.2	67.8	85.5	61.7	64.6	67.0	68.2	69.9	73.3	87.8	88.6	89.9	89.9	86.5	87.7	69.0	84.2	72.8	84.2	71.9	88.1
Kazakhstan	85 to 89	72.3	70.6	82.8	81.9	54.4	49.9	65.5	57.0	73.8	68.4	68.6	57.0	66.4	53.7	64.8	48.6	69.4	63.5	73.3	73.3	84.0	84.0	58.1	57.0
Kenya	85 to 89	87.5	86.5	93.4	92.5	78.3	74.7	63.0	59.9	71.8	70.0	65.1	61.8	88.9	89.7	94.6	93.2	86.0	86.1	92.2	90.1	95.4	94.1	88.4	84.0
Kiribati	85 to 89	80.9	78.8	89.1	87.2	68.3	66.7	50.5	47.2	67.5	63.8	44.5	43.7	78.5	77.7	74.4	71.7	85.1	86.5	88.4	85.4	93.3	91.2	82.5	79.1
Kuwait	85 to 89	86.8	88.8	89.6	92.3	84.5	83.4	77.1	79.0	82.5	85.6	78.5	79.2	86.2	89.5	88.0	93.4	84.7	87.5	86.9	88.7	89.6	92.0	88.0	86.4
Kyrgyzstan	85 to 89	63.1	77.9	77.8	87.2	36.3	57.7	46.6	65.0	62.0	75.0	44.1	62.3	65.3	61.8	66.2	64.1	64.2	60.0	66.3	79.7	80.4	88.5	45.7	64.0
Lao People's Democratic Republic	85 to 89	65.1	70.9	76.0	80.1	57.8	62.1	42.2	45.1	55.7	56.9	49.2	51.8	62.8	72.3	60.5	70.3	67.5	76.6	70.4	75.6	79.9	83.9	68.4	70.8
Latvia	85 to 89	47.9	69.7	65.0	77.0	38.5	70.1	45.7	59.7	58.6	70.2	53.7	61.1	68.2	64.8	69.0	66.4	63.2	55.9	48.2	70.6	67.2	78.3	44.3	74.8
Lebanon	85 to 89	61.8	80.8	74.8	85.7	48.0	76.1	44.5	64.1	65.4	74.9	35.2	63.1	54.2	74.6	66.9	79.9	45.1	71.3	66.4	82.6	77.0	86.8	61.9	82.0
Lesotho	85 to 89	80.8	77.3	88.5	85.1	61.8	71.0	57.2	50.8	69.1	62.0	54.6	65.1	88.0	86.0	90.2	85.8	82.1	85.9	85.3	81.4	91.1	87.9	72.6	78.2
Liberia	85 to 89	79.6	78.3	85.8	85.1	73.2	70.9	68.4	67.7	66.9	65.9	78.8	78.4	88.8	88.7	88.3	88.1	89.3	89.3	81.9	79.9	88.8	87.7	76.1	73.1
Libya	85 to 89	71.3	71.0	80.0	79.6	62.7	62.7	64.0	65.3	72.5	71.2	66.6	71.0	79.6	79.0	83.4	79.7	77.7	79.6	72.3	71.4	80.9	80.7	68.1	66.7
Lithuania	85 to 89	65.5	74.1	75.2	81.7	62.1	70.1	66.6	65.8	73.1	74.1	73.1	68.6	72.1	65.3	73.0	66.3	70.5	59.9	65.1	74.8	76.1	82.8	65.5	74.3
Luxembourg	85 to 89	71.0	86.0	80.8	90.5	61.6	80.7	68.4	74.1	76.4	81.1	70.1	74.6	79.7	82.0	77.9	82.5	83.4	82.4	71.3	87.1	81.4	91.0	65.7	85.5
Madagascar	85 to 89	81.2	81.8	90.4	90.4	68.0	67.4	48.2	46.3	60.5	60.3	51.3	48.5	82.5	85.1	91.2	91.4	79.4	81.6	87.8	87.3	93.6	93.0	80.7	79.2
Malawi	85 to 89	82.6	81.0	89.4	88.6	73.4	68.9	53.1	47.0	61.1	59.0	60.1	53.7	85.6	86.4	89.8	89.3	83.7	83.6	87.4	85.1	92.4	91.0	82.5	77.6
Malaysia	85 to 89	84.8	85.3	89.1	88.1	80.2	84.0	69.2	64.6	75.0	69.7	72.8	71.5	77.7	76.4	77.4	73.0	79.3	82.0	88.2	89.0	91.3	90.4	87.3	91.6
Maldives	85 to 89	74.8	79.9	81.5	86.7	66.0	72.1	51.3	64.0	60.2	71.1	54.4	67.1	65.8	78.2	65.2	76.7	71.7	81.1	80.2	82.9	85.4	88.8	76.4	78.8

Mali	85 to 89	80.9	82.0	86.1	87.2	76.3	76.4	69.5	74.4	67.8	71.3	79.5	81.6	87.7	89.3	87.2	88.6	88.3	89.7	83.9	83.5	89.1	89.5	80.4	78.8
Malta	85 to 89	78.0	85.2	85.0	89.3	71.7	81.8	74.2	84.3	79.6	86.5	78.0	88.0	91.5	90.8	92.0	91.9	90.3	89.2	77.7	84.1	85.0	88.5	74.6	83.5
Marshall Islands	85 to 89	74.6	76.8	83.2	84.1	65.6	68.9	43.9	46.1	59.0	61.1	45.5	47.1	61.0	73.2	48.6	58.2	81.7	85.2	83.0	84.1	88.3	88.4	80.9	82.6
Mauritania	85 to 89	80.9	81.6	86.9	87.8	75.5	74.5	64.8	71.5	64.9	69.2	80.0	81.7	89.5	91.2	88.3	89.9	90.5	91.8	84.4	83.1	90.2	89.9	78.7	76.6
Mauritius	85 to 89	79.4	86.7	86.0	91.4	73.6	80.5	64.4	72.0	72.9	79.5	67.7	71.9	71.9	76.0	72.1	76.6	70.3	75.0	81.0	87.8	87.2	91.8	78.9	85.5
Mexico	85 to 89	80.2	90.3	84.7	93.3	78.2	86.4	70.4	79.4	73.4	83.7	79.1	81.6	90.0	87.1	88.0	86.7	91.7	88.0	80.5	90.4	85.2	93.1	80.9	89.4
Micronesia (Federated States of)	85 to 89	76.4	78.1	84.6	85.5	67.1	70.0	48.8	50.6	64.5	64.8	45.6	49.5	65.1	71.3	52.7	61.6	82.8	85.5	84.5	85.0	89.2	89.5	82.8	83.5
Monaco	85 to 89	79.0	86.0	85.7	90.0	73.0	82.2	74.6	82.4	81.1	86.1	75.9	84.4	79.1	86.3	77.4	85.1	82.7	88.0	79.1	85.4	85.8	89.4	76.9	84.7
Mongolia	85 to 89	65.5	77.7	80.4	86.5	35.1	63.4	31.4	60.8	53.4	73.1	23.3	54.4	33.6	41.8	36.6	34.9	34.9	55.4	87.3	89.1	93.2	94.1	76.9	82.3
Montenegro	85 to 89	68.5	50.9	79.3	68.2	57.1	34.1	43.4	19.6	60.5	43.4	38.9	15.6	75.3	65.5	74.3	60.0	78.2	74.4	82.4	69.9	88.5	81.0	78.6	62.0
Morocco	85 to 89	60.4	64.0	68.8	71.1	54.1	59.5	56.2	60.6	63.3	66.4	63.4	67.7	70.0	72.4	74.3	72.8	69.4	74.5	61.1	64.4	69.7	72.2	59.6	64.0
Mozambique	85 to 89	81.0	80.5	88.8	88.8	69.8	64.6	56.9	50.5	66.8	63.6	60.4	52.3	85.5	87.2	91.0	90.9	82.3	82.4	85.7	84.6	91.5	91.1	79.2	74.1
Myanmar	85 to 89	65.6	70.4	76.8	81.3	54.7	57.1	40.7	41.2	53.2	57.8	48.4	41.5	65.7	72.3	65.5	72.6	68.4	73.1	71.5	75.6	81.6	84.9	65.1	67.9
Namibia	85 to 89	82.3	82.3	89.3	89.4	70.1	69.4	58.0	56.8	67.1	66.8	66.0	63.3	90.2	89.9	91.4	90.7	88.4	88.4	85.7	85.1	91.6	91.1	76.3	75.5
Nauru	85 to 89	80.3	77.8	85.8	83.6	73.3	73.0	56.0	37.6	68.3	50.0	56.9	45.9	75.9	68.4	53.1	52.1	87.1	89.4	86.3	86.4	89.3	88.5	84.5	86.9
Nepal	85 to 89	71.1	74.0	80.0	83.2	62.5	63.5	66.4	69.4	74.3	77.0	68.6	70.8	71.1	76.5	73.8	78.1	72.9	77.5	72.4	74.9	80.9	84.2	68.6	68.2
Netherlands	85 to 89	88.8	88.2	92.3	92.1	85.3	83.4	79.4	79.0	84.2	84.2	81.0	80.0	83.3	82.3	82.8	83.7	84.5	81.2	88.5	88.5	91.8	91.9	87.9	87.2
New Zealand	85 to 89	85.3	89.4	89.8	92.2	81.6	86.6	79.3	75.8	84.5	82.6	79.7	75.7	82.0	79.5	82.0	80.1	82.0	79.8	85.0	89.7	89.4	92.0	84.6	90.0
Nicaragua	85 to 89	85.0	91.7	91.4	94.8	74.5	86.4	74.8	80.7	81.6	85.2	74.4	81.5	91.8	94.9	93.2	95.7	88.9	92.7	85.7	91.6	91.7	94.4	78.6	89.4
Niger	85 to 89	80.8	79.7	87.5	87.1	73.0	70.8	65.4	67.3	69.7	69.4	74.6	75.0	87.1	87.0	88.1	88.0	86.5	87.0	85.0	82.5	90.8	89.9	78.5	74.9
Nigeria	85 to 89	79.5	84.6	86.6	90.7	71.3	76.2	65.6	75.1	68.5	76.7	76.6	81.7	88.6	93.1	88.9	93.5	88.5	92.6	82.6	85.9	89.7	92.4	74.6	78.7
Niue	85 to 89	80.4	83.1	87.6	89.4	71.0	74.8	53.7	55.0	68.3	70.1	47.4	48.9	69.6	76.6	60.9	69.2	86.1	89.3	86.9	89.1	91.2	92.4	84.2	87.1
North Macedonia	85 to 89	67.7	55.3	79.6	69.2	53.4	46.9	52.0	41.2	65.6	54.6	51.0	48.7	59.8	48.9	59.9	39.4	63.8	64.9	69.7	57.1	81.7	71.8	60.2	53.3
Northern Mariana Islands	85 to 89	81.2	84.2	86.3	89.3	76.3	78.8	56.4	58.6	65.3	68.8	61.0	61.2	66.5	68.3	44.2	53.1	88.3	87.8	88.9	91.4	90.9	93.4	89.1	91.0
Norway	85 to 89	85.7	96.1	89.7	96.8	82.7	94.4	80.9	83.2	86.4	86.4	80.2	85.8	80.3	84.8	77.6	84.9	85.9	85.1	85.2	95.4	89.1	95.9	85.2	96.0
Oman	85 to 89	71.0	73.9	82.7	85.0	53.3	58.5	69.4	72.2	80.0	82.9	57.4	61.3	81.8	84.5	85.4	87.3	74.5	80.8	70.2	73.2	82.2	84.5	59.8	63.6
Pakistan	85 to 89	79.0	79.5	84.3	85.1	73.2	74.0	72.2	71.8	77.4	76.9	74.9	75.6	76.0	78.6	77.0	77.6	78.4	81.1	80.6	80.8	85.6	86.4	78.6	78.6
Palau	85 to 89	81.5	83.2	88.3	85.7	72.8	80.7	60.0	60.1	73.8	67.4	55.7	64.4	77.0	77.7	67.7	59.9	88.0	90.9	86.3	87.6	90.5	87.9	84.0	89.9
Palestine	85 to 89	40.4	61.7	61.6	73.0	18.0	52.4	59.6	68.9	68.8	73.9	62.8	73.5	80.8	85.6	84.4	87.9	77.2	82.7	31.1	58.5	58.1	72.5	12.1	52.7
Panama	85 to 89	85.5	88.3	90.9	93.1	78.2	81.1	76.5	80.8	81.8	86.4	78.3	80.4	90.4	88.0	90.6	89.4	90.0	86.7	85.4	87.9	90.8	92.3	81.2	84.2
Papua New Guinea	85 to 89	73.9	74.6	80.1	80.3	68.7	69.7	40.5	36.8	53.7	50.8	45.6	42.1	66.3	71.0	45.3	51.2	83.7	85.7	84.8	85.4	86.3	86.4	87.4	87.8
Paraguay	85 to 89	76.4	81.1	83.5	86.9	69.5	74.9	64.6	66.6	73.7	75.5	65.9	67.4	84.1	85.5	84.3	85.1	84.7	86.9	77.2	81.5	84.2	87.2	74.4	79.0
Peru	85 to 89	84.8	90.7	89.4	94.1	80.0	85.6	71.8	79.1	76.9	83.2	76.1	81.5	89.9	91.6	90.6	91.7	89.1	91.2	85.5	90.5	89.8	93.6	83.9	88.3
Philippines	85 to 89	69.2	81.0	75.8	86.9	65.6	76.5	35.7	58.1	40.5	66.6	52.2	65.7	73.0	81.6	67.7	80.8	79.0	83.2	75.0	84.4	80.4	89.1	75.3	83.8
Poland	85 to 89	56.5	81.8	71.8	87.5	40.8	76.8	53.6	65.0	65.9	74.5	54.8	64.6	79.2	77.4	79.4	77.0	78.7	78.3	56.8	82.6	73.2	88.1	46.2	81.2
Portugal	85 to 89	64.3	78.6	76.6	85.7	51.9	71.1	63.4	71.0	72.5	78.7	66.0	71.6	75.0	75.2	73.1	74.9	79.3	77.2	64.6	79.6	77.6	86.4	56.5	76.1
Puerto Rico	85 to 89	80.9	92.7	87.4	95.9	73.6	87.2	78.1	85.5	82.8	89.1	80.2	85.9	91.1	92.7	91.6	94.7	90.1	89.2	79.8	91.9	86.6	94.7	75.7	89.7
Qatar	85 to 89	65.1	78.1	73.1	84.0	56.6	71.3	35.4	69.0	55.8	78.3	31.3	67.0	31.2	69.8	63.5	79.9	0.0	61.7	71.3	79.6	76.8	84.7	70.3	77.7
Republic of Korea	85 to 89	78.8	89.9	85.7	93.3	73.2	85.7	67.7	86.6	77.1	91.3	64.5	83.2	72.8	87.9	70.8	87.4	79.6	89.2	81.8	89.2	87.5	92.1	80.6	88.8
Republic of Moldova	85 to 89	42.1	73.0	59.8	77.9	32.3	72.2	34.6	54.1	51.6	65.3	38.7	57.7	59.8	73.0	63.4	73.6	55.2	73.2	45.8	75.3	63.5	80.2	43.7	78.0
Romania	85 to 89	59.8	71.8	72.0	80.5	50.7	64.9	38.8	48.4	51.4	61.3	48.8	52.0	65.9	62.8	62.8	59.4	72.3	69.5	62.2	73.7	74.9	82.2	57.3	70.8
Russian Federation	85 to 89	27.4	62.0	54.8	74.9	0.0	52.6	50.6	60.3	63.8	70.0	51.3	64.1	68.7	60.8	67.6	56.8	67.4	68.0	20.7	62.1	54.2	76.4	0.0	57.1
Rwanda	85 to 89	77.7	83.1	86.6	90.6	63.9	68.1	42.9	53.9	59.1	68.5	42.4	49.4	83.0	87.3	89.6	92.4	77.6	82.1	84.7	87.9	90.2	92.9	79.6	80.4
Saint Kitts and Nevis	85 to 89	59.9	68.2	74.7	77.8	42.2	63.6	54.1	63.8	67.9	71.7	50.8	68.9	80.9	83.4	84.1	84.9	74.7	81.1	60.2	67.7	75.4	78.1	49.7	68.0
Saint Lucia	85 to 89	55.2	71.8	71.5	81.9	37.3	60.2	51.8	63.7	65.5	76.2	51.3	60.4	81.6	87.4	82.0	88.7	80.2	85.5	54.3	71.6	71.8	81.5	43.1	66.1
Saint Vincent and the Grenadines	85 to 89	63.7	69.3	76.6	79.1	49.9	59.7	46.8	60.6	62.4	70.7	46.2	62.5	86.6	87.4	90.2	89.9	81.5	85.1	65.6	69.4	77.9	79.3	59.5	65.3
Samoa	85 to 89	78.3	80.8	86.4	86.9	67.2	74.3	46.9	43.8	65.2	60.9	36.1	41.8	66.6	73.1	56.4	61.2	83.8	88.0	86.9	89.5	91.0	91.8	84.9	89.8
San Marino	85 to 89	84.5	93.3	89.1	94.1	79.9	93.1	77.6	87.1	81.6	88.1	82.7	92.0	87.6	94.2	87.2	93.9	88.3	93.8	84.4	92.3	89.1	93.4	82.6	94.0

Sao Tome and Principe	85 to 89	83.2	83.9	90.1	89.5	69.3	77.5	69.0	72.0	74.8	74.4	76.7	82.0	92.4	93.2	93.0	92.5	90.5	93.4	86.0	85.6	92.3	90.9	72.0	80.2
Saudi Arabia	85 to 89	59.1	71.0	71.3	74.9	47.6	67.0	57.5	59.3	69.1	70.8	58.2	59.6	81.5	83.4	84.4	86.5	79.4	80.1	59.2	72.5	71.4	75.4	53.9	73.2
Senegal	85 to 89	82.3	80.3	88.6	87.8	74.7	70.8	70.0	68.0	70.9	69.2	79.1	78.0	89.7	89.3	90.1	89.0	89.6	89.7	84.8	82.4	91.1	90.0	78.1	73.5
Serbia	85 to 89	54.2	69.3	70.3	80.4	37.1	55.4	47.0	60.0	61.8	72.2	46.2	54.6	54.4	69.6	50.6	68.6	63.1	71.9	55.3	70.1	72.2	81.6	44.0	61.0
Seychelles	85 to 89	81.2	83.6	88.2	88.9	71.6	78.3	61.2	64.9	70.2	72.1	65.1	71.2	79.3	83.0	79.1	82.3	79.4	84.4	83.8	85.6	89.9	90.3	77.7	83.4
Sierra Leone	85 to 89	80.1	80.3	86.8	86.8	72.7	73.4	67.8	68.7	68.8	69.1	78.5	79.9	89.0	89.8	88.8	89.1	89.4	90.3	82.5	82.1	89.4	88.9	75.7	75.8
Singapore	85 to 89	94.6	100.0	95.2	99.5	94.9	100.0	95.3	100.0	96.4	100.0	95.8	100.0	99.6	100.0	98.8	100.0	100.0	98.2	92.5	97.4	93.3	96.9	95.4	100.0
Slovakia	85 to 89	84.9	88.9	89.5	92.7	80.9	84.4	44.8	58.1	58.3	68.6	49.9	61.1	73.8	76.3	72.0	74.9	77.5	79.2	86.3	89.3	90.1	92.5	86.0	88.1
Slovenia	85 to 89	76.6	83.7	84.2	89.4	69.9	76.3	70.5	68.8	75.9	77.8	75.6	67.4	78.5	77.2	77.8	76.8	80.1	78.6	77.0	84.4	85.0	89.8	73.2	80.8
Solomon Islands	85 to 89	77.0	77.8	84.1	84.6	69.8	71.1	50.5	50.9	65.0	64.7	50.2	51.2	66.9	73.0	50.5	61.0	82.8	84.9	86.1	85.9	89.0	89.1	86.5	86.6
Somalia	85 to 89	78.4	79.2	87.7	87.9	64.6	63.2	40.9	41.1	57.7	57.8	41.4	40.8	80.5	84.2	89.5	90.4	76.0	78.2	86.7	85.9	91.6	91.4	82.2	79.2
South Sudan	85 to 89	91.1	87.5	92.1	92.4	73.8	74.9	71.4	64.4	66.7	66.8	57.8	57.3	94.8	91.8	92.9	93.2	84.3	84.7	92.8	89.0	94.7	94.3	85.6	85.2
Spain	85 to 89	85.0	85.2	84.8	93.4	67.8	92.5	55.7	55.6	78.2	82.9	73.1	77.4	87.2	86.8	85.1	81.4	85.9	77.5	90.6	89.9	85.2	93.7	71.8	97.4
Sri Lanka	85 to 89	76.7	92.6	76.8	83.4	54.7	60.4	71.1	76.9	50.7	59.9	44.8	50.1	85.3	79.2	80.0	84.7	82.0	84.2	77.1	93.9	79.8	85.3	62.8	67.6
Sudan	85 to 89	65.8	73.4	63.4	72.0	43.1	58.7	38.0	46.3	57.9	65.4	49.2	66.0	80.5	84.4	71.0	72.1	58.4	72.1	69.2	76.1	64.5	73.5	53.0	63.7
Suriname	85 to 89	52.2	65.4	84.0	83.7	73.1	72.2	45.0	59.5	74.9	73.9	72.9	69.2	61.3	69.9	90.3	90.1	86.8	86.7	54.8	66.4	84.8	84.4	77.9	77.7
Sweden	85 to 89	78.0	77.1	91.2	95.3	86.2	93.0	68.7	66.0	86.5	89.1	88.8	90.5	88.6	88.7	80.6	85.1	89.3	88.5	78.9	78.1	90.7	94.5	87.9	94.4
Switzerland	85 to 89	88.1	94.4	86.7	94.3	75.2	90.6	84.1	87.3	81.9	88.7	83.9	92.2	83.7	86.4	78.2	82.3	83.1	81.3	87.5	93.5	86.8	93.7	77.8	92.5
Syrian Arab Republic	85 to 89	80.7	92.6	64.8	67.8	51.0	54.2	78.3	87.8	60.5	64.3	59.5	59.8	79.7	81.5	75.2	77.2	72.8	77.8	80.5	92.0	65.5	68.6	57.8	60.5
Taiwan (Province of China)	85 to 89	56.5	61.2	91.8	97.1	86.3	92.4	52.4	55.4	72.3	83.0	66.5	75.9	72.1	75.8	78.5	80.5	81.7	80.0	57.6	62.3	94.4	97.8	94.9	98.9
Tajikistan	85 to 89	89.0	95.6	77.9	82.0	50.2	55.8	63.7	76.4	61.1	67.6	58.0	64.7	79.6	79.9	64.1	69.0	69.2	78.1	93.6	98.0	81.5	84.8	57.5	61.4
Thailand	85 to 89	65.5	70.8	87.9	91.6	73.2	82.6	50.8	59.9	70.7	76.5	63.8	72.1	64.8	72.7	53.9	69.5	59.4	75.1	69.3	73.7	91.7	93.9	84.5	91.4
Timor-Leste	85 to 89	81.3	87.5	77.6	76.9	62.3	57.7	61.3	69.5	52.6	51.0	52.1	45.3	54.8	71.1	63.1	64.2	71.7	73.6	87.5	91.7	82.9	82.0	73.2	68.4
Togo	85 to 89	69.0	67.0	87.0	86.2	73.6	68.9	42.4	38.1	68.3	67.3	78.6	76.3	65.8	67.4	88.7	87.8	89.2	88.8	75.7	73.2	89.8	88.7	76.8	71.5
Tokelau	85 to 89	80.6	78.5	84.5	88.5	65.1	73.4	67.1	63.3	65.5	70.7	43.4	50.2	88.9	88.2	54.3	70.3	83.8	88.8	83.2	81.1	88.4	91.4	79.9	86.6
Tonga	85 to 89	75.8	82.0	90.7	90.5	79.3	81.8	48.6	55.9	74.9	74.1	64.6	65.4	67.7	78.2	72.7	74.3	89.0	90.3	82.9	87.8	94.2	93.2	89.1	90.8
Trinidad and Tobago	85 to 89	85.7	86.2	78.5	86.8	57.2	75.8	65.3	64.8	72.8	81.0	62.6	76.4	78.9	80.4	85.8	88.8	84.1	85.6	91.4	90.9	78.4	86.4	61.5	78.9
Tropical Latin America	85 to 89	67.7	81.1	82.0	89.9	65.1	79.6	62.8	74.9	75.8	79.7	69.4	73.1	85.0	87.4	89.4	83.2	89.5	83.1	67.1	80.7	82.0	89.9	68.4	83.1
Tunisia	85 to 89	55.9	67.4	71.0	78.0	39.5	56.1	53.4	63.2	67.3	72.4	52.2	65.2	68.3	76.3	76.2	78.8	62.8	75.2	56.2	67.7	71.3	78.8	46.6	60.9
Turkey	85 to 89	71.0	77.5	79.0	83.8	65.2	72.5	58.3	66.9	67.3	74.1	63.7	70.9	62.1	68.8	68.9	72.7	55.9	66.9	73.5	79.0	81.0	84.8	72.2	78.1
Turkmenistan	85 to 89	75.6	76.9	84.9	86.5	61.3	59.6	65.3	60.4	73.9	71.8	66.9	59.2	73.1	63.5	73.5	64.8	73.7	64.3	77.2	79.3	86.3	88.0	66.5	67.2
Tuvalu	85 to 89	72.2	77.6	81.7	85.0	62.6	69.9	39.9	47.2	57.5	61.8	37.7	48.0	58.7	71.5	45.3	59.8	81.8	86.3	80.9	84.2	86.9	88.9	78.9	82.3
Uganda	85 to 89	85.7	89.7	93.2	95.2	71.7	76.3	51.2	58.2	68.0	71.4	47.3	55.0	84.4	89.5	92.3	94.1	79.5	84.5	91.5	93.3	95.5	96.5	85.3	86.6
Ukraine	85 to 89	56.1	69.5	69.3	80.4	51.9	57.0	63.7	65.9	73.1	75.4	64.0	63.7	38.7	55.9	33.2	53.9	46.2	59.1	56.0	70.1	70.6	81.4	56.9	61.9
United Arab Emirates	85 to 89	77.3	81.0	83.4	63.2	70.4	77.8	71.9	71.3	79.4	54.3	72.1	76.3	64.6	63.6	69.5	26.8	64.7	74.1	78.2	82.1	84.2	66.3	75.1	81.6
United Kingdom	85 to 89	81.8	90.8	88.0	93.6	75.3	87.3	79.1	78.3	83.5	82.8	82.7	80.9	82.0	79.3	80.7	79.0	85.2	80.8	81.5	91.5	87.8	93.8	77.9	90.8
United Republic of Tanzania	85 to 89	83.9	86.5	91.6	92.7	71.2	75.7	52.9	59.5	68.4	69.8	50.3	60.9	86.5	89.7	94.0	93.7	81.6	86.4	89.8	89.5	94.2	94.2	84.4	83.1
United States Virgin Islands	85 to 89	73.4	85.4	82.7	91.4	62.8	74.9	62.5	76.3	72.9	84.3	62.8	71.9	84.9	90.8	86.7	93.1	82.1	86.0	73.5	85.3	82.8	91.0	68.6	79.4
United States of America	85 to 89	92.6	97.7	95.2	98.4	89.0	95.9	85.2	90.5	88.5	91.8	86.6	93.1	85.4	81.4	85.2	82.3	85.7	81.0	91.8	97.0	94.1	97.0	91.1	98.4
Uruguay	85 to 89	79.9	84.6	86.5	89.8	73.0	78.7	79.6	82.6	86.0	88.1	76.9	79.4	73.8	77.4	65.9	75.8	82.4	80.7	79.6	84.4	86.3	89.4	76.5	82.2
Uzbekistan	85 to 89	74.6	78.8	84.1	87.3	61.6	62.9	57.7	72.7	67.7	79.9	61.6	71.5	80.4	67.2	84.0	69.1	76.8	66.9	79.0	80.0	87.7	88.6	71.0	67.3
Vanuatu	85 to 89	77.9	79.8	86.0	86.9	68.2	71.6	47.5	49.1	65.1	64.5	44.9	47.6	70.8	74.5	54.7	63.6	84.0	86.2	85.3	86.6	90.1	90.8	82.0	84.1
Venezuela (Bolivarian Republic of)	85 to 89	85.7	84.3	89.8	89.9	81.9	77.0	71.5	71.9	76.2	79.0	77.4	73.2	93.5	87.5	95.0	88.5	91.0	85.9	86.8	84.8	90.5	89.9	85.8	81.4
Viet Nam	85 to 89	64.1	62.5	77.3	76.0	48.4	47.5	39.7	38.7	55.2	54.5	43.6	42.4	65.6	71.5	65.2	70.0	66.2	74.4	70.4	67.2	81.8	79.7	60.6	57.5
Yemen	85 to 89	45.4	48.1	63.8	64.1	31.0	32.7	41.9	45.7	58.4	57.8	41.1	51.0	62.5	61.2	70.5	67.9	48.2	59.6	46.5	48.4	64.8	65.3	43.1	38.9
Zambia	85 to 89	80.9	81.6	89.6	90.1	68.2	67.1	45.4	45.2	58.4	59.0	48.4	47.9	81.8	84.6	89.4	89.8	79.4	82.0	88.4	86.9	93.1	92.4	83.4	79.5
Zimbabwe	85 to 89	83.4	78.9	90.4	86.8	69.7	67.5	49.0	36.6	60.9	48.5	56.5	51.1	82.7	80.9	84.8	81.2	81.2	82.1	86.6	82.7	92.1	89.3	77.2	75.0
Afghanistan	95 plus	46.2	58.7	75.2	77.6	47.4	64.4	37.5	58.3	68.7	73.4	48.2	68.2	34.8	56.5	70.5	73.3	30.4	61.9	47.2	57.7	76.7	79.3	48.1	60.6

Albania	95 plus	69.6	55.0	87.6	79.8	46.9	55.9	51.7	24.7	76.0	58.5	35.8	46.2	66.6	60.0	79.6	71.6	52.4	68.6	75.0	65.7	91.3	86.8	55.1	61.9
Algeria	95 plus	45.6	52.5	0.0	72.2	59.6	57.7	43.0	53.9	0.0	67.7	64.0	63.6	43.1	51.3	20.4	57.5	60.2	62.1	47.5	54.2	0.0	76.0	57.0	55.8
American Samoa	95 plus	78.1	79.5	90.9	91.0	78.5	82.5	49.2	49.9	75.9	76.1	48.5	52.9	61.5	67.7	69.0	72.8	77.0	79.3	82.9	83.8	93.6	93.5	83.6	86.7
Andorra	95 plus	78.9	85.3	89.8	92.8	88.1	90.5	81.6	86.5	88.8	90.9	87.3	89.0	72.3	77.4	80.0	83.1	82.6	85.0	77.1	82.4	90.0	92.8	83.0	85.2
Angola	95 plus	86.4	89.0	95.8	96.7	82.5	84.3	64.6	65.3	80.3	80.1	70.4	73.0	87.5	90.8	97.0	97.6	81.7	85.2	87.7	88.7	97.0	97.1	81.7	82.2
Antigua and Barbuda	95 plus	63.0	69.5	83.1	85.8	69.5	77.5	59.1	64.8	77.6	80.3	68.8	73.0	80.5	81.1	89.2	88.7	75.7	78.1	61.7	68.4	84.2	87.1	65.9	74.5
Argentina	95 plus	74.1	86.6	88.4	93.7	79.5	90.6	73.7	81.8	86.6	89.7	73.0	81.8	58.2	74.4	56.6	78.7	79.3	83.2	73.4	84.6	89.2	94.0	76.7	86.7
Armenia	95 plus	68.8	72.1	86.2	83.9	69.9	84.3	65.4	72.7	80.5	84.0	70.8	77.4	62.0	62.7	74.7	72.7	67.3	72.7	69.0	71.6	88.2	85.3	67.0	80.9
Australia	95 plus	70.0	82.7	85.4	90.9	82.8	91.1	67.8	71.7	81.8	83.5	76.5	78.4	68.8	72.4	79.3	82.3	73.1	74.1	70.5	81.4	87.0	91.6	79.5	87.1
Austria	95 plus	72.5	99.0	87.1	100.0	81.9	100.0	96.5	91.3	96.5	94.0	100.0	93.7	89.2	86.4	92.5	91.1	93.1	88.9	66.2	93.4	85.7	98.2	73.0	93.3
Azerbaijan	95 plus	52.6	75.0	79.4	88.9	29.1	73.1	21.6	69.8	58.2	83.0	35.3	71.8	74.8	82.5	83.5	89.0	70.3	81.2	60.0	74.1	84.4	90.4	25.6	68.5
Bahamas	95 plus	74.8	79.1	88.1	90.4	82.9	83.1	68.9	75.0	81.8	86.9	78.7	75.3	87.9	89.4	93.5	95.0	83.9	85.2	73.6	77.0	89.1	90.7	79.1	79.9
Bahrain	95 plus	34.3	48.7	69.1	76.1	50.4	55.7	15.5	42.0	53.7	69.5	52.3	55.5	32.9	54.0	52.5	69.2	50.1	60.4	39.3	51.4	74.9	79.6	49.1	55.4
Bangladesh	95 plus	70.3	61.6	85.5	85.0	74.5	57.6	67.5	58.9	81.0	80.8	72.6	60.7	59.4	58.6	72.9	76.5	66.3	61.7	69.4	61.5	86.9	86.6	71.4	54.9
Barbados	95 plus	58.2	67.6	80.6	84.0	68.9	77.0	62.7	68.1	79.3	81.8	72.7	74.7	77.5	77.4	86.4	87.9	75.4	75.7	57.2	66.6	82.2	85.2	64.7	73.6
Belarus	95 plus	54.2	59.5	77.9	81.3	64.6	67.2	65.7	63.7	81.6	80.6	67.8	64.0	53.6	51.8	69.3	67.6	54.5	48.8	55.5	60.9	80.9	84.2	63.5	66.4
Belgium	95 plus	65.4	84.1	84.3	92.4	72.5	90.0	65.2	72.3	80.8	84.5	73.3	76.5	66.1	67.4	76.1	77.8	76.1	73.2	66.4	83.0	86.2	93.1	69.8	86.6
Belize	95 plus	79.2	76.3	90.7	88.2	81.6	82.5	75.9	69.7	86.2	81.1	78.8	77.6	89.1	85.1	95.1	90.0	86.0	87.1	76.7	74.8	91.1	89.2	77.0	78.5
Benin	95 plus	76.7	77.0	89.4	89.7	81.7	81.2	67.5	68.2	78.0	78.3	81.9	82.1	82.6	84.5	89.1	90.2	84.0	86.0	77.1	76.9	91.3	91.2	76.1	75.5
Bermuda	95 plus	75.4	84.7	89.2	93.5	77.2	86.0	81.8	87.6	89.8	93.1	81.5	84.8	89.3	89.7	94.3	95.4	81.1	82.1	72.3	81.2	89.2	93.0	72.3	81.2
Bhutan	95 plus	73.8	78.5	88.5	90.6	75.6	81.0	71.2	76.2	84.6	86.6	73.3	78.6	70.0	75.0	82.3	84.1	70.1	77.8	72.8	77.2	89.5	91.4	72.6	77.3
Bolivia (Plurinational State of)	95 plus	72.7	82.0	87.6	91.3	76.5	86.5	64.0	77.2	79.0	85.3	72.9	81.4	77.5	82.8	85.4	87.9	80.4	86.1	71.7	79.9	88.8	91.9	72.4	82.1
Bosnia and Herzegovina	95 plus	60.8	73.5	81.9	87.7	69.9	77.8	61.7	76.0	79.2	86.5	67.3	77.0	62.4	75.5	74.4	83.8	65.8	77.7	62.1	72.9	84.6	89.2	68.4	74.7
Botswana	95 plus	82.3	91.6	93.1	96.8	78.3	89.2	62.5	79.6	79.3	87.9	71.4	84.6	88.3	94.3	94.2	97.1	85.8	90.7	83.3	89.5	94.6	96.9	76.0	84.7
Brazil	95 plus	70.3	84.1	86.2	92.3	75.6	88.7	71.7	77.9	84.1	87.0	76.2	81.0	86.9	81.0	91.5	86.9	88.6	84.8	68.8	81.8	87.1	92.6	71.5	84.3
Brunei Darussalam	95 plus	66.9	67.0	85.5	85.0	88.1	77.9	62.6	58.1	81.3	79.4	48.8	54.9	73.4	77.3	82.6	85.2	81.9	81.6	67.4	67.7	86.4	86.2	86.4	77.2
Bulgaria	95 plus	56.7	43.3	78.6	73.8	64.9	49.4	51.5	42.8	73.1	69.1	61.8	54.2	62.7	47.4	72.2	62.2	71.4	59.2	58.6	47.6	81.6	78.4	64.2	50.1
Burkina Faso	95 plus	78.2	77.6	91.2	90.7	77.4	78.0	70.4	68.4	81.9	80.2	77.3	77.6	84.1	85.1	93.2	92.3	80.4	83.6	78.3	77.7	93.1	92.4	72.1	72.8
Burundi	95 plus	82.3	82.7	93.0	93.5	80.7	80.5	60.0	59.1	79.4	78.5	63.5	64.7	83.9	83.8	94.7	94.6	74.6	77.8	83.9	83.7	94.3	94.6	82.0	80.7
Cabo Verde	95 plus	85.9	82.9	94.8	93.8	84.5	80.0	79.8	79.0	87.0	87.1	84.7	82.8	92.7	94.3	97.6	98.5	88.1	90.2	85.3	81.3	95.9	94.3	78.8	74.1
Cambodia	95 plus	60.1	55.0	82.3	80.1	59.8	54.3	34.7	25.9	64.2	60.3	49.4	40.8	55.9	58.5	69.7	70.8	62.5	67.5	65.7	61.2	86.9	85.0	62.8	58.2
Cameroon	95 plus	76.6	76.7	90.0	89.7	78.3	79.8	68.4	68.7	79.9	79.5	77.7	80.1	83.4	85.9	91.3	91.6	82.4	86.2	76.6	76.4	91.9	91.2	73.1	74.3
Canada	95 plus	82.3	88.5	91.7	93.9	89.2	97.0	91.1	87.3	94.3	92.2	90.8	89.9	77.2	73.1	85.4	82.9	78.6	74.2	79.5	85.4	91.6	93.4	84.0	91.5
Central African Republic	95 plus	81.6	81.2	92.6	92.2	79.2	81.1	59.1	60.2	77.4	76.3	67.3	71.4	86.1	86.8	94.2	94.5	78.9	82.5	83.3	82.3	94.2	93.7	79.2	79.7
Chad	95 plus	78.2	76.2	90.6	90.0	79.5	77.5	71.7	70.2	81.9	79.5	80.1	79.1	85.2	84.2	92.2	91.1	83.7	84.7	77.5	75.3	92.1	91.5	73.6	71.6
Chile	95 plus	72.1	78.0	82.7	86.9	84.7	88.9	80.4	85.3	87.2	89.6	85.1	90.0	72.5	64.6	57.4	59.8	85.9	82.7	69.9	75.7	83.0	87.3	79.7	83.5
China	95 plus	62.5	76.5	83.3	90.9	62.8	58.4	7.2	25.8	50.8	64.6	17.9	0.0	0.0	49.8	27.7	67.1	23.1	46.2	73.0	81.2	89.4	93.7	72.9	66.5
Colombia	95 plus	88.2	94.4	94.3	97.8	90.4	94.5	82.6	91.3	88.8	94.4	83.9	88.5	92.4	89.1	96.4	93.6	91.6	88.6	85.0	89.8	94.1	96.7	85.5	89.2
Comoros	95 plus	86.8	88.5	95.4	96.5	84.6	86.2	60.1	65.0	77.2	79.1	69.3	72.8	86.7	87.2	96.0	96.5	80.3	83.3	88.2	88.3	96.6	96.9	84.5	84.5
Congo	95 plus	84.0	83.7	94.0	93.9	81.5	82.9	58.9	62.4	76.4	76.7	69.8	72.8	87.3	87.9	95.3	96.2	81.7	85.0	85.6	84.0	95.6	94.9	80.5	80.7
Cook Islands	95 plus	78.9	88.8	91.9	95.8	78.4	89.0	45.1	66.5	76.8	85.3	41.4	60.9	65.4	77.8	73.4	83.8	75.9	85.0	84.2	91.0	94.4	97.0	83.8	91.3
Costa Rica	95 plus	87.0	91.9	94.1	96.7	89.2	92.7	84.9	93.5	90.3	95.8	86.1	89.7	90.8	91.5	95.7	96.2	90.0	89.1	83.5	87.1	93.7	95.4	84.0	87.0
Coted'Ivoire	95 plus	79.2	78.4	91.5	91.1	79.5	78.8	72.8	70.8	82.8	81.4	80.8	80.9	86.0	87.2	92.3	92.6	85.2	87.1	78.5	77.9	92.8	92.4	73.9	73.2
Croatia	95 plus	46.8	68.2	77.2	84.7	44.5	77.7	44.1	53.4	73.2	75.3	41.7	60.3	39.5	40.2	58.2	56.4	49.1	56.8	51.4	70.0	81.0	87.3	47.3	76.0
Cuba	95 plus	67.2	75.0	82.4	88.2	76.1	79.9	67.6	75.9	79.0	85.8	76.4	79.8	85.7	89.7	91.0	94.9	87.6	88.2	65.1	72.5	83.2	88.5	71.3	75.2
Cyprus	95 plus	0.0	50.2	31.7	77.4	42.8	62.0	21.0	56.5	45.7	76.2	56.6	73.8	6.7	44.8	0.0	59.5	51.9	71.5	0.0	50.9	34.5	80.0	37.8	53.1
Czechia	95 plus	55.2	82.3	79.9	91.7	59.4	87.7	64.8	80.6	81.3	89.0	68.6	80.5	59.0	68.2	70.9	77.5	68.9	75.6	57.6	80.6	83.0	92.3	58.4	83.6
Democratic People's Republic of Korea	95 plus	75.2	74.6	89.2	88.9	75.9	76.6	40.4	23.0	69.0	59.1	40.7	33.9	49.3	57.0	65.2	70.4	55.7	63.3	81.0	81.2	92.9	93.0	81.6	81.4

Democratic Republic of the Congo	95 plus	81.6	79.8	93.4	91.9	79.2	77.2	62.1	55.3	78.1	74.5	69.5	66.9	85.1	86.4	95.8	94.9	80.2	80.1	82.8	81.2	94.8	93.4	78.1	76.4
Denmark	95 plus	74.5	79.3	87.9	90.5	82.4	85.0	70.2	75.9	82.6	86.6	78.6	79.6	75.7	70.1	83.1	79.3	82.4	77.5	74.0	78.1	89.1	91.2	78.7	81.3
Djibouti	95 plus	89.9	91.5	97.3	97.9	85.7	87.4	63.3	67.2	79.3	80.6	70.5	74.8	86.9	89.3	96.4	96.9	79.7	84.3	91.0	90.4	98.1	97.9	85.6	85.1
Dominica	95 plus	52.9	61.9	78.5	82.1	60.5	72.6	51.5	62.1	74.2	78.7	63.6	74.2	78.2	84.8	88.8	92.0	71.3	80.2	50.9	59.7	79.5	83.0	56.0	67.8
Dominican Republic	95 plus	68.7	74.8	84.3	87.9	76.8	80.4	58.8	71.2	74.8	83.6	71.5	76.3	81.8	88.3	88.8	92.9	83.9	88.8	68.5	72.8	85.8	88.3	73.6	76.5
Ecuador	95 plus	72.2	76.4	84.6	89.3	81.1	80.5	59.1	62.3	72.2	79.1	72.9	70.7	86.5	72.0	89.9	81.0	89.5	78.2	71.4	76.1	86.0	90.5	77.2	77.7
Egypt	95 plus	30.7	67.3	69.6	73.5	31.6	69.9	16.0	73.4	64.9	78.9	35.4	75.9	21.9	63.2	74.0	75.3	34.4	69.8	36.3	66.3	72.9	75.1	32.9	65.9
El Salvador	95 plus	89.5	90.7	95.2	96.2	90.5	91.1	82.5	86.4	88.9	91.9	83.6	84.9	91.4	91.2	95.3	95.1	90.4	89.5	86.3	87.0	95.1	95.6	86.1	86.3
Equatorial Guinea	95 plus	84.4	93.7	94.0	99.0	81.6	89.2	61.7	75.5	78.9	85.4	68.0	79.5	87.5	93.2	95.7	98.8	80.5	88.1	85.8	92.7	95.5	99.0	81.4	86.4
Eritrea	95 plus	82.4	82.6	92.7	92.9	81.7	81.0	62.3	53.0	79.1	73.7	68.4	65.3	84.6	86.7	94.2	94.3	77.0	79.4	84.0	84.1	94.4	94.5	82.5	81.3
Estonia	95 plus	36.1	61.2	70.1	80.9	50.1	72.2	54.9	66.0	75.4	82.4	63.6	63.4	55.8	38.3	71.0	59.3	52.0	34.6	38.5	62.3	74.5	83.3	49.8	71.2
Eswatini	95 plus	79.7	87.8	91.4	94.7	76.1	87.9	59.3	81.6	77.9	89.0	67.7	82.6	87.1	90.7	92.7	94.7	83.5	88.9	81.4	85.8	93.3	95.2	74.7	83.8
Ethiopia	95 plus	86.5	88.6	94.6	96.3	85.1	87.4	67.7	73.1	82.7	84.3	69.9	75.9	87.6	87.3	96.3	96.9	79.5	84.4	88.7	89.0	96.4	97.1	87.4	86.4
Fiji	95 plus	83.4	79.6	92.9	91.2	81.2	82.6	59.5	46.4	78.4	71.7	51.9	55.8	66.8	59.7	77.1	70.9	76.8	80.1	86.8	84.1	95.5	94.1	84.3	84.1
Finland	95 plus	81.2	90.2	91.0	95.2	91.6	95.8	87.5	90.0	92.2	92.5	91.0	95.5	81.8	82.8	86.9	88.1	93.0	89.7	78.7	86.2	91.0	94.6	85.6	89.3
France	95 plus	66.1	80.4	85.0	91.1	70.1	86.1	63.4	71.6	80.2	84.2	70.7	77.3	64.2	70.8	74.5	80.6	78.0	75.5	67.0	79.7	86.7	91.8	67.4	82.9
Gabon	95 plus	93.1	92.6	98.2	98.4	87.5	87.3	66.1	69.8	81.9	83.3	69.6	74.7	94.7	94.8	99.1	100.0	84.4	87.6	94.0	91.4	99.1	98.2	87.2	85.1
Gambia	95 plus	79.1	74.7	91.2	89.0	80.3	77.4	72.0	68.0	81.8	79.4	81.4	80.2	86.4	85.6	92.6	91.2	85.3	86.4	78.7	74.1	92.6	90.3	74.5	71.4
Georgia	95 plus	56.0	69.1	81.3	86.5	13.9	59.7	40.3	66.5	68.9	81.7	26.4	65.2	62.7	63.1	74.9	75.4	58.8	56.7	59.8	68.7	85.1	88.2	12.1	56.1
Germany	95 plus	75.9	92.0	89.7	96.8	76.9	93.9	78.1	81.5	88.1	89.6	78.8	83.9	74.2	74.7	82.6	83.8	80.9	76.9	75.0	88.5	90.4	96.0	73.6	89.0
Ghana	95 plus	74.2	74.2	89.0	89.2	75.7	74.3	68.0	66.1	79.0	78.9	79.9	79.2	86.3	87.2	92.6	92.4	86.0	87.3	73.5	74.0	90.6	90.6	69.3	68.6
Greece	95 plus	59.7	73.1	80.3	81.5	67.7	84.7	53.3	59.7	72.8	67.4	68.0	75.2	67.6	75.7	74.8	76.0	79.4	83.8	61.0	74.0	83.4	84.9	65.3	81.7
Greenland	95 plus	77.7	74.2	90.3	87.9	79.0	81.1	75.4	70.9	86.1	82.8	79.5	78.6	46.2	60.3	60.2	69.5	66.4	75.2	77.2	74.1	91.5	89.4	75.6	77.7
Grenada	95 plus	66.7	71.8	85.5	87.4	66.4	88.2	69.7	67.8	83.3	82.3	73.5	82.2	84.3	82.8	92.3	88.8	77.1	85.6	64.5	70.9	86.4	88.7	60.9	84.1
Guam	95 plus	88.0	97.2	93.2	99.6	90.4	95.2	68.9	85.0	81.1	91.8	73.3	81.8	76.9	86.4	69.2	91.0	84.4	88.9	91.2	96.6	95.1	100.0	91.1	93.7
Guatemala	95 plus	65.7	73.6	83.3	87.8	73.8	79.1	55.8	56.7	72.8	74.1	71.5	72.0	86.9	85.6	92.9	91.2	85.5	85.7	64.7	74.4	84.6	89.8	69.6	76.5
Guinea	95 plus	77.6	75.8	90.1	89.0	80.4	79.8	73.0	70.3	81.0	78.9	80.9	81.1	85.3	86.0	92.3	91.5	85.0	86.8	76.4	74.8	91.6	90.5	74.4	73.7
Guinea-Bissau	95 plus	75.2	72.9	89.3	87.9	77.1	76.6	67.1	64.6	79.0	76.9	78.6	79.4	83.5	84.3	90.8	90.0	82.9	86.0	74.9	72.6	91.0	89.6	71.1	70.2
Guyana	95 plus	64.4	61.6	78.8	77.6	76.0	73.8	64.3	62.4	74.7	73.4	76.9	75.3	83.8	81.7	89.2	89.5	85.6	82.4	61.5	59.6	79.7	79.0	70.4	68.9
Haiti	95 plus	49.9	58.5	76.5	78.6	58.6	66.9	52.6	59.2	74.2	75.6	63.8	69.4	67.0	71.1	81.0	82.4	65.3	74.4	44.4	55.1	76.4	79.2	52.0	61.1
Honduras	95 plus	74.6	73.7	89.2	87.6	72.0	77.9	66.6	66.3	82.2	79.8	68.8	74.2	80.6	75.6	87.9	83.1	79.2	80.4	73.9	73.1	90.2	89.2	68.9	74.3
Hungary	95 plus	58.8	79.6	81.5	90.8	63.4	83.5	53.7	78.1	75.1	87.5	61.9	79.3	66.8	76.3	77.3	84.0	71.9	79.5	61.3	78.3	84.4	91.6	62.7	79.8
Iceland	95 plus	85.5	90.7	92.7	95.6	91.4	94.6	88.6	89.8	92.4	93.6	89.3	89.2	80.1	79.8	85.4	86.3	86.4	83.8	82.4	87.0	92.6	95.0	86.0	89.2
India	95 plus	72.5	77.1	86.6	89.2	76.9	81.8	69.5	73.2	82.3	85.0	73.7	76.3	67.0	73.5	79.8	82.1	70.9	77.6	71.7	76.3	88.0	90.3	74.0	78.8
Indonesia	95 plus	67.8	62.0	84.6	82.5	74.4	67.7	34.8	24.8	61.3	57.2	56.6	49.3	59.4	56.8	70.5	67.8	69.1	70.3	72.5	67.4	88.7	86.9	75.4	69.1
Iran (Islamic Republic of)	95 plus	56.8	75.5	75.4	82.1	70.4	84.5	66.2	77.8	77.3	82.0	76.3	82.4	54.4	72.4	63.6	76.5	69.5	79.5	56.9	74.5	78.5	84.4	66.7	80.3
Iraq	95 plus	63.4	51.9	83.2	77.8	66.2	56.4	66.3	55.7	81.4	76.0	71.0	64.2	75.1	72.1	85.6	82.2	74.1	72.7	61.6	52.2	84.5	80.2	61.7	53.9
Ireland	95 plus	68.9	77.2	85.7	89.4	76.3	83.1	79.4	86.3	87.8	91.6	83.9	87.6	67.6	68.7	77.6	78.1	76.7	77.5	67.7	74.6	86.7	89.8	71.5	77.5
Israel	95 plus	82.5	89.1	89.4	93.5	90.7	95.4	64.1	71.8	77.9	83.2	74.1	78.3	81.6	78.5	87.0	85.9	85.2	81.4	81.9	87.4	90.8	94.0	87.0	91.5
Italy	95 plus	61.6	75.7	82.5	89.1	68.3	81.4	64.7	60.8	80.3	79.1	73.3	69.1	70.1	60.9	78.5	73.2	79.0	69.1	63.0	76.3	84.9	90.4	65.7	79.4
Jamaica	95 plus	70.5	72.4	85.8	86.9	77.9	79.0	72.1	71.0	84.3	83.3	77.2	77.5	89.2	87.7	94.2	92.5	88.2	88.0	67.8	70.0	86.3	87.4	73.0	74.2
Japan	95 plus	80.1	94.3	91.2	97.9	84.3	96.4	83.2	81.7	91.4	90.6	80.5	82.9	92.3	92.3	95.4	94.8	92.1	95.0	77.1	89.7	90.6	96.1	80.0	90.6
Jordan	95 plus	65.2	86.7	79.6	81.8	76.7	92.1	63.8	72.6	76.6	74.0	75.5	79.3	83.3	84.6	89.9	90.3	83.5	85.1	65.5	84.6	81.9	84.6	73.6	87.3
Kazakhstan	95 plus	63.3	52.9	83.5	77.9	65.0	63.7	66.4	44.4	80.6	67.4	73.6	69.2	53.5	35.0	66.1	49.8	66.7	62.6	62.0	55.8	85.0	81.7	59.6	60.0
Kenya	95 plus	89.4	90.3	97.0	96.6	86.8	87.1	67.7	68.3	82.5	82.2	71.6	73.9	86.9	90.6	97.4	96.4	81.9	84.8	90.8	89.9	98.0	97.2	86.9	85.8
Kiribati	95 plus	81.8	75.5	94.1	91.2	73.7	67.0	49.3	39.3	79.3	74.1	36.6	28.7	72.8	69.4	83.1	79.6	73.4	73.3	87.3	80.9	96.8	94.0	80.3	73.7
Kuwait	95 plus	84.4	86.5	92.1	93.1	89.7	89.5	79.9	82.0	88.6	89.9	80.0	81.4	82.8	86.5	89.5	92.8	82.6	85.2	82.4	84.0	92.6	93.4	85.8	85.3
Kyrgyzstan	95 plus	34.8	65.1	73.1	85.6	3.6	41.9	28.8	65.7	66.1	82.4	19.6	55.9	44.7	37.2	66.2	58.4	33.8	34.3	39.0	65.4	77.1	87.3	5.3	39.5

Lao People's Democratic Republic	95 plus	61.2	61.7	82.8	83.1	61.8	63.4	43.1	37.9	69.6	65.8	51.9	52.9	57.7	64.3	71.3	74.8	62.5	72.0	64.6	65.9	86.2	86.9	63.6	64.3
Latvia	95 plus	37.9	47.4	70.6	73.6	48.4	72.7	48.6	52.7	71.2	74.1	62.8	64.2	57.2	46.1	72.1	63.5	57.7	48.9	40.3	51.3	75.0	78.2	47.4	71.4
Lebanon	95 plus	60.1	71.1	82.4	86.5	60.7	76.2	50.0	64.2	76.9	81.8	49.9	68.4	48.3	67.2	71.6	80.1	40.0	67.5	62.2	71.5	84.6	88.2	63.4	74.6
Lesotho	95 plus	78.5	86.1	90.9	93.9	72.8	86.5	58.0	79.6	77.5	88.0	64.7	81.5	87.9	90.5	93.3	94.7	81.5	87.8	80.5	84.6	92.9	94.7	71.8	82.5
Liberia	95 plus	76.1	72.6	89.5	87.8	79.1	76.2	71.1	69.2	79.9	76.9	80.8	79.6	84.5	84.0	91.6	90.8	84.5	85.2	75.0	71.0	91.0	89.3	72.9	69.7
Libya	95 plus	69.0	71.1	84.7	85.4	74.7	77.1	67.0	72.9	80.4	82.3	74.3	79.2	73.9	74.7	84.0	82.4	75.1	78.5	68.4	70.2	86.4	87.1	71.3	73.0
Lithuania	95 plus	57.2	58.4	78.9	80.0	66.4	70.1	64.2	57.3	79.1	77.0	72.5	64.3	57.2	33.9	72.5	55.5	60.0	35.9	58.8	61.1	82.3	83.6	64.4	69.1
Luxembourg	95 plus	60.0	77.4	81.7	90.0	68.5	81.0	62.3	71.1	79.5	84.7	70.5	73.2	65.4	72.5	74.9	81.9	77.3	75.6	61.7	76.9	84.1	91.0	66.3	78.4
Madagascar	95 plus	82.5	83.8	94.7	94.3	79.4	81.9	53.9	54.3	74.5	73.6	63.6	65.6	79.9	83.4	94.9	94.5	74.3	79.0	86.0	85.7	96.5	95.7	81.0	81.9
Malawi	95 plus	82.3	86.7	92.8	94.4	82.8	87.5	59.0	68.0	75.1	80.8	70.3	78.0	82.8	89.5	92.8	94.9	79.4	85.4	83.9	86.0	94.5	95.2	82.0	84.5
Malaysia	95 plus	84.5	84.2	93.0	89.5	86.6	90.2	74.6	64.0	85.3	73.5	76.5	74.2	77.0	70.7	85.5	74.3	77.9	80.0	84.4	85.3	94.3	91.7	84.6	89.0
Maldives	95 plus	72.5	75.1	87.8	88.9	74.7	77.3	56.1	60.9	75.3	76.8	63.7	68.2	61.3	71.9	74.7	79.9	67.7	76.9	74.7	76.1	90.4	90.9	74.9	76.0
Mali	95 plus	76.7	81.0	89.2	90.4	80.6	84.5	72.0	79.2	80.5	82.6	80.0	82.7	83.3	86.4	91.1	92.1	82.9	86.9	75.5	78.8	90.6	91.7	74.7	79.1
Malta	95 plus	70.6	81.9	87.1	91.7	74.1	86.0	75.8	86.7	86.9	91.9	77.5	86.8	88.0	86.9	92.8	92.0	87.7	86.6	68.6	78.3	87.5	91.5	69.6	80.3
Marshall Islands	95 plus	71.8	70.7	87.4	88.1	74.5	70.7	40.2	34.1	67.9	70.8	52.2	39.8	52.1	62.7	62.0	70.7	69.2	72.0	77.9	77.0	91.2	91.3	79.6	77.2
Mauritania	95 plus	78.3	78.0	90.5	90.4	81.3	81.1	70.4	74.2	79.1	80.0	82.1	82.7	86.1	87.4	92.2	92.5	86.3	88.4	78.1	76.6	92.3	91.7	75.9	75.4
Mauritius	95 plus	70.9	84.9	86.8	93.5	75.4	86.1	59.4	77.1	77.8	87.4	67.1	77.5	62.4	73.3	74.7	82.9	66.9	73.5	72.1	83.2	88.9	94.0	73.9	82.9
Mexico	95 plus	84.1	91.1	91.7	94.8	86.7	94.1	79.0	84.2	85.1	88.4	81.2	86.6	88.5	84.8	91.9	89.2	90.0	87.9	81.8	87.9	92.1	94.6	82.4	89.0
Micronesia (Federated States of)	95 plus	74.2	73.7	89.5	89.2	71.6	73.5	46.9	42.9	75.8	73.7	41.3	43.9	56.9	63.7	69.5	72.7	65.5	73.2	79.8	79.1	92.6	92.2	79.1	79.1
Monaco	95 plus	68.8	79.7	86.1	90.8	74.4	84.5	74.3	83.6	86.4	90.8	76.4	84.1	67.7	78.4	77.7	85.1	76.0	83.4	68.9	77.7	87.5	91.2	71.4	80.1
Mongolia	95 plus	70.0	83.8	87.2	93.3	66.0	74.1	44.7	83.1	70.6	90.9	52.3	59.9	28.2	25.3	52.5	43.7	35.8	50.9	81.0	81.9	93.5	93.7	76.6	78.5
Montenegro	95 plus	65.0	62.6	84.9	80.9	62.0	71.8	47.3	46.9	73.1	68.8	45.7	63.4	65.2	66.0	76.5	71.0	69.5	77.8	71.7	67.7	89.3	85.2	69.4	73.3
Morocco	95 plus	53.4	48.3	74.6	71.2	65.9	61.0	53.0	53.5	70.6	70.1	68.8	68.4	59.5	54.5	73.2	66.4	64.7	65.1	52.9	49.0	76.6	74.3	62.6	57.8
Mozambique	95 plus	81.5	86.7	93.2	95.0	78.0	83.9	58.5	67.1	77.6	81.5	65.6	73.2	84.0	90.8	94.7	97.0	75.5	83.1	83.1	86.4	94.7	95.7	77.9	81.9
Myanmar	95 plus	59.7	61.6	82.4	84.2	57.3	54.9	37.2	35.0	66.0	66.9	50.5	42.0	59.2	66.5	72.6	78.3	62.9	67.1	64.2	66.0	86.4	87.6	58.8	57.3
Namibia	95 plus	80.1	87.2	91.9	94.7	77.1	86.7	61.7	74.1	78.6	84.8	72.7	81.5	88.9	92.7	94.2	96.0	86.4	90.3	81.3	86.1	93.5	95.4	74.0	82.6
Nauru	95 plus	77.9	79.6	90.3	92.6	80.8	81.8	51.4	32.3	78.1	70.8	56.7	38.3	66.7	64.1	69.1	71.3	76.4	77.2	81.9	87.2	92.7	95.2	83.4	88.6
Nepal	95 plus	67.7	70.1	85.6	86.8	70.2	71.9	66.6	68.2	82.1	82.6	70.1	72.2	64.6	69.1	79.1	80.8	65.7	71.8	66.3	69.1	86.6	88.1	66.9	68.1
Netherlands	95 plus	79.6	78.8	90.8	90.6	85.2	82.9	76.2	74.1	87.0	85.8	78.8	77.8	67.1	66.4	77.3	78.3	75.7	69.5	78.4	77.9	91.3	91.4	81.4	79.7
New Zealand	95 plus	82.1	82.7	92.0	92.1	87.3	88.0	80.6	71.6	89.6	85.4	78.3	72.2	75.3	66.2	83.8	78.5	77.8	67.7	80.1	81.3	92.2	92.4	83.5	84.5
Nicaragua	95 plus	85.6	81.6	94.3	92.0	81.4	85.7	81.6	70.7	90.1	84.2	77.4	76.3	92.4	89.9	96.1	93.6	86.7	90.5	82.8	79.8	94.2	92.0	77.5	81.9
Niger	95 plus	78.6	75.2	90.8	89.5	79.4	76.8	69.4	69.1	81.8	79.0	77.8	77.0	84.9	82.1	92.0	90.9	81.6	82.4	78.9	74.3	92.4	91.1	74.9	71.4
Nigeria	95 plus	78.8	84.5	91.0	93.6	80.4	85.7	70.7	78.5	81.2	85.5	79.8	84.5	87.6	93.0	93.7	96.6	86.0	92.0	78.6	83.4	92.6	94.6	75.6	80.9
Niue	95 plus	79.3	82.0	91.6	93.3	76.7	79.2	53.0	51.2	77.7	79.2	43.9	37.8	61.7	69.0	71.7	77.4	74.2	77.8	83.4	86.4	94.1	95.5	81.6	84.6
North Macedonia	95 plus	74.5	67.6	89.6	85.7	68.3	67.5	70.2	60.8	85.0	79.3	63.8	61.7	69.8	64.0	81.8	75.3	65.6	68.7	74.4	68.8	91.1	88.1	67.4	66.8
Northern Mariana Islands	95 plus	79.2	82.6	91.5	93.3	81.9	84.2	44.8	49.7	73.5	76.0	52.3	54.6	54.4	61.1	52.8	64.2	77.0	77.8	87.3	90.0	95.1	96.5	88.4	89.9
Norway	95 plus	86.0	95.6	93.4	97.7	90.4	99.2	87.9	81.8	93.1	88.7	85.4	86.5	75.7	75.5	82.5	83.6	83.7	80.0	83.1	91.4	93.2	96.8	85.4	93.2
Oman	95 plus	73.1	76.9	88.7	90.2	64.4	72.0	75.9	80.3	87.3	89.2	64.2	72.5	78.3	82.8	87.0	89.5	68.7	78.6	69.5	73.7	88.8	90.4	62.6	69.1
Pakistan	95 plus	75.3	75.7	88.4	88.5	78.7	80.0	70.8	70.6	83.5	82.7	74.3	75.6	67.2	71.1	80.4	80.5	71.1	75.6	74.9	75.3	89.8	89.9	76.1	77.0
Palau	95 plus	77.0	74.4	91.0	89.4	74.8	78.1	52.4	34.7	80.0	74.8	44.5	44.3	66.2	70.7	74.4	68.6	75.4	79.7	80.2	79.6	92.9	91.4	79.2	81.8
Palestine	95 plus	21.2	40.9	62.0	72.4	47.5	59.0	41.4	50.9	66.5	72.4	65.9	69.6	72.1	78.3	81.2	85.8	76.3	79.8	9.5	37.9	61.3	74.7	36.4	53.4
Panama	95 plus	82.8	87.3	92.3	94.8	85.1	87.9	79.3	86.6	88.4	93.0	80.5	83.8	89.3	86.7	94.1	92.2	88.4	86.2	80.2	83.7	92.3	94.2	80.8	83.2
Papua New Guinea	95 plus	71.4	72.7	87.1	86.9	75.8	79.6	29.6	22.8	64.2	59.5	41.2	40.7	52.8	60.9	62.2	65.3	69.4	75.5	80.2	82.6	91.4	91.4	84.9	88.1
Paraguay	95 plus	69.0	79.4	85.7	90.4	74.0	83.7	62.4	75.0	79.9	86.6	68.5	76.7	77.9	84.3	86.2	89.9	80.9	86.9	68.8	77.6	87.0	90.9	71.3	79.7
Peru	95 plus	82.9	90.6	92.1	95.9	85.3	91.9	75.3	83.6	84.8	89.3	78.4	84.9	86.7	90.3	92.6	93.9	87.4	90.7	80.8	87.0	92.5	95.4	81.3	86.8
Philippines	95 plus	62.5	77.8	82.3	87.4	67.3	83.8	25.5	53.9	55.1	63.9	44.3	68.6	67.3	77.1	76.8	82.9	72.5	80.5	67.1	79.3	86.2	89.9	69.6	82.7
Poland	95 plus	54.4	75.0	79.6	88.5	57.3	82.9	56.5	63.8	76.7	81.0	62.0	65.0	75.2	67.6	84.3	77.6	74.8	74.2	56.1	75.2	82.5	89.9	56.2	80.3
Portugal	95 plus	54.3	62.1	79.0	82.3	62.7	69.6	53.6	58.4	74.4	77.5	66.3	67.0	62.0	56.3	72.7	68.2	73.7	68.7	57.0	63.6	82.2	84.8	61.2	67.7

Puerto Rico	95 plus	77.2	93.3	90.1	97.3	79.2	93.9	81.4	92.6	89.5	94.8	82.1	90.3	84.5	93.2	91.7	97.2	81.5	89.9	74.5	88.6	90.2	96.2	74.3	88.2
Qatar	95 plus	49.8	67.3	70.6	85.2	71.6	69.2	21.3	66.3	53.3	82.3	54.1	64.4	5.8	50.7	44.1	70.3	0.0	35.8	58.2	66.6	76.9	86.6	73.8	68.8
Republic of Korea	95 plus	55.0	79.6	79.9	91.0	61.8	84.3	31.4	78.6	65.9	89.4	38.0	76.5	45.7	80.7	62.5	87.2	66.9	85.5	61.5	77.6	84.1	91.0	65.3	80.8
Republic of Moldova	95 plus	10.0	71.6	56.0	86.3	28.4	78.7	0.0	63.9	45.6	80.9	31.9	64.0	46.3	75.5	65.6	84.7	47.5	73.5	15.4	71.9	62.1	88.3	32.1	77.0
Romania	95 plus	50.6	60.7	77.2	80.9	56.2	69.9	45.8	54.1	70.2	74.7	59.3	64.4	59.9	64.1	70.0	73.5	70.3	73.4	54.2	62.7	81.2	84.0	55.9	68.5
Russian Federation	95 plus	7.4	56.3	58.6	79.3	0.0	66.8	39.0	57.6	68.0	76.6	42.6	66.6	53.7	48.9	69.6	63.0	50.0	63.6	8.7	58.5	63.9	82.9	0.0	65.5
Rwanda	95 plus	80.1	84.3	91.9	93.8	78.8	82.6	55.4	61.9	76.9	79.3	59.8	67.7	82.8	86.2	93.6	94.3	73.1	80.3	82.8	85.4	93.8	95.2	81.4	82.5
Saint Kitts and Nevis	95 plus	62.6	65.1	82.2	83.4	70.3	74.0	64.2	67.8	79.5	80.9	72.9	77.1	82.0	80.0	90.1	89.0	79.1	75.2	61.1	63.2	83.7	84.7	66.0	69.1
Saint Lucia	95 plus	60.4	48.1	80.8	76.7	71.8	53.1	63.7	51.1	77.7	75.4	75.2	58.0	74.7	76.6	83.3	85.4	78.4	77.6	59.7	47.7	82.9	78.5	66.7	49.9
Saint Vincent and the Grenadines	95 plus	49.8	47.0	77.5	75.1	54.9	59.4	40.4	46.5	69.0	71.6	53.3	59.3	83.6	80.8	89.3	89.0	79.5	76.5	50.2	45.5	80.0	76.5	53.1	56.9
Samoa	95 plus	77.1	78.3	90.7	91.1	74.2	81.3	44.2	25.9	74.5	67.3	28.5	27.2	56.9	62.8	68.7	69.3	68.1	76.5	83.1	87.1	93.8	94.7	83.2	92.0
San Marino	95 plus	72.1	84.9	87.5	92.5	78.1	89.9	72.0	85.4	83.8	89.8	80.2	88.5	76.8	87.0	83.7	90.7	82.5	89.6	71.8	81.9	88.6	92.5	74.2	84.4
Sao Tome and Principe	95 plus	82.5	77.2	92.5	90.3	81.6	79.3	73.7	73.1	85.0	82.3	82.6	82.4	92.7	89.2	95.6	93.9	87.4	89.3	82.4	75.9	93.6	91.2	75.7	73.5
Saudi Arabia	95 plus	51.4	55.2	76.3	73.4	58.2	67.4	51.2	48.9	72.4	71.3	63.2	61.2	74.6	74.7	83.8	84.4	76.1	75.5	50.0	57.7	77.9	75.6	54.7	66.9
Senegal	95 plus	79.4	76.8	91.3	90.2	80.8	78.6	71.7	69.5	81.8	79.1	81.6	80.3	86.9	85.7	93.0	91.8	85.4	86.1	79.1	76.3	92.7	91.6	75.5	73.2
Serbia	95 plus	36.6	69.3	69.8	87.1	49.9	62.8	40.3	67.2	66.5	82.7	58.3	66.0	27.7	67.5	42.4	78.9	58.3	68.1	40.9	69.7	74.7	89.1	49.5	61.4
Seychelles	95 plus	79.1	80.0	91.1	91.1	78.4	83.7	61.2	61.4	78.6	77.7	68.3	73.0	72.3	75.7	81.3	83.2	76.6	81.3	79.7	80.3	92.6	92.5	76.7	81.2
Sierra Leone	95 plus	77.2	75.3	90.2	89.1	79.4	78.6	72.4	71.1	81.2	79.3	81.5	81.1	85.7	85.8	92.3	92.1	85.4	86.2	76.0	74.1	91.5	90.4	73.5	72.6
Singapore	95 plus	87.7	96.1	94.2	98.2	95.8	98.4	95.2	100.0	97.3	100.0	91.4	93.4	100.0	98.7	99.9	99.9	100.0	95.4	83.1	90.0	93.0	96.3	89.6	91.9
Slovakia	95 plus	82.8	86.1	91.6	93.8	90.6	90.1	62.1	72.1	79.2	84.7	68.9	74.6	71.1	73.3	80.0	81.4	76.0	78.7	82.0	84.2	92.3	93.9	87.3	86.2
Slovenia	95 plus	76.8	75.0	87.7	89.1	83.8	76.1	79.1	72.7	86.1	85.4	81.6	71.8	72.8	66.4	79.4	77.1	79.3	71.8	75.7	74.6	89.3	90.3	79.9	73.7
Solomon Islands	95 plus	73.1	72.1	88.5	87.7	74.9	74.7	44.2	40.4	74.1	71.8	48.1	47.0	54.9	63.7	65.4	71.1	67.4	73.5	80.2	79.0	92.0	91.3	82.5	81.8
Somalia	95 plus	81.0	87.7	92.7	94.9	79.6	87.5	53.0	73.8	75.4	84.5	60.5	77.3	80.2	90.2	93.8	96.8	73.2	84.4	84.4	87.2	94.7	95.8	82.6	85.6
South Sudan	95 plus	86.2	85.7	96.3	96.1	86.3	87.1	69.6	65.9	80.3	80.1	70.9	71.9	93.3	90.3	95.8	95.8	81.7	83.1	85.9	84.9	97.6	96.8	86.7	86.3
Spain	95 plus	88.5	88.2	84.8	89.1	71.8	88.7	65.0	65.9	80.5	83.5	71.2	73.2	86.5	85.8	83.1	78.7	79.6	67.1	90.3	88.7	86.6	90.1	69.4	86.4
Sri Lanka	95 plus	66.4	78.6	79.7	83.9	59.4	58.2	64.3	69.4	56.5	61.2	48.0	43.5	75.0	66.2	83.0	85.7	77.8	79.7	67.2	78.4	83.8	86.9	59.9	59.2
Sudan	95 plus	55.4	61.4	71.5	75.4	56.1	65.1	27.5	26.1	63.6	71.5	56.8	70.6	74.9	78.1	68.8	69.4	50.7	66.6	59.5	65.3	74.2	78.0	54.9	61.5
Suriname	95 plus	45.7	55.6	87.6	89.3	79.9	85.1	38.0	57.0	83.5	85.2	78.0	81.4	47.5	57.5	93.4	93.7	84.6	87.4	46.8	55.5	88.2	89.9	75.4	80.7
Sweden	95 plus	73.5	78.8	92.1	96.2	90.9	97.4	71.2	76.4	91.9	91.6	90.9	90.3	87.7	88.5	76.8	84.6	85.1	86.2	71.2	76.5	92.0	95.6	85.4	91.2
Switzerland	95 plus	83.2	92.9	85.5	92.3	75.8	91.2	86.8	87.3	85.2	90.6	83.8	91.7	69.7	79.1	77.6	79.7	78.9	75.7	80.8	89.0	87.0	92.4	71.2	85.8
Syrian Arab Republic	95 plus	68.4	84.4	79.3	78.1	67.4	62.1	75.3	86.7	74.6	74.0	67.2	62.2	68.7	69.9	80.3	81.0	70.6	74.2	68.0	81.8	81.2	80.4	64.8	60.2
Taiwan (Province of China)	95 plus	61.1	59.6	94.5	99.9	92.5	99.9	57.8	53.6	69.7	87.4	45.9	75.7	67.6	69.7	74.7	85.8	69.5	80.2	60.7	60.7	97.1	100.0	96.9	100.0
Tajikistan	95 plus	88.0	100.0	79.6	81.8	50.9	62.7	40.0	76.0	71.6	76.7	59.5	67.0	63.2	78.3	69.6	72.5	63.0	71.9	93.3	100.0	82.3	83.6	45.6	57.6
Thailand	95 plus	52.8	60.6	92.2	93.6	80.0	88.6	48.0	58.8	80.8	80.8	64.9	72.5	55.3	62.8	66.8	73.3	48.7	71.5	53.2	59.2	94.4	95.3	81.6	88.5
Timor-Leste	95 plus	81.7	85.8	83.7	80.7	67.3	58.8	64.3	66.5	65.9	60.7	53.9	46.3	50.3	62.8	72.7	70.6	66.2	67.2	84.0	87.3	87.8	85.5	69.3	61.3
Togo	95 plus	64.1	56.7	89.7	88.6	78.5	74.5	38.7	28.8	80.1	77.6	80.5	78.3	60.0	58.7	92.1	91.0	84.7	84.8	68.9	62.4	91.2	90.2	72.5	68.4
Tokelau	95 plus	76.2	73.4	89.0	91.6	71.4	82.5	69.6	64.0	74.3	77.4	36.9	44.6	85.5	84.8	67.3	77.4	68.6	79.0	75.6	73.4	91.9	93.7	78.3	87.6
Tonga	95 plus	72.2	79.8	93.5	93.1	85.2	86.5	38.9	44.7	81.0	79.6	64.2	61.7	57.7	71.4	78.3	79.8	81.9	83.6	78.1	84.5	96.0	95.1	86.9	88.1
Trinidad and Tobago	95 plus	84.4	83.9	78.6	88.3	68.5	81.1	63.3	60.6	77.8	86.8	72.3	80.9	71.4	72.7	83.8	87.8	76.0	80.8	87.9	86.6	79.5	88.6	65.1	77.0
Tropical Latin America	95 plus	56.2	75.3	86.2	92.2	75.5	88.6	60.6	77.2	83.7	87.0	75.4	80.8	74.3	80.2	91.1	87.0	87.9	84.9	54.8	73.2	87.1	92.5	71.4	84.1
Tunisia	95 plus	50.7	57.8	77.3	79.7	57.8	65.1	51.8	62.5	73.7	78.4	64.5	71.7	61.2	65.4	74.7	76.3	61.2	70.6	48.6	56.6	78.9	81.4	53.8	61.1
Turkey	95 plus	67.5	74.5	83.9	86.3	78.6	85.7	60.5	71.3	77.1	82.0	73.8	80.8	54.3	64.4	70.2	75.2	56.1	71.5	68.7	74.2	86.4	88.0	76.3	82.0
Turkmenistan	95 plus	65.0	76.6	85.6	90.2	60.5	63.4	63.5	72.2	80.7	85.3	66.6	65.3	63.2	61.7	76.2	74.5	66.8	59.6	65.0	76.1	87.3	91.2	56.6	61.2
Tuvalu	95 plus	70.6	71.2	87.8	88.1	67.9	72.4	37.6	34.5	70.5	69.4	32.4	40.6	50.9	62.1	64.2	69.3	63.9	74.2	77.1	76.9	91.4	91.2	76.2	77.3
Uganda	95 plus	88.7	94.5	96.7	98.8	83.6	88.9	59.3	67.7	79.7	82.1	62.3	72.5	82.9	90.0	94.4	95.9	74.7	83.3	90.8	93.9	98.0	99.1	85.3	87.8
Ukraine	95 plus	47.8	64.2	74.8	84.1	59.6	65.5	69.8	70.7	84.1	84.5	66.6	65.3	5.4	35.7	33.0	55.7	21.0	38.5	50.9	65.7	78.9	86.6	59.7	65.0
United Arab Emirates	95 plus	77.4	76.6	89.9	80.7	79.1	79.4	77.9	70.4	88.4	76.8	77.3	74.2	61.3	56.3	77.0	57.0	64.3	65.8	75.9	76.5	90.7	83.6	75.9	76.9
United Kingdom	95 plus	74.2	85.2	87.7	93.3	84.9	89.8	77.9	72.6	86.7	84.4	85.5	79.2	67.1	63.8	76.3	75.3	80.2	71.1	73.6	84.1	88.8	93.8	80.5	86.1

United Republic of Tanzania	95 plus	83.9	87.5	94.0	95.2	81.8	87.4	58.1	66.5	78.4	80.0	62.7	74.9	84.9	88.5	95.3	95.5	77.1	84.5	86.1	87.3	95.5	96.0	83.4	85.1
United States Virgin Islands	95 plus	57.2	65.0	80.9	84.8	60.9	64.7	54.4	61.0	76.1	80.4	62.0	62.5	77.2	78.6	85.5	87.1	70.2	75.1	55.5	64.0	81.9	85.8	57.7	62.4
United States of America	95 plus	90.2	92.5	95.8	96.9	93.8	96.3	90.4	89.3	94.0	93.2	89.3	91.8	78.3	68.7	85.3	78.4	83.0	75.4	86.4	89.0	94.9	95.8	88.4	91.2
Uruguay	95 plus	68.8	76.1	86.0	88.6	74.4	83.6	80.6	82.7	90.4	90.6	78.1	82.7	57.7	64.8	59.4	69.4	77.2	79.0	68.0	74.5	87.0	89.3	70.7	79.3
Uzbekistan	95 plus	64.1	73.4	84.5	88.5	61.2	62.4	53.1	74.6	74.4	85.9	61.8	69.1	65.4	66.6	79.7	78.3	61.0	64.1	66.3	70.8	87.4	89.3	59.4	57.4
Vanuatu	95 plus	74.4	74.9	90.1	89.9	72.8	74.3	42.1	40.3	75.1	72.6	41.0	41.8	58.3	63.7	68.4	72.2	68.8	73.8	79.9	80.1	92.9	92.8	78.5	79.1
Venezuela (Bolivarian Republic of)	95 plus	84.2	88.6	92.2	95.0	88.0	90.7	74.2	82.5	84.2	90.0	79.5	82.6	91.0	91.5	95.9	95.4	89.8	89.7	82.4	85.4	92.6	94.6	84.1	86.2
Viet Nam	95 plus	53.4	51.1	79.7	78.8	48.1	44.3	25.9	26.5	60.8	61.2	39.0	39.3	53.5	62.5	68.5	74.2	56.5	68.4	59.8	56.4	84.5	83.2	52.2	46.8
Yemen	95 plus	39.2	37.0	71.6	68.7	48.3	46.7	33.3	38.1	64.2	63.0	49.7	59.3	48.8	45.5	68.6	64.0	39.1	52.6	38.3	36.4	73.9	71.8	48.3	42.2
Zambia	95 plus	82.5	89.1	93.6	95.8	81.1	88.7	54.7	72.7	75.5	83.2	63.1	77.6	79.9	89.7	93.6	97.0	74.9	85.6	85.7	88.7	95.4	96.4	83.6	87.2
Zimbabwe	95 plus	78.6	85.6	91.2	93.7	74.0	87.5	43.7	69.9	69.5	81.4	63.1	80.8	80.2	88.0	88.7	92.9	78.4	87.5	80.4	84.2	93.1	94.4	72.6	83.3

ICH, intracerebral hemorrhage; SAH, subarachnoid hemorrhage; IS, ischemic stroke; QCI, quality of care index

Table S9 QCI values by sex, age group and countires for stroke, ICH, SAH, and IS in 1990 and 2021

ICH, intracerebral hemorrhage; SAH, subarachnoid hemorrhage; IS, ischemic stroke; QCI, quality of care index

Table S10 Percent changes of the QCIs for stroke, ICH, SAH, and IS in SDI regions between 1990 and 2021 for both sexes, females, and males

	QCI											
	stroke			ICH			SAH			IS		
**location**	**both**	**female**	**male**	**both**	**female**	**male**	**both**	**female**	**male**	**both**	**female**	**male**
Global	28.1	2.3	33.8	46.6	79.7	75.2	84.7	12.1	80.5	84.3	71.6	77.2
High SDI	28.1	2.3	34.0	46.5	77.8	74.2	83.5	4.5	80.6	87.2	72.1	78.0
High-middle SDI	28.2	2.3	33.5	46.5	79.7	75.6	84.8	12.2	80.5	90.7	71.4	77.4
Low SDI	28.1	2.2	33.6	46.5	80.7	75.9	84.8	12.7	80.5	87.8	72.9	77.9
Low-middle SDI	28.2	2.3	33.1	46.5	79.7	74.6	84.8	12.6	80.5	86.4	71.8	79.1
Middle SDI	28.9	2.4	43.0	46.5	79.7	75.2	84.8	12.3	80.5	88.6	23.9	78.1

ICH, intracerebral hemorrhage; SAH, subarachnoid hemorrhage; IS, ischemic stroke; QCI, quality of care index; SDI, sociodemographic index

Table S11 Percent changes of the QCI for stroke, ICH, SAH, and IS in 21 GBD regions between 1990 and 2021 for both sexes, females, and males

	QCI											
	stroke			ICH			SAH			IS		
**location**	**both**	**female**	**male**	**both**	**female**	**male**	**both**	**female**	**male**	**both**	**female**	**male**
Andean Latin America	28.1	2.2	33.9	46.5	80.0	76.0	84.9	12.9	80.5	87.5	72.5	77.8
Australasia	28.0	2.3	34.0	46.6	77.0	75.1	84.2	10.5	80.4	87.5	72.5	77.8
Caribbean	28.0	2.3	34.3	46.5	79.6	76.5	84.6	12.1	80.1	87.0	71.8	77.1
Central Asia	27.9	2.2	33.7	46.3	77.3	77.1	85.2	12.3	75.9	87.4	72.9	75.3
Central Europe	28.1	2.2	33.9	46.5	79.6	76.6	84.8	12.3	80.3	87.5	72.5	77.7
Central Latin America	28.1	2.2	33.7	46.5	79.8	76.5	85.2	12.3	80.2	87.7	72.8	77.8
Central Sub-Saharan Africa	28.2	2.1	33.0	46.5	81.8	77.3	84.2	10.8	80.5	87.8	73.5	77.9
East Asia	25.9	2.8	50.1	46.5	79.7	74.8	84.9	12.6	80.5	88.3	74.9	78.1

Eastern Europe	28.1	2.3	34.1	46.5	78.8	76.5	84.9	12.9	80.3	87.3	72.3	78.0
Eastern Sub-Saharan Africa	28.1	2.2	33.8	46.5	81.3	76.6	84.8	12.6	80.4	87.6	73.0	77.2
High-income Asia Pacific	28.0	2.3	34.0	46.5	79.2	75.4	100.0	49.7	80.9	87.6	72.5	77.8
High-income North America	29.1	2.0	32.8	46.3	100.0	85.1	87.8	26.3	77.0	86.6	72.1	78.5
North Africa and Middle East	28.1	2.3	34.0	46.5	79.6	75.7	84.7	12.3	80.4	86.7	71.8	77.9
Oceania	28.1	2.2	33.8	46.5	81.5	76.6	84.7	12.5	80.4	87.7	72.9	77.4
South Asia	28.2	2.2	33.3	46.6	79.7	71.7	84.8	12.9	80.5	87.7	72.4	78.6
Southeast Asia	28.1	2.3	33.7	46.5	80.4	76.6	84.8	12.4	80.5	0.0	71.6	77.9
Southern Latin America	28.1	2.2	33.8	46.5	79.2	76.1	84.7	12.3	80.4	87.6	72.5	77.6
Southern Sub-Saharan Africa	29.2	1.4	30.1	46.5	86.0	78.5	85.5	13.8	80.3	77.5	100.0	71.1
Tropical Latin America	28.1	2.2	33.7	46.5	80.7	76.6	84.9	12.5	80.4	87.7	72.7	77.8
Western Europe	28.1	2.3	34.0	46.6	77.9	75.1	84.6	11.6	80.4	87.5	72.5	77.9
Western Sub-Saharan Africa	28.1	2.3	33.7	46.5	78.8	75.7	84.8	12.4	80.4	87.6	72.8	77.8

ICH, intracerebral hemorrhage; SAH, subarachnoid hemorrhage; IS, ischemic stroke; QCI, quality of care index

Table S12 Percent changes of the QCIs for stroke, ICH, SAH, and IS in countries between 1990 and 2021 for both sexes, females, and males

	QCI											
	stroke			ICH			SAH			IS		
location	both	female	male	both	female	male	both	female	male	both	female	male
Afghanistan	28.1	2.1	33.8	46.5	82.2	76.1	84.6	11.3	80.2	87.8	73.6	79.1
Albania	28.1	1.9	34.0	46.4	79.7	76.0	83.8	9.4	80.2	87.5	75.1	77.5
Algeria	28.1	2.4	33.7	46.5	80.9	76.3	84.9	12.6	80.4	86.3	86.6	78.1
American Samoa	28.1	2.2	33.5	46.5	81.9	76.9	85.0	13.4	80.5	87.7	73.1	77.5
Andorra	28.0	2.3	33.9	46.6	68.4	73.6	84.0	9.9	80.4	87.6	72.4	77.6
Angola	28.2	2.2	33.5	46.5	81.5	76.4	84.8	12.5	80.5	88.0	73.6	78.3
Antigua and Barbuda	28.1	2.3	33.7	46.5	78.2	76.2	84.6	11.9	80.4	87.5	72.3	77.7
Argentina	28.0	2.2	33.8	46.5	79.8	76.5	84.8	12.4	80.4	87.6	72.4	77.6
Armenia	28.2	2.2	33.5	46.5	77.4	75.9	84.8	12.3	80.5	87.8	72.9	78.7
Australia	28.0	2.3	34.0	46.6	77.0	75.1	83.7	8.5	80.4	87.5	72.5	77.8
Austria	36.2	2.9	33.7	46.5	79.9	76.3	84.7	11.5	80.5	89.8	69.4	78.3
Azerbaijan	29.5	2.1	9.0	47.2	88.1	30.8	84.7	12.0	80.5	73.7	74.1	80.9
Bahamas	28.0	2.3	34.1	46.5	77.9	75.5	84.5	11.7	80.4	87.5	72.6	77.8
Bahrain	28.1	2.2	33.8	46.5	79.0	75.3	84.7	12.0	80.5	87.6	72.7	77.9
Bangladesh	28.1	2.3	33.6	46.5	77.8	75.6	84.0	11.1	80.2	84.3	74.6	77.6
Barbados	28.1	2.3	33.8	46.5	77.9	76.0	84.8	12.5	80.5	87.5	72.2	77.8
Belarus	28.2	2.3	33.4	46.5	78.4	77.7	85.1	11.8	80.7	87.5	72.4	78.1
Belgium	28.1	2.3	33.9	46.5	78.5	75.8	84.6	11.4	80.3	87.2	72.3	78.1
Belize	28.1	2.2	35.2	46.5	79.9	76.0	84.5	12.3	80.3	87.6	73.1	71.9
Benin	28.1	2.2	33.2	46.5	78.5	76.1	84.7	12.1	80.4	87.9	73.2	77.7
Bermuda	28.1	2.3	33.9	46.6	76.8	75.5	84.6	11.5	80.4	87.6	72.5	77.6
Bhutan	28.1	2.3	33.7	46.5	79.6	75.4	84.8	12.5	80.5	85.8	71.6	78.3
Bolivia (Plurinational State of)	28.1	2.2	33.7	46.5	80.3	76.4	84.9	12.4	80.4	87.6	72.9	77.4
Bosnia and Herzegovina	28.3	2.3	33.5	46.5	75.2	75.8	84.6	11.7	80.4	86.1	71.9	78.2
Botswana	26.7	9.0	36.1	46.4	72.2	76.0	84.1	9.6	80.3	97.7	76.8	83.3
Brazil	28.1	2.2	33.7	46.5	80.7	76.6	84.9	12.5	80.3	87.7	72.7	77.8

Brunei Darussalam	28.1	2.1	33.1	46.5	80.7	76.8	84.9	12.6	80.5	87.8	73.2	77.6
Bulgaria	28.0	2.3	33.8	46.5	79.5	76.7	85.2	14.9	83.7	86.4	64.1	76.2
Burkina Faso	28.2	2.0	32.8	46.4	80.0	76.9	84.9	12.7	80.4	88.0	75.4	77.1
Burundi	28.1	2.1	33.5	46.5	81.4	76.7	84.5	12.0	80.3	87.7	73.1	77.5
Cabo Verde	29.5	2.1	45.6	46.5	80.3	77.8	84.6	11.0	80.4	95.2	66.1	77.8
Cambodia	28.1	2.4	33.4	46.5	77.5	76.0	84.5	11.1	80.5	90.5	74.6	78.8
Cameroon	28.2	2.1	34.7	46.5	81.5	75.3	84.9	13.0	80.3	95.8	78.0	78.4
Canada	28.2	2.3	33.8	46.9	70.1	69.0	84.7	12.3	80.4	87.0	72.2	78.1
Central African Republic	28.2	2.1	33.2	46.5	82.2	77.3	83.8	10.6	79.9	87.6	73.5	77.4
Chad	28.7	1.9	30.5	46.5	84.0	78.5	85.5	14.4	80.4	88.9	76.6	78.7
Chile	28.1	2.3	33.9	46.6	77.1	74.5	83.5	5.8	80.6	87.5	72.4	77.7
China	25.8	2.9	53.3	46.5	79.6	74.7	84.9	12.7	80.5	88.3	74.8	78.1
Colombia	28.1	2.2	33.6	46.5	79.4	76.1	84.7	11.5	80.1	87.7	72.8	77.7
Comoros	28.1	2.2	33.6	46.5	80.8	76.6	84.7	12.1	80.4	87.6	72.9	77.5
Congo	28.1	2.2	33.4	46.5	81.6	76.8	84.8	12.8	80.5	87.8	73.3	77.6
Cook Islands	28.5	2.4	89.2	46.6	76.2	73.0	84.5	12.1	80.5	94.0	70.7	77.6
Costa Rica	28.1	2.2	33.9	46.5	78.0	75.3	85.3	13.3	80.6	87.6	72.6	77.9
Coted'Ivoire	28.2	2.0	32.8	46.5	82.5	76.7	84.9	12.9	80.4	87.9	74.0	77.5
Croatia	28.1	2.3	34.0	46.5	79.6	76.5	84.8	12.3	80.5	87.3	72.3	78.0
Cuba	28.1	2.2	33.7	46.5	80.0	76.8	84.8	12.3	80.4	87.7	72.6	77.5
Cyprus	28.0	2.2	33.7	46.5	77.8	75.0	84.7	12.1	80.4	87.7	72.9	77.5
Czechia	28.1	2.3	34.0	46.5	76.9	75.7	84.7	12.2	80.4	87.5	72.4	77.9
Democratic People's Republic of Korea	28.0	2.2	33.7	46.5	83.3	78.2	84.8	12.5	80.5	87.8	75.0	79.4
Democratic Republic of the Congo	28.3	2.1	32.5	46.5	82.2	77.8	89.3	3.8	80.4	87.8	73.5	77.7
Denmark	28.1	2.2	33.4	46.6	77.3	75.7	84.7	12.3	80.4	87.8	72.9	77.7
Djibouti	28.1	2.3	33.9	46.5	81.0	76.6	84.6	12.1	80.4	87.2	72.2	88.4
Dominica	28.0	2.2	34.4	46.5	80.3	76.4	84.9	12.8	80.4	87.5	73.0	75.8
Dominican Republic	28.6	1.2	32.9	47.4	70.5	100.0	87.4	19.3	81.2	88.6	74.7	78.6
Ecuador	28.0	2.2	33.9	46.5	78.3	75.7	85.6	13.6	80.5	87.4	72.5	77.3
Egypt	32.4	1.1	16.2	46.4	76.6	73.4	84.5	11.7	80.5	88.3	74.0	77.2
El Salvador	28.1	2.2	33.5	46.5	80.4	76.9	84.9	12.6	80.4	87.6	72.8	77.6
Equatorial Guinea	28.1	2.3	33.7	46.5	79.8	76.0	84.7	12.3	80.5	87.7	73.0	78.1
Eritrea	28.2	2.3	33.5	46.5	80.5	76.5	84.2	10.4	80.5	87.8	72.5	78.5
Estonia	28.1	2.3	33.7	46.5	78.6	76.6	84.9	12.6	80.4	87.6	72.3	77.9
Eswatini	30.6	1.2	29.3	46.4	81.7	86.8	84.8	13.5	76.9	90.9	71.6	78.5
Ethiopia	28.1	2.2	33.6	46.5	81.2	76.6	84.9	12.8	80.4	87.8	73.2	77.9
Fiji	28.2	2.1	33.4	46.5	82.4	76.9	84.9	12.8	80.5	88.0	73.8	77.7
Finland	28.1	2.3	33.8	46.8	63.0	71.9	84.6	11.2	80.3	87.6	72.3	77.6
France	29.2	2.6	31.7	46.8	73.8	68.5	82.4	16.3	80.3	59.1	70.5	78.0
Gabon	28.1	2.1	33.6	46.5	81.5	76.6	84.9	12.8	80.5	87.8	73.8	77.0
Gambia	28.7	1.5	31.3	46.5	84.6	77.8	85.3	14.9	80.4	88.2	78.4	77.9
Georgia	28.3	2.2	51.0	46.5	80.4	76.7	84.3	2.0	80.5	86.8	64.9	77.0
Germany	28.0	2.3	34.2	46.6	75.3	72.8	84.7	12.2	80.5	87.4	72.2	77.7
Ghana	28.4	2.2	32.3	46.5	80.5	76.2	84.5	12.2	0.0	87.8	73.8	77.5
Greece	28.0	2.2	34.1	46.5	79.0	76.1	86.6	22.7	82.3	87.5	72.6	77.8
Greenland	28.1	2.2	33.4	46.5	80.2	76.4	88.6	6.1	81.1	87.8	73.0	77.5
Grenada	28.0	2.2	35.0	46.5	79.1	76.2	84.7	11.6	80.3	87.4	72.6	0.0

Guam	29.8	2.4	7.4	46.6	68.4	68.8	83.6	10.6	80.1	62.8	69.6	78.7
Guatemala	28.0	2.2	33.6	46.5	80.3	77.1	90.7	14.1	80.3	87.5	72.6	77.4
Guinea	28.4	2.2	36.7	46.4	80.0	79.0	84.9	13.1	79.4	97.3	74.3	77.3
Guinea-Bissau	28.2	2.0	33.2	46.5	82.3	76.8	84.9	13.0	80.3	87.8	75.9	77.5
Guyana	28.1	2.2	33.5	46.5	80.1	76.6	84.9	12.9	80.4	87.7	72.9	77.6
Haiti	28.1	2.2	33.6	46.5	80.9	76.7	83.6	10.2	79.8	87.4	72.3	77.5
Honduras	0.0	100.0	100.0	46.5	89.3	78.8	87.1	26.0	77.7	89.6	66.0	79.1
Hungary	28.1	2.2	33.5	46.5	79.1	76.6	84.6	12.1	80.4	87.7	72.8	77.5
Iceland	28.1	2.2	33.8	46.5	79.3	75.7	84.7	12.2	80.4	87.7	72.8	77.8
India	28.3	2.2	33.0	46.6	79.8	64.1	84.9	12.9	80.6	87.7	72.5	78.7
Indonesia	100.0	1.7	41.7	46.5	82.6	78.2	84.9	12.5	80.3	85.5	71.2	77.9
Iran (Islamic Republic of)	28.1	2.2	33.4	46.5	79.0	74.8	84.6	11.8	80.5	87.4	72.7	78.3
Iraq	28.2	2.2	32.9	46.5	79.7	75.7	85.0	12.9	80.5	82.3	70.3	78.4
Ireland	28.1	2.2	33.5	46.5	78.0	75.4	84.9	12.5	80.4	87.8	72.9	77.7
Israel	28.1	2.2	33.7	46.5	78.1	75.6	84.6	11.3	80.4	87.7	72.6	77.8
Italy	28.1	2.2	33.8	46.6	79.4	75.3	84.8	11.9	80.4	87.7	72.7	78.0
Jamaica	28.1	2.2	33.5	46.5	77.1	76.6	84.5	11.3	80.3	87.5	72.5	77.3
Japan	28.1	2.3	34.2	46.5	78.4	74.2	86.7	24.5	83.3	87.4	72.4	77.9
Jordan	28.7	2.3	44.2	46.5	79.5	75.3	84.6	12.2	80.5	98.8	71.9	78.6
Kazakhstan	28.4	2.0	31.9	46.5	83.8	78.4	85.4	10.5	80.8	87.8	73.4	77.2
Kenya	28.4	2.0	28.0	46.5	83.6	78.5	85.2	13.6	80.5	85.0	74.7	78.2
Kiribati	28.3	2.0	32.9	46.5	83.5	77.8	85.6	9.6	80.4	88.0	75.4	78.1
Kuwait	27.7	2.4	34.1	46.3	68.8	81.6	83.6	9.7	80.3	87.1	72.0	69.8
Kyrgyzstan	28.1	2.2	33.5	46.5	80.0	76.9	85.0	13.0	80.3	87.8	72.9	77.4
Lao People's Democratic Republic	28.1	2.3	33.9	46.5	78.5	75.5	84.6	11.7	80.5	86.3	71.0	77.6
Latvia	28.3	2.2	42.2	46.5	79.4	76.4	85.0	12.9	80.5	86.6	72.5	78.9
Lebanon	32.7	2.2	54.8	46.5	77.3	74.4	84.6	11.6	80.5	90.8	70.1	78.2
Lesotho	28.2	2.2	32.9	46.8	76.7	75.0	82.0	6.1	80.9	87.5	72.5	77.9
Liberia	28.3	2.1	32.7	46.5	82.8	77.4	85.1	13.4	80.4	88.1	73.7	78.1
Libya	27.7	1.5	32.5	46.5	82.2	77.1	85.1	13.3	80.5	87.9	73.4	77.9
Lithuania	28.4	2.3	55.6	46.6	76.9	75.2	84.7	10.2	80.6	86.8	72.5	79.1
Luxembourg	28.1	2.2	33.7	46.5	79.0	76.0	84.5	11.4	80.4	87.7	72.8	77.7
Madagascar	28.1	2.1	33.6	46.5	82.8	77.1	83.9	12.4	80.8	85.9	92.9	77.2
Malawi	28.3	2.1	32.6	46.5	82.8	78.2	85.1	12.7	80.5	88.5	74.1	73.7
Malaysia	28.3	2.2	33.7	46.5	80.1	75.9	84.9	12.9	80.5	84.7	71.6	78.6
Maldives	28.1	2.2	33.4	46.5	80.3	76.3	84.8	12.4	80.5	87.7	73.1	77.4
Mali	28.2	2.1	33.2	46.5	81.3	75.7	84.8	12.8	80.4	88.1	73.3	77.9
Malta	28.1	2.2	33.8	46.6	76.7	74.3	84.7	11.9	80.4	87.8	72.9	77.8
Marshall Islands	28.0	2.3	34.2	46.5	81.6	75.5	84.1	10.8	80.6	87.2	72.7	77.5
Mauritania	28.2	2.1	33.1	46.5	80.1	76.4	84.7	12.4	80.4	87.9	73.5	77.8
Mauritius	28.1	2.2	33.5	46.5	79.7	76.1	84.6	12.4	80.5	87.7	72.9	77.6
Mexico	28.0	2.2	34.1	46.5	79.4	76.3	86.3	10.8	80.7	87.7	72.7	77.9
Micronesia (Federated States of)	28.0	2.3	34.0	46.5	79.5	76.7	83.2	10.8	75.5	87.4	72.3	76.8
Monaco	28.1	2.2	33.8	46.6	75.3	74.6	84.6	12.0	80.5	87.7	72.7	77.7
Mongolia	87.7	2.7	28.9	45.9	73.8	55.6	85.6	20.0	80.8	88.3	74.2	77.9
Montenegro	29.8	1.0	27.8	46.5	91.0	78.5	84.7	12.1	80.3	92.4	58.3	69.2
Morocco	28.1	2.4	33.8	46.5	80.5	76.2	84.8	12.4	80.4	88.8	74.4	78.2

Mozambique	28.2	2.6	33.1	46.6	81.6	78.1	0.0	13.5	80.3	87.6	73.4	77.4
Myanmar	28.1	2.2	33.6	46.5	79.7	76.2	84.8	12.4	80.5	87.3	72.3	78.0
Namibia	28.2	2.1	32.9	46.5	81.4	77.6	84.9	12.6	80.4	87.6	73.9	75.4
Nauru	28.2	2.2	33.4	46.5	83.6	77.8	87.9	13.1	79.7	88.0	72.7	78.2
Nepal	28.0	2.3	33.3	46.5	78.3	76.3	84.6	11.6	80.4	87.2	72.1	74.8
Netherlands	28.1	2.2	33.3	46.6	77.0	75.0	84.7	12.3	80.5	87.8	73.1	77.7
New Zealand	28.1	2.2	33.8	46.6	77.1	75.4	84.9	12.6	80.5	87.6	72.6	77.9
Nicaragua	28.1	2.2	33.9	46.5	79.3	76.0	84.8	12.2	80.5	87.7	72.9	77.8
Niger	28.3	2.0	32.5	46.5	82.9	77.0	84.6	12.1	80.2	88.1	73.8	78.0
Nigeria	28.1	2.4	33.8	46.5	76.1	75.2	84.8	12.1	80.4	86.8	71.3	77.7
Niue	28.1	2.2	33.6	46.5	81.1	76.9	85.2	14.4	80.5	87.8	72.6	77.6
North Macedonia	28.8	1.0	32.0	46.4	80.5	76.8	85.9	15.7	84.3	86.8	0.0	76.7
Northern Mariana Islands	27.9	2.3	34.2	46.5	78.8	76.8	84.6	11.9	80.4	87.0	72.4	87.8
Norway	28.0	2.2	33.9	46.6	78.3	72.0	84.6	11.5	80.4	87.6	72.6	77.6
Oman	28.2	2.5	25.2	46.5	77.7	75.1	86.4	16.3	100.0	91.4	69.2	78.2
Pakistan	28.2	2.2	33.6	46.5	83.0	76.9	85.2	13.9	80.6	90.0	70.8	78.9
Palau	28.3	2.1	33.8	46.5	81.0	75.4	84.9	13.1	80.6	87.2	74.1	72.1
Palestine	27.7	2.4	35.9	46.5	79.3	75.9	84.3	11.2	80.4	84.6	67.8	100.0
Panama	28.1	2.2	33.6	46.5	78.9	76.2	85.4	13.2	80.5	87.7	72.6	77.9
Papua New Guinea	28.1	2.2	33.9	46.5	80.0	76.0	84.6	11.9	80.5	87.5	72.7	77.6
Paraguay	28.1	2.2	33.7	46.5	81.0	76.9	84.8	12.2	80.4	87.7	72.7	77.5
Peru	28.1	2.2	33.9	46.5	80.4	76.0	84.9	12.9	80.5	87.5	72.5	78.1
Philippines	28.5	1.8	32.4	46.5	90.0	78.5	85.2	13.6	80.3	88.5	74.2	78.6
Poland	28.0	2.3	34.3	46.5	79.3	76.5	84.9	12.6	80.4	86.3	71.9	77.6
Portugal	28.1	2.1	33.4	46.5	80.1	76.5	84.9	12.6	80.4	87.8	73.1	77.6
Puerto Rico	28.1	2.4	34.2	46.6	76.5	75.9	84.7	12.0	80.4	86.3	71.0	77.8
Qatar	28.0	2.2	33.5	46.5	78.0	75.8	83.7	10.9	80.3	87.6	73.0	77.3
Republic of Korea	28.1	2.2	33.5	46.5	80.4	76.4	84.7	12.2	80.5	87.8	73.0	77.6
Republic of Moldova	28.2	2.3	32.3	46.5	80.5	76.6	84.9	12.8	80.5	91.0	71.5	79.3
Romania	28.1	2.2	33.8	46.5	79.7	76.9	84.4	11.5	82.6	87.5	72.5	78.0
Russian Federation	28.1	2.3	33.7	46.5	79.2	76.5	86.2	21.0	78.6	87.0	72.2	78.3
Rwanda	28.1	2.2	33.4	46.5	80.8	76.8	84.7	12.3	80.4	87.7	73.0	77.5
Saint Kitts and Nevis	28.1	2.1	33.6	46.5	80.8	76.7	84.8	12.4	80.4	87.7	73.3	77.3
Saint Lucia	28.1	2.2	33.8	46.5	79.2	76.2	84.6	11.9	80.4	87.7	72.7	77.8
Saint Vincent and the Grenadines	28.0	2.2	33.9	46.3	76.7	77.2	85.0	12.8	80.5	87.8	73.0	77.6
Samoa	28.2	2.1	34.1	46.5	83.1	75.6	83.4	0.0	80.5	86.4	71.5	77.8
San Marino	28.0	2.3	34.1	46.6	71.4	73.8	84.0	9.9	80.4	87.6	72.4	77.5
Sao Tome and Principe	28.4	2.0	35.2	46.5	81.3	77.0	85.2	13.5	80.5	93.2	67.6	78.4
Saudi Arabia	30.1	2.3	25.3	46.5	75.6	74.6	84.6	11.2	80.5	91.3	71.4	78.6
Senegal	28.3	2.1	32.7	46.5	81.2	76.1	84.9	12.8	80.4	88.0	73.7	77.9
Serbia	28.0	2.2	33.7	46.5	79.8	76.9	84.5	11.4	80.1	87.5	72.9	77.4
Seychelles	28.1	2.2	33.9	46.5	78.8	75.9	84.7	12.0	80.5	87.6	72.8	77.7
Sierra Leone	28.2	2.0	33.2	46.5	82.6	76.2	84.9	15.4	80.3	87.9	74.5	77.7
Singapore	28.1	2.2	33.6	46.5	79.6	76.1	84.7	12.2	80.4	87.8	72.9	77.6
Slovakia	28.1	2.2	33.6	46.5	78.5	76.0	84.9	12.7	80.4	87.7	72.8	77.4
Slovenia	28.1	2.2	33.7	46.5	78.6	76.1	84.6	11.8	80.4	87.7	72.7	78.0
Solomon Islands	27.6	2.3	35.3	46.4	80.4	77.4	86.2	21.1	80.6	86.6	83.6	81.3
Somalia	28.1	2.2	33.6	46.5	81.3	77.0	82.4	9.8	82.9	87.5	72.8	77.3

South Africa	29.0	1.5	30.3	46.5	85.3	78.3	85.3	13.2	80.4	87.6	80.3	76.3
South Sudan	28.1	2.1	33.5	46.5	82.5	77.3	84.8	13.1	80.3	87.6	73.2	75.9
Spain	28.1	2.2	32.6	46.5	79.5	76.5	78.5	3.2	80.7	87.1	72.6	78.7
Sri Lanka	28.1	2.3	33.7	46.5	79.9	76.1	84.8	12.5	80.5	87.5	72.2	77.7
Sudan	28.5	2.4	33.1	46.5	80.3	75.8	84.8	12.4	80.4	87.4	76.1	79.2
Suriname	27.9	2.3	34.0	46.4	79.4	77.7	84.7	12.2	80.1	87.2	72.3	72.9
Sweden	28.1	2.3	34.1	46.8	70.6	65.3	84.6	11.9	80.4	87.4	72.3	77.8
Switzerland	28.1	2.3	34.1	46.6	75.1	74.5	84.1	9.6	80.5	87.4	72.3	77.8
Syrian Arab Republic	28.2	2.0	33.0	46.5	80.7	76.8	84.6	12.0	80.4	87.7	74.1	77.5
Taiwan (Province of China)	28.0	2.3	34.1	46.5	78.3	75.2	83.7	9.9	80.6	87.5	72.3	77.5
Tajikistan	28.8	2.1	32.5	100.0	73.0	72.2	84.7	12.1	80.9	86.9	75.4	80.1
Thailand	28.1	2.2	34.0	46.5	79.8	76.4	84.9	12.8	80.6	87.6	72.6	78.0
Timor-Leste	28.2	7.0	33.9	46.4	75.7	76.8	84.4	10.6	80.6	88.0	73.4	78.0
Togo	28.4	2.1	30.8	46.5	81.6	78.5	85.1	12.8	80.4	88.1	73.7	77.9
Tokelau	28.2	2.3	33.9	46.5	80.7	75.9	84.9	13.2	80.4	86.1	71.4	78.2
Tonga	28.1	2.1	34.4	46.5	81.6	75.9	84.9	12.9	80.5	87.2	74.7	58.4
Trinidad and Tobago	28.1	2.2	33.7	46.5	79.5	76.1	84.9	12.8	80.3	87.7	72.8	77.7
Tunisia	16.7	5.3	52.9	46.5	78.1	75.0	84.7	12.1	80.5	79.0	91.9	76.9
Turkey	28.1	2.2	33.4	46.5	79.4	76.3	84.8	12.3	80.4	87.7	73.0	77.5
Turkmenistan	27.9	17.1	33.7	48.1	85.5	89.3	84.8	12.8	80.4	87.5	74.9	76.9
Tuvalu	28.1	2.3	33.9	46.5	79.5	75.4	84.4	11.8	80.4	85.7	65.3	79.5
Uganda	27.9	2.3	34.1	46.5	79.1	76.0	84.4	11.5	80.4	87.0	72.4	76.4
Ukraine	28.1	2.3	34.1	46.5	76.0	75.2	87.8	9.2	89.4	87.6	72.4	77.7
United Arab Emirates	28.2	1.6	34.2	46.5	87.2	75.4	84.7	13.5	80.4	87.6	76.6	77.3
United Kingdom	28.0	2.3	33.9	46.5	78.1	75.8	84.7	12.0	80.4	87.6	72.5	77.7
United Republic of Tanzania	25.4	0.0	41.1	46.5	79.0	75.6	84.5	11.9	80.4	87.8	73.1	77.1
United States Virgin Islands	27.1	2.5	0.0	46.4	71.9	75.4	84.4	11.4	79.9	81.1	70.6	79.6
United States of America	29.8	0.9	32.6	46.4	95.6	82.9	86.8	21.3	79.7	86.5	72.0	78.5
Uruguay	28.1	2.2	33.4	46.5	78.9	76.2	84.8	12.2	80.4	87.8	73.1	77.6
Uzbekistan	29.7	1.8	29.9	47.3	97.8	68.0	97.3	100.0	80.3	86.3	72.6	79.2
Vanuatu	28.9	2.4	57.4	46.5	78.8	68.4	86.6	2.8	80.4	100.0	69.1	80.5
Venezuela (Bolivarian Republic of)	28.2	2.2	32.8	46.5	79.6	77.3	85.4	14.0	80.3	87.9	73.1	78.0
Viet Nam	26.4	2.3	49.2	46.5	79.1	76.8	84.7	12.0	80.4	87.6	73.3	77.1
Yemen	28.1	2.3	33.7	46.5	81.4	76.1	84.7	12.3	80.4	91.0	75.0	78.3
Zambia	25.9	2.2	33.6	46.4	81.4	77.7	84.5	12.5	84.3	87.6	71.4	78.0
Zimbabwe	27.9	2.3	34.6	0.0	0.0	0.0	84.4	11.1	80.4	87.3	72.4	77.5

ICH, intracerebral hemorrhage; SAH, subarachnoid hemorrhage; IS, ischemic stroke; QCI, quality of care index

Table S13 Values of age-standardized gender disparity ratio in burden of stroke, ICH, SAH, and IS at global and SDI levels

	**Age-Standardized GDR**		
**location**	**cause**	**1990**	**2021**
**Global**	Stroke	1.21	1.18
**Low SDI**	Stroke	1.28	1.23
**Low-middle SDI**	Stroke	1.16	1.17
**Middle SDI**	Stroke	1.40	1.31

**High-middle SDI**	Stroke	1.32	1.21
**High SDI**	Stroke	1.07	1.05
**Global**	ICH	1.39	1.29
**Low SDI**	ICH	1.37	1.24
**Low-middle SDI**	ICH	1.35	1.26
**Middle SDI**	ICH	1.54	1.44
**High-middle SDI**	ICH	1.49	1.36
**High SDI**	ICH	1.16	1.10
**Global**	SAH	1.03	1.00
**Low SDI**	SAH	1.01	0.96
**Low-middle SDI**	SAH	1.03	0.94
**Middle SDI**	SAH	0.90	1.01
**High-middle SDI**	SAH	1.14	1.03
**High SDI**	SAH	0.94	0.99
**Global**	IS	1.05	1.08
**Low SDI**	IS	1.10	1.14
**Low-middle SDI**	IS	1.02	1.09
**Middle SDI**	IS	1.14	1.15
**High-middle SDI**	IS	1.07	1.09
**High SDI**	IS	1.02	1.02

ICH, intracerebral hemorrhage; SAH, subarachnoid hemorrhage; IS, ischemic stroke; GDR, gender disparity ratio

Table S14 Values of age-standardized gender disparity ratio in burden of stroke, ICH, SAH, and IS at regional levels

	**Age-Standardized GDR**		
**location**	**cause**	**1990**	**2021**
Andean Latin America	Stroke	1.19	1.10
Australasia	Stroke	1.00	0.98
Caribbean	Stroke	1.20	1.22
Central Asia	Stroke	1.31	1.28
Central Europe	Stroke	1.23	1.16
Central Latin America	Stroke	1.15	1.16
Central Sub-Saharan Africa	Stroke	1.36	1.39
East Asia	Stroke	1.85	1.39
Eastern Europe	Stroke	1.35	1.26
Eastern Sub-Saharan Africa	Stroke	1.54	1.34
High-income Asia Pacific	Stroke	1.06	1.05
High-income North America	Stroke	1.05	1.00
North Africa and Middle East	Stroke	1.09	1.02
Oceania	Stroke	1.10	1.06
South Asia	Stroke	1.10	1.10
Southeast Asia	Stroke	1.28	1.32
Southern Latin America	Stroke	1.13	1.09
Southern Sub-Saharan Africa	Stroke	1.23	1.26
Tropical Latin America	Stroke	1.32	1.15
Western Europe	Stroke	1.08	1.02

Western Sub-Saharan Africa	Stroke	1.21	1.21
Andean Latin America	ICH	1.30	1.18
Australasia	ICH	1.11	1.06
Caribbean	ICH	1.43	1.41
Central Asia	ICH	1.32	1.28
Central Europe	ICH	1.47	1.42
Central Latin America	ICH	1.21	1.24
Central Sub-Saharan Africa	ICH	1.34	1.46
East Asia	ICH	1.95	1.56
Eastern Europe	ICH	1.30	1.34
Eastern Sub-Saharan Africa	ICH	1.76	1.53
High-income Asia Pacific	ICH	1.12	1.07
High-income North America	ICH	1.11	1.05
North Africa and Middle East	ICH	1.42	1.16
Oceania	ICH	1.43	1.32
South Asia	ICH	1.32	1.21
Southeast Asia	ICH	1.39	1.40
Southern Latin America	ICH	1.26	1.18
Southern Sub-Saharan Africa	ICH	1.20	1.32
Tropical Latin America	ICH	1.52	1.37
Western Europe	ICH	1.17	1.10
Western Sub-Saharan Africa	ICH	1.03	1.02
Andean Latin America	SAH	0.93	0.89
Australasia	SAH	0.94	1.00
Caribbean	SAH	0.94	0.93
Central Asia	SAH	1.01	1.03
Central Europe	SAH	0.99	1.00
Central Latin America	SAH	0.95	0.98
Central Sub-Saharan Africa	SAH	0.98	0.99
East Asia	SAH	1.05	1.08
Eastern Europe	SAH	1.09	1.07
Eastern Sub-Saharan Africa	SAH	1.08	1.02
High-income Asia Pacific	SAH	0.93	0.96
High-income North America	SAH	0.98	1.06
North Africa and Middle East	SAH	1.16	1.00
Oceania	SAH	0.74	0.77
South Asia	SAH	1.04	0.92
Southeast Asia	SAH	1.01	0.99
Southern Latin America	SAH	0.97	0.96
Southern Sub-Saharan Africa	SAH	0.98	0.96
Tropical Latin America	SAH	0.92	0.88
Western Europe	SAH	0.91	0.96
Western Sub-Saharan Africa	SAH	0.98	0.97
Andean Latin America	IS	1.10	1.07
Australasia	IS	0.95	0.96
Caribbean	IS	1.03	1.09
Central Asia	IS	1.19	1.19
Central Europe	IS	1.06	1.06

Central Latin America	IS	1.09	1.10
Central Sub-Saharan Africa	IS	1.13	1.15
East Asia	IS	1.19	1.19
Eastern Europe	IS	1.25	1.15
Eastern Sub-Saharan Africa	IS	1.08	1.10
High-income Asia Pacific	IS	0.99	1.03
High-income North America	IS	1.03	1.00
North Africa and Middle East	IS	0.93	0.96
Oceania	IS	0.96	0.97
South Asia	IS	0.95	1.02
Southeast Asia	IS	1.11	1.14
Southern Latin America	IS	1.01	1.03
Southern Sub-Saharan Africa	IS	1.15	1.14
Tropical Latin America	IS	1.16	1.09
Western Europe	IS	1.02	1.00
Western Sub-Saharan Africa	IS	1.31	1.31

ICH, intracerebral hemorrhage; SAH, subarachnoid hemorrhage; IS, ischemic stroke; GDR, gender disparity ratio

Table S15 Values of age-standardized gender disparity ratio in burden of stroke, ICH, SAH, and IS at national levels

		**Age-Standardized GDR**	
**cause**	**location**	**1990**	**2021**
Stroke	Afghanistan	1.90	1.01
Stroke	Albania	2.18	1.17
Stroke	Algeria	0.80	0.88
Stroke	American Samoa	1.22	1.14
Stroke	Andorra	0.96	0.97
Stroke	Angola	1.54	1.36
Stroke	Antigua and Barbuda	1.84	1.38
Stroke	Argentina	1.14	1.09
Stroke	Armenia	1.14	1.09
Stroke	Australia	0.99	0.98
Stroke	Austria	1.03	1.01
Stroke	Azerbaijan	1.44	1.16
Stroke	Bahamas	1.31	1.18
Stroke	Bahrain	1.33	1.10
Stroke	Bangladesh	1.06	1.03
Stroke	Barbados	1.25	1.16
Stroke	Belarus	1.08	1.20
Stroke	Belgium	1.10	1.04
Stroke	Belize	1.24	1.17
Stroke	Benin	1.04	1.11
Stroke	Bermuda	1.31	1.24
Stroke	Bhutan	0.95	1.04
Stroke	Bolivia (Plurinational State of)	1.23	1.10
Stroke	Bosnia and Herzegovina	1.02	1.05

Stroke	Botswana	1.33	1.20
Stroke	Brazil	1.33	1.15
Stroke	Brunei Darussalam	0.96	0.99
Stroke	Bulgaria	1.25	1.37
Stroke	Burkina Faso	1.57	1.51
Stroke	Burundi	2.20	1.43
Stroke	Cabo Verde	1.13	1.34
Stroke	Cambodia	1.05	1.29
Stroke	Cameroon	1.25	1.24
Stroke	Canada	1.04	0.99
Stroke	Central African Republic	2.19	2.07
Stroke	Chad	1.16	1.25
Stroke	Chile	1.11	1.08
Stroke	China	1.96	1.41
Stroke	Colombia	1.12	1.14
Stroke	Comoros	1.33	1.22
Stroke	Congo	1.34	1.17
Stroke	Cook Islands	1.30	1.15
Stroke	Costa Rica	1.12	1.13
Stroke	Coted'Ivoire	1.23	1.23
Stroke	Croatia	1.30	1.05
Stroke	Cuba	1.18	1.23
Stroke	Cyprus	0.94	1.03
Stroke	Czechia	1.22	1.06
Stroke	Democratic People's Republic of Korea	1.33	1.37
Stroke	Democratic Republic of the Congo	1.28	1.42
Stroke	Denmark	1.04	1.06
Stroke	Djibouti	1.40	1.32
Stroke	Dominica	2.06	1.46
Stroke	Dominican Republic	1.21	1.25
Stroke	Ecuador	1.19	1.13
Stroke	Egypt	0.27	0.64
Stroke	El Salvador	1.23	1.19
Stroke	Equatorial Guinea	1.81	1.21
Stroke	Eritrea	2.04	1.59
Stroke	Estonia	1.17	1.18
Stroke	Eswatini	1.87	1.40
Stroke	Ethiopia	1.63	1.27
Stroke	Fiji	1.31	1.24
Stroke	Finland	0.99	1.01
Stroke	France	1.10	1.01
Stroke	Gabon	1.41	1.28
Stroke	Gambia	1.23	1.26
Stroke	Georgia	1.71	1.56
Stroke	Germany	1.08	1.03
Stroke	Ghana	1.18	1.24
Stroke	Greece	0.93	0.99
Stroke	Greenland	0.97	1.06

Stroke	Grenada	2.12	1.34
Stroke	Guam	1.05	1.11
Stroke	Guatemala	1.15	1.17
Stroke	Guinea	0.98	1.09
Stroke	Guinea-Bissau	1.67	1.42
Stroke	Guyana	2.91	1.44
Stroke	Haiti	0.00	1.09
Stroke	Honduras	1.38	1.30
Stroke	Hungary	1.20	1.15
Stroke	Iceland	1.03	1.00
Stroke	India	1.11	1.10
Stroke	Indonesia	1.12	1.27
Stroke	Iran (Islamic Republic of)	1.12	0.99
Stroke	Iraq	1.48	1.39
Stroke	Ireland	1.04	1.04
Stroke	Israel	0.94	1.00
Stroke	Italy	1.12	1.05
Stroke	Jamaica	1.27	1.27
Stroke	Japan	1.07	1.06
Stroke	Jordan	0.87	0.92
Stroke	Kazakhstan	1.25	1.35
Stroke	Kenya	1.17	1.26
Stroke	Kiribati	1.42	1.43
Stroke	Kuwait	0.97	1.11
Stroke	Kyrgyzstan	1.44	1.45
Stroke	Lao People's Democratic Republic	1.21	1.22
Stroke	Latvia	1.24	1.08
Stroke	Lebanon	1.30	1.05
Stroke	Lesotho	2.61	3.09
Stroke	Liberia	1.04	1.07
Stroke	Libya	1.02	1.06
Stroke	Lithuania	1.16	1.16
Stroke	Luxembourg	1.14	1.08
Stroke	Madagascar	1.72	1.43
Stroke	Malawi	1.20	1.35
Stroke	Malaysia	1.10	1.05
Stroke	Maldives	0.91	1.07
Stroke	Mali	0.90	0.95
Stroke	Malta	1.09	1.03
Stroke	Marshall Islands	1.91	1.41
Stroke	Mauritania	1.02	1.04
Stroke	Mauritius	1.38	1.24
Stroke	Mexico	1.11	1.13
Stroke	Micronesia (Federated States of)	1.48	1.39
Stroke	Monaco	1.04	1.00
Stroke	Mongolia	2.03	1.47
Stroke	Montenegro	1.32	1.30
Stroke	Morocco	0.79	0.90

Stroke	Mozambique	1.68	2.20
Stroke	Myanmar	1.70	1.66
Stroke	Namibia	1.41	1.46
Stroke	Nauru	1.31	1.38
Stroke	Nepal	0.85	1.18
Stroke	Netherlands	1.05	1.03
Stroke	New Zealand	1.03	1.00
Stroke	Nicaragua	1.23	1.14
Stroke	Niger	1.19	1.17
Stroke	Nigeria	1.26	1.21
Stroke	Niue	1.30	1.27
Stroke	North Macedonia	1.19	1.03
Stroke	Northern Mariana Islands	1.01	1.15
Stroke	Norway	1.02	1.01
Stroke	Oman	1.34	1.16
Stroke	Pakistan	0.97	0.99
Stroke	Palau	1.55	1.22
Stroke	Palestine	1.63	1.29
Stroke	Panama	1.20	1.24
Stroke	Papua New Guinea	0.55	0.70
Stroke	Paraguay	1.11	1.15
Stroke	Peru	1.20	1.10
Stroke	Philippines	1.36	1.39
Stroke	Poland	1.45	1.17
Stroke	Portugal	1.16	1.06
Stroke	Puerto Rico	1.23	1.16
Stroke	Qatar	1.10	1.04
Stroke	Republic of Korea	1.05	1.03
Stroke	Republic of Moldova	1.16	1.17
Stroke	Romania	1.10	1.19
Stroke	Russian Federation	1.72	1.31
Stroke	Rwanda	2.08	1.49
Stroke	Saint Kitts and Nevis	1.71	1.39
Stroke	Saint Lucia	1.73	1.39
Stroke	Saint Vincent and the Grenadines	1.46	1.41
Stroke	Samoa	1.51	1.23
Stroke	San Marino	1.00	0.99
Stroke	Sao Tome and Principe	1.11	1.08
Stroke	Saudi Arabia	0.93	0.97
Stroke	Senegal	1.21	1.22
Stroke	Serbia	1.24	1.13
Stroke	Seychelles	1.38	1.14
Stroke	Sierra Leone	1.23	1.10
Stroke	Singapore	0.96	0.98
Stroke	Slovakia	1.11	1.09
Stroke	Slovenia	1.27	1.13
Stroke	Solomon Islands	1.30	1.28
Stroke	Somalia	2.12	1.70

Stroke	South Africa	1.19	1.22
Stroke	South Sudan	1.58	1.44
Stroke	Spain	1.09	1.00
Stroke	Sri Lanka	1.36	1.27
Stroke	Sudan	0.59	0.85
Stroke	Suriname	1.21	1.35
Stroke	Sweden	1.00	0.99
Stroke	Switzerland	1.04	1.00
Stroke	Syrian Arab Republic	0.98	0.96
Stroke	Taiwan (Province of China)	1.10	1.14
Stroke	Tajikistan	1.39	1.21
Stroke	Thailand	1.22	1.20
Stroke	Timor-Leste	0.89	1.19
Stroke	Togo	1.10	1.29
Stroke	Tokelau	1.69	1.32
Stroke	Tonga	1.15	1.11
Stroke	Trinidad and Tobago	1.26	1.24
Stroke	Tunisia	1.05	1.10
Stroke	Turkey	1.17	1.09
Stroke	Turkmenistan	1.28	1.49
Stroke	Tuvalu	1.78	1.31
Stroke	Uganda	1.66	1.39
Stroke	Ukraine	1.15	1.21
Stroke	United Arab Emirates	0.99	0.52
Stroke	United Kingdom	1.06	1.02
Stroke	United Republic of Tanzania	1.40	1.16
Stroke	United States of America	1.05	1.32
Stroke	United States Virgin Islands	1.41	1.00
Stroke	Uruguay	1.09	1.08
Stroke	Uzbekistan	1.25	1.23
Stroke	Vanuatu	1.53	1.35
Stroke	Venezuela (Bolivarian Republic of)	1.21	1.33
Stroke	Viet Nam	1.97	1.77
Stroke	Yemen	1.21	1.11
Stroke	Zambia	1.46	1.46
Stroke	Zimbabwe	1.31	1.53
SAH	Afghanistan	1.82	1.15
SAH	Albania	1.03	0.94
SAH	Algeria	0.94	0.91
SAH	American Samoa	0.84	0.82
SAH	Andorra	0.80	0.86
SAH	Angola	1.02	0.99
SAH	Antigua and Barbuda	1.16	1.00
SAH	Argentina	0.98	0.96
SAH	Armenia	0.99	1.05
SAH	Australia	0.96	1.00
SAH	Austria	0.92	0.95
SAH	Azerbaijan	1.00	0.97

SAH	Bahamas	1.01	1.02
SAH	Bahrain	0.97	0.97
SAH	Bangladesh	1.15	0.89
SAH	Barbados	1.06	1.00
SAH	Belarus	1.09	1.21
SAH	Belgium	0.91	0.97
SAH	Belize	1.05	0.98
SAH	Benin	0.90	0.92
SAH	Bermuda	1.12	1.11
SAH	Bhutan	0.87	0.89
SAH	Bolivia (Plurinational State of)	0.75	0.75
SAH	Bosnia and Herzegovina	0.99	0.97
SAH	Botswana	1.00	0.98
SAH	Brazil	0.92	0.88
SAH	Brunei Darussalam	0.78	0.89
SAH	Bulgaria	1.03	1.05
SAH	Burkina Faso	1.10	1.07
SAH	Burundi	1.19	1.06
SAH	Cabo Verde	1.02	1.01
SAH	Cambodia	0.91	0.94
SAH	Cameroon	0.99	0.98
SAH	Canada	1.00	1.01
SAH	Central African Republic	1.10	1.09
SAH	Chad	0.97	0.94
SAH	Chile	0.92	0.98
SAH	China	0.9923	1.08
SAH	Colombia	0.94	0.96
SAH	Comoros	0.99	0.98
SAH	Congo	0.96	0.90
SAH	Cook Islands	0.87	0.92
SAH	Costa Rica	0.95	1.00
SAH	Coted'Ivoire	1.00	0.97
SAH	Croatia	0.99	1.02
SAH	Cuba	0.96	0.98
SAH	Cyprus	0.82	0.91
SAH	Czechia	0.98	0.98
SAH	Democratic People's Republic of Korea	1.04	1.04
SAH	Democratic Republic of the Congo	0.97	0.99
SAH	Denmark	0.88	0.95
SAH	Djibouti	1.06	1.03
SAH	Dominica	1.16	1.09
SAH	Dominican Republic	0.99	0.96
SAH	Ecuador	0.97	0.96
SAH	Egypt	1.91	1.02
SAH	El Salvador	0.99	0.97
SAH	Equatorial Guinea	1.09	0.94
SAH	Eritrea	1.24	1.09
SAH	Estonia	1.24	1.16

SAH	Eswatini	1.05	1.00
SAH	Ethiopia	1.08	1.01
SAH	Fiji	0.90	0.87
SAH	Finland	0.90	0.94
SAH	France	0.94	0.97
SAH	Gabon	1.03	1.00
SAH	Gambia	0.98	0.94
SAH	Georgia	1.04	1.02
SAH	Germany	0.91	0.93
SAH	Ghana	0.96	0.96
SAH	Greece	0.92	0.98
SAH	Greenland	0.02	0.91
SAH	Grenada	1.03	1.00
SAH	Guam	0.81	0.93
SAH	Guatemala	0.97	0.98
SAH	Guinea	0.89	0.90
SAH	Guinea-Bissau	1.04	0.94
SAH	Guyana	1.18	1.13
SAH	Haiti	0.28	0.59
SAH	Honduras	0.90	0.62
SAH	Hungary	1.01	0.99
SAH	Iceland	0.86	0.93
SAH	India	1.03	0.92
SAH	Indonesia	0.90	0.90
SAH	Iran (Islamic Republic of)	1.02	0.99
SAH	Iraq	1.10	1.02
SAH	Ireland	0.90	0.96
SAH	Israel	0.93	1.00
SAH	Italy	0.91	0.98
SAH	Jamaica	0.94	0.94
SAH	Japan	0.94	0.97
SAH	Jordan	0.97	0.98
SAH	Kazakhstan	1.01	0.98
SAH	Kenya	0.99	0.99
SAH	Kiribati	1.17	1.04
SAH	Kuwait	0.93	1.02
SAH	Kyrgyzstan	1.17	1.13
SAH	Lao People's Democratic Republic	0.83	0.93
SAH	Latvia	1.15	1.12
SAH	Lebanon	1.42	1.06
SAH	Lesotho	1.16	1.10
SAH	Liberia	0.90	0.90
SAH	Libya	0.95	0.90
SAH	Lithuania	1.15	1.14
SAH	Luxembourg	0.86	0.95
SAH	Madagascar	1.11	0.99
SAH	Malawi	0.98	1.01
SAH	Malaysia	0.96	0.92

SAH	Maldives	0.86	0.93
SAH	Mali	0.82	0.83
SAH	Malta	0.91	0.96
SAH	Marshall Islands	1.02	0.90
SAH	Mauritania	0.89	0.89
SAH	Mauritius	1.25	1.10
SAH	Mexico	0.94	1.01
SAH	Micronesia (Federated States of)	0.96	0.92
SAH	Monaco	0.84	0.89
SAH	Mongolia	1.28	1.02
SAH	Montenegro	0.94	0.90
SAH	Morocco	0.90	0.89
SAH	Mozambique	1.14	1.23
SAH	Myanmar	1.08	1.06
SAH	Namibia	0.99	1.01
SAH	Nauru	0.75	0.76
SAH	Nepal	0.91	1.01
SAH	Netherlands	0.93	0.96
SAH	New Zealand	0.87	0.97
SAH	Nicaragua	0.95	0.95
SAH	Niger	0.97	0.96
SAH	Nigeria	1.00	0.99
SAH	Niue	0.91	0.85
SAH	North Macedonia	0.81	0.77
SAH	Northern Mariana Islands	0.61	0.80
SAH	Norway	0.85	0.98
SAH	Oman	1.04	0.98
SAH	Pakistan	0.93	0.87
SAH	Palau	0.96	0.89
SAH	Palestine	1.00	1.00
SAH	Panama	0.92	0.99
SAH	Papua New Guinea	0.51	0.64
SAH	Paraguay	0.81	0.81
SAH	Peru	0.96	0.89
SAH	Philippines	1.02	0.99
SAH	Poland	1.08	1.06
SAH	Portugal	0.91	0.93
SAH	Puerto Rico	1.00	1.02
SAH	Qatar	2.00	1.10
SAH	Republic of Korea	0.93	0.94
SAH	Republic of Moldova	1.13	1.09
SAH	Romania	0.91	1.01
SAH	Russian Federation	1.11	1.06
SAH	Rwanda	1.15	1.06
SAH	Saint Kitts and Nevis	0.96	1.02
SAH	Saint Lucia	0.91	0.97
SAH	Saint Vincent and the Grenadines	0.93	0.97
SAH	Samoa	0.93	0.85

SAH	San Marino	0.94	0.94
SAH	Sao Tome and Principe	0.94	0.91
SAH	Saudi Arabia	0.95	0.95
SAH	Senegal	0.96	0.94
SAH	Serbia	0.81	0.85
SAH	Seychelles	1.05	0.98
SAH	Sierra Leone	0.96	0.90
SAH	Singapore	0.90	0.93
SAH	Slovakia	0.97	0.98
SAH	Slovenia	0.99	0.99
SAH	Solomon Islands	0.91	0.92
SAH	Somalia	1.23	1.12
SAH	South Africa	0.96	0.95
SAH	South Sudan	1.05	1.03
SAH	Spain	0.99	1.00
SAH	Sri Lanka	1.03	1.01
SAH	Sudan	0.92	0.87
SAH	Suriname	0.98	1.00
SAH	Sweden	0.83	0.91
SAH	Switzerland	0.87	0.95
SAH	Syrian Arab Republic	0.99	0.97
SAH	Taiwan (Province of China)	0.94	1.01
SAH	Tajikistan	0.87	0.90
SAH	Thailand	1.23	1.13
SAH	Timor-Leste	0.82	0.91
SAH	Togo	0.93	0.96
SAH	Tokelau	0.84	0.82
SAH	Tonga	0.85	0.85
SAH	Trinidad and Tobago	1.02	1.07
SAH	Tunisia	1.02	0.99
SAH	Turkey	1.36	1.09
SAH	Turkmenistan	1.01	1.15
SAH	Tuvalu	0.87	0.86
SAH	Uganda	1.16	1.07
SAH	Ukraine	1.01	1.06
SAH	United Arab Emirates	0.84	0.54
SAH	United Kingdom	0.85	0.94
SAH	United Republic of Tanzania	1.07	0.98
SAH	United States of America	0.97	1.10
SAH	United States Virgin Islands	1.06	1.07
SAH	Uruguay	0.96	0.96
SAH	Uzbekistan	1.02	1.12
SAH	Vanuatu	0.95	0.92
SAH	Venezuela (Bolivarian Republic of)	0.96	1.00
SAH	Viet Nam	1.18	1.09
SAH	Yemen	1.43	1.08
SAH	Zambia	1.00	0.99
SAH	Zimbabwe	0.95	0.94

IS	Afghanistan	0.87	0.91
IS	Albania	1.26	1.04
IS	Algeria	0.75	0.86
IS	American Samoa	1.04	1.01
IS	Andorra	0.95	0.97
IS	Angola	1.18	1.17
IS	Antigua and Barbuda	1.22	1.12
IS	Argentina	1.01	1.02
IS	Armenia	1.09	1.03
IS	Australia	0.95	0.96
IS	Austria	0.95	1.01
IS	Azerbaijan	1.23	1.10
IS	Bahamas	1.04	1.02
IS	Bahrain	1.11	1.01
IS	Bangladesh	0.78	0.90
IS	Barbados	1.00	1.02
IS	Belarus	1.01	1.11
IS	Belgium	1.03	1.01
IS	Belize	1.06	1.04
IS	Benin	1.26	1.28
IS	Bermuda	1.20	1.16
IS	Bhutan	0.91	1.01
IS	Bolivia (Plurinational State of)	1.14	1.07
IS	Bosnia and Herzegovina	0.93	0.99
IS	Botswana	1.18	1.15
IS	Brazil	1.16	1.09
IS	Brunei Darussalam	0.95	0.96
IS	Bulgaria	1.02	1.22
IS	Burkina Faso	1.41	1.41
IS	Burundi	1.07	1.07
IS	Cabo Verde	1.21	1.46
IS	Cambodia	1.05	1.11
IS	Cameroon	1.26	1.32
IS	Canada	1.01	0.99
IS	Central African Republic	1.18	1.19
IS	Chad	1.27	1.38
IS	Chile	1.00	1.04
IS	China	1.20	1.20
IS	Colombia	1.08	1.10
IS	Comoros	1.10	1.10
IS	Congo	1.15	1.11
IS	Cook Islands	1.09	1.04
IS	Costa Rica	1.08	1.08
IS	Coted'Ivoire	1.28	1.34
IS	Croatia	1.06	0.96
IS	Cuba	1.07	1.14
IS	Cyprus	0.60	0.95
IS	Czechia	1.07	1.02

IS	Democratic People's Republic of Korea	1.08	1.12
IS	Democratic Republic of the Congo	1.11	1.15
IS	Denmark	1.00	1.02
IS	Djibouti	1.11	1.16
IS	Dominica	1.31	1.12
IS	Dominican Republic	1.04	1.10
IS	Ecuador	1.09	1.08
IS	Egypt	0.00	0.47
IS	El Salvador	1.15	1.12
IS	Equatorial Guinea	1.21	1.13
IS	Eritrea	1.06	1.13
IS	Estonia	1.02	1.12
IS	Eswatini	1.25	1.13
IS	Ethiopia	1.03	1.05
IS	Fiji	1.11	1.09
IS	Finland	0.95	1.00
IS	France	1.02	1.00
IS	Gabon	1.13	1.13
IS	Gambia	1.32	1.40
IS	Georgia	1.38	1.41
IS	Germany	1.04	1.02
IS	Ghana	1.32	1.33
IS	Greece	0.85	0.92
IS	Greenland	0.96	1.01
IS	Grenada	1.60	1.16
IS	Guam	1.00	1.07
IS	Guatemala	1.11	1.11
IS	Guinea	1.20	1.29
IS	Guinea-Bissau	1.63	1.58
IS	Guyana	1.33	1.13
IS	Haiti	0.61	0.79
IS	Honduras	1.28	1.24
IS	Hungary	1.10	1.09
IS	Iceland	1.01	0.99
IS	India	0.96	1.03
IS	Indonesia	1.06	1.15
IS	Iran (Islamic Republic of)	1.03	0.94
IS	Iraq	1.22	1.28
IS	Ireland	0.98	1.01
IS	Israel	0.91	0.97
IS	Italy	1.02	0.99
IS	Jamaica	1.00	1.06
IS	Japan	1.00	1.04
IS	Jordan	0.82	0.90
IS	Kazakhstan	1.19	1.23
IS	Kenya	1.03	1.08
IS	Kiribati	1.18	1.20
IS	Kuwait	0.94	1.08

IS	Kyrgyzstan	1.32	1.37
IS	Lao People's Democratic Republic	1.03	1.13
IS	Latvia	1.10	0.99
IS	Lebanon	0.97	1.01
IS	Lesotho	1.33	1.32
IS	Liberia	1.26	1.30
IS	Libya	0.99	1.06
IS	Lithuania	1.10	1.10
IS	Luxembourg	1.04	1.04
IS	Madagascar	1.16	1.13
IS	Malawi	1.06	1.12
IS	Malaysia	1.01	0.98
IS	Maldives	0.96	1.05
IS	Mali	1.09	1.14
IS	Malta	1.04	0.99
IS	Marshall Islands	1.11	1.05
IS	Mauritania	1.25	1.23
IS	Mauritius	1.17	1.11
IS	Mexico	1.07	1.08
IS	Micronesia (Federated States of)	1.06	1.07
IS	Monaco	0.96	0.96
IS	Mongolia	1.15	1.15
IS	Montenegro	1.08	1.07
IS	Morocco	0.78	0.87
IS	Mozambique	1.15	1.28
IS	Myanmar	1.21	1.24
IS	Namibia	1.25	1.24
IS	Nauru	1.08	1.11
IS	Nepal	0.80	1.06
IS	Netherlands	1.01	1.00
IS	New Zealand	0.99	0.98
IS	Nicaragua	1.17	1.09
IS	Niger	1.20	1.25
IS	Nigeria	1.35	1.31
IS	Niue	1.08	1.09
IS	North Macedonia	1.05	0.85
IS	Northern Mariana Islands	0.99	1.05
IS	Norway	0.98	1.00
IS	Oman	1.21	1.12
IS	Pakistan	0.93	0.98
IS	Palau	1.13	1.01
IS	Palestine	1.38	1.18
IS	Panama	1.11	1.13
IS	Papua New Guinea	0.83	0.86
IS	Paraguay	1.06	1.09
IS	Peru	1.10	1.07
IS	Philippines	1.05	1.13
IS	Poland	1.22	1.07

IS	Portugal	1.00	0.97
IS	Puerto Rico	1.12	1.10
IS	Qatar	1.00	1.00
IS	Republic of Korea	0.98	1.00
IS	Republic of Moldova	1.02	1.08
IS	Romania	0.98	1.06
IS	Russian Federation	1.71	1.19
IS	Rwanda	1.06	1.10
IS	Saint Kitts and Nevis	1.30	1.09
IS	Saint Lucia	1.11	1.11
IS	Saint Vincent and the Grenadines	1.08	1.12
IS	Samoa	1.09	1.07
IS	San Marino	0.98	0.98
IS	Sao Tome and Principe	1.21	1.17
IS	Saudi Arabia	0.83	0.89
IS	Senegal	1.31	1.35
IS	Serbia	0.97	0.98
IS	Seychelles	1.22	1.08
IS	Sierra Leone	1.37	1.30
IS	Singapore	0.92	0.97
IS	Slovakia	1.03	1.04
IS	Slovenia	1.14	1.07
IS	Solomon Islands	0.99	1.00
IS	Somalia	1.05	1.07
IS	South Africa	1.14	1.12
IS	South Sudan	1.09	1.09
IS	Spain	1.00	0.98
IS	Sri Lanka	1.13	1.13
IS	Sudan	0.67	0.87
IS	Suriname	1.02	1.09
IS	Sweden	0.97	0.99
IS	Switzerland	1.00	0.99
IS	Syrian Arab Republic	0.89	0.87
IS	Taiwan (Province of China)	1.01	1.06
IS	Tajikistan	1.37	1.18
IS	Thailand	1.10	1.07
IS	Timor-Leste	1.00	1.12
IS	Togo	1.27	1.42
IS	Tokelau	1.13	1.10
IS	Tonga	1.09	1.06
IS	Trinidad and Tobago	1.19	1.14
IS	Tunisia	0.95	1.06
IS	Turkey	1.07	1.02
IS	Turkmenistan	1.19	1.25
IS	Tuvalu	1.11	1.08
IS	Uganda	1.09	1.09
IS	Ukraine	1.06	1.12
IS	United Arab Emirates	0.95	0.49

IS	United Kingdom	1.03	1.01
IS	United Republic of Tanzania	1.07	1.09
IS	United States of America	1.03	1.16
IS	United States Virgin Islands	1.13	1.00
IS	Uruguay	1.01	1.02
IS	Uzbekistan	1.16	1.17
IS	Vanuatu	1.12	1.10
IS	Venezuela (Bolivarian Republic of)	1.09	1.15
IS	Viet Nam	1.28	1.29
IS	Yemen	0.82	1.04
IS	Zambia	1.06	1.11
IS	Zimbabwe	1.17	1.22
ICH	Afghanistan	3.34	1.50
ICH	Albania	2.69	1.52
ICH	Algeria	1.05	0.99
ICH	American Samoa	1.31	1.28
ICH	Andorra	1.03	1.00
ICH	Angola	1.60	1.40
ICH	Antigua and Barbuda	1.90	1.62
ICH	Argentina	1.26	1.20
ICH	Armenia	1.26	1.24
ICH	Australia	1.10	1.06
ICH	Austria	1.06	1.02
ICH	Azerbaijan	1.43	1.23
ICH	Bahamas	1.53	1.34
ICH	Bahrain	1.49	1.20
ICH	Bangladesh	1.43	1.26
ICH	Barbados	1.45	1.35
ICH	Belarus	1.15	1.29
ICH	Belgium	1.20	1.15
ICH	Belize	1.39	1.30
ICH	Benin	0.88	0.93
ICH	Bermuda	1.34	1.28
ICH	Bhutan	1.20	1.14
ICH	Bolivia (Plurinational State of)	1.47	1.29
ICH	Bosnia and Herzegovina	1.33	1.28
ICH	Botswana	1.17	1.15
ICH	Brazil	1.52	1.36
ICH	Brunei Darussalam	1.08	1.09
ICH	Bulgaria	1.51	1.42
ICH	Burkina Faso	1.32	1.26
ICH	Burundi	2.83	1.91
ICH	Cabo Verde	1.00	1.07
ICH	Cambodia	1.32	1.41
ICH	Cameroon	1.12	1.06
ICH	Canada	1.08	1.01
ICH	Central African Republic	1.97	2.16
ICH	Chad	1.01	1.02

ICH	Chile	1.25	1.12
ICH	China	1.99	1.58
ICH	Colombia	1.22	1.19
ICH	Comoros	1.42	1.31
ICH	Congo	1.27	1.14
ICH	Cook Islands	1.42	1.27
ICH	Costa Rica	1.14	1.15
ICH	Coted'Ivoire	1.07	1.02
ICH	Croatia	1.68	1.44
ICH	Cuba	1.27	1.34
ICH	Cyprus	1.25	1.09
ICH	Czechia	1.42	1.19
ICH	Democratic People's Republic of Korea	1.62	1.80
ICH	Democratic Republic of the Congo	1.25	1.49
ICH	Denmark	1.14	1.13
ICH	Djibouti	1.48	1.35
ICH	Dominica	2.28	1.81
ICH	Dominican Republic	1.45	1.38
ICH	Ecuador	1.31	1.20
ICH	Egypt	1.021	1.22
ICH	El Salvador	1.24	1.27
ICH	Equatorial Guinea	1.76	1.18
ICH	Eritrea	2.25	1.92
ICH	Estonia	1.26	1.30
ICH	Eswatini	2.00	1.40
ICH	Ethiopia	1.71	1.39
ICH	Fiji	1.62	1.63
ICH	Finland	1.08	1.03
ICH	France	1.25	1.07
ICH	Gabon	1.43	1.29
ICH	Gambia	1.00	1.00
ICH	Georgia	1.38	1.35
ICH	Germany	1.17	1.07
ICH	Ghana	0.98	1.00
ICH	Greece	1.12	1.13
ICH	Greenland	1.22	1.20
ICH	Grenada	1.84	1.49
ICH	Guam	1.16	1.16
ICH	Guatemala	1.16	1.20
ICH	Guinea	0.90	0.94
ICH	Guinea-Bissau	1.18	1.05
ICH	Guyana	2.16	1.68
ICH	Haiti	3.08	2.44
ICH	Honduras	1.56	1.85
ICH	Hungary	1.36	1.29
ICH	Iceland	1.08	1.05
ICH	India	1.33	1.21
ICH	Indonesia	1.28	1.38

ICH	Iran (Islamic Republic of)	1.21	1.09
ICH	Iraq	1.46	1.31
ICH	Ireland	1.08	1.04
ICH	Israel	1.15	1.14
ICH	Italy	1.22	1.15
ICH	Jamaica	1.43	1.45
ICH	Japan	1.12	1.08
ICH	Jordan	1.14	1.08
ICH	Kazakhstan	1.23	1.40
ICH	Kenya	1.26	1.32
ICH	Kiribati	1.23	1.28
ICH	Kuwait	1.10	1.13
ICH	Kyrgyzstan	1.26	1.30
ICH	Lao People's Democratic Republic	1.60	1.33
ICH	Latvia	1.39	1.37
ICH	Lebanon	2.44	1.18
ICH	Lesotho	2.67	3.09
ICH	Liberia	0.91	0.89
ICH	Libya	1.10	1.07
ICH	Lithuania	1.18	1.23
ICH	Luxembourg	1.25	1.17
ICH	Madagascar	1.69	1.58
ICH	Malawi	1.40	1.76
ICH	Malaysia	1.19	1.14
ICH	Maldives	1.08	1.06
ICH	Mali	0.86	0.83
ICH	Malta	1.12	1.05
ICH	Marshall Islands	1.84	1.53
ICH	Mauritania	0.82	0.86
ICH	Mauritius	1.45	1.27
ICH	Mexico	1.16	1.19
ICH	Micronesia (Federated States of)	1.53	1.47
ICH	Monaco	1.21	1.09
ICH	Mongolia	1.84	1.42
ICH	Montenegro	1.67	2.42
ICH	Morocco	1.07	1.05
ICH	Mozambique	2.03	2.89
ICH	Myanmar	1.59	1.62
ICH	Namibia	1.28	1.51
ICH	Nauru	1.44	1.61
ICH	Nepal	1.24	1.31
ICH	Netherlands	1.16	1.08
ICH	New Zealand	1.16	1.11
ICH	Nicaragua	1.28	1.21
ICH	Niger	1.09	1.04
ICH	Nigeria	1.05	1.03
ICH	Niue	1.42	1.45
ICH	North Macedonia	1.50	1.38

ICH	Northern Mariana Islands	1.09	1.20
ICH	Norway	1.18	1.06
ICH	Oman	1.47	1.20
ICH	Pakistan	1.13	1.11
ICH	Palau	1.59	1.37
ICH	Palestine	1.55	1.32
ICH	Panama	1.27	1.30
ICH	Papua New Guinea	1.06	1.01
ICH	Paraguay	1.33	1.41
ICH	Peru	1.28	1.16
ICH	Philippines	1.74	1.46
ICH	Poland	1.43	1.39
ICH	Portugal	1.30	1.22
ICH	Puerto Rico	1.23	1.19
ICH	Qatar	1.35	1.09
ICH	Republic of Korea	1.22	1.07
ICH	Republic of Moldova	1.39	1.36
ICH	Romania	1.41	1.63
ICH	Russian Federation	1.33	1.37
ICH	Rwanda	2.61	2.10
ICH	Saint Kitts and Nevis	1.65	1.59
ICH	Saint Lucia	1.80	1.59
ICH	Saint Vincent and the Grenadines	1.97	1.58
ICH	Samoa	1.74	1.43
ICH	San Marino	1.07	1.01
ICH	Sao Tome and Principe	0.97	0.97
ICH	Saudi Arabia	1.40	1.25
ICH	Senegal	1.00	0.97
ICH	Serbia	1.52	1.51
ICH	Seychelles	1.32	1.16
ICH	Sierra Leone	1.00	0.93
ICH	Singapore	1.05	1.00
ICH	Slovakia	1.58	1.35
ICH	Slovenia	1.35	1.21
ICH	Solomon Islands	1.38	1.38
ICH	Somalia	2.81	2.73
ICH	South Africa	1.14	1.28
ICH	South Sudan	1.96	1.99
ICH	Spain	1.15	1.14
ICH	Sri Lanka	1.58	1.42
ICH	Sudan	1.39	1.02
ICH	Suriname	1.45	1.58
ICH	Sweden	1.06	1.00
ICH	Switzerland	1.09	1.02
ICH	Syrian Arab Republic	1.18	1.17
ICH	Taiwan (Province of China)	1.26	1.18
ICH	Tajikistan	1.29	1.21
ICH	Thailand	1.19	1.21

ICH	Timor-Leste	1.21	1.37
ICH	Togo	0.94	1.03
ICH	Tokelau	2.01	1.61
ICH	Tonga	1.27	1.22
ICH	Trinidad and Tobago	1.46	1.42
ICH	Tunisia	1.24	1.15
ICH	Turkey	1.25	1.17
ICH	Turkmenistan	1.25	1.52
ICH	Tuvalu	1.81	1.45
ICH	Uganda	2.36	1.70
ICH	Ukraine	1.26	1.30
ICH	United Arab Emirates	1.19	0.79
ICH	United Kingdom	1.10	1.08
ICH	United Republic of Tanzania	1.65	1.27
ICH	United States of America	1.11	1.41
ICH	United States Virgin Islands	1.58	1.05
ICH	Uruguay	1.26	1.20
ICH	Uzbekistan	1.32	1.21
ICH	Vanuatu	1.66	1.45
ICH	Venezuela (Bolivarian Republic of)	1.32	1.47
ICH	Viet Nam	1.71	1.84
ICH	Yemen	2.52	1.36
ICH	Zambia	1.62	1.54
ICH	Zimbabwe	1.35	3.16

ICH, intracerebral hemorrhage; SAH, subarachnoid hemorrhage; IS, ischemic stroke; GDR, gender disparity ratio

Table S16 The number of countries for GDR values near one for stroke, ICH, SAH, and IS in 1990 and 2021

GDR range	year	stroke	ICH	SAH	IS
**<0.95**	1990	15	6	84	24
	2021	8	7	76	16
**[0.95,1.05]**	1990	33	10	74	61
	2021	42	22	91	60
**>1.05**	1990	156	188	46	119
	2021	154	175	37	128

ICH, intracerebral hemorrhage; SAH, subarachnoid hemorrhage; IS, ischemic stroke; GDR, gender disparity ratio

Table S17 GDR values by age group and SDI regions for stroke, ICH, SAH, and IS in 1990 and 2021

		GDR							
		stroke		ICH		SAH		IS	
location	age	1990	2021	1990	2021	1990	2021	1990	2021
Global	<5	1.12	1.05	1.06	1.01	1.08	1.04	0.99	1.04
High SDI	<5	1.02	1.00	1.03	1.00	1.03	1.02	1.00	1.00
High-middle SDI	<5	1.05	1.00	1.04	1.00	1.03	1.01	0.97	1.00

Low SDI	<5	1.19	1.11	0.98	1.00	1.10	1.07	1.13	1.13
Low-middle SDI	<5	1.29	1.07	1.13	1.01	1.29	1.07	0.94	1.04
Middle SDI	<5	1.08	1.02	1.06	1.01	1.02	1.02	0.98	1.01
Global	5 to 9	1.00	1.01	1.00	1.00	0.88	0.98	1.09	1.11
High SDI	5 to 9	0.99	1.00	1.00	0.99	0.95	1.00	1.03	1.01
High-middle SDI	5 to 9	1.01	1.00	1.01	1.00	0.91	0.99	1.06	1.04
Low SDI	5 to 9	1.01	1.04	0.98	1.00	0.95	1.00	1.19	1.27
Low-middle SDI	5 to 9	0.97	1.01	0.98	0.99	0.86	0.96	1.10	1.14
Middle SDI	5 to 9	1.00	1.01	1.01	1.00	0.83	0.97	1.09	1.06
Global	10 to 14	0.98	0.99	0.96	0.96	1.18	1.10	1.11	1.12
High SDI	10 to 14	0.98	0.99	0.99	1.00	1.13	1.05	1.08	1.03
High-middle SDI	10 to 14	1.01	1.01	0.99	1.00	1.19	1.05	1.15	1.09
Low SDI	10 to 14	0.90	0.94	0.84	0.88	1.06	1.05	1.07	1.13
Low-middle SDI	10 to 14	0.96	1.00	0.92	0.95	1.28	1.15	1.08	1.17
Middle SDI	10 to 14	0.99	1.02	0.98	0.99	1.19	1.11	1.14	1.12
Global	15 to 19	1.23	1.15	1.04	1.00	1.31	1.11	1.14	1.12
High SDI	15 to 19	1.09	1.05	1.04	1.01	1.17	1.04	1.10	1.03
High-middle SDI	15 to 19	1.35	1.17	1.14	1.08	1.47	1.10	1.33	1.16
Low SDI	15 to 19	1.13	1.08	0.83	0.82	1.11	1.07	1.02	1.08
Low-middle SDI	15 to 19	1.12	1.14	0.91	0.96	1.17	1.10	0.98	1.11
Middle SDI	15 to 19	1.31	1.23	1.09	1.10	1.41	1.15	1.20	1.21
Global	20 to 24	0.99	1.02	0.86	0.89	0.99	1.00	1.23	1.26
High SDI	20 to 24	0.99	1.01	0.93	0.97	0.99	1.00	1.12	1.08
High-middle SDI	20 to 24	1.05	1.05	0.94	0.99	1.03	1.01	1.42	1.31
Low SDI	20 to 24	0.91	0.95	0.68	0.73	0.95	0.97	1.10	1.19
Low-middle SDI	20 to 24	0.94	1.01	0.76	0.86	0.98	1.01	1.14	1.31
Middle SDI	20 to 24	1.02	1.05	0.89	0.95	0.98	1.01	1.29	1.35
Global	25 to 29	1.10	1.12	1.09	1.13	1.06	1.06	1.08	1.13
High SDI	25 to 29	1.03	1.03	1.04	1.04	1.02	1.03	1.04	1.04
High-middle SDI	25 to 29	1.16	1.15	1.15	1.20	1.10	1.05	1.19	1.19
Low SDI	25 to 29	0.96	1.05	0.89	0.98	0.98	1.03	0.98	1.06
Low-middle SDI	25 to 29	1.08	1.12	1.09	1.12	1.08	1.07	0.99	1.13
Middle SDI	25 to 29	1.14	1.18	1.13	1.21	1.05	1.08	1.12	1.20
Global	30 to 34	1.18	1.21	1.29	1.33	1.19	1.15	1.15	1.20
High SDI	30 to 34	1.07	1.07	1.14	1.14	1.13	1.07	1.07	1.07
High-middle SDI	30 to 34	1.26	1.27	1.33	1.40	1.23	1.14	1.33	1.31
Low SDI	30 to 34	1.01	1.10	1.07	1.16	1.06	1.08	1.00	1.10
Low-middle SDI	30 to 34	1.22	1.23	1.44	1.38	1.29	1.21	1.06	1.18
Middle SDI	30 to 34	1.24	1.29	1.35	1.43	1.18	1.17	1.19	1.27
Global	35 to 39	1.42	1.35	1.28	1.27	1.14	1.12	1.21	1.25
High SDI	35 to 39	1.20	1.14	1.19	1.18	1.15	1.09	1.12	1.09
High-middle SDI	35 to 39	1.58	1.48	1.35	1.38	1.20	1.16	1.52	1.42
Low SDI	35 to 39	1.15	1.22	1.10	1.14	1.03	1.05	1.03	1.17
Low-middle SDI	35 to 39	1.35	1.31	1.24	1.22	1.12	1.08	1.04	1.19
Middle SDI	35 to 39	1.63	1.49	1.35	1.36	1.14	1.14	1.28	1.33
Global	40 to 44	1.33	1.29	1.45	1.40	1.03	1.05	1.43	1.35
High SDI	40 to 44	1.16	1.12	1.27	1.22	1.02	1.04	1.18	1.12
High-middle SDI	40 to 44	1.50	1.39	1.53	1.46	1.09	1.09	2.33	1.68

Low SDI	40 to 44	1.23	1.26	1.42	1.42	1.02	1.03	1.10	1.23
Low-middle SDI	40 to 44	1.24	1.24	1.44	1.38	1.02	1.00	1.14	1.24
Middle SDI	40 to 44	1.48	1.38	1.53	1.47	1.02	1.05	1.69	1.48
Global	45 to 49	1.27	1.21	1.41	1.32	1.28	1.15	1.19	1.17
High SDI	45 to 49	1.12	1.08	1.26	1.18	1.20	1.13	1.08	1.05
High-middle SDI	45 to 49	1.38	1.27	1.44	1.36	1.34	1.18	1.51	1.29
Low SDI	45 to 49	1.29	1.23	1.47	1.39	1.21	1.15	1.06	1.15
Low-middle SDI	45 to 49	1.19	1.17	1.37	1.30	1.23	1.12	1.01	1.11
Middle SDI	45 to 49	1.42	1.28	1.48	1.37	1.36	1.17	1.34	1.22
Global	50 to 54	1.30	1.21	1.21	1.17	1.05	1.03	1.19	1.13
High SDI	50 to 54	1.10	1.06	1.13	1.09	0.99	1.01	1.02	1.00
High-middle SDI	50 to 54	1.36	1.23	1.21	1.17	1.08	1.05	1.49	1.21
Low SDI	50 to 54	1.37	1.25	1.23	1.17	1.05	1.01	1.08	1.14
Low-middle SDI	50 to 54	1.33	1.21	1.24	1.17	1.09	0.98	1.04	1.12
Middle SDI	50 to 54	1.52	1.30	1.25	1.21	1.01	1.04	1.41	1.20
Global	55 to 59	1.19	1.13	1.21	1.14	1.13	1.02	1.15	1.12
High SDI	55 to 59	1.06	1.04	1.10	1.04	1.00	1.00	1.01	1.00
High-middle SDI	55 to 59	1.27	1.16	1.23	1.14	1.23	1.05	1.45	1.20
Low SDI	55 to 59	1.19	1.13	1.18	1.11	1.05	0.95	1.05	1.13
Low-middle SDI	55 to 59	1.12	1.11	1.18	1.14	1.10	0.95	0.99	1.12
Middle SDI	55 to 59	1.37	1.21	1.28	1.19	1.21	1.03	1.35	1.17
Global	60 to 64	1.11	1.09	1.33	1.22	1.03	1.00	0.90	0.95
High SDI	60 to 64	1.04	1.04	1.14	1.07	0.94	1.00	0.90	0.95
High-middle SDI	60 to 64	1.18	1.13	1.35	1.24	1.15	1.05	0.94	0.96
Low SDI	60 to 64	1.15	1.13	1.41	1.26	0.99	0.93	0.96	1.05
Low-middle SDI	60 to 64	1.00	1.04	1.32	1.21	0.97	0.89	0.81	0.93
Middle SDI	60 to 64	1.19	1.15	1.42	1.29	0.95	1.00	0.95	0.97
Global	65 to 69	1.20	1.15	1.14	1.10	1.08	1.05	1.05	1.05
High SDI	65 to 69	1.07	1.04	1.06	1.03	0.98	1.03	1.00	1.00
High-middle SDI	65 to 69	1.27	1.17	1.18	1.14	1.22	1.09	1.08	1.07
Low SDI	65 to 69	1.43	1.29	1.10	1.05	1.06	0.99	1.14	1.21
Low-middle SDI	65 to 69	1.17	1.13	1.08	1.04	1.06	0.94	1.04	1.10
Middle SDI	65 to 69	1.37	1.23	1.18	1.15	0.90	1.04	1.16	1.09
Global	70 to 74	1.41	1.29	1.27	1.19	1.35	1.14	1.23	1.19
High SDI	70 to 74	1.17	1.10	1.11	1.06	1.08	1.06	1.12	1.06
High-middle SDI	70 to 74	1.50	1.30	1.33	1.22	1.58	1.18	1.29	1.20
Low SDI	70 to 74	1.73	1.54	1.29	1.18	1.17	1.12	1.35	1.40
Low-middle SDI	70 to 74	1.46	1.42	1.20	1.18	1.23	1.14	1.27	1.33
Middle SDI	70 to 74	1.72	1.41	1.36	1.27	1.77	1.18	1.35	1.26
Global	75 to 79	1.37	1.26	1.27	1.19	1.41	1.22	1.26	1.20
High SDI	75 to 79	1.19	1.11	1.15	1.10	1.13	1.13	1.17	1.07
High-middle SDI	75 to 79	1.45	1.26	1.30	1.18	1.66	1.25	1.34	1.21
Low SDI	75 to 79	1.54	1.43	1.28	1.19	1.25	1.18	1.32	1.34
Low-middle SDI	75 to 79	1.46	1.40	1.26	1.22	1.33	1.21	1.31	1.32
Middle SDI	75 to 79	1.56	1.35	1.31	1.21	1.77	1.26	1.33	1.27
Global	All ages	1.20	1.19	1.17	1.15	1.31	1.12	0.98	1.08
High SDI	All ages	1.02	1.01	1.09	1.06	1.06	1.04	0.89	0.96
High-middle SDI	All ages	1.20	1.18	1.18	1.19	1.44	1.14	0.85	1.02

Low SDI	All ages	1.32	1.24	1.10	1.04	1.15	1.07	1.15	1.19
Low-middle SDI	All ages	1.24	1.20	1.15	1.09	1.30	1.09	1.05	1.12
Middle SDI	All ages	1.39	1.33	1.21	1.23	1.55	1.15	1.18	1.19
Global	Age-standardized	1.22	1.18	1.25	1.19	1.04	1.01	1.31	1.24
High SDI	Age-standardized	1.07	1.04	1.12	1.08	0.95	1.00	1.15	1.07
High-middle SDI	Age-standardized	1.33	1.21	1.34	1.26	1.15	1.04	1.53	1.29
Low SDI	Age-standardized	1.29	1.23	1.17	1.08	1.02	0.97	1.34	1.37
Low-middle SDI	Age-standardized	1.17	1.17	1.18	1.13	1.04	0.95	1.29	1.35
Middle SDI	Age-standardized	1.42	1.32	1.34	1.31	0.90	1.02	1.47	1.37
Global	80 to 84	1.23	1.18	1.12	1.09	1.08	1.08	1.12	1.10
High SDI	80 to 84	1.15	1.07	1.06	1.04	1.03	1.07	1.10	1.04
High-middle SDI	80 to 84	1.27	1.17	1.22	1.11	1.15	1.07	1.12	1.09
Low SDI	80 to 84	1.34	1.30	1.04	0.98	1.15	1.09	1.21	1.22
Low-middle SDI	80 to 84	1.25	1.24	1.01	1.01	1.13	1.05	1.16	1.16
Middle SDI	80 to 84	1.32	1.26	1.13	1.13	0.84	1.07	1.15	1.15
Global	85 to 89	1.24	1.22	1.17	1.25	0.98	1.02	1.14	1.11
High SDI	85 to 89	1.13	1.06	1.05	1.04	0.98	1.03	1.08	1.02
High-middle SDI	85 to 89	1.40	1.30	1.47	1.56	0.95	1.00	1.22	1.15
Low SDI	85 to 89	1.25	1.25	1.04	1.03	1.06	1.04	1.16	1.17
Low-middle SDI	85 to 89	1.23	1.23	1.03	1.05	1.00	0.97	1.15	1.16
Middle SDI	85 to 89	1.38	1.40	1.35	1.73	0.76	1.01	1.18	1.20
Global	90 to 94	1.16	1.15	1.00	1.05	0.91	0.96	1.13	1.10
High SDI	90 to 94	1.09	1.03	0.98	0.99	0.93	0.98	1.08	1.03
High-middle SDI	90 to 94	1.32	1.27	1.08	1.16	0.79	0.87	1.22	1.18
Low SDI	90 to 94	1.17	1.17	0.91	0.90	1.01	0.99	1.16	1.16
Low-middle SDI	90 to 94	1.17	1.16	0.92	0.94	0.95	0.93	1.16	1.15
Middle SDI	90 to 94	1.27	1.34	0.96	1.20	0.72	0.92	1.19	1.21
Global	95 plus	1.10	1.06	1.10	1.12	1.03	1.06	1.16	1.11
High SDI	95 plus	1.05	1.02	1.07	1.08	1.02	1.06	1.11	1.06
High-middle SDI	95 plus	1.18	1.15	1.12	1.24	0.95	1.03	1.26	1.19
Low SDI	95 plus	1.16	1.13	1.10	1.06	1.18	1.11	1.21	1.19
Low-middle SDI	95 plus	1.15	1.13	1.10	1.11	1.13	1.10	1.21	1.18
Middle SDI	95 plus	1.13	1.11	1.04	1.14	0.91	1.02	1.19	1.14

ICH, intracerebral hemorrhage; SAH, subarachnoid hemorrhage; IS, ischemic stroke; GDR, gender disparity ratio; SDI, sociodemographic index

Table S18 GDR values by age group and 21 GBD regions for stroke, ICH, SAH, and IS in 1990 and 2021

		GDR							
		stroke		ICH		SAH		IS	
**location**	**age**	**1990**	**2021**	**1990**	**2021**	**1990**	**2021**	**1990**	**2021**
Andean Latin America	<5	1.24	1.05	1.14	1.02	1.17	1.07	1.03	1.02
Australasia	<5	1.01	0.99	1.00	0.99	1.04	1.01	1.02	1.01
Caribbean	<5	1.06	1.06	1.05	1.04	0.97	0.98	0.92	0.96
Central Asia	<5	1.02	1.01	1.04	1.00	1.02	1.01	0.99	1.01
Central Europe	<5	1.07	1.01	1.04	1.00	1.06	1.01	1.02	1.01
Central Latin America	<5	1.05	1.02	1.04	1.02	1.04	1.04	1.00	1.00

Central Sub-Saharan Africa	<5	1.03	1.02	0.92	0.98	0.99	1.03	1.03	1.04
East Asia	<5	1.07	0.99	1.05	1.00	0.98	1.01	0.98	0.99
Eastern Europe	<5	1.04	1.02	1.02	1.00	1.03	1.02	1.04	1.02
Eastern Sub-Saharan Africa	<5	1.11	1.04	1.02	1.00	1.09	1.06	1.01	1.02
High-income Asia Pacific	<5	1.03	1.00	1.02	1.00	1.03	1.01	1.02	1.01
High-income North America	<5	1.00	1.00	1.01	1.01	1.04	1.04	0.99	1.00
North Africa and Middle East	<5	1.19	1.02	1.13	1.01	1.23	1.04	0.74	0.98
Oceania	<5	0.99	1.00	0.95	0.95	0.95	0.94	0.93	0.96
South Asia	<5	1.28	1.07	1.18	1.05	1.41	1.10	0.96	1.01
Southeast Asia	<5	1.11	1.05	1.07	1.04	1.03	1.03	0.97	1.00
Southern Latin America	<5	1.05	1.00	1.02	0.99	1.06	1.01	0.99	1.01
Southern Sub-Saharan Africa	<5	1.02	1.02	0.95	0.99	0.99	1.00	1.04	1.03
Tropical Latin America	<5	1.04	1.02	1.02	1.01	1.01	1.01	1.01	1.03
Western Europe	<5	1.02	1.00	1.01	0.99	1.01	1.00	1.01	1.01
Western Sub-Saharan Africa	<5	1.26	1.15	1.00	0.99	1.12	1.08	1.50	1.32
Andean Latin America	5 to 9	0.96	1.00	0.99	0.99	0.74	0.88	1.10	1.06
Australasia	5 to 9	0.98	0.99	0.98	0.99	0.96	0.99	1.02	1.01
Caribbean	5 to 9	0.98	0.99	1.01	1.02	0.82	0.81	0.96	0.97
Central Asia	5 to 9	0.99	0.99	0.99	0.99	0.91	0.96	1.04	1.04
Central Europe	5 to 9	1.00	1.00	1.00	0.99	0.94	0.99	1.05	1.02
Central Latin America	5 to 9	0.98	1.00	0.99	0.99	0.87	0.95	1.07	1.03
Central Sub-Saharan Africa	5 to 9	0.93	0.99	0.91	0.97	0.92	0.99	1.07	1.06
East Asia	5 to 9	1.02	1.01	1.02	1.00	0.82	0.99	1.06	1.04
Eastern Europe	5 to 9	0.99	1.00	0.97	0.98	0.98	0.99	1.05	1.03
Eastern Sub-Saharan Africa	5 to 9	1.00	1.00	0.98	0.98	1.01	1.01	1.05	1.04
High-income Asia Pacific	5 to 9	0.99	1.00	1.00	0.99	0.93	0.99	1.07	1.02
High-income North America	5 to 9	1.00	1.00	1.00	1.00	0.96	1.00	1.00	1.00
North Africa and Middle East	5 to 9	0.93	0.99	0.96	0.99	0.87	0.99	1.05	1.06
Oceania	5 to 9	0.97	0.96	0.85	0.84	1.17	1.14	1.04	1.03
South Asia	5 to 9	0.96	0.99	0.96	0.98	0.90	0.95	1.06	1.06
Southeast Asia	5 to 9	0.98	1.01	0.99	1.01	0.76	0.93	1.09	1.08
Southern Latin America	5 to 9	0.98	1.00	1.00	0.99	0.88	0.98	1.04	1.03
Southern Sub-Saharan Africa	5 to 9	1.03	1.01	1.02	1.00	0.98	0.98	1.06	1.04
Tropical Latin America	5 to 9	0.98	0.99	0.99	0.98	0.85	0.92	1.07	1.04
Western Europe	5 to 9	0.98	0.99	0.99	0.99	0.87	0.98	1.06	1.02
Western Sub-Saharan Africa	5 to 9	1.11	1.08	1.05	1.03	1.05	1.05	1.65	1.80
Andean Latin America	10 to 14	0.97	0.98	0.94	0.97	1.65	1.31	1.00	1.00
Australasia	10 to 14	0.98	0.98	0.98	1.00	1.20	1.02	1.08	1.02
Caribbean	10 to 14	0.88	0.88	0.91	0.91	1.17	1.12	1.05	1.03
Central Asia	10 to 14	1.00	1.01	0.95	0.97	1.03	1.03	1.24	1.14
Central Europe	10 to 14	1.02	1.00	0.98	0.99	1.19	1.04	1.20	1.07
Central Latin America	10 to 14	0.99	1.03	0.98	0.99	1.16	1.24	1.23	1.12
Central Sub-Saharan	10 to 14	0.86	0.94	0.77	0.88	1.08	1.06	1.06	1.06

Africa									
East Asia	10 to 14	1.00	1.02	1.00	1.01	1.20	1.06	1.14	1.11
Eastern Europe	10 to 14	1.01	1.02	0.98	0.99	1.06	1.05	1.06	1.05
Eastern Sub-Saharan Africa	10 to 14	0.94	0.97	0.87	0.92	1.09	1.06	1.03	1.05
High-income Asia Pacific	10 to 14	0.99	0.99	0.99	0.99	1.18	1.03	1.15	1.04
High-income North America	10 to 14	0.96	0.97	1.01	1.01	1.10	1.10	1.04	1.02
North Africa and Middle East	10 to 14	0.92	1.00	0.88	0.98	1.31	1.09	1.03	1.13
Oceania	10 to 14	0.85	0.83	0.86	0.85	0.97	0.95	0.98	0.98
South Asia	10 to 14	0.97	0.99	0.93	0.95	1.21	1.12	1.05	1.07
Southeast Asia	10 to 14	0.97	1.04	0.93	0.99	1.25	1.22	1.14	1.17
Southern Latin America	10 to 14	1.00	1.00	0.99	0.99	1.43	1.09	1.09	1.03
Southern Sub-Saharan Africa	10 to 14	1.02	1.01	0.97	0.94	1.02	1.04	1.10	1.15
Tropical Latin America	10 to 14	1.00	1.00	0.97	0.98	1.23	1.23	1.23	1.07
Western Europe	10 to 14	0.97	0.99	0.97	0.99	1.15	1.02	1.16	1.02
Western Sub-Saharan Africa	10 to 14	0.92	0.94	0.86	0.87	1.04	1.04	1.24	1.35
Andean Latin America	15 to 19	2.17	1.22	1.18	1.02	2.16	1.15	1.41	1.07
Australasia	15 to 19	1.10	1.07	1.03	1.00	1.37	1.06	1.10	1.03
Caribbean	15 to 19	1.00	0.98	0.86	0.86	1.10	0.98	1.03	1.05
Central Asia	15 to 19	1.18	1.21	1.02	1.06	1.16	1.19	1.32	1.31
Central Europe	15 to 19	1.21	1.12	1.03	1.00	1.23	1.09	1.34	1.12
Central Latin America	15 to 19	1.12	1.15	0.96	0.98	1.18	1.29	1.25	1.16
Central Sub-Saharan Africa	15 to 19	1.14	1.09	0.82	0.83	1.16	1.11	1.04	1.03
East Asia	15 to 19	1.51	1.25	1.23	1.16	1.85	1.14	1.31	1.20
Eastern Europe	15 to 19	1.23	1.18	1.09	1.09	1.26	1.12	1.32	1.14
Eastern Sub-Saharan Africa	15 to 19	1.55	1.26	0.92	0.88	1.43	1.26	1.02	1.07
High-income Asia Pacific	15 to 19	1.18	1.07	1.05	1.01	1.27	1.06	1.18	1.03
High-income North America	15 to 19	1.00	1.01	1.00	1.01	1.05	1.04	1.02	1.01
North Africa and Middle East	15 to 19	1.27	1.19	1.09	1.08	1.17	1.06	1.15	1.26
Oceania	15 to 19	1.16	1.06	1.06	0.95	0.99	0.91	0.91	0.87
South Asia	15 to 19	1.03	1.06	0.87	0.92	1.10	1.04	0.92	0.98
Southeast Asia	15 to 19	1.16	1.44	0.93	1.17	1.18	1.30	1.12	1.38
Southern Latin America	15 to 19	1.22	1.17	1.00	1.02	2.24	1.28	1.15	1.09
Southern Sub-Saharan Africa	15 to 19	1.23	1.50	1.02	1.45	1.07	1.21	1.07	1.19
Tropical Latin America	15 to 19	1.24	1.23	1.04	1.09	1.19	1.20	1.41	1.16
Western Europe	15 to 19	1.15	1.06	1.05	1.01	1.20	1.04	1.24	1.03
Western Sub-Saharan Africa	15 to 19	0.97	1.01	0.73	0.76	0.97	0.97	1.15	1.19
Andean Latin America	20 to 24	0.93	1.00	0.74	0.89	0.87	0.91	1.95	1.30
Australasia	20 to 24	0.98	0.99	0.92	0.94	0.97	1.00	1.18	1.04
Caribbean	20 to 24	0.86	0.83	0.71	0.70	0.94	0.81	1.12	1.08
Central Asia	20 to 24	0.92	1.02	0.77	0.91	0.94	1.01	1.52	1.60
Central Europe	20 to 24	1.03	1.02	0.94	0.96	0.98	0.99	1.43	1.20
Central Latin America	20 to 24	0.96	1.01	0.86	0.92	0.95	1.03	1.45	1.22
Central Sub-Saharan Africa	20 to 24	0.91	0.93	0.66	0.68	0.97	0.97	1.08	1.09

East Asia	20 to 24	1.09	1.12	0.97	1.05	1.03	1.04	1.38	1.46
Eastern Europe	20 to 24	1.05	1.07	0.95	1.02	1.04	1.01	1.65	1.36
Eastern Sub-Saharan Africa	20 to 24	0.97	1.03	0.67	0.75	1.04	1.06	1.08	1.16
High-income Asia Pacific	20 to 24	1.00	1.01	0.92	0.96	1.03	0.99	1.17	1.07
High-income North America	20 to 24	0.97	1.00	0.93	0.96	0.96	1.01	1.03	1.03
North Africa and Middle East	20 to 24	0.94	0.98	0.78	0.87	0.99	0.99	1.18	1.40
Oceania	20 to 24	0.88	0.85	0.54	0.47	0.97	0.94	1.13	1.11
South Asia	20 to 24	0.94	1.00	0.79	0.86	0.98	0.99	1.06	1.13
Southeast Asia	20 to 24	0.92	1.06	0.74	0.91	0.94	1.02	1.31	1.56
Southern Latin America	20 to 24	0.95	0.99	0.87	0.92	0.88	0.96	1.14	1.06
Southern Sub-Saharan Africa	20 to 24	0.98	1.13	0.71	0.96	0.96	1.04	1.10	1.27
Tropical Latin America	20 to 24	0.97	0.99	0.86	0.91	0.89	0.91	1.56	1.23
Western Europe	20 to 24	1.01	1.00	0.94	0.95	0.94	0.98	1.25	1.04
Western Sub-Saharan Africa	20 to 24	0.97	0.99	0.76	0.77	1.01	1.02	1.31	1.43
Andean Latin America	25 to 29	1.13	1.15	1.10	1.15	1.01	1.05	1.18	1.13
Australasia	25 to 29	1.00	0.98	0.96	0.94	1.14	1.03	1.05	1.00
Caribbean	25 to 29	1.00	0.96	1.00	1.00	0.95	0.84	1.13	1.10
Central Asia	25 to 29	1.09	1.14	1.14	1.18	1.06	1.11	1.24	1.30
Central Europe	25 to 29	1.15	1.07	1.18	1.09	1.08	1.04	1.21	1.11
Central Latin America	25 to 29	1.05	1.10	1.04	1.05	1.03	1.12	1.11	1.10
Central Sub-Saharan Africa	25 to 29	0.99	1.04	0.89	0.95	1.00	1.02	0.97	1.01
East Asia	25 to 29	1.20	1.27	1.17	1.33	1.09	1.09	1.14	1.25
Eastern Europe	25 to 29	1.19	1.21	1.30	1.38	1.14	1.06	1.32	1.32
Eastern Sub-Saharan Africa	25 to 29	1.03	1.13	0.94	1.07	1.04	1.09	0.97	1.04
High-income Asia Pacific	25 to 29	1.11	1.06	1.04	1.03	1.19	1.04	1.09	1.05
High-income North America	25 to 29	0.96	1.00	0.96	0.98	0.98	1.05	0.99	1.00
North Africa and Middle East	25 to 29	1.05	1.06	1.13	1.10	1.08	1.04	0.94	1.14
Oceania	25 to 29	1.04	1.09	0.86	0.92	1.22	1.20	0.85	0.87
South Asia	25 to 29	1.06	1.08	1.05	1.06	1.08	1.05	0.96	1.04
Southeast Asia	25 to 29	1.12	1.25	1.09	1.26	1.06	1.10	1.19	1.32
Southern Latin America	25 to 29	1.07	1.04	1.03	1.02	1.10	1.02	1.04	1.03
Southern Sub-Saharan Africa	25 to 29	1.09	1.24	0.97	1.38	0.95	1.08	1.10	1.18
Tropical Latin America	25 to 29	1.22	1.11	1.36	1.17	0.99	1.01	1.30	1.11
Western Europe	25 to 29	1.06	1.01	1.06	0.99	0.98	0.99	1.11	1.01
Western Sub-Saharan Africa	25 to 29	1.01	1.05	0.90	0.94	1.04	1.07	1.13	1.21
Andean Latin America	30 to 34	1.11	1.13	1.27	1.27	1.04	1.06	1.08	1.10
Australasia	30 to 34	1.00	0.98	1.02	0.98	1.16	1.06	1.05	1.01
Caribbean	30 to 34	1.05	1.04	1.26	1.25	1.02	0.92	1.21	1.17
Central Asia	30 to 34	1.13	1.19	1.27	1.30	1.14	1.18	1.19	1.32
Central Europe	30 to 34	1.23	1.14	1.38	1.27	1.15	1.10	1.37	1.18
Central Latin America	30 to 34	1.06	1.14	1.13	1.20	1.09	1.20	1.14	1.15
Central Sub-Saharan Africa	30 to 34	1.02	1.10	1.00	1.11	1.01	1.06	1.00	1.06
East Asia	30 to 34	1.33	1.42	1.36	1.53	1.23	1.19	1.26	1.42

Eastern Europe	30 to 34	1.28	1.37	1.47	1.75	1.30	1.24	1.52	1.57
Eastern Sub-Saharan Africa	30 to 34	1.08	1.22	1.13	1.32	1.07	1.14	0.98	1.07
High-income Asia Pacific	30 to 34	1.21	1.13	1.19	1.15	1.55	1.12	1.11	1.08
High-income North America	30 to 34	0.98	1.01	1.04	1.03	1.11	1.12	1.00	0.99
North Africa and Middle East	30 to 34	1.04	1.02	1.17	1.09	1.12	1.03	0.92	1.06
Oceania	30 to 34	1.03	1.10	1.12	1.28	0.94	0.97	1.03	1.05
South Asia	30 to 34	1.28	1.23	1.53	1.36	1.38	1.21	1.07	1.13
Southeast Asia	30 to 34	1.26	1.39	1.37	1.55	1.21	1.21	1.23	1.35
Southern Latin America	30 to 34	1.05	1.02	1.11	1.06	1.14	1.02	1.01	1.03
Southern Sub-Saharan Africa	30 to 34	1.30	1.26	1.52	1.45	0.94	1.05	1.21	1.16
Tropical Latin America	30 to 34	1.48	1.10	2.25	1.32	1.23	1.04	1.60	1.08
Western Europe	30 to 34	1.05	1.01	1.10	1.01	1.00	1.00	1.13	1.04
Western Sub-Saharan Africa	30 to 34	0.96	1.05	0.94	1.03	1.07	1.12	1.12	1.25
Andean Latin America	35 to 39	1.27	1.17	1.19	1.19	1.02	1.02	1.08	1.09
Australasia	35 to 39	1.06	1.01	1.05	1.03	1.02	1.08	1.09	1.02
Caribbean	35 to 39	1.31	1.27	1.21	1.28	1.04	0.93	1.32	1.24
Central Asia	35 to 39	1.32	1.36	1.20	1.25	1.19	1.20	1.39	1.46
Central Europe	35 to 39	1.59	1.33	1.41	1.35	1.17	1.17	1.78	1.30
Central Latin America	35 to 39	1.18	1.27	1.16	1.27	1.03	1.15	1.20	1.19
Central Sub-Saharan Africa	35 to 39	1.19	1.25	1.07	1.13	1.02	1.06	1.07	1.13
East Asia	35 to 39	1.83	1.70	1.39	1.45	1.16	1.18	1.46	1.57
Eastern Europe	35 to 39	1.50	1.69	1.39	1.60	1.30	1.31	1.85	1.81
Eastern Sub-Saharan Africa	35 to 39	1.31	1.31	1.16	1.19	1.07	1.08	1.02	1.10
High-income Asia Pacific	35 to 39	1.40	1.21	1.24	1.20	1.45	1.14	1.12	1.09
High-income North America	35 to 39	1.05	1.03	1.12	1.08	1.13	1.11	1.03	0.99
North Africa and Middle East	35 to 39	1.16	1.07	1.17	1.09	1.12	1.02	0.75	1.03
Oceania	35 to 39	1.77	1.80	1.19	1.31	1.13	1.10	1.50	1.47
South Asia	35 to 39	1.34	1.28	1.26	1.19	1.17	1.09	1.08	1.13
Southeast Asia	35 to 39	1.78	1.85	1.35	1.46	1.20	1.18	1.26	1.43
Southern Latin America	35 to 39	1.23	1.10	1.16	1.10	1.00	1.02	1.05	1.05
Southern Sub-Saharan Africa	35 to 39	1.66	1.40	1.36	1.21	0.98	1.03	1.28	1.15
Tropical Latin America	35 to 39	4.00	1.25	1.97	1.30	1.33	0.98	2.00	1.14
Western Europe	35 to 39	1.14	1.05	1.10	1.03	1.00	1.02	1.19	1.07
Western Sub-Saharan Africa	35 to 39	1.05	1.12	1.00	1.07	1.05	1.09	1.23	1.39
Andean Latin America	40 to 44	1.16	1.08	1.31	1.22	0.93	0.94	1.31	1.14
Australasia	40 to 44	1.06	1.02	1.14	1.10	1.01	1.01	1.09	1.02
Caribbean	40 to 44	1.21	1.26	1.52	1.67	0.94	0.91	1.36	1.34
Central Asia	40 to 44	1.34	1.33	1.40	1.35	1.10	1.14	1.89	1.59
Central Europe	40 to 44	1.42	1.32	1.49	1.46	1.02	1.08	2.17	1.43
Central Latin America	40 to 44	1.13	1.21	1.26	1.38	0.97	1.05	1.26	1.17
Central Sub-Saharan Africa	40 to 44	1.27	1.29	1.33	1.41	1.00	1.01	1.12	1.14
East Asia	40 to 44	1.88	1.57	1.63	1.54	1.01	1.11	5.82	2.11
Eastern Europe	40 to 44	1.39	1.57	1.46	1.74	1.13	1.17	2.76	2.50

Eastern Sub-Saharan Africa	40 to 44	1.52	1.39	1.61	1.57	1.07	1.06	1.06	1.13
High-income Asia Pacific	40 to 44	1.24	1.15	1.32	1.21	1.04	1.04	1.13	1.07
High-income North America	40 to 44	1.03	1.01	1.15	1.09	1.03	1.07	1.03	1.00
North Africa and Middle East	40 to 44	1.06	1.02	1.30	1.15	1.05	0.98	1.05	1.13
Oceania	40 to 44	1.40	1.36	1.58	1.56	1.10	1.07	0.85	0.89
South Asia	40 to 44	1.22	1.21	1.40	1.31	1.05	1.02	1.07	1.12
Southeast Asia	40 to 44	1.57	1.61	1.59	1.63	1.11	1.09	1.46	1.60
Southern Latin America	40 to 44	1.24	1.09	1.32	1.16	1.03	0.99	1.10	1.04
Southern Sub-Saharan Africa	40 to 44	1.34	1.31	1.39	1.43	0.96	0.98	1.26	1.19
Tropical Latin America	40 to 44	1.94	1.13	2.16	1.46	1.09	0.84	2.26	1.20
Western Europe	40 to 44	1.10	1.05	1.17	1.09	0.95	0.97	1.22	1.06
Western Sub-Saharan Africa	40 to 44	1.09	1.18	1.22	1.28	1.02	1.06	1.39	1.61
Andean Latin America	45 to 49	1.16	1.04	1.40	1.23	1.06	0.99	1.06	1.05
Australasia	45 to 49	1.01	0.99	1.11	1.08	1.19	1.15	1.02	0.98
Caribbean	45 to 49	1.33	1.30	1.68	1.77	1.24	1.13	1.11	1.10
Central Asia	45 to 49	1.24	1.26	1.28	1.29	1.19	1.25	1.39	1.33
Central Europe	45 to 49	1.37	1.29	1.46	1.51	1.24	1.17	1.66	1.30
Central Latin America	45 to 49	1.14	1.18	1.28	1.39	1.08	1.17	1.18	1.14
Central Sub-Saharan Africa	45 to 49	1.26	1.26	1.31	1.41	1.12	1.10	1.05	1.06
East Asia	45 to 49	1.69	1.34	1.54	1.38	1.50	1.21	1.90	1.32
Eastern Europe	45 to 49	1.29	1.38	1.34	1.56	1.28	1.32	1.78	1.61
Eastern Sub-Saharan Africa	45 to 49	1.55	1.34	1.68	1.60	1.25	1.16	1.01	1.06
High-income Asia Pacific	45 to 49	1.14	1.09	1.27	1.17	1.29	1.15	1.02	1.02
High-income North America	45 to 49	1.01	0.99	1.15	1.10	1.19	1.17	1.02	0.99
North Africa and Middle East	45 to 49	1.08	1.02	1.34	1.15	1.25	1.07	0.86	0.97
Oceania	45 to 49	1.24	1.20	1.30	1.25	1.53	1.34	0.89	0.92
South Asia	45 to 49	1.12	1.08	1.30	1.19	1.22	1.10	0.95	1.00
Southeast Asia	45 to 49	1.50	1.49	1.56	1.57	1.35	1.24	1.24	1.31
Southern Latin America	45 to 49	1.22	1.08	1.34	1.17	1.40	1.13	1.06	1.01
Southern Sub-Saharan Africa	45 to 49	1.22	1.25	1.26	1.39	1.08	1.08	1.16	1.14
Tropical Latin America	45 to 49	1.83	1.17	2.13	1.57	1.59	1.10	3.02	1.22
Western Europe	45 to 49	1.09	1.03	1.20	1.10	1.08	1.05	1.11	1.02
Western Sub-Saharan Africa	45 to 49	1.25	1.28	1.32	1.33	1.21	1.19	1.50	1.67
Andean Latin America	50 to 54	1.26	1.08	1.23	1.14	0.95	0.90	1.12	1.05
Australasia	50 to 54	1.03	0.98	1.04	1.02	0.91	0.97	0.96	0.92
Caribbean	50 to 54	1.34	1.31	1.27	1.32	0.92	0.94	1.14	1.16
Central Asia	50 to 54	1.22	1.24	1.13	1.16	1.00	1.07	1.25	1.23
Central Europe	50 to 54	1.34	1.28	1.23	1.31	1.02	1.04	1.47	1.26
Central Latin America	50 to 54	1.16	1.20	1.16	1.26	0.98	1.04	1.12	1.10
Central Sub-Saharan Africa	50 to 54	1.33	1.30	1.15	1.20	0.99	1.00	1.03	1.02
East Asia	50 to 54	1.92	1.28	1.28	1.19	0.92	1.06	2.56	1.22
Eastern Europe	50 to 54	1.24	1.30	1.12	1.23	1.06	1.09	1.63	1.48
Eastern Sub-Saharan	50 to 54	1.54	1.32	1.30	1.26	1.07	1.02	0.96	1.00

Africa									
High-income Asia Pacific	50 to 54	1.08	1.05	1.12	1.08	1.00	1.02	0.95	0.96
High-income North America	50 to 54	1.02	1.00	1.08	1.05	1.00	1.06	0.96	0.99
North Africa and Middle East	50 to 54	1.11	1.03	1.17	1.05	1.07	0.98	0.90	0.98
Oceania	50 to 54	1.36	1.24	1.08	1.04	0.86	0.89	0.80	0.84
South Asia	50 to 54	1.30	1.16	1.26	1.14	1.13	1.01	0.98	1.03
Southeast Asia	50 to 54	1.45	1.46	1.22	1.26	1.10	1.06	1.21	1.28
Southern Latin America	50 to 54	1.22	1.10	1.22	1.14	1.16	1.03	1.00	0.97
Southern Sub-Saharan Africa	50 to 54	1.20	1.37	1.10	1.21	1.01	0.99	1.07	1.14
Tropical Latin America	50 to 54	1.69	1.23	1.46	1.34	1.15	0.96	1.89	1.18
Western Europe	50 to 54	1.08	1.05	1.12	1.08	0.96	0.97	1.02	0.97
Western Sub-Saharan Africa	50 to 54	1.29	1.29	1.07	1.09	1.01	1.02	1.69	1.86
Andean Latin America	55 to 59	1.19	1.06	1.22	1.11	0.98	0.90	1.14	1.04
Australasia	55 to 59	1.03	0.98	1.02	0.98	1.02	0.99	0.97	0.93
Caribbean	55 to 59	1.27	1.26	1.33	1.34	1.03	1.02	1.15	1.20
Central Asia	55 to 59	1.19	1.19	1.14	1.14	1.05	1.10	1.29	1.23
Central Europe	55 to 59	1.22	1.20	1.27	1.30	1.06	1.04	1.40	1.24
Central Latin America	55 to 59	1.14	1.16	1.19	1.23	1.00	1.04	1.12	1.10
Central Sub-Saharan Africa	55 to 59	1.29	1.27	1.17	1.23	0.98	0.98	1.06	1.06
East Asia	55 to 59	1.72	1.19	1.33	1.17	1.46	1.05	2.18	1.19
Eastern Europe	55 to 59	1.18	1.25	1.12	1.21	1.11	1.13	1.79	1.62
Eastern Sub-Saharan Africa	55 to 59	1.42	1.25	1.30	1.27	1.07	1.01	0.96	1.02
High-income Asia Pacific	55 to 59	1.03	1.02	1.06	1.00	0.99	0.98	0.96	0.98
High-income North America	55 to 59	1.00	1.00	1.06	1.02	1.04	1.09	0.95	1.00
North Africa and Middle East	55 to 59	1.09	1.02	1.24	1.07	1.24	1.01	1.04	1.03
Oceania	55 to 59	1.21	1.10	1.11	1.05	0.96	0.91	0.93	0.94
South Asia	55 to 59	1.02	0.99	1.15	1.07	1.09	0.88	0.86	0.95
Southeast Asia	55 to 59	1.34	1.36	1.26	1.28	1.16	1.07	1.18	1.27
Southern Latin America	55 to 59	1.14	1.08	1.20	1.13	1.24	1.03	1.00	0.97
Southern Sub-Saharan Africa	55 to 59	1.14	1.25	1.10	1.22	1.01	0.98	1.04	1.10
Tropical Latin America	55 to 59	1.40	1.16	1.56	1.38	1.40	1.06	1.58	1.17
Western Europe	55 to 59	1.06	1.04	1.12	1.04	0.97	0.96	1.01	0.97
Western Sub-Saharan Africa	55 to 59	1.15	1.16	1.00	1.02	1.00	0.98	1.60	1.75
Andean Latin America	60 to 64	1.14	1.07	1.32	1.21	0.94	0.90	1.07	1.03
Australasia	60 to 64	1.01	1.01	1.06	1.03	0.98	1.02	0.88	0.91
Caribbean	60 to 64	1.14	1.16	1.50	1.42	0.98	0.96	0.90	0.99
Central Asia	60 to 64	1.17	1.15	1.24	1.20	1.00	1.03	1.06	1.01
Central Europe	60 to 64	1.10	1.14	1.35	1.35	0.96	1.00	0.91	0.98
Central Latin America	60 to 64	1.12	1.13	1.27	1.27	0.97	0.97	0.99	1.03
Central Sub-Saharan Africa	60 to 64	1.25	1.26	1.37	1.45	0.99	0.99	0.98	1.01
East Asia	60 to 64	1.28	1.16	1.51	1.31	1.10	1.06	0.91	0.93
Eastern Europe	60 to 64	1.18	1.21	1.24	1.29	1.10	1.13	1.05	1.11
Eastern Sub-Saharan Africa	60 to 64	1.51	1.29	1.99	1.66	1.08	1.02	0.95	1.00

High-income Asia Pacific	60 to 64	1.01	1.01	1.08	1.01	0.92	0.97	0.88	0.94
High-income North America	60 to 64	1.02	1.00	1.10	1.05	0.96	1.06	0.91	1.00
North Africa and Middle East	60 to 64	1.00	1.00	1.39	1.15	1.15	1.02	0.65	0.79
Oceania	60 to 64	0.80	0.90	1.18	1.16	0.66	0.73	0.73	0.79
South Asia	60 to 64	0.88	0.93	1.26	1.13	0.92	0.83	0.64	0.80
Southeast Asia	60 to 64	1.17	1.23	1.36	1.34	1.02	1.00	1.04	1.09
Southern Latin America	60 to 64	1.09	1.06	1.27	1.18	1.01	0.98	0.93	0.94
Southern Sub-Saharan Africa	60 to 64	1.15	1.24	1.18	1.28	1.02	0.99	1.07	1.15
Tropical Latin America	60 to 64	1.17	1.11	1.47	1.36	0.89	0.81	1.13	1.04
Western Europe	60 to 64	1.06	1.04	1.17	1.09	0.93	0.98	0.91	0.93
Western Sub-Saharan Africa	60 to 64	1.24	1.24	1.12	1.10	1.02	1.00	1.55	1.66
Andean Latin America	65 to 69	1.22	1.08	1.12	1.07	0.94	0.89	1.17	1.07
Australasia	65 to 69	1.02	1.02	1.01	1.02	0.99	1.06	0.97	0.97
Caribbean	65 to 69	1.24	1.24	1.12	1.11	1.00	1.01	1.06	1.13
Central Asia	65 to 69	1.30	1.23	1.04	1.03	1.04	1.06	1.22	1.16
Central Europe	65 to 69	1.13	1.15	1.08	1.17	0.95	1.02	1.07	1.09
Central Latin America	65 to 69	1.18	1.17	1.12	1.15	0.99	0.99	1.12	1.12
Central Sub-Saharan Africa	65 to 69	1.53	1.53	1.05	1.15	1.04	1.04	1.16	1.20
East Asia	65 to 69	1.53	1.22	1.28	1.21	1.18	1.11	1.13	1.06
Eastern Europe	65 to 69	1.28	1.26	1.06	1.13	1.17	1.18	1.44	1.28
Eastern Sub-Saharan Africa	65 to 69	2.32	1.61	1.75	1.46	1.16	1.08	1.10	1.16
High-income Asia Pacific	65 to 69	1.03	1.00	1.03	1.00	0.96	0.99	0.96	0.97
High-income North America	65 to 69	1.06	1.00	1.03	1.02	1.02	1.08	1.00	1.00
North Africa and Middle East	65 to 69	1.10	1.02	1.21	1.03	1.20	1.05	0.86	0.90
Oceania	65 to 69	0.93	0.98	0.82	0.83	0.83	0.86	0.88	0.90
South Asia	65 to 69	1.03	0.98	1.09	1.02	1.03	0.87	0.87	0.93
Southeast Asia	65 to 69	1.26	1.32	1.05	1.09	1.05	1.03	1.17	1.23
Southern Latin America	65 to 69	1.13	1.09	1.15	1.12	1.02	1.02	1.04	1.01
Southern Sub-Saharan Africa	65 to 69	1.24	1.37	1.01	1.04	1.06	1.03	1.15	1.25
Tropical Latin America	65 to 69	1.26	1.16	1.13	1.13	0.90	0.84	1.39	1.15
Western Europe	65 to 69	1.09	1.04	1.07	1.04	0.95	1.03	1.02	0.98
Western Sub-Saharan Africa	65 to 69	1.23	1.22	0.79	0.85	1.00	0.98	1.66	1.69
Andean Latin America	70 to 74	1.39	1.20	1.26	1.17	1.05	1.02	1.28	1.15
Australasia	70 to 74	1.13	1.07	1.08	1.05	1.07	1.07	1.09	1.03
Caribbean	70 to 74	1.48	1.40	1.28	1.25	1.12	1.11	1.30	1.27
Central Asia	70 to 74	1.57	1.46	1.20	1.16	1.18	1.21	1.47	1.40
Central Europe	70 to 74	1.35	1.24	1.32	1.28	1.16	1.18	1.31	1.19
Central Latin America	70 to 74	1.30	1.27	1.18	1.19	1.05	1.07	1.26	1.21
Central Sub-Saharan Africa	70 to 74	1.80	1.84	1.36	1.56	1.11	1.11	1.35	1.39
East Asia	70 to 74	1.89	1.39	1.48	1.30	2.63	1.25	1.33	1.23
Eastern Europe	70 to 74	1.63	1.41	1.21	1.21	1.32	1.30	1.95	1.43
Eastern Sub-Saharan Africa	70 to 74	1.97	1.58	1.77	1.48	1.15	1.10	1.19	1.24
High-income Asia	70 to 74	1.13	1.06	1.07	1.01	1.03	1.00	1.10	1.04

Pacific									
High-income North America	70 to 74	1.14	1.06	1.08	1.04	1.10	1.12	1.09	1.02
North Africa and Middle East	70 to 74	1.37	1.19	1.43	1.17	1.33	1.15	1.26	1.15
Oceania	70 to 74	1.56	1.39	1.30	1.21	0.95	0.95	1.05	1.04
South Asia	70 to 74	1.32	1.30	1.19	1.14	1.26	1.13	1.11	1.20
Southeast Asia	70 to 74	1.52	1.53	1.22	1.26	1.18	1.13	1.29	1.35
Southern Latin America	70 to 74	1.27	1.18	1.21	1.16	1.13	1.07	1.17	1.10
Southern Sub-Saharan Africa	70 to 74	1.33	1.39	1.13	1.18	1.09	1.08	1.23	1.29
Tropical Latin America	70 to 74	1.48	1.27	1.25	1.20	1.03	1.07	1.51	1.22
Western Europe	70 to 74	1.18	1.08	1.11	1.06	1.05	1.06	1.14	1.04
Western Sub-Saharan Africa	70 to 74	1.68	1.54	1.01	1.00	1.08	1.04	1.92	1.84
Andean Latin America	75 to 79	1.38	1.21	1.31	1.20	1.11	1.08	1.29	1.16
Australasia	75 to 79	1.15	1.07	1.13	1.10	1.14	1.16	1.14	1.04
Caribbean	75 to 79	1.44	1.35	1.31	1.28	1.16	1.15	1.30	1.26
Central Asia	75 to 79	1.64	1.47	1.32	1.19	1.32	1.42	1.53	1.43
Central Europe	75 to 79	1.48	1.24	1.59	1.36	1.29	1.28	1.50	1.22
Central Latin America	75 to 79	1.31	1.25	1.20	1.20	1.10	1.12	1.29	1.20
Central Sub-Saharan Africa	75 to 79	1.60	1.63	1.43	1.52	1.18	1.18	1.32	1.34
East Asia	75 to 79	1.56	1.32	1.32	1.20	8.70	1.33	1.29	1.25
Eastern Europe	75 to 79	1.91	1.50	1.29	1.30	1.57	1.50	2.64	1.58
Eastern Sub-Saharan Africa	75 to 79	1.61	1.42	1.58	1.40	1.22	1.15	1.19	1.22
High-income Asia Pacific	75 to 79	1.18	1.09	1.11	1.06	1.08	1.03	1.18	1.06
High-income North America	75 to 79	1.15	1.08	1.13	1.08	1.17	1.20	1.12	1.03
North Africa and Middle East	75 to 79	1.49	1.26	1.51	1.24	1.60	1.28	1.41	1.27
Oceania	75 to 79	1.39	1.33	1.39	1.33	1.06	1.05	1.09	1.08
South Asia	75 to 79	1.40	1.32	1.26	1.19	1.40	1.21	1.22	1.24
Southeast Asia	75 to 79	1.47	1.47	1.31	1.34	1.28	1.21	1.29	1.32
Southern Latin America	75 to 79	1.31	1.18	1.29	1.21	1.17	1.14	1.24	1.13
Southern Sub-Saharan Africa	75 to 79	1.29	1.31	1.21	1.27	1.14	1.13	1.21	1.23
Tropical Latin America	75 to 79	1.49	1.25	1.41	1.29	1.07	1.13	1.48	1.22
Western Europe	75 to 79	1.22	1.09	1.17	1.10	1.10	1.14	1.21	1.05
Western Sub-Saharan Africa	75 to 79	1.40	1.36	1.01	1.01	1.10	1.07	1.52	1.50
Andean Latin America	All ages	1.25	1.12	1.16	1.11	1.11	0.98	1.16	1.10
Australasia	All ages	0.94	0.94	1.01	1.02	1.07	1.08	0.80	0.86
Caribbean	All ages	1.26	1.25	1.23	1.26	1.11	1.07	1.02	1.06
Central Asia	All ages	1.20	1.23	1.04	1.09	1.10	1.15	1.12	1.17
Central Europe	All ages	1.10	1.07	1.12	1.24	1.18	1.11	0.69	0.83
Central Latin America	All ages	1.18	1.19	1.13	1.20	1.04	1.08	1.16	1.15
Central Sub-Saharan Africa	All ages	1.35	1.26	0.98	1.01	1.06	1.05	1.17	1.10
East Asia	All ages	1.60	1.36	1.30	1.29	5.40	1.24	1.22	1.22
Eastern Europe	All ages	1.01	1.13	0.98	1.15	1.20	1.21	0.52	0.88
Eastern Sub-Saharan Africa	All ages	1.56	1.32	1.30	1.15	1.17	1.10	1.10	1.11
High-income Asia Pacific	All ages	1.04	1.00	1.09	1.05	1.07	0.98	0.90	0.93

High-income North America	All ages	1.02	0.97	1.07	1.05	1.09	1.17	0.97	0.96
North Africa and Middle East	All ages	1.14	1.05	1.17	1.04	1.36	1.06	0.92	0.96
Oceania	All ages	1.17	1.14	0.91	0.88	0.98	0.95	1.01	1.01
South Asia	All ages	1.18	1.12	1.17	1.09	1.32	1.07	0.97	1.01
Southeast Asia	All ages	1.32	1.37	1.12	1.15	1.22	1.13	1.16	1.20
Southern Latin America	All ages	1.14	1.08	1.20	1.16	1.22	1.03	0.94	0.98
Southern Sub-Saharan Africa	All ages	1.24	1.27	1.03	0.94	1.02	1.03	1.18	1.14
Tropical Latin America	All ages	1.37	1.16	1.32	1.26	1.21	1.05	1.23	1.09
Western Europe	All ages	0.99	0.97	1.06	1.04	0.98	1.01	0.80	0.89
Western Sub-Saharan Africa	All ages	1.22	1.22	0.86	0.92	1.07	1.05	1.42	1.45
Andean Latin America	Age-standardized	1.19	1.10	1.18	1.11	0.94	0.89	1.29	1.17
Australasia	Age-standardized	0.99	0.98	1.05	1.03	0.95	1.01	1.06	0.99
Caribbean	Age-standardized	1.21	1.22	1.26	1.28	0.95	0.93	1.33	1.30
Central Asia	Age-standardized	1.32	1.29	1.18	1.18	1.02	1.04	1.52	1.48
Central Europe	Age-standardized	1.24	1.16	1.30	1.32	1.00	1.01	1.72	1.29
Central Latin America	Age-standardized	1.14	1.16	1.14	1.18	0.95	0.99	1.27	1.20
Central Sub-Saharan Africa	Age-standardized	1.37	1.40	1.11	1.21	0.99	1.00	1.34	1.38
East Asia	Age-standardized	1.91	1.39	1.68	1.42	1.06	1.09	1.62	1.43
Eastern Europe	Age-standardized	1.36	1.26	1.18	1.25	1.10	1.08	2.78	1.55
Eastern Sub-Saharan Africa	Age-standardized	1.55	1.34	1.48	1.30	1.09	1.03	1.22	1.24
High-income Asia Pacific	Age-standardized	1.06	1.05	1.09	1.07	0.94	0.97	1.12	1.07
High-income North America	Age-standardized	1.04	1.00	1.08	1.03	0.99	1.07	1.09	1.00
North Africa and Middle East	Age-standardized	1.10	1.02	1.24	1.06	1.17	1.01	1.33	1.18
Oceania	Age-standardized	1.12	1.07	1.07	0.99	0.74	0.77	1.08	1.07
South Asia	Age-standardized	1.10	1.10	1.19	1.11	1.05	0.92	1.15	1.23
Southeast Asia	Age-standardized	1.29	1.33	1.20	1.24	1.02	1.00	1.37	1.40
Southern Latin America	Age-standardized	1.13	1.09	1.20	1.15	0.98	0.97	1.16	1.10
Southern Sub-Saharan Africa	Age-standardized	1.23	1.26	1.08	1.09	0.99	0.97	1.28	1.32
Tropical Latin America	Age-standardized	1.33	1.15	1.37	1.27	0.93	0.88	1.71	1.26
Western Europe	Age-standardized	1.08	1.02	1.11	1.07	0.91	0.97	1.21	1.05
Western Sub-Saharan Africa	Age-standardized	1.21	1.21	0.87	0.90	0.99	0.98	1.69	1.66
Andean Latin America	80 to 84	1.23	1.16	1.04	1.03	1.05	1.02	1.16	1.12
Australasia	80 to 84	1.09	1.04	1.02	1.01	1.06	1.10	1.06	1.02
Caribbean	80 to 84	1.24	1.21	1.05	1.07	1.09	1.09	1.14	1.13
Central Asia	80 to 84	1.49	1.39	1.08	1.03	1.14	1.05	1.34	1.30
Central Europe	80 to 84	1.40	1.18	1.24	1.15	1.10	1.09	1.25	1.12
Central Latin America	80 to 84	1.19	1.16	1.02	1.04	1.04	1.05	1.16	1.12
Central Sub-Saharan Africa	80 to 84	1.38	1.40	1.07	1.11	1.12	1.11	1.23	1.24
East Asia	80 to 84	1.33	1.25	1.36	1.15	0.20	1.07	1.11	1.15

Eastern Europe	80 to 84	1.67	1.47	1.08	1.19	1.31	1.24	1.46	1.35
Eastern Sub-Saharan Africa	80 to 84	1.38	1.29	1.30	1.12	1.18	1.11	1.16	1.16
High-income Asia Pacific	80 to 84	1.16	1.08	1.08	1.09	0.98	0.98	1.11	1.05
High-income North America	80 to 84	1.11	1.05	1.04	1.01	1.07	1.12	1.08	1.02
North Africa and Middle East	80 to 84	1.31	1.17	1.16	1.03	1.33	1.11	1.11	1.10
Oceania	80 to 84	1.21	1.19	1.09	1.03	0.97	0.96	1.06	1.06
South Asia	80 to 84	1.20	1.18	1.07	1.04	1.16	1.05	1.10	1.11
Southeast Asia	80 to 84	1.25	1.28	0.95	1.02	1.07	1.06	1.15	1.18
Southern Latin America	80 to 84	1.20	1.13	1.16	1.13	0.91	1.03	1.12	1.08
Southern Sub-Saharan Africa	80 to 84	1.21	1.22	0.99	0.98	1.06	1.05	1.17	1.16
Tropical Latin America	80 to 84	1.33	1.15	1.12	1.09	1.03	1.07	1.24	1.11
Western Europe	80 to 84	1.18	1.06	1.05	1.02	1.01	1.07	1.13	1.04
Western Sub-Saharan Africa	80 to 84	1.30	1.27	0.80	0.83	1.04	1.02	1.31	1.28
Andean Latin America	85 to 89	1.13	1.12	1.02	1.04	1.02	1.00	1.08	1.08
Australasia	85 to 89	1.07	1.03	1.01	1.02	1.01	1.05	1.03	1.00
Caribbean	85 to 89	1.21	1.17	1.05	1.07	1.02	1.04	1.14	1.11
Central Asia	85 to 89	1.53	1.41	1.18	1.14	1.00	0.92	1.36	1.30
Central Europe	85 to 89	1.48	1.21	1.27	1.19	0.97	0.95	1.34	1.14
Central Latin America	85 to 89	1.10	1.10	0.97	1.03	0.99	1.00	1.07	1.06
Central Sub-Saharan Africa	85 to 89	1.26	1.31	1.03	1.08	1.08	1.08	1.15	1.18
East Asia	85 to 89	1.70	1.51	1.78	3.92	0.29	1.13	1.23	1.24
Eastern Europe	85 to 89	1.82	1.36	1.18	1.11	0.97	0.87	1.61	1.27
Eastern Sub-Saharan Africa	85 to 89	1.27	1.25	1.20	1.15	1.13	1.10	1.11	1.13
High-income Asia Pacific	85 to 89	1.15	1.07	1.08	1.08	0.96	0.99	1.10	1.02
High-income North America	85 to 89	1.07	1.02	1.02	0.99	1.00	1.02	1.03	0.98
North Africa and Middle East	85 to 89	1.31	1.18	1.07	1.01	1.13	1.00	1.18	1.11
Oceania	85 to 89	1.22	1.19	1.37	1.31	0.65	0.71	1.04	1.03
South Asia	85 to 89	1.19	1.20	1.06	1.07	0.99	0.97	1.11	1.13
Southeast Asia	85 to 89	1.24	1.24	1.06	1.09	0.91	0.93	1.14	1.14
Southern Latin America	85 to 89	1.16	1.10	1.12	1.10	0.78	0.95	1.10	1.05
Southern Sub-Saharan Africa	85 to 89	1.19	1.17	1.04	1.03	1.05	1.02	1.13	1.11
Tropical Latin America	85 to 89	1.26	1.13	1.09	1.09	1.00	1.00	1.20	1.08
Western Europe	85 to 89	1.18	1.06	1.05	1.03	0.96	1.01	1.13	1.02
Western Sub-Saharan Africa	85 to 89	1.20	1.20	0.89	0.91	1.00	1.00	1.19	1.18
Andean Latin America	90 to 94	1.08	1.07	0.94	0.96	0.98	0.95	1.08	1.07
Australasia	90 to 94	1.02	1.00	0.95	0.95	0.94	0.98	1.03	1.01
Caribbean	90 to 94	1.14	1.10	0.93	0.97	0.99	1.01	1.13	1.10
Central Asia	90 to 94	1.48	1.30	0.98	1.00	0.90	0.83	1.45	1.28
Central Europe	90 to 94	1.39	1.14	1.00	1.00	0.86	0.84	1.31	1.12
Central Latin America	90 to 94	1.06	1.05	0.92	0.97	0.98	0.98	1.07	1.05
Central Sub-Saharan Africa	90 to 94	1.19	1.23	0.89	0.90	1.07	1.08	1.16	1.18
East Asia	90 to 94	1.99	1.72	6.99	3.03	0.62	1.06	1.35	1.37
Eastern Europe	90 to 94	1.55	1.22	0.98	0.96	0.72	0.66	1.44	1.21

Eastern Sub-Saharan Africa	90 to 94	1.16	1.14	0.92	0.91	1.09	1.06	1.10	1.10
High-income Asia Pacific	90 to 94	1.11	1.05	1.02	1.05	0.96	0.98	1.09	1.04
High-income North America	90 to 94	1.04	1.00	0.99	0.97	0.93	0.93	1.04	1.00
North Africa and Middle East	90 to 94	1.20	1.08	0.90	0.90	0.93	0.88	1.16	1.07
Oceania	90 to 94	1.15	1.10	0.97	0.92	0.71	0.75	1.07	1.03
South Asia	90 to 94	1.13	1.13	0.96	0.98	0.90	0.90	1.12	1.12
Southeast Asia	90 to 94	1.19	1.14	0.88	0.89	0.76	0.83	1.15	1.11
Southern Latin America	90 to 94	1.11	1.07	1.02	1.02	0.59	0.84	1.09	1.06
Southern Sub-Saharan Africa	90 to 94	1.18	1.13	0.95	0.91	1.02	0.99	1.17	1.11
Tropical Latin America	90 to 94	1.17	1.07	0.98	0.99	0.97	0.96	1.16	1.07
Western Europe	90 to 94	1.14	1.04	0.97	0.97	0.88	0.93	1.13	1.04
Western Sub-Saharan Africa	90 to 94	1.15	1.15	0.86	0.87	1.00	1.00	1.19	1.18
Andean Latin America	95 plus	1.07	1.05	1.07	1.05	1.05	1.02	1.14	1.10
Australasia	95 plus	1.04	1.00	1.09	1.08	1.09	1.11	1.10	1.06
Caribbean	95 plus	1.12	1.08	1.08	1.07	1.08	1.07	1.20	1.15
Central Asia	95 plus	1.40	1.28	1.17	1.20	1.15	1.15	1.53	1.35
Central Europe	95 plus	1.26	1.13	1.26	1.19	1.09	1.06	1.31	1.19
Central Latin America	95 plus	1.06	1.03	1.07	1.06	1.05	1.05	1.12	1.08
Central Sub-Saharan Africa	95 plus	1.18	1.17	1.13	1.11	1.19	1.17	1.21	1.20
East Asia	95 plus	1.20	1.24	2.29	3.59	1.11	1.24	1.15	1.20
Eastern Europe	95 plus	1.58	1.20	1.34	1.18	1.44	1.04	1.64	1.27
Eastern Sub-Saharan Africa	95 plus	1.15	1.11	1.19	1.10	1.23	1.15	1.15	1.13
High-income Asia Pacific	95 plus	1.08	1.02	1.13	1.10	1.03	1.00	1.13	1.06
High-income North America	95 plus	1.02	1.00	1.05	1.02	1.03	1.05	1.08	1.05
North Africa and Middle East	95 plus	1.20	1.06	1.13	1.04	1.18	1.04	1.27	1.13
Oceania	95 plus	1.17	1.09	1.65	1.33	0.98	0.98	1.11	1.07
South Asia	95 plus	1.13	1.12	1.12	1.14	1.12	1.08	1.19	1.18
Southeast Asia	95 plus	1.18	1.08	1.19	1.09	1.08	1.02	1.21	1.12
Southern Latin America	95 plus	1.09	1.03	1.13	1.06	0.71	0.89	1.15	1.08
Southern Sub-Saharan Africa	95 plus	1.14	1.09	1.09	1.05	1.07	1.06	1.19	1.14
Tropical Latin America	95 plus	1.14	1.04	1.11	1.08	1.04	1.03	1.22	1.10
Western Europe	95 plus	1.12	1.04	1.09	1.09	0.99	1.06	1.19	1.09
Western Sub-Saharan Africa	95 plus	1.13	1.11	1.01	1.01	1.09	1.06	1.23	1.20

ICH, intracerebral hemorrhage; SAH, subarachnoid hemorrhage; IS, ischemic stroke; GDR, gender disparity ratio

Table S19 The number of regions for GDR values near one for stroke, ICH, SAH, and IS in different age groups in 1990 and 2021

GDR range	cause	year	<5	5 to 9	10 to 14	15 to 19	20 to 24	25 to 29	30 to 34	35 to 39	40 to 44	45 to 49	50 to 54	55 to 59	60 to 64	65 to 69	70 to 74	75 to 79	80 to 84	85 to 89	90 to 94	95+
<0.95	stroke	1990	0	2	6	0	9	0	0	0	0	0	0	0	2	1	0	0	0	0	0	0
		2021	0	0	4	0	3	0	0	0	0	0	0	0	2	0	0	0	0	0	0	0
	ICH	1990	2	2	9	6	19	4	1	0	0	0	0	0	0	2	0	0	2	1	8	0

		2021	0	1	7	6	14	3	0	0	0	0	0	0	0	2	0	0	1	1	7	0
	SAH	1990	1	14	0	0	8	0	2	0	3	0	5	0	6	3	0	0	2	4	12	1
		2021	1	4	0	1	4	1	1	1	3	0	3	3	4	4	0	0	0	5	10	1
	IS	1990	3	0	0	2	0	2	1	1	1	3	2	2	12	3	0	0	0	0	0	0
		2021	0	0	0	1	0	1	0	0	1	1	2	3	8	3	0	0	0	0	0	0
[0.95,1.05]	stroke	1990	11	18	15	4	11	7	7	2	1	2	2	4	4	3	0	0	0	0	2	2
		2021	17	20	17	3	14	7	7	3	4	5	4	6	5	7	0	0	1	2	4	8
	ICH	1990	14	19	12	10	2	7	3	1	0	0	1	2	0	7	1	1	7	8	12	1
		2021	21	20	14	8	7	7	4	2	0	0	2	5	2	8	4	1	11	7	13	4
	SAH	1990	13	5	4	3	13	10	4	9	11	0	9	12	11	13	4	0	7	14	7	6
		2021	16	16	9	5	16	8	5	6	9	1	13	12	14	10	4	2	9	13	8	7
	IS	1990	17	10	7	4	1	6	6	3	1	5	8	8	4	5	1	0	0	3	2	0
		2021	20	15	10	6	3	8	4	3	3	8	9	7	9	5	5	2	4	6	5	1
>1.05	stroke	1990	10	1	0	17	1	14	14	19	20	19	19	17	15	17	21	21	21	21	19	19
		2021	4	1	0	18	4	14	14	18	17	16	17	15	14	14	21	21	20	19	17	13
	ICH	1990	5	0	0	5	0	10	17	20	21	21	20	19	21	12	20	20	12	12	1	20
		2021	0	0	0	7	0	11	17	19	21	21	19	16	19	11	17	20	9	13	1	17
	SAH	1990	7	2	17	18	0	11	15	12	7	21	7	9	4	5	17	21	12	3	2	14
		2021	4	1	12	15	1	12	15	14	9	20	5	6	3	7	17	19	12	3	3	13
	IS	1990	1	11	14	15	20	13	14	17	19	13	11	11	5	13	20	21	21	18	19	21
		2021	1	6	11	14	18	12	17	18	17	12	10	11	4	13	16	19	17	15	16	20

ICH, intracerebral hemorrhage; SAH, subarachnoid hemorrhage; IS, ischemic stroke; GDR, gender disparity ratio

Table S20 GDR values by age group and countries for stroke, ICH, SAH, and IS in 1990 and 2021

	GDR								
		**stroke**		**ICH**		**SAH**		**IS**	
**location**	**age**	**1990**	**2021**	**1990**	**2021**	**1990**	**2021**	**1990**	**2021**
Afghanistan	5	1.52	1.11	1.46	1.07	1.23	1.12	0.80	0.99
Albania	5	1.00	0.99	1.02	1.00	1.04	1.01	0.97	0.97
Algeria	5	1.09	0.99	1.05	0.99	1.07	1.01	0.90	0.99
American Samoa	5	0.99	0.98	0.98	0.99	0.99	0.99	0.99	0.99
Andorra	5	0.98	1.00	0.98	0.99	0.93	1.00	0.94	1.01
Angola	5	1.20	1.03	0.99	0.98	1.04	1.03	1.13	1.04
Antigua and Barbuda	5	1.00	0.99	0.99	0.98	1.01	1.00	1.00	1.00
Argentina	5	1.05	1.00	1.02	0.99	1.07	1.01	1.00	1.01
Armenia	5	1.03	1.00	1.03	0.99	1.02	1.01	1.01	1.01
Australia	5	1.01	0.99	1.00	0.99	1.04	1.01	1.02	1.01
Austria	5	1.02	0.99	1.01	0.99	1.01	1.00	1.02	1.01
Azerbaijan	5	1.05	1.01	1.06	0.99	1.01	1.01	1.03	1.02
Bahamas	5	1.00	0.99	0.99	0.99	1.01	1.01	1.00	1.00
Bahrain	5	1.02	1.01	1.04	1.00	1.06	1.01	0.96	1.00
Bangladesh	5	4.24	1.10	2.00	1.05	12.52	1.16	0.93	1.02
Barbados	5	1.00	0.99	0.99	0.98	1.00	1.00	0.99	1.00
Belarus	5	1.06	1.02	1.03	1.00	1.02	1.02	1.05	1.02
Belgium	5	1.03	1.00	1.03	1.00	1.03	1.00	1.02	1.02

Belize	5	1.07	1.00	1.04	0.99	1.05	1.01	1.00	1.01
Benin	5	1.13	1.09	0.96	0.97	1.07	1.05	1.33	1.20
Bermuda	5	0.99	0.98	0.99	0.98	1.00	1.00	1.00	1.00
Bhutan	5	1.45	1.06	1.28	1.03	1.81	1.08	0.96	1.03
Bolivia (Plurinational State of)	5	2.03	1.16	1.32	1.07	2.05	1.19	1.04	1.04
Bosnia and Herzegovina	5	1.03	1.01	1.00	0.99	1.03	1.01	1.02	1.02
Botswana	5	1.01	1.01	0.96	0.95	1.01	1.01	1.03	1.05
Brazil	5	1.04	1.03	1.03	1.01	1.01	1.01	1.01	1.03
Brunei Darussalam	5	1.04	1.02	1.02	1.01	1.04	1.03	1.02	1.02
Bulgaria	5	1.07	1.02	1.03	1.02	1.08	1.02	1.00	1.00
Burkina Faso	5	1.21	1.14	1.02	1.01	1.12	1.09	1.30	1.21
Burundi	5	1.10	1.04	0.98	1.00	1.17	1.07	0.96	1.00
Cabo Verde	5	1.08	1.02	0.97	0.97	1.07	1.03	1.34	1.07
Cambodia	5	1.14	1.04	1.10	1.02	1.03	1.03	0.94	0.99
Cameroon	5	1.21	1.13	1.01	0.99	1.12	1.09	1.31	1.26
Canada	5	0.98	0.99	1.00	0.99	1.03	1.03	0.98	1.00
Central African Republic	5	1.03	1.07	0.92	0.99	1.04	1.09	0.98	1.05
Chad	5	1.27	1.23	1.00	0.99	1.17	1.14	1.60	1.52
Chile	5	1.02	1.00	1.02	1.00	1.04	1.01	0.98	1.01
China	5	1.07	0.99	1.05	1.00	0.98	1.01	0.98	0.99
Colombia	5	1.03	1.01	1.01	1.00	1.03	1.03	1.00	1.00
Comoros	5	1.13	1.04	1.01	0.99	1.14	1.06	1.00	1.01
Congo	5	1.06	1.01	0.97	0.98	1.06	1.03	1.07	1.03
Cook Islands	5	0.99	0.98	0.98	0.98	0.96	0.99	0.98	0.99
Costa Rica	5	1.01	0.99	1.00	0.99	1.01	1.01	1.01	1.00
Coted'Ivoire	5	1.24	1.13	0.98	0.97	1.13	1.08	1.64	1.35
Croatia	5	1.03	1.00	1.01	0.99	1.05	1.01	1.02	1.01
Cuba	5	1.02	1.00	1.00	0.99	1.03	1.01	1.01	1.01
Cyprus	5	1.02	1.00	1.01	0.99	0.99	1.00	0.99	1.03
Czechia	5	1.03	1.00	1.01	0.99	1.05	1.01	1.02	1.02
Democratic People's Republic of Korea	5	1.03	1.00	1.01	1.00	1.03	1.01	0.99	1.00
Democratic Republic of the Congo	5	1.00	1.01	0.91	0.97	0.98	1.03	1.01	1.04
Denmark	5	1.04	1.00	1.04	0.99	1.00	1.00	1.01	1.01
Djibouti	5	1.11	1.03	1.03	0.99	1.15	1.05	1.03	1.02
Dominica	5	1.00	1.00	0.98	0.99	1.01	1.01	1.00	0.99
Dominican Republic	5	1.10	1.02	1.06	1.00	1.07	1.03	1.00	0.98
Ecuador	5	1.21	1.04	1.12	1.01	1.21	1.08	1.07	1.01
Egypt	5	46.59	1.01	2.13	0.98	#####	1.06	0.00	0.97
El Salvador	5	1.10	1.00	1.06	1.00	1.02	1.02	1.00	1.00
Equatorial Guinea	5	1.02	1.01	0.95	0.98	1.05	1.01	1.02	1.02
Eritrea	5	1.13	1.05	1.04	1.01	1.16	1.07	1.03	1.02
Estonia	5	1.06	1.02	1.02	0.99	1.04	1.01	1.06	1.02
Eswatini	5	1.06	1.04	0.99	1.01	1.01	1.01	1.04	1.03
Ethiopia	5	1.06	1.03	0.99	0.99	1.06	1.04	1.00	1.01
Fiji	5	1.01	1.01	0.99	1.00	1.02	0.99	1.00	1.00
Finland	5	1.00	0.99	1.00	0.99	0.99	0.99	1.00	1.01
France	5	1.02	1.00	1.01	1.00	1.01	1.00	1.02	1.02
Gabon	5	1.03	1.02	0.99	0.99	1.04	1.03	1.02	1.03

Gambia	5	1.17	1.07	0.99	0.98	1.10	1.05	1.38	1.15
Georgia	5	1.00	0.99	1.01	0.98	1.00	1.00	1.00	1.01
Germany	5	1.02	0.99	1.02	0.99	1.01	1.00	1.01	1.01
Ghana	5	1.20	1.08	0.99	0.99	1.12	1.06	1.62	1.14
Greece	5	1.04	1.00	1.05	1.00	1.01	1.00	1.01	1.01
Greenland	5	0.93	0.96	1.01	1.00	0.41	0.92	1.05	0.99
Grenada	5	1.00	0.98	0.99	0.99	1.00	1.00	0.97	0.99
Guam	5	1.01	1.00	0.99	0.99	0.99	0.99	1.01	1.01
Guatemala	5	1.04	1.04	1.04	1.03	1.02	1.05	0.96	1.00
Guinea	5	1.33	1.19	0.96	0.98	1.18	1.12	2.39	1.54
Guinea-Bissau	5	1.43	1.11	1.02	0.98	1.22	1.09	2.21	1.22
Guyana	5	1.09	1.02	1.03	1.00	1.11	1.03	0.97	0.99
Haiti	5	1.46	1.14	1.25	1.10	0.00	0.85	0.53	0.90
Honduras	5	1.05	1.02	1.04	1.01	1.06	1.04	0.96	1.00
Hungary	5	1.03	1.00	1.01	0.99	1.04	1.01	1.02	1.01
Iceland	5	1.02	1.00	1.01	0.99	1.00	1.00	1.01	1.02
India	5	1.18	1.06	1.11	1.04	1.26	1.08	0.97	1.02
Indonesia	5	1.15	1.11	1.11	1.11	1.01	1.05	0.96	1.00
Iran (Islamic Republic of)	5	1.03	0.99	1.03	0.99	1.01	1.01	0.93	1.00
Iraq	5	1.13	1.04	1.09	1.03	1.04	1.04	0.78	1.00
Ireland	5	1.03	1.00	1.01	0.99	1.05	1.01	1.02	1.02
Israel	5	1.01	0.99	1.05	0.99	1.01	1.00	0.99	1.01
Italy	5	1.00	1.00	1.01	1.00	1.01	1.01	0.98	1.02
Jamaica	5	1.02	1.00	0.98	0.99	1.03	1.01	0.92	1.00
Japan	5	1.02	1.00	1.02	1.00	1.02	1.01	1.02	1.02
Jordan	5	1.13	1.00	1.10	0.99	1.08	1.02	0.90	0.98
Kazakhstan	5	0.95	1.00	0.99	0.99	1.01	1.00	0.95	1.00
Kenya	5	1.02	1.01	0.98	0.99	1.02	1.02	1.01	1.01
Kiribati	5	1.08	1.04	0.97	1.00	1.09	1.01	1.00	1.01
Kuwait	5	1.06	1.03	1.09	1.03	1.03	1.04	0.97	1.01
Kyrgyzstan	5	1.03	1.00	1.03	0.98	1.02	1.00	1.00	1.01
Lao People's Democratic Republic	5	1.27	1.09	1.10	1.05	1.02	1.06	0.75	0.99
Latvia	5	1.05	1.01	1.03	0.99	1.04	1.02	1.03	1.02
Lebanon	5	1.01	0.99	1.00	0.99	1.02	1.02	0.93	1.00
Lesotho	5	1.04	1.05	1.00	1.01	1.02	1.03	1.03	1.04
Liberia	5	1.48	1.13	0.97	0.98	1.14	1.09	5.50	1.26
Libya	5	1.26	1.28	1.20	1.26	1.25	1.10	0.95	1.04
Lithuania	5	1.03	1.01	1.02	0.99	1.04	1.02	1.02	1.01
Luxembourg	5	1.04	1.00	1.03	0.99	1.02	0.99	1.02	1.02
Madagascar	5	1.20	1.09	1.05	1.03	1.23	1.14	1.06	1.03
Malawi	5	1.07	1.03	0.98	0.99	1.04	1.05	0.99	1.01
Malaysia	5	1.00	0.99	1.00	0.99	1.01	1.00	1.00	1.00
Maldives	5	1.02	1.00	1.00	1.00	1.01	1.01	0.95	1.00
Mali	5	1.27	1.13	1.00	0.98	1.13	1.08	1.45	1.25
Malta	5	1.04	1.00	1.01	0.99	1.03	1.00	1.03	1.02
Marshall Islands	5	1.02	1.01	1.00	1.00	1.02	1.00	1.00	1.00
Mauritania	5	1.12	1.06	0.98	0.98	1.10	1.05	1.27	1.13
Mauritius	5	1.00	0.99	0.99	0.99	1.01	1.01	0.98	1.00

Mexico	5	1.06	1.02	1.04	1.02	1.04	1.04	1.00	1.00
Micronesia (Federated States of)	5	1.01	1.01	0.99	1.00	1.00	1.00	0.99	1.00
Monaco	5	0.98	0.99	0.99	0.99	0.92	0.98	0.95	1.01
Mongolia	5	1.05	1.00	1.07	1.00	1.02	0.99	0.99	1.00
Montenegro	5	1.02	1.01	1.02	1.01	0.99	1.00	1.02	1.02
Morocco	5	1.10	0.99	1.03	0.98	1.13	1.02	0.79	0.98
Mozambique	5	1.26	1.04	1.15	1.01	1.14	1.06	1.09	1.03
Myanmar	5	1.50	1.11	1.24	1.08	1.20	1.07	0.89	1.00
Namibia	5	1.02	1.00	0.96	0.96	1.01	1.01	1.06	1.03
Nauru	5	1.01	1.01	0.99	0.99	0.97	0.96	1.00	1.00
Nepal	5	1.38	1.06	1.18	1.03	1.62	1.10	0.91	1.03
Netherlands	5	1.02	1.00	1.01	0.99	1.03	1.00	1.01	1.01
New Zealand	5	1.02	0.99	1.00	0.99	1.04	1.01	1.04	1.01
Nicaragua	5	1.22	1.01	1.17	1.00	1.06	1.02	1.00	1.01
Niger	5	1.23	1.09	0.93	0.97	1.12	1.08	1.53	1.16
Nigeria	5	1.28	1.18	1.02	1.00	1.10	1.07	1.37	1.39
Niue	5	1.04	1.08	1.01	0.99	1.05	1.00	1.02	1.09
North Macedonia	5	1.14	1.01	1.08	0.99	1.01	1.02	0.98	1.01
Northern Mariana Islands	5	1.00	0.98	0.99	0.98	1.00	1.00	1.01	1.00
Norway	5	1.02	1.00	1.01	1.00	1.04	1.00	1.03	1.01
Oman	5	1.04	0.99	1.16	1.01	1.07	1.01	0.84	0.98
Pakistan	5	1.26	1.10	1.17	1.05	1.50	1.13	0.93	0.99
Palau	5	1.02	1.01	0.98	0.99	0.99	0.98	0.99	1.00
Palestine	5	1.26	1.05	1.17	1.04	1.02	1.02	0.78	1.00
Panama	5	0.98	0.97	1.02	1.00	1.03	1.02	0.97	0.96
Papua New Guinea	5	0.97	0.99	0.91	0.94	0.91	0.93	0.88	0.95
Paraguay	5	0.99	1.00	0.98	0.99	0.94	1.00	0.99	1.01
Peru	5	1.15	1.03	1.10	1.01	1.09	1.03	1.01	1.02
Philippines	5	1.20	1.04	1.20	1.03	1.06	1.02	1.00	1.01
Poland	5	1.08	1.01	1.04	1.00	1.06	1.01	1.02	1.02
Portugal	5	1.06	0.99	1.03	0.99	1.01	1.00	0.93	1.01
Puerto Rico	5	1.00	0.99	0.99	0.98	1.01	1.01	1.00	1.00
Qatar	5	1.00	0.99	0.99	0.99	1.13	1.03	0.98	1.00
Republic of Korea	5	1.03	1.00	1.02	1.00	1.04	1.02	1.02	1.01
Republic of Moldova	5	1.11	1.03	1.12	1.01	1.07	1.02	1.04	1.02
Romania	5	1.11	1.01	1.08	1.00	1.04	0.99	1.02	1.01
Russian Federation	5	1.04	1.02	1.02	1.00	1.04	1.03	1.03	1.03
Rwanda	5	1.14	1.03	1.03	1.00	1.14	1.05	1.00	1.01
Saint Kitts and Nevis	5	1.02	1.00	1.01	0.99	1.02	1.01	0.97	1.00
Saint Lucia	5	1.01	0.99	1.00	0.99	1.00	1.00	1.00	1.00
Saint Vincent and the Grenadines	5	1.02	1.00	1.02	0.99	0.99	1.01	0.98	1.00
Samoa	5	1.02	1.00	1.01	1.00	1.00	1.00	1.00	1.01
San Marino	5	1.01	0.99	1.01	0.99	1.00	0.99	0.99	1.01
Sao Tome and Principe	5	1.16	1.04	1.01	0.99	1.10	1.04	1.36	1.09
Saudi Arabia	5	1.42	1.00	1.51	0.99	1.10	1.01	0.93	1.01
Senegal	5	1.26	1.08	0.97	0.98	1.13	1.07	1.69	1.14
Serbia	5	1.17	1.01	1.08	0.99	1.17	1.01	1.07	1.02
Seychelles	5	0.99	0.99	0.98	0.98	1.00	1.00	1.00	1.00

Sierra Leone	5	1.58	1.23	0.95	0.97	1.14	1.11	1.01	1.93
Singapore	5	1.03	1.00	1.02	0.99	1.02	1.00	1.02	1.02
Slovakia	5	1.03	1.01	1.01	1.00	1.03	1.01	1.01	1.01
Slovenia	5	1.01	1.00	1.00	0.99	1.02	1.01	1.02	1.02
Solomon Islands	5	1.00	1.00	0.97	0.99	0.98	0.99	0.98	1.00
Somalia	5	1.16	1.06	1.06	1.02	1.15	1.08	1.01	1.01
South Africa	5	1.00	1.02	0.93	0.99	0.99	1.01	1.03	1.02
South Sudan	5	1.34	1.14	1.12	1.04	1.20	1.09	1.02	1.00
Spain	5	1.02	0.99	1.00	1.00	1.02	1.00	1.02	1.01
Sri Lanka	5	1.01	0.99	1.00	0.99	1.00	1.00	1.00	1.00
Sudan	5	1.01	1.06	1.01	1.06	1.03	1.05	0.38	0.97
Suriname	5	1.08	1.02	1.05	1.00	1.04	1.02	1.00	1.00
Sweden	5	1.00	1.00	1.01	1.00	0.95	1.00	1.03	1.01
Switzerland	5	1.03	1.00	1.02	0.99	1.01	1.00	1.01	1.02
Syrian Arab Republic	5	1.20	1.04	1.20	1.05	1.10	1.03	0.82	1.00
Taiwan (Province of China)	5	1.00	0.99	1.00	0.99	1.00	1.00	1.01	1.00
Tajikistan	5	1.13	1.05	1.18	1.05	1.03	1.03	1.02	1.02
Thailand	5	1.03	1.00	1.00	1.00	1.04	1.01	1.01	1.00
Timor-Leste	5	1.48	1.12	1.31	1.08	1.04	1.07	0.87	1.00
Togo	5	1.21	1.09	0.98	0.98	1.12	1.07	1.53	1.20
Tokelau	5	0.97	1.00	0.97	0.98	0.91	0.89	0.97	1.01
Tonga	5	1.01	1.00	0.99	0.99	0.97	0.98	1.01	1.01
Trinidad and Tobago	5	1.02	1.00	1.00	0.99	1.02	1.01	0.99	1.00
Tunisia	5	1.00	1.00	0.99	0.99	1.01	1.01	0.87	1.00
Turkey	5	1.06	1.00	1.00	0.99	1.54	1.03	0.85	1.00
Turkmenistan	5	1.06	1.00	1.08	1.00	1.03	1.01	1.01	1.00
Tuvalu	5	1.16	1.02	1.02	1.00	1.12	1.00	1.03	1.01
Uganda	5	1.14	1.05	1.02	1.01	1.14	1.07	0.99	1.01
Ukraine	5	1.03	1.01	1.01	1.00	1.01	1.01	1.05	1.02
United Arab Emirates	5	1.05	1.00	1.05	1.00	1.07	1.02	1.02	1.00
United Kingdom	5	1.01	1.00	1.00	0.99	1.01	1.00	1.00	1.01
United Republic of Tanzania	5	1.08	1.03	1.01	0.99	1.07	1.06	1.00	1.02
United States Virgin Islands	5	1.01	0.99	1.00	0.99	1.02	1.00	1.00	1.00
United States of America	5	1.00	1.00	1.02	1.01	1.05	1.05	0.99	1.00
Uruguay	5	1.06	1.00	1.02	0.99	1.04	1.01	1.00	1.01
Uzbekistan	5	1.06	1.02	1.08	1.00	1.02	1.02	1.02	1.02
Vanuatu	5	1.02	1.01	0.98	0.99	1.01	0.99	0.99	1.00
Venezuela (Bolivarian Republic of)	5	1.07	1.03	1.05	1.01	1.03	1.05	1.01	1.00
Viet Nam	5	1.02	0.99	1.00	0.99	1.03	1.01	0.98	0.98
Yemen	5	0.93	0.99	0.92	0.98	1.04	1.01	0.61	0.93
Zambia	5	1.10	1.03	1.00	0.99	1.06	1.03	1.02	1.02
Zimbabwe	5	1.06	1.04	1.01	0.99	1.00	0.99	1.04	1.03
Afghanistan	5 to 9	1.03	1.05	1.06	1.05	1.04	1.06	1.03	1.13
Albania	5 to 9	0.98	1.00	1.01	0.99	0.93	1.00	1.00	1.02
Algeria	5 to 9	0.90	0.97	0.91	0.97	0.88	0.97	1.02	1.01
American Samoa	5 to 9	1.00	1.00	0.93	0.95	1.10	1.08	1.04	1.02
Andorra	5 to 9	0.95	0.99	0.96	0.98	0.77	0.97	1.01	1.02
Angola	5 to 9	0.95	0.99	0.93	0.97	0.92	0.99	1.09	1.06

Antigua and Barbuda	5 to 9	0.99	1.00	0.98	0.99	0.99	1.02	0.99	0.99
Argentina	5 to 9	0.98	0.99	1.00	0.99	0.86	0.98	1.03	1.02
Armenia	5 to 9	1.00	0.99	0.99	0.98	0.97	1.00	1.10	1.03
Australia	5 to 9	0.98	0.99	0.98	0.99	0.97	1.00	1.02	1.01
Austria	5 to 9	1.00	0.99	0.99	0.98	0.98	1.00	1.08	1.02
Azerbaijan	5 to 9	0.98	0.99	0.99	0.99	0.90	0.94	1.02	1.02
Bahamas	5 to 9	1.02	1.00	1.01	0.99	1.03	1.02	1.01	1.00
Bahrain	5 to 9	1.05	1.00	1.04	1.00	1.05	1.00	1.04	1.02
Bangladesh	5 to 9	1.09	1.02	1.13	1.01	0.99	0.92	1.32	1.15
Barbados	5 to 9	0.97	1.00	0.97	0.99	0.94	1.01	0.96	1.00
Belarus	5 to 9	0.99	0.99	0.97	0.98	0.94	1.00	1.05	1.02
Belgium	5 to 9	0.97	1.00	0.97	0.99	0.89	0.99	1.06	1.03
Belize	5 to 9	1.05	1.01	1.05	0.99	1.01	1.04	1.03	1.01
Benin	5 to 9	1.05	1.03	1.02	1.00	1.01	1.01	1.35	1.34
Bermuda	5 to 9	1.00	1.00	0.99	0.99	1.02	1.04	1.00	1.00
Bhutan	5 to 9	0.98	1.01	0.97	0.99	0.95	1.01	1.04	1.09
Bolivia (Plurinational State of)	5 to 9	0.85	1.00	0.92	1.00	0.17	0.84	1.17	1.09
Bosnia and Herzegovina	5 to 9	1.01	1.00	1.00	0.99	1.02	1.00	1.07	1.04
Botswana	5 to 9	1.02	1.01	1.00	0.99	1.00	1.00	1.04	1.05
Brazil	5 to 9	0.99	0.99	0.99	0.98	0.85	0.92	1.07	1.04
Brunei Darussalam	5 to 9	1.05	0.99	1.03	0.99	1.07	0.99	1.09	1.02
Bulgaria	5 to 9	0.98	1.00	1.00	1.00	0.95	0.99	1.06	1.03
Burkina Faso	5 to 9	1.07	1.05	1.04	1.03	1.04	1.03	1.26	1.29
Burundi	5 to 9	0.97	0.97	0.95	0.95	0.98	0.98	1.04	1.02
Cabo Verde	5 to 9	1.02	1.03	1.00	1.00	1.02	1.03	1.26	1.36
Cambodia	5 to 9	0.97	1.00	0.99	1.00	0.82	0.96	1.05	1.03
Cameroon	5 to 9	1.12	1.08	1.06	1.01	1.07	1.06	1.49	1.68
Canada	5 to 9	0.99	1.00	0.99	0.99	0.95	1.01	1.01	1.00
Central African Republic	5 to 9	0.97	1.01	0.94	0.98	0.96	1.03	1.10	1.10
Chad	5 to 9	1.12	1.12	1.05	1.03	1.07	1.08	1.69	2.50
Chile	5 to 9	0.99	1.00	0.99	0.99	0.94	1.00	1.07	1.04
China	5 to 9	1.02	1.01	1.02	1.00	0.81	0.99	1.06	1.04
Colombia	5 to 9	0.97	1.00	0.99	0.99	0.83	0.94	1.05	1.03
Comoros	5 to 9	0.99	0.98	0.97	0.96	0.99	0.99	1.07	1.04
Congo	5 to 9	1.02	1.00	0.99	0.98	1.02	1.01	1.12	1.06
Cook Islands	5 to 9	0.97	0.98	0.85	0.94	1.11	1.04	1.08	1.00
Costa Rica	5 to 9	0.99	1.00	0.99	0.99	0.98	1.01	1.01	1.01
Coted'Ivoire	5 to 9	1.11	1.09	1.04	1.01	1.06	1.06	2.03	2.44
Croatia	5 to 9	1.00	0.99	1.01	0.99	0.94	0.98	1.04	1.02
Cuba	5 to 9	1.00	1.01	0.99	1.00	1.01	1.04	1.01	1.01
Cyprus	5 to 9	0.99	1.00	1.01	0.99	0.86	1.00	1.04	1.02
Czechia	5 to 9	1.00	1.00	0.99	0.99	0.98	0.99	1.06	1.02
Democratic People's Republic of Korea	5 to 9	0.99	1.00	1.00	0.99	0.94	1.00	1.01	1.02
Democratic Republic of the Congo	5 to 9	0.91	0.98	0.89	0.96	0.91	0.99	1.06	1.05
Denmark	5 to 9	0.97	1.00	0.99	0.99	0.77	1.01	1.08	1.02
Djibouti	5 to 9	1.00	1.00	0.98	0.99	1.01	1.02	1.05	1.05
Dominica	5 to 9	1.00	1.01	0.99	1.00	1.01	1.02	1.00	1.00
Dominican Republic	5 to 9	1.06	1.04	1.08	1.03	0.93	1.01	1.03	1.03

Ecuador	5 to 9	1.01	1.00	1.02	0.98	0.88	0.92	1.22	1.05
Egypt	5 to 9	1.01	1.01	1.01	1.02	0.85	1.02	1.01	1.13
El Salvador	5 to 9	0.96	1.00	1.00	0.99	0.71	0.95	1.12	1.04
Equatorial Guinea	5 to 9	0.97	0.98	0.94	0.96	0.97	0.97	1.09	1.06
Eritrea	5 to 9	1.03	1.01	1.00	0.99	1.05	1.02	1.05	1.05
Estonia	5 to 9	0.99	0.99	0.97	0.97	0.99	1.00	1.05	1.02
Eswatini	5 to 9	1.06	1.05	1.05	1.04	1.01	1.01	1.05	1.07
Ethiopia	5 to 9	1.00	0.99	0.99	0.97	1.01	0.99	1.03	1.03
Fiji	5 to 9	0.98	0.98	0.88	0.86	1.13	1.15	1.06	1.09
Finland	5 to 9	0.98	0.99	0.99	0.98	0.79	0.97	1.09	1.02
France	5 to 9	1.00	0.99	0.99	0.99	0.95	0.98	1.04	1.02
Gabon	5 to 9	0.99	1.01	0.97	0.99	0.99	1.01	1.05	1.05
Gambia	5 to 9	1.09	1.07	1.03	1.01	1.05	1.06	1.62	1.60
Georgia	5 to 9	0.97	0.99	0.97	0.98	0.91	1.00	1.00	1.02
Germany	5 to 9	0.99	1.00	0.99	0.99	0.82	1.00	1.09	1.02
Ghana	5 to 9	1.12	1.04	1.04	1.00	1.08	1.03	2.38	1.34
Greece	5 to 9	0.97	0.99	0.97	0.99	0.89	1.00	1.07	1.02
Greenland	5 to 9	1.04	0.98	1.03	1.00	0.61	0.67	1.37	1.01
Grenada	5 to 9	0.97	1.01	0.98	1.00	0.93	1.02	0.95	1.00
Guam	5 to 9	1.03	1.01	0.94	0.98	1.15	1.08	1.11	1.01
Guatemala	5 to 9	0.94	0.98	0.99	0.99	0.88	0.91	1.10	1.02
Guinea	5 to 9	1.17	1.10	1.07	1.02	1.08	1.06	2.79	2.79
Guinea-Bissau	5 to 9	1.20	1.12	1.08	1.04	1.12	1.11	3.24	2.28
Guyana	5 to 9	1.01	1.02	1.02	1.01	0.95	1.02	1.00	1.01
Haiti	5 to 9	0.84	0.92	1.03	1.04	0.00	0.19	0.78	0.90
Honduras	5 to 9	0.83	1.01	0.95	1.00	0.26	0.99	1.04	1.04
Hungary	5 to 9	1.00	1.00	0.99	0.99	0.96	1.00	1.05	1.02
Iceland	5 to 9	0.93	0.99	0.96	0.98	0.63	1.01	1.02	1.02
India	5 to 9	0.96	0.99	0.95	0.98	0.90	0.99	1.04	1.03
Indonesia	5 to 9	0.95	1.03	0.97	1.02	0.65	0.91	1.08	1.10
Iran (Islamic Republic of)	5 to 9	1.00	1.00	1.01	0.99	0.90	1.00	1.21	1.08
Iraq	5 to 9	0.88	1.01	0.93	1.00	0.74	0.99	1.26	1.07
Ireland	5 to 9	1.00	1.00	0.99	0.99	0.90	1.02	1.07	1.02
Israel	5 to 9	0.97	0.99	0.98	0.99	0.91	0.99	1.00	1.02
Italy	5 to 9	0.98	0.99	0.99	0.98	0.83	0.96	1.04	1.02
Jamaica	5 to 9	0.97	1.01	0.98	1.00	0.92	1.04	0.95	1.01
Japan	5 to 9	0.99	1.00	0.99	0.99	0.97	0.99	1.04	1.02
Jordan	5 to 9	0.97	1.03	1.01	1.06	0.99	1.01	1.20	1.15
Kazakhstan	5 to 9	0.99	0.99	0.99	0.98	0.94	0.99	1.02	1.02
Kenya	5 to 9	0.99	0.99	0.97	0.98	0.99	1.00	1.03	1.03
Kiribati	5 to 9	1.07	1.09	0.86	0.89	1.72	1.52	1.21	1.25
Kuwait	5 to 9	1.05	1.05	1.09	1.06	0.92	1.04	1.13	1.16
Kyrgyzstan	5 to 9	0.97	0.99	0.98	0.98	0.87	0.97	1.03	1.03
Lao People's Democratic Republic	5 to 9	0.97	1.03	1.00	1.02	0.48	0.91	1.21	1.12
Latvia	5 to 9	0.99	0.99	0.98	0.98	1.01	0.99	1.04	1.02
Lebanon	5 to 9	1.00	1.01	1.00	1.00	0.98	1.01	1.01	1.02
Lesotho	5 to 9	1.01	1.08	0.99	1.08	1.00	1.01	1.03	1.07
Liberia	5 to 9	1.20	1.09	1.08	1.01	1.08	1.08	6.49	2.17

Libya	5 to 9	0.99	1.03	1.00	1.05	0.94	0.93	1.07	1.35
Lithuania	5 to 9	0.99	0.99	0.97	0.98	0.97	0.99	1.03	1.02
Luxembourg	5 to 9	0.91	1.01	0.93	1.00	0.73	1.03	0.96	1.03
Madagascar	5 to 9	1.04	1.01	1.00	0.99	1.03	1.03	1.13	1.08
Malawi	5 to 9	0.97	0.99	0.96	0.97	0.96	0.99	1.06	1.04
Malaysia	5 to 9	0.98	0.99	0.99	0.99	0.95	0.99	1.04	1.01
Maldives	5 to 9	0.88	1.00	0.93	0.99	0.71	0.98	1.00	1.03
Mali	5 to 9	1.11	1.07	1.06	1.04	1.05	1.03	1.45	1.49
Malta	5 to 9	0.97	1.01	0.98	1.00	0.90	1.01	1.08	1.04
Marshall Islands	5 to 9	1.01	1.03	0.92	0.91	1.17	1.25	1.03	1.07
Mauritania	5 to 9	1.08	1.04	1.02	1.00	1.05	1.04	1.58	1.36
Mauritius	5 to 9	0.99	1.00	0.98	0.99	0.97	1.00	1.02	1.03
Mexico	5 to 9	0.99	1.00	1.00	0.99	0.91	0.95	1.07	1.02
Micronesia (Federated States of)	5 to 9	1.02	1.01	0.88	0.93	1.36	1.18	1.08	1.04
Monaco	5 to 9	0.97	0.98	0.98	0.98	0.80	0.92	1.03	1.01
Mongolia	5 to 9	1.00	1.00	1.00	0.99	0.89	1.00	1.00	1.02
Montenegro	5 to 9	0.98	1.00	1.00	1.00	0.95	1.00	1.03	1.02
Morocco	5 to 9	0.78	0.97	0.78	0.96	0.75	0.96	0.92	1.00
Mozambique	5 to 9	1.04	1.02	1.03	1.01	1.05	1.05	1.09	1.07
Myanmar	5 to 9	1.02	1.04	1.02	1.04	0.66	0.94	1.18	1.11
Namibia	5 to 9	1.02	1.02	1.00	1.00	1.00	1.00	1.05	1.05
Nauru	5 to 9	1.02	1.01	0.88	0.85	1.40	1.31	1.16	1.16
Nepal	5 to 9	0.98	1.02	0.97	1.00	0.97	1.05	1.06	1.08
Netherlands	5 to 9	1.00	0.99	0.99	0.98	0.89	0.97	1.05	1.02
New Zealand	5 to 9	0.98	0.99	0.98	0.98	0.88	0.97	1.03	1.02
Nicaragua	5 to 9	0.97	1.00	0.99	0.99	0.79	1.00	1.10	1.03
Niger	5 to 9	1.14	1.04	1.06	1.01	1.08	1.03	1.63	1.27
Nigeria	5 to 9	1.10	1.10	1.05	1.04	1.04	1.05	1.46	2.01
Niue	5 to 9	1.00	0.82	0.88	0.49	1.27	1.01	1.07	1.33
North Macedonia	5 to 9	1.00	1.00	0.99	0.99	0.86	0.99	1.08	1.03
Northern Mariana Islands	5 to 9	1.01	1.00	0.96	0.97	1.09	1.07	1.03	1.01
Norway	5 to 9	1.00	1.00	0.97	0.98	0.90	1.01	1.12	1.02
Oman	5 to 9	1.01	1.00	1.10	0.99	0.98	1.02	1.01	1.02
Pakistan	5 to 9	0.95	0.97	0.95	0.95	0.88	0.81	1.03	1.12
Palau	5 to 9	1.00	0.98	0.89	0.92	1.14	1.02	1.11	1.03
Palestine	5 to 9	1.06	1.03	1.13	1.05	0.97	1.01	1.31	1.10
Panama	5 to 9	1.01	0.99	1.03	0.99	0.98	0.96	0.99	0.98
Papua New Guinea	5 to 9	0.95	0.95	0.81	0.81	1.17	1.14	1.03	1.03
Paraguay	5 to 9	0.93	0.99	0.96	0.98	0.64	0.92	1.02	1.02
Peru	5 to 9	0.96	1.00	0.99	1.00	0.79	0.87	1.04	1.05
Philippines	5 to 9	1.08	1.01	1.11	1.01	0.76	0.84	1.23	1.10
Poland	5 to 9	1.00	0.99	0.99	0.99	0.96	0.99	1.02	1.02
Portugal	5 to 9	0.91	0.98	0.95	0.99	0.59	0.93	1.17	1.02
Puerto Rico	5 to 9	1.01	1.00	0.99	0.99	1.02	1.03	1.00	1.00
Qatar	5 to 9	1.04	1.00	1.01	0.99	1.16	1.03	1.01	1.01
Republic of Korea	5 to 9	0.98	1.00	1.01	0.99	0.76	1.00	1.11	1.02
Republic of Moldova	5 to 9	0.98	0.99	0.97	0.98	0.99	0.99	1.03	1.02
Romania	5 to 9	1.00	0.99	1.00	0.99	0.89	0.97	1.07	1.02

Russian Federation	5 to 9	0.99	1.00	0.97	0.98	0.98	0.99	1.04	1.03
Rwanda	5 to 9	0.97	0.99	0.96	0.98	1.01	1.01	1.05	1.03
Saint Kitts and Nevis	5 to 9	1.00	1.02	1.00	1.01	0.93	1.04	0.99	1.01
Saint Lucia	5 to 9	1.00	1.01	0.99	1.00	0.99	1.01	1.00	1.00
Saint Vincent and the Grenadines	5 to 9	1.00	0.97	1.02	0.97	0.94	0.92	0.99	0.96
Samoa	5 to 9	1.00	1.00	0.90	0.94	1.21	1.11	1.05	1.03
San Marino	5 to 9	0.98	0.99	0.97	0.98	0.94	1.00	1.04	1.02
Sao Tome and Principe	5 to 9	1.20	1.02	1.09	1.00	1.12	1.03	4.73	1.19
Saudi Arabia	5 to 9	0.98	1.00	1.00	0.99	0.97	1.01	1.12	1.02
Senegal	5 to 9	1.10	1.05	1.03	1.00	1.06	1.05	1.83	1.43
Serbia	5 to 9	1.01	1.00	1.01	0.99	0.80	0.99	1.12	1.02
Seychelles	5 to 9	0.98	0.98	0.97	0.97	0.95	0.97	1.01	0.99
Sierra Leone	5 to 9	1.17	1.11	1.07	1.02	1.06	1.06	5.53	5.16
Singapore	5 to 9	0.98	1.00	0.98	0.99	0.97	1.01	1.00	1.02
Slovakia	5 to 9	1.00	1.00	0.99	0.99	0.95	0.99	1.04	1.02
Slovenia	5 to 9	1.00	0.99	0.99	0.99	0.98	0.99	1.04	1.02
Solomon Islands	5 to 9	0.99	0.99	0.93	0.93	1.10	1.11	1.00	1.00
Somalia	5 to 9	1.01	1.01	0.99	1.00	1.03	1.04	1.03	1.03
South Africa	5 to 9	1.04	1.01	1.04	1.00	0.97	0.98	1.07	1.04
South Sudan	5 to 9	1.00	0.99	0.98	0.98	0.99	0.99	1.05	1.06
Spain	5 to 9	0.98	0.99	1.00	0.99	0.90	0.98	1.07	1.02
Sri Lanka	5 to 9	0.97	0.99	0.97	0.98	0.92	0.99	1.08	1.03
Sudan	5 to 9	0.69	0.94	0.78	0.96	0.68	0.87	0.89	1.07
Suriname	5 to 9	1.02	1.00	1.04	1.00	0.94	0.95	1.00	0.99
Sweden	5 to 9	0.99	0.99	1.00	0.99	0.92	0.98	1.03	1.02
Switzerland	5 to 9	0.97	0.99	0.97	0.98	0.81	0.95	1.05	1.02
Syrian Arab Republic	5 to 9	0.89	1.00	1.17	1.02	0.89	0.97	0.99	1.03
Taiwan (Province of China)	5 to 9	1.00	1.00	1.00	1.00	1.00	1.01	1.02	1.01
Tajikistan	5 to 9	0.97	0.99	0.99	0.99	0.79	0.87	1.12	1.08
Thailand	5 to 9	0.95	1.00	0.96	0.99	0.65	0.98	1.07	1.03
Timor-Leste	5 to 9	1.00	1.03	1.01	1.02	0.76	0.94	1.11	1.10
Togo	5 to 9	1.09	1.06	1.02	1.01	1.05	1.05	1.82	1.60
Tokelau	5 to 9	1.01	0.90	0.91	0.64	1.16	1.33	1.06	1.20
Tonga	5 to 9	1.00	0.99	0.95	0.95	1.09	1.05	1.04	1.02
Trinidad and Tobago	5 to 9	1.02	1.01	1.01	0.99	1.01	1.03	1.04	1.01
Tunisia	5 to 9	0.89	0.98	0.90	0.97	0.88	0.99	0.99	1.01
Turkey	5 to 9	1.00	1.00	1.00	0.99	0.96	1.00	1.07	1.01
Turkmenistan	5 to 9	0.99	1.00	0.98	0.98	0.90	0.98	1.05	1.03
Tuvalu	5 to 9	1.12	1.03	0.84	0.91	2.35	1.31	1.30	1.11
Uganda	5 to 9	1.01	1.00	0.99	0.98	1.02	1.01	1.04	1.04
Ukraine	5 to 9	1.00	0.99	0.98	0.98	0.99	1.00	1.08	1.04
United Arab Emirates	5 to 9	1.05	0.99	1.07	0.99	1.01	1.00	1.11	1.01
United Kingdom	5 to 9	0.99	0.99	0.98	0.98	0.87	0.99	1.07	1.02
United Republic of Tanzania	5 to 9	1.00	1.00	0.99	0.99	1.01	1.01	1.04	1.06
United States Virgin Islands	5 to 9	1.01	1.00	1.00	0.99	1.02	1.03	1.01	1.00
United States of America	5 to 9	1.00	1.00	1.00	1.01	0.96	1.00	1.00	1.00
Uruguay	5 to 9	0.98	0.99	0.98	0.98	0.79	0.95	1.07	1.02
Uzbekistan	5 to 9	0.99	1.00	0.99	0.99	0.93	0.97	1.07	1.05

Vanuatu	5 to 9	1.00	1.00	0.89	0.89	1.24	1.20	1.07	1.07
Venezuela (Bolivarian Republic of)	5 to 9	1.00	1.00	1.00	1.00	0.91	0.94	1.05	1.02
Viet Nam	5 to 9	1.01	1.01	0.99	0.99	1.01	1.01	1.02	1.03
Yemen	5 to 9	0.80	0.93	0.84	0.94	0.83	0.91	0.91	1.01
Zambia	5 to 9	0.98	1.00	0.96	0.98	0.99	1.01	1.03	1.04
Zimbabwe	5 to 9	1.00	1.02	0.98	1.00	1.00	0.97	1.03	1.05
Afghanistan	10 to 14	0.90	0.96	0.85	0.92	1.19	1.13	0.93	1.06
Albania	10 to 14	0.99	1.00	0.96	0.98	1.19	1.08	1.08	1.06
Algeria	10 to 14	0.87	0.97	0.83	0.96	1.14	1.02	1.03	1.04
American Samoa	10 to 14	0.95	0.93	0.96	0.94	1.01	0.98	1.06	1.01
Andorra	10 to 14	0.94	0.98	0.95	0.98	1.01	0.99	1.04	1.01
Angola	10 to 14	0.88	0.95	0.79	0.89	1.11	1.06	1.08	1.07
Antigua and Barbuda	10 to 14	0.98	0.97	0.98	0.99	1.08	1.01	1.00	0.93
Argentina	10 to 14	0.99	1.00	0.99	0.99	1.46	1.10	1.08	1.03
Armenia	10 to 14	1.02	1.01	0.98	0.98	1.05	1.02	1.17	1.06
Australia	10 to 14	0.97	0.98	0.98	1.00	1.17	1.02	1.06	1.01
Austria	10 to 14	1.00	1.00	1.00	0.99	1.08	1.02	1.11	1.02
Azerbaijan	10 to 14	1.00	1.00	0.94	0.96	1.01	1.01	1.12	1.08
Bahamas	10 to 14	0.97	1.00	0.98	1.00	1.05	1.04	1.00	1.01
Bahrain	10 to 14	1.13	1.02	1.09	1.01	1.21	1.04	1.15	1.06
Bangladesh	10 to 14	1.56	1.31	1.22	1.15	41.76	2.60	1.48	1.50
Barbados	10 to 14	0.97	0.99	0.97	0.99	1.08	1.01	1.02	0.99
Belarus	10 to 14	1.01	1.00	0.99	0.98	1.02	1.00	1.10	1.03
Belgium	10 to 14	0.97	0.98	0.96	0.99	1.12	1.00	1.11	1.02
Belize	10 to 14	0.99	1.00	0.98	1.00	1.17	1.04	1.05	1.02
Benin	10 to 14	0.90	0.91	0.87	0.86	1.00	1.00	1.12	1.15
Bermuda	10 to 14	0.99	1.00	0.99	0.99	1.02	1.00	1.00	1.00
Bhutan	10 to 14	0.95	1.02	0.92	0.98	1.06	1.12	1.01	1.10
Bolivia (Plurinational State of)	10 to 14	0.74	0.98	0.78	0.95	2.58	1.38	0.92	1.00
Bosnia and Herzegovina	10 to 14	1.00	1.00	0.96	0.97	1.28	1.08	1.15	1.11
Botswana	10 to 14	1.03	1.02	0.96	0.98	1.04	1.03	1.13	1.15
Brazil	10 to 14	1.00	1.00	0.97	0.98	1.24	1.23	1.23	1.07
Brunei Darussalam	10 to 14	1.17	0.98	1.08	0.98	2.03	1.04	1.61	1.03
Bulgaria	10 to 14	1.02	1.01	0.97	0.99	1.30	1.10	1.51	1.12
Burkina Faso	10 to 14	0.97	0.94	0.95	0.91	1.02	1.02	1.10	1.14
Burundi	10 to 14	0.78	0.89	0.67	0.82	1.20	1.03	1.01	1.00
Cabo Verde	10 to 14	0.95	0.98	0.92	0.94	1.03	1.04	1.19	1.33
Cambodia	10 to 14	0.95	1.01	0.93	0.98	1.15	1.10	1.07	1.07
Cameroon	10 to 14	0.92	0.91	0.86	0.84	1.07	1.06	1.22	1.36
Canada	10 to 14	0.97	0.99	0.98	1.00	1.09	1.11	1.05	1.02
Central African Republic	10 to 14	0.87	0.94	0.74	0.84	1.20	1.16	1.10	1.10
Chad	10 to 14	0.89	0.83	0.83	0.75	1.06	1.06	1.25	1.44
Chile	10 to 14	1.00	0.99	1.00	0.99	1.33	1.07	1.10	1.03
China	10 to 14	1.00	1.02	1.00	1.01	1.21	1.06	1.15	1.11
Colombia	10 to 14	0.94	1.04	0.96	1.00	1.14	1.35	1.19	1.12
Comoros	10 to 14	0.88	0.93	0.78	0.87	1.09	1.03	1.04	1.03
Congo	10 to 14	0.95	0.95	0.89	0.90	1.09	1.05	1.10	1.06
Cook Islands	10 to 14	0.77	0.90	0.84	0.92	0.95	0.95	1.00	0.92

Costa Rica	10 to 14	1.01	1.02	0.99	0.99	1.07	1.09	1.07	1.03
Coted'Ivoire	10 to 14	0.94	0.92	0.88	0.84	1.09	1.07	1.54	1.71
Croatia	10 to 14	1.01	0.99	1.01	0.99	1.16	1.00	1.15	1.03
Cuba	10 to 14	1.01	1.02	1.00	1.01	1.11	1.07	1.08	1.03
Cyprus	10 to 14	0.98	0.94	1.01	0.97	1.11	0.97	1.05	1.00
Czechia	10 to 14	1.03	1.01	0.99	0.99	1.18	1.04	1.19	1.06
Democratic People's Republic of Korea	10 to 14	0.96	1.00	0.98	0.99	1.09	1.06	1.09	1.10
Democratic Republic of the Congo	10 to 14	0.84	0.94	0.76	0.87	1.07	1.05	1.05	1.05
Denmark	10 to 14	0.97	0.99	0.99	0.99	1.22	1.05	1.18	1.02
Djibouti	10 to 14	0.95	0.99	0.89	0.95	1.09	1.08	1.04	1.06
Dominica	10 to 14	0.99	1.00	0.98	0.98	1.07	1.10	1.01	1.03
Dominican Republic	10 to 14	1.01	1.04	1.00	1.01	1.38	1.24	1.14	1.13
Ecuador	10 to 14	1.04	0.94	0.99	0.96	1.50	1.19	1.07	0.99
Egypt	10 to 14	1.01	1.09	0.00	1.10	1.01	1.30	1.01	1.66
El Salvador	10 to 14	0.94	1.00	0.96	1.00	1.47	1.22	1.59	1.24
Equatorial Guinea	10 to 14	0.89	0.92	0.79	0.86	1.17	1.01	1.10	1.06
Eritrea	10 to 14	0.95	0.97	0.84	0.91	1.18	1.08	1.04	1.05
Estonia	10 to 14	1.01	1.01	0.98	0.98	1.09	1.01	1.06	1.03
Eswatini	10 to 14	1.07	1.07	1.00	0.99	1.06	1.07	1.15	1.23
Ethiopia	10 to 14	0.91	0.95	0.85	0.90	1.08	1.03	1.01	1.02
Fiji	10 to 14	0.89	0.93	0.93	0.96	1.02	1.11	1.08	1.17
Finland	10 to 14	0.99	0.98	1.01	0.98	1.24	1.05	1.23	1.02
France	10 to 14	0.97	0.98	0.98	1.01	1.04	1.01	1.07	1.02
Gabon	10 to 14	0.97	1.00	0.92	0.98	1.05	1.06	1.05	1.07
Gambia	10 to 14	0.93	0.91	0.86	0.82	1.07	1.07	1.40	1.49
Georgia	10 to 14	1.00	1.02	0.96	0.98	1.02	1.02	1.11	1.06
Germany	10 to 14	1.02	0.99	1.01	0.99	1.28	1.01	1.34	1.02
Ghana	10 to 14	0.92	0.98	0.83	0.91	1.09	1.05	1.65	1.36
Greece	10 to 14	0.98	1.00	0.97	1.00	1.09	1.05	1.24	1.04
Greenland	10 to 14	0.76	0.91	0.99	1.00	1.19	0.89	1.22	1.01
Grenada	10 to 14	0.92	1.02	0.93	1.01	1.08	1.08	0.96	1.05
Guam	10 to 14	0.98	1.18	0.98	1.10	1.14	1.37	1.44	1.54
Guatemala	10 to 14	0.97	1.04	0.94	0.99	1.13	1.34	1.59	1.19
Guinea	10 to 14	0.81	0.83	0.78	0.76	1.03	1.02	1.27	1.54
Guinea-Bissau	10 to 14	0.87	0.87	0.78	0.75	1.18	1.14	1.87	1.81
Guyana	10 to 14	1.00	1.02	0.95	0.98	1.47	1.27	1.29	1.10
Haiti	10 to 14	0.41	0.61	0.67	0.77	1.24	1.11	0.86	0.97
Honduras	10 to 14	0.67	1.02	0.79	0.99	1.41	1.24	1.31	1.15
Hungary	10 to 14	1.04	1.00	1.00	0.98	1.12	1.00	1.23	1.04
Iceland	10 to 14	0.97	0.91	0.98	0.95	1.31	0.90	1.27	0.99
India	10 to 14	0.95	0.99	0.91	0.95	1.10	1.05	1.03	1.04
Indonesia	10 to 14	0.93	1.04	0.90	0.99	1.21	1.29	1.11	1.21
Iran (Islamic Republic of)	10 to 14	0.98	1.01	0.99	1.00	1.09	1.04	1.21	1.15
Iraq	10 to 14	1.07	1.04	1.01	1.02	1.23	1.07	1.38	1.14
Ireland	10 to 14	0.98	1.00	0.98	0.99	1.22	1.02	1.14	1.02
Israel	10 to 14	0.96	0.99	0.97	1.00	1.11	1.03	1.03	1.02
Italy	10 to 14	0.97	0.97	0.96	0.97	1.11	0.99	1.14	1.01
Jamaica	10 to 14	0.97	1.00	0.95	1.00	1.13	1.05	1.11	1.03

Japan	10 to 14	0.99	0.99	0.99	0.99	1.09	1.03	1.07	1.04
Jordan	10 to 14	0.96	1.06	0.93	1.08	1.08	1.03	1.15	1.21
Kazakhstan	10 to 14	1.00	1.01	0.97	0.98	1.00	1.02	1.11	1.07
Kenya	10 to 14	0.97	1.00	0.93	0.97	1.02	1.03	1.02	1.03
Kiribati	10 to 14	1.02	1.02	0.95	0.97	1.24	1.17	1.26	1.41
Kuwait	10 to 14	1.15	1.12	1.20	1.12	1.19	1.09	1.30	1.36
Kyrgyzstan	10 to 14	1.00	1.01	0.95	0.98	1.03	1.01	1.34	1.10
Lao People's Democratic Republic	10 to 14	0.90	1.02	0.85	0.97	1.92	1.35	1.27	1.25
Latvia	10 to 14	1.02	1.00	1.00	0.98	1.10	1.00	1.10	1.03
Lebanon	10 to 14	1.02	1.02	1.00	1.01	1.11	1.04	1.02	1.04
Lesotho	10 to 14	1.04	1.10	0.99	0.99	1.04	1.10	1.08	1.26
Liberia	10 to 14	0.85	0.82	0.80	0.73	1.05	1.03	1.43	1.40
Libya	10 to 14	0.95	1.01	0.94	0.99	1.07	1.11	1.03	1.25
Lithuania	10 to 14	1.01	1.01	0.98	0.98	1.06	1.01	1.05	1.03
Luxembourg	10 to 14	0.90	1.00	0.93	1.01	1.04	1.02	0.99	1.03
Madagascar	10 to 14	0.91	0.93	0.78	0.83	1.26	1.15	1.18	1.09
Malawi	10 to 14	0.87	0.95	0.81	0.89	1.06	1.05	1.03	1.06
Malaysia	10 to 14	1.02	1.00	0.98	0.98	1.11	1.03	1.13	1.07
Maldives	10 to 14	0.87	1.02	0.87	1.00	1.17	1.05	1.12	1.09
Mali	10 to 14	0.82	0.82	0.77	0.75	1.01	0.99	1.11	1.19
Malta	10 to 14	0.88	1.14	0.90	1.09	0.99	1.44	1.01	1.24
Marshall Islands	10 to 14	0.88	0.87	0.91	0.90	0.96	1.03	1.00	1.07
Mauritania	10 to 14	0.85	0.91	0.77	0.85	1.03	1.01	1.29	1.20
Mauritius	10 to 14	1.04	1.03	0.99	1.00	1.16	1.12	1.24	1.12
Mexico	10 to 14	1.00	1.02	0.99	0.99	1.13	1.20	1.21	1.11
Micronesia (Federated States of)	10 to 14	0.90	0.91	0.90	0.93	1.10	1.06	1.08	1.08
Monaco	10 to 14	0.93	0.96	0.97	0.97	1.01	0.97	1.09	1.01
Mongolia	10 to 14	1.00	1.01	0.98	0.98	1.05	1.05	1.04	1.06
Montenegro	10 to 14	0.99	0.98	0.98	0.97	1.07	1.02	1.08	1.05
Morocco	10 to 14	0.70	0.93	0.67	0.91	1.00	1.00	0.82	0.95
Mozambique	10 to 14	1.02	1.04	0.99	1.02	1.09	1.11	1.07	1.10
Myanmar	10 to 14	0.95	1.06	0.88	1.01	2.10	1.40	1.32	1.26
Namibia	10 to 14	1.03	1.04	0.95	0.99	1.03	1.04	1.16	1.19
Nauru	10 to 14	0.89	0.85	0.91	0.87	1.12	1.05	1.32	1.35
Nepal	10 to 14	0.94	1.04	0.90	1.00	1.12	1.15	1.03	1.10
Netherlands	10 to 14	0.97	1.00	0.98	1.00	1.14	1.05	1.08	1.02
New Zealand	10 to 14	1.01	0.98	0.99	0.99	1.39	1.00	1.15	1.02
Nicaragua	10 to 14	0.99	0.99	0.96	0.98	1.20	1.05	1.27	1.05
Niger	10 to 14	0.85	0.85	0.80	0.81	1.04	1.00	1.14	1.10
Nigeria	10 to 14	0.95	0.98	0.91	0.93	1.03	1.04	1.14	1.36
Niue	10 to 14	0.92	0.00	0.94	0.39	1.10	0.00	1.08	0.48
North Macedonia	10 to 14	0.99	1.03	0.94	0.99	1.32	1.23	1.26	1.18
Northern Mariana Islands	10 to 14	0.93	0.96	0.96	0.98	0.98	1.00	1.06	1.04
Norway	10 to 14	1.02	0.98	1.01	0.99	1.30	0.99	1.11	1.01
Oman	10 to 14	1.08	1.01	1.11	0.99	1.09	0.99	1.13	1.04
Pakistan	10 to 14	0.93	0.91	0.90	0.86	1.05	1.12	1.00	1.07
Palau	10 to 14	0.93	0.96	0.95	0.97	0.99	0.98	1.12	1.06
Palestine	10 to 14	1.12	1.08	1.12	1.08	1.08	1.04	1.37	1.20

Panama	10 to 14	0.91	1.01	0.97	1.00	1.06	1.14	0.98	1.05
Papua New Guinea	10 to 14	0.80	0.80	0.81	0.81	0.94	0.93	0.93	0.95
Paraguay	10 to 14	0.89	0.99	0.91	0.98	1.17	1.15	1.06	1.07
Peru	10 to 14	1.01	1.00	0.97	0.97	1.61	1.37	1.01	1.00
Philippines	10 to 14	1.08	1.06	1.04	1.01	1.36	1.27	1.28	1.22
Poland	10 to 14	1.02	0.99	0.99	0.98	1.19	1.01	1.14	1.04
Portugal	10 to 14	0.81	1.01	0.85	1.02	1.38	1.09	2.70	1.06
Puerto Rico	10 to 14	0.99	1.00	0.99	0.99	1.04	1.00	1.02	1.00
Qatar	10 to 14	1.09	1.05	1.01	1.02	1.51	1.10	1.07	1.05
Republic of Korea	10 to 14	0.97	0.99	0.98	0.99	1.51	1.05	1.30	1.05
Republic of Moldova	10 to 14	0.98	1.00	0.93	0.98	1.06	1.02	1.04	1.03
Romania	10 to 14	1.01	1.01	0.99	0.99	1.16	1.11	1.25	1.09
Russian Federation	10 to 14	1.01	1.02	0.98	1.00	1.08	1.06	1.07	1.05
Rwanda	10 to 14	0.76	0.97	0.66	0.93	1.21	1.04	1.00	1.02
Saint Kitts and Nevis	10 to 14	1.01	1.01	0.98	1.00	1.35	1.15	1.20	1.03
Saint Lucia	10 to 14	1.04	1.04	1.01	1.02	1.13	1.18	1.07	1.05
Saint Vincent and the Grenadines	10 to 14	0.85	0.94	0.87	0.96	0.99	1.00	0.93	0.99
Samoa	10 to 14	0.95	0.93	0.95	0.94	1.09	1.01	1.06	1.05
San Marino	10 to 14	0.97	0.99	0.97	0.99	1.05	1.00	1.10	1.02
Sao Tome and Principe	10 to 14	1.05	1.00	0.97	0.96	1.15	1.05	1.98	1.22
Saudi Arabia	10 to 14	0.92	1.00	0.87	0.98	1.04	1.00	1.07	1.04
Senegal	10 to 14	0.85	0.91	0.78	0.84	1.06	1.04	1.40	1.27
Serbia	10 to 14	0.99	1.01	0.97	1.00	1.28	1.05	1.34	1.08
Seychelles	10 to 14	0.96	0.98	0.95	0.97	1.02	0.99	1.05	1.02
Sierra Leone	10 to 14	0.82	0.76	0.77	0.68	1.06	1.01	1.75	1.79
Singapore	10 to 14	0.95	0.98	0.97	0.99	1.09	1.00	1.04	1.02
Slovakia	10 to 14	1.00	1.00	0.97	0.99	1.09	1.02	1.14	1.05
Slovenia	10 to 14	1.02	1.00	1.00	0.98	1.06	0.99	1.10	1.03
Solomon Islands	10 to 14	0.91	0.90	0.92	0.91	0.95	0.94	0.99	0.98
Somalia	10 to 14	0.94	0.98	0.86	0.92	1.09	1.07	1.02	1.03
South Africa	10 to 14	1.03	1.01	0.97	0.95	1.02	1.02	1.10	1.14
South Sudan	10 to 14	0.92	0.91	0.86	0.82	1.06	1.08	1.03	1.04
Spain	10 to 14	0.93	0.98	0.95	1.00	1.17	1.02	1.12	1.01
Sri Lanka	10 to 14	0.97	1.00	0.94	0.98	1.06	1.02	1.20	1.10
Sudan	10 to 14	0.56	0.87	0.54	0.86	1.01	1.06	0.76	1.01
Suriname	10 to 14	0.88	0.83	0.91	0.88	1.08	0.92	1.02	0.95
Sweden	10 to 14	0.97	1.00	1.01	1.02	1.09	1.03	1.03	1.02
Switzerland	10 to 14	0.98	0.99	0.98	0.99	1.16	1.01	1.18	1.02
Syrian Arab Republic	10 to 14	1.01	1.08	1.01	1.09	1.18	1.05	1.14	1.17
Taiwan (Province of China)	10 to 14	1.00	0.99	1.00	1.01	1.02	1.01	1.08	1.05
Tajikistan	10 to 14	0.99	0.99	0.93	0.95	1.03	1.02	1.71	1.29
Thailand	10 to 14	0.98	1.04	0.94	1.00	1.35	1.21	1.09	1.09
Timor-Leste	10 to 14	0.95	1.03	0.93	0.98	1.23	1.32	1.13	1.22
Togo	10 to 14	0.87	0.91	0.79	0.82	1.04	1.05	1.35	1.42
Tokelau	10 to 14	0.90	0.22	0.93	0.57	1.03	0.73	1.08	0.81
Tonga	10 to 14	0.95	0.94	0.95	0.95	0.99	0.98	1.03	1.03
Trinidad and Tobago	10 to 14	1.00	1.00	0.98	0.98	1.26	1.11	1.15	1.03
Tunisia	10 to 14	0.94	0.99	0.91	0.98	1.08	1.02	1.03	1.05

Turkey	10 to 14	1.03	1.01	0.99	0.99	1.68	1.09	1.10	1.04
Turkmenistan	10 to 14	1.00	1.01	0.96	0.96	1.03	1.05	1.23	1.17
Tuvalu	10 to 14	0.98	0.96	0.96	0.96	1.37	1.16	1.37	1.22
Uganda	10 to 14	0.98	0.98	0.92	0.93	1.11	1.07	1.04	1.04
Ukraine	10 to 14	1.00	1.00	0.98	0.98	1.02	1.02	1.05	1.04
United Arab Emirates	10 to 14	1.14	1.00	1.13	0.99	1.39	1.01	1.27	1.02
United Kingdom	10 to 14	0.97	1.00	0.97	1.00	1.18	1.02	1.09	1.02
United Republic of Tanzania	10 to 14	0.96	0.98	0.91	0.94	1.08	1.08	1.03	1.07
United States Virgin Islands	10 to 14	1.14	1.02	1.06	1.00	1.47	1.05	1.16	1.01
United States of America	10 to 14	0.96	0.97	1.01	1.01	1.10	1.09	1.04	1.02
Uruguay	10 to 14	1.02	0.99	0.99	0.99	1.58	1.09	1.13	1.02
Uzbekistan	10 to 14	1.01	1.02	0.94	0.98	1.05	1.04	1.50	1.18
Vanuatu	10 to 14	0.93	0.91	0.94	0.92	1.10	1.01	1.10	1.08
Venezuela (Bolivarian Republic of)	10 to 14	1.02	1.06	1.00	1.00	1.19	1.45	1.26	1.14
Viet Nam	10 to 14	1.03	1.04	0.98	1.01	1.11	1.10	1.08	1.10
Yemen	10 to 14	0.72	0.88	0.69	0.87	1.01	1.04	0.83	0.97
Zambia	10 to 14	0.91	0.97	0.84	0.93	1.04	1.05	1.02	1.04
Zimbabwe	10 to 14	1.01	1.00	0.97	0.89	1.02	1.08	1.05	1.15
Afghanistan	15 to 19	1.25	1.14	1.03	0.87	1.14	1.10	0.72	0.85
Albania	15 to 19	1.23	1.09	1.07	0.99	1.26	1.06	1.09	1.04
Algeria	15 to 19	1.15	1.06	0.97	0.96	1.05	0.99	1.04	1.01
American Samoa	15 to 19	1.35	1.21	1.22	1.11	1.06	1.03	0.98	0.93
Andorra	15 to 19	1.02	1.02	0.96	0.97	0.93	0.96	1.02	1.00
Angola	15 to 19	1.21	1.08	0.88	0.84	1.18	1.09	1.05	1.02
Antigua and Barbuda	15 to 19	1.17	1.10	0.97	1.01	1.39	1.13	1.01	0.99
Argentina	15 to 19	1.29	1.19	1.00	1.03	3.24	1.31	1.18	1.10
Armenia	15 to 19	1.15	1.11	1.01	0.97	1.23	1.10	1.12	1.12
Australia	15 to 19	1.10	1.07	1.03	1.00	1.36	1.08	1.08	1.03
Austria	15 to 19	1.16	1.04	1.07	0.99	1.19	1.02	1.26	1.02
Azerbaijan	15 to 19	1.24	1.13	1.08	1.01	1.07	1.04	1.15	1.08
Bahamas	15 to 19	1.15	1.07	1.01	0.99	1.25	1.11	1.05	1.01
Bahrain	15 to 19	1.11	1.13	0.99	1.04	1.01	1.02	0.93	1.11
Bangladesh	15 to 19	3.54	1.43	1.53	1.11	1.01	1.53	1.21	1.13
Barbados	15 to 19	1.06	1.04	0.92	0.95	1.16	1.06	0.95	1.00
Belarus	15 to 19	1.18	1.08	1.06	0.98	1.12	1.06	1.27	1.02
Belgium	15 to 19	1.22	1.04	1.09	0.98	1.27	1.02	1.33	1.02
Belize	15 to 19	1.50	1.09	1.15	1.01	1.91	1.08	1.30	1.06
Benin	15 to 19	0.92	0.93	0.70	0.71	0.91	0.89	1.09	1.07
Bermuda	15 to 19	1.06	1.03	0.96	0.94	1.06	1.01	1.03	1.02
Bhutan	15 to 19	0.96	1.13	0.82	1.00	0.99	1.13	0.90	1.09
Bolivia (Plurinational State of)	15 to 19	0.35	1.12	0.40	0.90	0.73	1.02	0.86	1.05
Bosnia and Herzegovina	15 to 19	1.16	1.13	0.94	0.95	1.38	1.09	1.19	1.13
Botswana	15 to 19	1.93	1.24	1.50	1.08	1.13	1.05	1.25	1.14
Brazil	15 to 19	1.25	1.24	1.05	1.10	1.19	1.21	1.42	1.17
Brunei Darussalam	15 to 19	1.36	1.05	1.13	0.98	1.69	1.02	1.37	1.01
Bulgaria	15 to 19	1.40	1.27	1.07	1.10	1.42	1.26	1.98	1.48
Burkina Faso	15 to 19	1.00	0.98	0.82	0.78	0.97	0.96	1.08	1.10
Burundi	15 to 19	7.04	1.00	0.05	0.55	2.58	1.12	0.81	0.88

Cabo Verde	15 to 19	0.94	1.13	0.71	0.90	0.95	1.03	1.19	1.52
Cambodia	15 to 19	1.01	1.20	0.82	1.01	1.04	1.12	0.95	1.08
Cameroon	15 to 19	0.95	0.94	0.70	0.69	1.00	0.96	1.14	1.17
Canada	15 to 19	1.02	1.01	0.98	0.97	1.07	1.05	1.02	1.01
Central African Republic	15 to 19	1.80	1.30	1.02	0.80	1.56	1.35	1.20	1.12
Chad	15 to 19	1.03	0.98	0.77	0.71	1.02	0.99	1.22	1.28
Chile	15 to 19	1.12	1.12	1.00	1.01	1.34	1.18	1.11	1.08
China	15 to 19	1.52	1.24	1.24	1.16	1.89	1.14	1.31	1.20
Colombia	15 to 19	1.17	1.20	0.96	1.00	1.28	1.37	1.71	1.20
Comoros	15 to 19	1.05	0.97	0.54	0.66	1.16	1.03	0.92	0.94
Congo	15 to 19	1.36	1.05	0.99	0.80	1.25	1.07	1.19	1.05
Cook Islands	15 to 19	1.33	1.13	1.17	1.06	1.04	1.01	0.88	0.94
Costa Rica	15 to 19	1.07	1.05	0.95	0.94	1.10	1.08	1.07	1.02
Coted'Ivoire	15 to 19	1.06	0.98	0.76	0.71	1.03	0.96	1.40	1.32
Croatia	15 to 19	1.14	1.07	1.03	0.96	1.17	1.04	1.19	1.05
Cuba	15 to 19	1.11	1.12	0.99	1.03	1.11	1.10	1.15	1.13
Cyprus	15 to 19	1.10	1.05	1.03	0.99	1.19	1.03	1.07	1.03
Czechia	15 to 19	1.15	1.07	1.00	0.95	1.17	1.05	1.30	1.07
Democratic People's Republic of Korea	15 to 19	1.47	1.55	1.22	1.31	1.50	1.38	1.31	1.43
Democratic Republic of the Congo	15 to 19	1.08	1.09	0.77	0.82	1.13	1.11	1.02	1.03
Denmark	15 to 19	1.05	1.05	1.00	0.99	1.06	1.04	1.06	1.02
Djibouti	15 to 19	1.43	1.28	1.05	1.05	1.37	1.28	1.08	1.14
Dominica	15 to 19	1.19	1.09	1.01	0.96	1.33	1.15	1.10	1.05
Dominican Republic	15 to 19	1.26	1.68	1.06	1.37	1.36	1.57	1.30	2.42
Ecuador	15 to 19	2.59	1.23	1.51	1.06	2.00	1.23	2.07	1.11
Egypt	15 to 19	4.42	1.37	1.01	1.34	1.51	1.16	2.84	2.50
El Salvador	15 to 19	1.49	1.28	1.08	1.09	1.62	1.30	2.53	1.48
Equatorial Guinea	15 to 19	1.52	1.07	0.97	0.88	1.48	1.04	1.19	1.05
Eritrea	15 to 19	13.16	1.61	1.80	1.00	2.89	1.52	1.17	1.15
Estonia	15 to 19	1.24	1.08	1.10	0.98	1.32	1.04	1.32	1.03
Eswatini	15 to 19	1.93	1.94	1.68	1.46	1.09	1.11	1.16	1.29
Ethiopia	15 to 19	1.76	1.09	0.96	0.78	1.44	1.10	0.97	0.96
Fiji	15 to 19	1.35	1.21	1.25	1.13	1.04	1.00	0.93	0.90
Finland	15 to 19	1.12	1.05	1.04	0.98	1.11	1.05	1.27	1.02
France	15 to 19	1.10	1.06	1.04	1.04	1.05	1.05	1.13	1.03
Gabon	15 to 19	1.22	1.18	0.97	1.02	1.15	1.12	1.07	1.10
Gambia	15 to 19	1.04	1.01	0.76	0.68	1.02	1.03	1.32	1.47
Georgia	15 to 19	1.16	1.18	0.99	1.03	1.11	1.23	1.07	1.18
Germany	15 to 19	1.20	1.07	1.07	1.01	1.40	1.06	1.44	1.04
Ghana	15 to 19	0.93	1.07	0.62	0.79	0.97	1.00	1.36	1.32
Greece	15 to 19	1.18	1.06	1.03	1.00	1.16	1.05	1.26	1.03
Greenland	15 to 19	0.65	0.94	0.81	0.96	0.00	0.85	0.92	0.98
Grenada	15 to 19	1.37	1.05	1.04	0.98	2.06	1.03	1.38	1.03
Guam	15 to 19	1.45	1.29	1.33	1.25	1.17	1.09	1.10	1.04
Guatemala	15 to 19	1.01	1.21	0.81	0.98	1.10	1.26	0.97	1.20
Guinea	15 to 19	0.86	0.88	0.64	0.62	0.92	0.92	1.13	1.22
Guinea-Bissau	15 to 19	0.99	0.93	0.56	0.54	1.11	1.01	1.77	1.60
Guyana	15 to 19	1.41	1.19	1.05	0.98	1.54	1.33	1.81	1.21

Haiti	15 to 19	0.00	0.37	0.27	0.44	0.05	0.27	0.00	0.43
Honduras	15 to 19	0.93	1.06	0.79	0.91	1.13	1.10	1.06	1.12
Hungary	15 to 19	1.22	1.08	1.05	0.96	1.19	1.05	1.51	1.08
Iceland	15 to 19	1.18	1.10	1.07	1.02	1.28	1.16	1.28	1.04
India	15 to 19	0.98	1.07	0.83	0.93	1.00	1.03	0.90	0.99
Indonesia	15 to 19	0.99	1.44	0.81	1.15	1.01	1.29	1.00	1.40
Iran (Islamic Republic of)	15 to 19	1.18	1.19	1.11	1.11	1.08	1.05	1.56	1.59
Iraq	15 to 19	1.38	1.44	1.12	1.25	1.07	1.06	1.37	2.12
Ireland	15 to 19	1.06	1.03	0.98	0.98	1.09	1.00	1.10	1.01
Israel	15 to 19	1.14	1.05	1.11	1.00	1.17	1.03	1.07	1.01
Italy	15 to 19	1.12	1.04	1.01	0.98	1.06	0.96	1.22	1.01
Jamaica	15 to 19	1.17	1.05	0.94	0.99	1.29	1.01	1.14	1.03
Japan	15 to 19	1.10	1.08	1.02	1.01	1.10	1.06	1.08	1.03
Jordan	15 to 19	1.12	1.10	1.04	1.05	1.01	0.99	0.96	1.10
Kazakhstan	15 to 19	1.15	1.15	1.00	1.01	1.21	1.21	1.31	1.20
Kenya	15 to 19	1.11	1.24	0.89	1.01	1.09	1.18	0.98	1.06
Kiribati	15 to 19	1.93	2.16	1.44	1.73	1.40	1.34	1.06	1.08
Kuwait	15 to 19	1.25	1.14	1.23	1.07	1.03	0.99	1.26	1.28
Kyrgyzstan	15 to 19	1.28	1.13	1.09	0.99	1.26	1.10	1.46	1.17
Lao People's Democratic Republic	15 to 19	1.42	1.27	0.78	0.93	2.11	1.27	1.23	1.33
Latvia	15 to 19	1.32	1.10	1.19	1.00	1.44	1.08	1.54	1.06
Lebanon	15 to 19	1.34	1.18	1.24	1.12	1.13	1.05	1.04	1.08
Lesotho	15 to 19	1.67	2.97	1.59	2.28	1.09	1.14	1.10	1.31
Liberia	15 to 19	0.89	0.82	0.65	0.54	0.94	0.89	1.18	1.09
Libya	15 to 19	1.10	1.25	0.97	1.12	1.02	1.04	1.02	1.92
Lithuania	15 to 19	1.14	1.09	1.03	1.00	1.22	1.09	1.11	1.05
Luxembourg	15 to 19	1.55	1.05	1.30	1.00	1.62	1.02	2.88	1.02
Madagascar	15 to 19	1.01	1.67	0.82	0.26	4.09	2.10	1.39	1.19
Malawi	15 to 19	1.12	1.27	0.69	0.83	1.21	1.29	0.99	1.11
Malaysia	15 to 19	1.33	1.21	1.08	1.07	1.16	1.06	1.25	1.16
Maldives	15 to 19	1.13	1.21	0.91	1.05	1.17	1.11	1.08	1.23
Mali	15 to 19	0.71	0.74	0.45	0.45	0.84	0.81	0.98	1.00
Malta	15 to 19	1.13	1.08	1.00	1.01	1.11	1.09	1.29	1.04
Marshall Islands	15 to 19	1.38	1.86	1.23	1.58	1.10	1.18	0.96	0.92
Mauritania	15 to 19	0.90	0.96	0.61	0.73	0.96	0.94	1.25	1.12
Mauritius	15 to 19	1.16	1.27	1.00	1.09	1.14	1.24	1.27	1.30
Mexico	15 to 19	1.09	1.14	0.96	0.99	1.12	1.30	1.17	1.14
Micronesia (Federated States of)	15 to 19	1.78	1.71	1.46	1.42	1.24	1.22	0.92	0.93
Monaco	15 to 19	1.06	1.01	1.02	0.98	0.89	0.91	1.09	0.99
Mongolia	15 to 19	1.20	1.16	1.03	1.02	1.24	1.11	1.06	1.07
Montenegro	15 to 19	1.17	1.20	1.04	1.10	1.06	1.05	1.07	1.08
Morocco	15 to 19	0.90	1.04	0.72	0.93	0.98	0.99	0.69	0.93
Mozambique	15 to 19	1.52	1.90	1.23	1.44	1.42	1.91	1.16	1.44
Myanmar	15 to 19	2.10	2.29	0.95	1.46	3.10	1.94	1.71	2.14
Namibia	15 to 19	1.63	1.48	1.36	1.32	1.08	1.08	1.21	1.23
Nauru	15 to 19	1.88	2.22	1.71	2.05	1.19	1.16	0.92	0.89
Nepal	15 to 19	1.00	1.23	0.84	1.07	1.05	1.24	0.92	1.12
Netherlands	15 to 19	1.07	1.05	1.01	0.99	1.06	1.03	1.05	1.01

New Zealand	15 to 19	1.12	1.04	1.03	0.99	1.41	1.01	1.16	1.02
Nicaragua	15 to 19	1.01	1.08	0.87	0.96	1.04	1.08	1.09	1.08
Niger	15 to 19	0.91	0.90	0.67	0.69	0.96	0.91	1.09	1.04
Nigeria	15 to 19	1.00	1.05	0.81	0.86	0.97	0.99	1.08	1.18
Niue	15 to 19	1.39	1.04	1.25	0.88	1.20	0.78	0.95	0.68
North Macedonia	15 to 19	1.27	1.15	1.03	1.00	1.35	1.17	1.39	1.20
Northern Mariana Islands	15 to 19	1.26	1.21	1.21	1.15	1.01	1.07	0.98	1.00
Norway	15 to 19	1.09	1.03	1.01	0.98	1.18	0.99	1.00	1.00
Oman	15 to 19	1.45	1.12	1.47	1.06	1.07	0.99	1.44	1.08
Pakistan	15 to 19	0.93	0.89	0.80	0.73	0.92	0.84	0.89	0.87
Palau	15 to 19	1.33	0.67	1.13	0.55	0.97	0.46	0.86	0.27
Palestine	15 to 19	1.76	1.23	1.95	1.20	1.04	1.00	2.41	1.26
Panama	15 to 19	1.00	1.07	0.97	0.98	1.09	1.14	0.94	1.02
Papua New Guinea	15 to 19	1.05	0.98	0.94	0.84	0.94	0.86	0.89	0.84
Paraguay	15 to 19	1.03	1.08	0.87	0.98	1.09	1.03	1.05	1.08
Peru	15 to 19	2.34	1.25	1.28	1.04	2.39	1.12	1.38	1.07
Philippines	15 to 19	1.29	1.36	1.16	1.15	1.16	1.22	1.22	1.26
Poland	15 to 19	1.23	1.12	1.05	1.02	1.29	1.09	1.36	1.08
Portugal	15 to 19	1.60	1.04	1.20	1.01	1.89	1.03	1.01	1.03
Puerto Rico	15 to 19	1.07	1.05	0.97	0.96	1.07	1.04	1.10	1.04
Qatar	15 to 19	1.41	1.21	1.19	1.11	1.52	1.14	1.16	1.15
Republic of Korea	15 to 19	1.30	1.07	1.08	1.00	2.47	1.05	1.39	1.03
Republic of Moldova	15 to 19	1.15	1.11	1.01	1.01	1.14	1.09	1.07	1.04
Romania	15 to 19	1.15	1.17	1.00	1.02	1.13	1.22	1.25	1.18
Russian Federation	15 to 19	1.27	1.21	1.11	1.15	1.34	1.14	1.39	1.17
Rwanda	15 to 19	2.43	1.19	0.00	0.92	1.98	1.18	0.80	1.00
Saint Kitts and Nevis	15 to 19	1.11	1.19	0.95	1.04	1.22	1.27	1.08	1.14
Saint Lucia	15 to 19	1.12	1.17	1.01	1.08	1.13	1.14	1.05	1.18
Saint Vincent and the Grenadines	15 to 19	1.19	1.11	1.04	1.02	1.10	1.05	1.17	1.06
Samoa	15 to 19	1.42	1.31	1.29	1.19	1.15	1.08	0.95	0.95
San Marino	15 to 19	1.08	1.04	1.01	0.98	1.08	1.01	1.12	1.02
Sao Tome and Principe	15 to 19	0.95	1.14	0.74	0.88	0.94	1.09	1.09	1.42
Saudi Arabia	15 to 19	1.01	1.08	0.81	0.99	1.00	1.00	0.85	1.11
Senegal	15 to 19	0.92	0.99	0.60	0.71	0.96	0.98	1.27	1.21
Serbia	15 to 19	1.24	1.07	1.04	0.96	1.24	1.01	1.58	1.08
Seychelles	15 to 19	1.04	1.07	0.90	0.98	1.00	1.00	1.05	1.02
Sierra Leone	15 to 19	1.02	0.86	0.75	0.59	0.98	0.88	1.40	1.17
Singapore	15 to 19	1.04	1.00	0.97	0.95	1.02	0.94	0.97	0.98
Slovakia	15 to 19	1.19	1.08	1.04	0.98	1.14	1.03	1.29	1.08
Slovenia	15 to 19	1.13	1.06	1.00	0.94	1.11	1.03	1.18	1.03
Solomon Islands	15 to 19	1.13	1.15	1.03	1.05	1.00	1.01	0.96	0.96
Somalia	15 to 19	2.01	1.45	0.98	0.89	1.75	1.43	0.99	1.02
South Africa	15 to 19	1.15	1.27	0.95	1.27	1.01	1.05	1.05	1.14
South Sudan	15 to 19	1.33	1.21	0.82	0.69	1.27	1.26	1.00	1.00
Spain	15 to 19	1.20	1.09	1.07	1.05	1.33	1.07	1.26	1.03
Sri Lanka	15 to 19	1.19	1.15	0.98	1.03	1.04	1.02	1.39	1.27
Sudan	15 to 19	0.80	0.99	0.58	0.84	0.97	0.99	0.53	0.84
Suriname	15 to 19	1.18	1.28	0.96	1.07	1.22	1.30	1.26	1.31

Sweden	15 to 19	1.12	1.06	1.08	1.03	1.21	1.04	1.07	1.02
Switzerland	15 to 19	1.15	1.03	1.04	0.98	1.20	0.99	1.28	1.01
Syrian Arab Republic	15 to 19	1.76	1.46	1.47	1.37	1.08	1.05	1.61	1.55
Taiwan (Province of China)	15 to 19	1.14	1.08	1.03	1.03	1.02	1.01	1.17	1.08
Tajikistan	15 to 19	1.19	1.12	1.01	0.97	1.08	1.06	1.70	1.16
Thailand	15 to 19	1.40	1.67	1.06	1.27	1.60	1.69	1.22	1.41
Timor-Leste	15 to 19	1.10	1.39	0.89	1.04	1.18	1.36	1.09	1.39
Togo	15 to 19	0.86	0.95	0.57	0.67	0.91	0.94	1.16	1.23
Tokelau	15 to 19	1.54	1.56	1.42	1.62	1.14	0.83	0.94	0.63
Tonga	15 to 19	1.11	1.18	1.05	1.10	1.03	1.08	0.99	0.99
Trinidad and Tobago	15 to 19	1.36	1.19	1.09	1.00	1.52	1.52	1.98	1.23
Tunisia	15 to 19	1.12	1.09	1.00	1.00	1.03	1.01	1.05	1.10
Turkey	15 to 19	1.36	1.15	1.11	1.04	1.60	1.12	1.48	1.16
Turkmenistan	15 to 19	1.11	1.56	0.95	1.21	1.18	2.58	1.26	3.07
Tuvalu	15 to 19	2.60	1.83	2.04	1.58	1.47	1.29	0.98	0.97
Uganda	15 to 19	1.55	1.27	1.11	0.96	1.46	1.26	1.05	1.04
Ukraine	15 to 19	1.18	1.13	1.08	1.02	1.09	1.07	1.23	1.10
United Arab Emirates	15 to 19	1.46	1.16	1.44	1.11	1.20	1.06	1.61	1.17
United Kingdom	15 to 19	1.11	1.06	1.02	1.01	1.35	1.05	1.15	1.03
United Republic of Tanzania	15 to 19	1.42	1.18	1.01	0.95	1.39	1.21	1.03	1.10
United States Virgin Islands	15 to 19	1.33	1.30	1.13	1.13	1.42	1.33	1.28	1.33
United States of America	15 to 19	0.99	1.01	1.00	1.01	1.04	1.04	1.02	1.01
Uruguay	15 to 19	1.08	1.17	0.93	1.02	1.80	1.43	1.08	1.12
Uzbekistan	15 to 19	1.24	1.26	1.06	1.13	1.14	1.20	1.61	1.50
Vanuatu	15 to 19	1.55	1.48	1.47	1.33	1.22	1.12	0.97	0.94
Venezuela (Bolivarian Republic of)	15 to 19	1.19	1.22	0.98	0.98	1.38	1.42	1.29	1.32
Viet Nam	15 to 19	1.25	1.41	1.06	1.23	1.20	1.26	1.15	1.39
Yemen	15 to 19	1.10	1.09	1.00	0.98	1.03	1.02	0.96	1.05
Zambia	15 to 19	1.07	1.13	0.57	0.72	1.17	1.15	0.94	1.01
Zimbabwe	15 to 19	1.39	3.15	1.22	4.59	1.23	1.68	1.11	1.36
Afghanistan	20 to 24	0.68	0.72	0.48	0.43	0.88	0.87	0.66	0.84
Albania	20 to 24	1.04	1.00	0.94	0.92	1.04	1.00	1.14	1.08
Algeria	20 to 24	0.90	0.96	0.75	0.86	0.95	0.97	1.02	1.16
American Samoa	20 to 24	1.25	1.12	0.97	0.87	1.24	1.13	1.44	1.32
Andorra	20 to 24	0.93	0.97	0.89	0.93	0.80	0.92	1.01	1.01
Angola	20 to 24	0.93	0.94	0.69	0.70	0.97	0.96	1.06	1.09
Antigua and Barbuda	20 to 24	1.07	1.07	0.87	0.98	1.30	1.13	1.07	1.07
Argentina	20 to 24	0.92	0.99	0.85	0.92	0.80	0.96	1.10	1.06
Armenia	20 to 24	0.97	1.01	0.87	0.90	1.01	1.05	1.28	1.27
Australia	20 to 24	0.97	0.99	0.91	0.94	0.99	1.00	1.15	1.04
Austria	20 to 24	0.99	1.00	0.94	0.95	0.97	1.01	1.15	1.04
Azerbaijan	20 to 24	0.96	0.98	0.79	0.86	0.99	0.99	1.32	1.14
Bahamas	20 to 24	1.14	1.07	0.99	0.98	1.25	1.13	1.27	1.09
Bahrain	20 to 24	0.92	1.01	0.83	0.94	0.94	0.99	1.00	1.15
Bangladesh	20 to 24	1.88	1.50	1.23	1.20	2.99	1.62	2.23	2.19
Barbados	20 to 24	1.04	0.98	0.92	0.91	1.11	0.98	1.18	1.00
Belarus	20 to 24	1.02	1.01	0.93	0.94	0.94	1.00	1.50	1.12
Belgium	20 to 24	1.05	0.99	0.96	0.94	0.98	0.97	1.49	1.03

Belize	20 to 24	1.25	1.15	0.95	1.07	1.77	1.15	1.33	1.28
Benin	20 to 24	0.96	0.93	0.75	0.71	0.97	0.94	1.21	1.25
Bermuda	20 to 24	1.04	1.00	0.93	0.91	1.09	1.04	1.30	1.07
Bhutan	20 to 24	0.92	1.02	0.78	0.91	0.93	1.03	1.03	1.15
Bolivia (Plurinational State of)	20 to 24	0.37	0.79	0.29	0.70	0.24	0.61	1.09	1.12
Bosnia and Herzegovina	20 to 24	1.03	0.99	0.94	0.93	1.17	1.02	1.28	1.15
Botswana	20 to 24	1.16	1.02	0.86	0.86	1.04	1.00	1.24	1.11
Brazil	20 to 24	0.97	0.99	0.86	0.91	0.90	0.91	1.58	1.24
Brunei Darussalam	20 to 24	1.08	0.98	0.95	0.92	1.07	0.95	1.90	1.03
Bulgaria	20 to 24	1.01	1.14	0.86	1.04	0.97	1.08	1.91	4.59
Burkina Faso	20 to 24	1.00	0.99	0.82	0.79	1.02	1.02	1.17	1.25
Burundi	20 to 24	0.88	0.90	0.47	0.58	1.01	0.97	1.06	1.06
Cabo Verde	20 to 24	0.91	1.06	0.70	0.88	0.97	1.06	1.46	1.71
Cambodia	20 to 24	0.78	0.98	0.60	0.84	0.85	0.98	1.00	1.12
Cameroon	20 to 24	0.97	0.94	0.74	0.70	1.05	1.02	1.32	1.45
Canada	20 to 24	0.97	0.98	0.92	0.93	1.00	0.99	1.04	1.03
Central African Republic	20 to 24	1.00	0.95	0.67	0.63	1.05	1.01	1.17	1.16
Chad	20 to 24	0.99	0.95	0.76	0.70	1.05	1.04	1.33	1.52
Chile	20 to 24	1.01	0.98	0.94	0.93	1.04	0.93	1.22	1.07
China	20 to 24	1.09	1.12	0.98	1.05	1.03	1.04	1.38	1.46
Colombia	20 to 24	0.98	1.00	0.87	0.90	0.98	1.01	2.20	1.15
Comoros	20 to 24	0.76	0.88	0.42	0.60	0.85	0.92	1.01	1.06
Congo	20 to 24	1.00	0.89	0.74	0.61	1.02	0.92	1.20	1.09
Cook Islands	20 to 24	1.05	1.10	0.80	0.95	1.10	1.11	1.30	1.21
Costa Rica	20 to 24	0.98	1.00	0.89	0.91	1.00	1.05	1.19	1.10
Coted'Ivoire	20 to 24	1.03	0.97	0.78	0.73	1.08	1.01	1.62	1.73
Croatia	20 to 24	1.06	0.99	1.02	0.93	1.03	0.97	1.43	1.09
Cuba	20 to 24	1.01	1.06	0.90	1.00	1.04	1.05	1.24	1.18
Cyprus	20 to 24	1.02	1.05	0.96	0.98	1.03	1.06	1.09	1.09
Czechia	20 to 24	1.03	0.98	0.95	0.90	1.02	0.97	1.46	1.11
Democratic People's Republic of Korea	20 to 24	1.11	1.17	0.98	1.03	1.06	1.07	1.46	1.75
Democratic Republic of the Congo	20 to 24	0.89	0.93	0.64	0.68	0.95	0.97	1.06	1.08
Denmark	20 to 24	0.99	0.99	0.96	0.95	0.89	0.99	1.15	1.03
Djibouti	20 to 24	1.09	1.08	0.87	0.91	1.12	1.12	1.13	1.19
Dominica	20 to 24	1.05	1.01	0.92	0.91	1.14	1.06	1.11	1.08
Dominican Republic	20 to 24	1.05	1.22	0.90	1.12	1.08	1.15	1.46	2.50
Ecuador	20 to 24	1.18	1.08	0.95	0.96	1.17	1.09	5.17	1.40
Egypt	20 to 24	1.14	1.07	1.09	1.06	1.12	1.06	1.85	3.65
El Salvador	20 to 24	1.14	1.04	0.93	0.95	1.16	1.02	6.69	1.51
Equatorial Guinea	20 to 24	1.00	0.96	0.72	0.77	1.05	0.97	1.16	1.13
Eritrea	20 to 24	1.24	1.14	0.79	0.82	1.31	1.18	1.21	1.27
Estonia	20 to 24	1.03	0.99	0.95	0.90	1.09	0.99	1.33	1.06
Eswatini	20 to 24	1.10	1.18	0.84	0.88	1.00	1.03	1.11	1.32
Ethiopia	20 to 24	0.90	0.97	0.62	0.72	0.99	1.01	1.01	1.05
Fiji	20 to 24	1.05	1.01	0.74	0.73	1.12	1.07	1.41	1.29
Finland	20 to 24	0.97	0.98	0.94	0.93	0.85	0.94	1.21	1.02
France	20 to 24	1.02	1.02	0.97	1.00	0.96	1.04	1.22	1.05
Gabon	20 to 24	1.02	1.03	0.82	0.89	1.02	1.03	1.11	1.15

Gambia	20 to 24	1.06	1.01	0.83	0.72	1.10	1.10	1.71	2.16
Georgia	20 to 24	0.97	1.05	0.83	0.95	0.95	1.04	1.21	1.36
Germany	20 to 24	1.01	1.00	0.93	0.95	0.97	0.98	1.37	1.04
Ghana	20 to 24	0.93	1.01	0.67	0.79	1.05	1.09	1.80	1.84
Greece	20 to 24	1.00	1.01	0.87	0.94	0.93	1.02	1.53	1.08
Greenland	20 to 24	0.75	0.91	0.79	0.88	0.36	0.82	0.96	0.99
Grenada	20 to 24	1.18	0.98	1.08	0.91	1.11	0.98	1.01	1.03
Guam	20 to 24	1.05	1.18	0.82	1.03	1.06	1.24	1.41	1.30
Guatemala	20 to 24	0.94	1.03	0.77	0.88	1.03	1.07	1.59	1.30
Guinea	20 to 24	0.90	0.87	0.69	0.63	0.98	0.95	1.30	1.60
Guinea-Bissau	20 to 24	0.86	0.88	0.53	0.55	1.04	1.02	1.85	2.21
Guyana	20 to 24	1.26	1.08	0.96	0.87	1.35	1.22	4.27	1.86
Haiti	20 to 24	0.00	0.28	0.00	0.21	0.00	0.14	0.00	0.36
Honduras	20 to 24	0.59	0.86	0.47	0.77	0.43	0.72	2.55	1.19
Hungary	20 to 24	1.03	1.00	0.95	0.93	0.99	1.00	1.52	1.13
Iceland	20 to 24	1.01	0.99	0.96	0.93	0.92	0.97	1.27	1.02
India	20 to 24	0.89	0.99	0.75	0.87	0.91	0.99	1.01	1.09
Indonesia	20 to 24	0.83	1.02	0.66	0.85	0.81	0.97	1.19	1.58
Iran (Islamic Republic of)	20 to 24	0.97	1.03	0.90	0.98	0.98	1.01	1.34	1.72
Iraq	20 to 24	1.03	1.07	0.88	0.95	0.94	0.99	1.67	1.49
Ireland	20 to 24	0.96	0.99	0.92	0.94	0.86	0.99	1.15	1.02
Israel	20 to 24	1.02	0.98	0.97	0.95	1.04	0.98	1.09	1.02
Italy	20 to 24	0.99	0.99	0.92	0.92	0.89	0.93	1.16	1.03
Jamaica	20 to 24	1.12	0.97	0.90	0.91	1.31	0.94	1.39	1.05
Japan	20 to 24	1.02	1.02	0.96	0.97	1.06	1.00	1.13	1.08
Jordan	20 to 24	0.90	1.00	0.77	0.96	0.98	1.00	1.11	1.23
Kazakhstan	20 to 24	0.95	1.02	0.85	0.94	0.95	1.02	1.74	1.40
Kenya	20 to 24	1.00	1.06	0.82	0.87	1.01	1.05	1.05	1.12
Kiribati	20 to 24	1.22	1.35	0.82	0.94	1.56	1.42	1.50	1.97
Kuwait	20 to 24	1.02	1.02	0.99	0.96	0.98	1.01	1.20	1.27
Kyrgyzstan	20 to 24	0.99	1.00	0.84	0.90	0.97	0.98	2.61	1.54
Lao People's Democratic Republic	20 to 24	0.73	0.88	0.48	0.68	0.84	0.88	1.28	1.40
Latvia	20 to 24	1.02	1.03	0.92	0.98	1.04	1.04	1.34	1.19
Lebanon	20 to 24	1.09	1.04	1.03	1.00	1.04	1.03	1.09	1.10
Lesotho	20 to 24	1.10	1.16	0.94	0.84	1.02	1.02	1.08	1.30
Liberia	20 to 24	0.90	0.79	0.67	0.50	0.97	0.88	1.35	1.39
Libya	20 to 24	0.95	0.97	0.81	0.83	0.96	0.92	1.12	1.84
Lithuania	20 to 24	1.02	1.00	0.93	0.92	1.09	1.01	1.23	1.07
Luxembourg	20 to 24	1.07	0.99	0.97	0.96	1.00	0.97	1.72	1.03
Madagascar	20 to 24	1.02	0.92	0.53	0.38	1.14	0.99	1.63	1.50
Malawi	20 to 24	0.89	1.05	0.61	0.73	0.96	1.07	1.05	1.26
Malaysia	20 to 24	1.05	1.02	0.90	0.92	1.04	1.00	1.42	1.19
Maldives	20 to 24	0.84	1.04	0.71	0.93	0.92	1.03	1.15	1.27
Mali	20 to 24	0.74	0.77	0.46	0.42	0.86	0.84	1.14	1.27
Malta	20 to 24	0.95	1.02	0.86	0.95	0.94	1.04	1.23	1.05
Marshall Islands	20 to 24	1.01	1.05	#####	0.53	1.01	1.13	3.77	1.53
Mauritania	20 to 24	0.95	0.97	0.69	0.76	1.02	1.00	1.64	1.42
Mauritius	20 to 24	0.95	1.13	0.84	1.03	0.98	1.12	1.25	1.97

Mexico	20 to 24	0.95	1.03	0.87	0.96	0.92	1.06	1.26	1.21
Micronesia (Federated States of)	20 to 24	1.09	1.15	0.61	0.72	1.36	1.27	1.43	1.64
Monaco	20 to 24	0.92	0.96	0.90	0.93	0.71	0.83	1.13	1.04
Mongolia	20 to 24	0.95	1.01	0.82	0.89	0.87	0.91	1.07	1.10
Montenegro	20 to 24	1.01	1.03	0.87	0.97	0.95	0.99	1.16	1.09
Morocco	20 to 24	0.77	0.91	0.57	0.80	0.87	0.94	0.84	1.03
Mozambique	20 to 24	0.97	1.12	0.73	0.82	1.02	1.16	1.08	1.38
Myanmar	20 to 24	0.65	1.18	0.33	0.94	0.71	1.14	1.70	2.32
Namibia	20 to 24	1.06	1.11	0.81	0.96	1.00	1.02	1.14	1.21
Nauru	20 to 24	0.95	0.90	0.43	0.29	1.09	0.86	1.97	2.02
Nepal	20 to 24	0.93	1.10	0.78	0.98	0.99	1.14	1.06	1.28
Netherlands	20 to 24	0.97	0.98	0.93	0.94	0.92	0.94	1.06	1.01
New Zealand	20 to 24	1.00	1.00	0.95	0.95	0.90	0.98	1.34	1.07
Nicaragua	20 to 24	0.86	0.95	0.74	0.88	0.86	0.95	1.10	1.11
Niger	20 to 24	0.92	0.91	0.71	0.70	0.99	0.98	1.15	1.16
Nigeria	20 to 24	1.01	1.04	0.84	0.87	1.01	1.04	1.18	1.32
Niue	20 to 24	1.09	0.74	0.81	0.37	1.22	0.72	1.29	1.21
North Macedonia	20 to 24	0.86	0.98	0.79	0.89	0.64	0.91	1.18	1.19
Northern Mariana Islands	20 to 24	1.04	1.11	0.83	0.92	0.95	1.13	1.26	1.18
Norway	20 to 24	1.00	0.99	0.94	0.94	1.02	0.97	1.00	1.01
Oman	20 to 24	1.01	0.99	0.97	0.95	0.99	1.00	1.16	1.11
Pakistan	20 to 24	0.88	0.82	0.74	0.64	0.87	0.71	1.00	1.04
Palau	20 to 24	1.04	0.60	0.67	0.17	1.00	0.45	1.56	1.32
Palestine	20 to 24	1.06	1.06	1.03	1.06	1.00	1.00	1.73	1.48
Panama	20 to 24	0.87	0.96	0.83	0.90	0.90	0.95	1.06	1.07
Papua New Guinea	20 to 24	0.74	0.76	0.31	0.27	0.88	0.89	1.01	1.05
Paraguay	20 to 24	0.76	0.96	0.68	0.90	0.66	0.88	1.01	1.12
Peru	20 to 24	0.98	1.02	0.80	0.91	0.90	0.90	1.70	1.31
Philippines	20 to 24	1.09	1.07	1.03	0.92	1.00	0.98	1.52	1.50
Poland	20 to 24	1.08	1.01	1.01	0.98	1.03	0.99	1.52	1.11
Portugal	20 to 24	1.03	0.98	0.88	0.97	0.93	0.94	17.93	1.04
Puerto Rico	20 to 24	1.05	1.01	0.95	0.94	1.11	1.04	1.20	1.06
Qatar	20 to 24	0.99	1.00	0.89	0.92	1.12	1.03	1.05	1.05
Republic of Korea	20 to 24	0.98	0.99	0.89	0.93	0.90	0.97	1.22	1.05
Republic of Moldova	20 to 24	1.03	1.01	0.91	0.95	1.00	1.01	1.32	1.11
Romania	20 to 24	1.00	1.05	0.92	0.99	0.94	1.03	1.38	1.27
Russian Federation	20 to 24	1.04	1.09	0.94	1.08	1.05	1.02	1.69	1.43
Rwanda	20 to 24	0.69	1.03	0.35	0.83	0.87	1.06	0.98	1.08
Saint Kitts and Nevis	20 to 24	0.96	1.04	0.82	0.93	1.01	1.13	1.24	1.10
Saint Lucia	20 to 24	1.12	1.14	1.05	1.10	1.13	1.11	1.42	1.37
Saint Vincent and the Grenadines	20 to 24	0.88	1.00	0.71	0.92	0.96	0.99	0.95	1.09
Samoa	20 to 24	1.09	1.05	0.83	0.81	1.18	1.10	1.16	1.22
San Marino	20 to 24	0.99	0.99	0.93	0.93	0.98	0.98	1.12	1.04
Sao Tome and Principe	20 to 24	0.88	1.04	0.68	0.85	0.93	1.14	1.10	1.84
Saudi Arabia	20 to 24	0.76	0.98	0.50	0.89	0.93	0.98	0.93	1.54
Senegal	20 to 24	0.96	0.98	0.68	0.73	1.03	1.02	1.65	1.51
Serbia	20 to 24	0.98	0.99	0.89	0.93	0.80	0.94	1.51	1.14
Seychelles	20 to 24	1.03	0.87	0.87	0.76	0.99	0.87	2.36	1.03

Sierra Leone	20 to 24	0.99	0.84	0.76	0.58	1.02	0.90	1.54	1.50
Singapore	20 to 24	0.96	1.00	0.91	0.94	0.95	1.00	0.99	1.03
Slovakia	20 to 24	1.01	0.99	0.93	0.93	0.96	0.96	1.30	1.12
Slovenia	20 to 24	1.04	0.98	0.97	0.90	1.02	0.99	1.31	1.06
Solomon Islands	20 to 24	0.99	1.01	0.81	0.82	1.06	1.06	1.03	1.07
Somalia	20 to 24	1.03	1.05	0.67	0.74	1.12	1.08	1.06	1.09
South Africa	20 to 24	0.95	1.12	0.66	1.04	0.93	1.01	1.09	1.26
South Sudan	20 to 24	0.98	0.98	0.70	0.66	1.03	1.02	1.09	1.11
Spain	20 to 24	1.05	1.00	0.94	0.96	1.04	0.98	1.43	1.06
Sri Lanka	20 to 24	1.06	1.06	0.89	0.96	1.00	1.00	4.05	2.12
Sudan	20 to 24	0.64	0.83	0.39	0.64	0.83	0.88	0.66	1.00
Suriname	20 to 24	1.04	1.02	0.85	0.86	1.08	1.03	1.56	1.36
Sweden	20 to 24	0.99	1.01	1.00	1.00	0.95	0.98	1.05	1.03
Switzerland	20 to 24	0.99	0.99	0.94	0.95	0.90	0.99	1.31	1.02
Syrian Arab Republic	20 to 24	0.98	1.02	0.82	0.89	1.00	1.00	1.80	1.50
Taiwan (Province of China)	20 to 24	1.01	1.03	0.92	0.98	0.97	1.02	1.22	1.16
Tajikistan	20 to 24	0.80	0.96	0.61	0.83	0.80	0.94	1.51	1.40
Thailand	20 to 24	1.09	1.21	0.93	1.03	1.16	1.24	1.28	1.59
Timor-Leste	20 to 24	0.91	0.98	0.74	0.78	0.96	0.98	1.20	1.69
Togo	20 to 24	0.88	0.92	0.60	0.66	0.95	0.97	1.37	1.52
Tokelau	20 to 24	1.04	0.70	0.69	0.27	1.12	0.64	1.33	1.42
Tonga	20 to 24	1.00	1.07	0.83	0.88	1.06	1.12	1.10	1.20
Trinidad and Tobago	20 to 24	1.10	1.10	0.94	0.96	1.19	1.32	2.32	1.42
Tunisia	20 to 24	0.94	1.00	0.82	0.91	0.98	0.99	1.07	1.29
Turkey	20 to 24	1.01	1.02	0.88	0.94	1.08	1.05	1.29	1.19
Turkmenistan	20 to 24	0.92	1.08	0.79	0.95	0.91	1.03	1.63	5.50
Tuvalu	20 to 24	1.02	1.14	0.52	0.75	1.24	1.19	1.48	1.72
Uganda	20 to 24	1.10	1.07	0.85	0.83	1.12	1.09	1.09	1.12
Ukraine	20 to 24	1.06	1.04	0.98	0.94	1.03	1.01	1.68	1.32
United Arab Emirates	20 to 24	0.96	1.10	0.90	1.17	0.97	1.04	1.10	1.44
United Kingdom	20 to 24	0.99	1.00	0.94	0.96	0.88	0.97	1.11	1.03
United Republic of Tanzania	20 to 24	1.08	1.05	0.84	0.87	1.11	1.08	1.11	1.19
United States Virgin Islands	20 to 24	1.19	1.53	1.06	1.60	1.25	1.43	1.42	3.37
United States of America	20 to 24	0.97	1.00	0.94	0.97	0.96	1.01	1.03	1.03
Uruguay	20 to 24	0.93	1.02	0.86	0.94	0.81	1.04	1.10	1.11
Uzbekistan	20 to 24	0.86	1.03	0.65	0.91	0.93	1.02	1.48	1.90
Vanuatu	20 to 24	1.14	1.17	0.80	0.83	1.29	1.25	1.40	1.49
Venezuela (Bolivarian Republic of)	20 to 24	1.01	1.06	0.89	0.92	1.03	1.14	1.58	1.70
Viet Nam	20 to 24	1.08	1.14	0.95	1.05	1.10	1.11	1.35	1.53
Yemen	20 to 24	0.93	0.92	0.79	0.78	1.00	0.97	0.97	1.07
Zambia	20 to 24	0.82	0.99	0.50	0.67	0.93	1.02	1.01	1.14
Zimbabwe	20 to 24	1.09	1.15	0.92	0.69	1.07	1.02	1.12	1.32
Afghanistan	25 to 29	0.78	0.90	0.88	0.90	0.97	1.02	0.52	0.66
Albania	25 to 29	1.06	1.01	1.06	1.00	1.04	1.00	1.03	1.02
Algeria	25 to 29	0.93	1.01	0.92	1.01	0.99	1.00	0.83	1.03
American Samoa	25 to 29	1.72	1.46	1.70	1.51	1.53	1.32	1.07	0.98
Andorra	25 to 29	0.93	0.97	0.92	0.95	0.80	0.93	0.97	0.99
Angola	25 to 29	0.97	1.02	0.87	0.93	0.98	1.01	0.96	1.00

Antigua and Barbuda	25 to 29	1.16	1.05	1.13	1.02	1.13	1.04	1.11	1.03
Argentina	25 to 29	1.07	1.05	1.02	1.03	1.08	1.02	1.01	1.03
Armenia	25 to 29	1.09	1.05	1.21	1.00	1.08	1.10	1.21	1.15
Australia	25 to 29	1.00	0.97	0.96	0.94	1.16	1.03	1.04	0.99
Austria	25 to 29	1.01	0.99	1.02	0.95	0.97	1.00	1.04	1.00
Azerbaijan	25 to 29	1.11	1.06	1.16	1.08	1.03	1.02	1.18	1.09
Bahamas	25 to 29	1.17	1.19	1.20	1.20	1.06	1.13	1.13	1.17
Bahrain	25 to 29	1.08	1.08	1.15	1.13	1.05	1.03	1.05	1.12
Bangladesh	25 to 29	3.20	1.97	2.64	1.94	2.45	1.52	1.32	1.52
Barbados	25 to 29	1.19	1.10	1.13	1.06	1.17	1.10	1.23	1.11
Belarus	25 to 29	1.21	1.10	1.29	1.12	1.09	1.08	1.43	1.12
Belgium	25 to 29	1.07	0.99	1.08	0.96	0.94	1.00	1.16	1.00
Belize	25 to 29	2.68	1.21	3.01	1.26	2.21	1.08	2.10	1.25
Benin	25 to 29	0.98	0.99	0.86	0.87	1.01	1.00	1.09	1.11
Bermuda	25 to 29	1.09	1.01	1.02	0.92	1.09	1.04	1.26	1.19
Bhutan	25 to 29	0.96	1.06	0.93	1.03	0.97	1.03	0.93	1.05
Bolivia (Plurinational State of)	25 to 29	0.71	0.96	0.73	0.94	0.68	0.85	0.84	1.02
Bosnia and Herzegovina	25 to 29	1.08	0.99	1.09	1.01	1.23	1.06	1.12	1.03
Botswana	25 to 29	1.21	1.05	1.05	0.97	1.06	1.02	1.13	1.07
Brazil	25 to 29	1.23	1.11	1.38	1.18	0.99	1.01	1.31	1.11
Brunei Darussalam	25 to 29	1.21	1.30	1.13	1.25	1.21	1.55	1.19	1.18
Bulgaria	25 to 29	1.24	1.19	1.23	1.30	1.12	1.10	1.32	1.21
Burkina Faso	25 to 29	0.99	1.03	0.88	0.92	1.03	1.05	1.07	1.11
Burundi	25 to 29	1.06	1.05	0.98	0.96	1.04	1.05	0.88	0.97
Cabo Verde	25 to 29	1.24	1.06	1.13	0.96	1.22	1.07	1.78	1.25
Cambodia	25 to 29	0.87	1.06	0.80	1.02	0.93	1.02	0.92	1.04
Cameroon	25 to 29	1.03	1.04	0.94	0.93	1.08	1.09	1.12	1.19
Canada	25 to 29	0.96	0.98	0.94	0.95	0.99	1.02	1.01	1.00
Central African Republic	25 to 29	1.08	1.09	0.97	0.98	1.06	1.07	1.00	1.03
Chad	25 to 29	1.05	1.06	0.95	0.95	1.09	1.11	1.15	1.22
Chile	25 to 29	1.06	1.02	1.03	0.99	1.11	1.02	1.09	1.04
China	25 to 29	1.20	1.27	1.17	1.33	1.09	1.09	1.14	1.25
Colombia	25 to 29	1.10	1.07	1.04	0.98	1.10	1.11	1.35	1.08
Comoros	25 to 29	0.99	1.02	0.91	0.97	1.00	1.02	0.95	1.00
Congo	25 to 29	1.09	0.97	0.98	0.87	1.05	0.98	1.06	1.00
Cook Islands	25 to 29	1.24	1.25	1.15	1.28	1.25	1.19	0.91	1.01
Costa Rica	25 to 29	1.05	1.02	0.97	0.93	1.09	1.06	1.07	1.02
Coted'Ivoire	25 to 29	1.11	1.07	1.01	0.94	1.12	1.10	1.24	1.33
Croatia	25 to 29	1.17	1.01	1.20	0.96	1.14	1.01	1.19	1.05
Cuba	25 to 29	1.12	1.09	1.12	1.09	1.07	1.06	1.23	1.15
Cyprus	25 to 29	1.01	1.02	0.96	0.98	0.98	1.01	0.99	1.01
Czechia	25 to 29	1.09	1.00	1.07	0.94	1.08	0.98	1.19	1.05
Democratic People's Republic of Korea	25 to 29	1.27	1.42	1.29	1.58	1.09	1.09	1.15	1.30
Democratic Republic of the Congo	25 to 29	0.98	1.05	0.89	0.97	0.99	1.03	0.97	1.02
Denmark	25 to 29	0.99	0.98	1.02	0.94	0.86	0.95	1.03	0.99
Djibouti	25 to 29	1.16	1.13	1.08	1.10	1.12	1.12	1.04	1.09
Dominica	25 to 29	1.04	1.04	1.00	1.00	1.04	1.02	1.05	1.06
Dominican Republic	25 to 29	1.35	1.33	1.51	1.57	1.12	1.03	1.56	1.76

Ecuador	25 to 29	1.38	1.14	1.42	1.08	1.18	1.12	1.71	1.13
Egypt	25 to 29	1.58	1.18	9.44	1.43	1.25	1.09	1.08	1.68
El Salvador	25 to 29	1.63	1.17	1.44	1.19	1.41	1.10	2.08	1.22
Equatorial Guinea	25 to 29	1.05	1.01	0.93	0.93	1.06	1.00	1.02	1.03
Eritrea	25 to 29	1.32	1.27	1.17	1.21	1.22	1.18	1.02	1.08
Estonia	25 to 29	1.10	1.04	1.12	1.03	1.13	1.06	1.12	1.06
Eswatini	25 to 29	1.11	1.30	1.02	1.27	1.01	1.07	1.04	1.15
Ethiopia	25 to 29	0.94	1.07	0.87	0.99	0.99	1.05	0.93	0.99
Fiji	25 to 29	1.30	1.17	1.41	1.20	1.31	1.21	0.96	0.95
Finland	25 to 29	1.04	0.98	1.02	0.96	0.92	0.94	1.04	1.00
France	25 to 29	1.07	1.06	1.09	1.03	0.99	1.10	1.09	1.03
Gabon	25 to 29	1.07	1.07	0.98	1.02	1.04	1.05	1.02	1.05
Gambia	25 to 29	1.14	1.12	1.06	1.04	1.14	1.17	1.30	1.37
Georgia	25 to 29	1.20	1.15	1.15	1.12	1.09	1.15	1.13	1.21
Germany	25 to 29	1.06	0.99	1.04	0.96	1.02	0.97	1.18	1.01
Ghana	25 to 29	1.05	1.09	0.98	1.04	1.17	1.17	1.32	1.31
Greece	25 to 29	1.06	1.07	1.07	1.05	0.96	1.05	1.06	1.04
Greenland	25 to 29	0.74	0.87	0.95	0.89	0.30	0.78	0.93	0.97
Grenada	25 to 29	1.92	1.12	3.79	1.14	1.20	1.08	1.01	1.17
Guam	25 to 29	1.16	1.34	1.09	1.34	1.22	1.36	1.00	1.10
Guatemala	25 to 29	1.20	1.11	1.19	1.08	1.09	1.05	1.11	1.09
Guinea	25 to 29	0.94	0.99	0.84	0.87	1.03	1.05	1.11	1.25
Guinea-Bissau	25 to 29	0.94	1.08	0.84	0.99	1.07	1.19	1.17	1.42
Guyana	25 to 29	1.64	1.51	1.64	1.54	1.27	1.29	2.70	2.25
Haiti	25 to 29	0.00	0.48	0.28	0.59	0.00	0.30	0.00	0.55
Honduras	25 to 29	0.84	0.91	0.94	0.87	0.72	0.77	0.89	0.99
Hungary	25 to 29	1.19	1.05	1.23	1.03	1.10	1.03	1.41	1.11
Iceland	25 to 29	1.05	1.03	1.05	0.99	1.01	1.07	1.13	1.02
India	25 to 29	0.98	1.04	0.96	1.02	1.00	1.02	0.94	1.02
Indonesia	25 to 29	1.03	1.19	1.02	1.20	0.97	1.05	1.10	1.30
Iran (Islamic Republic of)	25 to 29	0.99	1.08	1.00	1.14	1.00	1.03	1.02	1.30
Iraq	25 to 29	1.20	1.11	1.30	1.10	1.02	1.01	1.11	1.12
Ireland	25 to 29	0.95	0.97	0.96	0.94	0.81	0.97	1.01	0.98
Israel	25 to 29	1.05	0.98	1.11	0.96	1.04	0.98	1.02	1.00
Italy	25 to 29	1.06	1.01	1.05	1.00	0.97	0.97	1.09	1.02
Jamaica	25 to 29	1.25	0.97	1.10	0.98	1.27	0.89	1.40	1.05
Japan	25 to 29	1.11	1.07	1.06	1.06	1.18	1.03	1.07	1.05
Jordan	25 to 29	1.02	1.05	1.24	1.13	1.01	1.01	1.00	1.15
Kazakhstan	25 to 29	1.10	1.08	1.15	1.11	1.14	1.09	1.38	1.16
Kenya	25 to 29	1.04	1.06	0.97	0.99	1.03	1.05	0.99	1.02
Kiribati	25 to 29	1.44	1.92	1.14	1.64	1.97	1.74	1.04	1.18
Kuwait	25 to 29	0.98	1.03	1.02	1.04	0.97	1.01	0.97	1.14
Kyrgyzstan	25 to 29	1.17	1.22	1.21	1.22	1.10	1.17	1.52	1.91
Lao People's Democratic Republic	25 to 29	0.97	1.13	0.95	1.08	0.92	1.03	0.93	1.27
Latvia	25 to 29	1.18	1.10	1.40	1.19	1.10	1.09	1.32	1.13
Lebanon	25 to 29	1.25	1.08	1.63	1.16	1.13	1.06	0.99	1.06
Lesotho	25 to 29	1.09	1.28	1.05	1.24	1.02	1.06	1.02	1.14
Liberia	25 to 29	0.99	0.96	0.87	0.83	1.04	1.03	1.16	1.19

Libya	25 to 29	1.04	1.05	1.08	1.10	1.01	0.97	1.02	1.33
Lithuania	25 to 29	1.12	1.03	1.18	1.01	1.16	1.03	1.19	1.05
Luxembourg	25 to 29	1.08	0.96	1.05	0.94	1.03	0.93	1.15	0.98
Madagascar	25 to 29	1.21	1.24	1.03	1.12	1.10	1.11	1.07	1.11
Malawi	25 to 29	0.93	1.15	0.82	1.11	0.97	1.10	0.94	1.07
Malaysia	25 to 29	1.15	1.07	1.08	1.04	1.08	1.02	1.21	1.11
Maldives	25 to 29	0.95	1.14	0.91	1.09	0.99	1.06	1.01	1.23
Mali	25 to 29	0.78	0.89	0.65	0.71	0.88	0.93	0.95	1.06
Malta	25 to 29	0.99	0.98	0.96	0.95	0.95	0.99	1.01	0.99
Marshall Islands	25 to 29	1.01	2.34	1.01	1.93	#####	1.69	1.02	0.90
Mauritania	25 to 29	1.06	1.02	0.96	0.90	1.12	1.05	1.31	1.20
Mauritius	25 to 29	1.18	1.35	1.16	1.38	1.12	1.21	1.42	1.58
Mexico	25 to 29	1.00	1.13	0.98	1.09	0.98	1.18	1.04	1.12
Micronesia (Federated States of)	25 to 29	4.12	2.48	2.84	2.14	5.90	1.98	0.92	1.00
Monaco	25 to 29	1.02	1.02	1.08	1.08	0.85	0.91	1.04	1.05
Mongolia	25 to 29	1.02	1.13	1.00	1.11	0.98	1.10	1.03	1.06
Montenegro	25 to 29	1.34	1.04	1.44	1.10	1.05	0.97	1.09	1.03
Morocco	25 to 29	0.91	0.94	0.90	0.89	0.98	0.97	0.76	0.88
Mozambique	25 to 29	1.06	1.37	0.99	1.36	1.06	1.27	1.01	1.21
Myanmar	25 to 29	0.97	1.62	0.92	1.66	0.98	1.26	1.19	1.64
Namibia	25 to 29	1.08	1.16	0.99	1.17	1.01	1.04	1.06	1.12
Nauru	25 to 29	1.62	1.48	1.54	1.55	2.13	1.21	0.84	0.82
Nepal	25 to 29	0.96	1.12	0.95	1.11	1.00	1.10	0.90	1.08
Netherlands	25 to 29	0.97	0.97	0.99	0.94	0.88	0.95	1.00	0.98
New Zealand	25 to 29	1.02	1.01	1.00	0.96	1.07	1.06	1.10	1.02
Nicaragua	25 to 29	0.96	1.02	0.91	0.98	0.96	1.00	0.99	1.06
Niger	25 to 29	0.97	0.98	0.87	0.89	1.02	1.03	1.04	1.07
Nigeria	25 to 29	1.01	1.06	0.91	0.96	1.03	1.06	1.08	1.19
Niue	25 to 29	1.45	0.91	1.36	0.81	1.59	0.93	0.98	0.78
North Macedonia	25 to 29	0.98	1.02	1.00	0.99	0.80	0.98	0.99	1.05
Northern Mariana Islands	25 to 29	1.27	1.26	1.24	1.15	1.05	1.23	0.98	1.01
Norway	25 to 29	1.01	0.99	0.99	0.96	0.91	0.99	1.07	1.01
Oman	25 to 29	1.02	1.00	1.14	1.06	1.00	1.00	1.00	1.03
Pakistan	25 to 29	0.95	0.95	0.91	0.89	0.97	0.89	0.94	0.96
Palau	25 to 29	2.45	3.29	2.43	5.39	1.90	1.90	1.17	1.34
Palestine	25 to 29	1.15	1.14	1.55	1.45	1.02	1.01	1.23	1.27
Panama	25 to 29	0.91	0.97	0.94	0.96	0.93	0.94	0.81	0.95
Papua New Guinea	25 to 29	0.72	0.93	0.00	0.36	1.07	1.14	0.75	0.82
Paraguay	25 to 29	0.89	1.05	0.88	1.05	0.83	0.97	0.96	1.06
Peru	25 to 29	1.13	1.22	1.11	1.25	1.02	1.08	1.11	1.16
Philippines	25 to 29	1.46	1.33	2.41	1.40	1.08	1.07	1.43	1.36
Poland	25 to 29	1.23	1.12	1.26	1.18	1.16	1.08	1.25	1.14
Portugal	25 to 29	1.19	1.01	1.14	1.02	0.99	0.95	1.92	1.00
Puerto Rico	25 to 29	1.12	1.04	1.10	0.99	1.09	1.06	1.27	1.07
Qatar	25 to 29	1.01	1.01	1.01	0.98	1.15	1.05	0.98	1.02
Republic of Korea	25 to 29	1.12	1.05	1.04	1.00	1.18	1.06	1.13	1.04
Republic of Moldova	25 to 29	1.20	1.12	1.42	1.32	1.08	1.06	1.15	1.09
Romania	25 to 29	1.12	1.11	1.17	1.15	1.01	1.06	1.21	1.16

Russian Federation	25 to 29	1.21	1.24	1.34	1.50	1.16	1.05	1.32	1.33
Rwanda	25 to 29	0.89	1.10	0.85	1.10	0.98	1.09	0.85	1.01
Saint Kitts and Nevis	25 to 29	0.67	1.06	0.61	1.01	0.82	1.08	0.61	1.07
Saint Lucia	25 to 29	1.06	1.19	1.06	1.29	0.98	1.03	1.00	1.26
Saint Vincent and the Grenadines	25 to 29	1.20	1.07	1.59	1.12	0.89	0.95	1.25	1.09
Samoa	25 to 29	1.40	1.40	1.35	1.42	1.48	1.34	0.95	0.98
San Marino	25 to 29	1.01	0.99	1.00	0.96	0.99	0.99	1.07	1.01
Sao Tome and Principe	25 to 29	0.96	1.08	0.85	1.05	1.02	1.16	1.08	1.25
Saudi Arabia	25 to 29	0.88	1.09	0.87	1.22	0.98	1.00	0.71	1.29
Senegal	25 to 29	1.05	1.05	0.93	0.93	1.09	1.08	1.25	1.19
Serbia	25 to 29	1.10	1.03	1.11	1.01	0.95	0.97	1.18	1.07
Seychelles	25 to 29	1.09	1.10	1.06	1.07	1.01	1.02	1.30	1.29
Sierra Leone	25 to 29	1.03	0.96	0.90	0.81	1.06	1.00	1.24	1.24
Singapore	25 to 29	1.03	1.03	1.01	0.99	1.00	1.02	0.96	1.01
Slovakia	25 to 29	1.07	1.03	1.11	1.02	1.01	1.00	1.13	1.07
Slovenia	25 to 29	1.10	1.00	1.09	0.94	1.07	1.01	1.16	1.04
Solomon Islands	25 to 29	1.32	1.32	1.26	1.28	1.39	1.29	0.96	0.97
Somalia	25 to 29	1.08	1.15	0.99	1.11	1.08	1.09	0.96	1.01
South Africa	25 to 29	1.09	1.24	0.96	1.44	0.91	1.04	1.11	1.19
South Sudan	25 to 29	1.05	1.05	0.96	0.98	1.04	1.04	0.98	1.00
Spain	25 to 29	1.14	1.01	1.10	1.00	1.10	0.99	1.14	1.01
Sri Lanka	25 to 29	1.21	1.18	1.25	1.23	1.04	1.04	1.80	1.66
Sudan	25 to 29	0.78	0.97	0.81	0.96	0.97	1.00	0.52	0.93
Suriname	25 to 29	1.32	1.18	1.38	1.21	1.09	1.03	1.51	1.29
Sweden	25 to 29	0.97	1.01	1.00	1.01	0.90	1.00	1.02	1.01
Switzerland	25 to 29	1.04	0.99	1.06	0.95	0.95	0.99	1.14	0.99
Syrian Arab Republic	25 to 29	1.20	1.14	1.38	1.25	1.07	1.03	1.15	1.19
Taiwan (Province of China)	25 to 29	1.11	1.06	1.10	1.05	1.00	1.03	1.14	1.09
Tajikistan	25 to 29	0.98	1.10	0.99	1.19	0.91	1.04	1.24	1.23
Thailand	25 to 29	1.20	1.62	1.10	1.54	1.21	1.44	1.17	1.55
Timor-Leste	25 to 29	1.16	1.24	1.15	1.23	1.10	1.10	1.16	1.36
Togo	25 to 29	0.96	1.03	0.83	0.91	1.02	1.07	1.14	1.21
Tokelau	25 to 29	1.49	1.13	1.40	1.33	1.50	1.04	0.85	0.75
Tonga	25 to 29	1.07	1.16	1.03	1.12	1.14	1.19	0.97	0.99
Trinidad and Tobago	25 to 29	1.31	1.26	1.29	1.20	1.29	1.37	1.77	1.51
Tunisia	25 to 29	1.01	1.07	1.02	1.10	1.02	1.02	0.97	1.21
Turkey	25 to 29	1.13	1.06	1.10	1.05	1.25	1.11	1.13	1.10
Turkmenistan	25 to 29	0.98	1.47	0.97	1.79	1.00	1.43	1.00	1.79
Tuvalu	25 to 29	1.95	1.91	1.47	1.85	2.23	1.62	0.79	0.97
Uganda	25 to 29	1.13	1.13	1.07	1.09	1.10	1.09	1.00	1.02
Ukraine	25 to 29	1.15	1.19	1.20	1.23	1.09	1.08	1.35	1.39
United Arab Emirates	25 to 29	0.94	0.96	0.92	0.99	0.97	0.95	0.97	0.98
United Kingdom	25 to 29	1.01	1.01	1.00	1.00	0.95	0.99	1.02	1.00
United Republic of Tanzania	25 to 29	1.11	1.09	1.02	1.07	1.09	1.08	1.00	1.07
United States Virgin Islands	25 to 29	1.19	1.75	1.22	2.35	1.08	1.54	1.25	2.01
United States of America	25 to 29	0.96	1.00	0.97	0.99	0.97	1.06	0.99	1.00
Uruguay	25 to 29	1.06	1.08	1.00	1.03	1.11	1.12	1.04	1.05
Uzbekistan	25 to 29	1.08	1.15	1.29	1.18	1.01	1.10	1.18	1.40

Vanuatu	25 to 29	3.50	2.30	6.11	2.53	4.10	2.15	1.10	1.10
Venezuela (Bolivarian Republic of)	25 to 29	1.09	1.12	1.08	1.06	1.04	1.19	1.06	1.13
Viet Nam	25 to 29	1.32	1.21	1.33	1.23	1.19	1.13	1.31	1.28
Yemen	25 to 29	1.04	1.02	1.15	1.03	1.07	1.04	0.84	1.00
Zambia	25 to 29	0.89	1.10	0.77	0.99	0.96	1.06	0.92	1.02
Zimbabwe	25 to 29	1.08	1.30	1.01	1.34	1.07	1.11	1.05	1.19
Afghanistan	30 to 34	0.81	0.87	1.16	0.98	0.97	1.00	0.51	0.65
Albania	30 to 34	1.03	1.04	1.06	1.07	1.03	1.02	1.03	1.04
Algeria	30 to 34	0.85	0.95	0.90	0.95	0.93	0.96	0.73	0.93
American Samoa	30 to 34	1.53	1.28	1.80	1.53	1.07	0.95	1.30	1.17
Andorra	30 to 34	0.91	0.97	0.94	0.97	0.77	0.93	1.00	1.02
Angola	30 to 34	0.99	1.08	0.99	1.08	1.00	1.05	0.99	1.06
Antigua and Barbuda	30 to 34	1.51	1.02	1.89	1.02	1.56	1.05	1.24	1.03
Argentina	30 to 34	1.06	1.02	1.11	1.06	1.16	1.02	0.99	1.02
Armenia	30 to 34	1.10	1.07	1.35	1.11	1.15	1.15	1.15	1.15
Australia	30 to 34	1.01	0.96	1.02	0.98	1.22	1.04	1.05	1.00
Austria	30 to 34	0.98	0.99	1.05	0.97	0.93	1.00	1.05	1.02
Azerbaijan	30 to 34	1.16	1.12	1.29	1.24	1.07	1.06	1.19	1.13
Bahamas	30 to 34	1.12	1.24	1.19	1.34	1.13	1.17	1.09	1.22
Bahrain	30 to 34	1.05	1.08	1.08	1.15	1.04	1.05	1.01	1.11
Bangladesh	30 to 34	1.01	3.09	1.01	4.34	14.38	2.07	1.70	1.92
Barbados	30 to 34	1.20	1.18	1.26	1.27	1.23	1.19	1.26	1.20
Belarus	30 to 34	1.30	1.25	1.51	1.51	1.15	1.21	1.40	1.22
Belgium	30 to 34	1.02	0.96	1.08	0.94	0.95	0.97	1.14	0.99
Belize	30 to 34	1.33	1.22	1.43	1.33	1.34	1.15	1.31	1.22
Benin	30 to 34	0.99	1.01	0.95	0.97	1.06	1.05	1.11	1.15
Bermuda	30 to 34	1.11	1.03	1.09	0.98	1.16	1.08	1.24	1.11
Bhutan	30 to 34	1.07	1.11	1.22	1.21	1.09	1.11	0.99	1.10
Bolivia (Plurinational State of)	30 to 34	0.85	0.99	1.10	1.10	0.80	0.91	0.86	1.02
Bosnia and Herzegovina	30 to 34	1.13	0.97	1.31	1.05	1.42	1.02	1.21	1.02
Botswana	30 to 34	1.26	1.09	1.15	1.08	1.11	1.05	1.15	1.10
Brazil	30 to 34	1.51	1.10	2.31	1.33	1.26	1.04	1.62	1.08
Brunei Darussalam	30 to 34	1.08	1.25	1.13	1.33	1.06	1.48	0.97	1.08
Bulgaria	30 to 34	1.35	1.27	1.44	1.55	1.22	1.20	1.51	1.29
Burkina Faso	30 to 34	0.95	1.06	0.94	1.06	1.07	1.12	1.07	1.15
Burundi	30 to 34	1.25	1.15	1.41	1.26	1.15	1.11	0.90	0.99
Cabo Verde	30 to 34	1.07	1.01	1.04	0.97	1.17	1.08	1.51	1.20
Cambodia	30 to 34	0.86	1.09	0.88	1.12	1.03	1.06	0.94	1.04
Cameroon	30 to 34	0.98	1.04	1.00	1.03	1.12	1.15	1.11	1.22
Canada	30 to 34	0.98	0.99	0.98	0.98	1.10	1.08	1.03	1.02
Central African Republic	30 to 34	1.17	1.27	1.16	1.32	1.11	1.16	1.03	1.09
Chad	30 to 34	0.98	1.04	0.96	1.02	1.09	1.14	1.12	1.22
Chile	30 to 34	1.03	1.01	1.09	1.04	1.08	1.02	1.05	1.04
China	30 to 34	1.33	1.42	1.36	1.53	1.24	1.19	1.27	1.43
Colombia	30 to 34	1.06	1.07	1.09	1.05	1.13	1.15	1.26	1.09
Comoros	30 to 34	1.05	1.08	1.08	1.18	1.01	1.06	0.99	1.04
Congo	30 to 34	1.17	1.00	1.16	0.98	1.08	0.99	1.10	1.04
Cook Islands	30 to 34	1.15	1.20	1.29	1.32	0.96	1.05	1.13	1.13

Costa Rica	30 to 34	1.03	1.06	1.04	1.03	1.07	1.18	1.07	1.05
Coted'Ivoire	30 to 34	1.06	1.06	1.05	1.03	1.16	1.16	1.20	1.37
Croatia	30 to 34	1.24	1.05	1.36	1.07	1.27	1.07	1.28	1.08
Cuba	30 to 34	1.12	1.11	1.24	1.17	1.05	1.07	1.23	1.22
Cyprus	30 to 34	0.97	1.00	0.97	0.96	0.96	0.99	1.00	1.01
Czechia	30 to 34	1.12	1.01	1.20	1.01	1.12	1.00	1.26	1.07
Democratic People's Republic of Korea	30 to 34	1.31	1.51	1.39	1.82	1.15	1.16	1.19	1.37
Democratic Republic of the Congo	30 to 34	1.00	1.11	0.97	1.12	1.00	1.07	0.99	1.06
Denmark	30 to 34	0.99	1.00	1.11	0.98	0.87	1.01	1.10	1.04
Djibouti	30 to 34	1.23	1.22	1.24	1.32	1.18	1.20	1.06	1.15
Dominica	30 to 34	1.04	1.10	1.07	1.15	1.07	1.10	1.06	1.13
Dominican Republic	30 to 34	1.41	1.27	1.98	1.63	1.18	1.02	1.62	1.53
Ecuador	30 to 34	1.34	1.12	1.77	1.24	1.15	1.10	1.47	1.11
Egypt	30 to 34	1.35	1.10	2.86	1.35	1.25	1.08	1.03	1.31
El Salvador	30 to 34	1.49	1.22	1.44	1.36	1.48	1.19	1.84	1.29
Equatorial Guinea	30 to 34	1.12	1.03	1.10	1.02	1.11	1.01	1.06	1.06
Eritrea	30 to 34	1.63	1.53	1.67	1.79	1.38	1.31	1.04	1.15
Estonia	30 to 34	1.18	1.04	1.29	1.08	1.27	1.07	1.27	1.08
Eswatini	30 to 34	1.21	1.45	1.27	1.56	1.05	1.13	1.06	1.20
Ethiopia	30 to 34	1.02	1.14	1.08	1.17	1.04	1.10	0.94	1.00
Fiji	30 to 34	1.09	0.97	1.26	1.03	0.95	0.90	1.12	1.06
Finland	30 to 34	1.19	1.00	1.31	1.00	1.12	1.00	1.39	1.04
France	30 to 34	1.13	1.06	1.20	1.05	1.02	1.09	1.16	1.09
Gabon	30 to 34	1.12	1.11	1.11	1.14	1.07	1.08	1.05	1.09
Gambia	30 to 34	1.09	1.10	1.10	1.13	1.18	1.23	1.28	1.38
Georgia	30 to 34	1.35	1.29	1.34	1.30	1.23	1.32	1.21	1.37
Germany	30 to 34	1.02	0.98	1.09	0.98	0.95	0.95	1.14	1.04
Ghana	30 to 34	0.92	1.05	0.93	1.08	1.09	1.18	1.25	1.32
Greece	30 to 34	0.98	1.03	0.99	1.02	0.97	1.07	1.02	1.05
Greenland	30 to 34	0.85	0.86	1.20	0.97	1.01	0.71	0.99	0.99
Grenada	30 to 34	1.79	1.09	8.17	1.18	0.93	1.06	#####	1.13
Guam	30 to 34	1.07	1.24	1.12	1.39	0.98	1.08	1.11	1.22
Guatemala	30 to 34	1.05	1.13	1.10	1.20	1.08	1.07	1.02	1.13
Guinea	30 to 34	0.86	0.97	0.83	0.95	1.02	1.08	1.09	1.29
Guinea-Bissau	30 to 34	0.82	1.05	0.82	1.11	1.03	1.23	1.13	1.48
Guyana	30 to 34	2.00	1.82	2.45	2.62	1.52	1.53	3.87	2.78
Haiti	30 to 34	0.00	0.68	0.86	1.24	0.00	0.47	0.00	0.71
Honduras	30 to 34	1.01	0.87	1.77	0.90	1.18	0.76	1.14	0.99
Hungary	30 to 34	1.27	1.08	1.44	1.17	1.16	1.02	1.69	1.17
Iceland	30 to 34	0.96	1.00	0.99	0.98	0.93	1.03	1.05	1.02
India	30 to 34	1.14	1.16	1.33	1.26	1.21	1.14	1.04	1.09
Indonesia	30 to 34	1.16	1.30	1.30	1.47	1.13	1.15	1.16	1.34
Iran (Islamic Republic of)	30 to 34	1.00	1.07	1.04	1.13	1.01	1.05	1.00	1.20
Iraq	30 to 34	1.18	1.07	1.34	1.08	1.02	1.00	1.02	1.03
Ireland	30 to 34	0.98	0.98	1.01	0.96	0.94	0.99	1.08	1.01
Israel	30 to 34	1.05	1.00	1.16	1.01	1.06	1.02	1.07	1.03
Italy	30 to 34	1.08	1.04	1.11	1.05	1.05	1.04	1.13	1.03
Jamaica	30 to 34	1.15	1.05	1.17	1.21	1.26	0.92	1.20	1.14

Japan	30 to 34	1.19	1.15	1.18	1.20	1.43	1.13	1.08	1.09
Jordan	30 to 34	0.97	1.05	1.19	1.13	1.01	1.02	0.91	1.12
Kazakhstan	30 to 34	1.14	1.16	1.30	1.29	1.33	1.20	1.26	1.20
Kenya	30 to 34	1.04	1.06	1.04	1.05	1.04	1.05	0.99	1.02
Kiribati	30 to 34	1.31	1.54	1.25	1.57	1.21	1.13	1.29	1.55
Kuwait	30 to 34	0.98	1.04	1.02	1.07	0.97	1.03	0.99	1.12
Kyrgyzstan	30 to 34	1.18	1.49	1.27	1.69	1.13	1.44	1.29	3.00
Lao People's Democratic Republic	30 to 34	1.09	1.23	1.32	1.33	1.13	1.13	0.95	1.28
Latvia	30 to 34	1.26	1.19	1.63	1.64	1.18	1.16	1.44	1.22
Lebanon	30 to 34	1.19	1.06	1.76	1.13	1.17	1.07	0.93	1.04
Lesotho	30 to 34	1.17	1.56	1.24	1.84	1.06	1.17	1.05	1.21
Liberia	30 to 34	0.98	0.99	0.96	0.97	1.08	1.09	1.17	1.24
Libya	30 to 34	0.90	0.99	0.91	1.00	0.94	0.93	0.83	1.13
Lithuania	30 to 34	1.17	1.11	1.34	1.23	1.27	1.17	1.28	1.14
Luxembourg	30 to 34	1.00	0.97	1.01	0.97	0.94	0.96	1.10	0.99
Madagascar	30 to 34	1.26	1.34	1.21	1.47	1.10	1.09	1.10	1.14
Malawi	30 to 34	0.89	1.31	0.86	1.66	0.94	1.19	0.93	1.12
Malaysia	30 to 34	1.21	1.15	1.21	1.19	1.15	1.07	1.23	1.15
Maldives	30 to 34	1.11	1.17	1.14	1.18	1.15	1.09	1.13	1.20
Mali	30 to 34	0.68	0.82	0.62	0.69	0.81	0.89	0.91	1.03
Malta	30 to 34	0.93	0.93	0.91	0.93	0.93	0.92	0.97	0.97
Marshall Islands	30 to 34	2.15	1.51	2.94	2.01	1.19	0.89	1.32	1.31
Mauritania	30 to 34	1.05	1.00	1.06	0.95	1.16	1.08	1.32	1.21
Mauritius	30 to 34	1.45	1.92	1.60	2.39	1.33	1.68	1.82	1.93
Mexico	30 to 34	1.03	1.19	1.08	1.29	1.06	1.28	1.10	1.19
Micronesia (Federated States of)	30 to 34	2.07	1.68	2.89	2.14	1.12	0.99	1.37	1.43
Monaco	30 to 34	1.02	1.05	1.15	1.17	0.87	0.95	1.08	1.09
Mongolia	30 to 34	1.02	1.20	1.06	1.21	1.01	1.21	1.03	1.11
Montenegro	30 to 34	1.30	1.14	1.41	1.32	1.04	1.01	1.11	1.07
Morocco	30 to 34	0.87	0.86	0.92	0.84	0.94	0.92	0.71	0.75
Mozambique	30 to 34	1.12	1.84	1.17	2.41	1.12	1.56	1.04	1.40
Myanmar	30 to 34	1.19	1.84	1.45	2.03	1.26	1.40	1.26	1.54
Namibia	30 to 34	1.14	1.26	1.13	1.39	1.04	1.09	1.09	1.17
Nauru	30 to 34	1.16	1.09	1.41	1.34	0.69	0.64	1.37	1.30
Nepal	30 to 34	1.12	1.24	1.34	1.44	1.22	1.24	0.97	1.15
Netherlands	30 to 34	0.97	0.96	1.03	0.95	0.95	0.94	1.03	1.00
New Zealand	30 to 34	0.95	1.03	1.01	1.01	0.94	1.18	1.07	1.05
Nicaragua	30 to 34	1.06	1.05	1.15	1.10	1.08	1.07	1.13	1.11
Niger	30 to 34	0.92	0.98	0.91	0.97	1.02	1.08	1.02	1.08
Nigeria	30 to 34	1.00	1.08	0.99	1.08	1.08	1.13	1.10	1.25
Niue	30 to 34	1.26	0.94	1.42	1.01	1.03	0.81	1.18	1.04
North Macedonia	30 to 34	1.05	1.01	1.14	1.06	0.92	0.91	1.12	1.06
Northern Mariana Islands	30 to 34	1.07	1.17	1.14	1.20	0.78	1.00	1.18	1.13
Norway	30 to 34	1.01	0.99	1.07	0.98	0.91	0.98	1.12	1.02
Oman	30 to 34	0.99	1.00	1.15	1.11	0.98	1.00	0.95	0.99
Pakistan	30 to 34	1.05	1.07	1.14	1.11	1.12	1.05	1.01	1.08
Palau	30 to 34	1.89	1.94	2.38	2.85	1.12	1.07	1.59	1.71
Palestine	30 to 34	1.09	1.07	1.45	1.27	1.02	1.02	1.10	1.11

Panama	30 to 34	0.99	1.06	1.07	1.15	1.01	1.06	0.98	1.04
Papua New Guinea	30 to 34	0.76	1.01	0.00	0.74	0.86	0.96	0.92	0.99
Paraguay	30 to 34	0.88	1.06	0.99	1.15	0.78	0.95	1.03	1.10
Peru	30 to 34	1.08	1.18	1.18	1.33	1.06	1.09	1.01	1.12
Philippines	30 to 34	1.97	1.51	93.96	1.76	1.21	1.16	1.40	1.35
Poland	30 to 34	1.32	1.24	1.49	1.41	1.26	1.20	1.47	1.30
Portugal	30 to 34	1.15	1.02	1.16	1.07	1.03	0.97	2.02	1.02
Puerto Rico	30 to 34	1.24	1.10	1.33	1.14	1.24	1.14	1.41	1.13
Qatar	30 to 34	1.00	1.01	1.03	1.00	1.13	1.05	0.99	1.02
Republic of Korea	30 to 34	1.23	1.10	1.21	1.10	2.25	1.11	1.14	1.07
Republic of Moldova	30 to 34	1.25	1.16	1.50	1.49	1.18	1.11	1.22	1.13
Romania	30 to 34	1.22	1.19	1.41	1.40	1.05	1.13	1.42	1.21
Russian Federation	30 to 34	1.31	1.42	1.51	1.89	1.35	1.24	1.61	1.66
Rwanda	30 to 34	0.93	1.17	1.14	1.37	0.99	1.14	0.83	1.02
Saint Kitts and Nevis	30 to 34	0.72	1.05	0.82	1.01	0.80	1.14	0.77	1.07
Saint Lucia	30 to 34	1.74	1.24	2.31	1.46	1.46	1.06	2.05	1.30
Saint Vincent and the Grenadines	30 to 34	1.34	1.18	2.51	1.36	0.92	1.04	1.34	1.19
Samoa	30 to 34	1.28	1.22	1.54	1.50	1.03	0.94	1.11	1.16
San Marino	30 to 34	1.03	1.00	1.07	0.98	1.02	1.00	1.12	1.04
Sao Tome and Principe	30 to 34	1.01	0.97	1.04	1.01	1.10	1.09	1.16	1.12
Saudi Arabia	30 to 34	0.85	1.08	0.92	1.33	0.96	0.98	0.62	1.20
Senegal	30 to 34	1.02	1.04	1.00	1.00	1.12	1.14	1.23	1.20
Serbia	30 to 34	1.13	1.08	1.26	1.16	0.97	1.00	1.27	1.13
Seychelles	30 to 34	1.17	1.13	1.21	1.15	1.12	1.08	1.30	1.19
Sierra Leone	30 to 34	1.00	0.96	0.97	0.91	1.09	1.04	1.25	1.29
Singapore	30 to 34	1.07	1.06	1.12	1.04	1.07	1.04	0.96	1.06
Slovakia	30 to 34	1.20	1.08	1.44	1.19	1.11	1.06	1.30	1.13
Slovenia	30 to 34	1.16	1.02	1.23	1.00	1.11	1.06	1.23	1.06
Solomon Islands	30 to 34	1.37	1.38	1.49	1.55	1.16	1.08	1.12	1.15
Somalia	30 to 34	1.20	1.31	1.28	1.60	1.15	1.18	0.97	1.04
South Africa	30 to 34	1.36	1.24	1.72	1.42	0.90	1.02	1.25	1.15
South Sudan	30 to 34	1.08	1.15	1.07	1.24	1.06	1.10	0.99	1.03
Spain	30 to 34	1.13	1.03	1.13	1.06	1.14	0.99	1.19	1.04
Sri Lanka	30 to 34	1.26	1.20	1.40	1.37	1.10	1.07	1.58	1.45
Sudan	30 to 34	0.74	0.92	0.92	0.93	0.93	0.97	0.44	0.85
Suriname	30 to 34	1.46	1.43	1.83	1.89	1.20	1.14	1.62	1.55
Sweden	30 to 34	0.98	1.00	1.04	1.00	0.91	0.99	1.03	1.00
Switzerland	30 to 34	0.98	0.97	1.03	0.95	0.88	0.96	1.09	1.01
Syrian Arab Republic	30 to 34	1.04	1.05	1.21	1.15	1.02	1.02	0.92	1.02
Taiwan (Province of China)	30 to 34	1.19	1.15	1.22	1.21	1.05	1.10	1.17	1.08
Tajikistan	30 to 34	1.00	1.14	1.09	1.37	0.92	1.08	1.25	1.27
Thailand	30 to 34	1.22	2.35	1.17	2.54	1.33	2.05	1.16	1.78
Timor-Leste	30 to 34	1.30	1.42	1.51	1.60	1.24	1.24	1.17	1.44
Togo	30 to 34	0.87	1.01	0.82	0.98	1.00	1.12	1.11	1.25
Tokelau	30 to 34	1.32	0.98	1.84	1.43	0.98	0.74	1.20	1.09
Tonga	30 to 34	0.95	1.03	1.02	1.08	0.93	0.96	1.03	1.06
Trinidad and Tobago	30 to 34	1.44	1.27	1.71	1.42	1.65	1.45	2.67	1.49
Tunisia	30 to 34	0.96	1.05	1.02	1.11	0.99	1.02	0.89	1.16

Turkey	30 to 34	1.26	1.06	1.31	1.08	1.55	1.13	1.26	1.06
Turkmenistan	30 to 34	1.03	1.32	1.10	1.52	1.08	1.32	1.01	1.34
Tuvalu	30 to 34	1.47	1.33	2.21	1.69	0.90	0.89	1.28	1.36
Uganda	30 to 34	1.22	1.25	1.29	1.35	1.16	1.18	1.01	1.04
Ukraine	30 to 34	1.20	1.32	1.35	1.56	1.18	1.28	1.38	1.53
United Arab Emirates	30 to 34	0.98	0.97	1.01	0.98	0.96	0.96	1.02	1.01
United Kingdom	30 to 34	1.02	1.00	1.03	1.02	1.05	0.99	1.08	1.03
United Republic of Tanzania	30 to 34	1.14	1.14	1.14	1.24	1.11	1.12	1.00	1.09
United States Virgin Islands	30 to 34	1.56	1.63	2.00	2.71	1.39	1.45	1.64	1.70
United States of America	30 to 34	0.98	1.01	1.04	1.03	1.11	1.13	1.00	0.99
Uruguay	30 to 34	0.98	1.05	1.10	1.09	1.00	1.15	0.98	1.03
Uzbekistan	30 to 34	1.08	1.18	1.35	1.27	1.04	1.15	1.12	1.38
Vanuatu	30 to 34	2.04	1.61	3.32	2.06	1.19	1.02	1.43	1.37
Venezuela (Bolivarian Republic of)	30 to 34	1.15	1.20	1.33	1.32	1.05	1.29	1.08	1.13
Viet Nam	30 to 34	1.61	1.39	1.80	1.54	1.37	1.23	1.43	1.36
Yemen	30 to 34	1.02	1.05	1.33	1.17	1.10	1.06	0.76	1.09
Zambia	30 to 34	0.85	1.18	0.83	1.21	0.91	1.08	0.90	1.04
Zimbabwe	30 to 34	1.08	1.34	1.08	2.03	1.06	1.10	1.06	1.22
Afghanistan	35 to 39	1.30	1.08	1.51	1.11	1.12	1.06	0.32	0.49
Albania	35 to 39	1.13	1.18	1.09	1.16	1.05	1.07	1.05	1.07
Algeria	35 to 39	0.87	0.95	0.91	0.94	0.93	0.94	0.56	0.81
American Samoa	35 to 39	2.03	1.65	1.49	1.36	1.16	1.04	2.16	1.71
Andorra	35 to 39	0.97	0.98	0.99	0.98	0.81	0.93	1.05	1.03
Angola	35 to 39	1.22	1.23	1.10	1.13	1.03	1.06	1.07	1.13
Antigua and Barbuda	35 to 39	1.82	1.08	1.73	1.03	1.16	1.05	1.42	1.04
Argentina	35 to 39	1.25	1.10	1.15	1.09	0.98	1.01	1.03	1.04
Armenia	35 to 39	1.20	1.16	1.24	1.16	1.22	1.20	1.20	1.22
Australia	35 to 39	1.08	1.01	1.07	1.03	1.05	1.08	1.09	1.02
Austria	35 to 39	1.06	1.02	1.07	1.01	0.98	1.01	1.09	1.04
Azerbaijan	35 to 39	1.39	1.23	1.25	1.19	1.12	1.10	1.34	1.17
Bahamas	35 to 39	1.65	1.53	1.39	1.39	1.11	1.16	1.24	1.30
Bahrain	35 to 39	1.07	1.10	1.07	1.10	1.02	1.04	0.98	1.08
Bangladesh	35 to 39	1.92	1.85	1.21	1.43	1.23	1.18	1.14	1.45
Barbados	35 to 39	1.80	1.17	1.62	1.11	1.33	1.12	1.83	1.09
Belarus	35 to 39	1.64	1.59	1.44	1.56	1.19	1.27	1.64	1.37
Belgium	35 to 39	1.07	0.97	1.06	0.95	0.90	0.94	1.13	1.03
Belize	35 to 39	1.12	1.37	1.01	1.25	1.12	1.13	1.12	1.30
Benin	35 to 39	1.04	1.07	0.96	1.00	1.04	1.04	1.20	1.25
Bermuda	35 to 39	1.22	1.09	1.12	1.04	1.23	1.12	1.28	1.14
Bhutan	35 to 39	1.12	1.12	1.12	1.11	1.02	1.02	0.97	1.08
Bolivia (Plurinational State of)	35 to 39	1.19	1.07	1.15	1.09	0.88	0.89	0.89	1.02
Bosnia and Herzegovina	35 to 39	1.26	1.07	1.34	1.15	1.43	1.08	1.27	1.11
Botswana	35 to 39	1.54	1.22	1.14	1.13	1.12	1.06	1.20	1.15
Brazil	35 to 39	4.29	1.26	2.01	1.30	1.36	0.99	2.04	1.14
Brunei Darussalam	35 to 39	1.02	1.37	1.02	1.34	0.58	1.21	0.85	1.03
Bulgaria	35 to 39	2.06	1.48	1.47	1.40	1.33	1.19	2.25	1.67
Burkina Faso	35 to 39	1.14	1.25	1.12	1.21	1.11	1.15	1.18	1.29
Burundi	35 to 39	1.74	1.26	1.35	1.16	1.11	1.07	0.98	1.03

Cabo Verde	35 to 39	1.39	1.02	1.21	0.99	1.20	1.05	2.35	1.29
Cambodia	35 to 39	1.19	1.27	0.99	1.12	1.02	1.04	0.92	1.03
Cameroon	35 to 39	1.10	1.16	1.06	1.09	1.08	1.11	1.21	1.38
Canada	35 to 39	1.03	1.03	1.06	1.03	1.10	1.09	1.05	1.04
Central African Republic	35 to 39	1.75	1.73	1.33	1.34	1.16	1.17	1.16	1.22
Chad	35 to 39	1.07	1.16	1.02	1.09	1.07	1.11	1.23	1.39
Chile	35 to 39	1.15	1.11	1.17	1.12	1.04	1.07	1.08	1.08
China	35 to 39	1.85	1.70	1.40	1.45	1.17	1.19	1.48	1.58
Colombia	35 to 39	1.19	1.15	1.13	1.12	1.06	1.09	1.32	1.15
Comoros	35 to 39	1.16	1.13	1.03	1.09	0.98	1.02	1.02	1.06
Congo	35 to 39	1.40	1.09	1.15	1.00	1.06	0.95	1.19	1.09
Cook Islands	35 to 39	1.54	1.35	1.27	1.26	1.09	1.07	1.71	1.33
Costa Rica	35 to 39	1.08	1.14	1.08	1.12	1.02	1.18	1.06	1.08
Coted'Ivoire	35 to 39	1.14	1.15	1.09	1.08	1.12	1.11	1.34	1.60
Croatia	35 to 39	1.64	1.18	1.46	1.20	1.32	1.16	1.56	1.12
Cuba	35 to 39	1.28	1.22	1.17	1.25	1.10	1.03	1.36	1.24
Cyprus	35 to 39	0.93	1.03	0.96	0.98	0.78	1.02	0.94	1.04
Czechia	35 to 39	1.32	1.07	1.27	1.07	1.17	1.02	1.46	1.08
Democratic People's Republic of Korea	35 to 39	1.53	2.02	1.29	1.59	1.06	1.09	1.13	1.39
Democratic Republic of the Congo	35 to 39	1.14	1.26	1.03	1.14	1.00	1.07	1.05	1.12
Denmark	35 to 39	1.09	1.03	1.13	1.03	0.91	0.98	1.16	1.06
Djibouti	35 to 39	1.34	1.27	1.16	1.17	1.13	1.12	1.10	1.17
Dominica	35 to 39	1.16	1.24	1.10	1.17	1.09	1.10	1.12	1.23
Dominican Republic	35 to 39	1.54	1.31	1.40	1.25	1.08	0.95	1.58	1.42
Ecuador	35 to 39	1.37	1.14	1.28	1.15	1.06	1.06	1.29	1.09
Egypt	35 to 39	1.66	1.18	2.23	1.28	1.24	1.07	0.38	1.41
El Salvador	35 to 39	1.46	1.48	1.23	1.50	1.15	1.18	1.57	1.52
Equatorial Guinea	35 to 39	1.51	1.10	1.22	1.05	1.13	1.00	1.18	1.12
Eritrea	35 to 39	2.23	1.59	1.33	1.29	1.26	1.16	1.08	1.17
Estonia	35 to 39	1.31	1.17	1.18	1.28	1.40	1.18	1.28	1.14
Eswatini	35 to 39	1.68	2.17	1.46	1.55	1.09	1.16	1.13	1.30
Ethiopia	35 to 39	1.29	1.17	1.14	1.09	1.05	1.06	0.98	1.02
Fiji	35 to 39	1.51	1.18	1.34	1.13	1.09	0.99	1.65	1.34
Finland	35 to 39	1.28	1.06	1.29	1.02	1.05	1.06	1.46	1.05
France	35 to 39	1.32	1.08	1.22	1.03	1.09	1.05	1.30	1.11
Gabon	35 to 39	1.30	1.21	1.15	1.14	1.08	1.06	1.12	1.14
Gambia	35 to 39	1.17	1.16	1.09	1.08	1.12	1.11	1.47	1.60
Georgia	35 to 39	1.54	1.55	1.25	1.31	1.27	1.30	1.29	1.80
Germany	35 to 39	1.11	1.02	1.09	1.03	0.96	0.98	1.24	1.08
Ghana	35 to 39	1.07	1.16	1.03	1.10	1.07	1.13	1.57	1.58
Greece	35 to 39	1.11	1.10	1.05	1.06	0.99	1.07	1.11	1.09
Greenland	35 to 39	1.14	1.03	1.45	1.16	1.01	0.88	1.09	1.03
Grenada	35 to 39	1.90	1.22	1.56	1.24	0.99	0.99	2.59	1.18
Guam	35 to 39	1.28	1.17	1.19	1.05	1.07	0.92	1.53	1.32
Guatemala	35 to 39	1.12	1.20	1.03	1.12	1.03	1.04	1.12	1.14
Guinea	35 to 39	0.96	1.09	0.93	1.02	1.00	1.06	1.21	1.49
Guinea-Bissau	35 to 39	1.09	1.26	1.00	1.15	1.03	1.10	1.49	1.99
Guyana	35 to 39	2.46	2.28	1.53	1.77	1.38	1.53	2.81	2.72

Haiti	35 to 39	0.00	1.50	1.33	1.63	0.00	0.59	0.04	0.97
Honduras	35 to 39	1.31	0.95	1.36	1.04	0.68	0.66	0.86	0.97
Hungary	35 to 39	1.67	1.19	1.46	1.26	1.14	1.05	2.57	1.21
Iceland	35 to 39	0.97	1.01	0.99	0.98	0.86	1.01	1.05	1.04
India	35 to 39	1.30	1.24	1.26	1.17	1.15	1.08	1.07	1.11
Indonesia	35 to 39	1.58	1.65	1.30	1.38	1.11	1.08	1.16	1.38
Iran (Islamic Republic of)	35 to 39	1.09	1.11	1.09	1.12	1.03	1.06	1.08	1.24
Iraq	35 to 39	1.42	1.14	1.30	1.07	1.07	0.99	0.97	0.98
Ireland	35 to 39	1.04	1.00	1.03	0.97	0.93	1.00	1.11	1.04
Israel	35 to 39	1.09	1.04	1.09	1.05	1.02	1.04	1.06	1.04
Italy	35 to 39	1.14	1.06	1.10	1.06	1.03	1.05	1.14	1.03
Jamaica	35 to 39	1.18	1.33	1.07	1.41	1.13	0.98	1.10	1.26
Japan	35 to 39	1.36	1.23	1.24	1.23	1.37	1.15	1.10	1.09
Jordan	35 to 39	1.01	1.10	1.19	1.15	1.01	1.03	0.83	1.16
Kazakhstan	35 to 39	1.27	1.32	1.20	1.29	1.33	1.18	1.44	1.24
Kenya	35 to 39	1.07	1.08	1.02	1.01	1.01	1.00	1.00	1.03
Kiribati	35 to 39	1.81	2.21	1.21	1.36	1.56	1.36	2.34	4.50
Kuwait	35 to 39	0.97	1.09	1.00	1.11	0.95	1.04	0.96	1.13
Kyrgyzstan	35 to 39	1.33	1.87	1.11	1.44	1.18	1.43	1.34	20.60
Lao People's Democratic Republic	35 to 39	6.87	1.78	1.35	1.34	1.05	1.10	0.78	1.44
Latvia	35 to 39	1.39	1.33	1.28	1.49	1.25	1.24	1.53	1.33
Lebanon	35 to 39	1.47	1.08	1.96	1.12	1.27	1.07	0.83	1.04
Lesotho	35 to 39	1.62	4.81	1.48	2.35	1.13	1.25	1.11	1.46
Liberia	35 to 39	1.06	1.07	1.02	1.03	1.05	1.05	1.32	1.43
Libya	35 to 39	0.90	1.03	0.96	1.01	0.92	0.92	0.73	1.15
Lithuania	35 to 39	1.43	1.27	1.37	1.35	1.43	1.28	1.46	1.25
Luxembourg	35 to 39	1.17	1.00	1.11	1.01	0.94	1.02	1.27	1.02
Madagascar	35 to 39	1.72	1.50	1.20	1.21	1.08	1.03	1.20	1.18
Malawi	35 to 39	1.01	1.39	0.97	1.27	0.95	1.09	0.97	1.16
Malaysia	35 to 39	1.28	1.28	1.14	1.18	1.11	1.07	1.15	1.16
Maldives	35 to 39	1.37	1.24	1.06	1.12	1.06	1.07	1.01	1.16
Mali	35 to 39	0.69	0.85	0.71	0.79	0.77	0.86	0.91	1.07
Malta	35 to 39	0.97	1.03	0.93	1.02	0.94	1.02	1.02	1.04
Marshall Islands	35 to 39	1.53	3.56	1.13	1.54	1.11	1.18	1.58	2.99
Mauritania	35 to 39	1.15	1.01	1.08	0.97	1.11	1.04	1.54	1.29
Mauritius	35 to 39	2.68	2.79	1.80	2.03	1.60	1.37	8.50	2.41
Mexico	35 to 39	1.12	1.31	1.14	1.32	1.01	1.24	1.17	1.23
Micronesia (Federated States of)	35 to 39	10.11	3.75	1.60	1.60	1.74	1.24	5.22	4.67
Monaco	35 to 39	1.21	1.11	1.24	1.18	0.97	0.94	1.21	1.12
Mongolia	35 to 39	1.10	1.49	1.06	1.23	0.98	1.35	1.04	1.18
Montenegro	35 to 39	1.68	1.24	1.32	1.24	1.06	1.01	1.17	1.08
Morocco	35 to 39	0.93	0.85	0.96	0.84	0.95	0.89	0.45	0.54
Mozambique	35 to 39	1.42	2.94	1.26	2.07	1.15	1.51	1.12	1.66
Myanmar	35 to 39	1.01	3.18	1.30	1.69	1.17	1.30	1.33	1.66
Namibia	35 to 39	1.39	1.58	1.21	1.42	1.07	1.12	1.15	1.24
Nauru	35 to 39	2.69	2.30	1.32	1.29	0.94	0.82	1.01	7.75
Nepal	35 to 39	1.23	1.29	1.20	1.27	1.09	1.13	0.96	1.15
Netherlands	35 to 39	1.03	1.02	1.04	1.01	0.98	1.02	1.09	1.05

New Zealand	35 to 39	0.95	1.03	0.96	1.00	0.90	1.11	1.08	1.05
Nicaragua	35 to 39	1.17	1.20	1.19	1.31	0.99	1.06	1.14	1.22
Niger	35 to 39	1.02	1.07	1.00	1.05	1.03	1.07	1.08	1.14
Nigeria	35 to 39	1.08	1.13	1.04	1.10	1.08	1.11	1.18	1.36
Niue	35 to 39	1.60	1.33	1.28	1.20	1.15	1.01	1.66	1.44
North Macedonia	35 to 39	1.44	1.16	1.34	1.16	0.93	0.98	1.50	1.15
Northern Mariana Islands	35 to 39	1.40	1.41	1.11	1.18	0.81	1.05	2.07	1.51
Norway	35 to 39	1.11	1.03	1.13	1.04	1.02	1.05	1.16	1.04
Oman	35 to 39	1.07	1.05	1.15	1.18	0.97	0.99	1.01	1.05
Pakistan	35 to 39	1.16	1.19	1.12	1.12	1.09	1.05	1.03	1.12
Palau	35 to 39	3.41	2.58	1.79	1.74	1.37	1.23	11.73	5.97
Palestine	35 to 39	1.19	1.11	1.38	1.22	1.02	1.03	1.18	1.10
Panama	35 to 39	1.20	1.19	1.19	1.26	1.06	1.02	1.17	1.08
Papua New Guinea	35 to 39	1.49	1.92	0.00	0.96	0.98	1.08	1.25	1.37
Paraguay	35 to 39	1.10	1.09	1.07	1.10	0.78	0.83	1.06	1.08
Peru	35 to 39	1.25	1.22	1.17	1.25	1.04	1.04	1.06	1.12
Philippines	35 to 39	6.26	2.16	1.01	1.62	1.19	1.12	1.52	1.40
Poland	35 to 39	1.77	1.52	1.48	1.50	1.27	1.28	2.06	1.45
Portugal	35 to 39	1.32	1.08	1.15	1.09	0.96	0.96	1.59	1.06
Puerto Rico	35 to 39	1.49	1.20	1.36	1.21	1.31	1.18	1.63	1.18
Qatar	35 to 39	1.04	1.05	1.05	1.05	1.10	1.07	1.01	1.06
Republic of Korea	35 to 39	1.48	1.19	1.26	1.17	2.03	1.13	1.16	1.09
Republic of Moldova	35 to 39	1.57	1.34	1.43	1.47	1.25	1.19	1.29	1.18
Romania	35 to 39	1.54	1.49	1.38	1.50	1.06	1.23	1.74	1.43
Russian Federation	35 to 39	1.56	1.81	1.41	1.67	1.33	1.32	2.12	2.01
Rwanda	35 to 39	1.55	1.29	1.25	1.27	1.03	1.09	0.92	1.04
Saint Kitts and Nevis	35 to 39	3.20	1.18	1.78	1.09	1.10	1.12	4.42	1.19
Saint Lucia	35 to 39	2.45	1.33	1.91	1.32	1.08	1.05	2.59	1.27
Saint Vincent and the Grenadines	35 to 39	1.94	1.30	2.04	1.29	1.04	0.89	1.48	1.15
Samoa	35 to 39	1.85	1.58	1.43	1.35	1.22	1.05	1.54	1.59
San Marino	35 to 39	1.11	1.02	1.11	1.00	1.04	1.01	1.17	1.06
Sao Tome and Principe	35 to 39	1.17	0.93	1.14	0.95	1.10	0.97	1.38	1.14
Saudi Arabia	35 to 39	0.92	1.17	0.98	1.30	0.95	0.96	0.27	1.09
Senegal	35 to 39	1.09	1.08	1.00	1.00	1.06	1.08	1.40	1.29
Serbia	35 to 39	1.55	1.22	1.40	1.26	0.96	1.01	1.80	1.25
Seychelles	35 to 39	1.98	1.07	1.42	0.96	1.23	1.00	1.90	1.02
Sierra Leone	35 to 39	1.10	1.05	1.03	0.99	1.07	1.01	1.49	1.57
Singapore	35 to 39	1.15	1.14	1.12	1.14	1.05	1.08	0.97	1.07
Slovakia	35 to 39	1.52	1.17	1.54	1.23	1.15	1.06	1.59	1.15
Slovenia	35 to 39	1.37	1.06	1.28	1.05	1.13	1.09	1.44	1.06
Solomon Islands	35 to 39	2.15	2.10	1.42	1.43	1.44	1.27	1.56	1.60
Somalia	35 to 39	1.62	1.49	1.31	1.36	1.16	1.12	1.02	1.07
South Africa	35 to 39	1.80	1.34	1.40	1.14	0.96	0.99	1.33	1.12
South Sudan	35 to 39	1.28	1.32	1.13	1.23	1.06	1.07	1.04	1.07
Spain	35 to 39	1.17	1.07	1.07	1.05	1.10	1.01	1.18	1.07
Sri Lanka	35 to 39	1.56	1.35	1.44	1.37	1.13	1.08	1.67	1.45
Sudan	35 to 39	0.86	0.95	1.03	0.92	0.96	0.95	0.00	0.67
Suriname	35 to 39	3.55	1.51	2.11	1.34	1.27	1.00	2.47	1.43

Sweden	35 to 39	1.05	1.00	1.06	1.00	0.98	1.02	1.07	1.00
Switzerland	35 to 39	1.03	1.01	1.03	0.97	0.89	0.99	1.10	1.04
Syrian Arab Republic	35 to 39	1.13	1.11	1.17	1.16	0.99	0.98	0.73	0.95
Taiwan (Province of China)	35 to 39	1.43	1.34	1.28	1.29	1.07	1.11	1.24	1.13
Tajikistan	35 to 39	1.16	1.24	1.06	1.20	0.94	1.08	1.82	1.33
Thailand	35 to 39	1.47	3.64	1.18	1.93	1.38	1.93	1.17	1.80
Timor-Leste	35 to 39	1.96	2.04	1.48	1.54	1.23	1.20	1.21	1.56
Togo	35 to 39	0.99	1.13	0.93	1.07	1.01	1.09	1.27	1.44
Tokelau	35 to 39	2.42	1.56	1.59	1.38	1.16	0.99	2.48	1.83
Tonga	35 to 39	1.19	1.20	1.13	1.13	1.02	1.01	1.37	1.32
Trinidad and Tobago	35 to 39	1.38	1.63	1.32	1.79	1.32	1.44	2.01	1.70
Tunisia	35 to 39	0.98	1.07	0.99	1.07	0.98	1.01	0.74	1.09
Turkey	35 to 39	1.42	1.12	1.25	1.12	1.44	1.14	1.27	1.08
Turkmenistan	35 to 39	1.35	1.68	1.21	1.48	1.18	1.33	1.44	1.61
Tuvalu	35 to 39	13.54	2.20	1.75	1.40	1.39	1.07	6.53	3.52
Uganda	35 to 39	1.43	1.34	1.26	1.23	1.16	1.13	1.05	1.07
Ukraine	35 to 39	1.34	1.54	1.33	1.46	1.18	1.32	1.49	1.66
United Arab Emirates	35 to 39	1.08	1.02	1.11	1.02	0.99	0.97	1.12	1.05
United Kingdom	35 to 39	1.08	1.06	1.05	1.05	1.02	1.04	1.13	1.06
United Republic of Tanzania	35 to 39	1.27	1.15	1.11	1.11	1.09	1.04	1.03	1.09
United States Virgin Islands	35 to 39	1.80	1.86	1.58	2.09	1.25	1.44	1.63	1.64
United States of America	35 to 39	1.05	1.03	1.12	1.08	1.14	1.12	1.02	0.98
Uruguay	35 to 39	1.14	1.06	1.17	1.09	0.91	1.00	1.04	1.04
Uzbekistan	35 to 39	1.37	1.31	1.31	1.21	1.12	1.18	1.42	1.58
Vanuatu	35 to 39	3.88	2.37	1.86	1.53	1.56	1.20	3.35	2.53
Venezuela (Bolivarian Republic of)	35 to 39	1.33	1.52	1.28	1.57	1.03	1.15	1.15	1.12
Viet Nam	35 to 39	2.60	1.95	1.54	1.58	1.36	1.24	1.59	1.62
Yemen	35 to 39	1.33	1.22	1.47	1.21	1.15	1.09	0.30	1.18
Zambia	35 to 39	1.05	1.31	0.97	1.12	0.91	1.02	0.95	1.08
Zimbabwe	35 to 39	1.17	1.72	1.11	1.79	1.04	1.09	1.09	1.29
Afghanistan	40 to 44	1.39	0.90	2.26	1.36	1.19	1.01	0.55	0.73
Albania	40 to 44	1.06	1.16	1.12	1.23	0.98	1.03	1.07	1.10
Algeria	40 to 44	0.84	0.89	1.01	0.96	0.90	0.90	0.75	0.84
American Samoa	40 to 44	1.62	1.30	1.63	1.45	1.08	0.99	1.03	0.96
Andorra	40 to 44	0.98	0.97	1.05	0.98	0.79	0.89	1.08	1.02
Angola	40 to 44	1.32	1.26	1.43	1.38	1.00	1.01	1.12	1.15
Antigua and Barbuda	40 to 44	1.72	1.05	2.23	1.11	0.98	0.97	1.50	1.03
Argentina	40 to 44	1.27	1.09	1.33	1.15	1.03	0.98	1.09	1.03
Armenia	40 to 44	1.21	1.30	1.40	1.45	1.19	1.25	1.33	1.57
Australia	40 to 44	1.08	1.01	1.15	1.11	1.05	1.01	1.09	1.02
Austria	40 to 44	1.09	1.00	1.17	1.00	0.95	0.94	1.30	1.03
Azerbaijan	40 to 44	1.43	1.20	1.45	1.22	1.09	1.08	1.73	1.22
Bahamas	40 to 44	1.63	1.45	1.96	1.58	0.94	1.11	1.39	1.36
Bahrain	40 to 44	1.07	1.02	1.16	1.06	1.00	0.99	1.07	1.02
Bangladesh	40 to 44	1.98	1.31	1.79	1.38	1.12	0.98	1.28	1.26
Barbados	40 to 44	1.27	1.18	1.43	1.30	1.02	1.05	1.32	1.14
Belarus	40 to 44	1.44	1.52	1.27	1.46	1.39	1.56	3.02	1.84
Belgium	40 to 44	1.07	1.01	1.17	1.03	0.91	0.92	1.18	1.06

Belize	40 to 44	0.97	1.31	1.05	1.48	1.00	1.02	1.05	1.27
Benin	40 to 44	1.06	1.11	1.12	1.16	1.01	1.02	1.31	1.38
Bermuda	40 to 44	1.34	1.14	1.44	1.19	1.15	1.12	1.74	1.15
Bhutan	40 to 44	0.96	1.03	1.17	1.16	0.87	0.93	0.92	1.04
Bolivia (Plurinational State of)	40 to 44	1.17	1.00	1.45	1.21	0.83	0.82	1.31	1.10
Bosnia and Herzegovina	40 to 44	1.13	1.09	1.36	1.23	1.00	1.03	1.33	1.18
Botswana	40 to 44	1.41	1.21	1.29	1.23	1.06	1.03	1.24	1.18
Brazil	40 to 44	1.98	1.13	2.19	1.46	1.11	0.85	2.31	1.20
Brunei Darussalam	40 to 44	0.94	1.11	1.10	1.27	0.60	0.89	0.98	1.04
Bulgaria	40 to 44	1.76	1.39	1.61	1.49	1.14	1.05	2.89	2.33
Burkina Faso	40 to 44	1.18	1.33	1.36	1.47	1.08	1.12	1.27	1.37
Burundi	40 to 44	2.39	1.39	2.16	1.64	1.14	1.05	1.07	1.06
Cabo Verde	40 to 44	1.31	1.16	1.32	1.19	1.14	1.06	2.50	1.63
Cambodia	40 to 44	1.10	1.19	1.28	1.25	0.98	1.00	1.15	1.12
Cameroon	40 to 44	1.19	1.25	1.36	1.36	1.05	1.07	1.38	1.61
Canada	40 to 44	1.02	1.01	1.06	1.04	1.05	1.04	1.04	1.01
Central African Republic	40 to 44	2.22	2.21	1.93	2.20	1.11	1.11	1.26	1.29
Chad	40 to 44	1.12	1.25	1.22	1.35	1.03	1.07	1.38	1.66
Chile	40 to 44	1.13	1.11	1.28	1.18	0.97	1.06	1.10	1.06
China	40 to 44	1.92	1.57	1.64	1.54	1.01	1.12	7.05	2.14
Colombia	40 to 44	1.13	1.13	1.29	1.24	0.98	1.02	1.26	1.15
Comoros	40 to 44	1.24	1.17	1.35	1.34	0.97	1.00	1.06	1.07
Congo	40 to 44	1.45	1.06	1.46	1.20	0.98	0.89	1.27	1.10
Cook Islands	40 to 44	1.31	1.23	1.41	1.35	1.05	1.04	0.96	0.99
Costa Rica	40 to 44	1.07	1.12	1.15	1.23	0.99	1.05	1.07	1.06
Coted'Ivoire	40 to 44	1.22	1.23	1.30	1.32	1.09	1.07	1.62	2.01
Croatia	40 to 44	1.47	1.21	1.49	1.29	1.09	1.04	1.65	1.17
Cuba	40 to 44	1.17	1.28	1.37	1.53	0.94	1.02	1.27	1.33
Cyprus	40 to 44	0.99	1.03	1.07	1.02	0.85	0.98	1.04	1.04
Czechia	40 to 44	1.38	1.12	1.52	1.18	1.07	1.01	1.94	1.12
Democratic People's Republic of Korea	40 to 44	1.39	1.78	1.40	1.83	0.98	1.01	1.61	2.35
Democratic Republic of the Congo	40 to 44	1.20	1.30	1.24	1.41	0.99	1.01	1.10	1.13
Denmark	40 to 44	1.12	1.03	1.29	1.06	0.92	0.95	1.31	1.04
Djibouti	40 to 44	1.35	1.28	1.40	1.40	1.07	1.07	1.10	1.17
Dominica	40 to 44	1.20	1.29	1.33	1.55	1.05	1.05	1.18	1.35
Dominican Republic	40 to 44	1.31	1.33	1.59	1.54	0.96	0.94	1.49	1.49
Ecuador	40 to 44	1.29	1.13	1.53	1.23	0.96	1.02	1.83	1.19
Egypt	40 to 44	1.37	1.12	2.22	1.37	1.19	1.03	1.38	1.63
El Salvador	40 to 44	1.27	1.40	1.30	1.74	1.02	1.07	1.58	1.55
Equatorial Guinea	40 to 44	1.87	1.13	1.82	1.30	1.12	0.96	1.29	1.14
Eritrea	40 to 44	4.03	1.67	2.55	1.76	1.24	1.12	1.11	1.16
Estonia	40 to 44	1.29	1.21	1.35	1.46	1.30	1.25	1.52	1.16
Eswatini	40 to 44	1.86	2.24	1.99	2.11	1.07	1.09	1.19	1.39
Ethiopia	40 to 44	1.64	1.21	1.62	1.33	1.08	1.03	1.01	1.01
Fiji	40 to 44	1.26	1.08	1.52	1.31	1.08	1.00	0.98	0.95
Finland	40 to 44	1.20	1.05	1.33	1.07	1.05	1.01	1.48	1.05
France	40 to 44	1.26	1.05	1.34	1.08	1.02	0.98	1.33	1.06
Gabon	40 to 44	1.40	1.24	1.42	1.33	1.05	1.02	1.15	1.16

Gambia	40 to 44	1.18	1.20	1.30	1.36	1.05	1.04	1.72	2.07
Georgia	40 to 44	1.63	1.61	1.40	1.43	1.25	1.25	1.55	2.52
Germany	40 to 44	1.07	1.06	1.14	1.10	0.90	0.96	1.26	1.10
Ghana	40 to 44	1.20	1.30	1.40	1.42	1.04	1.10	2.12	2.27
Greece	40 to 44	1.09	1.08	1.14	1.13	0.93	0.97	1.20	1.06
Greenland	40 to 44	1.06	1.05	1.54	1.29	1.01	0.97	1.19	1.06
Grenada	40 to 44	1.45	1.12	1.61	1.25	1.01	0.96	2.17	1.15
Guam	40 to 44	1.24	1.32	1.36	1.51	1.07	1.12	1.03	1.04
Guatemala	40 to 44	0.99	1.17	1.07	1.25	0.96	1.01	1.18	1.15
Guinea	40 to 44	0.93	1.13	1.06	1.20	0.93	1.00	1.32	1.78
Guinea-Bissau	40 to 44	1.44	1.46	1.60	1.71	1.04	1.02	2.71	3.65
Guyana	40 to 44	2.07	1.62	1.90	1.89	1.16	1.21	4.83	2.39
Haiti	40 to 44	1.01	1.62	3.12	3.30	0.26	0.65	0.46	1.37
Honduras	40 to 44	0.95	0.80	2.13	1.38	0.63	0.52	1.34	1.03
Hungary	40 to 44	1.42	1.24	1.49	1.34	0.94	1.01	3.48	1.43
Iceland	40 to 44	0.97	1.02	1.01	1.02	0.83	0.98	1.06	1.03
India	40 to 44	1.18	1.21	1.36	1.31	1.04	1.03	1.06	1.11
Indonesia	40 to 44	1.39	1.42	1.52	1.55	1.03	0.99	1.33	1.53
Iran (Islamic Republic of)	40 to 44	1.06	1.08	1.19	1.15	1.01	1.04	1.53	1.33
Iraq	40 to 44	1.34	1.13	1.47	1.19	1.07	0.97	1.85	1.39
Ireland	40 to 44	1.04	1.00	1.10	0.99	0.93	0.95	1.17	1.02
Israel	40 to 44	1.02	1.03	1.09	1.10	0.92	1.00	1.03	1.02
Italy	40 to 44	1.11	1.08	1.18	1.15	0.99	0.98	1.15	1.04
Jamaica	40 to 44	1.15	1.25	1.33	1.56	0.97	0.94	1.25	1.23
Japan	40 to 44	1.22	1.16	1.30	1.22	1.03	1.05	1.11	1.07
Jordan	40 to 44	0.91	1.07	1.14	1.15	0.97	1.00	0.92	1.11
Kazakhstan	40 to 44	1.27	1.34	1.33	1.49	1.12	1.09	1.92	1.40
Kenya	40 to 44	1.09	1.14	1.18	1.23	0.99	0.99	1.00	1.03
Kiribati	40 to 44	1.64	1.74	1.35	1.48	1.49	1.26	1.10	1.16
Kuwait	40 to 44	0.98	1.12	1.07	1.20	0.94	1.02	1.00	1.20
Kyrgyzstan	40 to 44	1.45	1.64	1.37	1.52	1.12	1.22	2.31	1.01
Lao People's Democratic Republic	40 to 44	4.52	1.56	2.04	1.57	0.85	1.03	1.90	1.90
Latvia	40 to 44	1.37	1.36	1.48	1.65	1.16	1.21	2.06	1.55
Lebanon	40 to 44	1.31	1.07	2.74	1.16	1.21	1.05	0.98	1.06
Lesotho	40 to 44	1.83	8.72	1.91	4.92	1.15	1.18	1.14	1.61
Liberia	40 to 44	1.06	1.12	1.17	1.32	0.99	0.99	1.44	1.70
Libya	40 to 44	0.82	0.92	0.95	0.98	0.89	0.86	0.83	1.15
Lithuania	40 to 44	1.31	1.31	1.43	1.44	1.18	1.29	1.76	1.45
Luxembourg	40 to 44	1.05	1.01	1.12	1.03	0.83	0.95	1.29	1.02
Madagascar	40 to 44	1.69	1.37	1.57	1.52	1.06	0.95	1.21	1.15
Malawi	40 to 44	1.17	1.68	1.40	2.49	0.97	1.09	1.04	1.26
Malaysia	40 to 44	1.18	1.23	1.21	1.26	1.05	1.03	1.20	1.23
Maldives	40 to 44	0.94	1.11	1.11	1.09	0.93	1.01	1.05	1.13
Mali	40 to 44	0.71	0.84	0.89	0.92	0.76	0.82	0.98	1.12
Malta	40 to 44	0.95	0.99	0.96	1.02	0.90	0.95	1.04	0.99
Marshall Islands	40 to 44	2.29	1.96	1.83	1.74	1.33	1.15	0.96	0.97
Mauritania	40 to 44	1.08	0.98	1.16	1.05	0.99	0.96	1.69	1.33
Mauritius	40 to 44	2.25	1.42	1.99	1.40	1.48	1.08	398.96	1.72

Mexico	40 to 44	1.11	1.23	1.23	1.38	0.98	1.11	1.26	1.20
Micronesia (Federated States of)	40 to 44	2.92	2.04	1.86	1.79	1.49	1.18	0.94	0.99
Monaco	40 to 44	1.16	1.11	1.37	1.27	0.89	0.93	1.29	1.16
Mongolia	40 to 44	1.10	1.55	1.23	1.39	0.86	1.23	1.10	1.29
Montenegro	40 to 44	1.31	1.28	1.29	1.45	0.97	1.00	1.17	1.12
Morocco	40 to 44	0.83	0.79	1.08	0.89	0.90	0.83	0.73	0.61
Mozambique	40 to 44	1.59	4.17	1.75	4.63	1.11	1.40	1.18	1.88
Myanmar	40 to 44	36.77	2.48	2.11	1.94	1.11	1.18	3.29	2.07
Namibia	40 to 44	1.34	1.60	1.37	1.74	1.02	1.08	1.19	1.28
Nauru	40 to 44	1.74	1.66	1.71	1.81	1.05	0.97	0.93	0.91
Nepal	40 to 44	0.96	1.15	1.22	1.35	0.91	1.00	0.87	1.09
Netherlands	40 to 44	1.03	1.01	1.09	0.99	0.92	0.97	1.10	1.04
New Zealand	40 to 44	0.95	1.04	1.08	1.10	0.88	1.02	1.09	1.05
Nicaragua	40 to 44	1.15	1.12	1.37	1.31	0.96	0.98	1.22	1.15
Niger	40 to 44	1.04	1.08	1.15	1.20	0.97	1.02	1.10	1.17
Nigeria	40 to 44	1.13	1.17	1.24	1.26	1.06	1.09	1.31	1.51
Niue	40 to 44	1.35	1.28	1.42	1.44	1.11	1.06	0.96	0.97
North Macedonia	40 to 44	1.26	1.17	1.38	1.26	0.85	0.93	1.60	1.27
Northern Mariana Islands	40 to 44	0.98	1.16	1.10	1.17	0.76	0.96	0.91	0.97
Norway	40 to 44	1.07	1.03	1.20	1.04	0.87	0.98	1.17	1.04
Oman	40 to 44	1.05	1.06	1.22	1.19	0.95	0.97	1.07	1.08
Pakistan	40 to 44	1.05	1.11	1.16	1.20	0.98	0.98	1.00	1.10
Palau	40 to 44	2.17	1.78	1.97	1.85	1.26	1.17	1.11	1.13
Palestine	40 to 44	1.09	1.13	1.41	1.35	0.99	1.01	1.44	1.34
Panama	40 to 44	1.15	1.17	1.28	1.36	0.97	0.95	1.31	1.08
Papua New Guinea	40 to 44	0.09	1.15	0.00	1.29	0.91	1.01	0.70	0.83
Paraguay	40 to 44	0.86	1.04	1.22	1.30	0.61	0.75	1.08	1.11
Peru	40 to 44	1.11	1.09	1.22	1.23	0.94	0.94	1.15	1.12
Philippines	40 to 44	4.02	1.85	1.01	1.81	1.12	1.04	1.84	1.57
Poland	40 to 44	1.51	1.48	1.53	1.59	1.10	1.16	2.89	1.67
Portugal	40 to 44	1.11	1.02	1.19	1.09	0.83	0.86	1.45	1.03
Puerto Rico	40 to 44	1.36	1.17	1.42	1.30	1.14	1.10	1.55	1.15
Qatar	40 to 44	1.06	1.06	1.13	1.08	1.11	1.07	1.04	1.06
Republic of Korea	40 to 44	1.29	1.14	1.36	1.19	1.19	1.02	1.19	1.07
Republic of Moldova	40 to 44	1.33	1.44	1.41	1.78	1.16	1.21	1.32	1.37
Romania	40 to 44	1.33	1.45	1.39	1.69	0.95	1.07	1.77	1.40
Russian Federation	40 to 44	1.44	1.66	1.49	1.85	1.12	1.15	4.10	3.31
Rwanda	40 to 44	2.30	1.36	2.11	1.64	1.11	1.07	1.03	1.04
Saint Kitts and Nevis	40 to 44	1.82	1.30	2.01	1.41	0.92	1.02	3.12	1.42
Saint Lucia	40 to 44	1.51	1.34	1.76	1.61	0.85	0.96	2.12	1.32
Saint Vincent and the Grenadines	40 to 44	1.39	1.31	1.84	1.57	0.97	0.92	1.25	1.28
Samoa	40 to 44	1.61	1.30	1.64	1.47	1.19	1.02	0.97	0.98
San Marino	40 to 44	1.10	1.03	1.17	1.02	0.99	0.98	1.19	1.05
Sao Tome and Principe	40 to 44	0.99	0.96	1.07	1.07	0.96	0.94	1.19	1.24
Saudi Arabia	40 to 44	0.83	1.04	1.22	1.44	0.92	0.93	0.59	1.56
Senegal	40 to 44	1.13	1.10	1.24	1.16	1.01	1.02	1.67	1.44
Serbia	40 to 44	1.34	1.24	1.49	1.39	0.82	0.96	2.07	1.41
Seychelles	40 to 44	1.75	1.56	1.58	1.61	1.15	1.12	2.47	1.82

Sierra Leone	40 to 44	1.16	1.08	1.26	1.20	1.03	0.96	1.93	1.92
Singapore	40 to 44	1.07	1.08	1.16	1.11	0.93	0.98	0.98	1.04
Slovakia	40 to 44	1.56	1.23	1.96	1.37	1.06	1.03	2.22	1.25
Slovenia	40 to 44	1.32	1.13	1.32	1.15	1.02	1.09	1.62	1.11
Solomon Islands	40 to 44	1.83	1.77	1.52	1.54	1.34	1.21	0.99	1.00
Somalia	40 to 44	2.18	1.76	2.08	2.09	1.20	1.12	1.05	1.08
South Africa	40 to 44	1.36	1.26	1.40	1.35	0.96	0.96	1.30	1.16
South Sudan	40 to 44	1.48	1.51	1.60	1.97	1.06	1.06	1.08	1.10
Spain	40 to 44	1.17	1.06	1.17	1.10	1.03	0.98	1.28	1.06
Sri Lanka	40 to 44	1.49	1.29	1.65	1.46	1.11	1.07	1.81	1.40
Sudan	40 to 44	0.66	0.84	1.22	0.99	0.87	0.88	0.23	0.87
Suriname	40 to 44	1.70	1.19	1.82	1.37	1.07	0.91	1.77	1.28
Sweden	40 to 44	1.05	1.00	1.13	1.00	0.90	0.98	1.17	1.00
Switzerland	40 to 44	1.04	1.05	1.08	1.00	0.91	1.02	1.16	1.05
Syrian Arab Republic	40 to 44	1.00	1.06	1.19	1.21	0.94	0.95	1.03	1.13
Taiwan (Province of China)	40 to 44	1.38	1.32	1.36	1.34	1.02	1.06	1.31	1.20
Tajikistan	40 to 44	1.08	1.18	1.23	1.28	0.84	1.01	3.58	1.40
Thailand	40 to 44	1.31	2.00	1.22	1.72	1.25	1.42	1.19	1.65
Timor-Leste	40 to 44	1.52	1.80	1.69	1.85	1.07	1.09	1.33	1.95
Togo	40 to 44	0.99	1.25	1.11	1.37	0.95	1.06	1.51	1.87
Tokelau	40 to 44	1.82	1.33	1.94	1.62	1.14	1.02	0.94	0.96
Tonga	40 to 44	1.17	1.10	1.37	1.24	1.02	0.99	0.98	0.97
Trinidad and Tobago	40 to 44	1.12	1.18	1.32	1.46	1.05	1.01	1.60	1.44
Tunisia	40 to 44	0.93	1.02	1.08	1.10	0.95	0.97	0.89	1.15
Turkey	40 to 44	1.21	1.10	1.24	1.15	1.18	1.07	1.29	1.09
Turkmenistan	40 to 44	1.43	1.70	1.48	1.81	1.09	1.27	2.97	2.07
Tuvalu	40 to 44	4.09	1.57	2.29	1.62	1.36	1.07	0.95	0.97
Uganda	40 to 44	1.65	1.44	1.81	1.56	1.15	1.12	1.08	1.09
Ukraine	40 to 44	1.26	1.42	1.43	1.56	1.09	1.16	1.65	1.83
United Arab Emirates	40 to 44	1.07	1.02	1.18	1.04	0.95	0.94	1.15	1.02
United Kingdom	40 to 44	1.04	1.05	1.07	1.09	0.94	0.98	1.16	1.07
United Republic of Tanzania	40 to 44	1.40	1.16	1.48	1.30	1.08	1.03	1.06	1.10
United States Virgin Islands	40 to 44	1.39	1.14	1.56	1.23	1.06	0.97	1.40	1.10
United States of America	40 to 44	1.03	1.01	1.16	1.10	1.03	1.08	1.03	1.00
Uruguay	40 to 44	1.20	1.04	1.35	1.18	1.14	0.93	1.16	1.04
Uzbekistan	40 to 44	1.39	1.24	1.74	1.24	1.05	1.16	2.16	1.57
Vanuatu	40 to 44	2.39	1.67	2.11	1.65	1.34	1.14	1.07	1.05
Venezuela (Bolivarian Republic of)	40 to 44	1.20	1.39	1.35	1.70	0.92	1.01	1.24	1.12
Viet Nam	40 to 44	2.33	2.06	1.79	1.95	1.28	1.20	2.04	2.04
Yemen	40 to 44	0.81	0.92	1.75	1.21	1.01	0.97	0.00	1.36
Zambia	40 to 44	1.35	1.51	1.54	1.64	0.95	1.01	1.01	1.13
Zimbabwe	40 to 44	1.12	1.44	1.16	2.01	0.97	0.95	1.07	1.26
Afghanistan	45 to 49	1.73	1.01	2.78	1.56	1.88	1.31	0.51	0.52
Albania	45 to 49	1.10	1.13	1.18	1.21	1.07	1.05	1.07	1.09
Algeria	45 to 49	0.86	0.90	0.99	0.94	1.01	0.98	0.65	0.80
American Samoa	45 to 49	1.36	1.19	1.35	1.26	1.22	1.15	1.07	0.99
Andorra	45 to 49	0.98	0.96	1.06	0.97	0.91	0.94	1.02	0.99
Angola	45 to 49	1.37	1.24	1.50	1.37	1.16	1.12	1.06	1.08

Antigua and Barbuda	45 to 49	1.57	1.10	1.74	1.26	1.32	1.05	1.18	1.00
Argentina	45 to 49	1.25	1.08	1.34	1.17	1.44	1.11	1.08	1.00
Armenia	45 to 49	1.13	1.24	1.24	1.39	1.26	1.32	1.11	1.37
Australia	45 to 49	1.03	0.99	1.12	1.07	1.18	1.14	1.02	0.98
Austria	45 to 49	1.09	0.98	1.17	1.00	1.04	0.99	1.22	0.99
Azerbaijan	45 to 49	1.30	1.15	1.31	1.17	1.14	1.10	1.36	1.14
Bahamas	45 to 49	1.37	1.28	1.57	1.46	1.18	1.24	1.11	1.07
Bahrain	45 to 49	1.03	0.99	1.18	1.02	1.05	1.03	0.97	1.00
Bangladesh	45 to 49	1.11	1.21	1.25	1.33	1.24	1.11	0.93	1.13
Barbados	45 to 49	1.42	1.23	1.75	1.45	1.27	1.33	1.19	1.07
Belarus	45 to 49	1.26	1.39	1.26	1.56	1.51	1.75	1.47	1.41
Belgium	45 to 49	1.10	1.01	1.18	1.06	1.08	1.06	1.10	1.01
Belize	45 to 49	1.05	1.46	1.21	1.85	1.11	1.22	0.99	1.22
Benin	45 to 49	1.12	1.17	1.12	1.18	1.15	1.12	1.39	1.42
Bermuda	45 to 49	1.38	1.16	1.56	1.30	1.38	1.24	1.40	1.10
Bhutan	45 to 49	0.92	0.97	1.14	1.08	1.01	1.00	0.81	0.95
Bolivia (Plurinational State of)	45 to 49	1.21	1.01	1.58	1.34	1.06	0.95	1.10	1.03
Bosnia and Herzegovina	45 to 49	1.11	1.06	1.42	1.22	1.19	1.04	1.14	1.10
Botswana	45 to 49	1.29	1.16	1.22	1.19	1.17	1.09	1.15	1.10
Brazil	45 to 49	1.86	1.17	2.16	1.57	1.61	1.10	3.14	1.22
Brunei Darussalam	45 to 49	0.95	1.07	1.08	1.27	0.97	1.16	0.90	0.97
Bulgaria	45 to 49	1.60	1.42	1.54	1.60	1.30	1.20	1.86	2.11
Burkina Faso	45 to 49	1.50	1.57	1.66	1.69	1.33	1.31	1.42	1.52
Burundi	45 to 49	2.39	1.36	2.39	1.71	1.47	1.19	1.01	1.01
Cabo Verde	45 to 49	1.35	1.29	1.29	1.26	1.28	1.19	2.19	1.89
Cambodia	45 to 49	1.41	1.29	1.46	1.34	1.33	1.16	1.09	1.06
Cameroon	45 to 49	1.36	1.39	1.45	1.43	1.27	1.27	1.45	1.67
Canada	45 to 49	1.01	0.98	1.08	1.00	1.17	1.13	1.00	0.97
Central African Republic	45 to 49	2.23	2.21	1.99	2.54	1.36	1.32	1.16	1.18
Chad	45 to 49	1.18	1.34	1.22	1.37	1.19	1.21	1.41	1.67
Chile	45 to 49	1.15	1.08	1.33	1.16	1.22	1.16	1.03	1.04
China	45 to 49	1.72	1.33	1.55	1.37	1.53	1.21	1.99	1.32
Colombia	45 to 49	1.06	1.07	1.21	1.23	1.07	1.08	1.11	1.10
Comoros	45 to 49	1.26	1.16	1.39	1.34	1.11	1.09	1.01	1.02
Congo	45 to 49	1.35	1.07	1.38	1.19	1.11	0.99	1.13	1.03
Cook Islands	45 to 49	1.25	1.12	1.29	1.22	1.27	1.10	1.02	0.98
Costa Rica	45 to 49	1.04	1.09	1.11	1.23	1.06	1.14	1.06	1.05
Coted'Ivoire	45 to 49	1.38	1.38	1.41	1.42	1.31	1.25	1.71	2.10
Croatia	45 to 49	1.43	1.21	1.57	1.43	1.28	1.13	1.37	1.12
Cuba	45 to 49	1.25	1.27	1.48	1.63	1.18	1.07	1.18	1.17
Cyprus	45 to 49	0.95	1.05	1.05	1.08	0.92	1.07	0.93	1.06
Czechia	45 to 49	1.38	1.13	1.56	1.26	1.27	1.02	1.70	1.10
Democratic People's Republic of Korea	45 to 49	1.37	1.73	1.40	1.92	1.22	1.22	1.19	1.45
Democratic Republic of the Congo	45 to 49	1.17	1.25	1.21	1.39	1.09	1.10	1.02	1.05
Denmark	45 to 49	1.11	1.03	1.26	1.09	1.14	1.02	1.15	1.01
Djibouti	45 to 49	1.30	1.25	1.39	1.42	1.19	1.16	1.04	1.11
Dominica	45 to 49	1.39	1.38	1.70	2.00	1.28	1.24	1.10	1.18
Dominican Republic	45 to 49	1.34	1.36	1.66	1.62	1.18	1.12	1.14	1.20

Ecuador	45 to 49	1.16	1.05	1.43	1.22	1.07	1.05	1.03	1.06
Egypt	45 to 49	1.33	1.14	2.32	1.39	1.47	1.14	0.88	1.26
El Salvador	45 to 49	1.15	1.31	1.20	1.70	1.13	1.17	1.25	1.32
Equatorial Guinea	45 to 49	1.77	1.12	1.77	1.27	1.31	1.03	1.18	1.10
Eritrea	45 to 49	3.59	1.58	2.94	1.85	1.60	1.25	1.05	1.08
Estonia	45 to 49	1.29	1.20	1.37	1.59	1.67	1.38	1.41	1.15
Eswatini	45 to 49	1.93	2.13	2.14	2.28	1.24	1.21	1.19	1.32
Ethiopia	45 to 49	1.72	1.17	1.68	1.30	1.24	1.10	0.97	0.96
Fiji	45 to 49	1.29	1.09	1.52	1.31	1.34	1.17	1.04	0.98
Finland	45 to 49	1.13	1.01	1.22	1.03	1.24	1.08	1.20	1.01
France	45 to 49	1.18	1.02	1.35	1.10	1.11	1.07	1.17	1.02
Gabon	45 to 49	1.33	1.21	1.38	1.31	1.15	1.09	1.06	1.07
Gambia	45 to 49	1.36	1.38	1.40	1.50	1.26	1.20	1.91	2.15
Georgia	45 to 49	1.44	1.49	1.30	1.38	1.27	1.38	1.37	1.82
Germany	45 to 49	1.09	1.03	1.24	1.08	1.02	1.01	1.14	1.03
Ghana	45 to 49	1.38	1.45	1.43	1.49	1.32	1.28	2.18	2.32
Greece	45 to 49	1.06	1.11	1.08	1.21	1.10	1.12	1.04	1.03
Greenland	45 to 49	1.00	1.04	1.49	1.33	1.01	1.14	1.06	1.02
Grenada	45 to 49	1.13	1.25	1.31	1.54	1.12	1.18	0.92	1.12
Guam	45 to 49	1.09	1.17	1.15	1.37	1.14	1.18	1.01	1.03
Guatemala	45 to 49	1.02	1.07	1.08	1.16	1.03	1.06	1.06	1.07
Guinea	45 to 49	1.02	1.22	1.09	1.23	1.08	1.13	1.36	1.74
Guinea-Bissau	45 to 49	2.49	1.97	2.07	2.01	1.81	1.42	4.31	4.63
Guyana	45 to 49	10.67	1.57	4.83	2.27	2.05	1.52	3.20	1.27
Haiti	45 to 49	6.57	1.75	3.71	3.88	1.76	1.19	0.42	0.88
Honduras	45 to 49	1.40	1.03	2.74	3.04	1.23	0.94	1.37	1.13
Hungary	45 to 49	1.36	1.24	1.42	1.42	1.31	1.16	1.88	1.28
Iceland	45 to 49	0.98	1.00	1.03	1.03	0.91	1.06	1.01	0.99
India	45 to 49	1.13	1.07	1.34	1.17	1.23	1.10	0.95	0.99
Indonesia	45 to 49	1.30	1.36	1.43	1.52	1.21	1.14	1.13	1.28
Iran (Islamic Republic of)	45 to 49	1.09	1.05	1.22	1.13	1.14	1.08	1.16	1.11
Iraq	45 to 49	1.76	1.21	1.98	1.28	1.35	1.09	1.01	1.33
Ireland	45 to 49	1.01	1.00	1.06	0.99	1.09	1.08	1.03	1.00
Israel	45 to 49	1.05	1.03	1.22	1.18	1.04	1.06	0.98	0.99
Italy	45 to 49	1.09	1.07	1.21	1.17	1.13	1.03	1.07	1.04
Jamaica	45 to 49	1.12	1.22	1.31	1.49	1.06	1.05	0.95	1.07
Japan	45 to 49	1.14	1.10	1.28	1.18	1.23	1.16	1.02	1.02
Jordan	45 to 49	0.95	1.02	1.14	1.09	1.00	1.00	0.87	0.97
Kazakhstan	45 to 49	1.22	1.30	1.27	1.48	1.22	1.23	1.52	1.28
Kenya	45 to 49	1.06	1.11	1.14	1.20	1.06	1.05	0.95	0.99
Kiribati	45 to 49	1.30	1.41	1.16	1.27	1.78	1.44	1.14	1.25
Kuwait	45 to 49	0.98	1.11	1.07	1.17	0.96	1.04	0.94	1.16
Kyrgyzstan	45 to 49	1.32	1.45	1.21	1.37	1.49	1.21	1.87	4.40
Lao People's Democratic Republic	45 to 49	2.64	1.42	2.10	1.49	1.44	1.20	1.14	1.35
Latvia	45 to 49	1.32	1.29	1.34	1.60	1.44	1.35	1.87	1.37
Lebanon	45 to 49	1.42	1.06	3.92	1.21	1.90	1.14	1.05	1.04
Lesotho	45 to 49	1.97	1.01	2.03	1.01	1.32	1.49	1.15	1.67
Liberia	45 to 49	1.12	1.21	1.17	1.32	1.11	1.13	1.50	1.77

Libya	45 to 49	0.84	0.95	0.94	0.99	0.96	0.94	0.81	1.03
Lithuania	45 to 49	1.23	1.26	1.30	1.49	1.51	1.35	1.39	1.39
Luxembourg	45 to 49	1.19	1.00	1.37	1.03	1.05	1.02	1.25	1.01
Madagascar	45 to 49	1.66	1.33	1.66	1.55	1.30	1.12	1.14	1.06
Malawi	45 to 49	1.22	1.61	1.45	2.91	1.16	1.23	1.00	1.17
Malaysia	45 to 49	1.15	1.16	1.19	1.22	1.15	1.10	1.05	1.09
Maldives	45 to 49	0.63	1.05	0.81	1.02	0.98	1.05	0.71	1.03
Mali	45 to 49	0.84	0.93	0.94	0.94	0.93	0.95	1.02	1.14
Malta	45 to 49	1.00	0.99	1.01	1.01	0.98	1.02	1.02	1.00
Marshall Islands	45 to 49	1.81	1.60	1.56	1.53	1.97	1.49	1.05	1.03
Mauritania	45 to 49	1.16	1.03	1.14	1.06	1.14	1.05	1.66	1.37
Mauritius	45 to 49	1.78	1.55	1.75	1.63	1.67	1.32	2.32	1.47
Mexico	45 to 49	1.13	1.20	1.28	1.40	1.06	1.24	1.21	1.17
Micronesia (Federated States of)	45 to 49	1.69	1.58	1.45	1.46	2.33	1.52	1.04	1.10
Monaco	45 to 49	1.11	1.06	1.36	1.21	1.07	1.01	1.09	1.06
Mongolia	45 to 49	1.35	1.50	1.37	1.40	1.39	1.58	1.12	1.25
Montenegro	45 to 49	1.22	1.25	1.27	1.54	1.00	1.00	1.12	1.09
Morocco	45 to 49	0.81	0.82	1.01	0.89	1.00	0.93	0.48	0.51
Mozambique	45 to 49	1.64	3.51	1.87	7.01	1.37	1.75	1.13	1.55
Myanmar	45 to 49	4.57	1.95	2.13	1.78	1.80	1.33	2.02	1.48
Namibia	45 to 49	1.33	1.55	1.37	1.80	1.14	1.17	1.16	1.23
Nauru	45 to 49	1.45	1.49	1.42	1.54	1.58	1.32	1.16	1.11
Nepal	45 to 49	0.90	1.08	1.17	1.29	1.07	1.12	0.72	1.00
Netherlands	45 to 49	1.02	1.00	1.09	1.00	1.04	1.04	1.03	1.01
New Zealand	45 to 49	0.95	1.01	1.08	1.11	1.24	1.19	0.99	1.01
Nicaragua	45 to 49	1.12	1.12	1.29	1.33	1.08	1.08	1.08	1.10
Niger	45 to 49	1.17	1.15	1.25	1.24	1.16	1.14	1.13	1.17
Nigeria	45 to 49	1.28	1.22	1.35	1.28	1.23	1.17	1.43	1.55
Niue	45 to 49	1.26	1.25	1.29	1.37	1.34	1.23	1.00	1.02
North Macedonia	45 to 49	1.25	1.11	1.37	1.21	1.04	0.94	1.37	1.12
Northern Mariana Islands	45 to 49	0.99	1.20	1.04	1.20	0.95	1.17	0.98	1.05
Norway	45 to 49	1.04	1.02	1.18	1.05	1.01	1.08	1.07	1.02
Oman	45 to 49	1.14	1.07	1.30	1.17	1.06	1.02	1.22	1.12
Pakistan	45 to 49	1.01	1.04	1.12	1.13	1.10	1.08	0.93	1.00
Palau	45 to 49	1.72	1.49	1.61	1.55	1.51	1.32	1.25	1.21
Palestine	45 to 49	1.20	1.13	1.53	1.33	1.04	1.03	1.49	1.23
Panama	45 to 49	1.22	1.30	1.37	1.57	1.10	1.20	1.28	1.12
Papua New Guinea	45 to 49	0.00	0.90	0.00	0.78	1.37	1.29	0.70	0.84
Paraguay	45 to 49	1.03	1.12	1.29	1.42	1.06	1.00	1.13	1.13
Peru	45 to 49	1.16	1.04	1.35	1.23	1.07	0.98	1.09	1.04
Philippines	45 to 49	3.48	1.72	15.93	1.78	1.31	1.19	1.63	1.33
Poland	45 to 49	1.55	1.38	1.56	1.56	1.45	1.31	2.94	1.39
Portugal	45 to 49	1.09	1.09	1.17	1.23	1.03	1.00	1.11	1.02
Puerto Rico	45 to 49	1.27	1.12	1.39	1.31	1.19	1.15	1.19	1.07
Qatar	45 to 49	0.98	1.04	1.04	1.08	1.18	1.15	0.94	1.05
Republic of Korea	45 to 49	1.14	1.08	1.27	1.15	1.86	1.15	1.01	1.01
Republic of Moldova	45 to 49	1.28	1.24	1.36	1.35	1.34	1.25	1.13	1.18
Romania	45 to 49	1.26	1.41	1.35	1.80	1.07	1.20	1.35	1.32

Russian Federation	45 to 49	1.35	1.44	1.34	1.63	1.28	1.32	2.48	1.81
Rwanda	45 to 49	2.44	1.39	2.36	1.81	1.46	1.18	0.98	1.01
Saint Kitts and Nevis	45 to 49	1.59	1.44	1.84	1.71	1.10	1.21	1.25	1.23
Saint Lucia	45 to 49	1.87	1.50	2.43	2.13	1.10	1.10	1.36	1.11
Saint Vincent and the Grenadines	45 to 49	1.69	1.64	3.47	2.26	1.06	1.15	1.16	1.14
Samoa	45 to 49	1.45	1.22	1.46	1.34	1.48	1.20	1.01	1.01
San Marino	45 to 49	1.07	1.01	1.16	1.01	1.06	1.02	1.07	1.01
Sao Tome and Principe	45 to 49	1.00	1.05	1.04	1.10	1.04	1.05	1.16	1.33
Saudi Arabia	45 to 49	0.91	1.05	1.23	1.35	1.02	1.00	0.45	0.96
Senegal	45 to 49	1.33	1.22	1.39	1.25	1.24	1.16	1.84	1.55
Serbia	45 to 49	1.21	1.22	1.31	1.46	1.08	1.04	1.38	1.33
Seychelles	45 to 49	1.49	1.31	1.42	1.38	1.27	1.13	1.54	1.29
Sierra Leone	45 to 49	1.33	1.21	1.35	1.26	1.21	1.12	2.14	2.00
Singapore	45 to 49	1.05	1.02	1.13	1.07	1.12	1.02	0.94	0.98
Slovakia	45 to 49	1.49	1.21	2.16	1.42	1.19	1.11	1.67	1.18
Slovenia	45 to 49	1.37	1.08	1.43	1.10	1.14	1.06	1.52	1.08
Solomon Islands	45 to 49	1.61	1.56	1.39	1.41	1.93	1.54	1.02	1.02
Somalia	45 to 49	2.24	1.64	2.28	2.11	1.45	1.25	1.00	1.01
South Africa	45 to 49	1.22	1.20	1.24	1.30	1.08	1.04	1.19	1.12
South Sudan	45 to 49	1.53	1.52	1.72	2.32	1.21	1.17	1.03	1.05
Spain	45 to 49	1.16	1.04	1.21	1.13	1.12	1.06	1.09	1.01
Sri Lanka	45 to 49	1.45	1.24	1.76	1.47	1.21	1.14	1.34	1.17
Sudan	45 to 49	0.70	0.86	1.16	0.96	1.03	0.98	0.08	0.70
Suriname	45 to 49	1.44	1.78	1.65	2.32	1.22	1.26	1.18	1.28
Sweden	45 to 49	1.02	0.98	1.07	0.98	1.07	1.03	1.07	1.00
Switzerland	45 to 49	1.00	1.02	1.04	0.99	1.00	1.06	1.03	1.01
Syrian Arab Republic	45 to 49	1.01	1.07	1.13	1.20	1.02	0.99	1.02	1.13
Taiwan (Province of China)	45 to 49	1.39	1.23	1.42	1.28	1.04	1.10	1.14	1.10
Tajikistan	45 to 49	1.14	1.11	1.19	1.17	1.00	1.07	2.06	1.20
Thailand	45 to 49	1.33	1.51	1.26	1.48	1.70	1.61	1.10	1.23
Timor-Leste	45 to 49	1.18	1.53	1.44	1.72	1.18	1.22	1.10	1.43
Togo	45 to 49	1.19	1.45	1.23	1.51	1.19	1.28	1.67	2.07
Tokelau	45 to 49	1.57	1.30	1.64	1.55	1.52	1.22	1.03	1.03
Tonga	45 to 49	1.13	1.07	1.26	1.18	1.14	1.08	1.03	1.00
Trinidad and Tobago	45 to 49	1.06	1.18	1.45	1.69	1.22	1.26	1.17	1.19
Tunisia	45 to 49	1.00	1.02	1.14	1.09	1.08	1.03	0.89	1.04
Turkey	45 to 49	1.17	1.08	1.21	1.15	1.48	1.16	1.18	1.07
Turkmenistan	45 to 49	1.32	1.51	1.31	1.65	1.31	1.38	1.73	1.56
Tuvalu	45 to 49	2.04	1.36	1.65	1.39	2.11	1.32	1.10	1.07
Uganda	45 to 49	1.66	1.39	1.93	1.63	1.34	1.21	1.04	1.04
Ukraine	45 to 49	1.18	1.28	1.35	1.44	1.22	1.27	1.27	1.36
United Arab Emirates	45 to 49	1.02	1.01	1.17	1.02	1.01	1.02	0.99	0.98
United Kingdom	45 to 49	1.05	1.04	1.11	1.09	1.16	1.10	1.10	1.03
United Republic of Tanzania	45 to 49	1.36	1.09	1.49	1.23	1.21	1.09	1.01	1.03
United States Virgin Islands	45 to 49	1.38	1.84	1.68	2.69	1.25	1.58	1.14	1.37
United States of America	45 to 49	1.01	0.99	1.16	1.11	1.19	1.18	1.02	1.00
Uruguay	45 to 49	1.13	1.07	1.35	1.18	1.36	1.26	1.05	1.03
Uzbekistan	45 to 49	1.19	1.18	1.31	1.18	1.10	1.27	1.19	1.27

Vanuatu	45 to 49	1.74	1.42	1.65	1.42	1.75	1.37	1.14	1.09
Venezuela (Bolivarian Republic of)	45 to 49	1.34	1.34	1.46	1.62	1.16	1.18	1.22	1.09
Viet Nam	45 to 49	1.89	1.86	1.65	1.90	1.47	1.33	1.48	1.57
Yemen	45 to 49	1.10	1.01	2.19	1.36	1.51	1.21	0.00	0.83
Zambia	45 to 49	1.39	1.51	1.51	1.65	1.15	1.15	0.98	1.07
Zimbabwe	45 to 49	1.08	1.40	1.11	2.77	1.04	1.10	1.03	1.22
Afghanistan	50 to 54	1.62	0.97	1.78	1.17	1.37	1.03	0.05	0.51
Albania	50 to 54	1.16	1.12	1.11	1.09	0.99	0.98	1.04	1.06
Algeria	50 to 54	0.89	0.94	0.94	0.93	0.94	0.94	0.70	0.82
American Samoa	50 to 54	1.46	1.26	1.25	1.17	0.94	0.90	1.00	0.94
Andorra	50 to 54	1.00	0.97	1.03	0.97	0.81	0.88	0.96	0.94
Angola	50 to 54	1.51	1.28	1.29	1.18	1.03	1.00	1.07	1.06
Antigua and Barbuda	50 to 54	2.60	1.24	1.80	1.28	1.51	0.91	1.47	1.02
Argentina	50 to 54	1.25	1.10	1.22	1.15	1.18	1.02	1.00	0.95
Armenia	50 to 54	1.07	1.24	1.08	1.31	0.97	1.10	1.02	1.33
Australia	50 to 54	1.04	0.98	1.05	1.02	0.94	0.98	0.97	0.92
Austria	50 to 54	1.10	1.00	1.07	0.99	0.97	0.97	1.17	0.93
Azerbaijan	50 to 54	1.27	1.12	1.14	1.06	1.03	1.02	1.25	1.06
Bahamas	50 to 54	1.48	1.36	1.32	1.33	0.99	0.97	1.10	1.08
Bahrain	50 to 54	0.86	0.98	0.98	0.97	0.77	0.98	0.67	0.99
Bangladesh	50 to 54	2.08	1.11	1.44	1.14	1.29	0.84	1.10	0.99
Barbados	50 to 54	1.19	1.16	1.10	1.19	1.03	1.02	1.01	0.98
Belarus	50 to 54	1.15	1.24	1.05	1.20	1.05	1.22	1.33	1.29
Belgium	50 to 54	1.10	1.05	1.08	1.09	0.99	0.95	1.00	0.97
Belize	50 to 54	1.06	1.09	1.05	1.05	0.90	0.92	0.97	0.97
Benin	50 to 54	1.12	1.21	0.93	1.02	0.93	0.97	1.54	1.60
Bermuda	50 to 54	1.32	1.17	1.29	1.25	1.14	1.12	1.33	1.08
Bhutan	50 to 54	1.04	1.04	1.12	1.08	0.91	0.93	0.84	0.96
Bolivia (Plurinational State of)	50 to 54	1.57	1.12	1.33	1.20	0.83	0.79	1.41	1.11
Bosnia and Herzegovina	50 to 54	1.12	1.09	1.16	1.17	0.95	0.98	1.12	1.10
Botswana	50 to 54	1.26	1.16	1.02	1.10	1.05	1.03	1.06	1.03
Brazil	50 to 54	1.70	1.23	1.46	1.35	1.16	0.97	1.91	1.18
Brunei Darussalam	50 to 54	0.91	1.01	0.96	1.07	0.76	0.94	0.84	0.91
Bulgaria	50 to 54	1.45	1.43	1.23	1.34	1.06	1.06	1.47	2.09
Burkina Faso	50 to 54	1.75	1.74	1.34	1.38	1.17	1.18	1.65	1.72
Burundi	50 to 54	2.48	1.35	1.63	1.33	1.16	1.05	0.97	0.95
Cabo Verde	50 to 54	1.46	1.36	1.18	1.12	1.15	1.07	3.05	2.48
Cambodia	50 to 54	1.33	1.34	1.10	1.16	1.01	1.02	1.04	1.04
Cameroon	50 to 54	1.36	1.37	1.14	1.13	1.03	1.02	1.59	1.81
Canada	50 to 54	1.02	0.99	1.05	0.99	0.99	1.04	0.95	0.93
Central African Republic	50 to 54	2.65	2.30	1.45	1.59	1.12	1.13	1.18	1.15
Chad	50 to 54	1.18	1.31	1.00	1.06	0.94	0.92	1.55	1.96
Chile	50 to 54	1.16	1.09	1.22	1.13	1.08	1.06	0.99	0.99
China	50 to 54	1.98	1.28	1.29	1.18	0.90	1.06	2.88	1.22
Colombia	50 to 54	1.12	1.10	1.14	1.16	0.96	0.97	1.07	1.07
Comoros	50 to 54	1.31	1.15	1.19	1.14	0.99	0.99	0.98	0.97
Congo	50 to 54	1.39	1.11	1.15	1.04	0.97	0.90	1.08	0.99
Cook Islands	50 to 54	1.37	1.16	1.24	1.18	1.02	0.97	0.98	0.95

Costa Rica	50 to 54	1.07	1.13	1.08	1.20	0.97	1.05	0.99	1.01
Coted'Ivoire	50 to 54	1.39	1.38	1.14	1.13	1.05	1.03	1.98	2.57
Croatia	50 to 54	1.41	1.21	1.37	1.36	1.10	1.08	1.18	1.02
Cuba	50 to 54	1.22	1.27	1.19	1.28	0.95	1.00	1.18	1.29
Cyprus	50 to 54	1.08	1.05	1.13	1.06	0.88	0.96	0.95	1.01
Czechia	50 to 54	1.29	1.16	1.26	1.20	1.00	1.03	1.41	1.09
Democratic People's Republic of Korea	50 to 54	1.39	1.60	1.19	1.36	0.98	1.00	1.15	1.32
Democratic Republic of the Congo	50 to 54	1.23	1.28	1.09	1.19	0.97	0.99	1.00	1.00
Denmark	50 to 54	1.06	1.04	1.09	1.06	0.96	0.97	1.00	0.96
Djibouti	50 to 54	1.35	1.24	1.21	1.20	1.05	1.04	0.99	1.05
Dominica	50 to 54	1.69	1.65	1.46	1.73	1.10	1.09	1.26	1.35
Dominican Republic	50 to 54	1.36	1.38	1.30	1.32	0.90	0.96	1.16	1.20
Ecuador	50 to 54	1.22	1.09	1.22	1.12	0.96	0.99	1.07	1.04
Egypt	50 to 54	1.51	1.17	1.94	1.25	1.30	1.05	1.36	1.45
El Salvador	50 to 54	1.30	1.31	1.18	1.39	1.04	1.04	1.28	1.25
Equatorial Guinea	50 to 54	2.01	1.16	1.37	1.11	1.12	0.95	1.17	1.09
Eritrea	50 to 54	3.16	1.57	1.56	1.41	1.25	1.09	1.00	1.02
Estonia	50 to 54	1.29	1.26	1.22	1.32	1.34	1.31	1.44	1.33
Eswatini	50 to 54	2.06	1.99	1.46	1.32	1.12	1.07	1.11	1.19
Ethiopia	50 to 54	1.62	1.18	1.27	1.13	1.07	1.00	0.90	0.92
Fiji	50 to 54	1.26	1.18	1.23	1.27	1.00	0.97	0.95	0.94
Finland	50 to 54	1.08	1.01	1.12	1.03	0.94	0.98	1.05	0.96
France	50 to 54	1.19	1.03	1.22	1.10	1.02	0.98	1.12	0.95
Gabon	50 to 54	1.35	1.24	1.20	1.17	1.05	1.01	1.02	1.03
Gambia	50 to 54	1.32	1.31	1.04	1.05	0.98	0.91	2.13	2.50
Georgia	50 to 54	1.42	1.41	1.16	1.21	1.09	1.20	1.18	1.53
Germany	50 to 54	1.06	1.05	1.12	1.07	0.95	0.92	1.02	0.99
Ghana	50 to 54	1.39	1.40	1.10	1.12	1.01	1.01	2.54	2.48
Greece	50 to 54	1.08	1.14	1.06	1.16	0.99	1.04	0.98	0.99
Greenland	50 to 54	1.11	1.10	1.36	1.26	1.01	1.03	1.03	0.98
Grenada	50 to 54	1.74	1.24	1.46	1.26	0.77	1.00	2.23	1.08
Guam	50 to 54	1.12	1.01	1.10	1.01	0.94	0.88	0.96	0.92
Guatemala	50 to 54	1.06	1.08	1.00	1.05	0.98	0.99	1.07	1.02
Guinea	50 to 54	0.94	1.14	0.88	0.98	0.84	0.89	1.42	1.86
Guinea-Bissau	50 to 54	3.01	1.90	1.31	1.20	1.18	0.98	14.02	7.30
Guyana	50 to 54	2.70	1.56	1.27	1.44	1.19	1.15	2.07	1.40
Haiti	50 to 54	12.00	1.84	1.63	1.82	0.26	0.62	0.62	0.97
Honduras	50 to 54	1.55	1.53	1.50	2.12	0.97	0.67	1.40	1.30
Hungary	50 to 54	1.33	1.27	1.22	1.32	1.07	0.98	1.60	1.30
Iceland	50 to 54	1.04	1.00	1.03	1.01	0.92	0.99	0.97	0.93
India	50 to 54	1.28	1.18	1.26	1.15	1.13	1.04	0.97	1.04
Indonesia	50 to 54	1.24	1.38	1.15	1.22	0.98	0.97	1.09	1.28
Iran (Islamic Republic of)	50 to 54	1.14	1.06	1.15	1.07	1.05	1.03	1.40	1.11
Iraq	50 to 54	1.53	1.27	1.29	1.16	1.09	0.99	1.01	1.60
Ireland	50 to 54	1.05	1.02	1.04	1.01	1.00	1.03	0.99	0.96
Israel	50 to 54	1.01	1.05	1.10	1.20	0.93	1.01	0.89	0.94
Italy	50 to 54	1.10	1.09	1.14	1.12	0.97	0.98	0.99	1.00
Jamaica	50 to 54	1.31	1.23	1.22	1.22	0.86	0.94	1.10	1.11

Japan	50 to 54	1.09	1.05	1.12	1.08	0.99	1.02	0.96	0.97
Jordan	50 to 54	0.98	1.00	1.05	1.01	0.97	1.00	0.90	0.91
Kazakhstan	50 to 54	1.16	1.27	1.10	1.25	0.95	1.02	1.28	1.24
Kenya	50 to 54	1.05	1.14	1.03	1.08	0.96	0.97	0.90	0.95
Kiribati	50 to 54	1.39	1.47	1.11	1.18	1.32	1.14	1.08	1.17
Kuwait	50 to 54	0.98	1.11	1.03	1.12	0.92	1.03	0.88	1.13
Kyrgyzstan	50 to 54	1.25	1.42	1.07	1.21	1.19	1.07	1.34	2.69
Lao People's Democratic Republic	50 to 54	2.10	1.35	1.26	1.17	0.87	1.01	1.14	1.27
Latvia	50 to 54	1.32	1.24	1.19	1.25	1.31	1.23	1.57	1.32
Lebanon	50 to 54	1.36	1.05	1.90	1.12	1.26	1.05	1.06	0.99
Lesotho	50 to 54	2.40	1.01	1.52	2.56	1.23	1.27	1.10	1.49
Liberia	50 to 54	1.03	1.10	0.90	0.94	0.85	0.87	1.56	1.85
Libya	50 to 54	0.91	0.97	0.93	0.92	0.91	0.89	0.84	1.03
Lithuania	50 to 54	1.19	1.20	1.08	1.23	1.26	1.17	1.21	1.17
Luxembourg	50 to 54	1.10	1.07	1.13	1.06	0.85	0.96	1.04	1.00
Madagascar	50 to 54	1.69	1.31	1.33	1.22	1.07	0.96	1.09	1.01
Malawi	50 to 54	1.21	1.53	1.16	1.51	0.98	1.02	0.96	1.10
Malaysia	50 to 54	1.22	1.15	1.12	1.10	1.04	0.99	1.03	1.05
Maldives	50 to 54	0.84	1.04	0.85	0.95	0.87	0.98	0.79	0.98
Mali	50 to 54	0.80	0.91	0.76	0.77	0.71	0.78	1.03	1.21
Malta	50 to 54	1.06	1.03	1.04	1.01	0.94	0.98	1.00	0.99
Marshall Islands	50 to 54	2.37	1.82	1.44	1.37	1.24	1.05	1.03	0.99
Mauritania	50 to 54	0.99	0.99	0.79	0.85	0.82	0.85	1.70	1.45
Mauritius	50 to 54	1.81	1.29	1.43	1.17	1.27	1.13	2.26	1.26
Mexico	50 to 54	1.14	1.20	1.15	1.25	0.99	1.09	1.13	1.12
Micronesia (Federated States of)	50 to 54	1.92	1.69	1.29	1.30	1.18	1.05	0.97	1.02
Monaco	50 to 54	1.07	1.04	1.15	1.08	0.87	0.92	0.99	0.98
Mongolia	50 to 54	1.38	1.47	1.17	1.23	1.13	1.06	1.08	1.18
Montenegro	50 to 54	1.43	1.26	1.23	1.27	0.99	0.94	1.12	1.06
Morocco	50 to 54	0.83	0.85	0.94	0.86	0.85	0.84	0.46	0.54
Mozambique	50 to 54	1.79	2.87	1.42	2.14	1.17	1.32	1.11	1.43
Myanmar	50 to 54	3.46	1.87	1.22	1.29	1.11	1.09	2.00	1.44
Namibia	50 to 54	1.37	1.60	1.14	1.39	1.05	1.08	1.10	1.16
Nauru	50 to 54	1.66	1.81	1.28	1.41	0.88	0.90	1.07	1.05
Nepal	50 to 54	1.02	1.19	1.13	1.22	0.94	1.03	0.73	1.03
Netherlands	50 to 54	1.04	1.02	1.07	1.02	1.00	0.95	0.98	0.98
New Zealand	50 to 54	0.95	0.99	1.01	1.04	0.78	0.91	0.94	0.95
Nicaragua	50 to 54	1.23	1.15	1.22	1.19	1.02	1.00	1.11	1.05
Niger	50 to 54	1.20	1.11	1.07	1.00	0.96	0.94	1.15	1.18
Nigeria	50 to 54	1.34	1.24	1.09	1.11	1.07	1.05	1.62	1.75
Niue	50 to 54	1.36	1.37	1.22	1.32	1.04	1.02	0.97	1.00
North Macedonia	50 to 54	1.21	1.14	1.16	1.14	0.84	0.82	1.22	1.11
Northern Mariana Islands	50 to 54	1.02	1.15	0.96	1.05	0.68	0.87	0.90	0.95
Norway	50 to 54	1.01	1.02	1.06	1.02	0.91	1.02	0.94	0.95
Oman	50 to 54	1.13	1.04	1.11	1.03	0.99	0.99	1.21	1.07
Pakistan	50 to 54	1.06	1.08	1.08	1.09	1.01	0.98	0.92	1.00
Palau	50 to 54	1.77	1.52	1.39	1.39	1.14	1.09	1.16	1.14
Palestine	50 to 54	1.26	1.15	1.29	1.21	1.00	1.00	1.89	1.27

Panama	50 to 54	1.21	1.29	1.21	1.29	0.94	1.10	1.14	1.06
Papua New Guinea	50 to 54	0.00	0.87	0.40	0.67	0.47	0.74	0.59	0.73
Paraguay	50 to 54	1.26	1.19	1.24	1.27	0.99	0.92	1.18	1.09
Peru	50 to 54	1.25	1.08	1.22	1.15	0.98	0.90	1.11	1.04
Philippines	50 to 54	2.34	1.63	1.72	1.34	1.09	1.04	1.52	1.26
Poland	50 to 54	1.59	1.37	1.32	1.34	1.18	1.14	2.91	1.42
Portugal	50 to 54	1.16	1.16	1.16	1.22	0.95	0.96	1.07	1.01
Puerto Rico	50 to 54	1.22	1.14	1.19	1.25	1.01	1.04	1.17	1.03
Qatar	50 to 54	1.00	1.01	1.04	1.01	1.20	1.11	0.89	1.00
Republic of Korea	50 to 54	1.08	1.04	1.14	1.07	1.12	1.02	0.95	0.96
Republic of Moldova	50 to 54	1.15	1.24	1.08	1.18	1.12	1.15	1.01	1.18
Romania	50 to 54	1.23	1.35	1.14	1.42	0.90	1.03	1.19	1.18
Russian Federation	50 to 54	1.30	1.34	1.13	1.24	1.08	1.08	2.08	1.63
Rwanda	50 to 54	2.27	1.38	1.50	1.40	1.14	1.06	0.93	0.96
Saint Kitts and Nevis	50 to 54	1.52	1.76	1.31	1.68	0.86	1.05	1.33	1.46
Saint Lucia	50 to 54	1.28	1.37	1.13	1.37	0.80	0.95	0.97	1.09
Saint Vincent and the Grenadines	50 to 54	1.13	1.62	1.11	1.53	0.94	1.00	0.93	1.15
Samoa	50 to 54	1.62	1.30	1.35	1.24	1.10	0.96	0.98	0.98
San Marino	50 to 54	1.07	1.02	1.08	1.01	0.99	0.98	1.01	0.96
Sao Tome and Principe	50 to 54	1.15	1.06	1.05	0.97	0.96	0.90	1.41	1.39
Saudi Arabia	50 to 54	0.94	1.05	1.06	1.15	0.92	0.93	0.45	0.91
Senegal	50 to 54	1.33	1.21	1.05	0.98	0.97	0.93	2.14	1.71
Serbia	50 to 54	1.24	1.23	1.17	1.29	0.82	0.82	1.43	1.29
Seychelles	50 to 54	1.46	1.23	1.19	1.14	1.11	1.02	1.47	1.19
Sierra Leone	50 to 54	1.38	1.19	1.08	1.01	0.99	0.90	2.79	2.36
Singapore	50 to 54	1.02	0.98	1.08	0.99	0.95	0.96	0.86	0.92
Slovakia	50 to 54	1.34	1.17	1.44	1.26	0.98	0.98	1.38	1.08
Slovenia	50 to 54	1.40	1.12	1.29	1.09	1.04	1.01	1.41	1.06
Solomon Islands	50 to 54	1.56	1.60	1.21	1.26	1.19	1.13	0.94	0.95
Somalia	50 to 54	2.17	1.62	1.45	1.47	1.19	1.09	0.94	0.95
South Africa	50 to 54	1.18	1.30	1.08	1.19	1.01	0.98	1.08	1.12
South Sudan	50 to 54	1.59	1.51	1.33	1.47	1.06	1.02	0.99	1.00
Spain	50 to 54	1.15	1.06	1.12	1.12	1.02	0.98	1.01	0.96
Sri Lanka	50 to 54	1.44	1.23	1.44	1.28	1.11	1.07	1.28	1.11
Sudan	50 to 54	0.74	0.85	1.01	0.86	0.84	0.84	0.09	0.65
Suriname	50 to 54	1.50	1.77	1.29	1.55	0.91	1.02	1.17	1.29
Sweden	50 to 54	1.03	0.98	1.03	0.97	0.87	0.95	1.02	0.97
Switzerland	50 to 54	1.05	1.03	1.05	0.99	0.93	1.02	1.02	0.96
Syrian Arab Republic	50 to 54	1.04	1.09	1.02	1.08	0.94	0.94	1.13	1.20
Taiwan (Province of China)	50 to 54	1.32	1.19	1.20	1.17	0.98	1.07	1.07	1.02
Tajikistan	50 to 54	1.26	1.12	1.12	1.07	0.89	0.96	2.08	1.11
Thailand	50 to 54	1.38	1.36	1.14	1.20	1.34	1.23	1.17	1.20
Timor-Leste	50 to 54	1.02	1.43	1.00	1.20	0.86	0.98	1.04	1.41
Togo	50 to 54	1.15	1.41	0.96	1.11	0.93	1.01	1.80	2.29
Tokelau	50 to 54	1.90	1.39	1.50	1.37	1.01	0.97	0.99	1.00
Tonga	50 to 54	1.10	1.12	1.10	1.11	0.91	0.93	0.96	0.97
Trinidad and Tobago	50 to 54	1.25	1.27	1.44	1.46	1.00	1.11	1.41	1.22
Tunisia	50 to 54	0.99	1.03	1.04	1.03	0.99	0.99	0.79	1.00

Turkey	50 to 54	1.16	1.10	1.10	1.11	1.14	1.05	1.16	1.07
Turkmenistan	50 to 54	1.26	1.44	1.12	1.31	0.98	1.12	1.45	1.32
Tuvalu	50 to 54	2.75	1.51	1.43	1.29	1.10	0.98	1.05	1.02
Uganda	50 to 54	1.71	1.40	1.50	1.36	1.17	1.09	0.99	0.98
Ukraine	50 to 54	1.14	1.24	1.12	1.21	0.99	1.11	1.21	1.28
United Arab Emirates	50 to 54	1.01	1.02	1.08	1.02	0.83	1.00	0.90	0.94
United Kingdom	50 to 54	1.05	1.05	1.07	1.05	0.92	1.00	1.02	0.98
United Republic of Tanzania	50 to 54	1.33	1.05	1.22	1.07	1.06	0.98	0.93	0.95
United States Virgin Islands	50 to 54	1.42	1.18	1.35	1.18	1.02	1.06	1.18	1.02
United States of America	50 to 54	1.02	1.00	1.08	1.06	1.00	1.07	0.97	1.00
Uruguay	50 to 54	1.14	1.06	1.25	1.10	1.08	1.05	0.99	0.99
Uzbekistan	50 to 54	1.21	1.19	1.15	1.11	1.00	1.12	1.18	1.18
Vanuatu	50 to 54	1.80	1.51	1.39	1.29	1.11	1.03	1.07	1.03
Venezuela (Bolivarian Republic of)	50 to 54	1.31	1.45	1.22	1.43	0.99	1.04	1.11	1.07
Viet Nam	50 to 54	1.96	1.80	1.32	1.46	1.25	1.17	1.49	1.50
Yemen	50 to 54	1.23	1.05	1.52	1.11	1.19	1.01	0.00	1.00
Zambia	50 to 54	1.35	1.47	1.16	1.22	0.99	1.00	0.93	1.01
Zimbabwe	50 to 54	1.14	1.97	0.83	0.00	0.93	0.95	0.99	1.25
Afghanistan	55 to 59	1.27	0.86	2.26	1.25	2.06	1.14	0.00	0.57
Albania	55 to 59	1.20	1.13	1.22	1.13	1.03	0.97	1.07	1.07
Algeria	55 to 59	0.93	0.94	0.99	0.94	1.00	0.96	0.82	0.87
American Samoa	55 to 59	1.31	1.16	1.17	1.09	0.92	0.87	1.07	1.02
Andorra	55 to 59	0.99	0.97	1.01	0.96	0.85	0.88	0.95	0.94
Angola	55 to 59	1.51	1.25	1.38	1.21	1.05	1.00	1.12	1.10
Antigua and Barbuda	55 to 59	2.04	1.36	1.71	1.44	1.37	1.04	1.37	1.07
Argentina	55 to 59	1.16	1.08	1.20	1.14	1.30	1.02	1.00	0.96
Armenia	55 to 59	1.03	1.19	1.02	1.29	1.01	1.08	1.10	1.31
Australia	55 to 59	1.04	0.98	1.03	0.98	1.05	0.98	0.97	0.92
Austria	55 to 59	1.10	1.00	1.04	0.96	0.97	0.96	1.18	0.94
Azerbaijan	55 to 59	1.23	1.09	1.17	1.06	1.00	0.96	1.23	1.06
Bahamas	55 to 59	1.54	1.33	1.49	1.38	1.09	1.07	1.15	1.08
Bahrain	55 to 59	0.81	0.91	1.00	0.91	0.78	0.93	0.66	0.88
Bangladesh	55 to 59	0.94	0.98	1.06	1.06	1.00	0.86	0.80	0.94
Barbados	55 to 59	1.44	1.20	1.44	1.30	1.16	1.05	1.21	1.05
Belarus	55 to 59	1.03	1.16	0.99	1.16	1.08	1.24	1.22	1.38
Belgium	55 to 59	1.10	1.07	1.13	1.09	0.98	1.00	1.01	0.99
Belize	55 to 59	1.39	1.21	1.44	1.20	1.05	1.03	1.17	1.09
Benin	55 to 59	1.01	1.09	0.88	0.95	0.91	0.93	1.54	1.61
Bermuda	55 to 59	1.24	1.19	1.23	1.28	1.21	1.17	1.35	1.11
Bhutan	55 to 59	0.86	0.97	1.04	1.03	0.85	0.87	0.74	0.93
Bolivia (Plurinational State of)	55 to 59	1.38	1.12	1.42	1.27	0.89	0.81	1.35	1.16
Bosnia and Herzegovina	55 to 59	1.06	1.09	1.18	1.23	0.96	1.01	1.08	1.12
Botswana	55 to 59	1.19	1.13	1.03	1.08	1.03	1.00	1.02	1.02
Brazil	55 to 59	1.41	1.16	1.57	1.38	1.42	1.06	1.59	1.17
Brunei Darussalam	55 to 59	0.93	0.95	0.99	0.97	0.83	0.93	0.88	0.91
Bulgaria	55 to 59	1.24	1.28	1.21	1.25	1.02	1.06	1.31	1.96
Burkina Faso	55 to 59	1.52	1.57	1.27	1.30	1.18	1.16	1.58	1.72
Burundi	55 to 59	2.35	1.32	1.82	1.42	1.21	1.05	0.98	0.99

Cabo Verde	55 to 59	1.27	1.28	1.09	1.07	1.09	1.04	2.28	2.43
Cambodia	55 to 59	1.07	1.29	1.14	1.21	1.05	1.03	1.03	1.08
Cameroon	55 to 59	1.16	1.19	1.05	1.05	1.00	0.99	1.47	1.75
Canada	55 to 59	1.03	1.00	1.05	0.97	1.06	1.02	0.95	0.97
Central African Republic	55 to 59	2.55	2.30	1.59	1.79	1.17	1.17	1.21	1.22
Chad	55 to 59	1.08	1.16	0.97	1.00	0.96	0.91	1.45	1.90
Chile	55 to 59	1.08	1.07	1.19	1.11	1.06	1.05	0.99	0.99
China	55 to 59	1.75	1.19	1.33	1.16	1.50	1.05	2.35	1.19
Colombia	55 to 59	1.12	1.11	1.20	1.15	1.01	1.03	1.09	1.06
Comoros	55 to 59	1.23	1.11	1.17	1.11	0.98	0.96	0.99	0.99
Congo	55 to 59	1.29	1.06	1.14	1.01	0.96	0.89	1.11	1.02
Cook Islands	55 to 59	1.32	1.15	1.20	1.13	1.00	0.91	1.08	1.00
Costa Rica	55 to 59	1.05	1.13	1.06	1.15	0.94	1.09	1.03	1.02
Coted'Ivoire	55 to 59	1.22	1.20	1.07	1.04	1.02	0.99	1.74	2.20
Croatia	55 to 59	1.22	1.16	1.50	1.40	1.12	1.23	1.11	1.02
Cuba	55 to 59	1.15	1.22	1.17	1.25	0.97	1.02	1.16	1.31
Cyprus	55 to 59	1.07	1.04	1.11	1.05	0.95	0.93	0.95	1.01
Czechia	55 to 59	1.19	1.10	1.31	1.13	1.05	1.00	1.31	1.04
Democratic People's Republic of Korea	55 to 59	1.36	1.44	1.28	1.46	1.09	1.05	1.21	1.35
Democratic Republic of the Congo	55 to 59	1.19	1.26	1.09	1.23	0.96	0.97	1.03	1.04
Denmark	55 to 59	1.03	1.06	1.09	1.09	0.98	0.97	0.96	0.98
Djibouti	55 to 59	1.28	1.21	1.20	1.20	1.04	1.01	1.00	1.08
Dominica	55 to 59	1.98	1.77	1.88	1.96	1.29	1.26	1.49	1.52
Dominican Republic	55 to 59	1.20	1.35	1.24	1.33	1.01	1.03	1.08	1.24
Ecuador	55 to 59	1.19	1.06	1.23	1.08	0.97	0.99	1.20	1.03
Egypt	55 to 59	1.69	1.14	3.79	1.32	1.85	1.10	1.01	1.74
El Salvador	55 to 59	1.18	1.24	1.15	1.36	1.02	1.02	1.18	1.21
Equatorial Guinea	55 to 59	1.90	1.14	1.49	1.10	1.15	0.93	1.20	1.14
Eritrea	55 to 59	2.63	1.44	1.63	1.41	1.29	1.07	0.99	1.03
Estonia	55 to 59	1.22	1.21	1.16	1.25	1.40	1.21	1.76	1.30
Eswatini	55 to 59	2.04	1.70	1.57	1.28	1.15	1.05	1.15	1.19
Ethiopia	55 to 59	1.41	1.16	1.22	1.14	1.04	0.98	0.89	0.92
Fiji	55 to 59	1.32	1.14	1.32	1.22	1.02	0.94	1.08	1.01
Finland	55 to 59	1.05	1.00	1.10	0.99	0.90	0.96	1.04	0.94
France	55 to 59	1.18	1.03	1.21	1.05	1.01	0.98	1.12	0.95
Gabon	55 to 59	1.35	1.21	1.25	1.16	1.04	1.00	1.05	1.07
Gambia	55 to 59	1.17	1.15	0.98	0.98	0.97	0.89	1.93	2.36
Georgia	55 to 59	1.41	1.41	1.17	1.24	1.07	1.14	1.18	1.70
Germany	55 to 59	1.03	1.04	1.13	1.03	0.97	0.92	1.01	0.99
Ghana	55 to 59	1.14	1.25	0.96	1.05	0.96	0.97	2.02	2.27
Greece	55 to 59	1.03	1.11	1.05	1.13	1.01	1.02	0.95	0.98
Greenland	55 to 59	0.99	1.11	1.17	1.22	1.01	1.25	1.01	1.00
Grenada	55 to 59	0.98	1.28	1.07	1.39	0.88	1.08	0.90	1.17
Guam	55 to 59	1.11	1.11	1.08	1.09	0.90	0.90	1.05	1.04
Guatemala	55 to 59	1.03	1.06	1.00	1.04	0.95	0.99	0.99	1.01
Guinea	55 to 59	0.86	1.00	0.83	0.90	0.84	0.85	1.33	1.79
Guinea-Bissau	55 to 59	2.04	1.46	1.23	1.08	1.21	0.96	9.23	6.16
Guyana	55 to 59	1.01	1.57	2.15	1.56	1.62	1.26	148.14	1.69

Haiti	55 to 59	49.92	1.49	2.01	2.10	0.81	0.77	0.43	0.93
Honduras	55 to 59	1.51	1.55	1.72	3.08	1.15	0.71	1.45	1.50
Hungary	55 to 59	1.23	1.22	1.18	1.30	1.15	1.00	1.73	1.32
Iceland	55 to 59	1.02	1.02	1.01	1.02	0.91	0.97	0.97	0.94
India	55 to 59	1.05	0.99	1.19	1.07	1.14	0.89	0.87	0.95
Indonesia	55 to 59	1.12	1.28	1.16	1.24	0.98	0.97	1.05	1.27
Iran (Islamic Republic of)	55 to 59	1.12	1.04	1.19	1.06	1.09	1.02	1.55	1.11
Iraq	55 to 59	1.32	1.25	1.31	1.22	1.21	1.05	8.09	2.05
Ireland	55 to 59	1.04	1.02	1.04	1.01	0.95	1.01	1.01	0.95
Israel	55 to 59	0.97	1.02	1.11	1.13	0.92	1.00	0.87	0.94
Italy	55 to 59	1.07	1.07	1.18	1.08	0.98	0.98	1.00	1.00
Jamaica	55 to 59	1.42	1.31	1.40	1.31	0.97	1.00	1.13	1.21
Japan	55 to 59	1.04	1.02	1.07	1.01	0.99	0.98	0.97	0.98
Jordan	55 to 59	0.89	0.95	0.99	0.95	0.95	0.99	0.73	0.86
Kazakhstan	55 to 59	1.13	1.24	1.12	1.24	1.06	1.12	1.32	1.32
Kenya	55 to 59	1.04	1.14	1.03	1.10	0.95	0.97	0.90	0.97
Kiribati	55 to 59	1.31	1.33	1.06	1.10	1.33	1.08	1.21	1.30
Kuwait	55 to 59	0.92	1.07	0.96	1.05	0.91	1.01	0.76	1.07
Kyrgyzstan	55 to 59	1.19	1.31	1.08	1.20	1.41	1.13	1.30	1.95
Lao People's Democratic Republic	55 to 59	1.53	1.25	1.39	1.20	1.05	1.01	1.18	1.29
Latvia	55 to 59	1.24	1.17	1.25	1.27	1.34	1.11	1.66	1.28
Lebanon	55 to 59	1.26	1.02	2.03	1.07	1.56	1.05	1.05	0.97
Lesotho	55 to 59	2.47	14.84	1.69	2.62	1.28	1.29	1.11	1.46
Liberia	55 to 59	0.92	0.97	0.84	0.85	0.85	0.83	1.48	1.90
Libya	55 to 59	0.92	0.98	0.94	0.93	0.92	0.91	0.86	1.09
Lithuania	55 to 59	1.12	1.16	1.05	1.19	1.18	1.19	1.24	1.21
Luxembourg	55 to 59	1.08	1.08	1.17	1.05	0.90	0.94	1.06	1.01
Madagascar	55 to 59	1.54	1.20	1.32	1.19	1.09	0.94	1.10	1.02
Malawi	55 to 59	1.11	1.36	1.15	1.57	0.99	1.02	0.95	1.12
Malaysia	55 to 59	1.16	1.11	1.14	1.09	1.03	0.97	1.02	1.04
Maldives	55 to 59	0.82	1.00	0.89	0.92	0.88	0.95	0.82	0.96
Mali	55 to 59	0.81	0.86	0.79	0.77	0.77	0.79	1.08	1.20
Malta	55 to 59	1.04	1.04	1.05	1.00	0.92	0.96	0.94	0.98
Marshall Islands	55 to 59	1.73	1.53	1.29	1.28	1.19	1.03	1.10	1.08
Mauritania	55 to 59	0.90	0.93	0.74	0.80	0.82	0.83	1.65	1.46
Mauritius	55 to 59	1.52	1.18	1.40	1.10	1.58	1.10	1.71	1.18
Mexico	55 to 59	1.12	1.14	1.18	1.21	1.00	1.07	1.14	1.10
Micronesia (Federated States of)	55 to 59	1.54	1.44	1.22	1.24	1.18	1.03	1.10	1.13
Monaco	55 to 59	1.03	1.02	1.10	1.03	0.89	0.91	0.97	0.96
Mongolia	55 to 59	1.64	1.44	1.30	1.23	1.40	1.34	1.07	1.15
Montenegro	55 to 59	1.42	1.24	1.30	1.35	0.99	0.88	1.13	1.09
Morocco	55 to 59	0.77	0.84	0.92	0.88	0.90	0.85	0.46	0.56
Mozambique	55 to 59	1.83	2.63	1.64	2.47	1.24	1.37	1.18	1.60
Myanmar	55 to 59	2.22	1.65	1.38	1.38	1.35	1.14	1.60	1.38
Namibia	55 to 59	1.29	1.51	1.13	1.42	1.04	1.07	1.09	1.17
Nauru	55 to 59	1.43	1.52	1.24	1.36	0.93	0.89	1.28	1.23
Nepal	55 to 59	0.77	1.08	1.07	1.17	0.89	1.04	0.56	1.00
Netherlands	55 to 59	1.03	1.02	1.08	1.00	1.02	0.99	0.99	0.97

New Zealand	55 to 59	0.95	1.00	1.00	1.00	0.87	1.05	0.96	0.95
Nicaragua	55 to 59	1.18	1.16	1.23	1.22	1.00	1.00	1.10	1.07
Niger	55 to 59	1.11	1.02	1.02	0.95	0.97	0.93	1.14	1.19
Nigeria	55 to 59	1.21	1.12	1.04	1.01	1.07	1.00	1.60	1.62
Niue	55 to 59	1.28	1.29	1.18	1.24	1.01	0.96	1.06	1.08
North Macedonia	55 to 59	1.13	1.07	1.25	1.16	0.83	0.75	1.22	1.11
Northern Mariana Islands	55 to 59	1.01	1.17	0.96	1.08	0.71	0.88	1.02	1.05
Norway	55 to 59	0.99	1.01	1.05	0.99	0.93	1.01	0.93	0.94
Oman	55 to 59	1.10	1.02	1.20	1.03	1.02	1.00	1.21	1.08
Pakistan	55 to 59	0.94	0.98	1.02	1.04	0.95	0.92	0.84	0.95
Palau	55 to 59	1.74	1.51	1.41	1.38	1.10	1.03	1.31	1.26
Palestine	55 to 59	1.20	1.12	1.38	1.24	1.00	0.99	2.28	1.34
Panama	55 to 59	1.13	1.24	1.15	1.25	0.93	1.09	1.03	1.06
Papua New Guinea	55 to 59	0.00	0.66	0.63	0.77	0.71	0.79	0.75	0.83
Paraguay	55 to 59	1.11	1.19	1.23	1.40	1.00	0.99	1.10	1.11
Peru	55 to 59	1.18	1.05	1.19	1.10	1.01	0.89	1.09	1.03
Philippines	55 to 59	1.72	1.49	1.60	1.35	1.12	1.05	1.35	1.24
Poland	55 to 59	1.36	1.27	1.30	1.31	1.18	1.10	2.40	1.38
Portugal	55 to 59	1.15	1.15	1.25	1.21	1.14	1.00	1.07	1.05
Puerto Rico	55 to 59	1.22	1.11	1.18	1.18	1.03	1.07	1.22	1.03
Qatar	55 to 59	0.93	0.95	0.96	0.91	1.32	1.11	0.85	0.94
Republic of Korea	55 to 59	1.01	1.01	1.08	1.00	1.14	0.98	0.95	0.97
Republic of Moldova	55 to 59	1.03	1.16	1.08	1.12	1.10	1.17	0.98	1.23
Romania	55 to 59	1.16	1.27	1.24	1.55	0.98	1.16	1.14	1.17
Russian Federation	55 to 59	1.24	1.28	1.15	1.22	1.12	1.12	2.66	1.82
Rwanda	55 to 59	1.97	1.34	1.59	1.49	1.18	1.04	0.93	0.98
Saint Kitts and Nevis	55 to 59	1.49	1.65	1.37	1.69	1.00	1.12	1.57	1.50
Saint Lucia	55 to 59	1.77	1.36	1.59	1.40	0.98	1.02	1.75	1.11
Saint Vincent and the Grenadines	55 to 59	1.05	1.48	1.11	1.53	0.96	1.02	0.92	1.12
Samoa	55 to 59	1.53	1.22	1.33	1.18	1.09	0.92	1.08	1.08
San Marino	55 to 59	1.04	1.01	1.06	0.98	0.97	0.95	1.00	0.96
Sao Tome and Principe	55 to 59	0.95	1.01	0.88	0.92	0.89	0.87	1.14	1.41
Saudi Arabia	55 to 59	0.86	0.99	1.10	1.14	0.93	0.93	0.48	0.92
Senegal	55 to 59	1.14	1.11	0.96	0.94	0.95	0.91	1.91	1.76
Serbia	55 to 59	1.24	1.20	1.33	1.38	0.88	0.87	1.63	1.32
Seychelles	55 to 59	1.66	1.25	1.43	1.20	1.22	1.03	1.74	1.25
Sierra Leone	55 to 59	1.20	1.04	1.00	0.92	0.97	0.87	2.35	2.08
Singapore	55 to 59	0.96	0.95	1.02	0.94	0.91	0.93	0.85	0.91
Slovakia	55 to 59	1.16	1.11	1.43	1.26	1.01	0.99	1.18	1.07
Slovenia	55 to 59	1.35	1.11	1.38	1.07	1.14	1.03	1.51	1.08
Solomon Islands	55 to 59	1.46	1.42	1.19	1.20	1.22	1.08	1.02	1.03
Somalia	55 to 59	2.10	1.55	1.57	1.60	1.24	1.10	0.94	0.96
South Africa	55 to 59	1.10	1.21	1.06	1.21	0.99	0.97	1.04	1.09
South Sudan	55 to 59	1.51	1.42	1.39	1.55	1.05	1.00	0.99	1.01
Spain	55 to 59	1.10	1.05	1.10	1.07	1.04	0.98	0.99	0.97
Sri Lanka	55 to 59	1.31	1.18	1.41	1.27	1.09	1.04	1.20	1.11
Sudan	55 to 59	0.62	0.82	1.03	0.86	0.92	0.86	0.11	0.68
Suriname	55 to 59	1.48	1.88	1.39	1.82	1.05	1.10	1.28	1.46

Sweden	55 to 59	1.04	0.98	1.04	0.95	0.90	0.94	1.03	0.97
Switzerland	55 to 59	1.05	1.01	1.05	0.95	0.94	0.97	1.00	0.95
Syrian Arab Republic	55 to 59	1.01	1.07	1.02	1.11	1.02	0.98	1.09	1.17
Taiwan (Province of China)	55 to 59	1.28	1.12	1.22	1.09	0.99	1.03	1.06	0.99
Tajikistan	55 to 59	1.24	1.06	1.16	1.05	0.89	0.91	2.14	1.08
Thailand	55 to 59	1.28	1.21	1.11	1.11	1.46	1.20	1.14	1.12
Timor-Leste	55 to 59	0.79	1.23	1.00	1.21	0.83	0.96	1.02	1.35
Togo	55 to 59	1.00	1.26	0.89	1.06	0.90	0.98	1.65	2.41
Tokelau	55 to 59	1.69	1.30	1.47	1.27	1.04	0.94	1.16	1.10
Tonga	55 to 59	1.12	1.13	1.11	1.09	0.92	0.90	1.07	1.07
Trinidad and Tobago	55 to 59	1.20	1.35	1.49	1.61	1.15	1.30	1.61	1.39
Tunisia	55 to 59	0.96	1.01	1.06	1.03	1.03	0.99	0.81	1.03
Turkey	55 to 59	1.16	1.08	1.13	1.09	1.46	1.10	1.21	1.07
Turkmenistan	55 to 59	1.18	1.36	1.12	1.33	1.08	1.21	1.37	1.31
Tuvalu	55 to 59	2.15	1.35	1.40	1.21	1.20	0.95	1.24	1.15
Uganda	55 to 59	1.61	1.33	1.62	1.39	1.17	1.06	0.97	0.98
Ukraine	55 to 59	1.09	1.20	1.10	1.21	1.06	1.12	1.23	1.37
United Arab Emirates	55 to 59	0.91	1.03	1.01	1.01	0.70	1.04	0.80	0.96
United Kingdom	55 to 59	1.03	1.03	1.07	1.02	0.93	0.97	1.02	0.97
United Republic of Tanzania	55 to 59	1.26	0.99	1.26	1.02	1.05	0.94	0.94	0.95
United States Virgin Islands	55 to 59	1.41	1.22	1.44	1.25	1.13	1.06	1.24	1.08
United States of America	55 to 59	1.00	1.00	1.06	1.03	1.04	1.10	0.95	1.00
Uruguay	55 to 59	1.11	1.07	1.28	1.15	1.14	1.08	1.04	1.02
Uzbekistan	55 to 59	1.17	1.13	1.15	1.08	1.04	1.17	1.29	1.16
Vanuatu	55 to 59	1.62	1.37	1.35	1.24	1.12	1.00	1.20	1.12
Venezuela (Bolivarian Republic of)	55 to 59	1.26	1.37	1.23	1.43	1.03	1.02	1.14	1.11
Viet Nam	55 to 59	2.08	1.73	1.51	1.62	1.44	1.23	1.52	1.49
Yemen	55 to 59	1.01	0.99	1.82	1.17	1.55	1.13	0.06	1.13
Zambia	55 to 59	1.31	1.37	1.17	1.20	0.98	0.97	0.93	1.02
Zimbabwe	55 to 59	1.14	1.73	0.90	0.00	0.94	0.97	1.00	1.23
Afghanistan	60 to 64	0.86	0.80	4.19	1.47	1.83	1.09	0.00	0.39
Albania	60 to 64	1.18	1.06	1.53	1.20	1.04	0.97	0.96	0.92
Algeria	60 to 64	0.94	0.95	1.08	1.01	1.02	0.99	0.73	0.78
American Samoa	60 to 64	1.19	1.12	1.26	1.22	0.81	0.80	0.97	0.94
Andorra	60 to 64	1.00	0.99	1.04	1.00	0.84	0.90	0.88	0.90
Angola	60 to 64	1.43	1.24	1.66	1.40	1.04	1.00	1.06	1.04
Antigua and Barbuda	60 to 64	1.51	1.32	1.74	1.62	1.05	1.03	1.03	1.01
Argentina	60 to 64	1.10	1.06	1.28	1.21	1.04	0.98	0.94	0.93
Armenia	60 to 64	1.03	1.09	1.14	1.24	0.95	1.03	0.90	0.97
Australia	60 to 64	1.01	1.01	1.05	1.03	1.01	1.02	0.88	0.91
Austria	60 to 64	1.07	1.02	1.08	1.02	0.98	0.99	0.92	0.92
Azerbaijan	60 to 64	1.17	1.06	1.28	1.12	1.00	0.99	1.03	0.95
Bahamas	60 to 64	1.31	1.18	1.64	1.35	0.98	0.99	0.92	0.95
Bahrain	60 to 64	0.89	0.89	1.34	1.00	0.75	0.95	0.58	0.76
Bangladesh	60 to 64	0.69	0.90	1.40	1.17	0.82	0.79	0.43	0.75
Barbados	60 to 64	1.24	1.10	1.40	1.25	1.07	0.97	0.98	0.90
Belarus	60 to 64	1.03	1.16	1.07	1.23	1.05	1.17	0.86	1.06
Belgium	60 to 64	1.09	1.07	1.19	1.16	0.95	0.98	0.91	0.94

Belize	60 to 64	1.23	1.01	1.60	1.12	1.00	0.93	1.01	0.89
Benin	60 to 64	1.08	1.16	0.95	1.02	0.93	0.95	1.53	1.60
Bermuda	60 to 64	1.24	1.21	1.36	1.34	1.14	1.16	1.12	1.07
Bhutan	60 to 64	0.72	0.92	1.09	1.08	0.73	0.82	0.55	0.79
Bolivia (Plurinational State of)	60 to 64	1.14	1.12	1.55	1.39	0.75	0.78	1.18	1.11
Bosnia and Herzegovina	60 to 64	1.01	1.07	1.33	1.27	0.86	0.99	0.77	0.93
Botswana	60 to 64	1.23	1.18	1.15	1.15	1.01	1.00	1.10	1.07
Brazil	60 to 64	1.17	1.11	1.47	1.36	0.89	0.81	1.13	1.04
Brunei Darussalam	60 to 64	1.00	0.96	1.09	1.02	0.86	0.92	0.85	0.87
Bulgaria	60 to 64	1.06	1.22	1.32	1.32	0.93	1.09	0.87	1.11
Burkina Faso	60 to 64	1.60	1.63	1.50	1.47	1.18	1.15	1.58	1.73
Burundi	60 to 64	2.38	1.33	5.09	2.19	1.19	1.05	0.95	0.93
Cabo Verde	60 to 64	1.10	1.36	1.04	1.16	1.03	1.05	1.32	2.05
Cambodia	60 to 64	0.83	1.14	1.23	1.32	0.91	0.96	0.90	0.96
Cameroon	60 to 64	1.26	1.27	1.20	1.15	1.03	1.00	1.47	1.72
Canada	60 to 64	1.05	1.01	1.09	1.01	1.00	0.99	0.91	0.95
Central African Republic	60 to 64	2.18	1.94	2.46	2.63	1.09	1.09	1.12	1.14
Chad	60 to 64	1.17	1.26	1.08	1.10	0.99	0.94	1.43	1.89
Chile	60 to 64	1.05	1.05	1.25	1.12	0.89	1.00	0.89	0.95
China	60 to 64	1.29	1.16	1.52	1.31	1.01	1.06	0.91	0.93
Colombia	60 to 64	1.11	1.11	1.29	1.20	0.94	0.97	0.99	1.03
Comoros	60 to 64	1.30	1.15	1.57	1.33	1.00	1.00	0.98	0.97
Congo	60 to 64	1.26	1.08	1.31	1.12	0.96	0.92	1.05	0.98
Cook Islands	60 to 64	1.24	1.15	1.35	1.25	0.88	0.91	0.98	0.96
Costa Rica	60 to 64	1.03	1.11	1.08	1.17	0.92	1.03	0.93	0.99
Coted'Ivoire	60 to 64	1.28	1.26	1.17	1.12	1.02	0.98	1.61	1.92
Croatia	60 to 64	1.05	1.09	1.46	1.47	0.87	1.01	0.86	0.91
Cuba	60 to 64	1.08	1.19	1.23	1.34	0.98	1.00	0.90	1.07
Cyprus	60 to 64	1.10	1.06	1.24	1.09	0.94	0.95	0.88	0.97
Czechia	60 to 64	1.13	1.08	1.38	1.17	0.94	1.04	0.94	0.93
Democratic People's Republic of Korea	60 to 64	1.23	1.25	1.56	1.78	1.08	1.06	0.88	0.91
Democratic Republic of the Congo	60 to 64	1.17	1.26	1.25	1.46	0.97	0.99	0.95	1.00
Denmark	60 to 64	1.02	1.08	1.12	1.16	0.90	0.99	0.87	0.97
Djibouti	60 to 64	1.33	1.23	1.60	1.40	1.06	1.02	0.98	1.05
Dominica	60 to 64	1.78	1.50	2.59	2.03	1.21	1.17	1.26	1.17
Dominican Republic	60 to 64	1.27	1.24	1.63	1.36	1.02	0.97	1.02	1.04
Ecuador	60 to 64	1.17	1.10	1.30	1.25	1.03	0.99	1.12	1.03
Egypt	60 to 64	1.27	1.04	26.17	1.31	1.67	1.06	0.11	0.77
El Salvador	60 to 64	1.15	1.16	1.28	1.32	0.97	0.96	1.05	1.07
Equatorial Guinea	60 to 64	1.71	1.14	2.02	1.18	1.07	0.96	1.13	1.05
Eritrea	60 to 64	2.45	1.52	4.19	2.34	1.23	1.09	0.97	1.04
Estonia	60 to 64	1.10	1.20	1.24	1.26	1.20	1.16	0.85	1.08
Eswatini	60 to 64	2.00	1.66	2.02	1.47	1.07	1.03	1.31	1.37
Ethiopia	60 to 64	1.79	1.22	1.94	1.42	1.10	1.01	0.90	0.92
Fiji	60 to 64	1.11	1.09	1.40	1.40	0.83	0.83	0.94	0.92
Finland	60 to 64	1.02	1.04	1.08	1.06	0.90	1.00	0.85	0.93
France	60 to 64	1.15	1.04	1.29	1.09	1.00	1.00	1.00	0.92
Gabon	60 to 64	1.35	1.21	1.49	1.29	1.04	1.01	1.02	1.02

Gambia	60 to 64	1.25	1.30	1.08	1.10	0.99	0.95	1.74	2.21
Georgia	60 to 64	1.37	1.27	1.32	1.27	1.01	1.00	1.14	1.14
Germany	60 to 64	1.04	1.04	1.16	1.08	0.95	0.96	0.91	0.92
Ghana	60 to 64	1.20	1.31	1.02	1.10	0.98	0.97	1.83	1.97
Greece	60 to 64	1.00	1.09	1.13	1.17	0.96	1.02	0.87	0.94
Greenland	60 to 64	0.97	1.07	1.18	1.20	0.29	0.95	0.91	0.95
Grenada	60 to 64	1.17	1.13	1.73	1.39	0.87	0.95	0.78	0.93
Guam	60 to 64	1.06	1.07	1.13	1.11	0.80	0.86	0.93	0.96
Guatemala	60 to 64	1.04	1.06	1.12	1.16	1.00	1.00	0.94	0.98
Guinea	60 to 64	0.96	1.09	0.90	0.96	0.90	0.91	1.32	1.70
Guinea-Bissau	60 to 64	2.04	1.56	1.40	1.17	1.11	0.97	5.62	4.71
Guyana	60 to 64	#####	1.48	3.63	1.81	1.15	1.11	1.01	1.32
Haiti	60 to 64	0.44	0.98	3.21	2.63	0.57	0.63	0.21	0.63
Honduras	60 to 64	1.23	1.23	1.77	2.64	0.96	0.59	1.20	1.30
Hungary	60 to 64	1.13	1.16	1.28	1.31	1.07	1.03	0.96	1.01
Iceland	60 to 64	1.01	1.02	1.03	1.03	0.89	0.93	0.85	0.91
India	60 to 64	0.89	0.93	1.28	1.14	0.94	0.84	0.65	0.80
Indonesia	60 to 64	1.03	1.17	1.26	1.30	0.90	0.91	0.97	1.09
Iran (Islamic Republic of)	60 to 64	1.07	1.02	1.23	1.09	1.07	1.04	0.81	0.88
Iraq	60 to 64	1.23	1.20	1.59	1.33	1.13	1.04	0.88	1.03
Ireland	60 to 64	1.05	1.03	1.08	1.05	0.89	1.00	0.91	0.93
Israel	60 to 64	0.96	1.04	1.17	1.18	0.95	1.03	0.82	0.94
Italy	60 to 64	1.07	1.06	1.25	1.11	0.92	0.99	0.90	0.94
Jamaica	60 to 64	1.16	1.10	1.46	1.29	0.88	0.93	0.85	0.91
Japan	60 to 64	1.03	1.01	1.09	1.02	0.93	0.98	0.90	0.94
Jordan	60 to 64	0.89	0.96	1.06	1.04	0.99	1.02	0.61	0.80
Kazakhstan	60 to 64	1.14	1.20	1.21	1.29	1.03	0.99	1.06	1.08
Kenya	60 to 64	1.17	1.24	1.34	1.39	1.01	1.02	0.91	0.98
Kiribati	60 to 64	1.30	1.30	1.21	1.24	1.06	0.96	1.09	1.17
Kuwait	60 to 64	0.96	1.10	1.05	1.10	0.95	1.06	0.76	0.98
Kyrgyzstan	60 to 64	1.18	1.22	1.16	1.21	1.16	1.17	1.12	1.17
Lao People's Democratic Republic	60 to 64	0.87	1.07	1.49	1.25	0.79	0.94	0.86	1.01
Latvia	60 to 64	1.15	1.11	1.32	1.27	1.14	1.03	0.94	0.95
Lebanon	60 to 64	1.15	1.03	2.52	1.14	1.28	1.06	0.80	0.90
Lesotho	60 to 64	2.45	10.20	2.39	4.20	1.13	1.12	1.26	2.63
Liberia	60 to 64	1.03	1.07	0.93	0.92	0.91	0.90	1.43	1.77
Libya	60 to 64	0.91	0.95	1.02	0.97	0.92	0.89	0.70	0.80
Lithuania	60 to 64	1.13	1.14	1.18	1.18	1.19	1.13	0.90	0.97
Luxembourg	60 to 64	1.10	1.07	1.26	1.13	0.91	0.96	0.96	0.94
Madagascar	60 to 64	1.55	1.23	1.92	1.56	1.06	0.96	1.05	0.99
Malawi	60 to 64	1.11	1.26	1.53	2.04	0.98	0.99	0.90	1.04
Malaysia	60 to 64	1.12	1.07	1.26	1.14	0.98	0.96	0.93	0.94
Maldives	60 to 64	0.86	1.03	1.04	1.01	0.88	0.97	0.82	0.93
Mali	60 to 64	0.89	0.95	0.87	0.86	0.87	0.87	1.06	1.23
Malta	60 to 64	1.04	1.02	1.14	1.01	0.94	0.97	0.83	0.91
Marshall Islands	60 to 64	2.42	1.32	2.12	1.46	0.96	0.84	1.15	0.97
Mauritania	60 to 64	1.01	1.05	0.80	0.89	0.88	0.91	1.51	1.44
Mauritius	60 to 64	1.47	1.19	1.58	1.18	1.27	1.12	1.46	1.07

Mexico	60 to 64	1.10	1.11	1.23	1.22	0.97	0.98	0.99	1.03
Micronesia (Federated States of)	60 to 64	1.33	1.32	1.45	1.44	0.84	0.87	0.97	1.01
Monaco	60 to 64	1.02	1.01	1.13	1.05	0.86	0.93	0.87	0.90
Mongolia	60 to 64	1.61	1.44	1.77	1.42	1.20	1.11	1.00	1.08
Montenegro	60 to 64	1.09	1.16	1.38	1.54	0.94	0.92	0.93	0.96
Morocco	60 to 64	0.73	0.86	0.98	0.99	0.88	0.88	0.35	0.53
Mozambique	60 to 64	1.92	2.42	3.47	5.88	1.20	1.23	1.17	1.53
Myanmar	60 to 64	1.09	1.35	1.47	1.48	1.00	1.02	1.09	1.15
Namibia	60 to 64	1.34	1.50	1.23	1.53	1.02	1.03	1.29	1.36
Nauru	60 to 64	1.31	1.41	1.54	1.85	0.70	0.75	1.13	1.12
Nepal	60 to 64	0.51	0.96	1.11	1.20	0.73	0.94	0.19	0.78
Netherlands	60 to 64	1.05	1.03	1.15	1.05	0.98	1.00	0.92	0.93
New Zealand	60 to 64	1.01	1.01	1.11	1.05	0.83	1.03	0.90	0.93
Nicaragua	60 to 64	1.13	1.15	1.25	1.25	0.97	0.96	0.97	1.00
Niger	60 to 64	1.21	1.15	1.18	1.11	1.02	0.99	1.17	1.28
Nigeria	60 to 64	1.28	1.20	1.16	1.08	1.07	1.02	1.56	1.54
Niue	60 to 64	1.24	1.25	1.37	1.39	0.87	0.88	0.99	1.01
North Macedonia	60 to 64	0.96	1.03	1.21	1.26	0.78	0.76	0.76	0.85
Northern Mariana Islands	60 to 64	0.95	1.10	1.05	1.14	0.62	0.78	0.89	0.96
Norway	60 to 64	1.01	1.04	1.13	1.04	0.87	1.06	0.87	0.92
Oman	60 to 64	1.08	0.98	1.44	1.06	1.07	1.00	0.83	0.84
Pakistan	60 to 64	0.85	0.89	1.08	1.07	0.85	0.81	0.70	0.80
Palau	60 to 64	1.78	1.63	1.79	1.75	0.96	0.97	1.24	1.26
Palestine	60 to 64	1.06	1.12	1.41	1.36	1.02	1.03	0.73	0.94
Panama	60 to 64	1.20	1.15	1.32	1.23	0.95	0.99	1.03	1.00
Papua New Guinea	60 to 64	0.00	0.45	0.78	0.88	0.45	0.59	0.51	0.62
Paraguay	60 to 64	1.07	1.12	1.38	1.42	0.83	0.80	1.01	1.05
Peru	60 to 64	1.15	1.05	1.30	1.14	0.96	0.89	1.04	1.01
Philippines	60 to 64	1.34	1.33	1.59	1.43	1.03	1.00	1.13	1.10
Poland	60 to 64	1.19	1.19	1.36	1.34	1.05	1.05	1.05	1.03
Portugal	60 to 64	1.12	1.08	1.31	1.22	0.97	1.02	0.95	0.96
Puerto Rico	60 to 64	1.19	1.15	1.23	1.24	1.02	1.05	1.07	1.04
Qatar	60 to 64	0.98	0.94	1.17	0.95	1.65	1.12	0.84	0.89
Republic of Korea	60 to 64	0.99	1.01	1.11	1.01	0.91	0.96	0.84	0.93
Republic of Moldova	60 to 64	1.04	1.16	1.30	1.26	1.07	1.10	0.88	1.01
Romania	60 to 64	1.04	1.17	1.32	1.60	0.93	1.02	0.87	1.00
Russian Federation	60 to 64	1.25	1.24	1.26	1.30	1.09	1.12	1.20	1.16
Rwanda	60 to 64	2.08	1.39	3.73	2.21	1.15	1.06	0.90	0.96
Saint Kitts and Nevis	60 to 64	1.14	1.57	1.39	1.97	0.93	1.04	0.94	1.25
Saint Lucia	60 to 64	1.74	1.32	2.43	1.56	0.77	1.02	1.02	0.99
Saint Vincent and the Grenadines	60 to 64	1.08	1.18	1.41	1.37	0.89	0.98	0.83	0.92
Samoa	60 to 64	1.41	1.17	1.55	1.32	0.91	0.83	1.00	1.00
San Marino	60 to 64	1.02	1.02	1.07	1.02	0.97	0.98	0.91	0.92
Sao Tome and Principe	60 to 64	1.05	1.08	0.99	0.99	0.95	0.93	1.22	1.32
Saudi Arabia	60 to 64	0.77	0.95	1.26	1.20	0.94	0.96	0.29	0.64
Senegal	60 to 64	1.23	1.23	1.07	1.05	0.99	0.95	1.71	1.79
Serbia	60 to 64	1.08	1.08	1.33	1.39	0.78	0.76	0.88	0.90
Seychelles	60 to 64	1.30	1.09	1.34	1.11	1.07	1.01	1.20	1.00

Sierra Leone	60 to 64	1.28	1.12	1.07	0.98	0.99	0.92	2.01	1.82
Singapore	60 to 64	0.94	0.96	1.03	0.98	0.88	0.94	0.77	0.89
Slovakia	60 to 64	1.10	1.08	1.55	1.33	0.96	1.00	0.92	0.92
Slovenia	60 to 64	1.21	1.09	1.39	1.14	1.00	0.99	1.02	0.94
Solomon Islands	60 to 64	1.25	1.25	1.32	1.32	0.88	0.89	0.90	0.92
Somalia	60 to 64	2.33	1.72	5.87	6.18	1.23	1.13	0.93	0.96
South Africa	60 to 64	1.11	1.21	1.11	1.26	1.01	0.99	1.05	1.12
South Sudan	60 to 64	1.56	1.42	2.34	2.57	1.06	1.02	0.98	0.98
Spain	60 to 64	1.09	1.05	1.16	1.12	1.02	1.01	0.88	0.91
Sri Lanka	60 to 64	1.19	1.15	1.43	1.32	1.05	1.04	1.02	1.01
Sudan	60 to 64	0.55	0.82	1.20	0.96	0.89	0.85	0.11	0.58
Suriname	60 to 64	1.05	1.48	1.30	1.79	0.94	1.05	0.93	1.15
Sweden	60 to 64	1.04	1.02	1.08	0.99	0.88	0.93	0.91	0.95
Switzerland	60 to 64	1.05	1.02	1.10	1.03	0.94	0.97	0.92	0.92
Syrian Arab Republic	60 to 64	0.98	1.04	1.14	1.21	1.02	1.02	0.76	0.84
Taiwan (Province of China)	60 to 64	1.14	1.14	1.25	1.15	0.98	1.05	0.92	0.96
Tajikistan	60 to 64	1.11	1.03	1.15	1.08	0.87	0.89	1.18	0.90
Thailand	60 to 64	1.20	1.14	1.20	1.14	1.23	1.10	1.01	1.02
Timor-Leste	60 to 64	0.67	1.01	1.02	1.20	0.76	0.86	0.86	1.03
Togo	60 to 64	1.11	1.32	1.00	1.15	0.96	0.99	1.53	2.13
Tokelau	60 to 64	1.50	1.25	1.91	1.40	0.79	0.86	1.05	1.03
Tonga	60 to 64	1.10	1.13	1.19	1.19	0.86	0.87	0.99	0.99
Trinidad and Tobago	60 to 64	1.15	1.30	1.49	1.64	0.99	1.11	1.27	1.20
Tunisia	60 to 64	0.92	1.01	1.15	1.10	1.02	1.01	0.65	0.84
Turkey	60 to 64	1.09	1.08	1.22	1.16	1.25	1.10	0.94	0.95
Turkmenistan	60 to 64	1.12	1.23	1.17	1.38	0.98	1.04	0.98	1.00
Tuvalu	60 to 64	1.49	1.23	1.65	1.39	0.76	0.81	1.02	1.01
Uganda	60 to 64	1.61	1.34	2.81	1.82	1.14	1.05	0.96	0.96
Ukraine	60 to 64	1.09	1.16	1.22	1.27	1.10	1.14	0.91	1.04
United Arab Emirates	60 to 64	0.90	1.02	1.19	1.04	0.48	0.97	0.65	0.87
United Kingdom	60 to 64	1.03	1.03	1.10	1.05	0.82	0.94	0.91	0.93
United Republic of Tanzania	60 to 64	1.17	1.02	1.48	1.17	1.01	0.95	0.89	0.92
United States Virgin Islands	60 to 64	1.34	1.24	1.70	1.39	1.04	1.07	1.05	1.01
United States of America	60 to 64	1.02	1.00	1.10	1.06	0.95	1.07	0.91	1.00
Uruguay	60 to 64	1.06	1.06	1.26	1.21	1.02	0.96	0.94	0.96
Uzbekistan	60 to 64	1.14	1.12	1.20	1.15	1.02	1.15	1.07	0.98
Vanuatu	60 to 64	1.42	1.29	1.57	1.41	0.87	0.87	1.09	1.04
Venezuela (Bolivarian Republic of)	60 to 64	1.21	1.27	1.37	1.50	1.01	0.99	1.02	1.06
Viet Nam	60 to 64	1.58	1.44	1.67	1.67	1.17	1.12	1.24	1.19
Yemen	60 to 64	0.77	0.90	2.59	1.29	1.39	1.04	0.05	0.56
Zambia	60 to 64	1.40	1.35	1.68	1.48	1.01	0.96	0.93	0.99
Zimbabwe	60 to 64	1.17	1.59	0.97	1.01	0.97	0.98	1.05	1.46
Afghanistan	65 to 69	1.15	0.87	3.02	1.16	1.86	1.16	0.26	0.59
Albania	65 to 69	1.47	1.12	1.39	1.08	1.06	1.01	1.11	1.00
Algeria	65 to 69	0.98	0.97	0.97	0.93	1.07	1.03	0.88	0.88
American Samoa	65 to 69	1.20	1.14	1.04	1.03	0.89	0.88	1.01	0.98
Andorra	65 to 69	1.01	0.98	0.97	0.97	0.88	0.93	0.95	0.95
Angola	65 to 69	1.78	1.46	1.21	1.15	1.09	1.04	1.24	1.23

Antigua and Barbuda	65 to 69	1.81	1.58	1.31	1.52	1.19	1.04	1.26	1.19
Argentina	65 to 69	1.14	1.09	1.16	1.14	1.03	1.02	1.04	1.00
Armenia	65 to 69	1.13	1.09	1.00	1.08	0.95	1.06	1.08	1.04
Australia	65 to 69	1.01	1.02	0.99	1.01	0.99	1.06	0.96	0.97
Austria	65 to 69	1.07	1.01	1.01	1.00	1.00	1.02	0.99	0.97
Azerbaijan	65 to 69	1.33	1.13	1.05	0.94	1.06	1.03	1.16	1.05
Bahamas	65 to 69	1.52	1.17	1.29	1.10	1.06	1.02	1.14	1.00
Bahrain	65 to 69	1.02	0.94	1.12	1.00	0.85	0.97	0.89	0.87
Bangladesh	65 to 69	1.11	0.94	1.17	1.03	1.09	0.84	0.78	0.86
Barbados	65 to 69	1.52	1.14	1.29	1.06	1.12	0.97	1.28	1.02
Belarus	65 to 69	1.06	1.21	0.99	1.12	1.17	1.29	0.96	1.20
Belgium	65 to 69	1.11	1.07	1.08	1.09	0.97	1.05	1.03	1.00
Belize	65 to 69	1.26	1.25	1.11	1.10	1.07	1.04	1.07	1.11
Benin	65 to 69	1.12	1.18	0.64	0.75	0.91	0.93	1.91	1.94
Bermuda	65 to 69	1.31	1.26	1.18	1.22	1.15	1.19	1.28	1.17
Bhutan	65 to 69	0.84	1.00	1.00	0.99	0.86	0.90	0.74	0.94
Bolivia (Plurinational State of)	65 to 69	1.23	1.14	1.05	1.08	0.74	0.74	1.28	1.16
Bosnia and Herzegovina	65 to 69	0.98	1.09	0.91	1.13	0.82	1.02	0.82	1.01
Botswana	65 to 69	1.47	1.28	0.91	0.98	1.05	1.03	1.34	1.23
Brazil	65 to 69	1.26	1.16	1.13	1.13	0.90	0.85	1.40	1.15
Brunei Darussalam	65 to 69	0.99	0.94	1.01	0.96	0.86	0.93	0.91	0.89
Bulgaria	65 to 69	1.14	1.28	1.07	1.14	0.98	1.07	1.06	1.31
Burkina Faso	65 to 69	1.52	1.46	0.98	0.96	1.13	1.09	1.55	1.64
Burundi	65 to 69	6.30	1.82	4.41	1.98	1.24	1.12	1.16	1.11
Cabo Verde	65 to 69	0.95	1.39	0.83	1.00	1.00	1.05	1.16	2.04
Cambodia	65 to 69	1.03	1.27	0.84	1.02	0.97	1.00	1.11	1.12
Cameroon	65 to 69	1.24	1.24	0.87	0.84	0.98	0.98	1.41	1.71
Canada	65 to 69	1.07	0.99	1.04	1.01	1.04	1.03	0.99	0.96
Central African Republic	65 to 69	4.66	3.55	1.55	1.77	1.14	1.11	1.41	1.43
Chad	65 to 69	1.15	1.30	0.74	0.75	0.97	0.92	1.57	2.10
Chile	65 to 69	1.13	1.07	1.15	1.07	0.98	1.02	1.05	1.02
China	65 to 69	1.57	1.22	1.29	1.21	1.01	1.10	1.14	1.06
Colombia	65 to 69	1.15	1.14	1.10	1.12	0.96	0.97	1.11	1.11
Comoros	65 to 69	1.66	1.36	1.21	1.16	1.07	1.05	1.15	1.12
Congo	65 to 69	1.52	1.24	0.94	0.86	1.01	0.97	1.26	1.17
Cook Islands	65 to 69	1.31	1.17	1.15	1.14	0.96	0.97	1.05	1.00
Costa Rica	65 to 69	1.15	1.13	1.09	1.10	1.01	0.99	1.07	1.06
Coted'Ivoire	65 to 69	1.24	1.23	0.87	0.84	0.99	0.97	1.59	1.88
Croatia	65 to 69	1.05	1.09	1.11	1.30	0.76	1.02	0.98	0.99
Cuba	65 to 69	1.16	1.24	1.10	1.14	1.00	1.04	1.05	1.22
Cyprus	65 to 69	1.09	1.06	1.08	1.02	0.98	0.99	0.97	1.01
Czechia	65 to 69	1.17	1.08	1.18	1.09	0.84	1.08	1.13	1.00
Democratic People's Republic of Korea	65 to 69	1.38	1.39	1.29	1.35	1.19	1.15	1.03	1.10
Democratic Republic of the Congo	65 to 69	1.40	1.56	0.99	1.16	1.02	1.04	1.12	1.19
Denmark	65 to 69	1.04	1.07	1.02	1.07	0.94	1.01	0.97	1.01
Djibouti	65 to 69	1.66	1.47	1.36	1.28	1.11	1.08	1.12	1.21
Dominica	65 to 69	2.44	1.56	1.60	1.36	1.22	1.13	1.57	1.28
Dominican Republic	65 to 69	1.28	1.30	1.17	1.12	1.03	1.02	1.10	1.16

Ecuador	65 to 69	1.22	1.11	1.12	1.10	1.00	0.96	1.19	1.07
Egypt	65 to 69	2.36	0.99	1.01	1.07	1.87	1.08	1.01	0.75
El Salvador	65 to 69	1.21	1.18	1.12	1.16	0.98	0.97	1.16	1.13
Equatorial Guinea	65 to 69	2.45	1.28	1.32	0.99	1.13	1.00	1.35	1.23
Eritrea	65 to 69	4.96	2.18	2.40	2.22	1.26	1.16	1.13	1.21
Estonia	65 to 69	1.13	1.20	1.03	1.11	1.29	1.27	1.06	1.15
Eswatini	65 to 69	2.31	1.70	1.19	0.94	1.09	1.04	1.37	1.37
Ethiopia	65 to 69	5.31	1.60	1.99	1.36	1.21	1.08	1.10	1.06
Fiji	65 to 69	1.19	1.14	1.10	1.10	0.94	0.91	1.01	0.99
Finland	65 to 69	1.01	1.06	0.99	1.04	0.90	1.01	0.96	1.01
France	65 to 69	1.13	1.03	1.13	1.04	0.99	1.05	1.06	0.97
Gabon	65 to 69	1.54	1.34	1.18	1.09	1.09	1.05	1.15	1.15
Gambia	65 to 69	1.25	1.31	0.76	0.77	0.97	0.93	1.80	2.30
Georgia	65 to 69	1.80	1.39	1.13	1.12	1.06	1.00	1.34	1.31
Germany	65 to 69	1.07	1.03	1.08	1.04	0.98	1.01	1.01	0.97
Ghana	65 to 69	1.17	1.27	0.69	0.79	0.95	0.94	1.77	1.87
Greece	65 to 69	1.01	1.07	1.02	1.06	0.96	1.05	0.96	0.99
Greenland	65 to 69	1.03	1.07	1.07	1.07	0.49	0.99	1.02	1.00
Grenada	65 to 69	1.47	1.19	1.14	1.07	1.00	0.98	1.30	1.05
Guam	65 to 69	1.14	1.04	1.04	0.97	0.90	0.89	1.04	0.98
Guatemala	65 to 69	1.13	1.16	1.03	1.10	1.03	1.04	1.06	1.08
Guinea	65 to 69	0.93	1.07	0.63	0.70	0.91	0.90	1.38	1.76
Guinea-Bissau	65 to 69	2.16	1.53	0.72	0.67	1.01	0.91	5.44	4.30
Guyana	65 to 69	2.51	1.80	1.17	1.29	1.07	1.13	2.60	1.63
Haiti	65 to 69	1.01	1.11	1.22	1.07	0.72	0.71	0.57	0.84
Honduras	65 to 69	1.34	1.46	1.23	1.35	0.93	0.63	1.43	1.78
Hungary	65 to 69	1.14	1.14	1.08	1.14	1.06	1.05	1.07	1.09
Iceland	65 to 69	1.05	1.04	1.02	1.04	0.94	1.01	0.97	0.98
India	65 to 69	1.05	0.99	1.10	1.02	1.05	0.87	0.88	0.93
Indonesia	65 to 69	1.09	1.28	1.01	1.03	0.93	0.94	1.09	1.25
Iran (Islamic Republic of)	65 to 69	1.13	1.01	1.11	1.01	1.12	1.05	1.04	0.94
Iraq	65 to 69	1.46	1.33	1.27	1.18	1.17	1.08	1.66	1.41
Ireland	65 to 69	1.07	1.02	1.00	1.01	0.94	1.00	1.03	0.98
Israel	65 to 69	0.98	1.05	1.05	1.08	1.00	1.07	0.91	0.99
Italy	65 to 69	1.10	1.05	1.10	1.05	0.94	1.03	1.01	0.98
Jamaica	65 to 69	1.37	1.27	1.06	1.10	0.92	0.99	1.15	1.09
Japan	65 to 69	1.03	1.01	1.02	1.00	0.96	1.00	0.97	0.98
Jordan	65 to 69	0.91	0.98	0.92	1.00	1.02	1.03	0.73	0.89
Kazakhstan	65 to 69	1.22	1.31	1.02	1.13	1.01	0.99	1.23	1.27
Kenya	65 to 69	1.31	1.42	1.18	1.21	1.06	1.06	1.00	1.09
Kiribati	65 to 69	1.42	1.40	1.04	1.06	1.12	1.03	1.14	1.19
Kuwait	65 to 69	0.90	1.09	0.92	1.02	0.94	1.07	0.74	1.02
Kyrgyzstan	65 to 69	1.36	1.40	0.98	1.06	1.21	1.25	1.36	1.59
Lao People's Democratic Republic	65 to 69	1.17	1.19	0.90	0.96	0.88	0.97	1.18	1.22
Latvia	65 to 69	1.17	1.14	1.08	1.12	1.18	1.15	1.09	1.10
Lebanon	65 to 69	1.21	1.05	1.78	1.09	1.30	1.10	0.94	0.96
Lesotho	65 to 69	4.50	128.63	1.62	1.64	1.18	1.14	1.50	2.51
Liberia	65 to 69	0.98	1.03	0.64	0.60	0.92	0.89	1.48	1.83

Libya	65 to 69	0.93	0.98	0.93	0.88	0.97	0.92	0.79	0.92
Lithuania	65 to 69	1.20	1.19	1.05	1.05	1.17	1.24	1.14	1.15
Luxembourg	65 to 69	1.13	1.05	1.13	1.08	0.93	1.01	1.09	0.98
Madagascar	65 to 69	2.61	1.72	1.72	1.42	1.15	1.05	1.22	1.17
Malawi	65 to 69	1.47	1.64	1.21	1.57	1.07	1.07	1.10	1.28
Malaysia	65 to 69	1.11	1.12	0.99	1.03	0.98	1.01	1.00	1.02
Maldives	65 to 69	0.90	1.06	0.77	0.92	0.92	1.00	0.95	1.01
Mali	65 to 69	0.85	0.88	0.57	0.59	0.87	0.86	1.14	1.25
Malta	65 to 69	1.08	1.05	1.02	1.01	0.99	1.03	1.00	0.98
Marshall Islands	65 to 69	4.32	1.46	1.60	1.14	1.09	0.95	1.24	1.04
Mauritania	65 to 69	0.98	1.02	0.55	0.67	0.89	0.91	1.53	1.46
Mauritius	65 to 69	1.33	1.26	1.08	1.09	1.25	1.16	1.34	1.15
Mexico	65 to 69	1.16	1.15	1.11	1.11	0.99	1.01	1.12	1.11
Micronesia (Federated States of)	65 to 69	1.50	1.41	1.12	1.12	0.96	0.96	1.05	1.08
Monaco	65 to 69	1.06	1.01	1.07	1.01	0.91	0.96	0.97	0.95
Mongolia	65 to 69	2.12	1.62	1.12	1.22	1.09	0.94	1.06	1.12
Montenegro	65 to 69	1.15	1.28	1.18	1.32	0.94	0.94	1.01	1.06
Morocco	65 to 69	0.73	0.89	0.82	0.86	0.95	0.95	0.47	0.69
Mozambique	65 to 69	2.59	3.40	1.98	3.11	1.20	1.22	1.34	1.79
Myanmar	65 to 69	1.66	1.58	0.91	1.14	1.08	1.06	1.48	1.33
Namibia	65 to 69	1.56	1.61	0.93	1.13	1.05	1.06	1.40	1.42
Nauru	65 to 69	1.45	1.77	1.26	2.16	0.85	0.85	1.19	1.18
Nepal	65 to 69	0.63	1.12	0.99	1.09	0.89	1.05	0.50	1.04
Netherlands	65 to 69	1.08	1.03	1.08	1.02	1.01	1.03	1.00	0.98
New Zealand	65 to 69	1.09	1.03	1.07	1.04	0.97	1.06	1.03	0.99
Nicaragua	65 to 69	1.20	1.15	1.10	1.07	1.00	0.97	1.11	1.07
Niger	65 to 69	1.23	1.17	0.84	0.80	0.99	0.99	1.29	1.40
Nigeria	65 to 69	1.32	1.20	0.84	0.90	1.03	1.00	1.79	1.59
Niue	65 to 69	1.33	1.30	1.15	1.18	0.96	0.95	1.05	1.07
North Macedonia	65 to 69	1.02	1.07	0.94	0.97	0.72	0.72	0.97	1.00
Northern Mariana Islands	65 to 69	1.01	1.19	0.91	1.05	0.74	0.88	0.97	1.04
Norway	65 to 69	1.04	1.05	1.08	1.00	0.90	1.06	0.97	0.98
Oman	65 to 69	1.16	1.01	1.33	1.04	1.10	1.03	1.07	0.93
Pakistan	65 to 69	0.93	0.98	0.99	0.98	0.93	0.87	0.87	0.98
Palau	65 to 69	1.74	1.26	1.35	1.13	0.97	0.90	1.21	1.06
Palestine	65 to 69	1.04	1.15	1.11	1.15	1.04	1.06	0.74	1.06
Panama	65 to 69	1.29	1.21	1.17	1.16	0.97	1.01	1.15	1.09
Papua New Guinea	65 to 69	0.16	0.52	0.46	0.55	0.69	0.75	0.73	0.79
Paraguay	65 to 69	1.15	1.17	1.08	1.15	0.87	0.78	1.19	1.18
Peru	65 to 69	1.24	1.06	1.15	1.06	0.98	0.89	1.15	1.05
Philippines	65 to 69	1.37	1.43	1.14	1.18	1.06	1.03	1.20	1.21
Poland	65 to 69	1.25	1.21	1.15	1.22	1.06	1.05	1.36	1.15
Portugal	65 to 69	1.16	1.08	1.12	1.10	0.97	1.04	1.10	1.00
Puerto Rico	65 to 69	1.21	1.22	1.11	1.16	1.03	1.07	1.10	1.13
Qatar	65 to 69	0.96	0.92	0.97	0.91	1.40	1.08	0.89	0.90
Republic of Korea	65 to 69	1.04	0.99	1.08	0.99	0.96	0.99	0.97	0.97
Republic of Moldova	65 to 69	1.09	1.17	0.99	1.07	1.18	1.15	1.00	1.07
Romania	65 to 69	1.08	1.18	0.94	1.23	0.90	1.05	1.03	1.10

Russian Federation	65 to 69	1.46	1.29	1.07	1.13	1.17	1.17	2.31	1.35
Rwanda	65 to 69	6.96	1.79	2.98	1.84	1.22	1.11	1.14	1.13
Saint Kitts and Nevis	65 to 69	1.20	1.60	1.07	1.33	0.95	1.02	1.06	1.39
Saint Lucia	65 to 69	1.44	1.20	1.14	1.08	0.93	0.96	1.13	0.98
Saint Vincent and the Grenadines	65 to 69	1.06	1.36	0.87	1.21	0.93	1.08	0.88	1.14
Samoa	65 to 69	1.55	1.23	1.26	1.11	1.00	0.91	1.06	1.05
San Marino	65 to 69	1.02	1.01	1.00	0.98	1.00	1.02	0.98	0.97
Sao Tome and Principe	65 to 69	1.07	1.08	0.83	0.82	0.95	0.93	1.30	1.35
Saudi Arabia	65 to 69	0.80	0.98	1.00	1.04	0.98	1.00	0.46	0.80
Senegal	65 to 69	1.23	1.24	0.75	0.77	0.96	0.94	1.77	1.85
Serbia	65 to 69	1.07	1.13	0.96	1.20	0.74	0.78	1.02	1.09
Seychelles	65 to 69	1.33	1.16	1.12	1.06	1.10	1.06	1.28	1.13
Sierra Leone	65 to 69	1.22	1.06	0.75	0.68	0.98	0.91	1.83	1.72
Singapore	65 to 69	0.93	0.95	0.95	0.97	0.92	0.96	0.86	0.92
Slovakia	65 to 69	1.09	1.10	1.16	1.16	0.97	1.04	1.03	1.02
Slovenia	65 to 69	1.26	1.12	1.15	1.13	1.01	1.03	1.24	1.03
Solomon Islands	65 to 69	1.31	1.30	1.07	1.09	0.96	0.96	0.98	0.99
Somalia	65 to 69	7.30	3.22	5.47	20.03	1.28	1.22	1.08	1.13
South Africa	65 to 69	1.16	1.31	0.97	1.06	1.04	1.02	1.11	1.21
South Sudan	65 to 69	2.50	2.01	2.29	2.07	1.12	1.10	1.14	1.17
Spain	65 to 69	1.12	1.04	1.07	1.06	1.04	1.07	1.01	0.97
Sri Lanka	65 to 69	1.27	1.19	1.20	1.14	1.08	1.07	1.16	1.10
Sudan	65 to 69	0.54	0.83	0.92	0.81	0.99	0.91	0.24	0.70
Suriname	65 to 69	1.41	1.48	1.15	1.19	1.02	1.04	1.25	1.24
Sweden	65 to 69	1.06	1.03	1.01	0.97	0.90	0.99	0.99	0.99
Switzerland	65 to 69	1.08	1.00	1.05	1.02	0.97	1.01	1.03	0.96
Syrian Arab Republic	65 to 69	1.05	1.05	1.02	1.05	1.05	1.04	0.89	0.91
Taiwan (Province of China)	65 to 69	1.12	1.18	1.09	1.10	0.98	1.10	1.00	1.04
Tajikistan	65 to 69	1.33	1.11	0.96	0.87	0.95	0.92	1.55	1.08
Thailand	65 to 69	1.23	1.16	1.02	1.04	1.23	1.11	1.10	1.06
Timor-Leste	65 to 69	0.79	1.11	0.70	0.88	0.83	0.91	1.03	1.19
Togo	65 to 69	1.08	1.35	0.69	0.77	0.95	0.96	1.60	2.38
Tokelau	65 to 69	1.77	1.32	1.37	1.16	0.93	0.93	1.13	1.07
Tonga	65 to 69	1.12	1.15	1.01	1.04	0.93	0.93	1.05	1.04
Trinidad and Tobago	65 to 69	1.24	1.30	1.15	1.22	0.97	1.05	1.40	1.28
Tunisia	65 to 69	0.96	1.04	1.02	1.01	1.06	1.04	0.79	0.96
Turkey	65 to 69	1.18	1.11	1.14	1.08	1.31	1.12	1.10	1.03
Turkmenistan	65 to 69	1.29	1.47	0.99	1.18	1.04	1.16	1.27	1.31
Tuvalu	65 to 69	1.70	1.33	1.11	1.11	0.92	0.91	1.11	1.08
Uganda	65 to 69	2.15	1.60	2.42	1.67	1.20	1.11	1.09	1.09
Ukraine	65 to 69	1.14	1.20	1.07	1.13	1.14	1.17	1.10	1.17
United Arab Emirates	65 to 69	0.82	1.00	0.91	0.93	0.48	0.98	0.66	0.92
United Kingdom	65 to 69	1.07	1.03	1.02	1.01	0.84	1.00	1.04	0.99
United Republic of Tanzania	65 to 69	1.54	1.21	1.41	1.18	1.10	1.03	1.02	1.09
United States Virgin Islands	65 to 69	1.51	1.24	1.37	1.15	1.07	1.11	1.23	1.08
United States of America	65 to 69	1.06	1.00	1.03	1.02	1.02	1.09	1.00	1.00
Uruguay	65 to 69	1.07	1.08	1.15	1.14	1.02	0.99	1.02	1.04
Uzbekistan	65 to 69	1.22	1.17	1.00	1.00	1.08	1.20	1.16	1.09

Vanuatu	65 to 69	1.54	1.37	1.23	1.17	0.97	0.95	1.15	1.09
Venezuela (Bolivarian Republic of)	65 to 69	1.26	1.34	1.17	1.30	1.01	1.00	1.12	1.16
Viet Nam	65 to 69	1.78	1.58	1.20	1.32	1.18	1.14	1.39	1.38
Yemen	65 to 69	0.94	0.97	1.81	0.91	1.47	1.10	0.27	0.84
Zambia	65 to 69	1.88	1.71	1.18	1.15	1.07	1.03	1.07	1.17
Zimbabwe	65 to 69	1.40	2.00	0.87	0.00	1.03	1.03	1.20	1.56
Afghanistan	70 to 74	3.46	1.37	6.82	1.75	2.18	1.42	1.22	1.28
Albania	70 to 74	1.93	1.31	1.78	1.27	1.20	1.08	1.29	1.12
Algeria	70 to 74	1.16	1.10	1.12	1.04	1.13	1.08	1.12	1.06
American Samoa	70 to 74	1.37	1.33	1.21	1.23	0.95	0.95	1.12	1.10
Andorra	70 to 74	1.07	1.03	1.02	1.01	1.01	0.99	1.03	1.00
Angola	70 to 74	2.09	1.70	1.58	1.46	1.15	1.10	1.43	1.40
Antigua and Barbuda	70 to 74	2.30	1.76	1.58	1.67	1.23	1.10	1.55	1.35
Argentina	70 to 74	1.27	1.18	1.21	1.19	1.15	1.07	1.16	1.09
Armenia	70 to 74	1.31	1.21	1.14	1.15	1.12	1.14	1.27	1.19
Australia	70 to 74	1.12	1.07	1.07	1.04	1.07	1.07	1.08	1.02
Austria	70 to 74	1.15	1.06	1.04	1.02	1.02	1.02	1.12	1.02
Azerbaijan	70 to 74	1.61	1.35	1.21	1.11	1.10	1.08	1.36	1.22
Bahamas	70 to 74	1.72	1.27	1.48	1.21	1.13	1.11	1.31	1.11
Bahrain	70 to 74	1.36	1.13	1.31	1.13	1.15	1.07	1.32	1.09
Bangladesh	70 to 74	1.73	1.38	1.32	1.21	1.44	1.14	1.23	1.22
Barbados	70 to 74	1.53	1.34	1.30	1.25	1.15	1.09	1.36	1.23
Belarus	70 to 74	1.21	1.29	1.11	1.17	1.28	1.32	1.20	1.27
Belgium	70 to 74	1.21	1.12	1.12	1.11	1.05	1.14	1.16	1.06
Belize	70 to 74	1.33	1.37	1.18	1.21	1.12	1.07	1.15	1.22
Benin	70 to 74	1.52	1.47	0.86	0.91	1.04	1.03	1.94	1.89
Bermuda	70 to 74	1.51	1.41	1.26	1.30	1.16	1.18	1.51	1.32
Bhutan	70 to 74	1.13	1.19	1.11	1.08	1.15	1.09	0.99	1.14
Bolivia (Plurinational State of)	70 to 74	1.59	1.32	1.32	1.23	1.05	1.00	1.43	1.26
Bosnia and Herzegovina	70 to 74	1.20	1.18	1.31	1.24	1.22	1.12	1.16	1.15
Botswana	70 to 74	1.57	1.34	1.16	1.09	1.10	1.06	1.42	1.29
Brazil	70 to 74	1.49	1.27	1.25	1.19	1.03	1.07	1.52	1.22
Brunei Darussalam	70 to 74	1.08	1.06	1.05	1.02	1.02	1.00	1.04	1.02
Bulgaria	70 to 74	1.48	1.50	1.45	1.29	1.16	1.38	1.37	1.54
Burkina Faso	70 to 74	2.60	2.16	1.37	1.21	1.18	1.13	2.22	2.18
Burundi	70 to 74	3.12	1.74	3.70	2.01	1.23	1.13	1.29	1.20
Cabo Verde	70 to 74	1.27	1.71	0.96	1.08	1.05	1.06	1.47	2.28
Cambodia	70 to 74	1.59	1.66	1.13	1.28	1.17	1.12	1.28	1.33
Cameroon	70 to 74	1.73	1.66	1.10	1.02	1.09	1.06	1.71	1.93
Canada	70 to 74	1.13	1.04	1.06	1.02	1.08	1.06	1.08	1.01
Central African Republic	70 to 74	4.28	3.68	2.17	2.55	1.21	1.18	1.65	1.67
Chad	70 to 74	1.63	1.83	0.99	0.98	1.08	1.06	1.87	2.41
Chile	70 to 74	1.30	1.17	1.19	1.10	1.05	1.09	1.25	1.13
China	70 to 74	1.94	1.39	1.49	1.31	1.01	1.25	1.34	1.24
Colombia	70 to 74	1.27	1.24	1.18	1.18	1.06	1.07	1.22	1.19
Comoros	70 to 74	1.64	1.41	1.34	1.23	1.10	1.07	1.26	1.22
Congo	70 to 74	1.75	1.44	1.25	1.12	1.09	1.05	1.40	1.31
Cook Islands	70 to 74	1.52	1.25	1.36	1.23	0.99	0.97	1.18	1.09

Costa Rica	70 to 74	1.24	1.21	1.13	1.12	1.06	1.05	1.19	1.15
Coted'Ivoire	70 to 74	1.62	1.57	1.06	1.01	1.07	1.04	1.83	1.95
Croatia	70 to 74	1.25	1.16	1.40	1.45	1.13	1.28	1.17	1.09
Cuba	70 to 74	1.34	1.36	1.20	1.22	1.07	1.08	1.26	1.32
Cyprus	70 to 74	1.10	1.18	1.06	1.07	1.05	1.05	1.02	1.13
Czechia	70 to 74	1.36	1.12	1.31	1.14	1.18	1.17	1.39	1.07
Democratic People's Republic of Korea	70 to 74	1.56	1.54	1.53	1.57	1.55	1.38	1.21	1.25
Democratic Republic of the Congo	70 to 74	1.69	1.95	1.30	1.64	1.10	1.11	1.32	1.40
Denmark	70 to 74	1.14	1.12	1.08	1.09	1.05	1.08	1.10	1.08
Djibouti	70 to 74	1.64	1.48	1.47	1.32	1.12	1.08	1.21	1.28
Dominica	70 to 74	3.22	1.83	1.86	1.52	1.29	1.19	2.01	1.52
Dominican Republic	70 to 74	1.49	1.39	1.31	1.20	1.12	1.07	1.28	1.26
Ecuador	70 to 74	1.33	1.20	1.24	1.17	1.03	1.07	1.26	1.14
Egypt	70 to 74	20.18	1.13	1.01	1.27	1.99	1.19	1.01	1.10
El Salvador	70 to 74	1.39	1.26	1.21	1.19	1.06	1.04	1.31	1.21
Equatorial Guinea	70 to 74	2.60	1.43	1.71	1.15	1.18	1.04	1.55	1.33
Eritrea	70 to 74	2.55	1.94	2.31	2.20	1.22	1.14	1.22	1.29
Estonia	70 to 74	1.52	1.23	1.24	1.16	1.29	1.30	1.74	1.20
Eswatini	70 to 74	2.12	1.62	1.52	1.18	1.16	1.10	1.51	1.40
Ethiopia	70 to 74	2.45	1.52	1.70	1.37	1.17	1.09	1.16	1.15
Fiji	70 to 74	1.48	1.38	1.43	1.46	1.00	0.98	1.17	1.13
Finland	70 to 74	1.09	1.10	1.03	1.03	0.99	1.03	1.06	1.07
France	70 to 74	1.16	1.06	1.16	1.03	1.05	1.06	1.12	1.03
Gabon	70 to 74	1.75	1.50	1.44	1.29	1.12	1.08	1.30	1.28
Gambia	70 to 74	1.68	1.87	0.99	0.99	1.06	1.05	1.97	2.60
Georgia	70 to 74	2.17	1.71	1.29	1.26	1.20	1.12	1.67	1.62
Germany	70 to 74	1.15	1.07	1.11	1.05	1.05	1.05	1.12	1.04
Ghana	70 to 74	1.55	1.56	0.92	0.97	1.04	1.02	1.90	1.88
Greece	70 to 74	1.11	1.12	1.07	1.09	1.04	1.08	1.06	1.04
Greenland	70 to 74	1.14	1.15	1.14	1.13	1.15	1.12	1.09	1.06
Grenada	70 to 74	2.43	1.44	1.56	1.31	1.25	1.11	2.31	1.28
Guam	70 to 74	1.31	1.19	1.21	1.14	0.98	0.96	1.18	1.10
Guatemala	70 to 74	1.40	1.29	1.21	1.19	1.05	1.08	1.27	1.16
Guinea	70 to 74	1.37	1.47	0.88	0.89	1.04	1.02	1.62	1.95
Guinea-Bissau	70 to 74	3.73	2.49	1.05	0.97	1.15	1.07	6.04	4.44
Guyana	70 to 74	1.01	1.83	1.84	1.38	1.21	1.18	7.83	1.62
Haiti	70 to 74	10.47	2.00	1.75	1.51	1.30	1.19	1.14	1.25
Honduras	70 to 74	1.60	1.83	1.42	1.56	1.11	1.05	1.55	1.81
Hungary	70 to 74	1.29	1.21	1.27	1.23	1.14	1.15	1.25	1.17
Iceland	70 to 74	1.13	1.09	1.06	1.06	1.02	1.03	1.10	1.05
India	70 to 74	1.34	1.30	1.19	1.13	1.26	1.13	1.13	1.20
Indonesia	70 to 74	1.30	1.52	1.14	1.21	1.08	1.08	1.18	1.37
Iran (Islamic Republic of)	70 to 74	1.35	1.11	1.22	1.08	1.20	1.08	1.40	1.08
Iraq	70 to 74	2.03	1.63	1.56	1.37	1.26	1.16	2.19	1.74
Ireland	70 to 74	1.17	1.11	1.05	1.04	1.07	1.07	1.14	1.07
Israel	70 to 74	1.03	1.07	1.06	1.10	1.03	1.09	0.99	1.02
Italy	70 to 74	1.23	1.11	1.16	1.08	1.05	1.09	1.18	1.04
Jamaica	70 to 74	1.81	1.63	1.29	1.32	1.10	1.14	1.53	1.35

Japan	70 to 74	1.13	1.07	1.05	1.02	1.03	1.00	1.10	1.05
Jordan	70 to 74	1.06	1.05	1.07	1.09	1.05	1.04	1.02	1.01
Kazakhstan	70 to 74	1.44	1.59	1.14	1.33	1.16	1.21	1.47	1.55
Kenya	70 to 74	1.35	1.44	1.25	1.27	1.08	1.08	1.09	1.18
Kiribati	70 to 74	1.89	1.74	1.33	1.31	1.11	1.04	1.34	1.36
Kuwait	70 to 74	1.02	1.17	1.03	1.11	1.02	1.07	0.98	1.13
Kyrgyzstan	70 to 74	1.75	1.59	1.17	1.15	1.23	1.28	1.80	1.72
Lao People's Democratic Republic	70 to 74	1.88	1.48	1.26	1.16	1.21	1.09	1.37	1.36
Latvia	70 to 74	1.42	1.22	1.23	1.20	1.29	1.29	1.46	1.19
Lebanon	70 to 74	1.45	1.14	2.52	1.20	1.55	1.16	1.15	1.07
Lesotho	70 to 74	3.42	4.12	1.89	1.78	1.26	1.21	1.74	2.22
Liberia	70 to 74	1.43	1.49	0.88	0.87	1.03	1.02	1.76	2.02
Libya	70 to 74	1.16	1.17	1.12	1.04	1.09	1.05	1.12	1.18
Lithuania	70 to 74	1.30	1.28	1.09	1.14	1.26	1.27	1.32	1.26
Luxembourg	70 to 74	1.29	1.15	1.17	1.13	1.04	1.06	1.29	1.09
Madagascar	70 to 74	2.40	1.82	2.07	1.78	1.19	1.11	1.35	1.30
Malawi	70 to 74	1.49	1.62	1.38	1.67	1.10	1.09	1.20	1.34
Malaysia	70 to 74	1.34	1.24	1.16	1.13	1.11	1.07	1.15	1.12
Maldives	70 to 74	1.30	1.20	1.14	1.04	1.10	1.04	1.11	1.12
Mali	70 to 74	1.31	1.28	0.86	0.83	1.03	1.01	1.41	1.51
Malta	70 to 74	1.26	1.10	1.10	1.03	1.03	1.04	1.26	1.04
Marshall Islands	70 to 74	2.31	1.77	1.59	1.49	1.05	1.00	1.26	1.18
Mauritania	70 to 74	1.40	1.35	0.80	0.87	1.02	1.00	1.79	1.67
Mauritius	70 to 74	1.45	1.32	1.21	1.18	1.30	1.22	1.36	1.21
Mexico	70 to 74	1.27	1.22	1.14	1.14	1.04	1.07	1.26	1.18
Micronesia (Federated States of)	70 to 74	1.96	1.67	1.49	1.36	1.02	1.01	1.21	1.20
Monaco	70 to 74	1.15	1.07	1.13	1.05	1.04	1.01	1.10	1.03
Mongolia	70 to 74	3.34	1.59	1.69	1.16	1.89	1.36	1.22	1.17
Montenegro	70 to 74	1.36	1.48	1.38	1.60	1.08	1.06	1.12	1.17
Morocco	70 to 74	0.95	1.09	1.04	1.04	1.11	1.10	0.90	1.05
Mozambique	70 to 74	2.03	2.52	1.73	2.23	1.18	1.19	1.39	1.73
Myanmar	70 to 74	2.23	1.92	1.21	1.42	1.27	1.17	1.59	1.49
Namibia	70 to 74	1.62	1.62	1.18	1.29	1.10	1.09	1.48	1.48
Nauru	70 to 74	1.82	2.17	1.65	5.46	0.96	0.92	1.29	1.35
Nepal	70 to 74	1.07	1.38	1.14	1.18	1.22	1.19	0.89	1.28
Netherlands	70 to 74	1.15	1.08	1.12	1.06	1.10	1.07	1.09	1.04
New Zealand	70 to 74	1.19	1.08	1.13	1.08	1.08	1.06	1.15	1.04
Nicaragua	70 to 74	1.29	1.22	1.15	1.14	1.05	1.04	1.21	1.15
Niger	70 to 74	1.67	1.59	1.10	1.03	1.11	1.07	1.54	1.65
Nigeria	70 to 74	1.77	1.46	1.03	1.01	1.10	1.04	2.05	1.73
Niue	70 to 74	1.60	1.53	1.43	1.46	1.00	0.99	1.20	1.21
North Macedonia	70 to 74	1.20	1.27	1.14	1.19	1.15	1.09	1.17	1.24
Northern Mariana Islands	70 to 74	1.25	1.31	1.12	1.18	0.87	0.95	1.09	1.13
Norway	70 to 74	1.13	1.08	1.14	1.03	1.03	1.05	1.09	1.04
Oman	70 to 74	1.50	1.20	1.54	1.17	1.18	1.08	1.50	1.17
Pakistan	70 to 74	1.11	1.16	1.07	1.07	1.12	1.08	1.03	1.13
Palau	70 to 74	1.69	1.28	1.41	1.19	0.99	0.93	1.27	1.11
Palestine	70 to 74	1.61	1.27	1.34	1.24	1.09	1.06	1.99	1.26

Panama	70 to 74	1.36	1.35	1.20	1.23	1.05	1.09	1.25	1.24
Papua New Guinea	70 to 74	1.34	1.16	1.15	1.03	0.88	0.90	0.91	0.94
Paraguay	70 to 74	1.35	1.29	1.26	1.29	1.04	0.99	1.29	1.23
Peru	70 to 74	1.39	1.19	1.27	1.16	1.07	1.00	1.28	1.15
Philippines	70 to 74	1.54	1.65	1.30	1.33	1.12	1.11	1.28	1.37
Poland	70 to 74	1.56	1.24	1.32	1.25	1.16	1.14	1.71	1.19
Portugal	70 to 74	1.35	1.14	1.20	1.12	1.09	1.10	1.32	1.07
Puerto Rico	70 to 74	1.32	1.28	1.16	1.17	1.05	1.06	1.25	1.19
Qatar	70 to 74	1.27	1.07	1.51	1.05	3.28	1.17	1.16	1.03
Republic of Korea	70 to 74	1.17	1.04	1.15	1.01	1.10	1.01	1.11	1.02
Republic of Moldova	70 to 74	1.40	1.39	1.32	1.30	1.30	1.28	1.21	1.35
Romania	70 to 74	1.31	1.29	1.27	1.39	1.19	1.28	1.26	1.23
Russian Federation	70 to 74	2.09	1.46	1.22	1.22	1.30	1.31	3.77	1.50
Rwanda	70 to 74	3.13	1.74	3.22	1.92	1.23	1.11	1.25	1.22
Saint Kitts and Nevis	70 to 74	1.97	1.69	1.45	1.36	1.13	1.05	1.68	1.54
Saint Lucia	70 to 74	1.98	1.51	1.47	1.37	1.09	1.08	1.48	1.26
Saint Vincent and the Grenadines	70 to 74	1.98	1.48	1.80	1.32	1.08	1.10	1.38	1.24
Samoa	70 to 74	1.88	1.47	1.59	1.36	1.03	0.97	1.21	1.18
San Marino	70 to 74	1.10	1.07	1.04	1.01	1.03	1.02	1.07	1.04
Sao Tome and Principe	70 to 74	1.10	1.36	0.89	0.97	1.00	1.01	1.16	1.54
Saudi Arabia	70 to 74	1.11	1.10	1.25	1.16	1.09	1.08	1.01	1.03
Senegal	70 to 74	1.67	1.73	0.99	0.99	1.07	1.04	1.96	2.17
Serbia	70 to 74	1.26	1.29	1.18	1.37	1.15	1.09	1.24	1.28
Seychelles	70 to 74	1.39	1.21	1.18	1.10	1.13	1.07	1.31	1.16
Sierra Leone	70 to 74	1.70	1.46	0.98	0.90	1.06	1.03	2.14	1.89
Singapore	70 to 74	1.04	0.99	1.00	0.98	0.98	0.98	1.00	0.97
Slovakia	70 to 74	1.18	1.17	1.31	1.26	1.11	1.13	1.13	1.12
Slovenia	70 to 74	1.38	1.20	1.19	1.22	1.14	1.12	1.41	1.14
Solomon Islands	70 to 74	1.66	1.54	1.33	1.31	1.01	1.00	1.10	1.10
Somalia	70 to 74	3.42	2.48	5.46	6.00	1.27	1.20	1.20	1.24
South Africa	70 to 74	1.25	1.34	1.07	1.16	1.05	1.06	1.20	1.26
South Sudan	70 to 74	2.07	1.69	2.31	1.69	1.14	1.10	1.23	1.22
Spain	70 to 74	1.22	1.07	1.13	1.09	1.06	1.10	1.16	1.02
Sri Lanka	70 to 74	1.57	1.35	1.43	1.29	1.09	1.08	1.41	1.25
Sudan	70 to 74	0.94	1.04	1.34	1.03	1.30	1.09	0.83	1.03
Suriname	70 to 74	1.35	1.57	1.18	1.30	1.10	1.13	1.22	1.35
Sweden	70 to 74	1.12	1.07	1.03	1.00	1.01	1.02	1.08	1.03
Switzerland	70 to 74	1.14	1.05	1.07	1.03	1.03	1.06	1.11	1.02
Syrian Arab Republic	70 to 74	1.29	1.21	1.20	1.17	1.15	1.10	1.23	1.15
Taiwan (Province of China)	70 to 74	1.22	1.21	1.20	1.11	1.08	1.14	1.11	1.12
Tajikistan	70 to 74	1.64	1.37	1.10	1.03	1.08	1.05	1.77	1.37
Thailand	70 to 74	1.37	1.24	1.13	1.12	1.34	1.17	1.21	1.14
Timor-Leste	70 to 74	1.18	1.41	0.99	1.11	1.07	1.08	1.12	1.30
Togo	70 to 74	1.53	1.86	0.94	1.00	1.05	1.06	1.85	2.48
Tokelau	70 to 74	2.46	1.56	1.75	1.45	1.02	0.99	1.36	1.21
Tonga	70 to 74	1.43	1.33	1.27	1.23	1.01	0.98	1.23	1.17
Trinidad and Tobago	70 to 74	1.48	1.37	1.23	1.23	1.13	1.11	1.60	1.36
Tunisia	70 to 74	1.20	1.18	1.18	1.10	1.15	1.09	1.16	1.16

Turkey	70 to 74	1.37	1.22	1.29	1.15	1.46	1.21	1.28	1.15
Turkmenistan	70 to 74	1.41	1.90	1.10	1.40	1.18	1.40	1.41	1.80
Tuvalu	70 to 74	2.58	1.62	1.62	1.43	1.01	0.98	1.32	1.22
Uganda	70 to 74	1.92	1.54	2.41	1.66	1.18	1.11	1.19	1.17
Ukraine	70 to 74	1.32	1.32	1.16	1.20	1.35	1.27	1.36	1.31
United Arab Emirates	70 to 74	1.02	0.00	1.06	0.00	1.07	0.00	0.98	0.00
United Kingdom	70 to 74	1.18	1.08	1.06	1.05	1.04	1.06	1.16	1.04
United Republic of Tanzania	70 to 74	1.63	1.32	1.68	1.28	1.13	1.06	1.15	1.20
United States Virgin Islands	70 to 74	1.61	1.34	1.43	1.23	1.16	1.13	1.36	1.20
United States of America	70 to 74	1.14	1.06	1.08	1.05	1.10	1.12	1.09	1.02
Uruguay	70 to 74	1.20	1.19	1.20	1.19	1.13	1.08	1.15	1.14
Uzbekistan	70 to 74	1.49	1.36	1.18	1.10	1.14	1.44	1.40	1.30
Vanuatu	70 to 74	1.84	1.58	1.57	1.45	1.02	1.00	1.27	1.22
Venezuela (Bolivarian Republic of)	70 to 74	1.41	1.47	1.27	1.34	1.06	1.08	1.27	1.32
Viet Nam	70 to 74	2.38	1.97	1.47	1.58	1.27	1.18	1.64	1.62
Yemen	70 to 74	2.16	1.49	2.68	1.30	1.81	1.35	1.37	1.64
Zambia	70 to 74	1.71	1.67	1.38	1.34	1.11	1.08	1.14	1.24
Zimbabwe	70 to 74	1.47	1.79	1.26	1.60	1.12	1.15	1.28	1.50
Afghanistan	75 to 79	4.63	1.68	5.15	2.08	3.72	1.84	1.69	1.61
Albania	75 to 79	2.61	1.50	2.29	1.48	1.24	1.14	1.37	1.20
Algeria	75 to 79	1.31	1.17	1.25	1.12	1.26	1.17	1.30	1.17
American Samoa	75 to 79	1.28	1.26	1.24	1.25	1.00	1.01	1.11	1.10
Andorra	75 to 79	1.08	1.04	1.06	1.04	1.06	1.03	1.07	1.02
Angola	75 to 79	1.71	1.55	1.59	1.46	1.21	1.16	1.34	1.34
Antigua and Barbuda	75 to 79	1.82	1.68	1.40	1.60	1.27	1.20	1.44	1.35
Argentina	75 to 79	1.30	1.18	1.30	1.23	1.18	1.13	1.22	1.12
Armenia	75 to 79	1.42	1.21	1.27	1.20	1.38	1.28	1.41	1.20
Australia	75 to 79	1.15	1.07	1.12	1.09	1.14	1.16	1.13	1.04
Austria	75 to 79	1.17	1.07	1.06	1.06	1.06	1.10	1.18	1.05
Azerbaijan	75 to 79	1.66	1.28	1.36	1.19	1.18	1.15	1.38	1.19
Bahamas	75 to 79	1.36	1.22	1.29	1.21	1.16	1.17	1.20	1.11
Bahrain	75 to 79	7.71	1.57	2.26	1.48	1.88	1.29	4.58	1.53
Bangladesh	75 to 79	2.00	1.37	1.48	1.26	1.74	1.25	1.36	1.23
Barbados	75 to 79	1.36	1.39	1.27	1.42	1.19	1.16	1.27	1.23
Belarus	75 to 79	1.24	1.29	1.19	1.23	1.49	1.53	1.28	1.31
Belgium	75 to 79	1.24	1.12	1.18	1.15	1.10	1.21	1.23	1.07
Belize	75 to 79	1.53	1.32	1.36	1.28	1.20	1.13	1.28	1.18
Benin	75 to 79	1.31	1.32	0.93	0.95	1.06	1.06	1.48	1.51
Bermuda	75 to 79	1.46	1.35	1.31	1.26	1.24	1.26	1.42	1.30
Bhutan	75 to 79	1.23	1.24	1.18	1.13	1.27	1.17	1.13	1.21
Bolivia (Plurinational State of)	75 to 79	1.50	1.30	1.35	1.27	1.11	1.05	1.35	1.24
Bosnia and Herzegovina	75 to 79	1.31	1.26	1.64	1.37	1.41	1.21	1.32	1.28
Botswana	75 to 79	1.44	1.28	1.34	1.19	1.16	1.11	1.30	1.23
Brazil	75 to 79	1.49	1.25	1.41	1.29	1.06	1.13	1.48	1.22
Brunei Darussalam	75 to 79	1.09	1.10	1.13	1.10	1.06	1.05	1.07	1.09
Bulgaria	75 to 79	1.48	1.54	1.72	1.44	1.24	1.52	1.41	1.59
Burkina Faso	75 to 79	1.64	1.54	1.18	1.13	1.18	1.14	1.54	1.54
Burundi	75 to 79	2.08	1.52	2.43	1.64	1.33	1.20	1.25	1.20

Cabo Verde	75 to 79	1.20	1.46	0.99	1.06	1.09	1.08	1.30	1.68
Cambodia	75 to 79	1.48	1.54	1.25	1.42	1.28	1.20	1.27	1.32
Cameroon	75 to 79	1.42	1.41	1.08	1.04	1.10	1.08	1.42	1.52
Canada	75 to 79	1.13	1.06	1.10	1.06	1.15	1.15	1.11	1.03
Central African Republic	75 to 79	2.26	2.25	1.85	1.93	1.26	1.25	1.45	1.50
Chad	75 to 79	1.36	1.47	1.02	1.03	1.10	1.08	1.45	1.64
Chile	75 to 79	1.35	1.19	1.27	1.15	1.12	1.17	1.32	1.17
China	75 to 79	1.58	1.33	1.32	1.20	11.51	1.33	1.30	1.25
Colombia	75 to 79	1.28	1.23	1.19	1.18	1.11	1.13	1.25	1.19
Comoros	75 to 79	1.48	1.33	1.39	1.26	1.17	1.13	1.24	1.21
Congo	75 to 79	1.55	1.39	1.36	1.25	1.16	1.11	1.33	1.28
Cook Islands	75 to 79	1.43	1.23	1.43	1.28	1.07	1.03	1.18	1.10
Costa Rica	75 to 79	1.25	1.22	1.18	1.15	1.10	1.12	1.22	1.18
Coted'Ivoire	75 to 79	1.36	1.38	1.03	1.02	1.08	1.06	1.44	1.53
Croatia	75 to 79	1.65	1.15	3.05	1.50	1.27	1.45	1.68	1.12
Cuba	75 to 79	1.38	1.34	1.25	1.27	1.13	1.11	1.31	1.30
Cyprus	75 to 79	1.17	1.17	1.13	1.09	1.12	1.09	1.14	1.14
Czechia	75 to 79	1.46	1.12	1.48	1.18	1.30	1.25	1.56	1.10
Democratic People's Republic of Korea	75 to 79	1.44	1.42	1.51	1.50	1.78	1.51	1.22	1.26
Democratic Republic of the Congo	75 to 79	1.56	1.71	1.38	1.57	1.18	1.19	1.30	1.36
Denmark	75 to 79	1.17	1.13	1.14	1.13	1.10	1.13	1.15	1.10
Djibouti	75 to 79	1.52	1.39	1.51	1.34	1.20	1.14	1.23	1.26
Dominica	75 to 79	2.95	1.60	2.05	1.45	1.38	1.23	1.85	1.37
Dominican Republic	75 to 79	1.36	1.33	1.29	1.24	1.16	1.12	1.21	1.23
Ecuador	75 to 79	1.33	1.22	1.29	1.23	1.09	1.13	1.26	1.16
Egypt	75 to 79	1.01	1.21	2.82	1.35	2.86	1.42	0.00	1.29
El Salvador	75 to 79	1.40	1.25	1.23	1.18	1.12	1.09	1.34	1.21
Equatorial Guinea	75 to 79	1.95	1.38	1.70	1.21	1.26	1.10	1.42	1.29
Eritrea	75 to 79	1.76	1.62	1.81	1.75	1.29	1.21	1.19	1.25
Estonia	75 to 79	1.54	1.19	1.23	1.20	1.48	1.51	1.82	1.16
Eswatini	75 to 79	1.72	1.44	1.60	1.33	1.21	1.14	1.36	1.29
Ethiopia	75 to 79	1.57	1.31	1.33	1.26	1.21	1.13	1.12	1.13
Fiji	75 to 79	1.34	1.33	1.46	1.58	1.09	1.08	1.16	1.15
Finland	75 to 79	1.11	1.09	1.07	1.05	1.05	1.07	1.11	1.07
France	75 to 79	1.15	1.06	1.18	1.07	1.09	1.14	1.13	1.03
Gabon	75 to 79	1.58	1.43	1.52	1.32	1.19	1.14	1.27	1.26
Gambia	75 to 79	1.39	1.47	0.99	0.99	1.08	1.06	1.51	1.67
Georgia	75 to 79	2.24	1.95	1.40	1.39	1.27	1.30	1.70	1.89
Germany	75 to 79	1.20	1.07	1.17	1.08	1.11	1.12	1.20	1.04
Ghana	75 to 79	1.35	1.33	0.91	0.94	1.06	1.03	1.51	1.47
Greece	75 to 79	1.22	1.11	1.16	1.11	1.12	1.13	1.21	1.06
Greenland	75 to 79	1.12	1.16	1.13	1.15	1.20	1.21	1.09	1.09
Grenada	75 to 79	2.19	2.29	1.61	2.14	1.37	1.37	1.92	1.79
Guam	75 to 79	1.25	1.16	1.24	1.17	1.05	1.03	1.16	1.10
Guatemala	75 to 79	1.34	1.29	1.24	1.20	1.09	1.11	1.23	1.20
Guinea	75 to 79	1.23	1.30	0.93	0.94	1.06	1.04	1.36	1.50
Guinea-Bissau	75 to 79	1.78	1.61	1.02	0.98	1.15	1.07	2.00	1.93
Guyana	75 to 79	3.59	1.52	1.83	1.34	1.24	1.23	2.28	1.40

Haiti	75 to 79	2.46	1.68	1.71	1.54	1.34	1.23	1.24	1.23
Honduras	75 to 79	1.55	1.72	1.37	1.41	1.16	1.10	1.48	1.65
Hungary	75 to 79	1.36	1.20	1.42	1.25	1.21	1.23	1.36	1.19
Iceland	75 to 79	1.16	1.10	1.13	1.11	1.09	1.09	1.15	1.07
India	75 to 79	1.39	1.33	1.24	1.18	1.39	1.20	1.23	1.25
Indonesia	75 to 79	1.29	1.43	1.20	1.28	1.15	1.15	1.20	1.32
Iran (Islamic Republic of)	75 to 79	1.44	1.15	1.26	1.13	1.33	1.16	1.59	1.16
Iraq	75 to 79	1.95	1.72	1.64	1.49	1.40	1.28	1.98	1.82
Ireland	75 to 79	1.18	1.14	1.09	1.08	1.13	1.15	1.19	1.12
Israel	75 to 79	1.07	1.06	1.15	1.12	1.08	1.18	1.04	1.01
Italy	75 to 79	1.27	1.13	1.23	1.13	1.10	1.20	1.28	1.08
Jamaica	75 to 79	1.74	1.49	1.42	1.36	1.12	1.20	1.49	1.32
Japan	75 to 79	1.18	1.10	1.10	1.06	1.07	1.03	1.18	1.07
Jordan	75 to 79	1.08	1.08	1.10	1.13	1.12	1.11	1.10	1.08
Kazakhstan	75 to 79	1.56	1.56	1.26	1.37	1.35	1.40	1.61	1.52
Kenya	75 to 79	1.30	1.38	1.25	1.28	1.15	1.14	1.12	1.19
Kiribati	75 to 79	1.62	1.54	1.44	1.42	1.20	1.12	1.28	1.29
Kuwait	75 to 79	1.04	1.17	1.08	1.17	1.11	1.15	1.02	1.15
Kyrgyzstan	75 to 79	1.86	1.59	1.33	1.25	1.45	1.54	1.88	1.67
Lao People's Democratic Republic	75 to 79	1.63	1.40	1.39	1.27	1.31	1.16	1.33	1.31
Latvia	75 to 79	1.53	1.19	1.40	1.21	1.56	1.51	1.63	1.19
Lebanon	75 to 79	1.56	1.16	2.67	1.28	2.29	1.31	1.27	1.12
Lesotho	75 to 79	2.22	2.02	1.80	1.69	1.31	1.23	1.47	1.51
Liberia	75 to 79	1.27	1.31	0.91	0.92	1.06	1.05	1.43	1.53
Libya	75 to 79	1.26	1.26	1.21	1.12	1.20	1.14	1.25	1.32
Lithuania	75 to 79	1.29	1.23	1.12	1.16	1.37	1.48	1.38	1.24
Luxembourg	75 to 79	1.48	1.17	1.26	1.20	1.11	1.15	1.54	1.12
Madagascar	75 to 79	1.83	1.62	1.77	1.66	1.27	1.18	1.31	1.28
Malawi	75 to 79	1.41	1.48	1.42	1.55	1.16	1.15	1.23	1.32
Malaysia	75 to 79	1.21	1.22	1.15	1.17	1.16	1.13	1.12	1.13
Maldives	75 to 79	1.27	1.21	1.27	1.12	1.17	1.10	1.13	1.15
Mali	75 to 79	1.14	1.16	0.91	0.89	1.04	1.02	1.22	1.29
Malta	75 to 79	1.38	1.11	1.18	1.06	1.10	1.10	1.42	1.08
Marshall Islands	75 to 79	1.81	1.48	1.72	1.55	1.17	1.09	1.22	1.15
Mauritania	75 to 79	1.24	1.23	0.85	0.90	1.05	1.03	1.42	1.38
Mauritius	75 to 79	1.45	1.27	1.36	1.26	1.45	1.29	1.37	1.19
Mexico	75 to 79	1.31	1.20	1.17	1.17	1.09	1.12	1.32	1.17
Micronesia (Federated States of)	75 to 79	1.58	1.45	1.56	1.46	1.12	1.09	1.17	1.17
Monaco	75 to 79	1.19	1.09	1.19	1.09	1.09	1.06	1.17	1.07
Mongolia	75 to 79	3.02	1.57	1.69	1.11	2.29	1.53	1.22	1.16
Montenegro	75 to 79	1.55	1.54	1.67	1.77	1.15	1.10	1.18	1.21
Morocco	75 to 79	1.12	1.18	1.14	1.15	1.29	1.23	1.16	1.22
Mozambique	75 to 79	1.78	2.05	1.58	1.81	1.25	1.24	1.37	1.58
Myanmar	75 to 79	1.83	1.64	1.36	1.51	1.39	1.26	1.45	1.38
Namibia	75 to 79	1.48	1.47	1.30	1.38	1.14	1.13	1.38	1.37
Nauru	75 to 79	1.50	1.45	1.54	1.70	1.04	0.95	1.21	1.23
Nepal	75 to 79	1.30	1.46	1.22	1.25	1.38	1.29	1.16	1.37
Netherlands	75 to 79	1.15	1.10	1.16	1.10	1.14	1.15	1.13	1.07

New Zealand	75 to 79	1.20	1.10	1.18	1.16	1.16	1.14	1.19	1.07
Nicaragua	75 to 79	1.24	1.18	1.17	1.14	1.10	1.09	1.21	1.14
Niger	75 to 79	1.38	1.43	1.09	1.08	1.12	1.11	1.34	1.45
Nigeria	75 to 79	1.47	1.35	1.02	1.02	1.11	1.07	1.62	1.50
Niue	75 to 79	1.45	1.42	1.52	1.56	1.09	1.06	1.18	1.19
North Macedonia	75 to 79	1.63	1.37	2.02	1.45	1.44	1.18	1.71	1.42
Northern Mariana Islands	75 to 79	1.20	1.27	1.14	1.22	0.93	1.02	1.10	1.13
Norway	75 to 79	1.14	1.08	1.19	1.08	1.07	1.12	1.12	1.06
Oman	75 to 79	1.66	1.26	1.60	1.26	1.33	1.17	1.64	1.27
Pakistan	75 to 79	1.18	1.21	1.15	1.14	1.23	1.18	1.11	1.18
Palau	75 to 79	1.44	1.18	1.43	1.18	1.06	0.99	1.20	1.09
Palestine	75 to 79	3.35	1.44	1.63	1.33	1.22	1.16	7.60	1.53
Panama	75 to 79	1.32	1.35	1.21	1.26	1.08	1.18	1.25	1.25
Papua New Guinea	75 to 79	1.30	1.22	1.27	1.20	1.00	0.99	1.00	1.02
Paraguay	75 to 79	1.33	1.34	1.37	1.45	1.08	1.07	1.27	1.29
Peru	75 to 79	1.40	1.20	1.33	1.19	1.13	1.05	1.29	1.15
Philippines	75 to 79	1.51	1.57	1.44	1.45	1.19	1.19	1.28	1.33
Poland	75 to 79	2.05	1.22	1.47	1.31	1.26	1.21	2.56	1.20
Portugal	75 to 79	1.45	1.17	1.28	1.16	1.18	1.15	1.46	1.12
Puerto Rico	75 to 79	1.31	1.23	1.21	1.17	1.09	1.13	1.25	1.16
Qatar	75 to 79	1.42	1.20	1.96	1.21	1.01	1.39	1.25	1.17
Republic of Korea	75 to 79	1.23	1.07	1.23	1.08	1.18	1.06	1.18	1.05
Republic of Moldova	75 to 79	1.47	1.26	1.58	1.32	1.59	1.39	1.29	1.24
Romania	75 to 79	1.37	1.30	1.49	1.53	1.34	1.48	1.37	1.27
Russian Federation	75 to 79	3.54	1.67	1.31	1.33	1.52	1.54	#####	1.84
Rwanda	75 to 79	2.11	1.55	2.40	1.72	1.31	1.18	1.25	1.22
Saint Kitts and Nevis	75 to 79	2.08	1.55	1.60	1.41	1.23	1.12	1.75	1.39
Saint Lucia	75 to 79	1.50	1.45	1.39	1.42	1.13	1.11	1.24	1.26
Saint Vincent and the Grenadines	75 to 79	2.59	1.54	2.58	1.40	1.18	1.15	1.53	1.30
Samoa	75 to 79	1.61	1.34	1.67	1.39	1.13	1.04	1.18	1.16
San Marino	75 to 79	1.12	1.09	1.09	1.06	1.09	1.07	1.13	1.08
Sao Tome and Principe	75 to 79	1.28	1.27	1.00	0.99	1.06	1.04	1.35	1.37
Saudi Arabia	75 to 79	1.31	1.11	1.34	1.24	1.21	1.19	1.30	1.09
Senegal	75 to 79	1.38	1.44	0.98	1.00	1.09	1.06	1.49	1.59
Serbia	75 to 79	3.63	1.13	1.01	1.28	1.62	1.13	4.80	1.14
Seychelles	75 to 79	1.59	1.38	1.41	1.31	1.25	1.18	1.47	1.31
Sierra Leone	75 to 79	1.38	1.31	0.98	0.95	1.08	1.05	1.54	1.50
Singapore	75 to 79	1.12	0.99	1.08	0.98	1.03	1.00	1.09	0.96
Slovakia	75 to 79	1.21	1.16	1.58	1.33	1.22	1.21	1.18	1.14
Slovenia	75 to 79	1.54	1.17	1.41	1.24	1.24	1.17	1.63	1.15
Solomon Islands	75 to 79	1.47	1.38	1.43	1.37	1.11	1.08	1.11	1.10
Somalia	75 to 79	2.14	1.78	2.56	2.19	1.37	1.26	1.20	1.21
South Africa	75 to 79	1.24	1.28	1.15	1.24	1.11	1.11	1.19	1.21
South Sudan	75 to 79	1.70	1.47	1.83	1.50	1.21	1.17	1.22	1.21
Spain	75 to 79	1.27	1.05	1.19	1.14	1.15	1.21	1.26	1.02
Sri Lanka	75 to 79	1.55	1.37	1.53	1.38	1.17	1.14	1.41	1.29
Sudan	75 to 79	1.21	1.13	1.55	1.14	1.67	1.23	1.14	1.18
Suriname	75 to 79	1.47	1.29	1.39	1.23	1.14	1.15	1.27	1.18

Sweden	75 to 79	1.13	1.08	1.08	1.05	1.04	1.08	1.13	1.06
Switzerland	75 to 79	1.17	1.06	1.10	1.06	1.09	1.10	1.18	1.03
Syrian Arab Republic	75 to 79	1.31	1.22	1.29	1.25	1.27	1.20	1.25	1.18
Taiwan (Province of China)	75 to 79	1.18	1.17	1.19	1.15	1.20	1.23	1.12	1.12
Tajikistan	75 to 79	1.91	1.42	1.33	1.14	1.22	1.12	1.91	1.42
Thailand	75 to 79	1.36	1.25	1.21	1.18	1.50	1.25	1.23	1.15
Timor-Leste	75 to 79	1.26	1.40	1.13	1.25	1.15	1.16	1.17	1.29
Togo	75 to 79	1.33	1.49	0.96	1.01	1.08	1.07	1.48	1.70
Tokelau	75 to 79	1.76	1.45	1.65	1.46	1.11	1.05	1.24	1.18
Tonga	75 to 79	1.27	1.23	1.28	1.24	1.06	1.03	1.16	1.14
Trinidad and Tobago	75 to 79	1.47	1.30	1.31	1.25	1.18	1.14	1.52	1.29
Tunisia	75 to 79	1.21	1.23	1.25	1.20	1.30	1.20	1.21	1.25
Turkey	75 to 79	1.49	1.27	1.47	1.22	1.97	1.37	1.39	1.22
Turkmenistan	75 to 79	1.46	1.65	1.23	1.41	1.35	1.75	1.45	1.53
Tuvalu	75 to 79	1.81	1.42	1.73	1.47	1.12	1.06	1.23	1.18
Uganda	75 to 79	1.69	1.43	2.14	1.59	1.26	1.17	1.20	1.18
Ukraine	75 to 79	1.40	1.37	1.22	1.27	1.68	1.44	1.54	1.43
United Arab Emirates	75 to 79	1.20	0.15	1.21	0.00	1.45	0.00	1.20	0.26
United Kingdom	75 to 79	1.22	1.09	1.12	1.11	1.09	1.12	1.25	1.06
United Republic of Tanzania	75 to 79	1.59	1.34	1.78	1.36	1.22	1.14	1.19	1.24
United States Virgin Islands	75 to 79	1.49	1.28	1.38	1.24	1.20	1.17	1.31	1.19
United States of America	75 to 79	1.15	1.08	1.13	1.09	1.17	1.21	1.12	1.04
Uruguay	75 to 79	1.25	1.19	1.29	1.23	1.13	1.14	1.22	1.15
Uzbekistan	75 to 79	1.48	1.42	1.24	1.14	1.31	1.78	1.38	1.40
Vanuatu	75 to 79	1.58	1.42	1.71	1.54	1.11	1.08	1.21	1.18
Venezuela (Bolivarian Republic of)	75 to 79	1.39	1.41	1.29	1.32	1.10	1.14	1.29	1.31
Viet Nam	75 to 79	2.18	1.99	1.62	1.84	1.41	1.30	1.56	1.60
Yemen	75 to 79	2.78	1.75	2.57	1.54	2.59	1.62	1.79	1.91
Zambia	75 to 79	1.62	1.58	1.56	1.51	1.21	1.16	1.18	1.26
Zimbabwe	75 to 79	1.42	1.57	1.45	1.80	1.20	1.22	1.26	1.35
Afghanistan	80 to 84	3.06	1.48	6.10	1.43	2.89	1.49	1.25	1.15
Albania	80 to 84	2.29	1.48	#####	1.20	1.11	0.98	1.27	1.15
Algeria	80 to 84	1.01	1.13	0.39	0.94	0.87	1.05	0.97	1.10
American Samoa	80 to 84	1.17	1.16	1.02	1.05	0.92	0.94	1.08	1.07
Andorra	80 to 84	1.05	1.04	0.97	0.98	0.98	0.99	1.03	1.02
Angola	80 to 84	1.42	1.36	1.20	1.07	1.15	1.10	1.24	1.23
Antigua and Barbuda	80 to 84	1.51	1.34	1.11	1.19	1.17	1.16	1.31	1.20
Argentina	80 to 84	1.20	1.12	1.15	1.15	0.89	1.03	1.12	1.07
Armenia	80 to 84	1.34	1.11	1.11	1.04	1.13	1.11	1.24	1.06
Australia	80 to 84	1.08	1.03	1.00	1.01	1.06	1.11	1.05	1.01
Austria	80 to 84	1.11	1.05	0.98	1.00	1.00	1.06	1.08	1.03
Azerbaijan	80 to 84	1.45	1.18	1.12	0.95	1.10	1.06	1.26	1.11
Bahamas	80 to 84	1.11	1.14	0.98	1.06	1.08	1.10	1.04	1.06
Bahrain	80 to 84	4.73	1.37	2.64	1.31	1.57	1.11	1.96	1.17
Bangladesh	80 to 84	1.44	1.45	1.12	1.14	1.31	1.09	1.18	1.24
Barbados	80 to 84	1.25	1.19	1.10	1.15	1.15	1.10	1.14	1.09
Belarus	80 to 84	1.13	1.20	1.08	1.15	1.27	1.32	1.07	1.14
Belgium	80 to 84	1.21	1.07	1.08	1.06	1.01	1.12	1.16	1.04

Belize	80 to 84	1.23	1.11	1.03	0.99	1.13	1.06	1.13	1.05
Benin	80 to 84	1.22	1.23	0.65	0.73	0.98	0.99	1.26	1.27
Bermuda	80 to 84	1.29	1.22	1.12	1.16	1.18	1.20	1.21	1.15
Bhutan	80 to 84	1.18	1.18	1.04	1.02	1.13	1.05	1.10	1.13
Bolivia (Plurinational State of)	80 to 84	1.31	1.20	0.95	0.96	1.01	0.95	1.21	1.14
Bosnia and Herzegovina	80 to 84	1.25	1.20	1.18	1.06	1.07	1.03	1.14	1.14
Botswana	80 to 84	1.29	1.20	0.99	0.96	1.07	1.06	1.21	1.16
Brazil	80 to 84	1.33	1.15	1.12	1.10	1.02	1.08	1.24	1.11
Brunei Darussalam	80 to 84	1.04	1.02	1.13	1.00	0.91	0.95	0.98	1.00
Bulgaria	80 to 84	1.34	1.50	1.33	1.12	1.09	1.10	1.19	1.38
Burkina Faso	80 to 84	1.49	1.40	1.04	0.96	1.14	1.09	1.42	1.37
Burundi	80 to 84	1.56	1.38	1.92	1.39	1.26	1.17	1.19	1.17
Cabo Verde	80 to 84	1.20	1.34	0.86	0.92	1.06	1.05	1.23	1.36
Cambodia	80 to 84	1.25	1.30	0.82	1.00	1.09	1.04	1.13	1.16
Cameroon	80 to 84	1.32	1.30	0.89	0.83	1.05	1.03	1.30	1.31
Canada	80 to 84	1.08	1.03	1.02	1.01	1.09	1.11	1.06	1.01
Central African Republic	80 to 84	1.60	1.63	1.25	1.29	1.16	1.15	1.30	1.32
Chad	80 to 84	1.27	1.33	0.82	0.81	1.04	1.02	1.28	1.35
Chile	80 to 84	1.22	1.14	1.17	1.10	0.94	1.03	1.14	1.11
China	80 to 84	1.34	1.25	1.38	1.15	0.00	1.07	1.11	1.15
Colombia	80 to 84	1.19	1.17	1.01	1.04	1.04	1.05	1.14	1.13
Comoros	80 to 84	1.30	1.25	1.01	0.97	1.12	1.09	1.17	1.16
Congo	80 to 84	1.34	1.26	0.90	0.85	1.08	1.05	1.22	1.18
Cook Islands	80 to 84	1.26	1.15	1.22	1.18	0.99	0.99	1.13	1.07
Costa Rica	80 to 84	1.18	1.15	1.06	1.04	1.05	1.06	1.14	1.12
Coted'Ivoire	80 to 84	1.27	1.29	0.87	0.86	1.03	1.01	1.27	1.30
Croatia	80 to 84	1.64	1.08	1.84	1.23	1.14	1.11	1.43	1.05
Cuba	80 to 84	1.23	1.22	1.05	1.09	1.08	1.06	1.15	1.15
Cyprus	80 to 84	1.33	1.14	0.93	0.99	0.67	1.00	1.10	1.12
Czechia	80 to 84	1.35	1.08	1.16	1.08	1.07	1.09	1.25	1.05
Democratic People's Republic of Korea	80 to 84	1.24	1.25	1.42	1.34	1.17	1.14	1.10	1.13
Democratic Republic of the Congo	80 to 84	1.36	1.45	1.05	1.16	1.12	1.12	1.22	1.26
Denmark	80 to 84	1.12	1.10	1.02	1.05	1.03	1.06	1.09	1.06
Djibouti	80 to 84	1.35	1.29	1.17	1.00	1.16	1.10	1.19	1.19
Dominica	80 to 84	1.76	1.31	1.24	1.01	1.24	1.14	1.40	1.16
Dominican Republic	80 to 84	1.19	1.17	1.04	1.04	1.09	1.07	1.09	1.11
Ecuador	80 to 84	1.18	1.17	1.04	1.07	1.05	1.07	1.13	1.12
Egypt	80 to 84	0.00	0.56	0.00	0.77	2.12	1.11	0.00	0.43
El Salvador	80 to 84	1.23	1.16	1.01	1.01	1.05	1.03	1.19	1.12
Equatorial Guinea	80 to 84	1.52	1.26	1.24	0.96	1.17	1.06	1.28	1.18
Eritrea	80 to 84	1.42	1.42	1.33	1.35	1.22	1.15	1.16	1.21
Estonia	80 to 84	1.58	1.12	1.08	1.14	1.42	1.38	1.39	1.07
Eswatini	80 to 84	1.42	1.27	1.13	0.94	1.10	1.05	1.27	1.19
Ethiopia	80 to 84	1.37	1.25	1.17	1.09	1.19	1.12	1.11	1.12
Fiji	80 to 84	1.20	1.23	1.22	1.39	1.01	1.00	1.11	1.12
Finland	80 to 84	1.05	1.06	0.97	0.98	0.97	1.00	1.03	1.04
France	80 to 84	1.13	1.05	1.06	1.01	0.98	1.06	1.08	1.02
Gabon	80 to 84	1.38	1.29	1.21	0.98	1.13	1.08	1.21	1.19

Gambia	80 to 84	1.28	1.32	0.80	0.78	1.03	1.01	1.29	1.34
Georgia	80 to 84	1.96	2.41	1.15	1.22	1.13	1.03	1.57	2.02
Germany	80 to 84	1.18	1.05	1.07	1.02	1.02	1.06	1.14	1.03
Ghana	80 to 84	1.27	1.27	0.73	0.73	1.01	0.98	1.30	1.28
Greece	80 to 84	1.17	1.07	1.00	1.01	1.04	1.04	1.11	1.03
Greenland	80 to 84	1.07	1.11	0.95	1.01	0.82	1.04	1.04	1.07
Grenada	80 to 84	3.73	1.50	1.82	1.19	1.47	1.15	2.71	1.38
Guam	80 to 84	1.13	1.14	1.02	1.11	0.95	1.02	1.09	1.09
Guatemala	80 to 84	1.20	1.18	0.99	1.00	1.07	1.05	1.15	1.14
Guinea	80 to 84	1.21	1.23	0.76	0.75	1.02	1.00	1.23	1.27
Guinea-Bissau	80 to 84	1.43	1.39	0.73	0.66	1.05	0.99	1.45	1.43
Guyana	80 to 84	1.60	1.25	1.28	0.97	1.16	1.13	1.34	1.16
Haiti	80 to 84	1.55	1.27	1.17	1.07	1.27	1.10	1.06	1.00
Honduras	80 to 84	1.34	1.39	1.04	0.88	1.08	0.96	1.26	1.28
Hungary	80 to 84	1.31	1.14	1.17	1.07	1.09	1.09	1.20	1.10
Iceland	80 to 84	1.10	1.04	1.04	1.01	1.00	1.01	1.08	1.02
India	80 to 84	1.18	1.15	1.06	1.03	1.15	1.04	1.09	1.09
Indonesia	80 to 84	1.16	1.25	0.88	0.91	1.00	0.98	1.09	1.17
Iran (Islamic Republic of)	80 to 84	1.28	1.06	1.02	0.98	1.10	1.02	1.16	1.02
Iraq	80 to 84	1.53	1.52	1.26	1.21	1.23	1.13	1.32	1.35
Ireland	80 to 84	1.13	1.10	1.01	1.00	1.02	1.07	1.09	1.08
Israel	80 to 84	1.03	1.03	1.01	1.03	1.04	1.12	1.00	0.99
Italy	80 to 84	1.23	1.10	1.06	1.04	1.01	1.11	1.18	1.06
Jamaica	80 to 84	1.30	1.29	1.05	1.09	1.06	1.10	1.20	1.17
Japan	80 to 84	1.16	1.09	1.08	1.09	0.99	0.98	1.11	1.05
Jordan	80 to 84	1.02	1.03	0.93	0.97	1.07	1.06	0.98	1.02
Kazakhstan	80 to 84	1.56	1.55	1.06	1.15	1.10	1.01	1.46	1.41
Kenya	80 to 84	1.23	1.26	1.03	1.02	1.11	1.09	1.11	1.15
Kiribati	80 to 84	1.39	1.34	1.36	1.22	1.12	1.04	1.21	1.21
Kuwait	80 to 84	1.02	1.12	1.05	1.09	1.06	1.10	0.98	1.09
Kyrgyzstan	80 to 84	1.82	1.48	1.08	1.12	1.26	1.20	1.59	1.39
Lao People's Democratic Republic	80 to 84	1.29	1.22	0.95	0.91	1.10	1.02	1.13	1.14
Latvia	80 to 84	1.39	1.09	1.07	1.12	1.37	1.40	1.26	1.05
Lebanon	80 to 84	1.47	1.11	4.05	1.21	1.77	1.17	1.13	1.06
Lesotho	80 to 84	1.64	1.51	1.43	1.19	1.19	1.12	1.35	1.31
Liberia	80 to 84	1.22	1.25	0.72	0.69	1.01	1.01	1.25	1.29
Libya	80 to 84	1.22	1.25	1.07	0.98	1.12	1.05	1.13	1.19
Lithuania	80 to 84	1.20	1.15	1.01	1.00	1.21	1.33	1.17	1.12
Luxembourg	80 to 84	1.38	1.13	1.07	1.09	1.00	1.07	1.29	1.09
Madagascar	80 to 84	1.48	1.42	1.28	1.28	1.21	1.13	1.24	1.22
Malawi	80 to 84	1.28	1.32	1.02	1.05	1.11	1.10	1.17	1.21
Malaysia	80 to 84	1.10	1.07	0.97	0.92	1.05	0.99	1.05	1.02
Maldives	80 to 84	1.08	1.15	0.80	0.93	1.01	1.01	1.02	1.10
Mali	80 to 84	1.15	1.16	0.73	0.71	1.01	0.99	1.16	1.20
Malta	80 to 84	1.26	1.11	1.05	1.02	1.05	1.08	1.20	1.07
Marshall Islands	80 to 84	1.37	1.28	1.30	1.30	1.06	1.00	1.14	1.11
Mauritania	80 to 84	1.20	1.20	0.65	0.73	1.00	0.99	1.24	1.23
Mauritius	80 to 84	1.22	1.18	1.07	1.09	1.20	1.14	1.15	1.12

Mexico	80 to 84	1.18	1.13	1.02	1.04	1.03	1.04	1.16	1.10
Micronesia (Federated States of)	80 to 84	1.32	1.27	1.34	1.25	1.01	1.00	1.12	1.12
Monaco	80 to 84	1.18	1.09	1.08	1.03	1.01	1.01	1.12	1.05
Mongolia	80 to 84	2.47	1.41	2.14	1.00	1.83	1.19	1.24	1.18
Montenegro	80 to 84	1.44	1.31	1.92	1.50	1.03	0.90	1.13	1.11
Morocco	80 to 84	1.08	1.11	0.85	0.91	1.13	1.06	0.99	1.05
Mozambique	80 to 84	1.39	1.51	1.19	1.32	1.15	1.13	1.22	1.30
Myanmar	80 to 84	1.45	1.38	0.88	1.11	1.18	1.10	1.27	1.23
Namibia	80 to 84	1.32	1.31	0.94	1.00	1.06	1.05	1.26	1.24
Nauru	80 to 84	1.27	1.18	1.21	0.39	0.92	0.83	1.13	1.10
Nepal	80 to 84	1.27	1.31	1.08	1.09	1.20	1.13	1.14	1.22
Netherlands	80 to 84	1.11	1.08	1.06	1.03	1.03	1.08	1.08	1.05
New Zealand	80 to 84	1.15	1.06	1.10	1.07	1.07	1.03	1.10	1.04
Nicaragua	80 to 84	1.20	1.13	1.06	1.04	1.06	1.04	1.17	1.10
Niger	80 to 84	1.29	1.33	0.88	0.83	1.06	1.05	1.25	1.32
Nigeria	80 to 84	1.33	1.26	0.81	0.87	1.04	1.02	1.35	1.27
Niue	80 to 84	1.28	1.26	1.31	1.36	0.99	0.98	1.13	1.13
North Macedonia	80 to 84	1.58	1.50	1.25	1.25	1.05	0.91	1.36	1.33
Northern Mariana Islands	80 to 84	1.13	1.15	0.88	0.99	0.82	0.91	1.06	1.08
Norway	80 to 84	1.10	1.05	1.11	1.01	0.97	1.04	1.07	1.04
Oman	80 to 84	1.61	1.19	1.60	1.21	1.21	1.09	1.37	1.13
Pakistan	80 to 84	1.13	1.13	1.02	0.98	1.10	1.04	1.07	1.09
Palau	80 to 84	1.26	1.07	1.26	0.94	0.98	0.90	1.13	1.03
Palestine	80 to 84	3.82	1.49	1.17	1.02	1.15	1.12	3.14	1.42
Panama	80 to 84	1.22	1.22	1.03	1.10	1.01	1.09	1.18	1.17
Papua New Guinea	80 to 84	1.14	1.11	0.81	0.72	0.89	0.89	0.98	0.99
Paraguay	80 to 84	1.20	1.16	1.07	1.06	1.05	1.01	1.13	1.11
Peru	80 to 84	1.24	1.16	1.07	1.03	1.06	1.01	1.18	1.12
Philippines	80 to 84	1.22	1.32	1.03	1.18	1.08	1.09	1.13	1.20
Poland	80 to 84	1.92	1.16	1.20	1.17	1.10	1.10	1.62	1.11
Portugal	80 to 84	1.37	1.14	1.06	1.06	1.00	1.04	1.28	1.08
Puerto Rico	80 to 84	1.21	1.15	1.04	1.08	1.05	1.09	1.16	1.11
Qatar	80 to 84	1.28	1.13	2.38	1.15	1.01	1.30	1.08	1.07
Republic of Korea	80 to 84	1.19	1.09	1.20	1.11	0.97	1.00	1.10	1.04
Republic of Moldova	80 to 84	1.28	1.07	1.22	1.06	1.53	1.19	1.10	1.03
Romania	80 to 84	1.31	1.20	1.04	1.07	1.10	1.10	1.21	1.13
Russian Federation	80 to 84	1.01	1.76	1.10	1.22	1.28	1.25	1.01	1.58
Rwanda	80 to 84	1.56	1.39	1.86	1.49	1.25	1.15	1.19	1.19
Saint Kitts and Nevis	80 to 84	1.59	1.37	1.19	1.14	1.15	1.11	1.36	1.22
Saint Lucia	80 to 84	1.52	1.39	1.24	1.31	1.09	1.08	1.29	1.23
Saint Vincent and the Grenadines	80 to 84	1.38	1.46	1.07	1.20	1.12	1.12	1.15	1.32
Samoa	80 to 84	1.36	1.20	1.63	1.17	1.02	0.95	1.12	1.10
San Marino	80 to 84	1.10	1.04	0.98	0.98	1.02	1.03	1.08	1.04
Sao Tome and Principe	80 to 84	1.18	1.21	0.84	0.85	1.02	1.00	1.19	1.22
Saudi Arabia	80 to 84	1.23	1.03	1.13	1.11	1.11	1.12	1.07	0.97
Senegal	80 to 84	1.29	1.32	0.80	0.78	1.04	1.01	1.30	1.33
Serbia	80 to 84	1.56	1.09	1.58	1.18	0.85	0.93	1.23	1.01
Seychelles	80 to 84	1.32	1.14	1.03	0.95	1.11	1.05	1.23	1.10

Sierra Leone	80 to 84	1.27	1.23	0.79	0.75	1.02	1.00	1.28	1.26
Singapore	80 to 84	1.03	1.00	1.02	1.00	0.96	1.00	1.00	0.98
Slovakia	80 to 84	1.14	1.11	1.20	1.10	1.05	1.07	1.09	1.08
Slovenia	80 to 84	1.40	1.15	1.09	1.19	1.09	1.06	1.32	1.10
Solomon Islands	80 to 84	1.24	1.21	1.23	1.18	1.00	1.00	1.06	1.06
Somalia	80 to 84	1.58	1.49	2.03	1.74	1.29	1.20	1.17	1.18
South Africa	80 to 84	1.19	1.20	0.98	0.98	1.05	1.04	1.16	1.15
South Sudan	80 to 84	1.40	1.31	1.34	1.17	1.15	1.12	1.18	1.16
Spain	80 to 84	1.24	1.02	1.07	1.06	1.06	1.13	1.19	0.99
Sri Lanka	80 to 84	1.43	1.29	1.04	1.01	1.08	1.07	1.25	1.18
Sudan	80 to 84	1.19	1.06	1.26	0.86	1.45	1.03	0.95	1.02
Suriname	80 to 84	1.21	1.23	1.03	1.12	1.08	1.11	1.11	1.13
Sweden	80 to 84	1.07	1.05	0.98	1.00	0.95	1.01	1.05	1.03
Switzerland	80 to 84	1.13	1.04	0.96	0.99	1.00	1.05	1.10	1.03
Syrian Arab Republic	80 to 84	1.15	1.10	1.02	1.05	1.11	1.06	1.03	1.01
Taiwan (Province of China)	80 to 84	1.10	1.10	1.02	1.06	1.09	1.10	1.05	1.07
Tajikistan	80 to 84	1.62	1.37	0.99	0.92	1.06	0.95	1.44	1.30
Thailand	80 to 84	1.15	1.16	0.92	1.03	1.08	1.07	1.08	1.09
Timor-Leste	80 to 84	1.16	1.22	0.74	0.79	1.00	0.98	1.08	1.14
Togo	80 to 84	1.26	1.35	0.76	0.76	1.03	1.01	1.28	1.37
Tokelau	80 to 84	1.39	1.34	1.39	1.37	0.99	0.99	1.16	1.14
Tonga	80 to 84	1.17	1.13	1.09	1.05	0.98	0.96	1.11	1.08
Trinidad and Tobago	80 to 84	1.28	1.22	1.07	1.07	1.08	1.08	1.22	1.16
Tunisia	80 to 84	1.38	1.18	1.26	1.02	1.21	1.06	1.17	1.12
Turkey	80 to 84	1.30	1.19	1.20	1.09	1.50	1.20	1.16	1.12
Turkmenistan	80 to 84	1.39	1.48	1.07	1.16	1.13	1.22	1.30	1.32
Tuvalu	80 to 84	1.41	1.25	1.42	1.17	0.99	0.97	1.16	1.12
Uganda	80 to 84	1.44	1.31	1.81	1.36	1.22	1.14	1.18	1.16
Ukraine	80 to 84	1.23	1.30	1.06	1.15	1.28	1.18	1.15	1.23
United Arab Emirates	80 to 84	1.12	0.51	1.04	0.17	1.07	0.08	1.07	0.56
United Kingdom	80 to 84	1.18	1.08	1.00	1.02	0.98	1.05	1.14	1.05
United Republic of Tanzania	80 to 84	1.42	1.28	1.62	1.13	1.21	1.10	1.18	1.18
United States Virgin Islands	80 to 84	1.32	1.22	1.11	1.15	1.13	1.11	1.18	1.15
United States of America	80 to 84	1.11	1.05	1.04	1.01	1.07	1.12	1.08	1.02
Uruguay	80 to 84	1.17	1.15	1.14	1.16	0.96	1.02	1.12	1.10
Uzbekistan	80 to 84	1.30	1.35	1.00	1.03	1.18	1.08	1.20	1.30
Vanuatu	80 to 84	1.33	1.25	1.47	1.28	1.00	0.99	1.15	1.12
Venezuela (Bolivarian Republic of)	80 to 84	1.17	1.27	1.01	1.08	1.05	1.09	1.12	1.19
Viet Nam	80 to 84	1.73	1.64	1.17	1.27	1.24	1.13	1.40	1.39
Yemen	80 to 84	2.21	1.64	2.28	1.09	2.08	1.32	1.28	1.34
Zambia	80 to 84	1.41	1.38	1.20	1.15	1.18	1.11	1.16	1.19
Zimbabwe	80 to 84	1.35	1.42	1.01	0.95	1.05	1.02	1.24	1.28
Afghanistan	85 to 89	2.36	1.89	1.56	1.21	1.65	1.24	1.53	1.55
Albania	85 to 89	2.41	1.61	3.27	1.28	1.13	0.87	1.40	1.20
Algeria	85 to 89	0.80	1.12	0.20	0.50	0.66	0.67	0.82	1.06
American Samoa	85 to 89	1.16	1.14	1.17	1.17	0.69	0.72	1.04	1.02
Andorra	85 to 89	1.05	1.05	0.97	0.97	0.95	0.96	1.02	1.03
Angola	85 to 89	1.25	1.27	1.07	1.07	1.09	1.07	1.14	1.17

Antigua and Barbuda	85 to 89	1.36	1.21	1.12	1.08	1.08	1.06	1.25	1.13
Argentina	85 to 89	1.16	1.09	1.12	1.11	0.75	0.95	1.09	1.03
Armenia	85 to 89	1.32	1.07	1.07	1.03	0.93	0.95	1.23	1.02
Australia	85 to 89	1.06	1.03	1.00	1.01	1.01	1.05	1.02	0.99
Austria	85 to 89	1.10	1.02	0.95	0.98	1.00	1.01	1.08	0.99
Azerbaijan	85 to 89	1.65	1.24	1.31	1.10	1.04	1.02	1.36	1.14
Bahamas	85 to 89	1.16	1.14	1.08	1.09	1.06	1.06	1.08	1.07
Bahrain	85 to 89	2.65	1.46	1.56	1.30	1.17	1.05	1.87	1.28
Bangladesh	85 to 89	1.35	1.54	1.08	1.18	0.95	0.95	1.21	1.38
Barbados	85 to 89	1.22	1.20	1.06	1.10	1.03	1.04	1.14	1.13
Belarus	85 to 89	1.16	1.23	1.08	1.18	1.03	1.07	1.10	1.16
Belgium	85 to 89	1.20	1.05	1.06	1.06	0.96	1.02	1.14	1.01
Belize	85 to 89	1.17	1.13	1.05	1.03	1.06	1.02	1.10	1.07
Benin	85 to 89	1.16	1.18	0.81	0.85	0.96	0.97	1.15	1.17
Bermuda	85 to 89	1.21	1.15	1.07	1.10	1.09	1.12	1.16	1.09
Bhutan	85 to 89	1.21	1.18	1.06	1.04	1.01	0.98	1.13	1.13
Bolivia (Plurinational State of)	85 to 89	1.22	1.19	1.02	1.03	0.97	0.94	1.16	1.13
Bosnia and Herzegovina	85 to 89	1.34	1.22	1.25	1.10	0.98	0.94	1.23	1.16
Botswana	85 to 89	1.25	1.18	1.05	1.03	1.04	1.04	1.17	1.13
Brazil	85 to 89	1.26	1.13	1.09	1.09	1.00	1.00	1.20	1.08
Brunei Darussalam	85 to 89	0.96	1.07	1.12	1.16	0.86	0.97	0.92	1.01
Bulgaria	85 to 89	1.80	1.55	1.67	1.14	0.96	0.83	1.48	1.43
Burkina Faso	85 to 89	1.29	1.27	1.02	0.99	1.09	1.05	1.26	1.24
Burundi	85 to 89	1.33	1.31	1.37	1.30	1.16	1.14	1.13	1.14
Cabo Verde	85 to 89	1.14	1.23	0.92	0.95	1.04	1.04	1.13	1.22
Cambodia	85 to 89	1.35	1.41	1.09	1.27	0.90	0.90	1.20	1.23
Cameroon	85 to 89	1.20	1.22	0.91	0.91	1.01	1.00	1.18	1.20
Canada	85 to 89	1.06	1.00	1.00	0.98	1.04	1.06	1.03	0.97
Central African Republic	85 to 89	1.32	1.39	1.09	1.11	1.07	1.07	1.17	1.22
Chad	85 to 89	1.19	1.22	0.90	0.90	1.01	0.99	1.17	1.21
Chile	85 to 89	1.15	1.11	1.11	1.07	0.88	0.97	1.09	1.07
China	85 to 89	1.79	1.54	1.01	4.39	0.00	1.14	1.25	1.26
Colombia	85 to 89	1.12	1.10	1.01	1.02	1.00	1.01	1.08	1.06
Comoros	85 to 89	1.23	1.23	1.05	1.05	1.09	1.09	1.11	1.13
Congo	85 to 89	1.24	1.21	0.95	0.93	1.05	1.04	1.14	1.14
Cook Islands	85 to 89	1.20	1.11	1.32	1.21	0.75	0.88	1.06	1.01
Costa Rica	85 to 89	1.10	1.10	1.02	1.03	1.03	1.05	1.07	1.06
Coted'Ivoire	85 to 89	1.20	1.22	0.93	0.91	1.00	1.00	1.17	1.20
Croatia	85 to 89	1.65	1.09	1.56	1.22	0.94	0.81	1.46	1.04
Cuba	85 to 89	1.21	1.18	1.05	1.09	1.03	1.03	1.15	1.12
Cyprus	85 to 89	186.02	1.22	1.96	1.02	0.45	0.95	4.02	1.21
Czechia	85 to 89	1.32	1.09	1.12	1.11	0.95	0.97	1.24	1.05
Democratic People's Republic of Korea	85 to 89	1.25	1.21	1.59	1.42	0.88	0.94	1.08	1.08
Democratic Republic of the Congo	85 to 89	1.26	1.34	1.02	1.11	1.08	1.09	1.15	1.20
Denmark	85 to 89	1.09	1.10	1.01	1.06	0.98	0.98	1.05	1.05
Djibouti	85 to 89	1.25	1.25	1.12	1.07	1.12	1.10	1.12	1.15
Dominica	85 to 89	1.45	1.28	1.16	1.06	1.10	1.07	1.29	1.18
Dominican Republic	85 to 89	1.18	1.12	1.03	1.05	0.99	1.02	1.09	1.06

Ecuador	85 to 89	1.13	1.16	1.06	1.10	1.03	1.02	1.08	1.10
Egypt	85 to 89	0.00	0.37	0.00	0.64	0.96	0.80	0.00	0.28
El Salvador	85 to 89	1.13	1.11	1.03	1.02	1.03	1.02	1.08	1.07
Equatorial Guinea	85 to 89	1.28	1.20	1.09	1.01	1.09	1.06	1.15	1.12
Eritrea	85 to 89	1.28	1.35	1.16	1.28	1.15	1.13	1.11	1.17
Estonia	85 to 89	1.93	1.08	1.13	1.11	1.20	1.07	1.72	1.03
Eswatini	85 to 89	1.31	1.13	1.12	0.92	1.05	0.99	1.19	1.08
Ethiopia	85 to 89	1.25	1.22	1.17	1.14	1.13	1.10	1.07	1.10
Fiji	85 to 89	1.35	1.33	2.53	2.38	0.79	0.77	1.13	1.13
Finland	85 to 89	1.03	1.03	0.97	0.96	0.97	0.99	1.01	1.01
France	85 to 89	1.21	1.05	1.09	1.02	0.92	1.01	1.15	1.01
Gabon	85 to 89	1.23	1.23	1.12	1.05	1.09	1.07	1.11	1.13
Gambia	85 to 89	1.19	1.25	0.89	0.88	1.00	0.98	1.17	1.23
Georgia	85 to 89	2.09	2.57	1.36	1.26	0.95	0.92	1.71	2.34
Germany	85 to 89	1.16	1.04	1.05	1.00	0.96	1.02	1.11	1.00
Ghana	85 to 89	1.20	1.23	0.86	0.87	0.99	0.98	1.19	1.21
Greece	85 to 89	1.25	1.10	1.03	1.05	0.95	0.99	1.18	1.04
Greenland	85 to 89	1.13	1.11	1.01	1.02	0.70	0.90	1.08	1.06
Grenada	85 to 89	2.03	1.20	1.45	1.09	1.25	1.06	1.79	1.13
Guam	85 to 89	0.99	1.06	0.86	1.05	0.53	0.97	0.95	1.01
Guatemala	85 to 89	1.19	1.16	1.03	1.02	1.07	1.03	1.13	1.10
Guinea	85 to 89	1.16	1.18	0.87	0.86	0.99	0.98	1.15	1.18
Guinea-Bissau	85 to 89	1.23	1.29	0.86	0.82	0.99	0.95	1.22	1.29
Guyana	85 to 89	1.27	1.24	1.08	1.03	1.04	1.05	1.18	1.17
Haiti	85 to 89	1.43	1.37	1.14	1.12	1.03	0.99	1.22	1.21
Honduras	85 to 89	1.22	1.24	1.05	1.01	1.01	0.92	1.17	1.18
Hungary	85 to 89	1.35	1.15	1.20	1.10	0.99	1.00	1.25	1.10
Iceland	85 to 89	1.08	1.02	1.02	0.99	0.96	0.97	1.05	0.99
India	85 to 89	1.16	1.16	1.05	1.05	1.00	0.97	1.10	1.10
Indonesia	85 to 89	1.19	1.27	1.01	1.05	0.90	0.84	1.10	1.17
Iran (Islamic Republic of)	85 to 89	1.30	1.09	1.01	0.99	0.96	0.96	1.22	1.05
Iraq	85 to 89	1.41	1.50	1.12	1.12	1.11	1.06	1.31	1.38
Ireland	85 to 89	1.14	1.11	1.01	0.99	0.98	0.99	1.10	1.08
Israel	85 to 89	1.02	1.01	0.99	1.04	1.00	1.04	0.98	0.97
Italy	85 to 89	1.26	1.12	1.06	1.06	0.95	1.01	1.21	1.06
Jamaica	85 to 89	1.19	1.23	1.03	1.07	1.02	1.04	1.13	1.16
Japan	85 to 89	1.16	1.07	1.08	1.08	0.97	0.99	1.10	1.03
Jordan	85 to 89	1.06	0.97	0.96	0.93	1.04	1.03	1.01	0.96
Kazakhstan	85 to 89	1.52	1.64	1.08	1.20	0.93	0.77	1.45	1.48
Kenya	85 to 89	1.19	1.24	1.10	1.13	1.10	1.08	1.08	1.12
Kiribati	85 to 89	1.30	1.31	1.52	1.46	0.87	0.83	1.13	1.15
Kuwait	85 to 89	1.06	1.11	1.05	1.08	1.04	1.07	1.02	1.06
Kyrgyzstan	85 to 89	2.14	1.51	1.40	1.20	1.03	1.07	1.76	1.38
Lao People's Democratic Republic	85 to 89	1.31	1.29	1.13	1.10	0.90	0.92	1.17	1.18
Latvia	85 to 89	1.69	1.10	1.09	1.15	1.09	1.19	1.52	1.05
Lebanon	85 to 89	1.56	1.13	1.86	1.19	1.48	1.12	1.24	1.06
Lesotho	85 to 89	1.43	1.20	1.27	0.95	1.10	1.00	1.25	1.12
Liberia	85 to 89	1.17	1.20	0.85	0.84	0.99	0.99	1.17	1.20

Libya	85 to 89	1.28	1.27	1.09	1.00	1.07	1.00	1.19	1.21
Lithuania	85 to 89	1.21	1.17	1.00	1.08	1.04	1.11	1.16	1.11
Luxembourg	85 to 89	1.31	1.12	1.09	1.09	0.93	1.00	1.24	1.06
Madagascar	85 to 89	1.33	1.34	1.18	1.24	1.15	1.12	1.16	1.18
Malawi	85 to 89	1.22	1.29	1.02	1.10	1.07	1.07	1.12	1.17
Malaysia	85 to 89	1.11	1.05	1.03	0.97	0.98	0.89	1.05	0.99
Maldives	85 to 89	1.23	1.20	1.11	1.06	0.91	0.95	1.12	1.13
Mali	85 to 89	1.13	1.14	0.85	0.87	0.99	0.99	1.11	1.14
Malta	85 to 89	1.19	1.09	1.02	0.98	1.02	1.03	1.14	1.06
Marshall Islands	85 to 89	1.27	1.22	1.30	1.30	0.59	0.68	1.09	1.07
Mauritania	85 to 89	1.15	1.18	0.81	0.85	0.98	0.98	1.15	1.17
Mauritius	85 to 89	1.17	1.13	1.08	1.11	1.03	1.02	1.11	1.07
Mexico	85 to 89	1.08	1.08	0.93	1.03	0.96	0.99	1.05	1.04
Micronesia (Federated States of)	85 to 89	1.26	1.22	1.42	1.31	0.64	0.72	1.08	1.07
Monaco	85 to 89	1.17	1.09	1.07	1.02	0.94	0.97	1.12	1.05
Mongolia	85 to 89	2.29	1.36	2.30	1.34	1.05	0.63	1.21	1.14
Montenegro	85 to 89	1.39	2.00	1.56	2.78	0.95	0.81	1.13	1.31
Morocco	85 to 89	1.27	1.20	1.00	0.98	1.07	0.98	1.17	1.13
Mozambique	85 to 89	1.27	1.37	1.11	1.21	1.11	1.10	1.16	1.23
Myanmar	85 to 89	1.41	1.42	1.10	1.39	0.96	0.99	1.25	1.25
Namibia	85 to 89	1.27	1.29	1.02	1.06	1.03	1.03	1.20	1.21
Nauru	85 to 89	1.17	1.15	1.20	1.09	0.61	0.58	1.06	1.02
Nepal	85 to 89	1.28	1.31	1.08	1.09	1.01	1.01	1.18	1.23
Netherlands	85 to 89	1.08	1.10	1.04	1.05	0.98	1.03	1.04	1.05
New Zealand	85 to 89	1.10	1.06	1.06	1.09	1.00	1.00	1.06	1.02
Nicaragua	85 to 89	1.23	1.10	1.10	1.05	1.05	1.03	1.17	1.06
Niger	85 to 89	1.20	1.23	0.93	0.93	1.02	1.01	1.16	1.20
Nigeria	85 to 89	1.22	1.19	0.89	0.94	1.01	1.01	1.20	1.17
Niue	85 to 89	1.23	1.19	1.44	1.43	0.71	0.77	1.08	1.06
North Macedonia	85 to 89	1.49	1.47	1.29	1.12	0.94	0.61	1.36	1.35
Northern Mariana Islands	85 to 89	1.13	1.13	1.07	1.12	0.50	0.60	1.02	1.03
Norway	85 to 89	1.08	1.03	1.08	1.01	0.90	1.00	1.04	1.00
Oman	85 to 89	1.55	1.45	1.39	1.35	1.15	1.08	1.38	1.33
Pakistan	85 to 89	1.15	1.15	1.03	1.02	0.98	0.96	1.09	1.10
Palau	85 to 89	1.21	1.06	1.32	1.05	0.77	0.66	1.08	0.98
Palestine	85 to 89	3.43	1.39	1.09	1.01	1.09	1.06	4.81	1.37
Panama	85 to 89	1.16	1.15	1.04	1.08	1.01	1.03	1.12	1.10
Papua New Guinea	85 to 89	1.17	1.15	1.18	1.21	0.54	0.60	0.99	0.98
Paraguay	85 to 89	1.20	1.16	1.12	1.12	1.00	0.98	1.13	1.10
Peru	85 to 89	1.12	1.10	1.01	1.02	1.02	1.01	1.07	1.06
Philippines	85 to 89	1.16	1.14	0.78	1.01	0.86	0.97	1.07	1.06
Poland	85 to 89	1.76	1.14	1.20	1.15	1.01	0.98	1.58	1.08
Portugal	85 to 89	1.48	1.21	1.10	1.10	0.92	0.97	1.37	1.14
Puerto Rico	85 to 89	1.19	1.10	1.03	1.04	1.02	1.06	1.14	1.06
Qatar	85 to 89	1.29	1.18	1.78	1.17	1.01	1.29	1.09	1.09
Republic of Korea	85 to 89	1.17	1.09	1.20	1.10	0.89	0.98	1.09	1.04
Republic of Moldova	85 to 89	1.85	1.08	1.34	1.13	1.15	1.01	1.45	1.03
Romania	85 to 89	1.42	1.24	1.05	1.18	0.87	0.85	1.31	1.16

Russian Federation	85 to 89	1.01	1.42	1.24	1.09	1.00	0.83	1.01	1.34
Rwanda	85 to 89	1.36	1.33	1.39	1.39	1.15	1.13	1.13	1.16
Saint Kitts and Nevis	85 to 89	1.77	1.22	1.34	1.04	1.13	1.05	1.52	1.15
Saint Lucia	85 to 89	1.91	1.36	1.28	1.26	1.02	1.04	1.67	1.23
Saint Vincent and the Grenadines	85 to 89	1.53	1.32	1.35	1.13	1.11	1.06	1.31	1.22
Samoa	85 to 89	1.29	1.17	1.81	1.46	0.67	0.69	1.07	1.02
San Marino	85 to 89	1.11	1.01	0.99	0.96	0.99	1.00	1.08	0.99
Sao Tome and Principe	85 to 89	1.30	1.15	0.98	0.91	1.03	0.99	1.28	1.13
Saudi Arabia	85 to 89	1.50	1.12	1.19	1.19	1.06	1.08	1.33	1.03
Senegal	85 to 89	1.19	1.24	0.90	0.89	1.01	0.99	1.17	1.22
Serbia	85 to 89	1.90	1.45	1.34	1.32	0.80	0.96	1.64	1.34
Seychelles	85 to 89	1.23	1.14	1.08	1.01	1.00	0.97	1.16	1.08
Sierra Leone	85 to 89	1.19	1.18	0.88	0.86	0.99	0.99	1.18	1.17
Singapore	85 to 89	1.00	0.99	1.01	1.00	0.99	1.02	0.98	0.97
Slovakia	85 to 89	1.11	1.10	1.17	1.12	0.93	0.95	1.05	1.05
Slovenia	85 to 89	1.20	1.17	1.00	1.15	0.97	0.98	1.16	1.11
Solomon Islands	85 to 89	1.20	1.19	1.29	1.26	0.61	0.72	1.03	1.03
Somalia	85 to 89	1.36	1.39	1.39	1.41	1.18	1.16	1.11	1.15
South Africa	85 to 89	1.18	1.16	1.04	1.02	1.04	1.02	1.12	1.10
South Sudan	85 to 89	1.25	1.23	1.15	1.16	1.10	1.10	1.11	1.11
Spain	85 to 89	1.25	1.01	1.07	1.07	0.99	1.05	1.19	0.96
Sri Lanka	85 to 89	1.40	1.38	1.13	1.20	0.98	1.01	1.27	1.26
Sudan	85 to 89	1.47	1.23	1.18	0.99	1.22	1.00	1.22	1.15
Suriname	85 to 89	1.15	1.16	1.03	1.07	1.04	1.04	1.09	1.09
Sweden	85 to 89	1.06	1.02	0.97	0.98	0.90	0.96	1.03	1.00
Switzerland	85 to 89	1.15	1.04	0.98	0.96	0.94	1.01	1.11	1.01
Syrian Arab Republic	85 to 89	1.27	1.25	1.02	1.07	1.03	0.99	1.13	1.13
Taiwan (Province of China)	85 to 89	1.06	1.05	1.09	1.09	0.96	1.01	0.99	0.99
Tajikistan	85 to 89	1.55	1.47	1.05	1.04	0.93	0.88	1.42	1.38
Thailand	85 to 89	1.20	1.11	1.11	1.06	0.91	0.92	1.09	1.03
Timor-Leste	85 to 89	1.25	1.33	1.01	1.13	0.88	0.87	1.13	1.20
Togo	85 to 89	1.18	1.25	0.87	0.88	0.99	0.99	1.17	1.24
Tokelau	85 to 89	1.30	1.21	1.51	1.41	0.65	0.79	1.11	1.06
Tonga	85 to 89	1.14	1.11	1.16	1.13	0.82	0.82	1.06	1.03
Trinidad and Tobago	85 to 89	1.37	1.14	1.16	1.06	1.02	1.04	1.27	1.10
Tunisia	85 to 89	1.80	1.39	1.29	1.11	1.21	1.05	1.53	1.29
Turkey	85 to 89	1.21	1.16	1.06	1.04	1.23	1.09	1.12	1.09
Turkmenistan	85 to 89	1.39	1.45	1.11	1.21	1.00	1.01	1.30	1.31
Tuvalu	85 to 89	1.30	1.22	1.53	1.29	0.55	0.69	1.10	1.08
Uganda	85 to 89	1.30	1.25	1.44	1.30	1.16	1.11	1.12	1.11
Ukraine	85 to 89	1.34	1.41	1.14	1.18	0.72	0.91	1.24	1.31
United Arab Emirates	85 to 89	1.18	0.81	1.10	0.71	1.07	0.36	1.12	0.81
United Kingdom	85 to 89	1.17	1.07	1.01	1.02	0.95	0.98	1.13	1.03
United Republic of Tanzania	85 to 89	1.29	1.23	1.36	1.15	1.15	1.09	1.12	1.13
United States Virgin Islands	85 to 89	1.32	1.22	1.16	1.17	1.06	1.08	1.21	1.15
United States of America	85 to 89	1.07	1.03	1.02	0.99	0.99	1.02	1.03	0.99
Uruguay	85 to 89	1.18	1.14	1.12	1.11	0.80	0.94	1.13	1.09
Uzbekistan	85 to 89	1.36	1.39	1.10	1.12	1.09	1.03	1.23	1.32

Vanuatu	85 to 89	1.26	1.21	1.45	1.35	0.65	0.74	1.10	1.08
Venezuela (Bolivarian Republic of)	85 to 89	1.10	1.17	0.98	1.08	1.04	1.03	1.05	1.11
Viet Nam	85 to 89	1.60	1.60	1.26	1.29	0.98	0.94	1.35	1.39
Yemen	85 to 89	2.06	1.96	1.42	1.13	1.46	1.14	1.51	1.68
Zambia	85 to 89	1.31	1.34	1.21	1.23	1.13	1.10	1.12	1.16
Zimbabwe	85 to 89	1.30	1.29	1.08	0.95	1.04	0.99	1.19	1.19
Afghanistan	90 to 94	1.71	1.43	1.02	0.87	1.23	0.91	1.44	1.36
Albania	90 to 94	2.17	1.47	1.31	0.81	0.97	0.75	1.49	1.26
Algeria	90 to 94	0.25	0.74	0.09	0.19	0.39	0.09	0.29	0.80
American Samoa	90 to 94	1.13	1.08	0.97	0.96	0.71	0.74	1.07	1.03
Andorra	90 to 94	1.01	1.03	0.94	0.95	0.90	0.90	1.02	1.04
Angola	90 to 94	1.18	1.20	0.92	0.91	1.08	1.07	1.15	1.17
Antigua and Barbuda	90 to 94	1.27	1.10	0.97	0.95	1.04	1.01	1.23	1.09
Argentina	90 to 94	1.12	1.06	1.03	1.02	0.56	0.86	1.10	1.05
Armenia	90 to 94	1.29	0.98	0.94	0.94	0.85	0.81	1.28	0.98
Australia	90 to 94	1.02	1.00	0.94	0.95	0.94	0.98	1.02	1.00
Austria	90 to 94	1.06	1.00	0.95	0.97	0.97	0.98	1.08	1.01
Azerbaijan	90 to 94	2.26	1.20	1.11	0.97	1.00	0.98	1.92	1.19
Bahamas	90 to 94	1.08	1.08	0.93	0.99	1.02	1.02	1.08	1.07
Bahrain	90 to 94	2.57	1.39	0.94	0.99	0.86	0.89	2.05	1.28
Bangladesh	90 to 94	1.19	1.37	0.92	0.98	0.80	0.84	1.18	1.35
Barbados	90 to 94	1.14	1.13	0.89	0.98	0.96	1.01	1.14	1.11
Belarus	90 to 94	1.15	1.21	0.98	1.03	0.85	0.87	1.13	1.18
Belgium	90 to 94	1.16	1.02	0.97	0.98	0.88	0.92	1.15	1.02
Belize	90 to 94	1.13	1.07	0.96	0.91	1.03	0.99	1.12	1.07
Benin	90 to 94	1.10	1.13	0.79	0.82	0.95	0.97	1.15	1.18
Bermuda	90 to 94	1.17	1.11	0.99	1.03	1.06	1.08	1.16	1.10
Bhutan	90 to 94	1.18	1.13	0.98	0.97	0.95	0.93	1.16	1.13
Bolivia (Plurinational State of)	90 to 94	1.15	1.09	0.91	0.92	0.93	0.89	1.15	1.09
Bosnia and Herzegovina	90 to 94	1.25	1.14	0.97	0.96	0.82	0.87	1.21	1.14
Botswana	90 to 94	1.23	1.10	0.93	0.91	1.01	1.01	1.20	1.12
Brazil	90 to 94	1.18	1.07	0.98	0.99	0.97	0.96	1.17	1.07
Brunei Darussalam	90 to 94	0.93	1.10	1.09	1.17	0.83	0.95	0.89	1.02
Bulgaria	90 to 94	3.02	1.45	1.06	0.95	0.70	0.64	2.05	1.40
Burkina Faso	90 to 94	1.23	1.22	0.93	0.90	1.06	1.03	1.27	1.25
Burundi	90 to 94	1.16	1.19	0.94	0.95	1.10	1.08	1.09	1.12
Cabo Verde	90 to 94	1.11	1.20	0.89	0.92	1.04	1.04	1.15	1.24
Cambodia	90 to 94	1.33	1.45	0.86	0.92	0.77	0.78	1.26	1.33
Cameroon	90 to 94	1.17	1.19	0.87	0.87	1.01	1.00	1.21	1.24
Canada	90 to 94	1.03	0.97	0.98	0.97	0.98	0.99	1.03	0.98
Central African Republic	90 to 94	1.19	1.21	0.88	0.83	1.05	1.03	1.15	1.16
Chad	90 to 94	1.16	1.18	0.88	0.86	1.01	0.99	1.20	1.23
Chile	90 to 94	1.04	1.08	1.00	1.01	0.75	0.82	1.03	1.08
China	90 to 94	2.54	1.92	1.01	4.04	0.00	1.10	1.46	1.46
Colombia	90 to 94	1.05	1.05	0.93	0.98	0.98	0.99	1.06	1.05
Comoros	90 to 94	1.13	1.13	0.84	0.84	1.06	1.06	1.09	1.11
Congo	90 to 94	1.16	1.16	0.81	0.83	1.04	1.04	1.14	1.14
Cook Islands	90 to 94	1.16	1.07	1.05	1.04	0.76	0.87	1.09	1.03

Costa Rica	90 to 94	1.06	1.05	0.97	1.00	1.03	1.02	1.07	1.06
Coted'Ivoire	90 to 94	1.17	1.19	0.88	0.87	1.00	0.99	1.20	1.22
Croatia	90 to 94	1.75	1.07	1.19	1.01	0.69	0.64	1.54	1.06
Cuba	90 to 94	1.15	1.11	0.95	0.97	1.00	1.02	1.15	1.11
Cyprus	90 to 94	1.14	1.20	0.89	0.91	0.59	0.71	1.12	1.28
Czechia	90 to 94	1.32	1.05	0.98	1.00	0.80	0.88	1.28	1.06
Democratic People's Republic of Korea	90 to 94	1.19	1.16	1.10	0.99	0.75	0.84	1.09	1.09
Democratic Republic of the Congo	90 to 94	1.19	1.26	0.88	0.90	1.07	1.08	1.16	1.21
Denmark	90 to 94	1.06	1.08	0.93	0.99	0.92	0.89	1.06	1.07
Djibouti	90 to 94	1.15	1.15	0.88	0.86	1.09	1.06	1.11	1.13
Dominica	90 to 94	1.32	1.17	0.94	0.91	1.05	1.03	1.26	1.15
Dominican Republic	90 to 94	1.11	1.07	0.86	0.97	0.95	1.00	1.08	1.07
Ecuador	90 to 94	1.07	1.11	0.93	0.99	0.99	0.93	1.07	1.11
Egypt	90 to 94	0.00	0.36	0.00	0.59	0.58	0.61	0.00	0.29
El Salvador	90 to 94	1.07	1.07	0.96	0.98	1.00	1.00	1.08	1.07
Equatorial Guinea	90 to 94	1.18	1.14	0.91	0.92	1.07	1.06	1.14	1.13
Eritrea	90 to 94	1.14	1.20	0.88	0.87	1.08	1.08	1.09	1.13
Estonia	90 to 94	1.54	1.07	0.94	1.03	0.97	0.84	1.48	1.05
Eswatini	90 to 94	1.26	1.02	0.96	0.88	1.01	0.97	1.21	1.05
Ethiopia	90 to 94	1.11	1.13	0.91	0.94	1.08	1.07	1.05	1.09
Fiji	90 to 94	1.46	1.36	1.71	1.38	0.84	0.79	1.25	1.22
Finland	90 to 94	1.00	1.00	0.95	0.94	0.93	0.97	1.01	1.02
France	90 to 94	1.22	1.04	0.98	0.96	0.83	0.92	1.20	1.04
Gabon	90 to 94	1.15	1.16	0.95	0.92	1.08	1.07	1.11	1.13
Gambia	90 to 94	1.15	1.22	0.87	0.85	1.00	0.99	1.19	1.27
Georgia	90 to 94	2.46	1.91	1.14	1.04	0.88	0.93	2.28	1.95
Germany	90 to 94	1.12	1.03	0.98	0.97	0.89	0.96	1.11	1.03
Ghana	90 to 94	1.18	1.25	0.84	0.86	0.99	0.99	1.23	1.29
Greece	90 to 94	1.21	1.05	0.90	0.93	0.85	0.90	1.19	1.05
Greenland	90 to 94	1.13	1.08	0.96	0.95	0.57	0.76	1.13	1.08
Grenada	90 to 94	1.52	1.09	1.06	0.96	1.12	1.00	1.50	1.09
Guam	90 to 94	0.89	1.04	0.63	1.00	0.32	0.95	0.89	1.03
Guatemala	90 to 94	1.18	1.13	0.92	0.93	1.05	1.01	1.18	1.13
Guinea	90 to 94	1.13	1.16	0.85	0.84	1.00	0.98	1.18	1.21
Guinea-Bissau	90 to 94	1.17	1.23	0.84	0.78	0.99	0.95	1.22	1.29
Guyana	90 to 94	1.14	1.14	0.86	0.86	0.97	1.00	1.17	1.15
Haiti	90 to 94	1.30	1.24	0.93	0.92	0.97	0.93	1.25	1.21
Honduras	90 to 94	1.23	1.17	1.00	0.92	1.00	0.90	1.22	1.17
Hungary	90 to 94	1.31	1.08	0.95	0.99	0.89	0.92	1.26	1.09
Iceland	90 to 94	1.03	0.99	0.97	0.95	0.91	0.90	1.04	1.00
India	90 to 94	1.12	1.11	0.97	0.98	0.92	0.91	1.12	1.10
Indonesia	90 to 94	1.13	1.23	0.81	0.81	0.79	0.74	1.11	1.19
Iran (Islamic Republic of)	90 to 94	1.12	1.00	0.88	0.90	0.74	0.82	1.12	1.01
Iraq	90 to 94	1.30	1.44	0.96	0.97	1.00	0.97	1.29	1.40
Ireland	90 to 94	1.12	1.09	0.96	0.97	0.90	0.91	1.12	1.10
Israel	90 to 94	0.98	0.98	0.90	0.96	0.95	0.97	0.99	0.98
Italy	90 to 94	1.21	1.11	0.96	0.98	0.89	0.89	1.20	1.08
Jamaica	90 to 94	1.13	1.12	0.94	0.93	1.00	0.98	1.13	1.12

Japan	90 to 94	1.12	1.05	1.03	1.05	0.98	0.99	1.10	1.04
Jordan	90 to 94	0.99	0.87	0.86	0.82	0.99	0.98	0.99	0.90
Kazakhstan	90 to 94	1.38	1.34	0.94	0.89	0.81	0.55	1.43	1.34
Kenya	90 to 94	1.12	1.14	0.93	0.93	1.08	1.05	1.08	1.10
Kiribati	90 to 94	1.23	1.27	1.09	1.05	0.89	0.85	1.16	1.20
Kuwait	90 to 94	1.02	1.05	0.98	1.00	0.98	1.02	1.02	1.06
Kyrgyzstan	90 to 94	3.20	1.60	1.20	1.10	1.00	0.97	2.57	1.53
Lao People's Democratic Republic	90 to 94	1.29	1.28	0.93	0.90	0.78	0.83	1.21	1.23
Latvia	90 to 94	1.52	1.05	0.88	0.98	0.89	0.89	1.48	1.03
Lebanon	90 to 94	1.44	1.11	1.19	1.02	1.13	1.00	1.25	1.08
Lesotho	90 to 94	1.37	1.03	1.02	0.86	1.05	0.97	1.29	1.05
Liberia	90 to 94	1.15	1.19	0.84	0.82	0.99	0.99	1.20	1.24
Libya	90 to 94	1.17	1.13	0.94	0.88	0.98	0.89	1.16	1.15
Lithuania	90 to 94	1.15	1.13	0.92	0.97	0.87	0.86	1.16	1.12
Luxembourg	90 to 94	1.23	1.09	0.97	0.99	0.84	0.92	1.20	1.08
Madagascar	90 to 94	1.21	1.21	0.86	0.87	1.10	1.08	1.14	1.14
Malawi	90 to 94	1.12	1.10	0.81	0.77	1.03	1.00	1.10	1.09
Malaysia	90 to 94	1.07	1.01	0.95	0.90	0.92	0.84	1.06	1.00
Maldives	90 to 94	1.20	1.17	0.93	0.93	0.81	0.87	1.15	1.15
Mali	90 to 94	1.10	1.10	0.85	0.86	0.99	0.98	1.14	1.15
Malta	90 to 94	1.14	1.06	0.96	0.98	0.99	1.01	1.14	1.08
Marshall Islands	90 to 94	1.16	1.16	0.87	0.93	0.63	0.71	1.11	1.11
Mauritania	90 to 94	1.13	1.15	0.82	0.84	0.99	0.99	1.17	1.19
Mauritius	90 to 94	1.15	1.10	0.97	0.99	0.93	0.92	1.13	1.09
Mexico	90 to 94	1.03	1.03	0.88	0.96	0.93	0.96	1.04	1.04
Micronesia (Federated States of)	90 to 94	1.20	1.16	1.02	0.97	0.71	0.75	1.11	1.10
Monaco	90 to 94	1.15	1.08	0.99	0.99	0.86	0.92	1.13	1.08
Mongolia	90 to 94	1.63	1.27	1.02	1.16	0.55	0.27	1.21	1.15
Montenegro	90 to 94	1.32	1.56	1.09	0.93	0.87	0.63	1.16	1.26
Morocco	90 to 94	1.15	1.12	0.84	0.86	0.92	0.83	1.13	1.11
Mozambique	90 to 94	1.21	1.21	0.91	0.88	1.09	1.07	1.16	1.16
Myanmar	90 to 94	1.37	1.46	0.88	1.04	0.83	0.92	1.31	1.33
Namibia	90 to 94	1.25	1.14	0.91	0.87	1.01	0.99	1.24	1.15
Nauru	90 to 94	1.09	1.07	0.94	0.80	0.64	0.67	1.06	1.01
Nepal	90 to 94	1.23	1.24	0.98	0.98	0.93	0.92	1.21	1.24
Netherlands	90 to 94	1.06	1.10	0.99	1.00	0.90	0.95	1.06	1.09
New Zealand	90 to 94	1.06	1.04	1.03	1.00	0.93	0.93	1.05	1.04
Nicaragua	90 to 94	1.17	1.08	1.02	1.01	1.03	1.03	1.17	1.08
Niger	90 to 94	1.16	1.19	0.89	0.87	1.02	1.01	1.18	1.22
Nigeria	90 to 94	1.16	1.13	0.86	0.90	1.01	1.01	1.19	1.16
Niue	90 to 94	1.17	1.14	1.05	1.05	0.73	0.78	1.11	1.07
North Macedonia	90 to 94	1.36	1.22	1.08	0.84	0.91	0.47	1.32	1.20
Northern Mariana Islands	90 to 94	1.09	1.08	0.90	0.92	0.51	0.63	1.04	1.03
Norway	90 to 94	1.04	0.99	1.00	0.94	0.85	0.92	1.04	1.00
Oman	90 to 94	1.40	1.49	1.13	1.17	1.06	1.04	1.33	1.43
Pakistan	90 to 94	1.12	1.11	0.96	0.95	0.92	0.90	1.11	1.11
Palau	90 to 94	1.16	1.01	1.04	0.84	0.77	0.61	1.10	0.99
Palestine	90 to 94	2.67	1.38	0.93	0.92	0.99	0.99	5.69	1.47

Panama	90 to 94	1.11	1.10	0.98	1.02	1.01	1.00	1.12	1.10
Papua New Guinea	90 to 94	1.09	1.08	0.83	0.79	0.60	0.65	1.01	1.00
Paraguay	90 to 94	1.15	1.10	0.99	1.01	0.96	0.96	1.12	1.09
Peru	90 to 94	1.07	1.06	0.94	0.97	0.99	0.98	1.08	1.06
Philippines	90 to 94	1.00	1.06	0.27	0.81	0.66	0.91	1.00	1.05
Poland	90 to 94	1.73	1.07	1.00	1.01	0.93	0.88	1.58	1.06
Portugal	90 to 94	1.43	1.17	0.96	0.98	0.82	0.80	1.37	1.16
Puerto Rico	90 to 94	1.12	1.04	0.95	0.98	0.99	1.02	1.13	1.05
Qatar	90 to 94	1.15	1.20	0.99	1.05	1.01	1.22	1.02	1.13
Republic of Korea	90 to 94	1.18	1.07	1.07	1.05	0.77	0.93	1.12	1.06
Republic of Moldova	90 to 94	1.72	1.06	0.89	1.01	0.90	0.95	1.49	1.05
Romania	90 to 94	1.39	1.16	0.83	0.94	0.73	0.75	1.33	1.14
Russian Federation	90 to 94	#####	1.20	1.02	0.93	0.85	0.66	1.01	1.20
Rwanda	90 to 94	1.18	1.20	0.93	0.99	1.09	1.08	1.09	1.13
Saint Kitts and Nevis	90 to 94	2.26	1.12	1.14	0.91	1.15	1.02	2.04	1.13
Saint Lucia	90 to 94	1.90	1.40	0.97	1.07	0.94	0.98	1.92	1.32
Saint Vincent and the Grenadines	90 to 94	1.58	1.27	1.07	0.98	1.05	1.03	1.42	1.22
Samoa	90 to 94	1.21	1.11	1.15	0.99	0.71	0.70	1.09	1.02
San Marino	90 to 94	1.10	0.97	0.93	0.93	0.93	0.96	1.10	1.00
Sao Tome and Principe	90 to 94	1.46	1.15	0.97	0.88	1.05	1.00	1.51	1.18
Saudi Arabia	90 to 94	1.37	1.07	0.94	0.98	0.97	1.00	1.33	1.01
Senegal	90 to 94	1.15	1.21	0.87	0.85	1.01	0.99	1.18	1.24
Serbia	90 to 94	2.11	1.51	1.05	1.08	0.62	0.89	1.87	1.45
Seychelles	90 to 94	1.18	1.10	0.94	0.91	0.90	0.91	1.16	1.10
Sierra Leone	90 to 94	1.16	1.16	0.86	0.84	1.00	0.99	1.20	1.20
Singapore	90 to 94	0.98	0.99	1.01	1.02	0.99	1.03	0.98	1.00
Slovakia	90 to 94	1.03	1.05	0.92	0.97	0.83	0.85	1.02	1.04
Slovenia	90 to 94	1.07	1.16	0.93	1.03	0.87	0.90	1.09	1.15
Solomon Islands	90 to 94	1.13	1.12	0.96	0.95	0.66	0.74	1.05	1.06
Somalia	90 to 94	1.19	1.10	0.91	0.84	1.11	1.05	1.09	1.07
South Africa	90 to 94	1.17	1.13	0.96	0.92	1.03	0.99	1.16	1.11
South Sudan	90 to 94	1.13	1.13	0.90	0.91	1.06	1.06	1.09	1.09
Spain	90 to 94	1.19	0.99	0.97	1.00	0.93	0.95	1.18	0.97
Sri Lanka	90 to 94	1.35	1.36	0.84	0.96	0.91	0.95	1.29	1.30
Sudan	90 to 94	1.32	1.18	0.88	0.86	0.99	0.84	1.20	1.17
Suriname	90 to 94	1.09	1.04	0.93	0.91	1.01	0.99	1.09	1.04
Sweden	90 to 94	1.02	1.00	0.96	0.96	0.83	0.92	1.03	1.01
Switzerland	90 to 94	1.12	1.02	0.93	0.94	0.86	0.95	1.13	1.03
Syrian Arab Republic	90 to 94	1.16	1.12	0.84	0.84	0.92	0.87	1.12	1.09
Taiwan (Province of China)	90 to 94	1.01	1.01	0.99	1.01	0.92	0.92	0.98	0.98
Tajikistan	90 to 94	1.51	1.36	0.90	0.93	0.83	0.79	1.53	1.41
Thailand	90 to 94	1.16	1.07	0.95	0.96	0.65	0.82	1.10	1.04
Timor-Leste	90 to 94	1.23	1.34	0.86	0.86	0.80	0.78	1.18	1.27
Togo	90 to 94	1.15	1.23	0.85	0.85	0.99	0.99	1.20	1.27
Tokelau	90 to 94	1.21	1.14	1.04	1.06	0.67	0.79	1.13	1.05
Tonga	90 to 94	1.09	1.06	0.96	0.95	0.81	0.82	1.07	1.03
Trinidad and Tobago	90 to 94	1.22	1.10	0.96	0.98	0.96	0.98	1.17	1.10
Tunisia	90 to 94	1.38	1.36	0.92	0.95	0.98	0.92	1.33	1.34

Turkey	90 to 94	1.08	1.02	0.88	0.90	0.96	0.89	1.06	1.01
Turkmenistan	90 to 94	1.38	1.44	0.99	1.05	0.91	0.89	1.38	1.38
Tuvalu	90 to 94	1.23	1.16	0.99	0.92	0.61	0.71	1.14	1.11
Uganda	90 to 94	1.18	1.16	1.00	0.97	1.10	1.07	1.10	1.11
Ukraine	90 to 94	1.21	1.39	1.02	1.05	0.22	0.63	1.18	1.33
United Arab Emirates	90 to 94	1.14	0.97	1.00	0.87	0.94	0.63	1.13	0.98
United Kingdom	90 to 94	1.11	1.05	0.96	0.97	0.88	0.91	1.11	1.05
United Republic of Tanzania	90 to 94	1.17	1.13	0.99	0.90	1.11	1.05	1.10	1.11
United States Virgin Islands	90 to 94	1.25	1.27	0.99	1.10	1.01	1.05	1.20	1.23
United States of America	90 to 94	1.04	1.01	0.99	0.97	0.93	0.92	1.04	1.00
Uruguay	90 to 94	1.16	1.10	1.04	1.03	0.62	0.78	1.14	1.10
Uzbekistan	90 to 94	1.32	1.36	0.94	1.03	1.02	0.95	1.29	1.38
Vanuatu	90 to 94	1.18	1.16	1.00	0.97	0.69	0.75	1.12	1.11
Venezuela (Bolivarian Republic of)	90 to 94	1.05	1.07	0.92	0.97	1.03	1.00	1.06	1.07
Viet Nam	90 to 94	1.62	1.67	0.93	0.92	0.81	0.83	1.45	1.52
Yemen	90 to 94	1.62	1.74	0.95	0.86	1.11	0.91	1.41	1.69
Zambia	90 to 94	1.19	1.11	0.86	0.80	1.08	1.02	1.10	1.07
Zimbabwe	90 to 94	1.30	1.09	0.88	0.77	1.00	0.95	1.24	1.11
Afghanistan	95	1.59	1.21	1.42	1.08	2.32	1.18	1.59	1.31
Albania	95	1.87	1.43	2.13	1.27	1.52	1.04	1.66	1.40
Algeria	95	0.00	1.25	0.00	1.07	0.34	0.93	0.00	1.36
American Samoa	95	1.16	1.10	1.56	1.44	0.90	0.92	1.12	1.08
Andorra	95	1.02	1.03	1.02	1.02	0.97	0.98	1.08	1.09
Angola	95	1.16	1.15	1.14	1.10	1.19	1.15	1.19	1.18
Antigua and Barbuda	95	1.20	1.11	1.13	1.10	1.18	1.14	1.28	1.17
Argentina	95	1.11	1.03	1.19	1.10	0.71	0.95	1.16	1.08
Armenia	95	1.23	1.00	1.14	1.08	1.11	1.00	1.32	1.05
Australia	95	1.03	1.00	1.07	1.07	1.08	1.11	1.10	1.05
Austria	95	1.06	1.00	0.97	1.00	0.99	1.03	1.17	1.05
Azerbaijan	95	2.73	1.22	1.65	1.16	1.19	1.10	3.29	1.32
Bahamas	95	1.06	1.09	1.04	1.15	1.11	1.11	1.13	1.14
Bahrain	95	1.37	1.36	1.03	1.25	1.05	1.14	1.52	1.44
Bangladesh	95	1.15	1.47	1.12	1.33	1.10	1.24	1.22	1.58
Barbados	95	1.17	1.09	1.09	1.10	1.15	1.16	1.27	1.16
Belarus	95	1.21	1.21	1.20	1.26	1.27	1.38	1.27	1.27
Belgium	95	1.16	1.03	1.10	1.10	1.00	1.06	1.24	1.08
Belize	95	1.11	1.07	1.09	1.05	1.11	1.03	1.18	1.14
Benin	95	1.09	1.11	0.95	0.95	1.06	1.05	1.20	1.21
Bermuda	95	1.15	1.09	1.10	1.10	1.16	1.16	1.23	1.14
Bhutan	95	1.17	1.12	1.15	1.10	1.17	1.08	1.23	1.18
Bolivia (Plurinational State of)	95	1.15	1.05	1.08	1.05	1.06	1.02	1.23	1.12
Bosnia and Herzegovina	95	1.17	1.13	1.18	1.12	1.13	1.08	1.24	1.19
Botswana	95	1.19	1.08	1.11	1.04	1.10	1.07	1.24	1.14
Brazil	95	1.14	1.04	1.10	1.07	1.03	1.02	1.22	1.10
Brunei Darussalam	95	0.97	1.09	1.67	1.45	1.01	1.04	1.00	1.12
Bulgaria	95	1.21	1.49	1.18	1.28	1.01	1.05	1.27	1.56
Burkina Faso	95	1.18	1.16	1.06	1.03	1.16	1.10	1.29	1.27
Burundi	95	1.15	1.16	1.25	1.21	1.27	1.22	1.15	1.17

Cabo Verde	95	1.12	1.17	1.03	1.05	1.11	1.09	1.22	1.27
Cambodia	95	1.38	1.47	1.30	1.48	1.11	1.05	1.38	1.46
Cameroon	95	1.15	1.12	1.03	0.99	1.11	1.06	1.26	1.23
Canada	95	1.03	0.97	1.04	1.03	1.09	1.12	1.09	1.02
Central African Republic	95	1.17	1.14	1.15	1.07	1.20	1.14	1.19	1.18
Chad	95	1.14	1.16	1.02	1.01	1.10	1.08	1.25	1.28
Chile	95	0.98	0.98	1.02	1.00	0.67	0.72	1.04	1.05
China	95	1.33	1.56	2.84	1.01	1.20	1.45	1.23	1.41
Colombia	95	1.04	1.03	1.06	1.07	1.05	1.06	1.10	1.08
Comoros	95	1.13	1.12	1.11	1.09	1.20	1.16	1.14	1.15
Congo	95	1.15	1.13	1.10	1.05	1.17	1.13	1.19	1.18
Cook Islands	95	1.17	1.08	1.85	1.40	0.97	0.99	1.13	1.06
Costa Rica	95	1.05	1.04	1.05	1.07	1.06	1.08	1.11	1.10
Coted'Ivoire	95	1.15	1.16	1.02	1.01	1.08	1.06	1.25	1.26
Croatia	95	1.73	1.09	1.76	1.25	1.18	0.99	1.71	1.15
Cuba	95	1.08	1.10	1.03	1.08	1.04	1.08	1.17	1.18
Cyprus	95	0.74	1.25	0.81	1.03	0.00	0.83	0.91	1.51
Czechia	95	1.35	1.05	1.18	1.11	1.03	1.02	1.42	1.10
Democratic People's Republic of Korea	95	1.17	1.16	1.69	1.74	1.17	1.11	1.14	1.14
Democratic Republic of the Congo	95	1.18	1.19	1.12	1.11	1.20	1.18	1.21	1.22
Denmark	95	1.07	1.06	1.05	1.09	1.01	1.02	1.13	1.12
Djibouti	95	1.14	1.12	1.13	1.08	1.21	1.15	1.15	1.15
Dominica	95	1.30	1.13	1.17	1.06	1.25	1.15	1.42	1.22
Dominican Republic	95	1.10	1.09	1.04	1.10	1.06	1.05	1.16	1.15
Ecuador	95	1.04	1.11	0.99	1.12	1.00	1.04	1.11	1.17
Egypt	95	2.21	1.05	1.83	1.04	2.15	1.08	2.22	1.14
El Salvador	95	1.05	1.06	1.06	1.08	1.05	1.06	1.11	1.11
Equatorial Guinea	95	1.15	1.11	1.16	1.07	1.19	1.12	1.17	1.15
Eritrea	95	1.13	1.15	1.16	1.13	1.22	1.19	1.14	1.16
Estonia	95	1.40	1.12	1.19	1.30	1.37	1.72	1.50	1.17
Eswatini	95	1.20	1.08	1.15	1.08	1.11	1.06	1.25	1.14
Ethiopia	95	1.11	1.10	1.18	1.11	1.21	1.15	1.10	1.12
Fiji	95	1.14	1.10	1.51	1.29	1.00	0.89	1.13	1.12
Finland	95	0.99	0.99	1.01	0.97	0.93	0.98	1.06	1.06
France	95	1.21	1.06	1.13	1.09	0.96	1.07	1.29	1.11
Gabon	95	1.12	1.13	1.18	1.11	1.17	1.14	1.14	1.15
Gambia	95	1.14	1.15	1.00	0.99	1.09	1.06	1.24	1.26
Georgia	95	5.83	1.45	2.61	1.25	1.27	1.33	7.05	1.57
Germany	95	1.17	1.03	1.12	1.07	1.02	1.09	1.23	1.08
Ghana	95	1.17	1.20	0.99	1.00	1.08	1.06	1.31	1.32
Greece	95	1.19	0.96	1.07	0.90	0.94	0.91	1.28	1.04
Greenland	95	1.14	1.08	1.08	1.05	0.91	0.93	1.21	1.15
Grenada	95	1.29	0.99	1.13	1.00	1.20	1.04	1.42	1.06
Guam	95	1.03	1.05	1.11	1.12	0.82	1.02	1.04	1.07
Guatemala	95	1.13	1.11	1.02	1.03	1.09	1.06	1.21	1.17
Guinea	95	1.12	1.11	1.00	0.97	1.09	1.05	1.23	1.23
Guinea-Bissau	95	1.16	1.15	1.01	0.97	1.10	1.05	1.28	1.28
Guyana	95	1.04	1.05	0.97	0.97	1.04	1.09	1.13	1.15

Haiti	95	1.30	1.18	1.16	1.09	1.24	1.11	1.47	1.29
Honduras	95	1.24	1.13	1.19	1.08	1.11	1.03	1.31	1.20
Hungary	95	1.29	1.09	1.21	1.10	1.07	1.06	1.35	1.15
Iceland	95	1.01	1.01	1.03	1.05	0.99	1.03	1.08	1.07
India	95	1.13	1.09	1.12	1.11	1.13	1.06	1.19	1.15
Indonesia	95	1.14	1.22	1.08	1.16	1.02	0.96	1.18	1.26
Iran (Islamic Republic of)	95	1.07	0.97	1.01	1.00	0.91	0.96	1.18	1.05
Iraq	95	1.26	1.38	1.15	1.18	1.15	1.13	1.37	1.49
Ireland	95	1.12	1.08	1.05	1.05	1.01	1.01	1.21	1.16
Israel	95	0.99	0.98	1.05	1.06	1.02	1.06	1.04	1.03
Italy	95	1.21	1.09	1.10	1.14	0.99	1.06	1.29	1.14
Jamaica	95	1.10	1.10	1.09	1.07	1.07	1.05	1.18	1.18
Japan	95	1.08	1.02	1.14	1.09	1.03	1.00	1.13	1.06
Jordan	95	1.04	0.89	1.01	0.93	1.08	1.06	1.11	0.97
Kazakhstan	95	1.28	1.22	1.10	0.97	0.99	0.80	1.43	1.36
Kenya	95	1.12	1.11	1.15	1.11	1.19	1.14	1.13	1.13
Kiribati	95	1.28	1.36	2.17	2.58	1.13	1.09	1.21	1.28
Kuwait	95	1.03	1.04	1.11	1.10	1.08	1.09	1.08	1.10
Kyrgyzstan	95	20.07	2.04	3.37	1.47	1.96	1.70	14.67	2.21
Lao People's Democratic Republic	95	1.34	1.31	1.34	1.25	1.14	1.04	1.35	1.35
Latvia	95	1.46	1.01	1.13	1.15	1.25	1.30	1.58	1.09
Lebanon	95	1.36	1.14	1.54	1.20	1.79	1.19	1.34	1.18
Lesotho	95	1.25	1.09	1.20	1.08	1.15	1.08	1.29	1.15
Liberia	95	1.13	1.15	0.99	0.97	1.08	1.07	1.25	1.28
Libya	95	1.13	1.11	1.08	1.04	1.12	1.05	1.21	1.19
Lithuania	95	1.19	1.14	1.09	1.20	1.21	1.55	1.28	1.21
Luxembourg	95	1.19	1.11	1.13	1.16	0.97	1.08	1.27	1.16
Madagascar	95	1.19	1.15	1.17	1.12	1.28	1.20	1.19	1.17
Malawi	95	1.12	1.08	1.07	1.04	1.17	1.11	1.15	1.13
Malaysia	95	1.07	0.99	1.12	0.99	1.10	0.93	1.11	1.03
Maldives	95	1.18	1.15	1.18	1.13	1.10	1.04	1.21	1.20
Mali	95	1.11	1.07	1.01	1.00	1.10	1.06	1.21	1.16
Malta	95	1.17	1.07	1.12	1.06	1.06	1.06	1.26	1.14
Marshall Islands	95	1.17	1.25	1.30	1.78	0.90	0.98	1.15	1.18
Mauritania	95	1.11	1.11	0.96	0.97	1.07	1.05	1.22	1.22
Mauritius	95	1.15	1.09	1.16	1.13	1.12	1.13	1.20	1.13
Mexico	95	1.06	1.01	1.05	1.02	1.02	1.01	1.12	1.06
Micronesia (Federated States of)	95	1.25	1.21	1.83	1.68	1.06	0.99	1.17	1.17
Monaco	95	1.16	1.07	1.13	1.08	1.02	1.02	1.23	1.14
Mongolia	95	1.32	1.26	1.35	1.52	1.47	0.86	1.22	1.19
Montenegro	95	1.37	1.13	1.60	1.09	1.10	0.91	1.29	1.16
Morocco	95	1.13	1.17	1.03	1.02	1.13	1.02	1.22	1.29
Mozambique	95	1.19	1.13	1.18	1.11	1.25	1.17	1.21	1.17
Myanmar	95	1.44	1.53	1.31	1.59	1.15	1.17	1.47	1.53
Namibia	95	1.19	1.09	1.08	1.04	1.09	1.06	1.26	1.16
Nauru	95	1.12	1.13	1.38	1.85	0.91	0.92	1.11	1.07
Nepal	95	1.22	1.21	1.17	1.14	1.20	1.12	1.29	1.29
Netherlands	95	1.07	1.09	1.10	1.10	1.02	1.13	1.12	1.15

New Zealand	95	1.05	1.05	1.14	1.18	1.08	1.16	1.10	1.09
Nicaragua	95	1.16	1.07	1.16	1.10	1.11	1.03	1.22	1.12
Niger	95	1.14	1.17	1.05	1.03	1.13	1.10	1.23	1.28
Nigeria	95	1.13	1.09	1.02	1.01	1.09	1.05	1.23	1.17
Niue	95	1.19	1.18	1.77	2.09	0.97	1.00	1.15	1.13
North Macedonia	95	1.31	1.27	1.33	1.29	1.25	1.10	1.35	1.32
Northern Mariana Islands	95	1.12	1.11	1.41	1.39	0.69	0.83	1.08	1.07
Norway	95	1.03	0.98	1.09	1.03	0.99	1.05	1.09	1.04
Oman	95	1.38	1.25	1.36	1.23	1.27	1.14	1.42	1.31
Pakistan	95	1.12	1.11	1.12	1.09	1.13	1.06	1.18	1.17
Palau	95	1.22	1.14	1.80	1.69	0.99	0.86	1.17	1.12
Palestine	95	1.31	1.23	1.01	1.04	1.06	1.08	1.69	1.40
Panama	95	1.09	1.08	1.10	1.11	1.06	1.07	1.14	1.13
Papua New Guinea	95	1.15	1.09	1.56	1.46	0.90	0.86	1.08	1.04
Paraguay	95	1.16	1.08	1.17	1.13	1.07	1.04	1.22	1.14
Peru	95	1.08	1.04	1.08	1.05	1.06	1.03	1.14	1.10
Philippines	95	1.22	1.04	1.24	0.93	1.06	1.03	1.24	1.09
Poland	95	1.39	1.07	1.24	1.25	1.13	1.05	1.47	1.12
Portugal	95	1.26	1.18	1.12	1.16	0.99	0.99	1.34	1.25
Puerto Rico	95	1.14	1.04	1.09	1.05	1.13	1.08	1.22	1.09
Qatar	95	0.99	1.23	0.98	1.28	1.01	1.96	1.04	1.26
Republic of Korea	95	1.29	1.08	1.73	1.17	0.93	1.02	1.29	1.13
Republic of Moldova	95	1.97	1.10	1.43	1.26	1.38	1.15	1.94	1.15
Romania	95	1.37	1.16	1.18	1.16	1.00	1.00	1.45	1.23
Russian Federation	95	1.01	1.19	1.60	1.15	1.39	0.99	1.01	1.27
Rwanda	95	1.17	1.14	1.29	1.17	1.28	1.18	1.15	1.15
Saint Kitts and Nevis	95	1.17	1.13	1.09	1.05	1.14	1.18	1.27	1.23
Saint Lucia	95	1.12	1.45	1.03	1.30	1.06	1.10	1.24	1.57
Saint Vincent and the Grenadines	95	1.41	1.27	1.30	1.21	1.12	1.16	1.51	1.34
Samoa	95	1.22	1.12	2.61	2.48	1.01	0.91	1.13	1.03
San Marino	95	1.12	1.03	1.04	1.01	1.01	1.01	1.19	1.10
Sao Tome and Principe	95	1.13	1.14	1.03	1.00	1.09	1.05	1.24	1.24
Saudi Arabia	95	1.31	1.09	1.14	1.17	1.10	1.12	1.42	1.13
Senegal	95	1.13	1.15	1.00	0.99	1.09	1.07	1.23	1.25
Serbia	95	1.40	1.39	1.14	1.25	0.73	1.16	1.51	1.45
Seychelles	95	1.16	1.09	1.15	1.06	1.06	1.02	1.21	1.14
Sierra Leone	95	1.14	1.13	1.00	0.98	1.08	1.07	1.25	1.25
Singapore	95	0.98	1.00	1.06	1.07	1.00	1.05	1.04	1.05
Slovakia	95	1.01	1.04	1.15	1.14	1.05	1.03	1.06	1.09
Slovenia	95	1.05	1.17	1.06	1.19	1.00	1.07	1.12	1.22
Solomon Islands	95	1.18	1.17	1.54	1.53	0.97	0.97	1.11	1.12
Somalia	95	1.17	1.09	1.25	1.09	1.28	1.15	1.15	1.12
South Africa	95	1.14	1.08	1.09	1.04	1.07	1.05	1.19	1.14
South Sudan	95	1.12	1.10	1.13	1.11	1.17	1.15	1.13	1.12
Spain	95	1.18	1.00	1.13	1.14	1.04	1.17	1.25	1.04
Sri Lanka	95	1.34	1.44	1.18	1.40	1.07	1.07	1.40	1.47
Sudan	95	1.28	1.16	1.12	1.01	1.36	1.04	1.35	1.27
Suriname	95	1.10	1.05	1.07	1.05	1.10	1.07	1.17	1.11

Sweden	95	1.01	0.99	1.01	1.01	0.90	0.98	1.08	1.05
Switzerland	95	1.13	1.01	1.02	0.99	0.98	1.05	1.22	1.08
Syrian Arab Republic	95	1.18	1.26	1.11	1.19	1.14	1.09	1.25	1.33
Taiwan (Province of China)	95	1.02	1.00	1.52	1.15	1.08	1.07	1.00	1.00
Tajikistan	95	1.56	1.31	1.20	1.14	1.11	1.01	1.80	1.45
Thailand	95	1.15	1.06	1.24	1.11	1.37	1.02	1.16	1.08
Timor-Leste	95	1.24	1.37	1.22	1.31	1.10	1.05	1.27	1.40
Togo	95	1.14	1.19	1.00	0.99	1.09	1.07	1.26	1.32
Tokelau	95	1.25	1.11	2.01	1.74	0.98	0.98	1.17	1.07
Tonga	95	1.10	1.08	1.26	1.29	0.96	0.95	1.10	1.08
Trinidad and Tobago	95	1.15	1.09	1.08	1.07	1.10	1.09	1.22	1.15
Tunisia	95	1.34	1.22	1.14	1.09	1.22	1.08	1.47	1.33
Turkey	95	1.07	1.01	1.05	1.01	1.25	1.05	1.13	1.07
Turkmenistan	95	1.41	1.42	1.21	1.31	1.14	1.25	1.54	1.49
Tuvalu	95	1.29	1.22	2.18	1.71	1.00	0.93	1.20	1.18
Uganda	95	1.16	1.11	1.28	1.13	1.26	1.15	1.15	1.13
Ukraine	95	1.25	1.28	1.26	1.29	1.57	1.45	1.32	1.33
United Arab Emirates	95	1.14	1.02	1.14	1.04	1.20	0.87	1.20	1.09
United Kingdom	95	1.03	1.04	1.01	1.07	0.95	1.06	1.10	1.09
United Republic of Tanzania	95	1.15	1.09	1.25	1.07	1.24	1.13	1.15	1.13
United States Virgin Islands	95	1.33	1.31	1.23	1.29	1.22	1.16	1.42	1.38
United States of America	95	1.02	1.01	1.05	1.02	1.03	1.04	1.07	1.05
Uruguay	95	1.16	1.06	1.16	1.10	0.77	0.88	1.23	1.13
Uzbekistan	95	1.38	1.42	1.20	1.24	1.31	1.22	1.47	1.56
Vanuatu	95	1.24	1.21	1.83	1.74	0.99	0.98	1.18	1.17
Venezuela (Bolivarian Republic of)	95	1.05	1.05	1.06	1.09	1.07	1.06	1.10	1.10
Viet Nam	95	1.66	1.78	1.56	1.56	1.21	1.09	1.62	1.78
Yemen	95	1.48	1.47	1.29	1.06	1.76	1.22	1.53	1.70
Zambia	95	1.15	1.08	1.20	1.07	1.25	1.13	1.14	1.11
Zimbabwe	95	1.23	1.07	1.10	1.01	1.13	1.06	1.28	1.13

ICH, intracerebral hemorrhage; SAH, subarachnoid hemorrhage; IS, ischemic stroke; GDR, gender disparity ratio

Table S21 The number of countries for GDR values near one for stroke, ICH, SAH, and IS in different age groups in 1990 and 2021

GDR range	cause	year	<5	5 to 9	10 to 14	15 to 19	20 to 24	25 to 29	30 to 34	35 to 39	40 to 44	45 to 49	50 to 54	55 to 59	60 to 64	65 to 69	70 to 74	75 to 79	80 to 84	85 to 89	90 to 94	95+
<0.95	storke	1990	2	18	79	19	57	23	24	9	16	13	12	22	25	18	1	0	1	2	5	2
		2021	0	5	46	12	33	8	10	5	8	6	5	9	11	9	1	1	2	2	3	1
	ICH	1990	8	31	89	59	174	44	21	6	3	4	11	12	5	39	8	6	33	24	106	2
		2021	2	16	57	50	156	34	9	5	2	4	9	21	5	32	7	7	35	23	101	3
	SAH	1990	8	90	1	13	62	26	34	25	52	4	60	52	81	58	3	1	13	53	104	18
		2021	4	26	8	17	40	15	21	16	31	7	46	51	57	46	6	1	11	49	103	20
	IS	1990	30	4	8	25	6	30	23	21	17	24	35	37	107	29	4	1	1	4	4	2
		2021	2	1	6	19	2	11	5	4	6	8	22	25	79	23	2	1	2	2	3	0
[0.95,1.05]	storke	1990	110	160	113	24	102	58	58	20	23	29	23	36	39	28	6	2	7	7	19	25
		2021	164	183	145	35	121	78	64	27	33	41	39	45	46	40	6	2	16	16	35	43
	ICH	1990	152	154	107	73	25	58	34	34	5	9	24	37	15	52	19	11	59	52	78	44

		2021	191	183	139	97	38	72	51	42	18	19	32	37	31	51	37	14	68	56	89	56
	**SAH**	1990	130	79	53	40	88	80	47	55	69	30	78	74	75	76	53	8	66	100	70	46
		2021	160	152	103	72	113	90	58	69	103	35	106	91	107	93	50	20	76	106	82	59
	**IS**	1990	147	98	48	40	23	65	49	29	30	49	60	46	41	54	12	3	17	21	22	8
		2021	182	142	92	69	34	74	55	37	46	70	80	59	71	63	29	13	37	47	46	13
**>1.05**	**storke**	1990	92	26	12	161	45	123	122	175	165	162	169	146	140	158	197	202	196	195	180	177
		2021	40	16	13	157	50	118	130	172	163	157	160	150	147	155	197	201	186	186	166	160
	**ICH**	1990	44	19	8	72	5	102	149	164	196	191	169	155	184	113	177	187	112	128	20	158
		2021	11	5	8	57	10	98	144	157	184	181	163	146	168	121	160	183	101	125	14	145
	**SAH**	1990	66	35	150	151	54	98	123	124	83	170	66	78	48	70	148	195	125	51	30	140
		2021	40	26	93	115	51	99	125	119	70	162	52	62	40	65	148	183	117	49	19	125
	**IS**	1990	27	102	148	139	175	109	132	154	157	131	109	121	56	121	188	200	186	179	178	194
		2021	20	61	106	116	168	119	144	163	152	126	102	120	54	118	173	190	165	155	155	191

ICH, intracerebral hemorrhage; SAH, subarachnoid hemorrhage; IS, ischemic stroke; GDR, gender disparity ratio

Table S22 The ASPR, ASIR, ASMR, and DALYs rate of stroke and its subtypes in China in 2021

measure	location	cause	
Deaths	China	Ischemic stroke	64.5(54.0, 74.8)
Deaths	China	Intracerebral hemorrhage	68.8(57.6, 81.2)
Deaths	China	Subarachnoid hemorrhage	4.7(3.4, 6.0)
Deaths	China	Stroke	138.0(116.7, 160.3)
DALYs (Disability-Adjusted Life Years)	China	Stroke	2648.0(2253.4, 3076.9)
DALYs (Disability-Adjusted Life Years)	China	Ischemic stroke	1181.0(1009.7, 1356.7)
DALYs (Disability-Adjusted Life Years)	China	Intracerebral hemorrhage	1351.6(1129.1, 1600.9)
DALYs (Disability-Adjusted Life Years)	China	Subarachnoid hemorrhage	115.5(86.9, 142.5)
Prevalence	China	Stroke	1301.4(1200.6, 1405.7)
Prevalence	China	Ischemic stroke	1018.8(918.5, 1123.4)
Prevalence	China	Subarachnoid hemorrhage	68.9(61.5, 76.9)
Prevalence	China	Intracerebral hemorrhage	222.1(200.1, 246.5)
Incidence	China	Stroke	204.8(181.0, 231.5)
Incidence	China	Ischemic stroke	135.8(113.3, 159.8)
Incidence	China	Intracerebral hemorrhage	61.2(53.0, 69.1)
Incidence	China	Subarachnoid hemorrhage	7.8(6.9, 8.9)

Table S23 The EAPC results in China from 1990 to 2021

**location**	**measure**	**sex**	**cause**	**age**	**EAPC_95CI**
China	Deaths	Both	Stroke	Age-standardized	-1.92(-2.16 to -1.68)
China	Deaths	Both	Ischemic stroke	Age-standardized	-0.47(-0.73 to -0.22)
China	Deaths	Both	Intracerebral hemorrhage	Age-standardized	-2.38(-2.73 to -2.04)
China	Deaths	Both	Subarachnoid hemorrhage	Age-standardized	-6.66(-7.32 to -6.01)
China	DALYs	Both	Stroke	Age-standardized	-2.05(-2.24 to -1.87)
China	DALYs	Both	Ischemic stroke	Age-standardized	-0.5(-0.69 to -0.32)
China	DALYs	Both	Intracerebral hemorrhage	Age-standardized	-2.47(-2.78 to -2.17)
China	DALYs	Both	Subarachnoid hemorrhage	Age-standardized	-6.29(-6.89 to -5.69)
China	Prevalence	Both	Stroke	Age-standardized	0.34(0.31 to 0.38)
China	Prevalence	Both	Ischemic stroke	Age-standardized	1.02(0.96 to 1.08)
China	Prevalence	Both	Intracerebral hemorrhage	Age-standardized	-1.26(-1.37 to -1.15)
China	Prevalence	Both	Subarachnoid hemorrhage	Age-standardized	-1.65(-1.76 to -1.54)
China	Incidence	Both	Stroke	Age-standardized	-0.6(-0.72 to -0.48)
China	Incidence	Both	Ischemic stroke	Age-standardized	0.94(0.88 to 1)
China	Incidence	Both	Intracerebral hemorrhage	Age-standardized	-2.24(-2.5 to -1.98)
China	Incidence	Both	Subarachnoid hemorrhage	Age-standardized	-3.7(-4.08 to -3.31)

EAPC, Estimated Annual Percentage Change
