# Supplementary material for: Quality of care index and gender disparity ratio for stroke and its subtypes from the Global Burden of Disease Study 2021
Source: PLoS One. 2026 Apr 29;21(4):e0345938. doi: 10.1371/journal.pone.0345938 (PMC13127941; doi:10.1371/journal.pone.0345938)
Supplement: Attachment — Submitted filename: Respond to reviewers.docx [file pone.0345938.s005.docx]

**Response to the Reviewers’ Comments**

Dear Reviewers,

We really appreciate all of your constructive notes. All of them have helped us improve the quality of this manuscript significantly. Please find our revised manuscript in the attachments where we have addressed your notes. In the following sections, we provide point-by-point responses to your concerns.

Best regards,

Jingjing An,

MD MHA

**ACADEMIC EDITOR:**

**1. 1. What has previously been published on this topic and what does this work add to the existing literature?**

**Response:** Many thanks for your precious advice.

We have added a content your mentioned in line 60-90 in manuscript-revised.

**2. GBD showed that The annual number of strokes and deaths due to stroke increased substantially from 1990 to 2021. Data on the burden of stroke in your county should be increased so that readers can understand the need for stroke-related studies. Additional citations could be added: Estimated Burden of Stroke in China in 2020. JAMA Netw Open. 2023;6(3): e231455.**

**Response:** Many thanks for your valuable advice.

We have added a content your mentioned in line 44-47 in manuscript-revised.

**3. This reviewer would expect to see some points regarding how to translate these observations to help address this public health concern.**

**Response:** Many thanks for your constructive advice.

We have added a content your mentioned in line 360-370 in manuscript-revised.

**Reviewers' comments:**

**Reviewer's Responses to Questions**

**Comments to the Author**

**1. Is the manuscript technically sound, and do the data support the conclusions?**

**The manuscript must describe a technically sound piece of scientific research with data that supports the conclusions. Experiments must have been conducted rigorously, with appropriate controls, replication, and sample sizes. The conclusions must be drawn appropriately based on the data presented.**

**Reviewer #1: Partly**

**Response:** Many thanks for your professional advice.

We have revised the manuscript carefully.

**2. Has the statistical analysis been performed appropriately and rigorously?**

**Reviewer #1: Yes**

**Response:** Thanks for your feedback! And many thanks for sharing your time and energy in reviewing our work.

**3. Have the authors made all data underlying the findings in their manuscript fully available?**

**The PLOS Data policy requires authors to make all data underlying the findings described in their manuscript fully available without restriction, with rare exception (please refer to the Data Availability Statement in the manuscript PDF file). The data should be provided as part of the manuscript or its supporting information, or deposited to a public repository. For example, in addition to summary statistics, the data points behind means, medians and variance measures should be available. If there are restrictions on publicly sharing data—e.g. participant privacy or use of data from a third party—those must be specified.**

**Reviewer #1: Yes**

**Response:** Thanks for your feedback! And many thanks for sharing your time and energy in reviewing our work.

**4. Is the manuscript presented in an intelligible fashion and written in standard English?**

**PLOS ONE does not copyedit accepted manuscripts, so the language in submitted articles must be clear, correct, and unambiguous. Any typographical or grammatical errors should be corrected at revision, so please note any specific errors here.**

**Reviewer #1: No**

**Response:** Many thanks for your professional advice.

We have polished our manuscript.

**5. Review Comments to the Author**

**Please use the space provided to explain your answers to the questions above. You may also include additional comments for the author, including concerns about dual publication, research ethics, or publication ethics. (Please upload your review as an attachment if it exceeds 20,000 characters)**

**Reviewer #1: General Comment**

**This manuscript presents an important analysis of the QCI and GDR for stroke and its major subtypes, leveraging data from the GBD Study 2021. The topic aligns well with current global health priorities, particularly the pursuit of equitable and effective care delivery. However, the manuscript would benefit from stronger conceptual framing, more precise reporting of methodological procedures, and clarification of some key terms and interpretations. Specific comments are presented.**

**Response:** Thanks for your feedback! And many thanks for sharing your time and energy in reviewing our work.

**Specific comments**

**Comment 1. The authors refer to the QCI as a composite index derived through PCA based on four secondary metrics. However, a clearer theoretical rationale for how this index captures “quality of care” is needed. What dimensions does it intend to reflect? How has it been validated across conditions or settings?**

**Response:** Many thanks for your professional advice.

We have added a content your mentioned in line 113-171 in manuscript-revised.

The Healthcare Access and Quality Index (HAQ) in 2019 has been provided in <https://ghdx.healthdata.org/record/ihme-data/gbd-2019-healthcare-access-and-quality-1990-2019>. However, the HAQ in 2021 is not available in public. Due to lack of the HAQ in 2021, we are unable to validate our results. We have added the limitation in line 378-379 in manuscript-revised.

**Comment 2: The GDR is defined as the ratio of female-to-male QCI. A ratio close to 1 is assumed to reflect equity, but this may oversimplify interpretability. What does a GDR >1 or <1 indicate in real-world terms? Consider illustrating implications with country-level examples where GDR diverges notably from parity.**

**Response:** We appreciate the reviewer’s insightful observation.

As defined in the study, the Gender Disparity Ratio (GDR) represents the ratio of female-to-male QCI. A value of 1 indicates parity between sexes, whereas values above 1 suggest relatively higher CKD QCI in women, and values below 1 indicate relatively higher CKD QCI in men. We have added a content your mentioned in line 172-175 and 247-286 in manuscript-revised.

**Comment 3: The analysis presents decreasing YLDs alongside increasing QCI and improved GDR. Please clarify how much of the improvement in QCI reflects actual system performance versus changes in stroke incidence/severity. The limitations of indirect attribution need emphasis.**

**Response:** We appreciate the reviewer’s insightful observation and valuable advice.

As shown in line 113-171 for QCI and line 171-175 in manuscript-revised, QCI and GDR reflects a comprehensive indicator to represent the difference in stroke care, comparing with prevalence, incidence, and mortality. Therefore, QCI and GDR reflects the whole comprehensive indicator, not a reflects the changes of prevalence, incidence, and mortality separately. In addition, the changes in stroke incidence/severity have been presented in line 181-195 in manuscript-revised.

The limitation has been added in line 377-378 in manuscript-revised.

**Comment 4: While the results are rich in numerical detail, the manuscript would benefit from a concise interpretive table outlining how to read QCI and GDR values, including thresholds for high/low quality or parity gaps. This would enhance accessibility for both technical and policy audiences.**

**Response:** We appreciate the reviewer’s insightful observation.

We have added a **Table 1** in manuscript-revised.

**Comment 5: Sentences such as “The situation intensifies in most regions and countries with the increase in age” (line 267) or “There was a positive association between QCI and SDI” (Abstract) are vague or grammatically unclear. Please clarify.**

**Response:** We appreciate the reviewer’s precious suggestions.

We have revised the content in manuscript-revised.

**Comment 6: While age-specific results are reported, the manuscript stops short of interpreting why older age groups exhibit different QCI or GDR patterns. Are care gaps among the elderly driven by biological vulnerability, health system neglect, or cultural inequities? A deeper exploration could enhance interpretive value.**

**Response:** We appreciate the reviewer’s constructive advice.

We have performed the analysis of QCI and GDR focusing on older age groups. The manuscript is submitting to another journal and reviewing.

**Comment 7: Phrases like “measures should be taken to improve disparities” (line 297) are too general. Consider offering more grounded recommendations, such as improving stroke recognition in women, investing in regional stroke units, or enhancing guideline adherence monitoring in low-SDI countries.**

**Response:** Many thanks for valuable suggestions.

We have added the content you suggested in line 384-387 in manuscript-revised in manuscript-revised.

**Comment 8: Given the emphasis on disparities, integrating a brief discussion of how health systems might inadvertently reinforce or mitigate inequities, especially in gender and aging populations, could enrich the policy relevance of the manuscript.**

**Response:** We appreciate the reviewer’s insightful observation.

We have added the content in line 360-370 in manuscript-revised.
