# Supplementary material for: Quality of care index and gender disparity ratio for stroke and its subtypes from the Global Burden of Disease Study 2021
Source: PLoS One. 2026 Apr 29;21(4):e0345938. doi: 10.1371/journal.pone.0345938 (PMC13127941; doi:10.1371/journal.pone.0345938)
Supplement: Attachment — Submitted filename: Respond_to_reviewers_auresp_2.docx [file pone.0345938.s006.docx]

**Response to the Editor’s Comments**

Dear Editor,

We really appreciate all of your constructive notes. All of them have helped us improve the quality of this manuscript significantly. Please find our revised manuscript in the attachments where we have addressed your notes. In the following sections, we provide point-by-point responses to your concerns.

Best regards,

Jingjing An,

MD MHA

**Additional Editor Comments:**

**The core metric of the study, QCI, is derived from principal component analysis (PCA) of four secondary indices (MIR, DPR, PIR, YLR) and primary GBD metrics. However:The study explicitly states that validation using the Healthcare Access and Quality (HAQ) Index—an established benchmark for healthcare quality—was not possible due to the unavailability of 2021 HAQ data. Without external validation, the QCI’s ability to accurately reflect "quality of care" (as defined by the WHO) remains unsubstantiated. The authors rely on internal PCA variance explanation but provide no evidence that QCI correlates with real-world indicators of care quality (e.g., adherence to stroke treatment guidelines, hospital readmission rates, or patient-reported outcomes).**

**Response:** Many thanks for your professional advice.

We agree that external validation against established healthcare quality metrics such as the Healthcare Access and Quality (HAQ) Index would further strengthen the interpretability of QCI. However, as noted in the manuscript, the HAQ Index data were not available for 2021, which prevented direct validation within the same study period.

Several points support the validity of QCI as a proxy for quality of care:

First, the QCI is constructed from four well-established epidemiological ratios, all of which have been widely used in previous studies as indirect indicators of healthcare effectiveness, survival, disease management, and disability burden ^[1,2,3,4]^. These metrics reflect different dimensions of care quality, including survival outcomes, disability prevention, and long-term disease management.

In addition, it is important to emphasize that the QCI primarily reflects health outcomes and does not directly measure structural or process-related dimensions of healthcare delivery, such as adherence to stroke treatment guidelines, hospital readmission rates, or patient-reported outcomes. Moreover, the index may be influenced by factors beyond the healthcare system itself, including underlying population health status, disease ecology, and the completeness and accuracy of disease surveillance systems. Due to the limited availability of comprehensive, longitudinal data covering multiple decades and diverse geographic settings, empirical validation of the QCI against direct and independent indicators of healthcare quality remains constrained. Therefore, future research should prioritize the validation and refinement of the QCI as more granular and reliable data on healthcare delivery become available, which will further enhance its robustness and utility as a proxy measure of healthcare quality.

We have mentioned in line 400-416 in manuscript-revised.

References

1. Xu C, Zhu L, Lai J, Luo Z, Ying J, Hu S, Song P, Yang J. Global and regional quality of care index in major depressive disorder: the global burden of disease study 2021. Int J Equity Health. 2026 Feb 7. doi: 10.1186/s12939-026-02775-5. Epub ahead of print. PMID: 41654951.
2. Xia Z, Wang J, Wen X. Global assessment of pain care quality for musculoskeletal disorders: Insights from the quality of care index (QCI) from 1990 to 2021. PLoS One. 2025 Oct 31;20(10):e0335235. doi: 10.1371/journal.pone.0335235. PMID: 41171723;
3. Lin S, Liu Q, Yin L, Liu W, Zhu X, Shen Y, Li Z, Feng B. Global, regional, and national trends and inequality analysis of the total cancers and 29 cancer types from 1990 to 2021. BMC Public Health. 2025 Oct 15;25(1):3494. doi: 10.1186/s12889-025-24671-3. PMID: 41094448;
4. He R, Zhu W, Hui S, Yu M, Li H, Li Y, Huang P, Yu R. Inequalities in disease burden and care quality of neglected tropical diseases and malaria, 1990-2021: Findings from the 2021 Global Burden of Disease Study. PLoS One. 2025 Aug 7;20(8):e0329475. doi: 10.1371/journal.pone.0329475. PMID: 40773432; P

**While the study correlates QCI/GDR with the Socio-Demographic Index (SDI), it does not adjust for granular factors within SDI tiers (e.g., healthcare funding, workforce density, or health insurance coverage). For example, low-SDI countries may have similar SDI scores but vastly different healthcare infrastructures, which the study fails to disentangle.**

**Response:** Many thanks for your professional advice.

Thank you for this insightful comment. We fully agree that countries within the same SDI category may differ substantially in specific healthcare system characteristics, such as healthcare expenditure, workforce density, insurance coverage, and infrastructure capacity, which are not fully captured by the composite SDI measure.

However, SDI is a well-established composite indicator developed within the Global Burden of Disease (GBD) framework, incorporating income per capita, educational attainment, and fertility rate, and has been widely used as a proxy for overall socioeconomic development and health system capacity in global health research ^[1,2,3]^. The use of SDI in this study was intended to examine broad sociodemographic gradients in quality of care rather than to isolate the independent effects of specific healthcare system components.

Importantly, the primary aim of this study was to characterize global, regional, and national patterns and disparities in stroke care quality, rather than to establish causal relationships between healthcare system inputs and quality outcomes. Given the global scope of the analysis and the inclusion of 204 countries and territories over multiple decades, consistent and comparable data on granular healthcare system variables, such as healthcare expenditure, workforce density, and insurance coverage, are not uniformly available across all locations and time periods.

We acknowledge that residual heterogeneity in healthcare system characteristics may exist within SDI strata. This limitation has now been explicitly acknowledged in line 416-430 in manuscript-revised. Future studies incorporating more detailed healthcare system indicators will be essential to further disentangle the relative contributions of specific healthcare resources and policies to variations in care quality.

References

1. GBD 2019 Diseases and Injuries Collaborators. Global burden of 369 diseases and injuries in 204 countries and territories, 1990-2019: a systematic analysis for the Global Burden of Disease Study 2019. Lancet. 2020 Oct 17;396(10258):1204-1222. doi: 10.1016/S0140-6736(20)30925-9. Erratum in: Lancet. 2020 Nov 14;396(10262):1562. doi: 10.1016/S0140-6736(20)32226-1. PMID: 33069326;
2. GBD 2021 Asthma and Allergic Diseases Collaborators. Global, regional, and national burden of asthma and atopic dermatitis, 1990-2021, and projections to 2050: a systematic analysis of the Global Burden of Disease Study 2021. Lancet Respir Med. 2025 May;13(5):425-446. doi: 10.1016/S2213-2600(25)00003-7. Epub 2025 Mar 24. PMID: 40147466.
3. GBD 2021 Adult BMI Collaborators. Global, regional, and national prevalence of adult overweight and obesity, 1990-2021, with forecasts to 2050: a forecasting study for the Global Burden of Disease Study 2021. Lancet. 2025 Mar 8;405(10481):813-838. doi: 10.1016/S0140-6736(25)00355-1.

**GBD showed that The annual number of strokes and deaths due to stroke increased substantially from 1990 to 2021. Data on the burden of stroke in your county should be increased so that readers can understand the need for stroke-related studies. Additional citations could be added: Estimated Burden of Stroke in China in 2020. JAMA Netw Open. 2023;6(3): e231455.’**

**Response:** Many thanks for your professional advice.

We have added the content in line 295-304 and 323-327 in manuscript-revised.
